# Whole-exome sequencing in eccrine porocarcinoma indicates promising therapeutic strategies

**DOI:** 10.1038/s41417-021-00347-z

**Published:** 2021-05-27

**Authors:** Evgeniya Denisova, Dana Westphal, Harald M. Surowy, Friedegund Meier, Barbara Hutter, Julia Reifenberger, Arno Rütten, Alexander Schulz, Mildred Sergon, Mirjana Ziemer, Benedikt Brors, Regina C. Betz, Silke Redler

**Affiliations:** 1 Division of Applied Bioinformatics, German Cancer Research Center (DKFZ), Heidelberg, Germany; 2 Department of Dermatology, Carl Gustav Carus Medical Center, TU Dresden, Dresden, Germany; 3 National Centre for Tumour Diseases (NCT), Partner Site Dresden, Dresden, Germany; 4 Institute of Human Genetics, Medical Faculty and University Hospital Düsseldorf, Heinrich-Heine-University Düsseldorf, Düsseldorf, Germany; 5 Computational Oncology, Molecular Diagnostics Program, National Center for Tumor Diseases (NCT), Heidelberg, Germany; 6 Department of Dermatology, Medical Faculty and University Hospital Düsseldorf, Heinrich-Heine-University Düsseldorf, Düsseldorf, Germany; 7 Dermatopathology, Bodensee, Siemensstrasse 6/1, 88048 Friedrichshafen, Germany; 8 Institute of Pathology, Carl Gustav Carus Medical Center, TU Dresden, Dresden, Germany; 9 Department of Dermatology, Venereology and Allergology, University Medical Center, Leipzig, Germany; 10 National Center for Tumor Diseases (NCT), Heidelberg, Germany; 11 German Cancer Consortium (DKTK), Heidelberg, Germany; 12 Institute of Human Genetics, University of Bonn, Medical Faculty and University Hospital Bonn, Bonn, Germany

**Keywords:** Cancer genetics, Cancer

## Abstract

Malignant sweat gland tumours are rare, with the most common form being Eccrine porocarcinoma (EP). To investigate the mutational landscape of EP, we performed whole-exome sequencing (WES) on 14 formalin-fixed paraffin-embedded samples of matched primary EP and healthy surrounding tissue. Mutational profiling revealed a high overall median mutation rate. This was attributed to signatures of mutational processes related to ultraviolet (UV) exposure, APOBEC enzyme dysregulation, and defective homologous double-strand break repair. All of these processes cause genomic instability and are implicated in carcinogenesis. Recurrent driving somatic alterations were detected in the EP candidate drivers *TP53, FAT2*, *CACNA1S*, and *KMT2D*. The analyses also identified copy number alterations and recurrent gains and losses in several chromosomal regions including that containing *BRCA2*, as well as deleterious alterations in multiple HRR components. In accordance with this reduced or even a complete loss of BRCA2 protein expression was detected in 50% of the investigated EP tumours. Our results implicate crucial oncogenic driver pathways and suggest that defective homologous double-strand break repair and the p53 pathway are involved in EP aetiology. Targeting of the p53 axis and PARP inhibition, and/or immunotherapy may represent promising treatment strategies.

## Introduction

Malignant tumours of the sweat gland are rare, with Eccrine porocarcinoma (EP) being the most common form. Research suggests that EP arises from the intraepidermal ductal portion/acrosyringium of the eccrine cutaneous sweat glands [1–3]. EP accounts for an estimated 0.005%–0.01% of all cutaneous malignancies [4]. EP shows an equal sex distribution and has a peak incidence between the ages of 50 and 80 years [4, 5]. Although the disease may occur de novo, a substantial proportion of EP tumours develop from pre-existing poromas, which represent their benign counterpart [4, 5]. The clinical presentation of EP is highly variable. Contrary to its aggressive nature and its pronounced tendency to local recurrence and metastatic spread, EP is frequently slow-growing and may present in a manner that mimics other skin conditions [6, 7]. EP diagnosis is therefore often delayed, with a poor prognosis for local recurrence after standard wide local excision and no approved treatment approaches in cases of metastatic spread. This renders the identification of effective treatment a key priority for research.

**Fig. 1:**
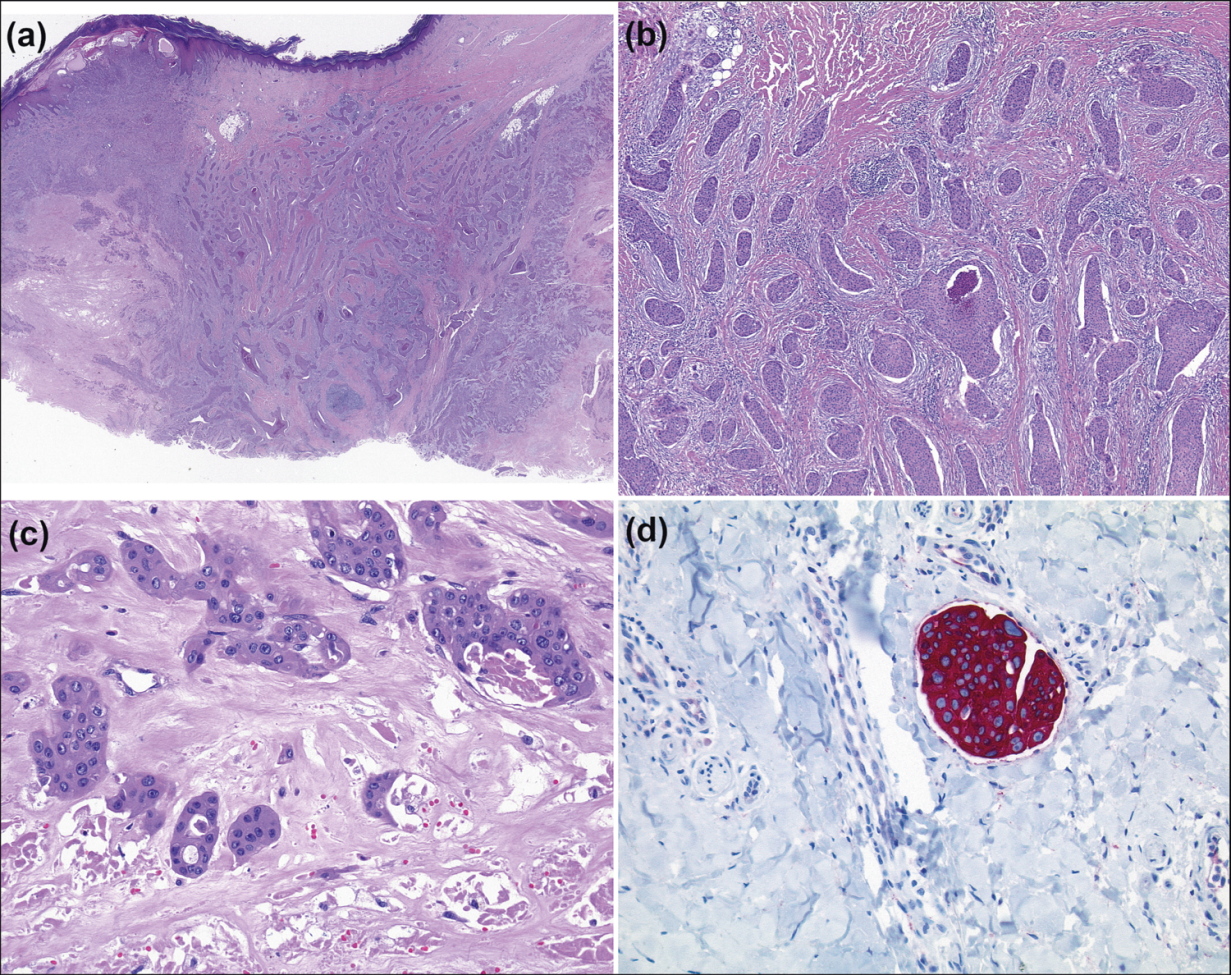
Histopathological and immunohistochemical features of eccrine porocarcinoma, as exemplified by case 1. **a** Scanning image of a porocarcinoma. The neoplasm has an infiltrating growth pattern and is connected to the overlying epidermis. **b** Areas of the neoplasm recapitulating the morphological structures of a poroma. Poroid cell type, necrosis (HE). **c** Epithelial strands with ductal differentiation and cytological atypia surrounding the necrotic areas (HE). **d** Intralymphatic invasion by tumour aggregates (cytokeratin 7).

Histopathological classification is a prerequisite for EP diagnosis and clinical management [1, 2]. Histopathological hallmarks are an infiltrating growth pattern, a connection to the overlying epidermis, and a tendency to ulceration (Fig. 1a). Intraepithelial extensions occur, which resemble those observed in Bowen’s disease. The tumour aggregates have small eosinophilic poroid tumour cells similar to those of a benign poroma and are often characterised by necrosis (Fig. 1b). Tumour cells display atypical nuclei, mitoses, and ductal differentiation. The latter is characterised by intracytoplasmic vacuoles, or true ductal lumina, surrounded by cuticular cells (Fig. 1c). In terms of immunochemistry, these lumina show positive staining for carcinoembryonic antigen (CEA) or epithelial membrane antigen (EMA). In a substantial proportion of cases, EP is associated with early intralymphatic spread; high rates of mitosis (>14/10 high power fields); and a tumour depth of <7 mm [8]. All three features represent poor prognostic signs. Staining with cytokeratin 7 typically reveals intralymphatic invasion by tumour aggregates (Fig. 1d). Differential diagnosis includes benign poroma, which has no cytological atypia and very few mitotic figures. However, even benign poromas may show necrosis, which may render histological classification problematic [1, 2].

Limited data are available concerning EP biology. To date, few porocarcinoma investigations have been performed, and available reports involved sample sizes of only 1 to 6 tumours. These studies focused on known tumour genes and their underlying pathways [9–12]. Their findings suggest that EPs are driven by somatic alterations [9–12]. In particular, these studies suggest that cell-cycle dysregulation, and specific oncogenic drivers and signalling pathways, are involved in EP tumourigenesis. Implicated oncogenic drivers include *TP53* (tumour protein p53); *CDKN2A* (cyclin-dependent kinase inhibitor 2A); *HRAS* (HRas proto-oncogene, GTPase); and *EGFR* (epidermal growth factor receptor). Implicated signalling pathways include *MAPK* (mitogen-activated protein kinase), and *PI3K*-*AKT* (phosphatidylinositol-4,5-bisphosphate 3-kinase, AKT serine/threonine kinase) [9–11].

The aim of this study was to generate insights into EP biology via a comprehensive analysis of tumour-specific alterations using whole-exome sequencing (WES). Primary EP and matched surrounding healthy tissue were investigated. To our knowledge, this represents both the first systematic genome-wide approach to porocarcinoma, and the largest reported EP sample to date.

## Materials and methods

### Patient samples

Matched primary EP and healthy surrounding tissue samples were obtained from 16 unrelated individuals. The study inclusion criteria were: (1) Central European origin; (2) no history of recurrence or metastases; (3) a negative family history of EP; and (4) a confirmed histopathological diagnosis, as assigned by two experienced and designated histopathologists (AR and JR). Ethical approval was obtained from the respective ethics committees, and all participants provided written informed consent prior to inclusion (Ethics vote 5360/13, Heinrich Heine University Düsseldorf). All study procedures were conducted in accordance with the principles of the Declaration of Helsinki.

### Whole-exome sequencing

Exome capture was performed using the Agilent SureSelect XT Human All Exon V7 Enrichment Kit (Agilent). The capture area comprised 35.7 Mb and targeted 99.7% of all coding exons (213,994). Paired-end 100 bp DNA sequencing was performed on a HiSeq 4000 system (Illumina).

### Read mapping and data preprocessing

Mapping and preprocessing were performed using DKFZ OTP (One Touch Pipeline) workflows [13]. Briefly, reads were mapped to the human reference genome build hs37d5 (phase II reference of the 1000 Genomes Project including decoy sequences) using the Burrows-Wheeler Aligner (BWA) version 0.7.15 mem function with all default parameters, except for the invoking of -T 0 [14, 15]. BAM files were sorted using SAMtools version 0.1.19, and PCR duplicates were marked using Sambamba version 0.6.5 [16, 17].

### Detection of somatic single nucleotide variations (SNVs)

SNVs were detected using an in-house workflow based on SAMtools/BCFtools (version 0.1.19). Parameters were adjusted to allow for somatic variant calling and heuristic filtering, as described elsewhere [16, 18, 19].

Briefly, SAMtools mpileup was called on the tumour sample BAM file. Minimum mapping quality was set at 20, and minimum base quality was set at 13. The output was piped to bcftools view. All positions with at least one high-quality non-reference base were reported. From these raw SNV calls, only those supported by at least five variant reads and with a variant allele frequency of at least 5% were selected. Variant calls that were only supported on one strand were discarded if one of the Illumina-specific error profiles [20] occurred in a sequence context of +/−10 bases around the SNV. To categorise variants as germline or somatic, a pileup of the bases in the tumour-matched control sample was generated for each SNV position using SAMtools mpileup. Here, uniquely mapped reads were considered, without base quality restriction. For high confidence somatic SNVs, the coverage at the position in the control was required to be at least 10, with less than 1/30 of the control bases supporting the variant observed in the tumour. To remove artefacts, a ‘confidence score’ was calculated for each mutation. This score was first defined as 10 and then decreased if the mutation overlapped with genomic regions that are known to be prone to artefacts [21, 22]. The latter were identified using annotations from the UCSC genome browser (http://genome.ucsc.edu/cgi-bin/hgTables). Three points were deducted if the overlap was observed with: (1) regions of low mappability, according to wgEncodeCrgMapabilityAlign100mer or hiSeqDepthTopPt1Pct track; (2) Encode DAC Blackslisted regions; or (3) Duke Excluded Region. Two points were deducted if the overlap was observed for any two of the following features: short tandem repeats, simple repeats, low complexity, satellite repeats, or segmental duplications. SNVs with a score of at least 8 were classified as “high confidence”. Annotation was performed with ANNOVAR (version 2016Feb01) [23]. With the exception of the analysis of mutational signatures, only high confidence somatic, non-silent coding variants (i.e., nonsynonymous, stopgain, stoploss, or splicing in a vicinity of 2 bp of exon boundaries) were selected. In the analysis of mutational signatures, all high confidence variants, including non-coding and silent types, were included.

### Detection of somatic indels

Short insertions/deletions (indels) were identified using Platypus (version 0.8.1) with default parameters. A scoring scheme similar to that applied in the SNV workflow was used. To be classified as high confidence, somatic calls (control genotype 0/0) were required to have the Platypus filter flag PASS or to pass custom filters allowing for low variant frequency. Variants marked with the badReads, alleleBias, or strandBias flags were discarded if the variant allele frequency was <10%. In addition, combinations of Platypus non-PASS filter flags, bad quality values, low genotype quality, very low variant counts in the tumour, and the presence of variant reads in the control, were not tolerated. “Indels were annotated with ANNOVAR. Somatic high-confidence indels (minimum confidence of 8) located within a coding sequence or splice site were selected for the analysis. In addition, visual inspection using Integrative Genomics Viewer (IGV) was performed for: (1) HRAS codons 13 and 61, which were identified as being frequently mutated in porocarcinoma in previous reports (refs); and (2) alterations in the recurrently mutated genes discussed in the manuscript [10, 11, 24]. Tumour mutation burden was calculated as the number of non-silent somatic mutations (SNVs and indels) per mega-base of the target region. The oncoprint plot was generated using the ComplexHeatmap R package [25].

### Detection of copy number alterations (CNAs)

CNAs and loss of heterozygosity regions were identified using an in-house workflow based on the software package CNVkit with default parameter settings [26]. Tumour cell content and ploidy were estimated using a method adapted from ACEseq (https://aceseq.readthedocs.io/en/latest/, manuscript in preparation, preprint available at https://www.biorxiv.org/content/10.1101/210807v1.full). Segments with a total copy number (TCN) at least 0.7 above the tumour ploidy were defined as gains. Segments with a TCN at least 0.7 below the tumour ploidy were defined as losses. High-level CNAs were defined as homozygous deletions and amplifications (>2.5× the average ploidy). CNA analysis in Formalin-Fixed Paraffin-Embedded (FFPE) samples is challenging, due to suboptimal DNA quality and consequent uneven coverage. In individual tumours, this may result in artefacts, particularly with respect to copy number losses. To address this, the GISTIC2.0 workflow with default parameters (except for q = 0.1 and log2 ratio amplification/deletion threshold = 0.3/−0.3) was used to identify regions of significantly recurring gains or losses [27].

### Detection of structural variations (SVs)

SVs were called using the in-house tool Sophia (https://bitbucket.org/utoprak/sophia/src/master/). The algorithm is based on the supplementary alignments from BWA mem and discordantly mapping mates. It uses a panel of 3261 normal controls for filtering artefacts and common SVs in the germline. For all events, overlapping as well as adjacent genes, and the closest cancer genes, are annotated.

### Analysis of mutational signatures

Mutational signature contributions were calculated using the R/Bioconductor package YAPSA (Yet Another Package for Signature Analysis, https://bioconductor.org/packages/release/bioc/html/YAPSA.html), as based on Alexandrov-COSMIC signatures-v2 (https://cancer.sanger.ac.uk/cosmic/signatures_v2) [28].

#### Immunohistochemistry (IHC)

To explore whether the deleterious genetic alterations in HRR components such as *BRCA2* lead to corresponding changes in the protein level, we investigated BRCA2 via immunohistochemistry in a cohort of 11 EP tumours. One µm serial section of the different EP tumour tissues was stained for BRCA2 using the BenchMark XT automated stainer (Ventana Medical Systems Inc.). The sections were treated with cell conditioning 1 (CC1) buffer (#950-124, Ventana), and then treated for 60 min with a 1:25 dilution of the anti-BRCA2 antibody (#10741, Cell Signalling). This was followed by incubation with the Optiview DAB IHC Detection Kit (#760-500, Ventana). Stained slides were scanned at ×200 magnification using the Panoramic Scan II (3DHistech) and visualised with the CaseViewer v2.3 (3DHistech). Snapshot images of representative regions were taken at ×100 and ×400 magnification and processed for white balance using Photoshop (Adobe). Overall staining intensity, in comparison to the positive control tissue, was classified by one examiner as: no colour reaction (SI = 0), mild reaction (SI = 1), moderate reaction (SI = 2), or intense reaction (SI = 3). The results were then confirmed by a dermapathologist. Data from individual samples were displayed using Prism v7 (GraphPad). Immunohistochemistry and Western blot analyses of BRCA2 expressing cells treated with BRCA2 siRNA were performed to confirm BRCA2 antibody specificity.

## Results

### Mutational landscape of EP

The tumour and normal tissue samples of two patients were excluded from the analysis due to poor sequencing performance (<30×). In the remaining 14 sample pairs, a median target region coverage of 162× was achieved (Supplemental Table 1).

In the 14 included EP tumour samples, the analyses identified a total of 15,213 (median: 905; range: 13–2265) somatic tumour-specific SNVs (Table 1 and Supplemental Table 2) within the WES target regions. Of these, 10,024 (median, 630; range, 11–1488) were non-silent. The analyses also identified 51 somatic small insertions and deletions (median, 15; range, 0–24) (Table 1 and Supplemental Table 3). Of these, 49 were non-silent (median, 1; range, 0–23). The median rate of somatic mutations was 25.35 (range, 0.36–63.47), and the median rate of somatic protein-altering mutations was 17.66 (range, 0.31–41.71) per megabase of the target sequence.

**Table 1:** Tumour-specific variant counts and mutation rates.

PID	SNV in target regions	Functional SNV	INDEL in target regions	Functional INDEL	Mutation frequency/Mb**	Functional mutation frequency/Mb**
C000-EXKBCS	2265	1488	1	1	63,47	41,71
C000-6KNGDL	2241	1434	4	4	62,89	40,28
C000-HZGGV2	2185	1396	10	9	61,46	39,38
C000-RCWM1J	2176	1361	4	4	61,06	38,24
C000-X88E7Z	1576	1066	5	5	44,29	30,00
C000-ESMKCH	1468	947	0	0	41,12	26,53
C000-CFLP5N	923	640	0	0	25,85	17,93
C000-ZZP8UU	887	621	0	0	24,85	17,39
C000-2SXE39	590	437	24	23	17,17	12,91
C000-S5ZHWR	415	293	2	2	11,68	8,26
C000-JMUDMJ	354	247	1	1	9,94	6,95
C000-648QQH	90	63	0	0	2,52	1,76
C000-W91YB6	30	20	0	0	0,84	0,56
C000-AX1WLG	13	11	0	0	0,36	0,31
**Total**	15213	10024	51	49		
**Median**	905	630,5	1	1	25,35	17,66

*Including variants outside target regions.

**Per Mb of target sequence (35.7 Mb).

The genes showing the highest number of coding mutations in EP were the following tumour suppressor genes: *TP53* and *FAT2* (FAT atypical cadherin 2) (mutated in 6 patients, respectively); and *CACNA1S* (calcium voltage-gated channel subunit alpha1 S) and *KMT2D* (lysine methyltransferase 2D) (mutated in 5 patients, respectively). One of the samples (C000-HZGGV2) with an SNV in *TP53* also carried a small deletion (Fig. 2.1).

**Fig. 2:**
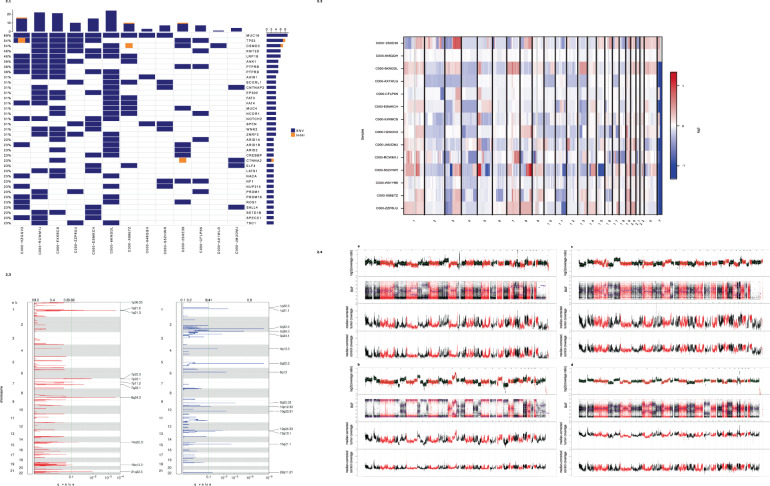
Frequently mutated genes in Ecrrine porocarcinoma. Rows represent individual genes, columns represent individual tumours. Genes are sorted according to the frequency of coding single nucleotide variations. One tumour (C000-W91YB6) is not included in the oncoprint as it did not carry alterations in the most frequently altered genes. 2.2: Overall copy number alterations pattern in the present cohort. Relative chromosomal gains (red) and losses (blue) in tumour/normal pairs. Chromosomes are represented along the horizontal axis, tumour samples are represented along the vertical axis. 2.3: Recurrent copy number alterations. GISTIC2.0 plot of recurrent focal gains (**a**) and losses (**b**). Chromosomes are represented along the vertical axis, q values are marked along the horizontal axis. The green lines mark the cut-off for the significance threshold (*q* = 0.1). 2.4: High-level copy number alterations in porocarcinoma. Examples of coverage profiles of highly rearranged tumours. Each plot depicts, from top to bottom: log2 ratio of coverage between tumour and normal samples, B allele frequency (BAF) plot, median-corrected tumour coverage, and median-corrected control coverage. The presented cases include the most prominent CNAs, including *MYC* (8q24) and *TERT* (5p15) amplification in C000-S5ZHWR (**a**); *BCL3* (19q13) amplification and *RB1* (13q14) loss in C000-X88E7Z (**b**); *EGFR* (7p11) amplification in C000-2SXE39 (**c**); *ERBB2* (17q12) amplification and losses of *STK11* (19p13), *CDKN2A*/*CDKN2B* (9p21) in C000-RCWM1J (**d**).

In addition, the analyses revealed several recurrently mutated genes with long protein-coding sequences, including *TTN* (titin); *CSMD3* (CUB and Sushi multiple domains 3); *MUC16* (mucin 16, cell surface-associated); *LRP1B* (LDL receptor-related protein 1B); and *ZFHX4* (zinc finger homeobox 4).

Among genes with a previously reported EP-associated mutation, coding SNVs were detected in: *ARID1A* (AT-rich interaction domain 1A) (mutated in 3 patients); *APC* (APC regulator of WNT signalling pathway), *ERBB4* (erb-b2 receptor tyrosine kinase 4), *NCOR1* (nuclear receptor corepressor 1), and *PDGFRA* (platelet-derived growth factor receptor alpha) (mutated in 2 patients, respectively); and *ATM* (ATM serine/threonine kinase) and *CDKN2A* (mutated in 1 patient, respectively) [10]. No recurrent indels were identified. No mutations were identified in *HRAS*, *EGFR*, or *RB1* (RB transcriptional corepressor 1) [11].

### Mutational signature analysis

**Fig. 3:**
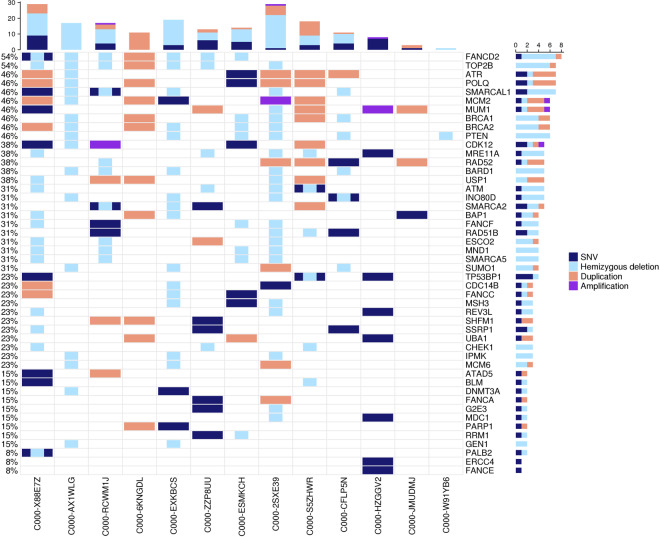
Alterations in genes previously implicated in sensitivity/resistance to poly ADP ribose polymerase (PARP) inhibitors. Rows represent individual genes, columns represent individual tumours. Genes are categorised according to the frequency of mutations and copy number alterations. Bars depict the number of alterations for individual tumours (top) and genes (right). Tumour C000-648QQH is not included in the oncoprint as it did not carry alterations in the respective genes.

We investigated the prevalence of mutation signatures using YAPSA, which includes Alexandrov-COSMIC-v2 (AC) signatures [28]. The most prominent mutational signature (detected in 11/14 samples) was the AC7-UV-induced signature, which is typically observed in skin cancers that occur on sun-exposed areas of the body. Another frequent signature (detected in 10 samples) was AC11, which has been associated with alkylating agents (specifically temozolomide) [28]. APOBEC signatures AC2 and AC13 were detected in 8 and 7 samples respectively. These signatures have been attributed to the activity of the AID/APOBEC family of cytidine deaminases [28]. The -“BRCAness” signature AC3 was detected in 4 samples. This mutational signature has been associated with defective homologous recombination repair (HRR) [28]. The presence of this signature in several cases prompted us to investigate whether EP tumours exhibit other signs of defective HRR. Here, we examined genes whose disruption has previously been implicated in sensitivity/resistance to PARP (poly(ADP-ribose) polymerase) inhibitors [29]. In nearly all (13/14) EP tumours, deleterious alterations were identified in multiple HRR components, including *PTEN* (phosphatase and tensin homologue); *BARD1* (BRCA1 associated RING domain 1); *ATM* (ataxia telangiectasia mutated); *BRCA1* (breast cancer type 1 susceptibility protein); *BRCA2* (breast cancer type 2 susceptibility protein); *CHEK1* (checkpoint kinase 1); and *ATR* (ataxia telangiectasia and Rad3-related protein). Deleterious alterations were also identified in nearly all (13/14) tumours in members of the Fanconi anaemia complementation groups, including *FANCD2*, *FANCF*, *FANCC*, *FANCA*, and *FANCE* (Figs. 2.2, 3).

### Copy number alterations (CNAs)

**Fig. 4:**
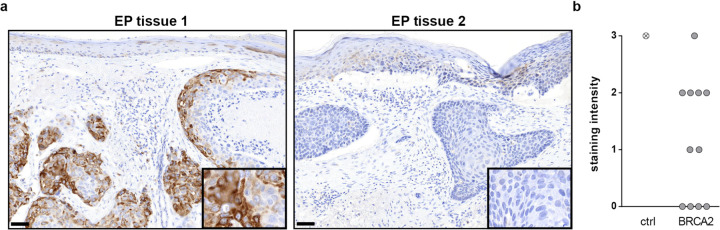
Immunohistochemistry analyses of eccrine porocarcinoma tissue reveal deregulation of the HRR component BRCA2. **a** Representative images of positive (left panel) and negative (right panel) BRCA2 staining in EP tumour tissue. All images are shown at ×100 magnification. The inlays are shown at ×400 magnification. Scale bar in the bottom left corner indicates 100 µm. Representative images of the positive control are displayed Supplemental Fig. S1. **b** IHC analyses of 11 EP tumours reveal diverse BRCA2 staining. A qualitative score of 0 (no colour reaction), 1 (mild reaction), 2 (moderate reaction), and 3 (intense reaction) was assigned and displayed in an *XY* graph.

Analysis of somatic CNAs identified recurrent gains in regions of chromosomes 1p21, 7p22, 8q24, and 21q22. Recurrent losses were identified in chromosomal regions 1p21, 1p22; 2q32, 2q36; 3p24; 4q13; 5q22 (containing *APC*); 6q13; 9q22; 10p12, 10q23 (containing *PTEN* (phosphatase and tensin homologue)); 12q24; 13q13 (containing *BRCA2* (BRCA2 DNA repair associated)); 15q21; and 22q11 (Figs. 2.2, 2.3, 4 and Supplemental Table 4).

Furthermore, in individual tumours, multiple known cancer drivers were affected by high-level CNAs (Supplemental Table 5). Amplifications were observed for *EGFR; TERT* (telomerase reverse transcriptase); *MYC* (MYC proto-oncogene, bHLH transcription factor [Homo sapiens); *BCL3* (BCL3 transcription coactivator); and *ERBB2* (erb-b2 receptor tyrosine kinase 2). Homozygous deletions were observed for *RB1; CDKN2A*/*CDKN2B* (cyclin-dependent kinase inhibitor 2B); *BRCA2; APC; STK11* (serine/threonine kinase 11); *TSC1* (TSC complex subunit 1); *VHL* (von Hippel-Lindau tumour suppressor); *MSH2* (mutS homologue 2); *WT1* (WT1 transcription factor); and *MLH1* (mutL homologue 1). Several tumours displayed highly rearranged genomes (Fig. 2.4), and two tumours (C000-2SXE39 and C000-CFLP5N) were estimated to be tetraploid.

### Structural variations (SVs)

The high number of cross-linking artefacts (typical for FFPE samples) precluded a comprehensive SV analysis. A specific search was made for evidence of the gene fusions *YAP1*-*MAML2* and *YAP1*-*NUTM1* since these were reported in previous EP research [30]. None were detected.

#### Immunohistochemistry results

Cytoplasmic BRCA2 staining (SI = 2 or 3), comparable to that of the positive control (comparison Fig. 4a left panel and Supplemental Fig. 1), was detected in around 50% of the investigated EP tumours (Fig. 4a, b). Intriguingly, in the other 50% of EP tumours, BRCA2 staining was either weak or completely absent (SI = 0 or 1) (Fig. 4, Supplemental Fig. 1).

## Discussion

The aim of this study was to generate insights into the mutational landscape and pathogenic causes of the clinically well-defined sweat gland carcinoma subtype EP. To study the underlying molecular mechanisms of tumour origin, we performed WES in the largest reported EP cohort to date. The analyses were performed using histologically well-characterised FFPE samples of matched primary EP and healthy surrounding tissue. To our knowledge, only one study to date has performed genomic profiling in EP using fresh frozen tissue. However, the study investigated only a single metastatic EP sample and did not analyse tissue from the primary tumour [9].

Compared to fresh frozen tissue or blood samples, DNA samples isolated from FFPE tissue pose an analytical challenge, since the FFPE process usually leads to shorter DNA library insert sizes, and necessitates the generation of more DNA sequencing data. Although this leads to increased duplication rates and more variability in median coverage (Table 1), research has shown that FFPE samples do permit the reliable detection of genetic alterations, if sufficient sequence coverage can be obtained [31, 32]. In this study, only four of 32 samples were excluded due to low sequencing depths, which is favourable compared to other WES studies of FFPE material [31]. The four low-quality samples were obtained from both the EP as well as the matched surrounding tissue samples of two patients, indicating that the low quality reflected a general aspect of the fixation process.

The 14 primary EP tumours exhibited a median of 25.35 mutations per Mb of target sequence (approximate 100fold range). These mutations can be attributed to signatures of known mutational processes. The influence of chemotherapy as an alternative cause for such mutations is unlikely in non-metastatic primary tumour tissue. The identified mutational processes—which are frequently implicated in carcinogenesis—may be related to UV exposure, dysregulation of the APOBEC enzyme, or defective homologous double-strand break repair, and may cause genomic instability [33–35]. These observations are consistent with known mutation signatures in melanoma, as well as in basal-cell-carcinoma and squamous-cell carcinoma (SCC) of the skin (https://cancer.sanger.ac.uk/cosmic/signatures) [35, 36]. The high overall median mutation rate of EP is most comparable to that of SCC, which may suggest common mechanisms of tumourigenesis [35, 36]. Notably, this similarity between EP and SCC is consistent with: i) the histopathological overlap between EP and SCC, which leads to difficulties in differential diagnosis within the clinical practice; and ii) the hypothesis that a combination of anti-cancer drugs used in the management of advanced SCC may be an effective treatment strategy for metastatic EP [1, 2, 37]. In general, hypermutated tumours are sensitive to immunotherapy, and this emerging treatment option is now revolutionising the therapeutic paradigm in an increasing number of tumours. The present data suggest that EP may also be responsive to this therapeutic strategy. In particular, checkpoint blockade using antibodies that impede immune inhibitory pathways represents a novel strategy that might also serve as a therapeutic option in advanced or nonresectable EP [38, 39]. The hypothesis that EP is sensitive to immunotherapy is supported by a successful therapeutic trial in a patient with metastatic EP. In this patient, EGFR-directed therapy and immune checkpoint inhibition during anti-PD-1 therapy with nivolumab prevented tumour progression for 7 and 5 months, respectively (preliminary data, Westphal et al., Manuscript in preparation).

In the present EP cohort, analysis of WES data revealed that *TP53*, *FAT2*, *KMT2D*, and *CACNA1S* were the most frequently mutated genes. Mutations in the cell cycle regulator and tumour suppressor gene *TP53* constitute the most common genetic lesion in human cancer. Mutations in *TP53* are found in nearly half of all reported cancer cases. Partial inactivation of the p53 pathway is a key event in both tumourigenesis and tumour progression [40–42]. Attempts to develop cancer therapies that target specific mutant genes have proven highly effective, and clinical trials of agents that exploit the p53 system are ongoing [40–42]. These developments raise the hope that the targeting of mutant p53 represents a potential treatment approach for patients with EP. However, further studies are warranted to test this hypothesis. These investigations should include both a more detailed characterisation of this locus in a larger cohort and a comparison of somatic and germline findings. In the present cohort, p53 staining revealed increased or decreased p53 levels in the majority of EP samples, indicating stabilising gain-of-function or destabilising loss-of-function mutations respectively (preliminary data, manuscript in preparation, Westphal et al.).

*FAT2* is an atypical cadherin that may act as a receptor for the Hippo pathway, and which has recently been shown to play a critical role in tissue homoeostasis via canonical signalling. Dysregulation of the Hippo pathway is implicated in multiple non-malignant pathologies, immunological processes, and cancer [43–45].

*KMT2D* is a member of the histone-lysine N-methyltransferase (KMT2) family, which is of key relevance in terms of transcription regulation. *KMT2D* is crucial for H3K4 histone methylation at distal enhancers, and the interplay between KMT2-dependent H3K4 methylation and DNA methylation suggests that these histone modifications show high epigenetic stability. Mutations in the KMT2 family are a frequent finding in many cancer entities, including haematological malignancies and solid tumours [46]. First-generation inhibitors of KMT2 function, which target the wild-type and fusion proteins of KMT2A, have now been developed within the research setting and may represent a future, novel treatment strategy for multiple cancer entities [46].

*CACNA1S* encodes the calcium channel α-subunit of the slowly inactivating L-type voltage-dependent calcium channel that facilitates L-Type calcium currents in skeletal muscle cells*. CACNA1S* is associated with susceptibility to hypokalaemic periodic paralysis, thyrotoxic periodic paralysis, and malignant hyperthermia [44, 45]. Voltage-gated calcium channels are also implicated in mitogenesis, proliferation, differentiation, apoptosis, and metastasis. In addition, previous research has identified an association between voltage-gated calcium channels and prostate cancer [46, 47].

The present analyses also identified a number of recurrently mutated long genes, such as *TTN*, *CSMD3*, *MUC16*, *LRP1B*, and *ZFHX4*. However, gene length is one determinant of the probability of a mutation occurring in a given gene, and frequently mutated long genes have been reported for a wide range of tumour entities [48, 49]. Whether these mutations constitute passenger events or suggest instead that the respective genes play an active role in cancer development and tumour evolution, remains controversial [50]. This effect is more prominent in tumours with a high mutational burden, as was the case in the present EP cohort, and was therefore expected.

Previous research has implicated a number of additional genes in EP development, i.e., *ARID1A*, *APC*, *ERBB4*, *NCOR1*, *PDGFRA*, *ATM CDKN2A, HRAS*, *EGFR*, and *RB1* [9–12]. In this study, mutations in the genes *ARID1A*, *APC*, *ERBB4*, *NCOR1*, *PDGFRA*, *ATM*, and *CDKN2A* were either non-recurrent or of very low frequency, while none of the 14 sequenced EP tumours harboured mutations in the genes *HRAS*, *EGFR, or RB1*. To our knowledge, the present WES approach represents the most comprehensive molecular profiling in EP to date. Therefore, a plausible hypothesis is that mutations in these genes—in particular *HRAS*, *EGFR*, and *RB1*—were coincidental findings in individual tumours, and are not recurrent driver events in EP tumourigenesis. An alternative hypothesis is that these cross-study differences in mutational profiles are attributable to the well-known cancer-related phenomena of: (i) genetic heterogeneity; (ii) inter-tumour and intra-tumour heterogeneity and thus differences in mutational profiles secondary to the nature of the investigated cell clone; and (iii) rapid genomic changes in response to therapeutic pressure [51–53]. Furthermore, current standard high-throughput DNA sequencing approaches are predominantly suited to the detection of small point mutations, and are—in the case of WES—focused on mutations with disrupting or deleterious effects on protein-coding sequences. However, recent studies have underlined that across nearly all tumour types, genetic architecture is also determined by structural variations, such as deletions or duplications of whole gene regions, or complex, genome-wide, and localised rearrangements [51, 52].

In accordance with these findings, our results suggest that individual EP tumours arise secondary to CNAs in multiple known cancer drivers, such as the previously reported genes *RB1* and *EGFR*. Notably, the analyses identified CNAs in distinct, high frequency, and high penetrance cancer genes, which are consistent with the involvement of crucial cancer pathways. These include tumour suppressor genes and pathways implicated in previous cancer research, e.g., the WNT signalling pathway; the AKT/PKB signalling pathway; HRR; DNA mismatch repair; regulation of anabolic cell growth; cell cycle progression; apoptosis; and cellular transformation [54–61]. The present findings will thus facilitate mechanistic understanding of this rare, and relatively unexplored, tumour entity.

An unexpected finding of this study was that the HRR pathway for double-strand DNA repair also appears to play a key role in EP biology. The “BRCAness” point mutation signature (AC3), which is associated with defective HRR, was detected in a number of EP samples. Furthermore, our analyses revealed recurrent losses in chromosomal region 13q, which contains *BRCA2*, as well as high-level CNAs of *BRCA2*. In accordance with this, BRCA2 protein expression was weak or completely absent in around 50% of the tested EP tumour samples (Fig. 4). BRCA2 protein staining was mainly observed in the cytoplasm of EP cells. This may be attributed to a novel role in the motility and migration of cancer cells that were proposed for cytoplasmic and membranous localised BRCA1 and BRCA2 proteins [62, 63].

Failure of HRR is important since several HRR processes play a critical role in cancer development. In accordance with this, BRCA2 protein expression was weak or completely absent in around 50% of the tested EP tumour samples (Fig. 4). Failure of HRR is important since several HRR processes play a critical role in cancer development. Interestingly, *BRCA2* is a key and well-characterised player in the crucial cancer-related processes of HRR [35]. Poly-ADP ribose polymerase (PARP) inhibitors are approved drugs for tumours harbouring mutations in either *BRCA1* or *BRCA2*. Furthermore, previous research has shown that p53—which was one of the key cancer drivers in the present EP cohort—regulates key processes of HRR, and interacts directly with BRCA2 [59, 60]. Of note, next to BRCA2 deleterious alterations, we also observed sporadic BRCA2 duplications. These duplications, however, did not lead to higher protein expression, suggesting that not all genetic alterations may be translated to the protein level. The present analyses generated evidence for further signs of defective HRR. Deleterious alterations were not only found in BRCA2 but in multiple other HRR components, including several genes for which disruption has been implicated in sensitivity/resistance to PARP (poly(ADP-ribose) polymerase) inhibitors in previous research (Figs. 2.2, 3). These HRR pathway findings are surprising, since although it plays a crucial role in the development of various tumour entities [54, 55], the HRR pathway has not been the focus of previous skin tumour research, with the exception of melanoma. Here, previous studies have proposed that germline variants in the genes *BRCA1* and *BRCA2* may represent predisposing factors. However, this currently remains controversial [61, 62]. In particular, it remains unclear whether the BRCA and HRR pathway is associated with melanoma subtypes in general, i.e., cutaneous, uveal, acral, and mucosal, or whether each melanoma subtype has a distinct aetiology. Previous studies found that the p53 and BRCA pathways played a key role in uveal melanoma patients with a poor prognosis [61, 62].

Our study is limited by the comparatively small number of samples, which means that we could not immunohistochemically investigate every genetic variation in a sufficient number of samples [64–70]. Of note, only 2 samples showed duplication of *BRCA2*. Larger studies in independent samples are warranted to substantiate our findings and to better understand the role of different genetic variants including duplications. This will be a major step towards a better understanding of the HRR pathway/ the “BRCA-ness” phenotype in the development of EP. Nevertheless, our findings open new avenues for an in-depth understanding of EP pathogenesis and promising drug candidates. Further clinical trials must prove direct evidence for suggested therapeutic interventions.

In conclusion, this study generated novel insights into the previously unexplored mutational landscape of EP and rare sweat gland carcinomas. The results implicate several crucial oncogenic pathways in EP aetiology. The possible involvement of the p53 pathway and HRR suggest that targeted PARP inhibition represents a potential future therapeutic approach. In addition, primary EP may be responsive to immunotherapy, and checkpoint blockade using antibodies that impede immune inhibitory pathways may represent a novel treatment strategy. Further research is warranted to elucidate the genetic architecture and functional effects of risk loci and to determine the effectiveness of existing therapeutic strategies, such as PARP inhibitors and immune checkpoint inhibitors, in the clinical management of EP.

## Supplementary information

Supplementerial Material

Supplemental Table 3

Supplemental Table 4

Supplemental Table 2

Supplemental Table 5

Supplemental Table 1

## Data Availability

The generated sequencing data were deposited at the European Genome-phenome Archive (EGA) under accession number EGAS00001004632.
