# Supplementary material for: Whole-exome sequencing in eccrine porocarcinoma indicates promising therapeutic strategies
Source: Cancer Gene Ther. 2021 May 27;29(6):697–708. doi: 10.1038/s41417-021-00347-z (PMC9209330; doi:10.1038/s41417-021-00347-z)
Supplement: Supplementary file 1 — Supplementerial Material [file 41417_2021_347_MOESM1_ESM.pdf]

Supporting information

Whole-exome sequencing in eccrine porocarcinoma indicates promising therapeutic strategies

Evgeniya Denisova[1], Dana Westphal[2,3], Harald M. Surowy[4], Friedegund Meier[2,3], Barbara Hutter[1,5], Julia Reifenberger[6], Arno Rütten[7], Alexander Schulz[3], Mildred Sergon[8], Mirjana Ziemer[9], Benedikt Brors[1,10,11], Regina C. Betz[12,*], Silke Redler[4,*,#]

[1]Division of Applied Bioinformatics, German Cancer Research Center (DKFZ), Heidelberg, Germany

[2]Department of Dermatology, Carl Gustav Carus Medical Center, TU Dresden, Dresden, Germany

[3]National Centre for Tumour Diseases (NCT), Partner Site Dresden, Germany

[4]Institute of Human Genetics, Medical Faculty and University Hospital Düsseldorf, Heinrich-Heine-University Düsseldorf, Germany

[5]Computational Oncology, Molecular Diagnostics Program, National Center for Tumor Diseases (NCT), Heidelberg, Germany

[6]Department of Dermatology, University Hospital Düsseldorf, Düsseldorf, Germany

[7]Dermatopathology, Bodensee, Siemensstrasse 6/1, 88048 Friedrichshafen, Germany

[8]Institute of Pathology, Carl Gustav Carus Medical Center, TU Dresden, Dresden, Germany

[9]Department of Dermatology, Venereology and Allergology, University Medical Center, Leipzig, Germany

[10]National Center for Tumor Diseases (NCT), Heidelberg, Germany

[11]German Cancer Consortium (DKTK), Heidelberg, Germany

[12]Institute of Human Genetics, University of Bonn, Medical Faculty & University Hospital Bonn, Bonn, Germany

[*]contributed equally.

#Correspondence to:

Prof Silke Redler, MD
Institute of Human Genetics
University of Düsseldorf
Universitätsstr. 1
D-40225 Düsseldorf
Germany
Phone: +49 211 81 10423
Fax: +49 211 81 12358
Email: silke.redler@med.uni-duesseldorf.de

Supporting Information

Supplemental Fig. 1: Immunohistochemistry and Western Blot analyses of BRCA2-expressing cells treated with BRCA2 siRNA confirm BRCA2 antibody specificity.

The BRCA2 expressing prostata carcinoma cells line LNCAP was treated for 48 h with 43 nM BRCA2 siRNA (siBRCA2, 5‘-CCAACUUUGUCCUUAACUA-3‘, Eurofins) or scrambled siRNA (siSCR, 5‘-GCAGCUAUAUGAAUGUUGU-3‘, Eurofins), according to the Lipofectamine RNAiMAX protocols. **a,** In immunohistochmical analyses, LNCAP cells treated with the BRCA2 siRNA show almost no BRCA2 protein expression. Treated cells were resuspended in HistoGel (#HG4000012, Thermoscientific) in accordance with the manufacturers‘ protocols, then fixed with 4% formalin and embedded in paraffin. One µm sections of these cell blocks were stained for BRCA2 (see Methods section in main text). Representative images at 100x and 400x (inlays) magnification were taken of the siSCR (left panel) and siBRCA2 (right panel) treated cells showing strong and no BRCA2 staining, respectively. **b,** In Western blot analyses, LNCAP cells treated with the BRCA2 siRNA do not display any discernible BRCA2 protein band at 380 kDa. Treated cells were lysed in IP-lysis buffer (50 mM Hepes, pH7.5; 150 mM NaCl; 10% glycerol; 1% Triton-X-100; 1.5 mM $MgCl_2$; 1 mM EDTA). 25 and 50 µg protein were loaded onto 4-20% gradient gels (#456-1083, BioRad) and blotted onto nitrocellulose membranes (#170-4159, BioRad). Membranes were incubated with BRCA2 (#10741) or GAPDH (#2118) antibodies from Cell Signaling at a 1:1000 dilution o/n at 4°C, followed by an incubation with a HRP-conjugated, rabbit secondary antibody (#NA934, GE Healthcare) at 1:3000 dilution for 1 h at RT. Proteins were detected with Dura Extended Duration substrate (#34075, Thermo Fisher Scientific) and visualised using the ImageQuant LAS 4000 detection system (GE Healthcare). Images of the uncut BRCA2- (left panel) and GAPDH- (right panel) stained membranes are shown. *is a non-specific band at 35 kDa detected with the BRCA2 antibody.

Supplemental table 1: Whole-exome sequencing coverage statistics

Supplemental table 2: Tumour-specific coding single nucleotide variants

Supplemental table 3: Tumour-specific coding small indels

Supplemental table 4: Recurrent tumour-specific copy number gains and losses

Supplemental table 5: Mutational signatures of the single tumours.

Supplemental Figure 1

a

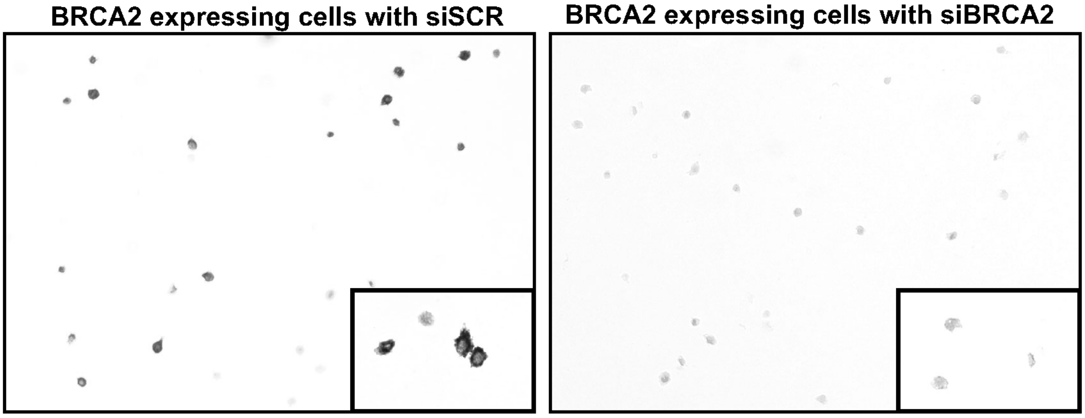

b

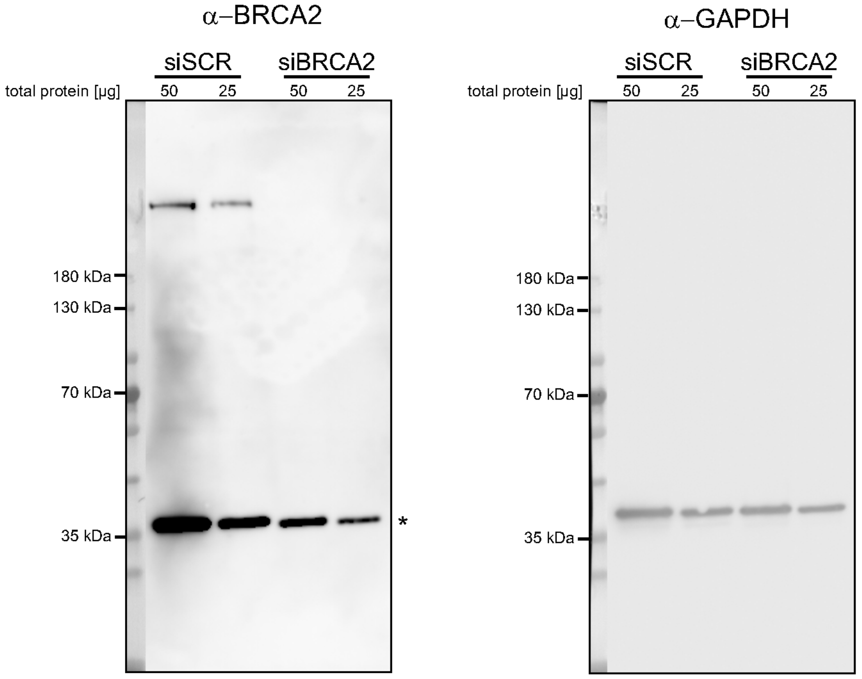

Supplemental Fig. 1: Immunohistochemistry and Western blot analyses of BRCA2 expressing cells treated with BRCA2 siRNA confirm BRCA2 antibody specificity. The BRCA2 expressing prostate carcinoma cells line LNCAP was treated for 48 h with 43 nM BRCA2 siRNA (siBRCA2, 5'-CCAACUUUGUCCUUAACUA-3', Eurofins) or scrambled siRNA (siSCR, 5'-GCAGCUAUAUGAAUGUUGU-3', Eurofins), according to the Lipofectamine RNAiMAX protocols. **a,** In immunohistochmical analyses, LNCAP cells treated with the BRCA2 siRNA show hardly any BRCA2 protein expression. Treated cells were resuspended in HistoGel (#HG4000012, Thermoscientific) following the manufacturers' protocols, then fixed with 4% formalin and embedded in paraffin. One µm sections of these cell blocks were stained for BRCA2 (see method section in main text). Representative images at 100x and 400x (inlays) magnification were taken of the siSCR (left panel) and siBRCA2 (right panel) treated cells showing strong and no BRCA2 staining, respectively. **b,** In Western blot analyses, LNCAP cells treated with the BRCA2 siRNA do not display any discernible BRCA2 protein band at 380 kDa. Treated cells were lysed in IP-lysis buffer (50 mM Hepes, pH7.5; 150 mM NaCl; 10% glycerol; 1% Triton-X-100; 1.5 mM $MgCl_2$; 1 mM EDTA). 25 and 50 µg protein were loaded onto 4-20% gradient gels (#456-1083, BioRad) and blotted onto nitrocellulose membranes (#170-4159, BioRad). Membranes were incubated with BRCA2 (#10741) or GAPDH (#2118) antibodies from Cell Signaling at a 1:1000 dilution o/n at 4°C, followed by an incubation with a HRP-conjugated, rabbit secondary antibody (#NA934, GE Healthcare) at 1:3000 dilution for 1 h at RT. Proteins were detected with Dura Extended Duration substrate (#34075, Thermo Fisher Scientific) and visualized using the ImageQuant LAS 4000 detection system (GE Healthcare). Images of the uncut BRCA2- (left panel) and GAPDH- (right panel) stained membranes are shown. *is a non-specific band at 35 kDa detected with the BRCA2 antibody.

Supplemental Table 1: Whole-exome sequencing coverage statistics*

Patient ID	Sample Type	Coverage	Mapped Reads %	Duplicates%	Properly Paired%	Insert Size (Median)
**C000-2SXE39**	CONTROL	224,98	99,99	33,68	97,55	149
**C000-2SXE39**	TUMOR	278,03	99,99	24,65	96,87	162
**C000-648QQH**	CONTROL	78,02	99,99	68,38	96,97	132
**C000-648QQH**	TUMOR	71,98	99,99	67,19	97,27	134
**C000-6KNGDL**	CONTROL	108,14	99,98	55,07	96,58	137
**C000-6KNGDL**	TUMOR	178,62	99,98	29,92	91,94	135
**C000-AX1WLG**	CONTROL	257,71	99,99	28,49	97,93	161
**C000-AX1WLG**	TUMOR	243,26	99,99	23,6	97,78	159
**C000-CFLP5N**	CONTROL	34,15	99,97	80,74	95,23	120
**C000-CFLP5N**	TUMOR	35,65	99,98	81,84	96,05	120
**C000-ESMKCH**	CONTROL	37,32	99,98	78,11	94,86	123
**C000-ESMKCH**	TUMOR	54,64	99,99	72,62	96,74	123
**C000-EXKBCS**	CONTROL	221,06	99,98	41,05	96,97	152
**C000-EXKBCS**	TUMOR	176,44	99,99	45,4	96,91	149
**C000-HZGGV2**	CONTROL	113,57	99,98	56,14	95,87	132
**C000-HZGGV2**	TUMOR	145,67	99,99	43,49	94,66	126
**C000-JMUDMJ**	CONTROL	170,8	99,99	45,81	97,68	148
**C000-JMUDMJ**	TUMOR	169,39	99,99	45,27	97,25	147
**C000-RCWM1J**	CONTROL	77,21	99,96	58,57	93,74	133
**C000-RCWM1J**	TUMOR	185,73	99,98	24,87	90,85	137
**C000-S5ZHWR**	CONTROL	144,49	99,98	47,84	97,31	144
**C000-S5ZHWR**	TUMOR	237,27	99,99	35,62	97,04	151
**C000-W91YB6**	CONTROL	155,08	99,99	40,2	97,87	149
**C000-W91YB6**	TUMOR	287,49	99,99	34,14	97,72	146
**C000-X88E7Z**	CONTROL	162,19	99,98	33,21	97,58	149
**C000-X88E7Z**	TUMOR	488,43	99,99	48,13	96,17	140
**C000-ZZP8UU**	CONTROL	87,77	99,99	65,91	96,17	125
**C000-ZZP8UU**	TUMOR	60,2	99,98	78,65	95,36	125
**C000-XWCDE5***	CONTROL	4,76	99,95	91,63	97,29	110
**C000-XWCDE5***	TUMOR	19,05	99,97	88,5	97,06	115
**C000-DZCVJ8***	CONTROL	19,49	99,96	88,44	95,27	117
**C000-DZCVJ8***	TUMOR	14,61	99,97	91,27	97,59	116

*samples with a median coverage of < 20X. Those were excluded from the analysis.

C000-2SXE39

Chromosome	Position (hg'	Reference al	Mutant allele	Sequence context	ANNOVAR fu	Gene	Mutation type	ANNOVAR output (alternative transcripts)
1	1290861	G	T	TGCCGTCGTC,AAGGCCGAGG	exonic	MXRA8	nonsynonymous SNV	MXRA8:ENST00000309212.6:exon3:c.C345A:p.F115L,MXRA8:ENST00000477278.2:exon3:c.C318A:p.F106L,MXRA8:ENST00000445648.2:exon3:c.C345A:p.F115L,
1	3415750	C	A	TGGCACTGCC,GTGGACAGGG	exonic	MEGF6	nonsynonymous SNV	MEGF6:ENST00000485002.2:exon24:c.G3067T:p.G1023W,MEGF6:ENST00000356575.4:exon24:c.G3046T:p.G1016W,
1	5967219	T	C	AGCTGTGAAG,AGGCCTGGCC	exonic	NPHP4	nonsynonymous SNV	NPHP4:ENST00000378156.4:exon13:c.A1567G:p.T523A,NPHP4:ENST00000489180.2:exon13:c.A1567G:p.T523A,
1	22927288	C	A	CCTACTGGAA,ATGACCAACC	exonic	EPHA8	nonsynonymous SNV	EPHA8:ENST00000166244.3:exon14:c.C2523A:p.N841K,
1	28856416	C	A	AGGTCCCCCC,AGCAGATGCC	exonic	RCC1	nonsynonymous SNV	RCC1:ENST00000419074.1:exon3:c.C38A:p.P13Q,RCC1:ENST00000486790.2:exon2:c.C344A:p.P115Q,RCC1:ENST00000373833.6:exon5:c.C38A:p.P13Q,RCC1:ENST00000430407.1:exon2:c.C38A:p.P13Q,RCC1:ENST00000373832.1:exon2:c.C38A:p.P13Q,RCC1:ENST00000398958.2:exon4:c.C38A:p.P13
1	35851074	G	C	TGTGCGCTTT,CAAATCATTG	exonic	ZMYM4	nonsynonymous SNV	ZMYM4:ENST00000314607.6:exon10:c.G1601C:p.C534S,
1	38184427	C	T	TCCAGCCACG,GCCCACAGAG	exonic	EPHA10	nonsynonymous SNV	EPHA10:ENST00000427468.2:exon16:c.G2818A:p.A940T,EPHA10:ENST00000373048.4:exon16:c.G2818A:p.A940T,EPHA10:ENST00000330210.7:exon12:c.G1303A:p.A435T,
1	39798850	A	G	CCCCAAAGCA,AAAGTTACAA	exonic	MACF1	nonsynonymous SNV	MACF1:ENST00000372915.3:exon36:c.A6605G:p.K2202R,MACF1:ENST00000564288.1:exon37:c.A6590G:p.K2197R,MACF1:ENST00000289893.4:exon1:c.A1910G:p.K637R,MACF1:ENST00000567887.1:exon37:c.A6701G:p.K2234R,
1	53541638	C	G	AGAAGCCAAA,TTGAGGTCAG	exonic	PODN	nonsynonymous SNV	PODN:ENST00000371500.3:exon7:c.C663G:p.N221K,PODN:ENST00000312553.5:exon5:c.C720G:p.N240K,
1	65849879	G	T	TTGACAGGAT,GACGGGCGGC	exonic	DNAJC6	stopgain	DNAJC6:ENST00000371069.4:exon6:c.G670T:p.G224X,DNAJC6:ENST00000494710.2:exon6:c.G592T:p.G198X,DNAJC6:ENST00000263441.7:exon7:c.G460T:p.G154X,DNAJC6:ENST00000395325.3:exon6:c.G499T:p.G167X,
1	71542483	C	G	CATACCTGTT,TTTCTTCTAA	exonic	ZRANB2	nonsynonymous SNV	ZRANB2:ENST00000370920.3:exon4:c.G296C:p.R99T,ZRANB2:ENST00000254821.6:exon4:c.G296C:p.R99T,
1	79095535	A	G	AGTCAAGTCT,TTTTTCATGG	exonic	IFI44L	nonsynonymous SNV	IFI44L:ENST00000370751.5:exon4:c.A658G:p.I220V,IFI44L:ENST00000450498.1:exon3:c.A589G:p.I197V,
1	82372786	G	T	CGATGCCCGG,CAGTGATGTC	exonic	LPHN2	nonsynonymous SNV	LPHN2:ENST00000359929.3:exon4:c.G158T:p.G53V,LPHN2:ENST00000370713.1:exon3:c.G158T:p.G53V,LPHN2:ENST00000370717.2:exon3:c.G158T:p.G53V,LPHN2:ENST00000370715.1:exon3:c.G158T:p.G53V,LPHN2:ENST00000370727.1:exon6:c.G158T:p.G53V,LPHN2:ENST00000370730.1:exon6:c.G1
1	89618035	G	A	GTAAAATCTC,AACAGTCCAA	exonic	GBP7	stopgain	GBP7:ENST00000294671.2:exon5:c.C541T:p.R181X,
1	92428390	C	T	GAAAAATGGG,GATTGACAAA	exonic	BRDT	stopgain	BRDT:ENST00000423434.1:exon3:c.C79T:p.R27X,BRDT:ENST00000548992.1:exon4:c.C79T:p.R27X,BRDT:ENST00000448194.1:exon2:c.C79T:p.R27X,BRDT:ENST00000394530.3:exon2:c.C79T:p.R27X,BRDT:ENST00000440509.1:exon3:c.C79T:p.R27X,BRDT:ENST00000457265.1:exon3:c.C79T:p.R27X,BR
1	99418749	C	A	ATTGTGTATA,TGCTGACATC	exonic	LPPR5	nonsynonymous SNV	LPPR5:ENST00000370188.3:exon3:c.G498T:p.Q166H,LPPR5:ENST00000263177.4:exon3:c.G498T:p.Q166H,
1	103480128	G	T	TGGTCCTTTG,AACCATCACC	exonic	COL11A1	nonsynonymous SNV	COL11A1:ENST00000353414.4:exon12:c.C1394A:p.S465Y,COL11A1:ENST00000358392.2:exon13:c.C1547A:p.S516Y,COL11A1:ENST00000512756.1:exon11:c.C1163A:p.S388Y,COL11A1:ENST00000427239.1:exon13:c.C1547A:p.S516Y,COL11A1:ENST00000370096.3:exon13:c.C1511A:p.S504Y,
1	103487280	G	T	ACCACTGCTG,TTCTCCTTTC	exonic	COL11A1	nonsynonymous SNV	COL11A1:ENST00000353414.4:exon8:c.C1174A:p.P392T,COL11A1:ENST00000358392.2:exon9:c.C1327A:p.P443T,COL11A1:ENST00000512756.1:exon7:c.C943A:p.P315T,COL11A1:ENST00000427239.1:exon9:c.C1327A:p.P443T,COL11A1:ENST00000370096.3:exon9:c.C1291A:p.P431T,
1	115113346	C	T	TCCTTCTGTG,GTGTTCAATC	exonic	BCAS2	nonsynonymous SNV	BCAS2:ENST00000369541.3:exon5:c.G445A:p.A149T,
1	120057096	G	C	CCCTTCAACC,CCACATAGTC	exonic	HSD3B1	nonsynonymous SNV	HSD3B1:ENST00000528909.1:exon3:c.G950C:p.R317P,HSD3B1:ENST00000235547.6:exon4:c.G956C:p.R319P,HSD3B1:ENST00000369413.3:exon4:c.G950C:p.R317P,
1	152323721	T	A	CTGGACTCAC,GTGACTAGAT	exonic	FLG2	nonsynonymous SNV	FLG2:ENST00000388718.5:exon3:c.A6541T:p.S2181C,
1	152636741	A	G	CAGCTCTGGG,GCTGCTGTGG	exonic	LCE2D	nonsynonymous SNV	LCE2D:ENST00000368784.1:exon2:c.A160G:p.S54G,
1	152944487	A	G	ATGTGCACCC,AAACCAAGGA	exonic	SPRR4	nonsynonymous SNV	SPRR4:ENST00000328051.2:exon2:c.A121G:p.K41E,
1	155271120	A	C	GACTTTGCTA,GTCTCTTTGG	exonic	PKLR	nonsynonymous SNV	PKLR:ENST00000342741.4:exon1:c.T67G:p.L23V,
1	156518025	G	A	AGTGACCTTG,TGACAGCTGA	exonic	IQGAP3	nonsynonymous SNV	IQGAP3:ENST00000361170.2:exon19:c.C2144T:p.T715I,IQGAP3:ENST00000491900.1:exon18:c.C2015T:p.T672I,
1	158299228	T	G	GCCAGCCGCC,CCCCATCTGC	exonic	CD1B	nonsynonymous SNV	CD1B:ENST00000368168.3:exon4:c.A818C:p.E273A,
1	158984470	G	C	TGTCTGTAAA,ATGGGAAAAA	exonic	IFI16	nonsynonymous SNV	IFI16:ENST00000430894.2:exon2:c.G12C:p.K4N,
1	159779957	G	A	TTGGCCTGAT,GTTATTGCTG	exonic	FCRL6	nonsynonymous SNV	FCRL6:ENST00000339348.5:exon6:c.G960A:p.M320I,FCRL6:ENST00000392235.3:exon5:c.G675A:p.M225I,FCRL6:ENST00000368106.3:exon6:c.G960A:p.M320I,FCRL6:ENST00000321935.6:exon7:c.G981A:p.M327I,
1	163117231	G	T	TGTCAAAGCT,CTCAGGGAAG	exonic	RGS5	nonsynonymous SNV	RGS5:ENST00000527988.1:exon3:c.C123A:p.S41R,RGS5:ENST00000367903.3:exon6:c.C507A:p.S169R,RGS5:ENST00000313961.5:exon5:c.C447A:p.S149R,RGS5:ENST00000530507.1:exon5:c.C459A:p.S153R,
1	166960675	C	A	GAATGTTTCA,CTCCAGATAT	exonic	MAEL	nonsynonymous SNV	MAEL:ENST00000367870.2:exon2:c.C193A:p.P65T,MAEL:ENST00000447624.1:exon2:c.C193A:p.P65T,MAEL:ENST00000367872.4:exon3:c.C286A:p.P96T,
1	169697258	G	A	GAGCCTTTTG,ATCCCTTCAA	exonic	SELE	nonsynonymous SNV	SELE:ENST00000367777.1:exon7:c.C1220T:p.S407F,SELE:ENST00000333360.7:exon8:c.C1220T:p.S407F,SELE:ENST00000367782.4:exon8:c.C1220T:p.S407F,
1	176526284	C	T	GCTGCTGCGT,CAGAAGTGCT	exonic	PAPPA2	nonsynonymous SNV	PAPPA2:ENST00000367661.3:exon2:c.C826T:p.P276S,PAPPA2:ENST00000367662.3:exon2:c.C826T:p.P276S,
1	182369014	A	T	ACATGCCAGC,GAAGAAGGTG	exonic	TEDDM1	nonsynonymous SNV	TEDDM1:ENST00000367565.1:exon1:c.T607A:p.C203S,
1	197072907	G	C	AGCAGCTATA,ATTGTTGTTT	exonic	ASPM	nonsynonymous SNV	ASPM:ENST00000367409.4:exon18:c.C5474G:p.S1825C,
1	197390585	C	T	TGGCTACATT,ACTTATCAAT	exonic	CRB1	nonsynonymous SNV	CRB1:ENST00000544212.1:exon2:c.C70T:p.H24Y,CRB1:ENST00000367399.2:exon4:c.C1291T:p.H431Y,CRB1:ENST00000535699.1:exon8:c.C1420T:p.H474Y,CRB1:ENST00000367400.3:exon6:c.C1627T:p.H543Y,CRB1:ENST00000484075.1:exon6:c.C1627T:p.H543Y,CRB1:ENST00000543483.1:exon5:c.C7
1	198672464	C	A	CCACTCTGAG,CCTTCTGGAA	exonic	PTPRC	nonsynonymous SNV	PTPRC:ENST00000348564.6:exon4:c.C138A:p.S46R,PTPRC:ENST00000367367.4:exon6:c.C423A:p.S141R,PTPRC:ENST00000352140.3:exon6:c.C471A:p.S157R,PTPRC:ENST00000442510.2:exon7:c.C621A:p.S207R,PTPRC:ENST00000529828.1:exon6:c.C477A:p.S159R,PTPRC:ENST00000530727.1:exon
1	200950187	G	A	GGGGCCGTCC,TGCACCCTTG	exonic	KIF21B	nonsynonymous SNV	KIF21B:ENST00000360529.5:exon28:c.C3841T:p.R1281W,KIF21B:ENST00000332129.2:exon28:c.C3841T:p.R1281W,KIF21B:ENST00000461742.2:exon29:c.C3880T:p.R1294W,KIF21B:ENST00000422435.2:exon29:c.C3880T:p.R1294W,
1	215914862	T	A	AACATCCTAC,GCTAACTCCA	exonic	USH2A	nonsynonymous SNV	USH2A:ENST00000366943.2:exon60:c.A11566T:p.S3856C,USH2A:ENST00000307340.3:exon60:c.A11566T:p.S3856C,
1	216373209	C	A	GGCTGACCAC,AGCCAAAGGG	exonic	USH2A	nonsynonymous SNV	USH2A:ENST00000366942.3:exon17:c.G3571T:p.G1191C,USH2A:ENST00000366943.2:exon17:c.G3571T:p.G1191C,USH2A:ENST00000307340.3:exon17:c.G3571T:p.G1191C,
1	218578622	G	T	AGAGTCTTTC,TTTGCAGAAC	exonic	TGFB2	nonsynonymous SNV	TGFB2:ENST00000366930.4:exon2:c.G458T:p.R153L,TGFB2:ENST00000366929.4:exon3:c.G542T:p.R181L,
1	226340173	C	A	GAGATATGAT,CTTCTTCATG	exonic	ACBD3	nonsynonymous SNV	ACBD3:ENST00000366812.5:exon7:c.G1238T:p.G413V,
1	228464152	G	A	GCACCGCAGT,CGCCAGGTAC	exonic	OBSCN	nonsynonymous SNV	OBSCN:ENST00000570156.2:exon26:c.G7347A:p.M2449I,OBSCN:ENST00000284548.11:exon22:c.G6222A:p.M2074I,OBSCN:ENST00000422127.1:exon22:c.G6222A:p.M2074I,OBSCN:ENST00000359599.6:exon11:c.G2763A:p.M921I,
1	240256829	G	C	GGCCGACGGC,GCCTTGCGGC	exonic	FMN2	nonsynonymous SNV	FMN2:ENST00000319653.9:exon1:c.G1420C:p.G474R,
1	240370931	C	G	CTACCCGGAG,GGGAATACCT	exonic	FMN2	nonsynonymous SNV	FMN2:ENST00000319653.9:exon5:c.C2819G:p.A940G,
1	244715869	A	T	GAGACCTTTC,CACAACTGAT	exonic	C1orf101	nonsynonymous SNV	C1orf101:ENST00000366531.3:exon6:c.A329T:p.H110L,C1orf101:ENST00000366534.4:exon9:c.A782T:p.H261L,C1orf101:ENST00000366533.4:exon9:c.A782T:p.H261L,C1orf101:ENST00000428042.1:exon5:c.A542T:p.H181L,
1	244868937	G	T	CTACAGAGTT,CCTCCCGAAG	exonic	DESI2	nonsynonymous SNV	DESI2:ENST00000302550.11:exon5:c.G431T:p.C144F,DESI2:ENST00000263831.7:exon4:c.G332T:p.C111F,
1	247582124	C	A	CCGCTGCAAG,TGGCCAGGTA	exonic	NLRP3	nonsynonymous SNV	NLRP3:ENST00000336119.3:exon1:c.C28A:p.L10M,NLRP3:ENST00000366497.2:exon2:c.C28A:p.L10M,NLRP3:ENST00000348069.2:exon1:c.C28A:p.L10M,NLRP3:ENST00000366496.2:exon1:c.C28A:p.L10M,NLRP3:ENST00000391828.3:exon3:c.C28A:p.L10M,NLRP3:ENST00000391827.2:exon1:c.C28A:p.L
1	248085208	G	T	TGAGGTGAAG,GAGCCCTGAC	exonic	OR2T8	stopgain	OR2T8:ENST00000319968.4:exon1:c.G889T:p.G297X,
1	248097309	A	T	ATCGTTCCCA,AATGGTCACT	exonic	OR2AJ1	nonsynonymous SNV	OR2AJ1:ENST00000318244.3:exon1:c.A239T:p.K80I,
1	248185622	A	G	TTATGTGGCC,TTTGCTTTCC	exonic	OR2L5	nonsynonymous SNV	OR2L5:ENST00000355281.1:exon1:c.A373G:p.I125V,
2	1842980	G	T	CTGGCGTGGG,CAGGACATGC	exonic	MYT1L	stopgain	MYT1L:ENST00000407844.1:exon3:c.C9A:p.C3X,MYT1L:ENST00000428368.2:exon21:c.C3015A:p.C1005X,MYT1L:ENST00000399157.2:exon2:c.C183A:p.C61X,MYT1L:ENST00000399161.2:exon21:c.C3021A:p.C1007X,
2	1926468	C	A	GACATGCTGA,GGATGTTCAT	exonic	MYT1L	nonsynonymous SNV	MYT1L:ENST00000602387.1:exon1:c.G35T:p.R12L,MYT1L:ENST00000428368.2:exon10:c.G1073T:p.R358L,MYT1L:ENST00000399161.2:exon10:c.G1073T:p.R358L,
2	3469388	G	T	TGGCCGTGGA,GCGTATCATT	exonic	TRAPPC12	nonsynonymous SNV	TRAPPC12:ENST00000324266.5:exon9:c.G1698T:p.E566D,TRAPPC12:ENST00000382110.2:exon9:c.G1698T:p.E566D,TRAPPC12:ENST00000415624.1:exon4:c.G192T:p.E64D,
2	16082862	G	T	GGCGGGTATT,CCGCCCCAGC	exonic	MYCN	nonsynonymous SNV	MYCN:ENST00000281043.3:exon2:c.G676T:p.A226S,
2	21360734	T	A	ATACTACCAG,TGGGGAAAAA	exonic	TDRD15	nonsynonymous SNV	TDRD15:ENST00000405799.1:exon4:c.T395A:p.V132D,
2	21361591	G	T	GAAGACTGTA,GCACACAAGT	exonic	TDRD15	nonsynonymous SNV	TDRD15:ENST00000405799.1:exon4:c.G1252T:p.G418C,
2	24090746	C	A	GCCAAGCTTG,ACCCACTTTC	exonic	ATAD2B	nonsynonymous SNV	ATAD2B:ENST00000238789.5:exon10:c.G1147T:p.A383S,
2	27360502	A	C	GGTGCTGCAA,AGCAGTAGCC	exonic	C2orf53	nonsynonymous SNV	C2orf53:ENST00000432962.1:exon4:c.T202G:p.F68V,
2	31409336	C	T	AGTTGAACTG,ATTAATCTCT	exonic	CAPN14	nonsynonymous SNV	CAPN14:ENST00000403897.3:exon16:c.G1603A:p.A535T,CAPN14:ENST00000444918.2:exon16:c.G1603A:p.A535T,
2	89266034	A	C	AGATGCAGAC,GGGAGGATGG	exonic	IGKV1-6	nonsynonymous SNV	IGKV1-6:ENST00000464162.1:exon2:c.T98G:p.L33R,
2	97482267	G	T	GGAGGGGGCG,GCTGCCGGGA	exonic	CNNM3	nonsynonymous SNV	CNNM3:ENST00000305510.3:exon1:c.G253T:p.G85C,CNNM3:ENST00000377060.3:exon1:c.G253T:p.G85C,
2	103334932	G	C	CGCTTGTTTA,AGACATTATG	exonic	MFSD9	nonsynonymous SNV	MFSD9:ENST00000258436.5:exon6:c.C1372G:p.L458V,
2	105889413	C	A	GGCGGAGGCC,TCTGCAGCAG	exonic	TGFBRAP1	nonsynonymous SNV	TGFBRAP1:ENST00000258449.1:exon9:c.G1871T:p.R624M,TGFBRAP1:ENST00000393359.2:exon10:c.G1871T:p.R624M,
2	130897660	G	A	AGGGACTTGA,GTGCTGCAGC	exonic	CCDC74B	nonsynonymous SNV	CCDC74B:ENST00000409943.3:exon6:c.C688T:p.L230F,CCDC74B:ENST00000310463.6:exon6:c.C886T:p.L296F,CCDC74B:ENST00000392984.3:exon5:c.C1192T:p.L398F,
2	133402778	G	A	CTACTTCCGG,CGTACATGAT	exonic	GPR39	nonsynonymous SNV	GPR39:ENST00000329321.3:exon2:c.G961A:p.A321T,
2	152487834	C	G	TGCCTCTCAG,CACTGGAGAT	exonic	NEB	nonsynonymous SNV	NEB:ENST00000603639.1:exon64:c.G9441C:p.W3147C,NEB:ENST00000397345.3:exon66:c.G9441C:p.W3147C,NEB:ENST00000427231.2:exon66:c.G9441C:p.W3147C,NEB:ENST00000604864.1:exon64:c.G9441C:p.W3147C,
2	153527790	T	A	AGAATACCTT,TCTTTTTTTG	exonic	PRPF40A	nonsynonymous SNV	PRPF40A:ENST00000410080.1:exon14:c.A1476T:p.E492D,
2	163055283	A	T	AGACAAGTGC,TAGTACTTGG	exonic	FAP	stopgain	FAP:ENST00000443424.1:exon15:c.T1311A:p.Y437X,FAP:ENST00000422436.1:exon1:c.T54A:p.Y18X,FAP:ENST00000188790.4:exon16:c.T1386A:p.Y462X,
2	176948160	G	T	CCACGTCGCT,CTCATGTCGG	exonic	EVX2	nonsynonymous SNV	EVX2:ENST00000308618.4:exon1:c.C345A:p.S115R,
2	178740618	C	G	ACTTTGGGGA,ATCAATTCAA	exonic	PDE11A	nonsynonymous SNV	PDE11A:ENST00000409504.1:exon4:c.G261C:p.M87I,PDE11A:ENST00000449286.2:exon4:c.G261C:p.M87I,PDE11A:ENST00000358450.4:exon6:c.G585C:p.M195I,PDE11A:ENST00000286063.6:exon5:c.G1335C:p.M445I,PDE11A:ENST00000389683.3:exon2:c.G3C:p.M1I,
2	179401752	A	T	CGGAAGTAAT,GCCAGCATTT	exonic	TTN	nonsynonymous SNV	TTN:ENST00000589042.1:exon356:c.T100084A:p.Y33362N,TTN:ENST00000460472.2:exon184:c.T72889A:p.Y24297N,TTN:ENST00000342175.6:exon185:c.T73465A:p.Y24489N,TTN:ENST00000359218.5:exon185:c.T73264A:p.Y24422N,TTN:ENST00000342992.6:exon305:c.T92380A:p.Y30794N,TTN:ENST0
2	179452665	C	A	TTTAGCTCTG,AGGGCGCCCA	exonic	TTN	nonsynonymous SNV	TTN:ENST00000589042.1:exon305:c.G63469T:p.A21157S,TTN:ENST00000460472.2:exon133:c.G36274T:p.A12092S,TTN:ENST00000342175.6:exon134:c.G36850T:p.A12284S,TTN:ENST00000359218.5:exon134:c.G36649T:p.A12217S,TTN:ENST00000342992.6:exon254:c.G55765T:p.A18589S,TTN:ENST0
2	179455185	T	C	AGTTCATCTT,ATTAATCCTG	exonic	TTN	nonsynonymous SNV	TTN:ENST00000589042.1:exon304:c.A61267G:p.K20423E,TTN:ENST00000460472.2:exon132:c.A34072G:p.K11358E,TTN:ENST00000342175.6:exon133:c.A34648G:p.K11550E,TTN:ENST00000359218.5:exon133:c.A34447G:p.K11483E,TTN:ENST00000342992.6:exon253:c.A53563G:p.K17855E,TTN:ENST0
2	189927933	C	T	CCCATGCTCC,GGGCTGCCCT	exonic	COL5A2	nonsynonymous SNV	COL5A2:ENST00000374866.3:exon27:c.G1834A:p.G612R,
2	190719359	G	A	ACGGAATATA,TAAAACTTGT	exonic	PMS1	nonsynonymous SNV	PMS1:ENST00000418224.3:exon8:c.G833A:p.S278N,PMS1:ENST00000441310.2:exon9:c.G1361A:p.S454N,PMS1:ENST00000432292.3:exon7:c.G833A:p.S278N,PMS1:ENST00000409593.1:exon4:c.G716A:p.S239N,PMS1:ENST00000450931.1:exon9:c.G1244A:p.S415N,PMS1:ENST00000409823.3:exon8:c.
2	198570409	G	T	TGGTACCGAC,AGCACGGGCT	exonic	MARS2	stopgain	MARS2:ENST00000282276.6:exon1:c.G280T:p.E94X,
2	201438572	A	T	TCTTCTGGTA,AAATGTGATA	exonic	SGOL2	nonsynonymous SNV	SGOL2:ENST00000357799.4:exon7:c.A3503T:p.K1168I,
2	202632036	T	A	CTCTCTGGTG,TATGGGAAAG	exonic	ALS2	nonsynonymous SNV	ALS2:ENST00000467448.1:exon3:c.A91T:p.T31S,ALS2:ENST00000410052.1:exon4:c.A91T:p.T31S,ALS2:ENST00000409632.2:exon4:c.A91T:p.T31S,ALS2:ENST00000264276.6:exon3:c.A91T:p.T31S,
2	205978244	G	T	GAGATTTCTG,GGAAGGAGGC	exonic	PARD3B	nonsynonymous SNV	PARD3B:ENST00000351153.1:exon6:c.G623T:p.G208V,PARD3B:ENST00000406610.2:exon6:c.G623T:p.G208V,PARD3B:ENST00000462231.1:exon6:c.G623T:p.G208V,PARD3B:ENST00000349953.3:exon6:c.G623T:p.G208V,PARD3B:ENST00000358768.2:exon6:c.G623T:p.G208V,
2	207414866	T	G	AACTTATGAT,GTTAATGATC	exonic	ADAM23	nonsynonymous SNV	ADAM23:ENST00000264377.3:exon9:c.T915G:p.I305M,ADAM23:ENST00000374415.3:exon9:c.T915G:p.I305M,ADAM23:ENST00000374416.1:exon9:c.T915G:p.I305M,
2	212426794	C	G	ATGATCCATA,TTGCCATGAT	exonic	ERBB4	nonsynonymous SNV	ERBB4:ENST00000402597.1:exon20:c.G2291C:p.S764T,ERBB4:ENST00000342788.4:exon20:c.G2321C:p.S774T,ERBB4:ENST00000436443.1:exon20:c.G2321C:p.S774T,
2	219870770	G	T	GGGAAACTCT,AGAAGAAGTT	exonic	CCDC108	stopgain	CCDC108:ENST00000341552.5:exon31:c.C4895A:p.S1632X,CCDC108:ENST00000453220.1:exon29:c.C4895A:p.S1632X,CCDC108:ENST00000441968.1:exon30:c.C4895A:p.S1632X,
2	220336997	C	T	GTGGCTGTGA,GGGGAGGATG	exonic	SPEG	nonsynonymous SNV	SPEG:ENST00000312358.7:exon15:c.C3884T:p.T1295M,
2	226446875	A	T	GTACATCGAG,TGGTGGGGAA	exonic	NYAP2	nonsynonymous SNV	NYAP2:ENST00000272907.6:exon4:c.A742T:p.M248L,
2	234526477	C	A	GTTCACCATG,AGTCGGTGGT	exonic	UGT1A1	nonsynonymous SNV	UGT1A1:ENST00000373450.4:exon1:c.C124A:p.Q42K,
2	234545199	C	T	CAGCCCCGTT,CTTTATGTGT	exonic	UGT1A10	nonsynonymous SNV	UGT1A10:ENST00000344644.5:exon1:c.C31T:p.P11S,UGT1A10:ENST00000373445.1:exon1:c.C31T:p.P11S,
2	238283535	C	T	ACCACGGCCA,GCGGACCCGG	exonic	COL6A3	nonsynonymous SNV	COL6A3:ENST00000472056.1:exon5:c.G1378A:p.V460M,COL6A3:ENST00000392004.3:exon7:c.G2581A:p.V861M,COL6A3:ENST00000392003.2:exon6:c.G1978A:p.V660M,COL6A3:ENST00000409809.1:exon6:c.G2581A:p.V861M,COL6A3:ENST00000346358.4:exon7:c.G2599A:p.V867M,COL6A3:ENST0000
2	239072806	G	T	CTACGCTCTC,CCGCCACGCT	exonic	FAM132B	nonsynonymous SNV	FAM132B:ENST00000546354.1:exon5:c.G778T:p.A260S,
3	9482359	A	G	CTGTAATAAC,CAGTTATTGG	exonic	SETD5	nonsynonymous SNV	SETD5:ENST00000402466.1:exon9:c.A493G:p.T165A,SETD5:ENST00000407969.1:exon6:c.A844G:p.T282A,SETD5:ENST00000406341.1:exon7:c.A787G:p.T263A,SETD5:ENST00000302463.6:exon7:c.A493G:p.T165A,SETD5:ENST00000402198.1:exon8:c.A787G:p.T263A,SETD5:ENST00000442373.1:exon6
3	16336457	G	A	GATAGAATTG,CAGTGAAATA	exonic	OXNAD1	nonsynonymous SNV	OXNAD1:ENST00000285083.5:exon6:c.G385A:p.A129T,OXNAD1:ENST00000435829.2:exon6:c.G439A:p.A147T,OXNAD1:ENST00000544043.1:exon5:c.G439A:p.A147T,OXNAD1:ENST00000605932.1:exon6:c.G385A:p.A129T,OXNAD1:ENST00000606098.1:exon6:c.G385A:p.A129T,
3	42738571	T	C	TGCCACAGTG,TGACAACACC	exonic	HHATL	nonsynonymous SNV	HHATL:ENST00000441594.1:exon8:c.A932G:p.N311S,HHATL:ENST00000310417.5:exon9:c.A932G:p.N311S,HHATL:ENST00000457462.1:exon7:c.A737G:p.N246S,
3	73673416	C	G	CGCGCAGCGC,TCCTTCTTGA	exonic	PDZRN3	nonsynonymous SNV	PDZRN3:ENST00000308537.4:exon1:c.G561C:p.E187D,PDZRN3:ENST00000263666.4:exon1:c.G561C:p.E187D,
3	74414730	G	A	TAGCACCAGG,CTGCTCCATT	exonic	CNTN3	nonsynonymous SNV	CNTN3:ENST00000263665.6:exon8:c.C1070T:p.A357V,
3	93761946	G	C	AATGGAGCAT,AGCAAATAGA	exonic	ARL13B	nonsynonymous SNV	ARL13B:ENST00000394222.3:exon7:c.G886C:p.E296Q,ARL13B:ENST00000535334.1:exon6:c.G577C:p.E193Q,ARL13B:ENST00000539730.1:exon6:c.G49C:p.E17Q,ARL13B:ENST00000471138.1:exon7:c.G886C:p.E296Q,ARL13B:ENST00000303097.7:exon5:c.G565C:p.E189Q,
3	96533658	A	G	GAGGAAGAAG,CGTGGACAAG	exonic	EPHA6	nonsynonymous SNV	EPHA6:ENST00000470610.2:exon1:c.A191G:p.D64G,EPHA6:ENST00000389672.5:exon1:c.A191G:p.D64G,
3	97888095	G	C	CTATCCCATT,TCTAAGATTT	exonic	OR5H15	nonsynonymous SNV	OR5H15:ENST00000356526.2:exon1:c.G552C:p.L184F,
3	99568371	T	C	TCCACTTCTC,TGAGAGGTGC	exonic	FILIP1L	nonsynonymous SNV	FILIP1L:ENST00000331335.5:exon5:c.A2149G:p.R717G,FILIP1L:ENST00000495625.2:exon3:c.A1429G:p.R477G,FILIP1L:ENST00000487087.1:exon2:c.A877G:p.R293G,FILIP1L:ENST00000471562.1:exon2:c.A1429G:p.R477G,FILIP1L:ENST00000354552.3:exon5:c.A2149G:p.R717G,FILIP1L:ENST000003836
3	111899464	C	G	TCACCTTCTT,AAATATATCA	exonic	SLC9C1	nonsynonymous SNV	SLC9C1:ENST00000487372.1:exon21:c.G2551C:p.E851Q,SLC9C1:ENST00000305815.5:exon22:c.G2695C:p.E899Q,
3	113172600	C	A	ACAAAAGGAG,CTGAGTTTTG	exonic	SPICE1	nonsynonymous SNV	SPICE1:ENST00000295872.4:exon14:c.G1855T:p.A619S,
3	115342547	G	A	ATGCTGTGCT,TATGAGAAGA	exonic	GAP43	nonsynonymous SNV	GAP43:ENST00000305124.6:exon1:c.G11A:p.C4Y,
3	128720481	G	T	GATGGAGCCG,TCAGCACGGG	exonic	EFCC1	nonsynonymous SNV	EFCC1:ENST00000480450.1:exon1:c.G10T:p.V4F,
3	130145247	C	A	GGAACATAGT,GTGAGTATCT	exonic	COL6A5	nonsynonymous SNV	COL6A5:ENST00000312481.7:exon31:c.C5236A:p.P1746T,COL6A5:ENST00000265379.6:exon31:c.C5236A:p.L1746I,COL6A5:ENST00000432398.2:exon31:c.C5236A:p.L1746I,
3	133748558	T	C	CACCTTAATG,TGCCGAAGAC	exonic	SLCO2A1	nonsynonymous SNV	SLCO2A1:ENST00000493729.1:exon1:c.A89G:p.N30S,SLCO2A1:ENST00000481359.3:exon1:c.A89G:p.N30S,SLCO2A1:ENST00000310926.4:exon1:c.A89G:p.N30S,
3	134670233	C	A	TCAGTGGCTA,GATGAAAACC	exonic	EPHB1	stopgain	EPHB1:ENST00000473867.1:exon5:c.C78A:p.Y26X,EPHB1:ENST00000497173.1:exon3:c.C78A:p.Y26X,EPHB1:ENST00000482618.1:exon3:c.C144A:p.Y48X,EPHB1:ENST00000398015.3:exon3:c.C144A:p.Y48X,EPHB1:ENST00000474732.1:exon3:c.C78A:p.Y26X,EPHB1:ENST00000460895.1:exon4:c.C78A:p
3	134670267	G	T	CACCTACCAG,TGTGCAATGT	exonic	EPHB1	nonsynonymous SNV	EPHB1:ENST00000473867.1:exon5:c.G112T:p.V38L,EPHB1:ENST00000497173.1:exon3:c.G112T:p.V38L,EPHB1:ENST00000482618.1:exon3:c.G178T:p.V60L,EPHB1:ENST00000398015.3:exon3:c.G178T:p.V60L,EPHB1:ENST00000474732.1:exon3:c.G112T:p.V38L,EPHB1:ENST00000460895.1:exon4:c.G11
3	138669358	G	A	CCTCTGCTTG,AGGGATGGCC	exonic	C3orf72	nonsynonymous SNV	C3orf72:ENST00000383165.3:exon3:c.G472A:p.E158K,
3	140123418	G	T	TCCATATACA,GTGAAGGATG	exonic	CLSTN2	nonsynonymous SNV	CLSTN2:ENST00000458420.3:exon4:c.G447T:p.Q149H,
3	147113753	C	A	ATGTGATTTA,AAGTTTGTAT	exonic	ZIC4	nonsynonymous SNV	ZIC4:ENST00000462748.2:exon3:c.G574T:p.V192L,ZIC4:ENST00000425731.3:exon3:c.G688T:p.V230L,ZIC4:ENST00000484399.1:exon3:c.G574T:p.V192L,ZIC4:ENST00000473123.1:exon3:c.G574T:p.V192L,ZIC4:ENST00000383075.3:exon3:c.G574T:p.V192L,ZIC4:ENST00000525172.2:exon3:c.G724T:p.V242L,
3	148601450	C	A	GGGCTCTGCA,CAGAGTCCGA	exonic	CPA3	nonsynonymous SNV	CPA3:ENST00000296046.3:exon9:c.C829A:p.P277T,
3	148768072	A	G	TCCTGTTTTG,AAGTAAGGCC	exonic	HLTF	nonsynonymous SNV	HLTF:ENST00000465259.1:exon15:c.T1561C:p.S521P,HLTF:ENST00000392912.2:exon15:c.T1564C:p.S522P,HLTF:ENST00000494055.1:exon15:c.T1564C:p.S522P,HLTF:ENST00000310053.5:exon15:c.T1564C:p.S522P,
3	150285688	G	A	ATGTATCTTA,GTGGCTGTCT	splicing	EIF2A(ENST(	.	.
3	155199773	C	A	AGGCTGCTCT,CCCAGAATTG	exonic	PLCH1	stopgain	PLCH1:ENST00000334686.6:exon22:c.G3952T:p.E1318X,PLCH1:ENST00000414191.1:exon22:c.G3952T:p.E1318X,PLCH1:ENST00000460012.1:exon23:c.G3952T:p.E1318X,PLCH1:ENST00000340059.7:exon23:c.G4066T:p.E1356X,
3	161214944	G	C	AGAACCTGGA,AGACTGGACA	exonic	OTOL1	nonsynonymous SNV	OTOL1:ENST00000327928.4:exon1:c.G349C:p.E117Q,
3	164709174	G	T	CTCTTGACAT,GTAGGATGTG	exonic	SI	nonsynonymous SNV	SI:ENST00000264382.3:exon44:c.C5075A:p.P1692Q,
3	164716460	C	T	TTCTGCAATG,ACTAATATAG	exonic	SI	nonsynonymous SNV	SI:ENST00000264382.3:exon38:c.G4408A:p.A1470T,
3	164766991	C	G	TGCACAGCAT,CATGCAAATT	exonic	SI	nonsynonymous SNV	SI:ENST00000264382.3:exon15:c.G1639C:p.D547H,
3	164907347	G	T	AATCAGAACG,TATATTTTCT	exonic	SLITRK3	stopgain	SLITRK3:ENST00000241274.3:exon2:c.C1272A:p.Y424X,SLITRK3:ENST00000475390.1:exon2:c.C1272A:p.Y424X,
3	184037489	A	G	GGCGCCTACT,TCCAGCCCAA	exonic	EIF4G1	nonsynonymous SNV	EIF4G1:ENST00000456033.1:exon5:c.A287G:p.Y96C,EIF4G1:ENST00000427141.2:exon8:c.A176G:p.Y59C,EIF4G1:ENST00000411531.1:exon4:c.A317G:p.Y106C,EIF4G1:ENST00000450424.1:exon6:c.A437G:p.Y146C,EIF4G1:ENST00000382330.3:exon8:c.A458G:p.Y153C,EIF4G1:ENST00000414031.1:exon
3	184049288	C	A	GGAGAGGAGT,GGAAGCCCCT	exonic	EIF4G1	stopgain	EIF4G1:ENST00000411531.1:exon27:c.C4172A:p.S1391X,EIF4G1:ENST00000382330.3:exon31:c.C4310A:p.S1437X,EIF4G1:ENST00000434061.2:exon23:c.C3704A:p.S1235X,EIF4G1:ENST00000414031.1:exon30:c.C4169A:p.S1390X,EIF4G1:ENST00000346169.2:exon30:c.C4289A:p.S1430X,EIF4G1:ENST
3	185905997	C	A	TTACCTTGAA,GCAGAATTTC	exonic	DGKG	nonsynonymous SNV	DGKG:ENST00000544847.1:exon20:c.G1912T:p.V638F,DGKG:ENST00000265022.3:exon22:c.G2089T:p.V697F,DGKG:ENST00000344484.4:exon21:c.G2014T:p.V672F,DGKG:ENST00000382164.4:exon21:c.G1972T:p.V658F,
3	192125840	C	A	CGGCCTCTTG,GGCCGCTGCA	exonic	FGF12	nonsynonymous SNV	FGF12:ENST00000454309.2:exon1:c.G173T:p.R58L,
3	195481222	C	T	AAGGTGCCCC,GTCACACTTG	exonic	MUC4	nonsynonymous SNV	MUC4:ENST00000349607.4:exon17:c.G2329A:p.G777R,MUC4:ENST00000463781.3:exon19:c.G15190A:p.G5064R,MUC4:ENST00000475231.1:exon18:c.G15034A:p.G5012R,MUC4:ENST00000346145.4:exon18:c.G2482A:p.G828R,
4	10079005	T	A	GGAGGCAAAG,GTTCATTGTC	exonic	WDR1	nonsynonymous SNV	WDR1:ENST00000499869.2:exon14:c.A1637T:p.H546L,WDR1:ENST00000382451.2:exon11:c.A1217T:p.H406L,WDR1:ENST00000382452.2:exon14:c.A1637T:p.H546L,WDR1:ENST00000502702.1:exon11:c.A1217T:p.H406L,
4	20618688	C	G	TGGCTGTGAG,CATGCCACAA	exonic	SLIT2	nonsynonymous SNV	SLIT2:ENST00000273739.5:exon37:c.C4042G:p.P1348A,SLIT2:ENST00000504154.1:exon35:c.C4003G:p.P1335A,SLIT2:ENST00000503837.1:exon35:c.C3991G:p.P1331A,SLIT2:ENST00000503823.1:exon34:c.C3979G:p.P1327A,
4	48493080	G	A	GCAGCCGGCC,ACGGTCCCGA	exonic	ZAR1	nonsynonymous SNV	ZAR1:ENST00000327939.4:exon1:c.G772A:p.D258N,
4	48625145	G	C	TGTCATACCT,ATCAAACTGA	exonic	FRYL	nonsynonymous SNV	FRYL:ENST00000505759.1:exon3:c.C448G:p.Q150E,FRYL:ENST00000503238.1:exon2:c.C172G:p.Q58E,FRYL:ENST00000507711.1:exon5:c.C172G:p.Q58E,FRYL:ENST00000358350.4:exon5:c.C172G:p.Q58E,FRYL:ENST00000537810.1:exon5:c.C172G:p.Q58E,
4	66361168	G	C	ACAGACACAA,AGGTTGAAGC	exonic	EPHA5	nonsynonymous SNV	EPHA5:ENST00000273854.3:exon4:c.C1004G:p.S335C,EPHA5:ENST00000511294.1:exon4:c.C1004G:p.S335C,EPHA5:ENST00000354839.4:exon4:c.C1004G:p.S335C,
4	73169785	T	A	GCCTGTAGCC,GGTTCTTAAT	exonic	ADAMTS3	nonsynonymous SNV	ADAMTS3:ENST00000286657.4:exon17:c.A2273T:p.Q758L,
4	75178018	T	G	GAAAGCCATC,GCAGGTAAAT	exonic	EPGN	nonsynonymous SNV	EPGN:ENST00000514968.1:exon3:c.T223G:p.C75G,EPGN:ENST00000332112.4:exon3:c.T223G:p.C75G,EPGN:ENST00000509145.1:exon2:c.T160G:p.C54G,EPGN:ENST00000505212.1:exon3:c.T223G:p.C75G,EPGN:ENST00000413830.1:exon3:c.T250G:p.C84G,EPGN:ENST00000502358.1:exon3:c.T250G
4	81966915	T	C	AAAAGGACTG,ATATCTTCAA	exonic	BMP3	nonsynonymous SNV	BMP3:ENST00000282701.2:exon2:c.T340C:p.Y114H,
4	104510940	C	T	AAACTTGGGT,TCTTGGCGTT	exonic	TACR3	nonsynonymous SNV	TACR3:ENST00000304883.2:exon5:c.G1297A:p.D433N,
4	109088721	T	C	CTCGTGTCCG,TGCTGGCCGG	exonic	LEF1	nonsynonymous SNV	LEF1:ENST00000506680.1:exon1:c.A203G:p.N68S,LEF1:ENST00000438313.2:exon1:c.A203G:p.N68S,LEF1:ENST00000379951.2:exon1:c.A203G:p.N68S,LEF1:ENST00000265165.1:exon1:c.A203G:p.N68S,
4	114275566	G	T	GTATCGCCTG,GAGAACAGAA	exonic	ANK2	nonsynonymous SNV	ANK2:ENST00000264366.6:exon37:c.G5693T:p.G1898V,ANK2:ENST00000357077.4:exon38:c.G5792T:p.G1931V,
4	119631356	G	A	AACTTCTGCT,GCCGTGGACG	exonic	METTL14	nonsynonymous SNV	METTL14:ENST00000388822.5:exon11:c.G1270A:p.G424S,METTL14:ENST00000506780.1:exon11:c.G1156A:p.G386S,
4	134073905	C	A	TCATCTCCAA,GGAAGCATTT	exonic	PCDH10	nonsynonymous SNV	PCDH10:ENST00000264360.5:exon1:c.C2610A:p.N870K,
4	144617670	A	T	GTGTGGATAT,GCAGATGAGG	exonic	FREM3	nonsynonymous SNV	FREM3:ENST00000329798.5:exon1:c.T4159A:p.Y1387N,
4	147561434	T	A	GCGCTCAGCA,GGCCCACGCG	exonic	POU4F2	nonsynonymous SNV	POU4F2:ENST00000281321.3:exon2:c.T704A:p.M235K,
4	152640638	T	A	TATGTGGCAG,TCAGAGCCAG	exonic	PET112	nonsynonymous SNV	PET112:ENST00000511538.1:exon3:c.A368T:p.N123I,PET112:ENST00000512306.1:exon3:c.A380T:p.N127I,PET112:ENST00000263985.6:exon3:c.A380T:p.N127I,PET112:ENST00000515812.1:exon3:c.A380T:p.N127I,
4	156631897	G	C	TCTATGCCTG,ATAAGGAGGA	exonic	GUCY1A3	nonsynonymous SNV	GUCY1A3:ENST00000513574.1:exon5:c.G580C:p.D194H,GUCY1A3:ENST00000506455.1:exon6:c.G580C:p.D194H,GUCY1A3:ENST00000511507.1:exon6:c.G580C:p.D194H,GUCY1A3:ENST00000296518.7:exon6:c.G580C:p.D194H,GUCY1A3:ENST00000511108.1:exon7:c.G580C:p.D194H,GUCY1A3:ENST
4	159791505	C	T	GAACTTTGGC,GCTCACTTCT	exonic	FNIP2	nonsynonymous SNV	FNIP2:ENST00000379346.3:exon14:c.C2902T:p.R968C,FNIP2:ENST00000264433.6:exon14:c.C2833T:p.R945C,
4	170322874	T	G	ATCTGTCCCA,TTGCAGTTGG	exonic	NEK1	nonsynonymous SNV	NEK1:ENST00000512193.1:exon30:c.A3221C:p.N1074T,NEK1:ENST00000511633.1:exon32:c.A3380C:p.N1127T,NEK1:ENST00000507142.1:exon33:c.A3512C:p.N1171T,NEK1:ENST00000439128.2:exon31:c.A3428C:p.N1143T,NEK1:ENST00000510533.1:exon30:c.A3296C:p.N1099T,
4	177041224	G	C	TCATATTGAT,GAAGTCTATC	exonic	WDR17	nonsynonymous SNV	WDR17:ENST00000508596.1:exon4:c.G514C:p.G172R,WDR17:ENST00000393643.2:exon4:c.G514C:p.G172R,WDR17:ENST00000280190.4:exon5:c.G586C:p.G196R,WDR17:ENST00000507824.2:exon4:c.G586C:p.G196R,

5	1432603	G	C	AGCAGGTGTG,TCCCAAAAGT	exonic	SLC6A3	nonsynonymous SNV	SLC6A3:ENST00000453492.2:exon3:c.C629G:p.T210S,SLC6A3:ENST00000513308.1:exon3:c.C407G:p.T136S,SLC6A3:ENST00000270349.9:exon4:c.C629G:p.T210S,
5	5235182	A	G	GAAGCTCTGC,ACAGTCAGAA	exonic	ADAMTS16	nonsynonymous SNV	ADAMTS16:ENST00000274181.7:exon13:c.A1906G:p.N636D,
5	21802441	G	A	GTCTTTGAAA,GGCCCGCCGA	exonic	CDH12	nonsynonymous SNV	CDH12:ENST00000504376.2:exon9:c.C1091T:p.P364L,CDH12:ENST00000522262.1:exon7:c.C971T:p.P324L,CDH12:ENST00000382254.1:exon10:c.C1091T:p.P364L,
5	38350682	G	T	GGCAAAGGGC,GCTGAGCTCT	exonic	EGFLAM	nonsynonymous SNV	EGFLAM:ENST00000322350.5:exon4:c.G371T:p.R124L,EGFLAM:ENST00000354891.3:exon4:c.G371T:p.R124L,
5	39390585	T	G	GATGCTGGCC,TCTCCTCCAC	exonic	DAB2	nonsynonymous SNV	DAB2:ENST00000509337.1:exon4:c.A423C:p.E141D,DAB2:ENST00000545653.1:exon5:c.A423C:p.E141D,DAB2:ENST00000320816.6:exon5:c.A423C:p.E141D,DAB2:ENST00000339788.6:exon5:c.A423C:p.E141D,
5	41054913	T	A	GTTCTTTCTA,TGAAATGATG	exonic	MROH2B	nonsynonymous SNV	MROH2B:ENST00000399564.4:exon11:c.A1063T:p.I355L,
5	54573046	C	T	TAGATCAGAA,GTTTCTGTAA	exonic	DHX29	nonsynonymous SNV	DHX29:ENST00000251636.5:exon13:c.G2174A:p.R725H,
5	63256511	T	C	ATGCCCAGCG,CTTCACTGTC	exonic	HTR1A	nonsynonymous SNV	HTR1A:ENST00000323865.3:exon1:c.A1036G:p.T346A,
5	76708023	T	A	GAGTTGGGAC,TCAACATCTT	exonic	PDE8B	nonsynonymous SNV	PDE8B:ENST00000505283.1:exon3:c.T70A:p.F24I,PDE8B:ENST00000342343.4:exon15:c.T1615A:p.F539I,PDE8B:ENST00000264917.5:exon16:c.T1675A:p.F559I,PDE8B:ENST00000333194.4:exon15:c.T1510A:p.F504I,PDE8B:ENST00000346042.3:exon13:c.T1384A:p.F462I,PDE8B:ENST00000340978.3:exo
5	94826640	C	G	ATAACAGTGC,AAGTATAAAT	exonic	TTC37	nonsynonymous SNV	TTC37:ENST00000358746.2:exon37:c.G3903C:p.L1301F,
5	102526563	G	C	GTATTCCATG,TGCCATCTAT	exonic	PPIP5K2	nonsynonymous SNV	PPIP5K2:ENST00000414217.1:exon27:c.G3310C:p.V1104L,PPIP5K2:ENST00000358359.3:exon29:c.G3373C:p.V1125L,PPIP5K2:ENST00000321521.9:exon28:c.G3310C:p.V1104L,PPIP5K2:ENST00000509597.1:exon8:c.G838C:p.V280L,
5	127448596	G	A	TAAAGGAGTC,TGAAGTTTGG	exonic	SLC12A2	nonsynonymous SNV	SLC12A2:ENST00000509205.1:exon2:c.G847A:p.V283M,SLC12A2:ENST00000343225.4:exon2:c.G847A:p.V283M,SLC12A2:ENST00000262461.2:exon2:c.G847A:p.V283M,
5	128448660	G	T	ATTAAGGCGA,TGCTCCAGAG	exonic	ISOC1	nonsynonymous SNV	ISOC1:ENST00000173527.5:exon5:c.G863T:p.S288I,
5	131944366	A	T	AGCAAGAAAA,GAAGAATTAA	exonic	RAD50	nonsynonymous SNV	RAD50:ENST00000378823.3:exon17:c.A2361T:p.K787N,RAD50:ENST00000265335.6:exon17:c.A2778T:p.K926N,
5	140811257	G	T	GGACCACGAG,AGTCAGGATT	exonic	PCDHGA12	stopgain	PCDHGA12:ENST00000252085.3:exon1:c.G931T:p.E311X,
5	149215841	C	G	ACCCCACCCA,CACACCACCG	exonic	PPARGC1B	nonsynonymous SNV	PPARGC1B:ENST00000394320.3:exon8:c.C1823G:p.T608S,PPARGC1B:ENST00000403750.1:exon7:c.C1631G:p.T544S,PPARGC1B:ENST00000360453.4:exon7:c.C1706G:p.T569S,PPARGC1B:ENST00000309241.5:exon8:c.C1823G:p.T608S,
5	150275911	T	C	ACCACAATCA,ATGGTTTCTT	exonic	ZNF300	nonsynonymous SNV	ZNF300:ENST00000274599.5:exon6:c.A890G:p.Y297C,ZNF300:ENST00000446148.2:exon7:c.A938G:p.Y313C,ZNF300:ENST00000394226.2:exon6:c.A890G:p.Y297C,ZNF300:ENST00000418587.2:exon5:c.A782G:p.Y261C,
5	150911459	C	A	GCGGATCACC,CCGTGGTGGC	exonic	FAT2	nonsynonymous SNV	FAT2:ENST00000261800.5:exon13:c.G9500T:p.G3167V,
5	162896669	G	A	CTACAGGAAC,TGGTGCCCAG	exonic	HMMR	nonsynonymous SNV	HMMR:ENST00000393915.4:exon5:c.G296A:p.R99H,HMMR:ENST00000432118.2:exon2:c.G35A:p.R12H,HMMR:ENST00000358715.3:exon5:c.G293A:p.R98H,HMMR:ENST00000353866.3:exon4:c.G248A:p.R83H,
5	169018103	C	G	ACTCAAGAGT,GAATGTTAGA	exonic	SPDL1	nonsynonymous SNV	SPDL1:ENST00000513795.1:exon3:c.C211G:p.R71G,SPDL1:ENST00000265295.4:exon3:c.C211G:p.R71G,SPDL1:ENST00000515224.1:exon4:c.C211G:p.R71G,SPDL1:ENST00000506574.1:exon4:c.C211G:p.R71G,SPDL1:ENST00000513941.1:exon4:c.C211G:p.R71G,SPDL1:ENST00000508247.1:exon3:c.C
6	401663	C	G	TGCGAAAAGA,TGTGCCAGAG	exonic	IRF4	nonsynonymous SNV	IRF4:ENST00000380956.4:exon7:c.C985G:p.L329V,IRF4:ENST00000493114.1:exon7:c.C985G:p.L329V,
6	1390350	C	A	CCGCCTCCTG,GCCTCGTCCT	exonic	FOXF2	stopgain	FOXF2:ENST00000259806.1:exon1:c.C168A:p.C56X,
6	24828487	G	T	CAGAGGCTCA,CCCGGTCCAG	exonic	FAM65B	nonsynonymous SNV	FAM65B:ENST00000538035.1:exon18:c.C2543A:p.A848D,FAM65B:ENST00000259698.4:exon19:c.C2606A:p.A869D,
6	26390248	G	C	TTTATGCCCA,CGCATCTCCC	exonic	BTN2A2	nonsynonymous SNV	BTN2A2:ENST00000490025.1:exon3:c.G125C:p.S42T,BTN2A2:ENST00000469230.1:exon5:c.G740C:p.S247T,BTN2A2:ENST00000416795.2:exon5:c.G740C:p.S247T,BTN2A2:ENST00000482536.1:exon3:c.G110C:p.S37T,BTN2A2:ENST00000356709.4:exon5:c.G740C:p.S247T,BTN2A2:ENST00000352867.2:
6	29141668	C	A	GCTGGTGAAT,TCCGGGGCCC	exonic	OR2J2	nonsynonymous SNV	OR2J2:ENST00000377167.2:exon1:c.C256A:p.L86I,
6	29394596	A	G	AGCAGGGAGA,GACCTTGGAG	exonic	OR11A1	nonsynonymous SNV	OR11A1:ENST00000377149.1:exon5:c.T823C:p.F275L,OR11A1:ENST00000377148.1:exon2:c.T823C:p.F275L,OR11A1:ENST00000377147.2:exon1:c.T823C:p.F275L,
6	31797471	G	A	CTCGTGGCTG,ACGCCAACAC	exonic	HSPA1B	nonsynonymous SNV	HSPA1B:ENST00000545241.1:exon3:c.G1471A:p.D491N,HSPA1B:ENST00000375650.3:exon1:c.G1744A:p.D582N,
6	32065193	T	A	AAACACACCA,GGAGACTGCA	exonic	TNXB	nonsynonymous SNV	TNXB:ENST00000479795.1:exon3:c.A437T:p.H146L,TNXB:ENST00000375247.2:exon3:c.A437T:p.H146L,TNXB:ENST00000375244.3:exon3:c.A437T:p.H146L,
6	34003852	G	A	TCGAAGATGC,GTAGATGCGG	exonic	GRM4	nonsynonymous SNV	GRM4:ENST00000535756.1:exon8:c.C1636T:p.R546C,GRM4:ENST00000609222.1:exon9:c.C1636T:p.R546C,GRM4:ENST00000374177.3:exon7:c.C1687T:p.R563C,GRM4:ENST00000544773.2:exon8:c.C1528T:p.R510C,GRM4:ENST00000374181.4:exon8:c.C2035T:p.R679C,GRM4:ENST00000538487.2:exo
6	41162400	C	A	GGAGGCAGGT,TGGTGGAGGC	exonic	TREML2	nonsynonymous SNV	TREML2:ENST00000483722.1:exon3:c.G548T:p.R183I,
6	42074665	C	G	TTGGTGGCCC,CTCTTCCTTT	exonic	C6orf132	nonsynonymous SNV	C6orf132:ENST00000341865.4:exon4:c.G985C:p.G329R,
6	44921105	C	T	GAAGGGGTCC,CTGCCTTGGT	exonic	SUPT3H	nonsynonymous SNV	SUPT3H:ENST00000306867.5:exon8:c.G743A:p.G248E,SUPT3H:ENST00000475057.1:exon9:c.G743A:p.G248E,SUPT3H:ENST00000371458.1:exon3:c.G92A:p.G31E,SUPT3H:ENST00000371460.1:exon11:c.G776A:p.G259E,SUPT3H:ENST00000371459.1:exon9:c.G743A:p.G248E,SUPT3H:ENST0000037146
6	56469061	G	T	TAATTTTTTG,CTATGTTGAT	exonic	DST	nonsynonymous SNV	DST:ENST00000446842.2:exon26:c.C8754A:p.S2918R,DST:ENST00000312431.6:exon36:c.C9732A:p.S3244R,DST:ENST00000439203.1:exon26:c.C8754A:p.S2918R,DST:ENST00000361203.3:exon36:c.C9732A:p.S3244R,DST:ENST00000370754.5:exon39:c.C10266A:p.S3422R,DST:ENST00000370769.4:ex
6	65146067	C	G	ATTATCTTAC,TGATAAGCAG	exonic	EYS	nonsynonymous SNV	EYS:ENST00000370616.2:exon25:c.G5927C:p.R1976T,EYS:ENST00000503581.1:exon28:c.G5927C:p.R1976T,EYS:ENST00000370621.3:exon28:c.G5927C:p.R1976T,
6	76715207	T	A	TTCTGCACTG,GTCTCTTAAA	exonic	IMPG1	nonsynonymous SNV	IMPG1:ENST00000369950.3:exon10:c.A932T:p.H311L,
6	90327855	G	A	ACTTAAATAT,GAATGTAACC	exonic	ANKRD6	nonsynonymous SNV	ANKRD6:ENST00000485637.1:exon9:c.G897A:p.M299I,
6	99328454	T	A	TAAACTGAGG,GCTGAAGCTC	exonic	FBXL4	nonsynonymous SNV	FBXL4:ENST00000229971.1:exon7:c.A1364T:p.H455L,FBXL4:ENST00000369244.2:exon8:c.A1364T:p.H455L,
6	117622263	C	G	GGTCTTTGGT,GGGTTCTTGA	exonic	ROS1	nonsynonymous SNV	ROS1:ENST00000368507.3:exon43:c.G6589C:p.D2197H,ROS1:ENST00000368508.3:exon42:c.G6607C:p.D2203H,
6	117996952	G	A	TGGCGGCGCT,CTGCCGCGCC	exonic	NUS1	nonsynonymous SNV	NUS1:ENST00000368494.3:exon1:c.G119A:p.C40Y,
6	119295727	T	G	TGTTTAAATC,TGTTCATGCA	exonic	FAM184A	nonsynonymous SNV	FAM184A:ENST00000521043.1:exon3:c.A270C:p.Q90H,FAM184A:ENST00000338891.7:exon14:c.A2781C:p.Q927H,
6	123892206	G	T	GTCCTCTTCA,CACTTTTCCG	exonic	TRDN	nonsynonymous SNV	TRDN:ENST00000334268.4:exon2:c.C94A:p.L32M,TRDN:ENST00000546248.1:exon2:c.C94A:p.L32M,TRDN:ENST00000398178.3:exon2:c.C94A:p.L32M,TRDN:ENST00000542443.1:exon2:c.C94A:p.L32M,
6	130415460	G	A	CTCAACCCCG,GATGTAAAGG	exonic	L3MBTL3	nonsynonymous SNV	L3MBTL3:ENST00000529410.1:exon20:c.G1684A:p.G562R,L3MBTL3:ENST00000368139.2:exon16:c.G1609A:p.G537R,L3MBTL3:ENST00000368136.2:exon18:c.G1684A:p.G562R,L3MBTL3:ENST00000533560.1:exon17:c.G1609A:p.G537R,L3MBTL3:ENST00000526019.1:exon17:c.G1609A:p.G537R,L3MBTL
6	132190603	C	T	TTGAGACCCT,TGATGTCCCA	exonic	ENPP1	nonsynonymous SNV	ENPP1:ENST00000360971.2:exon13:c.C1379T:p.S460F,
6	152647742	C	A	ACCTTTGACA,TGGCTATATA	exonic	SYNE1	nonsynonymous SNV	SYNE1:ENST00000341594.5:exon82:c.G14223T:p.Q4741H,SYNE1:ENST00000448038.1:exon79:c.G14769T:p.Q4923H,SYNE1:ENST00000423061.1:exon78:c.G14769T:p.Q4923H,SYNE1:ENST00000265368.4:exon79:c.G14982T:p.Q4994H,SYNE1:ENST00000367255.5:exon79:c.G14982T:p.Q4994H,
6	152652190	C	A	AAAACACTGT,ATAGACACTC	exonic	SYNE1	nonsynonymous SNV	SYNE1:ENST00000448038.1:exon78:c.G13417T:p.D4473Y,SYNE1:ENST00000423061.1:exon77:c.G13417T:p.D4473Y,SYNE1:ENST00000265368.4:exon78:c.G13630T:p.D4544Y,SYNE1:ENST00000367255.5:exon78:c.G13630T:p.D4544Y,
6	157507508	G	T	TGGAGCCGAG,GCCTGTGACA	exonic	ARID1B	nonsynonymous SNV	ARID1B:ENST00000319584.6:exon12:c.G1956T:p.R652S,
6	160148328	G	A	TCGGCGGCGG,CGAGTGACTG	exonic	SOD2	nonsynonymous SNV	SOD2:ENST00000545162.1:exon1:c.C62T:p.A21V,
6	166571930	G	C	TCCCGAGGAA,AGGGCGCCGA	exonic	T	nonsynonymous SNV	T:ENST00000366876.2:exon8:c.C1184G:p.S395C,T:ENST00000366871.3:exon8:c.C1007G:p.S336C,T:ENST00000296946.2:exon9:c.C1181G:p.S394C,
7	6193769	C	T	TCCGCCTGCG,GGTCGGAGGA	exonic	USP42	nonsynonymous SNV	USP42:ENST00000306177.5:exon15:c.C2584T:p.R862W,USP42:ENST00000426246.2:exon13:c.C2122T:p.R708W,
7	11500300	C	A	TCTTGCCTGT,GCCCAGGCCC	exonic	THSD7A	nonsynonymous SNV	THSD7A:ENST00000423059.4:exon11:c.G2594T:p.R865L,
7	11676115	C	A	GGGGGCGCCA,CACATGACGC	exonic	THSD7A	nonsynonymous SNV	THSD7A:ENST00000423059.4:exon2:c.G664T:p.V222L,
7	12409677	C	A	AAAGTTCTGC,GTTTCCATCT	exonic	VWDE	nonsynonymous SNV	VWDE:ENST00000452576.2:exon12:c.G2255T:p.R752L,VWDE:ENST00000275358.3:exon12:c.G2255T:p.R752L,
7	20441689	G	T	AAAAGTGTAT,GAAAATACTG	exonic	ITGB8	stopgain	ITGB8:ENST00000222573.4:exon10:c.G1627T:p.G543X,ITGB8:ENST00000537992.1:exon11:c.G1222T:p.G408X,
7	21745124	T	A	TCGGTCTTTG,AGTTGGAAAT	exonic	DNAH11	nonsynonymous SNV	DNAH11:ENST00000409508.3:exon39:c.T6515A:p.V2172E,DNAH11:ENST00000328843.6:exon40:c.T6536A:p.V2179E,
7	27839632	G	T	TGCAAAAATG,AGCTGAAATG	exonic	TAX1BP1	stopgain	TAX1BP1:ENST00000396319.2:exon13:c.G1687T:p.E563X,TAX1BP1:ENST00000265393.6:exon13:c.G1687T:p.E563X,TAX1BP1:ENST00000543117.1:exon13:c.G1687T:p.E563X,TAX1BP1:ENST00000409980.1:exon13:c.G1687T:p.E563X,TAX1BP1:ENST00000433216.2:exon12:c.G1216T:p.E406X,TAX1BP1:
7	37253013	A	G	TAGAACATAC,GCTGGTGCGC	exonic	ELMO1	nonsynonymous SNV	ELMO1:ENST00000442504.1:exon12:c.T881C:p.L294P,ELMO1:ENST00000310758.4:exon12:c.T881C:p.L294P,ELMO1:ENST00000424212.1:exon2:c.T104C:p.L35P,ELMO1:ENST00000448602.1:exon12:c.T881C:p.L294P,ELMO1:ENST00000420636.1:exon2:c.T95C:p.L32P,
7	43532745	C	T	GCTGCAAGAG,GTCAGCCAAG	exonic	HECW1	nonsynonymous SNV	HECW1:ENST00000453890.1:exon17:c.C3301T:p.R1101C,HECW1:ENST00000395891.2:exon19:c.C3403T:p.R1135C,
7	45222963	G	T	CTGTCGCCAT,GCTGGCTGG	exonic	RAMP3	nonsynonymous SNV	RAMP3:ENST00000242249.4:exon3:c.G399T:p.M133I,RAMP3:ENST00000496212.1:exon3:c.G399T:p.M133I,RAMP3:ENST00000481345.1:exon3:c.G399T:p.M133I,
7	47872751	C	A	TGGTCAGCCC,ATGAACCTGC	exonic	PKD1L1	nonsynonymous SNV	PKD1L1:ENST00000289672.2:exon41:c.G6274T:p.G2092W,
7	72178616	A	T	GTGACCAGGA,GCTGGAGATT	exonic	TYW1B	nonsynonymous SNV	TYW1B:ENST00000343721.5:exon3:c.T211A:p.F71I,
7	73973253	G	T	GAGTAACCCC,GCTCCGTGAT	exonic	GTF2IRD1	nonsynonymous SNV	GTF2IRD1:ENST00000476977.1:exon21:c.G2170T:p.G724C,GTF2IRD1:ENST00000455841.2:exon21:c.G2266T:p.G756C,GTF2IRD1:ENST00000424337.2:exon21:c.G2170T:p.G724C,GTF2IRD1:ENST00000265755.3:exon21:c.G2215T:p.G739C,
7	80456749	C	G	ACTGTGGGAT,TTTGCCAGCC	exonic	SEMA3C	nonsynonymous SNV	SEMA3C:ENST00000419255.2:exon4:c.G319C:p.D107H,SEMA3C:ENST00000265361.3:exon4:c.G319C:p.D107H,SEMA3C:ENST00000544525.1:exon4:c.G373C:p.D125H,
7	82595351	C	A	ATGTTTTTGC,TCTGGGAGTA	exonic	PCLO	nonsynonymous SNV	PCLO:ENST00000423517.2:exon4:c.G3753T:p.E1251D,PCLO:ENST00000333891.9:exon4:c.G3753T:p.E1251D,
7	92764351	T	A	ATAGAGTGTT,TGGAATAGTA	exonic	SAMD9L	stopgain	SAMD9L:ENST00000318238.4:exon5:c.A934T:p.K312X,SAMD9L:ENST00000437805.1:exon6:c.A934T:p.K312X,SAMD9L:ENST00000411955.1:exon6:c.A934T:p.K312X,
7	100190423	G	C	CGGACCCAAG,TGTGACACAG	exonic	FBXO24	nonsynonymous SNV	FBXO24:ENST00000241071.6:exon5:c.G576C:p.R192S,FBXO24:ENST00000360609.2:exon5:c.G534C:p.R178S,FBXO24:ENST00000427939.2:exon5:c.G690C:p.R230S,FBXO24:ENST00000468962.1:exon5:c.G540C:p.R180S,FBXO24:ENST00000465843.1:exon5:c.G534C:p.R178S,
7	128481259	G	A	AGCCAAGATC,AATGTGACGA	exonic	FLNC	nonsynonymous SNV	FLNC:ENST00000325888.8:exon12:c.G1849A:p.E617K,FLNC:ENST00000346177.6:exon12:c.G1849A:p.E617K,
7	129824056	G	T	GAATCCATAT,ATCAAGAAGT	exonic	TMEM209	nonsynonymous SNV	TMEM209:ENST00000462753.1:exon8:c.C988A:p.H330N,TMEM209:ENST00000473456.1:exon8:c.C991A:p.H331N,TMEM209:ENST00000397622.2:exon8:c.C991A:p.H331N,TMEM209:ENST00000336804.8:exon8:c.C988A:p.H330N,
7	136700820	A	G	TGGGCCCCAT,CAATGTCATG	exonic	CHRM2	nonsynonymous SNV	CHRM2:ENST00000397608.3:exon4:c.A1208G:p.Y403C,CHRM2:ENST00000401861.1:exon5:c.A1208G:p.Y403C,CHRM2:ENST00000445907.2:exon3:c.A1208G:p.Y403C,CHRM2:ENST00000320658.5:exon3:c.A1208G:p.Y403C,CHRM2:ENST00000453373.1:exon3:c.A1208G:p.Y403C,CHRM2:ENST0000040
7	137374714	C	T	TGTGCAGGAG,CAGATGCTGG	exonic	DGKI	nonsynonymous SNV	DGKI:ENST00000424189.2:exon2:c.G436A:p.A146T,DGKI:ENST00000446122.1:exon2:c.G436A:p.A146T,DGKI:ENST00000288490.5:exon2:c.G436A:p.A146T,
7	141755858	G	C	GAGGACGGCA,TGCCCATGGA	exonic	MGAM	nonsynonymous SNV	MGAM:ENST00000549489.2:exon29:c.G3542C:p.S1181T,MGAM:ENST00000475668.2:exon29:c.G3542C:p.S1181T,
7	148979196	G	T	TGGCCCGCCT,CCCCTACTGC	exonic	ZNF783	nonsynonymous SNV	ZNF783:ENST00000434415.1:exon6:c.G1403T:p.C468F,
7	150656662	G	A	ACACTTACCT,GGGCCAGCCA	exonic	KCNH2	nonsynonymous SNV	KCNH2:ENST00000430723.3:exon3:c.C470T:p.P157L,KCNH2:ENST00000262186.5:exon3:c.C470T:p.P157L,KCNH2:ENST00000392968.2:exon1:c.C182T:p.P61L,
7	158706999	G	T	AGCTTTCTGC,GGCTGCTTGT	exonic	WDR60	nonsynonymous SNV	WDR60:ENST00000444851.1:exon10:c.G1109T:p.R370L,WDR60:ENST00000407559.3:exon14:c.G1778T:p.R593L,
8	35579837	C	A	GCCAGAGTGA,TATGGCGTGG	exonic	UNC5D	nonsynonymous SNV	UNC5D:ENST00000416672.1:exon10:c.C1242A:p.D414E,UNC5D:ENST00000287272.2:exon8:c.C1059A:p.D353E,UNC5D:ENST00000404895.2:exon9:c.C1227A:p.D409E,UNC5D:ENST00000420357.1:exon7:c.C1026A:p.D342E,UNC5D:ENST00000453357.2:exon9:c.C1212A:p.D404E,
8	41363518	G	A	CCTATTGAGC,AGGACTGCGA	exonic	GOLGA7	nonsynonymous SNV	GOLGA7:ENST00000520817.1:exon4:c.G353A:p.R118Q,GOLGA7:ENST00000357743.4:exon3:c.G353A:p.R118Q,GOLGA7:ENST00000405786.2:exon4:c.G353A:p.R118Q,GOLGA7:ENST00000518270.1:exon4:c.G353A:p.R118Q,
8	56015797	T	G	TCCTGCTCCT,CTGCATCTGG	exonic	XKR4	nonsynonymous SNV	XKR4:ENST00000327381.6:exon1:c.T749G:p.F250C,
8	66605980	T	A	AAGGAGAGTG,TCAGCAAGAC	exonic	MTFR1	stopgain	MTFR1:ENST00000458689.2:exon2:c.T168A:p.C56X,MTFR1:ENST00000262146.4:exon4:c.T267A:p.C89X,
8	77764058	G	C	CATGTGGCTG,TGGGCACAGC	exonic	ZFHX4	nonsynonymous SNV	ZFHX4:ENST00000455469.2:exon10:c.G4766C:p.G1589A,ZFHX4:ENST00000518282.1:exon9:c.G4823C:p.G1608A,ZFHX4:ENST00000050961.6:exon10:c.G4766C:p.G1589A,ZFHX4:ENST00000521891.2:exon10:c.G4901C:p.G1634A,
8	106814813	A	G	TCTCAGCAAA,AGTGTTTATC	exonic	ZFPM2	nonsynonymous SNV	ZFPM2:ENST00000520492.1:exon8:c.A2107G:p.K703E,ZFPM2:ENST00000378472.4:exon5:c.A1696G:p.K566E,ZFPM2:ENST00000517361.1:exon6:c.A2107G:p.K703E,ZFPM2:ENST00000407775.2:exon8:c.A2503G:p.K835E,
8	109468140	A	C	AGGATTTTAC,AGAAGATCCA	exonic	EMC2	nonsynonymous SNV	EMC2:ENST00000220853.3:exon5:c.A344C:p.Q115P,
8	110447423	A	T	CACAAGAAAA,GAGCTCAAGT	exonic	PKHD1L1	nonsynonymous SNV	PKHD1L1:ENST00000378402.5:exon29:c.A3345T:p.K1115N,
8	113871468	C	T	ACCACTTGGT,CTTGAAGATT	exonic	CSMD3	nonsynonymous SNV	CSMD3:ENST00000352409.3:exon11:c.G1661A:p.G554E,CSMD3:ENST00000297405.5:exon11:c.G1661A:p.G554E,CSMD3:ENST00000455883.2:exon10:c.G1349A:p.G450E,CSMD3:ENST00000343508.3:exon12:c.G1541A:p.G514E,
8	120638941	C	T	AGATGTTGGT,CAGGGGGAGT	exonic	ENPP2	stopgain	ENPP2:ENST00000518958.1:exon3:c.G156A:p.W52X,ENPP2:ENST00000259486.6:exon3:c.G156A:p.W52X,ENPP2:ENST00000427067.2:exon3:c.G144A:p.W48X,ENPP2:ENST00000522826.1:exon3:c.G156A:p.W52X,ENPP2:ENST00000520066.1:exon3:c.G156A:p.W52X,ENPP2:ENST00000075322.6:exon3:c
8	134232854	G	A	GTCCTGGATG,GGTGCGCTAC	exonic	WISP1	nonsynonymous SNV	WISP1:ENST00000250160.6:exon3:c.G380A:p.G127E,
8	139601541	G	C	GGCTGGCGAA,TAGGCACACT	exonic	COL22A1	stopgain	COL22A1:ENST00000303045.6:exon65:c.C4836G:p.Y1612X,COL22A1:ENST00000435777.1:exon64:c.C4776G:p.Y1592X,
8	139603665	A	G	AATGAGTCAT,CCTGGGATTC	splicing	COL22A1(EN		.
8	143927909	T	A	AGGAAAAATC,TCAAAACTAA	exonic	GML	nonsynonymous SNV	GML:ENST00000220940.1:exon4:c.T280A:p.F94I,
8	146016862	T	C	AGGGAGCACA,TGCCAATGTT	exonic	RPL8	nonsynonymous SNV	RPL8:ENST00000394920.2:exon4:c.A299G:p.N100S,RPL8:ENST00000532702.1:exon4:c.A299G:p.N100S,RPL8:ENST00000262584.3:exon4:c.A299G:p.N100S,RPL8:ENST00000528957.1:exon3:c.A299G:p.N100S,RPL8:ENST00000533397.1:exon3:c.A299G:p.N100S,RPL8:ENST00000531975.1:exon3:c.A185(
9	4118217	C	A	GACTGGCTGT,GGGGCCCGGC	exonic	GLIS3	nonsynonymous SNV	GLIS3:ENST00000324333.10:exon3:c.G796T:p.D266Y,GLIS3:ENST00000381971.3:exon4:c.G1261T:p.D421Y,
9	14851380	T	A	ATTGGTCTGG,GTGATCCAAC	exonic	FREM1	nonsynonymous SNV	FREM1:ENST00000380875.3:exon7:c.A1054T:p.T352S,FREM1:ENST00000380881.4:exon7:c.A1057T:p.T353S,FREM1:ENST00000422223.2:exon7:c.A1054T:p.T352S,FREM1:ENST00000380880.3:exon6:c.A1054T:p.T352S,
9	27949478	T	A	AGGGCAGTGC,ATGGAAATCC	exonic	LINGO2	nonsynonymous SNV	LINGO2:ENST00000379992.2:exon6:c.A1192T:p.S398C,LINGO2:ENST00000308675.3:exon4:c.A1192T:p.S398C,
9	37854934	G	T	TTTAGAAGTA,GCAGCTATCC	exonic	DCAF10	nonsynonymous SNV	DCAF10:ENST00000242323.7:exon4:c.G1009T:p.G337C,DCAF10:ENST00000377724.3:exon4:c.G1009T:p.G337C,
9	43627245	G	C	TTGGGGTGTG,ATGAAATAAA	exonic	SPATA31A6	nonsynonymous SNV	SPATA31A6:ENST00000332857.6:exon4:c.C1442G:p.S481C,
9	86293513	G	A	AAGTTCCAAC,TCTACGAAAA	exonic	UBQLN1	nonsynonymous SNV	UBQLN1:ENST00000376395.4:exon5:c.C713T:p.T238M,UBQLN1:ENST00000529923.1:exon2:c.C104T:p.T35M,UBQLN1:ENST00000257468.7:exon5:c.C713T:p.T238M,
9	99284801	C	A	AGAGGAATAG,GTTTGTTTTG	exonic	CDC14B	nonsynonymous SNV	CDC14B:ENST00000375240.3:exon12:c.G1330T:p.A444S,CDC14B:ENST00000375242.3:exon12:c.G1219T:p.A407S,CDC14B:ENST00000463569.1:exon12:c.G1330T:p.A444S,CDC14B:ENST00000375241.1:exon12:c.G1330T:p.A444S,CDC14B:ENST00000474602.1:exon12:c.G1330T:p.A444S,CDC14B:ENST
9	101787274	G	A	GGTGAACCTG,GCCCCCGGTG	exonic	COL15A1	nonsynonymous SNV	COL15A1:ENST00000375001.3:exon15:c.G1973A:p.G658E,
9	101797417	C	A	CCTGGATGCA,AATGGGACTT	exonic	COL15A1	nonsynonymous SNV	COL15A1:ENST00000375001.3:exon18:c.C2201A:p.T734K,
9	101907076	A	G	GAAGAATGGA,CTTGCTGTAT	exonic	TGFBR1	nonsynonymous SNV	TGFBR1:ENST00000552516.1:exon6:c.A1048G:p.T350A,TGFBR1:ENST00000549766.1:exon6:c.A1048G:p.T350A,TGFBR1:ENST00000374990.2:exon5:c.A805G:p.T269A,TGFBR1:ENST00000374994.4:exon6:c.A1036G:p.T346A,TGFBR1:ENST00000550253.1:exon6:c.A829G:p.T277A,
9	104189868	C	A	CGCCACTTCC,AAAGTCAACA	exonic	ALDOB	nonsynonymous SNV	ALDOB:ENST00000374855.4:exon5:c.G436T:p.G146W,
9	107361008	G	C	CCTCCGAAGA,CTAATTTTGA	exonic	OR13C5	nonsynonymous SNV	OR13C5:ENST00000374779.2:exon1:c.C687G:p.S229R,
9	107591392	C	A	TGAAGAGGGG,ATTGACCGGC	exonic	ABCA1	nonsynonymous SNV	ABCA1:ENST00000374736.3:exon15:c.G1920T:p.M640I,
9	116929974	G	C	CTCAGATGTG,ACATCCTCCA	exonic	COL27A1	nonsynonymous SNV	COL27A1:ENST00000356083.3:exon3:c.G139C:p.D47H,
10	7627975	T	C	AATCCAATGA,ACTGAAACGG	exonic	ITIH5	nonsynonymous SNV	ITIH5:ENST00000256861.6:exon8:c.A997G:p.I333V,ITIH5:ENST00000298441.6:exon4:c.A355G:p.I119V,ITIH5:ENST00000397145.2:exon8:c.A997G:p.I333V,ITIH5:ENST00000397146.2:exon8:c.A997G:p.I333V,ITIH5:ENST00000446830.2:exon8:c.A343G:p.I115V,
10	15617507	A	T	ATGTTCCACC,ATGGTCCAAC	exonic	ITGA8	stopgain	ITGA8:ENST00000378076.3:exon24:c.T2459A:p.L820X,
10	15726056	T	A	AATTGCTACA,AGCAGGTGCC	exonic	ITGA8	nonsynonymous SNV	ITGA8:ENST00000378076.3:exon4:c.A515T:p.Y172F,
10	15726057	A	T	ATTGCTACAT,GCAGGTGCCA	exonic	ITGA8	nonsynonymous SNV	ITGA8:ENST00000378076.3:exon4:c.T514A:p.Y172N,
10	17726738	C	T	CGTTGCTATG,AGGCTTTGAC	exonic	STAM	stopgain	STAM:ENST00000377524.3:exon3:c.C190T:p.Q64X,
10	18828547	G	T	AAGCAGCGCA,CCGTCATAAA	exonic	CACNB2	nonsynonymous SNV	CACNB2:ENST00000377319.3:exon13:c.G1598T:p.S533I,CACNB2:ENST00000282343.8:exon14:c.G1793T:p.S598I,CACNB2:ENST00000377331.2:exon14:c.G1721T:p.S574I,CACNB2:ENST00000352115.6:exon14:c.G1805T:p.S602I,CACNB2:ENST00000377315.4:exon13:c.G1733T:p.S578I,CACNB2:ENST00(
10	26357739	C	G	TTCCAGAGAT,AGATCTACGT	exonic	MYO3A	nonsynonymous SNV	MYO3A:ENST00000543632.1:exon11:c.C1096G:p.Q366E,MYO3A:ENST00000265944.5:exon12:c.C1096G:p.Q366E,
10	48429183	G	T	CTGGGCCGGG,CACCCCCGGG	exonic	GDF10	nonsynonymous SNV	GDF10:ENST00000224605.2:exon2:c.C703A:p.P235T,
10	50373407	C	T	GACGTGGGCT,TGTGCAGAAG	splicing	C10orf128(EN		.
10	61835726	G	C	CATAGTAATT,ATGACCTCTC	exonic	ANK3	stopgain	ANK3:ENST00000280772.2:exon37:c.C4913G:p.S1638X,
10	75525219	A	T	GCTTCACCGT,TGTTGTGGAC	exonic	SEC24C	nonsynonymous SNV	SEC24C:ENST00000411652.2:exon9:c.A881T:p.Y294F,SEC24C:ENST00000546025.1:exon5:c.A572T:p.Y191F,SEC24C:ENST00000345254.4:exon9:c.A1238T:p.Y413F,SEC24C:ENST00000339365.2:exon10:c.A1238T:p.Y413F,SEC24C:ENST00000465076.1:exon9:c.A1238T:p.Y413F,
10	83635677	C	A	GCGACGGTCC,GTCCACCACG	exonic	NRG3	nonsynonymous SNV	NRG3:ENST00000404547.1:exon1:c.C581A:p.P194Q,NRG3:ENST00000372141.2:exon1:c.C581A:p.P194Q,
10	87373320	G	C	CGCTGGCATG,CTGGTGAGGT	exonic	GRID1	nonsynonymous SNV	GRID1:ENST00000327946.7:exon15:c.C2445G:p.S815R,GRID1:ENST00000536331.1:exon8:c.C1158G:p.S386R,
10	119029883	C	A	ATTGGATTTC,ATGGCTCATG	exonic	SLC18A2	nonsynonymous SNV	SLC18A2:ENST00000298472.5:exon15:c.C1349A:p.P450Q,
10	119043537	T	C	CTAAGGTTTT,CAGTGTCCTG	exonic	PDZD8	nonsynonymous SNV	PDZD8:ENST00000334464.5:exon5:c.A2707G:p.K903E,
10	134692994	G	A	CTCGGCGCCC,GCGACAGCAC	exonic	TTC40	nonsynonymous SNV	TTC40:ENST00000368582.2:exon29:c.C3941T:p.P1314L,TTC40:ENST00000368586.5:exon29:c.C3941T:p.P1314L,
10	134981802	G	A	CGACGTGACC,GGAACACCTT	exonic	KNDC1	nonsynonymous SNV	KNDC1:ENST00000368572.2:exon3:c.G346A:p.G116R,KNDC1:ENST00000304613.3:exon3:c.G346A:p.G116R,KNDC1:ENST00000368571.2:exon2:c.G151A:p.G51R,
11	376897	C	T	GCCCCCAGCC,GGGCGCAGGC	exonic	B4GALNT4	nonsynonymous SNV	B4GALNT4:ENST00000329962.6:exon14:c.C1774T:p.R592W,
11	5809344	C	A	TTCTGTCGAG,ATCTGCTGAT	exonic	OR52N1	nonsynonymous SNV	OR52N1:ENST00000317078.1:exon1:c.G703T:p.A235S,
11	6024223	G	T	GAGATGCCAT,TAAAGTTTCC	exonic	OR56A4	stopgain	OR56A4:ENST00000330728.4:exon1:c.C156A:p.Y52X,
11	6292763	G	T	AGCACACTGG,CCCTGGCTGA	exonic	CCKBR	nonsynonymous SNV	CCKBR:ENST00000532715.1:exon4:c.G1082T:p.G361V,CCKBR:ENST00000525462.1:exon4:c.G1541T:p.G514V,CCKBR:ENST00000334619.2:exon5:c.G1334T:p.G445V,
11	6643450	C	A	TCCTCCTCGC,GGCCACAATG	exonic	DCHS1	nonsynonymous SNV	DCHS1:ENST00000299441.3:exon21:c.G9457T:p.G3153C,
11	13733476	C	A	TCAACAAAGG,AGGGAAAGGA	exonic	FAR1	nonsynonymous SNV	FAR1:ENST00000354817.3:exon7:c.C770A:p.A257E,FAR1:ENST00000532701.1:exon7:c.C770A:p.A257E,
11	17464267	T	A	GTGAACTCAC,GGAGATGGAG	exonic	ABCC8	nonsynonymous SNV	ABCC8:ENST00000527905.1:exon10:c.A1630T:p.I544F,ABCC8:ENST00000389817.3:exon10:c.A1630T:p.I544F,ABCC8:ENST00000302539.4:exon10:c.A1630T:p.I544F,
11	20178090	C	A	TCCGCCATTT,ATGCGTCGGT	exonic	DBX1	nonsynonymous SNV	DBX1:ENST00000227256.3:exon4:c.G819T:p.M273I,DBX1:ENST00000524983.2:exon4:c.G702T:p.M234I,
11	26484636	C	A	TGACCGATCT,GTCTCATTAA	exonic	ANO3	nonsynonymous SNV	ANO3:ENST00000531646.1:exon4:c.C373A:p.R125S,ANO3:ENST00000256737.3:exon4:c.C373A:p.R125S,ANO3:ENST00000537978.1:exon5:c.C325A:p.R109S,ANO3:ENST00000525139.1:exon4:c.C325A:p.R109S,
11	33368839	G	T	TGTTTCACAG,TATTCCTGCA	exonic	HIPK3	nonsynonymous SNV	HIPK3:ENST00000303296.4:exon9:c.G1898T:p.G633V,HIPK3:ENST00000456517.1:exon9:c.G1898T:p.G633V,HIPK3:ENST00000379016.3:exon9:c.G1898T:p.G633V,HIPK3:ENST00000525975.1:exon9:c.G1898T:p.G633V,
11	40137040	G	T	CTCCACTAGT,ACTGAAGGTT	exonic	LRRC4C	stopgain	LRRC4C:ENST00000528697.1:exon7:c.C803A:p.S268X,LRRC4C:ENST00000530763.1:exon5:c.C803A:p.S268X,LRRC4C:ENST00000278198.2:exon2:c.C803A:p.S268X,LRRC4C:ENST00000527150.1:exon3:c.C803A:p.S268X,
11	43342402	A	G	GAACTGCCTC,ATTTGCCACT	exonic	API5	nonsynonymous SNV	API5:ENST00000455725.2:exon4:c.A230G:p.Q77R,API5:ENST00000531273.1:exon3:c.A263G:p.Q88R,API5:ENST00000378852.3:exon3:c.A263G:p.Q88R,API5:ENST00000534600.1:exon3:c.A263G:p.Q88R,API5:ENST00000420461.2:exon2:c.A101G:p.Q34R,
11	46671864	A	G	GTCATATTAT,CAGGTAAACA	exonic	ATG13	nonsynonymous SNV	ATG13:ENST00000312040.4:exon7:c.A455G:p.Y152C,ATG13:ENST00000528494.1:exon6:c.A455G:p.Y152C,ATG13:ENST00000526508.1:exon7:c.A455G:p.Y152C,ATG13:ENST00000451945.1:exon6:c.A455G:p.Y152C,ATG13:ENST00000530500.1:exon5:c.A218G:p.Y73C,ATG13:ENST00000434074.1:exon6:
11	47612180	G	T	TGGCCACATC,AAGTCGCCCC	exonic	C1QTNF4	nonsynonymous SNV	C1QTNF4:ENST00000530097.1:exon1:c.C183A:p.F61L,C1QTNF4:ENST00000302514.3:exon2:c.C183A:p.F61L,
11	51412355	C	T	CTGAGAAAAG,CCAGGAGGAC	exonic	OR4A5	nonsynonymous SNV	OR4A5:ENST00000319760.6:exon1:c.G41A:p.G14D,
11	55587661	C	A	CTCCTCACTA,TCTCCCTTTC	exonic	OR5D18	nonsynonymous SNV	OR5D18:ENST00000333976.4:exon1:c.C556A:p.L186I,
11	55798231	C	G	TGCTGAGTGC,TTATCCTGGC	exonic	OR5AS1	nonsynonymous SNV	OR5AS1:ENST00000313555.1:exon1:c.C337G:p.L113V,
11	55890601	C	G	TCACCATCTT,TATGGAACTA	exonic	OR8H3	nonsynonymous SNV	OR8H3:ENST00000313472.3:exon1:c.C753G:p.F251L,
11	55904825	C	A	CAAATGGCCA,ATAGCGGTCA	exonic	OR8J3	nonsynonymous SNV	OR8J3:ENST00000301529.1:exon1:c.G370T:p.V124L,
11	55904942	A	T	TTTACTAAAA,GTTCATCAGC	exonic	OR8J3	nonsynonymous SNV	OR8J3:ENST00000301529.1:exon1:c.T253A:p.F85I,
11	59224442	G	T	TCATGGACCA,ATCAACCACA	exonic	OR4D6	nonsynonymous SNV	OR4D6:ENST00000300127.2:exon1:c.G9T:p.Q3H,
11	59245473	G	A	GGCCCATACA,ACATTTTCAT	exonic	OR4D10	nonsynonymous SNV	OR4D10:ENST00000530162.1:exon1:c.G571A:p.D191N,
11	103870919	G	T	GACTCTGCAC,TAGCCGTTTC	exonic	PDGFD	stopgain	PDGFD:ENST00000302251.5:exon2:c.C171A:p.Y57X,PDGFD:ENST00000393158.2:exon2:c.C189A:p.Y63X,PDGFD:ENST00000529268.1:exon2:c.C258A:p.Y86X,
11	123476077	C	A	AATGACAGCT,ATCCAAGAGC	exonic	GRAMD1B	stopgain	GRAMD1B:ENST00000529432.1:exon9:c.C665A:p.S222X,GRAMD1B:ENST00000534764.1:exon9:c.C773A:p.S258X,GRAMD1B:ENST00000529750.1:exon9:c.C785A:p.S262X,GRAMD1B:ENST00000456860.2:exon10:c.C806A:p.S269X,GRAMD1B:ENST00000322282.7:exon9:c.C785A:p.S262X,
11	123900375	C	T	CATGGGCCTT,CCCATGCCCC	exonic	OR10G8	nonsynonymous SNV	OR10G8:ENST00000431524.1:exon1:c.C46T:p.P16S,

11	124266858	C	G	CCTTATACAG,AATGGATTAC	exonic	OR8B3	nonsynonymous SNV	OR8B3:ENST00000354597.3:exon1:c.G390C:p.L130F,
11	124766093	C	T	CCTGCCCTTA,CCTGGATGGA	splicing	ROBO4(ENST	.	.
12	417080	C	T	TTCTTCTATG,GGTCCACCAT	exonic	KDM5A	nonsynonymous SNV	KDM5A:ENST00000399788.2:exon23:c.G3470A:p.R1157H,KDM5A:ENST00000382815.4:exon23:c.G3470A:p.R1157H,
12	5744378	C	A	AGGATGACCA,GAGGTTGATG	exonic	ANO2	nonsynonymous SNV	ANO2:ENST00000546188.1:exon17:c.G1759T:p.V587L,ANO2:ENST00000356134.5:exon18:c.G1759T:p.V587L,ANO2:ENST00000327087.8:exon17:c.G1756T:p.V586L,ANO2:ENST00000545860.1:exon5:c.G436T:p.V146L,
12	11420229	G	C	AGGCTGCTGG,GATTGCCTCC	exonic	PRB3	nonsynonymous SNV	PRB3:ENST00000538488.1:exon6:c.C827G:p.P276R,PRB3:ENST00000381842.3:exon5:c.C827G:p.P276R,
12	21327559	A	T	TCCAAACTAC,TAGACCAAAG	exonic	SLCO1B1	nonsynonymous SNV	SLCO1B1:ENST00000256958.2:exon4:c.A275T:p.H92L,
12	40499101	G	T	GTGTCTCCTT,TTGTTGGCCG	exonic	SLC2A13	nonsynonymous SNV	SLC2A13:ENST00000280871.4:exon1:c.C510A:p.N170K,SLC2A13:ENST00000380858.1:exon1:c.C510A:p.N170K,
12	40873367	C	A	GACAACTGGA,CATCAGCTGA	exonic	MUC19	nonsynonymous SNV	MUC19:ENST00000454784.4:exon49:c.C4912A:p.P1638T,
12	40873368	C	A	ACAACTGGAC,ATCAGCTGAC	exonic	MUC19	nonsynonymous SNV	MUC19:ENST00000454784.4:exon49:c.C4913A:p.P1638Q,
12	40878932	G	A	ATTATCAGCT,GAGTGACAGG	exonic	MUC19	nonsynonymous SNV	MUC19:ENST00000454784.4:exon49:c.G10477A:p.G3493R,
12	46240644	A	T	TTTAGCTTCC,GAGCAGTTGT	exonic	ARID2	stopgain	ARID2:ENST00000334344.6:exon12:c.A1504T:p.R502X,ARID2:ENST00000422737.1:exon12:c.A1057T:p.R353X,ARID2:ENST00000444670.1:exon4:c.A334T:p.R112X,
12	50357892	C	A	TCACTGGCTG,TCCATGAACC	exonic	AQP5	stopgain	AQP5:ENST00000293599.6:exon3:c.C546A:p.C182X,
12	54428068	A	T	CCAGAGACGG,CGGCAAGCGG	exonic	HOXC5,RP11	nonsynonymous SNV	RP11-834C11.12:ENST00000513209.1:exon2:c.A173T:p.D58V,HOXC5:ENST00000312492.2:exon2:c.A461T:p.D154V,
12	56558167	A	T	GGCGGATCCG,GCCCCGGCCC	exonic	SMARCC2	nonsynonymous SNV	SMARCC2:ENST00000267064.4:exon27:c.T3488A:p.L1163H,SMARCC2:ENST00000550164.1:exon28:c.T3581A:p.L1194H,
12	71002978	C	A	GGGCACAACG,GGCCCCCAGG	exonic	PTPRB	nonsynonymous SNV	PTPRB:ENST00000451516.2:exon2:c.G196T:p.A66S,PTPRB:ENST00000538708.1:exon2:c.G196T:p.A66S,PTPRB:ENST00000261266.5:exon2:c.G196T:p.A66S,PTPRB:ENST00000548122.1:exon4:c.G487T:p.A163S,PTPRB:ENST00000334414.6:exon4:c.G850T:p.A284S,PTPRB:ENST00000550358.1:exon4:c.
12	78415588	A	G	CAGTCCTACA,AGATGGACTT	exonic	NAV3	nonsynonymous SNV	NAV3:ENST00000266692.7:exon9:c.A1969G:p.K657E,NAV3:ENST00000228327.6:exon9:c.A1969G:p.K657E,NAV3:ENST00000397909.2:exon9:c.A1969G:p.K657E,NAV3:ENST00000536525.2:exon9:c.A1969G:p.K657E,NAV3:ENST00000549464.1:exon9:c.A1969G:p.K657E,
12	85413482	C	T	TCAGAAATGA,AAAATCAATT	exonic	TSPAN19	nonsynonymous SNV	TSPAN19:ENST00000532498.2:exon6:c.G373A:p.V125I,
12	85677512	G	A	GTATCCAGCA,TAAGAAACGG	exonic	ALX1	nonsynonymous SNV	ALX1:ENST00000316824.3:exon2:c.G389A:p.S130N,
12	90049539	G	A	TGCATCTGTG,ACCTGAGCTC	exonic	ATP2B1	nonsynonymous SNV	ATP2B1:ENST00000348959.3:exon1:c.C125T:p.S42F,ATP2B1:ENST00000551310.1:exon2:c.C125T:p.S42F,ATP2B1:ENST00000261173.2:exon1:c.C125T:p.S42F,ATP2B1:ENST00000428670.3:exon2:c.C125T:p.S42F,ATP2B1:ENST00000359142.3:exon1:c.C125T:p.S42F,
12	95927351	C	A	TGAACAGAAA,TATTTCTATT	exonic	USP44	nonsynonymous SNV	USP44:ENST00000552440.1:exon1:c.G682T:p.V228F,USP44:ENST00000537435.2:exon2:c.G682T:p.V228F,USP44:ENST00000258499.3:exon2:c.G682T:p.V228F,USP44:ENST00000393091.2:exon2:c.G682T:p.V228F,
12	100904642	C	A	TCAGTTTCCC,AAGTTCAACC	exonic	NR1H4	nonsynonymous SNV	NR1H4:ENST00000188403.7:exon2:c.C196A:p.Q66K,NR1H4:ENST00000548884.1:exon4:c.C166A:p.Q56K,NR1H4:ENST00000549996.1:exon4:c.C166A:p.Q56K,NR1H4:ENST00000392986.3:exon4:c.C166A:p.Q56K,NR1H4:ENST00000321046.5:exon4:c.C166A:p.Q56K,NR1H4:ENST00000551379.1:exon2:c.C
12	103699824	C	A	ATGCCCTGAG,CTCCAGGTGA	exonic	C12orf42	nonsynonymous SNV	C12orf42:ENST00000548048.1:exon8:c.G358T:p.A120S,C12orf42:ENST00000552578.1:exon5:c.G559T:p.A187S,C12orf42:ENST00000378113.2:exon5:c.G559T:p.A187S,C12orf42:ENST00000548883.1:exon5:c.G559T:p.A187S,
12	108686193	G	T	TTCCCATGCA,GTTGGCTGTG	exonic	CMKLR1	nonsynonymous SNV	CMKLR1:ENST00000412676.1:exon3:c.C547A:p.L183M,CMKLR1:ENST00000312143.7:exon3:c.C547A:p.L183M,CMKLR1:ENST00000552995.1:exon3:c.C541A:p.L181M,CMKLR1:ENST00000397688.2:exon3:c.C541A:p.L181M,CMKLR1:ENST00000550402.1:exon4:c.C547A:p.L183M,
12	113549913	C	T	CTGGGGGAAG,GCTCCTCCAC	exonic	RASAL1	nonsynonymous SNV	RASAL1:ENST00000446861.3:exon13:c.G1352A:p.R451H,RASAL1:ENST00000261729.5:exon14:c.G1352A:p.R451H,RASAL1:ENST00000548055.1:exon13:c.G1352A:p.R451H,RASAL1:ENST00000546530.1:exon14:c.G1352A:p.R451H,
12	113591064	C	A	GGAGGAGAGG,ACCAGGCGGG	exonic	CCDC42B	nonsynonymous SNV	CCDC42B:ENST00000335621.6:exon4:c.C319A:p.H107N,
12	124221633	A	T	GGACTATTTG,GGCAAGTGCT	exonic	ATP6V0A2	nonsynonymous SNV	ATP6V0A2:ENST00000330342.3:exon9:c.A853T:p.R285W,ATP6V0A2:ENST00000504192.2:exon6:c.A463T:p.R155W,
12	129373205	T	A	CCTTTTGGAG,CATCTTTGGT	exonic	GLT1D1	nonsynonymous SNV	GLT1D1:ENST00000539044.1:exon3:c.T239A:p.V80D,GLT1D1:ENST00000537468.1:exon3:c.T206A:p.V69D,GLT1D1:ENST00000281703.6:exon3:c.T239A:p.V80D,GLT1D1:ENST00000442111.2:exon3:c.T239A:p.V80D,GLT1D1:ENST00000441390.2:exon3:c.T239A:p.V80D,GLT1D1:ENST00000413816.2:exon3
13	23905839	A	C	AAAACCTTTA,CTCTTAATGT	exonic	SACS	nonsynonymous SNV	SACS:ENST00000382298.3:exon10:c.T12176G:p.V4059G,SACS:ENST00000402364.1:exon8:c.T9926G:p.V3309G,SACS:ENST00000382292.3:exon9:c.T12176G:p.V4059G,
13	31232061	A	G	GTAGCAGAAA,TACAGGAATT	exonic	USPL1	nonsynonymous SNV	USPL1:ENST00000255304.4:exon9:c.A1847G:p.N616S,
13	32828466	A	T	TGACACAAAC,GCGAGCAGCA	exonic	FRY	nonsynonymous SNV	FRY:ENST00000380250.3:exon51:c.A7351T:p.S2451C,
13	38164612	G	T	ATAGCGCTGC,TTGTGGTGGC	exonic	POSTN	nonsynonymous SNV	POSTN:ENST00000541179.1:exon4:c.C338A:p.T113K,POSTN:ENST00000379742.4:exon4:c.C338A:p.T113K,POSTN:ENST00000379747.4:exon4:c.C338A:p.T113K,POSTN:ENST00000379749.4:exon4:c.C338A:p.T113K,POSTN:ENST00000379743.4:exon4:c.C338A:p.T113K,POSTN:ENST00000541481.1:exo
13	44462942	G	A	TGAATGCTAT,ATAGCAGAAT	exonic	LACC1	nonsynonymous SNV	LACC1:ENST00000441843.1:exon5:c.G957A:p.M319I,LACC1:ENST00000325686.6:exon5:c.G957A:p.M319I,
13	72147141	A	T	AGGAAGTTCC,GTCCTATAAA	exonic	DACH1	nonsynonymous SNV	DACH1:ENST00000305425.4:exon4:c.T1136A:p.L379Q,DACH1:ENST00000359684.2:exon5:c.T1292A:p.L431Q,
13	96242075	T	C	AGATGTCCTA,CAGTAGAAAC	exonic	DZIP1	nonsynonymous SNV	DZIP1:ENST00000361156.3:exon15:c.A1898G:p.D633G,DZIP1:ENST00000376829.2:exon18:c.A1955G:p.D652G,DZIP1:ENST00000361396.2:exon17:c.A1898G:p.D633G,DZIP1:ENST00000347108.3:exon16:c.A1955G:p.D652G,
13	97485134	A	T	TCTTTGAGAG,ACATTCAACC	exonic	HS6ST3	nonsynonymous SNV	HS6ST3:ENST00000376705.2:exon2:c.A1098T:p.R366S,
13	111156284	G	A	CAGGGGAGGC,AGGCCCCCCT	exonic	COL4A2	nonsynonymous SNV	COL4A2:ENST00000360467.5:exon44:c.G4229A:p.R1410Q,
14	20248944	C	T	GGGCTTCATT,ATTCTATAAT	exonic	OR4M1	nonsynonymous SNV	OR4M1:ENST00000315957.4:exon1:c.C463T:p.H155Y,
14	20612174	T	A	GGTAATCTCC,ATAGAAGCTG	exonic	OR4N5	nonsynonymous SNV	OR4N5:ENST00000333629.1:exon1:c.T280A:p.Y94N,
14	21547081	T	A	TATCAGGAAG,GGACGAGGCC	exonic	ARHGEF40	nonsynonymous SNV	ARHGEF40:ENST00000298694.4:exon11:c.T2285A:p.V762E,ARHGEF40:ENST00000298693.3:exon11:c.T2285A:p.V762E,
14	38724933	T	C	TGGGAACGCC,GCGCTCCAGT	exonic	CLEC14A	nonsynonymous SNV	CLEC14A:ENST00000342213.2:exon1:c.A295G:p.R99G,
14	45700451	C	T	ACGGACAGAT,TTCCAGTTTT	exonic	MIS18BP1	nonsynonymous SNV	MIS18BP1:ENST00000310806.4:exon8:c.G1487A:p.R496K,
14	47426642	T	C	ATAAACCCCA,AGTTTTCATT	exonic	MDGA2	nonsynonymous SNV	MDGA2:ENST00000439988.3:exon3:c.A2024G:p.Y675C,MDGA2:ENST00000399232.2:exon9:c.A1817G:p.Y606C,MDGA2:ENST00000357362.3:exon9:c.A1130G:p.Y377C,MDGA2:ENST00000426342.1:exon9:c.A1130G:p.Y377C,
14	54418810	C	A	TGAGCGGCGT,CTCCCGCGTG	exonic	BMP4	nonsynonymous SNV	BMP4:ENST00000558984.1:exon2:c.G131T:p.G44V,BMP4:ENST00000245451.4:exon3:c.G131T:p.G44V,BMP4:ENST00000559642.1:exon3:c.G131T:p.G44V,BMP4:ENST00000417573.1:exon3:c.G131T:p.G44V,BMP4:ENST00000559087.1:exon3:c.G131T:p.G44V,BMP4:ENST00000558961.1:exon1:c.G56T:p.G
14	65009192	A	G	GTCGCGCCA,AAACGCCCTG	exonic	HSPA2	nonsynonymous SNV	HSPA2:ENST00000394709.1:exon2:c.A1625G:p.K542R,HSPA2:ENST00000247207.6:exon1:c.A1625G:p.K542R,
14	70633564	C	T	GCATGGTCAT,ATCCAAGATG	exonic	SLC8A3	nonsynonymous SNV	SLC8A3:ENST00000356921.2:exon2:c.G1576A:p.D526N,SLC8A3:ENST00000357887.3:exon2:c.G1576A:p.D526N,SLC8A3:ENST00000534137.1:exon1:c.G1576A:p.D526N,SLC8A3:ENST00000528359.1:exon1:c.G1576A:p.D526N,SLC8A3:ENST00000494208.1:exon2:c.G1576A:p.D526N,SLC8A3:ENST00003
14	72191424	G	A	AGCCCACACC,GGCTGCGTGC	exonic	SIPA1L1	nonsynonymous SNV	SIPA1L1:ENST00000537413.1:exon15:c.A2911G:p.R971G,SIPA1L1:ENST00000358550.2:exon16:c.A4486G:p.R1496G,SIPA1L1:ENST00000555818.1:exon17:c.A4549G:p.R1517G,SIPA1L1:ENST00000381232.3:exon16:c.A4486G:p.R1496G,
14	92959961	G	A	GATGTGCCGG,AAGACGATTA	exonic	SLC24A4	nonsynonymous SNV	SLC24A4:ENST00000532405.1:exon17:c.G1750A:p.E584K,SLC24A4:ENST00000298877.1:exon17:c.G1807A:p.E603K,SLC24A4:ENST00000393265.2:exon17:c.G1666A:p.E556K,SLC24A4:ENST00000532405.1:exon17:c.G1858A:p.E620K,SLC24A4:ENST00000531433.1:exon18:c.G1801A:p.E601K,
14	94526799	T	G	TTATGAAGGA,TCGAGCAGGA	exonic	DDX24	nonsynonymous SNV	DDX24:ENST00000555054.1:exon5:c.A1429C:p.I477L,DDX24:ENST00000330836.5:exon5:c.A1558C:p.I520L,DDX24:ENST00000544005.1:exon4:c.A808C:p.I270L,
14	106067416	G	A	CTCCAGCCAG,TGATGTTGAT	exonic	IGHE	nonsynonymous SNV	IGHE:ENST00000390541.2:exon2:c.C443T:p.T148I,
15	22369457	G	T	TGAGAAACAA,GAAGTAAAGG	exonic	OR4M2	nonsynonymous SNV	OR4M2:ENST00000332663.2:exon1:c.G882T:p.K294N,
15	22369458	G	T	GAGAAACAAG,AAGTAAAGGC	exonic	OR4M2	stopgain	OR4M2:ENST00000332663.2:exon1:c.G883T:p.E295X,
15	23685782	C	T	TCTCCTGCTC,TGCGTCTTCT	exonic	GOLGA6L2	nonsynonymous SNV	GOLGA6L2:ENST00000567107.1:exon8:c.G1840A:p.G614R,
15	24922981	C	A	GATGGGCAGC,GCAGAAAGCT	exonic	NPAP1	nonsynonymous SNV	NPAP1:ENST00000329468.2:exon1:c.C1967A:p.P656Q,
15	44630016	A	T	ATAAACAATC,AGTAGTACCT	exonic	CASC4	nonsynonymous SNV	CASC4:ENST00000345795.2:exon5:c.A632T:p.Q211L,CASC4:ENST00000299957.6:exon5:c.A632T:p.Q211L,CASC4:ENST00000559222.1:exon6:c.A275T:p.Q92L,
15	53081336	G	T	GGGATGGTGC,GAGGAAGGCC	exonic	ONECUT1	nonsynonymous SNV	ONECUT1:ENST00000305901.5:exon1:c.C746A:p.P249Q,
15	55677892	C	A	TCACAGAATT,AACATTTCTA	exonic	CCPG1	nonsynonymous SNV	CCPG1:ENST00000563171.1:exon3:c.G81T:p.L27F,CCPG1:ENST00000569205.1:exon3:c.G81T:p.L27F,CCPG1:ENST00000564092.1:exon4:c.G81T:p.L27F,CCPG1:ENST00000425574.3:exon3:c.G81T:p.L27F,CCPG1:ENST00000310958.6:exon3:c.G81T:p.L27F,CCPG1:ENST00000568808.1:exon3:c.G81T:p.L
15	59192116	C	T	TCTTGTGTAC,ATCACCAGAA	exonic	SLTM	nonsynonymous SNV	SLTM:ENST00000380516.2:exon7:c.G610A:p.G204S,SLTM:ENST00000558486.1:exon5:c.G364A:p.G122S,
15	65157774	G	T	TTCCATCCCC,CTGCTCCTCC	exonic	PLEKHO2	nonsynonymous SNV	PLEKHO2:ENST00000323544.4:exon6:c.G1160T:p.R387L,
15	78369750	A	T	CGCGATGTCC,AGTGGCCGAG	exonic	TBC1D2B	stopgain	TBC1D2B:ENST00000300584.3:exon1:c.T245A:p.L82X,TBC1D2B:ENST00000409931.3:exon1:c.T245A:p.L82X,
15	81633828	C	A	ACGTTGAACG,TGGGAGACAT	exonic	TMC3	nonsynonymous SNV	TMC3:ENST00000558726.1:exon16:c.G1750T:p.A584S,TMC3:ENST00000359440.5:exon16:c.G1747T:p.A583S,
15	84488635	C	T	GTATCAGGGG,ATTACTATGA	exonic	ADAMTSL3	nonsynonymous SNV	ADAMTSL3:ENST00000567476.1:exon6:c.C436T:p.H146Y,ADAMTSL3:ENST00000286744.5:exon6:c.C436T:p.H146Y,
16	3304494	T	C	TCTAGCGCCC,GCAGGGGCCG	exonic	MEFV	nonsynonymous SNV	MEFV:ENST00000542898.1:exon2:c.A574G:p.R192G,MEFV:ENST00000538326.1:exon2:c.A574G:p.R192G,MEFV:ENST00000537682.1:exon2:c.A574G:p.R192G,MEFV:ENST00000570511.1:exon2:c.A574G:p.R192G,MEFV:ENST00000219596.1:exon2:c.A574G:p.R192G,
16	3778993	C	A	TGCCACTGCC,GGGAGGCATG	exonic	CREBBP	nonsynonymous SNV	CREBBP:ENST00000382070.3:exon30:c.G5941T:p.G1981W,CREBBP:ENST00000262367.5:exon31:c.G6055T:p.G2019W,
16	31283247	C	A	AACCCAAGAT,ACTGGTGAAG	exonic	ITGAM	stopgain	ITGAM:ENST00000287497.8:exon7:c.C638A:p.S213X,ITGAM:ENST00000544665.3:exon7:c.C638A:p.S213X,
16	32859394	A	T	ATCATCTCCA,GGACACCTCC	exonic	IGHV20R16-	nonsynonymous SNV	IGHV20R16-5:ENST00000567458.2:exon2:c.A275T:p.K92M,IGHV20R16-5:ENST00000560724.1:exon1:c.A218T:p.K73M,
16	33020686	C	T	AGGCTTGGTA,AGCCTGGGGG	exonic	IGHV30R16-	stopgain	IGHV30R16-8:ENST00000565407.2:exon2:c.C94T:p.Q32X,
16	49671394	G	A	ATGAAGGACT,CGTGGGCTGC	exonic	ZNF423	stopgain	ZNF423:ENST00000562871.1:exon3:c.C1489T:p.Q497X,ZNF423:ENST00000262383.2:exon5:c.C1669T:p.Q557X,ZNF423:ENST00000567169.1:exon2:c.C1318T:p.Q440X,ZNF423:ENST00000561648.1:exon4:c.C1669T:p.Q557X,ZNF423:ENST00000535559.1:exon2:c.C1318T:p.Q440X,ZNF423:ENST0000563
16	49671414	G	T	CACCACCGGA,ACTCTAGTTT	exonic	ZNF423	nonsynonymous SNV	ZNF423:ENST00000562871.1:exon3:c.C1469A:p.S490Y,ZNF423:ENST00000262383.2:exon5:c.C1649A:p.S550Y,ZNF423:ENST00000567169.1:exon2:c.C1298A:p.S433Y,ZNF423:ENST00000561648.1:exon4:c.C1649A:p.S550Y,ZNF423:ENST00000535559.1:exon2:c.C1298A:p.S433Y,ZNF423:ENST000005631
16	50709805	G	T	GGTCATGCTG,AGTTGGAGCT	exonic	SNX20	nonsynonymous SNV	SNX20:ENST00000330943.4:exon3:c.C158A:p.S53Y,SNX20:ENST00000300590.3:exon3:c.C158A:p.S53Y,SNX20:ENST00000423026.2:exon3:c.C158A:p.S53Y,SNX20:ENST00000568993.1:exon3:c.C158A:p.S53Y,
16	56906622	T	G	GATGGCACCT,CTTCGGAATG	exonic	SLC12A3	nonsynonymous SNV	SLC12A3:ENST00000563236.1:exon8:c.T1019G:p.F340C,SLC12A3:ENST00000262502.5:exon8:c.T1016G:p.F339C,SLC12A3:ENST00000566786.1:exon8:c.T1016G:p.F339C,SLC12A3:ENST00000438926.2:exon8:c.T1019G:p.F340C,
16	65032737	C	A	GTTCCCATCA,CAGAGTCAAT	exonic	CDH11	nonsynonymous SNV	CDH11:ENST00000268603.4:exon4:c.G251T:p.G84V,CDH11:ENST00000567934.1:exon4:c.G251T:p.G84V,CDH11:ENST00000394156.3:exon4:c.G251T:p.G84V,
16	87446772	G	A	GGCAGCTGGT,ATGGGGCTGC	exonic	ZCCHC14	nonsynonymous SNV	ZCCHC14:ENST00000268616.4:exon11:c.C1222T:p.H408Y,ZCCHC14:ENST00000568020.1:exon11:c.C1222T:p.H408Y,
16	88798288	C	T	ATGCGCTGCC,GATCACGTTC	exonic	PIEZO1	nonsynonymous SNV	PIEZO1:ENST00000301015.9:exon22:c.G3022A:p.G1008R,
17	3336679	C	G	AGCGCCACCA,GGAGAGACAG	exonic	OR1E2	nonsynonymous SNV	OR1E2:ENST00000248384.1:exon1:c.G457C:p.V153L,
17	3572605	C	A	TCATCTTCTA,CTGCTCGCCT	exonic	EMC6	stopgain	EMC6:ENST00000248378.5:exon2:c.C165A:p.Y55X,EMC6:ENST00000397133.2:exon2:c.C165A:p.Y55X,
17	7578416	C	A	CGCCTCACAA,CTCCGTCATG	exonic	TP53	nonsynonymous SNV	TP53:ENST00000445888.2:exon5:c.G514T:p.V172F,TP53:ENST00000514944.1:exon4:c.G235T:p.V79F,TP53:ENST00000413465.2:exon4:c.G514T:p.V172F,TP53:ENST00000359597.4:exon4:c.G514T:p.V172F,TP53:ENST00000455263.2:exon5:c.G514T:p.V172F,TP53:ENST00000269305.4:exon5:c.G514T:p.V
17	9705758	C	T	AGAAACATTC,TGAAGGCTTG	exonic	RP11-477N12	nonsynonymous SNV	RP11-477N12.3:ENST00000399363.4:exon5:c.G827A:p.R276K,
17	10305019	C	G	TTTCAGTCAC,TCTTTGATTT	exonic	MYH8	nonsynonymous SNV	MYH8:ENST00000403437.2:exon23:c.G2772C:p.E924D,
17	10395777	C	A	CTGCTCTTCA,CCTCAGCTTG	exonic	MYH1	nonsynonymous SNV	MYH1:ENST00000226207.5:exon40:c.G5776T:p.V1926L,
17	18024015	G	A	GCCCAGCCAC,CGCTCGCAGC	exonic	MYO15A	nonsynonymous SNV	MYO15A:ENST00000205890.5:exon2:c.G1901A:p.R634H,
17	18167807	G	A	GCTGTCTGCC,TGGTTGCTCG	exonic	MIEF2	nonsynonymous SNV	MIEF2:ENST00000323019.4:exon4:c.G1094A:p.R365H,MIEF2:ENST00000395706.2:exon4:c.G1127A:p.R376H,
17	21438692	G	T	TGCCCACCTG,GCAAAGCTTC	exonic	C17orf51	nonsynonymous SNV	C17orf51:ENST00000391411.5:exon2:c.C535A:p.P179T,
17	27417925	C	T	ACGCTGAAGG,GGCTCAGCTG	exonic	MYO18A	nonsynonymous SNV	MYO18A:ENST00000533112.1:exon34:c.G5096A:p.R1699H,MYO18A:ENST00000354329.4:exon34:c.G5207A:p.R1736H,MYO18A:ENST00000531253.1:exon35:c.G5207A:p.R1736H,MYO18A:ENST00000527372.1:exon35:c.G5207A:p.R1736H,
17	29665114	T	C	AGCAAACGAG,GTCTCATGGG	exonic	NF1	nonsynonymous SNV	NF1:ENST00000444181.2:exon2:c.T155C:p.V52A,NF1:ENST00000356175.3:exon44:c.T6713C:p.V2238A,NF1:ENST00000456735.2:exon36:c.T5711C:p.V1904A,NF1:ENST00000358273.4:exon45:c.T6776C:p.V2259A,
17	31322490	T	G	GTGAGCTGCC,GTCATCCCAA	exonic	SPACA3	nonsynonymous SNV	SPACA3:ENST00000269053.3:exon2:c.T98G:p.L33R,
17	42945720	T	C	TAATGTCACC,ATGGGAGAAG	splicing	EFTUD2(ENS	.	.
17	54431274	G	T	TCCTGTATCA,TACACCACAG	exonic	ANKFN1	nonsynonymous SNV	ANKFN1:ENST00000566473.2:exon5:c.G477T:p.Q159H,ANKFN1:ENST00000318698.2:exon5:c.G477T:p.Q159H,
17	56540471	G	C	GCCTCTGTAA,ACGGAGATGT	exonic	HSF5	nonsynonymous SNV	HSF5:ENST00000323777.3:exon4:c.C1214G:p.S405C,
17	59761412	T	A	CAGCTAACTC,CTTTGTTTGT	exonic	BRIP1	stopgain	BRIP1:ENST00000259008.2:exon20:c.A2995T:p.R999X,
17	60765658	G	T	AGTGTTTTCA,GTCAGGGCCC	exonic	MRC2	nonsynonymous SNV	MRC2:ENST00000303375.5:exon21:c.G2955T:p.Q985H,
17	72999477	G	T	CCAGCTGTGC,AGATGGAGGC	exonic	CDR2L	stopgain	CDR2L:ENST00000337231.5:exon5:c.G706T:p.E236X,
17	73953594	C	A	AGAATGAACT,CTGGGTTTCA	exonic	ACOX1	stopgain	ACOX1:ENST00000293217.5:exon4:c.G484T:p.E162X,ACOX1:ENST00000301608.4:exon4:c.G484T:p.E162X,ACOX1:ENST00000537812.1:exon4:c.G370T:p.E124X,
17	73953595	C	T	GAATGAACTC,TGGGTTTCAG	exonic	ACOX1	nonsynonymous SNV	ACOX1:ENST00000573078.1:exon5:c.G644A:p.R215K,
17	79650824	C	A	CTCGGCGCCG,GGCTCAAGGC	exonic	ARL16	nonsynonymous SNV	ARL16:ENST00000397498.4:exon1:c.G32T:p.R11L,
18	641491	G	A	CTGGGTGTCT,AACTGGCAAA	exonic	CLUL1	nonsynonymous SNV	CLUL1:ENST00000581619.1:exon7:c.G1234A:p.E412K,CLUL1:ENST00000400606.2:exon7:c.G1159A:p.E387K,CLUL1:ENST00000338387.7:exon7:c.G1159A:p.E387K,CLUL1:ENST00000540035.1:exon9:c.G1315A:p.E439K,CLUL1:ENST00000579494.1:exon7:c.G1159A:p.E387K,
18	5419739	G	C	GCCGAGATGG,CGTGACTTCT	exonic	EPB41L3	nonsynonymous SNV	EPB41L3:ENST00000341928.2:exon12:c.C1477G:p.P493A,EPB41L3:ENST00000342933.3:exon12:c.C1477G:p.P493A,EPB41L3:ENST00000400111.3:exon12:c.C1531G:p.P511A,EPB41L3:ENST00000544123.1:exon12:c.C1531G:p.P511A,EPB41L3:ENST00000540638.2:exon12:c.C1531G:p.P511A,
18	8705783	C	A	CACCGTGCGC,CTCGCCCGCC	exonic	SOGA2	nonsynonymous SNV	SOGA2:ENST00000306329.11:exon1:c.C125A:p.P42H,
18	14763859	C	A	GAACAGTCAA,AGAAGAAACA	exonic	ANKRD30B	nonsynonymous SNV	ANKRD30B:ENST00000447268.2:exon7:c.C995A:p.T332K,ANKRD30B:ENST00000358984.4:exon7:c.C995A:p.T332K,ANKRD30B:ENST00000580206.1:exon7:c.C995A:p.T332K,
18	33048625	C	A	CCGGTGACGA,GTCAGAGGGC	exonic	INO80C	nonsynonymous SNV	INO80C:ENST00000590757.1:exon2:c.G238T:p.V80F,INO80C:ENST00000586489.1:exon5:c.G364T:p.V122F,INO80C:ENST00000334598.7:exon5:c.G529T:p.V177F,INO80C:ENST00000441607.2:exon7:c.G637T:p.V213F,
18	55024556	G	C	AAAAAGCTTT,ACGGGGCCAT	exonic	ST8SIA3	nonsynonymous SNV	ST8SIA3:ENST00000324000.3:exon3:c.G715C:p.D239H,
19	1912330	G	T	AAGCGGGTGC,GCCCAGCCGC	exonic	ADAT3	nonsynonymous SNV	ADAT3:ENST00000602400.1:exon2:c.G236T:p.R79L,ADAT3:ENST00000329478.2:exon2:c.G284T:p.R95L,
19	4298049	C	A	TCTACGGCCG,CCAGCACACG	exonic	TMIGD2	nonsynonymous SNV	TMIGD2:ENST00000595645.1:exon2:c.G340T:p.A114S,TMIGD2:ENST00000301272.2:exon2:c.G340T:p.A114S,
19	21607683	A	G	GACAAACCCT,CAAATGTGAA	exonic	ZNF493	nonsynonymous SNV	ZNF493:ENST00000355504.4:exon4:c.A1838G:p.Y613C,ZNF493:ENST00000392288.2:exon4:c.A2222G:p.Y741C,
19	22499193	T	C	GGAGAAACCC,ACAAATGCGA	exonic	ZNF729	nonsynonymous SNV	ZNF729:ENST00000357491.6:exon5:c.T2890C:p.Y964H,ZNF729:ENST00000601693.1:exon4:c.T2974C:p.Y992H,
19	22574536	G	C	TGTGAGGACT,GTTAAAAGCT	exonic	ZNF98	nonsynonymous SNV	ZNF98:ENST00000357774.5:exon4:c.C1501G:p.Q501E,
19	22574537	G	T	GTGAGGACTG,TTAAAAGCTT	exonic	ZNF98	nonsynonymous SNV	ZNF98:ENST00000357774.5:exon4:c.C1500A:p.N500K,
19	33291146	T	C	CATCAGATTG,TGCAGTTGGA	exonic	TDRD12	nonsynonymous SNV	TDRD12:ENST00000444215.2:exon17:c.T1832C:p.V611A,
19	33370231	C	T	TCGGATTCTT,GGTTTTCCCT	exonic	CEP89	nonsynonymous SNV	CEP89:ENST00000305768.5:exon19:c.G2189A:p.R730Q,
19	33792748	G	C	GCGGGTGCGG,TGCGAGGGCG	exonic	CEBPA	nonsynonymous SNV	CEBPA:ENST00000498907.2:exon1:c.C573G:p.H191Q,
19	35831786	C	T	TCTTCCAGAT,CTCCCAAGAA	exonic	CD22	nonsynonymous SNV	CD22:ENST00000594125.1:exon2:c.C181T:p.P61S,CD22:ENST00000419549.2:exon6:c.C736T:p.P246S,CD22:ENST00000085219.5:exon7:c.C1252T:p.P418S,CD22:ENST00000341773.6:exon5:c.C721T:p.P241S,CD22:ENST00000536635.2:exon6:c.C988T:p.P330S,CD22:ENST00000544992.2:exon7:c.C1252T
19	35941565	G	T	CAGGGGTGTG,GTCAAGGAGA	exonic	FFAR2	nonsynonymous SNV	FFAR2:ENST00000246549.2:exon1:c.G949T:p.G317C,FFAR2:ENST00000599180.2:exon2:c.G949T:p.G317C,
19	39994719	C	T	AGTGGTGTGC,GAGCAGCCTG	exonic	DLL3	nonsynonymous SNV	DLL3:ENST00000356433.5:exon5:c.C661T:p.R221X,DLL3:ENST00000205143.4:exon5:c.C661T:p.R221X,
19	42343016	C	A	CCATGTTCCT,ATCAGAAGCC	exonic	LYPD4	nonsynonymous SNV	LYPD4:ENST00000330743.3:exon3:c.C150T:p.M50I,
19	42801384	G	A	GGTGCCATCT,AGTGCACAAA	exonic	PAFAH1B3	nonsynonymous SNV	PAFAH1B3:ENST00000262890.3:exon5:c.C542T:p.S181L,PAFAH1B3:ENST00000538771.1:exon6:c.C542T:p.S181L,
19	42819171	G	T	TGGAGTATGA,ATGGTCCTCA	exonic	TMEM145	nonsynonymous SNV	TMEM145:ENST00000301204.3:exon6:c.G447T:p.E149D,TMEM145:ENST00000598766.1:exon6:c.G519T:p.E173D,
19	51170947	C	T	CCCAGCCCGG,CCTGTACCCC	exonic	SHANK1	nonsynonymous SNV	SHANK1:ENST00000391814.1:exon24:c.G4294A:p.A1432T,SHANK1:ENST00000293441.1:exon22:c.G4270A:p.A1424T,SHANK1:ENST00000359082.3:exon21:c.G4243A:p.A1415T,SHANK1:ENST00000391813.1:exon8:c.G2431A:p.A811T,
19	52580333	G	T	GAATGTCAAA,ATCCCTGAAA	exonic	ZNF841	nonsynonymous SNV	ZNF841:ENST00000389534.4:exon5:c.C20A:p.S7Y,ZNF841:ENST00000594440.1:exon5:c.C20A:p.S7Y,ZNF841:ENST00000594295.1:exon4:c.C20A:p.S7Y,
19	54744327	C	A	GGATGGGCTG,CCCTTCTTTG	exonic	LILRA6	nonsynonymous SNV	LILRA6:ENST00000245621.5:exon6:c.G1081T:p.A361S,LILRA6:ENST00000396365.2:exon6:c.G1081T:p.A361S,LILRA6:ENST00000419410.2:exon6:c.G1081T:p.A361S,
19	55143514	G	A	AGGAGAAGAT,AACACCCACA	exonic	LILRB1	nonsynonymous SNV	LILRB1:ENST00000396327.3:exon5:c.G487A:p.E163K,LILRB1:ENST00000427581.2:exon4:c.G595A:p.E199K,LILRB1:ENST00000421584.1:exon4:c.G487A:p.E163K,LILRB1:ENST00000448689.1:exon4:c.G487A:p.E163K,LILRB1:ENST00000434867.2:exon5:c.G487A:p.E163K,LILRB1:ENST00000396315.1:exo
19	56538535	G	A	ACCAGGGAAT,TTCTCCTACG	exonic	NLRP5	nonsynonymous SNV	NLRP5:ENST00000390649.3:exon7:c.G936A:p.M312I,
19	57175679	G	C	GCACGCGCCG,TGCTGGGTCA	exonic	ZNF835	nonsynonymous SNV	ZNF835:ENST00000537055.2:exon2:c.C888G:p.H296Q,
19	58117052	A	T	CTCTTCTTTC,GGTTGCTGGT	splicing	ZNF530(ENS	.	.
20	2777089	C	T	CTCAGGCTCC,CTGGGGACAC	exonic	CPXM1	nonsynonymous SNV	CPXM1:ENST00000380605.2:exon9:c.G1046A:p.G349E,
20	9417794	A	G	GTGGAAGCCA,GAAAGGTGAG	exonic	PLCB4	nonsynonymous SNV	PLCB4:ENST00000334005.3:exon28:c.A2723G:p.K908R,PLCB4:ENST00000278655.4:exon27:c.A2723G:p.K908R,PLCB4:ENST00000378501.2:exon26:c.A2723G:p.K908R,PLCB4:ENST00000378473.3:exon29:c.A2759G:p.K920R,PLCB4:ENST00000378493.1:exon26:c.A2723G:p.K908R,PLCB4:ENST0000041
20	20079336	C	T	AGTGTGTGCT,AAGAGTGAAC	exonic	C20orf26	nonsynonymous SNV	C20orf26:ENST00000472660.1:exon6:c.C425T:p.S142L,C20orf26:ENST00000442372.2:exon2:c.C14T:p.S5L,C20orf26:ENST00000340348.6:exon7:c.C599T:p.S200L,C20orf26:ENST00000451767.2:exon7:c.C737T:p.S246L,C20orf26:ENST00000377306.1:exon8:c.C737T:p.S246L,C20orf26:ENST00000245957.5
20	31631145	A	G	GCCATTGAAT,CAACCTGGCT	exonic	BPIFB6	nonsynonymous SNV	BPIFB6:ENST00000349552.1:exon14:c.A1301G:p.Y434C,
20	42885940	G	A	CATCATCAGT,ACTATGACCA	exonic	GDAP1L1	nonsynonymous SNV	GDAP1L1:ENST00000342560.5:exon2:c.G328A:p.D110N,GDAP1L1:ENST00000438466.1:exon2:c.G328A:p.D110N,GDAP1L1:ENST00000372952.3:exon2:c.G328A:p.D110N,
20	47887160	C	A	AGGCCCTGGT,TTCAAAGCTT	exonic	ZNFX1	nonsynonymous SNV	ZNFX1:ENST00000371754.4:exon3:c.G1189T:p.D397Y,ZNFX1:ENST00000396105.1:exon3:c.G1189T:p.D397Y,ZNFX1:ENST00000371752.1:exon3:c.G1189T:p.D397Y,ZNFX1:ENST00000371744.1:exon3:c.G1189T:p.D397Y,ZNFX1:ENST00000455070.1:exon1:c.G601T:p.D201Y,
20	49366363	G	A	TATTCTCCCA,AAACGCATCG	exonic	PARD6B	nonsynonymous SNV	PARD6B:ENST00000371610.2:exon3:c.G457A:p.E153K,
20	58330263	G	T	TTGCAACCCC,ATGGAGGACC	exonic	PHACTR3	nonsynonymous SNV	PHACTR3:ENST00000541461.1:exon4:c.G262T:p.D88Y,PHACTR3:ENST00000361300.4:exon4:c.G262T:p.D88Y,PHACTR3:ENST00000359926.3:exon4:c.G376T:p.D126Y,PHACTR3:ENST00000355648.4:exon4:c.G262T:p.D88Y,PHACTR3:ENST00000395639.4:exon4:c.G262T:p.D88Y,PHACTR3:ENST00003
20	58348423	C	G	GTCTCCGCAT,TCACCACGGT	exonic	PHACTR3	nonsynonymous SNV	PHACTR3:ENST00000541461.1:exon6:c.C718G:p.L240V,PHACTR3:ENST00000361300.4:exon5:c.C508G:p.L170V,PHACTR3:ENST00000359926.3:exon6:c.C832G:p.L278V,PHACTR3:ENST00000355648.4:exon6:c.C718G:p.L240V,PHACTR3:ENST00000395639.4:exon5:c.C508G:p.L170V,PHACTR3:ENST000
20	61867671	T	A	CGGGGCCACC,TGTCCAGGGG	exonic	BIRC7	nonsynonymous SNV	BIRC7:ENST00000217169.3:exon1:c.T223A:p.L75M,BIRC7:ENST00000342412.6:exon1:c.T223A:p.L75M,
21	41496174	A	C	GATGCCGTTC,GCTTGAGAGG	exonic	DSCAM	nonsynonymous SNV	DSCAM:ENST00000400454.1:exon20:c.T3644G:p.L1215R,DSCAM:ENST00000404019.2:exon16:c.T2900G:p.L967R,
21	44492239	T	A	CTTCGCCGAG,GTGGCCCTGA	exonic	CBS	nonsynonymous SNV	CBS:ENST00000359624.3:exon3:c.A65T:p.H22L,CBS:ENST00000398165.3:exon3:c.A65T:p.H22L,CBS:ENST00000398158.1:exon3:c.A65T:p.H22L,CBS:ENST00000398168.1:exon3:c.A65T:p.H22L,CBS:ENST00000352178.5:exon3:c.A65T:p.H22L,CBS:ENST00000441030.1:exon3:c.A65T:p.H22L,
21	47422618	T	A	CGCCAGATC,GCATAGGTGG	exonic	COL6A1	nonsynonymous SNV	COL6A1:ENST00000361866.3:exon33:c.T2428A:p.C810S,
22	18387421	C	A	GGCTCCAGCA,AGAACTTGCC	exonic	MICAL3	nonsynonymous SNV	MICAL3:ENST00000414725.2:exon4:c.G449T:p.C150F,MICAL3:ENST00000441493.2:exon3:c.G449T:p.C150F,MICAL3:ENST00000585038.1:exon2:c.G449T:p.C150F,MICAL3:ENST00000429452.1:exon3:c.G449T:p.C150F,MICAL3:ENST00000400561.2:e
22	19394797	G	A	GTTCACACAT,CTGGGAAGAA	exonic	HIRA	nonsynonymous SNV	HIRA:ENST00000541063.1:exon4:c.C80T:p.A27V,HIRA:ENST00000340170.4:exon4:c.C212T:p.A71V,HIRA:ENST00000263208.5:exon4:c.C212T:p.A71V,HIRA:ENST00000546308.1:exon5:c.C80T:p.A27V,
22	22599577	G	A	CCCTTCTCTG,ATCCAAAGAT	exonic	VPREB1	nonsynonymous SNV	VPREB1:ENST00000302273.2:exon2:c.G263A:p.G88E,VPREB1:ENST00000403807.3:exon2:c.G266A:p.G89E,
22	39994226	G	C	CCAGCCGTGC,ACGACATGGA	exonic	CACNA1I	nonsynonymous SNV	CACNA1I:ENST00000400164.3:exon4:c.G307C:p.D103H,CACNA1I:ENST00000401624.1:exon2:c.G307C:p.D103H,CACNA1I:ENST00000404898.1:exon2:c.G307C:p.D103H,CACNA1I:ENST00000336649.4:exon4:c.G307C:p.D103H,CACNA1I:ENST00000402142.3:exon2:c.G307C:p.D103H,CACNA1I:ENST0000
22	50303672	G	C	CGATGATGGC,AAGGCTGACA	exonic	ALG12	nonsynonymous SNV	ALG12:ENST00000330817.6:exon5:c.C534G:p.F178L,
X	1748826	C	T	TATGTGTGAC,TTGGTGGTAA	exonic	ASMT	nonsynonymous SNV	ASMT:ENST00000381233.3:exon5:c.C556T:p.L186F,ASMT:ENST00000381229.4:exon5:c.C556T:p.L186F,ASMT:ENST00000381241.3:exon5:c.C556T:p.L186F,
X	41055561	G	A	GATGTTGCAT,GGACACCGGC	exonic	USP9X	nonsynonymous SNV	USP9X:ENST00000378308.2:exon27:c.G4035A:p.M1345I,USP9X:ENST00000324545.8:exon27:c.G4035A:p.M1345I,
X	41055562	G	T	ATGTTGCATG,GACACCGGCC	exonic	USP9X	nonsynonymous SNV	USP9X:ENST00000378308.2:exon27:c.G4036T:p.G1346X,USP9X:ENST00000324545.8:exon27:c.G4036T:p.G1346X,
X	41204494	A	T	TCAGATTCGT,GAATAGTCGA	exonic	DDX3X	stopgain	DDX3X:ENST00000457138.2:exon10:c.A1039T:p.R347X,DDX3X:ENST00000399959.2:exon11:c.A1087T:p.R363X,

X	41586623	T	A	TGGATTGCCA,AAGCCGCTAT	exonic	GPR82	nonsynonymous SNV	GPR82:ENST00000302548.4:exon3:c.T344A:p.I115K,
X	66765355	C	A	GCAGTCGGCC,TGGAGTGCCA	exonic	AR	nonsynonymous SNV	AR:ENST00000396044.3:exon1:c.C367A:p.L123M,AR:ENST00000374690.3:exon1:c.C367A:p.L123M,AR:ENST00000514029.1:exon1:c.C367A:p.L123M,AR:ENST00000504326.1:exon1:c.C367A:p.L123M,
X	68749626	G	T	CAACAAGCCC,CCCTGCTGCC	exonic	FAM155B	nonsynonymous SNV	FAM155B:ENST00000252338.4:exon3:c.G1246T:p.A416S,
X	107977066	G	T	CCCCTCCCCA,GAACTTTCCA	exonic	IRS4	nonsynonymous SNV	IRS4:ENST00000372129.2:exon1:c.C2509A:p.L837M,
X	107977067	G	T	CCCTCCCCAG,AACTTTCCAG	exonic	IRS4	nonsynonymous SNV	IRS4:ENST00000372129.2:exon1:c.C2508A:p.F836L,
X	144904814	C	A	TACAATGAAT,CTGCTCTCAA	exonic	SLITRK2	nonsynonymous SNV	SLITRK2:ENST00000335565.4:exon5:c.C871A:p.P291T,SLITRK2:ENST00000413937.2:exon3:c.C871A:p.P291T,SLITRK2:ENST00000447897.2:exon2:c.C871A:p.P291T,SLITRK2:ENST00000434188.2:exon3:c.C871A:p.P291T,SLITRK2:ENST00000370490.1:exon1:c.C871A:p.P291T,SLITRK2:ENST000004285[illegible]
X	151900103	C	A	GTCCTCCCTC,CATCAGATGC	exonic	MAGEA12	nonsynonymous SNV	MAGEA12:ENST00000393900.3:exon3:c.G698T:p.G233V,MAGEA12:ENST00000357916.4:exon2:c.G698T:p.G233V,MAGEA12:ENST00000393869.3:exon3:c.G698T:p.G233V,

Cytoband	COSMIC
1p36.33	.
1p36.32	.
1p36.31	.
1p36.12	.
1p35.3	.
1p34.3	.
1p34.3	.
1p34.3	.
1p32.3	.
1p31.3	.
1p31.1	.
1p31.1	.
1p31.1	.
1p22.2	.
1p22.1	.
1p21.3	PAP2D;basechange=c.498G>T;AAchange
1p21.1	.
1p21.1	.
1p13.2	BCAS2;basechange=c.445G>A;AAchange
1p12	.
1q21.3	.
1q21.3	.
1q21.3	.
1q22	.
1q23.1	.
1q23.1	.
1q23.1	.
1q23.2	.
1q23.3	.
1q24.1	.
1q24.2	.
1q25.2	.
1q25.3	.
1q31.3	.
1q31.3	.
1q31.3	.
1q32.1	.
1q41	.
1q41	USH2A;basechange=c.3571G>T;AAchang
1q41	TGFB2_ENST00000366929;basechange=
1q42.12	.
1q42.13	.
1q43	.
1q43	.
1q44	.
1q44	.
1q44	.
1q44	OR2T8;basechange=c.889G>A;AAchange
1q44	.
1q44	.
2p25.3	.
2p25.3	.
2p25.3	.
2p24.3	.
2p24.1	.
2p24.1	.
2p23.3	.
2p23.3	.
2p23.1	.
2p11.2	.
2q11.2	.
2q12.1	.
2q12.1	.
2q21.1	.
2q21.2	.
2q23.3	NEB_ENST00000397345;basechange=c.
2q23.3	.
2q24.2	.
2q31.1	.
2q31.2	.
2q31.2	.
2q31.2	.
2q31.2	.
2q32.2	COL5A2;basechange=c.1834G>T;AAchar
2q32.2	.
2q33.1	.
2q33.1	.
2q33.1	.
2q33.3	.
2q33.3	.
2q34	.
2q35	.
2q35	.
2q36.3	.
2q37.1	.
2q37.1	.
2q37.3	.
2q37.3	.
3p25.3	.
3p25.1	.
3p22.1	.
3p13	.
3p12.3	.
3q11.1	.
3q11.2	.
3q11.2	.
3q12.1	.
3q13.2	.
3q13.2	SPICE1;basechange=c.1855G>A;AAchan
3q13.31	.
3q21.3	.
3q22.1	.
3q22.2	.
3q22.2	EPHB1;basechange=c.144C>T;AAchange
3q22.2	.
3q22.3	.
3q23	.
3q24	.
3q24	.
3q24	.
3q25.1	.
3q25.31	.
3q26.1	.
3q26.1	.
3q26.1	.
3q26.1	SI;basechange=c.1639G>A;AAchange=p.
3q26.1	.
3q27.1	.
3q27.1	.
3q27.2	.
3q28	FGF12;basechange=c.173G>A;AAchange
3q29	.
4p16.1	.
4p15.31	.
4p11	.
4p11	.
4q13.1	.
4q13.3	.
4q13.3	.
4q21.21	.
4q24	TACR3;basechange=c.1297G>T;AAchang
4q25	.
4q26	.
4q26	.
4q28.3	.
4q31.21	.
4q31.22	.
4q31.3	.
4q32.1	.
4q32.1	.
4q33	.

4q34.2	.
5p15.33	.
5p15.32	.
5p14.3	.
5p13.2	.
5p13.1	.
5p13.1	.
5q11.2	.
5q12.3	.
5q13.3	.
5q15	.
5q21.1	.
5q23.3	.
5q23.3	.
5q31.1	.
5q31.3	.
5q32	.
5q33.1	.
5q33.1	.
5q34	.
5q35.1	.
6p25.3	.
6p25.3	.
6p22.3	.
6p22.2	.
6p22.1	.
6p22.1	.
6p21.33	.
6p21.33	.
6p21.31	.
6p21.1	.
6p21.1	.
6p21.1	.
6p12.1	.
6q12	.
6q14.1	.
6q15	.
6q16.1	.
6q22.1	.
6q22.1	.
6q22.31	.
6q22.31	.
6q23.1	.
6q23.2	.
6q25.2	.
6q25.2	.
6q25.3	.
6q25.3	.
6q27	.
7p22.1	.
7p21.3	.
7p21.3	.
7p21.3	.
7p21.1	.
7p15.3	DNAH11;basechange=c.6536T>C;AAchan
7p15.2	.
7p14.1	.
7p13	.
7p13	.
7p12.3	.
7q11.22	.
7q11.23	.
7q21.11	.
7q21.11	.
7q21.2	.
7q22.1	.
7q32.1	.
7q32.2	.
7q33	.
7q33	.
7q34	.
7q36.1	.
7q36.1	.
7q36.3	.
8p12	.
8p11.21	.
8q12.1	.
8q13.1	.
8q21.11	.
8q23.1	.
8q23.1	.
8q23.1	.
8q23.3	CSMD3;basechange=c.1661G>A;AAchan
8q24.12	ENPP2_ENST00000522826;basechange=
8q24.22	.
8q24.23	.
8q24.23	.
8q24.3	.
8q24.3	.
9p24.2	.
9p22.3	.
9p21.2	.
9p13.2	.
9p11.2	.
9q21.32	.
9q22.32	.
9q22.33	.
9q22.33	.
9q22.33	.
9q31.1	.
9q31.1	.
9q31.1	.
9q32	.
10p14	.
10p13	.
10p13	.
10p13	.
10p12.33	.
10p12.31	.
10p12.1	.
10q11.22	.
10q11.23	.
10q21.2	.
10q22.2	.
10q23.1	.
10q23.1	.
10q25.3	.
10q25.3	.
10q26.3	.
10q26.3	.
11p15.5	.
11p15.4	.
11p15.4	.
11p15.4	.
11p15.4	.
11p15.2	.
11p15.1	.
11p15.1	.
11p14.2	ANO3;basechange=c.373C>T;AAchange=
11p13	.
11p12	LRRC4C;basechange=c.803C>A;AAchang
11p12	.
11p11.2	.
11p11.2	C1QTNF4;basechange=c.183C>A;AAchan
11p11.12	.
11q11	.
11q12.1	.
11q12.1	.
11q12.1	.
11q12.1	.
11q12.1	.
11q12.1	.
11q22.3	PDGFD_ENST00000393158;basechange
11q24.1	.

11q24.2	.
11q24.2	.
11q24.2	.
12p13.33	KDM5A;basechange=c.3470G>A;AAchan
12p13.31	.
12p13.2	.
12p12.1	.
12q12	.
12q12	.
12q12	.
12q12	.
12q12	.
12q13.12	.
12q13.13	.
12q13.2	.
12q15	.
12q21.2	.
12q21.31	.
12q21.31	.
12q21.33	.
12q22	.
12q23.1	.
12q23.2	.
12q23.3	.
12q24.13	.
12q24.13	.
12q24.31	.
12q24.33	.
13q12.12	.
13q12.3	.
13q13.1	.
13q13.3	.
13q14.11	.
13q21.33	.
13q32.1	.
13q32.1	.
13q34	COL4A2;basechange=c.4229G>A;AAchar
14q11.2	.
14q11.2	.
14q11.2	.
14q21.1	.
14q21.2	.
14q21.3	.
14q22.2	.
14q23.3	.
14q24.2	.
14q24.2	.
14q32.12	.
14q32.12	.
14q32.33	.
15q11.2	.
15q11.2	.
15q11.2	.
15q11.2	.
15q15.3	.
15q21.3	.
15q21.3	.
15q22.1	.
15q22.31	.
15q25.1	.
15q25.1	.
15q25.2	.
16p13.3	.
16p13.3	.
16p11.2	.
16p11.2	.
16p11.2	.
16q12.1	.
16q12.1	.
16q12.1	.
16q13	.
16q21	.
16q24.2	.
16q24.3	.
17p13.2	.
17p13.2	.
17p13.1	TP53_ENST00000545858;basechange=c
17p13.1	.
17p13.1	.
17p13.1	.
17p11.2	.
17p11.2	.
17p11.2	.
17q11.2	.
17q11.2	.
17q11.2	.
17q21.31	.
17q22	.
17q22	.
17q23.2	.
17q23.2	.
17q25.1	.
17q25.1	.
17q25.1	.
17q25.3	.
18p11.32	.
18p11.31	.
18p11.22	.
18p11.21	.
18q12.2	.
18q21.31	.
19p13.3	.
19p13.3	.
19p12	.
19p12	.
19p12	.
19p12	.
19q13.11	.
19q13.11	.
19q13.11	CEBPA;basechange=c.573C>T;AAchange
19q13.12	.
19q13.12	.
19q13.2	DLL3;basechange=c.661C>T;AAchange=
19q13.2	.
19q13.2	.
19q13.2	TMEM145;basechange=c.447G>T;AAcha
19q13.33	.
19q13.41	.
19q13.42	.
19q13.42	.
19q13.43	.
19q13.43	.
19q13.43	.
20p13	.
20p12.2	PLCB4;basechange=c.2723A>T;AAchang
20p11.23	.
20q11.21	.
20q13.12	.
20q13.13	.
20q13.13	.
20q13.32	.
20q13.32	.
20q13.33	.
21q22.2	.
21q22.3	.
21q22.3	COL6A1;basechange=c.2428T>G;AAchar
22q11.21	.
22q11.21	.
22q11.22	.
22q13.1	.
22q13.33	.
Xp22.33	.
Xp11.4	.
Xp11.4	.

Xp11.4	.
Xp11.4	.
Xq12	.
Xq13.1	FAM155B;basechange=c.1246G>T;AAcha
Xq22.3	IRS4;basechange=c.2509C>A;AAchange=
Xq22.3	.
Xq27.3	.
Xq28	.

C000-6KNGDL

Chromosome	Position (hg19)	Reference allele	Mutant allele	Sequence context	ANNOVAR function	Gene	Mutation type	ANNOVAR output (alternative transcripts)	Cytoband	COSMIC
1	1355551	C	A	CCCGCAGCGG,AGCCACGAGC	exonic	ANKRD65	nonsynonymous SNV	[illegible]	1p36.33	.
1	3329333	C	T	CTACGCCAAG,CCTCGCCCTT	exonic	PRDM16	nonsynonymous SNV	[illegible]	1p36.32	.
1	3646694	C	T	GGCAGCTACA,CCAACCTGGG	exonic	TP73	nonsynonymous SNV	[illegible]	1p36.32	.
1	3669112	G	A	GGAAAAGCCG,GCCTGTCCTC	exonic	CCDC27	nonsynonymous SNV	[illegible]	1p36.32	.
1	4772718	G	A	AGCAACAACG,GGAAGTCACC	exonic	AJAP1	nonsynonymous SNV	[illegible]	1p36.32	.
1	6145840	C	T	CTCACTGTTC,CATCTGTAAG	exonic	KCNAB2	nonsynonymous SNV	[illegible]	1p36.31	.
1	17752090	G	A	TGCAGCACAC,AGCCCGAGAC	exonic	RCC2	nonsynonymous SNV	[illegible]	1p36.13	.
1	18691784	C	T	GCTGCGAGCT,CGGCCCCCTA	exonic	IGSF21	nonsynonymous SNV	[illegible]	1p36.13	.
1	20640891	G	A	ACGAGGATTT,GAGCGGATCC	exonic	VWA5B1	nonsynonymous SNV	[illegible]	1p36.12	.
1	23403750	C	A	CTGGCAGCCA,CTGACAGTAA	exonic	KDM1A	nonsynonymous SNV	[illegible]	1p36.12	.
1	25553969	G	A	TTCCATGTCA,GTTTGATCTG	exonic	SYF2	nonsynonymous SNV	[illegible]	1p36.11	.
1	25553970	G	A	TCCATGTCAG,TTTGATCTGC	exonic	SYF2	nonsynonymous SNV	[illegible]	1p36.11	.
1	27023828	C	T	CCAGGGCTAC,CCGGGGGCGA	exonic	ARID1A	nonsynonymous SNV	[illegible]	1p36.11	.
1	29587133	C	T	GCCCCCAACT,CCATCGCGCC	exonic	PTPRU	nonsynonymous SNV	[illegible]	1p35.3	.
1	29587134	C	T	CCCCCAACTC,CATCGCGCCC	exonic	PTPRU	nonsynonymous SNV	[illegible]	1p35.3	.
1	31901870	C	A	GGTCATCACC,TCTACACCAT	exonic	SERINC2	nonsynonymous SNV	[illegible]	1p35.2	.
1	32198210	G	A	ACCACATCCT,GACCTGGGGA	exonic	BAI2	stopgain	[illegible]	1p35.2	.
1	33160876	C	T	GTCAGCACTT,CCGGAAATCG	exonic	SYNC	nonsynonymous SNV	[illegible]	1p35.1	.
1	35915575	A	C	AAGAAAAAGG,TAGGGTGATG	exonic	KIAA0319L	nonsynonymous SNV	[illegible]	1p34.3	.
1	35925910	A	T	ATTTACCTGA,AGTGTAGTTC	exonic	KIAA0319L	nonsynonymous SNV	[illegible]	1p34.3	.
1	36202603	G	A	AGGAGCACTG,GGTTATGGTC	exonic	CLSPN	nonsynonymous SNV	[illegible]	1p34.3	.
1	36226381	G	A	GAAACTACAG,GAGTGCATTA	exonic	CLSPN	nonsynonymous SNV	[illegible]	1p34.3	.
1	39907970	G	A	GAGAACCTGG,TGAGAAAATT	exonic	MACF1	nonsynonymous SNV	[illegible]	1p34.3	.
1	40777784	C	T	CCAGGAGGCC,CTGGGGAGCA	exonic	COL9A2	nonsynonymous SNV	[illegible]	1p34.2	.
1	42046538	G	A	ATGGTGTCTG,ACAGGCAGGC	exonic	HIVEP3	nonsynonymous SNV	[illegible]	1p34.2	.
1	43675708	C	T	ATTCATCCAT,ATTCATTGAA	exonic	WDR65	nonsynonymous SNV	[illegible]	1p34.2	.
1	45166651	G	A	GACCCAGGAG,AAGTGCAGGT	exonic	C1orf228	nonsynonymous SNV	[illegible]	1p34.1	.
1	45278136	T	G	TCAGCACTTT,GGAGGCCGAG	exonic	BTBD19	nonsynonymous SNV	[illegible]	1p34.1	.
1	46499789	C	A	AAGCAAGCAT,CCTGCTCCAC	exonic	MAST2	nonsynonymous SNV	[illegible]	1p34.1	.
1	49224710	C	T	CGCTCCAGCT,CTGCTGGAGG	exonic	BEND5	nonsynonymous SNV	[illegible]	1p33	.
1	54694472	G	A	CCGCCCATCG,ACCGTCCGAG	exonic	SSBP3	nonsynonymous SNV	[illegible]	1p32.3	.
1	55074634	C	T	CTGCAGGTAT,CTACTACAAC	exonic	ACOT11	nonsynonymous SNV	[illegible]	1p32.3	.
1	57420382	C	T	TGCCAATTCC,CAGTATTGGT	exonic	C8B	stopgain	[illegible]	1p32.2	.
1	57476424	C	A	CTGGGCTCAC,AAATGGATCA	exonic	DAB1	nonsynonymous SNV	[illegible]	1p32.2	.
1	57476425	A	T	TGGGCTCACC,AATGGATCAC	exonic	DAB1	nonsynonymous SNV	[illegible]	1p32.2	.
1	57611058	C	T	CGGACCCCTT,ACCTTTAAAC	exonic	DAB1	nonsynonymous SNV	[illegible]	1p32.2	.
1	63867980	C	T	TTACCCACCT,TTACAGCTTA	exonic	ALG6	nonsynonymous SNV	[illegible]	1p31.3	.
1	65877022	G	A	AGACCAAGTG,AAACCAGTTG	exonic	DNAJC6	stopgain	[illegible]	1p31.3	.
1	70488837	G	A	CCCTGGGAAA,GGGCCAGCGT	exonic	LRRC7	nonsynonymous SNV	[illegible]	1p31.1	.
1	71535037	G	A	AGAACTTCTT,AACGGGACCT	exonic	ZRANB2	nonsynonymous SNV	[illegible]	1p31.1	.
1	74507089	G	A	AGCCTTTTGG,ATTTTTCTTT	exonic	LRRIQ3	nonsynonymous SNV	[illegible]	1p31.1	LRRIQ3;basechange=c.1526C>T;AAchange=p.S509F;tissue=endometrium
1	75038023	G	A	CTCAGCATCA,ATCCCATTTC	exonic	C1orf173	nonsynonymous SNV	[illegible]	1p31.1	.
1	75038443	C	T	AACCTCTTTT,TCTCTTTGGC	exonic	C1orf173	nonsynonymous SNV	[illegible]	1p31.1	.
1	75622680	C	T	GCTGTCTCCA,CCATGTTAGA	exonic	LHX8	nonsynonymous SNV	[illegible]	1p31.1	.
1	75622758	G	A	TAGTTATATG,ATGGTAGGTA	exonic	LHX8	nonsynonymous SNV	[illegible]	1p31.1	.
1	77949026	G	A	AAATGTATTT,GAGAGGACAC	exonic	AK5	nonsynonymous SNV	[illegible]	1p31.1	.
1	79095541	C	T	GTCTATTTTT,ATGGCCATGT	exonic	IFI44L	nonsynonymous SNV	[illegible]	1p31.1	.
1	86362092	C	T	CCTCTTGATC,CTAGAGGTGA	exonic	COL24A1	nonsynonymous SNV	[illegible]	1p22.3	.
1	87043678	G	A	CGGGCTCATG,AGGAGCAAAC	exonic	CLCA4	nonsynonymous SNV	[illegible]	1p22.3	CLCA4;basechange=c.2045G>A;AAchange=p.G682E;tissue=autonomic_ganglia
1	91788732	C	T	TCTAGAAATT,CTTGCAGCCA	exonic	HFM1	nonsynonymous SNV	[illegible]	1p22.2	.
1	92645881	G	C	GCAGTCTGAT,AAAATGTGGC	exonic	KIAA1107	nonsynonymous SNV	[illegible]	1p22.1	.
1	94012454	C	T	AATGGGGGAT,CAGGGAGTTT	exonic	FNBP1L	nonsynonymous SNV	[illegible]	1p22.1	.
1	99771439	C	T	TCTGACAAAT,TCAAAAGAGC	exonic	LPPR4	nonsynonymous SNV	[illegible]	1p21.2	.
1	103412437	G	A	GGACCAGGAG,ACCCTGAGGT	exonic	COL11A1	nonsynonymous SNV	[illegible]	1p21.1	.
1	109391399	G	A	CAAATGGTAA,GAGAGTCCTG	exonic	AKNAD1	nonsynonymous SNV	[illegible]	1p13.3	.
1	109779094	C	T	GAGTTGGTCT,CTGTTCTAAT	exonic	SARS	nonsynonymous SNV	[illegible]	1p13.3	.
1	110018321	C	T	TTTGGCTATC,CTTCAGGTGA	exonic	SYPL2	nonsynonymous SNV	[illegible]	1p13.3	.
1	110037192	G	C	GGCGCGGTGG,TCATGCCTGA	exonic	CYB561D1	nonsynonymous SNV	[illegible]	1p13.3	.
1	114438070	G	A	CCAGAATCAG,GAGTTCTTGT	exonic	AP4B1	nonsynonymous SNV	[illegible]	1p13.2	.
1	114682571	G	A	ACTGCGAGGA,GCTGACAGAG	exonic	SYT6	nonsynonymous SNV	[illegible]	1p13.2	.
1	115164683	G	A	GGTTTTGGAG,GAGCTAAAAC	exonic	DENND2C	nonsynonymous SNV	[illegible]	1p13.2	.
1	115165607	C	T	TCTGGTTATA,CTTGGGTGCT	splicing	DENND2C(ENST00000393276.3[illegible]	.	.	1p13.2	.
1	118623910	C	T	AAATTCTGAT,CACAAATAGT	exonic	SPAG17	nonsynonymous SNV	[illegible]	1p12	.
1	120502126	C	T	CAATTAACCC,TGGAAGAGAA	splicing	NOTCH2(ENST00000256646.2:e[illegible]	.	.	1p12	.
1	120611960	C	T	TGCGCGGGGG,CGCGCAGCAC	exonic	NOTCH2	nonsynonymous SNV	[illegible]	1p11.2	NOTCH2;basechange=c.61G>A;AAchange=p.A21T;tissue=liver
1	151787791	G	A	CCTGCTGGAG,GGTCTTGACC	exonic	RORC	nonsynonymous SNV	[illegible]	1q21.3	.
1	152058185	G	A	TCCTGCTGTG,ATTCCTGTGC	exonic	TCHHL1	nonsynonymous SNV	[illegible]	1q21.3	.
1	152282959	G	A	CTCATGGCGG,ATCCTTGTCT	exonic	FLG	nonsynonymous SNV	[illegible]	1q21.3	FLG;basechange=c.4403C>T;AAchange=p.S1468F;tissue=lung
1	152329565	C	T	CCTTTCCTTT,CCAACTGTTT	exonic	FLG2	nonsynonymous SNV	[illegible]	1q21.3	.
1	152383413	G	A	GCTGGATCGT,GGGTTTCTGA	exonic	CRNN	nonsynonymous SNV	[illegible]	1q21.3	.
1	152800010	C	T	CCCAAGTGCC,TCCCAAGTGC	exonic	LCE1A	nonsynonymous SNV	[illegible]	1q21.3	.
1	152882979	C	T	GCTGGAGCTC,CACAGCAGCA	exonic	IVL	nonsynonymous SNV	[illegible]	1q21.3	IVL;basechange=c.706C>T;AAchange=p.P236S;tissue=large_intestine
1	153658651	G	A	GAGCTGACAC,AAAAGTCCTG	exonic	NPR1	nonsynonymous SNV	[illegible]	1q21.3	.
1	153924640	G	A	CAGTGGTGGG,GAAAGTGCAG	exonic	CRTC2	nonsynonymous SNV	[illegible]	1q21.3	.
1	154474399	G	A	CGCCATGAGG,GGCCCCGGCC	exonic	SHE	nonsynonymous SNV	[illegible]	1q21.3	.
1	154519914	C	G	GGTTGTCCAT,GAATCCTCAA	exonic	TDRD10	nonsynonymous SNV	[illegible]	1q21.3	.
1	154561912	G	A	TTTAGGCTGA,AGAATCTCCT	exonic	ADAR	nonsynonymous SNV	[illegible]	1q21.3	.
1	154987341	G	A	GGGCGGTGGC,GAGCTGTCAT	exonic	ZBTB7B	nonsynonymous SNV	[illegible]	1q21.3	.
1	155408820	G	A	GTCATCCCCA,AAGCAACTAA	exonic	ASH1L	nonsynonymous SNV	[illegible]	1q22	.
1	157767670	C	T	GCTACTGATT,CTGCTCCGGC	exonic	FCRL1	nonsynonymous SNV	[illegible]	1q23.1	.
1	157772251	C	T	GCATCACTCT,CCTCACTGAA	exonic	FCRL1	nonsynonymous SNV	[illegible]	1q23.1	FCRL1;basechange=c.523G>T;AAchange=p.E175*;tissue=lung&FCRL1_ENST00000358292;basechange=c.523G>T;AAchange=p.E175
1	157804470	C	T	TCCACGCGTC,CTTGCAATGC	exonic	CD5L	nonsynonymous SNV	[illegible]	1q23.1	.
1	158532904	G	A	GGACAATTGG,AAATAAAAAG	exonic	OR6P1	nonsynonymous SNV	[illegible]	1q23.1	.
1	159683347	C	T	GTGAACACTT,GCCTTGCACT	exonic	CRP	nonsynonymous SNV	[illegible]	1q23.2	CRP;basechange=c.643G>A;AAchange=p.E215K;tissue=large_intestine
1	159922144	G	A	ACTGTCCCCC,GCCTCCAGGA	exonic	SLAMF9	nonsynonymous SNV	[illegible]	1q23.2	.
1	160012275	T	A	GGGGCCGGCT,TCTGTCTGAG	exonic	KCNJ10	nonsynonymous SNV	[illegible]	1q23.2	KCNJ10;basechange=c.48A>T;AAchange=p.E16D;tissue=lung
1	160064964	G	A	GCAGGAGATG,AGACAGCTGT	exonic	IGSF8	nonsynonymous SNV	[illegible]	1q23.2	IGSF8;basechange=c.137C>G;AAchange=p.S46C;tissue=liver
1	160192457	C	T	TCCCTTGTCC,CCTCCATGAA	exonic	DCAF8	nonsynonymous SNV	[illegible]	1q23.2	.
1	160851928	C	T	GCCGCCACCC,CAGAGGTCAT	exonic	ITLN1	nonsynonymous SNV	[illegible]	1q23.3	.
1	162353006	C	T	TCGGCTGGAT,CTCCACCTCC	exonic	C1orf226	nonsynonymous SNV	[illegible]	1q23.3	.
1	167849810	T	G	AGGTACCTTT,CCCCAGGGAA	exonic	ADCY10	nonsynonymous SNV	[illegible]	1q24.2	.
1	176525511	C	T	GCTGGGTGGG,ACTCTGTTCT	exonic	PAPPA2	nonsynonymous SNV	[illegible]	1q25.2	.
1	176927551	C	T	ATGGCCAGTT,GGCTGGTGGA	exonic	ASTN1	nonsynonymous SNV	[illegible]	1q25.2	.
1	178858813	C	T	GAAGAGACCT,ATGGCCTGCT	exonic	RALGPS2	nonsynonymous SNV	[illegible]	1q25.2	.
1	182025645	C	T	AATCCCTTCT,CCCGGAGTGG	exonic	ZNF648	nonsynonymous SNV	[illegible]	1q25.3	.
1	182852448	C	T	GTTAATTGTC,TTTTAGTAGC	exonic	DHX9	nonsynonymous SNV	[illegible]	1q25.3	.
1	183835196	G	A	AGTCCAAAAT,GTGATCAGGA	exonic	RGL1	nonsynonymous SNV	[illegible]	1q25.3	.
1	183885671	C	T	CCTCTCCTCC,CTCCGTCCTG	exonic	RGL1	nonsynonymous SNV	[illegible]	1q25.3	.
1	183885813	G	A	GACAATAACG,CAACATGTAC	exonic	RGL1	nonsynonymous SNV	[illegible]	1q25.3	.
1	183938597	T	C	TGGGGTCCTC,TATAGAAGCC	exonic	COLGALT2	nonsynonymous SNV	[illegible]	1q25.3	.
1	185878628	C	T	GATTGAAATT,GCAATCCTTT	exonic	HMCN1	nonsynonymous SNV	[illegible]	1q31.1	HMCN1;basechange=c.781C>T;AAchange=p.R261C;tissue=endometrium
1	185964039	C	T	GACTCCTCTT,CTGTAATCAC	exonic	HMCN1	nonsynonymous SNV	[illegible]	1q31.1	HMCN1;basechange=c.3598C>A;AAchange=p.P1200T;tissue=lung
1	186055467	C	T	CTCTGGTTTT,CACCTCCTGA	exonic	HMCN1	nonsynonymous SNV	[illegible]	1q31.1	.
1	190067482	C	T	GCCCAGGTTC,GGGAAGGGTC	exonic	BRINP3	nonsynonymous SNV	[illegible]	1q31.1	.
1	196274381	G	C	AGTTTTGAAA,AGCAAGAGAG	exonic	KCNT2	nonsynonymous SNV	[illegible]	1q31.3	.
1	196871658	G	A	CTATTACTGT,ATCAAAATTT	exonic	CFHR4	nonsynonymous SNV	[illegible]	1q31.3	CFHR4;basechange=c.169G>A;AAchange=p.D57N;tissue=pancreas
1	196977808	G	A	TCCTATATGT,AATGAAGCAA	exonic	CFHR5	nonsynonymous SNV	[illegible]	1q31.3	.
1	200818283	G	A	AAAGAAAGGG,ATGGGATATC	exonic	CAMSAP2	nonsynonymous SNV	[illegible]	1q32.1	.
1	201035010	G	A	AACTGTAGGA,GGTAGTGACC	exonic	CACNA1S	nonsynonymous SNV	[illegible]	1q32.1	.
1	202092357	C	T	AGCCCTAACC,CGGCAAGGAT	exonic	GPR37L1	nonsynonymous SNV	[illegible]	1q32.1	.
1	202920164	C	T	AGCCCCATTC,CCTGTGCCAC	exonic	ADIPOR1	nonsynonymous SNV	[illegible]	1q32.1	.
1	205074169	G	A	TCTCGTGGAT,ATATTGGACT	exonic	RBBP5	nonsynonymous SNV	[illegible]	1q32.1	.
1	205812915	C	T	GACTGCAATC,TGTAGAAGAG	splicing	PM20D1(ENST00000367136.4:ex[illegible]	.	.	1q32.1	.
1	206225233	C	T	GCTGCCATCT,GGGTCAGCAG	exonic	AVPR1B	nonsynonymous SNV	[illegible]	1q32.1	AVPR1B;basechange=c.793C>T;AAchange=p.R265W;tissue=endometrium
1	215824023	C	T	ATGTTGACCA,TGCTTGGGTA	exonic	USH2A	nonsynonymous SNV	[illegible]	1q41	.
1	215914871	C	T	CTGCTAACTC,ACAACTTCCT	exonic	USH2A	nonsynonymous SNV	[illegible]	1q41	USH2A;basechange=c.11557G>A;AAchange=p.G3853R;tissue=large_intestine
1	222757634	C	T	CTTTTCCCTC,AATGGCTATA	exonic	TAF1A	nonsynonymous SNV	[illegible]	1q41	.
1	223938625	C	T	GAAACTGATC,GCATCCGAAA	exonic	CAPN2	nonsynonymous SNV	[illegible]	1q41	.
1	223947140	G	A	CAAGGATGGG,ATTTCTGCAT	exonic	CAPN2	nonsynonymous SNV	[illegible]	1q41	.
1	227198755	G	A	GATGGATAAG,GAAACCTGGC	exonic	CDC42BPA	nonsynonymous SNV	[illegible]	1q42.13	.
1	231299518	C	A	TCCGGGCCCA,TGGCACCGCC	exonic	TRIM67	nonsynonymous SNV	[illegible]	1q42.2	.
1	233135106	C	T	CCAAAGTCCT,GGACGCATTC	exonic	PCNXL2	nonsynonymous SNV	[illegible]	1q42.2	.
1	236416792	C	T	AGGACAAGGT,GCTTCAGATT	exonic	ERO1LB	nonsynonymous SNV	[illegible]	1q42.3	.
1	237527657	G	A	TGCCCCTACA,AAATTCATGA	splicing	RYR2(ENST00000366574.2:exon[illegible]	.	.	1q43	.
1	240371818	C	T	CCCACCGCCC,CTCTGCTTCC	exonic	FMN2	nonsynonymous SNV	[illegible]	1q43	.
1	240492733	C	T	TTGCTCAATT,GTCGCAAACT	exonic	FMN2	nonsynonymous SNV	[illegible]	1q43	FMN2;basechange=c.4831C>T;AAchange=p.R1611C;tissue=endometrium
1	241904931	C	T	ATATTTTCAT,AAGTACTCAC	exonic	WDR64	stopgain	[illegible]	1q43	.
1	241920591	G	A	AAAGGGTAAG,AAGATGATAT	exonic	WDR64	nonsynonymous SNV	[illegible]	1q43	.
1	245582914	G	A	TAGCCGGGAG,GACTAACAGA	exonic	KIF26B	nonsynonymous SNV	[illegible]	1q44	.
1	246707932	G	A	AAATTTTGAC,AGGAATCATT	exonic	TFB2M	nonsynonymous SNV	[illegible]	1q44	.
1	247752061	C	T	CCGTCCTCTC,ATTACACTGT	exonic	OR2G2	nonsynonymous SNV	[illegible]	1q44	.
1	248039598	C	T	GGTGAAATTT,ATTCTACAAT	exonic	TRIM58	nonsynonymous SNV	[illegible]	1q44	.
1	248186046	C	T	TCCCTGCGAT,TCTGACAGAG	exonic	OR2L5	nonsynonymous SNV	[illegible]	1q44	.
1	248569944	C	T	CATGAGCTTT,CCTTCTGCAA	exonic	OR2T1	nonsynonymous SNV	[illegible]	1q44	OR2T1;basechange=c.649C>T;AAchange=p.P217S;tissue=skin
2	3392281	C	T	CCCTTTGCCA,CGCCCTGAGC	exonic	TRAPPC12	nonsynonymous SNV	[illegible]	2p25.3	.
2	11053759	C	T	CCTGCCCATC,ACCCCATCAT	exonic	KCNF1	nonsynonymous SNV	[illegible]	2p25.1	.
2	11756768	G	A	CTCTACCTCG,AGAAGAGAAG	exonic	GREB1	nonsynonymous SNV	[illegible]	2p25.1	GREB1_ENST00000381486;basechange=c.3334G>T;AAchange=p.E1112*;tissue=lung
2	20818949	G	T	CTTAAGCTGG,GCTTGGGTTT	exonic	HS1BP3	nonsynonymous SNV	[illegible]	2p24.1	.
2	21234291	C	T	TTCAGGGGTT,TAGCCGTAGT	exonic	APOB	nonsynonymous SNV	[illegible]	2p24.1	.
2	21235409	C	T	TGGGTTGTTT,CAAGTTTTTC	exonic	APOB	nonsynonymous SNV	[illegible]	2p24.1	.
2	25315993	G	A	GGTCGCCATC,ATATGGGTAA	exonic	EFR3B	nonsynonymous SNV	[illegible]	2p23.3	.
2	25367909	G	A	GAACAGTCCG,AGAAGGAGGA	exonic	EFR3B	nonsynonymous SNV	[illegible]	2p23.3	.
2	27167591	G	A	GTGCACCCTG,GAAAAAAGAG	exonic	DPYSL5	nonsynonymous SNV	[illegible]	2p23.3	.
2	27447950	G	A	CTGTGGTGTG,AGCTGACCAA	exonic	CAD	nonsynonymous SNV	[illegible]	2p23.3	.
2	27789903	G	A	TCAGCTCTCA,AGCCCCAGAA	exonic	AC109829.1	nonsynonymous SNV	[illegible]	2p23.3	.
2	29240675	G	A	TCCCCTCCGG,GCAGCGGGAC	exonic	FAM179A	nonsynonymous SNV	[illegible]	2p23.2	.
2	32710820	C	T	AAATACATCT,CTACATGTAA	exonic	BIRC6	nonsynonymous SNV	[illegible]	2p22.3	.
2	36943565	G	A	AATGTTCCTT,GTAAGTACTT	exonic	VIT	nonsynonymous SNV	[illegible]	2p22.2	.
2	37195642	C	T	TTTGGGAGGC,GAGGCGGGTA	exonic	HEATR5B	nonsynonymous SNV	[illegible]	2p22.2	.
2	37291926	C	T	CTGATGTACT,CACCTAACAA	exonic	HEATR5B	nonsynonymous SNV	[illegible]	2p22.2	.
2	43514071	G	A	GGGTTGGTGA,GAAAAGTGGT	exonic	THADA	nonsynonymous SNV	[illegible]	2p21	.
2	48809121	G	A	TTTGTGAATG,GAACCTGGAA	exonic	STON1,STON1-GTF2A1L	nonsynonymous SNV	[illegible]	2p16.3	.
2	50282132	G	A	CGACCAAGTC,ATATGGAATT	exonic	NRXN1	stopgain	[illegible]	2p16.3	.
2	50765498	G	A	GCTAAGGCAC,GTTTTGCTGT	exonic	NRXN1	nonsynonymous SNV	[illegible]	2p16.3	.
2	54864863	C	T	TAGGAAGAAT,GTGAGACAGC	exonic	SPTBN1	nonsynonymous SNV	[illegible]	2p16.2	.
2	56145354	C	T	GCTGGCTCAC,TTTGCATTGC	exonic	EFEMP1	nonsynonymous SNV	[illegible]	2p16.1	EFEMP1;basechange=c.130G>T;AAchange=p.D44Y;tissue=ovary
2	62901223	G	A	TTCAAGGTAT,AACTGGTAGG	exonic	EHBP1	nonsynonymous SNV	[illegible]	2p15	.
2	63265952	G	T	GCCATTGAAG,TAAGAAATGC	splicing	EHBP1(ENST00000405015.3:exo[illegible]	.	.	2p15	.
2	64778904	C	T	CTTTCTGCTC,TGTGAAAGGA	exonic	AFTPH	nonsynonymous SNV	[illegible]	2p14	AFTPH;basechange=c.296C>A;AAchange=p.P99H;tissue=breast
2	69093245	C	T	TCCTTCCTCT,CTTGTCACTG	exonic	BMP10	nonsynonymous SNV	[illegible]	2p13.3	.
2	70488518	G	A	AAGTTCATGA,GTAATTTTTT	exonic	PCYOX1	nonsynonymous SNV	[illegible]	2p13.3	.
2	71653657	C	T	GAAGTGAATC,TTCTCAGGCC	exonic	ZNF638	nonsynonymous SNV	[illegible]	2p13.2	.
2	71795129	G	A	GCAGATACGG,TCAAGCTGTG	exonic	DYSF	nonsynonymous SNV	[illegible]	2p13.2	.
2	73496788	C	T	CACGCGGGCT,CACCGCGGCC	exonic	FBXO41	nonsynonymous SNV	[illegible]	2p13.2	.
2	73677027	G	A	GAAAATTTCA,TAGCTCCTGG	exonic	ALMS1	nonsynonymous SNV	[illegible]	2p13.1	.
2	73762028	C	G	ACAAATTCCT,CATCTCCGGA	exonic	ALMS1	nonsynonymous SNV	[illegible]	2p13.1	.
2	74042870	C	A	CATAAAGCTT,CGAGCCTATC	exonic	C2orf78	nonsynonymous SNV	[illegible]	2p13.1	.
2	74641530	C	G	TCTTTGTCCT,TTGTTCAGGC	exonic	C2orf81	nonsynonymous SNV	[illegible]	2p13.1	.
2	74783913	C	T	CCAGTCCCTC,CCAGGGCCTT	exonic	DOK1	nonsynonymous SNV	[illegible]	2p13.1	.
2	79255334	G	A	TGTCTCCCTA,GATTTCTGAA	splicing	REG3G(ENST00000393897.2:exo[illegible]	.	.	2p12	.
2	79348736	A	C	AGCTGCCCAG,AGGCACCAAT	exonic	REG1A	nonsynonymous SNV	[illegible]	2p12	.
2	80529855	C	T	GCCCCATCCT,GCACAGGTGG	exonic	LRRTM1	nonsynonymous SNV	[illegible]	2p12	.
2	84989948	G	A	ACCACCTGTG,ACCTGCCTAC	exonic	DNAH6	nonsynonymous SNV	[illegible]	2p11.2	.
2	85890800	G	T	GGCCAGCGCT,TCATCCATGG	exonic	SFTPB	nonsynonymous SNV	[illegible]	2p11.2	.
2	86678306	T	A	AAAGAGTATT,CTTTCTAAAG	exonic	KDM3A	nonsynonymous SNV	[illegible]	2p11.2	.
2	88425841	C	T	ATATCCTTCC,CTTCTGGATG	exonic	FABP1	nonsynonymous SNV	[illegible]	2p11.2	.
2	88828599	C	A	AACTCCGGGA,AAAGAGTTTT	exonic	TEX37	nonsynonymous SNV	[illegible]	2p11.2	.
2	90199113	G	A	ATCTGGGACA,ATTTCACTCT	exonic	IGKV1D-12	nonsynonymous SNV	[illegible]	2p11.2	.
2	95780908	G	T	TCAAGCTGGC,TAGGGATGGG	exonic	MRPS5	stopgain	[illegible]	2q11.1	.
2	96697846	G	A	CCCAGGAATG,AGTGACAGCC	exonic	GPAT2	nonsynonymous SNV	[illegible]	2q11.1	.
2	97017568	G	T	TCTGTGTTCA,ATTGATCCCA	splicing	NCAPH(ENST00000240423.4:exo[illegible]	.	.	2q11.2	.
2	100011016	G	A	AAGATGTTAT,AAGGCTTCAG	exonic	EIF5B	nonsynonymous SNV	[illegible]	2q11.2	.
2	103013181	A	C	TCTTGCCCCA,TCACTAAAGC	exonic	IL18R1	nonsynonymous SNV	[illegible]	2q12.1	.
2	103124583	C	T	CGGACTTTCC,CTTCTCCATC	exonic	SLC9A4	nonsynonymous SNV	[illegible]	2q12.1	.
2	112761519	G	A	TCTTAAGCAG,AAGATGGGAC	exonic	MERTK	nonsynonymous SNV	[illegible]	2q13	.
2	113788703	G	A	CGAGAATCAC,AATAGCATAG	exonic	IL36B	nonsynonymous SNV	[illegible]	2q13	.
2	121746551	C	T	CGGCCTGGCC,GCGGCGCCTA	exonic	GLI2	nonsynonymous SNV	[illegible]	2q14.2	.
2	121747940	C	T	GGACAGCATC,AGCCCCAGCC	exonic	GLI2	nonsynonymous SNV	[illegible]	2q14.2	.
2	128088072	G	A	GTAGTTAATG,AATTACCAAC	exonic	MAP3K2	nonsynonymous SNV	[illegible]	2q14.3	.
2	128892522	C	T	CCTTAGTAAT,ATATACCACT	exonic	UGGT1	nonsynonymous SNV	[illegible]	2q14.3	.
2	130941408	C	T	GCAAGGCCCT,CTTTTCCTCC	exonic	MZT2B	nonsynonymous SNV	[illegible]	2q21.1	.
2	131521072	C	A	GCACCAGGCC,TGACCTGCTC	exonic	AMER3	nonsynonymous SNV	[illegible]	2q21.1	.
2	131521116	A	G	CTGGAAGGGC,AGGAGTGCCT	exonic	AMER3	nonsynonymous SNV	[illegible]	2q21.1	.
2	136566687	C	T	GGGAAATTCC,CTGAGCCATA	exonic	LCT	nonsynonymous SNV	[illegible]	2q21.3	.
2	136566688	C	T	GGAAATTCCC,TGAGCCATAA	exonic	LCT	nonsynonymous SNV	[illegible]	2q21.3	.
2	137813992	C	T	GCCTGTAGGT,CGTGGGGAAG	exonic	THSD7B	nonsynonymous SNV	[illegible]	2q22.1	.
2	142237979	C	T	ACAATGTACT,CTTCGTCATA	exonic	LRP1B	nonsynonymous SNV	[illegible]	2q22.2	LRP1B;basechange=c.329G>A;AAchange=p.G110E;tissue=large_intestine
2	152348624	C	T	GTAATAGTCT,CTGATCTTGG	exonic	NEB	nonsynonymous SNV	[illegible]	2q23.3	.
2	158634824	T	A	AACCTCCAAG,GGAAATTCTG	exonic	ACVR1	nonsynonymous SNV	[illegible]	2q24.1	.
2	160027239	C	T	GCAATCATTT,CAAGTTGGTG	exonic	TANC1	nonsynonymous SNV	[illegible]	2q24.2	.
2	162820724	G	A	GTACCGCTTC,AAAAGTGCAT	exonic	SLC4A10	nonsynonymous SNV	[illegible]	2q24.2	.
2	163279967	A	T	CAGCATCTGC,TGTGGTACCT	exonic	KCNH7	nonsynonymous SNV	[illegible]	2q24.2	.
2	166019246	G	A	AGCCCAATGA,AGCAAACACG	exonic	SCN3A	nonsynonymous SNV	[illegible]	2q24.3	.
2	166894549	G	A	GCCAAGACGA,GGTTAAATTT	exonic	SCN1A	nonsynonymous SNV	[illegible]	2q24.3	.
2	166901669	C	T	TCATCCTCAT,TTTCTCTTCC	exonic	SCN1A	nonsynonymous SNV	[illegible]	2q24.3	.
2	167055790	C	T	TCAAACTTCT,CCAAACCTCA	exonic	SCN9A	nonsynonymous SNV	[illegible]	2q24.3	.
2	168103331	C	T	GACATCATCC,TGGAGATGTG	exonic	XIRP2	nonsynonymous SNV	[illegible]	2q24.3	.
2	170806358	C	T	CTACTATCCT,CTTGGCTTGA	exonic	UBR3	nonsynonymous SNV	[illegible]	2q31.1	.
2	175711098	A	G	CAGCAGCATC,ACAGTCCCAT	exonic	CHN1	nonsynonymous SNV	[illegible]	2q31.1	.
2	176964996	C	T	CTCCAGGGGG,TCCCTGCGCC	exonic	HOXD12	nonsynonymous SNV	[illegible]	2q31.1	.
2	179402342	G	T	GGGTAACCAG,ATGGAACTGC	exonic	TTN	nonsynonymous SNV	[illegible]	2q31.2	.
2	179433506	G	A	GGAACTGCAA,GGACCGAGGA	exonic	TTN	nonsynonymous SNV	[illegible]	2q31.2	.
2	179449219	C	T	GGGCTCCCTC,ATCACTATCT	exonic	TTN	nonsynonymous SNV	[illegible]	2q31.2	.
2	179499248	T	G	ATCAGGTCCT,TGGACCATAT	exonic	TTN	nonsynonymous SNV	[illegible]	2q31.2	.

2	179593451	G	A	CAAGGTCATG,GATCTTTCTC	exonic	TTN	nonsynonymous SNV	[illegible]	[illegible]	.
2	179597266	C	T	AAATAGAGTT,CAGGGAACTC	exonic	TTN	nonsynonymous SNV	[illegible]	[illegible]	TTN_ENST00000342992;basechange=c.12790G>A;AAchange=p.E4264K;tissue=breast
2	179615383	C	T	TTCATCAATT,GTGTAGAAGC	exonic	TTN	nonsynonymous SNV	[illegible]	[illegible]	TTN_ENST00000360870;basechange=c.11744G>A;AAchange=p.R3915Q;tissue=endometrium
2	179642264	C	T	TGAGTACCAT,TTCTTTAATG	exonic	TTN	nonsynonymous SNV	[illegible]	[illegible]	.
2	179650730	C	T	GAAATGCGTT,CTTATATCCG	exonic	TTN	nonsynonymous SNV	[illegible]	[illegible]	.
2	183866770	C	T	TATTCTCTGT,ACTTAAAACA	exonic	NCKAP1	nonsynonymous SNV	[illegible]	[illegible]	.
2	183993144	C	T	ATTAGTGGCC,TTCAGTAGGA	exonic	NUP35	nonsynonymous SNV	[illegible]	[illegible]	.
2	186659973	G	A	GACCCTAAAA,ACAGCAGATC	exonic	FSIP2	nonsynonymous SNV	[illegible]	[illegible]	.
2	186678369	G	A	CTTATTGTTC,ATTAGTAGCT	exonic	FSIP2	nonsynonymous SNV	[illegible]	[illegible]	.
2	189925479	C	G	TTTCCACCTT,TCCAGGAGGC	exonic	COL5A2	nonsynonymous SNV	[illegible]	[illegible]	.
2	196753695	C	T	TAACCATTTC,TATCTGGAGT	exonic	DNAH7	nonsynonymous SNV	[illegible]	[illegible]	.
2	197183600	G	A	GAAAAGGCTG,GGTTTCAGGA	exonic	HECW2	nonsynonymous SNV	[illegible]	[illegible]	.
2	202260150	T	A	ATTCTGGACA,CTGAGCATTT	exonic	TRAK2	nonsynonymous SNV	[illegible]	[illegible]	.
2	202446922	C	T	TTCCGCTTAT,ATCATAACGT	exonic	ALS2CR11	nonsynonymous SNV	[illegible]	[illegible]	.
2	206166551	C	T	ATACGGCTCT,AATTCTGTGC	exonic	PARD3B	stopgain	[illegible]	[illegible]	.
2	207426961	C	G	CAAGTATTAT,GCAGAGCCTG	exonic	ADAM23	nonsynonymous SNV	[illegible]	[illegible]	.
2	210783265	C	T	GATTCCGCCT,CCAGTATCAA	exonic	UNC80	nonsynonymous SNV	[illegible]	[illegible]	.
2	212251833	C	T	ACAGACACTC,TTGTTCAGCA	exonic	ERBB4	nonsynonymous SNV	[illegible]	[illegible]	.
2	212543810	C	T	TATGCAGATC,TTCCTCTACT	exonic	ERBB4	nonsynonymous SNV	[illegible]	[illegible]	.
2	215440537	C	T	GGCAAGCAGA,TGTGTAGGAC	exonic	VWC2L	nonsynonymous SNV	[illegible]	[illegible]	.
2	216251542	T	A	GCACCCGATC,TCTCGAGGTC	exonic	FN1	nonsynonymous SNV	[illegible]	[illegible]	.
2	218683405	G	A	GCGGAAGCCA,ACTCCAACAG	exonic	TNS1	nonsynonymous SNV	[illegible]	[illegible]	.
2	218713109	C	T	GCTAAACCCT,CTGTGCAGAG	exonic	TNS1	nonsynonymous SNV	[illegible]	[illegible]	.
2	219691657	C	T	GTACCACATT,GTTGACCACA	exonic	PRKAG3	nonsynonymous SNV	[illegible]	[illegible]	.
2	219874773	C	T	AGGTATGTTT,CCAGGGAAAT	exonic	CCDC108	nonsynonymous SNV	[illegible]	[illegible]	.
2	220037589	G	A	AATGGTGGAG,AGTCAGTGAG	exonic	CNPPD1	nonsynonymous SNV	[illegible]	[illegible]	.
2	220193670	C	T	ATAATTTCTT,CTTAGAGGGT	exonic	RESP18	nonsynonymous SNV	[illegible]	[illegible]	.
2	220493898	C	T	CACCCACCAC,CGCTCTCAGC	exonic	SLC4A3	nonsynonymous SNV	[illegible]	[illegible]	.
2	220494003	C	T	CTCTGCTCCT,CCTCCGAGGG	exonic	SLC4A3	nonsynonymous SNV	[illegible]	[illegible]	.
2	225669817	C	T	GCTGGGAGCT,TCTGCCAGTA	exonic	DOCK10	nonsynonymous SNV	[illegible]	[illegible]	DOCK10;basechange=c.4070G>A;AAchange=p.R1357K;tissue=lung
2	225702498	G	A	TGACTGGACA,AATGATCCAG	exonic	DOCK10	nonsynonymous SNV	[illegible]	[illegible]	.
2	227915865	C	T	CCCGGGAGTT,CTTTATCACC	exonic	COL4A4	nonsynonymous SNV	[illegible]	[illegible]	.
2	227953494	C	T	CCAGGGGGGC,CATGGGTCCA	exonic	COL4A4	nonsynonymous SNV	[illegible]	[illegible]	.
2	228122347	G	A	GGCCCTCCAG,ATTTCGTGGT	exonic	COL4A3	nonsynonymous SNV	[illegible]	[illegible]	.
2	228396908	C	T	TCAGCTTTTC,TCTTCAGGCT	exonic	AGFG1	nonsynonymous SNV	[illegible]	[illegible]	.
2	232263085	G	A	GACCCTCAAG,AGATCCACTT	exonic	B3GNT7	nonsynonymous SNV	[illegible]	[illegible]	.
2	233386543	C	T	GCGCCCCAGG,CTCAAGCTCT	exonic	PRSS56	nonsynonymous SNV	[illegible]	[illegible]	.
2	234526843	C	T	TTTCTCCCTC,CCTCTGTGGT	exonic	UGT1A1	nonsynonymous SNV	[illegible]	[illegible]	.
2	234676907	C	G	TGCTGGTTCC,ATGGTGTTTA	exonic	UGT1A1,UGT1A10,UGT1A3,UGT1A4,UGT1A5,UGT1A6,UGT1A7,UGT1A8,UGT1A9	nonsynonymous SNV	[illegible]	[illegible]	.
2	234845963	C	T	TCAATGAAAT,CTTCCTTCCT	exonic	TRPM8	nonsynonymous SNV	[illegible]	[illegible]	.
2	237123235	C	T	CGCTCGTCCT,GTCGCGCGCG	exonic	ASB18	nonsynonymous SNV	[illegible]	[illegible]	.
2	237240039	A	T	CACTGGATTC,TGCTGGTTAT	exonic	IQCA1	nonsynonymous SNV	[illegible]	[illegible]	.
2	238882313	G	T	GGCGAATCTT,TGAAAAATTA	exonic	UBE2F	nonsynonymous SNV	[illegible]	[illegible]	.
2	239013429	G	A	TCGATGGCAT,AGCGCCCTGC	exonic	ESPNL	nonsynonymous SNV	[illegible]	[illegible]	.
2	242346993	C	T	AAAATTTTTT,CACCTGATCC	exonic	FARP2	nonsynonymous SNV	[illegible]	[illegible]	.
3	4714929	G	A	CATGTCTGAC,AGAACCTGCC	exonic	ITPR1	nonsynonymous SNV	[illegible]	[illegible]	ITPR1;basechange=c.2269G>A;AAchange=p.E757K;tissue=endometrium&ITPR1_ENST00000302640;basechange=c.2269G>A;AAchan
3	9800940	G	A	GCCTCCTGAA,AATTACAGAC	exonic	OGG1	nonsynonymous SNV	[illegible]	[illegible]	.
3	9860253	T	C	GGGGTTTCAC,GTGTTAGCCA	exonic	TTLL3	nonsynonymous SNV	[illegible]	[illegible]	.
3	10251335	C	T	AGATGCCCCT,ATTCCTTGAG	exonic	IRAK2	nonsynonymous SNV	[illegible]	[illegible]	.
3	10384559	C	T	AGCAGGGTCT,CGTGGGCGGC	exonic	ATP2B2	nonsynonymous SNV	[illegible]	[illegible]	.
3	16242227	G	A	CCGGATGCTG,GGGCCACCAG	exonic	GALNT15	nonsynonymous SNV	[illegible]	[illegible]	.
3	17131327	G	A	ACCAGGCACC,AAAATGCTGA	exonic	PLCL2	nonsynonymous SNV	[illegible]	[illegible]	PLCL2;basechange=c.2929G>A;AAchange=p.E977K;tissue=large_intestine
3	27343218	T	A	TTTCTTGTAT,TCCCTAGGGC	exonic	NEK10	nonsynonymous SNV	[illegible]	[illegible]	.
3	27453181	G	A	GGCCTCTTTA,GAGTCTCACC	exonic	SLC4A7	nonsynonymous SNV	[illegible]	[illegible]	.
3	32586472	G	A	AGCAGTCCAA,GCTTTGACAT	exonic	DYNC1LI1	nonsynonymous SNV	[illegible]	[illegible]	.
3	35770897	C	T	GCTGGCTCTC,AGGCTGTGTG	exonic	ARPP21	nonsynonymous SNV	[illegible]	[illegible]	.
3	35781022	C	T	GGTCCTCCAG,CCCCTCCCTC	exonic	ARPP21	nonsynonymous SNV	[illegible]	[illegible]	.
3	37114319	G	A	GTCTCCTGGA,GTCAAACACC	exonic	LRRFIP2	nonsynonymous SNV	[illegible]	[illegible]	.
3	37547509	G	A	GTGACCGCTG,CCACTTCTCT	exonic	ITGA9	nonsynonymous SNV	[illegible]	[illegible]	.
3	38595782	G	A	ACGATGGAGA,GATGACAACC	exonic	SCN5A	nonsynonymous SNV	[illegible]	[illegible]	.
3	39227123	C	T	CGGTGGGCTC,AGCTGGAAAG	exonic	XIRP1	nonsynonymous SNV	[illegible]	[illegible]	.
3	39307301	G	A	ACCACCAGAA,GATCAGTTTA	exonic	CX3CR1	nonsynonymous SNV	[illegible]	[illegible]	.
3	41877454	G	A	TCAGTTAAGA,AACAATTGCC	exonic	ULK4	nonsynonymous SNV	[illegible]	[illegible]	.
3	44488602	G	A	ATCTAAAAGG,TTCTTTTACG	exonic	ZNF445	nonsynonymous SNV	[illegible]	[illegible]	.
3	46902205	C	T	AGCACTTCTG,CTGTGTGGGG	exonic	MYL3	nonsynonymous SNV	[illegible]	[illegible]	.
3	49161053	C	T	AGTGGCCTCC,CAATTTCACG	exonic	LAMB2	nonsynonymous SNV	[illegible]	[illegible]	.
3	52356528	C	T	CAGCAGTGCT,CTGCAGTCCA	exonic	DNAH1	nonsynonymous SNV	[illegible]	[illegible]	.
3	52485443	C	T	TTCTTGTCTC,GTCCTTCATG	exonic	TNNC1	nonsynonymous SNV	[illegible]	[illegible]	.
3	52514258	C	T	AGCTCCCCTC,CACTGTGGCT	exonic	NISCH	nonsynonymous SNV	[illegible]	[illegible]	.
3	52535218	G	T	AACCACGTTT,TCACTCATGT	exonic	STAB1	nonsynonymous SNV	[illegible]	[illegible]	.
3	52783707	C	G	GCAAAAATTA,CTGGGCGTGG	splicing	NEK4(ENST00000233027.5:exon6:c.1506+1G>C,ENST00000535191.1:exon8:c.1239+1G>C,ENST00000461689.1:exon6:c.1239+1G>C,ENST00000487068.1:exon3:c.277+1G>C)	.	.	[illegible]	.
3	53912416	C	T	CGTCTTGTAC,ATTGGACATC	exonic	ACTR8	nonsynonymous SNV	[illegible]	[illegible]	.
3	57488155	G	T	TCTGTGTCAA,ATTTACTGGT	exonic	DNAH12	nonsynonymous SNV	[illegible]	[illegible]	.
3	58849298	C	T	TCACTGCTGT,GCCGCCGTAA	exonic	C3orf67	nonsynonymous SNV	[illegible]	[illegible]	.
3	62248547	C	A	AGCACAAAAA,AGATACATCA	exonic	PTPRG	nonsynonymous SNV	[illegible]	[illegible]	.
3	65415195	C	T	CCCTACTTAC,TCCTCGTTGC	exonic	MAGI1	nonsynonymous SNV	[illegible]	[illegible]	.
3	73004261	G	A	GATTTTAAAG,ATGTGGACTC	exonic	GXYLT2	nonsynonymous SNV	[illegible]	[illegible]	.
3	77147191	C	T	GGAGGACTTT,CCCCGCGGAT	exonic	ROBO2	nonsynonymous SNV	[illegible]	[illegible]	.
3	97852023	G	A	ATCCATGAAG,ATTTTTATTC	exonic	OR5H1	nonsynonymous SNV	[illegible]	[illegible]	.
3	100513823	G	A	GGGTATAAAA,GGATGCCTCG	exonic	ABI3BP	nonsynonymous SNV	[illegible]	[illegible]	.
3	100553599	C	T	TGTTCTTGTT,GTTGCGATGT	exonic	ABI3BP	nonsynonymous SNV	[illegible]	[illegible]	.
3	105495366	G	A	GAAGATAAGG,ACAGTTTTGT	exonic	CBLB	nonsynonymous SNV	[illegible]	[illegible]	.
3	109031452	C	T	ACGCTTGGTT,CATTTGTTCC	exonic	DPPA2	nonsynonymous SNV	[illegible]	[illegible]	.
3	109046851	C	T	CCATTGGAGG,TTTTTATTAA	exonic	DPPA4	nonsynonymous SNV	[illegible]	[illegible]	.
3	112068598	C	T	AGCATTGTTT,CCTGGTAATT	exonic	CD200	nonsynonymous SNV	[illegible]	[illegible]	.
3	112328947	A	C	AAAAAGCAGG,CACCATTACT	exonic	CCDC80	nonsynonymous SNV	[illegible]	[illegible]	.
3	112357108	C	T	TTTTTCTTCT,CTTCTCTGGT	exonic	CCDC80	nonsynonymous SNV	[illegible]	[illegible]	.
3	118623520	C	T	ATTTCTTCTT,TTCCTCCTCT	exonic	IGSF11	nonsynonymous SNV	[illegible]	[illegible]	.
3	121634166	T	C	TGCTGAGAGG,TAGGATTTTT	splicing	SLC15A2(ENST00000489711.1:exon6:c.819+2T>C,ENST00000295605.2:exon5:c.526+2T>C,ENST00000469013.1:exon6:c.433+2T>C)	.	.	[illegible]	.
3	123038565	G	A	AGGAACTTGC,GACGTGCTCA	exonic	ADCY5	nonsynonymous SNV	[illegible]	[illegible]	.
3	123699153	T	G	TCCGCGGTTT,ACAGGACCCT	exonic	ROPN1	stoploss	[illegible]	[illegible]	.
3	126741054	G	A	CCGCGGGAAT,TGGCCTCGCT	exonic	PLXNA1	nonsynonymous SNV	[illegible]	[illegible]	.
3	128344468	A	T	CTCCTGCAGC,TGAGCACCTT	exonic	RPN1	nonsynonymous SNV	[illegible]	[illegible]	.
3	129546990	C	T	ATTTCAGGTT,TGGATCTGCC	exonic	TMCC1	nonsynonymous SNV	[illegible]	[illegible]	.
3	130368357	C	T	GAAATCATTC,CGTGGTGATC	exonic	COL6A6	nonsynonymous SNV	[illegible]	[illegible]	.
3	130799340	C	T	GGAAGCCCAA,TCCTCTCCAA	exonic	NEK11	nonsynonymous SNV	[illegible]	[illegible]	.
3	132420290	G	A	GATAAAAGAA,AGACTTCCCA	exonic	NPHP3	nonsynonymous SNV	[illegible]	[illegible]	.
3	133098661	G	A	ATCTCCCAGG,AATCTCTTCA	exonic	TMEM108	nonsynonymous SNV	[illegible]	[illegible]	.
3	134670294	A	T	GCCCAACCAG,ACAATTGGCT	exonic	EPHB1	nonsynonymous SNV	[illegible]	[illegible]	.
3	140265437	G	A	TGGCAAAATG,AAAGCCAGAA	exonic	CLSTN2	nonsynonymous SNV	[illegible]	[illegible]	.
3	145924354	G	A	TTTGCCATAG,AGTTGGCCCT	exonic	PLSCR4	nonsynonymous SNV	[illegible]	[illegible]	.
3	151155798	G	A	AATGGAGAAG,AAATCATGTC	exonic	IGSF10	nonsynonymous SNV	[illegible]	[illegible]	.
3	151163831	G	T	TGTTTGCGTT,ATATGATGCT	exonic	IGSF10	stopgain	[illegible]	[illegible]	.
3	157155547	G	A	GGCGACCCGC,ACGCGGGCCG	exonic	PTX3	nonsynonymous SNV	[illegible]	[illegible]	PTX3;basechange=c.376G>A;AAchange=p.D126N;tissue=oesophagus
3	158261187	G	A	AGCAGTTGCT,ATCCACCCAG	exonic	RSRC1	nonsynonymous SNV	[illegible]	[illegible]	.
3	159482356	C	T	TCTTCATCGT,GCCGGGGGGC	exonic	IQCJ-SCHIP1	nonsynonymous SNV	[illegible]	[illegible]	.
3	164748550	C	T	GTTGCCAAAT,TGCATCTGGA	exonic	SI	nonsynonymous SNV	[illegible]	[illegible]	.
3	164776967	C	A	AAGATGATGA,ATATTTCTGT	exonic	SI	nonsynonymous SNV	[illegible]	[illegible]	.
3	164776968	A	T	AGATGATGAC,TATTTCTGTC	exonic	SI	stopgain	[illegible]	[illegible]	.
3	164906757	C	T	TGGGGATTCT,CAGCTGGTGC	exonic	SLITRK3	nonsynonymous SNV	[illegible]	[illegible]	.
3	169511469	G	A	TGCATTAGAT,AGTGGTCATA	exonic	LRRC34	nonsynonymous SNV	[illegible]	[illegible]	.
3	169524668	C	T	TCTCCACCTT,GGGCCCAATA	exonic	LRRC34	nonsynonymous SNV	[illegible]	[illegible]	.
3	172477988	C	T	TGGAGTTATT,GAAAAGACTT	exonic	ECT2	stopgain	[illegible]	[illegible]	ECT2;basechange=c.526C>T;AAchange=p.R176*;tissue=large_intestine
3	173993243	C	T	GGATCTGGTG,TGGGGGTTCA	exonic	NLGN1	nonsynonymous SNV	[illegible]	[illegible]	.
3	179470150	C	T	GACTGGGTTC,CAAAAAATTT	exonic	USP13	nonsynonymous SNV	[illegible]	[illegible]	.
3	183777792	G	A	GCCCCAGAAG,GAAATAAGGG	exonic	HTR3C	nonsynonymous SNV	[illegible]	[illegible]	.
3	186394974	G	A	CAAGCCACAC,AACATGGACC	exonic	HRG	nonsynonymous SNV	[illegible]	[illegible]	HRG;basechange=c.880G>A;AAchange=p.E294K;tissue=stomach,large_intestine,endometrium
3	188592138	G	A	TACTGTTCTA,GATTGCGGTG	splicing	LPP(ENST00000312675.4:exon11:c.1711-1G>A,ENST00000543006.1:exon11:c.1711-1G>A)	.	.	[illegible]	.
3	188592139	G	A	ACTGTTCTAG,ATTGCGGTGG	exonic	LPP	nonsynonymous SNV	[illegible]	[illegible]	.
3	195453015	G	A	ACAGCACCCG,GGCCACGACC	exonic	MUC20	nonsynonymous SNV	[illegible]	[illegible]	.
3	195515980	G	A	CGTCTCTCCT,AGGTGGATAT	exonic	MUC4	nonsynonymous SNV	[illegible]	[illegible]	.
3	195516356	G	A	TCCCCGGTGG,AGCTGGGGCA	exonic	MUC4	nonsynonymous SNV	[illegible]	[illegible]	.
3	197408100	G	A	CAGGTCCTTG,AGAAGTTGCT	exonic	KIAA0226	nonsynonymous SNV	[illegible]	[illegible]	.
3	197547251	C	T	AACCTGGAGG,CTTGAGAGAC	exonic	LRCH3	nonsynonymous SNV	[illegible]	[illegible]	.
4	367533	C	T	AAACAATTTT,GCTCCTGAGT	exonic	ZNF141	nonsynonymous SNV	[illegible]	[illegible]	ZNF141;basechange=c.1307C>T;AAchange=p.S436L;tissue=large_intestine
4	1222094	G	A	GAGAGTGATG,TGTGGTACAT	exonic	CTBP1	nonsynonymous SNV	[illegible]	[illegible]	.
4	3105569	C	T	GGATTCTAAT,TTCCAAGGTT	exonic	HTT	nonsynonymous SNV	[illegible]	[illegible]	.
4	3117082	C	T	CCCCACCATT,GGCGGACAGC	exonic	HTT	nonsynonymous SNV	[illegible]	[illegible]	.
4	15985950	G	A	GGTGGCCACA,GTTTGCACGA	exonic	PROM1	nonsynonymous SNV	[illegible]	[illegible]	.
4	38054771	C	T	CATTCTTCTT,CTCTGTGCCA	exonic	TBC1D1	nonsynonymous SNV	[illegible]	[illegible]	.
4	39000392	T	C	TTGGAGGGAT,TAGAAAGATT	exonic	TMEM156	nonsynonymous SNV	[illegible]	[illegible]	.
4	39435999	A	G	TGGTTTGCCA,CCCTATCCAT	exonic	KLB	nonsynonymous SNV	[illegible]	[illegible]	.
4	40104531	C	T	GCTGTTGCCT,CTCCGCCACC	exonic	N4BP2	nonsynonymous SNV	[illegible]	[illegible]	.
4	40104532	C	T	CTGTTGCCTC,TCCGCCACCT	exonic	N4BP2	nonsynonymous SNV	[illegible]	[illegible]	.
4	40245177	G	A	TGGGCCTCTG,GACACAGCCG	exonic	RHOH	stopgain	[illegible]	[illegible]	.
4	41682102	G	A	GAAGAACGCA,ATACTATGAG	exonic	LIMCH1	nonsynonymous SNV	[illegible]	[illegible]	.
4	42895461	G	A	TGATTCCGAT,GACAGCAGAA	exonic	GRXCR1	nonsynonymous SNV	[illegible]	[illegible]	.
4	46307659	G	A	AGAGCCATCA,GAGCAACCTG	exonic	GABRA2	nonsynonymous SNV	[illegible]	[illegible]	.
4	48486138	C	T	AATCTTATGT,CCTGCTGGTT	exonic	SLC10A4	nonsynonymous SNV	[illegible]	[illegible]	.
4	55129962	G	A	CAACAGTGAG,GGGTGGTACC	exonic	PDGFRA	nonsynonymous SNV	[illegible]	[illegible]	.
4	55130020	G	A	TTCACTGTAG,GCCCTATATC	exonic	PDGFRA	nonsynonymous SNV	[illegible]	[illegible]	PDGFRA;basechange=c.554G>A;AAchange=p.G185E;tissue=ovary
4	57180881	G	A	GGGGGAAGAG,AGGAGGAGGG	exonic	KIAA1211	nonsynonymous SNV	[illegible]	[illegible]	.
4	57181997	G	A	GGAGAAGTCG,AGATGCACCG	exonic	KIAA1211	nonsynonymous SNV	[illegible]	[illegible]	.
4	57676318	T	A	GCAGGGTCCA,TTCGAATGAT	exonic	SPINK2	nonsynonymous SNV	[illegible]	[illegible]	.
4	69344690	G	A	TCCTTAGAAG,AAAAACAGAT	exonic	TMPRSS11E	nonsynonymous SNV	[illegible]	[illegible]	.
4	71347015	C	T	TCCTCAGCTC,ACCAGAGACC	exonic	MUC7	nonsynonymous SNV	[illegible]	[illegible]	.
4	71472058	G	A	CCCTGAGAAC,AAGAAGGAGG	exonic	AMBN	nonsynonymous SNV	[illegible]	[illegible]	AMBN;basechange=c.955G>A;AAchange=p.E319K;tissue=skin
4	71509967	C	T	GAGGGAAAGC,AAAACCCTTT	exonic	ENAM	stopgain	[illegible]	[illegible]	ENAM_ENST00000396073;basechange=c.2824C>T;AAchange=p.Q942*;tissue=large_intestine&ENAM;basechange=c.2824C>G;AAcha
4	71835671	A	G	CAATCAGATC,ACATGCTTTA	exonic	MOB1B	nonsynonymous SNV	[illegible]	[illegible]	.
4	74964357	G	A	AACCATGGGC,ATGCGGGGTT	exonic	CXCL2	nonsynonymous SNV	[illegible]	[illegible]	.
4	79188491	G	A	GTACCAGGAC,AAATGTGGAA	exonic	FRAS1	nonsynonymous SNV	[illegible]	[illegible]	FRAS1_ENST00000325942;basechange=c.886G>A;AAchange=p.E296K;tissue=upper_aerodigestive_tract&FRAS1_ENST00000264895
4	79340146	C	T	CATTCCCTCT,CTTCATAAAC	exonic	FRAS1	nonsynonymous SNV	[illegible]	[illegible]	.
4	81866037	G	A	CGATAATCCA,AAGGCTTACT	exonic	C4orf22	nonsynonymous SNV	[illegible]	[illegible]	.
4	83793092	G	A	ACAGCAGCAC,ATACCTGGCA	exonic	SEC31A	nonsynonymous SNV	[illegible]	[illegible]	.
4	85636505	C	T	GTAATTCCGT,CATCTCCAGA	exonic	WDFY3	nonsynonymous SNV	[illegible]	[illegible]	.
4	88403667	C	T	GTCTCTCATC,GTAGAGGAAA	exonic	SPARCL1	nonsynonymous SNV	[illegible]	[illegible]	.
4	88767428	C	T	CAGAAAATAT,ATTATGTACC	exonic	MEPE	nonsynonymous SNV	[illegible]	[illegible]	.
4	89618407	C	T	TCATGTTTGG,CCCCGCGGCA	exonic	NAP1L5	nonsynonymous SNV	[illegible]	[illegible]	.
4	96163723	C	T	CCACTTGCTC,ATGGCGTCCA	exonic	UNC5C	stopgain	[illegible]	[illegible]	.
4	96199480	C	T	CACTTCCTTT,CTAGGGGTTC	exonic	UNC5C	nonsynonymous SNV	[illegible]	[illegible]	.
4	101108989	C	T	GAGCAGTTCT,CTGCTTAAAC	exonic	DDIT4L	nonsynonymous SNV	[illegible]	[illegible]	.
4	110481543	C	T	CTGCCGACCC,TGGCACCTGG	exonic	CCDC109B	nonsynonymous SNV	[illegible]	[illegible]	.
4	111398069	G	A	CAAAAAGCAG,AGTACGTGGT	exonic	ENPEP	nonsynonymous SNV	[illegible]	[illegible]	.
4	114203991	T	C	GTTACCTTGC,TCTGGATAAG	exonic	ANK2	nonsynonymous SNV	[illegible]	[illegible]	.
4	114223939	A	C	ACAGAAAAAC,CAAACTAAAT	exonic	ANK2	nonsynonymous SNV	[illegible]	[illegible]	.
4	114257124	G	A	TGGCCCAGAA,GAGGTGTACT	exonic	ANK2	nonsynonymous SNV	[illegible]	[illegible]	.
4	114277893	G	A	CAGTCCAGAA,AAGTACAATT	exonic	ANK2	nonsynonymous SNV	[illegible]	[illegible]	.
4	114278559	G	A	TAGCCCTTAT,AAAATGTCCC	exonic	ANK2	nonsynonymous SNV	[illegible]	[illegible]	.
4	115597146	G	A	CCTGTCAATC,AACTATCTAT	exonic	UGT8	nonsynonymous SNV	[illegible]	[illegible]	UGT8;basechange=c.1328G>A;AAchange=p.R443Q;tissue=large_intestine
4	119161783	A	T	TGCAGAAGAG,TGTTTGGTCC	exonic	NDST3	nonsynonymous SNV	[illegible]	[illegible]	.
4	119951702	C	T	ACGGCCAAAC,CTTCCCAGGG	exonic	SYNPO2	nonsynonymous SNV	[illegible]	[illegible]	.
4	120528133	C	T	CTAGAAATAT,CTTCACTAAT	exonic	PDE5A	nonsynonymous SNV	[illegible]	[illegible]	.
4	122854151	C	T	CGGCCCTTCT,CCGCATCACT	exonic	TRPC3	nonsynonymous SNV	[illegible]	[illegible]	.
4	123301396	G	A	GCAAGTAACG,GTACGACTTT	exonic	ADAD1	nonsynonymous SNV	[illegible]	[illegible]	.
4	126238444	G	A	CAGGACGAGG,GACCCCCTTC	exonic	FAT4	nonsynonymous SNV	[illegible]	[illegible]	.
4	126241866	C	T	TGAGAACATT,CCATCGGTAC	exonic	FAT4	nonsynonymous SNV	[illegible]	[illegible]	.
4	126242247	G	A	AGGTGCTAAT,GAGAAATAGA	exonic	FAT4	nonsynonymous SNV	[illegible]	[illegible]	.
4	129209015	C	T	CGGCCCCGCC,ATCTGGGGGC	exonic	PGRMC2	nonsynonymous SNV	[illegible]	[illegible]	.
4	148653510	G	A	GTGGTTCCGG,AGAGGATCCG	exonic	ARHGAP10	nonsynonymous SNV	[illegible]	[illegible]	.
4	149075912	C	T	GCTGTGTTGA,CGAGGGTTCT	exonic	NR3C2	nonsynonymous SNV	[illegible]	[illegible]	.
4	151000301	G	A	GGCCCCAAGG,GAACGGGCTC	exonic	DCLK2	nonsynonymous SNV	[illegible]	[illegible]	.
4	155219254	C	T	AACCACAGTT,CATTGGGCCC	exonic	DCHS2	nonsynonymous SNV	[illegible]	[illegible]	.
4	155219255	C	G	ACCACAGTTC,ATTGGGCCCC	exonic	DCHS2	nonsynonymous SNV	[illegible]	[illegible]	.
4	155244460	C	T	TGCTGTTTTT,CCTTCAGAGG	exonic	DCHS2	nonsynonymous SNV	[illegible]	[illegible]	.
4	155298454	C	T	ATCCCTTTCC,TGTCGATATC	exonic	DCHS2	nonsynonymous SNV	[illegible]	[illegible]	.
4	159514632	G	C	ATCCTTTTGA,GCAGAAACAC	exonic	RXFP1	nonsynonymous SNV	[illegible]	[illegible]	.
4	164533937	C	T	TGACTCTCAT,TGTGGAAGAT	exonic	MARCH1	nonsynonymous SNV	[illegible]	[illegible]	.
4	167658744	G	A	TCACACAGGG,GATATACTGT	exonic	SPOCK3	nonsynonymous SNV	[illegible]	[illegible]	.
4	169842772	G	A	GCTGGTGCGT,AGAACGGGGT	exonic	PALLD	nonsynonymous SNV	[illegible]	[illegible]	.
4	173269793	G	A	CGAACCCCAG,GAGTCTGATA	exonic	GALNTL6	nonsynonymous SNV	[illegible]	[illegible]	.
4	174223236	G	A	GCCATTGAAC,AGAGTTCTTC	exonic	GALNT7	nonsynonymous SNV	[illegible]	[illegible]	.
4	174254256	C	T	CTTTTTCTTC,CCTTCTTATC	exonic	HMGB2	nonsynonymous SNV	[illegible]	[illegible]	.
4	175649711	C	T	GCACCCTTTT,ATTGGCAAAG	exonic	GLRA3	nonsynonymous SNV	[illegible]	[illegible]	.
4	176622807	G	A	TCCAGAAAGC,CTTCATGACC	exonic	GPM6A	nonsynonymous SNV	[illegible]	[illegible]	GPM6A;basechange=c.149C>T;AAchange=p.A50V;tissue=kidney,prostate
4	177116668	C	T	TCTAGGGCTG,CGCAGTCTGT	exonic	SPATA4	nonsynonymous SNV	[illegible]	[illegible]	.
4	183652092	C	T	ATCCCCATTC,TCACTCAGTA	exonic	TENM3	nonsynonymous SNV	[illegible]	[illegible]	.
4	184174983	G	A	TATTGAGAAG,AAAAACTGAT	exonic	WWC2	nonsynonymous SNV	[illegible]	[illegible]	.
4	186185599	G	A	TAGACATGAA,AACAGCCAAG	exonic	SNX25	nonsynonymous SNV	[illegible]	[illegible]	SNX25;basechange=c.247G>T;AAchange=p.E83*;tissue=lung
4	187173227	G	C	TTCTTGGGGA,AGTGGCCCTG	exonic	KLKB1	nonsynonymous SNV	[illegible]	[illegible]	.
4	187549878	G	A	GTAGAAAACT,AGGACGATGG	exonic	FAT1	stopgain	[illegible]	[illegible]	.
5	163162	C	A	AAGCCACCCT,ATTCCCCAGC	exonic	PLEKHG4B	stopgain	[illegible]	[illegible]	.
5	1032303	C	T	GGGGCCTTTC,GGGAGGACCA	exonic	NKD2	nonsynonymous SNV	[illegible]	[illegible]	.
5	1034362	G	A	ACTCCAGTGC,ATGTCTCGGT	exonic	NKD2	nonsynonymous SNV	[illegible]	[illegible]	.
5	5306831	C	T	TTTGCCTCAC,CTGGTCTCAG	exonic	ADAMTS16	nonsynonymous SNV	[illegible]	[illegible]	ADAMTS16_ENST00000274181;basechange=c.3401C>A;AAchange=p.P1134H;tissue=lung
5	7789754	G	A	TCTTGTAACA,AATGAATATT	splicing	ADCY2(ENST00000338316.4:exon20:c.2470-1G>A,ENST00000571121.1:exon17:c.1930-1G>A)	.	.	[illegible]	.
5	9629675	C	T	GAAAAATTTC,TTAGGAAGTA	exonic	TAS2R1	nonsynonymous SNV	[illegible]	[illegible]	.
5	13914019	G	A	TTTGCATTTG,ATTTTGTTTA	exonic	DNAH5	nonsynonymous SNV	[illegible]	[illegible]	.
5	15928101	C	T	TCCACCTCCT,GTCCTCCATC	exonic	FBXL7	nonsynonymous SNV	[illegible]	[illegible]	FBXL7;basechange=c.230C>T;AAchange=p.S77L;tissue=lung
5	23527515	A	G	CACACAGGGG,GAAGCCCTAT	exonic	PRDM9	nonsynonymous SNV	[illegible]	[illegible]	.
5	31299662	G	A	GCGGCCAGAT,GGAGGATTAT	exonic	CDH6	nonsynonymous SNV	[illegible]	[illegible]	.
5	31299732	C	T	TCCCCGATTC,CCCAGAGTAG	exonic	CDH6	nonsynonymous SNV	[illegible]	[illegible]	.
5	31799618	G	A	CTGAGTTTTG,GAACATCCCT	exonic	PDZD2	nonsynonymous SNV	[illegible]	[illegible]	.
5	33947368	G	A	GACCAGGGTG,AGTAGACATT	exonic	SLC45A2	nonsynonymous SNV	[illegible]	[illegible]	.
5	34826453	G	A	GATGTCGAAG,AAGTCACCAA	exonic	RAI14	nonsynonymous SNV	[illegible]	[illegible]	.
5	34915883	G	A	AGGCTTTGCA,TTCAGGCGAA	exonic	BRIX1	nonsynonymous SNV	[illegible]	[illegible]	.
5	35764750	G	A	CTTTCCAAAA,ATTTTGTGAA	exonic	SPEF2	nonsynonymous SNV	[illegible]	[illegible]	.
5	36039726	G	A	AAGATTTCCG,ATTCTGACAG	exonic	UGT3A2	nonsynonymous SNV	[illegible]	[illegible]	.

5	37168978	G	A	AGGTGTTGAG,GAACTGTAAT	exonic	C5orf42	[illegible]
5	39202415	C	T	ACACATTCTT,ATGGGGCTTT	exonic	FYB	[illegible]
5	39315936	G	A	TATGAAAACC,AAAACTACCC	exonic	C9	[illegible]
5	42719083	G	A	CCAGGTGAGC,ACATTACACC	exonic	GHR	[illegible]
5	43453776	C	G	AGACATGGAT,CAGCTAGAAA	exonic	C5orf28	[illegible]
5	72359743	C	T	AAGCTTACTT,AGGCAAACTC	exonic	FCHO2	[illegible]
5	73144873	C	T	AGGAAAAACT,GTTTGGTGCG	exonic	ARHGEF28	[illegible]
5	90107143	G	A	TTTTGCCATG,TTATTATTAC	exonic	GPR98	[illegible]
5	94206603	G	A	TCTATTTCAA,ATAGATGACC	exonic	MCTP1	[illegible]
5	101572591	C	T	TTGTTGAGAT,CTGTTCTGCT	exonic	SLCO4C1	[illegible]
5	109155571	C	T	TCGGTTTGAT,AAACTGGACT	exonic	MAN2A1	[illegible]
5	112164556	A	T	TTTGCAGGTT,TTGCGAGTGT	exonic	APC,CTC-554D6.1	[illegible]
5	115176247	A	G	CACCCGCCTC,GCCTACCAAA	exonic	ATG12	[illegible]
5	131034665	C	T	CTCTGACTGG,ATCAGCTGAT	exonic	FNIP1	[illegible]
5	131554331	G	A	GTCACAGAGG,AAGTGTCTGA	exonic	P4HA2	[illegible]
5	140255286	G	A	CCTTCTGGAG,TAAATCTGCA	exonic	PCDHA12	[illegible]
5	140515188	G	A	TGGGGTGGGG,AACTGGCCAC	exonic	PCDHB5	[illegible]
5	140554724	C	T	ACCAATTATC,CCAACCTGCT	exonic	PCDHB7	[illegible]
5	140580557	G	A	CTACACGTTG,AAACAGAGAG	exonic	PCDHB11	[illegible]
5	140784419	G	A	GGCCCTGCTA,ATAGAGATGC	exonic	PCDHGA9	[illegible]
5	151179498	C	T	ACAAAGACCT,AGCGGGATCA	exonic	G3BP1	[illegible]
5	169780953	G	T	GCTCATCACT,GGACCCTCAG	exonic	KCNIP1	[illegible]
5	176024329	C	T	GGCCGAGCCC,CAGCGCCGTC	exonic	GPRIN1	[illegible]
5	176829609	G	A	GGGGAGGATG,AGGATCCGTG	exonic	F12	[illegible]
5	178507054	C	T	CTCACTTACC,AGTATCAGAG	exonic	ZNF354C	[illegible]
6	2678521	C	T	TGAAATTCTT,ATCCTCTAAG	exonic	MYLK4	[illegible]
6	2779649	C	T	CAATCCTATT,TCCCAGTAGA	exonic	WRNIP1	[illegible]
6	6002692	C	T	AAGACCGCAT,GCACTTGCCC	exonic	NRN1	[illegible]
6	6145941	G	A	ATCAGCTTCC,ATGCCCAGAG	exonic	F13A1	[illegible]
6	7373595	G	A	CTCCTGTAAA,ACAAGAACTC	exonic	CAGE1	[illegible]
6	7386060	G	A	TCTTCACAAA,TGTGGATTCA	exonic	CAGE1	[illegible]
6	11104385	C	G	TTGGCTATTT,CTTTGAGAGC	exonic	ERVFRD-1	[illegible]
6	17804615	G	A	CTGATGATAG,GACTGCATAC	exonic	KIF13A	[illegible]
6	24588943	G	A	TGTGAGCACT,GGCTTTTCTC	exonic	KIAA0319	[illegible]
6	25861881	C	T	GAGTCTGCTT,GTTCTTGTGG	exonic	SLC17A3	[illegible]
6	26507008	G	A	TTTTCACTTG,AGACTATACA	exonic	BTN1A1	[illegible]
6	28269228	T	A	GAATAAAAAT,TCCTCTTGTA	exonic	PGBD1	[illegible]
6	28333474	A	T	AACCCTACGA,TGTGAAGAGT	exonic	ZKSCAN3	[illegible]
6	29430096	C	T	ATCTCTGATT,GACTCTCCTG	exonic	OR2H1	[illegible]
6	30630479	C	T	GAGGACAGTT,GCTCTGATGT	exonic	DHX16	[illegible]
6	31557903	C	T	AGCACAGGAT,CTGGGGGCAG	exonic	NCR3	[illegible]
6	31597511	C	T	CCCACCCATG,CCCCAATGAA	exonic	PRRC2A	[illegible]
6	31860275	G	A	CTCCTCCAGG,ACCCGGGGTC	exonic	EHMT2	[illegible]
6	31938450	T	C	ACATAGCAGG,TGGGGGCTGT	exonic	DXO	[illegible]
6	32057012	T	G	GTGGTGATGG,CTTGGAGGCA	exonic	TNXB	[illegible]
6	33747922	G	A	GGTCAGTGCG,CTTCAAACTT	exonic	LEMD2	[illegible]
6	34826789	C	T	GCTTATGCAT,CTGCACCCGG	exonic	UHRF1BP1	[illegible]
6	39563871	C	T	TACTTGGCCT,TGTTAGAAGA	exonic	KIF6	[illegible]
6	39602695	C	T	TTGTAGATTT,CAAATAGGAA	exonic	KIF6	[illegible]
6	40360041	C	T	AGCAGCTCCT,CTTTCTCTGA	exonic	LRFN2	[illegible]
6	41309598	G	A	CAGACCCAGA,CTGTGTGCCT	exonic	NCR2	[illegible]
6	42072312	C	T	GTGCAGGCCT,CGGGGGGCGC	exonic	C6orf132	[illegible]
6	42890942	C	T	TATGGCCCTT,CCCAGCAACG	exonic	PTCRA	[illegible]
6	43155670	G	A	GTCCAAGTCG,AGGCCAGCTT	exonic	CUL9	[illegible]
6	44273437	G	A	GAGTCTAGCT,GACCCCTTTC	exonic	AARS2	[illegible]
6	46135549	G	A	TAATGAGTAG,AAAGCGCTTA	exonic	ENPP5	[illegible]
6	46828528	C	T	TCCCAGACTC,CAGGAGAAGA	exonic	GPR116	[illegible]
6	51917985	G	A	TTTGCCGGAG,GGGCTGGAGA	exonic	PKHD1	[illegible]
6	52884052	G	A	ATCTGTATAT,GAGGTTTTGA	exonic	ICK	[illegible]
6	55128576	G	A	GTGTCTCATG,TGTTGGCTTA	exonic	HCRTR2	[illegible]
6	56470197	G	A	TTTTTACCAG,CAAAAGTTTG	exonic	DST	[illegible]
6	56506788	G	A	CCATCAATAA,AATCTGTACA	exonic	DST	[illegible]
6	70411421	G	A	CCAGCTGAAT,AAGAGCTTTA	exonic	LMBRD1	[illegible]
6	70744173	G	A	TGGTCCACCT,GCCCAAAAGG	exonic	COL19A1	[illegible]
6	70744174	G	A	GGTCCACCTG,CCCAAAAGGA	exonic	COL19A1	[illegible]
6	80223225	T	C	TTCTCCTGTC,GTACTGAAGC	exonic	LCA5	[illegible]
6	86347013	C	T	TGGAGGTGGT,CTCCATACTT	exonic	SYNCRIP	[illegible]
6	89814880	G	A	TCCCCATGAA,AACTTCTACT	exonic	SRSF12	[illegible]
6	93979264	C	T	TTTCCATAAC,AGCAGCAGTA	exonic	EPHA7	[illegible]
6	94124474	C	T	GCTTTAGAAT,CAGCAGTAGT	exonic	EPHA7	[illegible]
6	96651941	G	A	GTATCTGAAG,AAGTCGACAA	exonic	FUT9	[illegible]
6	105845762	G	A	TTCAAGCCAG,CGTAAGGGTC	exonic	PREP	[illegible]
6	117642495	C	T	TCTTTGTCTT,GTTTATAAGC	exonic	ROS1	[illegible]
6	117715853	C	T	AGATCTCTTT,TTAGAGAAGT	exonic	ROS1	[illegible]
6	127476413	C	T	GAATGGAATC,TTGGAGTCCA	exonic	RSPO3	[illegible]
6	127517127	C	T	AAACAGAAAT,GGTATCAGTC	exonic	RSPO3	[illegible]
6	128134294	G	A	CACTCCGTGG,GTTGGCAAAG	exonic	THEMIS	[illegible]
6	131216174	G	A	TTTCGGCCAA,CAAAACGATT	exonic	EPB41L2	[illegible]
6	131490326	T	C	GACTTTGCCC,TTCAAGGGAT	exonic	AKAP7	[illegible]
6	132061519	C	T	GAGAGCTGTC,TGTGAGTATG	exonic	ENPP3	[illegible]
6	135265068	G	A	GACCAGCTGG,AAAGGCTTCT	exonic	ALDH8A1	[illegible]
6	145157053	C	T	AGGATCATTG,TGACCTGGAG	exonic	UTRN	[illegible]
6	150210569	C	A	TCCTGAAATA,TTTTCCAGCC	exonic	RAET1E	[illegible]
6	150385754	G	A	CAGATGCCAG,GAGGATGAAG	exonic	ULBP3	[illegible]
6	152631852	G	A	TTCTGCAAAC,AATTTTGATC	exonic	SYNE1	[illegible]
6	152730788	G	A	AGATACAGCT,ATTTCAGGTT	exonic	SYNE1	[illegible]
6	154411260	C	T	TGGATCCTCT,TTCAGCCATT	exonic	OPRM1	[illegible]
6	157522148	C	T	TATGCCTTAT,CCTACCAGAA	exonic	ARID1B	[illegible]
6	161012086	G	A	ATAACACCAA,GACTAATCTC	exonic	LPA	[illegible]
6	165863793	G	A	GTGTCCAACC,TTGTTCTATA	exonic	PDE10A	[illegible]
7	1022924	C	T	CAAGACCCCT,CCCAGCTGCC	exonic	CYP2W1	[illegible]
7	5372426	C	T	GTCCACAGGT,GTCGTCGCTG	exonic	TNRC18	[illegible]
7	5765051	T	A	TGCCATTTTT,AATGGCATCA	exonic	RNF216	[illegible]
7	6370187	G	A	GAGCACTTCG,GATACACGTG	exonic	FAM220A	[illegible]
7	11441595	C	T	ATAACAGTCA,CTGTAAAACA	exonic	THSD7A	[illegible]
7	18833016	C	A	CGGTGCTGCA,GCATGGCTGT	exonic	HDAC9	[illegible]
7	18875162	C	T	TTCACTCCAT,GCTATGATGA	exonic	HDAC9	[illegible]
7	19184765	C	T	CTCCTCTTCT,CGTCCCCCTG	exonic	FERD3L	[illegible]
7	19763907	T	A	GCGTACCTTG,AGGTAACTCT	exonic	TMEM196	[illegible]
7	27147962	G	A	TAGGTACCCT,GGGGGGCTTG	exonic	HOXA3	[illegible]
7	28547275	G	A	GAACTGCGAG,AGGTGGGCCT	exonic	CREB5	[illegible]
7	31682521	G	A	GGCCATGGAG,GGCCACCAGA	exonic	CCDC129	[illegible]
7	34867012	G	A	TCTTTGGGCA,AAAAGCAAGC	splicing	NPSR1(ENST00000381542.1:exon3:c.281-1G>A,ENST00000360581.1:exon5:c.479-1G>A,ENS[illegible]	[illegible]
7	36763739	C	T	TAAGGATTTT,CAGGGGGACT	exonic	AOAH	[illegible]
7	37947169	C	T	AGGAGGCTTT,CCTTTGGTTT	exonic	SFRP4	[illegible]
7	39243918	G	T	TTTCCAGTTG,GCCACAGGTC	exonic	POU6F2	[illegible]
7	45124460	G	T	CCAGGACACA,TCCCATCCTG	exonic	NACAD	[illegible]
7	45748057	C	T	CTTTGTCCTC,GAGTTGGTAT	exonic	ADCY1	[illegible]
7	47342608	G	A	TTTGGGAAGG,GATACTGATG	exonic	TNS3	[illegible]
7	47942026	C	T	TGGCAGGGCT,GCACACGATG	exonic	PKD1L1	[illegible]
7	48318242	G	A	GCTATTTCAA,AGCAAGTGAA	exonic	ABCA13	[illegible]
7	48318922	G	A	TATAAAATGG,AAATAATTCA	exonic	ABCA13	[illegible]
7	64452195	G	A	GCTTCAAGTT,GTACCAAGAA	exonic	ERV3-1	[illegible]
7	65419185	C	T	TCATCTGCAT,GTCACGTACG	exonic	VKORC1L1	[illegible]
7	82580738	C	T	CTAATCCCAG,TCCTGAAATG	exonic	PCLO	[illegible]
7	87069054	G	A	TCCAGCAGAA,GATCTTGGGG	exonic	ABCB4	[illegible]
7	90585030	C	T	AGAGCAAAAT,CGTCCCTAGC	exonic	CDK14	[illegible]
7	92404042	G	A	CCAGGCTCTG,AACTTTATCC	exonic	CDK6	[illegible]
7	92760805	C	T	AAGTACTGCT,TATTTTGGCC	exonic	SAMD9L	[illegible]
7	94049938	C	T	CCCACAGGCC,CGTTGGAGCT	exonic	COL1A2	[illegible]
7	100151828	C	T	GGTGGAGACC,CTTTGCTGCA	exonic	AGFG2	[illegible]
7	100349760	C	T	GCCTGTCATC,CTACAGAAAA	exonic	ZAN	[illegible]
7	102608488	A	G	CAGTGAGTTT,GTTGTGTCAT	exonic	FBXL13	[illegible]
7	103194237	C	T	TTATTTCCAT,GATAATGAAG	exonic	RELN	[illegible]
7	103243770	G	A	GTACAGAGAT,ATCCAGAAGA	exonic	RELN	[illegible]
7	106513328	A	T	TGTTACAAAA,GTCACCCTTG	exonic	PIK3CG	[illegible]
7	108131887	C	T	CTGTTTCAAT,ATCAGTGCAG	exonic	PNPLA8	[illegible]
7	111584927	C	T	TGCCCAAATC,TACAAACAAA	splicing	DOCK4(ENST00000428084.1:exon11:c.784-1G>A,ENST00000437633.1:exon11:c.784-1G>A,E[illegible]	[illegible]
7	112723940	C	T	TTCTTTTCTC,ATTTTGAACT	exonic	GPR85	[illegible]
7	114271596	A	T	CAGCAGCAGC,GCAGCAGCAA	exonic	FOXP2	[illegible]
7	116422046	C	T	CTGCAGAATC,AACTGTAAAA	exonic	MET	[illegible]
7	121731807	C	T	ATCAAAACTC,CACTGGACTC	exonic	AASS	[illegible]
7	123143309	C	T	TCCATCTATT,TTCTGACTGG	exonic	IQUB	[illegible]
7	123509127	C	T	GTCTGGAAAT,CCTTGGAGAC	exonic	HYAL4	[illegible]
7	128357012	G	A	AACCAGACAT,GAACAGACCA	exonic	FAM71F1	[illegible]
7	128432444	G	A	GCCAACGCTG,GGGTCCCTGG	exonic	CCDC136	[illegible]
7	128432445	G	A	CCAACGCTGG,GGTCCCTGGA	exonic	CCDC136	[illegible]
7	128478755	C	T	CAGCACGTTC,GCTGCACATA	exonic	FLNC	[illegible]
7	132070005	C	T	TCTGTGGGAT,GGGGTCCCGT	exonic	PLXNA4	[illegible]
7	139167964	G	A	CCTGGGCGAT,GCGTGCTGCT	exonic	KLRG2	[illegible]
7	140287545	G	A	GCTCTCAGAG,AAGACTGCAG	exonic	DENND2A	[illegible]
7	141170537	C	T	CAACCTGTCC,GAGGACCAAC	exonic	TMEM178B	[illegible]
7	142749553	G	A	GCCTTCATGG,AAACACCATC	exonic	OR6V1	[illegible]
7	143079514	G	A	GGGAGGGCCT,AGGCCCCCAT	exonic	ZYX	[illegible]
7	143632486	G	T	CTGGACAGCC,ACTCCACACT	exonic	OR2F2	[illegible]
7	147600720	C	T	TGGGGAGGCT,TGGGCCTGGA	exonic	CNTNAP2	[illegible]
7	148949862	G	A	CTTGGAAGAG,AAGGTCCTGG	exonic	ZNF212	[illegible]
7	150171133	C	T	TCCACAGGAC,CGAGCAGAAT	exonic	GIMAP8	[illegible]
7	150488678	C	T	ACTGAATTCT,CCCCAGTAGC	exonic	TMEM176B	[illegible]
7	151962134	G	T	TGCAGTTCTG,CACACTTTGC	exonic	KMT2C	[illegible]
7	151962259	G	A	AGGTCTCCCG,GCTGTCGCAC	exonic	KMT2C	[illegible]
8	2886864	C	T	CCTCTCATCT,CTATGTTCCA	exonic	CSMD1	[illegible]
8	3000164	C	T	TGATTAGCTT,GGTAGAAAAA	exonic	CSMD1	[illegible]
8	9592976	C	T	AATTCCTCTT,ATAATGCGGC	exonic	TNKS	[illegible]
8	11412366	C	T	ATCACCTTCC,CTCGCTCCAG	exonic	BLK	[illegible]
8	13948001	C	T	GGAACCTACT,CTGCTGGAGA	exonic	SGCZ	[illegible]
8	19684066	C	T	AGAGAAAAAA,CATGGTAAGG	exonic	INTS10	[illegible]
8	21985239	C	T	CTCCCCGGCT,TCTGCAGGTG	exonic	HR	[illegible]
8	25167981	T	A	AGAAGAATTT,TCACACTTGG	exonic	DOCK5	[illegible]
8	27880877	C	T	GGGGGATGCC,TTAGGAGGAA	exonic	NUGGC	[illegible]
8	30002033	G	A	ATGATGCAGA,ATAATTGAAG	exonic	MBOAT4	[illegible]
8	30703397	A	C	ATAACCTTGA,GGTCACAACT	exonic	TEX15	[illegible]
8	32505319	C	T	GCAGACCCAT,TCTTGATGGG	exonic	NRG1	[illegible]
8	33247239	G	A	TTGTTTTTCG,GGACTCTTCA	exonic	FUT10	[illegible]
8	33367406	C	A	TTGCAGAGTC,ATGAGAAAAC	exonic	TTI2	[illegible]
8	36768629	C	G	CCGTCACCCT,AGTGTCAGGT	exonic	KCNU1	[illegible]
8	37729154	G	C	CTCTTGCCAA,ATGTGGTTTG	exonic	RAB11FIP1	[illegible]
8	37793376	C	T	ACTAGCATCC,CACTCCTTCA	exonic	GOT1L1	[illegible]
8	38386061	G	A	CAGCTTCCCG,ACTGGCTCAG	exonic	C8orf86	[illegible]
8	38386063	C	A	GCTTCCCGGA,TGGCTCAGGC	exonic	C8orf86	[illegible]
8	39103675	C	T	CAACAGTGTT,TGGACATGGA	exonic	ADAM32	[illegible]
8	41164366	C	T	TATGGGGTTT,CCCATCCCAT	exonic	SFRP1	[illegible]
8	42611783	C	T	ATTAGAAGAT,AATTTCAGCT	exonic	CHRNA6	[illegible]
8	48715868	C	A	TCAACTTACC,AACAAAGAGA	exonic	PRKDC	[illegible]
8	49831395	C	A	CCAGATTCCT,ATGTTTGTGC	exonic	SNAI2	[illegible]
8	53126851	C	A	GAGGTCATTC,TTTGACATTG	splicing	ST18	[illegible]
8	56637794	G	A	GTTTATATGT,AAAGCATGCC	exonic	TMEM68	[illegible]
8	63978498	G	A	GCTACGGATG,AGTGATTTGA	exonic	TTPA	[illegible]
8	67590068	T	C	CGGTGGCTCA,GCCTGTAATC	exonic	C8orf44	[illegible]
8	67590079	C	T	GCCTGTAATC,CAGCACTTTG	exonic	C8orf44	[illegible]
8	69380955	G	A	ATCCAAAGTG,AACTGGAGGA	exonic	C8orf34	[illegible]
8	72964843	C	T	CCTTTTGCTC,TGATGATCGT	exonic	TRPA1	[illegible]
8	75227481	C	T	GAGTTGGCAT,GCTGGAACTA	exonic	JPH1	[illegible]
8	76471270	G	A	ATGCTATTAG,TGGTGAGTAC	exonic	HNF4G	[illegible]
8	77775566	G	A	CAAAGAGGAG,AATTAGAGGC	exonic	ZFHX4	[illegible]
8	81083672	G	A	TGCTCGCCGC,GTCCATGTCT	exonic	TPD52	[illegible]
8	81733756	G	A	CATGGCCAAG,AAAACACGTG	exonic	ZNF704	[illegible]
8	94746870	G	C	CTCCTCAGAA,GCCGCCTGAA	exonic	RBM12B	[illegible]
8	98831380	C	T	TCCTAATTTT,CCTACAGAGA	exonic	LAPTM4B	[illegible]
8	99019788	C	T	TGTCAGTGTC,TGAGGGACAC	exonic	MATN2	[illegible]
8	99441162	G	A	CCGCTCCCTG,GGGCCACTTT	exonic	KCNS2	[illegible]
8	99441163	G	A	CGCTCCCTGG,GGCCACTTTG	exonic	KCNS2	[illegible]
8	100899771	C	T	TTCCTCATCT,CTCAAAATCT	exonic	COX6C	[illegible]
8	101719207	C	A	GCGGATAGCA,CGGGCATATT	exonic	PABPC1	[illegible]
8	109797062	C	T	TTGGTTGTTC,CTGAGTGGAG	exonic	TMEM74	[illegible]
8	113812430	C	T	CCAACACTTT,GTCCGTTTGA	exonic	CSMD3	[illegible]
8	116427180	C	T	TTGACTTGCT,CTCATTGCTG	exonic	TRPS1	[illegible]
8	124748096	C	T	TGACTGCCTT,TGAGAGGGTG	exonic	ANXA13	[illegible]
8	125076650	C	T	CCAGGATCCC,CAGCAGATCA	exonic	FER1L6	[illegible]
8	133895131	G	A	AGCTGCCGCC,AAATGGCGAC	exonic	TG	[illegible]
8	136594240	C	T	CTTCTCACTC,CAGAGCAAGA	exonic	KHDRBS3	[illegible]
8	143561141	C	T	GTCCGGTGTC,CCGCAACGCC	exonic	BAI1	[illegible]
8	143614825	G	A	CCTCCGTAGA,AGGTGGGTGA	exonic	BAI1	[illegible]
8	145138336	G	A	CGGTCAGTAG,GCTGGAGGTC	exonic	GPAA1	[illegible]
9	1051569	G	A	TGTTTCCCCA,AGTGAGGGCC	splicing	DMRT2	[illegible]
9	5231687	G	A	ACCACCCCAG,AGGGTGGCTG	exonic	INSL4	[illegible]
9	8471007	C	T	CATCTAATTC,ATTTCATCTG	exonic	PTPRD	[illegible]
9	8485939	C	T	ATGTTGATAT,CCTATAAAGA	exonic	PTPRD	[illegible]
9	14722664	G	A	AATAAGAGGA,ATGCATGCTG	exonic	CER1	[illegible]
9	14859183	G	A	CATCATACGG,ACTCTGGGAA	exonic	FREM1	[illegible]
9	16436826	G	A	TGAATTTTGA,AGTACCTTTG	exonic	BNC2	[illegible]
9	18928418	C	T	TTGACTGGCT,TGTGCGCATG	exonic	FAM154A	[illegible]
9	20949664	C	T	GTCATTAGAA,CTTAACTCAG	exonic	FOCAD	[illegible]
9	21409338	G	A	CTGCCTGAAG,ACAGACATGA	exonic	IFNA8	[illegible]
9	24545479	C	T	TGAATCATCT,CTTAATCTGC	exonic	IZUMO3	[illegible]
9	27950602	C	T	AATGGTGGAT,CCATGAAGAT	exonic	LINGO2	[illegible]
9	32784458	G	A	GGAGGTGGTA,AGCTGCTGAG	exonic	TMEM215	[illegible]

9	33548603	C	T	GAAAGTCAAT,CATTGGAAAG	exonic	ANKRD18B	[illegible]	[illegible]	9p13.3	.
9	34401033	G	A	GACTTGGGGG,GATGAGGTCT	exonic	FAM219A	[illegible]	[illegible]	9p13.3	.
9	35754116	C	T	CCAGAGCATC,TTAGGGCCAT	exonic	MSMP	[illegible]	[illegible]	9p13.3	.
9	35794014	C	T	GGAGAGTCTC,GTGCAGGCCC	exonic	NPR2	[illegible]	[illegible]	9p13.3	.
9	36170127	G	A	CTTCCGGAAG,AGGATCGGGA	exonic	CCIN	[illegible]	[illegible]	9p13.3	CCIN;basechange=c.628G>A;AAchange=p.E210K;tissue=skin
9	37708447	A	G	CTCCAAATGA,CAATGAGCCT	exonic	FRMPD1	[illegible]	[illegible]	9p13.2	.
9	37745789	C	T	TAATCCTGAG,CTTCCCTGCC	exonic	FRMPD1	[illegible]	[illegible]	9p13.2	.
9	73376575	C	T	AGCTTGGGTT,GAGTGTATGT	exonic	TRPM3	[illegible]	[illegible]	9q21.12	.
9	73391357	C	T	CCTTTGCTTT,CTCTTCCTTT	exonic	TRPM3	[illegible]	[illegible]	9q21.12	.
9	77411734	G	A	TGGGACTGGG,AACATGTGAC	exonic	TRPM6	[illegible]	[illegible]	9q21.13	.
9	78547359	C	T	TCAGTTATCT,GAGCAGAGGG	exonic	PCSK5	[illegible]	[illegible]	9q21.13	.
9	79325970	G	A	ATTGAGATCT,GAGGGTTCTC	exonic	PRUNE2	[illegible]	[illegible]	9q21.2	.
9	79325971	G	A	TTGAGATCTG,AGGGTTCTCT	exonic	PRUNE2	[illegible]	[illegible]	9q21.2	.
9	80336429	C	T	TCTCTGGGGT,CTTTCGGCCA	exonic	GNAQ	[illegible]	[illegible]	9q21.2	.
9	84606275	G	A	CGTCATCACG,ACCACCAATC	exonic	SPATA31D1	[illegible]	[illegible]	9q21.32	.
9	84608375	G	A	GTCCAAGAAG,GCAGGGGACC	exonic	SPATA31D1	[illegible]	[illegible]	9q21.32	.
9	86912823	G	A	AAACTTACCT,AACTTGTCTG	exonic	SLC28A3	[illegible]	[illegible]	9q21.33	.
9	87482238	T	C	CACTCCATCT,CTTCGGAAGG	exonic	NTRK2	[illegible]	[illegible]	9q21.33	.
9	90586064	G	A	AGACTGACCC,ATGTACAATG	exonic	CDK20	[illegible]	[illegible]	9q22.1	.
9	98775222	C	T	AAAGAAAGTT,TTTCAGGGCC	exonic	ERCC6L2	[illegible]	[illegible]	9q22.32	C9orf102_ENST00000407474;basechange=c.1333C>A;AAchange=p.L445I;tissue=endometrium
9	99017188	C	G	TTCCAGCGTC,GGCTAATAAG	exonic	HSD17B3	[illegible]	[illegible]	9q22.32	.
9	99735179	G	A	AAGAATTCAA,GAAGATGACA	exonic	HIATL2	[illegible]	[illegible]	9q22.33	.
9	100092924	G	A	GAAGGGTCAA,GAGAAAAGAA	exonic	CCDC180	[illegible]	[illegible]	9q22.33	.
9	100111488	G	A	CCAGTTCCAA,AAGAGCAAAA	exonic	CCDC180	[illegible]	[illegible]	9q22.33	.
9	100983435	C	T	TCCTCAGCCG,CGCCAGGTAT	exonic	TBC1D2	[illegible]	[illegible]	9q22.33	.
9	103348606	G	A	GAGGCAGCCA,GCCGGTGTAT	exonic	MURC	[illegible]	[illegible]	9q31.1	.
9	104161443	C	T	TGGGAGATGG,TTCCCCGAGC	exonic	ZNF189	[illegible]	[illegible]	9q31.1	.
9	104187217	C	T	AGGGCCCGTC,ATAAGAGAAA	exonic	ALDOB	[illegible]	[illegible]	9q31.1	.
9	111617829	C	T	TCCCAGATGT,CTGCACGCAG	exonic	ACTL7B	[illegible]	[illegible]	9q31.3	.
9	112899182	G	A	GAAAAGGAGA,GAGAGAGCTC	exonic	AKAP2,PALM2-AKAP2	[illegible]	[illegible]	9q31.3	.
9	112963737	G	A	GATTCTGCAG,AGCAGGTGTG	exonic	C9orf152	[illegible]	[illegible]	9q31.3	.
9	113510000	G	A	ACCAAGCCAG,ACTCTACACA	exonic	MUSK	[illegible]	[illegible]	9q31.3	.
9	114173400	G	A	TGCAAGCAAG,ATCCATGCTG	exonic	KIAA0368	[illegible]	[illegible]	9q31.3	.
9	116855594	G	A	GCAGGGGCAG,AGTAGAGGGG	exonic	KIF12	[illegible]	[illegible]	9q32	.
9	117552979	C	T	CTTGTTTGGT,GGCCTGCTTG	exonic	TNFSF15	[illegible]	[illegible]	9q32	.
9	118949681	G	A	CACCTCTGGG,AACAAGTGGG	exonic	PAPPA	[illegible]	[illegible]	9q33.1	.
9	119188149	C	T	CTTGGACTCC,CGTACGTGTT	exonic	ASTN2	[illegible]	[illegible]	9q33.1	.
9	120475640	A	T	CAATGGTGTT,TTACCATGAG	exonic	TLR4	[illegible]	[illegible]	9q33.1	.
9	121929579	G	A	CATCACTGAC,AAGAGGAATA	exonic	BRINP1	[illegible]	[illegible]	9q33.1	.
9	124929162	G	A	AAGCCAATAC,ACGCCATTTG	exonic	MORN5	[illegible]	[illegible]	9q33.2	.
9	125681240	G	A	ACACTGGAAA,GTTTAATTCC	exonic	ZBTB26	[illegible]	[illegible]	9q33.2	.
9	127572366	C	T	GAGGCCCAGC,CGAGGTGATC	exonic	OLFML2A	[illegible]	[illegible]	9q33.3	.
9	133352330	G	A	TGACATTCTC,AGATCGAGTT	exonic	ASS1	[illegible]	[illegible]	9q34.11	.
9	134073533	C	T	GTCAGCAACT,TGGCACTGCA	exonic	NUP214	[illegible]	[illegible]	9q34.13	.
9	135267547	G	A	TCATTCCCAA,GAGAGAATGG	exonic	TTF1	[illegible]	[illegible]	9q34.13	.
9	135517422	G	A	TCTGGAATGT,AAAATTCCTG	exonic	DDX31	[illegible]	[illegible]	9q34.13	.
9	135771690	G	A	TCCGGGGTCG,GGGAGACGGG	exonic	TSC1	[illegible]	[illegible]	9q34.13	.
9	136915581	G	A	GACTGGGACC,ACGTGACGTT	exonic	BRD3	[illegible]	[illegible]	9q34.2	.
9	137019599	C	T	TGACGACAAC,CCCCCGTGTC	exonic	WDR5	[illegible]	[illegible]	9q34.2	.
9	137019600	C	T	GACGACAACC,CCCCGTGTCT	exonic	WDR5	[illegible]	[illegible]	9q34.2	.
9	137620555	G	A	CTATTACTAC,AATACCCCTA	exonic	COL5A1	[illegible]	[illegible]	9q34.3	COL5A1;basechange=c.826G>A;AAchange=p.E276K;tissue=endometrium
9	138453712	C	T	ATGGACATCC,CCAGACCAAG	exonic	PAEP	[illegible]	[illegible]	9q34.3	.
9	139325534	G	A	GGGAGGAAGT,GATGTCCGGC	exonic	INPP5E	[illegible]	[illegible]	9q34.3	.
9	139333442	G	A	AGGACCCCGC,GGACTTGGGG	exonic	INPP5E	[illegible]	[illegible]	9q34.3	.
9	139333781	G	A	TGGGCCGGCG,AGCGCCGGGA	exonic	INPP5E	[illegible]	[illegible]	9q34.3	.
10	6005720	G	A	TCCAGAAGGG,AGAGGCTCTC	exonic	IL15RA	[illegible]	[illegible]	10p15.1	.
10	14563993	C	T	TTCTGCAATT,TGGTTTGTTC	exonic	FAM107B	[illegible]	[illegible]	10p13	.
10	15614218	C	T	ACCTTTATAT,CTGTGGATTG	exonic	ITGA8	[illegible]	[illegible]	10p13	.
10	15639298	C	T	GGTCGAAATC,CTACAATTGC	exonic	ITGA8	[illegible]	[illegible]	10p13	.
10	15688970	T	G	CAAATACAGG,AGATTTGCCC	exonic	ITGA8	[illegible]	[illegible]	10p13	ITGA8;basechange=c.1082A>T;AAchange=p.Y361F;tissue=skin
10	15761572	C	T	GTGCGGGCGT,GGGTATGTGG	exonic	ITGA8	[illegible]	[illegible]	10p13	.
10	16737103	G	A	GGTCACCCAG,GATTGTTCTC	exonic	RSU1	[illegible]	[illegible]	10p13	.
10	16975127	G	A	CGTTCGGTGA,ATTCAATGTC	exonic	CUBN	[illegible]	[illegible]	10p13	.
10	21104571	G	A	ATATTTTCTT,ATTCTTCTTC	exonic	NEBL	[illegible]	[illegible]	10p12.31	.
10	27337757	G	A	TTCCTTCCTG,GAAATTGCTG	exonic	ANKRD26	[illegible]	[illegible]	10p12.1	ANKRD26;basechange=c.1787C>A;AAchange=p.P596H;tissue=large_intestine
10	35929628	C	T	GGGTGGCGCT,GCTGGACACG	exonic	FZD8	[illegible]	[illegible]	10p11.21	.
10	36812589	G	A	TTAACCAAAT,AGCAGATGCT	exonic	NAMPTL	[illegible]	[illegible]	10p11.21	.
10	37431003	G	A	GACACACCTA,GGAAATTATG	exonic	ANKRD30A	[illegible]	[illegible]	10p11.21	.
10	45907642	G	T	TGCTCAGATG,ATGGAGTGGA	exonic	ALOX5	[illegible]	[illegible]	10q11.21	.
10	45919493	G	A	CTGCAGGATG,AGAACCTGTT	exonic	ALOX5	[illegible]	[illegible]	10q11.21	.
10	48390309	C	T	GATGGTGTTC,CTGGGTGCAG	exonic	RBP3	[illegible]	[illegible]	10q11.22	.
10	48429084	G	A	GCAGGGAAGG,GTCGTATCTC	exonic	GDF10	[illegible]	[illegible]	10q11.22	.
10	49663044	C	T	GAGCCACTGA,CTCCCCCTCG	splicing	ARHGAP22(ENST00000249601.4:exon7:c.792+1G>A,ENS[illegible]			10q11.22	.
10	67748504	C	T	ATTCTGATAT,ATTTTCGCTG	exonic	CTNNA3	[illegible]	[illegible]	10q21.3	.
10	70043986	A	G	TCTAATGTCA,CCCTTCCGTC	exonic	PBLD	[illegible]	[illegible]	10q21.3	.
10	72631618	C	T	CAAAATACCC,TTCATGTCGA	exonic	SGPL1	[illegible]	[illegible]	10q22.1	.
10	73326512	G	A	ACACCAGTGG,GACGCCCATC	exonic	CDH23	[illegible]	[illegible]	10q22.1	.
10	74631253	C	T	TGGGAATATT,CTGGGACATC	exonic	MCU	[illegible]	[illegible]	10q22.1	.
10	75000739	G	A	ATCTCAAAGC,GAGGCAGACC	exonic	FAM149B1	[illegible]	[illegible]	10q22.2	.
10	75523356	C	T	GGTGAAAGAC,AAGGTGAGAA	exonic	SEC24C	[illegible]	[illegible]	10q22.2	.
10	79584152	G	A	CTTCAGGAGG,ACAGGGTCAG	exonic	DLG5	[illegible]	[illegible]	10q22.3	.
10	91222265	T	C	TTTTTCTTCT,TTCCAGGTTG	exonic	SLC16A12	[illegible]	[illegible]	10q23.31	.
10	94700583	T	C	AGTTTTCAGG,TAGTCTAAGT	splicing	EXOC6(ENST00000371547.4:exon14:c.1358+2T>C,ENST0[illegible]			10q23.33	.
10	94834704	G	A	CATCCTACTG,GCTGCGAACC	exonic	CYP26A1	[illegible]	[illegible]	10q23.33	.
10	95791497	G	A	AAACTATGTG,CATATACCTG	exonic	PLCE1	[illegible]	[illegible]	10q23.33	.
10	97733858	G	A	GAGCTTAGAT,AAAATGGTCT	exonic	RP11-248J23.6,RP11-690P14.4	[illegible]	[illegible]	10q24.1	.
10	98097971	G	A	CCGTGGGAAA,AAATGCCTAG	exonic	DNTT	[illegible]	[illegible]	10q24.1	.
10	98802741	C	T	TCGCGGGTTC,CCGTCACGAT	exonic	SLIT1	[illegible]	[illegible]	10q24.1	.
10	99655719	C	T	ATCCCGTCTC,GTCGAAGTCG	exonic	CRTAC1	[illegible]	[illegible]	10q24.2	.
10	101090066	G	A	GCTGCCGCCG,GCTTCGGGGG	exonic	CNNM1	[illegible]	[illegible]	10q24.2	.
10	101090067	G	A	CTGCCGCCGG,CTTCGGGGGC	exonic	CNNM1	[illegible]	[illegible]	10q24.2	.
10	101464367	G	A	CGACAAACAC,AGCCTCAGCT	exonic	ENTPD7	[illegible]	[illegible]	10q24.2	.
10	101553692	C	T	TTCTTCATCT,CTACGGATTC	exonic	ABCC2	[illegible]	[illegible]	10q24.2	.
10	102738674	G	A	TGATGACAAG,TGTACTACTT	exonic	SEMA4G	[illegible]	[illegible]	10q24.31	.
10	104156053	C	T	AAATTGAACT,CTCCATTGTG	exonic	NFKB2	[illegible]	[illegible]	10q24.32	.
10	105218346	C	T	AGCAGGATGC,CGCGCTGTAG	exonic	CALHM1	[illegible]	[illegible]	10q24.33	.
10	105799742	C	T	TTGGTGTCCT,TGGGGCCTGG	exonic	COL17A1	[illegible]	[illegible]	10q24.33	.
10	108431071	G	A	ATACACTTCC,CTCTGATTTT	exonic	SORCS1	[illegible]	[illegible]	10q25.1	SORCS1;basechange=c.2113C>T;AAchange=p.R705W;tissue=large_intestine,endometrium&SORCS1_ENST00000344440;basechange
10	111884004	G	A	TCTCGGAACG,AGAAACCAGT	exonic	ADD3	[illegible]	[illegible]	10q25.1	ADD3;basechange=c.1373G>A;AAchange=p.G458E;tissue=skin
10	115406696	C	T	CTAGCGAGTT,GTGAGCTTTC	exonic	NRAP	[illegible]	[illegible]	10q25.3	NRAP;basechange=c.979G>A;AAchange=p.E327K;tissue=large_intestine,endometrium&NRAP;basechange=c.979G>T;AAchange=p.E3
10	116247757	G	A	CTTACAGTCG,GATGCCAAAC	exonic	ABLIM1	[illegible]	[illegible]	10q25.3	.
10	118891798	C	G	TCCTTTTCTT,TTTTGTTTTT	exonic	VAX1	[illegible]	[illegible]	10q25.3	.
10	119013973	G	A	AACGTCATGG,AATCGCCTTG	exonic	SLC18A2	[illegible]	[illegible]	10q25.3	.
10	121182718	C	T	GACCTGGTCT,CCAGACGGAG	exonic	GRK5	[illegible]	[illegible]	10q26.11	.
10	123263338	G	A	GGGTCCTCTG,AAGTTCATAC	exonic	FGFR2	[illegible]	[illegible]	10q26.13	.
10	129903656	G	A	TCATCTCCTG,TACTTTGTCT	exonic	MKI67	[illegible]	[illegible]	10q26.2	.
10	131565181	G	C	GCCAGGCTTG,GAGGGAGCTC	exonic	MGMT	[illegible]	[illegible]	10q26.3	.
10	133948654	G	A	TCGGAAGAGA,GAATGAGCTG	exonic	JAKMIP3	[illegible]	[illegible]	10q26.3	.
10	134660573	C	T	AGCAGCACCT,TGACGCCTGA	exonic	TTC40	[illegible]	[illegible]	10q26.3	TTC40;basechange=c.6130G>T;AAchange=p.E2044*;tissue=lung&C10orf92;basechange=c.613G>T;AAchange=p.E205*;tissue=lung
10	134660720	C	T	CTGCAGTGCT,CTCCGGGGCT	exonic	TTC40	[illegible]	[illegible]	10q26.3	.
10	134942917	G	A	CAGCCAGAAC,GGCTGCCCAA	exonic	GPR123	[illegible]	[illegible]	10q26.3	.
10	135192851	C	T	CGGGGAGGCC,CGGGCGGACC	exonic	PAOX	[illegible]	[illegible]	10q26.3	.
11	609061	C	T	AGGGAGGCTT,CCCAGCGCCC	exonic	PHRF1	[illegible]	[illegible]	11p15.5	.
11	639718	G	A	GCTGCTCATC,GCGCCACGTG	exonic	DRD4	[illegible]	[illegible]	11p15.5	.
11	639719	G	A	CTGCTCATCG,CGCCACGTGG	exonic	DRD4	[illegible]	[illegible]	11p15.5	.
11	1103555	G	A	TTCCCTCTAG,GCTCCATCAC	exonic	MUC2	[illegible]	[illegible]	11p15.5	.
11	1262641	G	A	TCAGTGGACA,AGTGGTTTGA	exonic	MUC5B	[illegible]	[illegible]	11p15.5	.
11	1780312	G	A	TACTTGTGGT,GATCCCTGCC	exonic	CTSD	[illegible]	[illegible]	11p15.5	.
11	1993862	G	A	TGCAGCAAGG,GTGGTCAACC	exonic	MRPL23	[illegible]	[illegible]	11p15.5	.
11	2154246	G	A	ATCTCTGGGG,GGCGCCCCCG	exonic	IGF2	[illegible]	[illegible]	11p15.5	.
11	2441626	G	A	TAGTGGACAG,AAAATCCTCC	exonic	TRPM5	[illegible]	[illegible]	11p15.5	.
11	2979739	G	A	GGTTTTGACA,TAACATTCTT	exonic	NAP1L4	[illegible]	[illegible]	11p15.4	.
11	3129002	G	A	CCCACTCACG,GAACAGGTCT	exonic	OSBPL5	[illegible]	[illegible]	11p15.4	.
11	4113009	A	G	AAAATCTTTA,GAAGCCTCTT	exonic	STIM1	[illegible]	[illegible]	11p15.4	.
11	4903613	C	T	ACTTCCCCTT,TTGTAGCCAT	exonic	OR51T1	[illegible]	[illegible]	11p15.4	OR51T1;basechange=c.565C>A;AAchange=p.L189I;tissue=large_intestine&ENSG00000176900;basechange=c.484C>A;AAchange=p.L
11	5009491	C	T	TGGTGTTTCG,CGTTCCAGTG	exonic	MMP26	[illegible]	[illegible]	11p15.4	.
11	5364438	G	A	CTCGAGAAAG,AAAGTGAGTG	exonic	OR51B5	[illegible]	[illegible]	11p15.4	.
11	5536435	C	T	AGCTGGAGAT,ACCCTTTAAT	exonic	UBQLNL	[illegible]	[illegible]	11p15.4	.
11	5629602	G	A	TCCTTCCTAG,ATGTGAGTGA	exonic	TRIM6,TRIM6-TRIM34	[illegible]	[illegible]	11p15.4	.
11	5809785	A	T	AGATTAAACC,CAATATGAAG	exonic	OR52N1	[illegible]	[illegible]	11p15.4	.
11	5969489	C	T	AGAAATTAAG,AGGGAATGCA	exonic	OR56A3	[illegible]	[illegible]	11p15.4	.
11	6292364	C	T	CTGACGGCTC,TGGGCCGGGA	exonic	CCKBR	[illegible]	[illegible]	11p15.4	.
11	7111211	G	A	GAGAGCTACG,AGAGCTGCGC	exonic	RBMXL2	[illegible]	[illegible]	11p15.4	.
11	7334901	C	T	ACTTCAGATC,TTATGTCAAG	exonic	SYT9	[illegible]	[illegible]	11p15.4	.
11	7689769	G	A	TCACTCGTCT,GTCTGGTCTG	exonic	CYB5R2	[illegible]	[illegible]	11p15.4	.
11	10781728	C	T	AGCGGAAGTT,GTTTAGGAAT	exonic	CTR9	[illegible]	[illegible]	11p15.3	.
11	10876166	T	A	TTTCATCATA,TTTCTTCTTT	exonic	ZBED5	[illegible]	[illegible]	11p15.3	.
11	15994594	C	T	GTGGTAGTTG,CATAGTGATA	exonic	SOX6	[illegible]	[illegible]	11p15.2	.
11	17527396	G	A	AGATTTCACA,CTGGCCTGTA	exonic	USH1C	[illegible]	[illegible]	11p15.1	.
11	18048153	G	A	CCACAGGACG,ATGGAAAAAC	exonic	TPH1	[illegible]	[illegible]	11p15.1	.
11	18267031	C	T	GCACCACGGC,TGTGAGTCTC	exonic	SAA2	[illegible]	[illegible]	11p15.1	.
11	18309206	G	A	ATTAAGGATC,AAGAGCAGAC	exonic	HPS5	[illegible]	[illegible]	11p15.1	.
11	22363126	G	A	GACGGAGGAT,GGAAGCCCCT	exonic	SLC17A6	[illegible]	[illegible]	11p14.3	.
11	27528286	C	T	CACGGGTTCC,CTAGCGCCGC	exonic	LIN7C	[illegible]	[illegible]	11p14.1	.
11	33631370	G	A	CAAGAGGAAG,GATATTACGA	exonic	KIAA1549L	[illegible]	[illegible]	11p13	C11orf41_ENST00000321505;basechange=c.4246G>T;AAchange=p.G1416*;tissue=urinary_tract
11	33631371	G	A	AAGAGGAAGG,ATATTACGAC	exonic	KIAA1549L	[illegible]	[illegible]	11p13	.
11	34112131	C	T	CTTCCTGCTG,GTCTCAGCCT	exonic	CAPRIN1	[illegible]	[illegible]	11p13	.
11	35313868	G	A	ATCCAAGCTT,GAAAATGCCA	exonic	SLC1A2	[illegible]	[illegible]	11p13	.
11	43436207	C	T	GCACTTGAGG,CTTTAGACAG	exonic	TTC17	[illegible]	[illegible]	11p12	.
11	45274136	G	A	TCCGCCAGGG,GAGCACCAGG	exonic	SYT13	[illegible]	[illegible]	11p11.2	SYT13;basechange=c.682C>A;AAchange=p.P228T;tissue=lung
11	45891243	C	T	GACAGGCTTC,CTTGGATTGA	exonic	CRY2	[illegible]	[illegible]	11p11.2	.
11	46678698	C	T	CACCATCACT,TTTCTTGTGC	exonic	ATG13	[illegible]	[illegible]	11p11.2	.
11	46678699	T	A	ACCATCACTC,TTCTTGTGCT	exonic	ATG13	[illegible]	[illegible]	11p11.2	.
11	46832559	G	A	TGTCTCACCT,AACAGAGTTT	exonic	CKAP5	[illegible]	[illegible]	11p11.2	.
11	55563809	C	T	CATCCTTTTC,TTTACTGTGT	exonic	OR5D14	[illegible]	[illegible]	11q11	.
11	55588024	G	A	CGAGATACTG,ACACCAAAGT	exonic	OR5D18	[illegible]	[illegible]	11q11	.
11	56510593	G	A	GTTGACATAG,AAATCAGGAT	exonic	OR9G4	[illegible]	[illegible]	11q12.1	.
11	58035012	C	T	GCCATGGCAG,CAAGAGGAAG	exonic	OR10W1	[illegible]	[illegible]	11q12.1	.
11	58893131	G	A	CACTTATACA,AGTTCTGCCC	exonic	FAM111B	[illegible]	[illegible]	11q12.1	.
11	60150732	G	C	CCTGCAGAAC,GGCTGCCAAC	exonic	MS4A7	[illegible]	[illegible]	11q12.2	MS4A7;basechange=c.118G>A;AAchange=p.G40R;tissue=large_intestine
11	60154223	G	A	AATTATCTCT,GAAAACAATC	exonic	MS4A7	[illegible]	[illegible]	11q12.2	.
11	60184114	A	G	AATCAAACTA,AGAGCAACTC	exonic	MS4A14	[illegible]	[illegible]	11q12.2	.
11	60709581	G	A	TTTGGGATGT,GAGGTGAAGA	exonic	SLC15A3	[illegible]	[illegible]	11q12.2	.
11	60774107	G	A	GTGAGCTCTG,GAGCCAGGTG	exonic	CD6	[illegible]	[illegible]	11q12.2	.
11	60774108	G	A	TGAGCTCTGG,AGCCAGGTGA	exonic	CD6	[illegible]	[illegible]	11q12.2	.
11	62782337	C	T	TTGTGGTTGG,CATGTTGAGG	exonic	SLC22A8	[illegible]	[illegible]	11q12.3	.
11	63143205	G	A	ACACAGGAGT,GAATGAAGAA	exonic	SLC22A9	[illegible]	[illegible]	11q12.3	.
11	63283123	C	T	CTGGATCTCC,GCTGGGGGCA	exonic	LGALS12	[illegible]	[illegible]	11q12.3	.
11	64067082	G	A	TACGCAGAGC,AGCGCGGCTG	exonic	KCNK4	[illegible]	[illegible]	11q13.1	.
11	64480619	C	T	GGCCGCTCGC,CAGCTTCAGG	exonic	NRXN2	[illegible]	[illegible]	11q13.1	.
11	64787912	C	T	AGCAACCCTC,TCATCTTTGC	exonic	ARL2	[illegible]	[illegible]	11q13.1	.
11	65985059	C	T	GATCACTCTC,CATCACAATG	exonic	PACS1	[illegible]	[illegible]	11q13.2	.
11	66512350	C	T	TGCCGCTGTT,CCTGCGCGGC	exonic	C11orf80	[illegible]	[illegible]	11q13.2	.
11	67223124	G	A	CCCAGCACTG,AGAGGGGCCG	exonic	CABP4	[illegible]	[illegible]	11q13.2	.
11	67833950	G	A	CTGTTTTTCA,AATTCTCTCG	exonic	CHKA	[illegible]	[illegible]	11q13.2	.
11	68181343	C	A	GTGTTCCACT,CTCCCGCCAG	exonic	LRP5	[illegible]	[illegible]	11q13.2	.
11	72437681	G	A	AGCAGGCGGG,GGGTCCGGTG	exonic	ARAP1	[illegible]	[illegible]	11q13.4	.
11	73676073	C	T	ATTAAGATCT,CAGAAGGGTG	exonic	DNAJB13	[illegible]	[illegible]	11q13.4	.
11	73717321	G	A	CCCATTGTAG,GGCTGCAGGG	exonic	UCP3	[illegible]	[illegible]	11q13.4	.
11	77921059	G	A	AGAGAAGGTG,AGAAGGAGAC	exonic	USP35	[illegible]	[illegible]	11q14.1	.
11	83641473	G	A	GTGCCTGGGA,AAGCAGGCTT	exonic	DLG2	[illegible]	[illegible]	11q14.1	.
11	84245725	C	T	GTGGGTTAGT,GAGGTAAGGA	exonic	DLG2	[illegible]	[illegible]	11q14.1	DLG2_ENST00000376104;basechange=c.407G>A;AAchange=p.R136Q;tissue=large_intestine&DLG2;basechange=c.92G>A;AAchange=
11	92085636	G	A	ATTAAATAGG,AAATCCAGGA	exonic	FAT3	[illegible]	[illegible]	11q14.3	.
11	102197524	C	T	CTGCAACCTC,GCCTCTTGGG	exonic	BIRC3	[illegible]	[illegible]	11q22.2	.
11	102248611	C	T	GAAGTATATT,CAACAGAAGA	exonic	BIRC2	[illegible]	[illegible]	11q22.2	.
11	103818449	C	T	GGTTGGAAAT,TTCCTGGAAG	exonic	PDGFD	[illegible]	[illegible]	11q22.3	.
11	104758012	C	T	CCATTTGTTT,ATGTCTCCAA	exonic	CASP12	[illegible]	[illegible]	11q22.3	.
11	107920770	G	T	AAAATCAAAT,TGGAAGACAG	exonic	CUL5	[illegible]	[illegible]	11q22.3	.
11	111583004	C	T	TGCCCTCCTC,CCCAGGCATC	exonic	SIK2	[illegible]	[illegible]	11q23.1	.
11	111746497	G	A	ATCTTGGCCT,GCCACAGGTT	exonic	FDXACB1	[illegible]	[illegible]	11q23.1	.
11	113856861	G	A	AGTTCAGCAT,GAAAGCAGTA	exonic	HTR3A	[illegible]	[illegible]	11q23.2	.
11	114167411	C	T	TCTGAAAAAT,TTTTCAAGAT	exonic	NNMT	[illegible]	[illegible]	11q23.2	NNMT;basechange=c.133C>A;AAchange=p.L45I;tissue=endometrium
11	116728580	G	A	TGACCATAGG,ATGTATCCCC	exonic	SIK3	[illegible]	[illegible]	11q23.3	.
11	118944071	C	T	TGGGCGGGTC,ACGCACTGCA	exonic	VPS11	[illegible]	[illegible]	11q23.3	.
11	121039591	C	T	CACACCCACC,GAGATAGCAA	exonic	TECTA	[illegible]	[illegible]	11q23.3	.
11	121490478	C	T	GTAGTGGTAC,CTACCAGGGG	exonic	SORL1	[illegible]	[illegible]	11q24.1	.
11	122647790	C	T	GGAGTACGTC,TCTACCTCCG	exonic	UBASH3B	[illegible]	[illegible]	11q24.1	.
11	123811110	C	T	AAGGCCTTTT,GGACATTCCC	exonic	OR4D5	[illegible]	[illegible]	11q24.1	OR4D5;basechange=c.787C>T;AAchange=p.R263W;tissue=liver
11	124619634	G	A	GAAGGTGTAG,AAAAGTTCCA	exonic	VSIG2	[illegible]	[illegible]	11q24.2	.
11	124747497	G	A	CTCGGCCCAG,AACCCAGGGG	exonic	ROBO3	[illegible]	[illegible]	11q24.2	.
11	124765523	G	A	GCCTCCCGGG,CAGTCTGGGT	exonic	ROBO4	[illegible]	[illegible]	11q24.2	.
11	128332452	T	C	GTTTTTCCTC,TTCCCCATCT	exonic	ETS1	[illegible]	[illegible]	11q24.3	.
11	128677081	C	T	ACAGGTCCTC,CCTTGGAGGG	exonic	FLI1	[illegible]	[illegible]	11q24.3	.
11	129788520	T	G	ATGCGGGGCT,GAACGTCTTG	exonic	PRDM10	[illegible]	[illegible]	11q24.3	.
11	130011818	C	T	ACGTAGGAAT,CGTGGGCCCA	exonic	APLP2	[illegible]	[illegible]	11q24.3	APLP2;basechange=c.2039C>T;AAchange=p.S680F;tissue=skin
11	130297788	C	T	ATGGTGAACT,CTCGCCGTCC	exonic	ADAMTS8	[illegible]	[illegible]	11q24.3	.
11	130332559	G	A	GAAGGCCAAG,GACAGATGGT	exonic	ADAMTS15	[illegible]	[illegible]	11q24.3	.
11	130784219	A	T	TCGGGCTGTA,TGGTGCCAGT	exonic	SNX19	[illegible]	[illegible]	11q24.3	.
11	133714162	C	T	GAACTGGATT,GTGTTCTCTC	exonic	SPATA19	[illegible]	[illegible]	11q25	.
12	300254	C	T	GTCTCCTTCT,CCCGGCTATC	exonic	SLC6A12	[illegible]	[illegible]	12p13.33	.
12	402017	G	A	TACTTTGAGG,GATGTCCAGC	exonic	KDM5A	[illegible]	[illegible]	12p13.33	.
12	1995190	G	A	TCGATGTAAT,GACGTAGTCA	exonic	CACNA2D4	[illegible]	[illegible]	12p13.33	.
12	2774004	G	A	TGCCACCGGG,AGGCCTGGCA	exonic	CACNA1C	[illegible]	[illegible]	12p13.33	.
12	4698143	C	T	AAGCCACCTT,CAATATCAAG	exonic	DYRK4	[illegible]	[illegible]	12p13.32	.
12	5153407	G	T	GGCCACAGGG,GAGAGCTCCA	exonic	KCNA5	[illegible]	[illegible]	12p13.32	.
12	5153408	G	A	GCCACAGGGG,AGAGCTCCAG	exonic	KCNA5	[illegible]	[illegible]	12p13.32	.
12	5154361	G	A	[illegible]	exonic	KCNA5	[illegible]	[illegible]	12p13.32	.

12	6840220	C	T	ACTGTCGTTT,CTCCCTGGGG	exonic	COPS7A	nonsynonymous SNV	COPS7A:ENST00000538410.1:exon7:c.C691T:p.P231S,	12p13.31	.
12	7551037	C	T	TTTCCACATC,CAATTGTTTA	exonic	CD163L1	nonsynonymous SNV	CD163L1:ENST00000313599.3:exon7:c.G1552A:p.G518R,CD163L1:ENST00000396630.1:exon7:c.G1552A:p.G518R,CD163L1:ENST00000416109.2:exon7:c.G1582A:p.G528R,	12p13.31	.
12	7653981	C	T	GTTCCCCACT,CTCCTGGACT	exonic	CD163	nonsynonymous SNV	CD163:ENST00000541972.1:exon3:c.G175A:p.E59K,CD163:ENST00000396620.3:exon3:c.G211A:p.E71K,CD163:ENST00000359156.4:exon3:c.G211A:p.E71K,CD163:ENST00000432237.2:exon3:c.G211A:p.E71K,	12p13.31	.
12	7864239	G	A	AAATTCCCGG,ACGATTCAGG	exonic	DPPA3	nonsynonymous SNV	DPPA3:ENST00000345088.2:exon1:c.G73A:p.D25N,	12p13.31	.
12	8628749	C	T	CTGAATGAGT,ATTTTCTTAT	exonic	CLEC6A	nonsynonymous SNV	CLEC6A:ENST00000382073.3:exon5:c.C398T:p.S133L,	12p13.31	.
12	9001352	C	T	TGGTCACTAC,CCTATCAAGT	exonic	A2ML1	nonsynonymous SNV	A2ML1:ENST00000539547.1:exon5:c.C397T:p.P133S,A2ML1:ENST00000299698.7:exon16:c.C1870T:p.P624S,A2ML1:ENST00000541459.1:exon5:c.C520T:p.P174S,A2ML1:ENST00000545692.1:exon5:c.C406T:p.P136S,	12p13.31	.
12	9354971	G	A	AATCTTACTG,AAAAGTAGTT	exonic	PZP	nonsynonymous SNV	PZP:ENST00000381997.2:exon1:c.C31T:p.P11S,	12p13.31	.
12	10233983	C	T	GTGTCTTGAC,AGTATTGGAG	exonic	CLEC1A	nonsynonymous SNV	CLEC1A:ENST00000457018.2:exon2:c.G145A:p.G49S,CLEC1A:ENST00000315330.4:exon3:c.G244A:p.G82S,	12p13.2	.
12	11420194	G	A	GGTGGTCCCT,GGGCTTTCCA	exonic	PRB3	stopgain	PRB3:ENST00000381842.3:exon5:c.C862T:p.Q288X,PRB3:ENST00000538488.1:exon6:c.C862T:p.Q288X,	12p13.2	.
12	11461790	C	T	TGGTTTCCTC,TTGTGGGCGT	exonic	PRB4	nonsynonymous SNV	PRB4:ENST00000445719.2:exon3:c.G127A:p.G43R,PRB4:ENST00000279575.1:exon3:c.G127A:p.G43R,PRB4:ENST00000535904.1:exon3:c.G127A:p.G43R,	12p13.2	.
12	11506727	C	T	TGGTTACCTC,TTGTGGGGGT	exonic	PRB1	nonsynonymous SNV	PRB1:ENST00000545626.1:exon3:c.G310A:p.G104R,PRB1:ENST00000546254.1:exon3:c.G310A:p.G104R,PRB1:ENST00000500254.2:exon3:c.G310A:p.G104R,	12p13.2	.
12	11546300	G	A	TTTCCTGGAG,AGATCGGGCA	exonic	PRB2	nonsynonymous SNV	PRB2:ENST00000389362.4:exon3:c.C712T:p.P238S,	12p13.2	.
12	11546639	C	T	TGGTTGCCTC,TTGTGGGGGT	exonic	PRB2	nonsynonymous SNV	PRB2:ENST00000389362.4:exon3:c.G373A:p.G125R,	12p13.2	.
12	12312887	C	T	CCATTCAGTC,AATACATAAA	exonic	LRP6	stopgain	LRP6:ENST00000261349.4:exon11:c.G2291A:p.W764X,LRP6:ENST00000543091.1:exon11:c.G2291A:p.W764X,	12p13.2	.
12	12337036	G	A	AATTCCACAT,GATTTGTGGC	exonic	LRP6	nonsynonymous SNV	LRP6:ENST00000261349.4:exon5:c.C854T:p.P285L,LRP6:ENST00000543091.1:exon5:c.C854T:p.P285L,	12p13.2	.
12	12356138	G	A	TAATCTTACC,ATTTGTTCCA	exonic	LRP6	nonsynonymous SNV	LRP6:ENST00000261349.4:exon3:c.C646T:p.R216W,LRP6:ENST00000535731.1:exon2:c.C193T:p.R65W,LRP6:ENST00000543091.1:exon3:c.C646T:p.R216W,	12p13.2	LRP6;basechange=c.646C>T;AAchange=p.R216W;tissue=large_intestine
12	13102607	C	T	ACCGGGTCGT,CCACTGGGGC	exonic	GPRC5D	nonsynonymous SNV	GPRC5D:ENST00000396333.3:exon1:c.G712A:p.D238N,GPRC5D:ENST00000228887.1:exon1:c.G712A:p.D238N,	12p13.1	.
12	13716799	C	T	CCTTCCTTGT,CCTGAAGTAG	exonic	GRIN2B	nonsynonymous SNV	GRIN2B:ENST00000609686.1:exon13:c.G3373A:p.D1125N,	12p13.1	.
12	13717090	G	A	TTGGAGGAGG,GAGGCCGATG	exonic	GRIN2B	nonsynonymous SNV	GRIN2B:ENST00000609686.1:exon13:c.C3082T:p.P1028S,	12p13.1	.
12	13768059	G	A	TAAGAAGGCA,AAGGTGAGAC	exonic	GRIN2B	nonsynonymous SNV	GRIN2B:ENST00000609686.1:exon7:c.C1643T:p.S548F,	12p13.1	.
12	16410641	C	T	TTACGTTTTT,TGACTGATAA	exonic	SLC15A5	nonsynonymous SNV	SLC15A5:ENST00000344941.3:exon3:c.G748A:p.E250K,	12p12.3	.
12	21176141	G	A	AATGTAATGG,AATGACTGGT	exonic	LST3,SLCO1B7	nonsynonymous SNV	LST3:ENST00000381541.3:exon6:c.G647A:p.G216E,SLCO1B7:ENST00000421593.2:exon5:c.G506A:p.G169E,SLCO1B7:ENST00000554957.1:exon6:c.G647A:p.G216E,	12p12.2	.
12	26220623	C	T	GAAATAGAGG,CTCACATGCA	exonic	RASSF8	nonsynonymous SNV	RASSF8:ENST00000541490.1:exon4:c.C1115T:p.A372V,RASSF8:ENST00000539545.1:exon2:c.C482T:p.A161V,RASSF8:ENST00000381352.3:exon5:c.C1115T:p.A372V,RASSF8:ENST00000405154.2:exon4:c.C1115T:p.A372V,RASSF8:ENST00000542865.1:exon5:c.C1115T:p.A372V,RASSF8:ENST000002	12p12.1	.
12	26596540	C	T	TGGATCCGAT,CTGGTTTGAG	exonic	ITPR2	nonsynonymous SNV	ITPR2:ENST00000381340.3:exon46:c.G6386A:p.G2129E,	12p11.23	.
12	27523133	G	A	CTTTCAACAA,GATAGAAATA	exonic	ARNTL2	nonsynonymous SNV	ARNTL2:ENST00000266503.5:exon3:c.G254A:p.R85K,ARNTL2:ENST00000311001.5:exon3:c.G254A:p.R85K,	12p11.23	.
12	27832527	C	T	CCTCCATCTC,AGATTCCAAA	exonic	PPFIBP1	nonsynonymous SNV	PPFIBP1:ENST00000318304.8:exon19:c.C1739T:p.P580L,PPFIBP1:ENST00000542629.1:exon18:c.C1646T:p.P549L,PPFIBP1:ENST00000228425.6:exon20:c.C1721T:p.P574L,PPFIBP1:ENST00000540114.1:exon16:c.C1232T:p.P411L,PPFIBP1:ENST00000537927.1:exon17:c.C1280T:p.P427L,	12p11.22	.
12	29630316	C	T	AGCTCAAAGC,ACTGCCACTG	exonic	OVCH1	nonsynonymous SNV	OVCH1:ENST00000318184.5:exon11:c.G1204A:p.G402S,	12p11.22	.
12	30869480	G	A	TACATGTATA,ATGGCAGTTG	exonic	CAPRIN2	nonsynonymous SNV	CAPRIN2:ENST00000417045.1:exon14:c.C2426T:p.S809F,CAPRIN2:ENST00000308433.5:exon13:c.C1427T:p.S476F,CAPRIN2:ENST00000251071.5:exon14:c.C2426T:p.S809F,CAPRIN2:ENST00000433722.2:exon11:c.C1664T:p.S555F,CAPRIN2:ENST00000395805.2:exon13:c.C2321T:p.S774F,CAPRIN2:EN	12p11.21	.
12	31136069	G	A	GTGGGCATCG,GGTGGCCTGC	exonic	TSPAN11	nonsynonymous SNV	TSPAN11:ENST00000546076.1:exon7:c.G686A:p.G229E,TSPAN11:ENST00000535215.1:exon6:c.G473A:p.G158E,TSPAN11:ENST00000261177.9:exon7:c.G686A:p.G229E,TSPAN11:ENST00000544427.1:exon6:c.G656A:p.G219E,	12p11.21	.
12	33559894	G	A	TATGCTACAG,AAATTGAAAA	exonic	SYT10	nonsynonymous SNV	SYT10:ENST00000535526.1:exon5:c.C364T:p.P122S,SYT10:ENST00000228567.3:exon3:c.C907T:p.P303S,	12p11.1	.
12	40626172	C	T	TTGGGAAGTC,TTGGTGTTCA	exonic	LRRK2	nonsynonymous SNV	LRRK2:ENST00000416796.1:exon3:c.C121T:p.L41F,LRRK2:ENST00000298910.7:exon3:c.C334T:p.L112F,LRRK2:ENST00000343742.2:exon3:c.C334T:p.L112F,	12q12	.
12	40866955	G	A	TCAACAGAAG,GTCTGTGGAA	exonic	MUC19	nonsynonymous SNV	MUC19:ENST00000454784.4:exon45:c.G3962A:p.G1321E,	12q12	.
12	40873169	G	A	ATCAGCTGAA,GATCAGGGAC	exonic	MUC19	nonsynonymous SNV	MUC19:ENST00000454784.4:exon49:c.G4714A:p.G1572R,	12q12	.
12	40873170	G	A	TCAGCTGAAG,ATCAGGGACA	exonic	MUC19	nonsynonymous SNV	MUC19:ENST00000454784.4:exon49:c.G4715A:p.G1572E,	12q12	.
12	40874640	G	A	GTCACAACTG,ACCACCTCTT	exonic	MUC19	nonsynonymous SNV	MUC19:ENST00000454784.4:exon49:c.G6185A:p.G2062E,	12q12	.
12	40878966	T	C	TCAACTGAAG,GACAGGAACA	exonic	MUC19	nonsynonymous SNV	MUC19:ENST00000454784.4:exon49:c.T10511C:p.V3504A,	12q12	.
12	43821236	C	T	CAGACCACAG,CCTATGCTGA	exonic	ADAMTS20	nonsynonymous SNV	ADAMTS20:ENST00000549670.1:exon9:c.G1372A:p.A458T,ADAMTS20:ENST00000553158.1:exon27:c.G3982A:p.A1328T,ADAMTS20:ENST00000389420.3:exon27:c.G3982A:p.A1328T,ADAMTS20:ENST00000395541.2:exon9:c.G1336A:p.A446T,	12q12	.
12	43860511	G	T	AAGGACTCAT,TGAAAACTTA	exonic	ADAMTS20	nonsynonymous SNV	ADAMTS20:ENST00000553158.1:exon9:c.C1311A:p.H437Q,ADAMTS20:ENST00000389420.3:exon9:c.C1311A:p.H437Q,	12q12	.
12	46245204	C	T	GGTGGTCTAT,AGGTGGCCAG	exonic	ARID2	stopgain	ARID2:ENST00000334344.6:exon15:c.C3298T:p.Q1100X,ARID2:ENST00000422737.1:exon15:c.C2851T:p.Q951X,ARID2:ENST00000444670.1:exon7:c.C2128T:p.Q710X,	12q12	ARID2;basechange=c.3298C>G;AAchange=p.Q1100E;tissue=lung
12	48189109	T	A	AGAGAGCCGC,CGGTGGTCAT	exonic	HDAC7	nonsynonymous SNV	HDAC7:ENST00000354334.3:exon10:c.A1031T:p.E344V,HDAC7:ENST00000380610.4:exon11:c.A1193T:p.E398V,HDAC7:ENST00000080059.7:exon11:c.A1142T:p.E381V,HDAC7:ENST00000552960.1:exon10:c.A1091T:p.E364V,HDAC7:ENST00000427332.2:exon11:c.A1025T:p.E342V,	12q13.11	.
12	48501854	G	A	TTGTTTCTCA,CTGGGGAAGC	splicing	PFKM(ENST00000550257.1:exon2:c.92-1G>A,[illegible])	.	.	12q13.11	.
12	49086827	G	A	TACTTAGGAA,GGGTGGAAGT	exonic	CCNT1	nonsynonymous SNV	CCNT1:ENST00000261900.3:exon9:c.C2170T:p.L724F,	12q13.11	.
12	49165654	C	T	TTGCGGCGCT,CCGGGCCAGG	exonic	ADCY6	nonsynonymous SNV	ADCY6:ENST00000357869.3:exon17:c.G2731A:p.E911K,ADCY6:ENST00000550422.1:exon18:c.G2731A:p.E911K,ADCY6:ENST00000307885.4:exon18:c.G2890A:p.E964K,	12q13.12	.
12	49434136	G	A	TCAGGGGGCT,GGGTCGGGGT	exonic	KMT2D	stopgain	KMT2D:ENST00000301067.7:exon31:c.C7417T:p.Q2473X,	12q13.12	.
12	49434960	G	A	ACTAGGATAG,GGGGATAGGT	exonic	KMT2D	nonsynonymous SNV	KMT2D:ENST00000301067.7:exon31:c.C6593T:p.P2198L,	12q13.12	.
12	49719875	C	T	CCTTCAGGAC,TTCCTTTCAC	exonic	TROAP	nonsynonymous SNV	TROAP:ENST00000257909.3:exon6:c.C650T:p.P217L,TROAP:ENST00000547807.1:exon6:c.C650T:p.P217L,TROAP:ENST00000551245.1:exon6:c.C650T:p.P217L,TROAP:ENST00000550346.1:exon4:c.C299T:p.P100L,	12q13.12	.
12	50047080	C	T	ATCATGTTTT,ATTCTCTAGG	exonic	FMNL3	nonsynonymous SNV	FMNL3:ENST00000352151.5:exon12:c.G1099A:p.E367K,FMNL3:ENST00000335154.5:exon13:c.G1252A:p.E418K,FMNL3:ENST00000550488.1:exon13:c.G1252A:p.E418K,FMNL3:ENST00000293590.5:exon13:c.G1252A:p.E418K,	12q13.12	.
12	50484348	C	T	GCCACCAGAA,CTATCATCAT	exonic	SMARCD1	nonsynonymous SNV	SMARCD1:ENST00000381513.4:exon9:c.C1108T:p.P370S,SMARCD1:ENST00000394963.4:exon9:c.C1108T:p.P370S,SMARCD1:ENST00000548573.1:exon5:c.C502T:p.P168S,	12q13.12	.
12	51065110	C	T	TCCGCATCTT,TACGCTGTCC	exonic	DIP2B	nonsynonymous SNV	DIP2B:ENST00000301180.5:exon5:c.C569T:p.S190F,	12q13.12	.
12	51504761	G	A	ACAGACATCG,GATATCTGAG	exonic	TFCP2	nonsynonymous SNV	TFCP2:ENST00000549867.1:exon6:c.C463T:p.P155S,TFCP2:ENST00000548108.1:exon5:c.C169T:p.P57S,TFCP2:ENST00000307660.4:exon5:c.C463T:p.P155S,TFCP2:ENST00000257915.5:exon5:c.C463T:p.P155S,TFCP2:ENST00000548115.1:exon5:c.C463T:p.P155S,	12q13.13	.
12	52180356	C	T	GGGCGCCATC,CCTCCATCAT	exonic	SCN8A	nonsynonymous SNV	SCN8A:ENST00000354534.6:exon22:c.C3973T:p.P1325S,SCN8A:ENST00000355133.3:exon20:c.C3850T:p.P1284S,SCN8A:ENST00000545061.1:exon20:c.C3850T:p.P1284S,	12q13.13	.
12	52636887	G	A	GCCATCCAGA,GCTGCAGGCT	exonic	KRT7	nonsynonymous SNV	KRT7:ENST00000331817.5:exon6:c.G950A:p.R317K,	12q13.13	.
12	52681806	G	A	GCCCGGCTGC,GGTGACAATG	exonic	KRT81	nonsynonymous SNV	KRT81:ENST00000327741.5:exon5:c.C862T:p.R288C,	12q13.13	.
12	52911445	C	T	GCCTTGACCT,AGCGATGATG	exonic	KRT5	nonsynonymous SNV	KRT5:ENST00000252242.4:exon5:c.G1021A:p.E341K,	12q13.13	.
12	52946833	C	T	GGCGGCAGCT,CCGACTTGCA	exonic	KRT71	nonsynonymous SNV	KRT71:ENST00000267119.5:exon1:c.G29A:p.G10E,	12q13.13	.
12	52946834	C	T	GCGGCAGCTC,CGACTTGCAG	exonic	KRT71	nonsynonymous SNV	KRT71:ENST00000267119.5:exon1:c.G28A:p.G10R,	12q13.13	.
12	55357556	C	T	TCTTCCATTT,AATCCTCCGC	exonic	TESPA1	nonsynonymous SNV	TESPA1:ENST00000449076.1:exon8:c.G625A:p.E209K,TESPA1:ENST00000526532.1:exon6:c.G211A:p.E71K,TESPA1:ENST00000524622.1:exon6:c.G211A:p.E71K,TESPA1:ENST00000532757.1:exon6:c.G211A:p.E71K,TESPA1:ENST00000316577.8:exon8:c.G625A:p.E209K,TESPA1:ENST00000531122.1:e	12q13.2	.
12	56495414	C	T	ATACATGAAC,GGAGGAGAAG	exonic	ERBB3	nonsynonymous SNV	ERBB3:ENST00000553131.1:exon11:c.C1327T:p.R443W,ERBB3:ENST00000550070.2:exon13:c.C1525T:p.R509W,ERBB3:ENST00000549832.1:exon6:c.C964T:p.R322W,ERBB3:ENST00000450146.2:exon15:c.C1675T:p.R559W,ERBB3:ENST00000267101.3:exon28:c.C3604T:p.R1202W,ERBB3:ENST000004	12q13.2	.
12	56625278	C	T	TCAGGGGCTT,GCCTGGGACA	exonic	SLC39A5	nonsynonymous SNV	SLC39A5:ENST00000419753.1:exon2:c.C220T:p.R74C,SLC39A5:ENST00000417965.1:exon3:c.C220T:p.R74C,SLC39A5:ENST00000424625.1:exon3:c.C220T:p.R74C,SLC39A5:ENST00000454355.2:exon4:c.C220T:p.R74C,SLC39A5:ENST00000266980.4:exon2:c.C220T:p.R74C,SLC39A5:ENST00000436633	12q13.3	SLC39A5;basechange=c.220C>T;AAchange=p.R74C;tissue=endometrium
12	57110912	G	A	GTCACTGCTG,GAGGGTGGGA	exonic	NACA	nonsynonymous SNV	NACA:ENST00000454682.1:exon3:c.C4402T:p.P1468S,	12q13.3	.
12	57849976	C	T	GTGCTCCCCA,CCTTCCTGGC	exonic	INHBE	nonsynonymous SNV	INHBE:ENST00000266646.2:exon2:c.C398T:p.T133I,INHBE:ENST00000547970.1:exon3:c.C233T:p.T78I,	12q13.3	.
12	57849978	C	T	GCTCCCCACC,TTCCTGGCAC	exonic	INHBE	nonsynonymous SNV	INHBE:ENST00000266646.2:exon2:c.C400T:p.L134F,INHBE:ENST00000547970.1:exon3:c.C235T:p.L79F,	12q13.3	.
12	57919871	C	T	TACTGTATTT,GATTGCTAGA	exonic	MBD6	stopgain	MBD6:ENST00000355673.3:exon6:c.C1120T:p.R374X,MBD6:ENST00000431731.2:exon6:c.C1120T:p.R374X,	12q13.3	.
12	57963904	G	A	GCAGAAATAC,AGGAGGAGAT	exonic	KIF5A	nonsynonymous SNV	KIF5A:ENST00000286452.5:exon9:c.G985A:p.E329K,KIF5A:ENST00000455537.2:exon12:c.G1252A:p.E418K,	12q13.3	.
12	58157552	G	A	AACTGGGCAG,GTCCCTTGAA	exonic	CYP27B1	nonsynonymous SNV	CYP27B1:ENST00000228606.4:exon8:c.C1255T:p.P419S,	12q14.1	.
12	70149201	C	T	GGCTAATGAT,CCTTGGAAGG	exonic	RAB3IP	nonsynonymous SNV	RAB3IP:ENST00000483530.2:exon2:c.C13T:p.P5S,RAB3IP:ENST00000550536.1:exon2:c.C61T:p.P21S,RAB3IP:ENST00000362025.5:exon2:c.C61T:p.P21S,RAB3IP:ENST00000247833.7:exon2:c.C13T:p.P5S,RAB3IP:ENST00000378815.6:exon2:c.C13T:p.P5S,RAB3IP:ENST00000549760.1:exon2:c.C13T:p.P5	12q15	.
12	70149202	C	T	GCTAATGATC,CTTGGAAGGC	exonic	RAB3IP	nonsynonymous SNV	RAB3IP:ENST00000483530.2:exon2:c.C14T:p.P5L,RAB3IP:ENST00000550536.1:exon2:c.C62T:p.P21L,RAB3IP:ENST00000362025.5:exon2:c.C62T:p.P21L,RAB3IP:ENST00000247833.7:exon2:c.C14T:p.P5L,RAB3IP:ENST00000378815.6:exon2:c.C14T:p.P5L,RAB3IP:ENST00000549760.1:exon2:c.C14T:p.P5L	12q15	.
12	72026153	G	A	TTAAGGGCAC,GGCTTCTTTT	exonic	ZFC3H1	nonsynonymous SNV	ZFC3H1:ENST00000378743.3:exon15:c.C2959T:p.R987C,ZFC3H1:ENST00000552994.1:exon15:c.C2959T:p.R987C,	12q21.1	.
12	72338426	G	A	TGCTCTCACA,AGTTCTCATG	exonic	TPH2	nonsynonymous SNV	TPH2:ENST00000333850.3:exon4:c.G500A:p.R167K,	12q21.1	.
12	75737587	G	A	TGAATATGTT,GAGAAAATAT	exonic	GLIPR1L1	nonsynonymous SNV	GLIPR1L1:ENST00000378695.4:exon2:c.G289A:p.G97R,GLIPR1L1:ENST00000312442.2:exon2:c.G289A:p.G97R,	12q21.2	.
12	81472017	C	T	TTTAGTGGTC,CGGGCCCGCG	exonic	ACSS3	nonsynonymous SNV	ACSS3:ENST00000548058.1:exon1:c.C118T:p.P40S,ACSS3:ENST00000261206.3:exon1:c.C118T:p.P40S,	12q21.31	.
12	85623422	G	A	CCAGAAATAT,AAATGGTGAG	exonic	LRRIQ1	nonsynonymous SNV	LRRIQ1:ENST00000393217.2:exon25:c.G4950A:p.M1650I,	12q21.31	.
12	94634297	G	A	ACACCCACAT,AAATTCTCTC	exonic	PLXNC1	nonsynonymous SNV	PLXNC1:ENST00000258526.4:exon11:c.G2157A:p.M719I,	12q22	.
12	99223005	C	T	AAGTAGTTTT,ATTTCTTCTC	exonic	ANKS1B	nonsynonymous SNV	ANKS1B:ENST00000548447.1:exon3:c.G31A:p.E11K,ANKS1B:ENST00000546960.1:exon5:c.G691A:p.E231K,ANKS1B:ENST00000552407.1:exon3:c.G31A:p.E11K,ANKS1B:ENST00000552748.1:exon3:c.G31A:p.E11K,ANKS1B:ENST00000546364.3:exon4:c.G31A:p.E11K,ANKS1B:ENST00000341752.7:exon3	12q23.1	.
12	99640529	G	A	CCATAGAGAT,AAATGGATTT	exonic	ANKS1B	nonsynonymous SNV	ANKS1B:ENST00000547010.1:exon6:c.C610T:p.H204Y,ANKS1B:ENST00000329257.7:exon13:c.C1870T:p.H624Y,ANKS1B:ENST00000547776.2:exon13:c.C1870T:p.H624Y,	12q23.1	.
12	100732346	C	T	AATATGTCAT,CTTGACCAGC	exonic	SCYL2	nonsynonymous SNV	SCYL2:ENST00000360820.2:exon18:c.C2186T:p.S729F,	12q23.1	.
12	101560374	A	G	TTGGATATCA,GGTGCAATGG	exonic	SLC5A8	nonsynonymous SNV	SLC5A8:ENST00000536262.2:exon12:c.T1424C:p.L475P,	12q23.1	.
12	103984756	C	T	AGTCAAGTGC,CGGATGGTTA	exonic	STAB2	nonsynonymous SNV	STAB2:ENST00000388887.2:exon2:c.C163T:p.P55S,	12q23.3	.
12	104015819	G	A	GTGTTTTGCA,GAAGGGTTTG	splicing	STAB2(ENST00000388887.2:exon5:c.418-1G>A)	.	.	12q23.3	.
12	104098337	G	A	TTTACCAAGG,AAGATGTAGG	exonic	STAB2	nonsynonymous SNV	STAB2:ENST00000388887.2:exon36:c.G3845A:p.G1282E,STAB2:ENST00000549474.1:exon2:c.G44A:p.G15E,	12q23.3	.
12	104151948	G	A	TTCTCCCCAG,AAGTGCTGGC	exonic	STAB2	nonsynonymous SNV	STAB2:ENST00000388887.2:exon64:c.G6988A:p.E2330K,	12q23.3	.
12	107048076	G	A	GCATTACCTG,ATTTCTGCGA	exonic	RFX4	nonsynonymous SNV	RFX4:ENST00000536722.1:exon4:c.G289A:p.D97N,RFX4:ENST00000551640.1:exon2:c.G97A:p.D33N,RFX4:ENST00000392842.1:exon4:c.G262A:p.D88N,RFX4:ENST00000549040.1:exon4:c.G13A:p.D5N,RFX4:ENST00000546882.1:exon3:c.G175A:p.D59N,RFX4:ENST00000357881.4:exon4:c.G289A:p.D97N,	12q23.3	.
12	108985604	G	A	GCCCTGGTGG,GGACTTGAGG	exonic	TMEM119	nonsynonymous SNV	TMEM119:ENST00000392806.3:exon2:c.C556T:p.P186S,	12q23.3	.
12	111772337	G	A	CCTGTCCCTG,AGAGCAGCAA	exonic	CUX2	nonsynonymous SNV	CUX2:ENST00000261726.6:exon19:c.G3019A:p.E1007K,	12q24.12	.
12	111856338	C	T	CCGCGGCCGC,CGGGCCCTGC	exonic	SH2B3	nonsynonymous SNV	SH2B3:ENST00000341259.2:exon2:c.C389T:p.P130L,	12q24.12	.
12	113285562	G	A	GAAGCAGGAA,AGCTGACTGA	exonic	RPH3A	nonsynonymous SNV	RPH3A:ENST00000547728.1:exon5:c.G145A:p.E49K,RPH3A:ENST00000551052.1:exon4:c.G133A:p.E45K,RPH3A:ENST00000420983.2:exon3:c.G145A:p.E49K,RPH3A:ENST00000552667.1:exon5:c.G145A:p.E49K,RPH3A:ENST00000543106.2:exon5:c.G145A:p.E49K,RPH3A:ENST00000551198.1:exon5:c.G	12q24.13	.
12	113872017	C	T	GTTTGACCAC,CCCTAATATG	exonic	SDSL	nonsynonymous SNV	SDSL:ENST00000553248.1:exon4:c.C259T:p.P87S,SDSL:ENST00000345635.4:exon6:c.C433T:p.P145S,SDSL:ENST00000403593.4:exon5:c.C433T:p.P145S,	12q24.13	.
12	117768490	G	A	GCTTTGGTGG,GGGACCCAGG	exonic	NOS1	nonsynonymous SNV	NOS1:ENST00000344089.3:exon2:c.C385T:p.P129S,NOS1:ENST00000317775.6:exon2:c.C385T:p.P129S,NOS1:ENST00000338101.4:exon1:c.C385T:p.P129S,	12q24.22	.
12	117768859	A	T	TGAACACCGA,CATGTGATCC	exonic	NOS1	nonsynonymous SNV	NOS1:ENST00000344089.3:exon2:c.T16A:p.F6I,NOS1:ENST00000317775.6:exon2:c.T16A:p.F6I,NOS1:ENST00000338101.4:exon1:c.T16A:p.F6I,	12q24.22	.
12	117768860	C	T	GAACACCGAA,ATGTGATCCT	exonic	NOS1	nonsynonymous SNV	NOS1:ENST00000344089.3:exon2:c.G15A:p.M5I,NOS1:ENST00000317775.6:exon2:c.G15A:p.M5I,NOS1:ENST00000338101.4:exon1:c.G15A:p.M5I,	12q24.22	.
12	121078772	G	A	CGGGACCCGG,GCTGCCTAGC	exonic	CABP1	nonsynonymous SNV	CABP1:ENST00000316803.3:exon1:c.G284A:p.G95E,	12q24.31	.
12	123471930	C	T	GAACTCTGTG,CCTTGGGCAG	exonic	PITPNM2	nonsynonymous SNV	PITPNM2:ENST00000280562.5:exon22:c.G3293A:p.G1098D,PITPNM2:ENST00000392428.1:exon20:c.G2474A:p.G825D,PITPNM2:ENST00000542749.1:exon21:c.G3311A:p.G1104D,PITPNM2:ENST00000320201.4:exon22:c.G3311A:p.G1104D,	12q24.31	.
12	123471931	C	T	AACTCTGTGC,CTTGGGCAGC	exonic	PITPNM2	nonsynonymous SNV	PITPNM2:ENST00000280562.5:exon22:c.G3292A:p.G1098S,PITPNM2:ENST00000392428.1:exon20:c.G2473A:p.G825S,PITPNM2:ENST00000542749.1:exon21:c.G3310A:p.G1104S,PITPNM2:ENST00000320201.4:exon22:c.G3310A:p.G1104S,	12q24.31	.
12	123706321	G	A	ACTGCTTAGA,AAAAAAAACC	exonic	MPHOSPH9	nonsynonymous SNV	MPHOSPH9:ENST00000606320.1:exon5:c.C470T:p.S157F,MPHOSPH9:ENST00000302373.4:exon1:c.C74T:p.S25F,MPHOSPH9:ENST00000392425.3:exon1:c.C14T:p.S5F,MPHOSPH9:ENST00000541076.2:exon3:c.C380T:p.S127F,MPHOSPH9:ENST00000302349.5:exon1:c.C14T:p.S5F,	12q24.31	.
12	124352654	G	A	GGCCAACCCT,GGGAACTGCC	exonic	DNAH10	nonsynonymous SNV	DNAH10:ENST00000409039.3:exon42:c.G7153A:p.G2385R,	12q24.31	.
12	126138670	C	T	CCCAATAATC,TAGTGACCTC	exonic	TMEM132B	nonsynonymous SNV	TMEM132B:ENST00000535886.1:exon5:c.C1187T:p.P396L,TMEM132B:ENST00000299308.3:exon9:c.C2651T:p.P884L,	12q24.32	.
12	126139074	C	T	TTCTTCGGGC,CAAAGAGGAA	exonic	TMEM132B	nonsynonymous SNV	TMEM132B:ENST00000535886.1:exon5:c.C1591T:p.P531S,TMEM132B:ENST00000299308.3:exon9:c.C3055T:p.P1019S,	12q24.32	TMEM132B;basechange=c.3055C>A;AAchange=p.P1019T;tissue=lung
12	128899937	G	A	TTCAGGAAGG,CAACGCCATC	exonic	TMEM132C	nonsynonymous SNV	TMEM132C:ENST00000435159.2:exon2:c.G746A:p.G249D,	12q24.32	.
13	23898591	G	A	GAGCCTCTAC,AAATCTGTGT	exonic	SGCG	nonsynonymous SNV	SGCG:ENST00000545013.1:exon9:c.G787A:p.E263K,SGCG:ENST00000537476.1:exon9:c.G787A:p.E263K,SGCG:ENST00000218867.3:exon8:c.G787A:p.E263K,	13q12.12	SGCG;basechange=c.787G>A;AAchange=p.E263K;tissue=large_intestine
13	26104724	G	A	ATCTCCAACA,GAAGATATAC	exonic	ATP8A2	nonsynonymous SNV	ATP8A2:ENST00000255283.8:exon3:c.G226A:p.G76R,ATP8A2:ENST00000381655.2:exon4:c.G346A:p.G116R,	13q12.13	.
13	27255259	C	T	CACCCCCAGC,TGTGACCCCT	exonic	WASF3	nonsynonymous SNV	WASF3:ENST00000361042.4:exon8:c.C776T:p.P259L,WASF3:ENST00000335327.5:exon8:c.C785T:p.P262L,	13q12.13	.
13	28830626	G	A	AACTGTTGGT,GGACGACTTA	exonic	PAN3	nonsynonymous SNV	PAN3:ENST00000380958.3:exon7:c.G1198A:p.G400R,PAN3:ENST00000399613.1:exon6:c.G598A:p.G200R,PAN3:ENST00000282391.5:exon2:c.G262A:p.G88R,	13q12.2	.
13	31233380	C	T	CTTGGAGAGT,CGATGAAGAC	exonic	USPL1	nonsynonymous SNV	USPL1:ENST00000255304.4:exon9:c.C3166T:p.P1056S,	13q12.3	.
13	38137489	C	T	ACGACCTTCC,TTAATCGTCT	exonic	POSTN	nonsynonymous SNV	POSTN:ENST00000379742.4:exon21:c.G2321A:p.R774K,POSTN:ENST00000379747.4:exon23:c.G2492A:p.R831K,POSTN:ENST00000379743.4:exon22:c.G2411A:p.R804K,POSTN:ENST00000541179.1:exon21:c.G2327A:p.R776K,POSTN:ENST00000541481.1:exon20:c.G2231A:p.R744K,POSTN:ENST0000	13q13.3	POSTN;basechange=c.2492G>A;AAchange=p.R831K;tissue=lung
13	39338470	C	T	CCAGAATTTT,GTCTGAATTG	exonic	FREM2	nonsynonymous SNV	FREM2:ENST00000280481.7:exon3:c.C5293T:p.R1765C,	13q13.3	FREM2;basechange=c.5293C>T;AAchange=p.R1765C;tissue=skin
13	39454470	C	T	ACAGTGAGAT,GAAAGACAAT	exonic	FREM2	nonsynonymous SNV	FREM2:ENST00000280481.7:exon24:c.C9056T:p.S3019L,	13q13.3	FREM2;basechange=c.9056C>T;AAchange=p.S3019L;tissue=large_intestine,endometrium
13	41556157	A	C	GCAAATTCAA,TACTAGGTCG	exonic	ELF1	nonsynonymous SNV	ELF1:ENST00000239882.3:exon2:c.T34G:p.F12V,ELF1:ENST00000442101.1:exon1:c.T34G:p.F12V,ELF1:ENST00000405737.2:exon2:c.T34G:p.F12V,	13q14.11	.
13	42273298	G	A	GGCTGTTCTT,GGAAGATATC	exonic	VWA8	nonsynonymous SNV	VWA8:ENST00000379310.3:exon29:c.C3473T:p.P1158L,	13q14.11	.
13	53421599	G	A	TCCTGCGCCC,CACGTCCAGC	exonic	PCDH8	nonsynonymous SNV	PCDH8:ENST00000377942.3:exon1:c.C973T:p.R325W,PCDH8:ENST00000338862.4:exon1:c.C973T:p.R325W,	13q14.3	.
13	70281852	G	A	CTGTCACCAA,GAGACAGACC	exonic	KLHL1	nonsynonymous SNV	KLHL1:ENST00000377844.4:exon10:c.C2092T:p.L698F,KLHL1:ENST00000545028.1:exon9:c.C1513T:p.L505F,	13q21.33	.
13	70514239	C	T	GGCTCGAATT,CTAAACAGTT	exonic	KLHL1	nonsynonymous SNV	KLHL1:ENST00000377844.4:exon4:c.G947A:p.G316E,KLHL1:ENST00000545028.1:exon3:c.G368A:p.G123E,	13q21.33	KLHL1;basechange=c.947G>C;AAchange=p.G316A;tissue=liver
13	77751920	C	T	TTCATGAATT,CACCTCCTCC	exonic	MYCBP2	nonsynonymous SNV	MYCBP2:ENST00000544440.2:exon35:c.G5189A:p.G1730E,MYCBP2:ENST00000407578.2:exon35:c.G5303A:p.G1768E,MYCBP2:ENST00000357337.6:exon36:c.G5189A:p.G1730E,	13q22.3	.
13	77847786	G	A	ATTTCCAAAA,AAGCTGGAAG	exonic	MYCBP2	nonsynonymous SNV	MYCBP2:ENST00000544440.2:exon5:c.C652T:p.L218F,MYCBP2:ENST00000407578.2:exon5:c.C766T:p.L256F,MYCBP2:ENST00000357337.6:exon6:c.C652T:p.L218F,	13q22.3	.
13	102227873	G	A	ATGCATAGAC,ATGAAACAGA	exonic	ITGBL1	nonsynonymous SNV	ITGBL1:ENST00000376180.3:exon4:c.G562A:p.D188N,ITGBL1:ENST00000545560.2:exon3:c.G139A:p.D47N,ITGBL1:ENST00000376162.3:exon3:c.G283A:p.D95N,	13q33.1	ITGBL1;basechange=c.562G>A;AAchange=p.D188N;tissue=skin
13	103443656	A	C	CGTAGGCATC,CGATATCCTT	exonic	KDELC1	nonsynonymous SNV	KDELC1:ENST00000376004.4:exon5:c.T797G:p.V266G,	13q33.1	.
13	108882558	C	T	GCAAAAACTT,ATCTAATGTA	exonic	ABHD13	nonsynonymous SNV	ABHD13:ENST00000375898.3:exon2:c.C992T:p.S331L,	13q33.3	.
13	109318281	G	A	GATGGAAATC,ACCAGTGCTT	exonic	MYO16	nonsynonymous SNV	MYO16:ENST00000251041.5:exon1:c.G10A:p.D4N,MYO16:ENST00000357550.2:exon1:c.G10A:p.D4N,MYO16:ENST00000356711.2:exon2:c.G10A:p.D4N,	13q33.3	.
13	109535435	G	A	AGCTCCTCAG,GAAGCTGTGT	exonic	MYO16	nonsynonymous SNV	MYO16:ENST00000251041.5:exon12:c.G1388A:p.G463E,MYO16:ENST00000357550.2:exon12:c.G1388A:p.G463E,MYO16:ENST00000356711.2:exon13:c.G1388A:p.G463E,	13q33.3	.
13	110819508	G	A	GTCTTACCTT,AAATCCTGGA	exonic	COL4A1	stopgain	COL4A1:ENST00000375820.4:exon44:c.C3946T:p.Q1316X,	13q34	.
13	111532441	G	A	ATTCGATACG,AGCTACTGTT	exonic	ANKRD10	nonsynonymous SNV	ANKRD10:ENST00000267339.2:exon6:c.C806T:p.S269F,	13q34	.
14	20296115	G	A	GCCTTTTTGT,GCCCAAACCA	exonic	OR4N2	nonsynonymous SNV	OR4N2:ENST00000315947.1:exon1:c.G508A:p.G170S,OR4N2:ENST00000568211.1:exon1:c.G508A:p.G170S,	14q11.2	.
14	20389727	C	T	GTGAGAACTT,CTTTCATTAA	exonic	OR4K5	nonsynonymous SNV	OR4K5:ENST00000315915.4:exon1:c.C962T:p.S321F,	14q11.2	.
14	20711329	G	A	CTTTCTGGCA,TAATGGCTTA	exonic	OR11H4	nonsynonymous SNV	OR11H4:ENST00000315409.2:exon1:c.G379A:p.V127I,	14q11.2	.
14	21058501	C	T	AGAACAAACC,CTCTGTGGGG	exonic	RNASE11,RNASE12	nonsynonymous SNV	RNASE12:ENST00000556526.1:exon1:c.G382A:p.G128R,RNASE11:ENST00000335950.4:exon1:c.G196A:p.G66R,	14q11.2	.
14	22180575	G	A	GAGCACTCTG,GGGCGGTGTG	exonic	TRAV2	nonsynonymous SNV	TRAV2:ENST00000390424.2:exon1:c.G22A:p.G8R,	14q11.2	.
14	22265763	G	A	GATTTTTTCA,GAGATGCCAG	splicing	TRAV8-1(ENST00000390430.2:exon2:c.47-1G>A)	.	.	14q11.2	.
14	23444286	G	A	CAGCCTGGAT,ATAGGACTTC	exonic	AJUBA	nonsynonymous SNV	AJUBA:ENST00000361265.4:exon5:c.C1267T:p.H423Y,AJUBA:ENST00000262713.2:exon5:c.C1267T:p.H423Y,AJUBA:ENST00000397388.3:exon3:c.C16T:p.H6Y,AJUBA:ENST00000553592.1:exon4:c.C16T:p.H6Y,AJUBA:ENST00000556731.1:exon4:c.C16T:p.H6Y,AJUBA:ENST00000553911.1:exon4:c.C16T:p	14q11.2	.
14	23829912	G	A	GCCGTGTAGG,AGCAGAGGCA	exonic	EFS	nonsynonymous SNV	EFS:ENST00000216733.3:exon2:c.C149T:p.S50F,	14q11.2	.
14	23863400	C	T	ACTCTTCCTT,ATGGTGGCCA	exonic	MYH6	nonsynonymous SNV	MYH6:ENST00000405093.3:exon21:c.G2562A:p.M854I,MYH6:ENST00000356287.3:exon20:c.G2562A:p.M854I,	14q11.2	.
14	24040265	G	A	GTGCCTGCTG,GGCCCTCAGC	exonic	JPH4	nonsynonymous SNV	JPH4:ENST00000397118.3:exon6:c.C1675T:p.P559S,JPH4:ENST00000544177.1:exon3:c.C670T:p.P224S,JPH4:ENST00000356300.4:exon5:c.C1675T:p.P559S,	14q11.2	.
14	31642744	G	A	TGTTACTACC,GAGAGCCTCT	exonic	HECTD1	nonsynonymous SNV	HECTD1:ENST00000553700.1:exon5:c.C872T:p.P291L,HECTD1:ENST00000556224.1:exon5:c.C872T:p.P291L,HECTD1:ENST00000399332.1:exon5:c.C872T:p.P291L,	14q12	.
14	33242896	G	A	CCTCTGTCGT,AAATCAAGCA	exonic	AKAP6	nonsynonymous SNV	AKAP6:ENST00000280979.4:exon12:c.G3385A:p.E1129K,AKAP6:ENST00000557272.1:exon12:c.G3385A:p.E1129K,	14q12	AKAP6;basechange=c.3385G>T;AAchange=p.E1129*;tissue=lung
14	33299099	G	A	TCCCGCCCTG,GCTGGCCTCT	exonic	AKAP6	nonsynonymous SNV	AKAP6:ENST00000557272.1:exon13:c.G3634A:p.G1212S,	14q12	.
14	45644696	T	G	GTACTATTAA,GAAAATGTTA	exonic	FANCM	nonsynonymous SNV	FANCM:ENST00000556250.1:exon7:c.T1287G:p.N429K,FANCM:ENST00000267430.5:exon14:c.T2739G:p.N913K,FANCM:ENST00000542564.2:exon13:c.T2661G:p.N887K,	14q21.2	.
14	47351373	G	A	TCTCCCTTTT,AATATTCCCA	exonic	MDGA2	stopgain	MDGA2:ENST00000439988.3:exon11:c.C2290T:p.Q764X,MDGA2:ENST00000357362.3:exon11:c.C1396T:p.Q466X,MDGA2:ENST00000399232.2:exon11:c.C2083T:p.Q695X,MDGA2:ENST00000426342.1:exon11:c.C1396T:p.Q466X,	14q21.3	.
14	52507393	C	T	TGGTACAGCT,CTTGTAGGGA	exonic	NID2	nonsynonymous SNV	NID2:ENST00000541773.1:exon9:c.G1843A:p.E615K,NID2:ENST00000216286.5:exon8:c.G2002A:p.E668K,	14q22.1	.
14	53619765	C	T	CCGCCGCCGC,TCGGCCGTTA	exonic	DDHD1	nonsynonymous SNV	DDHD1:ENST00000395606.1:exon1:c.G52A:p.G18S,DDHD1:ENST00000357758.3:exon1:c.G52A:p.G18S,DDHD1:ENST00000323669.5:exon1:c.G52A:p.G18S,	14q22.1	.
14	56139414	G	A	TAAGGATATT,AAAATGTATG	exonic	KTN1	nonsynonymous SNV	KTN1:ENST00000438792.2:exon37:c.G3511A:p.E1171K,KTN1:ENST00000395311.1:exon37:c.G3529A:p.E1177K,KTN1:ENST00000554507.1:exon20:c.G1396A:p.E466K,KTN1:ENST00000459737.1:exon39:c.G3598A:p.E1200K,KTN1:ENST00000395314.3:exon38:c.G3598A:p.E1200K,KTN1:ENST0000055557	14q22.3	.
14	57675371	G	A	TAATCAGCAC,AAGTTGTACG	exonic	EXOC5	nonsynonymous SNV	EXOC5:ENST00000340918.7:exon17:c.C1888T:p.R630C,EXOC5:ENST00000413566.2:exon18:c.C2083T:p.R695C,	14q22.3	EXOC5;basechange=c.2083C>T;AAchange=p.R695C;tissue=large_intestine,endometrium
14	58056080	C	A	TTTTTACAAT,TGTGTAGTTC	exonic	SLC35F4	nonsynonymous SNV	SLC35F4:ENST00000556826.1:exon3:c.G441T:p.Q147H,SLC35F4:ENST00000557254.1:exon3:c.G72T:p.Q24H,SLC35F4:ENST00000339762.6:exon3:c.G549T:p.Q183H,SLC35F4:ENST00000554729.1:exon3:c.G72T:p.Q24H,	14q22.3	.
14	58063511	C	T	GTCCTGGCTT,CATACCTCCT	exonic	SLC35F4	stopgain	SLC35F4:ENST00000339762.6:exon1:c.G105A:p.W35X,	14q22.3	.
14	58820380	G	A	CTGCAGGGAA,AAACTGAAAG	exonic	ARID4A	nonsynonymous SNV	ARID4A:ENST00000355431.3:exon17:c.G1660A:p.E554K,ARID4A:ENST00000395168.3:exon17:c.G1660A:p.E554K,ARID4A:ENST00000348476.3:exon17:c.G1660A:p.E554K,ARID4A:ENST00000431317.2:exon16:c.G1660A:p.E554K,ARID4A:ENST00000417477.2:exon7:c.G694A:p.E232K,	14q23.1	.
14	60489576	C	T	TTTAAAATTC,TCTTTCTACA	exonic	LRRC9	nonsynonymous SNV	LRRC9:ENST00000445360.1:exon27:c.C3691T:p.L1231F,	14q23.1	.
14	60585233	C	T	ACTCTCTTCT,CCTTACTCCC	exonic	PCNXL4	nonsynonymous SNV	PCNXL4:ENST00000406949.1:exon6:c.C1063T:p.P355S,PCNXL4:ENST00000404681.2:exon6:c.C1765T:p.P589S,PCNXL4:ENST00000406854.1:exon7:c.C1765T:p.P589S,PCNXL4:ENST00000555740.1:exon1:c.C73T:p.P25S,PCNXL4:ENST00000317623.4:exon6:c.C1063T:p.P355S,	14q23.1	.
14	60587958	C	T	TTTTCAGGAT,GTTTAATGTG	exonic	PCNXL4	nonsynonymous SNV	PCNXL4:ENST00000406949.1:exon7:c.C1285T:p.R429C,PCNXL4:ENST00000404681.2:exon7:c.C1987T:p.R663C,PCNXL4:ENST00000406854.1:exon8:c.C1987T:p.R663C,PCNXL4:ENST00000555740.1:exon2:c.C295T:p.R99C,PCNXL4:ENST00000317623.4:exon7:c.C1285T:p.R429C,	14q23.1	.
14	60923693	C	A	GGTGCTTTCA,AGCTTTGGGA	exonic	C14orf39	nonsynonymous SNV	C14orf39:ENST00000321731.3:exon15:c.G1300T:p.V434L,	14q23.1	.
14	61186535	C	A	CCAATGCTCC,ATTAAGGAAC	exonic	SIX4	nonsynonymous SNV	SIX4:ENST00000216513.4:exon2:c.G1492T:p.G498W,SIX4:ENST00000556952.3:exon3:c.G1468T:p.G490W,	14q23.1	.
14	71444298	C	T	AGCATCCTGT,AGAGCATGAG	exonic	PCNX	nonsynonymous SNV	PCNX:ENST00000439984.3:exon6:c.C1244T:p.S415L,PCNX:ENST00000304743.2:exon6:c.C1244T:p.S415L,PCNX:ENST00000238570.5:exon6:c.C1244T:p.S415L,	14q24.2	.
14	79175641	G	A	AATCTATGGC,AAGTTGTGTT	exonic	NRXN3	nonsynonymous SNV	NRXN3:ENST00000553631.1:exon3:c.G16A:p.E6K,NRXN3:ENST00000335750.5:exon4:c.G184A:p.E62K,NRXN3:ENST00000554719.1:exon4:c.G184A:p.E62K,NRXN3:ENST00000557081.1:exon3:c.G16A:p.E6K,NRXN3:ENST00000554738.1:exon6:c.G1297A:p.E433K,	14q24.3	.
14	79434518	G	A	CTTTCCAGGT,ATCTCTATAT	exonic	NRXN3	nonsynonymous SNV	NRXN3:ENST00000335750.5:exon11:c.G1852A:p.D618N,NRXN3:ENST00000554719.1:exon11:c.G1852A:p.D618N,NRXN3:ENST00000554738.1:exon12:c.G2938A:p.D980N,	14q31.1	.
14	79454437	C	T	GAGGGCTTCA,CTGTGATTGT	exonic	NRXN3	nonsynonymous SNV	NRXN3:ENST00000335750.5:exon12:c.C2096T:p.T699I,NRXN3:ENST00000554719.1:exon12:c.C2096T:p.T699I,NRXN3:ENST00000554738.1:exon13:c.C3182T:p.T1061I,	14q31.1	.
14	80164155	C	T	TGGAGAAGTC,CATCAATTTT	exonic	NRXN3	nonsynonymous SNV	NRXN3:ENST00000335750.5:exon15:c.C2680T:p.P894S,NRXN3:ENST00000554719.1:exon15:c.C2680T:p.P894S,NRXN3:ENST00000281127.7:exon4:c.C784T:p.P262S,NRXN3:ENST00000428277.2:exon5:c.C874T:p.P292S,NRXN3:ENST00000557594.1:exon4:c.C784T:p.P262S,NRXN3:ENST00000555387.1:	14q31.1	.
14	89817051	G	T	GAGGTGTATT,AACACGTGTG	exonic	FOXN3	nonsynonymous SNV	FOXN3:ENST00000261302.5:exon2:c.C645A:p.F215L,FOXN3:ENST00000554005.1:exon2:c.C204A:p.F68L,FOXN3:ENST00000345097.4:exon3:c.C645A:p.F215L,FOXN3:ENST00000557718.1:exon2:c.C186A:p.F62L,FOXN3:ENST00000553353.1:exon2:c.C198A:p.F66L,FOXN3:ENST00000557258.1:exon3:c.C	14q32.11	.
14	92922925	G	A	GGTGGAGGCT,ACTTCCTGTC	exonic	SLC24A4	nonsynonymous SNV	SLC24A4:ENST00000298877.1:exon12:c.G1177A:p.D393N,SLC24A4:ENST00000351924.5:exon12:c.G1120A:p.D374N,SLC24A4:ENST00000531433.1:exon13:c.G1171A:p.D391N,SLC24A4:ENST00000532405.1:exon12:c.G1228A:p.D410N,SLC24A4:ENST00000393265.2:exon12:c.G1036A:p.D346N,	14q32.12	.
14	93934110	T	G	GCAAACACCT,GAAATACTTT	exonic	UNC79	nonsynonymous SNV	UNC79:ENST00000393151.2:exon2:c.T116G:p.L39W,UNC79:ENST00000553484.1:exon2:c.T116G:p.L39W,UNC79:ENST00000555664.1:exon2:c.T116G:p.L39W,	14q32.12	.
14	94770815	C	T	GGTCGAAGAT,ATGATGATGA	exonic	SERPINA6	nonsynonymous SNV	SERPINA6:ENST00000341584.3:exon5:c.G1158A:p.M386I,	14q32.13	SERPINA6;basechange=c.1158G>A;AAchange=p.M386I;tissue=lung
14	95035919	C	T	GTCGTCGACC,CACGAAACCA	exonic	SERPINA4	nonsynonymous SNV	SERPINA4:ENST00000555095.1:exon5:c.C1271T:p.P424L,SERPINA4:ENST00000557004.1:exon5:c.C1271T:p.P424L,SERPINA4:ENST00000298841.5:exon5:c.C1271T:p.P424L,	14q32.13	.
14	100118820	G	A	TCAAACCTGG,CCACGTGGTA	exonic	HHIPL1	nonsynonymous SNV	HHIPL1:ENST00000357223.2:exon2:c.G515A:p.G172D,HHIPL1:ENST00000330710.5:exon2:c.G515A:p.G172D,	14q32.2	.
14	101347500	C	T	AGGGAGGTGG,CCTCCTGGGG	exonic	RTL1	nonsynonymous SNV	RTL1:ENST00000534062.1:exon1:c.G3626A:p.G1209D,	14q32.2	.
14	102808307	G	A	TGGCCAGAGA,AACTGGAGAG	exonic	ZNF839	nonsynonymous SNV	ZNF839:ENST00000262236.5:exon8:c.G2233A:p.E745K,ZNF839:ENST00000561251.1:exon6:c.G1219A:p.E407K,ZNF839:ENST00000442396.2:exon8:c.G2575A:p.E859K,ZNF839:ENST00000558850.1:exon8:c.G2227A:p.E743K,ZNF839:ENST00000559185.1:exon8:c.G2227A:p.E743K,	14q32.31	.
14	104641734	G	A	GCTCAGGCCA,CCCCGCCCGA	exonic	KIF26A	nonsynonymous SNV	KIF26A:ENST00000315264.7:exon11:c.G2192A:p.S731N,KIF26A:ENST00000423312.2:exon12:c.G2609A:p.S870N,	14q32.33	.
14	106204166	G	A	CACCACCGAG,AGAAGATCCA	exonic	IGHG1	nonsynonymous SNV	IGHG1:ENST00000390548.2:exon6:c.C1133T:p.S378F,	14q32.33	.
15	23685799	C	T	TTCTTCTCCT,CCGCATCTTC	exonic	GOLGA6L2	nonsynonymous SNV	GOLGA6L2:ENST00000567107.1:exon8:c.G1823A:p.G608E,	15q11.2	.
15	23689316	T	G	CACCTCTATC,CCCGCCTTAG	exonic	GOLGA6L2	nonsynonymous SNV	GOLGA6L2:ENST00000567107.1:exon4:c.A287C:p.E96A,GOLGA6L2:ENST00000566571.1:exon3:c.A257C:p.E86A,GOLGA6L2:ENST00000312015.5:exon4:c.A287C:p.E96A,	15q11.2	GOLGA6L2;basechange=c.287A>G;AAchange=p.E96G;tissue=oesophagus&ENSG00000174450;basechange=c.287A>G;AAchange=p.E
15	24921709	C	T	CCAGCGAGCT,CTGCTTGGAA	exonic	NPAP1	nonsynonymous SNV	NPAP1:ENST00000329468.2:exon1:c.C695T:p.S232F,	15q11.2	.
15	41805267	G	A	CGGCAGGGGC,AGGACCGCAG	exonic	LTK	nonsynonymous SNV	LTK:ENST00000453182.2:exon2:c.C95T:p.S32L,LTK:ENST00000355166.5:exon2:c.C95T:p.S32L,LTK:ENST00000263800.6:exon2:c.C95T:p.S32L,	15q15.1	.
15	42178465	C	T	TCCCGCGCCT,CAGCTGCATC	exonic	SPTBN5	nonsynonymous SNV	SPTBN5:ENST00000320955.6:exon7:c.G988A:p.E330K,	15q15.1	.
15	42438054	C	T	GGAACATAAG,CCCGTACTTG	exonic	PLA2G4F	nonsynonymous SNV	PLA2G4F:ENST00000382396.4:exon15:c.G1582A:p.A528T,PLA2G4F:ENST00000397272.3:exon15:c.G1588A:p.A530T,	15q15.1	.
15	42805586	C	T	GCTTTTCCCC,CTTGTCTTAG	exonic	SNAP23	nonsynonymous SNV	SNAP23:ENST00000563765.1:exon4:c.C152T:p.P51L,	15q15.2	.
15	44092893	C	T	TCCGGAGAAG,CACGGAAACA	exonic	HYPK,SERF2	nonsynonymous SNV	SERF2:ENST00000600633.1:exon1:c.C97T:p.P33S,SERF2:ENST00000594896.1:exon3:c.C235T:p.P79S,HYPK:ENST00000406925.1:exon2:c.C97T:p.P33S,HYPK:ENST00000442995.2:exon1:c.C97T:p.P33S,HYPK:ENST00000458412.1:exon1:c.C97T:p.P33S,	15q15.3	.
15	44092894	C	T	CCGGAGAAGC,ACGGAAACAT	exonic	HYPK,SERF2	nonsynonymous SNV	SERF2:ENST00000600633.1:exon1:c.C98T:p.P33L,SERF2:ENST00000594896.1:exon3:c.C236T:p.P79L,HYPK:ENST00000406925.1:exon2:c.C98T:p.P33L,HYPK:ENST00000442995.2:exon1:c.C98T:p.P33L,HYPK:ENST00000458412.1:exon1:c.C98T:p.P33L,	15q15.3	.
15	48722909	C	T	ATACCCGGGT,CACAGATGCA	exonic	FBN1	nonsynonymous SNV	FBN1:ENST00000316623.5:exon56:c.G6830A:p.G2277E,	15q21.1	.
15	48773869	C	T	GCCAGTTTTT,CTTTTTTGCC	exonic	FBN1	nonsynonymous SNV	FBN1:ENST00000316623.5:exon32:c.G3947A:p.G1316E,	15q21.1	.
15	48892427	T	A	TATTGCAGTG,TGTACTTGAA	exonic	FBN1	nonsynonymous SNV	FBN1:ENST00000316623.5:exon5:c.A351T:p.Q117H,FBN1:ENST00000537463.2:exon5:c.A351T:p.Q117H,	15q21.1	.
15	49420933	C	A	AAGCTCTCCA,ATCAGCTACA	exonic	COPS2	nonsynonymous SNV	COPS2:ENST00000388901.5:exon12:c.G1153T:p.V385L,COPS2:ENST00000299259.6:exon12:c.G1174T:p.V392L,COPS2:ENST00000542928.1:exon10:c.G961T:p.V321L,	15q21.1	.
15	51792089	G	A	TCCTCCTGTA,ATTCACATTC	exonic	DMXL2	nonsynonymous SNV	DMXL2:ENST00000543779.2:exon18:c.C3332T:p.S1111F,DMXL2:ENST00000251076.5:exon18:c.C3332T:p.S1111F,	15q21.2	.
15	59192088	G	A	CCCTTCTGAA,GAAGAGGTTT	exonic	SLTM	nonsynonymous SNV	SLTM:ENST00000380516.2:exon7:c.C638T:p.P213L,SLTM:ENST00000558486.1:exon5:c.C392T:p.P131L,	15q22.1	.
15	63632497	G	A	CTGCTCCTGG,AAATTTGCAC	exonic	CA12	nonsynonymous SNV	CA12:ENST00000422263.2:exon6:c.C557T:p.S186F,CA12:ENST00000178638.3:exon7:c.C737T:p.S246F,CA12:ENST00000344366.3:exon7:c.C737T:p.S246F,	15q22.2	.
15	63961860	G	A	CGACGTGTGG,AAGTACAGTG	exonic	HERC1	nonsynonymous SNV	HERC1:ENST00000443617.2:exon40:c.C8083T:p.P2695S,	15q22.31	.
15	65686856	G	A	GTTGGGGCTG,ACAGGGAGAG	exonic	IGDCC4	nonsynonymous SNV	IGDCC4:ENST00000352385.2:exon9:c.C1607T:p.S536F,	15q22.31	.
15	68624252	C	T	GTAGATGGCT,CTGCGTGGTT	exonic	ITGA11	nonsynonymous SNV	ITGA11:ENST00000423218.2:exon14:c.G1715A:p.G572E,ITGA11:ENST00000315757.7:exon14:c.G1715A:p.G572E,	15q23	.
15	69076847	G	A	AGTTGCGGGA,GAGCTTGAAC	exonic	ANP32A	nonsynonymous SNV	ANP32A:ENST00000560303.1:exon4:c.C415T:p.L139F,ANP32A:ENST00000465139.2:exon4:c.C415T:p.L139F,	15q23	.
15	72021038	C	T	GCGGAAGGTC,CACCAACGAG	exonic	THSD4	nonsynonymous SNV	THSD4:ENST00000355327.3:exon9:c.C1508T:p.P503L,THSD4:ENST00000357769.4:exon3:c.C428T:p.P143L,THSD4:ENST00000261862.6:exon8:c.C1508T:p.P503L,	15q23	.
15	73615025	G	A	CTCCCAGGGG,ACCGAGGCCC	exonic	HCN4	nonsynonymous SNV	HCN4:ENST00000261917.3:exon8:c.C3409T:p.P1137S,	15q24.1	.
15	74177155	C	T	GACAAAGATC,CCTGGGCAAC	exonic	TBC1D21	nonsynonymous SNV	TBC1D21:ENST00000300504.2:exon5:c.C401T:p.P134L,TBC1D21:ENST00000535547.2:exon4:c.C293T:p.P98L,	15q24.1	.
15	74703027	G	C	GAGGCGGCCA,GGCACAGGCA	exonic	SEMA7A	nonsynonymous SNV	SEMA7A:ENST00000261918.4:exon14:c.C1939G:p.L647V,SEMA7A:ENST00000542748.1:exon14:c.C1444G:p.L482V,SEMA7A:ENST00000543145.2:exon13:c.C1897G:p.L633V,	15q24.1	.
15	75657981	T	C	GCAGGAGAAT,GCTTGAACCT	exonic	MAN2C1	nonsynonymous SNV	MAN2C1:ENST00000569355.1:exon5:c.A481G:p.N161D,	15q24.2	.
15	79750014	G	A	GCACTCAGAC,ATGACTCAGA	exonic	KIAA1024	nonsynonymous SNV	KIAA1024:ENST00000305428.3:exon2:c.G1525A:p.D509N,KIAA1024:ENST00000559272.1:exon1:c.G1525A:p.D509N,	15q25.1	KIAA1024;basechange=c.1525G>T;AAchange=p.D509Y;tissue=large_intestine
15	85166542	T	C	CCAGGATGGT,TCGATCTCCT	exonic	ZSCAN2	nonsynonymous SNV	ZSCAN2:ENST00000541040.1:exon3:c.T613C:p.F205L,	15q25.2	.
15	89401043	C	T	ATCAGGGTTT,CTGACACTAG	exonic	ACAN	nonsynonymous SNV	ACAN:ENST00000561243.1:exon11:c.C5227T:p.P1743S,ACAN:ENST00000559004.1:exon12:c.C5227T:p.P1743S,ACAN:ENST00000352105.7:exon12:c.C5227T:p.P1743S,ACAN:ENST00000439576.2:exon12:c.C5227T:p.P1743S,	15q26.1	.
15	93540297	A	G	GCATAAATCT,TCCCTGTGGA	exonic	CHD2	nonsynonymous SNV	CHD2:ENST00000557381.1:exon29:c.A3706G:p.I1236V,CHD2:ENST00000394196.4:exon29:c.A3706G:p.I1236V,	15q26.1	.
15	99759184	G	A	CTCACTATAG,GAGGCACAAT	exonic	TTC23	nonsynonymous SNV	TTC23:ENST00000558613.1:exon4:c.C374T:p.P125L,TTC23:ENST00000394130.1:exon6:c.C374T:p.P125L,TTC23:ENST00000560279.1:exon5:c.C374T:p.P125L,TTC23:ENST00000394136.1:exon6:c.C374T:p.P125L,TTC23:ENST00000394132.2:exon7:c.C374T:p.P125L,TTC23:ENST00000394135.3:exon5:c.C	15q26.3	.
15	100657178	C	T	ACCGCATGTT,TACACTGGCA	exonic	ADAMTS17	nonsynonymous SNV	ADAMTS17:ENST00000268070.4:exon13:c.G1762A:p.E588K,	15q26.3	.
16	343558	G	A	TCCTCCAGCT,GGTTAGGGGG	exonic	AXIN1	stopgain	AXIN1:ENST00000262320.3:exon8:c.C2116T:p.Q706X,AXIN1:ENST00000354866.3:exon8:c.C2116T:p.Q706X,	16p13.3	.
16	427050	G	A	AGGTAGCTGG,ATGGGAGAGG	exonic	TMEM8A	nonsynonymous SNV	TMEM8A:ENST00000431232.2:exon4:c.C622T:p.P208S,TMEM8A:ENST00000250930.3:exon4:c.C43T:p.P15S,TMEM8A:ENST00000427313.1:exon4:c.C43T:p.P15S,	16p13.3	.
16	460259	C	T	CCGGGAACTT,CTGTGCCCCG	exonic	DECR2	nonsynonymous SNV	DECR2:ENST00000439661.1:exon6:c.C541T:p.P181S,DECR2:ENST00000397710.1:exon6:c.C541T:p.P181S,	16p13.3	.
16	734415	G	A	GGCAACGGCC,GGAACCGGGA	exonic	JMJD8	nonsynonymous SNV	JMJD8:ENST00000454700.1:exon1:c.C115T:p.R39W,JMJD8:ENST00000293882.4:exon1:c.C115T:p.R39W,	16p13.3	.
16	765366	C	T	GCCGGCTACT,CGAGGAGCGC	exonic	METRN	nonsynonymous SNV	METRN:ENST00000219542.3:exon1:c.C29T:p.S10F,METRN:ENST00000570132.1:exon1:c.C77T:p.S26F,METRN:ENST00000568223.2:exon1:c.C77T:p.S26F,	16p13.3	.
16	1447183	G	A	ACGTACCTCG,GATGGAACTG	exonic	UNKL	nonsynonymous SNV	UNKL:ENST00000397462.1:exon8:c.C1157T:p.P386L,UNKL:ENST00000389221.4:exon6:c.C848T:p.P283L,UNKL:ENST00000508903.2:exon6:c.C848T:p.P283L,	16p13.3	.
16	1568341	G	A	GGACTCCTTG,GGTCCTCTGT	exonic	IFT140	nonsynonymous SNV	IFT140:ENST00000426508.2:exon30:c.C4058T:p.P1353L,IFT140:ENST00000568837.1:exon2:c.C176T:p.P59L,IFT140:ENST00000361339.5:exon12:c.C1640T:p.P547L,	16p13.3	.
16	3777900	G	A	TCCGGGGTGG,GGGAACCAGT	exonic	CREBBP	nonsynonymous SNV	CREBBP:ENST00000382070.3:exon30:c.C7034T:p.P2345L,CREBBP:ENST00000262367.5:exon31:c.C7148T:p.P2383L,	16p13.3	.
16	4812289	G	A	CAGGCCGAAA,GAAGGGCGAG	exonic	ZNF500	nonsynonymous SNV	ZNF500:ENST00000545009.1:exon4:c.C646T:p.L216F,ZNF500:ENST00000219478.6:exon4:c.C646T:p.L216F,	16p13.3	.
16	5097914	G	A	CACTCTCCTC,AAAAATGCTG	exonic	C16orf89	nonsynonymous SNV	C16orf89:ENST00000474471.3:exon8:c.C1016T:p.S339L,C16orf89:ENST00000422873.1:exon7:c.C1034T:p.S345L,C16orf89:ENST00000472572.3:exon7:c.C920T:p.S307L,C16orf89:ENST00000350219.4:exon7:c.C1034T:p.S345L,C16orf89:ENST00000315997.5:exon7:c.C920T:p.S307L,	16p13.3	C16orf89_ENST00000422873;basechange=c.1034C>T;AAchange=p.S345L;tissue=large_intestine&AC026458.2;basechange=c.920C>T
16	16205284	C	T	CTCTTCATCT,CTTCCTCAGC	exonic	ABCC1	nonsynonymous SNV	ABCC1:ENST00000399408.2:exon23:c.C2954T:p.S985F,ABCC1:ENST00000349029.5:exon20:c.C2579T:p.S860F,ABCC1:ENST00000345148.5:exon22:c.C2924T:p.S975F,ABCC1:ENST00000399410.3:exon22:c.C2924T:p.S975F,ABCC1:ENST00000346370.5:exon21:c.C2756T:p.S919F,ABCC1:ENST0000035	16p13.11	.
16	22268126	T	A	CAAGCCCCTC,TCCACCTGGA	exonic	EEF2K	nonsynonymous SNV	EEF2K:ENST00000263026.5:exon7:c.T676A:p.F226I,EEF2K:ENST00000568269.1:exon7:c.T676A:p.F226I,	16p12.2	.
16	22545815	C	T	GGGCCGCTTC,ACCCTCAGCG	exonic	NPIPB5	nonsynonymous SNV	NPIPB5:ENST00000528249.1:exon7:c.C1511T:p.P504L,NPIPB5:ENST00000517539.1:exon8:c.C1511T:p.P504L,NPIPB5:ENST00000424340.1:exon7:c.C1511T:p.P504L,NPIPB5:ENST00000415833.2:exon11:c.C1511T:p.P504L,	16p12.2	.
16	23226602	C	T	TCCCCGTAGC,CACAGGGCCA	exonic	SCNN1G	nonsynonymous SNV	SCNN1G:ENST00000300061.2:exon13:c.C1762T:p.P588S,	16p12.2	.
16	23226603	C	T	CCCCGTAGCC,ACAGGGCCAG	exonic	SCNN1G	nonsynonymous SNV	SCNN1G:ENST00000300061.2:exon13:c.C1763T:p.P588L,	16p12.2	.
16	23568941	G	A	GGTCTCCATA,AAACCTCCAC	exonic	UBFD1	nonsynonymous SNV	UBFD1:ENST00000219638.4:exon4:c.G550A:p.E184K,	16p12.2	.
16	23690749	G	A	ACCCAGCAAG,GAGAGCCTGG	exonic	PLK1	nonsynonymous SNV	PLK1:ENST00000570220.1:exon1:c.G496A:p.G166R,	16p12.2	.
16	24801036	C	T	GGGTTAAATC,AAGCACTTTG	exonic	TNRC6A	nonsynonymous SNV	TNRC6A:ENST00000395799.3:exon6:c.C1073T:p.P358L,TNRC6A:ENST00000315183.7:exon6:c.C1073T:p.P358L,	16p12.1	.
16	24942678	G	A	GGGTGGCCAG,AGGTGGGTTG	exonic	ARHGAP17	nonsynonymous SNV	ARHGAP17:ENST00000303665.5:exon18:c.C1708T:p.P570S,ARHGAP17:ENST00000289968.6:exon19:c.C1942T:p.P648S,	16p12.1	.
16	27445698	C	A	GTCTGCTACA,CGATTACCTC	exonic	IL21R	nonsynonymous SNV	IL21R:ENST00000564089.1:exon4:c.C80A:p.T27N,IL21R:ENST00000337929.3:exon3:c.C80A:p.T27N,IL21R:ENST00000395755.1:exon3:c.C80A:p.T27N,IL21R:ENST00000395754.4:exon3:c.C80A:p.T27N,	16p12.1	.
16	28074432	A	T	CGAGTACCAG,TGCCGGTGTG	exonic	GSG1L	nonsynonymous SNV	GSG1L:ENST00000447459.2:exon1:c.T314A:p.I105N,GSG1L:ENST00000395724.3:exon1:c.T314A:p.I105N,	16p12.1	.
16	29756022	G	A	GGTGCCCATG,GCTCAATCCA	exonic	C16orf54	nonsynonymous SNV	C16orf54:ENST00000329410.3:exon2:c.C251T:p.P84L,	16p11.2	.
16	29756023	G	A	GTGCCCATGG,CTCAATCCAC	exonic	C16orf54	nonsynonymous SNV	C16orf54:ENST00000329410.3:exon2:c.C250T:p.P84S,	16p11.2	.
16	29992924	C	T	CAGTTCCATT,CATTGTCCTC	exonic	TAOK2	nonsynonymous SNV	TAOK2:ENST00000416441.2:exon1:c.C79T:p.P27S,	16p11.2	.

16	29992925	C	T	AGTTCCATTC,ATTGTCCTCT	exonic	TAOK2	nonsynonymous SNV	[illegible]	16p11.2	.
16	30518075	T	A	CCAGCCTCTC,GTGGAGCTGA	exonic	ITGAL	nonsynonymous SNV	[illegible]	16p11.2	.
16	30532930	G	A	TGGGGATCCC,GCTGCCTGAA	exonic	ITGAL	nonsynonymous SNV	[illegible]	16p11.2	.
16	30536661	C	T	GAAGCTCTTC,CGCAGATGCC	exonic	ZNF768	nonsynonymous SNV	[illegible]	16p11.2	.
16	30536662	C	T	AAGCTCTTCC,GCAGATGCCA	exonic	ZNF768	nonsynonymous SNV	[illegible]	16p11.2	.
16	30997455	C	T	ATGTGGTCAT,CACACGGCTG	exonic	AC135048.1,HSD3B7	nonsynonymous SNV	[illegible]	16p11.2	.
16	31072503	C	G	CGCTGGCACT,AAGAAGGCCT	exonic	ZNF668	nonsynonymous SNV	[illegible]	16p11.2	.
16	31141474	C	T	GGCTACTTCT,CAAGGTGCTG	exonic	KAT8	nonsynonymous SNV	[illegible]	16p11.2	.
16	31154936	C	T	CAGTTCTCCT,CCTGGGCTCC	exonic	PRSS36	nonsynonymous SNV	[illegible]	16p11.2	.
16	31202307	C	A	GGATGATCGT,GTGGTGGCAG	exonic	FUS	nonsynonymous SNV	[illegible]	16p11.2	.
16	31332855	G	A	GAATGTATTT,AGTGTAATGA	exonic	ITGAM	nonsynonymous SNV	[illegible]	16p11.2	ITGAM;basechange=c.1909G>T;AAchange=p.E637*;tissue=endometrium
16	31519561	G	A	GAAGGCCCTG,AGAGCCCCCA	exonic	C16orf58	nonsynonymous SNV	[illegible]	16p11.2	.
16	46771704	C	T	AGGGCCAGGC,CTGGAGTCAG	exonic	MYLK3	nonsynonymous SNV	[illegible]	16q11.2	.
16	47732410	C	T	GCTAGAATTT,AAGACAAAGT	exonic	PHKB	stopgain	[illegible]	16q12.1	.
16	49672471	C	T	GCCTCGCACT,GTGGCAGTGA	exonic	ZNF423	nonsynonymous SNV	[illegible]	16q12.1	.
16	50112681	C	T	GGACATTATT,CATGCAAGAG	exonic	HEATR3	nonsynonymous SNV	[illegible]	16q12.1	.
16	54967069	A	T	GACGGAGGGC,GCCTCAGCGA	exonic	IRX5	nonsynonymous SNV	[illegible]	16q12.2	.
16	57761269	C	T	CTGACCATCT,CATCTATGAC	exonic	CCDC135	nonsynonymous SNV	[illegible]	16q21	.
16	66426109	G	A	ATCGACCTCC,ATACATGAGC	exonic	CDH5	nonsynonymous SNV	[illegible]	16q21	.
16	67320954	G	A	CCTGCGCTTC,AGATCTGGTT	exonic	PLEKHG4	nonsynonymous SNV	[illegible]	16q22.1	.
16	67580054	G	A	GCTCCCGAGG,CATTGAGCCC	exonic	FAM65A	nonsynonymous SNV	[illegible]	16q22.1	.
16	67645285	C	T	AGGGGAACTT,CACCCCAGGA	exonic	CTCF	nonsynonymous SNV	[illegible]	16q22.1	.
16	67650716	C	T	GGTTCGGCAT,GTCGTTACAA	exonic	CTCF	nonsynonymous SNV	[illegible]	16q22.1	.
16	68215394	G	A	GACCTCAGTG,GAGGTAGAAG	exonic	NFATC3	stopgain	[illegible]	16q22.1	.
16	68282535	T	C	AGTGATCCAC,CACCTCGGCC	exonic	PLA2G15	nonsynonymous SNV	[illegible]	16q22.1	.
16	70381820	C	T	CTCGAACTCC,GACCTCAAGT	exonic	DDX19A	stopgain	[illegible]	16q22.1	.
16	70698280	G	A	CGGGATGGTG,ATGACTTGTC	exonic	MTSS1L	nonsynonymous SNV	[illegible]	16q22.1	.
16	71417897	G	A	CTTGAACGGG,ATGGCAAATT	exonic	CALB2	nonsynonymous SNV	[illegible]	16q22.2	.
16	77775837	G	A	AAAGCTACAA,CAGGTTATGA	exonic	NUDT7	nonsynonymous SNV	[illegible]	16q23.1	.
16	77918616	C	T	GGGCCTCATT,GGGGAGTGGT	exonic	VAT1L	nonsynonymous SNV	[illegible]	16q23.1	.
16	81703820	G	A	CTCAACGCGG,GATGGAAGTG	exonic	CMIP	nonsynonymous SNV	[illegible]	16q23.3	.
16	84002455	G	T	CATCCTGGAC,TGAGTACGCG	splicing	NECAB2(ENST00000305202.4:exon1:c.201+1G>T)	.		16q23.3	.
16	84203638	G	A	GCCGCCTGTG,AGGCTAAAAG	exonic	DNAAF1	nonsynonymous SNV	[illegible]	16q24.1	.
16	84256179	A	G	ATGCTGGTGA,CTCCAGCACC	exonic	KCNG4	nonsynonymous SNV	[illegible]	16q24.1	.
16	88496316	G	A	GACCCCCTGA,GACAGGCTTC	exonic	ZNF469	nonsynonymous SNV	[illegible]	16q24.2	.
16	88498466	C	T	GACGTGTCCC,CTGAACGGAC	exonic	ZNF469	nonsynonymous SNV	[illegible]	16q24.2	ZNF469;basechange=c.4504C>T;AAchange=p.P1502S;tissue=kidney
16	88499582	A	T	GAGCCCCCCC,TACGTCAGCT	exonic	ZNF469	nonsynonymous SNV	[illegible]	16q24.2	.
16	88499583	T	C	AGCCCCCCCA,ACGTCAGCTC	exonic	ZNF469	nonsynonymous SNV	[illegible]	16q24.2	.
16	88505196	G	A	TTCCCCAGCC,GAGCCCTGCA	exonic	ZNF469	nonsynonymous SNV	[illegible]	16q24.2	.
16	88901629	G	A	ACCTTGTTCG,GGGCGGAAAT	exonic	GALNS	nonsynonymous SNV	[illegible]	16q24.3	.
16	89017688	C	T	TCACCCGTCC,CTGGACTGGC	exonic	RP11-830F9.6	nonsynonymous SNV	[illegible]	16q24.3	.
16	89965278	G	A	CCAGGAGTGG,AGGTGGGTGC	exonic	TCF25	nonsynonymous SNV	[illegible]	16q24.3	.
17	1628666	T	A	CTCACTCGCT,CATGCAGGAG	exonic	WDR81	nonsynonymous SNV	[illegible]	17p13.3	.
17	3715928	G	A	ACTTTTCTTG,GAAAAGGGTG	exonic	C17orf85	nonsynonymous SNV	[illegible]	17p13.2	.
17	4458561	G	A	GGCAGGCCGG,CGCCACTCTG	exonic	MYBBP1A	nonsynonymous SNV	[illegible]	17p13.2	.
17	4794408	G	A	GCCCCAGGAG,GACCGCACAA	exonic	MINK1	nonsynonymous SNV	[illegible]	17p13.2	.
17	4794409	G	A	CCCCAGGAGG,ACCGCACAAG	exonic	MINK1	nonsynonymous SNV	[illegible]	17p13.2	.
17	7415900	C	T	CGCCTGGTCC,CCAGTGTTGG	exonic	POLR2A	nonsynonymous SNV	[illegible]	17p13.1	.
17	9631414	G	A	GAAACCACCA,GTGCCTCCGT	exonic	USP43	nonsynonymous SNV	[illegible]	17p13.1	.
17	10543900	A	G	CCAAATTTGT,CTGAGTGTGG	exonic	MYH3	nonsynonymous SNV	[illegible]	17p13.1	.
17	15968217	G	A	TACGGCCTGG,AGGGAAAGTA	exonic	NCOR1	nonsynonymous SNV	[illegible]	17p12	.
17	28601155	G	A	TCTTTGTCTC,ATATTCCCAG	exonic	BLMH	stopgain	[illegible]	17q11.2	.
17	35921587	G	A	AGGTGGAACA,TATTTTCAGA	exonic	SYNRG	nonsynonymous SNV	[illegible]	17q12	.
17	36093793	C	T	ATTTCCAGAA,TCTGGACTGT	exonic	HNF1B	nonsynonymous SNV	[illegible]	17q12	.
17	37319059	C	T	AGAATGATCT,CTCCACGTTG	exonic	ARL5C	nonsynonymous SNV	[illegible]	17q12	.
17	40452816	C	T	CTGCCCATCC,CGGCCCAGTG	exonic	STAT5A	nonsynonymous SNV	[illegible]	17q21.2	.
17	43005563	G	A	ATGGCGGAAG,GCCCAGGGGC	exonic	KIF18B	nonsynonymous SNV	[illegible]	17q21.31	.
17	45362052	C	T	CTCGAAAACC,CTGCTATGAG	exonic	ITGB3	nonsynonymous SNV	[illegible]	17q21.32	.
17	62024421	C	T	GCCCTCGAAT,GGGACAGTGC	exonic	SCN4A	nonsynonymous SNV	[illegible]	17q23.3	.
17	63923713	C	T	TTGTTCATAC,GCTGTCTGAT	exonic	CEP112	nonsynonymous SNV	[illegible]	17q24.1	.
17	65900953	G	A	GTCGTTAGAA,GAAGTAAGTA	exonic	BPTF	nonsynonymous SNV	[illegible]	17q24.2	.
17	73043385	G	A	GGCGGGGCTG,GAGGGGGCGG	exonic	KCTD2	nonsynonymous SNV	[illegible]	17q25.1	.
17	76523005	G	A	GACCTCGTTG,CCTGGAGTGG	exonic	DNAH17	nonsynonymous SNV	[illegible]	17q25.3	.
17	79249803	C	T	CAGGGCCTGC,TGCATGGCAG	exonic	SLC38A10	nonsynonymous SNV	[illegible]	17q25.3	.
17	80207385	G	A	GCTGTGGAAG,GAGGCCGCGG	exonic	CSNK1D	nonsynonymous SNV	[illegible]	17q25.3	.
18	2931275	C	T	TCTCCATTTT,ACTGAGGCCC	exonic	LPIN2	nonsynonymous SNV	[illegible]	18p11.31	.
18	9887014	C	T	GGGTGACATC,CCAAGGCCCC	exonic	TXNDC2	nonsynonymous SNV	[illegible]	18p11.22	.
18	9887015	C	T	GGTGACATCC,CAAGGCCCCA	exonic	TXNDC2	nonsynonymous SNV	[illegible]	18p11.22	.
18	18562725	G	A	CTCACTTACC,AGAAATATTG	exonic	ROCK1	nonsynonymous SNV	[illegible]	18q11.1	.
18	19029543	G	A	TTTACTCTGA,AGAAAGAGCC	exonic	GREB1L	nonsynonymous SNV	[illegible]	18q11.2	.
18	21053410	A	T	GATGAAAAGG,AGATAGTAAA	exonic	RIOK3	nonsynonymous SNV	[illegible]	18q11.2	.
18	21355764	T	G	AGCCTGCAGC,GTGACCCTGA	exonic	LAMA3	nonsynonymous SNV	[illegible]	18q11.2	.
18	21355765	G	T	GCCTGCAGCT,TGACCCTGAG	exonic	LAMA3	nonsynonymous SNV	[illegible]	18q11.2	.
18	25565627	C	T	TCTGGAGTTT,GCAAGTCTCT	exonic	CDH2	nonsynonymous SNV	[illegible]	18q12.1	CDH2;basechange=c.1840G>A;AAchange=p.E614K;tissue=breast
18	29432455	G	A	TTTTTCAGAG,ACTGTGTGGC	exonic	TRAPPC8	nonsynonymous SNV	[illegible]	18q12.1	.
18	31324187	G	A	ACCACCAGGG,GCTTTGCACC	exonic	ASXL3	nonsynonymous SNV	[illegible]	18q12.1	.
18	33606907	G	A	TTTTGACAAC,AAGAAAATCT	exonic	RPRD1A	nonsynonymous SNV	[illegible]	18q12.2	.
18	40854218	C	T	AATATCAACT,CCTTAAGCAC	exonic	SYT4	nonsynonymous SNV	[illegible]	18q12.3	.
18	47318646	G	A	TCCTTTCTTT,TCTTCACTTC	exonic	ACAA2	nonsynonymous SNV	[illegible]	18q21.1	.
18	51025778	C	T	AGCTTGTGTT,GACCAACTCA	exonic	DCC	stopgain	[illegible]	18q21.2	DCC;basechange=c.4009C>T;AAchange=p.R1337*;tissue=large_intestine
18	56203728	C	T	TTGCCTGCCT,CCAATTTCCA	exonic	ALPK2	nonsynonymous SNV	[illegible]	18q21.32	.
18	56939795	C	T	GACGGGCAGC,CTGGGCTCGG	exonic	RAX	nonsynonymous SNV	[illegible]	18q21.32	.
18	59221917	C	T	GGGACCCGCG,CGCTGTGGTG	exonic	CDH20	nonsynonymous SNV	[illegible]	18q21.33	.
18	60582234	C	A	ATGTGAACTT,CTGCCCGGTG	exonic	PHLPP1	nonsynonymous SNV	[illegible]	18q21.33	.
18	63430284	G	A	CTCTATGTAG,AAAGGTAGGG	exonic	CDH7	nonsynonymous SNV	[illegible]	18q22.1	.
18	66504350	G	A	AACAGTGCCA,GGAGGAAGGA	exonic	CCDC102B	nonsynonymous SNV	[illegible]	18q22.1	CCDC102B;basechange=c.350G>T;AAchange=p.R117M;tissue=lung
18	70451089	C	T	CACAAAATTC,TCTTGCACTC	exonic	NETO1	nonsynonymous SNV	[illegible]	18q22.3	.
18	77440203	G	A	GCTGAGGTCG,AGCGCGCGGG	exonic	CTDP1	nonsynonymous SNV	[illegible]	18q23	.
19	746504	C	T	GAGGCCACGT,CGGCCCGCCG	exonic	PALM	nonsynonymous SNV	[illegible]	19p13.3	.
19	757289	G	A	CCGCCCAGAG,ACGGGGAGGA	exonic	MISP	nonsynonymous SNV	[illegible]	19p13.3	.
19	1009246	G	A	CGGGCGCGCC,GGCCGTGGAC	exonic	GRIN3B	nonsynonymous SNV	[illegible]	19p13.3	.
19	1220715	C	T	TGGGGTCACC,TGTAAGTGCC	exonic	STK11	nonsynonymous SNV	[illegible]	19p13.3	STK11;basechange=c.733C>T;AAchange=p.L245F;tissue=lung
19	2114270	G	A	CTGTGTTTCT,AATAGGCAGT	exonic	AP3D1	stopgain	[illegible]	19p13.3	.
19	2980180	G	A	CAGTTCCCCA,GTGAGAGCAA	exonic	TLE6	nonsynonymous SNV	[illegible]	19p13.3	.
19	3251010	G	A	TACTGTGCCA,GGATTCCGCC	exonic	CELF5	nonsynonymous SNV	[illegible]	19p13.3	.
19	3595721	G	A	AGCTGGGGCT,GAGGGACAGC	exonic	TBXA2R	stopgain	[illegible]	19p13.3	.
19	3651833	G	A	CGTGTCATCC,ATTCGATGGC	exonic	PIP5K1C	nonsynonymous SNV	[illegible]	19p13.3	.
19	4217490	G	A	GGGGCCAGCG,GGGCGGTGGC	exonic	ANKRD24	nonsynonymous SNV	[illegible]	19p13.3	.
19	4652404	G	A	CAGGATCTGC,AGGGCCTGGG	exonic	TNFAIP8L1	nonsynonymous SNV	[illegible]	19p13.3	.
19	5976532	A	G	CGAGGCGGGC,GATCACGAGG	exonic	RANBP3	nonsynonymous SNV	[illegible]	19p13.3	.
19	7527157	C	T	AGGCCTATTC,GTGGAGGGGA	exonic	ARHGEF18,CTD-2207O23.3	nonsynonymous SNV	[illegible]	19p13.2	.
19	8384211	G	A	TTGGGAGGCC,AGGCAGGCGG	exonic	NDUFA7	nonsynonymous SNV	[illegible]	19p13.2	.
19	8555553	G	A	TCCCCGCGCC,GATCCCGAGG	exonic	PRAM1	nonsynonymous SNV	[illegible]	19p13.2	.
19	8662126	C	T	ACCTGGTCCT,CGTGAGCAGG	exonic	ADAMTS10	nonsynonymous SNV	[illegible]	19p13.2	.
19	8841976	G	A	CTGTGCCTAC,AGATGGCGCT	exonic	OR2Z1	nonsynonymous SNV	[illegible]	19p13.2	.
19	9047463	C	T	GTTGGAAAAT,TGAAGTGGCT	exonic	MUC16	nonsynonymous SNV	[illegible]	19p13.2	.
19	9048441	G	A	AGTTCACCAG,AGAAAGAATC	exonic	MUC16	nonsynonymous SNV	[illegible]	19p13.2	.
19	9063952	C	A	TCCCTCAATC,AGGGGTCAGG	exonic	MUC16	stopgain	[illegible]	19p13.2	.
19	9074436	G	A	GACATGAGTA,TAGCATCACC	exonic	MUC16	nonsynonymous SNV	[illegible]	19p13.2	.
19	9452137	T	A	TCAGCTAAGC,CTATTTAGAA	exonic	ZNF559	nonsynonymous SNV	[illegible]	19p13.2	.
19	9452138	C	T	CAGCTAAGCT,TATTTAGAAG	exonic	ZNF559	nonsynonymous SNV	[illegible]	19p13.2	.
19	10405140	C	T	GGGGCTGAGG,TTCCGCGCTG	exonic	ICAM5	nonsynonymous SNV	[illegible]	19p13.2	.
19	10426662	G	A	CCGGGCCATG,AGGCGGCCAT	exonic	CTD-2369P2.10,FDX1L	nonsynonymous SNV	[illegible]	19p13.2	FDX1L;basechange=c.11C>T;AAchange=p.S4F;tissue=NS
19	10746126	G	A	CACTTCCAAC,AAGCGGTCTA	exonic	SLC44A2	nonsynonymous SNV	[illegible]	19p13.2	SLC44A2;basechange=c.1168G>A;AAchange=p.E390K;tissue=upper_aerodigestive_tract
19	11289087	C	T	CTCCTCTTTC,GTTTCATGAT	exonic	KANK2	nonsynonymous SNV	[illegible]	19p13.2	.
19	11453782	G	A	TAAGGCTCAG,AACAGCAGGT	exonic	TMEM205	nonsynonymous SNV	[illegible]	19p13.2	.
19	11489027	C	T	GCCACCAGGC,CTGGGAGGTC	exonic	EPOR	nonsynonymous SNV	[illegible]	19p13.2	.
19	12541677	G	C	TCATGTCTTT,AAATACACTG	exonic	ZNF443	nonsynonymous SNV	[illegible]	19p13.2	.
19	14674800	C	T	TCAGGTCTTC,TAACAGAGTA	exonic	TECR	nonsynonymous SNV	[illegible]	19p13.12	.
19	14710852	G	A	GACTGCCGCC,AATTACCTGT	exonic	CLEC17A	nonsynonymous SNV	[illegible]	19p13.12	.
19	14951992	C	T	CTTATACTTC,CCTGAGCTGA	exonic	OR7A10	nonsynonymous SNV	[illegible]	19p13.12	.
19	16860429	C	T	TGGGCAGCAG,TCAGGCACGA	exonic	NWD1	nonsynonymous SNV	[illegible]	19p13.11	.
19	17085945	G	A	ATGCCCCAAG,CCACGGGAAG	exonic	CPAMD8	nonsynonymous SNV	[illegible]	19p13.11	.
19	17412086	G	A	AGGGCGGCCG,CGGCTCCCCA	exonic	ABHD8	nonsynonymous SNV	[illegible]	19p13.11	.
19	17746890	G	A	CACTATGAGG,TAATCAGCTT	exonic	UNC13A	nonsynonymous SNV	[illegible]	19p13.11	.
19	17837689	C	T	CAGGAGCGCC,TGGGGTGGCC	exonic	MAP1S	nonsynonymous SNV	[illegible]	19p13.11	.
19	19790253	C	T	GCCTCCATTT,CCCCAGTAGT	exonic	ZNF101	nonsynonymous SNV	[illegible]	19p13.11	.
19	19902508	A	C	GTTGGCCAGG,TGGTCTTGAA	exonic	ZNF506	nonsynonymous SNV	[illegible]	19p13.11	.
19	21719699	G	A	TCATTCTGGA,AGAAGCCCTA	exonic	ZNF429	nonsynonymous SNV	[illegible]	19p12	.
19	21910240	C	T	CCACATTCTT,ACATCTGTAT	exonic	ZNF100	nonsynonymous SNV	[illegible]	19p12	.
19	22499079	C	A	CCTTATGAAG,ATAAGATAAT	exonic	ZNF729	nonsynonymous SNV	[illegible]	19p12	.
19	22697255	G	A	CTCACCATAG,GCTCCACATG	exonic	ZNF98	nonsynonymous SNV	[illegible]	19p12	.
19	22939380	G	A	TGTTTAGTAA,GTGTGAGGAT	exonic	ZNF99	nonsynonymous SNV	[illegible]	19p12	.
19	23710769	C	T	TGATGCATTT,ATTGCTTCTT	exonic	ZNF675	nonsynonymous SNV	[illegible]	19p12	.
19	23710817	C	T	CCCACAGATC,TGGCAGACAG	splicing	ZNF675(ENST00000601935.1:exon5:c.227-1G>A)	.		19p12	.
19	35434688	C	T	AGTACCTTTT,ATACCTGGTT	exonic	ZNF30	nonsynonymous SNV	[illegible]	19q13.11	.
19	35501318	C	T	CCACGCGGTC,CCCGCTCAGC	exonic	GRAMD1A	nonsynonymous SNV	[illegible]	19q13.12	.
19	35837090	G	A	GACCTCCCCC,GACTGCGATG	exonic	CD22	nonsynonymous SNV	[illegible]	19q13.12	CD22;basechange=c.2364G>A;AAchange=p.P788P;tissue=pancreas
19	35993773	G	A	CGGGTGCTGG,GGGCGGGACC	exonic	DMKN	nonsynonymous SNV	[illegible]	19q13.12	.
19	35994328	G	A	GGAGGTCACG,GATGCGAGAG	exonic	DMKN	nonsynonymous SNV	[illegible]	19q13.12	.
19	36509847	C	T	GAAGTCCATC,GGGGGGTCCG	exonic	CLIP3	nonsynonymous SNV	[illegible]	19q13.12	.
19	37487911	C	T	ACACCTTTTT,GACATCAAAG	exonic	ZNF568	stopgain	[illegible]	19q13.12	.
19	37905169	G	A	TGTCTGGAAG,GAAAAAATCA	exonic	ZNF569	nonsynonymous SNV	[illegible]	19q13.12	.
19	38873350	C	T	AGTTCGAGAC,AGCCTGGTCA	exonic	PSMD8	nonsynonymous SNV	[illegible]	19q13.2	.
19	40900054	G	A	CTCTCTGACG,GGGACTTGGG	exonic	PRX	nonsynonymous SNV	[illegible]	19q13.2	.
19	40978512	A	T	CCCTATGTCC,GGCCTCACCT	splicing	SPTBN4	.		19q13.2	.
19	41727125	C	T	ACCTTCGTGT,CCAGCCTGGC	exonic	AXL	nonsynonymous SNV	[illegible]	19q13.2	.
19	42595456	T	A	CCTCCAGGGG,CTGTGCTGCC	exonic	POU2F2	nonsynonymous SNV	[illegible]	19q13.2	.
19	42867401	G	A	GTTTGGAGCA,CAGCCTGCAG	exonic	MEGF8	nonsynonymous SNV	[illegible]	19q13.2	.
19	43411959	C	T	TCCTTCTTCT,CCTGGGGTTT	exonic	PSG6	nonsynonymous SNV	[illegible]	19q13.31	.
19	44635518	C	T	TTATGTTCAT,GTAAATTACA	exonic	ZNF225	nonsynonymous SNV	[illegible]	19q13.31	.
19	44635775	G	A	GTCATCGCAT,GTCCACACAG	exonic	ZNF225	nonsynonymous SNV	[illegible]	19q13.31	.
19	44635776	G	A	TCATCGCATG,TCCACACAGG	exonic	ZNF225	nonsynonymous SNV	[illegible]	19q13.31	.
19	44778464	C	T	GAGTTCGCAT,TCCAAGACCA	exonic	ZNF233	nonsynonymous SNV	[illegible]	19q13.31	.
19	45515239	G	A	GGCCTGGACG,GGGACAGCCG	exonic	RELB	nonsynonymous SNV	[illegible]	19q13.32	.
19	46256972	C	T	CCCTCCAGCT,CCCAAGCTCT	exonic	AC074212.3	nonsynonymous SNV	[illegible]	19q13.32	.
19	46832662	C	T	GGGGAGTGAC,CCCGGCTGAG	exonic	HIF3A	nonsynonymous SNV	[illegible]	19q13.32	.
19	47023111	C	T	GTGGTGCATG,CTTCGGTCAC	exonic	PPP5D1	nonsynonymous SNV	[illegible]	19q13.32	.
19	47763981	C	T	GAGGGCAGCC,CGGGGAGCAG	exonic	CCDC9	nonsynonymous SNV	[illegible]	19q13.32	.
19	47944675	C	T	TCGTCATCAA,TATCTTCACC	exonic	SLC8A2	nonsynonymous SNV	[illegible]	19q13.32	.
19	48342995	C	T	TACCTTTCTC,CATGGTGCCC	exonic	CRX	nonsynonymous SNV	[illegible]	19q13.33	.
19	48364171	C	T	CCCCTAGTCC,TGTAGCCGCT	exonic	TPRX2P	nonsynonymous SNV	[illegible]	19q13.33	.
19	48892818	G	A	ACCTCCAGAG,GGTGAAGTCA	exonic	KDELR1	nonsynonymous SNV	[illegible]	19q13.33	.
19	49102448	C	T	AGCCTTCGAT,GTGCCTACCG	exonic	SULT2B1	nonsynonymous SNV	[illegible]	19q13.33	.
19	49253932	G	A	CGGCCCAGGC,GAGCTGACCC	exonic	FUT1	nonsynonymous SNV	[illegible]	19q13.33	.
19	49303004	G	A	GAGGGAGACC,GGGTCACGGA	exonic	BCAT2	nonsynonymous SNV	[illegible]	19q13.33	.
19	49442932	C	T	GAGAAGATTT,TGTCGTGGGA	exonic	DHDH	nonsynonymous SNV	[illegible]	19q13.33	.
19	51519399	G	A	TCCCCTACTC,AGCCCACAGT	exonic	KLK10	nonsynonymous SNV	[illegible]	19q13.41	.
19	51647926	C	T	CAGGACCATC,AACTCAATGT	exonic	SIGLEC7	stopgain	[illegible]	19q13.41	.
19	51885767	G	A	GATGATGCCG,AGATGGCGCA	exonic	LIM2	nonsynonymous SNV	[illegible]	19q13.41	.
19	52129300	C	T	TGATGGTACC,ATAATGGGGT	exonic	SIGLEC5	nonsynonymous SNV	[illegible]	19q13.41	.
19	52327570	G	A	GCTGTAGAGA,GTTGAACGTG	exonic	FPR3	nonsynonymous SNV	[illegible]	19q13.41	.
19	52394946	G	A	TTCAATTTCC,TAGGCATTTG	exonic	ZNF649	nonsynonymous SNV	[illegible]	19q13.41	.
19	52448172	T	G	ACCCTATGAA,GCACTGAATG	exonic	ZNF613	nonsynonymous SNV	[illegible]	19q13.41	.
19	53572724	T	C	GTAGTTAGGT,TGAATGTCCT	exonic	ZNF160	nonsynonymous SNV	[illegible]	19q13.41	.
19	53645099	G	A	TGTGAATTTC,ACTAAAGACC	exonic	ZNF347	nonsynonymous SNV	[illegible]	19q13.42	.
19	53740775	G	A	ACATATGTAA,GCTTCTCTCC	exonic	ZNF677	nonsynonymous SNV	[illegible]	19q13.42	.
19	54600415	C	T	CGGCTGAGCT,CCAGCCATGG	exonic	OSCAR	nonsynonymous SNV	[illegible]	19q13.42	.
19	54609345	C	T	CAGGGATCCC,GGAATAGGCC	exonic	NDUFA3	nonsynonymous SNV	[illegible]	19q13.42	.
19	54691138	A	C	AGATAGGAGA,AGTCCAGGCC	exonic	MBOAT7	nonsynonymous SNV	[illegible]	19q13.42	.
19	54802106	C	T	ATCAGCTGCC,CCTCCTTGGT	exonic	LILRA3	nonsynonymous SNV	[illegible]	19q13.42	.
19	54802107	C	T	TCAGCTGCCC,CTCCTTGGTC	exonic	LILRA3	nonsynonymous SNV	[illegible]	19q13.42	.
19	54867856	A	G	CCTCTCTAGA,CATCAACAGC	exonic	LAIR1	nonsynonymous SNV	[illegible]	19q13.42	.
19	54976568	C	T	GGGGGGCGGC,CGCCGCCGTG	exonic	CDC42EP5	nonsynonymous SNV	[illegible]	19q13.42	.
19	54976569	C	T	GGGGGCGGCC,GCCGCCGTGG	exonic	CDC42EP5	nonsynonymous SNV	[illegible]	19q13.42	.
19	55019154	C	T	ACTGTGATCT,CCCGGGGAGC	exonic	LAIR2	nonsynonymous SNV	[illegible]	19q13.42	.
19	55086975	C	T	AACCTCTCCT,CGAGTGGTCG	exonic	LILRA2	nonsynonymous SNV	[illegible]	19q13.42	.
19	55106832	C	T	CATGTGTGGT,TCTACCCAGT	exonic	LILRA1	nonsynonymous SNV	[illegible]	19q13.42	.
19	55107662	C	A	AGGACAGTTC,GTGGCAGACC	exonic	LILRA1	nonsynonymous SNV	[illegible]	19q13.42	.
19	55175241	G	C	CCTCTGGGCT,AGCCAGGCTC	exonic	LILRB4	nonsynonymous SNV	[illegible]	19q13.42	.
19	55316301	G	A	GGTGCCTCAA,GAGGACACGT	exonic	KIR2DL4	nonsynonymous SNV	[illegible]	19q13.42	.
19	55450818	C	A	GAGACTCTGT,CTGGCGGAGG	exonic	NLRP7	nonsynonymous SNV	[illegible]	19q13.42	.
19	55526389	C	T	CGGAAGCGGC,TCTGCACAGC	exonic	GP6	nonsynonymous SNV	[illegible]	19q13.42	.
19	56249613	G	A	CTCAGCCCAG,GGATTGGCTT	exonic	NLRP9	nonsynonymous SNV	[illegible]	19q13.42	.
19	57058941	C	T	TGGCTGAACC,CATTCAGAGG	exonic	ZFP28	nonsynonymous SNV	[illegible]	19q13.43	.
19	57065112	G	A	ATATGCTGAA,GGGTAACAGA	exonic	ZFP28	nonsynonymous SNV	[illegible]	19q13.43	.
19	57065791	C	T	AGGTATGGTT,CTCCCTTACT	exonic	ZFP28	nonsynonymous SNV	[illegible]	19q13.43	.
19	57335671	A	T	CAGCAGAGTG,CGAGCTTCTC	exonic	PEG3	nonsynonymous SNV	[illegible]	19q13.43	.
19	57335672	C	T	AGCAGAGTGA,GAGCTTCTCA	exonic	PEG3	nonsynonymous SNV	[illegible]	19q13.43	.
19	57647256	C	T	TTTTCTGTAT,CATTATCATC	exonic	ZIM3	nonsynonymous SNV	[illegible]	19q13.43	.
19	57840476	C	T	CGCGGCTCAT,CCTCACACAT	exonic	ZNF543	nonsynonymous SNV	[illegible]	19q13.43	.
19	58087261	G	A	TTCTTCCTGG,AGAAGTAAAT	exonic	ZNF416	nonsynonymous SNV	[illegible]	19q13.43	.
19	58596191	T	C	GCTTTTCTCC,TCTCGTGGGT	exonic	ZSCAN18	nonsynonymous SNV	[illegible]	19q13.43	.
20	1143811	G	A	CGGGGGAGAA,ACTTAGACCC	exonic	PSMF1	nonsynonymous SNV	[illegible]	20p13	.
20	5843759	G	A	GTCAGAGAAG,AAGAGGATGA	exonic	C20orf196	nonsynonymous SNV	[illegible]	20p12.3	.
20	6759096	C	T	ACAGCCAACT,GAAATTCCCC	exonic	BMP2	nonsynonymous SNV	[illegible]	20p12.3	.
20	10626030	C	T	TCCTTTCCAC,CATTTTTACA	exonic	JAG1	nonsynonymous SNV	[illegible]	20p12.2	.
20	10626031	C	T	CCTTTCCACC,ATTTTTACAG	exonic	JAG1	nonsynonymous SNV	[illegible]	20p12.2	.
20	17208110	G	A	AGCAGAACAC,GCTTTGGAGT	exonic	PCSK2	nonsynonymous SNV	[illegible]	20p12.1	.
20	17641058	G	A	TTCCTTCATG,AGAAAGTCGA	exonic	RRBP1	nonsynonymous SNV	[illegible]	20p12.1	.
20	31035611	C	T	ATGCTGAGGT,CGTGGGCCCT	exonic	C20orf112	nonsynonymous SNV	[illegible]	20q11.21	.
20	33330872	G	A	CTGGGAGTCG,GGTTCAGGGG	exonic	NCOA6	nonsynonymous SNV	[illegible]	20q11.22	NCOA6;basechange=c.3188C>T;AAchange=p.P1063L;tissue=lung
20	35802520	C	T	ACTACAGATC,TAGGAATAAA	exonic;splicing	MROH8;MROH8(ENST00000343811.4:exon3:c.338-1G>A,ENST00000441008.2:exon6:c.216-1G>A,ENST00000400441.3:exon4:c.258-1G>A,ENST00000400440.2:exon3:c.350-1G>A,ENST00000421643.1:exon3:c.367-1G>A)	nonsynonymous SNV	[illegible]	20q11.23	.
20	42682943	C	T	GGGCAGATCT,GGGAGAAAAG	exonic	TOX2	nonsynonymous SNV	[illegible]	20q13.12	.
20	43031193	G	A	TCTCTGAGCA,ATTTTGTTGC	splicing	HNF4A(ENST00000372920.1:exon2:c.116-1G>A)	.		20q13.12	.
20	43246928	G	A	CTCTCCAGAA,GACAGGTGGA	exonic	PKIG	nonsynonymous SNV	[illegible]	20q13.12	.
20	43752573	C	T	ACATCTTCAT,CTTGTTTCCT	exonic	WFDC12	nonsynonymous SNV	[illegible]	20q13.12	.

20	43836780	G	T	CAGCATGGCC,AAAGGCAAAT	exonic	SEMG1	nonsynonymous SNV	[illegible]	20q13.12	.
20	43850733	G	A	ATCTGGAAAG,GATTATCCAG	exonic	SEMG2	nonsynonymous SNV	[illegible]	20q13.12	.
20	44838970	G	A	GGCGGCGTCG,GGTCCCGCGC	exonic	CDH22	nonsynonymous SNV	[illegible]	20q13.12	.
20	47266698	G	A	CTCCAGGGGG,CTTGTTTGAA	exonic	PREX1	nonsynonymous SNV	[illegible]	20q13.13	.
20	50769778	G	A	GTCGCAATGA,GACACTTGAA	exonic	ZFP64	nonsynonymous SNV	[illegible]	20q13.2	.
20	57766789	G	A	GGGGATGCAC,AAGGCGCCTC	exonic	ZNF831	nonsynonymous SNV	[illegible]	20q13.32	.
20	58180139	C	T	CCCCAGCGTC,TCACCGACTC	exonic	PHACTR3	nonsynonymous SNV	[illegible]	20q13.32	.
20	58519369	C	T	GAAATTGATC,AGTTTACTTT	exonic	FAM217B	nonsynonymous SNV	[illegible]	20q13.33	.
20	60888501	G	A	CCGGGGAAGC,AAGCAGGGGA	exonic	LAMA5	nonsynonymous SNV	[illegible]	20q13.33	.
20	61430837	G	A	TTTGGGGAAA,CATCAGAAAA	exonic	MRGBP	nonsynonymous SNV	[illegible]	20q13.33	C20orf20;basechange=c.457G>T;AAchange=p.A153S;tissue=endometrium
20	62321725	C	T	GTCGCCTGGC,CCTTCTTCTC	exonic	RTEL1,RTEL1-TNFRSF6B	nonsynonymous SNV	[illegible]	20q13.33	.
20	62371854	C	T	CCCCGGACTC,GTCGGCGCAC	exonic	SLC2A4RG	nonsynonymous SNV	[illegible]	20q13.33	.
20	62680686	G	A	CCCGGCTCGG,GCTGCGCGGG	exonic	SOX18	nonsynonymous SNV	[illegible]	20q13.33	.
21	18976514	G	A	GTAATCCAGC,CTTTGGGAGG	exonic	BTG3	nonsynonymous SNV	[illegible]	21q21.1	.
21	27066082	C	T	TTTTAAAAAT,GAGCTGAGAT	exonic	JAM2	stopgain	[illegible]	21q21.3	.
21	28296683	C	T	ATTAGAATTT,CTTCGTGGCA	exonic	ADAMTS5	nonsynonymous SNV	[illegible]	21q21.3	.
21	28338088	C	T	GGTTTCGCAG,TGGCGCGCGG	exonic	ADAMTS5	nonsynonymous SNV	[illegible]	21q21.3	.
21	31965011	G	A	CTATGGCTGT,GCTATGGCTA	exonic	KRTAP6-3	nonsynonymous SNV	[illegible]	21q22.11	KRTAP6-3;basechange=c.226G>A;AAchange=p.G76S;tissue=breast
21	32638597	G	A	GTCACCCAAG,AATTGGCTCT	exonic	TIAM1	nonsynonymous SNV	[illegible]	21q22.11	.
21	35124139	C	T	AAGAGTTCTT,CTTTAGTAGA	exonic	ITSN1	nonsynonymous SNV	[illegible]	21q22.11	.
21	38117209	G	A	CTTCCCTCTG,ACTCTCACGT	exonic	SIM2	nonsynonymous SNV	[illegible]	21q22.13	.
21	38564476	G	A	ACTTTCTAAA,TGCAGATTTC	exonic	TTC3	nonsynonymous SNV	[illegible]	21q22.13	.
21	40568255	A	C	ATGGTATCTC,CAGTCCTTCT	exonic	BRWD1	nonsynonymous SNV	[illegible]	21q22.2	.
21	40571196	C	T	TCTCTGTTTT,AGATTCATCA	exonic	BRWD1	nonsynonymous SNV	[illegible]	21q22.2	.
21	43258151	G	A	AGGGACCTCG,TAATGATCTC	exonic	PRDM15	nonsynonymous SNV	[illegible]	21q22.3	.
21	43913189	C	T	CCCTCATATT,CTGGGTAATG	exonic	RSPH1	nonsynonymous SNV	[illegible]	21q22.3	.
21	45537793	G	A	TTTGCTTCTG,AATCTTCCAT	exonic	PWP2	nonsynonymous SNV	[illegible]	21q22.3	.
21	46000169	G	A	GCAGCACACA,GCTTGCAGCA	exonic	KRTAP10-5	nonsynonymous SNV	[illegible]	21q22.3	.
21	46101761	G	A	GCAGCAGGAA,GGGTGCTGCA	exonic	KRTAP12-1	nonsynonymous SNV	[illegible]	21q22.3	.
21	46314976	C	T	CGGTGAGTTT,TGTTGGGCAA	splicing	ITGB2(ENST00000397852.1:exon9:c.994-1G>A,ENST00000397857.1:exon10:c.994-1G>A,ENST00000397854.3:exon9:c.823-1G>A,ENST00000355153.4:exon10:c.994-1G>A,ENST00000397850.2:exon11:c.994-1G>A,ENST00000523323.1:exon10:c.1037-1G>A,ENST00000302347.5:exon10:c.994-1G>A)	.	.	21q22.3	.
21	47532369	G	A	GAACCTGAAG,AGCAGGGCCT	exonic	COL6A2	nonsynonymous SNV	[illegible]	21q22.3	.
21	47544598	C	T	GCCAGGCCCT,GGGGGCCAAG	exonic	COL6A2	nonsynonymous SNV	[illegible]	21q22.3	.
21	47625828	A	C	GGTGCACTCC,CATAGGTGTA	exonic	LSS	nonsynonymous SNV	[illegible]	21q22.3	.
21	47686986	G	A	ACCATCCAGG,GAAAGATGGT	exonic	MCM3AP	nonsynonymous SNV	[illegible]	21q22.3	.
22	17451003	C	T	ACTGTATCCA,TGTGCTGGGC	exonic	GAB4	nonsynonymous SNV	[illegible]	22q11.1	.
22	18368805	G	A	CTGTGGCCCC,AGGAGCTTCC	exonic	MICAL3	nonsynonymous SNV	[illegible]	22q11.21	.
22	20127024	G	A	GGACAAGGAG,ACGACTTCCG	exonic	ZDHHC8	nonsynonymous SNV	[illegible]	22q11.21	.
22	21377844	C	T	GACCTGGACA,CGGGGACTCT	exonic	P2RX6	nonsynonymous SNV	[illegible]	22q11.21	.
22	22724297	C	T	TGGCCAAGCC,CCAGGACACT	exonic	IGLV7-46	nonsynonymous SNV	[illegible]	22q11.22	.
22	22869629	C	T	ATCTGTCGAT,GCCCCTCAGA	exonic	ZNF280A	nonsynonymous SNV	[illegible]	22q11.22	.
22	23154303	C	T	TCTCCTGCTC,CCCTCCTCAC	exonic	IGLV3-10	nonsynonymous SNV	[illegible]	22q11.22	.
22	24180592	C	T	GCCGTGCGGC,GCGGAAGGAG	exonic	DERL3	nonsynonymous SNV	[illegible]	22q11.23	.
22	26317230	C	T	TCAGATTGGC,ATCGGGACTT	exonic	MYO18B	nonsynonymous SNV	[illegible]	22q12.1	.
22	26317308	C	T	CGTGCAGCTC,TTCTGGGCAC	exonic	MYO18B	nonsynonymous SNV	[illegible]	22q12.1	.
22	26860215	G	A	CTTCTGGGGA,AGGGTCTGCT	exonic	HPS4	nonsynonymous SNV	[illegible]	22q12.1	.
22	28193353	G	A	AGAGCTGGAC,ACACCTCGTC	exonic	MN1	nonsynonymous SNV	[illegible]	22q12.1	.
22	28692256	C	T	CCGGTAATTT,CTTTGGAGAA	exonic	TTC28	nonsynonymous SNV	[illegible]	22q12.1	.
22	29446766	C	T	ACCCCACGGC,CCACAGGGGC	exonic	ZNRF3	nonsynonymous SNV	[illegible]	22q12.1	.
22	29622514	G	A	AGAGCTGACA,AGCGGCTGAA	exonic	EMID1	nonsynonymous SNV	[illegible]	22q12.2	.
22	32193616	A	T	GAAGAGAAGA,TGGACTTCAC	exonic	DEPDC5	nonsynonymous SNV	[illegible]	22q12.2	.
22	35480391	C	T	GTTCCAGAAT,AGCGAGCCAA	exonic	ISX	stopgain	[illegible]	22q12.3	.
22	35796489	C	T	GGGCGACGCC,AGGCCGACGA	exonic	MCM5	stopgain	[illegible]	22q12.3	.
22	37603329	C	T	ACCACCACGG,TGAGGCCACC	exonic	SSTR3	nonsynonymous SNV	[illegible]	22q13.1	.
22	38379623	C	T	TCCTGCTGCT,CTTCTTGACC	exonic	SOX10	nonsynonymous SNV	[illegible]	22q13.1	.
22	38621599	C	T	CCACTGGTGA,GCTGCGGGAC	exonic	TMEM184B	nonsynonymous SNV	[illegible]	22q13.1	.
22	39477097	G	A	CCTGGCCGAG,ACCCGAAGGT	exonic	APOBEC3G	nonsynonymous SNV	[illegible]	22q13.1	.
22	40082043	C	T	CACCACGACT,CATGGACCCC	exonic	CACNA1I	nonsynonymous SNV	[illegible]	22q13.1	.
22	41546029	C	T	ACATCCCCCT,CAAGGCAGAC	exonic	EP300	nonsynonymous SNV	[illegible]	22q13.2	.
22	42139121	G	A	GCAGTATGGG,AACTGCAGGC	exonic	MEI1	nonsynonymous SNV	[illegible]	22q13.2	.
22	45603578	G	A	CACTGACTCG,GCTCCGAGGG	exonic	KIAA0930	nonsynonymous SNV	[illegible]	22q13.31	.
22	46219073	C	T	TGTCCCGTCT,CCTCACTGCC	exonic	ATXN10	nonsynonymous SNV	[illegible]	22q13.31	.
22	46653465	T	G	TTAAAAGTTG,TAATTCCAAA	exonic	PKDREJ	nonsynonymous SNV	[illegible]	22q13.31	PKDREJ;basechange=c.5755A>G;AAchange=p.T1919A;tissue=breast
22	50471736	C	T	GAACCACTGT,TGATGTCGAA	exonic	TTLL8	nonsynonymous SNV	[illegible]	22q13.33	.
22	50616384	G	A	CGCCGAGCCC,ACGGCGCCGC	exonic	PANX2	nonsynonymous SNV	[illegible]	22q13.33	.
22	50728200	G	A	GCAGCGGCGT,GATGTCGGGG	exonic	PLXNB2	nonsynonymous SNV	[illegible]	22q13.33	.
22	50845153	A	T	CTGACTTGTG,TGTGCCGCAG	exonic	PPP6R2	nonsynonymous SNV	[illegible]	22q13.33	.
22	51009441	G	A	CTTCTTAGCA,CCTTCTGGAA	exonic	CPT1B	nonsynonymous SNV	[illegible]	22q13.33	.
22	51169505	C	T	CCCGGCGCCC,CGGCCCACGC	exonic	SHANK3	nonsynonymous SNV	[illegible]	22q13.33	.
X	10181765	G	T	CATCATGTTT,AATTAACCGG	exonic	CLCN4	stopgain	[illegible]	Xp22.2	.
X	15339830	G	A	GCCGCAGTGA,AAATAAGTCT	exonic	PIGA	nonsynonymous SNV	[illegible]	Xp22.2	.
X	15819612	A	C	CCTCGGCCTC,CAAAGTGCTG	exonic	ZRSR2	nonsynonymous SNV	[illegible]	Xp22.2	.
X	16867383	C	T	TTCTGGAGGC,CATCTTCTGC	exonic	RBBP7	nonsynonymous SNV	[illegible]	Xp22.2	.
X	18192262	C	T	TGGAGTTATT,ATTGGCTGAA	exonic	BEND2	nonsynonymous SNV	[illegible]	Xp22.13	.
X	18664169	C	T	CATTCTGGAC,CCAAGATAGA	exonic	CDKL5	nonsynonymous SNV	[illegible]	Xp22.13	.
X	19372660	G	A	TGGAAAAGAT,AGGTCTGCCT	exonic	PDHA1	nonsynonymous SNV	[illegible]	Xp22.12	.
X	21674916	C	T	TGGGTGAGCT,CCACTCCTCC	exonic	KLHL34	nonsynonymous SNV	[illegible]	Xp22.12	.
X	23019055	G	A	AGAGAGCAAA,GAATGGGCCT	exonic	DDX53	nonsynonymous SNV	[illegible]	Xp22.11	.
X	30260394	G	A	TGTTTTGAGG,ATACTGCCTC	exonic	MAGEB4	nonsynonymous SNV	[illegible]	Xp21.2	MAGEB4;basechange=c.142G>A;AAchange=p.D48N;tissue=lung
X	30260533	C	T	AATGCAAGTT,CTCCCAGGCC	exonic	MAGEB4	nonsynonymous SNV	[illegible]	Xp21.2	.
X	31227743	C	T	GCAGGATATC,ATGGGCTGGT	exonic	DMD	nonsynonymous SNV	[illegible]	Xp21.2	.
X	32398737	C	T	ACATTCATTT,CTTTCGCATC	exonic	DMD	nonsynonymous SNV	[illegible]	Xp21.1	.
X	36328958	G	A	TTTACAGAAT,GAAATTCTAC	exonic	CXorf30	stopgain	[illegible]	Xp21.1	.
X	37027654	C	T	ACCTCCTCTC,GCCCAGAGAC	exonic	FAM47C	nonsynonymous SNV	[illegible]	Xp21.1	.
X	37979716	C	T	AGAACAACTC,AAGGTAGAGT	exonic	SYTL5	stopgain	[illegible]	Xp11.4	.
X	46713472	C	T	AAGCATTGTT,CAATATCCCG	exonic	RP2	nonsynonymous SNV	[illegible]	Xp11.23	.
X	48418020	C	A	GCGGTACCTG,TGAACGTGTA	exonic	TBC1D25	nonsynonymous SNV	[illegible]	Xp11.23	.
X	53423417	G	A	AGTTTCTTAC,AATCTCCTCC	exonic	SMC1A	nonsynonymous SNV	[illegible]	Xp11.22	.
X	53432269	C	T	AGGTCACTGG,CCCACCAGAG	exonic	SMC1A	nonsynonymous SNV	[illegible]	Xp11.22	.
X	53432271	C	T	GTCACTGGCC,CACCAGAGAT	exonic	SMC1A	nonsynonymous SNV	[illegible]	Xp11.22	.
X	53561113	G	A	AACTTGGCAC,GTCAGCTTGA	exonic	HUWE1	nonsynonymous SNV	[illegible]	Xp11.22	.
X	54275974	G	A	CTTGTGTTCT,ATTTGGAGGT	exonic	WNK3	nonsynonymous SNV	[illegible]	Xp11.22	.
X	63445521	C	T	AATTGGAGAA,TATTCTCATT	exonic	ASB12,MTMR8	nonsynonymous SNV	[illegible]	Xq11.2	.
X	65393484	C	T	TGACTCTGTT,CCCCGGGGGG	exonic	HEPH	nonsynonymous SNV	[illegible]	Xq12	.
X	68049720	C	T	AACCTGGAGC,CGTATCCTGG	exonic	EFNB1	nonsynonymous SNV	[illegible]	Xq13.1	.
X	69262274	G	A	GCCCAGAAAG,TAGGGACCTT	exonic	AWAT2	nonsynonymous SNV	[illegible]	Xq13.1	.
X	69455634	T	C	AGTGCTTTAC,TTGCCTGGTT	exonic	AWAT1	nonsynonymous SNV	[illegible]	Xq13.1	.
X	70386953	G	A	CCTGCTGGCA,ACAAAGTGGG	exonic	NLGN3	nonsynonymous SNV	[illegible]	Xq13.1	.
X	70621460	C	T	AATGCGCCAC,TTCCAACCCT	exonic	TAF1	nonsynonymous SNV	[illegible]	Xq13.1	.
X	74318802	G	A	AAATCCCAGC,AAATGGCAAC	exonic	ABCB7	nonsynonymous SNV	[illegible]	Xq13.3	.
X	85218948	C	T	GACTCATCCT,CGTAGGCAGG	exonic	CHM	nonsynonymous SNV	[illegible]	Xq21.2	.
X	100105348	C	T	ATCTGCAATT,CAAAACTTTG	exonic	NOX1	nonsynonymous SNV	[illegible]	Xq22.1	.
X	100356237	C	T	TGCTGATGAT,AAGCTGAAGA	exonic	CENPI	stopgain	[illegible]	Xq22.1	.
X	100748347	G	A	TGGTGCTGGG,ACCAGACTGG	exonic	ARMCX4	nonsynonymous SNV	[illegible]	Xq22.1	.
X	100910986	T	A	CGAAAGGATT,TCAAAATCTC	exonic	ARMCX2	nonsynonymous SNV	[illegible]	Xq22.1	.
X	101910717	G	A	AGCTTGTGTG,AGGGTGATGT	exonic	GPRASP1	nonsynonymous SNV	[illegible]	Xq22.1	.
X	103349463	C	T	GAATTGAATT,CTTGAGAATG	exonic	SLC25A53	nonsynonymous SNV	[illegible]	Xq22.2	.
X	105011151	G	A	GAATTGCCAG,AAGTGGAATC	exonic	IL1RAPL2	nonsynonymous SNV	[illegible]	Xq22.3	.
X	106092478	C	T	TTTTTAGGTT,AGATGATTTT	exonic	TBC1D8B	nonsynonymous SNV	[illegible]	Xq22.3	.
X	106796024	G	A	CTGTCCCACA,GATGTGATGT	splicing	FRMPD3(ENST00000276185.4:exon8:c.610-1G>A,ENST00000439554.1:exon6:c.454-1G>A)	.	.	Xq22.3	.
X	108631736	G	A	AGGCCAATTC,AATTCGGACC	exonic	GUCY2F	stopgain	[illegible]	Xq22.3	.
X	109697207	C	T	AAGCCCACAT,GCACATGACT	exonic	RGAG1	nonsynonymous SNV	[illegible]	Xq23	RGAG1;basechange=c.3362C>T;AAchange=p.S1121L;tissue=large_intestine
X	117053571	C	T	GAAGGTTGTC,ATATTAAGAG	exonic	KLHL13	nonsynonymous SNV	[illegible]	Xq24	.
X	119293142	G	A	CAGCGGCAGC,ATGGCAACGT	exonic	RHOXF2	nonsynonymous SNV	[illegible]	Xq24	.
X	120181890	C	T	TGTGCTGAGT,TCTCCTTCCC	exonic	GLUD2	nonsynonymous SNV	[illegible]	Xq24	.
X	123019706	G	A	CGGTGCTTTA,TTGTCATGCA	exonic	XIAP	nonsynonymous SNV	[illegible]	Xq25	.
X	124029907	G	A	GGACAAACAG,AACTATGCTC	exonic	TENM1	nonsynonymous SNV	[illegible]	Xq25	.
X	125685418	C	T	GAAGAGGACT,CAGGGTGGCG	exonic	DCAF12L1	nonsynonymous SNV	[illegible]	Xq25	.
X	129149948	G	A	ACCTGCTTCC,GGCTGATGGC	exonic	BCORL1	nonsynonymous SNV	[illegible]	Xq26.1	.
X	133941600	G	A	GAGCCGGAAA,CCTTGAGAAC	exonic	FAM122C	nonsynonymous SNV	[illegible]	Xq26.3	.
X	134711227	C	T	CGGAGTATGT,CCTGCTGTTG	exonic	DDX26B	nonsynonymous SNV	[illegible]	Xq26.3	.
X	135288658	G	A	TGTGCAAAAG,ATGGCCACCA	exonic	FHL1	nonsynonymous SNV	[illegible]	Xq26.3	.
X	135428198	C	T	CCTTTGACAC,AAGGGAGACT	exonic	GPR112	nonsynonymous SNV	[illegible]	Xq26.3	.
X	141291840	C	T	TCCAACAGGC,TGTGGAAGAG	splicing	MAGEC2	.	.	Xq27.2	.
X	148564505	C	T	TGTCAGAATT,CACTGAGGGA	exonic	IDS	stopgain	[illegible]	Xq28	.
X	148797867	G	A	TCGAGTCAAG,GGCTGATCAC	exonic	MAGEA11	nonsynonymous SNV	[illegible]	Xq28	.
X	148797868	G	A	CGAGTCAAGG,GCTGATCACA	exonic	MAGEA11	nonsynonymous SNV	[illegible]	Xq28	.
X	149912873	G	A	GATGCTGACC,ATCTCCCATA	exonic	MTMR1	nonsynonymous SNV	[illegible]	Xq28	.
X	152225653	G	A	GCTCCCAAGG,AAATACCAGG	exonic	PNMA3	nonsynonymous SNV	[illegible]	Xq28	.
X	153033315	G	A	CAGCGGCGCA,AGGAAGCCAC	exonic	PLXNB3	nonsynonymous SNV	[illegible]	Xq28	.
X	153128152	G	A	GATGGGGGAA,TGGCCCCTGA	exonic	L1CAM	nonsynonymous SNV	[illegible]	Xq28	.
X	153218081	G	A	CAGCTCCTCG,GGGTGAGCCC	exonic	HCFC1	nonsynonymous SNV	[illegible]	Xq28	.
X	153578037	G	A	GGCCTTGAAG,GGCTGCCCCC	exonic	FLNA	nonsynonymous SNV	[illegible]	Xq28	.
X	153697230	C	T	GAGAAGGGCC,CATTGATGCC	exonic	PLXNA3	nonsynonymous SNV	[illegible]	Xq28	.
X	154213073	T	G	GAGTGCCAAC,TTTCCCTGAT	exonic	F8	nonsynonymous SNV	[illegible]	Xq28	.
X	154508447	G	T	CTTTAATAAT,TTCAGCTTGG	exonic	CLIC2	nonsynonymous SNV	[illegible]	Xq28	.

M1164I;tissue=large_inte

C000-648QQH

Chromosome	Position (hg1	Reference all	Mutant allele	Sequence context	ANNOVAR fu	Gene	Mutation type	ANNOVAR output (alternative transcripts)
1	977501	G	A	GCTGAGTGCC,GCAGCAGCGT	exonic	AGRN	nonsynonymous SNV	AGRN:ENST00000379370.2:exon7:c.G1343A:p.R448Q,
1	16257037	T	A	TTTACTCTTT,GCATTGGATA	exonic	SPEN	nonsynonymous SNV	SPEN:ENST00000375759.3:exon11:c.T4302A:p.F1434L,
1	28209143	C	G	AGATGAGTGA,AGCCGGCGCT	exonic	THEMIS2	nonsynonymous SNV	THEMIS2:ENST00000328928.7:exon5:c.C921G:p.D307E,THEMIS2:ENST00000373921.3:exon4:c.C1308G:p.D436E,
1	46476534	G	A	AGACTGCCTG,ATAAATCTCG	exonic	MAST2	nonsynonymous SNV	MAST2:ENST00000361297.2:exon10:c.G1111A:p.D371N,MAST2:ENST00000372008.2:exon8:c.G766A:p.D256N,
1	155340754	G	A	AGTGTTGGGG,AACACTCAGC	exonic	ASH1L	nonsynonymous SNV	ASH1L:ENST00000368346.3:exon11:c.C6368T:p.S2123F,ASH1L:ENST00000392403.3:exon11:c.C6353T:p.S2118F,
1	157771290	C	T	AATAAGGCCA,GGTGGCTGGA	exonic	FCRL1	nonsynonymous SNV	FCRL1:ENST00000368176.3:exon6:c.G964A:p.V322M,FCRL1:ENST00000491942.1:exon6:c.G964A:p.V322M,
1	237946993	C	T	GTTAATGGAA,GATTGGCAAA	exonic	RYR2	nonsynonymous SNV	RYR2:ENST00000360064.6:exon92:c.C11999T:p.T4000M,RYR2:ENST00000366574.2:exon90:c.C11981T:p.T3994M,RYR2:ENST00000542537.1:exon89:c.C11933T:p.T3978M,
2	21362279	C	T	GTTGAAGCCT,AGAAAATATT	exonic	TDRD15	nonsynonymous SNV	TDRD15:ENST00000405799.1:exon4:c.C1940T:p.S647L,
2	128044527	C	T	AGCTGAGTTG,CCAGCACCAG	exonic	ERCC3	nonsynonymous SNV	ERCC3:ENST00000285398.2:exon8:c.G1094A:p.G365D,ERCC3:ENST00000493187.2:exon8:c.G902A:p.G301D,
2	135911351	C	G	GATGAAGATT,CAAGCAATAT	exonic	RAB3GAP1	nonsynonymous SNV	RAB3GAP1:ENST00000539493.1:exon21:c.C2062G:p.P688A,RAB3GAP1:ENST00000264158.8:exon19:c.C2194G:p.P732A,RAB3GAP1:ENST00000442034.1:exon19:c.C2194G:p.P732A,
2	158397693	C	T	TATTCACATT,ATTGTATCAT	exonic	ACVR1C	nonsynonymous SNV	ACVR1C:ENST00000243349.8:exon7:c.G1134A:p.M378I,ACVR1C:ENST00000409680.3:exon7:c.G984A:p.M328I,ACVR1C:ENST00000335450.7:exon6:c.G894A:p.M298I,ACVR1C:ENST00000348328.5:exon5:c.G663A:p.M221I,
2	198949938	G	A	GAAATGTCTC,AAGGATGTCG	exonic	PLCL1	nonsynonymous SNV	PLCL1:ENST00000437704.2:exon1:c.G1403A:p.R468Q,PLCL1:ENST00000428675.1:exon2:c.G1697A:p.R566Q,PLCL1:ENST00000487695.2:exon2:c.G1475A:p.R492Q,
2	204571453	C	A	CAACTTATTC,CTTCAATTCA	exonic	CD28	nonsynonymous SNV	CD28:ENST00000374478.4:exon1:c.C34A:p.P12T,CD28:ENST00000324106.8:exon1:c.C34A:p.P12T,CD28:ENST00000374481.3:exon1:c.C34A:p.P12T,
3	63981393	C	A	CCTGCTGCTT,AGGGGCGATG	exonic	ATXN7	stopgain	ATXN7:ENST00000538065.1:exon10:c.C1895A:p.S632X,ATXN7:ENST00000398590.3:exon12:c.C1895A:p.S632X,ATXN7:ENST00000295900.6:exon12:c.C1895A:p.S632X,ATXN7:ENST00000484332.1:exon8:c.C1460A:p.S487X,ATXN7:ENST00000487717.1:exon11:c.C1895A:p.S632X,
3	124906162	G	A	AGACGCCACC,CTGCCGATGC	exonic	SLC12A8	nonsynonymous SNV	SLC12A8:ENST00000473262.1:exon2:c.C262T:p.R88W,SLC12A8:ENST00000479826.1:exon2:c.C187T:p.R63W,
3	159583953	C	G	AAATTTTAGG,ACAGAAAAAT	exonic	IQCJ-SCHIP1	nonsynonymous SNV	IQCJ-SCHIP1:ENST00000527095.1:exon2:c.C65G:p.A22G,IQCJ-SCHIP1:ENST00000412423.2:exon3:c.C722G:p.A241G,IQCJ-SCHIP1:ENST00000337808.6:exon3:c.C761G:p.A254G,SCHIP1:ENST00000495954.1:exon2:c.C32G:p.A11G,SCHIP1:ENST00000445224.2:exon2:c.C32G:p.A11G,IQCJ-SCHIP1:ENS
4	68719851	T	C	AACTGACTAT,ATATTCAACA	exonic	TMPRSS11D	nonsynonymous SNV	TMPRSS11D:ENST00000283916.6:exon3:c.A184G:p.N62D,
4	72121118	T	A	AACCTCTCAG,GAGTACTCTC	splicing	SLC4A4(ENS	.	.
4	83839488	T	A	TTACTAGACC,GTCAGATCAT	exonic	THAP9	nonsynonymous SNV	THAP9:ENST00000302236.5:exon5:c.T2123A:p.L708Q,
5	434722	G	C	GGAGCCCACA,ACGGCCTTCC	exonic	AHRR	nonsynonymous SNV	AHRR:ENST00000506456.1:exon7:c.G1447C:p.D483H,AHRR:ENST00000316418.5:exon12:c.G1933C:p.D645H,AHRR:ENST00000512529.1:exon9:c.G1417C:p.D473H,AHRR:ENST00000505113.1:exon11:c.G1879C:p.D627H,
5	79616978	A	G	CATGACACCT,TTCATTGCAG	exonic	SPZ1	nonsynonymous SNV	SPZ1:ENST00000296739.4:exon1:c.A944G:p.Y315C,
5	89979440	T	A	CCAGTGACTT,GCATTGGAAC	exonic	GPR98	stopgain	GPR98:ENST00000405460.2:exon28:c.T5702A:p.L1901X,
5	89979447	G	A	CTTTGCATTG,AACATAGACT	exonic	GPR98	stopgain	GPR98:ENST00000405460.2:exon28:c.G5709A:p.W1903X,
5	140711111	G	A	GTAGACCACA,AGTGGCCCAA	exonic	PCDHGA1	nonsynonymous SNV	PCDHGA1:ENST00000378105.3:exon1:c.G860A:p.R287K,PCDHGA1:ENST00000517417.1:exon1:c.G860A:p.R287K,
6	46977415	T	C	GTGATCCATT,TACAACGGGG	exonic	GPR110	nonsynonymous SNV	GPR110:ENST00000371253.2:exon11:c.A1756G:p.K586E,GPR110:ENST00000283297.5:exon5:c.A1165G:p.K389E,
6	91263302	C	A	ACTGTAATTA,TACCTAGAAA	exonic	MAP3K7	nonsynonymous SNV	MAP3K7:ENST00000369327.3:exon7:c.G611T:p.S204I,MAP3K7:ENST00000369325.3:exon7:c.G611T:p.S204I,MAP3K7:ENST00000369329.3:exon7:c.G611T:p.S204I,MAP3K7:ENST00000369332.3:exon7:c.G611T:p.S204I,
7	56088902	C	T	GAGTGGGAGA,CATCGCTGGA	exonic	PSPH	nonsynonymous SNV	PSPH:ENST00000424596.1:exon4:c.G4A:p.V2I,PSPH:ENST00000413218.1:exon5:c.G4A:p.V2I,PSPH:ENST00000275605.3:exon4:c.G4A:p.V2I,PSPH:ENST00000427797.1:exon3:c.G4A:p.V2I,PSPH:ENST00000437355.2:exon2:c.G4A:p.V2I,PSPH:ENST00000416592.1:exon5:c.G4A:p.V2I,PSPH:ENST0000041
7	103051902	C	T	ACAGACATGG,GACTTTCACT	exonic	SLC26A5	nonsynonymous SNV	SLC26A5:ENST00000339444.6:exon6:c.G535A:p.A179T,SLC26A5:ENST00000393735.2:exon6:c.G535A:p.A179T,SLC26A5:ENST00000423416.1:exon5:c.G535A:p.A179T,SLC26A5:ENST00000393723.1:exon4:c.G535A:p.A179T,SLC26A5:ENST00000306312.3:exon6:c.G535A:p.A179T,SLC26A5:ENST000004
8	17157580	G	A	GAAGGGCTCC,GGATGGAAAT	exonic	MTMR7	nonsynonymous SNV	MTMR7:ENST00000180173.5:exon14:c.C1774T:p.R592W,MTMR7:ENST00000398099.3:exon5:c.C547T:p.R183W,
8	82713785	C	G	TCTGATACAG,ATTTTCAGGT	exonic	SNX16	nonsynonymous SNV	SNX16:ENST00000345957.4:exon8:c.G982C:p.A328P,SNX16:ENST00000396330.2:exon9:c.G982C:p.A328P,SNX16:ENST00000353788.4:exon7:c.G895C:p.A299P,
8	102643866	G	A	TCCTATCAGG,AGCAGAAAGA	exonic	GRHL2	nonsynonymous SNV	GRHL2:ENST00000395927.1:exon10:c.G1211A:p.G404E,GRHL2:ENST00000251808.3:exon10:c.G1259A:p.G420E,
10	12111132	C	T	TTACGGCTAC,GGCCGAGGAA	exonic	DHTKD1	nonsynonymous SNV	DHTKD1:ENST00000263035.4:exon1:c.C100T:p.R34W,DHTKD1:ENST00000437298.1:exon1:c.C100T:p.R34W,
11	5862937	C	A	CATGGCCAGG,AGTAGTACAT	exonic	OR52E6	nonsynonymous SNV	OR52E6:ENST00000379946.2:exon2:c.G203T:p.C68F,OR52E6:ENST00000329322.5:exon1:c.G191T:p.C64F,
11	40137022	A	C	ATTGTGTGCC,GGTTGATCTC	exonic	LRRC4C	nonsynonymous SNV	LRRC4C:ENST00000527150.1:exon3:c.T821G:p.L274R,LRRC4C:ENST00000530763.1:exon5:c.T821G:p.L274R,LRRC4C:ENST00000278198.2:exon2:c.T821G:p.L274R,LRRC4C:ENST00000528697.1:exon7:c.T821G:p.L274R,
11	46916353	G	C	TTGGCGAACA,CAGCACAGGC	exonic	LRP4	nonsynonymous SNV	LRP4:ENST00000378623.1:exon12:c.C1327G:p.L443V,
11	88323821	C	A	TGCATGTGTA,TCATCAAAGA	exonic	GRM5	nonsynonymous SNV	GRM5:ENST00000305432.5:exon6:c.G1638T:p.E546D,GRM5:ENST00000455756.2:exon7:c.G1638T:p.E546D,GRM5:ENST00000305447.4:exon6:c.G1638T:p.E546D,GRM5:ENST00000393297.1:exon6:c.G1638T:p.E546D,GRM5:ENST00000418177.2:exon7:c.G1638T:p.E546D,
11	89955872	A	G	GCGCTCGCCA,GGGAACGCGG	exonic	CHORDC1	nonsynonymous SNV	CHORDC1:ENST00000529402.1:exon1:c.T251C:p.L84P,
12	40876346	A	G	AGGGACCACT,GACCATCATC	exonic	MUC19	nonsynonymous SNV	MUC19:ENST00000454784.4:exon49:c.A7891G:p.R2631G,
12	86373308	T	C	TTGCCGATCT,CTGTTCCAGT	exonic	MGAT4C	nonsynonymous SNV	MGAT4C:ENST00000604798.1:exon8:c.A1196G:p.E399G,MGAT4C:ENST00000332156.1:exon7:c.A1196G:p.E399G,MGAT4C:ENST00000549405.2:exon9:c.A1196G:p.E399G,MGAT4C:ENST00000393205.2:exon4:c.A1283G:p.E428G,MGAT4C:ENST00000548651.1:exon4:c.A1196G:p.E399G,MGAT4C:ENST0
12	94805553	C	T	TGAAGTTTTT,CTGCTGAGTT	exonic	CCDC41	nonsynonymous SNV	CCDC41:ENST00000397809.5:exon4:c.G244A:p.E82K,CCDC41:ENST00000339839.5:exon3:c.G244A:p.E82K,CCDC41:ENST00000547232.1:exon4:c.G145A:p.E49K,CCDC41:ENST00000397807.2:exon3:c.G145A:p.E49K,CCDC41:ENST00000547575.1:exon4:c.G244A:p.E82K,
12	104376635	C	T	TGGATGATCA,ACTCTACCAG	exonic	TDG	nonsynonymous SNV	TDG:ENST00000542036.1:exon3:c.C8T:p.T3I,
13	67801082	G	C	TTTTCCCACT,TCTTCATCTG	exonic	PCDH9	nonsynonymous SNV	PCDH9:ENST00000377861.3:exon2:c.C1491G:p.D497E,PCDH9:ENST00000544246.1:exon2:c.C1491G:p.D497E,PCDH9:ENST00000328454.5:exon1:c.C1491G:p.D497E,PCDH9:ENST00000456367.1:exon2:c.C1491G:p.D497E,PCDH9:ENST00000377865.2:exon1:c.C1491G:p.D497E,
13	76197424	G	A	TTTGAGGACT,AACAAAATTC	exonic	LMO7	nonsynonymous SNV	LMO7:ENST00000377534.3:exon2:c.G193A:p.E65K,LMO7:ENST00000357063.3:exon2:c.G193A:p.E65K,LMO7:ENST00000341547.4:exon2:c.G193A:p.E65K,
14	23240226	G	A	TTCTCACCCA,GCAGTGTTTA	splicing	OXA1L(ENST	.	.
14	105766818	C	T	GGCCGAGGAG,CGCCGCCGCT	exonic	BRF1	nonsynonymous SNV	BRF1:ENST00000546474.1:exon1:c.G149A:p.G50D,BRF1:ENST00000379937.2:exon1:c.G149A:p.G50D,BRF1:ENST00000548421.1:exon1:c.G149A:p.G50D,
16	347176	C	T	GGCGCTCTTC,CCGACTCAGC	exonic	AXIN1	nonsynonymous SNV	AXIN1:ENST00000354866.3:exon7:c.G1835A:p.G612E,AXIN1:ENST00000262320.3:exon7:c.G1835A:p.G612E,
17	42426831	G	T	CATCTGGGTG,CCCCTGCCAG	exonic	GRN	nonsynonymous SNV	GRN:ENST00000053867.3:exon3:c.G176T:p.G59V,GRN:ENST00000586782.1:exon3:c.G176T:p.G59V,GRN:ENST00000589536.1:exon4:c.G176T:p.G59V,GRN:ENST00000589265.1:exon3:c.G176T:p.G59V,GRN:ENST00000587109.1:exon3:c.G176T:p.G59V,GRN:ENST00000588237.1:exon3:c.G176T:p.G59V,
17	47296905	C	A	CTCGCAGGAC,TCAGCACGCA	exonic	ABI3	nonsynonymous SNV	ABI3:ENST00000419580.2:exon4:c.C448A:p.L150I,ABI3:ENST00000225941.1:exon4:c.C466A:p.L156I,
17	66364691	C	G	TGTTTCAGCA,CAGCGGGAGG	exonic	ARSG	nonsynonymous SNV	ARSG:ENST00000452479.2:exon6:c.C215G:p.T72S,ARSG:ENST00000448504.2:exon7:c.C707G:p.T236S,
17	67285394	T	C	AGCTTGGTTA,CTTTGATAGT	exonic	ABCA5	nonsynonymous SNV	ABCA5:ENST00000586995.1:exon7:c.A839G:p.D280G,ABCA5:ENST00000392677.2:exon14:c.A1826G:p.D609G,ABCA5:ENST00000593153.1:exon15:c.A1826G:p.D609G,ABCA5:ENST00000392676.3:exon14:c.A1826G:p.D609G,ABCA5:ENST00000588877.1:exon13:c.A1826G:p.D609G,
17	80356187	G	A	CGAAGGAGCC,TAGGTCACCT	exonic	OGFOD3	nonsynonymous SNV	OGFOD3:ENST00000582593.1:exon2:c.C40T:p.R14W,
18	210461	G	T	AGTTCTTCAG,TCATTATGTA	exonic	USP14	nonsynonymous SNV	USP14:ENST00000383589.2:exon13:c.G1163T:p.G388V,USP14:ENST00000582707.1:exon14:c.G1196T:p.G399V,USP14:ENST00000400266.3:exon14:c.G1268T:p.G423V,USP14:ENST00000261601.7:exon15:c.G1301T:p.G434V,
19	7697292	G	T	GGACCCCACG,GCTGGAAGCC	exonic	PCP2	nonsynonymous SNV	PCP2:ENST00000311069.5:exon3:c.C278A:p.P93H,PCP2:ENST00000598935.1:exon3:c.C230A:p.P77H,
19	9061509	G	A	ATTGACTACA,GTGTGTTTGA	exonic	MUC16	nonsynonymous SNV	MUC16:ENST00000397910.4:exon3:c.C25937T:p.P8646L,
19	12383772	G	T	AAGGGAACTG,AAGTACGGAA	exonic	ZNF44	nonsynonymous SNV	ZNF44:ENST00000355684.5:exon4:c.C1298A:p.S433Y,ZNF44:ENST00000397742.3:exon4:c.C1298A:p.S433Y,ZNF44:ENST00000356109.5:exon5:c.C1442A:p.S481Y,ZNF44:ENST00000393337.3:exon5:c.C1442A:p.S481Y,
19	18255359	G	A	TCTGGATGCC,GCCGGGGCCG	exonic	MAST3	nonsynonymous SNV	MAST3:ENST00000262811.6:exon22:c.G2581A:p.G861S,
19	35843227	G	A	GGCTCCTGGC,GAAGCTGGGG	exonic	FFAR1	nonsynonymous SNV	FFAR1:ENST00000246553.2:exon1:c.G773A:p.R258Q,
19	38573347	T	C	GCCTCGGCCA,GGCCTCCCTC	exonic	SIPA1L3	nonsynonymous SNV	SIPA1L3:ENST00000222345.6:exon3:c.T1142C:p.M381T,
19	38876352	G	A	GGGCGCAGCC,CGGGTGCGGG	exonic	GGN	nonsynonymous SNV	GGN:ENST00000585737.1:exon3:c.C1301T:p.A434V,GGN:ENST00000334928.6:exon3:c.C1550T:p.A517V,
21	38098614	T	A	TATTCCTGGA,TCCAGGTGAG	exonic	SIM2	nonsynonymous SNV	SIM2:ENST00000430056.3:exon6:c.T738A:p.D246E,SIM2:ENST00000290399.6:exon6:c.T738A:p.D246E,
X	47464462	G	A	AACCATCTGG,CAAACTATGA	exonic	SYN1	nonsynonymous SNV	SYN1:ENST00000340666.4:exon5:c.C689T:p.A230V,SYN1:ENST00000295987.7:exon5:c.C689T:p.A230V,
X	70524095	T	C	GTGGTGACTG,ATATGGCCAG	exonic	ITGB1BP2	nonsynonymous SNV	ITGB1BP2:ENST00000373829.3:exon9:c.T698C:p.V233A,ITGB1BP2:ENST00000538820.1:exon8:c.T644C:p.V215A,
X	105937584	G	C	TGATGCTGTT,TGATTTACAA	exonic	RNF128	nonsynonymous SNV	RNF128:ENST00000324342.3:exon1:c.G352C:p.V118L,RNF128:ENST00000418562.1:exon2:c.G271C:p.V91L,

ne

C16_ENST00000331986;basechange=c.12836C>A;AAchange=p.P4279H;tissue=lung&MUC16_ENST00000397910;basechange=c.25937C>A;AAchange=p.P8646H;tissue=lung

ne

C000-648QQH

Chromosom	Position (hg1	Reference all	Mutant allele	Sequence context	ANNOVAR function	Gene	Mutation type	ANNOVAR output (alternative transcripts)	Cytoband	COSMIC
2	152584365	G	C	GGAAGTTTCT,ATTGCTCATA	exonic	NEB	stopgain	NEB:ENST00000427231.2:exon5:c.C134G:p.S45X,NEB:ENST00000397345.3:exon5:c.C134G:p.S45X,NEB:ENST00000604864.1:exon3:c.C134G:p.S45X,NEB:ENST00000603639.1:exon3:c.C134G:p.S45X,NEB:ENST00000409198.1:exon5:c.C134G:p.S45X,NEB:ENST00000172853.10:exon5:c.C134G:p.S45X,	2q23.3	.
2	187703836	C	T	AGTTTGAACT,GATGTATCCC	exonic	ZSWIM2	nonsynonymous SNV	ZSWIM2:ENST00000295131.2:exon4:c.G344A:p.R115Q,	2q32.1	.
5	35800251	T	C	GCAGAAAAGG,AATTGCATTC	splicing	SPEF2(ENST00000356031.3:exon34:c.5010+2T>C,ENST00000440995.2:exon34:c.4995+2T>C,ENST00000506526.1:exon12:c.2629+2T>C,ENST00000303129.4:exon10:c.1401+2T>C,ENST00000513078.1:exon10:c.1625+2T>C)	.	.	5p13.2	.
6	25850052	C	A	ATATCTAAGA,ATTGATATAA	exonic	SLC17A3	nonsynonymous SNV	SLC17A3:ENST00000361703.6:exon9:c.G1018T:p.V340F,SLC17A3:ENST00000360657.3:exon9:c.G1018T:p.V340F,SLC17A3:ENST00000397060.4:exon10:c.G1252T:p.V418F,	6p22.2	.
6	30916386	C	T	TCACATATTT,CCCTCACTCC	exonic	DPCR1	nonsynonymous SNV	DPCR1:ENST00000462446.1:exon2:c.C145T:p.P49S,	6p21.33	.
7	123152327	G	A	AGTATCAAAA,CATCATCACT	exonic	IQUB	nonsynonymous SNV	IQUB:ENST00000434450.1:exon2:c.C68T:p.A23V,IQUB:ENST00000324698.6:exon2:c.C68T:p.A23V,IQUB:ENST00000469057.1:exon2:c.C68T:p.A23V,IQUB:ENST00000466202.1:exon2:c.C68T:p.A23V,IQUB:ENST00000484508.1:exon2:c.C68T:p.A23V,	7q31.32	.
8	113668499	C	T	ATTGTAAGAT,CAAGCAAGGG	exonic	CSMD3	nonsynonymous SNV	CSMD3:ENST00000339701.3:exon5:c.G908A:p.G303E,CSMD3:ENST00000297405.5:exon18:c.G2888A:p.G963E,CSMD3:ENST00000343508.3:exon19:c.G2768A:p.G923E,CSMD3:ENST00000455883.2:exon17:c.G2576A:p.G859E,CSMD3:ENST00000352409.3:exon18:c.G2888A:p.G963E,	8q23.3	CSMD3;basechange=c.2888G>T;AAchange=p.G963V;tissue=lung
11	8496269	C	T	ATATTTTTTT,TTTTTTTCTC	exonic	STK33	nonsynonymous SNV	STK33:ENST00000396673.1:exon3:c.G184A:p.E62K,STK33:ENST00000447869.1:exon1:c.G184A:p.E62K,STK33:ENST00000431279.2:exon5:c.G184A:p.E62K,STK33:ENST00000534493.1:exon3:c.G61A:p.E21K,STK33:ENST00000396672.1:exon2:c.G184A:p.E62K,STK33:ENST00000418597.1:exon2:c.G61A:p.E21K,STK33:ENST00000457885.2:exon2:c.G184A:p.E62K,STK33:ENST00000524760.1:exon1:c.G34A:p.E12K,STK33:ENST00000422559.2:exon3:c.G61A:p.E21K,STK33:ENST00000315204.1:exon3:c.G184A:p.E62K,	11p15.4	.
14	24780924	G	A	GATGGAGAAG,ACGGTCCGGA	exonic	LTB4R2	nonsynonymous SNV	LTB4R2:ENST00000533293.1:exon2:c.G1054A:p.D352N,LTB4R2:ENST00000528054.1:exon1:c.G1147A:p.D383N,LTB4R2:ENST00000543919.1:exon2:c.G1054A:p.D352N,	14q12	.
19	10480547	G	T	GTGAAACCCC,TCTCTACTAA	exonic	TYK2	nonsynonymous SNV	TYK2:ENST00000530829.1:exon4:c.C200A:p.T67K,	19p13.2	.
22	26224742	G	T	TCTCTCCCTA,ATCCTTTTCC	splicing	MYO18B(ENST00000536101.1:exon15:c.2787-1G>T,ENST00000335473.7:exon15:c.2787-1G>T,ENST00000407587.2:exon15:c.2787-1G>T,ENST00000539302.1:exon13:c.2642-1G>T)	.	.	22q12.1	.

Chromosom	Position (hg1	Reference all	Mutant allele	Sequence context	ANNOVAR fu	Gene	Mutation type	ANNOVAR output (alternative transcripts)	Cytoband	COSMIC
1	984654	C	T	CTGACCAGTG,CGTGCCGGTA	exonic	AGRN	nonsynonymous SNV	AGRN:ENST00000379370.2:exon25:c.C4337T:p.A1446V	1p36.33	.
1	1322777	G	A	AGAACGGGAA,AACTCCGGCT	exonic	CCNL2	nonsynonymous SNV	CCNL2:ENST00000408952.5:exon10:c.C731T:p.S244F,CCNL2:ENST00000400809.3:exon11:c.C1397T:p.S466F	1p36.33	.
1	4772246	G	A	CAGGCCCCGG,ACCAGGCGGC	exonic	AJAP1	nonsynonymous SNV	AJAP1:ENST00000378190.3:exon2:c.G316A:p.D106N,AJAP1:ENST00000378191.4:exon2:c.G316A:p.D106N	1p36.32	.
1	6635048	T	A	GTTTTTCGAG,CCGTGGTGCT	exonic	TAS1R1	nonsynonymous SNV	TAS1R1:ENST00000328191.4:exon3:c.T856A:p.S286T,TAS1R1:ENST00000333172.6:exon3:c.T856A:p.S286T	1p36.31	.
1	6635051	G	A	TTTCGAGTCC,TGGTGCTGAC	exonic	TAS1R1	nonsynonymous SNV	TAS1R1:ENST00000328191.4:exon3:c.G859A:p.V287M,TAS1R1:ENST00000333172.6:exon3:c.G859A:p.V287M	1p36.31	.
1	6662413	G	C	GCTGCGCCGC,CTCGCCAGTC	exonic	KLHL21	nonsynonymous SNV	KLHL21:ENST00000377658.4:exon1:c.C465G:p.S155R,KLHL21:ENST00000377663.3:exon1:c.C465G:p.S155R	1p36.31	.
1	6681495	C	T	GACCTCGTGA,CTGCTTCTGC	exonic	PHF13	nonsynonymous SNV	PHF13:ENST00000377648.4:exon4:c.C701T:p.T234I	1p36.31	.
1	10479766	G	T	AACTGGACAG,CCATGGTGGC	exonic	PGD	nonsynonymous SNV	PGD:ENST00000270776.8:exon13:c.G1412T:p.G471V,PGD:ENST00000538557.1:exon13:c.G1373T:p.G458V,PGD:ENST00000541529.1:exon12:c.G1346T:p.G449V	1p36.22	.
1	12336353	C	A	CTCCTCACCA,CCCAGAAATG	exonic	VPS13D	nonsynonymous SNV	VPS13D:ENST00000356315.4:exon19:c.C2708A:p.T903N,VPS13D:ENST00000358136.3:exon19:c.C2708A:p.T903N	1p36.22	.
1	12460295	A	C	GACTCCCCTG,GCAATGAGAA	exonic	VPS13D	nonsynonymous SNV	VPS13D:ENST00000356315.4:exon60:c.A11617C:p.S3873R,VPS13D:ENST00000358136.3:exon61:c.A11692C:p.S3898R	1p36.22	.
1	12836053	C	T	GCTGTCCATT,TTATAAGGTT	exonic	PRAMEF12	nonsynonymous SNV	PRAMEF12:ENST00000357726.4:exon2:c.C655T:p.L219F	1p36.21	PRAMEF12;b
1	19513051	T	A	GCTGTCCTCC,CCGTGCTACT	exonic	UBR4	nonsynonymous SNV	UBR4:ENST00000375254.3:exon14:c.A1733T:p.E578V,UBR4:ENST00000375217.2:exon14:c.A1733T:p.E578V,UBR4:ENST00000375226.2:exon14:c.A1733T:p.E578V,UBR4 NST00000375267.2:exon14:c.A1733T:p.E578V,	1p36.13	.
1	20249137	G	A	CGGCCAGTGG,AGCCACCGAT	exonic	PLA2G2E	nonsynonymous SNV	PLA2G2E:ENST00000375116.3:exon2:c.C152T:p.S51F	1p36.13	.
1	22172604	G	A	CCCTCACCGG,GACGTGGACA	exonic	HSPG2	nonsynonymous SNV	HSPG2:ENST00000374695.3:exon64:c.C8461T:p.P2821S	1p36.12	.
1	22182297	G	A	CTCGAGGGTG,GCGTGGGGCT	exonic	HSPG2	nonsynonymous SNV	HSPG2:ENST00000374695.3:exon45:c.C5684T:p.P1895L	1p36.12	.
1	22182298	G	A	TCGAGGGTGG,CGTGGGGCTC	exonic	HSPG2	nonsynonymous SNV	HSPG2:ENST00000374695.3:exon45:c.C5683T:p.P1895S	1p36.12	.
1	26156250	C	T	TCTCTTTGTC,AAGGCCAGCA	exonic	MTFR1L	nonsynonymous SNV	MTFR1L:ENST00000474295.1:exon6:c.C593T:p.P198L,MTFR1L:ENST00000526894.1:exon6:c.C524T:p.P175L,MTFR1L:ENST00000466284.1:exon5:c.C593T:p.P198	1p36.11	.
1	27057967	C	T	GGCTCAGTCT,CTTACCAGCA	exonic	ARID1A	nonsynonymous SNV	ARID1A:ENST00000324856.7:exon3:c.C1675T:p.P559S,ARID1A:ENST00000374152.2:exon2:c.C526T:p.P176S,ARID1A:ENST00000457599.2:exon3:c.C1675T:p.P559	1p36.11	.
1	28176773	C	T	GGTGGCTTGC,CATGCCATAC	exonic	PPP1R8	nonsynonymous SNV	PPP1R8:ENST00000311772.5:exon7:c.C872T:p.P291L,PPP1R8:ENST00000431586.1:exon7:c.C446T:p.P149L,PPP1R8:ENST00000236412.7:exon6:c.C200T:p.P67L,PPP1R ENST00000373931.4:exon7:c.C446T:p.P149L,	1p35.3	.
1	31897552	G	A	TGTGAGGAGG,GGCCGGGATC	exonic	SERINC2	nonsynonymous SNV	SERINC2:ENST00000536859.1:exon3:c.G236A:p.G79E,SERINC2:ENST00000536384.1:exon3:c.G236A:p.G79E,SERINC2:ENST00000373709.3:exon3:c.G224A:p.G75E,SEI NC2:ENST00000373710.1:exon4:c.G251A:p.G84E,	1p35.2	.
1	33960561	A	T	CTCAGGGGAG,AACTTTATGA	exonic	ZSCAN20	stopgain	ZSCAN20:ENST00000361328.3:exon8:c.A2617T:p.K873X	1p35.1	.
1	33960577	C	T	TATGAGTGTT,TGAATGTGGA	exonic	ZSCAN20	nonsynonymous SNV	ZSCAN20:ENST00000361328.3:exon8:c.C2633T:p.S878F	1p35.1	.
1	33960597	T	A	AAGAAGCTTC,CTAAGAGCTC	exonic	ZSCAN20	nonsynonymous SNV	ZSCAN20:ENST00000361328.3:exon8:c.T2653A:p.S885T	1p35.1	.
1	33960598	C	G	AGAAGCTTCT,TAAGAGCTCT	exonic	ZSCAN20	nonsynonymous SNV	ZSCAN20:ENST00000361328.3:exon8:c.C2654G:p.S885C	1p35.1	.
1	36474365	G	T	AAGGAGGCCT,CCAGTCATCA	exonic	AGO3	nonsynonymous SNV	AGO3:ENST00000373191.4:exon7:c.G868T:p.A290S,AGO3:ENST00000246314.6:exon5:c.G166T:p.A56S	1p34.3	.
1	36938171	G	A	TCACACTTCT,ATTTATGTGC	exonic	CSF3R	stopgain	CSF3R:ENST00000440588.2:exon5:c.C790T:p.Q264X,CSF3R:ENST00000418048.2:exon5:c.C790T:p.Q264X,CSF3R:ENST00000361632.4:exon6:c.C790T:p.Q264X,CSF3R NST00000373104.1:exon7:c.C790T:p.Q264X,CSF3R:ENST00000373103.1:exon7:c.C790T:p.Q264X,CSF3R:ENST00000331941.5:exon5:c.C790T:p.Q264X,CSF3R:ENST00000 373106.1:exon7:c.C790T:p.Q264X,CSF3R:ENST00000338937.5:exon5:c.C790T:p.Q264X,	1p34.3	.
1	38149083	G	A	CTGGATAGAC,AAAGAAGATA	exonic	C1orf109	nonsynonymous SNV	C1orf109:ENST00000464085.1:exon4:c.C482T:p.S161L,C1orf109:ENST00000491981.1:exon4:c.C482T:p.S161L,C1orf109:ENST00000358011.4:exon4:c.C482T:p.S161	1p34.3	.
1	41232364	A	C	ACTTGCCACC,ATGCTCAACA	exonic	NFYC	nonsynonymous SNV	NFYC:ENST00000427410.2:exon7:c.A703C:p.N235H,NFYC:ENST00000308733.5:exon7:c.A817C:p.N273H,NFYC:ENST00000372654.1:exon9:c.A817C:p.N273H,NFYC:ENST 0000456393.2:exon8:c.A817C:p.N273H,NFYC:ENST00000425457.2:exon8:c.A817C:p.N273H,NFYC:ENST00000372651.1:exon8:c.A817C:p.N273H,NFYC:ENST00000372653.1 :exon13:c.A715C:p.N239H,NFYC:ENST00000372652.1:exon8:c.A817C:p.N273H,NFYC:ENST00000447388.3:exon8:c.A817C:p.N273H,NFYC:ENST00000440226.3:exon8:c.A81	1p34.2	.
1	43220632	G	C	AATCTCCTGG,AGATGCGTAC	exonic	LEPRE1	nonsynonymous SNV	LEPRE1:ENST00000397054.3:exon8:c.C1253G:p.S418C,LEPRE1:ENST00000236040.4:exon8:c.C1253G:p.S418C,LEPRE1:ENST00000296388.5:exon8:c.C1253G:p.S418	1p34.2	.
1	43240893	G	C	TGGGGGACAA,TTGTCCACAT	exonic	C1orf50	nonsynonymous SNV	C1orf50:ENST00000372525.5:exon5:c.G428C:p.S143T	1p34.2	.
1	47138755	C	T	GCCATCAACA,GGGCCCTGCC	exonic	TEX38	nonsynonymous SNV	TEX38:ENST00000334122.4:exon2:c.C248T:p.T83M,TEX38:ENST00000564373.1:exon2:c.C86T:p.T29M,TEX38:ENST00000564071.1:exon2:c.C251T:p.T84M,TEX38:ENST0 00415500.1:exon2:c.C20T:p.T7M,TEX38:ENST00000569393.1:exon2:c.C410T:p.T137M,	1p33	.
1	52238345	G	A	GCACAAGAGC,TTATCATGCA	exonic	OSBPL9	nonsynonymous SNV	OSBPL9:ENST00000486942.1:exon9:c.G586A:p.V196I,OSBPL9:ENST00000428468.1:exon14:c.G1120A:p.V374I,OSBPL9:ENST00000453295.1:exon13:c.G1069A:p.V357I,O BPL9:ENST00000361556.5:exon10:c.G790A:p.V264I,OSBPL9:ENST00000447887.1:exon14:c.G1150A:p.V384I,OSBPL9:ENST00000371714.1:exon16:c.G1081A:p.V361I,OSBP L9:ENST00000530544.1:exon11:c.G877A:p.V293I,OSBPL9:ENST00000337809.4:exon13:c.G1135A:p.V379I,OSBPL9:ENST00000435686.2:exon13:c.G625A:p.V209I,OSBPL9:E NST00000462759.1:exon10:c.G586A:p.V196I,OSBPL9:ENST00000371710.3:exon14:c.G1174A:p.V392I,OSBPL9:ENST00000531828.1:exon11:c.G625A:p.V209I,	1p32.3	OSBPL9;bas
1	52803548	G	A	GGACGCGGAG,AACCCCAGGA	exonic	ZFYVE9	nonsynonymous SNV	ZFYVE9:ENST00000287727.3:exon16:c.G3775A:p.E1259K,ZFYVE9:ENST00000371591.1:exon15:c.G3775A:p.E1259K,ZFYVE9:ENST00000357206.2:exon15:c.G3598A:p.E1	1p32.3	.
1	53347262	C	G	CTGCAGGTAT,ATAGCCCACC	exonic	ZYG11A	nonsynonymous SNV	ZYG11A:ENST00000371528.1:exon11:c.C1869G:p.I623M,ZYG11A:ENST00000371532.1:exon10:c.C843G:p.I281M	1p32.3	.
1	74937217	C	G	CTTTTCAGAA,CCATGAATTA	exonic	LRRC53	nonsynonymous SNV	LRRC53:ENST00000294635.4:exon5:c.G2089C:p.V697L	1p31.1	.
1	86172039	C	T	ACAGGGCAAA,TGAAAAGACA	splicing	ZNHIT6(ENST00000431532.2:exon5:c.606-1G>A,ENST00000370574.3:exon4:c.723-		.	1p22.3	.
1	94645447	G	A	TCATTTACAT,TTGGATCTCT	exonic	ARHGAP29	nonsynonymous SNV	ARHGAP29:ENST00000260526.6:exon20:c.C2314T:p.H772Y,ARHGAP29:ENST00000552844.1:exon20:c.C2314T:p.H772Y	1p22.1	.
1	100533569	C	T	AGTCACCAGT,CTGCAATTGG	exonic	HIAT1	nonsynonymous SNV	HIAT1:ENST00000370152.3:exon6:c.C523T:p.P175S	1p21.2	.
1	110882612	A	C	ATGAGTTCAA,CGCTTCGGTG	exonic	RBM15	nonsynonymous SNV	RBM15:ENST00000602849.1:exon1:c.A585C:p.K195N,RBM15:ENST00000369784.3:exon1:c.A585C:p.K195N,RBM15:ENST00000487146.2:exon1:c.A585C:p.K195	1p13.3	.
1	110884273	C	G	GAAGACCGCT,TGATGGGAGT	exonic	RBM15	nonsynonymous SNV	RBM15:ENST00000602849.1:exon1:c.C2246G:p.S749C,RBM15:ENST00000369784.3:exon1:c.C2246G:p.S749C,RBM15:ENST00000487146.2:exon1:c.C2246G:p.S749	1p13.3	.
1	111146690	C	G	AAGAACCTCA,CAAGAATTCA	exonic	KCNA2	nonsynonymous SNV	KCNA2:ENST00000369770.3:exon3:c.G715C:p.V239L,KCNA2:ENST00000440270.1:exon2:c.G715C:p.V239L,KCNA2:ENST00000316361.4:exon3:c.G715C:p.V239L,KCNA2 NST00000485317.1:exon3:c.G715C:p.V239L,	1p13.3	.
1	117945045	A	T	TAAGAGAGAA,AGGGAAAAAA	exonic	MAN1A2	nonsynonymous SNV	MAN1A2:ENST00000356554.3:exon2:c.A540T:p.K180N	1p12	.
1	150445031	A	C	GGAACATGGG,CACCCTTCCA	exonic	RPRD2	nonsynonymous SNV	RPRD2:ENST00000369068.4:exon11:c.A3607C:p.T1203P,RPRD2:ENST00000401000.4:exon10:c.A3529C:p.T1177P	1q21.3	.
1	152082242	C	G	AGCAGCTGCT,TTCCTCCTGC	exonic	TCHH	nonsynonymous SNV	TCHH:ENST00000368804.1:exon2:c.G3451C:p.E1151Q	1q21.3	.
1	152128136	T	C	CTGGCCTTGT,TGTCTGGCTG	exonic	RPTN	nonsynonymous SNV	RPTN:ENST00000316073.3:exon3:c.A1439G:p.K480R	1q21.3	RPTN;basecl
1	153903303	C	T	AGGTACAGGG,CTGGCCATAC	exonic	DENND4B	nonsynonymous SNV	DENND4B:ENST00000361217.4:exon26:c.G4145A:p.G1382D	1q21.3	.
1	154306643	G	A	ACCCTCTACT,GAAGGAAAAT	exonic	ATP8B2	stopgain	ATP8B2:ENST00000368487.3:exon10:c.G650A:p.W217X,ATP8B2:ENST00000341822.2:exon9:c.G707A:p.W236X,ATP8B2:ENST00000368489.3:exon10:c.G749A:p.W250X	1q21.3	.
1	154919957	C	T	CAGCAGAAGA,CCATGTTTTG	exonic	PBXIP1	nonsynonymous SNV	PBXIP1:ENST00000368465.1:exon8:c.G719A:p.G240D,PBXIP1:ENST00000368460.3:exon9:c.G713A:p.G238D,PBXIP1:ENST00000542459.1:exon6:c.G341A:p.G114D,PBXIP ENST00000539880.1:exon6:c.G287A:p.G96D,PBXIP1:ENST00000368463.3:exon9:c.G806A:p.G269D,	1q21.3	.
1	156518462	G	A	GGCCACTGCG,GGTTCCTCAA	exonic	IQGAP3	nonsynonymous SNV	IQGAP3:ENST00000361170.2:exon17:c.C1904T:p.P635L,IQGAP3:ENST00000491900.1:exon16:c.C1775T:p.P592L	1q23.1	.
1	158436210	C	T	ATTGTTCAAT,CAATGATTTA	exonic	OR10K1	nonsynonymous SNV	OR10K1:ENST00000289451.2:exon1:c.C859T:p.P287S	1q23.1	.
1	160011650	C	T	TTCTCCCCTT,CTTGGTTTGG	exonic	KCNJ10	nonsynonymous SNV	KCNJ10:ENST00000368089.3:exon2:c.G673A:p.E225K	1q23.2	.
1	161165296	C	G	GCGGGCACTG,AGGGGGACCA	exonic	ADAMTS4	nonsynonymous SNV	ADAMTS4:ENST00000367996.5:exon4:c.G1220C:p.C407S	1q23.3	.
1	172579260	T	C	CGATCTAAAG,CCAAGACCAA	exonic	SUCO	nonsynonymous SNV	SUCO:ENST00000610051.1:exon23:c.T2513C:p.V838A,SUCO:ENST00000367723.4:exon23:c.T4079C:p.V1360A,SUCO:ENST00000263688.3:exon24:c.T3626C:p.V1209A,S CO:ENST00000608151.1:exon23:c.T4082C:p.V1361A,	1q24.3	.
1	174846639	G	A	CAAGATGATG,AAGAAATCTC	exonic	RABGAP1L	nonsynonymous SNV	RABGAP1L:ENST00000478442.1:exon2:c.G7A:p.E3K,RABGAP1L:ENST00000465412.1:exon2:c.G7A:p.E3K,RABGAP1L:ENST00000486220.1:exon2:c.G7A:p.E3K,RABGAP1 :ENST00000392064.2:exon1:c.G7A:p.E3K,	1q25.1	.
1	180012278	C	A	CCAACGGCAG,ACCTTCCAGA	exonic	CEP350	nonsynonymous SNV	CEP350:ENST00000367607.3:exon20:c.C4450A:p.H1484N	1q25.2	.
1	183197562	C	G	CTTTGGTGAA,ATGGCCCAGT	exonic	LAMC2	nonsynonymous SNV	LAMC2:ENST00000264144.4:exon11:c.C1522G:p.H508D,LAMC2:ENST00000493293.1:exon11:c.C1522G:p.H508D	1q25.3	.
1	201181106	C	T	GGAGATGGTT,AGGGAGGCTT	exonic	IGFN1	nonsynonymous SNV	IGFN1:ENST00000335211.4:exon12:c.C7085T:p.S2362L	1q32.1	.
1	203134567	G	T	GTTCGAGAAG,TCATCAGCAT	exonic	ADORA1	nonsynonymous SNV	ADORA1:ENST00000367236.4:exon3:c.G520T:p.V174F,ADORA1:ENST00000309502.3:exon6:c.G520T:p.V174F,ADORA1:ENST00000337894.4:exon4:c.G520T:p.V174	1q32.1	.
1	203148911	T	C	GCTTTCCTGG,CGTCGTATCC	exonic	CHI3L1	nonsynonymous SNV	CHI3L1:ENST00000255409.3:exon9:c.A989G:p.D330G	1q32.1	.
1	206651655	A	T	GCAGTCCTGC,CCACATCTAT	exonic	IKBKE	nonsynonymous SNV	IKBKE:ENST00000537984.1:exon8:c.A710T:p.H237L,IKBKE:ENST00000367120.3:exon9:c.A965T:p.H322L	1q32.1	.
1	206651677	C	A	TCCATGCCCA,AACACGTAAG	exonic	IKBKE	nonsynonymous SNV	IKBKE:ENST00000537984.1:exon8:c.C732A:p.H244Q,IKBKE:ENST00000367120.3:exon9:c.C987A:p.H329Q	1q32.1	.
1	223178283	G	T	AGATCCCAGG,GCCCAAAATC	exonic	DISP1	nonsynonymous SNV	DISP1:ENST00000284476.6:exon8:c.G3544T:p.G1182C	1q41	.
1	228465349	G	A	CACTGACAAC,TGCGCATCCG	exonic	OBSCN	nonsynonymous SNV	OBSCN:ENST00000570156.2:exon29:c.G8023A:p.V2675M,OBSCN:ENST00000366706.2:exon2:c.G280A:p.V94M	1q42.13	.
1	229606446	G	A	TGGTTCTTGA,AACAATGGCG	exonic	NUP133	nonsynonymous SNV	NUP133:ENST00000537506.1:exon16:c.C1909T:p.L637F,NUP133:ENST00000261396.3:exon15:c.C1957T:p.L653F	1q42.13	.
1	231061316	G	A	TTGCATCAGG,ATCCCAAACG	exonic	TTC13	nonsynonymous SNV	TTC13:ENST00000366662.4:exon11:c.C1376T:p.S459F,TTC13:ENST00000366661.4:exon13:c.C1535T:p.S512F,TTC13:ENST00000414259.1:exon11:c.C1376T:p.S459	1q42.2	.
1	241680490	G	A	ACTGGCATGC,TTCTGTCACA	exonic	FH	nonsynonymous SNV	FH:ENST00000366560.3:exon2:c.C259T:p.R87C	1q43	.
1	246755112	C	T	GAGGTGGCTG,AGGTGAGAAC	exonic	CNST	nonsynonymous SNV	CNST:ENST00000366512.3:exon2:c.C248T:p.A83V,CNST:ENST00000366511.1:exon2:c.C248T:p.A83V,CNST:ENST00000366513.4:exon2:c.C248T:p.A83V	1q44	.
1	247902059	T	C	CTCCTCATGA,TCTGGACCAT	exonic	OR14K1	nonsynonymous SNV	OR14K1:ENST00000283225.2:exon1:c.T143C:p.I48T	1q44	.
2	3483137	T	G	CGAGCTGGAG,CCTCACGGAG	exonic	TRAPPC12	nonsynonymous SNV	TRAPPC12:ENST00000382110.2:exon12:c.T2113G:p.S705A,TRAPPC12:ENST00000415624.1:exon7:c.T610G:p.S204A,TRAPPC12:ENST00000324266.5:exon12:c.T2113G:	2p25.3	.
2	17699177	C	T	ATTTCTAATT,CATGTATATC	exonic	RAD51AP2	nonsynonymous SNV	RAD51AP2:ENST00000399080.2:exon1:c.G506A:p.G169E	2p24.2	.
2	27463163	G	C	TGGCATCCCA,GGCTTCCTGA	exonic	CAD	nonsynonymous SNV	CAD:ENST00000403525.1:exon34:c.G5338C:p.G1780R,CAD:ENST00000264705.4:exon35:c.G5527C:p.G1843R	2p23.3	.
2	27804907	G	T	AGGAGCCATC,CAGTCCCTCT	exonic	C2orf16	nonsynonymous SNV	C2orf16:ENST00000408964.2:exon1:c.G5468T:p.R1823L	2p23.3	.
2	31489321	C	G	AGATCTTCTA,ACCCTGTCAC	exonic	EHD3	stopgain	EHD3:ENST00000322054.5:exon6:c.C1359G:p.Y453X	2p23.1	.
2	39081195	C	T	CTTTGTTTTA,CTTTCTTCTG	splicing	DHX57(ENST00000295373.6:exon10:c.2030+1G>A,ENST00000457308.1:exon9:c.17		.	2p22.1	.
2	44566440	T	G	AGAAGTAGTC,TGTTCATAAT	exonic	PREPL	nonsynonymous SNV	PREPL:ENST00000410081.1:exon7:c.A815C:p.K272T,PREPL:ENST00000409957.1:exon6:c.A548C:p.K183T,PREPL:ENST00000541738.1:exon6:c.A548C:p.K183T,PREPL:E ST00000378511.3:exon6:c.A815C:p.K272T,PREPL:ENST00000426481.1:exon6:c.A815C:p.K272T,PREPL:ENST00000425263.1:exon6:c.A815C:p.K272T,PREPL:ENST0000040 9936.1:exon7:c.A815C:p.K272T,PREPL:ENST00000260648.6:exon6:c.A815C:p.K272T,PREPL:ENST00000378520.3:exon6:c.A815C:p.K272T,PREPL:ENST00000409272.1:exo	2p21	.
2	45640334	T	C	CACTGCAGTT,TGCTCTTCTT	exonic	SRBD1	nonsynonymous SNV	SRBD1:ENST00000263736.4:exon19:c.A2432G:p.K811R,SRBD1:ENST00000535761.1:exon11:c.A989G:p.K330R	2p21	.
2	47748351	C	T	TTGCGCCGGG,CAGGGGCGCG	exonic	KCNK12	nonsynonymous SNV	KCNK12:ENST00000327876.4:exon2:c.G988A:p.A330T	2p21	.
2	47748369	C	T	GCGCCAGGAG,CGGGCAGCAG	exonic	KCNK12	nonsynonymous SNV	KCNK12:ENST00000327876.4:exon2:c.G970A:p.A324T	2p21	.
2	55895079	C	T	TATGGATCGG,TTCTGGAAAT	exonic	PNPT1	nonsynonymous SNV	PNPT1:ENST00000447944.2:exon12:c.G991A:p.A331T,PNPT1:ENST00000415374.1:exon12:c.G991A:p.A331T	2p16.1	.
2	69741809	G	T	AGCTGCTGTT,AGAGCCTCCT	exonic	AAK1	nonsynonymous SNV	AAK1:ENST00000406297.3:exon13:c.C1570A:p.Q524K,AAK1:ENST00000409085.4:exon13:c.C1570A:p.Q524K,AAK1:ENST00000409068.1:exon13:c.C1570A:p.Q524	2p13.3	.
2	72362015	C	T	TTCTCCAGCC,CTTCTGCAGG	exonic	CYP26B1	nonsynonymous SNV	CYP26B1:ENST00000474509.1:exon3:c.G511A:p.G171R,CYP26B1:ENST00000001146.2:exon4:c.G736A:p.G246R,CYP26B1:ENST00000546307.1:exon3:c.G511A:p.G171R YP26B1:ENST00000412253.1:exon3:c.G163A:p.G55R,	2p13.2	CYP26B1;ba
2	74328686	A	C	ATCCAGCGAG,AGCTGTTTGG	exonic	TET3	nonsynonymous SNV	TET3:ENST00000409262.3:exon9:c.A4366C:p.K1456Q	2p13.1	.
2	109393639	G	C	GCTCTCATTT,CAGACTTGGC	exonic	RANBP2	nonsynonymous SNV	RANBP2:ENST00000283195.6:exon25:c.G8551C:p.A2851P	2q12.3	.
2	113479673	C	T	CCCCACCTTC,CCACCAGGTG	exonic	NT5DC4	nonsynonymous SNV	NT5DC4:ENST00000327581.4:exon3:c.C196T:p.P66S	2q13	.
2	125204447	C	T	GTGAACTTCA,GGTGGACAAG	exonic	CNTNAP5	nonsynonymous SNV	CNTNAP5:ENST00000431078.1:exon6:c.C851T:p.T284M	2q14.3	.
2	128388468	C	A	GAGGAGTTCT,CTATGAGTTC	exonic	MYO7B	nonsynonymous SNV	MYO7B:ENST00000389524.4:exon35:c.C4844A:p.S1615Y,MYO7B:ENST00000428314.1:exon35:c.C4844A:p.S1615Y,MYO7B:ENST00000409090.1:exon11:c.C1403A:p.S468 MYO7B:ENST00000409816.2:exon34:c.C4844A:p.S1615Y,	2q14.3	.
2	128903449	G	C	AATGCCCTTT,AAAGGGAACA	exonic	UGGT1	nonsynonymous SNV	UGGT1:ENST00000375990.3:exon18:c.G1852C:p.E618Q,UGGT1:ENST00000259253.6:exon18:c.G1924C:p.E642Q	2q14.3	.
2	128944271	C	G	TGCCCAATAA,ATGATTCATC	exonic	UGGT1	nonsynonymous SNV	UGGT1:ENST00000375990.3:exon39:c.C4302G:p.N1434K,UGGT1:ENST00000259253.6:exon39:c.C4374G:p.N1458K	2q14.3	.
2	130877909	C	A	ACTTGCCCAT,TTGCTCCTGA	exonic	POTEF	nonsynonymous SNV	POTEF:ENST00000360967.5:exon1:c.G180T:p.K60N,POTEF:ENST00000357462.5:exon1:c.G180T:p.K60N,POTEF:ENST00000409914.2:exon3:c.G180T:p.K60N,POTEF:EN T00000361163.4:exon1:c.G180T:p.K60N,	2q21.1	.
2	135988122	G	A	TCACAATAAG,TAACTCTGAA	exonic	ZRANB3	nonsynonymous SNV	ZRANB3:ENST00000264159.6:exon13:c.C1915T:p.P639S,ZRANB3:ENST00000401392.1:exon13:c.C1915T:p.P639S,ZRANB3:ENST00000536680.1:exon13:c.C1915T:p.P639	2q21.3	.
2	137748489	T	A	TGAGGTGGTT,TGTATCACAA	exonic	THSD7B	nonsynonymous SNV	THSD7B:ENST00000413152.2:exon1:c.T28A:p.L10M	2q22.1	.
2	145157561	T	A	TTTATAGTCA,TGAAGTCTAG	exonic	ZEB2	nonsynonymous SNV	ZEB2:ENST00000558170.2:exon8:c.A1193T:p.N398I,ZEB2:ENST00000409487.3:exon8:c.A1193T:p.N398I,ZEB2:ENST00000539609.3:exon7:c.A1121T:p.N374I,ZEB2:ENST 000427902.1:exon8:c.A1193T:p.N398I,ZEB2:ENST00000303660.4:exon8:c.A1193T:p.N398I,ZEB2:ENST00000392861.2:exon8:c.A1193T:p.N398I,	2q22.3	.
2	162091890	C	T	GACAGTGACT,GGTGGTACTA	exonic	TANK	nonsynonymous SNV	TANK:ENST00000439442.1:exon3:c.C458T:p.S153L,TANK:ENST00000392749.2:exon8:c.C1139T:p.S380L,TANK:ENST00000405852.1:exon9:c.C1216T:p.R406W,TANK:EN T00000259075.2:exon8:c.C1139T:p.S380L,	2q24.2	TANK;basech
2	166872235	T	G	TTTCATTCAG,TTCTGTAAGT	exonic	SCN1A	nonsynonymous SNV	SCN1A:ENST00000423058.2:exon17:c.A3432C:p.K1144N,SCN1A:ENST00000409050.1:exon17:c.A3348C:p.K1116N,SCN1A:ENST00000375405.3:exon17:c.A3399C:p.K1133 ,SCN1A:ENST00000303395.4:exon17:c.A3432C:p.K1144N,	2q24.3	.
2	174125792	C	T	GTTTTTCAGC,ACCATTTGTA	exonic	MLTK	nonsynonymous SNV	MLTK:ENST00000375213.3:exon18:c.C1478T:p.P493L,MLTK:ENST00000409176.2:exon18:c.C1478T:p.P493L	2q31.1	.
2	179615431	C	T	CTTTTTTATT,TTTCACTAGC	exonic	TTN	nonsynonymous SNV	TTN:ENST00000360870.5:exon46:c.G11696A:p.R3899K	2q31.2	.
2	179641201	T	A	AAGACTTTTC,CATCTTTAAC	exonic	TTN	nonsynonymous SNV	TTN:ENST00000359218.5:exon27:c.A5252T:p.E1751V,TTN:ENST00000342175.6:exon27:c.A5252T:p.E1751V,TTN:ENST00000591111.1:exon28:c.A5390T:p.E1797V,TTN:E ST00000589042.1:exon28:c.A5390T:p.E1797V,TTN:ENST00000460472.2:exon27:c.A5252T:p.E1751V,TTN:ENST00000342992.6:exon28:c.A5390T:p.E1797V,TTN:ENST0000	2q31.2	.
2	186670315	A	T	AAACACCTTT,CACAAATAAG	exonic	FSIP2	nonsynonymous SNV	FSIP2:ENST00000343098.5:exon17:c.A16549T:p.T5517S,FSIP2:ENST00000424728.1:exon17:c.A16282T:p.T5428S	2q32.1	.
2	186670316	C	T	AACACCTTTA,ACAAATAAGC	exonic	FSIP2	nonsynonymous SNV	FSIP2:ENST00000343098.5:exon17:c.C16550T:p.T5517I,FSIP2:ENST00000424728.1:exon17:c.C16283T:p.T5428	2q32.1	.
2	198257848	A	C	TCACAACCAA,TCCATAAACC	exonic	SF3B1	nonsynonymous SNV	SF3B1:ENST00000335508.6:exon24:c.T3604G:p.F1202V	2q33.1	.
2	206870021	C	T	ACTATACGCC,ATTCAATAGC	exonic	INO80D	nonsynonymous SNV	INO80D:ENST00000403263.1:exon11:c.G2155A:p.G719R	2q33.3	INO80D;base
2	227984657	T	C	AGTTGGACCC,AAAATCCCAG	splicing	COL4A4(ENST00000396625.3:exon7:c.328-2A>G,ENST00000329662.7:exon7:c.328		.	2q36.3	.
3	5252810	G	A	TGTAGGTGTG,GTACGCCACG	exonic	EDEM1	nonsynonymous SNV	EDEM1:ENST00000445686.1:exon10:c.G1004A:p.G335E,EDEM1:ENST00000256497.4:exon10:c.G1589A:p.G530E	3p26.1	.
3	9831632	G	T	TTCTTATCCT,CCAGTCGGTG	exonic	TADA3	nonsynonymous SNV	TADA3:ENST00000301964.2:exon3:c.C223A:p.Q75K,TADA3:ENST00000343450.2:exon3:c.C223A:p.Q75K,TADA3:ENST00000440161.1:exon3:c.C223A:p.Q75K,TADA3:ENS 00000439043.1:exon3:c.C223A:p.Q75K,	3p25.3	.
3	14211956	C	T	TCTTCCCAAT,ATTTTCACTT	exonic	XPC	nonsynonymous SNV	XPC:ENST00000476581.2:exon3:c.G394A:p.D132N,XPC:ENST00000449060.2:exon3:c.G394A:p.D132N,XPC:ENST00000511155.1:exon3:c.G376A:p.D126N,XPC:ENST000	3p25.1	.
3	16306305	G	A	TCTCCACATG,GCAGGGATAG	exonic	DPH3	nonsynonymous SNV	DPH3:ENST00000383775.4:exon1:c.C79T:p.P27S,DPH3:ENST00000488423.1:exon1:c.C79T:p.P27S	3p25.1	.
3	32022583	G	A	GCAGGAGGGC,AGGAGCCCGC	exonic	OSBPL10	nonsynonymous SNV	OSBPL10:ENST00000396556.2:exon1:c.C89T:p.S30L,OSBPL10:ENST00000438237.2:exon1:c.C89T:p.S30L	3p23	.
3	37367249	C	T	CTAAATATTT,TTTTCAACAG	exonic	GOLGA4	nonsynonymous SNV	GOLGA4:ENST00000361924.2:exon14:c.C3872T:p.S1291F,GOLGA4:ENST00000437131.1:exon12:c.C3485T:p.S1162F,GOLGA4:ENST00000356847.4:exon15:c.C3938T:p.S	3p22.2	.
3	42242395	C	T	CATGAACATC,CCGGCTCCAA	exonic	TRAK1	nonsynonymous SNV	TRAK1:ENST00000449246.1:exon11:c.C1054T:p.P352S,TRAK1:ENST00000341421.3:exon11:c.C1102T:p.P368S,TRAK1:ENST00000327628.5:exon12:c.C1276T:p.P426S,T AK1:ENST00000396175.1:exon11:c.C1102T:p.P368S,TRAK1:ENST00000427771.1:exon4:c.C430T:p.P144S,	3p22.1	.
3	42264786	G	A	CCCTCCCTAC,ACAATTTCCT	exonic	TRAK1	nonsynonymous SNV	TRAK1:ENST00000327628.5:exon16:c.G2419A:p.D807N,TRAK1:ENST00000396175.1:exon15:c.G2245A:p.D749N	3p22.1	.
3	44776017	C	G	ACTCAGAGAT,ATCTCTTACC	exonic	ZNF501	stopgain	ZNF501:ENST00000396048.2:exon3:c.C104G:p.S35X	3p21.31	.
3	47950858	C	A	GGCAAGGAAA,GCTTTCCAAT	exonic	MAP4	nonsynonymous SNV	MAP4:ENST00000383736.3:exon1:c.G874T:p.V292F,MAP4:ENST00000426837.2:exon10:c.G5104T:p.V1702F,MAP4:ENST00000264724.11:exon1:c.G874T:p.V292	3p21.31	.
3	48611148	C	T	TCCAGGGTCT,CATGACCACC	exonic	COL7A1	nonsynonymous SNV	COL7A1:ENST00000454817.1:exon80:c.G6452A:p.G2151E,COL7A1:ENST00000328333.8:exon81:c.G6548A:p.G2183E	3p21.31	.
3	48611149	C	T	CCAGGGTCTC,ATGACCACCC	exonic	COL7A1	nonsynonymous SNV	COL7A1:ENST00000454817.1:exon80:c.G6451A:p.G2151R,COL7A1:ENST00000328333.8:exon81:c.G6547A:p.G2183R	3p21.31	.
3	49152305	A	C	CTCAGCTACC,CCTGAGAGGC	exonic	USP19	nonsynonymous SNV	USP19:ENST00000434032.2:exon15:c.T2177G:p.V726G,USP19:ENST00000398898.2:exon14:c.T1994G:p.V665G,USP19:ENST00000453664.1:exon15:c.T2147G:p.V716G,U P19:ENST00000417901.1:exon15:c.T2183G:p.V728G,USP19:ENST00000398888.2:exon14:c.T1874G:p.V625G,USP19:ENST00000398892.3:exon15:c.T1994G:p.V665G,USP1	3p21.31	.
3	49153297	G	A	CCTGTCAGGG,GTGGGGAGCA	exonic	USP19	nonsynonymous SNV	USP19:ENST00000434032.2:exon11:c.C1546T:p.P516S,USP19:ENST00000398898.2:exon10:c.C1363T:p.P455S,USP19:ENST00000453664.1:exon11:c.C1516T:p.P506S,US 19:ENST00000417901.1:exon11:c.C1552T:p.P518S,USP19:ENST00000398888.2:exon10:c.C1243T:p.P415S,USP19:ENST00000398892.3:exon11:c.C1363T:p.P455S,USP19:E NST00000306026.5:exon11:c.C1507T:p.P503S,USP19:ENST00000398896.1:exon8:c.C667T:p.P223S,	3p21.31	.
3	49694275	A	T	GTGCTGCAGC,GCAGCAAGAG	exonic	BSN	nonsynonymous SNV	BSN:ENST00000296452.4:exon5:c.A7286T:p.Q2429L	3p21.31	.
3	52283238	G	A	GGATGATGTG,TTGTCATGGC	exonic	PPM1M	nonsynonymous SNV	PPM1M:ENST00000409502.3:exon8:c.G499A:p.V167I,PPM1M:ENST00000323588.4:exon9:c.G652A:p.V218I,PPM1M:ENST00000296487.4:exon9:c.G652A:p.V218I,PPM1M:E ST00000457351.2:exon9:c.G1135A:p.V379I,	3p21.2	.
3	52457226	C	T	GACTGATTGG,CAGAACCTTC	exonic	PHF7	nonsynonymous SNV	PHF7:ENST00000347025.2:exon10:c.C922T:p.P308S,PHF7:ENST00000327906.3:exon11:c.C1039T:p.P347S	3p21.1	.
3	52457230	A	C	GATTGGCCAG,ACCTTCCTTA	exonic	PHF7	nonsynonymous SNV	PHF7:ENST00000347025.2:exon10:c.A926C:p.E309A,PHF7:ENST00000327906.3:exon11:c.A1043C:p.E348A	3p21.1	.
3	62636688	G	A	CAGGTTGACA,ATGACTTCAG	exonic	CADPS	nonsynonymous SNV	CADPS:ENST00000490353.2:exon5:c.C1037T:p.S346F,CADPS:ENST00000283269.9:exon5:c.C1037T:p.S346F,CADPS:ENST00000383710.4:exon5:c.C1037T:p.S346F,CAD S:ENST00000357948.3:exon5:c.C1037T:p.S346F,	3p14.2	.
3	63975935	C	T	CCTCCCCTGC,TGCCACTGAG	exonic	ATXN7	nonsynonymous SNV	ATXN7:ENST00000398590.3:exon10:c.C1445T:p.P482L,ATXN7:ENST00000487717.1:exon9:c.C1445T:p.P482L,ATXN7:ENST00000484332.1:exon6:c.C1010T:p.P337L,ATXN ENST00000295900.6:exon10:c.C1445T:p.P482L,ATXN7:ENST00000538065.1:exon8:c.C1445T:p.P482L,	3p14.1	.
3	63981343	C	A	CCCCAAATAG,AAATCGGTAC	exonic	ATXN7	nonsynonymous SNV	ATXN7:ENST00000398590.3:exon12:c.C1845A:p.S615R,ATXN7:ENST00000487717.1:exon11:c.C1845A:p.S615R,ATXN7:ENST00000484332.1:exon8:c.C1410A:p.S470R,AT N7:ENST00000295900.6:exon12:c.C1845A:p.S615R,ATXN7:ENST00000538065.1:exon10:c.C1845A:p.S615R,	3p14.1	.
3	107097354	G	A	ACCACAATCT,TCTCTCCATC	exonic	CCDC54	nonsynonymous SNV	CCDC54:ENST00000261058.1:exon1:c.G920A:p.C307Y	3q13.12	.
3	112736411	C	T	GACAGTATGA,GAGATGGCAT	exonic	C3orf17	nonsynonymous SNV	C3orf17:ENST00000383675.2:exon2:c.G145A:p.V49I,C3orf17:ENST00000314400.5:exon2:c.G145A:p.V49I,C3orf17:ENST00000496340.1:exon2:c.G145A:p.V49I,C3orf17:ENS 00000461381.1:exon2:c.G145A:p.V49I,C3orf17:ENST00000496206.1:exon2:c.G142A:p.V48I,	3q13.2	.
3	118865928	G	A	GTATGGCCTC,TTGACCACAA	exonic	C3orf30	nonsynonymous SNV	C3orf30:ENST00000295622.1:exon1:c.G892A:p.V298I,C3orf30:ENST00000494105.2:exon1:c.G892A:p.V298	3q13.32	.
3	119892161	C	T	GAGCTTACTT,TGTGAGGAGC	exonic	GPR156	nonsynonymous SNV	GPR156:ENST00000464295.1:exon9:c.G1090A:p.E364K,GPR156:ENST00000315843.3:exon8:c.G1090A:p.E364K,GPR156:ENST00000461057.1:exon8:c.G1078A:p.E360	3q13.33	.
3	120371448	G	C	CACTCACAAA,TCTACTTTCT	exonic	HGD	nonsynonymous SNV	HGD:ENST00000283871.5:exon5:c.C333G:p.D111E,HGD:ENST00000476082.2:exon4:c.C210G:p.D70E	3q13.33	.
3	127390401	C	A	ATCATCATTG,GCTTGGCCTG	exonic	PODXL2	nonsynonymous SNV	PODXL2:ENST00000342480.6:exon7:c.C1550A:p.A517E	3q21.3	.
3	133099571	C	T	CTTACTGTTA,CCCTGGCACC	exonic	TMEM108	nonsynonymous SNV	TMEM108:ENST00000321871.6:exon4:c.C1016T:p.T339I,TMEM108:ENST00000515826.1:exon3:c.C1016T:p.T339I,TMEM108:ENST00000393130.3:exon4:c.C1016T:p.T339	3q22.1	.
3	151155444	T	G	AAGCCTCACA,TCCTAATTTC	exonic	IGSF10	nonsynonymous SNV	IGSF10:ENST00000282466.3:exon6:c.A6905C:p.N2302T	3q25.1	.
3	158980427	G	A	CAGAGAAGCT,AGCAGCTCTG	exonic	IQCJ,IQCJ-SCHIP1	nonsynonymous SNV	IQCJ-SCHIP1:ENST00000471575.1:exon4:c.G238A:p.E80K	3q25.32	.
3	159986212	A	T	CCTTCTGCAT,ATGCAAGTAT	exonic	IFT80,RP11-432B6.3	nonsynonymous SNV	IFT80:ENST00000326448.7:exon19:c.T2212A:p.Y738N,RP11 432B6.3:ENST00000483754.1:exon17:c.T2725A:p.Y909N,IFT80:ENST00000496589.1:exon20:c.T1801A:p.Y601N,IFT80:ENST00000483465.1:exon18:c.T1801A:p.Y601N,	3q25.33	.
3	170724939	G	A	GTTTTTACCT,ACTAATAAGA	exonic	SLC2A2	stopgain	SLC2A2:ENST00000382808.4:exon4:c.C253T:p.Q85X,SLC2A2:ENST00000461867.1:exon2:c.C91T:p.Q31X,SLC2A2:ENST00000314251.3:exon5:c.C610T:p.Q204	3q26.2	.
3	182925404	T	C	ACCTTCATGG,CTTCTTGAAG	exonic	MCF2L2	nonsynonymous SNV	MCF2L2:ENST00000473233.1:exon23:c.A2704G:p.T902A,MCF2L2:ENST00000328913.3:exon23:c.A2704G:p.T902A	3q27.1	.
3	184086043	C	T	TCGAGGTGGA,TCCAGAGTTT	exonic	POLR2H	nonsynonymous SNV	POLR2H:ENST00000429568.1:exon5:c.C478T:p.L160F	3q27.1	.
3	190347293	A	T	GAAAGGTAAT,GATGCGGTCA	exonic	IL1RAP	stopgain	IL1RAP:ENST00000422485.1:exon9:c.A1057T:p.R353X,IL1RAP:ENST00000422940.1:exon8:c.A1057T:p.R353X,IL1RAP:ENST00000434491.1:exon6:c.A634T:p.R212	3q28	.
3	196612688	G	T	GGGGACCCTT,ATTCCAAAAA	exonic	SENP5	nonsynonymous SNV	SENP5:ENST00000445299.2:exon2:c.G636T:p.L212F,SENP5:ENST00000323460.5:exon2:c.G636T:p.L212F	3q29	.
4	1388429	G	A	CGGAGTGCCC,CCTGCTCACA	exonic	CRIPAK	nonsynonymous SNV	CRIPAK:ENST00000324803.4:exon1:c.G130A:p.A44T	4p16.3	CRIPAK;base
4	1803443	C	T	TGGCAGCATC,GGCAGACGTA	exonic	FGFR3	nonsynonymous SNV	FGFR3:ENST00000260795.2:exon5:c.C712T:p.R238W,FGFR3:ENST00000481110.2:exon6:c.C712T:p.R238W,FGFR3:ENST00000440486.2:exon6:c.C712T:p.R238W,FGFR3 ENST00000507588.1:exon2:c.C172T:p.R58W,FGFR3:ENST00000412135.2:exon6:c.C712T:p.R238W,FGFR3:ENST00000340107.4:exon6:c.C712T:p.R238W,FGFR3:ENST000	4p16.3	.
4	1824003	G	A	CCCGGCCTTT,GGGAGCAGCT	exonic	LETM1	stopgain	LETM1:ENST00000302787.2:exon10:c.C1513T:p.Q505X	4p16.3	.
4	13602114	T	C	CTCATCTTTC,CTTCACTTCT	exonic	BOD1L1	nonsynonymous SNV	BOD1L1:ENST00000040738.5:exon10:c.A6410G:p.E2137G	4p15.33	.
4	13602119	A	C	CTTTCTCTTC,CTTCTGCTGG	exonic	BOD1L1	nonsynonymous SNV	BOD1L1:ENST00000040738.5:exon10:c.T6405G:p.S2135R	4p15.33	.
4	15575932	G	A	GGTTCCTGGA,AGTCCATTCG	exonic	CC2D2A	nonsynonymous SNV	CC2D2A:ENST00000503292.1:exon30:c.G3754A:p.E1252K,CC2D2A:ENST00000424120.1:exon29:c.G3754A:p.E1252K,CC2D2A:ENST00000413206.1:exon29:c.G3754A:p.E	4p15.32	.
4	20547673	T	A	TGGAAACCAA,TTACACTGGT	exonic	SLIT2	nonsynonymous SNV	SLIT2:ENST00000503837.1:exon22:c.T2284A:p.F762I,SLIT2:ENST00000273739.5:exon23:c.T2308A:p.F770I,SLIT2:ENST00000503823.1:exon21:c.T2272A:p.F758I,SLIT2:EN T00000504154.1:exon22:c.T2296A:p.F766I,	4p15.31	.
4	20555577	A	T	CCCTCCAAAA,ATTTACCTGT	exonic	SLIT2	nonsynonymous SNV	SLIT2:ENST00000511508.1:exon6:c.A314T:p.K105I,SLIT2:ENST00000503837.1:exon26:c.A2699T:p.K900I,SLIT2:ENST00000273739.5:exon27:c.A2723T:p.K908I,SLIT2:ENS 00000503823.1:exon25:c.A2687T:p.K896I,SLIT2:ENST00000504154.1:exon26:c.A2711T:p.K904I,	4p15.31	.
4	49032891	G	C	AGCCCTATAT,GGGAACAATG	exonic	CWH43	nonsynonymous SNV	CWH43:ENST00000513409.1:exon11:c.G1341C:p.M447I,CWH43:ENST00000226432.4:exon11:c.G1422C:p.M474	4p11	.
4	52951137	G	T	AGTCCCATTT,CCCCCGTAGC	exonic	SPATA18	nonsynonymous SNV	SPATA18:ENST00000419395.2:exon10:c.G1439T:p.C480F,SPATA18:ENST00000295213.4:exon11:c.G1535T:p.C512F	4q12	.
4	74353509	G	T	GGGCACTTTT,AAATTTGGAA	exonic	AFM	nonsynonymous SNV	AFM:ENST00000226355.3:exon6:c.G684T:p.L228F	4q13.3	.
4	76452419	T	A	GGACTATATT,CATGAAATAA	exonic	THAP6	nonsynonymous SNV	THAP6:ENST00000514480.1:exon5:c.T664A:p.S222T,THAP6:ENST00000380837.3:exon4:c.T538A:p.S180T,THAP6:ENST00000311638.3:exon5:c.T664A:p.S222T	4q21.1	.
4	76721531	G	T	AGAAAATCTT,GAGAAGAAGA	exonic	USO1	stopgain	USO1:ENST00000514213.2:exon15:c.G1546T:p.G516X,USO1:ENST00000538159.1:exon16:c.G1618T:p.G540X	4q21.1	.
4	84205872	C	A	GCCAGTGCCA,AGCCCGCAGG	exonic	COQ2	nonsynonymous SNV	COQ2:ENST00000311469.4:exon1:c.G196T:p.V66L,COQ2:ENST00000439031.2:exon1:c.G85T:p.V29L,COQ2:ENST00000503391.1:exon1:c.G46T:p.V16L,COQ2:ENST0000	4q21.23	.
4	84367181	A	G	AGTGTAGAGG,TTTTTGCTAG	exonic	HELQ	nonsynonymous SNV	HELQ:ENST00000510985.1:exon4:c.T1250C:p.I417T,HELQ:ENST00000508591.1:exon5:c.T1451C:p.I484T,HELQ:ENST00000295488.3:exon5:c.T1451C:p.I484	4q21.23	.
4	84525837	A	T	AGGACTAAAG,GAGCAAAGGT	exonic	AGPAT9	stopgain	AGPAT9:ENST00000264409.4:exon12:c.A1222T:p.R408X,AGPAT9:ENST00000395226.2:exon13:c.A1222T:p.R408X	4q21.23	.
4	100540189	T	G	GGTCTAGCTA,TGATATTTCA	exonic	MTTP	nonsynonymous SNV	MTTP:ENST00000457717.1:exon17:c.T2276G:p.I759S,MTTP:ENST00000511045.1:exon16:c.T2357G:p.I786S,MTTP:ENST00000265517.5:exon16:c.T2276G:p.I759S	4q23	.
4	106197536	C	G	TGCTGAGCCA,ATGAAACTTC	exonic	TET2	nonsynonymous SNV	TET2:ENST00000380013.4:exon11:c.C5869G:p.H1957D,TET2:ENST00000513237.1:exon11:c.C5932G:p.H1978D,TET2:ENST00000540549.1:exon11:c.C5869G:p.H1957D	4q24	.
4	109774063	C	G	GTTACCGTGA,AGTTAAGGTG	exonic	COL25A1	nonsynonymous SNV	COL25A1:ENST00000494183.1:exon22:c.G1123C:p.V375L,COL25A1:ENST00000399126.1:exon25:c.G1339C:p.V447L,COL25A1:ENST00000399132.1:exon25:c.G1339C:p.V	4q25	.
4	110882079	C	T	TAAAAACACC,CTGGATCCTA	exonic	EGF	nonsynonymous SNV	EGF:ENST00000503392.1:exon7:c.C1123T:p.P375S,EGF:ENST00000265171.5:exon7:c.C1123T:p.P375S,EGF:ENST00000509793.1:exon6:c.C997T:p.P333	4q25	.
4	114290798	C	T	CAGACCCCAA,ATCCAGCGAG	exonic	ANK2	nonsynonymous SNV	ANK2:ENST00000505342.1:exon6:c.C2477T:p.T826I,ANK2:ENST00000357077.4:exon43:c.C11447T:p.T3816I,ANK2:ENST00000264366.6:exon42:c.C11348T:p.T3783I,ANK ENST00000510275.2:exon10:c.C1148T:p.T383I,ANK2:ENST00000509550.1:exon21:c.C2720T:p.T907I,ANK2:ENST00000506722.1:exon44:c.C5165T:p.T1722I,ANK2:ENST000	4q26	.
4	134076158	C	T	CATGATGCCA,CAACCGTGCC	exonic	PCDH10	nonsynonymous SNV	PCDH10:ENST00000264360.5:exon3:c.C2777T:p.T926I	4q28.3	.
4	143159099	G	A	ATCTGCTCTC,AATTCGCATC	exonic	INPP4B	stopgain	INPP4B:ENST00000513000.1:exon13:c.C754T:p.R252X,INPP4B:ENST00000308502.4:exon9:c.C754T:p.R252X,INPP4B:ENST00000510812.1:exon11:c.C754T:p.R252X,INPP B:ENST00000514525.1:exon5:c.C367T:p.R123X,INPP4B:ENST00000512630.1:exon11:c.C754T:p.R252X,INPP4B:ENST00000509777.1:exon12:c.C754T:p.R252X,INPP4B:ENS T00000262992.4:exon10:c.C754T:p.R252X,INPP4B:ENST00000508116.1:exon11:c.C754T:p.R252X,INPP4B:ENST00000511838.1:exon5:c.C199T:p.R67X,	4q31.21	.
4	152550936	T	A	CTACTTGAGA,CTTCCTCCGT	exonic	FAM160A1	nonsynonymous SNV	FAM160A1:ENST00000435205.1:exon8:c.T1061A:p.I354N,FAM160A1:ENST00000505231.1:exon6:c.T1061A:p.I354N	4q31.3	.
4	153332686	G	C	TTCCTGAGGA,TCCTCATCTA	exonic	FBXW7	nonsynonymous SNV	FBXW7:ENST00000604872.1:exon4:c.C270G:p.D90E,FBXW7:ENST00000281708.4:exon2:c.C270G:p.D90E,FBXW7:ENST00000605042.1:exon2:c.C270G:p.D90E,FBXW7:E ST00000603548.1:exon3:c.C270G:p.D90E,FBXW7:ENST00000603841.1:exon1:c.C270G:p.D90E,	4q31.3	.
4	187629061	G	A	CTCAGACTGT,GAAAGACACC	exonic	FAT1	nonsynonymous SNV	FAT1:ENST00000441802.2:exon2:c.C1921T:p.H641Y	4q35.2	.
5	14471569	G	A	TCTTTAAAGA,AAGACAGTAA	exonic	TRIO	nonsynonymous SNV	TRIO:ENST00000537187.1:exon38:c.G5906A:p.R1969K,TRIO:ENST00000513206.1:exon34:c.G5105A:p.R1702K,TRIO:ENST00000344204.4:exon38:c.G5906A:p.R1969K,TI O:ENST00000512070.2:exon38:c.G5729A:p.R1910K,	5p15.2	.
5	59982992	G	T	CATAGCTCTT,AAACGACAGC	exonic	DEPDC1B	nonsynonymous SNV	DEPDC1B:ENST00000265036.5:exon2:c.C111A:p.F37L,DEPDC1B:ENST00000545085.1:exon3:c.C30A:p.F10L,DEPDC1B:ENST00000453022.2:exon2:c.C111A:p.F37	5q12.1	.
5	64824833	C	T	TTCATCCAAC,ACCGTTGTTC	exonic	CENPK	stopgain	CENPK:ENST00000508421.1:exon8:c.G293A:p.W98X,CENPK:ENST00000515497.1:exon8:c.G389A:p.W130X,CENPK:ENST00000502997.1:exon6:c.G383A:p.W128X,CENPK ENST00000514814.1:exon8:c.G383A:p.W128X,CENPK:ENST00000242872.3:exon6:c.G383A:p.W128X,CENPK:ENST00000396679.1:exon8:c.G383A:p.W128X,	5q12.3	.
5	73932057	C	T	GTTGACCTCA,TGTCCTGGCT	exonic	ENC1	nonsynonymous SNV	ENC1:ENST00000510316.1:exon2:c.G35A:p.S12N,ENC1:ENST00000537006.1:exon3:c.G254A:p.S85N,ENC1:ENST00000302351.4:exon2:c.G254A:p.S85N	5q13.3	.

5	74400503	G	C	CATGGTCTTT,TCTGTAGCTT	exonic	ANKRD31	nonsynonymous SNV	ANKRD31:ENST00000506364.2:exon22:c.C4881G:p.D1627E,ANKRD31:ENST00000274361.3:exon21:c.C4710G:p.D1570E	5q13.3	.
5	79033487	C	A	TTCAATCGAT,AGGAAGAAAG	exonic	CMYA5	nonsynonymous SNV	CMYA5:ENST00000446378.2:exon2:c.C8899A:p.Q2967K	5q14.1	.
5	110820000	G	A	GGTGCCCAAG,CAGTGGAGGA	exonic	CAMK4	nonsynonymous SNV	CAMK4:ENST00000512453.1:exon12:c.G1258A:p.A420T,CAMK4:ENST00000282356.4:exon11:c.G1258A:p.A420T	5q22.1	.
5	130766998	G	T	CACAGACGAT,AGACAGCTAA	exonic	CTC-432M15.3,RAPGEF6	stopgain	RAPGEF6:ENST00000509018.1:exon26:c.C4019A:p.S1340X,RAPGEF6:ENST00000307984.5:exon28:c.C4058A:p.S1353X,RAPGEF6:ENST00000296859.6:exon27:c.C4043A<br>.S1348X,CTC-432M15.3:ENST00000514667.1:exon27:c.C4169A:p.S1390X,RAPGEF6:ENST00000507093.1:exon27:c.C4043A:p.S1348X,	5q31.1	.
5	133534805	C	A	TCGTCAAGTT,CATGATTGCA	exonic	PPP2CA	stopgain	PPP2CA:ENST00000481195.1:exon6:c.G829T:p.E277X	5q31.1	.
5	135496703	T	C	TTTTCCCTTA,CTCCAAACAG	exonic	SMAD5	nonsynonymous SNV	SMAD5:ENST00000545620.1:exon3:c.T562C:p.S188P,SMAD5:ENST00000545279.1:exon4:c.T562C:p.S188P	5q31.1	.
5	140348795	C	A	GGGGCCCAGA,AGGACCAGGG	exonic	PCDHAC2	nonsynonymous SNV	PCDHAC2:ENST00000289269.5:exon1:c.C2444A:p.T815K	5q31.3	.
5	140730615	C	T	CGGGTGATGG,CACAGACCAG	exonic	PCDHGB1	nonsynonymous SNV	PCDHGB1:ENST00000523390.1:exon1:c.C788T:p.A263V	5q31.3	.
5	140742062	G	A	AATGCCTCTT,GGAACAAAAT	exonic	PCDHGB2	stopgain	PCDHGB2:ENST00000522605.1:exon1:c.G2360A:p.W787X	5q31.3	.
5	140783631	C	G	CTTTTCAATG,TCATGACCGA	exonic	PCDHGA9	nonsynonymous SNV	PCDHGA9:ENST00000573521.1:exon1:c.C1112G:p.A371G	5q31.3	.
5	140783996	G	A	CTCCTTGGCA,AGGATACCAT	exonic	PCDHGA9	nonsynonymous SNV	PCDHGA9:ENST00000573521.1:exon1:c.G1477A:p.E493K	5q31.3	.
5	150924597	C	T	TCCTGCTGCT,CCGGTCAAAC	exonic	FAT2	nonsynonymous SNV	FAT2:ENST00000261800.5:exon9:c.G6091A:p.E2031K	5q33.1	.
5	150942951	C	T	GGTCAGCTTC,CTTTGGAGCT	exonic	FAT2	nonsynonymous SNV	FAT2:ENST00000261800.5:exon2:c.G3509A:p.G1170E	5q33.1	.
5	153857106	G	C	TCGCCAGACT,TGCATCCTTG	exonic	HAND1	nonsynonymous SNV	HAND1:ENST00000231121.2:exon1:c.C463G:p.Q155E	5q33.2	.
5	159707642	G	A	CCGGGCTGCA,GGCAGAGCTG	exonic	CCNJL	nonsynonymous SNV	CCNJL:ENST00000541762.1:exon3:c.C167T:p.P56L,CCNJL:ENST00000393977.3:exon3:c.C170T:p.P57L,CCNJL:ENST00000257536.7:exon3:c.C170T:p.P57L,CCNJL:ENST<br>0000519673.1:exon3:c.C170T:p.P57L,CCNJL:ENST00000520748.1:exon3:c.C275T:p.P92L,CCNJL:ENST00000505287.2:exon3:c.C305T:p.P102L,	5q33.3	.
5	169139226	A	T	GTTGTCCTCA,GGTACCATGA	exonic	DOCK2	nonsynonymous SNV	DOCK2:ENST00000256935.8:exon17:c.A1658T:p.K553M,DOCK2:ENST00000524185.1:exon17:c.A1658T:p.K553M	5q35.1	.
5	179028914	G	A	GCTTCAGGAC,AGAAGGCAGA	exonic	RUFY1	nonsynonymous SNV	RUFY1:ENST00000319449.4:exon15:c.G1783A:p.E595K,RUFY1:ENST00000393438.2:exon15:c.G1459A:p.E487K,RUFY1:ENST00000437570.2:exon14:c.G1459A:p.E487	5q35.3	.
6	4069925	C	T	AATGTTTCAC,ATCATTGTTT	exonic	FAM217A	nonsynonymous SNV	FAM217A:ENST00000274673.3:exon7:c.G532A:p.G178S	6p25.2	.
6	10687725	C	A	TTGCCAGTAG,AGTAATAGTT	exonic	C6orf52	nonsynonymous SNV	C6orf52:ENST00000426700.2:exon1:c.G59T:p.C20F,C6orf52:ENST00000259983.3:exon2:c.G59T:p.C20F	6p24.2	.
6	17837261	T	A	TCTTCATAGT,GTCTGCGGCT	exonic	KIF13A	nonsynonymous SNV	KIF13A:ENST00000378826.2:exon11:c.A1003T:p.N335Y,KIF13A:ENST00000378843.2:exon11:c.A1003T:p.N335Y,KIF13A:ENST00000259711.6:exon11:c.A1003T:p.N335Y,K<br>13A:ENST00000378816.5:exon11:c.A1003T:p.N335Y,KIF13A:ENST00000378814.5:exon11:c.A1003T:p.N335Y,	6p22.3	.
6	26273414	T	G	CAACGACATT,TCGAGCGCAT	exonic	HIST1H2BI	nonsynonymous SNV	HIST1H2BI:ENST00000377733.2:exon1:c.T211G:p.F71V	6p22.2	.
6	28293984	G	A	TGTTTCTTAA,AAGTGTCCGC	exonic	ZSCAN31	nonsynonymous SNV	ZSCAN31:ENST00000396838.2:exon8:c.C1180T:p.L394F,ZSCAN31:ENST00000439158.1:exon4:c.C1180T:p.L394F,ZSCAN31:ENST00000446474.1:exon3:c.C703T:p.L235F<br>SCAN31:ENST00000344279.6:exon4:c.C1180T:p.L394F,ZSCAN31:ENST00000414429.1:exon8:c.C1180T:p.L394F,	6p22.1	.
6	30624011	T	G	CAGCATGGGG,CCACCGGCAG	exonic	DHX16	nonsynonymous SNV	DHX16:ENST00000376442.3:exon16:c.A2462C:p.D821A,DHX16:ENST00000376437.5:exon8:c.A1019C:p.D340A	6p21.33	.
6	30653074	C	T	GTCAGGTCTC,ATTTATGGGC	exonic	PPP1R18	stopgain	PPP1R18:ENST00000399199.3:exon2:c.G722A:p.W241X,PPP1R18:ENST00000274853.3:exon1:c.G722A:p.W241X	6p21.33	.
6	30994298	A	T	TATCACAGGC,CTGAGACCAC	exonic	MUC22	nonsynonymous SNV	MUC22:ENST00000561890.1:exon2:c.A1090T:p.T364S	6p21.33	.
6	31685394	T	A	CCAGCCTGCG,CGCAGCCCAT	exonic	LY6G6D,LY6G6F,MEGT1	nonsynonymous SNV	LY6G6D:ENST00000375825.3:exon3:c.T215A:p.V72D,LY6G6F:ENST00000556581.1:exon6:c.T962A:p.V321D,MEGT1:ENST00000503322.1:exon6:c.T962A:p.V321	6p21.33	.
6	32056634	C	T	ACCACATATT,TACGCCTGGC	exonic	TNXB	nonsynonymous SNV	TNXB:ENST00000375244.3:exon6:c.G2707A:p.E903K,TNXB:ENST00000375247.2:exon6:c.G2707A:p.E903K	6p21.33	.
6	32632589	A	G	CCTCTGCAAG,TCCCGCGGAA	exonic	HLA-DQB1	nonsynonymous SNV	HLA-DQB1:ENST00000399084.1:exon3:c.T365C:p.I122T,HLA-DQB1:ENST00000434651.2:exon2:c.T365C:p.I122T,HLA<br>DQB1:ENST00000484729.2:exon2:c.T365C:p.I122T,HLA-DQB1:ENST00000374943.4:exon2:c.T365C:p.I122T,HLA-DQB1:ENST00000399079.3:exon2:c.T365C:p.I122T,	6p21.32	.
6	32632592	C	G	CTGCAAGATC,CGCGGAACGC	exonic	HLA-DQB1	nonsynonymous SNV	HLA-DQB1:ENST00000399084.1:exon3:c.G362C:p.G121A,HLA-DQB1:ENST00000434651.2:exon2:c.G362C:p.G121A,HLA<br>DQB1:ENST00000484729.2:exon2:c.G362C:p.G121A,HLA-DQB1:ENST00000374943.4:exon2:c.G362C:p.G121A,HLA-DQB1:ENST00000399079.3:exon2:c.G362C:p.G121A,	6p21.32	.
6	32632593	C	T	TGCAAGATCC,GCGGAACGCC	exonic	HLA-DQB1	nonsynonymous SNV	HLA-DQB1:ENST00000399084.1:exon3:c.G361A:p.G121R,HLA-DQB1:ENST00000434651.2:exon2:c.G361A:p.G121R,HLA<br>DQB1:ENST00000484729.2:exon2:c.G361A:p.G121R,HLA-DQB1:ENST00000374943.4:exon2:c.G361A:p.G121R,HLA-DQB1:ENST00000399079.3:exon2:c.G361A:p.G121R,	6p21.32	.
6	32632599	A	G	ATCCCGCGGA,CGCCACCTCG	exonic	HLA-DQB1	nonsynonymous SNV	HLA-DQB1:ENST00000399084.1:exon3:c.T355C:p.F119L,HLA-DQB1:ENST00000434651.2:exon2:c.T355C:p.F119L,HLA<br>DQB1:ENST00000484729.2:exon2:c.T355C:p.F119L,HLA-DQB1:ENST00000374943.4:exon2:c.T355C:p.F119L,HLA-DQB1:ENST00000399079.3:exon2:c.T355C:p.F119L,	6p21.32	.
6	32632601	G	T	CCCGCGGAAC,CCACCTCGTA	exonic	HLA-DQB1	nonsynonymous SNV	HLA-DQB1:ENST00000399084.1:exon3:c.C353A:p.A118E,HLA-DQB1:ENST00000434651.2:exon2:c.C353A:p.A118E,HLA<br>DQB1:ENST00000484729.2:exon2:c.C353A:p.A118E,HLA-DQB1:ENST00000374943.4:exon2:c.C353A:p.A118E,HLA-DQB1:ENST00000399079.3:exon2:c.C353A:p.A118E,	6p21.32	.
6	32632605	C	A	CGGAACGCCA,CTCGTAGTTG	exonic	HLA-DQB1	nonsynonymous SNV	HLA-DQB1:ENST00000399084.1:exon3:c.G349T:p.V117L,HLA-DQB1:ENST00000434651.2:exon2:c.G349T:p.V117L,HLA<br>DQB1:ENST00000484729.2:exon2:c.G349T:p.V117L,HLA-DQB1:ENST00000374943.4:exon2:c.G349T:p.V117L,HLA-DQB1:ENST00000399079.3:exon2:c.G349T:p.V117L,	6p21.32	.
6	33632864	C	A	GACAAGGAGG,CTTTGCCATC	exonic	ITPR3	nonsynonymous SNV	ITPR3:ENST00000605930.1:exon13:c.C1283A:p.A428D,ITPR3:ENST00000374316.5:exon14:c.C1283A:p.A428D	6p21.31	.
6	35212427	T	C	TTGCCTACAG,CCAGTGCTCC	exonic	SCUBE3	nonsynonymous SNV	SCUBE3:ENST00000274938.7:exon18:c.T2240C:p.V747A,SCUBE3:ENST00000394681.1:exon19:c.T2288C:p.V763A	6p21.31	.
6	36182011	G	C	AGCCCAGACA,GGTCCTGGAG	exonic	BRPF3	nonsynonymous SNV	BRPF3:ENST00000357641.6:exon8:c.G2837C:p.R946T,BRPF3:ENST00000534400.1:exon7:c.G2837C:p.R946T	6p21.31	.
6	36262066	C	T	CATCTGTCCC,GGGACTGCCC	exonic	PNPLA1	nonsynonymous SNV	PNPLA1:ENST00000457797.1:exon4:c.C607T:p.R203W,PNPLA1:ENST00000394571.2:exon4:c.C604T:p.R202W,PNPLA1:ENST00000312917.5:exon4:c.C346T:p.R116W,PN<br>LA1:ENST00000388715.3:exon4:c.C319T:p.R107W,	6p21.31	.
6	36953907	C	G	CCCGCGGCAC,GCCGCGAGCC	exonic	MTCH1	nonsynonymous SNV	MTCH1:ENST00000373627.5:exon1:c.G43C:p.G15R,MTCH1:ENST00000373616.5:exon1:c.G43C:p.G15R	6p21.2	.
6	43018070	C	A	ATGTCTTCCT,AAAGCCCAAG	exonic	CUL7	stopgain	CUL7:ENST00000265348.3:exon5:c.G1300T:p.E434X,CUL7:ENST00000535468.1:exon5:c.G1552T:p.E518X	6p21.1	.
6	43487162	G	A	GCTTTTCGAC,AATTCTGCTA	exonic	POLR1C	nonsynonymous SNV	POLR1C:ENST00000304004.3:exon3:c.G233A:p.R78Q,POLR1C:ENST00000372344.2:exon3:c.G233A:p.R78Q,POLR1C:ENST00000372389.3:exon3:c.G233A:p.R78Q,POLR<br>C:ENST00000428025.2:exon3:c.G68A:p.R23Q,	6p21.1	POLR1C;bas
6	44197153	T	G	CTTATCTTCT,CATGCTGGGT	exonic	SLC29A1	nonsynonymous SNV	SLC29A1:ENST00000371755.3:exon3:c.T56G:p.F19C,SLC29A1:ENST00000371708.1:exon2:c.T56G:p.F19C,SLC29A1:ENST00000393841.1:exon4:c.T56G:p.F19C,SLC29A1<br>NST00000427851.2:exon3:c.T56G:p.F19C,SLC29A1:ENST00000393844.1:exon3:c.T56G:p.F19C,SLC29A1:ENST00000313248.7:exon4:c.T293G:p.F98C,SLC29A1:ENST00000<br>371731.1:exon4:c.T56G:p.F19C,SLC29A1:ENST00000371740.5:exon4:c.T56G:p.F19C,SLC29A1:ENST00000371724.1:exon4:c.T56G:p.F19C,SLC29A1:ENST00000371713.1:c	6p21.1	.
6	44223138	C	T	TTAAGAGCTT,GTATTGGCAC	exonic	SLC35B2	nonsynonymous SNV	SLC35B2:ENST00000538577.1:exon3:c.G325A:p.E109K,SLC35B2:ENST00000537814.1:exon2:c.G205A:p.E69K,SLC35B2:ENST00000393812.3:exon4:c.G604A:p.E202	6p21.1	.
6	56473409	G	A	TTCTTCAACA,TCATTCTTTG	exonic	DST	nonsynonymous SNV	DST:ENST00000439203.1:exon26:c.C4406T:p.T1469I,DST:ENST00000361203.3:exon36:c.C5384T:p.T1795I,DST:ENST00000370754.5:exon39:c.C5918T:p.T1973I,DST:ENS<br>00000370769.4:exon36:c.C5384T:p.T1795I,DST:ENST00000446842.2:exon26:c.C4406T:p.T1469I,DST:ENST00000312431.6:exon36:c.C5384T:p.T1795I,	6p12.1	.
6	71235998	G	A	GGAAACTTCT,AAAAAGAAAT	exonic	FAM135A	nonsynonymous SNV	FAM135A:ENST00000370479.3:exon15:c.G2572A:p.E858K,FAM135A:ENST00000505868.1:exon12:c.G3211A:p.E1071K,FAM135A:ENST00000361499.3:exon15:c.G2623A:p<br>875K,FAM135A:ENST00000418814.2:exon15:c.G3211A:p.E1071K,FAM135A:ENST00000505769.1:exon15:c.G1951A:p.E651K,FAM135A:ENST00000457062.2:exon14:c.G257	6q13	.
6	82459651	C	A	ACTGTGCTCT,ATTTACCACT	exonic	FAM46A	stopgain	FAM46A:ENST00000369756.3:exon3:c.G1333T:p.E445X,FAM46A:ENST00000369754.3:exon3:c.G1147T:p.E383X,FAM46A:ENST00000320172.6:exon3:c.G1090T:p.E364	6q14.1	.
6	106552952	C	A	GCCCCTCTGC,AGAAGACTTT	exonic	PRDM1	nonsynonymous SNV	PRDM1:ENST00000369089.3:exon3:c.C515A:p.P172Q,PRDM1:ENST00000369091.2:exon5:c.C809A:p.P270Q,PRDM1:ENST00000369096.4:exon5:c.C917A:p.P306	6q21	.
6	116600375	C	T	AATCTATCCT,CTCCGCCACT	exonic	TSPYL1	nonsynonymous SNV	TSPYL1:ENST00000368608.3:exon1:c.G619A:p.E207K	6q22.1	.
6	126080712	C	T	TCTCTTGTCC,TCTCTGCCAC	exonic	HEY2	nonsynonymous SNV	HEY2:ENST00000368365.1:exon5:c.C640T:p.L214F,HEY2:ENST00000368364.3:exon5:c.C778T:p.L260F	6q22.31	.
6	129704300	G	A	CGAGCAGACC,GACAGGATGC	exonic	LAMA2	nonsynonymous SNV	LAMA2:ENST00000421865.2:exon35:c.G4993A:p.G1665R	6q22.33	.
6	133013368	A	C	TTACCTGTAG,TAATAGCGCC	exonic	VNN1	stopgain	VNN1:ENST00000367928.4:exon5:c.T1182G:p.Y394X	6q23.2	.
6	139609922	G	A	ACTGGGGTTG,AGAATCCTGG	exonic	TXLNB	nonsynonymous SNV	TXLNB:ENST00000358430.3:exon2:c.C115T:p.P39S	6q24.1	.
7	1132436	G	A	TGCCCTGAAG,CCGTCATTCC	exonic	GPER1	nonsynonymous SNV	GPER1:ENST00000297469.3:exon2:c.G1072A:p.A358T,GPER1:ENST00000397092.1:exon3:c.G1072A:p.A358T,GPER1:ENST00000401670.1:exon2:c.G1072A:p.A358T,GPE<br>1:ENST00000397088.3:exon2:c.G1072A:p.A358T,	7p22.3	.
7	1785619	A	G	TGGCAGCCTC,AGAAGACCAT	exonic	ELFN1	nonsynonymous SNV	ELFN1:ENST00000561626.1:exon2:c.A1387G:p.K463E,ELFN1:ENST00000424383.2:exon3:c.A1387G:p.K463E,ELFN1:ENST00000541472.1:exon1:c.A1387G:p.K463	7p22.3	.
7	6066520	C	T	GTTAGAACTT,TGCTCGCTCC	exonic	EIF2AK1	nonsynonymous SNV	EIF2AK1:ENST00000199389.6:exon14:c.G1603A:p.E535K,EIF2AK1:ENST00000536084.1:exon13:c.G1231A:p.E411K	7p22.1	.
7	32914648	C	T	ACAGTGCTGT,ATTCATTGCC	exonic	KBTBD2	nonsynonymous SNV	KBTBD2:ENST00000304056.4:exon3:c.G292A:p.D98N	7p14.3	.
7	38781635	G	A	CTCAAATTGG,GATCTCCATT	exonic	VPS41	nonsynonymous SNV	VPS41:ENST00000395969.2:exon24:c.C2134T:p.P712S,VPS41:ENST00000439468.1:exon3:c.C232T:p.P78S,VPS41:ENST00000310301.4:exon25:c.C2209T:p.P737	7p14.1	VPS41;basec
7	48141503	G	C	GACTGCCCAG,TAGAGACTAT	exonic	UPP1	nonsynonymous SNV	UPP1:ENST00000331803.4:exon6:c.G245C:p.G82A,UPP1:ENST00000416681.1:exon5:c.G245C:p.G82A,UPP1:ENST00000395564.4:exon5:c.G245C:p.G82A,UPP1:ENST00<br>0421046.1:exon5:c.G245C:p.G82A,UPP1:ENST00000341253.4:exon6:c.G245C:p.G82A,UPP1:ENST00000436673.1:exon6:c.G245C:p.G82A,	7p12.3	.
7	65419129	C	T	CGTGGGGTCC,TGTACCTGGC	exonic	VKORC1L1	nonsynonymous SNV	VKORC1L1:ENST00000434382.2:exon2:c.C263T:p.P88L	7q11.21	.
7	75511227	G	A	CTGGCGCTTT,CTGGCAATTT	exonic	RHBDD2	nonsynonymous SNV	RHBDD2:ENST00000006777.6:exon2:c.G259A:p.A87T	7q11.23	.
7	75513096	A	T	CTTCAGCCTG,TGAGGAGGAT	exonic	RHBDD2	nonsynonymous SNV	RHBDD2:ENST00000428119.1:exon2:c.A244T:p.M82L,RHBDD2:ENST00000318622.4:exon4:c.A244T:p.M82L,RHBDD2:ENST00000006777.6:exon3:c.A667T:p.M223	7q11.23	.
7	94293747	C	G	GAAAGCTGAA,CTGTGCCTCT	exonic	PEG10	nonsynonymous SNV	PEG10:ENST00000482108.1:exon2:c.C879G:p.N293K,PEG10:ENST00000488574.1:exon2:c.C879G:p.N293K	7q21.3	.
7	94913595	G	A	TTCAATCTTT,GAAGGCATTC	exonic	PPP1R9A	nonsynonymous SNV	PPP1R9A:ENST00000433360.1:exon17:c.G3562A:p.G1188R	7q21.3	.
7	98495423	G	C	CCCCAGAAAT,GTTGGTATGA	exonic	TRRAP	nonsynonymous SNV	TRRAP:ENST00000355540.3:exon8:c.G567C:p.M189I,TRRAP:ENST00000359863.4:exon8:c.G567C:p.M189I,TRRAP:ENST00000446306.3:exon7:c.G567C:p.M189	7q22.1	.
7	99129847	T	A	ATAAATACCT,AAGTGTAGAG	exonic	ZKSCAN5	stopgain	ZKSCAN5:ENST00000451158.1:exon7:c.T2495A:p.L832X,ZKSCAN5:ENST00000394170.2:exon7:c.T2495A:p.L832X,ZKSCAN5:ENST00000326775.5:exon7:c.T2495A:p.L832	7q22.1	.
7	102105205	C	G	CGCTGCCGCT,GCAGATCAGA	exonic	ALKBH4	nonsynonymous SNV	ALKBH4:ENST00000490528.1:exon1:c.G79C:p.E27Q,ALKBH4:ENST00000292566.3:exon1:c.G79C:p.E27Q	7q22.1	.
7	104747026	C	T	TCGGTTTACT,AGAAACCTCC	exonic	KMT2E	nonsynonymous SNV	KMT2E:ENST00000311117.3:exon20:c.C2654T:p.S885L,KMT2E:ENST00000334877.4:exon20:c.C2654T:p.S885L,KMT2E:ENST00000257745.4:exon19:c.C2654T:p.S885	7q22.3	.
7	105255247	C	G	GGGCTATCTG,CGCAGGAGGG	exonic	ATXN7L1	nonsynonymous SNV	ATXN7L1:ENST00000484475.1:exon4:c.G637C:p.A213P,ATXN7L1:ENST00000388807.4:exon4:c.G514C:p.A172P,ATXN7L1:ENST00000419735.3:exon10:c.G1534C:p.A512<br>ATXN7L1:ENST00000472195.1:exon8:c.G1162C:p.A388P,ATXN7L1:ENST00000477775.1:exon8:c.G1162C:p.A388P,	7q22.3	.
7	116778558	C	T	CTCGGGAGGA,CAGGGAAGCA	exonic	ST7	nonsynonymous SNV	ST7:ENST00000422922.1:exon8:c.C728T:p.T243I,ST7:ENST00000323984.3:exon9:c.C935T:p.T312I,ST7:ENST00000393443.1:exon9:c.C716T:p.T239I,ST7:ENST00000432<br>8.1:exon9:c.C797T:p.T266I,ST7:ENST00000393451.3:exon8:c.C866T:p.T289I,ST7:ENST00000393447.4:exon9:c.C806T:p.T269I,ST7:ENST00000393449.1:exon9:c.C935T:p.T3<br>12I,ST7:ENST00000446490.1:exon8:c.C866T:p.T289I,ST7:ENST00000490039.1:exon8:c.C779T:p.T260I,ST7:ENST00000265437.5:exon9:c.C935T:p.T312I,ST7:ENST0000039	7q31.2	.
7	128487840	C	T	TGTCAGGGCC,GGGTTCCTCA	exonic	FLNC	nonsynonymous SNV	FLNC:ENST00000346177.6:exon25:c.C4378T:p.R1460W,FLNC:ENST00000325888.8:exon25:c.C4378T:p.R1460W	7q32.1	.
7	132990692	G	A	AGTGACCTTC,ACTAGAGCTT	exonic	EXOC4	nonsynonymous SNV	EXOC4:ENST00000393161.2:exon4:c.G533A:p.R178Q,EXOC4:ENST00000253861.4:exon4:c.G533A:p.R178Q,EXOC4:ENST00000539845.1:exon4:c.G230A:p.R77	7q33	.
7	133863303	C	A	GGTTGCAGCT,AAGACCACCT	exonic	LRGUK	nonsynonymous SNV	LRGUK:ENST00000285928.2:exon10:c.C1156A:p.Q386K	7q33	.
7	135272651	G	A	GCTTGCTCTA,AATATTGGTG	exonic	NUP205	nonsynonymous SNV	NUP205:ENST00000285968.6:exon10:c.G1384A:p.E462K	7q33	.
7	138447099	C	T	ACTTTCCGGT,ATATATGCAG	exonic	ATP6V0A4	nonsynonymous SNV	ATP6V0A4:ENST00000310018.2:exon7:c.G498A:p.M166I,ATP6V0A4:ENST00000393054.1:exon6:c.G498A:p.M166I,ATP6V0A4:ENST00000353492.4:exon6:c.G498A:p.M16	7q34	.
7	138603078	C	T	GTGGCCAGAA,TTGCTGAGGT	exonic	KIAA1549	nonsynonymous SNV	KIAA1549:ENST00000440172.1:exon2:c.G1294A:p.V432I,KIAA1549:ENST00000422774.1:exon2:c.G1294A:p.V432I,KIAA1549:ENST00000242365.4:exon1:c.G1144A:p.V38	7q34	.
7	142637630	C	T	GCCATTGAGG,GGACATTCCC	exonic	C7orf34	nonsynonymous SNV	C7orf34:ENST00000409607.3:exon2:c.C400T:p.R134W	7q34	.
7	151127216	G	C	TGATTGAAGA,AGCTGCTCAG	exonic	CRYGN	nonsynonymous SNV	CRYGN:ENST00000337323.2:exon4:c.C467G:p.S156C	7q36.1	.
8	1497142	G	C	CACGCGCGAC,ACTGCGCTGT	exonic	DLGAP2	nonsynonymous SNV	DLGAP2:ENST00000421627.2:exon2:c.G283C:p.D95H	8p23.3	.
8	1581083	C	T	TGCCCTCGAC,TCCCGGGATG	exonic	DLGAP2	nonsynonymous SNV	DLGAP2:ENST00000421627.2:exon5:c.C1441T:p.L481F	8p23.3	.
8	12879009	C	A	TTGAAAAACA,AGAAGTTTGG	exonic	KIAA1456	nonsynonymous SNV	KIAA1456:ENST00000524591.2:exon5:c.C821A:p.T274K	8p22	.
8	13357322	C	T	CTGTCATTTT,GTCCACATCC	exonic	DLC1	nonsynonymous SNV	DLC1:ENST00000511869.1:exon2:c.G259A:p.E87K,DLC1:ENST00000276297.4:exon2:c.G259A:p.E87K,DLC1:ENST00000316609.5:exon2:c.G259A:p.E87	8p22	.
8	22460060	C	T	CGGGATATCT,CCACTGGGAC	exonic	C8orf58	nonsynonymous SNV	C8orf58:ENST00000289989.5:exon6:c.C890T:p.S297F,C8orf58:ENST00000475994.1:exon3:c.C406T:p.P136S	8p21.3	C8orf58;base
8	42611273	A	T	TCCAGAGGCC,CCTCATCAGC	exonic	CHRNA6	nonsynonymous SNV	CHRNA6:ENST00000276410.2:exon5:c.T1069A:p.W357R,CHRNA6:ENST00000534622.1:exon4:c.T1024A:p.W342R	8p11.21	.
8	43033291	G	A	TCAAAATTCA,ATTGCTGGGG	exonic	HGSNAT	nonsynonymous SNV	HGSNAT:ENST00000379644.4:exon10:c.G926A:p.R309K,HGSNAT:ENST00000458501.2:exon10:c.G1010A:p.R337K,HGSNAT:ENST00000522082.1:exon3:c.G167A:p.R56I	8p11.21	.
8	48626134	C	A	TTCCTGTGAG,TGCCTGGCCC	exonic	SPIDR	nonsynonymous SNV	SPIDR:ENST00000518074.1:exon16:c.C2092A:p.L698M,SPIDR:ENST00000517693.1:exon9:c.C697A:p.L233M,SPIDR:ENST00000297423.4:exon16:c.C2272A:p.L758M,SPID<br>ENST00000541342.1:exon15:c.C2062A:p.L688M,	8q11.21	.
8	48626137	C	G	CTGTGAGCTG,CTGGCCCGGT	exonic	SPIDR	nonsynonymous SNV	SPIDR:ENST00000518074.1:exon16:c.C2095G:p.P699A,SPIDR:ENST00000517693.1:exon9:c.C700G:p.P234A,SPIDR:ENST00000297423.4:exon16:c.C2275G:p.P759A,SPID<br>:ENST00000541342.1:exon15:c.C2065G:p.P689A,	8q11.21	.
8	55539300	C	T	AATAGTTTTT,AGGGAATGAT	exonic	RP1	nonsynonymous SNV	RP1:ENST00000220676.1:exon4:c.C2858T:p.S953L	8q12.1	.
8	82592967	G	A	AAATCAACTG,AGAACTTTTC	exonic	IMPA1	nonsynonymous SNV	IMPA1:ENST00000256108.5:exon3:c.C115T:p.P39S,IMPA1:ENST00000518202.1:exon3:c.C115T:p.P39S,IMPA1:ENST00000519816.1:exon3:c.C115T:p.P39S,IMPA1:ENST0<br>00518188.1:exon3:c.C115T:p.P39S,IMPA1:ENST00000521360.1:exon3:c.C115T:p.P39S,IMPA1:ENST00000449740.2:exon4:c.C292T:p.P98S,IMPA1:ENST00000311489.4:exon<br>3:c.C115T:p.P39S,IMPA1:ENST00000521979.1:exon3:c.C115T:p.P39S,IMPA1:ENST00000519964.1:exon3:c.C91T:p.P31S,IMPA1:ENST00000522997.1:exon3:c.C292T:p.P98S	8q21.13	.
8	100974969	C	T	GAAGAGCTAG,CTTAAAATTT	exonic	RGS22	nonsynonymous SNV	RGS22:ENST00000523437.1:exon26:c.G3700A:p.A1234T,RGS22:ENST00000360863.6:exon26:c.G3736A:p.A1246T,RGS22:ENST00000523287.1:exon24:c.G3193A:p.A1065	8q22.2	.
8	144378400	G	C	CGTGCGCCGA,TGCGGCAAGG	exonic	ZNF696	nonsynonymous SNV	ZNF696:ENST00000518575.1:exon4:c.G555C:p.E185D,ZNF696:ENST00000330143.3:exon3:c.G555C:p.E185D	8q24.3	.
8	144406773	G	A	CTGGTGCCCC,CCGGCGGCTC	exonic	TOP1MT	nonsynonymous SNV	TOP1MT:ENST00000519591.1:exon7:c.C404T:p.A135V,TOP1MT:ENST00000329245.4:exon6:c.C698T:p.A233V,TOP1MT:ENST00000523676.1:exon7:c.C404T:p.A135V,TOP<br>MT:ENST00000519148.1:exon6:c.C404T:p.A135V,TOP1MT:ENST00000521193.1:exon7:c.C404T:p.A135V,TOP1MT:ENST00000519139.2:exon6:c.C404T:p.A135V,TOP1MT:E	8q24.3	TOP1MT;bas
8	144899579	G	A	GTCAGTGCTG,GGACACCAGT	exonic	PUF60	nonsynonymous SNV	PUF60:ENST00000453551.2:exon10:c.C937T:p.P313S,PUF60:ENST00000526683.1:exon10:c.C1066T:p.P356S,PUF60:ENST00000456095.2:exon9:c.C979T:p.P327S,PUF60<br>NST00000313352.7:exon9:c.C886T:p.P296S,PUF60:ENST00000527197.1:exon8:c.C928T:p.P310S,PUF60:ENST00000349157.6:exon9:c.C1015T:p.P339S,	8q24.3	.
8	144945944	G	A	GGCTGTGGCC,TGCTCAGGGC	exonic	EPPK1	nonsynonymous SNV	EPPK1:ENST00000525985.1:exon2:c.C1478T:p.T493M	8q24.3	.
8	145512873	C	G	ATCATCCTCA,TGCTGTCACT	exonic	BOP1	nonsynonymous SNV	BOP1:ENST00000307404.5:exon2:c.G212C:p.S71T	8q24.3	.
8	146032974	C	A	GCGGCACCAG,TGATCCACAC	exonic	ZNF517	nonsynonymous SNV	ZNF517:ENST00000359971.3:exon5:c.C673A:p.L225M,ZNF517:ENST00000531720.1:exon4:c.C673A:p.L225M,ZNF517:ENST00000533965.1:exon5:c.C673A:p.L225	8q24.3	.
9	21971208	C	T	TCATCATGAC,TGCCAGAGAG	splicing	CDKN2A(ENST00000361570.3:exon3:c.317-1G>A,ENST00000304494.5:exon3:c.151<br>1G>A,ENST00000579755.1:exon3:c.194-1G>A,ENST00000579122.1:exon3:c.151-	.	.	9p21.3	.
9	33037072	C	T	GCCAATTTAT,GTAGACCATA	exonic	DNAJA1	nonsynonymous SNV	DNAJA1:ENST00000544625.1:exon7:c.C463T:p.R155C,DNAJA1:ENST00000330899.4:exon8:c.C934T:p.R312C	9p21.1	.
9	34991409	A	G	TAGAGCAGCT,GCAGGTGGGT	exonic	DNAJB5	nonsynonymous SNV	DNAJB5:ENST00000539059.1:exon1:c.A70G:p.S24G	9p13.3	.
9	35753101	C	T	TTGAGTTTAG,TGGAGTGGGG	exonic	MSMP	nonsynonymous SNV	MSMP:ENST00000436428.2:exon3:c.G416A:p.S139N	9p13.3	.
9	35801926	G	A	GCCCCAGCGG,GATCCAGTTA	exonic	NPR2	nonsynonymous SNV	NPR2:ENST00000342694.2:exon9:c.G1561A:p.G521R	9p13.3	.
9	79320007	G	A	AAATCAAAGG,AGGTGTGTAC	exonic	PRUNE2	nonsynonymous SNV	PRUNE2:ENST00000428286.1:exon8:c.C6106T:p.P2036S,PRUNE2:ENST00000376718.3:exon8:c.C7183T:p.P2395S	9q21.2	.
9	82227583	A	G	AGAGATTGTC,AGAGGCTGAA	exonic	TLE4	nonsynonymous SNV	TLE4:ENST00000376552.2:exon5:c.A265G:p.K89E,TLE4:ENST00000376544.3:exon5:c.A265G:p.K89E,TLE4:ENST00000425506.1:exon5:c.A259G:p.K87E,TLE4:ENST0000<br>76520.4:exon5:c.A265G:p.K89E,TLE4:ENST00000435650.1:exon5:c.A265G:p.K89E,TLE4:ENST00000265284.6:exon5:c.A265G:p.K89E,TLE4:ENST00000428713.1:exon4:c.A2<br>20G:p.K74E,TLE4:ENST00000414465.1:exon4:c.A220G:p.K74E,TLE4:ENST00000462803.1:exon5:c.A265G:p.K89E,TLE4:ENST00000376537.4:exon5:c.A265G:p.K89E,	9q21.31	.
9	92220888	C	T	CCGCAGAACC,CAACGAGGAC	exonic	GADD45G	nonsynonymous SNV	GADD45G:ENST00000252506.6:exon4:c.C374T:p.P125L,GADD45G:ENST00000375769.1:exon3:c.C320T:p.P107L	9q22.2	.
9	94984900	T	C	GGTGAGTCCA,TCTGCCCAAG	exonic	IARS	nonsynonymous SNV	IARS:ENST00000447699.2:exon32:c.A3278G:p.N1093S,IARS:ENST00000443024.2:exon33:c.A3608G:p.N1203S,IARS:ENST00000375643.3:exon33:c.A3608G:p.N1203	9q22.31	.
9	104314719	C	T	TACTGAGGAC,CGAAGGATGA	exonic	RNF20	nonsynonymous SNV	RNF20:ENST00000389120.3:exon13:c.C1585T:p.P529S	9q31.1	.
9	104314720	C	T	ACTGAGGACC,GAAGGATGAG	exonic	RNF20	nonsynonymous SNV	RNF20:ENST00000389120.3:exon13:c.C1586T:p.P529L	9q31.1	.
9	113173608	C	T	CTGCCCACCT,TCATACATTC	exonic	SVEP1	nonsynonymous SNV	SVEP1:ENST00000374469.1:exon37:c.G6314A:p.R2105K,SVEP1:ENST00000297826.5:exon2:c.G161A:p.R54K,SVEP1:ENST00000401783.2:exon37:c.G6383A:p.R2128	9q31.3	.
9	113308444	G	T	TTTCACAGAT,CACTCAAAAT	exonic	SVEP1	stopgain	SVEP1:ENST00000302728.8:exon3:c.C915A:p.C305X,SVEP1:ENST00000374469.1:exon3:c.C846A:p.C282X,SVEP1:ENST00000374461.1:exon3:c.C846A:p.C282X,SVEP1:E<br>ST00000401783.2:exon3:c.C915A:p.C305X,	9q31.3	.
9	116931342	C	G	GAGTACTCGG,CACCAGCCAC	exonic	COL27A1	nonsynonymous SNV	COL27A1:ENST00000356083.3:exon3:c.C1507G:p.P503A,COL27A1:ENST00000451716.1:exon1:c.C1348G:p.P450A	9q32	.
9	125920688	G	T	CGCTCACAAG,ATCATGAAGA	exonic	STRBP	nonsynonymous SNV	STRBP:ENST00000447404.2:exon9:c.C856A:p.P286T,STRBP:ENST00000360998.3:exon10:c.C814A:p.P272T,STRBP:ENST00000348403.5:exon10:c.C856A:p.P286T	9q33.3	.
9	127969880	C	T	CCCCTGTGCT,GAGTTGGCCA	exonic	RABEPK	stopgain	RABEPK:ENST00000394124.4:exon4:c.C91T:p.R31X,RABEPK:ENST00000373538.3:exon3:c.C91T:p.R31X,RABEPK:ENST00000259460.8:exon4:c.C91T:p.R31X,RABEPK:E<br>ST00000373544.1:exon4:c.C91T:p.R31X,RABEPK:ENST00000416065.1:exon2:c.C340T:p.R114X,RABEPK:ENST00000394125.4:exon4:c.C91T:p.R31X,	9q33.3	.
9	130258294	G	A	GCTGTCGGCT,AGCACTACCT	exonic	LRSAM1	nonsynonymous SNV	LRSAM1:ENST00000300417.6:exon23:c.G1750A:p.E584K,LRSAM1:ENST00000373324.4:exon22:c.G1669A:p.E557K,LRSAM1:ENST00000373322.1:exon22:c.G1750A:p.E5<br>K,LRSAM1:ENST00000323301.4:exon22:c.G1750A:p.E584K,	9q33.3	.
9	130479981	G	A	CAGAACCTGC,AAGGGCCTAC	exonic	TTC16	nonsynonymous SNV	TTC16:ENST00000373289.3:exon4:c.G356A:p.R119Q	9q34.11	.
9	130670691	G	A	CTCCAGGACG,ATGGGCGAAC	exonic	ST6GALNAC4	nonsynonymous SNV	ST6GALNAC4:ENST00000343609.2:exon5:c.C637T:p.P213S,ST6GALNAC4:ENST00000335791.5:exon6:c.C889T:p.P297S	9q34.11	ST6GALNAC
9	132618640	G	C	CTGTCTGCAG,TAAAAGACCC	splicing	USP20(ENST00000372429.3:exon4:c.135+1G>C,ENST00000315480.4:exon4:c.135<br>1G>C,ENST00000358355.1:exon4:c.135+1G>C)	.	.	9q34.11	.
9	135205376	G	A	CTAATCAGCT,CACATAAGCA	exonic	SETX	stopgain	SETX:ENST00000393220.1:exon10:c.C1609T:p.Q537X,SETX:ENST00000224140.5:exon10:c.C1609T:p.Q537X,SETX:ENST00000372169.2:exon10:c.C1609T:p.Q537	9q34.13	.
9	139811069	T	G	CTCGAGACGG,GAGTCGGGGG	splicing	TRAF2(ENST00000536468.1:exon8:c.678+2T>G,ENST00000247668.2:exon7:c.678<br>2T>G,ENST00000359662.3:exon7:c.834+2T>G)	.	.	9q34.3	.
10	4877958	A	G	GACGAGAGCA,CATGGTTATT	exonic	AKR1E2	nonsynonymous SNV	AKR1E2:ENST00000533295.1:exon5:c.A428G:p.N143S,AKR1E2:ENST00000298375.7:exon4:c.A416G:p.N139S,AKR1E2:ENST00000532248.1:exon4:c.A416G:p.N139S,AKR<br>E2:ENST00000463345.1:exon4:c.A416G:p.N139S,AKR1E2:ENST00000345253.5:exon4:c.A416G:p.N139S,AKR1E2:ENST00000474119.1:exon4:c.A104G:p.N35S,AKR1E2:EN	10p15.1	.
10	5415832	A	G	CAGTGGGAGG,TGCATCCCTG	exonic	UCN3	nonsynonymous SNV	UCN3:ENST00000380433.3:exon2:c.A149G:p.D50G	10p15.1	.
10	5496276	G	T	CCAGCTGGCA,CCAAAGCTCT	exonic	NET1	nonsynonymous SNV	NET1:ENST00000449083.1:exon4:c.G271T:p.A91S,NET1:ENST00000542715.1:exon9:c.G274T:p.A92S,NET1:ENST00000355029.4:exon9:c.G817T:p.A273S,NET1:ENST000<br>0380359.3:exon7:c.G655T:p.A219S,	10p15.1	.
10	8007221	A	C	CTTAAAGAGG,AGAGGCAGAT	exonic	TAF3	nonsynonymous SNV	TAF3:ENST00000344293.5:exon3:c.A1748C:p.E583A	10p14	.
10	17152939	G	A	GCCTGGCAGT,GGAAGACCCA	exonic	CUBN	nonsynonymous SNV	CUBN:ENST00000377833.4:exon9:c.C994T:p.H332Y	10p13	.
10	17202340	G	A	GGTACTGAAA,CCACAATTTT	exonic	TRDMT1	nonsynonymous SNV	TRDMT1:ENST00000377766.5:exon3:c.C208T:p.L70F	10p13	.
10	19780551	A	G	GACTGCTCTG,TAGGTCTGAT	exonic	C10orf112	nonsynonymous SNV	C10orf112:ENST00000377266.3:exon17:c.A3272G:p.D1091G,C10orf112:ENST00000454679.2:exon16:c.A3233G:p.D1078G	10p12.31	.
10	22605395	C	T	GCTGGCGGAT,CCCGCTCCTT	exonic	COMMD3,COMMD3-BMI1	nonsynonymous SNV	COMMD3:ENST00000376836.3:exon1:c.C49T:p.P17S,COMMD3-BMI1:ENST00000602390.1:exon1:c.C49T:p.P17S,COMMD3	10p12.2	.
10	26463161	G	A	GAGGAAGGCA,AGGCCGTCTG	exonic	MYO3A	nonsynonymous SNV	MYO3A:ENST00000265944.5:exon30:c.G3968A:p.R1323K	10p12.1	.
10	35928811	T	A	GCCGTCCTGT,GCTTGATGAC	exonic	FZD8	nonsynonymous SNV	FZD8:ENST00000374694.1:exon1:c.A1547T:p.Q516L	10p11.21	.
10	45498988	A	G	CTATAAATGT,CTGAATGTGA	exonic	ZNF22	nonsynonymous SNV	ZNF22:ENST00000298299.3:exon2:c.A172G:p.T58A	10q11.21	.
10	54531253	C	T	CCCATCTTTG,CTGGGAAGCC	exonic	MBL2	nonsynonymous SNV	MBL2:ENST00000373968.3:exon1:c.G143A:p.G48D	10q21.1	.
10	71007133	C	T	GAAGGCCTTG,TAATACAAGA	exonic	HKDC1	nonsynonymous SNV	HKDC1:ENST00000354624.5:exon9:c.C1049T:p.A350V,HKDC1:ENST00000395086.2:exon9:c.C1049T:p.A350V	10q22.1	.
10	74890539	G	A	GAAGAGAAAG,GCCCCTATAC	exonic	NUDT13	nonsynonymous SNV	NUDT13:ENST00000349051.5:exon7:c.G670A:p.G224S,NUDT13:ENST00000537969.1:exon9:c.G346A:p.G116S,NUDT13:ENST00000544879.1:exon10:c.G559A:p.G187S,N<br>DT13:ENST00000357321.4:exon9:c.G937A:p.G313S,	10q22.1	.
10	75560450	C	A	ATCACCCCAA,AACTTCTCCC	exonic	ZSWIM8	nonsynonymous SNV	ZSWIM8:ENST00000605216.1:exon24:c.C5064A:p.N1688K,ZSWIM8:ENST00000604729.1:exon24:c.C5055A:p.N1685K,ZSWIM8:ENST00000398706.2:exon24:c.C5079A:p.N1<br>93K,ZSWIM8:ENST00000604524.1:exon24:c.C4518A:p.N1506K,ZSWIM8:ENST00000603114.1:exon25:c.C4941A:p.N1647K,	10q22.2	.
10	81930632	G	T	GGGCATGCTG,GCGGAGGAGG	exonic	ANXA11	nonsynonymous SNV	ANXA11:ENST00000438331.1:exon5:c.C95A:p.P32H,ANXA11:ENST00000360615.4:exon4:c.C95A:p.P32H,ANXA11:ENST00000422982.3:exon4:c.C95A:p.P32H,ANXA11:EN<br>T00000372231.3:exon3:c.C95A:p.P32H,ANXA11:ENST00000265447.4:exon3:c.C95A:p.P32H,ANXA11:ENST00000437799.1:exon2:c.C95A:p.P32H,ANXA11:ENST0000053599	10q22.3	.
10	82182208	A	T	GCTATGGGAA,AAAATGGAGC	exonic	FAM213A	stopgain	FAM213A:ENST00000372185.1:exon3:c.A181T:p.K61X,FAM213A:ENST00000372181.1:exon2:c.A214T:p.K72X,FAM213A:ENST00000372187.5:exon3:c.A214T:p.K72X,FAM<br>3A:ENST00000372188.1:exon3:c.A214T:p.K72X,	10q23.1	.
10	85991873	T	C	GGAGTCCTTG,TGAAGCACTT	exonic	LRIT1	nonsynonymous SNV	LRIT1:ENST00000372105.3:exon4:c.A1682G:p.N561S	10q23.1	.
10	85997430	G	T	CGGGGTCGTT,CACACTACTG	exonic	LRIT1	stopgain	LRIT1:ENST00000372105.3:exon2:c.C135A:p.C45X	10q23.1	.
10	88822534	C	G	AGAGATGCGT,CATGGATTCC	exonic	GLUD1	nonsynonymous SNV	GLUD1:ENST00000544149.1:exon6:c.G401C:p.G134A,GLUD1:ENST00000277865.4:exon6:c.G800C:p.G267A,GLUD1:ENST00000537649.1:exon9:c.G299C:p.G100	10q23.2	.
10	91497690	A	C	CTTTTAGGTA,TGATTATTTG	exonic	KIF20B	nonsynonymous SNV	KIF20B:ENST00000394289.2:exon20:c.A3092C:p.N1031T,KIF20B:ENST00000416354.1:exon20:c.A3182C:p.N1061T,KIF20B:ENST00000260753.4:exon20:c.A2972C:p.N991<br>KIF20B:ENST00000371728.3:exon20:c.A3092C:p.N1031T,	10q23.31	.
10	93586806	G	A	TGGACTTGTG,CAATTCACTC	exonic	TNKS2	stopgain	TNKS2:ENST00000371627.4:exon8:c.G828A:p.W276X	10q23.32	.
10	93617198	A	G	AAGGTTTGTA,CAAGAAACTA	exonic	TNKS2	nonsynonymous SNV	TNKS2:ENST00000371627.4:exon24:c.A3005G:p.N1002S	10q23.32	.
10	101841277	A	T	TGCACCTTGT,CAGCGTCCGC	exonic	CPN1	nonsynonymous SNV	CPN1:ENST00000370418.3:exon1:c.T106A:p.Y36N	10q24.2	.
10	103900988	C	T	TCTATGGGGC,AGTACTACCT	exonic	PPRC1	nonsynonymous SNV	PPRC1:ENST00000413464.2:exon5:c.C2723T:p.P908L,PPRC1:ENST00000278070.2:exon5:c.C2723T:p.P908L	10q24.32	.
10	105198566	G	A	TGTTCTTTCG,TGAGTGAGGG	splicing	PDCD11(ENST00000369797.3:exon27:c.4025+1G>A)	.	.	10q24.33	.
10	115350588	C	T	GGGTCATCGT,GACACTGCGG	exonic	NRAP	nonsynonymous SNV	NRAP:ENST00000359988.3:exon40:c.G4705A:p.D1569N,NRAP:ENST00000369360.3:exon39:c.G4624A:p.D1542N,NRAP:ENST00000369358.4:exon40:c.G4729A:p.D1577N<br>RAP:ENST00000360478.3:exon39:c.G4600A:p.D1534N,	10q25.3	.
10	125447510	C	T	AAGTGCTTGG,GTACAGCAAG	exonic	GPR26	nonsynonymous SNV	GPR26:ENST00000284674.1:exon3:c.C848T:p.A283V	10q26.13	.
10	127430787	A	T	TGGAATTGTC,GGCTTTAAAA	splicing	C10orf137(ENST00000419769.2:exon18:c.2372-2A>T)	.	.	10q26.13	.
10	129902458	C	T	CTTTTCCTTA,GAGTTCTCAG	exonic	MKI67	nonsynonymous SNV	MKI67:ENST00000368653.3:exon12:c.G6566A:p.R2189H,MKI67:ENST00000368654.3:exon13:c.G7646A:p.R2549H	10q26.2	.
10	134755571	T	A	GGTTGGCCGA,TTGATCAACT	exonic	TTC40	nonsynonymous SNV	TTC40:ENST00000368585.3:exon2:c.A87T:p.K29N,TTC40:ENST00000368586.5:exon2:c.A87T:p.K29N,TTC40:ENST00000368582.2:exon2:c.A87T:p.K29N	10q26.3	.
11	608968	G	A	TCGGAGCACA,GGCACGGGAG	exonic	PHRF1	nonsynonymous SNV	PHRF1:ENST00000534320.1:exon14:c.G3512A:p.R1171K,PHRF1:ENST00000413872.2:exon14:c.G3506A:p.R1169K,PHRF1:ENST00000416188.2:exon14:c.G3509A:p.R117<br>,PHRF1:ENST00000264555.5:exon14:c.G3512A:p.R1171K,PHRF1:ENST00000533464.1:exon14:c.G3500A:p.R1167K,	11p15.5	.
11	792435	T	G	GAGCGGGAAG,ACACCACAGA	exonic	SLC25A22	nonsynonymous SNV	SLC25A22:ENST00000531214.1:exon8:c.A611C:p.Y204S,SLC25A22:ENST00000320230.5:exon8:c.A611C:p.Y204S,SLC25A22:ENST00000481290.1:exon7:c.A686C:p.Y229	11p15.5	.
11	904752	G	T	ATCTGACTTT,ACAGGGTAGT	exonic	CHID1	stopgain	CHID1:ENST00000454838.2:exon1:c.C65A:p.S22X,CHID1:ENST00000525840.1:exon2:c.C65A:p.S22X,CHID1:ENST00000528154.1:exon2:c.C65A:p.S22X,CHID1:ENST0000<br>31859.1:exon2:c.C65A:p.S22X,CHID1:ENST00000429789.2:exon2:c.C65A:p.S22X,CHID1:ENST00000528581.1:exon2:c.C65A:p.S22X,CHID1:ENST00000436108.2:exon3:c.C6<br>5A:p.S22X,CHID1:ENST00000534254.1:exon2:c.C65A:p.S22X,CHID1:ENST00000533056.1:exon2:c.C65A:p.S22X,CHID1:ENST00000533059.1:exon3:c.C65A:p.S22X,CHID1:E<br>NST00000525225.1:exon4:c.C65A:p.S22X,CHID1:ENST00000323578.8:exon2:c.C65A:p.S22X,CHID1:ENST00000323541.7:exon2:c.C155A:p.S52X,CHID1:ENST00000528426.<br>1:exon3:c.C65A:p.S22X,CHID1:ENST00000530939.1:exon2:c.C65A:p.S22X,CHID1:ENST00000336845.5:exon3:c.C65A:p.S22X,CHID1:ENST00000449825.1:exon2:c.C65A:p.S	11p15.5	.
11	2398786	G	A	CGCCATGGGA,TGGAGGGCTG	exonic	CD81	nonsynonymous SNV	CD81:ENST00000263645.5:exon1:c.G7A:p.V3M	11p15.5	.
11	5290829	C	T	CTTGGGGTTG,CCAGGATGGC	exonic	HBE1	nonsynonymous SNV	HBE1:ENST00000292896.2:exon2:c.G170A:p.G57D,HBE1:ENST00000396895.1:exon3:c.G170A:p.G57D,HBE1:ENST00000380237.1:exon4:c.G170A:p.G57I	11p15.4	.
11	5536911	G	T	ATATGGCTGT,GGTTCAGTGG	exonic	UBQLNL	nonsynonymous SNV	UBQLNL:ENST00000380184.1:exon1:c.C761A:p.P254Q	11p15.4	.
11	5776677	G	A	GCAGATGCTC,GCAGAAGGCC	exonic	OR52N4	nonsynonymous SNV	OR52N4:ENST00000317254.3:exon1:c.G707A:p.R236Q	11p15.4	.
11	5842356	A	G	TTTTTCACTC,TCGTTTTGTA	exonic	OR52N2	nonsynonymous SNV	OR52N2:ENST00000317037.2:exon1:c.A791G:p.H264R	11p15.4	.
11	6643849	G	A	CCACCACGGG,GTAGGGTCCT	exonic	DCHS1	nonsynonymous SNV	DCHS1:ENST00000299441.3:exon21:c.C9058T:p.P3020S	11p15.4	.
11	12315966	A	G	TTCCCCTGGG,GAAACCAGTC	exonic	MICALCL	nonsynonymous SNV	MICALCL:ENST00000256186.2:exon3:c.A988G:p.R330G	11p15.3	.
11	12903456	G	A	CAAGCCTTTT,TGCAGCAGGC	exonic	TEAD1	nonsynonymous SNV	TEAD1:ENST00000527636.1:exon8:c.G526A:p.V176M,TEAD1:ENST00000527575.1:exon7:c.G526A:p.V176M,TEAD1:ENST00000361985.2:exon7:c.G526A:p.V176M,TEAD1<br>NST00000361905.4:exon8:c.G481A:p.V161M,TEAD1:ENST00000334310.6:exon8:c.G493A:p.V165M,TEAD1:ENST00000526600.1:exon3:c.G238A:p.V80M,	11p15.2	.
11	16208376	C	T	CGTTGTTTCT,AATTTGTGAC	exonic	SOX6	nonsynonymous SNV	SOX6:ENST00000396356.3:exon5:c.G661A:p.E221K,SOX6:ENST00000528252.1:exon5:c.G661A:p.E221K,SOX6:ENST00000316399.6:exon4:c.G661A:p.E221K,SOX6:ENST<br>000528429.1:exon5:c.G661A:p.E221K,SOX6:ENST00000527619.1:exon5:c.G670A:p.E224K,SOX6:ENST00000352083.6:exon5:c.G661A:p.E221K,	11p15.1	.
11	18210601	G	A	CAAGCAGATC,AGACTGGGCC	exonic	RP11-113D6.6	nonsynonymous SNV	RP11-113D6.6:ENST00000527671.1:exon1:c.C317T:p.S106L	11p15.1	.
11	46911975	G	A	GCAATGATGC,GCGTCCAGAG	exonic	LRP4	nonsynonymous SNV	LRP4:ENST00000378623.1:exon14:c.C1768T:p.R590C	11p11.2	.
11	46916239	G	T	CAGAAGACAA,CTCGCGGCGG	exonic	LRP4	nonsynonymous SNV	LRP4:ENST00000378623.1:exon12:c.C1441A:p.L481I	11p11.2	.
11	55861061	C	T	AGATCCATCT,CTTTGTTGGC	exonic	OR8I2	nonsynonymous SNV	OR8I2:ENST00000302124.2:exon1:c.C278T:p.S93F	11q12.1	OR8I2;basec

11	57094268 A	T	GCTCTCGCTT,GCTGCCTTGA	exonic	SSRP1	nonsynonymous SNV	SSRP1:ENST00000278412.2:exon16:c.T1967A:p.L656Q	11q12.1	.
11	60152655 C	T	GCAATTTCCA,CACTTTGATG	exonic	MS4A7	nonsynonymous SNV	MS4A7:ENST00000300184.3:exon3:c.C242T:p.T81I,MS4A7:ENST00000530234.2:exon3:c.C242T:p.T81	11q12.2	.
11	61089840 C	T	TAAAGGTTTC,ATGGCCACTA	exonic	DDB1	nonsynonymous SNV	DDB1:ENST00000301764.7:exon9:c.G1050A:p.M350I,DDB1:ENST00000540166.1:exon11:c.G1050A:p.M350I,DDB1:ENST00000539739.1:exon2:c.G207A:p.M69I,DDB1:ENS 0000535174.1:exon5:c.G399A:p.M133I,DDB1:ENST00000535967.1:exon1:c.G3A:p.M1I,DDB1:ENST00000541513.1:exon4:c.G495A:p.M165I,	11q12.2	.
11	62561869 A	T	ATCTCTTCAG,ACTGGCATTC	exonic	NXF1	nonsynonymous SNV	NXF1:ENST00000294172.2:exon19:c.T1621A:p.S541T,NXF1:ENST00000532297.1:exon20:c.T1621A:p.S541T,NXF1:ENST00000530875.1:exon20:c.T1750A:p.S584	11q12.3	.
11	64854441 G	C	CAGCGCCTCT,CTGCCCCAGC	exonic	ZFPL1	nonsynonymous SNV	ZFPL1:ENST00000294258.3:exon6:c.G523C:p.A175P,ZFPL1:ENST00000526334.1:exon6:c.G523C:p.A175P,ZFPL1:ENST00000532200.1:exon5:c.G523C:p.A175P,ZFPL1:EN T00000526945.1:exon6:c.G505C:p.A169P,	11q13.1	.
11	64854447 C	T	CTCTGCTGCC,CAGCCTTCTA	exonic	ZFPL1	nonsynonymous SNV	ZFPL1:ENST00000294258.3:exon6:c.C529T:p.P177S,ZFPL1:ENST00000526334.1:exon6:c.C529T:p.P177S,ZFPL1:ENST00000532200.1:exon5:c.C529T:p.P177S,ZFPL1:ENS 00000526945.1:exon6:c.C511T:p.P171S,	11q13.1	.
11	65362999 C	T	TGAGCTGTTG,CCAGGGTGGA	exonic	KCNK7	nonsynonymous SNV	KCNK7:ENST00000340313.4:exon1:c.G245A:p.G82D,KCNK7:ENST00000394217.2:exon1:c.G245A:p.G82D,KCNK7:ENST00000342202.4:exon1:c.G245A:p.G82D,KCNK7:EN T00000394216.2:exon1:c.G245A:p.G82D,	11q13.1	.
11	67266319 T	A	ATCCAGGCCC,GGGGGGAACA	splicing	PITPNM1(ENST00000534749.1:exon10:c.1234-2A>T,ENST00000436757.2:exon11:c.1234-2A>T,ENST00000356404.3:exon11:c.1234-2A>T)	.	.	11q13.2	.
11	68216420 C	A	CCACCCCCAC,CACGCCCCAC	exonic	LRP5	nonsynonymous SNV	LRP5:ENST00000294304.7:exon23:c.C4730A:p.P1577H	11q13.2	.
11	73063977 G	A	GGAGCACCAC,AGCAATTCCT	exonic	ARHGEF17	nonsynonymous SNV	ARHGEF17:ENST00000263674.3:exon3:c.G3373A:p.E1125K	11q13.4	.
11	73076815 G	T	GGGCACCAGT,CTGGTGTCGT	exonic	ARHGEF17	nonsynonymous SNV	ARHGEF17:ENST00000263674.3:exon20:c.G5818T:p.A1940S	11q13.4	.
11	76909569 G	A	CGACGTCATC,TGGCCGTCAA	exonic	MYO7A	nonsynonymous SNV	MYO7A:ENST00000409619.2:exon35:c.G4438A:p.V1480M,MYO7A:ENST00000458637.2:exon34:c.G4471A:p.V1491M,MYO7A:ENST00000409709.3:exon34:c.G4471A:p.V14 M,MYO7A:ENST00000458169.2:exon14:c.G2014A:p.V672M,	11q13.5	.
11	83180399 G	A	TCGTAGTCTC,CTTTGGCCTC	exonic	DLG2	stopgain	DLG2:ENST00000531015.1:exon19:c.C2050T:p.R684X,DLG2:ENST00000330014.6:exon19:c.C1912T:p.R638X,DLG2:ENST00000537455.1:exon20:c.C1399T:p.R467X,DLG2 NST00000420775.2:exon8:c.C595T:p.R199X,DLG2:ENST00000532653.1:exon20:c.C2095T:p.R699X,DLG2:ENST00000404783.3:exon9:c.C583T:p.R195X,DLG2:ENST0000045 7267.1:exon3:c.C205T:p.R69X,DLG2:ENST00000426717.2:exon8:c.C541T:p.R181X,DLG2:ENST00000376104.2:exon25:c.C2464T:p.R822X,DLG2:ENST00000524982.1:exon21 :c.C2137T:p.R713X,DLG2:ENST00000398309.2:exon20:c.C2149T:p.R717X,DLG2:ENST00000376106.3:exon19:c.C541T:p.R181X,DLG2:ENST00000543673.1:exon23:c.C2464	11q14.1	DLG2_ENST
11	108547912 T	C	ACCTTTGAGG,TCTCCGAAAA	exonic	DDX10	nonsynonymous SNV	DDX10:ENST00000526794.1:exon4:c.T479C:p.V160A,DDX10:ENST00000322536.3:exon4:c.T479C:p.V160/	11q22.3	.
11	113935221 G	A	TCAGAGAGCC,GACCATCGGA	exonic	ZBTB16	nonsynonymous SNV	ZBTB16:ENST00000392996.2:exon2:c.G1199A:p.R400Q,ZBTB16:ENST00000335953.4:exon2:c.G1199A:p.R400C	11q23.2	.
11	118220499 T	A	AGAAGATGGT,CGGTACTTCT	exonic	CD3G	nonsynonymous SNV	CD3G:ENST00000532917.1:exon3:c.T121A:p.S41T	11q23.3	.
11	118376002 T	A	GGAGGTGGTC,CACCCTTACC	exonic	KMT2A	nonsynonymous SNV	KMT2A:ENST00000354520.4:exon26:c.T9272A:p.L3091H,KMT2A:ENST00000534358.1:exon27:c.T9395A:p.L3132H,KMT2A:ENST00000389506.5:exon27:c.T9386A:p.L3129	11q23.3	.
11	128840385 A	T	GAGGAGCTCA,AGAAGACACA	exonic	ARHGAP32	nonsynonymous SNV	ARHGAP32:ENST00000527272.1:exon12:c.T3634A:p.L1212M,ARHGAP32:ENST00000392657.3:exon13:c.T3634A:p.L1212M,ARHGAP32:ENST00000310343.9:exon22:c.T4	11q24.3	.
11	130542980 C	T	CAGCTCCAGC,GTCCTGTGAG	exonic	C11orf44	nonsynonymous SNV	C11orf44:ENST00000317019.2:exon1:c.C107T:p.P36L	11q24.3	.
12	661232 C	T	CTTCCAGGTT,ATCTGTCTTT	exonic	B4GALNT3	nonsynonymous SNV	B4GALNT3:ENST00000322843.3:exon10:c.C817T:p.H273Y,B4GALNT3:ENST00000266383.5:exon12:c.C1111T:p.H371\	12p13.33	.
12	1025971 C	T	TTAGTTAAAT,CACTTCAAGA	exonic	RAD52	nonsynonymous SNV	RAD52:ENST00000536177.1:exon8:c.G559A:p.D187N,RAD52:ENST00000430095.2:exon8:c.G559A:p.D187N,RAD52:ENST00000539046.1:exon6:c.G328A:p.D110N,RAD52 NST00000358495.3:exon8:c.G559A:p.D187N,	12p13.33	.
12	4791382 A	T	CCCAGTCGGT,CCTCCTTTTC	exonic	NDUFA9,RP11-234B24.6	nonsynonymous SNV	RP11-234B24.6:ENST00000544741.2:exon2:c.A89T:p.Y30F,NDUFA9:ENST00000266544.5:exon9:c.A812T:p.Y271I	12p13.32	.
12	7045839 C	T	GGGGCCCAGT,CACCGCCCAC	exonic	ATN1	nonsynonymous SNV	ATN1:ENST00000356654.4:exon5:c.C1409T:p.S470F,ATN1:ENST00000396684.2:exon5:c.C1409T:p.S470F	12p13.31	.
12	7076380 T	A	AAGTTTGATG,AGCCAGGGTT	exonic	PHB2	nonsynonymous SNV	PHB2:ENST00000535923.1:exon7:c.A743T:p.Y248F,PHB2:ENST00000440277.1:exon6:c.A629T:p.Y210F,PHB2:ENST00000542912.1:exon7:c.A743T:p.Y248F,PHB2:ENST0 00399433.2:exon7:c.A743T:p.Y248F,	12p13.31	.
12	7848231 A	G	AAGCCCAGAA,TTGGAGAAAG	exonic	GDF3	nonsynonymous SNV	GDF3:ENST00000329913.3:exon1:c.T94C:p.F32L	12p13.31	.
12	9353622 C	T	TAGCTTGAGA,TCTGCCATTG	exonic	PZP	nonsynonymous SNV	PZP:ENST00000381997.2:exon3:c.G143A:p.S48N,PZP:ENST00000261336.2:exon6:c.G536A:p.S179N	12p13.31	.
12	9885661 G	T	GACCACAAAT,ATGTCCCACT	exonic	CLECL1	stopgain	CLECL1:ENST00000327839.3:exon1:c.C200A:p.S67X	12p13.31	.
12	12871066 C	T	CCCAAAGGTG,CTGCAAGGTG	exonic	CDKN1B	nonsynonymous SNV	CDKN1B:ENST00000396340.1:exon1:c.C293T:p.A98V,CDKN1B:ENST00000228872.4:exon1:c.C293T:p.A98\	12p13.1	.
12	12940029 G	T	CTCGCTGAGC,CAACGGGCGC	exonic	APOLD1	nonsynonymous SNV	APOLD1:ENST00000326765.6:exon2:c.G283T:p.A95S,APOLD1:ENST00000356591.4:exon2:c.G190T:p.A64S	12p13.1	.
12	12940266 C	A	GTTTTTCTGC,GCTGGCAGGG	exonic	APOLD1	nonsynonymous SNV	APOLD1:ENST00000326765.6:exon2:c.C520A:p.R174S,APOLD1:ENST00000356591.4:exon2:c.C427A:p.R143S	12p13.1	.
12	13214586 G	A	TCTTCTCCCT,AGGAGGCTCG	exonic	KIAA1467	nonsynonymous SNV	KIAA1467:ENST00000416494.2:exon4:c.G610A:p.E204K,KIAA1467:ENST00000197268.8:exon4:c.G610A:p.E204I	12p13.1	.
12	27066471 C	T	TTCCCTCTTT,TTCCTCGTTT	exonic	ASUN	nonsynonymous SNV	ASUN:ENST00000539625.1:exon12:c.G1421A:p.R474K,ASUN:ENST00000538155.1:exon5:c.G665A:p.R222K,ASUN:ENST00000261191.7:exon14:c.G1724A:p.R575	12p11.23	.
12	27066493 C	T	TTTCGTTCTT,CTCTTCTGGG	exonic	ASUN	nonsynonymous SNV	ASUN:ENST00000539625.1:exon12:c.G1399A:p.E467K,ASUN:ENST00000538155.1:exon5:c.G643A:p.E215K,ASUN:ENST00000261191.7:exon14:c.G1702A:p.E568	12p11.23	.
12	32137512 C	G	GAGCAGTGTT,TCCTTTGGAT	exonic	KIAA1551	nonsynonymous SNV	KIAA1551:ENST00000312561.4:exon4:c.C3623G:p.S1208C	12p11.21	.
12	40879148 A	G	TGAAGAATCA,GGACAACTAG	exonic	MUC19	nonsynonymous SNV	MUC19:ENST00000454784.4:exon49:c.A10693G:p.R3565G	12q12	.
12	43822539 T	C	TCTGCTATTC,ATCAAGAGCA	exonic	ADAMTS20	nonsynonymous SNV	ADAMTS20:ENST00000549670.1:exon7:c.A943G:p.R315G,ADAMTS20:ENST00000395541.2:exon7:c.A907G:p.R303G,ADAMTS20:ENST00000553158.1:exon25:c.A3553G: R1185G,ADAMTS20:ENST00000389420.3:exon25:c.A3553G:p.R1185G,	12q12	.
12	48501881 C	T	TTCCAGCATG,TCATACCAAA	exonic	PFKM	nonsynonymous SNV	PFKM:ENST00000546755.1:exon3:c.C109T:p.L37F,PFKM:ENST00000549366.1:exon3:c.C109T:p.L37F,PFKM:ENST00000340802.6:exon3:c.C109T:p.L37F,PFKM:ENST000 547581.1:exon3:c.C109T:p.L37F,PFKM:ENST00000550257.1:exon2:c.C118T:p.L40F,PFKM:ENST00000552792.1:exon3:c.C109T:p.L37F,PFKM:ENST00000548288.1:exon3:c.C	12q13.11	.
12	49425425 G	A	ACCCTCCCAG,AGGCGGCTCC	exonic	KMT2D	nonsynonymous SNV	KMT2D:ENST00000301067.7:exon39:c.C13063T:p.P4355S	12q13.12	.
12	50045880 G	A	ACTGTCCACA,ACTCCAGGCC	exonic	FMNL3	nonsynonymous SNV	FMNL3:ENST00000550488.1:exon14:c.C1439T:p.S480F,FMNL3:ENST00000352151.5:exon13:c.C1286T:p.S429F,FMNL3:ENST00000335154.5:exon14:c.C1439T:p.S480F,FM L3:ENST00000293590.5:exon14:c.C1439T:p.S480F,	12q13.12	.
12	50744417 C	A	ATCGGGATTT,TGGTACCATT	exonic	FAM186A	nonsynonymous SNV	FAM186A:ENST00000543111.1:exon4:c.G6198T:p.Q2066H,FAM186A:ENST00000543096.1:exon1:c.G231T:p.Q77H,FAM186A:ENST00000327337.5:exon4:c.G6198T:p.Q20	12q13.12	.
12	50745003 C	T	CAGGAGGTCC,CAGGGATGGA	exonic	FAM186A	nonsynonymous SNV	FAM186A:ENST00000543111.1:exon4:c.G5612A:p.G1871E,FAM186A:ENST00000327337.5:exon4:c.G5612A:p.G1871I	12q13.12	.
12	52779093 C	T	CCCAGACCAA,ACCTCTCCCA	exonic	KRT84	nonsynonymous SNV	KRT84:ENST00000257951.3:exon1:c.G277A:p.V93I	12q13.13	.
12	52800050 G	T	GCTGGAAAGA,TGGTACGACA	exonic	KRT82	nonsynonymous SNV	KRT82:ENST00000257974.2:exon1:c.C12A:p.H4Q	12q13.13	.
12	53215801 A	T	ACCCAGGGAG,TGCCACCTCC	exonic	KRT79	nonsynonymous SNV	KRT79:ENST00000330553.5:exon9:c.T1463A:p.I488N	12q13.13	.
12	53227645 C	T	CGCTGGATCT,GGGGTCAATC	exonic	KRT79	nonsynonymous SNV	KRT79:ENST00000330553.5:exon1:c.G400A:p.E134K	12q13.13	.
12	53588083 C	T	ATGTGGACCC,AGGAGGGAGT	exonic	ITGB7	nonsynonymous SNV	ITGB7:ENST00000542497.1:exon9:c.G1207A:p.G403R,ITGB7:ENST00000550743.2:exon8:c.G1207A:p.G403R,ITGB7:ENST00000422257.3:exon10:c.G1207A:p.G403R,ITGB ENST00000338737.4:exon10:c.G1207A:p.G403R,ITGB7:ENST00000267082.5:exon10:c.G1207A:p.G403R,	12q13.13	.
12	53647479 G	C	ATCTTCTCCA,CTTCATGGCA	exonic	MFSD5	nonsynonymous SNV	MFSD5:ENST00000534842.1:exon2:c.G1181C:p.S394T,MFSD5:ENST00000329548.4:exon2:c.G860C:p.S287T,MFSD5:ENST00000551660.2:exon2:c.G1181C:p.S394	12q13.13	.
12	54756524 G	C	GTTCATGGCT,CATAGAGCAC	exonic	GPR84	nonsynonymous SNV	GPR84:ENST00000551809.1:exon1:c.C1112G:p.A371G,GPR84:ENST00000267015.3:exon2:c.C1112G:p.A371C	12q13.13	.
12	56334094 C	A	CCTTTCTCCC,AAGGTCCAAT	exonic	DGKA	nonsynonymous SNV	DGKA:ENST00000557180.1:exon3:c.C203A:p.P68Q	12q13.2	.
12	57009020 G	A	TTCTGGTCAG,AAAGGAATCA	exonic	BAZ2A	nonsynonymous SNV	BAZ2A:ENST00000379441.3:exon3:c.C514T:p.P172S,BAZ2A:ENST00000179765.5:exon3:c.C508T:p.P170S,BAZ2A:ENST00000549884.1:exon3:c.C508T:p.P170S,BAZ2A:EN T00000551812.1:exon3:c.C514T:p.P172S,	12q13.3	.
12	58345662 A	T	AACATCAGAC,TTTGGCGTGC	exonic	XRCC6BP1	nonsynonymous SNV	XRCC6BP1:ENST00000300145.3:exon4:c.A437T:p.H146L	12q14.1	.
12	59272767 C	T	GAAGTCTGTG,CCCCATCCTT	exonic	LRIG3	nonsynonymous SNV	LRIG3:ENST00000320743.3:exon14:c.G1922A:p.G641D,LRIG3:ENST00000379141.4:exon14:c.G1742A:p.G581D	12q14.1	.
12	70925879 G	A	AATGTCCACA,AGTCTTTGGA	exonic	PTPRB	nonsynonymous SNV	PTPRB:ENST00000451516.2:exon29:c.C5516T:p.S1839F,PTPRB:ENST00000538708.1:exon29:c.C5516T:p.S1839F,PTPRB:ENST00000334414.6:exon32:c.C6440T:p.S2147 PTPRB:ENST00000261266.5:exon30:c.C5786T:p.S1929F,PTPRB:ENST00000550358.1:exon31:c.C6176T:p.S2059F,PTPRB:ENST00000550857.1:exon29:c.C5516T:p.S1839F	12q15	.
12	70946726 G	A	TGATACACCC,AATGGAGGCA	exonic	PTPRB	nonsynonymous SNV	PTPRB:ENST00000451516.2:exon18:c.C4294T:p.R1432W,PTPRB:ENST00000538708.1:exon18:c.C4294T:p.R1432W,PTPRB:ENST00000334414.6:exon21:c.C5218T:p.R174 W,PTPRB:ENST00000261266.5:exon19:c.C4564T:p.R1522W,PTPRB:ENST00000550358.1:exon20:c.C4954T:p.R1652W,PTPRB:ENST00000550857.1:exon18:c.C4294T:p.R1	12q15	.
12	78452873 G	A	AGGGGTTCAC,ATGTGACAGT	exonic	NAV3	nonsynonymous SNV	NAV3:ENST00000397909.2:exon12:c.G2614A:p.D872N,NAV3:ENST00000228327.6:exon12:c.G2614A:p.D872N,NAV3:ENST00000536525.2:exon12:c.G2614A:p.D872N,NAV ENST00000266692.7:exon12:c.G2614A:p.D872N,	12q21.2	.
12	91347456 T	C	GTGAAATTCT,CCCCTCTCTG	exonic	CCER1	nonsynonymous SNV	CCER1:ENST00000358859.2:exon1:c.A1064G:p.E355G	12q21.33	.
12	101760410 G	A	ACTCCAGTTC,AGGTGGACAG	exonic	UTP20	nonsynonymous SNV	UTP20:ENST00000261637.4:exon47:c.G6200A:p.R2067Q	12q23.2	.
12	105540305 G	T	CAACAATCAG,TGAGTAGGGT	splicing	KIAA1033(ENST00000332180.5:exon23:c.2409+1G>T,ENST00000550053.1:exon23:	.	.	12q23.3	.
12	106461689 G	A	GTGGCCCGGC,ATCGGGGTTC	exonic	NUAK1	nonsynonymous SNV	NUAK1:ENST00000549704.1:exon4:c.C127T:p.R43C,NUAK1:ENST00000261402.2:exon7:c.C877T:p.R293C,NUAK1:ENST00000553094.1:exon2:c.C22T:p.R8	12q23.3	.
12	110032888 C	G	GGCCACGCCT,TCTGGACCAG	exonic	MVK	nonsynonymous SNV	MVK:ENST00000228510.3:exon10:c.C941G:p.S314C,MVK:ENST00000392727.3:exon9:c.C785G:p.S262C,MVK:ENST00000539696.1:exon4:c.C98G:p.S33C,MVK:ENST0000 41384.1:exon7:c.C359G:p.S120C,MVK:ENST00000539575.1:exon8:c.C785G:p.S262C,	12q24.11	.
12	112499158 G	A	TGTTGGTGTC,ACAAAGGAAC	exonic	NAA25	nonsynonymous SNV	NAA25:ENST00000261745.4:exon12:c.C1184T:p.S395L	12q24.13	.
12	112638500 G	A	GCAGTCCTTC,GTAGCCTGCT	exonic	HECTD4	stopgain	HECTD4:ENST00000550722.1:exon55:c.C8071T:p.R2691X,HECTD4:ENST00000377560.5:exon54:c.C7993T:p.R2665X,HECTD4:ENST00000430131.2:exon54:c.C7243T:p.F	12q24.13	C12orf51_EN
12	123001833 G	A	TTGATCTTGA,CGGGAACGAG	exonic	RSRC2	nonsynonymous SNV	RSRC2:ENST00000526560.2:exon4:c.C224T:p.A75V	12q24.31	.
12	123907655 C	T	GTAACCACAG,ATCTTTTTCT	exonic	RILPL2	nonsynonymous SNV	RILPL2:ENST00000280571.8:exon3:c.G541A:p.A181T	12q24.31	.
12	125292475 T	G	TTCATGGATC,GCAGGGGACA	splicing	SCARB1(ENST00000339570.5:exon8:c.843-2A>C,ENST00000415380.2:exon8:c.843	.	.	12q24.31	.
12	131293186 C	T	CTGGGTTCTT,GTATCCGAAG	exonic	STX2	nonsynonymous SNV	STX2:ENST00000261653.6:exon5:c.G344A:p.R115Q,STX2:ENST00000392373.2:exon5:c.G344A:p.R115C	12q24.33	.
12	133304726 T	A	CAAAACATCC,GATCGAGTTT	exonic	ANKLE2	nonsynonymous SNV	ANKLE2:ENST00000539605.1:exon11:c.A2321T:p.Q774L,ANKLE2:ENST00000538766.1:exon3:c.A572T:p.Q191L,ANKLE2:ENST00000357997.5:exon12:c.A2507T:p.Q836L,A KLE2:ENST00000542282.1:exon2:c.A572T:p.Q191L,ANKLE2:ENST00000542657.1:exon3:c.A572T:p.Q191L,	12q24.33	.
13	21562501 G	A	GGCGGGGGCG,GGGCCGGGGC	exonic	LATS2	nonsynonymous SNV	LATS2:ENST00000382592.4:exon4:c.C1418T:p.P473L,LATS2:ENST00000542899.1:exon4:c.C1418T:p.P473I	13q12.11	.
13	21562820 C	T	CACTGCTGGA,GGAGGTGCTG	exonic	LATS2	nonsynonymous SNV	LATS2:ENST00000382592.4:exon4:c.G1099A:p.V367I,LATS2:ENST00000542899.1:exon4:c.G1099A:p.V367	13q12.11	.
13	21729846 G	T	TTTCTTTGTT,CTGACATCTC	exonic	SKA3	nonsynonymous SNV	SKA3:ENST00000314759.5:exon8:c.C1224A:p.S408R	13q12.11	.
13	33316866 G	A	GGAAAATTAG,TATGCAATTA	splicing	PDS5B(ENST00000315596.10:exon23:c.2612+1G>A,ENST00000450460.1:exon23:c	.	.	13q13.1	.
13	35735950 G	A	AGGATTTCGA,GAATGCCAAT	exonic	NBEA	nonsynonymous SNV	NBEA:ENST00000400445.3:exon23:c.G3925A:p.G1309R,NBEA:ENST00000310336.4:exon24:c.G3925A:p.G1309R,NBEA:ENST00000379939.2:exon23:c.G3925A:p.G1309R BEA:ENST00000540320.1:exon23:c.G3925A:p.G1309R,	13q13.3	NBEA;basecl
13	42042938 G	A	AAAAGAGCTA,AAGCCTTCAT	exonic	RGCC	nonsynonymous SNV	RGCC:ENST00000379359.3:exon4:c.G370A:p.E124K	13q14.11	.
13	42277520 C	T	TCCAGTAGCC,ATGAACGTTT	exonic	VWA8	nonsynonymous SNV	VWA8:ENST00000379310.3:exon27:c.G3144A:p.M1048I	13q14.11	.
13	51523613 C	T	CCAGAACCTT,AGCCTCATTG	exonic	RNASEH2B	nonsynonymous SNV	RNASEH2B:ENST00000422660.1:exon9:c.C713T:p.S238L,RNASEH2B:ENST00000336617.3:exon9:c.C713T:p.S238I	13q14.3	.
13	60240768 C	T	GGAACTGATT,ATTTTTAGAA	exonic	DIAPH3	nonsynonymous SNV	DIAPH3:ENST00000400330.1:exon28:c.G3532A:p.E1178K,DIAPH3:ENST00000400320.1:exon26:c.G3394A:p.E1132K,DIAPH3:ENST00000377908.2:exon27:c.G3499A:p.E1 7K,DIAPH3:ENST00000400324.4:exon28:c.G3532A:p.E1178K,DIAPH3:ENST00000400319.1:exon26:c.G3322A:p.E1108K,	13q21.2	.
13	74420138 G	A	GGCGGTACGG,ATGGATAATG	exonic	KLF12	nonsynonymous SNV	KLF12:ENST00000377666.4:exon4:c.C496T:p.P166S,KLF12:ENST00000377669.2:exon3:c.C496T:p.P166S	13q22.1	.
13	99378449 G	A	AGCCACAAAC,TATGGTAGAT	exonic	SLC15A1	nonsynonymous SNV	SLC15A1:ENST00000376503.5:exon4:c.C173T:p.T58M	13q32.3	.
13	99449430 T	C	TTTCATTTCT,CCTGATACTC	exonic	DOCK9	nonsynonymous SNV	DOCK9:ENST00000376460.1:exon55:c.A6137G:p.E2046G,DOCK9:ENST00000339416.2:exon53:c.A6098G:p.E2033C	13q32.3	.
13	99896768 G	C	TTATTTTGTA,TCCTTTTTGC	splicing	UBAC2(ENST00000403766.3:exon4:c.280-1G>C,ENST00000457666.1:exon4:c.298-	.	.	13q32.3	.
13	100517100 G	A	CTGAAGGTCG,GCCTCAAGTA	exonic	CLYBL	nonsynonymous SNV	CLYBL:ENST00000444838.2:exon4:c.G467A:p.G156E,CLYBL:ENST00000416504.1:exon3:c.G320A:p.G107E,CLYBL:ENST00000376360.1:exon5:c.G569A:p.G190E,CLYBL:E ST00000443887.1:exon2:c.G218A:p.G73E,CLYBL:ENST00000376354.1:exon4:c.G467A:p.G156E,CLYBL:ENST00000339105.4:exon5:c.G569A:p.G190E,CLYBL:ENST0000037	13q32.3	.
13	110839508 G	T	GGGCCAGGAA,ACCCGGATGG	exonic	COL4A1	nonsynonymous SNV	COL4A1:ENST00000375820.4:exon25:c.C1705A:p.L569I	13q34	.
13	111132653 G	A	CAGAGGAGAT,CTGGCTTCAC	exonic	COL4A2	nonsynonymous SNV	COL4A2:ENST00000360467.5:exon31:c.G2674A:p.A892T	13q34	.
13	113864071 G	A	CAAGAAGCTG,TCATCAAGAA	exonic	CUL4A	nonsynonymous SNV	CUL4A:ENST00000375440.4:exon1:c.G130A:p.V44I	13q34	.
14	23240546 T	C	CAGTTGGAGC,AGCAGCCAGG	exonic	OXA1L	nonsynonymous SNV	OXA1L:ENST00000285848.5:exon9:c.T1352C:p.L451P,OXA1L:ENST00000604262.1:exon9:c.T1172C:p.L391P,OXA1L:ENST00000358043.5:exon9:c.T1124C:p.L375	14q11.2	.
14	23518900 A	T	AGACATGGCT,CTGTTGCCAA	exonic	CDH24	nonsynonymous SNV	CDH24:ENST00000267383.5:exon10:c.T1647A:p.S549R,CDH24:ENST00000487137.2:exon10:c.T1533A:p.S511R,CDH24:ENST00000397359.3:exon11:c.T1647A:p.S549R,C H24:ENST00000554034.1:exon9:c.T1533A:p.S511R,	14q11.2	.
14	23894144 G	A	CTTCAGCAGC,GCTTGATCTT	exonic	MYH7	nonsynonymous SNV	MYH7:ENST00000355349.3:exon22:c.C2513T:p.P838L	14q11.2	.
14	24586589 G	A	AGCTTTCCTC,AATGTTGCAC	exonic	DCAF11	nonsynonymous SNV	DCAF11:ENST00000559593.1:exon4:c.G398A:p.R133Q,DCAF11:ENST00000561001.1:exon4:c.G320A:p.R107Q,DCAF11:ENST00000396936.1:exon3:c.G163A:p.E55K,DCAF 1:ENST00000561375.1:exon4:c.G320A:p.R107Q,DCAF11:ENST00000446197.3:exon4:c.G398A:p.R133Q,DCAF11:ENST00000559115.1:exon4:c.G398A:p.R133Q,DCAF11:EN ST00000558638.1:exon5:c.G320A:p.R107Q,DCAF11:ENST00000558914.1:exon3:c.G343A:p.E115K,DCAF11:ENST00000559382.1:exon4:c.G343A:p.E115K,DCAF11:ENST000 00561041.1:exon4:c.G398A:p.R133Q,DCAF11:ENST00000560713.1:exon4:c.G320A:p.R107Q,DCAF11:ENST00000560901.1:exon4:c.G343A:p.E115K,DCAF11:ENST0000039	14q11.2	WDR23;base
14	31574845 C	T	ACCATATATT,TTGAGGAAGG	exonic	HECTD1	nonsynonymous SNV	HECTD1:ENST00000553700.1:exon39:c.G7256A:p.R2419K,HECTD1:ENST00000399332.1:exon39:c.G7256A:p.R2419I	14q12	.
14	33243085 A	G	ACAAAAGAAG,TGGGAAAGGA	exonic	AKAP6	nonsynonymous SNV	AKAP6:ENST00000280979.4:exon12:c.A3574G:p.M1192V,AKAP6:ENST00000557272.1:exon12:c.A3574G:p.M1192\	14q12	.
14	42356857 C	G	GAACACTTGA,ATTCTTATCA	exonic	LRFN5	nonsynonymous SNV	LRFN5:ENST00000298119.4:exon3:c.C1029G:p.D343E,LRFN5:ENST00000554120.1:exon3:c.C1029G:p.D343E,LRFN5:ENST00000554171.1:exon5:c.C1029G:p.D343	14q21.1	.
14	45599011 C	T	TCCAGGGTCT,TTCAGACTTG	exonic	FKBP3	nonsynonymous SNV	FKBP3:ENST00000396062.3:exon3:c.G304A:p.E102K,FKBP3:ENST00000216330.3:exon4:c.G304A:p.E102I	14q21.2	.
14	64066302 C	T	AATATCAAAA,TGATAAAAAT	exonic	WDR89	nonsynonymous SNV	WDR89:ENST00000394942.2:exon2:c.G359A:p.S120N,WDR89:ENST00000267522.3:exon3:c.G359A:p.S120N,WDR89:ENST00000554717.1:exon4:c.G359A:p.S120I	14q23.2	.
14	64066324 A	T	TTGGAAGGGT,ACCCTTGAAG	exonic	WDR89	nonsynonymous SNV	WDR89:ENST00000394942.2:exon2:c.T337A:p.Y113N,WDR89:ENST00000267522.3:exon3:c.T337A:p.Y113N,WDR89:ENST00000554717.1:exon4:c.T337A:p.Y113I	14q23.2	.
14	64990308 A	T	AGCAAAATTT,ATTTGAGGAA	exonic	ZBTB1	nonsynonymous SNV	ZBTB1:ENST00000394712.2:exon2:c.A2086T:p.N696Y,ZBTB1:ENST00000554015.1:exon4:c.A2086T:p.N696\	14q23.3	.
14	68123146 C	T	ACTCTTGGTA,GTTCTAACTG	exonic	VTI1B	nonsynonymous SNV	VTI1B:ENST00000554659.1:exon4:c.G527A:p.R176H	14q24.1	.
14	68290341 T	A	TCCTTACCTG,CAGGTAAATT	exonic	RAD51B	stopgain	RAD51B:ENST00000487861.1:exon2:c.T81A:p.C27X,RAD51B:ENST00000471583.1:exon2:c.T81A:p.C27X,RAD51B:ENST00000485181.1:exon2:c.T81A:p.C27X,RAD51B:EN T00000479335.1:exon2:c.T81A:p.C27X,RAD51B:ENST00000390683.3:exon1:c.T81A:p.C27X,RAD51B:ENST00000553334.1:exon1:c.T81A:p.C27X,RAD51B:ENST00000488612 .1:exon2:c.T81A:p.C27X,RAD51B:ENST00000487270.1:exon2:c.T81A:p.C27X,	14q24.1	.
14	70991356 G	A	CACCACTTCT,GAGAAGTGAA	exonic	ADAM20	nonsynonymous SNV	ADAM20:ENST00000256389.3:exon2:c.C269T:p.P90L	14q24.2	.
14	71199500 C	A	GGCTGACTGG,ATCTCATACA	exonic	MAP3K9	nonsynonymous SNV	MAP3K9:ENST00000554146.1:exon11:c.G1770T:p.M590I,MAP3K9:ENST00000554752.2:exon11:c.G2586T:p.M862I,MAP3K9:ENST00000381250.4:exon10:c.G2517T:p.M83 MAP3K9:ENST00000555993.2:exon12:c.G2628T:p.M876I,MAP3K9:ENST00000553414.1:exon10:c.G1785T:p.M595I,	14q24.2	.
14	77964599 C	A	CCCTGGCCCG,CACTCCGCAA	exonic	ISM2	nonsynonymous SNV	ISM2:ENST00000216481.6:exon2:c.G214T:p.A72S	14q24.3	.
14	81297630 G	A	TGTTTTTCCC,CTCAACCCCT	exonic	CEP128	nonsynonymous SNV	CEP128:ENST00000216517.6:exon13:c.C1066T:p.R356W,CEP128:ENST00000555265.1:exon13:c.C1066T:p.R356W,CEP128:ENST00000281129.3:exon12:c.C1066T:p.R3	14q31.1	CEP128;base
14	86088651 A	T	CAACCAGATA,ACCACATTCC	exonic	FLRT2	nonsynonymous SNV	FLRT2:ENST00000330753.4:exon2:c.A793T:p.N265Y,FLRT2:ENST00000554746.1:exon2:c.A793T:p.N265\	14q31.3	.
14	89319389 A	T	AGATTGGAAA,TGTTACTACA	exonic	TTC8	nonsynonymous SNV	TTC8:ENST00000380656.2:exon8:c.A699T:p.K233N,TTC8:ENST00000345383.5:exon7:c.A669T:p.K223N,TTC8:ENST00000338104.6:exon8:c.A747T:p.K249N,TTC8:ENST00 00358622.5:exon2:c.A105T:p.K35N,TTC8:ENST00000346301.4:exon6:c.A579T:p.K193N,	14q31.3	.
14	103871461 C	T	TCGTACCAGC,GCTCAGGAGC	exonic	MARK3	nonsynonymous SNV	MARK3:ENST00000560417.1:exon2:c.C100T:p.R34C,MARK3:ENST00000335102.5:exon2:c.C100T:p.R34C,MARK3:ENST00000440884.3:exon2:c.C100T:p.R34C,MARK3:EN T00000303622.9:exon2:c.C100T:p.R34C,MARK3:ENST00000561225.1:exon2:c.C64T:p.R22C,MARK3:ENST00000553942.1:exon2:c.C100T:p.R34C,MARK3:ENST0000041668 2.2:exon2:c.C100T:p.R34C,MARK3:ENST00000216288.7:exon2:c.C100T:p.R34C,MARK3:ENST00000429436.2:exon2:c.C100T:p.R34C,	14q32.32	.
14	104642460 C	T	TCCATCAGCT,CTGGCTCAGC	exonic	KIF26A	nonsynonymous SNV	KIF26A:ENST00000315264.7:exon11:c.C2918T:p.S973F,KIF26A:ENST00000423312.2:exon12:c.C3335T:p.S1112I	14q32.33	.
15	30033617 C	G	GGTTGCCCAA,TTGGGCATAC	exonic	TJP1	nonsynonymous SNV	TJP1:ENST00000356107.6:exon10:c.G1174C:p.V392L,TJP1:ENST00000545208.2:exon10:c.G1174C:p.V392L,TJP1:ENST00000346128.6:exon10:c.G1174C:p.V392L,TJP1:E ST00000400011.2:exon11:c.G1186C:p.V396L,	15q13.1	.
15	41105073 A	G	GCGACTAGCC,TGCTGCGGGG	exonic	ZFYVE19	nonsynonymous SNV	ZFYVE19:ENST00000570108.1:exon8:c.A934G:p.M312V,ZFYVE19:ENST00000355341.4:exon7:c.A1003G:p.M335V,ZFYVE19:ENST00000564258.1:exon7:c.A478G:p.M160\ FYVE19:ENST00000336455.5:exon8:c.A973G:p.M325V,	15q15.1	.
15	43477796 C	G	GCTCCTGCCT,GAGTGCGGGG	exonic	CCNDBP1	nonsynonymous SNV	CCNDBP1:ENST00000566515.1:exon1:c.C100G:p.R34G,CCNDBP1:ENST00000562553.1:exon1:c.C100G:p.R34G,CCNDBP1:ENST00000300213.4:exon1:c.C100G:p.R34G, CNDBP1:ENST00000568507.1:exon1:c.C100G:p.R34G,CCNDBP1:ENST00000569745.1:exon1:c.C100G:p.R34G,CCNDBP1:ENST00000563065.1:exon1:c.C100G:p.R34G,CC NDBP1:ENST00000565296.1:exon1:c.C100G:p.R34G,CCNDBP1:ENST00000567434.1:exon1:c.C100G:p.R34G,	15q15.2	.
15	65350841 C	A	GCGTGCAAGG,AAGCAGCTCC	exonic	RASL12	nonsynonymous SNV	RASL12:ENST00000434605.2:exon4:c.G316T:p.A106S,RASL12:ENST00000220062.4:exon4:c.G349T:p.A117S,RASL12:ENST00000421977.3:exon3:c.G292T:p.A98	15q22.31	.
15	71535236 C	T	CTGCAGGCTG,GGAGGCCCCC	exonic	THSD4	nonsynonymous SNV	THSD4:ENST00000261862.6:exon4:c.C713T:p.A238V,THSD4:ENST00000355327.3:exon5:c.C713T:p.A238\	15q23	.
15	71535266 C	T	CTACCTTTGA,CCATGATCAA	exonic	THSD4	nonsynonymous SNV	THSD4:ENST00000261862.6:exon4:c.C743T:p.T248I,THSD4:ENST00000355327.3:exon5:c.C743T:p.T248	15q23	.
15	73566193 C	T	TGCAGAGATA,ATGACTGGGT	exonic	NEO1	nonsynonymous SNV	NEO1:ENST00000261908.6:exon20:c.C3007T:p.H1003Y,NEO1:ENST00000339362.5:exon21:c.C3007T:p.H1003Y,NEO1:ENST00000558964.1:exon20:c.C3007T:p.H1003Y,N O1:ENST00000560262.1:exon20:c.C3007T:p.H1003Y,NEO1:ENST00000560328.1:exon16:c.C2047T:p.H683Y,	15q24.1	.
15	76170358 C	A	TGTTAAACTG,AGAAGTAAGT	exonic	UBE2Q2	nonsynonymous SNV	UBE2Q2:ENST00000338677.4:exon7:c.C730A:p.Q244K,UBE2Q2:ENST00000267938.4:exon7:c.C730A:p.Q244K,UBE2Q2:ENST00000561851.1:exon7:c.C682A:p.Q228K,UBE Q2:ENST00000569423.1:exon6:c.C625A:p.Q209K,	15q24.2	.
15	77328143 C	T	CTGCTCTTAG,GTCCACAGAG	exonic	PSTPIP1	nonsynonymous SNV	PSTPIP1:ENST00000559295.1:exon13:c.C929T:p.A310V,PSTPIP1:ENST00000267939.5:exon13:c.C926T:p.A309V,PSTPIP1:ENST00000558012.1:exon14:c.C986T:p.A329	15q24.3	.
15	90173634 G	A	CGAAGCACCC,CTGGCGGCAT	exonic	KIF7	nonsynonymous SNV	KIF7:ENST00000394412.3:exon16:c.C3202T:p.R1068W	15q26.1	.
15	99671936 C	A	AGCCAGGCTG,AAGGCACATA	exonic	SYNM	nonsynonymous SNV	SYNM:ENST00000328642.7:exon5:c.C3368A:p.A1123E,SYNM:ENST00000560674.1:exon4:c.C2513A:p.A838E,SYNM:ENST00000336292.6:exon5:c.C3368A:p.A1123	15q26.3	.
16	1435356 G	A	GGGGCCGGCG,GTGGACCGCT	exonic	UNKL	nonsynonymous SNV	UNKL:ENST00000508903.2:exon10:c.C1117T:p.P373S,UNKL:ENST00000397462.1:exon14:c.C1714T:p.P572S,UNKL:ENST00000389221.4:exon10:c.C1108T:p.P370	16p13.3	.
16	1470507 C	G	ATGGGGCGGT,AGGGTCGTCC	exonic	C16orf91	nonsynonymous SNV	C16orf91:ENST00000442039.2:exon2:c.G139C:p.D47H,C16orf91:ENST00000310355.1:exon4:c.G610C:p.D204I	16p13.3	.
16	1552987 G	C	CTCCGGCAGC,CATGGACATC	exonic	TELO2	nonsynonymous SNV	TELO2:ENST00000262319.6:exon15:c.G1826C:p.R609P	16p13.3	TELO2;basec
16	1838696 C	T	CTGGACGCGA,GCCCGCGTGC	exonic	NUBP2	nonsynonymous SNV	NUBP2:ENST00000262302.9:exon7:c.C797T:p.T266M,NUBP2:ENST00000543305.1:exon6:c.C374T:p.T125M,NUBP2:ENST00000568706.1:exon6:c.C374T:p.T125M,NUBP2 NST00000565987.1:exon8:c.C617T:p.T206M,	16p13.3	.
16	3169670 G	A	GAAGCCCTAC,CCTGCACTGA	exonic	ZNF205	nonsynonymous SNV	ZNF205:ENST00000382192.3:exon7:c.G1009A:p.A337T,ZNF205:ENST00000219091.4:exon7:c.G1009A:p.A337	16p13.3	.
16	3340443 G	A	AGCTCCAATC,GATTCGCCAC	exonic	ZNF263	nonsynonymous SNV	ZNF263:ENST00000219069.5:exon6:c.G1937A:p.R646Q,ZNF263:ENST00000538765.1:exon2:c.G881A:p.R294Q,ZNF263:ENST00000574674.1:exon1:c.G41A:p.R14	16p13.3	.
16	3458552 G	C	GAATACATCA,CAGCCCCCTA	exonic	ZNF174	nonsynonymous SNV	ZNF174:ENST00000268655.4:exon3:c.G857C:p.S286T,ZNF174:ENST00000571936.1:exon4:c.G857C:p.S286	16p13.3	.
16	3613817 C	T	CTGCCCCTCC,CGCTGAGGGC	exonic	NLRC3	nonsynonymous SNV	NLRC3:ENST00000301749.7:exon4:c.G1121A:p.G374E,NLRC3:ENST00000359128.5:exon5:c.G1121A:p.G374E,NLRC3:ENST00000324659.8:exon6:c.G1067A:p.G356E,NLR 3:ENST00000448023.2:exon3:c.G1262A:p.G421E,	16p13.3	.
16	4935868 C	T	ACCACCGATT,CTGAGGCCCC	exonic	PPL	nonsynonymous SNV	PPL:ENST00000590782.2:exon22:c.G2782A:p.E928K,PPL:ENST00000592772.1:exon10:c.G1051A:p.E351K,PPL:ENST00000345988.2:exon22:c.G2788A:p.E930	16p13.3	.
16	15814718 T	C	TTGCCTCCTC,TCTCCTCATT	exonic	MYH11	nonsynonymous SNV	MYH11:ENST00000300036.5:exon33:c.A4769G:p.K1590R,MYH11:ENST00000396324.3:exon34:c.A4790G:p.K1597R,MYH11:ENST00000576790.2:exon33:c.A4769G:p.K159 ,MYH11:ENST00000452625.2:exon34:c.A4790G:p.K1597R,	16p13.11	.
16	20974744 C	T	AACTCCCGGA,CGCTGGCACC	exonic	DNAH3	nonsynonymous SNV	DNAH3:ENST00000261383.3:exon53:c.G10462A:p.V3488I	16p12.3	.
16	20976570 G	A	CTCGCAGGCC,ACGATACATT	exonic	DNAH3	nonsynonymous SNV	DNAH3:ENST00000261383.3:exon53:c.C8636T:p.S2879L	16p12.3	.
16	21245089 G	T	AGCTGCTAGT,ATAGTTACCT	exonic	ANKS4B	nonsynonymous SNV	ANKS4B:ENST00000311620.5:exon1:c.G31T:p.D11Y	16p12.2	.
16	23718360 G	A	GCTGCGGCAG,GAGAGGCATG	exonic	ERN2	nonsynonymous SNV	ERN2:ENST00000256797.4:exon5:c.C491T:p.P164L,ERN2:ENST00000457008.2:exon5:c.C347T:p.P116I	16p12.2	.
16	23718361 G	A	CTGCGGCAGG,AGAGGCATGA	exonic	ERN2	nonsynonymous SNV	ERN2:ENST00000256797.4:exon5:c.C490T:p.P164S,ERN2:ENST00000457008.2:exon5:c.C346T:p.P116S	16p12.2	.
16	28506640 G	A	CATGAAGTGG,GAGCTCAGCT	exonic	APOBR	nonsynonymous SNV	APOBR:ENST00000431282.1:exon2:c.G278A:p.G93E,APOBR:ENST00000328423.5:exon2:c.G278A:p.G93E,APOBR:ENST00000564831.1:exon2:c.G278A:p.G93I	16p11.2	.
16	28855381 C	T	AGGTTATCTC,GGCCTCGGCC	exonic	TUFM	nonsynonymous SNV	TUFM:ENST00000313511.3:exon8:c.G964A:p.G322R	16p11.2	.
16	31073354 G	A	TGGGCGCGCT,GTGGCGACGG	exonic	ZNF668	stopgain	ZNF668:ENST00000538906.1:exon3:c.C895T:p.Q299X,ZNF668:ENST00000300849.4:exon3:c.C895T:p.Q299X,ZNF668:ENST00000394983.2:exon3:c.C895T:p.Q299X,ZNF66 ENST00000539836.3:exon4:c.C964T:p.Q322X,ZNF668:ENST00000417110.2:exon1:c.G542A:p.W181X,ZNF668:ENST00000535577.1:exon3:c.C895T:p.Q299X,ZNF668:ENST0	16p11.2	.
16	48175187 A	G	TGTCCTCTGG,ATTTCCACAC	exonic	ABCC12	nonsynonymous SNV	ABCC12:ENST00000416054.1:exon3:c.T353C:p.F118S,ABCC12:ENST00000497206.2:exon3:c.T353C:p.F118S,ABCC12:ENST00000311303.3:exon3:c.T353C:p.F118S,ABC 2:ENST00000448542.1:exon3:c.T353C:p.F118S,ABCC12:ENST00000529504.1:exon3:c.T353C:p.F118S,ABCC12:ENST00000534418.1:exon3:c.T353C:p.F118S,ABCC12:ENST 00000533639.1:exon4:c.T353C:p.F118S,ABCC12:ENST00000532494.1:exon3:c.T353C:p.F118S,ABCC12:ENST00000529084.1:exon5:c.T353C:p.F118S,	16q12.1	.
16	54966695 T	A	TAAAATGACG,GGACGCCGCG	exonic	IRX5	nonsynonymous SNV	IRX5:ENST00000320990.5:exon2:c.T535A:p.W179R,IRX5:ENST00000394636.4:exon2:c.T535A:p.W179R,IRX5:ENST00000558597.1:exon1:c.T337A:p.W113I	16q12.2	.
16	55363086 C	T	AGAGACTCCG,GTGCGACGAG	exonic	IRX6	nonsynonymous SNV	IRX6:ENST00000290552.7:exon5:c.C1196T:p.A399V	16q12.2	.
16	57054811 G	A	CAAGCTGCAT,TCCAGGGTTC	exonic	NLRC5	nonsynonymous SNV	NLRC5:ENST00000308149.7:exon3:c.G187A:p.V63I,NLRC5:ENST00000544641.1:exon3:c.G187A:p.V63I,NLRC5:ENST00000436936.1:exon3:c.G187A:p.V63I,NLRC5:ENST 000539144.1:exon1:c.G187A:p.V63I,NLRC5:ENST00000539881.1:exon3:c.G187A:p.V63I,NLRC5:ENST00000262510.6:exon3:c.G187A:p.V63I,	16q13	.
16	57789270 C	A	GTGAAGATCC,CCAGCAGGCC	exonic	KATNB1	nonsynonymous SNV	KATNB1:ENST00000379661.3:exon16:c.C1430A:p.P477H	16q21	.
16	67262476 G	C	AGCGTTCTCT,AGGATGGGGC	exonic	TMEM208	nonsynonymous SNV	TMEM208:ENST00000562235.1:exon4:c.G241C:p.E81Q,TMEM208:ENST00000563953.1:exon4:c.G31C:p.E11Q,TMEM208:ENST00000565201.1:exon4:c.G241C:p.E81Q,TM M208:ENST00000304800.9:exon4:c.G241C:p.E81Q,	16q22.1	.
16	67644983 C	G	GGCACAGTGG,TCCAGAAGCA	exonic	CTCF	nonsynonymous SNV	CTCF:ENST00000264010.4:exon3:c.C248G:p.A83G	16q22.1	.
16	67688578 G	A	GGCAACGCTG,GTGCCAGACC	exonic	RLTPR	nonsynonymous SNV	RLTPR:ENST00000334583.6:exon31:c.G3565A:p.G1189S,RLTPR:ENST00000545661.1:exon32:c.G3457A:p.G1153S	16q22.1	.
16	67719479 T	A	GATGTTCATC,CCTCAGCAAG	exonic	GFOD2	nonsynonymous SNV	GFOD2:ENST00000268797.7:exon2:c.A140T:p.E47V,GFOD2:ENST00000602496.1:exon2:c.A140T:p.E47\	16q22.1	.
16	68405225 C	T	CCCTGAATCC,CGTCCTGCTG	exonic	SMPD3	nonsynonymous SNV	SMPD3:ENST00000568373.1:exon1:c.G860A:p.G287E,SMPD3:ENST00000563226.1:exon1:c.G860A:p.G287E,SMPD3:ENST00000219334.5:exon3:c.G860A:p.G287	16q22.1	.
16	69725775 A	T	ACAGCAGCAG,TTCAGCCCAA	exonic	NFAT5	nonsynonymous SNV	NFAT5:ENST00000393742.2:exon13:c.A1765T:p.I589F,NFAT5:ENST00000354436.2:exon12:c.A1993T:p.I665F,NFAT5:ENST00000349945.1:exon14:c.A1765T:p.I589F,NFAT ENST00000567239.1:exon13:c.A2044T:p.I682F,NFAT5:ENST00000432919.1:exon13:c.A2047T:p.I683F,NFAT5:ENST00000566899.1:exon13:c.A1765T:p.I589F,	16q22.1	.
16	76569569 T	A	GTAGGGGACA,TGCAGCAGCT	exonic	CNTNAP4	nonsynonymous SNV	CNTNAP4:ENST00000476707.1:exon17:c.T2892A:p.H964Q,CNTNAP4:ENST00000377504.4:exon18:c.T2736A:p.H912Q,CNTNAP4:ENST00000307431.8:exon19:c.T2880A: H960Q,CNTNAP4:ENST00000478060.1:exon17:c.T2664A:p.H888Q,	16q23.1	.
16	85699839 C	G	GCCGCTGTCC,ACAGCACCAA	exonic	GSE1	nonsynonymous SNV	GSE1:ENST00000253458.7:exon13:c.C3016G:p.H1006D,GSE1:ENST00000405402.2:exon12:c.C2704G:p.H902D,GSE1:ENST00000393243.1:exon12:c.C2797G:p.H933	16q24.1	.
16	88501827 C	T	AGCATTCTTC,AGTCTCTGCT	exonic	ZNF469	nonsynonymous SNV	ZNF469:ENST00000437464.1:exon2:c.C7865T:p.P2622L,ZNF469:ENST00000565624.1:exon1:c.C7949T:p.P2650I	16q24.2	.
16	88505055 C	T	ACCCCCAAAG,CAAACCCGGC	exonic	ZNF469	nonsynonymous SNV	ZNF469:ENST00000437464.1:exon2:c.C11093T:p.A3698V,ZNF469:ENST00000565624.1:exon1:c.C11177T:p.A3726\	16q24.2	.
16	89713612 G	A	GCCACATACC,ATGTATGGAC	exonic	CHMP1A	nonsynonymous SNV	CHMP1A:ENST00000535997.2:exon4:c.C188T:p.S63L,CHMP1A:ENST00000550102.1:exon5:c.C353T:p.S118L,CHMP1A:ENST00000549328.1:exon5:c.C380T:p.S127L,CHM 1A:ENST00000397901.3:exon5:c.C380T:p.S127L,	16q24.3	.
17	1946256 T	G	ACTGACAAAG,CATCCCCTCT	exonic	OVCA2	nonsynonymous SNV	OVCA2:ENST00000572195.1:exon2:c.T542G:p.V181G	17p13.3	.
17	6927794 G	A	CCTCCACCAG,TAGTCCCAGG	exonic	BCL6B	nonsynonymous SNV	BCL6B:ENST00000293805.5:exon4:c.G476A:p.G159D	17p13.1	.
17	7221894 C	T	TGGTCCACCC,ATTAACATGA	exonic	NEURL4,RP11-542C16.2	nonsynonymous SNV	NEURL4:ENST00000570460.1:exon22:c.G3712A:p.G1238R,NEURL4:ENST00000315614.7:exon23:c.G3778A:p.G1260R,NEURL4:ENST00000399464.2:exon23:c.G3784A:p.(262R,NEURL4:ENST00000573186.1:exon22:c.G3036A:p.M1012I,	17p13.1	.
17	7496316 G	A	GCTAATAGAT,AAGAATTGTA	exonic	FXR2	nonsynonymous SNV	FXR2:ENST00000250113.7:exon13:c.C1514T:p.S505L	17p13.1	.
17	7577538 C	T	GATGGGCCTC,GGTTCATGCC	exonic	TP53	nonsynonymous SNV	TP53:ENST00000509690.1:exon4:c.G347A:p.R116Q,TP53:ENST00000514944.1:exon6:c.G464A:p.R155Q,TP53:ENST00000445888.2:exon7:c.G743A:p.R248Q,TP53:ENST(000455263.2:exon7:c.G743A:p.R248Q,TP53:ENST00000269305.4:exon7:c.G743A:p.R248Q,TP53:ENST00000413465.2:exon6:c.G743A:p.R248Q,TP53:ENST00000359597.4:ex on6:c.G743A:p.R248Q,TP53:ENST00000420246.2:exon7:c.G743A:p.R248Q,	17p13.1	TP53_ENST(
17	11513769 G	A	GTGATCAAAT,GAGCTACCAA	exonic	DNAH9	stopgain	DNAH9:ENST00000579828.1:exon3:c.G671A:p.W224X,DNAH9:ENST00000454412.2:exon3:c.G671A:p.W224X,DNAH9:ENST00000262442.4:exon3:c.G671A:p.W224)	17p12	.
17	16004826 C	A	GGATCACAAA,AGAACCCTCT	exonic	NCOR1	nonsynonymous SNV	NCOR1:ENST00000395848.1:exon17:c.G2149T:p.V717F,NCOR1:ENST00000395851.1:exon19:c.G2476T:p.V826F,NCOR1:ENST00000268712.3:exon20:c.G2428T:p.V810	17p11.2	.
17	16456710 G	A	ATCCTCCACT,GAGTACATGC	exonic	ZNF287	nonsynonymous SNV	ZNF287:ENST00000395825.3:exon6:c.C746T:p.P249L,ZNF287:ENST00000395824.1:exon6:c.C746T:p.P249I	17p11.2	.

17	19284226	C	T	CTCATGCTCT,TTTGCATGAG	exonic	MAPK7	nonsynonymous SNV	MAPK7:ENST00000482850.1:exon2:c.C287T:p.S96F,MAPK7:ENST00000299612.7:exon2:c.C287T:p.S96F,MAPK7:ENST00000308406.5:exon4:c.C704T:p.S235F,MAPK7:EN T00000395604.3:exon4:c.C704T:p.S235F,MAPK7:ENST00000395602.4:exon4:c.C704T:p.S235F,MAPK7:ENST00000443215.1:exon4:c.C704T:p.S235F,	17p11.2	.
17	26864425	C	T	GTACCTCAGC,CCAGCTCCAA	exonic	FOXN1	nonsynonymous SNV	FOXN1:ENST00000226247.2:exon8:c.C1918T:p.P640S,FOXN1:ENST00000579795.1:exon9:c.C1918T:p.P640S	17q11.2	.
17	27018029	G	A	GATGAGACAG,GCTGTGGCAG	exonic	SUPT6H	nonsynonymous SNV	SUPT6H:ENST00000314616.6:exon27:c.G3581A:p.G1194E,SUPT6H:ENST00000347486.4:exon28:c.G3581A:p.G1194E	17q11.2	.
17	27185622	T	A	AAGTCAGTTC,CCTGGAGCTC	exonic	ERAL1	nonsynonymous SNV	ERAL1:ENST00000254928.5:exon7:c.T740A:p.L247H	17q11.2	.
17	28417577	G	C	GCCAAAATAG,ATGTTGTTGT	exonic	EFCAB5	nonsynonymous SNV	EFCAB5:ENST00000419434.1:exon15:c.G2868C:p.R956S,EFCAB5:ENST00000394835.3:exon20:c.G3822C:p.R1274S,EFCAB5:ENST00000320856.5:exon18:c.G3450C:p.R	17q11.2	.
17	29654653	C	T	TTCACCTTAA,CATTGCAAAC	exonic	NF1	nonsynonymous SNV	NF1:ENST00000356175.3:exon37:c.C5342T:p.T1781I,NF1:ENST00000456735.2:exon29:c.C4340T:p.T1447I,NF1:ENST00000358273.4:exon38:c.C5405T:p.T1802	17q11.2	.
17	29687617	C	T	CTCCTGACTC,CACATCTCCT	exonic	NF1	nonsynonymous SNV	NF1:ENST00000444181.2:exon14:c.C1652T:p.P551L,NF1:ENST00000356175.3:exon56:c.C8210T:p.P2737L,NF1:ENST00000456735.2:exon48:c.C7208T:p.P2403L,NF1:ENS 00000358273.4:exon57:c.C8273T:p.P2758L,	17q11.2	.
17	30519248	C	T	AAAGCCCTTA,TCGTATATTT	exonic	RHOT1	nonsynonymous SNV	RHOT1:ENST00000394692.2:exon9:c.C569T:p.T190I,RHOT1:ENST00000333942.6:exon9:c.C569T:p.T190I,RHOT1:ENST00000358365.3:exon9:c.C569T:p.T190I,RHOT1:EN T00000583994.1:exon10:c.C188T:p.T63I,RHOT1:ENST00000354266.3:exon9:c.C506T:p.T169I,RHOT1:ENST00000581031.1:exon9:c.C569T:p.T190I,RHOT1:ENST000005810 94.1:exon9:c.C569T:p.T190I,RHOT1:ENST00000545287.2:exon9:c.C569T:p.T190I,	17q11.2	.
17	38907572	G	A	TGCAGAACTT,CATTTCCTAG	exonic	KRT25	stopgain	KRT25:ENST00000312150.4:exon4:c.C676T:p.Q226X	17q21.2	.
17	40259705	G	C	ATCCTGCAGC,CAGCCAAGGC	exonic	DHX58	nonsynonymous SNV	DHX58:ENST00000251642.3:exon8:c.C914G:p.A305G	17q21.2	.
17	40343043	G	A	CACCTGGTGA,GCTGGCGCGG	exonic	GHDC	nonsynonymous SNV	GHDC:ENST00000414034.3:exon6:c.C1075T:p.L359F,GHDC:ENST00000436923.2:exon5:c.C1075T:p.L359F,GHDC:ENST00000301671.8:exon5:c.C1075T:p.L359F,GHDC:E ST00000593209.1:exon6:c.C1075T:p.L359F,GHDC:ENST00000587427.1:exon6:c.C1075T:p.L359F,GHDC:ENST00000428494.2:exon5:c.C958T:p.L320F,	17q21.2	.
17	40947060	T	C	GAGTTTCCGG,CCCACTCTCT	exonic	WNK4	nonsynonymous SNV	WNK4:ENST00000246914.5:exon14:c.T2621C:p.V874A	17q21.31	.
17	44115983	G	A	TCGCACCAAG,GACTGTGTGG	exonic	KANSL1	nonsynonymous SNV	KANSL1:ENST00000575318.1:exon8:c.C2273T:p.P758L,KANSL1:ENST00000572904.1:exon10:c.C2462T:p.P821L,KANSL1:ENST00000574590.1:exon10:c.C2462T:p.P821L ANSL1:ENST00000262419.6:exon10:c.C2462T:p.P821L,KANSL1:ENST00000393476.3:exon9:c.C344T:p.P115L,KANSL1:ENST00000432791.1:exon9:c.C2462T:p.P821L,	17q21.31	.
17	45232232	T	A	TTAGAAGGTC,AAAAGGAAGA	splicing	CDC27(ENST00000576484.1:exon4:c.251-2A>T)	.	.	17q21.32	.
17	45679046	A	G	GCCAAACTAA,AATTCTAATG	exonic	NPEPPS	nonsynonymous SNV	NPEPPS:ENST00000530173.1:exon16:c.A1661G:p.K554R,NPEPPS:ENST00000544660.1:exon14:c.A1433G:p.K478R,NPEPPS:ENST00000322157.4:exon15:c.A1673G:p.K5	17q21.32	.
17	48186744	G	A	CTTTGGGACC,ATGCTGTCAT	exonic	PDK2	nonsynonymous SNV	PDK2:ENST00000007708.3:exon11:c.G871A:p.D291N,PDK2:ENST00000503176.1:exon10:c.G1063A:p.D355N	17q21.33	.
17	48267080	A	G	AGCACCAGGG,AACCAGTAGC	exonic	COL1A1	nonsynonymous SNV	COL1A1:ENST00000225964.5:exon38:c.T2627C:p.F876S	17q21.33	.
17	48272944	C	A	GCCAGCAGCA,CAGCAGGGCC	exonic	COL1A1	nonsynonymous SNV	COL1A1:ENST00000225964.5:exon17:c.G1139T:p.G380V	17q21.33	.
17	61890719	A	C	TACCCTACTC,CTCCCAAGGA	exonic	DDX42	nonsynonymous SNV	DDX42:ENST00000359353.5:exon14:c.A1450C:p.T484P,DDX42:ENST00000389924.2:exon15:c.A1807C:p.T603P,DDX42:ENST00000578681.1:exon16:c.A1807C:p.T603P,DD 42:ENST00000583590.1:exon16:c.A1807C:p.T603P,DDX42:ENST00000457800.2:exon14:c.A1807C:p.T603P,	17q23.3	.
17	62020390	G	A	AGGCAGATGA,GATCATGATG	exonic	SCN4A	nonsynonymous SNV	SCN4A:ENST00000578147.1:exon23:c.C4084T:p.L1362F,SCN4A:ENST00000435607.1:exon23:c.C4084T:p.L1362F	17q23.3	.
17	72248512	C	T	CACTTCACCA,CAGGTGGGCT	exonic	TTYH2	nonsynonymous SNV	TTYH2:ENST00000529107.1:exon11:c.C1193T:p.T398I,TTYH2:ENST00000269346.4:exon11:c.C1256T:p.T419I,TTYH2:ENST00000441391.2:exon5:c.C293T:p.T98	17q25.1	.
17	72305432	G	A	CTGGAGCCCC,TGAGGCCGAC	exonic	DNAI2	nonsynonymous SNV	DNAI2:ENST00000311014.6:exon10:c.G1252A:p.V418M,DNAI2:ENST00000579490.1:exon9:c.G1423A:p.V475M,DNAI2:ENST00000307504.5:exon9:c.G823A:p.V275M,DNA ENST00000446837.2:exon9:c.G1252A:p.V418M,DNAI2:ENST00000582036.1:exon10:c.G1252A:p.V418M,	17q25.1	.
17	73915936	C	T	TACGATGTTT,TAGCACCTTG	exonic	FBF1	nonsynonymous SNV	FBF1:ENST00000586717.1:exon19:c.G1909A:p.E637K,FBF1:ENST00000319129.5:exon19:c.G1906A:p.E636K,FBF1:ENST00000389570.4:exon19:c.G1909A:p.E637	17q25.1	.
17	74387502	C	G	CCGCCAGCCA,CAGTGCTAAA	exonic	UBE2O	nonsynonymous SNV	UBE2O:ENST00000319380.7:exon18:c.G3401C:p.G1134A	17q25.1	.
17	76450732	G	A	GGCCACGTCC,CGTCATCTGT	exonic	DNAH17	nonsynonymous SNV	DNAH17:ENST00000585328.1:exon64:c.C10211T:p.A3404V,DNAH17:ENST00000389840.5:exon64:c.C10184T:p.A3395V	17q25.3	.
17	79166311	C	T	CTGCCCTTCT,AAACTCAGCC	exonic	AZI1	nonsynonymous SNV	AZI1:ENST00000573053.1:exon7:c.G829A:p.E277K,AZI1:ENST00000450824.2:exon20:c.G2512A:p.E838K,AZI1:ENST00000374782.3:exon19:c.G2404A:p.E802K,AZI1:ENST 000269392.4:exon20:c.G2521A:p.E841K,AZI1:ENST00000575907.1:exon19:c.G2413A:p.E805K,	17q25.3	.
17	79517375	T	A	TTCTTCGGGC,GCTCAGGGCC	exonic	C17orf70	nonsynonymous SNV	C17orf70:ENST00000537152.1:exon3:c.A692T:p.Q231L,C17orf70:ENST00000327787.8:exon3:c.A1145T:p.Q382L,C17orf70:ENST00000541246.1:exon2:c.A692T:p.Q231L,C1 rf70:ENST00000544302.1:exon3:c.A692T:p.Q231L,	17q25.3	.
18	2890790	G	A	GATGCCAGTA,AACACGGGCA	exonic	EMILIN2	nonsynonymous SNV	EMILIN2:ENST00000254528.3:exon4:c.G665A:p.R222K	18p11.32	.
18	7231237	T	C	GACGATGCCC,GCTCTCGGGA	exonic	LRRC30	nonsynonymous SNV	LRRC30:ENST00000383467.2:exon1:c.T101C:p.L34P	18p11.23	.
18	19994626	C	A	CTTGCCACAG,AAGAGAAAAG	exonic	CTAGE1	nonsynonymous SNV	CTAGE1:ENST00000525417.1:exon1:c.G192T:p.L64F	18q11.2	.
18	21109149	G	A	TCAGGCGGTG,AAGCAGGGCA	exonic	C18orf8	nonsynonymous SNV	C18orf8:ENST00000269221.3:exon15:c.G1303A:p.E435K,C18orf8:ENST00000590868.1:exon13:c.G1159A:p.E387K	18q11.2	.
18	21526168	G	A	GAGGGCCCGG,AGGGAAGTTT	exonic	LAMA3	nonsynonymous SNV	LAMA3:ENST00000399516.3:exon69:c.G9103A:p.E3035K,LAMA3:ENST00000269217.6:exon33:c.G4444A:p.E1482K,LAMA3:ENST00000587184.1:exon32:c.G4276A:p.E1426 LAMA3:ENST00000313654.9:exon70:c.G9271A:p.E3091K,	18q11.2	.
18	21805063	G	A	TTTTTCTTCG,ACATTCTGTG	exonic	OSBPL1A	nonsynonymous SNV	OSBPL1A:ENST00000319481.3:exon17:c.C1544T:p.S515F,OSBPL1A:ENST00000578055.1:exon3:c.C5T:p.S2F,OSBPL1A:ENST00000357041.4:exon5:c.C398T:p.S133F,OS PL1A:ENST00000399443.3:exon3:c.C5T:p.S2F,	18q11.2	.
18	22807637	G	A	AGAGCAAGTC,GATCATCTTC	exonic	ZNF521	nonsynonymous SNV	ZNF521:ENST00000580488.1:exon4:c.C245T:p.P82L,ZNF521:ENST00000399425.2:exon4:c.C245T:p.P82L,ZNF521:ENST00000361524.3:exon4:c.C245T:p.P82L,ZNF521:EN T00000538137.2:exon4:c.C245T:p.P82L,ZNF521:ENST00000577801.1:exon4:c.C245T:p.P82L,	18q11.2	.
18	29039846	G	A	AGATGCAGAT,AACCAAACCA	exonic	DSG3	nonsynonymous SNV	DSG3:ENST00000257189.4:exon6:c.G556A:p.E186K	18q12.1	.
18	29055826	A	T	GGTGAAGGCA,AGAAGTTCAG	exonic	DSG3	nonsynonymous SNV	DSG3:ENST00000257189.4:exon16:c.A2603T:p.K868I	18q12.1	.
18	40854156	C	T	TTCTTTACTT,ATTTTTATCA	exonic	SYT4	nonsynonymous SNV	SYT4:ENST00000590752.1:exon2:c.G184A:p.E62K,SYT4:ENST00000255224.3:exon2:c.G238A:p.E80K	18q12.3	SYT4;basech
18	44104822	C	T	GTTGTCATGG,GGAGCTTGAT	exonic	LOXHD1	nonsynonymous SNV	LOXHD1:ENST00000441551.2:exon28:c.G3971A:p.R1324H,LOXHD1:ENST00000536736.1:exon30:c.G4589A:p.R1530H,LOXHD1:ENST00000300591.6:exon12:c.G1256A:p.F 19H,LOXHD1:ENST00000441893.2:exon11:c.G1388A:p.R463H,LOXHD1:ENST00000398722.4:exon23:c.G3755A:p.R1252H,LOXHD1:ENST00000579038.1:exon10:c.G968A:p.F	18q21.1	.
18	48434487	C	T	AGGACTTCTA,CTCCCAAAAT	exonic	ME2	nonsynonymous SNV	ME2:ENST00000382927.3:exon3:c.C163T:p.P55S,ME2:ENST00000321341.5:exon3:c.C163T:p.P55S	18q21.2	.
18	55103876	C	T	GGTGCTGGCA,CCAGTCGCGA	exonic	ONECUT2	nonsynonymous SNV	ONECUT2:ENST00000491143.2:exon1:c.C928T:p.P310S	18q21.31	.
19	1391126	C	A	AGGTCTACGA,CAGATGCCGG	exonic	NDUFS7	nonsynonymous SNV	NDUFS7:ENST00000233627.9:exon6:c.C417A:p.D139E,NDUFS7:ENST00000546283.1:exon7:c.C417A:p.D139E,NDUFS7:ENST00000414651.2:exon6:c.C507A:p.D169E,ND FS7:ENST00000313408.7:exon6:c.C417A:p.D139E,NDUFS7:ENST00000539480.1:exon6:c.C417A:p.D139E,	19p13.3	.
19	3179679	G	T	GGCCCTGGCC,TCCTCAACTC	exonic	S1PR4	nonsynonymous SNV	S1PR4:ENST00000246115.3:exon1:c.G889T:p.V297F	19p13.3	.
19	4357269	T	A	AGCCTGTTCC,GCGGGGCCTG	exonic	MPND	nonsynonymous SNV	MPND:ENST00000262966.8:exon9:c.T1016A:p.L339Q,MPND:ENST00000596722.1:exon8:c.T1112A:p.L371Q,MPND:ENST00000599840.1:exon9:c.T1016A:p.L339Q,MPND:E ST00000594716.1:exon9:c.T1016A:p.L339Q,MPND:ENST00000359935.4:exon7:c.T866A:p.L289Q,	19p13.3	.
19	4839221	G	A	TTCTCCTCCG,GGCTTTCTCA	exonic	PLIN3	nonsynonymous SNV	PLIN3:ENST00000585479.1:exon8:c.C1285T:p.P429S,PLIN3:ENST00000592528.1:exon8:c.C1252T:p.P418S,PLIN3:ENST00000221957.4:exon8:c.C1288T:p.P430	19p13.3	.
19	6374386	A	G	GACAGCATCA,GGTGGGCAGA	exonic	ALKBH7	nonsynonymous SNV	ALKBH7:ENST00000245812.3:exon2:c.A377G:p.K126R,ALKBH7:ENST00000599849.1:exon2:c.A194G:p.K65R	19p13.3	.
19	6501337	G	A	TGACCGAAGG,GCCAGAACGG	exonic	TUBB4A	nonsynonymous SNV	TUBB4A:ENST00000601640.1:exon4:c.C238T:p.P80S,TUBB4A:ENST00000594290.1:exon3:c.C238T:p.P80S,TUBB4A:ENST00000596291.1:exon2:c.C22T:p.P8S,TUBB4A:E ST00000596926.1:exon3:c.C238T:p.P80S,TUBB4A:ENST00000540257.1:exon3:c.C238T:p.P80S,TUBB4A:ENST00000597686.1:exon4:c.C373T:p.P125S,TUBB4A:ENST00000 264071.2:exon3:c.C238T:p.P80S,TUBB4A:ENST00000598635.1:exon4:c.C391T:p.P131S,TUBB4A:ENST00000595324.1:exon3:c.C238T:p.P80S,	19p13.3	.
19	6697700	G	A	GTAGAGAACG,CTCGGATTTC	exonic	C3	nonsynonymous SNV	C3:ENST00000245907.6:exon20:c.C2546T:p.A849V	19p13.3	.
19	6754134	C	T	CTCCGTGTGG,TCCTTCGGAG	exonic	SH2D3A	nonsynonymous SNV	SH2D3A:ENST00000245908.6:exon8:c.G1313A:p.S438N,SH2D3A:ENST00000437152.3:exon6:c.G1034A:p.S345N	19p13.3	.
19	7083276	G	A	GTGTTTTCGT,AGTTCCGCAC	exonic	ZNF557	nonsynonymous SNV	ZNF557:ENST00000414706.1:exon8:c.G814A:p.E272K,ZNF557:ENST00000252840.6:exon8:c.G814A:p.E272K,ZNF557:ENST00000439035.2:exon8:c.G793A:p.E265	19p13.2	.
19	7585686	C	T	CAGCCCAGAC,CTGATCCTGT	exonic	ZNF358	nonsynonymous SNV	ZNF358:ENST00000394341.2:exon1:c.C1558T:p.P520S,ZNF358:ENST00000597229.1:exon2:c.C1558T:p.P520S	19p13.2	.
19	8651249	A	T	TGGGCCGAGC,CTTGGTCCAG	exonic	ADAMTS10	nonsynonymous SNV	ADAMTS10:ENST00000597188.1:exon21:c.T2509A:p.C837S,ADAMTS10:ENST00000270328.4:exon20:c.T2509A:p.C837S,ADAMTS10:ENST00000595838.1:exon8:c.T970A:	19p13.2	.
19	10083593	A	T	CCCAGGACTC,CCACGCTCCC	splicing	COL5A3(ENST00000264828.3:exon52:c.3774+2T>A)	.	.	19p13.2	.
19	10083630	C	T	CCAGGGGGAC,TTTCTTGCCT	exonic	COL5A3	nonsynonymous SNV	COL5A3:ENST00000264828.3:exon51:c.G3739A:p.G1247S	19p13.2	.
19	10398514	G	A	ACTGATGCTC,GTGAGGCACC	exonic	ICAM4	nonsynonymous SNV	ICAM4:ENST00000393717.2:exon2:c.G697A:p.G233S,ICAM4:ENST00000340992.4:exon2:c.G620A:p.R207H,ICAM4:ENST00000380770.3:exon2:c.G697A:p.A233	19p13.2	.
19	11285273	G	T	ACATGGCCCG,AACGTGACCA	exonic	KANK2	nonsynonymous SNV	KANK2:ENST00000586659.1:exon9:c.C1944A:p.F648L,KANK2:ENST00000355150.5:exon7:c.C1944A:p.F648L,KANK2:ENST00000589359.1:exon9:c.C1968A:p.F656L,KANK ENST00000432929.2:exon9:c.C1968A:p.F656L,KANK2:ENST00000589894.1:exon7:c.C1944A:p.F648L,	19p13.2	.
19	11350611	G	C	GGAGACTCAG,TGGGCACCGT	splicing	C19orf80(ENST00000252453.8:exon1:c.297+1G>C,ENST00000587543.1:exon1:c.38	.	.	19p13.2	.
19	11978756	G	C	CATGAAAGGA,TCACATGGGA	exonic	ZNF439	nonsynonymous SNV	ZNF439:ENST00000304030.2:exon3:c.G872C:p.S291T,ZNF439:ENST00000455282.1:exon3:c.G464C:p.S155T	19p13.2	.
19	12461879	G	A	CCAGTGTGAG,TCTTTCCTGT	exonic	ZNF442	nonsynonymous SNV	ZNF442:ENST00000438182.1:exon3:c.C313T:p.P105S,ZNF442:ENST00000242804.4:exon6:c.C520T:p.P174S,ZNF442:ENST00000424168.1:exon3:c.C313T:p.P105	19p13.2	.
19	13135920	G	A	GCGCGGAAGC,CAAGTACTTC	exonic	NFIX	nonsynonymous SNV	NFIX:ENST00000587760.1:exon2:c.G89A:p.R30H,NFIX:ENST00000397661.2:exon2:c.G113A:p.R38H,NFIX:ENST00000585575.1:exon2:c.G89A:p.R30H,NFIX:ENST0000059 99.1:exon2:c.G113A:p.R38H,NFIX:ENST00000358552.3:exon1:c.G110A:p.R37H,NFIX:ENST00000586797.1:exon2:c.G124A:p.A42T,NFIX:ENST00000360105.4:exon2:c.G122A: p.R41H,NFIX:ENST00000591028.1:exon2:c.G161A:p.R54H,NFIX:ENST00000587260.1:exon1:c.G110A:p.R37H,	19p13.2	.
19	13210484	C	G	CGTTAACGTT,TGCTGCCGCC	exonic	LYL1	nonsynonymous SNV	LYL1:ENST00000264824.4:exon4:c.G492C:p.Q164H	19p13.2	.
19	15220523	C	T	AGGTGCAGCC,CCGCCAGCCC	exonic	SYDE1	nonsynonymous SNV	SYDE1:ENST00000342784.2:exon3:c.C439T:p.P147S,SYDE1:ENST00000600440.1:exon3:c.C238T:p.P80S	19p13.12	.
19	19338729	C	T	GCAGAAAGCC,CACTTCTGGC	exonic	NCAN	nonsynonymous SNV	NCAN:ENST00000252575.6:exon8:c.C2300T:p.P767L,NCAN:ENST00000538881.1:exon2:c.C653T:p.P218L	19p13.11	.
19	19375495	G	A	CTTGTGGAGG,CTAGGGGGTC	exonic	TM6SF2	nonsynonymous SNV	TM6SF2:ENST00000389363.4:exon10:c.C1112T:p.A371V	19p13.11	.
19	19375504	T	A	GGCTAGGGGG,CGGAGGGTGG	exonic	TM6SF2	nonsynonymous SNV	TM6SF2:ENST00000389363.4:exon10:c.A1103T:p.D368V	19p13.11	.
19	19747558	C	T	GACTCGCTGC,GCCACCCACG	exonic	GMIP	nonsynonymous SNV	GMIP:ENST00000587238.1:exon13:c.G1285A:p.G429S,GMIP:ENST00000203556.4:exon13:c.G1285A:p.G429S,GMIP:ENST00000445806.2:exon13:c.G1276A:p.G426	19p13.11	.
19	19790712	G	C	AGAACTCATA,TGGAGGAAAA	exonic	ZNF101	nonsynonymous SNV	ZNF101:ENST00000318110.5:exon4:c.G914C:p.S305T,ZNF101:ENST00000415784.2:exon5:c.G554C:p.S185T,ZNF101:ENST00000592502.1:exon4:c.G914C:p.S305	19p13.11	.
19	36386974	C	G	GGACAGAGGT,GGAAGGGCGC	exonic	NFKBID	nonsynonymous SNV	NFKBID:ENST00000352614.2:exon9:c.G970C:p.D324H,NFKBID:ENST00000396901.1:exon9:c.G514C:p.D172H,NFKBID:ENST00000606253.1:exon9:c.G514C:p.D172H	19q13.12	NFKBID;base
19	37310014	C	T	TCGCTGATGT,GAGCAAGGTG	exonic	ZNF790	nonsynonymous SNV	ZNF790:ENST00000356725.4:exon5:c.G1232A:p.R411Q	19q13.12	.
19	38189887	C	T	ACCCTGATGT,GAGTAAGTCG	exonic	ZNF607	nonsynonymous SNV	ZNF607:ENST00000395835.3:exon5:c.G1142A:p.R381Q,ZNF607:ENST00000355202.4:exon5:c.G1145A:p.R382Q	19q13.12	.
19	38384991	G	C	ATTTGGTGAG,TGCCTAAGAG	exonic	WDR87	nonsynonymous SNV	WDR87:ENST00000447313.2:exon4:c.C1352G:p.T451S,WDR87:ENST00000303868.5:exon4:c.C1235G:p.T412S	19q13.13	.
19	38633340	T	C	ATACAAGCCA,CCCCAGAAAG	exonic	SIPA1L3	nonsynonymous SNV	SIPA1L3:ENST00000222345.6:exon12:c.T3523C:p.S1175P	19q13.13	.
19	38895719	C	A	CCAGGGACCC,CGCCAGCCAG	exonic	FAM98C	nonsynonymous SNV	FAM98C:ENST00000343358.7:exon4:c.C521A:p.P174H,FAM98C:ENST00000252530.5:exon4:c.C521A:p.P174H	19q13.2	.
19	38939379	G	T	CGTGCAGCAT,TGGCCTCAGG	exonic	RYR1	nonsynonymous SNV	RYR1:ENST00000355481.4:exon11:c.G1048T:p.V350L,RYR1:ENST00000359596.3:exon11:c.G1048T:p.V350L,RYR1:ENST00000360985.3:exon11:c.G1048T:p.V350	19q13.2	.
19	39380781	C	T	GATGACTCTG,GACCTGGAGG	exonic	SIRT2	nonsynonymous SNV	SIRT2:ENST00000414941.1:exon6:c.G119A:p.R40H,SIRT2:ENST00000437828.1:exon4:c.G119A:p.R40H,SIRT2:ENST00000381766.4:exon3:c.G119A:p.R40H,SIRT2:ENST0 000420440.1:exon4:c.G119A:p.R40H,SIRT2:ENST00000358931.5:exon5:c.G230A:p.R77H,SIRT2:ENST00000447739.1:exon4:c.G119A:p.R40H,SIRT2:ENST00000392081.2:ex on4:c.G119A:p.R40H,SIRT2:ENST00000249396.7:exon5:c.G230A:p.R77H,SIRT2:ENST00000407552.1:exon5:c.G119A:p.R40H,	19q13.2	.
19	40519623	G	A	AAGAATGTTT,CAAAATCTAT	exonic	ZNF546	nonsynonymous SNV	ZNF546:ENST00000347077.4:exon7:c.G446A:p.C149Y,ZNF546:ENST00000599504.1:exon6:c.G332A:p.C111Y,ZNF546:ENST00000600094.1:exon7:c.G368A:p.C123	19q13.2	.
19	40904586	G	A	GTCCCGGGCC,CAGAGCCAGG	exonic	PRX	nonsynonymous SNV	PRX:ENST00000324001.7:exon6:c.C322T:p.R108W,PRX:ENST00000291825.7:exon6:c.C322T:p.R108W	19q13.2	.
19	42507533	G	A	TTCCATGACC,CCACGAAGAC	exonic	GRIK5	nonsynonymous SNV	GRIK5:ENST00000593562.1:exon19:c.C2465T:p.A822V,GRIK5:ENST00000301218.4:exon18:c.C2465T:p.A822V,GRIK5:ENST00000262895.3:exon18:c.C2465T:p.A822	19q13.2	.
19	44605038	C	T	GAAGCTGTAT,GAGATGTGAT	exonic	ZNF224	stopgain	ZNF224:ENST00000336976.6:exon4:c.C100T:p.R34X,ZNF224:ENST00000589680.1:exon4:c.C100T:p.R34X,ZNF224:ENST00000591511.1:exon2:c.C100T:p.R34X,ZNF224:E ST00000589870.1:exon3:c.C100T:p.R34X,	19q13.31	.
19	44635453	A	T	CACACTGGAG,GAAACCATTC	exonic	ZNF225	nonsynonymous SNV	ZNF225:ENST00000262894.6:exon5:c.A686T:p.E229V,ZNF225:ENST00000590612.1:exon4:c.A686T:p.E229V	19q13.31	.
19	45871997	A	C	AAGCTCTTCA,TCACCTACTC	exonic	ERCC2	nonsynonymous SNV	ERCC2:ENST00000586856.1:exon3:c.T101G:p.I34S,ERCC2:ENST00000591309.1:exon5:c.T251G:p.I84S,ERCC2:ENST00000485403.2:exon4:c.T179G:p.I60S,ERCC2:ENST 0000391944.3:exon5:c.T251G:p.I84S,ERCC2:ENST00000221481.6:exon5:c.T251G:p.I84S,ERCC2:ENST00000391941.2:exon4:c.T179G:p.I60S,ERCC2:ENST00000391940.4:e xon5:c.T179G:p.I60S,ERCC2:ENST00000391945.4:exon5:c.T251G:p.I84S,ERCC2:ENST00000586131.1:exon5:c.T179G:p.I60S,	19q13.32	.
19	46271817	C	G	AGCAGCGCCT,GCAGACGCAC	exonic	SIX5	nonsynonymous SNV	SIX5:ENST00000317578.6:exon1:c.G286C:p.E96Q	19q13.32	SIX5;basecha
19	47282134	C	A	ATCTTGCCAG,CACCAGGAAC	exonic	SLC1A5	nonsynonymous SNV	SLC1A5:ENST00000412532.2:exon5:c.G172T:p.A58S,SLC1A5:ENST00000434726.2:exon4:c.G250T:p.A84S,SLC1A5:ENST00000594991.1:exon4:c.G328T:p.A110S,SLC1A5 NST00000542575.2:exon5:c.G856T:p.A286S,	19q13.32	.
19	48244472	C	A	GGCTCCAAGG,CAAGACCTGG	exonic	EHD2	nonsynonymous SNV	EHD2:ENST00000263277.3:exon6:c.C1415A:p.A472D,EHD2:ENST00000538399.1:exon5:c.C1007A:p.A336D	19q13.33	.
19	49131486	C	T	GGCATCCTCC,CTGCGGCTCG	exonic	SPHK2	nonsynonymous SNV	SPHK2:ENST00000599748.1:exon5:c.C716T:p.P239L,SPHK2:ENST00000245222.4:exon6:c.C824T:p.P275L,SPHK2:ENST00000599029.1:exon4:c.C716T:p.P239L,SPHK2:E ST00000600537.1:exon6:c.C647T:p.P216L,SPHK2:ENST00000443164.1:exon4:c.C1010T:p.P337L,SPHK2:ENST00000340932.3:exon6:c.C716T:p.P239L,SPHK2:ENST000005 98088.1:exon6:c.C824T:p.P275L,SPHK2:ENST00000601712.1:exon6:c.C716T:p.P239L,SPHK2:ENST00000597434.1:exon5:c.C716T:p.P239L,	19q13.33	.
19	49692310	G	C	GGCCATGCAA,CTGACGCCCG	exonic	TRPM4	nonsynonymous SNV	TRPM4:ENST00000427978.2:exon14:c.G1981C:p.A661P,TRPM4:ENST00000252826.5:exon14:c.G1981C:p.A661P,TRPM4:ENST00000355712.5:exon9:c.G919C:p.A307	19q13.33	.
19	51170661	G	A	GCTGGGCGGC,GGACCCCCGG	exonic	SHANK1	nonsynonymous SNV	SHANK1:ENST00000293441.1:exon22:c.C4556T:p.P1519L,SHANK1:ENST00000391814.1:exon24:c.C4580T:p.P1527L,SHANK1:ENST00000359082.3:exon21:c.C4529T:p.P 10L,SHANK1:ENST00000391813.1:exon8:c.C2717T:p.P906L,	19q13.33	.
19	51645914	C	A	ACCGATTCCA,CTCCTTGGGG	exonic	SIGLEC7	nonsynonymous SNV	SIGLEC7:ENST00000600577.1:exon1:c.C288A:p.H96Q,SIGLEC7:ENST00000305628.7:exon1:c.C288A:p.H96Q,SIGLEC7:ENST00000536156.1:exon1:c.C288A:p.H96Q,SIGL C7:ENST00000601682.1:exon1:c.C288A:p.H96Q,SIGLEC7:ENST00000317643.6:exon1:c.C288A:p.H96Q,	19q13.41	.
19	51841341	C	A	CCAGGGGCCT,CCTTCCCGGG	exonic	VSIG10L	nonsynonymous SNV	VSIG10L:ENST00000600663.1:exon3:c.G380T:p.G127V	19q13.41	.
19	51958800	G	A	CCCAGGGTTT,AGGACCGTGA	exonic	SIGLEC8	nonsynonymous SNV	SIGLEC8:ENST00000321424.3:exon4:c.C923T:p.S308L,SIGLEC8:ENST00000430817.1:exon2:c.C596T:p.S199L,SIGLEC8:ENST00000340550.5:exon3:c.C644T:p.S215	19q13.41	.
19	52091635	C	A	AACAACAGGT,AAACTTCAAT	exonic	ZNF175	stopgain	ZNF175:ENST00000262259.2:exon5:c.C2051A:p.S684X	19q13.41	.
19	54697104	C	A	ACTCCAAGAC,TGGTTGCTGC	exonic	TSEN34	nonsynonymous SNV	TSEN34:ENST00000429671.2:exon5:c.C820A:p.L274M,TSEN34:ENST00000302937.4:exon5:c.C820A:p.L274M,TSEN34:ENST00000396383.1:exon5:c.C820A:p.L274M,TSEN 4:ENST00000396388.2:exon4:c.C820A:p.L274M,	19q13.42	.
19	55798624	C	G	CGAACACCCA,ATGTCCTCAA	exonic	BRSK1	nonsynonymous SNV	BRSK1:ENST00000585418.1:exon3:c.C274G:p.H92D,BRSK1:ENST00000590333.1:exon5:c.C322G:p.H108D,BRSK1:ENST00000309383.1:exon3:c.C274G:p.H92D	19q13.42	.
19	55816218	G	A	GAGCCGCCTG,AGGAGTCGTC	exonic	BRSK1	stopgain	BRSK1:ENST00000590333.1:exon16:c.G1695A:p.W565X,BRSK1:ENST00000309383.1:exon14:c.G1647A:p.W549X,BRSK1:ENST00000326848.7:exon6:c.G732A:p.W244X	19q13.42	.
19	56369189	G	A	TCCCAAGGAA,CTGGGAAACA	exonic	NLRP4	nonsynonymous SNV	NLRP4:ENST00000587891.1:exon1:c.G205A:p.A69T,NLRP4:ENST00000346986.5:exon3:c.G430A:p.A144T,NLRP4:ENST00000301295.6:exon3:c.G430A:p.A144T,NLRP4:EN T00000587464.1:exon3:c.G430A:p.A144T,	19q13.43	.
19	57955602	G	T	GTGGGAAATT,TTTATGGACA	exonic	ZNF749	nonsynonymous SNV	ZNF749:ENST00000334181.4:exon3:c.G1086T:p.L362F	19q13.43	.
20	368931	G	A	TACAGGCACT,AGTATACCTG	exonic	TRIB3	nonsynonymous SNV	TRIB3:ENST00000449710.1:exon2:c.G277A:p.E93K,TRIB3:ENST00000422053.2:exon3:c.G358A:p.E120K,TRIB3:ENST00000217233.3:exon2:c.G277A:p.E93	20p13	.
20	1433207	C	T	GTCCTCGTCC,GATGGTCCTC	exonic	NSFL1C	nonsynonymous SNV	NSFL1C:ENST00000216879.4:exon7:c.G716A:p.R239Q,NSFL1C:ENST00000476071.1:exon8:c.G722A:p.R241Q,NSFL1C:ENST00000350991.4:exon8:c.G722A:p.R241Q,NSF 1C:ENST00000381658.4:exon6:c.G383A:p.R128Q,NSFL1C:ENST00000381653.5:exon7:c.G716A:p.R239Q,NSFL1C:ENST00000353088.2:exon6:c.G623A:p.R208Q,	20p13	NSFL1C;base
20	2796254	C	T	CGGTGAAGGT,CTGGACAGCA	exonic	C20orf141	nonsynonymous SNV	C20orf141:ENST00000603872.1:exon3:c.C331T:p.P111S,C20orf141:ENST00000380589.4:exon2:c.C331T:p.P111S	20p13	.
20	2818908	C	T	GGCAGCTCCA,GCCCCAGGCG	exonic	PCED1A	nonsynonymous SNV	PCED1A:ENST00000360652.2:exon6:c.G811A:p.V271M,PCED1A:ENST00000439542.1:exon6:c.G811A:p.V271M,PCED1A:ENST00000448755.1:exon6:c.G658A:p.V220M,PC D1A:ENST00000356872.3:exon6:c.G658A:p.V220M,	20p13	.
20	3063296	C	T	TCGGGCTGGG,GGGCTCGAAG	exonic	AVP	nonsynonymous SNV	AVP:ENST00000380293.3:exon3:c.G475A:p.A159T	20p13	.
20	17601390	C	A	CGGCGCCCAC,TTGGCCCTCC	exonic	RRBP1	nonsynonymous SNV	RRBP1:ENST00000377813.1:exon17:c.G3477T:p.K1159N,RRBP1:ENST00000360807.4:exon17:c.G2178T:p.K726N,RRBP1:ENST00000455029.2:exon16:c.G1500T:p.K500N,R RBP1:ENST00000377807.2:exon18:c.G2178T:p.K726N,RRBP1:ENST00000246043.4:exon15:c.G3477T:p.K1159N,	20p12.1	.
20	25271193	C	T	AATCATGACC,AGTTGTGGGT	exonic	PYGB	nonsynonymous SNV	PYGB:ENST00000216962.4:exon16:c.C1904T:p.P635L	20p11.21	.
20	30816208	C	T	GATTCATGCC,ACCTTGTCCG	exonic	POFUT1	nonsynonymous SNV	POFUT1:ENST00000539210.1:exon2:c.C52T:p.H18Y,POFUT1:ENST00000375749.3:exon5:c.C685T:p.H229Y	20q11.21	.
20	33855219	T	A	TGTTTGTCTT,AAGGTAGGGC	exonic	MMP24	nonsynonymous SNV	MMP24:ENST00000246186.6:exon6:c.T1191A:p.F397L	20q11.22	.
20	33874441	A	G	CTCATCCCTG,CCAGGTCACT	exonic	FAM83C	nonsynonymous SNV	FAM83C:ENST00000374408.3:exon4:c.T2141C:p.V714A	20q11.22	.
20	36640181	G	A	CCACAAGCAC,GCAAACGTCC	exonic	TTI1	nonsynonymous SNV	TTI1:ENST00000373447.3:exon2:c.C2038T:p.R680C,TTI1:ENST00000373448.2:exon3:c.C2038T:p.R680C,TTI1:ENST00000449821.1:exon1:c.C2038T:p.R680C	20q11.23	.
20	36770543	G	C	TGCTGTTCTG,TCATGGGCCG	exonic	TGM2	nonsynonymous SNV	TGM2:ENST00000536701.1:exon6:c.C675G:p.D225E,TGM2:ENST00000361475.2:exon7:c.C918G:p.D306E,TGM2:ENST00000536724.1:exon6:c.C738G:p.D246E	20q11.23	.
20	39791154	G	C	CCCAACATGC,CTTCCTCCGA	exonic	PLCG1	nonsynonymous SNV	PLCG1:ENST00000373271.1:exon5:c.G575C:p.R192P,PLCG1:ENST00000244007.3:exon6:c.G575C:p.R192P,PLCG1:ENST00000373272.2:exon5:c.G575C:p.R192	20q12	.
20	49211992	T	A	TGCCACTTCT,CCAGGAGGCA	exonic	FAM65C	nonsynonymous SNV	FAM65C:ENST00000327979.2:exon16:c.A1952T:p.E651V,FAM65C:ENST00000535356.1:exon16:c.A1964T:p.E655V,FAM65C:ENST00000045083.2:exon16:c.A1952T:p.E651	20q13.13	.
20	52193128	T	A	GGTGCATCAT,AAAACTTCTG	exonic	ZNF217	nonsynonymous SNV	ZNF217:ENST00000302342.3:exon3:c.A2175T:p.L725F,ZNF217:ENST00000371471.2:exon4:c.A2175T:p.L725I	20q13.2	.
20	56186930	C	T	ACTAGGTCCC,CCAAGTTGAG	exonic	ZBP1	nonsynonymous SNV	ZBP1:ENST00000395822.3:exon5:c.G502A:p.G168R,ZBP1:ENST00000340462.4:exon5:c.G658A:p.G220R,ZBP1:ENST00000371173.3:exon6:c.G727A:p.G243R,ZBP1:ENST(000343535.4:exon6:c.G727A:p.G243R,	20q13.31	.
20	60427882	G	A	CTACGTCATC,ACATGAATGA	exonic	CDH4	nonsynonymous SNV	CDH4:ENST00000543233.1:exon5:c.G583A:p.D195N,CDH4:ENST00000360469.5:exon6:c.G805A:p.D269N	20q13.33	.
20	60740545	G	T	CCTTCGAGGA,TCCACGCAGC	exonic	SS18L1	nonsynonymous SNV	SS18L1:ENST00000421564.1:exon8:c.G891T:p.E297D,SS18L1:ENST00000370848.4:exon7:c.G900T:p.E300D,SS18L1:ENST00000331758.3:exon8:c.G891T:p.E297	20q13.33	.
20	62626746	C	T	TGGAATGACG,CAGGACTGAT	exonic	PRPF6	nonsynonymous SNV	PRPF6:ENST00000266079.4:exon6:c.C682T:p.P228S,PRPF6:ENST00000535781.1:exon6:c.C682T:p.P228S	20q13.33	.
21	19716290	G	A	AAGGCAAGCT,GCTCCAAAGT	exonic	TMPRSS15	nonsynonymous SNV	TMPRSS15:ENST00000284885.3:exon11:c.C1259T:p.P420L	21q21.1	.
21	34926498	A	G	AGTGAAGCTG,CATTGAAGGG	exonic	SON	nonsynonymous SNV	SON:ENST00000356577.4:exon3:c.A4961G:p.D1654G,SON:ENST00000290239.6:exon3:c.A4961G:p.D1654G,SON:ENST00000300278.4:exon3:c.A4961G:p.D1654G,SON:E ST00000455528.1:exon3:c.A4961G:p.D1654G,SON:ENST00000381679.4:exon3:c.A4961G:p.D1654G,	21q22.11	.
21	43771013	A	C	CCGCCAGGGC,CATAGCCCCA	exonic	TFF2	nonsynonymous SNV	TFF2:ENST00000291526.4:exon1:c.T54G:p.C18W	21q22.3	.
21	45168897	C	T	GGACCTCCTT,CCGTCTACAA	exonic	PDXK	nonsynonymous SNV	PDXK:ENST00000468090.1:exon5:c.C316T:p.P106S,PDXK:ENST00000291565.4:exon6:c.C400T:p.P134S,PDXK:ENST00000467908.1:exon5:c.C280T:p.P94	21q22.3	.
21	45655358	G	A	GTTGGGCCTG,GGTAGCCGTT	exonic	ICOSLG	nonsynonymous SNV	ICOSLG:ENST00000344330.4:exon4:c.C494T:p.P165L,ICOSLG:ENST00000400377.3:exon3:c.C143T:p.P48L,ICOSLG:ENST00000400379.3:exon4:c.C494T:p.P165L,ICOSL(ENST00000407780.3:exon4:c.C494T:p.P165L,	21q22.3	.
21	45953713	G	A	TCCTCGCGAA,GAACAGGAAG	exonic	TSPEAR	nonsynonymous SNV	TSPEAR:ENST00000397916.1:exon3:c.C193T:p.L65F,TSPEAR:ENST00000323084.4:exon3:c.C397T:p.L133F	21q22.3	.
21	47986563	C	T	ATTGAGACCT,TGTCATCCGA	exonic	DIP2A	nonsynonymous SNV	DIP2A:ENST00000400274.1:exon37:c.C4418T:p.S1473F,DIP2A:ENST00000417564.2:exon37:c.C4430T:p.S1477F,DIP2A:ENST00000318711.7:exon37:c.C4433T:p.S1478	21q22.3	.
22	18028592	T	G	ACTATGCCTG,CCACAGAGCT	exonic	CECR2	nonsynonymous SNV	CECR2:ENST00000400573.5:exon17:c.T3549G:p.C1183W,CECR2:ENST00000400585.2:exon17:c.T3123G:p.C1041W,CECR2:ENST00000262608.8:exon16:c.T3552G:p.C11	22q11.21	.
22	18387597	G	C	CAGCCCCAAT,ATGAGACACT	exonic	MICAL3	nonsynonymous SNV	MICAL3:ENST00000444520.1:exon3:c.C273G:p.I91M,MICAL3:ENST00000424046.1:exon3:c.C273G:p.I91M,MICAL3:ENST00000207726.7:exon3:c.C273G:p.I91M,MICAL3:EN T00000441493.2:exon3:c.C273G:p.I91M,MICAL3:ENST00000495076.1:exon3:c.C273G:p.I91M,MICAL3:ENST00000585038.1:exon2:c.C273G:p.I91M,MICAL3:ENST0000041472 5.2:exon2:c.C273G:p.I91M,MICAL3:ENST00000429452.1:exon3:c.C273G:p.I91M,MICAL3:ENST00000400561.2:exon2:c.C273G:p.I91M,MICAL3:ENST00000383094.3:exon2:c.C	22q11.21	.
22	18566433	C	T	GCCATTCACA,AGCGAGGCAG	exonic	PEX26	nonsynonymous SNV	PEX26:ENST00000399744.3:exon3:c.C602T:p.T201I,PEX26:ENST00000329627.7:exon4:c.C602T:p.T201I,PEX26:ENST00000428061.2:exon3:c.C602T:p.T20	22q11.21	.
22	20082303	C	G	AGCCCAAAGA,AGTGAAGAAC	exonic	DGCR8	nonsynonymous SNV	DGCR8:ENST00000383024.2:exon8:c.C1674G:p.D558E,DGCR8:ENST00000351989.3:exon9:c.C1773G:p.D591E,DGCR8:ENST00000407755.1:exon7:c.C1674G:p.D558	22q11.21	.
22	20937239	C	T	CTCACCTGGA,CTTTAAACAC	exonic	MED15	nonsynonymous SNV	MED15:ENST00000292733.7:exon10:c.C1402T:p.P468S,MED15:ENST00000263205.7:exon11:c.C1522T:p.P508S,MED15:ENST00000425759.2:exon9:c.C1069T:p.P357S,ME 15:ENST00000406969.1:exon10:c.C1324T:p.P442S,MED15:ENST00000541476.1:exon10:c.C1324T:p.P442S,MED15:ENST00000382974.2:exon9:c.C1189T:p.P397S,	22q11.21	.
22	24122797	G	C	CCTGCCGTTT,ATGGGCCTGG	exonic	MMP11	nonsynonymous SNV	MMP11:ENST00000437086.1:exon4:c.G463C:p.D155H,MMP11:ENST00000215743.3:exon4:c.G511C:p.D171H	22q11.23	.
22	26100170	G	A	GGCTGTCACG,AGGCGGGTAG	exonic	ADRBK2	nonsynonymous SNV	ADRBK2:ENST00000324198.6:exon15:c.G1322A:p.G441E	22q12.1	.
22	30690009	C	T	AGGAGCGGGT,GATCTTCTGA	exonic	RP1-130H16.18,TBC1D10A	nonsynonymous SNV	RP1-130H16.18:ENST00000330168.5:exon4:c.G379A:p.D127N,RP1 130H16.18:ENST00000434291.1:exon5:c.G418A:p.D140N,TBC1D10A:ENST00000215790.7:exon7:c.G796A:p.D266N,TBC1D10A:ENST00000403477.3:exon7:c.G817A:p.D273 N,TBC1D10A:ENST00000403362.1:exon7:c.G532A:p.D178N,TBC1D10A:ENST00000433426.1:exon7:c.G595A:p.D199N,RP1- 130H16.18:ENST00000447976.1:exon5:c.G418A:p.D140N,RP1-130H16.18:ENST00000434987.1:exon5:c.G412A:p.D138N,RP1-	22q12.2	.
22	30741029	G	C	AGCTGGGTCA,AAACTGGCGC	exonic	SF3A1	nonsynonymous SNV	SF3A1:ENST00000439242.1:exon4:c.C349G:p.L117V,SF3A1:ENST00000215793.8:exon4:c.C544G:p.L182V	22q12.2	.
22	31840618	C	T	GCAGCAGGGG,AGGGGAAGAG	exonic	EIF4ENIF1	nonsynonymous SNV	EIF4ENIF1:ENST00000344710.5:exon13:c.G1525A:p.A509T,EIF4ENIF1:ENST00000330125.5:exon15:c.G2047A:p.A683T,EIF4ENIF1:ENST00000397523.1:exon13:c.G1975A: A659T,EIF4ENIF1:ENST00000382180.2:exon7:c.G1012A:p.A338T,EIF4ENIF1:ENST00000397525.1:exon15:c.G2047A:p.A683T,	22q12.2	.
22	37469575	C	T	GCCCTACCTT,CTGGCACTGC	exonic	TMPRSS6	nonsynonymous SNV	TMPRSS6:ENST00000406856.1:exon13:c.G1552A:p.E518K,TMPRSS6:ENST00000381792.2:exon13:c.G1552A:p.E518K,TMPRSS6:ENST00000346753.3:exon13:c.G1579A: E527K,TMPRSS6:ENST00000406725.1:exon13:c.G1552A:p.E518K,	22q12.3	.
22	40802646	A	T	GGGGGCCGGC,CCCTCACCAA	exonic	SGSM3	nonsynonymous SNV	SGSM3:ENST00000248929.9:exon10:c.A1165T:p.T389S,SGSM3:ENST00000454798.2:exon9:c.A964T:p.T322S	22q13.1	.
22	42607774	C	T	TTCTGGGAGC,ATGCTTTAAT	exonic	TCF20	nonsynonymous SNV	TCF20:ENST00000335626.4:exon1:c.G3538A:p.G1180S,TCF20:ENST00000359486.3:exon1:c.G3538A:p.G1180S	22q13.2	.
22	42609941	A	T	TACTCAGAGC,CTCAAACTAC	exonic	TCF20	nonsynonymous SNV	TCF20:ENST00000335626.4:exon1:c.T1371A:p.S457R,TCF20:ENST00000359486.3:exon1:c.T1371A:p.S457R	22q13.2	.
22	43568412	T	G	CACTTACCGG,TGGAGAACCG	exonic	TTLL12	nonsynonymous SNV	TTLL12:ENST00000216129.6:exon10:c.A1490C:p.N497T	22q13.2	.
22	50537902	G	A	GTGGGAAGAC,ACAGCAGAAA	exonic	MOV10L1	nonsynonymous SNV	MOV10L1:ENST00000262794.5:exon3:c.G313A:p.D105N,MOV10L1:ENST00000545383.1:exon4:c.G313A:p.D105N,MOV10L1:ENST00000540615.1:exon3:c.G253A:p.D85N OV10L1:ENST00000395858.3:exon3:c.G313A:p.D105N,	22q13.33	.
22	51042055	G	A	TCCACCCCAG,ACTCCCTAAA	exonic	MAPK8IP2	nonsynonymous SNV	MAPK8IP2:ENST00000329492.3:exon4:c.G448A:p.D150N,MAPK8IP2:ENST00000442429.2:exon4:c.G448A:p.D150N	22q13.33	.
X	591870	G	A	CAATGACAAG,AGAAACTGAA	exonic	SHOX	nonsynonymous SNV	SHOX:ENST00000381575.1:exon1:c.G238A:p.E80K,SHOX:ENST00000381578.1:exon2:c.G238A:p.E80K,SHOX:ENST00000554971.1:exon1:c.G238A:p.E80K,SHOX:ENST0(00334060.3:exon2:c.G238A:p.E80K,	Xp22.33	.
X	14927001	A	T	AAATTGTGAA,ACCTGGCTTG	exonic	MOSPD2	nonsynonymous SNV	MOSPD2:ENST00000482354.1:exon8:c.A597T:p.K199N,MOSPD2:ENST00000380492.3:exon8:c.A597T:p.K199N	Xp22.2	.
X	30236800	G	A	TGAAGCAGAG,AAGAAGAGGC	exonic	MAGEB2	nonsynonymous SNV	MAGEB2:ENST00000378988.4:exon2:c.G103A:p.E35K	Xp21.2	.
X	30260859	C	G	GCTTCTGATG,CTCTACTGAG	exonic	MAGEB4	nonsynonymous SNV	MAGEB4:ENST00000378982.2:exon1:c.C607G:p.P203A	Xp21.2	.
X	39932692	C	A	AAATATAGAG,TTGGTGGAAG	exonic	BCOR	nonsynonymous SNV	BCOR:ENST00000406200.2:exon4:c.G1907T:p.S636I,BCOR:ENST00000378444.4:exon4:c.G1907T:p.S636I,BCOR:ENST00000342274.4:exon4:c.G1907T:p.S636I,BCOR:EN T00000397354.3:exon4:c.G1907T:p.S636I,BCOR:ENST00000378455.4:exon4:c.G1907T:p.S636I,	Xp11.4	.
X	44037677	T	C	ATATTTTTCT,GAACTTTTCG	exonic	EFHC2	nonsynonymous SNV	EFHC2:ENST00000420999.1:exon12:c.A1885G:p.K629E	Xp11.3	.
X	47308477	G	T	CTCATTCTTA,TAGAGGAAGG	exonic	ZNF41	nonsynonymous SNV	ZNF41:ENST00000377065.4:exon5:c.C692A:p.T231N,ZNF41:ENST00000313116.7:exon5:c.C692A:p.T231N,ZNF41:ENST00000397050.2:exon5:c.C722A:p.T241	Xp11.23	.
X	53642771	A	C	CATCGACAGC,CTCCCCAGGT	exonic	HUWE1	nonsynonymous SNV	HUWE1:ENST00000342160.3:exon21:c.T1983G:p.S661R,HUWE1:ENST00000218328.8:exon22:c.T1983G:p.S661R,HUWE1:ENST00000262854.6:exon22:c.T1983G:p.S661F	Xp11.22	.
X	67937760	G	A	CCAGGTAGGC,GTGGGGCTGT	exonic	STARD8	nonsynonymous SNV	STARD8:ENST00000374597.3:exon5:c.G764A:p.R255Q,STARD8:ENST00000374599.3:exon6:c.G1004A:p.R335Q,STARD8:ENST00000252336.6:exon5:c.G764A:p.R255(	Xq13.1	.
X	70468020	C	T	ACCTTCGCTG,AGAAGCTTTT	exonic	ZMYM3	nonsynonymous SNV	ZMYM3:ENST00000373998.1:exon11:c.G1967A:p.C656Y,ZMYM3:ENST00000373984.3:exon11:c.G1973A:p.C658Y,ZMYM3:ENST00000353904.2:exon11:c.G1967A:p.C656Y MYM3:ENST00000314425.5:exon11:c.G1967A:p.C656Y,ZMYM3:ENST00000373988.1:exon11:c.G1973A:p.C658Y,	Xq13.1	.
X	107417689	G	T	TGGCATTCCT,GGAAACCTGT	exonic	COL4A6	nonsynonymous SNV	COL4A6:ENST00000334504.7:exon31:c.C3119A:p.P1040Q,COL4A6:ENST00000538570.1:exon31:c.C3119A:p.P1040Q,COL4A6:ENST00000545689.1:exon31:c.C3119A:p.P 40Q,COL4A6:ENST00000372216.4:exon31:c.C3122A:p.P1041Q,COL4A6:ENST00000394872.2:exon31:c.C3122A:p.P1041Q,	Xq22.3	.
X	122387313	C	A	CAGATGCGCC,AGCCTTGAAG	exonic	GRIA3	nonsynonymous SNV	GRIA3:ENST00000542149.1:exon3:c.C428A:p.P143Q,GRIA3:ENST00000541091.1:exon3:c.C380A:p.P127Q,GRIA3:ENST00000371256.5:exon3:c.C428A:p.P143Q,GRIA3:EN T00000371251.1:exon3:c.C428A:p.P143Q,GRIA3:ENST00000264357.5:exon3:c.C428A:p.P143Q,	Xq25	.
Y	6954421	G	T	CACCGGGCCT,CCACCAGCAA	exonic	TBL1Y	nonsynonymous SNV	TBL1Y:ENST00000383032.1:exon16:c.G1243T:p.A415S,TBL1Y:ENST00000346432.3:exon15:c.G1243T:p.A415S,TBL1Y:ENST00000355162.2:exon15:c.G1243T:p.A415	Yp11.2	.

C000-ESMKCH

Chromosome	Position (hg1	Reference all	Mutant allele	Sequence context	ANNOVAR fu	Gene	Mutation type	ANNOVAR output (alternative transcripts)	Cytoband	COSMIC
1	880503	G	A	ACCCCATGCC,AGTGCTCAGG	exonic	NOC2L	nonsynonymous SNV	NOC2L:ENST00000327044.6:exon18:c.C2077T:p.R693W,	[illegible]	
1	1022925	C	T	GGGTTCCGTT,CCACAGCGGA	exonic	C1orf159	nonsynonymous SNV	C1orf159:ENST00000467751.1:exon6:c.G298A:p.E100K,	[illegible]	
1	1271752	G	A	TGGCCGGCCG,ACGCTCTCGC	exonic	DVL1	nonsynonymous SNV	DVL1:ENST00000378891.5:exon15:c.C1783T:p.P595S,DVL1:ENST00000378888.5:exon15:c.C1858T:p.P620S, [illegible]	[illegible]	
1	8420193	C	A	GCTGGCGTGA,TGGGGGTGTC	exonic	RERE	nonsynonymous SNV	RERE:ENST00000337907.3:exon19:c.G3374T:p.S1125I,RERE:ENST00000377464.1:exon12:c.G2570T:p.S857I,R [illegible]	[illegible]	
1	8421531	G	A	TTCAGATGGC,AGTTGGGCCT	exonic	RERE	nonsynonymous SNV	RERE:ENST00000337907.3:exon19:c.C2036T:p.S679L,RERE:ENST00000377464.1:exon12:c.C1232T:p.S411L,R [illegible]	[illegible]	
1	16260975	C	T	GGTGCGGTTA,TGCTGCATCT	exonic	SPEN	nonsynonymous SNV	SPEN:ENST00000375759.3:exon11:c.C8240T:p.T2747I,	[illegible]	
1	17740179	C	T	ACCAAAGCCC,AGGAGAAGAC	exonic	RCC2	stopgain	RCC2:ENST00000375433.3:exon8:c.G1061A:p.W354X,RCC2:ENST00000375436.4:exon9:c.G1061A:p.W354X,	[illegible]	
1	18807815	G	A	CACTTCCACA,ACAGGAAGCC	exonic	KLHDC7A	nonsynonymous SNV	KLHDC7A:ENST00000400664.1:exon1:c.G340A:p.D114N,	[illegible]	
1	20998685	C	T	CTCTGCTGCC,GAAGCTGAAA	exonic	KIF17	nonsynonymous SNV	KIF17:ENST00000400463.3:exon12:c.G2468A:p.R823Q,KIF17:ENST00000247986.2:exon12:c.G2468A:p.R823Q, [illegible]	[illegible]	
1	21151665	C	A	CAGCCTCCCT,CATAAGGCTC	exonic	EIF4G3	nonsynonymous SNV	EIF4G3:ENST00000264211.8:exon27:c.G4128T:p.W1376C,EIF4G3:ENST00000374935.3:exon26:c.G3288T:p.W1 [illegible]	[illegible]	
1	21563319	C	T	TTCTTAATCT,CAGGATGATC	exonic	ECE1	nonsynonymous SNV	ECE1:ENST00000436918.2:exon12:c.G1408A:p.E470K,ECE1:ENST00000374893.6:exon12:c.G1408A:p.E470K,E [illegible]	[illegible]	
1	22157788	C	T	ACGATCTCCT,GCCCTGGATG	exonic	HSPG2	nonsynonymous SNV	HSPG2:ENST00000374695.3:exon84:c.G11482A:p.E3828K,	[illegible]	
1	22446620	T	A	AATTTGCAGC,GCAGCGTTCA	exonic	WNT4	nonsynonymous SNV	WNT4:ENST00000542383.1:exon5:c.A814T:p.S272C,WNT4:ENST00000290167.6:exon5:c.A979T:p.S327C,	[illegible]	
1	23758313	G	A	GGATCCCCAG,TTCCAAGGGG	exonic	ASAP3	nonsynonymous SNV	ASAP3:ENST00000336689.3:exon23:c.C2422T:p.P808S,ASAP3:ENST00000495646.1:exon8:c.C934T:p.P312S,AS [illegible]	[illegible]	
1	24496071	C	T	CACTTCGCGC,ACCGTCTACG	exonic	IFNLR1	stopgain	IFNLR1:ENST00000374418.3:exon3:c.G203A:p.W68X,IFNLR1:ENST00000374421.3:exon3:c.G203A:p.W68X,IFNL [illegible]	[illegible]	
1	25228968	G	A	GCTGGTGGCC,GCATGCCCGC	exonic	RUNX3	nonsynonymous SNV	RUNX3:ENST00000540420.1:exon5:c.C614T:p.P205L,RUNX3:ENST00000308873.6:exon5:c.C893T:p.P298L,RUN [illegible]	[illegible]	
1	25228969	G	A	CTGGTGGCCG,CATGCCCGCC	exonic	RUNX3	nonsynonymous SNV	RUNX3:ENST00000540420.1:exon5:c.C613T:p.P205S,RUNX3:ENST00000308873.6:exon5:c.C892T:p.P298S,RUN [illegible]	[illegible]	
1	25245835	C	T	GAAACTCTTC,CTGGGGAGAG	exonic	RUNX3	nonsynonymous SNV	RUNX3:ENST00000540420.1:exon3:c.G161A:p.G54E,RUNX3:ENST00000308873.6:exon3:c.G440A:p.G147E,RUN [illegible]	[illegible]	
1	26310490	G	A	CGACGGAAAG,GATCCATTCC	exonic	PAFAH2	nonsynonymous SNV	PAFAH2:ENST00000374284.1:exon6:c.C499T:p.P167S,PAFAH2:ENST00000374282.3:exon6:c.C499T:p.P167S,P [illegible]	[illegible]	
1	26665835	G	A	CAGACCCTGG,AATTCTTCAA	exonic	AIM1L	nonsynonymous SNV	AIM1L:ENST00000308182.5:exon5:c.C155T:p.S52F,AIM1L:ENST00000527815.1:exon5:c.C668T:p.S223F,	[illegible]	
1	28477208	C	T	GTGATGACGC,CAGGAAGGCC	exonic	PTAFR	nonsynonymous SNV	PTAFR:ENST00000305392.3:exon2:c.G325A:p.G109S,PTAFR:ENST00000373857.3:exon2:c.G325A:p.G109S,PT [illegible]	[illegible]	
1	29185765	C	T	TGGGTCCTGG,CTCAGGCGTT	exonic	OPRD1	nonsynonymous SNV	OPRD1:ENST00000234961.2:exon2:c.C527T:p.A176V,	[illegible]	
1	29475079	C	T	GGATCTCGAC,TGGACCGGGT	exonic	SRSF4	nonsynonymous SNV	SRSF4:ENST00000373795.4:exon6:c.G1328A:p.R443K,	[illegible]	
1	33402826	C	T	GGTTTGGCTT,AATGTCCACT	exonic	RNF19B	nonsynonymous SNV	RNF19B:ENST00000373456.7:exon9:c.G1780A:p.E594K,RNF19B:ENST00000235150.4:exon9:c.G1777A:p.E593K [illegible]	[illegible]	
1	34038169	C	T	TCTGCAGTGG,CTCAGCGCCT	exonic	CSMD2	nonsynonymous SNV	CSMD2:ENST00000373381.4:exon50:c.G7699A:p.A2567T,CSMD2:ENST00000241312.4:exon51:c.G7705A:p.A25 [illegible]	[illegible]	
1	34383805	C	T	CCAGGATGGT,CATGTGCAGT	exonic	CSMD2	stopgain	CSMD2:ENST00000373381.4:exon5:c.G810A:p.W270X,CSMD2:ENST00000241312.4:exon5:c.G690A:p.W230X,	[illegible]	
1	36101943	T	A	CACGTTTTTC,GAATATATTC	exonic	PSMB2	nonsynonymous SNV	PSMB2:ENST00000373237.3:exon2:c.A182T:p.Q61L,	[illegible]	
1	36785279	G	A	ACGGCAGGAG,AAGAGCACAG	exonic	SH3D21	nonsynonymous SNV	SH3D21:ENST00000453908.2:exon14:c.G1015A:p.E339K,SH3D21:ENST00000505871.1:exon11:c.G682A:p.E228 [illegible]	[illegible]	
1	43637997	C	A	CGGCGGCAAA,CATACTTCCG	exonic	EBNA1BP2	nonsynonymous SNV	EBNA1BP2:ENST00000431635.2:exon1:c.G96T:p.W32C,	[illegible]	
1	45220458	C	T	GGAATTTGCC,GAATGATTAA	exonic	KIF2C	stopgain	KIF2C:ENST00000372224.4:exon8:c.C700T:p.R234X,KIF2C:ENST00000452259.1:exon7:c.C577T:p.R193X,KIF2C [illegible]	[illegible]	
1	45294713	C	T	AATACGTCAT,CACGCCGATT	exonic	PTCH2	nonsynonymous SNV	PTCH2:ENST00000447098.2:exon11:c.G1405A:p.D469N,PTCH2:ENST00000372192.3:exon11:c.G1405A:p.D469N [illegible]	[illegible]	
1	52492975	G	A	AAAATTATGG,AGAGTTCTGA	exonic	TXNDC12	nonsynonymous SNV	TXNDC12:ENST00000371626.4:exon4:c.C257T:p.S86F,	[illegible]	
1	52704513	C	T	AATTATCTAT,TAATGGTTGT	exonic	ZFYVE9	nonsynonymous SNV	ZFYVE9:ENST00000287727.3:exon4:c.C1424T:p.S475F,ZFYVE9:ENST00000371591.1:exon3:c.C1424T:p.S475F, [illegible]	[illegible]	
1	53792664	C	A	CTTGGCCGGC,CTGCGAGGGG	exonic	LRP8	nonsynonymous SNV	LRP8:ENST00000354412.3:exon2:c.G125T:p.G42V,LRP8:ENST00000371454.2:exon2:c.G125T:p.G42V,LRP8:EN [illegible]	[illegible]	
1	54640474	G	A	TAACTCCAGG,AAGCTGCTCT	exonic	CYB5RL	nonsynonymous SNV	CYB5RL:ENST00000542737.1:exon8:c.C766T:p.P256S,CYB5RL:ENST00000401046.3:exon6:c.C322T:p.P108S,C [illegible]	[illegible]	
1	63018492	G	A	CTTCGAGAAC,ATTTAAATTA	exonic	DOCK7	nonsynonymous SNV	DOCK7:ENST00000251157.5:exon22:c.C2677T:p.R893C,DOCK7:ENST00000340370.5:exon22:c.C2677T:p.R893 [illegible]	[illegible]	
1	63299779	G	A	TACTTTGCTG,ATTTCAAGGT	exonic	ATG4C	nonsynonymous SNV	ATG4C:ENST00000317868.4:exon8:c.G1004A:p.G335E,ATG4C:ENST00000371120.3:exon8:c.G1004A:p.G335E, [illegible]	[illegible]	
1	67861494	G	A	CAAGGTGCTG,AGAGCAGGGG	exonic	IL12RB2	nonsynonymous SNV	IL12RB2:ENST00000544434.1:exon14:c.G2053A:p.E685K,IL12RB2:ENST00000262345.1:exon16:c.G2311A:p.E77 [illegible]	[illegible]	
1	70831859	C	T	CCTTGTATTT,GTCTGGCAAC	exonic	HHLA3	nonsynonymous SNV	HHLA3:ENST00000525653.1:exon2:c.C187T:p.R63C,	[illegible]	
1	76262756	C	T	CCTCAGGGTC,CCGCTACAAT	exonic	MSH4	nonsynonymous SNV	MSH4:ENST00000263187.3:exon1:c.C86T:p.P29L,	[illegible]	
1	86965534	G	A	TGATAAGGTC,ATCTGAAATC	exonic	CLCA1	nonsynonymous SNV	CLCA1:ENST00000394711.1:exon14:c.G2551A:p.D851N,CLCA1:ENST00000234701.3:exon15:c.G2551A:p.D851N [illegible]	[illegible]	
1	93300361	A	G	ATAGAGGGGG,TATGATAGTC	exonic	RPL5	nonsynonymous SNV	RPL5:ENST00000370321.3:exon4:c.A215G:p.D72G,RPL5:ENST00000315741.5:exon4:c.A65G:p.D22G,	[illegible]	
1	103354167	C	T	ACCACTGTCA,CTTTCTGGCC	exonic	COL11A1	nonsynonymous SNV	COL11A1:ENST00000512756.1:exon60:c.G4226A:p.G1409D,COL11A1:ENST00000370096.3:exon62:c.G4574A:p. [illegible]	[illegible]	
1	114380964	G	A	AAAGTCAAAG,AAGAAGATTC	exonic	PTPN22	nonsynonymous SNV	PTPN22:ENST00000525799.1:exon8:c.C677T:p.S226F,PTPN22:ENST00000528414.1:exon11:c.C893T:p.S298F,P [illegible]	[illegible]	
1	114640427	C	T	GCATCTCGTT,CAGTGGTCCC	exonic	SYT6	stopgain	SYT6:ENST00000369547.1:exon6:c.G1182A:p.W394X,SYT6:ENST00000607941.1:exon6:c.G1182A:p.W394X,SY [illegible]	[illegible]	
1	114640428	C	T	CATCTCGTTC,AGTGGTCCCT	exonic	SYT6	stopgain	SYT6:ENST00000369547.1:exon6:c.G1181A:p.W394X,SYT6:ENST00000607941.1:exon6:c.G1181A:p.W394X,SY [illegible]	[illegible]	
1	118065485	C	A	GACCTTTTAC,TTTAGACCAC	exonic	MAN1A2	nonsynonymous SNV	MAN1A2:ENST00000356554.3:exon13:c.C1832A:p.P611H,MAN1A2:ENST00000422329.1:exon2:c.C149A:p.P50H [illegible]	[illegible]	
1	119427803	G	A	AGGCAACGAG,GAGGAGTTGG	exonic	TBX15	nonsynonymous SNV	TBX15:ENST00000369429.3:exon8:c.C1361T:p.P454L,TBX15:ENST00000449873.1:exon4:c.C545T:p.P182L,TBX [illegible]	[illegible]	
1	119467345	C	T	CCAGGTGTCT,CAGAAGCTAG	exonic	TBX15	nonsynonymous SNV	TBX15:ENST00000369429.3:exon4:c.G617A:p.G206E,TBX15:ENST00000207157.3:exon4:c.G299A:p.G100E,	[illegible]	
1	120468168	G	A	GCTCAGACAG,TGGCAGGAGG	exonic	NOTCH2	nonsynonymous SNV	NOTCH2:ENST00000256646.2:exon25:c.C4271T:p.T1424I,	[illegible]	
1	120468328	G	A	CAGTCCCGGG,ACTGGGGCAG	exonic	NOTCH2	nonsynonymous SNV	NOTCH2:ENST00000256646.2:exon25:c.C4111T:p.P1371S,	[illegible]	
1	120480579	G	A	GCTTTTTTCT,AACGCAAGTA	exonic	NOTCH2	stopgain	NOTCH2:ENST00000256646.2:exon20:c.C3238T:p.Q1080X,	[illegible]	
1	147131611	C	T	TGCATGCCCA,CTTGGTCAGC	exonic	ACP6	nonsynonymous SNV	ACP6:ENST00000392988.2:exon3:c.G379A:p.V127M,ACP6:ENST00000369238.6:exon3:c.G379A:p.V127M,	[illegible]	
1	152323423	C	T	TTGTTGAGAT,CAGCTTGGCC	exonic	FLG2	nonsynonymous SNV	FLG2:ENST00000388718.5:exon3:c.G6839A:p.G2280E,	[illegible]	
1	152382581	C	T	CTCAGTCCCT,TGTTCTGGCC	exonic	CRNN	nonsynonymous SNV	CRNN:ENST00000271835.3:exon3:c.G977A:p.R326K,	[illegible]	
1	152552312	G	A	ACAGCCAGAG,AAGCTGGAGG	exonic	LCE3D	nonsynonymous SNV	LCE3D:ENST00000368787.3:exon2:c.C101T:p.S34F,	[illegible]	
1	152692341	C	T	TGCCCAGCTC,CTGCCAGATG	exonic	C1orf68	nonsynonymous SNV	C1orf68:ENST00000368775.2:exon1:c.C344T:p.P115L,	[illegible]	
1	153430286	T	A	GCTGGGTCAC,GGCTGCCCCC	exonic	S100A7	nonsynonymous SNV	S100A7:ENST00000368723.3:exon3:c.A302T:p.Q101L,S100A7:ENST00000368722.1:exon3:c.A302T:p.Q101L,	[illegible]	
1	153658598	G	A	TCCATGCCCA,GGCAACCTCG	splicing	NPR1(ENST0	.	.	[illegible]	
1	154573959	C	T	AGGAACTCTG,GTTTCTTTTG	exonic	ADAR	nonsynonymous SNV	ADAR:ENST00000292205.5:exon2:c.G1288A:p.A430T,ADAR:ENST00000368471.3:exon2:c.G274A:p.A92T,ADAR [illegible]	[illegible]	
1	155265499	C	T	AATGTTCATC,CGGCCTTGAT	exonic	PKLR	nonsynonymous SNV	PKLR:ENST00000392414.3:exon3:c.G239A:p.G80E,PKLR:ENST00000342741.4:exon3:c.G332A:p.G111E,	[illegible]	
1	155838100	G	A	CATCAAAATG,ACTATGGGGA	exonic	SYT11	nonsynonymous SNV	SYT11:ENST00000368324.4:exon2:c.G379A:p.D127N,	[illegible]	
1	156035773	C	T	TGAGTTCAGC,ACGACAGCCG	exonic	RAB25	nonsynonymous SNV	RAB25:ENST00000361084.5:exon2:c.C115T:p.H39Y,	[illegible]	
1	156640829	G	A	TGGAGGCCTG,GGCCCCCACC	exonic	NES	nonsynonymous SNV	NES:ENST00000368223.3:exon4:c.C3151T:p.P1051S,	[illegible]	
1	158614107	G	A	ACTGTCTAAG,AACTTTTGTC	exonic	SPTA1	nonsynonymous SNV	SPTA1:ENST00000368147.4:exon30:c.C4274T:p.S1425F,	[illegible]	[illegible]
1	158725075	C	T	ATCTGCAATC,ACTCCGTTAC	exonic	OR6K6	nonsynonymous SNV	OR6K6:ENST00000368144.2:exon1:c.C470T:p.P157L,	[illegible]	
1	159828562	G	A	GAGTTGACCT,CATCCACTCG	exonic	VSIG8	stopgain	VSIG8:ENST00000368100.1:exon2:c.C190T:p.Q64X,	[illegible]	
1	161993049	G	A	CATCCTACCT,AGATAAAACG	exonic	OLFML2B	stopgain	OLFML2B:ENST00000294794.3:exon1:c.C172T:p.Q58X,OLFML2B:ENST00000367940.2:exon1:c.C172T:p.Q58X,	[illegible]	
1	165386362	C	T	ATGAGGCAGT,TTTATTATCC	exonic	RXRG	nonsynonymous SNV	RXRG:ENST00000359842.5:exon4:c.G538A:p.D180N,	[illegible]	
1	167095323	G	A	AGAGGAGGAC,ACAGCGCCAG	exonic	DUSP27	nonsynonymous SNV	DUSP27:ENST00000271385.5:exon6:c.G955A:p.D319N,DUSP27:ENST00000443333.1:exon5:c.G955A:p.D319N, [illegible]	[illegible]	[illegible]
1	168698136	C	T	TTGATCTCCT,CCACCAGCAC	exonic	DPT	nonsynonymous SNV	DPT:ENST00000367817.3:exon1:c.G277A:p.E93K,	[illegible]	
1	169823683	C	T	TCGATTTGGT,TGCGTCATCC	exonic	SCYL3	nonsynonymous SNV	SCYL3:ENST00000367772.4:exon13:c.G1897A:p.D633N,SCYL3:ENST00000367770.1:exon12:c.G1897A:p.D633N [illegible]	[illegible]	
1	170688939	A	G	AATAGCAGCC,GCTGCAGGCT	exonic	PRRX1	nonsynonymous SNV	PRRX1:ENST00000367760.3:exon2:c.A314G:p.Q105R,PRRX1:ENST00000239461.6:exon2:c.A314G:p.Q105R,PR [illegible]	[illegible]	
1	175048531	G	A	CTGCCACTGC,AAGAGGGCAG	exonic	TNN	nonsynonymous SNV	TNN:ENST00000239462.4:exon3:c.G472A:p.E158K,	[illegible]	
1	176740228	C	T	TCACTGCCTC,AGGACAACCA	exonic	PAPPA2	stopgain	PAPPA2:ENST00000367662.3:exon17:c.C4627T:p.Q1543X,	[illegible]	
1	176999980	G	A	CTGGCTGCTG,AGGTGTGAGA	exonic	ASTN1	nonsynonymous SNV	ASTN1:ENST00000367657.3:exon4:c.C974T:p.S325F,ASTN1:ENST00000367654.3:exon4:c.C974T:p.S325F,AST [illegible]	[illegible]	
1	178855234	C	T	AAGTTCTACT,TTTCTAGTGG	exonic	RALGPS2	nonsynonymous SNV	RALGPS2:ENST00000367634.2:exon13:c.C1171T:p.L391F,RALGPS2:ENST00000367635.3:exon13:c.C1171T:p.L [illegible]	[illegible]	
1	180063171	C	T	CAGGACATTT,TGGGGTACTT	exonic	CEP350	nonsynonymous SNV	CEP350:ENST00000367607.3:exon34:c.C7931T:p.S2644F,CEP350:ENST00000417046.1:exon1:c.C323T:p.S108F [illegible]	[illegible]	
1	181701902	C	T	AAACTGTGAC,CGACTCAGCA	exonic	CACNA1E	nonsynonymous SNV	CACNA1E:ENST00000360108.3:exon19:c.C2623T:p.P875S,CACNA1E:ENST00000367570.1:exon20:c.C2680T:p. [illegible]	[illegible]	
1	181740487	G	A	AACAACTTCC,GAGTTTCTTT	exonic	CACNA1E	nonsynonymous SNV	CACNA1E:ENST00000360108.3:exon35:c.G4883A:p.R1628Q,CACNA1E:ENST00000367570.1:exon36:c.G4940A: [illegible]	[illegible]	[illegible]
1	183106862	A	T	ACAGATCTGG,TAATGAGGTG	exonic	LAMC1	nonsynonymous SNV	LAMC1:ENST00000258341.4:exon26:c.A4373T:p.D1458V,	[illegible]	
1	185191084	C	T	ACTGTTTTCT,GAGTTCTCAT	exonic	SWT1	nonsynonymous SNV	SWT1:ENST00000367500.4:exon15:c.C2225T:p.S742L,SWT1:ENST00000367501.3:exon15:c.C2225T:p.S742L,	[illegible]	[illegible]
1	186113704	G	T	TGGAGTGCCT,TTCTGTGTCA	exonic	HMCN1	nonsynonymous SNV	HMCN1:ENST00000271588.4:exon91:c.G14135T:p.C4712F,HMCN1:ENST00000367492.2:exon91:c.G14135T:p.C [illegible]	[illegible]	
1	186294905	G	T	CCACTGTTTT,AGAATCAGCA	exonic	TPR	nonsynonymous SNV	TPR:ENST00000367478.4:exon42:c.C6103A:p.Q2035K,	[illegible]	[illegible]
1	186294913	G	T	TTGAGAATCA,CAGCTCTGTG	exonic	TPR	nonsynonymous SNV	TPR:ENST00000367478.4:exon42:c.C6095A:p.A2032D,	[illegible]	
1	196965187	G	A	ACCTGAACTC,AGTACGGTTA	exonic	CFHR5	nonsynonymous SNV	CFHR5:ENST00000256785.4:exon6:c.G826A:p.E276K,CFHR5:ENST00000367414.5:exon6:c.G898A:p.E300K,	[illegible]	
1	197112835	G	T	TCAACTTTTT,GGAAACACTA	exonic	ASPM	nonsynonymous SNV	ASPM:ENST00000294732.7:exon3:c.C547A:p.Q183K,ASPM:ENST00000367409.4:exon3:c.C547A:p.Q183K,	[illegible]	
1	197398613	C	T	GTTTGCCATT,CCGGTGGGAT	exonic	CRB1	nonsynonymous SNV	CRB1:ENST00000484075.1:exon8:c.C2711T:p.S904F,CRB1:ENST00000367399.2:exon6:c.C2375T:p.S792F,CRB [illegible]	[illegible]	
1	201022631	C	T	CGCACTCCTT,TGCCCGGCTC	exonic	CACNA1S	nonsynonymous SNV	CACNA1S:ENST00000362061.3:exon30:c.G3751A:p.E1251K,CACNA1S:ENST00000367338.3:exon29:c.G3694A: [illegible]	[illegible]	
1	201178726	G	A	TTATAGGAAG,ATTTGGGGGC	exonic	IGFN1	nonsynonymous SNV	IGFN1:ENST00000335211.4:exon12:c.G4705A:p.D1569N,	[illegible]	
1	201180256	G	A	GGCTCCTAAG,GAATGGGTTC	exonic	IGFN1	nonsynonymous SNV	IGFN1:ENST00000335211.4:exon12:c.G6235A:p.G2079R,	[illegible]	
1	201181964	C	T	CCCGCAGGCT,CAGAGCTTCC	exonic	IGFN1	nonsynonymous SNV	IGFN1:ENST00000335211.4:exon12:c.C7943T:p.S2648F,	[illegible]	
1	201182107	G	A	GGATAGCAAA,GTTCAAGTCC	exonic	IGFN1	nonsynonymous SNV	IGFN1:ENST00000335211.4:exon12:c.G8086A:p.G2696S,	[illegible]	
1	203192347	C	T	GCGTTCCTTC,CTGAGGTCTG	exonic	CHIT1	nonsynonymous SNV	CHIT1:ENST00000535569.1:exon5:c.G494A:p.G165E,CHIT1:ENST00000367229.1:exon6:c.G521A:p.G174E,CHIT [illegible]	[illegible]	
1	203317182	G	A	CAGTCGCGGG,ATCTGGAGGG	exonic	FMOD	nonsynonymous SNV	FMOD:ENST00000354955.4:exon2:c.C217T:p.P73S,	[illegible]	
1	203467877	G	A	TGACCTAGAG,ACATTCCTCC	exonic	OPTC	nonsynonymous SNV	OPTC:ENST00000448911.1:exon3:c.G373A:p.D125N,OPTC:ENST00000367222.2:exon4:c.G439A:p.D147N,	[illegible]	
1	203690445	C	T	CCCCTATGGC,GAAATAAGCC	exonic	ATP2B4	stopgain	ATP2B4:ENST00000367219.3:exon16:c.C2683T:p.R895X,ATP2B4:ENST00000341360.2:exon16:c.C2719T:p.R907 [illegible]	[illegible]	
1	204167617	C	T	TTGAAGTCTC,GGAACAGCTG	exonic	GOLT1A	nonsynonymous SNV	GOLT1A:ENST00000308302.3:exon5:c.G368A:p.R123Q,	[illegible]	
1	210857326	C	T	CCGGCCCCCT,TCTCAGCTGC	exonic	KCNH1	nonsynonymous SNV	KCNH1:ENST00000271751.4:exon11:c.G2267A:p.R756K,KCNH1:ENST00000367007.4:exon11:c.G2186A:p.R729 [illegible]	[illegible]	
1	216680456	T	A	AGCTTCATAA,CCTGCAGCGC	exonic	ESRRG	nonsynonymous SNV	ESRRG:ENST00000361395.2:exon8:c.A1133T:p.D378V,ESRRG:ENST00000493603.1:exon9:c.A1133T:p.D378V, [illegible]	[illegible]	
1	222713514	C	T	GTGATGGGGT,CCCTCGGTCC	exonic	HHIPL2	nonsynonymous SNV	HHIPL2:ENST00000343410.6:exon4:c.G1288A:p.D430N,	[illegible]	
1	223568150	G	A	CAAGGCTGAG,AGCTCCTTAG	exonic	C1orf65	nonsynonymous SNV	C1orf65:ENST00000366875.3:exon1:c.G1333A:p.E445K,	[illegible]	
1	224619416	G	A	ACATGATTTC,GAATTTGGTA	exonic	WDR26	stopgain	WDR26:ENST00000295024.6:exon2:c.C49T:p.R17X,WDR26:ENST00000414423.2:exon2:c.C490T:p.R164X,WDR [illegible]	[illegible]	
1	236740167	A	T	ATGCCACTCC,CTGAGGGCCT	exonic	HEATR1	nonsynonymous SNV	HEATR1:ENST00000366582.3:exon21:c.T2838A:p.S946R,HEATR1:ENST00000366581.2:exon21:c.T2838A:p.S94 [illegible]	[illegible]	
1	237586451	G	A	GTCACAACAG,AAAATACTTG	exonic	RYR2	nonsynonymous SNV	RYR2:ENST00000360064.6:exon13:c.G902A:p.G301E,RYR2:ENST00000542537.1:exon11:c.G860A:p.G287E,RY [illegible]	[illegible]	
1	237620001	G	A	GAGAGTCTTG,AAATCCATTC	exonic	RYR2	stopgain	RYR2:ENST00000360064.6:exon17:c.G1572A:p.W524X,RYR2:ENST00000542537.1:exon15:c.G1530A:p.W510X, [illegible]	[illegible]	
1	238050775	G	A	AGGGCCAAGC,CACAGAATCC	exonic	ZP4	nonsynonymous SNV	ZP4:ENST00000366570.4:exon5:c.C640T:p.R214C,	[illegible]	
1	246498741	T	A	TTTTAAGGCA,TTGCATTCCC	exonic	SMYD3	nonsynonymous SNV	SMYD3:ENST00000403792.3:exon3:c.A264T:p.K88N,SMYD3:ENST00000490107.1:exon3:c.A87T:p.K29N,SMYD3 [illegible]	[illegible]	
1	248004713	C	T	ACCTGGAAAT,ATCAGGGAAG	exonic	OR11L1	nonsynonymous SNV	OR11L1:ENST00000355784.2:exon1:c.G486A:p.M162I,	[illegible]	[illegible]
2	1544473	C	T	CCGCAGCGGG,CGCAGCTCAG	exonic	TPO	nonsynonymous SNV	TPO:ENST00000382201.3:exon15:c.C2555T:p.A852V,TPO:ENST00000469607.1:exon6:c.C1016T:p.A339V,TPO: [illegible]	[illegible]	
2	3391969	C	T	AGCGAGGCCT,GGCCAGGACA	exonic	TRAPPC12	nonsynonymous SNV	TRAPPC12:ENST00000382110.2:exon2:c.C575T:p.S192L,TRAPPC12:ENST00000324266.5:exon2:c.C575T:p.S1 [illegible]	[illegible]	
2	3392179	C	T	CGCCCCGAAC,CGTGGCCATG	exonic	TRAPPC12	nonsynonymous SNV	TRAPPC12:ENST00000382110.2:exon2:c.C785T:p.P262L,TRAPPC12:ENST00000324266.5:exon2:c.C785T:p.P2 [illegible]	[illegible]	
2	5834079	C	T	AGCCTGGGCT,CCACTTCGAG	exonic	SOX11	nonsynonymous SNV	SOX11:ENST00000322002.3:exon1:c.C1226T:p.S409F,	[illegible]	
2	11053814	C	T	GTCCTGGAGA,CGCGGCCAAG	exonic	KCNF1	nonsynonymous SNV	KCNF1:ENST00000295082.1:exon1:c.C1262T:p.T421I,	[illegible]	
2	11718443	C	T	CCGGCAGATC,CCGCCAGTAC	exonic	GREB1	nonsynonymous SNV	GREB1:ENST00000234142.5:exon5:c.C658T:p.P220S,GREB1:ENST00000389825.3:exon5:c.C328T:p.P110S,GR [illegible]	[illegible]	
2	11718444	C	T	CGGCAGATCC,CGCCAGTACT	exonic	GREB1	nonsynonymous SNV	GREB1:ENST00000234142.5:exon5:c.C659T:p.P220L,GREB1:ENST00000389825.3:exon5:c.C329T:p.P110L,GR [illegible]	[illegible]	[illegible]
2	11725308	C	T	AACTCCGGGC,CCCCAAAAAA	exonic	GREB1	nonsynonymous SNV	GREB1:ENST00000234142.5:exon7:c.C923T:p.P308L,GREB1:ENST00000381486.2:exon8:c.C923T:p.P308L,GR [illegible]	[illegible]	
2	18112844	C	T	ATCGCTATCT,CTCCTTGAGC	exonic	KCNS3	nonsynonymous SNV	KCNS3:ENST00000304101.4:exon3:c.C569T:p.S190F,KCNS3:ENST00000403915.1:exon3:c.C569T:p.S190F,	[illegible]	
2	26410600	C	T	GTCCTGGAGC,CTTCGATCCC	exonic	GAREML	nonsynonymous SNV	GAREML:ENST00000407684.1:exon6:c.C1469T:p.P490L,GAREML:ENST00000401533.2:exon6:c.C2099T:p.P700 [illegible]	[illegible]	
2	26532924	G	A	GGAGATGGTG,AGCTGGGGGC	exonic	GPR113	nonsynonymous SNV	GPR113:ENST00000447444.1:exon10:c.C2531T:p.S844F,GPR113:ENST00000421160.2:exon12:c.C2921T:p.S97 [illegible]	[illegible]	
2	27606414	C	A	CTACAATCAA,TGCTTCCCTC	exonic	PPM1G	nonsynonymous SNV	PPM1G:ENST00000350803.4:exon8:c.G1020T:p.Q340H,PPM1G:ENST00000344034.4:exon7:c.G1020T:p.Q340H, [illegible]	[illegible]	
2	27886962	G	A	CAATTCTGGG,AGCCCGACAT	exonic	SLC4A1AP	nonsynonymous SNV	SLC4A1AP:ENST00000326019.6:exon1:c.G343A:p.E115K,	[illegible]	
2	27997320	C	T	GTGAGCGAAG,TGGGACAGGT	exonic	MRPL33	nonsynonymous SNV	MRPL33:ENST00000296102.3:exon3:c.C71T:p.A24V,	[illegible]	
2	28635113	C	T	GGTGAGGAGC,CCTGCACACC	exonic	FOSL2	nonsynonymous SNV	FOSL2:ENST00000545753.1:exon4:c.C662T:p.P221L,FOSL2:ENST00000379619.1:exon4:c.C755T:p.P252L,FOSL [illegible]	[illegible]	
2	29297125	C	T	GTGTACACCC,ATGATTTCAG	exonic	C2orf71	nonsynonymous SNV	C2orf71:ENST00000331664.5:exon1:c.G3A:p.M1I,	[illegible]	
2	31483577	C	T	GTGTACGGGG,CCTCATGTGG	exonic	EHD3	nonsynonymous SNV	EHD3:ENST00000322054.5:exon4:c.C704T:p.A235V,	[illegible]	
2	47596784	C	T	GGCCCCCGGC,CTCGGCCCCC	exonic	EPCAM	nonsynonymous SNV	EPCAM:ENST00000419334.1:exon1:c.C140T:p.P47L,	[illegible]	
2	54856315	G	A	CAAGCACCGG,CGTTCGAGGA	exonic	SPTBN1	nonsynonymous SNV	SPTBN1:ENST00000389980.3:exon14:c.G2044A:p.A682T,SPTBN1:ENST00000356805.4:exon14:c.G2044A:p.A6 [illegible]	[illegible]	
2	54891675	C	T	CCCATCCCCT,CCCGACCTCT	exonic	SPTBN1	nonsynonymous SNV	SPTBN1:ENST00000356805.4:exon33:c.C6506T:p.S2169F,	[illegible]	
2	61508257	G	A	GCATCAGGAA,AAATTTCAAT	exonic	USP34	nonsynonymous SNV	USP34:ENST00000398571.2:exon38:c.C5119T:p.L1707F,	[illegible]	
2	74328483	C	T	AACAGCACCT,GGCCTTGGCT	exonic	TET3	nonsynonymous SNV	TET3:ENST00000409262.3:exon9:c.C4163T:p.S1388L,	[illegible]	
2	79385574	G	A	CCAGAGGGCC,CTTCTGGCAG	exonic	REG3A	nonsynonymous SNV	REG3A:ENST00000305165.2:exon4:c.C211T:p.R71W,REG3A:ENST00000409839.3:exon4:c.C211T:p.R71W,REG [illegible]	[illegible]	
2	86115992	G	A	GGCTGCAGGG,ACGCCGCTCC	exonic	ST3GAL5	nonsynonymous SNV	ST3GAL5:ENST00000306262.5:exon1:c.C37T:p.P13S,ST3GAL5:ENST00000433665.1:exon1:c.C37T:p.P13S,ST3 [illegible]	[illegible]	
2	88425751	C	T	ACCGTGAATT,GTTTTGGATC	exonic	FABP1	nonsynonymous SNV	FABP1:ENST00000295834.3:exon2:c.G184A:p.E62K,FABP1:ENST00000393750.3:exon2:c.G184A:p.E62K,	[illegible]	[illegible]
2	88474274	G	A	TGCATTTAAG,ACCTGTCCCT	exonic	THNSL2	nonsynonymous SNV	THNSL2:ENST00000402102.1:exon3:c.G340A:p.D114N,THNSL2:ENST00000324166.5:exon2:c.G340A:p.D114N,T [illegible]	[illegible]	
2	88892898	C	T	TTGGCGACAA,CCAGAGCTGA	exonic	EIF2AK3	nonsynonymous SNV	EIF2AK3:ENST00000415570.1:exon3:c.G296A:p.G99D,EIF2AK3:ENST00000419748.1:exon4:c.G206A:p.G69D,EI [illegible]	[illegible]	
2	111408326	G	A	CATGTGAGAC,ACAAGGCCTG	exonic	BUB1	nonsynonymous SNV	BUB1:ENST00000302759.6:exon18:c.C2000T:p.S667L,BUB1:ENST00000409311.1:exon18:c.C2000T:p.S667L,BU [illegible]	[illegible]	
2	112917364	G	A	GAGCCGGCTG,CCGCGCTGCA	exonic	FBLN7	nonsynonymous SNV	FBLN7:ENST00000409450.3:exon2:c.G187A:p.A63T,FBLN7:ENST00000441565.1:exon1:c.G40A:p.A14T,FBLN7: [illegible]	[illegible]	
2	113479640	G	A	GTACCCGCAT,AGATCCTGCG	exonic	NT5DC4	nonsynonymous SNV	NT5DC4:ENST00000327581.4:exon3:c.G163A:p.E55K,	[illegible]	
2	113940833	C	T	GAGTGCTTTT,CTGGGGGGCT	exonic	PSD4	nonsynonymous SNV	PSD4:ENST00000441564.3:exon2:c.C800T:p.S267F,PSD4:ENST00000245796.6:exon2:c.C800T:p.S267F,	[illegible]	
2	119726772	C	T	GTGAACTTCT,CCTAGCTGTG	exonic	MARCO	nonsynonymous SNV	MARCO:ENST00000327097.4:exon2:c.C134T:p.S45F,	[illegible]	
2	128178930	G	A	CTCCTTCCTG,AGGAGCTCCG	exonic	PROC	nonsynonymous SNV	PROC:ENST00000431364.1:exon2:c.G107A:p.G36E,PROC:ENST00000442644.1:exon3:c.G142A:p.E48K,PROC: [illegible]	[illegible]	
2	131487556	G	A	GAGCACAGGG,TGGTGTTCTC	exonic	GPR148	nonsynonymous SNV	GPR148:ENST00000309926.4:exon1:c.G832A:p.V278M,	[illegible]	
2	136567434	G	A	GGGAGTCCTT,ACTTGCTGCT	exonic	LCT	nonsynonymous SNV	LCT:ENST00000264162.2:exon8:c.C2483T:p.S828L,LCT:ENST00000452974.1:exon2:c.C779T:p.S260L,	[illegible]	
2	141299395	G	T	TGGCTGATGT,GAATATCGGA	exonic	LRP1B	nonsynonymous SNV	LRP1B:ENST00000389484.3:exon44:c.C7340A:p.P2447Q,	[illegible]	
2	152321841	G	A	ACCAAAACTG,TATTTCTGAA	exonic	RIF1	nonsynonymous SNV	RIF1:ENST00000243326.5:exon29:c.G5807A:p.G1936D,RIF1:ENST00000430328.2:exon29:c.G5807A:p.G1936D, [illegible]	[illegible]	
2	170537695	G	A	TACTTTGCTA,GTAGAAGAGG	exonic	CCDC173	nonsynonymous SNV	CCDC173:ENST00000447353.1:exon2:c.C116T:p.P39L,	[illegible]	
2	178986065	C	T	TGAAGTTCAA,GAGATCCTTA	exonic	RBM45	stopgain	RBM45:ENST00000286070.5:exon5:c.C832T:p.R278X,	[illegible]	
2	179439461	G	A	GCTTTTACAC,GAACTGATAC	exonic	TTN	nonsynonymous SNV	TTN:ENST00000342992.6:exon275:c.C63694T:p.R21232C,TTN:ENST00000589042.1:exon326:c.C71398T:p.R23 [illegible]	[illegible]	
2	179440541	G	A	CGCAGGTTGA,GACTGGGCCT	exonic	TTN	nonsynonymous SNV	TTN:ENST00000342992.6:exon275:c.C62614T:p.L20872F,TTN:ENST00000589042.1:exon326:c.C70318T:p.L234 [illegible]	[illegible]	
2	179610460	C	T	GAACCTTCTT,CTTGGACTAA	exonic	TTN	nonsynonymous SNV	TTN:ENST00000360870.5:exon46:c.G16667A:p.G5556E,	[illegible]	
2	179614852	G	T	GTCTCCAGAG,GAGGAACTGG	exonic	TTN	nonsynonymous SNV	TTN:ENST00000360870.5:exon46:c.C12275A:p.P4092H,	[illegible]	
2	179616629	C	T	TTAAAATATT,AGGCCAGGAG	exonic	TTN	nonsynonymous SNV	TTN:ENST00000360870.5:exon46:c.G10498A:p.E3500K,	[illegible]	
2	179665278	C	T	CTCTGGATTT,GGCTCCATCC	exonic	TTN	nonsynonymous SNV	TTN:ENST00000342992.6:exon4:c.G427A:p.E143K,TTN:ENST00000589042.1:exon4:c.G427A:p.E143K,TTN:ENS [illegible]	[illegible]	
2	179826118	G	A	GTCAACTCAG,ATTCACATTA	exonic	CCDC141	nonsynonymous SNV	CCDC141:ENST00000420890.2:exon5:c.C619T:p.P207S,CCDC141:ENST00000443758.1:exon5:c.C619T:p.P207S [illegible]	[illegible]	
2	192252073	G	A	AGGGAATCCC,CCAAGATCAA	exonic	MYO1B	nonsynonymous SNV	MYO1B:ENST00000392316.1:exon16:c.G1678A:p.A560T,MYO1B:ENST00000304164.4:exon17:c.G1678A:p.A560 [illegible]	[illegible]	[illegible]
2	197183719	G	A	CTCTCCCTCA,GCCTGGTGCT	exonic	HECW2	nonsynonymous SNV	HECW2:ENST00000260983.3:exon9:c.C1895T:p.P632L,HECW2:ENST00000409111.1:exon7:c.C827T:p.P276L,	[illegible]	
2	197183720	G	A	TCTCCCTCAG,CCTGGTGCTG	exonic	HECW2	nonsynonymous SNV	HECW2:ENST00000260983.3:exon9:c.C1894T:p.P632S,HECW2:ENST00000409111.1:exon7:c.C826T:p.P276S,	[illegible]	
2	207814409	C	T	GAGACGTATT,CTATAACATA	exonic	CPO	nonsynonymous SNV	CPO:ENST00000272852.3:exon2:c.C137T:p.S46F,	[illegible]	
2	214149256	G	A	GGTCCTGGAA,AGGCGTTGGG	exonic	SPAG16	nonsynonymous SNV	SPAG16:ENST00000420497.2:exon1:c.G49A:p.E17K,SPAG16:ENST00000406979.2:exon1:c.G49A:p.E17K,SPAG [illegible]	[illegible]	
2	215866380	G	A	TACACAAGAG,AAATATTTAC	exonic	ABCA12	nonsynonymous SNV	ABCA12:ENST00000272895.7:exon21:c.C2765T:p.S922F,ABCA12:ENST00000389661.4:exon13:c.C1811T:p.S60 [illegible]	[illegible]	
2	217724700	G	A	CCCTTGTGAG,AGATCGGCTC	exonic	TNP1	nonsynonymous SNV	TNP1:ENST00000236979.2:exon1:c.C58T:p.P20S,	[illegible]	
2	219495466	G	A	GATCCTGGGG,AGCAGCTGCT	exonic	PLCD4	nonsynonymous SNV	PLCD4:ENST00000450993.2:exon9:c.G1213A:p.E405K,PLCD4:ENST00000432688.1:exon9:c.G1213A:p.E405K,P [illegible]	[illegible]	
2	219875545	G	C	TCTTGGCCTC,ATAGGTGAGA	exonic	CCDC108	nonsynonymous SNV	CCDC108:ENST00000441968.1:exon24:c.C4131G:p.I1377M,CCDC108:ENST00000341552.5:exon25:c.C4131G:p [illegible]	[illegible]	
2	220102332	A	T	GCTTGAGGAT,TGGCCATTTT	exonic	GLB1L	nonsynonymous SNV	GLB1L:ENST00000356283.3:exon14:c.T1321A:p.Y441N,GLB1L:ENST00000392089.2:exon16:c.T1591A:p.Y531N, [illegible]	[illegible]	
2	220313760	C	T	AAAGCACCCC,CGGTCGGAAG	exonic	SPEG	nonsynonymous SNV	SPEG:ENST00000312358.7:exon4:c.C1880T:p.P627L,SPEG:ENST00000396698.1:exon5:c.C1568T:p.P523L,	[illegible]	
2	223158858	G	A	CACCCAGCAA,TGCGCCGCCC	exonic	PAX3	nonsynonymous SNV	PAX3:ENST00000409828.3:exon4:c.C614T:p.T205I,	[illegible]	
2	227915812	C	T	CTGTGAAATC,AGGTGGTCCG	exonic	COL4A4	nonsynonymous SNV	COL4A4:ENST00000329662.7:exon33:c.G3031A:p.G1011R,COL4A4:ENST00000396625.3:exon33:c.G3031A:p.G [illegible]	[illegible]	
2	227924276	G	A	AGGGCCTGGG,GCCCAACAGG	exonic	COL4A4	nonsynonymous SNV	COL4A4:ENST00000329662.7:exon28:c.C2228T:p.P743L,COL4A4:ENST00000396625.3:exon28:c.C2228T:p.P74 [illegible]	[illegible]	
2	231223774	G	T	TGTCAGTTTT,GAAGCACTGT	exonic	SP140L	nonsynonymous SNV	SP140L:ENST00000396563.4:exon4:c.G366T:p.L122F,SP140L:ENST00000458341.1:exon3:c.G105T:p.L35F,SP14 [illegible]	[illegible]	
2	233834931	C	T	TACCTCATTT,CTGGGCTCCT	exonic	NGEF	nonsynonymous SNV	NGEF:ENST00000264051.3:exon3:c.G376A:p.E126K,	[illegible]	[illegible]
2	233839497	A	G	TTTTTCTGGG,GTAACTCAGG	exonic	NGEF	nonsynonymous SNV	NGEF:ENST00000264051.3:exon2:c.T104C:p.L35P,	[illegible]	
2	234355374	T	A	CGTCATGGCA,ACGAGGCCAA	exonic	DGKD	nonsynonymous SNV	DGKD:ENST00000409813.3:exon11:c.T1219A:p.Y407N,DGKD:ENST00000264057.2:exon12:c.T1351A:p.Y451N,	[illegible]	
2	234590687	G	A	CCCATGGATG,GAGCCACTGG	exonic	UGT1A7	nonsynonymous SNV	UGT1A7:ENST00000373426.3:exon1:c.G104A:p.G35E,	[illegible]	
3	5024917	G	T	GAGCAGCCGT,CTTCAAAAGT	exonic	BHLHE40	nonsynonymous SNV	BHLHE40:ENST00000256495.3:exon5:c.G779T:p.C260F,	[illegible]	
3	9948710	C	T	ATCTTCAGGC,CTGAGGTCAG	exonic	IL17RE	nonsynonymous SNV	IL17RE:ENST00000295980.3:exon7:c.C592T:p.P198S,IL17RE:ENST00000454992.1:exon5:c.C472T:p.P158S,IL17 [illegible]	[illegible]	
3	12858719	C	T	TTCCTGCAGG,CCTGGTAGGG	exonic	CAND2	nonsynonymous SNV	CAND2:ENST00000456430.2:exon10:c.C2288T:p.A763V,CAND2:ENST00000295989.5:exon8:c.C2009T:p.A670V, [illegible]	[illegible]	
3	38104186	C	T	CCTAAAAAAT,CCCGTTTTTT	exonic	DLEC1	nonsynonymous SNV	DLEC1:ENST00000308059.6:exon5:c.C988T:p.P330S,DLEC1:ENST00000452631.2:exon5:c.C988T:p.P330S,DLE [illegible]	[illegible]	
3	47933885	C	T	TGACTGATTT,TTTTGGGGAG	exonic	MAP4	nonsynonymous SNV	MAP4:ENST00000420772.2:exon1:c.G97A:p.E33K,	[illegible]	
3	48476337	C	T	ATGCTGTGCC,CAGGGCTCTT	exonic	CCDC51	nonsynonymous SNV	CCDC51:ENST00000446140.1:exon2:c.G202A:p.G68R,CCDC51:ENST00000395696.1:exon2:c.G202A:p.G68R,CC [illegible]	[illegible]	
3	48679362	T	C	GTCCGGCCAT,GTCCTCGCTT	exonic	CELSR3	nonsynonymous SNV	CELSR3:ENST00000164024.4:exon32:c.A8746G:p.N2916D,CELSR3:ENST00000544264.1:exon33:c.A8761G:p.N [illegible]	[illegible]	
3	50153387	G	T	GCAGGAGCTG,AAGCCTTGGA	exonic	RBM5	stopgain	RBM5:ENST00000347869.3:exon22:c.G2068T:p.E690X,	[illegible]	
3	50402826	G	A	GTTGTAGGAG,CGTTTACCGA	exonic	CACNA2D2	nonsynonymous SNV	CACNA2D2:ENST00000395083.1:exon35:c.C3059T:p.A1020V,CACNA2D2:ENST00000435965.1:exon36:c.C3080 [illegible]	[illegible]	
3	56707663	G	T	GTTAAAATTT,TTACTGAGGT	exonic	FAM208A	nonsynonymous SNV	FAM208A:ENST00000493960.2:exon2:c.C422A:p.T141K,FAM208A:ENST00000355628.5:exon2:c.C422A:p.T141K [illegible]	[illegible]	
3	56707667	C	T	AAATTTGTTA,TGAGGTTGGT	exonic	FAM208A	nonsynonymous SNV	FAM208A:ENST00000493960.2:exon2:c.G418A:p.V140I,FAM208A:ENST00000355628.5:exon2:c.G418A:p.V140I, [illegible]	[illegible]	
3	64085073	C	T	CTGGAAGCTC,GCTGGCGCAT	exonic	PRICKLE2	nonsynonymous SNV	PRICKLE2:ENST00000564377.1:exon9:c.G2357A:p.R786Q,PRICKLE2:ENST00000295902.6:exon8:c.G2189A:p.R [illegible]	[illegible]	
3	98519549	C	T	TGCTTCATTC,TTTCCACCAA	exonic	DCBLD2	nonsynonymous SNV	DCBLD2:ENST00000326857.9:exon15:c.G1774A:p.G592R,DCBLD2:ENST00000326840.6:exon15:c.G1732A:p.G5 [illegible]	[illegible]	
3	100352174	G	T	ATTACAAAAA,TGACAATAGG	exonic	GPR128	nonsynonymous SNV	GPR128:ENST00000273352.3:exon4:c.G400T:p.V134L,	[illegible]	
3	101447691	G	A	CCATGGAACA,ATCAATAGCT	exonic	CEP97	nonsynonymous SNV	CEP97:ENST00000465011.1:exon4:c.G379A:p.D127N,	[illegible]	
3	121208413	C	A	GTTCCATGAA,AATTTTGCTT	exonic	POLQ	nonsynonymous SNV	POLQ:ENST00000264233.5:exon16:c.G3365T:p.C1122F,	[illegible]	
3	121415343	C	T	TGAAATACCT,TTCTGATTTT	exonic	GOLGB1	nonsynonymous SNV	GOLGB1:ENST00000393667.3:exon13:c.G4027A:p.E1343K,GOLGB1:ENST00000494517.1:exon12:c.G3904A:p.E [illegible]	[illegible]	[illegible]
3	122288331	A	T	GAAAGCACTT,CATCAGACCA	exonic	DTX3L	nonsynonymous SNV	DTX3L:ENST00000296161.4:exon3:c.A1395T:p.L465F,	[illegible]	
3	122646813	C	T	GCGGGCCACA,CATTGATCTT	exonic	SEMA5B	nonsynonymous SNV	SEMA5B:ENST00000357599.3:exon8:c.G674A:p.G225D,SEMA5B:ENST00000475244.1:exon8:c.G674A:p.G225D [illegible]	[illegible]	
3	123419433	C	T	CTTGGAAGTC,CCTTCTTGGC	exonic	MYLK	nonsynonymous SNV	MYLK:ENST00000360772.3:exon19:c.G2882A:p.G961E,MYLK:ENST00000359169.1:exon18:c.G2882A:p.G961E, [illegible]	[illegible]	
3	126133119	C	T	GAGCACTTTC,TCAGGTCCTT	exonic	CCDC37	nonsynonymous SNV	CCDC37:ENST00000510833.1:exon4:c.C322T:p.L108F,	[illegible]	
3	126446005	G	A	CCTTCAAGAT,GCAACTTGAG	exonic	CHCHD6	nonsynonymous SNV	CHCHD6:ENST00000503119.1:exon2:c.G172A:p.G58S,CHCHD6:ENST00000290913.3:exon2:c.G172A:p.G58S,C [illegible]	[illegible]	
3	126446006	G	A	CTTCAAGATG,CAACTTGAGA	exonic	CHCHD6	nonsynonymous SNV	CHCHD6:ENST00000503119.1:exon2:c.G173A:p.G58D,CHCHD6:ENST00000290913.3:exon2:c.G173A:p.G58D,C [illegible]	[illegible]	
3	126915988	C	T	CAGCCACTGT,CCCAGGTGGG	exonic	C3orf56	nonsynonymous SNV	C3orf56:ENST00000398112.1:exon2:c.C460T:p.P154S,	[illegible]	
3	127292554	G	A	CACAGCGTAG,GCTGGGGTAG	exonic	TPRA1	nonsynonymous SNV	TPRA1:ENST00000450633.2:exon10:c.C932T:p.P311L,TPRA1:ENST00000355552.3:exon11:c.C932T:p.P311L,TP [illegible]	[illegible]	
3	127292555	G	A	ACAGCGTAGG,CTGGGGTAGG	exonic	TPRA1	nonsynonymous SNV	TPRA1:ENST00000450633.2:exon10:c.C931T:p.P311S,TPRA1:ENST00000296210.7:exon9:c.C767T:p.A256V,TP [illegible]	[illegible]	
3	128060226	C	T	GCACACTGTC,ATGCGGCCCT	exonic	EEFSEC	nonsynonymous SNV	EEFSEC:ENST00000254730.6:exon5:c.C937T:p.H313Y,EEFSEC:ENST00000483457.1:exon4:c.C772T:p.H258Y, [illegible]	[illegible]	
3	129284280	G	A	GTTCTTGGCG,CCGAGGCGTC	exonic	PLXND1	nonsynonymous SNV	PLXND1:ENST00000324093.4:exon25:c.C4424T:p.A1475V,PLXND1:ENST00000393239.1:exon25:c.C4424T:p.A1 [illegible]	[illegible]	
3	129370533	G	A	TTGCCCAGAA,GTTCCGGGCA	exonic	TMCC1	nonsynonymous SNV	TMCC1:ENST00000426664.2:exon6:c.C1411T:p.L471F,TMCC1:ENST00000393238.3:exon6:c.C1753T:p.L585F,T [illegible]	[illegible]	
3	133534483	G	A	AGAGGTGAAA,ATGTGGCTGA	exonic	SRPRB	nonsynonymous SNV	SRPRB:ENST00000466490.2:exon6:c.G460A:p.D154N,	[illegible]	[illegible]
3	137850089	C	T	AGCTGGAGCT,CTTCCGCATG	exonic	A4GNT	nonsynonymous SNV	A4GNT:ENST00000236709.3:exon2:c.G10A:p.E4K,	[illegible]	
3	140785119	C	T	GCGTGGAACC,CGAGGACCGC	exonic	SPSB4	nonsynonymous SNV	SPSB4:ENST00000310546.2:exon2:c.C173T:p.P58L,	[illegible]	
3	142224040	C	T	CTCAGCAAGC,AAGGCTTTCA	exonic	ATR	nonsynonymous SNV	ATR:ENST00000383101.3:exon28:c.G4945A:p.G1649S,ATR:ENST00000350721.4:exon29:c.G5137A:p.G1713S,	[illegible]	
3	142742856	G	A	TATCCCAACA,AAAGGTACAT	exonic	U2SURP	nonsynonymous SNV	U2SURP:ENST00000463563.2:exon13:c.G1276A:p.E426K,U2SURP:ENST00000493598.2:exon13:c.G1267A:p.E4 [illegible]	[illegible]	
3	147128072	C	T	GAGCTGGCTT,GGCCGGCCAG	exonic	ZIC1	nonsynonymous SNV	ZIC1:ENST00000282928.4:exon1:c.C173T:p.S58L,	[illegible]	
3	148863279	C	T	TACCAGTATC,TGAAAAGTCT	exonic	HPS3	nonsynonymous SNV	HPS3:ENST00000296051.2:exon5:c.C1109T:p.P370L,HPS3:ENST00000460120.1:exon4:c.C614T:p.P205L,	[illegible]	
3	150916791	G	A	GGCAAATCCG,GTTCTTGTAT	exonic	GPR171	nonsynonymous SNV	GPR171:ENST00000309180.5:exon3:c.C383T:p.P128L,	[illegible]	
3	151094939	G	A	AGTAGCACGA,ACAGAATGGA	exonic	MED12L	nonsynonymous SNV	MED12L:ENST00000273432.4:exon26:c.G3764A:p.R1255K,MED12L:ENST00000474524.1:exon28:c.G4184A:p.R [illegible]	[illegible]	
3	151164159	G	A	TGTTGCCAGG,AATTTTCCTT	exonic	IGSF10	nonsynonymous SNV	IGSF10:ENST00000282466.3:exon4:c.C3610T:p.P1204S,	[illegible]	
3	156746100	G	A	GAAAACTTGA,GAAGGAAATG	exonic	LEKR1	nonsynonymous SNV	LEKR1:ENST00000356539.4:exon12:c.G1577A:p.R526K,LEKR1:ENST00000470811.1:exon13:c.G665A:p.R222K,	[illegible]	
3	161214845	C	T	CACTGCCACT,TCTCTCCCTT	exonic	OTOL1	nonsynonymous SNV	OTOL1:ENST00000327928.4:exon1:c.C250T:p.L84F,	[illegible]	
3	164906170	C	T	AGGGCCCACT,CTTCTCTTTT	exonic	SLITRK3	nonsynonymous SNV	SLITRK3:ENST00000475390.1:exon2:c.G2449A:p.E817K,SLITRK3:ENST00000241274.3:exon2:c.G2449A:p.E817 [illegible]	[illegible]	
3	167742343	C	T	TGGTACTGTT,ATCAACATTG	exonic	GOLIM4	nonsynonymous SNV	GOLIM4:ENST00000309027.4:exon13:c.G1747A:p.E583K,GOLIM4:ENST00000470487.1:exon14:c.G1831A:p.E61 [illegible]	[illegible]	
3	170802910	C	T	TGCTTACCTG,GGCTGATGAT	exonic	TNIK	nonsynonymous SNV	TNIK:ENST00000341852.6:exon23:c.G2743A:p.A915T,TNIK:ENST00000460047.1:exon23:c.G2806A:p.A936T,TN [illegible]	[illegible]	
3	178942540	T	C	GTGGTTGAAT,GGGAGAACCC	exonic	PIK3CA	nonsynonymous SNV	PIK3CA:ENST00000263967.3:exon16:c.T2347C:p.W783R,	[illegible]	
3	179069703	C	T	TATAAGAATC,GGAACTTGAT	exonic	MFN1	nonsynonymous SNV	MFN1:ENST00000471841.1:exon3:c.C128T:p.P43L,MFN1:ENST00000263969.5:exon2:c.C128T:p.P43L,MFN1:EN [illegible]	[illegible]	
3	183754243	G	A	CAATGTGGGT,GTCAGCATCT	exonic	HTR3D	stopgain	HTR3D:ENST00000334128.2:exon3:c.G56A:p.W19X,HTR3D:ENST00000382489.3:exon4:c.G461A:p.W154X,HTR [illegible]	[illegible]	
3	183960330	C	T	GTGTTGGGTG,TCTTGGGGAA	exonic	ALG3	nonsynonymous SNV	ALG3:ENST00000397676.3:exon9:c.G1289A:p.S430N,ALG3:ENST00000445626.2:exon9:c.G1145A:p.S382N,ALG [illegible]	[illegible]	
3	184039406	T	C	TCCAGTCCTC,CCAGGCTCCC	exonic	EIF4G1	nonsynonymous SNV	EIF4G1:ENST00000435046.2:exon3:c.T446C:p.L149P,EIF4G1:ENST00000444861.1:exon5:c.T542C:p.L181P,EIF4 [illegible]	[illegible]	
3	184073314	A	G	TCTTTCTGTG,GAGCTAGAGT	exonic	CLCN2	nonsynonymous SNV	CLCN2:ENST00000457512.1:exon12:c.T1174C:p.S392P,CLCN2:ENST00000423355.2:exon12:c.T97C:p.S33P,CL [illegible]	[illegible]	
3	190347251	G	A	AAGTGCCAAA,GCGAAGTTGC	exonic	IL1RAP	nonsynonymous SNV	IL1RAP:ENST00000443369.2:exon8:c.G1015A:p.G339S,IL1RAP:ENST00000434491.1:exon6:c.G592A:p.G198S,IL [illegible]	[illegible]	
3	196863429	C	A	AAGTTTATCT,CAATCTGAAG	exonic	DLG1	nonsynonymous SNV	DLG1:ENST00000447466.1:exon5:c.G530T:p.G177V,DLG1:ENST00000357674.4:exon10:c.G1004T:p.G335V,DLG [illegible]	[illegible]	

4	1164893	C	T	CGCCTGTTCC,GCCAACGGTC	exonic	SPON2	nonsynonymous SNV	SPON2:ENST00000290902.5:exon4:c.G530A:p.R177Q,SPON2:ENST00000431380.1:exon5:c.G530A:p.R177Q,	4p16.3	.
4	1643151	T	C	ACCGACTCCC,AGAGGCCCAC	exonic	FAM53A	nonsynonymous SNV	FAM53A:ENST00000461064.1:exon4:c.A1066G:p.R356G,FAM53A:ENST00000472884.2:exon5:c.A1066G:p.R356G,FAM53A:ENST00000308132.6:exon5:c.A1066G:p.R356G,	4p16.3	.
4	3449352	G	A	CAGCGACCAC,ACCTCGGTGA	exonic	HGFAC	nonsynonymous SNV	HGFAC:ENST00000511533.1:exon12:c.G1510A:p.D504N,HGFAC:ENST00000382774.3:exon11:c.G1489A:p.D497N,	4p16.3	.
4	13605878	G	C	TACTATCCAT,TTAGTGGAGT	exonic	BOD1L1	nonsynonymous SNV	BOD1L1:ENST00000040738.5:exon10:c.C2646G:p.N882K,	4p15.33	.
4	76584080	G	T	ACTCCACCAT,AACATAGGAA	exonic	G3BP2	nonsynonymous SNV	G3BP2:ENST00000511146.1:exon3:c.C124A:p.H42N,G3BP2:ENST00000507745.1:exon4:c.C124A:p.H42N,G3BP2:ENST00000509561.1:exon4:c.C124A:p.H42N,G3BP2:ENST00000508510.1:exon5:c.C124A:p.H42N,G3BP2:ENST00000357854.3:exon3:c.C124A:p.H42N,G3BP2:ENST00000515457.1:exon3:c.C124A:p.H42N,G	4q21.1	.
4	80246922	G	A	CTGGGGCCAG,AAAGGCCATG	exonic	NAA11	nonsynonymous SNV	NAA11:ENST00000286794.4:exon1:c.C110T:p.S37F,	4q21.21	.
4	80327898	C	T	TGGTTCAAAT,GTTCCATCCT	exonic	GK2	nonsynonymous SNV	GK2:ENST00000358842.3:exon1:c.G1457A:p.R486Q,	4q21.21	.
4	94751105	C	T	GAATTTTCCC,CCATTCCCAT	exonic	ATOH1	nonsynonymous SNV	ATOH1:ENST00000306011.3:exon1:c.C1028T:p.P343L,	4q22.2	.
4	106580268	C	T	GTCCTTATTC,CAGGGCCTCA	exonic	ARHGEF38	nonsynonymous SNV	ARHGEF38:ENST00000420470.2:exon10:c.C1291T:p.P431S,	4q24	.
4	128637526	C	T	TGTCTGTTTT,ATGACTCAGT	exonic	INTU	nonsynonymous SNV	INTU:ENST00000335251.6:exon16:c.C2764T:p.H922Y,	4q28.1	.
4	141583234	G	T	ATAGTACCCA,GATGTGTGGC	exonic	TBC1D9	nonsynonymous SNV	TBC1D9:ENST00000442267.2:exon10:c.C1616A:p.P539H,	4q31.21	.
4	153897234	C	T	CTCCCAGAAT,CCCCCAGCAG	exonic	FHDC1	nonsynonymous SNV	FHDC1:ENST00000260008.3:exon11:c.C2791T:p.P931S,FHDC1:ENST00000511601.1:exon12:c.C2791T:p.P931S,	4q31.3	.
4	153897235	C	T	TCCCAGAATC,CCCCAGCAGC	exonic	FHDC1	nonsynonymous SNV	FHDC1:ENST00000260008.3:exon11:c.C2792T:p.P931L,FHDC1:ENST00000511601.1:exon12:c.C2792T:p.P931L,	4q31.3	.
4	170322917	C	T	ACTGACTCTT,TTCTTCACTG	exonic	NEK1	nonsynonymous SNV	NEK1:ENST00000439128.2:exon31:c.G3385A:p.E1129K,NEK1:ENST00000512193.1:exon30:c.G3178A:p.E1060K,NEK1:ENST00000507142.1:exon33:c.G3469A:p.E1157K,NEK1:ENST00000510533.1:exon30:c.G3253A:p.E1085K,NEK1:ENST00000511633.1:exon32:c.G3337A:p.E1113K,	4q33	.
5	155080	C	T	TCCCTACAGC,ATGGTGACTG	exonic	PLEKHG4B	nonsynonymous SNV	PLEKHG4B:ENST00000502646.1:exon6:c.C757T:p.H253Y,PLEKHG4B:ENST00000283426.6:exon6:c.C1015T:p.H339Y,	5p15.33	.
5	1246038	C	T	GTCCTTGCTG,CCGTGCTGTG	exonic	SLC6A18	nonsynonymous SNV	SLC6A18:ENST00000324642.3:exon12:c.C1732T:p.P578S,	5p15.33	.
5	1282725	G	A	TCTGCGGCCG,AACACAGCCA	exonic	TERT	nonsynonymous SNV	TERT:ENST00000508104.2:exon3:c.C1588T:p.P530S,TERT:ENST00000334602.6:exon3:c.C1588T:p.P530S,TERT:ENST00000296820.5:exon3:c.C1588T:p.P530S,TERT:ENST00000460137.2:exon3:c.C1588T:p.P530S,TERT:ENST00000310581.5:exon3:c.C1588T:p.P530S,	5p15.33	.
5	6600322	G	A	TAAGACGATG,GACACTGCAG	exonic	NSUN2	nonsynonymous SNV	NSUN2:ENST00000506139.1:exon18:c.C1916T:p.P639L,NSUN2:ENST00000539938.1:exon18:c.C1313T:p.P438L,NSUN2:ENST00000264670.6:exon19:c.C2021T:p.P674L,	5p15.31	.
5	10354031	T	A	CGGAGGAAGG,AAGTCGGCGA	splicing	MARCH6(ENS	.	.	5p15.2	.
5	10681154	G	A	GGGGCAGGCT,GTGGACTCCA	exonic	DAP	stopgain	DAP:ENST00000432074.2:exon3:c.C280T:p.Q94X,	5p15.2	.
5	13824358	C	T	ATCGTGGACT,CGTATCCATT	exonic	DNAH5	nonsynonymous SNV	DNAH5:ENST00000265104.4:exon39:c.G6529A:p.E2177K,	5p15.2	.
5	14498257	G	T	GGAGACCGTT,TTCTTAGATG	exonic	TRIO	nonsynonymous SNV	TRIO:ENST00000344204.4:exon52:c.G8107T:p.V2703F,TRIO:ENST00000513206.1:exon48:c.G7306T:p.V2436F,TRIO:ENST00000344135.5:exon5:c.G604T:p.V202F,TRIO:ENST00000537187.1:exon53:c.G7579T:p.V2527F,	5p15.2	.
5	32089762	C	T	CCAACCCAGG,CGACTGGCGA	exonic	PDZD2	nonsynonymous SNV	PDZD2:ENST00000438447.1:exon20:c.C6208T:p.P2070S,PDZD2:ENST00000282493.3:exon19:c.C6208T:p.P2070S,	5p13.3	.
5	32420156	A	T	TGGACAGTGT,GCTAGCAACT	exonic	ZFR	nonsynonymous SNV	ZFR:ENST00000265069.8:exon3:c.T190A:p.Y64N,	5p13.3	.
5	33881453	G	T	CCAGTCCTCT,AGCCATCCAA	exonic	ADAMTS12	stopgain	ADAMTS12:ENST00000352040.3:exon2:c.C260A:p.S87X,ADAMTS12:ENST00000515401.1:exon2:c.C260A:p.S87X,ADAMTS12:ENST00000504830.1:exon2:c.C260A:p.S87X,	5p13.2	.
5	34823148	C	A	CCTGGGAAAA,CTGGTGAAAC	exonic	RAI14	nonsynonymous SNV	RAI14:ENST00000512629.1:exon14:c.C1114A:p.P372T,RAI14:ENST00000503673.1:exon16:c.C1201A:p.P401T,RAI14:ENST00000428746.2:exon15:c.C1201A:p.P401T,RAI14:ENST00000506376.1:exon14:c.C1177A:p.P393T,RAI14:ENST00000265109.3:exon15:c.C1201A:p.P401T,RAI14:ENST00000397449.1:exon14:c.C118	5p13.2	.
5	43280391	C	T	ATACAGTGTA,CGCCGCACGT	exonic	NIM1	nonsynonymous SNV	NIM1:ENST00000512796.1:exon4:c.C871T:p.P291S,NIM1:ENST00000326035.2:exon4:c.C871T:p.P291S,	5p12	.
5	71494635	C	T	CACAGTTCCT,TTCTCCACCA	exonic	MAP1B	nonsynonymous SNV	MAP1B:ENST00000296755.7:exon5:c.C5453T:p.S1818F,	5q13.2	.
5	71627170	G	A	GTTGGATCTC,AGGAATCTGC	exonic	PTCD2	nonsynonymous SNV	PTCD2:ENST00000380639.5:exon4:c.G436A:p.E146K,PTCD2:ENST00000543322.1:exon4:c.G436A:p.E146K,PTCD2:ENST00000515198.1:exon4:c.G436A:p.E146K,PTCD2:ENST00000308077.5:exon4:c.G436A:p.E146K,	5q13.2	.
5	74325249	G	A	ATTTAAATCA,CCTGGAGTCT	exonic	GCNT4	nonsynonymous SNV	GCNT4:ENST00000322348.4:exon1:c.C614T:p.A205V,	5q13.3	.
5	78944902	C	T	TCGAGTTCGT,CGTTAGTGCT	exonic	PAPD4	nonsynonymous SNV	PAPD4:ENST00000428308.2:exon12:c.C916T:p.P306S,PAPD4:ENST00000296783.3:exon11:c.C916T:p.P306S,PAPD4:ENST00000453514.1:exon10:c.C916T:p.P306S,PAPD4:ENST00000423041.2:exon11:c.C904T:p.P302S,	5q14.1	.
5	78944903	C	T	CGAGTTCGTC,GTTAGTGCTG	exonic	PAPD4	nonsynonymous SNV	PAPD4:ENST00000428308.2:exon12:c.C917T:p.P306L,PAPD4:ENST00000296783.3:exon11:c.C917T:p.P306L,PAPD4:ENST00000453514.1:exon10:c.C917T:p.P306L,PAPD4:ENST00000423041.2:exon11:c.C905T:p.P302L,	5q14.1	.
5	79029794	G	A	AGCTGAAGGA,GCAACCCAGA	exonic	CMYA5	nonsynonymous SNV	CMYA5:ENST00000446378.2:exon2:c.G5206A:p.G1736S,	5q14.1	.
5	80109473	T	C	ATAAAACAAG,TGCATTGATT	exonic	MSH3	nonsynonymous SNV	MSH3:ENST00000265081.6:exon20:c.T2726C:p.V909A,	5q14.1	.
5	82832882	G	A	ATCTAAAGAA,ATGAACCTTG	exonic	VCAN	nonsynonymous SNV	VCAN:ENST00000343200.5:exon7:c.G1099A:p.D367N,VCAN:ENST00000265077.3:exon8:c.G4060A:p.D1354N,VCAN:ENST00000513960.1:exon7:c.G1099A:p.D367N,	5q14.3	.
5	90074268	G	A	CATTCCCGAG,AAAAAAGCTT	exonic	GPR98	nonsynonymous SNV	GPR98:ENST00000405460.2:exon63:c.G12691A:p.E4231K,	5q14.3	.
5	102440503	T	C	GTTTCGGTGC,ACAATAACTG	exonic	GIN1	nonsynonymous SNV	GIN1:ENST00000512248.1:exon3:c.A187G:p.S63G,GIN1:ENST00000513747.1:exon3:c.A187G:p.S63G,	5q21.1	.
5	115298357	G	A	GAGCCGCGCA,TGGCCCTGCT	exonic	AQPEP	nonsynonymous SNV	AQPEP:ENST00000504467.1:exon1:c.G43A:p.V15M,AQPEP:ENST00000357872.4:exon1:c.G43A:p.V15M,	5q23.1	.
5	121187699	C	T	CACATCAGCC,TTCGCTGGCG	exonic	FTMT	nonsynonymous SNV	FTMT:ENST00000321339.1:exon1:c.C41T:p.P14L,	5q23.1	FTMT;basechange=c.41C>G;AAchange=p.P14R;tissue=lung
5	131647875	A	T	GTGTGAGGAC,ACTGGAAGGT	exonic	SLC22A4	nonsynonymous SNV	SLC22A4:ENST00000200652.3:exon2:c.A415T:p.N139Y,	5q31.1	.
5	132161796	G	A	GACGAGGCCG,GGAGGCGCGG	exonic	SHROOM1	nonsynonymous SNV	SHROOM1:ENST00000378679.3:exon4:c.C37T:p.P13S,SHROOM1:ENST00000378676.1:exon1:c.C37T:p.P13S,SHROOM1:ENST00000440118.1:exon2:c.C37T:p.P13S,SHROOM1:ENST00000319854.3:exon1:c.C37T:p.P13S,	5q31.1	.
5	132232718	G	A	CTTTTGGATG,TTTTGGAGTC	exonic	AFF4	nonsynonymous SNV	AFF4:ENST00000265343.5:exon11:c.C1604T:p.T535I,AFF4:ENST00000378595.3:exon11:c.C1604T:p.T535I,	5q31.1	.
5	133473877	G	A	GGATGGGGAG,GGCCCAGTGA	exonic	TCF7	nonsynonymous SNV	TCF7:ENST00000520652.1:exon2:c.G206A:p.G69E,	5q31.1	.
5	137089301	G	A	GGCCTTGTCT,CCGCGTCGTG	exonic	HNRNPA0	nonsynonymous SNV	HNRNPA0:ENST00000314940.4:exon1:c.C455T:p.A152V,	5q31.2	.
5	137454511	G	A	TCTTACCAAA,GATCTGCTGG	exonic	NME5	nonsynonymous SNV	NME5:ENST00000265191.2:exon5:c.C551T:p.P184L,	5q31.2	.
5	137454512	G	A	CTTACCAAAG,ATCTGCTGGT	exonic	NME5	nonsynonymous SNV	NME5:ENST00000265191.2:exon5:c.C550T:p.P184S,	5q31.2	.
5	137488275	C	T	GGTTCTCTCT,CGGGCTGCTT	exonic	BRD8	nonsynonymous SNV	BRD8:ENST00000427976.1:exon1:c.G70A:p.E24K,BRD8:ENST00000254900.5:exon21:c.G2752A:p.E918K,	5q31.2	.
5	138730307	G	C	GGACTGGCGT,GGACCGCGGC	exonic	PROB1	nonsynonymous SNV	PROB1:ENST00000434752.2:exon1:c.C464G:p.P155R,	5q31.2	.
5	140202762	C	T	GGAGAACAAC,CGCCAGGCTG	exonic	PCDHA5	nonsynonymous SNV	PCDHA5:ENST00000378126.3:exon1:c.C1402T:p.P468S,PCDHA5:ENST00000529859.1:exon1:c.C1402T:p.P468S,PCDHA5:ENST00000529619.1:exon1:c.C1402T:p.P468S,	5q31.3	.
5	140262130	G	A	GATCGACCGC,AGGAGCTGTG	exonic	PCDHA13	nonsynonymous SNV	PCDHA13:ENST00000289272.2:exon1:c.G277A:p.E93K,PCDHA13:ENST00000409494.1:exon1:c.G277A:p.E93K,	5q31.3	PCDHA13;basechange=c.277G>C;AAchange=p.E93Q;tissue=lung&PCDHA13;basechange=c.277G>T;AAchange=p.
5	140480510	G	A	ATTGGACCGG,AGGAGCTATG	exonic	PCDHB3	nonsynonymous SNV	PCDHB3:ENST00000231130.2:exon1:c.G277A:p.E93K,	5q31.3	.
5	140589668	G	A	AAAATCTTCG,TAAATAATTA	exonic	PCDHB12	nonsynonymous SNV	PCDHB12:ENST00000541609.1:exon2:c.G178A:p.V60I,PCDHB12:ENST00000239450.2:exon1:c.G1189A:p.V397I,	5q31.3	.
5	140741954	C	T	TTGCCCTATT,CTACAACCTG	exonic	PCDHGB2	nonsynonymous SNV	PCDHGB2:ENST00000522605.1:exon1:c.C2252T:p.S751F,	5q31.3	.
5	140764640	T	A	TCACGCCTGC,GCAGGCTTCA	exonic	PCDHGA7	nonsynonymous SNV	PCDHGA7:ENST00000518325.1:exon1:c.T2174A:p.L725Q,	5q31.3	.
5	140798702	G	A	CGCAGCCACA,ACAGGGGCAA	exonic	PCDHGB7	nonsynonymous SNV	PCDHGB7:ENST00000398594.2:exon1:c.G1276A:p.D426N,	5q31.3	.
5	140870921	C	T	AGTCTCTTAT,CCTAGTCACC	exonic	PCDHGC5	nonsynonymous SNV	PCDHGC5:ENST00000252087.1:exon1:c.C2114T:p.S705F,	5q31.3	.
5	141334712	C	T	GGCTGGTGGC,TCTGTGGGGC	exonic	PCDH12	nonsynonymous SNV	PCDH12:ENST00000231484.3:exon1:c.G2705A:p.R902K,	5q31.3	.
5	143853639	C	T	GAGAAAACAT,CTTGGCAATC	exonic	KCTD16	nonsynonymous SNV	KCTD16:ENST00000507359.3:exon3:c.C1249T:p.P417S,KCTD16:ENST00000512467.1:exon4:c.C1249T:p.P417S,	5q31.3	KCTD16;basechange=c.1249C>T;AAchange=p.P417S;tissue=lung
5	147807304	C	T	TCCGCCTTTT,CTTTAGGACT	exonic	FBXO38	nonsynonymous SNV	FBXO38:ENST00000340253.5:exon15:c.C2447T:p.S816F,	5q32	.
5	148998579	G	T	GAAGAACAAC,TGGCTGCTTA	exonic	ARHGEF37	nonsynonymous SNV	ARHGEF37:ENST00000333677.6:exon7:c.G868T:p.V290L,	5q32	.
5	149460362	C	A	GGCGCTGCCT,CCAGGGGGTC	exonic	CSF1R	nonsynonymous SNV	CSF1R:ENST00000543093.1:exon2:c.G275T:p.G92V,CSF1R:ENST00000504875.1:exon2:c.G275T:p.G92V,CSF1R:ENST00000286301.3:exon3:c.G275T:p.G92V,	5q32	.
5	150512697	T	C	AAGATGTAAA,GAACTGGGCT	exonic	ANXA6	nonsynonymous SNV	ANXA6:ENST00000354546.5:exon9:c.A598G:p.I200V,ANXA6:ENST00000356496.5:exon9:c.A598G:p.I200V,ANXA6:ENST00000523714.1:exon8:c.A502G:p.I168V,	5q33.1	.
5	150922698	G	A	CCTCCATCTT,GGCTTTGATG	exonic	FAT2	stopgain	FAT2:ENST00000261800.5:exon9:c.C7990T:p.Q2664X,	5q33.1	.
5	156890310	G	T	AAGGCCTCTT,CGACTCGTGG	exonic	NIPAL4	nonsynonymous SNV	NIPAL4:ENST00000435489.2:exon2:c.G432T:p.L144F,NIPAL4:ENST00000311946.7:exon2:c.G432T:p.L144F,NIPAL4:ENST00000519150.1:exon3:c.G344T:p.C115F,	5q33.3	.
5	160016614	C	T	TCTTCATTTC,ATTGCCTGGT	exonic	ATP10B	nonsynonymous SNV	ATP10B:ENST00000327245.5:exon24:c.G3735A:p.M1245I,	5q34	ATP10B;basechange=c.3735G>A;AAchange=p.M1245I;tissue=autonomic_ganglia
5	160721254	C	T	CGCCACATGT,GTTCCAGAGC	exonic	GABRB2	nonsynonymous SNV	GABRB2:ENST00000274547.2:exon11:c.G1373A:p.R458Q,GABRB2:ENST00000520240.1:exon10:c.G1259A:p.R420Q,GABRB2:ENST00000517901.1:exon9:c.G1070A:p.R357Q,GABRB2:ENST00000393959.1:exon10:c.G1373A:p.R458Q,GABRB2:ENST00000353437.6:exon10:c.G1259A:p.R420Q,GABRB2:ENST0000051754	5q34	.
5	161318012	C	T	TCACAAGTCT,CTTCTGGCTC	exonic	GABRA1	nonsynonymous SNV	GABRA1:ENST00000023897.6:exon9:c.C812T:p.S271F,GABRA1:ENST00000444819.1:exon8:c.C812T:p.S271F,GABRA1:ENST00000420560.1:exon8:c.C812T:p.S271F,GABRA1:ENST00000437025.2:exon8:c.C812T:p.S271F,GABRA1:ENST00000428797.2:exon9:c.C812T:p.S271F,GABRA1:ENST00000393943.4:exon8:c.C8	5q34	.
5	167643884	C	A	GACCTCACTG,CGTCCGGCCG	exonic	TENM2	nonsynonymous SNV	TENM2:ENST00000520394.1:exon18:c.C3473A:p.A1158D,TENM2:ENST00000403607.2:exon20:c.C3662A:p.A1221D,TENM2:ENST00000519204.1:exon21:c.C3827A:p.A1276D,TENM2:ENST00000545108.1:exon25:c.C4187A:p.A1396D,TENM2:ENST00000518659.1:exon22:c.C4190A:p.A1397D,	5q34	.
5	169111249	C	T	TCCTCATCCC,CACCCATAGC	exonic	DOCK2	nonsynonymous SNV	DOCK2:ENST00000524185.1:exon8:c.C656T:p.P219L,DOCK2:ENST00000256935.8:exon8:c.C656T:p.P219L,	5q35.1	DOCK2;basechange=c.656C>T;AAchange=p.P219L;tissue=endometrium
5	173493229	C	T	AGCACAGGCC,AGTGAAAGTG	exonic	NSG2	nonsynonymous SNV	NSG2:ENST00000517587.1:exon4:c.C236T:p.P79L,	5q35.2	.
5	176471462	G	A	CCCTGTCGTG,CCCAGTCGCA	exonic	ZNF346	nonsynonymous SNV	ZNF346:ENST00000503039.1:exon5:c.G520A:p.A174T,ZNF346:ENST00000261948.4:exon5:c.G520A:p.A174T,ZNF346:ENST00000503425.1:exon3:c.G349A:p.A117T,ZNF346:ENST00000511834.1:exon4:c.G445A:p.A149T,ZNF346:ENST00000358149.3:exon4:c.G445A:p.A149T,	5q35.2	.
6	4069103	G	T	TGATTTTCGG,AAAAGTCAGA	exonic	FAM217A	nonsynonymous SNV	FAM217A:ENST00000274673.3:exon7:c.C1354A:p.P452T,	6p25.2	.
6	4131030	C	T	GCATTCCATG,GTCCCATTTG	exonic	ECI2	nonsynonymous SNV	ECI2:ENST00000361538.2:exon3:c.G193A:p.A65T,ECI2:ENST00000380125.2:exon3:c.G193A:p.A65T,ECI2:ENST00000496241.2:exon3:c.G193A:p.A65T,ECI2:ENST00000495548.1:exon3:c.G424A:p.A142T,ECI2:ENST00000380118.3:exon3:c.G283A:p.A95T,ECI2:ENST00000465828.1:exon3:c.G193A:p.A65T,ECI2:ENST000	6p25.2	.
6	5260952	A	T	CTTGTGCGCG,CTGGAGGCTG	exonic	LYRM4	nonsynonymous SNV	LYRM4:ENST00000330636.4:exon1:c.T15A:p.S5R,LYRM4:ENST00000480566.1:exon1:c.T15A:p.S5R,LYRM4:ENST00000500576.2:exon1:c.T15A:p.S5R,LYRM4:ENST00000464010.1:exon1:c.T15A:p.S5R,LYRM4:ENST00000468929.1:exon1:c.T15A:p.S5R,	6p25.1	.
6	26459790	C	T	CATCTGTCAC,CGAGAAAAAT	exonic	BTN2A1	nonsynonymous SNV	BTN2A1:ENST00000312541.5:exon3:c.C164T:p.P55L,BTN2A1:ENST00000429381.1:exon3:c.C164T:p.P55L,BTN2A1:ENST00000469185.1:exon3:c.C164T:p.P55L,BTN2A1:ENST00000377600.2:exon3:c.C164T:p.P55L,	6p22.2	.
6	28294395	G	A	ATTCTCTGGT,CTCAATGAGT	exonic	ZSCAN31	nonsynonymous SNV	ZSCAN31:ENST00000396838.2:exon8:c.C769T:p.H257Y,ZSCAN31:ENST00000435857.1:exon3:c.C292T:p.H98Y,ZSCAN31:ENST00000439158.1:exon4:c.C769T:p.H257Y,ZSCAN31:ENST00000414431.1:exon4:c.C292T:p.H98Y,ZSCAN31:ENST00000344279.6:exon4:c.C769T:p.H257Y,ZSCAN31:ENST00000414429.1:exon8:	6p22.1	.
6	28297112	G	C	TCTTGTTCCA,ATCTTCAACT	exonic	ZSCAN31	nonsynonymous SNV	ZSCAN31:ENST00000396838.2:exon6:c.C349G:p.L117V,ZSCAN31:ENST00000439158.1:exon2:c.C349G:p.L117V,ZSCAN31:ENST00000447021.1:exon2:c.C349G:p.L117V,ZSCAN31:ENST00000344279.6:exon2:c.C349G:p.L117V,ZSCAN31:ENST00000414429.1:exon6:c.C349G:p.L117V,ZSCAN31:ENST00000439636.1:exo	6p22.1	.
6	30553113	C	T	TGTCATCTGG,TTAATAAGTG	exonic	ABCF1	nonsynonymous SNV	ABCF1:ENST00000326195.8:exon15:c.C1468T:p.L490F,ABCF1:ENST00000376545.3:exon14:c.C1354T:p.L452F,	6p21.33	.
6	31113494	G	A	TGGACCTTGC,GACAGCATAG	exonic	CCHCR1	nonsynonymous SNV	CCHCR1:ENST00000396268.3:exon11:c.C1672T:p.R558C,CCHCR1:ENST00000451521.2:exon11:c.C1564T:p.R522C,CCHCR1:ENST00000376266.5:exon11:c.C1405T:p.R469C,	6p21.33	CCHCR1;basechange=c.1405C>T;AAchange=p.R469C;tissue=endometrium&CCHCR1_ENST00000396268;basecha
6	31129599	C	T	CTGCCTGTTC,CGCCCCACCT	exonic	TCF19	nonsynonymous SNV	TCF19:ENST00000376255.4:exon3:c.C614T:p.P205L,TCF19:ENST00000542218.1:exon1:c.C374T:p.P125L,TCF19:ENST00000376257.3:exon3:c.C614T:p.P205L,	6p21.33	.
6	31852265	G	A	GGAGCGCTCG,GAGTCAGGTC	exonic	EHMT2	nonsynonymous SNV	EHMT2:ENST00000395728.3:exon20:c.C2846T:p.P949L,EHMT2:ENST00000375537.4:exon21:c.C2675T:p.P892L,EHMT2:ENST00000375530.4:exon20:c.C2573T:p.P858L,EHMT2:ENST00000375528.4:exon19:c.C2744T:p.P915L,	6p21.33	.
6	32189093	C	G	CCGAAGCTGG,ACTGCTCACC	exonic	NOTCH4	nonsynonymous SNV	NOTCH4:ENST00000375023.3:exon4:c.G461C:p.C154S,	6p21.32	NOTCH4;basechange=c.461G>A;AAchange=p.C154Y;tissue=lung
6	32362757	C	T	CTGCATTTCT,CATCTTCTTG	exonic	BTNL2	nonsynonymous SNV	BTNL2:ENST00000414363.1:exon4:c.G494A:p.G165E,BTNL2:ENST00000544175.1:exon5:c.G293A:p.G98E,BTNL2:ENST00000374995.3:exon6:c.G842A:p.G281E,BTNL2:ENST00000374993.1:exon6:c.G1124A:p.G375E,BTNL2:ENST00000540315.1:exon4:c.G494A:p.G165E,BTNL2:ENST00000454136.3:exon6:c.G1124A:p.G	6p21.32	.
6	32489735	G	T	TCTGCAGTAG,TGTCCACCGC	exonic	HLA-DRB5	nonsynonymous SNV	HLA-DRB5:ENST00000374975.3:exon2:c.C317A:p.T106N,	6p21.32	.
6	32609806	C	T	CAGCCCAACA,CCTCATTTGT	exonic	HLA-DQA1	nonsynonymous SNV	HLA-DQA1:ENST00000343139.5:exon3:c.C389T:p.T130I,HLA-DQA1:ENST00000374949.2:exon3:c.C389T:p.T130I,HLA-DQA1:ENST00000395363.1:exon3:c.C389T:p.T130I,HLA-DQA1:ENST00000496318.1:exon3:c.C389T:p.T130I,	6p21.32	.
6	33134541	C	T	CCAGGAAGTC,CCGATCTCCC	exonic	COL11A2	nonsynonymous SNV	COL11A2:ENST00000374713.1:exon57:c.G4153A:p.G1385R,COL11A2:ENST00000395197.1:exon58:c.G4114A:p.G1372R,COL11A2:ENST00000374712.1:exon57:c.G4051A:p.G1351R,COL11A2:ENST00000341947.2:exon59:c.G4294A:p.G1432R,COL11A2:ENST00000374708.4:exon57:c.G4036A:p.G1346R,COL11A2:ENST(	6p21.32	.
6	34100964	G	T	TCCAGAATGC,GGCGCCCAGC	exonic	GRM4	nonsynonymous SNV	GRM4:ENST00000538487.2:exon2:c.C310A:p.R104S,GRM4:ENST00000374181.4:exon1:c.C310A:p.R104S,GRM4:ENST00000609278.1:exon2:c.C310A:p.R104S,	6p21.31	.
6	39507895	G	A	TAGGCGGAAG,GTGGGCTCTG	exonic	KIF6	nonsynonymous SNV	KIF6:ENST00000287152.7:exon13:c.C1529T:p.P510L,KIF6:ENST00000373216.3:exon13:c.C1529T:p.P510L,KIF6:ENST00000373215.3:exon13:c.C1529T:p.P510L,KIF6:ENST00000373213.4:exon9:c.C1046T:p.P349L,	6p21.2	.
6	41738514	G	A	GGGCATGGGG,CTTGGGGGCT	exonic	FRS3	nonsynonymous SNV	FRS3:ENST00000373018.3:exon7:c.C1322T:p.A441V,FRS3:ENST00000259748.2:exon6:c.C1322T:p.A441V,	6p21.1	.
6	42893169	G	A	ACTGCACCCG,CCACGGAGAC	exonic	PTCRA	nonsynonymous SNV	PTCRA:ENST00000304672.1:exon4:c.G595A:p.A199T,PTCRA:ENST00000446507.1:exon3:c.G274A:p.A92T,PTCRA:ENST00000441198.1:exon5:c.G520A:p.A174T,	6p21.1	.
6	43013011	G	A	GCCATGTCCT,GCTCCAGGCC	exonic	CUL7	stopgain	CUL7:ENST00000535468.1:exon15:c.C3244T:p.Q1082X,CUL7:ENST00000265348.3:exon15:c.C2992T:p.Q998X,	6p21.1	.
6	47846964	C	T	ACAGAGTCTT,TTAGGTCATC	exonic	PTCHD4	nonsynonymous SNV	PTCHD4:ENST00000339488.4:exon3:c.G1616A:p.R539K,	6p12.3	.
6	49580127	C	A	AACTTGTATC,AAGCACAGAG	exonic	RHAG	stopgain	RHAG:ENST00000229810.7:exon6:c.G928T:p.G310X,RHAG:ENST00000371175.4:exon6:c.G928T:p.G310X,	6p12.3	.
6	50810932	G	A	CATCACGCAC,GCTTCGGCGC	exonic	TFAP2B	nonsynonymous SNV	TFAP2B:ENST00000263046.4:exon8:c.G1237A:p.G413S,TFAP2B:ENST00000393655.3:exon7:c.G1210A:p.G404S,	6p12.3	.
6	64421500	C	T	CTGCATAGAC,TAATCTATTG	exonic	PHF3	nonsynonymous SNV	PHF3:ENST00000262043.3:exon16:c.C4016T:p.P1339L,PHF3:ENST00000515594.1:exon14:c.C1823T:p.P608L,PHF3:ENST00000393387.1:exon15:c.C4016T:p.P1339L,	6q12	.
6	85457745	C	T	AGATCGTCTC,ACAGTCTTTA	exonic	TBX18	nonsynonymous SNV	TBX18:ENST00000606784.1:exon5:c.G358A:p.G120R,TBX18:ENST00000369663.5:exon5:c.G832A:p.G278R,TBX18:ENST00000606325.1:exon5:c.G358A:p.G120R,	6q14.3	.
6	87725678	C	T	TACCACGCGG,CAAGAGCCTT	exonic	HTR1E	nonsynonymous SNV	HTR1E:ENST00000305344.5:exon2:c.C626T:p.A209V,	6q14.3	.
6	106968885	A	G	CACTATGACC,CGGCTTTCAG	exonic	AIM1	nonsynonymous SNV	AIM1:ENST00000369066.3:exon2:c.A2578G:p.T860A,	6q21	.
6	108041918	C	T	ACATCTGTTT,CTTCAAGAGA	exonic	SCML4	nonsynonymous SNV	SCML4:ENST00000369020.3:exon6:c.G962A:p.G321E,SCML4:ENST00000369021.3:exon5:c.G875A:p.G292E,SCML4:ENST00000369025.2:exon2:c.G236A:p.G79E,SCML4:ENST00000369022.2:exon5:c.G788A:p.G263E,	6q21	.
6	108093509	C	T	TCGCTTTCTC,CCGGGATCCT	exonic	SCML4	nonsynonymous SNV	SCML4:ENST00000369020.3:exon2:c.G23A:p.G8E,	6q21	.
6	116264279	C	T	CTACGAATGG,TTCGGGCGCA	exonic	FRK	nonsynonymous SNV	FRK:ENST00000538210.1:exon6:c.G784A:p.A262T,FRK:ENST00000606080.1:exon7:c.G1210A:p.A404T,	6q22.1	.
6	117128234	C	T	GTTATGATGC,AATCCAGTTC	exonic	GPRC6A	nonsynonymous SNV	GPRC6A:ENST00000530250.1:exon3:c.G634A:p.G212S,GPRC6A:ENST00000310357.3:exon3:c.G634A:p.G212S,GPRC6A:ENST00000368549.3:exon3:c.G634A:p.G212S,	6q22.1	.
6	126243944	T	G	CACTAGACAG,CCTGTCCTAT	exonic	NCOA7	nonsynonymous SNV	NCOA7:ENST00000392477.2:exon13:c.T2487G:p.S829R,NCOA7:ENST00000368357.3:exon14:c.T2487G:p.S829R,NCOA7:ENST00000229634.9:exon12:c.T2142G:p.S714R,NCOA7:ENST00000433571.1:exon4:c.T417G:p.S139R,	6q22.32	.
6	133783819	C	T	ACTCAGTCCC,ATTACAGAGT	exonic	EYA4	nonsynonymous SNV	EYA4:ENST00000367895.5:exon9:c.C641T:p.P214L,EYA4:ENST00000355167.3:exon9:c.C641T:p.P214L,EYA4:ENST00000430974.2:exon7:c.C479T:p.P160L,EYA4:ENST00000452339.2:exon8:c.C479T:p.P160L,EYA4:ENST00000525849.1:exon8:c.C572T:p.P191L,EYA4:ENST00000431403.2:exon8:c.C641T:p.P214L,EYA4:E	6q23.2	.
6	150039043	G	A	GGACGCTGAG,CGGCCAGAGT	exonic	LATS1	nonsynonymous SNV	LATS1:ENST00000458696.2:exon1:c.C128T:p.A43V,	6q25.1	.
6	158516755	C	T	CCCTCGAGTC,CTCCTGTTCC	exonic	SYNJ2	nonsynonymous SNV	SYNJ2:ENST00000355585.4:exon27:c.C3850T:p.P1284S,SYNJ2:ENST00000367122.2:exon26:c.C3715T:p.P1239S,SYNJ2:ENST00000367112.1:exon10:c.C1105T:p.P369S,	6q25.3	.
6	158516756	C	T	CCTCGAGTCC,TCCTGTTCCC	exonic	SYNJ2	nonsynonymous SNV	SYNJ2:ENST00000355585.4:exon27:c.C3851T:p.P1284L,SYNJ2:ENST00000367122.2:exon26:c.C3716T:p.P1239L,SYNJ2:ENST00000367112.1:exon10:c.C1106T:p.P369L,	6q25.3	.
6	159457715	G	A	CACCGAACCC,GGGCCAAGTT	exonic	TAGAP	nonsynonymous SNV	TAGAP:ENST00000326965.6:exon9:c.C806T:p.P269L,TAGAP:ENST00000367066.3:exon10:c.C1340T:p.P447L,	6q25.3	.
7	1477834	G	A	TATCTCCTCC,GGGAGAGGTA	exonic	MICALL2	nonsynonymous SNV	MICALL2:ENST00000297508.7:exon12:c.C2210T:p.P737L,MICALL2:ENST00000405088.4:exon8:c.C1574T:p.P525L,	7p22.3	.
7	1527080	G	A	CCTCCGGGGG,GCCCCCTGAG	exonic	INTS1	nonsynonymous SNV	INTS1:ENST00000404767.3:exon20:c.C2522T:p.P841L,INTS1:ENST00000389470.4:exon21:c.C2951T:p.P984L,	7p22.3	.
7	5352880	G	A	TGCTCAGCCT,GGCCTTGTCT	exonic	TNRC18	stopgain	TNRC18:ENST00000430969.1:exon27:c.C7642T:p.Q2548X,TNRC18:ENST00000399537.4:exon27:c.C7642T:p.Q2548X,	7p22.1	.
7	6196494	G	T	AGAGGACTTT,TTAAAGATTC	exonic	USP42	nonsynonymous SNV	USP42:ENST00000306177.5:exon16:c.G3751T:p.V1251F,	7p22.1	.
7	6662015	G	A	GGGCCCGGCA,GCAGCGCGGC	exonic	ZNF853	nonsynonymous SNV	ZNF853:ENST00000457543.3:exon3:c.G1393A:p.G465S,	7p22.1	.
7	34125662	T	G	TCCTGGGAGT,TCAGACCTGC	exonic	BMPER	nonsynonymous SNV	BMPER:ENST00000426693.1:exon13:c.T1703G:p.F568C,BMPER:ENST00000297161.2:exon14:c.T1703G:p.F568C,	7p14.3	.
7	39247079	C	T	ATCCTCATTC,CTTCAACATG	exonic	POU6F2	nonsynonymous SNV	POU6F2:ENST00000520104.1:exon4:c.C347T:p.P116L,POU6F2:ENST00000524147.1:exon4:c.C347T:p.P116L,POU6F2:ENST00000451021.1:exon4:c.C374T:p.P125L,POU6F2:ENST00000403058.1:exon5:c.C371T:p.P124L,POU6F2:ENST00000518318.2:exon4:c.C371T:p.P124L,POU6F2:ENST00000559001.1:exon3:c.C347	7p14.1	.
7	48313301	T	G	TGTTTTCCTG,GCTGATATTT	exonic	ABCA13	nonsynonymous SNV	ABCA13:ENST00000435803.1:exon17:c.T4038G:p.C1346W,	7p12.3	.
7	50467675	G	A	GAAGGAGAAC,AAATGATGAA	exonic	IKZF1	nonsynonymous SNV	IKZF1:ENST00000359197.5:exon7:c.G784A:p.E262K,IKZF1:ENST00000426121.1:exon2:c.G169A:p.E57K,IKZF1:ENST00000439701.1:exon7:c.G784A:p.E262K,IKZF1:ENST00000357364.4:exon6:c.G649A:p.E217K,IKZF1:ENST00000349824.4:exon5:c.G481A:p.E161K,IKZF1:ENST00000346667.4:exon4:c.G220A:p.E74K,IKZF	7p12.2	.
7	86415968	C	T	CTCATTGCAG,CGCCAGCCGC	exonic	GRM3	nonsynonymous SNV	GRM3:ENST00000454217.1:exon2:c.C476T:p.A159V,GRM3:ENST00000439827.1:exon3:c.C860T:p.A287V,GRM3:ENST00000394720.2:exon3:c.C854T:p.A285V,GRM3:ENST00000361669.2:exon3:c.C860T:p.A287V,GRM3:ENST00000536043.1:exon2:c.C476T:p.A159V,	7q21.12	.
7	87179570	C	T	ACCAGGGCCA,CGTCTGCCCA	exonic	ABCB1	nonsynonymous SNV	ABCB1:ENST00000265724.3:exon13:c.G1267A:p.V423M,ABCB1:ENST00000543898.1:exon10:c.G1075A:p.V359M,	7q21.12	.
7	87280158	T	A	AAGACCCTGA,TGATCGGTCT	exonic	RUNDC3B	nonsynonymous SNV	RUNDC3B:ENST00000394654.3:exon2:c.T143A:p.I48N,RUNDC3B:ENST00000338056.3:exon2:c.T143A:p.I48N,RUNDC3B:ENST00000493037.1:exon2:c.T143A:p.I48N,	7q21.12	.
7	87912102	G	A	AGCCAGTCTG,GAATCGACGG	exonic	STEAP4	nonsynonymous SNV	STEAP4:ENST00000380079.4:exon3:c.C838T:p.P280S,STEAP4:ENST00000414498.1:exon4:c.C838T:p.P280S,	7q21.12	.
7	94049739	G	A	TGGCCCCAAT,GATTTGCTGG	exonic	COL1A2	nonsynonymous SNV	COL1A2:ENST00000297268.6:exon36:c.G2170A:p.G724R,	7q21.3	.
7	99217399	A	T	GCTGGACCCC,GGAAGCTCTT	exonic	ZSCAN25	nonsynonymous SNV	ZSCAN25:ENST00000431485.2:exon5:c.A170T:p.Q57L,ZSCAN25:ENST00000394152.2:exon4:c.A170T:p.Q57L,ZSCAN25:ENST00000262941.6:exon1:c.A170T:p.Q57L,ZSCAN25:ENST00000394150.1:exon4:c.A170T:p.Q57L,ZSCAN25:ENST00000334715.3:exon1:c.A170T:p.Q57L,	7q22.1	ZNF498;basechange=c.170A>T;AAchange=p.Q57L;tissue=liver
7	99971282	G	A	ACAAGGCCAT,GGTCGGCCCC	exonic	PILRA	nonsynonymous SNV	PILRA:ENST00000394000.2:exon1:c.G3A:p.M1I,PILRA:ENST00000432297.2:exon2:c.G3A:p.M1I,PILRA:ENST00000198536.2:exon1:c.G3A:p.M1I,PILRA:ENST00000453419.1:exon1:c.G3A:p.M1I,PILRA:ENST00000350573.2:exon1:c.G3A:p.M1I,	7q22.1	.
7	100401195	C	T	CAGAGTGACT,CGATTCGGAG	exonic	EPHB4	nonsynonymous SNV	EPHB4:ENST00000360620.3:exon16:c.G2696A:p.G899E,EPHB4:ENST00000358173.3:exon17:c.G2852A:p.G951E,	7q22.1	.
7	100624667	G	A	AAGAACCTGG,CTTGGTGGAG	exonic	MUC12	nonsynonymous SNV	MUC12:ENST00000379442.3:exon4:c.G329A:p.G110D,	7q22.1	.
7	100684865	C	A	TACCAGCATA,CAACCTCAAG	exonic	MUC17	nonsynonymous SNV	MUC17:ENST00000306151.4:exon3:c.C10168A:p.P3390T,MUC17:ENST00000379439.3:exon3:c.C10168A:p.P3390T,	7q22.1	.
7	107635339	G	C	TACCTTGAAA,TCATTATGAG	exonic	LAMB1	nonsynonymous SNV	LAMB1:ENST00000222399.6:exon5:c.C416G:p.T139S,LAMB1:ENST00000393560.1:exon5:c.C416G:p.T139S,LAMB1:ENST00000393561.1:exon3:c.C488G:p.T163S,	7q31.1	.
7	117227857	G	A	ACACTGAGTG,AGGTCAACGA	exonic	CFTR	nonsynonymous SNV	CFTR:ENST00000426809.1:exon11:c.G1559A:p.G520E,CFTR:ENST00000454343.1:exon11:c.G1466A:p.G489E,CFTR:ENST00000003084.6:exon12:c.G1649A:p.G550E,	7q31.2	.
7	127724841	G	A	CCCTGTAGAG,GCTCCTATGC	exonic	SND1	nonsynonymous SNV	SND1:ENST00000354725.3:exon19:c.G2176A:p.G726S,SND1:ENST00000486037.1:exon6:c.G634A:p.G212S,	7q32.1	.
7	129825187	G	A	CTGCTGGAAG,AGAGGTAGAA	exonic	TMEM209	nonsynonymous SNV	TMEM209:ENST00000336804.8:exon7:c.C793T:p.P265S,TMEM209:ENST00000462753.1:exon7:c.C793T:p.P265S,TMEM209:ENST00000473456.1:exon7:c.C796T:p.P266S,TMEM209:ENST00000397622.2:exon7:c.C796T:p.P266S,	7q32.2	.
7	130021651	C	T	CTTCCGGTCC,GGGCGCGCTC	exonic	CPA1	nonsynonymous SNV	CPA1:ENST00000481342.1:exon3:c.C64T:p.R22W,CPA1:ENST00000484324.1:exon2:c.C64T:p.R22W,CPA1:ENST00000011292.3:exon3:c.C328T:p.R110W,CPA1:ENST00000476062.1:exon3:c.C64T:p.R22W,	7q32.2	.
7	130024417	C	T	ACAGCAGGCT,CCTCTGTATT	exonic	CPA1	nonsynonymous SNV	CPA1:ENST00000604896.1:exon4:c.C293T:p.S98F,CPA1:ENST00000484324.1:exon6:c.C473T:p.S158F,CPA1:ENST00000011292.3:exon7:c.C737T:p.S246F,CPA1:ENST00000476062.1:exon7:c.C473T:p.S158F,	7q32.2	.
7	141536203	C	T	TCTCACTTGT,CTTAGCAGTG	exonic	PRSS37	nonsynonymous SNV	PRSS37:ENST00000438520.1:exon6:c.G700A:p.D234N,PRSS37:ENST00000350549.3:exon5:c.G700A:p.D234N,	7q34	.
7	141734592	G	C	GATGACCTGA,ATGGTCCATC	exonic	MGAM	nonsynonymous SNV	MGAM:ENST00000549489.2:exon16:c.G1910C:p.R637T,MGAM:ENST00000475668.2:exon16:c.G1910C:p.R637T,	7q34	.
7	144077003	G	A	CTGGCTGGAG,GCGTGAGGCT	exonic	ARHGEF5	nonsynonymous SNV	ARHGEF5:ENST00000056217.5:exon15:c.G4648A:p.G1550S,ARHGEF5:ENST00000471847.2:exon13:c.G1414A:p.G472S,	7q35	.
7	144094533	G	A	AATAGATCAG,AAAGTAGCCA	exonic	NOBOX	nonsynonymous SNV	NOBOX:ENST00000223140.5:exon8:c.C1525T:p.P509S,NOBOX:ENST00000467773.1:exon10:c.C1876T:p.P626S,NOBOX:ENST00000483238.1:exon10:c.C1780T:p.P594S,	7q35	.
7	148767516	C	A	CACTCTGCCT,AACTCCAATC	exonic	ZNF786	stoploss	ZNF786:ENST00000491431.1:exon4:c.G2348T:p.X783L,ZNF786:ENST00000451334.3:exon5:c.G2237T:p.X746L,ZNF786:ENST00000316286.9:exon3:c.G2090T:p.X697L,	7q36.1	.
7	150034168	C	T	CCGCTGGCCT,CTTGCGCCAG	exonic	LRRC61	nonsynonymous SNV	LRRC61:ENST00000493307.1:exon4:c.C218T:p.S73F,LRRC61:ENST00000323078.7:exon2:c.C218T:p.S73F,LRRC61:ENST00000359623.4:exon3:c.C218T:p.S73F,	7q36.1	.
7	151072950	C	T	CTCAGCAATC,TCAGATGTGG	exonic	NUB1	nonsynonymous SNV	NUB1:ENST00000568733.1:exon13:c.C1484T:p.P495L,NUB1:ENST00000566856.1:exon13:c.C1370T:p.P457L,NUB1:ENST00000355851.4:exon13:c.C1412T:p.P471L,NUB1:ENST00000413040.2:exon13:c.C1442T:p.P481L,	7q36.1	.
8	2815317	C	T	CTGAAAAAAA,CGTGCTTCCA	exonic	CSMD1	nonsynonymous SNV	CSMD1:ENST00000520002.1:exon64:c.G9718A:p.V3240I,CSMD1:ENST00000602557.1:exon64:c.G9718A:p.V3240I,CSMD1:ENST00000537824.1:exon63:c.G9715A:p.V3239I,CSMD1:ENST00000400186.3:exon61:c.G9187A:p.V3063I,CSMD1:ENST00000542608.1:exon60:c.G9184A:p.V3062I,CSMD1:ENST00000602723.1:ex	8p23.2	CSMD1_ENST00000318252;basechange=c.8902G>T;AAchange=p.V2968F;tissue=large_intestine&CSMD1_ENST0(
8	10464767	G	A	CCTGGGGAAG,GGGTGGAGTG	exonic	RP1L1	nonsynonymous SNV	RP1L1:ENST00000382483.3:exon4:c.C6841T:p.P2281S,	8p23.1	.
8	17804704	C	T	GGCCAGAGAT,CTCAGCAGGA	exonic	PCM1	nonsynonymous SNV	PCM1:ENST00000523055.1:exon6:c.C793T:p.P265S,PCM1:ENST00000519253.1:exon7:c.C793T:p.P265S,PCM1:ENST00000517730.1:exon8:c.C910T:p.P304S,PCM1:ENST00000518537.1:exon8:c.C910T:p.P304S,PCM1:ENST00000325083.8:exon7:c.C793T:p.P265S,PCM1:ENST00000524226.1:exon6:c.C793T:p.P265S,	8p22	.
8	23082429	C	T	TCGGCCCCCG,CTCGTGGTTC	exonic	TNFRSF10A	nonsynonymous SNV	TNFRSF10A:ENST00000221132.3:exon1:c.G146A:p.G49D,	8p21.3	.
8	24209499	G	A	CTTCTTTTCA,ATGAGTCAGA	splicing	ADAM28(ENS	.	.	8p21.2	.
8	24774774	T	A	AAGTCAGAAA,GGAAGAGGCC	exonic	NEFM	nonsynonymous SNV	NEFM:ENST00000221166.5:exon3:c.T1406A:p.M469K,NEFM:ENST00000433454.2:exon3:c.T278A:p.M93K,NEFM:ENST00000437366.2:exon3:c.T1406A:p.M469K,NEFM:ENST00000518131.1:exon3:c.T1406A:p.M469K,	8p21.2	.
8	27303365	C	T	TATGGAGTAT,CATCTCCCGT	exonic	PTK2B	nonsynonymous SNV	PTK2B:ENST00000346049.5:exon24:c.C2269T:p.P757S,PTK2B:ENST00000544172.1:exon28:c.C2269T:p.P757S,PTK2B:ENST00000397501.1:exon29:c.C2269T:p.P757S,	8p21.2	.
8	37691576	G	C	AAGGCCTGCA,CCGCATCGTG	exonic	GPR124	nonsynonymous SNV	GPR124:ENST00000315215.7:exon11:c.G1538C:p.S513T,GPR124:ENST00000412232.2:exon11:c.G1538C:p.S513T,	8p11.23	.
8	37693148	T	C	GCTCCCCCGG,GCCCCCAGAC	exonic	GPR124	nonsynonymous SNV	GPR124:ENST00000412232.2:exon13:c.T1910C:p.V637A,	8p11.23	.
8	48625345	C	T	TATGTGGCCC,CTTGTGTGTG	exonic	SPIDR	nonsynonymous SNV	SPIDR:ENST00000518074.1:exon15:c.C1919T:p.P640L,SPIDR:ENST00000541342.1:exon14:c.C1889T:p.P630L,SPIDR:ENST00000297423.4:exon15:c.C2099T:p.P700L,SPIDR:ENST00000517693.1:exon8:c.C524T:p.P175L,	8q11.21	.
8	56879417	G	A	GGTCACCAGG,AGGAGCCCAT	exonic	LYN	nonsynonymous SNV	LYN:ENST00000520220.2:exon9:c.G871A:p.E291K,LYN:ENST00000519728.1:exon9:c.G934A:p.E312K,	8q12.1	.
8	61749528	G	A	GCTGATACCT,CATCATCTTT	exonic	CHD7	nonsynonymous SNV	CHD7:ENST00000423902.2:exon17:c.G4142A:p.C1381Y,	8q12.2	CHD7_ENST00000423902;basechange=c.4142G>T;AAchange=p.C1381F;tissue=ovary&CHD7;basechange=c.4142(
8	67047308	C	T	TGCGAAGTAC,CACCTGCTCT	exonic	TRIM55	nonsynonymous SNV	TRIM55:ENST00000315962.4:exon3:c.C425T:p.P142L,TRIM55:ENST00000353317.5:exon3:c.C425T:p.P142L,TRIM55:ENST00000350034.4:exon3:c.C425T:p.P142L,TRIM55:ENST00000276573.7:exon3:c.C425T:p.P142L,	8q13.1	.
8	71069041	C	A	TGTGCTGCTG,AAACTCCCAC	exonic	NCOA2	nonsynonymous SNV	NCOA2:ENST00000518287.2:exon11:c.G1559T:p.C520F,NCOA2:ENST00000452400.2:exon11:c.G1559T:p.C520F,	8q13.3	.
8	77768387	C	T	CTGGGCTTGA,GGTACAGCAG	exonic	ZFHX4	nonsynonymous SNV	ZFHX4:ENST00000518282.1:exon9:c.C9152T:p.T3051M,ZFHX4:ENST00000521891.2:exon10:c.C9230T:p.T3077M,ZFHX4:ENST00000050961.6:exon10:c.C9095T:p.T3032M,ZFHX4:ENST00000455469.2:exon10:c.C9095T:p.T3032M,	8q21.11	ZFHX4;basechange=c.9230C>T;AAchange=p.T3077M;tissue=breast,large_intestine
8	89128882	G	A	TCAGCCGGAG,AATAGAGCGG	exonic	MMP16	nonsynonymous SNV	MMP16:ENST00000286614.6:exon6:c.C937T:p.P313S,	8q21.3	.
8	98289325	C	T	CCCCAGAAGC,CGGGATATTT	exonic	TSPYL5	nonsynonymous SNV	TSPYL5:ENST00000322128.3:exon1:c.G748A:p.G250S,	8q22.1	.
8	100865833	C	T	TGGTCCCCTC,CTGGGGAAGA	exonic	VPS13B	nonsynonymous SNV	VPS13B:ENST00000358544.2:exon56:c.C10291T:p.P3431S,VPS13B:ENST00000357162.2:exon56:c.C10216T:p.P3406S,	8q22.2	.
8	101237462	C	T	GTCACCTATT,CTGCTGTGCC	exonic	SPAG1	nonsynonymous SNV	SPAG1:ENST00000251809.3:exon14:c.C1750T:p.P584S,SPAG1:ENST00000388798.2:exon14:c.C1750T:p.P584S,	8q22.2	.
8	103664057	T	A	TGGGCAGGTC,GGTGGTTACA	exonic	KLF10	nonsynonymous SNV	KLF10:ENST00000285407.6:exon3:c.A503T:p.Q168L,KLF10:ENST00000395884.3:exon3:c.A470T:p.Q157L,	8q22.3	.
8	133112346	C	T	AATGCCACCC,CTTCTCCTTC	exonic	HHLA1	nonsynonymous SNV	HHLA1:ENST00000414222.1:exon3:c.G175A:p.G59R,HHLA1:ENST00000434736.2:exon5:c.G283A:p.G95R,	8q24.22	.
8	139825193	G	A	GGACCCGAGG,GATATCACAA	exonic	COL22A1	nonsynonymous SNV	COL22A1:ENST00000435777.1:exon8:c.C1315T:p.P439S,COL22A1:ENST00000303045.6:exon8:c.C1315T:p.P439S,	8q24.23	.
8	144408459	G	A	GTGGATCTCC,TGAAGTCACA	exonic	TOP1MT	nonsynonymous SNV	TOP1MT:ENST00000521193.1:exon5:c.C122T:p.T41M,TOP1MT:ENST00000329245.4:exon4:c.C416T:p.T139M,TOP1MT:ENST00000519148.1:exon4:c.C122T:p.T41M,TOP1MT:ENST00000518007.1:exon5:c.C323T:p.T108M,TOP1MT:ENST00000522041.1:exon4:c.C122T:p.T41M,TOP1MT:ENST00000519591.1:exon5:c.C12	8q24.3	.
8	144810193	G	A	AAACCCGCGC,GCCCCCCGCGA	exonic	FAM83H	nonsynonymous SNV	FAM83H:ENST00000388913.3:exon5:c.C1438T:p.R480C,	8q24.3	.
8	144906541	G	A	CGCCGGCTCG,ACCCCCCTCC	exonic	PUF60	nonsynonymous SNV	PUF60:ENST00000349157.6:exon2:c.C53T:p.S18F,PUF60:ENST00000526459.1:exon2:c.C50T:p.S17F,PUF60:ENST00000526683.1:exon2:c.C53T:p.S18F,PUF60:ENST00000529999.1:exon3:c.C164T:p.S55F,PUF60:ENST00000533162.1:exon3:c.C164T:p.S55F,PUF60:ENST00000531897.1:exon3:c.C164T:p.S55F,	8q24.3	.
8	144990926	C	T	CCGCAGAAGG,CTTCTGGGCC	exonic	PLEC	nonsynonymous SNV	PLEC:ENST00000356346.3:exon32:c.G13021A:p.A4341T,PLEC:ENST00000398774.2:exon32:c.G12967A:p.A4323T,PLEC:ENST00000354958.2:exon32:c.G12997A:p.A4333T,PLEC:ENST00000527096.1:exon32:c.G13132A:p.A4378T,PLEC:ENST00000357649.2:exon32:c.G13075A:p.A4359T,PLEC:ENST00000345136.3:exon	8q24.3	.
8	145095753	C	T	CAGCTACACA,CCTCAAGCAC	exonic	SPATC1	nonsynonymous SNV	SPATC1:ENST00000447830.2:exon3:c.C1051T:p.P351S,SPATC1:ENST00000377470.3:exon3:c.C1051T:p.P351S,	8q24.3	.
8	145095754	C	T	AGCTACACAC,CTCAAGCACC	exonic	SPATC1	nonsynonymous SNV	SPATC1:ENST00000447830.2:exon3:c.C1052T:p.P351L,SPATC1:ENST00000377470.3:exon3:c.C1052T:p.P351L,	8q24.3	.
8	145758604	C	T	GGGTAGCGCT,TCTCTGCATG	exonic	ARHGAP39	nonsynonymous SNV	ARHGAP39:ENST00000276826.5:exon7:c.G2701A:p.E901K,ARHGAP39:ENST00000540274.1:exon8:c.G2701A:p.E901K,ARHGAP39:ENST00000377307.2:exon8:c.G2794A:p.E932K,	8q24.3	.
8	146107628	C	T	TGGTTGAAGG,TTTCCCACAC	exonic	ZNF250	nonsynonymous SNV	ZNF250:ENST00000417550.2:exon6:c.G940A:p.A314T,ZNF250:ENST00000292579.7:exon6:c.G955A:p.A319T,	8q24.3	.
9	414900	C	T	CCTTTACCTA,CTTTAGTTGG	exonic	DOCK8	nonsynonymous SNV	DOCK8:ENST00000453981.1:exon29:c.C3649T:p.P1217S,DOCK8:ENST00000469391.1:exon27:c.C3349T:p.P1117S,DOCK8:ENST00000382329.1:exon16:c.C2050T:p.P684S,DOCK8:ENST00000432829.2:exon29:c.C3445T:p.P1149S,	9p24.3	.
9	414901	C	T	CTTTACCTAC,TTTAGTTGGC	exonic	DOCK8	nonsynonymous SNV	DOCK8:ENST00000453981.1:exon29:c.C3650T:p.P1217L,DOCK8:ENST00000469391.1:exon27:c.C3350T:p.P1117L,DOCK8:ENST00000382329.1:exon16:c.C2051T:p.P684L,DOCK8:ENST00000432829.2:exon29:c.C3446T:p.P1149L,	9p24.3	.
9	8317921	G	A	AGTGCGGCAC,ATAGGAAAAC	exonic	PTPRD	nonsynonymous SNV	PTPRD:ENST00000397611.3:exon30:c.C4462T:p.R1488C,PTPRD:ENST00000381196.4:exon43:c.C5692T:p.R1898C,PTPRD:ENST00000397617.3:exon29:c.C4471T:p.R1491C,PTPRD:ENST00000397606.3:exon29:c.C4471T:p.R1491C,PTPRD:ENST00000486161.1:exon31:c.C4471T:p.R1491C,PTPRD:ENST00000355233.5	9p24.1	PTPRD_ENST00000355233;basechange=c.4474C>T;AAchange=p.R1492C;tissue=breast,prostate,endometrium&PT
9	14824968	G	A	GTCATCCACT,GAGTTATATG	exonic	FREM1	nonsynonymous SNV	FREM1:ENST00000380880.3:exon11:c.C1904T:p.P635L,FREM1:ENST00000380881.4:exon12:c.C1907T:p.P636L,FREM1:ENST00000380875.3:exon12:c.C1904T:p.P635L,FREM1:ENST00000422223.2:exon12:c.C1904T:p.P635L,	9p22.3	.
9	19126249	G	A	ATAGGCTGAG,ACATGAGGTC	exonic	PLIN2	nonsynonymous SNV	PLIN2:ENST00000380465.3:exon3:c.C89T:p.S30F,PLIN2:ENST00000434144.1:exon4:c.C89T:p.S30F,PLIN2:ENST00000276914.2:exon3:c.C89T:p.S30F,PLIN2:ENST00000411567.1:exon3:c.C89T:p.S30F,PLIN2:ENST00000380464.3:exon3:c.C89T:p.S30F,	9p22.1	.
9	20986335	G	A	AGTCTCTCAA,GACGATTCCC	exonic	FOCAD	nonsynonymous SNV	FOCAD:ENST00000605086.1:exon28:c.G3085A:p.G1029R,FOCAD:ENST00000380249.1:exon42:c.G4777A:p.G1593R,FOCAD:ENST00000338382.6:exon39:c.G4777A:p.G1593R,	9p21.3	.
9	32784530	C	T	GAAGGCGGGC,TCAGGGGGAA	exonic	TMEM215	nonsynonymous SNV	TMEM215:ENST00000342743.5:exon2:c.C349T:p.L117F,	9p21.1	.
9	33053136	C	T	CCTGCATGTT,ATGATGACCA	exonic	SMU1	nonsynonymous SNV	SMU1:ENST00000397149.3:exon10:c.G1275A:p.M425I,SMU1:ENST00000536631.1:exon8:c.G792A:p.M264I,	9p21.1	.
9	33442395	G	A	GAAGCCCATG,AGGTGCCAAT	exonic	AQP3	nonsynonymous SNV	AQP3:ENST00000297991.4:exon5:c.C614T:p.S205F,	9p13.3	.
9	34379648	G	A	ACTTACCTAA,GTTCGGATAT	exonic	C9orf24	nonsynonymous SNV	C9orf24:ENST00000297623.2:exon6:c.C785T:p.P262L,C9orf24:ENST00000379127.1:exon4:c.C385T:p.L129F,C9orf24:ENST00000379126.3:exon3:c.C226T:p.L76F,C9orf24:ENST00000379124.1:exon3:c.C385T:p.L129F,C9orf24:ENST00000379133.3:exon3:c.C380T:p.P127L,	9p13.3	.
9	35100138	G	A	TGGCACTGGG,CTTTGGTGAG	exonic	STOML2	nonsynonymous SNV	STOML2:ENST00000356493.5:exon10:c.C965T:p.A322V,STOML2:ENST00000452248.2:exon9:c.C830T:p.A277V,	9p13.3	.
9	35310573	G	A	TCGGGAGACG,ACTCGATTCA	exonic	UNC13B	nonsynonymous SNV	UNC13B:ENST00000378495.3:exon9:c.G871A:p.D291N,UNC13B:ENST00000378496.4:exon9:c.G871A:p.D291N,UNC13B:ENST00000396787.1:exon10:c.G907A:p.D303N,	9p13.3	.
9	77377094	G	A	ATCAGATAGG,AGCTGTCCTG	exonic	TRPM6	nonsynonymous SNV	TRPM6:ENST00000451710.3:exon26:c.C4493T:p.S1498F,TRPM6:ENST00000361255.3:exon26:c.C4478T:p.S1493F,TRPM6:ENST00000360774.1:exon26:c.C4493T:p.S1498F,TRPM6:ENST00000376864.4:exon26:c.C4493T:p.S1498F,TRPM6:ENST00000449912.2:exon26:c.C4478T:p.S1493F,	9q21.13	.
9	77390931	A	G	GTCATGATGT,GCGATAGCGG	exonic	TRPM6	nonsynonymous SNV	TRPM6:ENST00000451710.3:exon24:c.T3271C:p.Y1091H,TRPM6:ENST00000361255.3:exon24:c.T3256C:p.Y1086H,TRPM6:ENST00000360774.1:exon24:c.T3271C:p.Y1091H,TRPM6:ENST00000376864.4:exon24:c.T3271C:p.Y1091H,TRPM6:ENST00000449912.2:exon24:c.T3256C:p.Y1086H,	9q21.13	.
9	84267142	T	A	GATGATGGCA,TCAATTCTGC	exonic	TLE1	nonsynonymous SNV	TLE1:ENST00000418319.1:exon6:c.A359T:p.N120I,TLE1:ENST00000376499.3:exon6:c.A359T:p.N120I,TLE1:ENST00000376463.1:exon5:c.A191T:p.N64I,	9q21.32	.
9	88247860	C	T	AGAACATTTC,ACAAGTAGCC	exonic	AGTPBP1	nonsynonymous SNV	AGTPBP1:ENST00000432218.1:exon10:c.G1246A:p.G416R,AGTPBP1:ENST00000357081.3:exon14:c.G1732A:p.G578R,AGTPBP1:ENST00000376109.3:exon13:c.G1768A:p.G590R,AGTPBP1:ENST00000376083.3:exon14:c.G1612A:p.G538R,	9q21.33	.
9	96021718	C	T	CCCACGCTGC,CCCTCAACCT	exonic	WNK2	nonsynonymous SNV	WNK2:ENST00000297954.4:exon11:c.C2888T:p.P963L,WNK2:ENST00000395477.2:exon11:c.C2888T:p.P963L,WNK2:ENST00000349097.3:exon11:c.C1724T:p.P575L,WNK2:ENST00000427277.2:exon11:c.C1724T:p.P575L,	9q22.31	.
9	97873869	C	T	CTCAGCCCAT,CTCCGAAGTG	exonic	FANCC	nonsynonymous SNV	FANCC:ENST00000289081.3:exon13:c.G1205A:p.G402E,FANCC:ENST00000375305.1:exon13:c.G1205A:p.G402E,	9q22.32	FANCC_ENST00000289081;basechange=c.1205G>T;AAchange=p.G402V;tissue=endometrium
9	100076901	C	T	TCTGCGTAGG,CCTGCTGGAG	exonic	CCDC180	nonsynonymous SNV	CCDC180:ENST00000411667.2:exon21:c.C713T:p.A238V,CCDC180:ENST00000357054.1:exon21:c.C1139T:p.A380V,CCDC180:ENST00000375202.2:exon21:c.C722T:p.A241V,CCDC180:ENST00000395220.1:exon21:c.C1139T:p.A380V,CCDC180:ENST00000529487.1:exon7:c.C722T:p.A241V,	9q22.33	.
9	100092747	G	A	AGAAGAAAAC,ATCAAGAAAT	exonic	CCDC180	nonsynonymous SNV	CCDC180:ENST00000411667.2:exon32:c.G2095A:p.D699N,CCDC180:ENST00000357054.1:exon32:c.G2521A:p.D841N,CCDC180:ENST00000375202.2:exon32:c.G2104A:p.D702N,CCDC180:ENST00000529487.1:exon18:c.G2104A:p.D702N,	9q22.33	.
9	100692830	C	T	TCATTGTATT,CCTTGGTTCT	exonic	HEMGN	nonsynonymous SNV	HEMGN:ENST00000259456.3:exon4:c.G847A:p.E283K,	9q22.33	.
9	111624703	G	A	GCCTCTTTAA,GGATGGCCCG	exonic	ACTL7A	nonsynonymous SNV	ACTL7A:ENST00000333999.3:exon1:c.G101A:p.R34K,	9q31.3	ACTL7A;basechange=c.101G>T;AAchange=p.R34M;tissue=upper_aerodigestive_tract
9	111706249	G	C	TACCTGTTTT,AAAAAGCTTG	exonic	CTNNAL1	stopgain	CTNNAL1:ENST00000325580.6:exon14:c.C1682G:p.S561X,CTNNAL1:ENST00000374595.4:exon16:c.C1934G:p.S645X,CTNNAL1:ENST00000325551.4:exon16:c.C1934G:p.S645X,CTNNAL1:ENST00000374594.1:exon5:c.C137G:p.S46X,	9q31.3	.
9	111976086	C	T	GGCAGGTGTT,CTGGACTCAG	exonic	EPB41L4B	nonsynonymous SNV	EPB41L4B:ENST00000374566.3:exon17:c.G1646A:p.G549E,	9q31.3	.
9	113217884	G	A	GTAACCTGAT,GGCACTCACA	exonic	SVEP1	nonsynonymous SNV	SVEP1:ENST00000302728.8:exon23:c.C3773T:p.P1258L,SVEP1:ENST00000374469.1:exon22:c.C3704T:p.P1235L,SVEP1:ENST00000401783.2:exon22:c.C3773T:p.P1258L,	9q31.3	.
9	115422233	C	T	GCGCTCTCTT,GGGGAATTGT	exonic	KIAA1958	nonsynonymous SNV	KIAA1958:ENST00000536272.1:exon5:c.C2119T:p.R707W,KIAA1958:ENST00000337530.6:exon4:c.C2035T:p.R679W,	9q32	.
9	123292356	T	G	AGATTTCTCC,CTTTAAGGCA	exonic	CDK5RAP2	nonsynonymous SNV	CDK5RAP2:ENST00000481266.1:exon8:c.A725C:p.E242A,CDK5RAP2:ENST00000480112.1:exon8:c.A722C:p.E241A,CDK5RAP2:ENST00000359309.3:exon8:c.A725C:p.E242A,CDK5RAP2:ENST00000349780.4:exon8:c.A725C:p.E242A,CDK5RAP2:ENST00000473282.2:exon8:c.A722C:p.E241A,CDK5RAP2:ENST0000036(	9q33.2	.
9	123725197	C	T	ATAACATGTC,ATCTTTGATT	exonic	C5	nonsynonymous SNV	C5:ENST00000223642.1:exon35:c.G4372A:p.G1458R,	9q33.2	.
9	124632959	G	A	GTTGCAGTCT,GAGCACTGGT	exonic	TTLL11	nonsynonymous SNV	TTLL11:ENST00000321582.5:exon7:c.C1820T:p.P607L,	9q33.2	.
9	127110084	G	A	ACTCCCCGGG,AGCACTACTC	exonic	NEK6	nonsynonymous SNV	NEK6:ENST00000320246.5:exon9:c.G814A:p.E272K,NEK6:ENST00000373603.1:exon9:c.G814A:p.E272K,NEK6:ENST00000546191.1:exon9:c.G814A:p.E272K,NEK6:ENST00000540326.1:exon9:c.G868A:p.E290K,NEK6:ENST00000394199.2:exon10:c.G916A:p.E306K,NEK6:ENST00000545174.1:exon9:c.G814A:p.E272K,N	9q33.3	.
9	128003091	T	C	AATCAGACGT,CCCCTTCAGG	exonic	HSPA5	nonsynonymous SNV	HSPA5:ENST00000324460.6:exon2:c.A218G:p.E73G,	9q33.3	.
9	129870542	G	A	TTCTCCAGCT,GGAGAGCTCC	exonic	ANGPTL2	stopgain	ANGPTL2:ENST00000373425.3:exon2:c.C469T:p.Q157X,	9q33.3	.
9	130440783	G	A	CTCTCACGGT,GACTCCGATT	exonic	STXBP1	stopgain	STXBP1:ENST00000373299.1:exon16:c.G1433A:p.W478X,STXBP1:ENST00000373302.3:exon16:c.G1433A:p.W478X,	9q34.11	.
9	131198161	C	T	CAGCCTCCAA,CCCAGCCCCG	exonic	CERCAM	nonsynonymous SNV	CERCAM:ENST00000372838.4:exon12:c.C1765T:p.P589S,CERCAM:ENST00000372842.1:exon13:c.C1531T:p.P511S,	9q34.11	.
9	131198162	C	T	AGCCTCCAAC,CCAGCCCCGA	exonic	CERCAM	nonsynonymous SNV	CERCAM:ENST00000372838.4:exon12:c.C1766T:p.P589L,CERCAM:ENST00000372842.1:exon13:c.C1532T:p.P511L,	9q34.11	.
9	131897136	G	A	GCTCCGGGTG,ATGACCAAAT	exonic	PPP2R4	nonsynonymous SNV	PPP2R4:ENST00000393370.2:exon6:c.G523A:p.D175N,PPP2R4:ENST00000337738.1:exon7:c.G628A:p.D210N,PPP2R4:ENST00000452489.2:exon7:c.G628A:p.D210N,PPP2R4:ENST00000417728.1:exon6:c.G418A:p.D140N,PPP2R4:ENST00000455292.1:exon8:c.G628A:p.D210N,PPP2R4:ENST00000357197.4:exon5:c.G4	9q34.11	.
9	132985397	C	T	GGAGAACACT,CTGAGAAGAG	exonic	NCS1	nonsynonymous SNV	NCS1:ENST00000372398.3:exon6:c.C433T:p.P145S,NCS1:ENST00000458469.1:exon6:c.C379T:p.P127S,	9q34.11	.
9	134136225	G	A	GTGCTTGGGC,GGATCACCAC	exonic	FAM78A	nonsynonymous SNV	FAM78A:ENST00000464831.1:exon4:c.C743T:p.P248L,FAM78A:ENST00000372271.3:exon2:c.C836T:p.P279L,FAM78A:ENST00000372269.3:exon4:c.C827T:p.P276L,	9q34.13	.
9	136083940	G	A	CCTGTCCTCC,GAAAGTCCTT	exonic	OBP2B	nonsynonymous SNV	OBP2B:ENST00000372034.3:exon2:c.C122T:p.P41L,OBP2B:ENST00000473737.1:exon2:c.C122T:p.P41L,	9q34.2	.
9	138377435	C	T	TCCGAGGCGT,CGACTCCAGC	exonic	PPP1R26	nonsynonymous SNV	PPP1R26:ENST00000401470.3:exon3:c.C1079T:p.S360F,PPP1R26:ENST00000605660.1:exon1:c.C1079T:p.S360F,PPP1R26:ENST00000356818.2:exon4:c.C1079T:p.S360F,PPP1R26:ENST00000605286.1:exon4:c.C1079T:p.S360F,PPP1R26:ENST00000604351.1:exon3:c.C1079T:p.S360F,	9q34.3	.
9	138714660	C	T	TCTCCCTTCC,CCCGTTCATC	exonic	CAMSAP1	nonsynonymous SNV	CAMSAP1:ENST00000389532.4:exon11:c.G1847A:p.G616E,CAMSAP1:ENST00000409386.3:exon12:c.G1880A:p.G627E,CAMSAP1:ENST00000312405.6:exon9:c.G1013A:p.G338E,	9q34.3	.
9	139402755	C	A	GCCGCTGGGG,ACTCGCAGCG	exonic	NOTCH1	nonsynonymous SNV	NOTCH1:ENST00000277541.6:exon20:c.G3254T:p.C1085F,	9q34.3	.
9	139902956	G	C	CTCCGTGGGG,CAGACCGGGG	exonic	ABCA2	nonsynonymous SNV	ABCA2:ENST00000448336.1:exon4:c.C434G:p.A145G,ABCA2:ENST00000490486.1:exon5:c.C464G:p.A155G,ABCA2:ENST00000371605.3:exon47:c.C7184G:p.A2395G,ABCA2:ENST00000265662.5:exon48:c.C7187G:p.A2396G,ABCA2:ENST00000341511.6:exon48:c.C7187G:p.A2396G,	9q34.3	.
9	139912773	G	A	AGGCGGGAGC,AGCCGTCCTT	exonic	ABCA2	nonsynonymous SNV	ABCA2:ENST00000487109.1:exon13:c.C1937T:p.S646L,ABCA2:ENST00000371605.3:exon13:c.C1847T:p.S616L,ABCA2:ENST00000265662.5:exon14:c.C1850T:p.S617L,ABCA2:ENST00000341511.6:exon14:c.C1850T:p.S617L,ABCA2:ENST00000459850.1:exon13:c.C1937T:p.S646L,	9q34.3	.
9	140063341	C	T	CTCCCCGGGT,CGCGGGCGAG	exonic	LRRC26	nonsynonymous SNV	LRRC26:ENST00000371542.3:exon2:c.G970A:p.D324N,	9q34.3	.
9	140086983	G	A	GAGTGCAAAG,GCTTCTCCTC	exonic	TPRN	nonsynonymous SNV	TPRN:ENST00000333046.4:exon2:c.C1280T:p.P427L,TPRN:ENST00000321773.2:exon2:c.C1703T:p.P568L,TPRN:ENST00000409012.4:exon2:c.C1886T:p.P629L,	9q34.3	.
9	140972643	C	T	CAGGCCAATG,CACCGAGTGT	exonic	CACNA1B	nonsynonymous SNV	CACNA1B:ENST00000277551.2:exon36:c.C5027T:p.A1676V,CACNA1B:ENST00000371365.2:exon2:c.C119T:p.A40V,CACNA1B:ENST00000371357.1:exon35:c.C5024T:p.A1675V,CACNA1B:ENST00000371363.1:exon35:c.C5021T:p.A1674V,CACNA1B:ENST00000371372.1:exon36:c.C5027T:p.A1676V,CACNA1B:ENST000(	9q34.3	.
10	697091	G	A	GGGCCTCAGG,GCACCTCCAC	exonic	PRR26	nonsynonymous SNV	PRR26:ENST00000441152.2:exon3:c.C584A:p.G195E,	10p15.3	.
10	3174601	G	A	CGGAGAAAAT,AAGACCACCA	exonic	PFKP	nonsynonymous SNV	PFKP:ENST00000381072.1:exon2:c.G132A:p.M44I,PFKP:ENST00000381075.2:exon20:c.G1854A:p.M618I,PFKP:ENST00000381125.4:exon18:c.G1878A:p.M626I,	10p15.2	.
10	7618500	C	T	TCCTCCAGGC,ATCATGCGT	exonic	ITIH5	nonsynonymous SNV	ITIH5:ENST00000446830.2:exon10:c.G1240A:p.G414S,ITIH5:ENST00000397146.2:exon10:c.G1894A:p.G632S,ITIH5:ENST00000298441.6:exon6:c.G1252A:p.G418S,ITIH5:ENST00000397145.2:exon10:c.G1894A:p.G632S,ITIH5:ENST00000256861.6:exon10:c.G1894A:p.G632S,	10p14	.
10	7786083	C	T	TGCCAAGAAG,CCAACAATGG	exonic	ITIH2	nonsynonymous SNV	ITIH2:ENST00000358415.4:exon18:c.C2248T:p.P750S,ITIH2:ENST00000379587.4:exon17:c.C2215T:p.P739S,	10p14	.
10	7786084	C	T	GCCAAGAAGC,CAACAATGGA	exonic	ITIH2	nonsynonymous SNV	ITIH2:ENST00000358415.4:exon18:c.C2249T:p.P750L,ITIH2:ENST00000379587.4:exon17:c.C2216T:p.P739L,	10p14	.
10	11911683	G	A	CACGCCCCTC,TTATGGCGCA	exonic	PROSER2	nonsynonymous SNV	PROSER2:ENST00000277570.5:exon4:c.G586A:p.V196I,	10p14	.
10	15255131	C	T	CCGACTCGAC,CCTCCAACAA	exonic	FAM171A1	nonsynonymous SNV	FAM171A1:ENST00000378116.4:exon8:c.G2456A:p.G819E,	10p13	FAM171A1;basechange=c.2456G>T;AAchange=p.G819V;tissue=lung&FAM171A1_ENST00000378116;basechange=
10	17243630	C	T	CGCAGGGGCT,CATCCCCGCG	exonic	TRDMT1	nonsynonymous SNV	TRDMT1:ENST00000358282.7:exon1:c.G4A:p.E2K,TRDMT1:ENST00000495022.1:exon1:c.G4A:p.E2K,TRDMT1:ENST00000377766.5:exon1:c.G4A:p.E2K,TRDMT1:ENST00000412821.3:exon1:c.G4A:p.E2K,TRDMT1:ENST00000354631.3:exon1:c.G4A:p.E2K,TRDMT1:ENST00000377799.3:exon1:c.G4A:p.E2K,TRDMT1:ENS	10p13	.
10	25886965	G	A	GAAATCCAAG,AGGAGACCCT	exonic	GPR158	nonsynonymous SNV	GPR158:ENST00000376351.3:exon11:c.G2410A:p.E804K,	10p12.1	.
10	43977067	G	A	CATCTGGAAT,AATTTGAATA	exonic	ZNF487	nonsynonymous SNV	ZNF487:ENST00000456416.1:exon3:c.G228A:p.M76I,ZNF487:ENST00000437590.2:exon4:c.G324A:p.M108I,ZNF487:ENST00000431662.1:exon4:c.G516A:p.M172I,	10q11.21	.
10	44868490	G	A	CTGAGTCAGA,GAGTGGCTCC	exonic	CXCL12	nonsynonymous SNV	CXCL12:ENST00000395793.3:exon3:c.C158T:p.P53L,	10q11.21	.
10	49951310	G	A	AGCCCTGGAG,AAGAGGGCAA	exonic	WDFY4	nonsynonymous SNV	WDFY4:ENST00000413659.2:exon11:c.G2176A:p.E726K,WDFY4:ENST00000325239.5:exon11:c.G2176A:p.E726K,	10q11.23	.
10	52103554	C	A	ACCCATTTGG,ATCCCGTTGG	exonic	SGMS1	nonsynonymous SNV	SGMS1:ENST00000361543.2:exon1:c.G321T:p.M107I,SGMS1:ENST00000361781.2:exon7:c.G321T:p.M107I,	10q11.23	.

10	55582847	C	T	TCTACAAAAT,TGTTCTCTGT	exonic	PCDH15	nonsynonymous SNV	[illegible]
10	60560077	C	T	CAAATCAAGC,CCACTGAAGG	exonic	BICC1	nonsynonymous SNV	[illegible]
10	71683559	G	A	CTTCCCCAGG,AGAAGCAGGT	exonic	COL13A1	nonsynonymous SNV	[illegible]
10	72539390	A	T	TGGGTCTCCA,TGGGGACAGA	exonic	TBATA	nonsynonymous SNV	[illegible]
10	73468890	C	T	CGTGGGTTAC,GCGATGCCGT	exonic	CDH23	nonsynonymous SNV	[illegible]
10	73560477	G	A	CAACCCTGGG,ATGTAGCCAG	exonic	CDH23	nonsynonymous SNV	[illegible]
10	75864984	G	T	TGGCTAAGAG,GAGGTGGAGA	exonic	VCL	nonsynonymous SNV	[illegible]
10	91144374	G	A	CAGAATGTAC,GGTTGTGGAA	exonic	IFIT1B	nonsynonymous SNV	[illegible]
10	94828440	G	A	TGCGGGGAAT,GGCTATGCCT	exonic	CYP26C1	nonsynonymous SNV	[illegible]
10	96602608	G	A	AGTCCAGGAA,AGATTGAACG	exonic	CYP2C19	nonsynonymous SNV	[illegible]
10	100995513	G	A	GGGGGGGCGG,AGTTGCTGGA	exonic	HPSE2	nonsynonymous SNV	[illegible]
10	102749625	G	A	GACTTTCCAT,GACAGCAAAG	exonic	C10orf2	nonsynonymous SNV	[illegible]
10	103530091	G	A	AGCACCTATC,GGGCTCGGGG	exonic	FGF8	stopgain	[illegible]
10	103868896	G	A	GACATCTTCC,TTTCCGCCGT	exonic	LDB1	nonsynonymous SNV	[illegible]
10	113940284	G	A	GGCCTTTTCC,ACTCATTAGG	exonic	GPAM	nonsynonymous SNV	[illegible]
10	129868630	C	G	CAGAGCTGGA,GTGTCCTCCC	exonic	PTPRE	nonsynonymous SNV	[illegible]
11	251558	C	T	GACTTTCACA,GACCTGCCAA	exonic	PSMD13	stopgain	[illegible]
11	279404	G	A	CAGCTGAAGC,CTTCCGCCAC	exonic	NLRP6	nonsynonymous SNV	[illegible]
11	764838	G	A	GCAGAGAATG,AAAGTAGCGC	exonic	TALDO1	nonsynonymous SNV	[illegible]
11	1272716	C	T	ACCGCCTCCT,CACTCTGGGA	exonic	MUC5B	nonsynonymous SNV	[illegible]
11	1585147	C	T	AGGAAGCTGT,TGCGGCCAGC	exonic	DUSP8	nonsynonymous SNV	[illegible]
11	4411372	C	T	GCACACCGTT,CCCCTGTGTG	exonic	TRIM21	nonsynonymous SNV	[illegible]
11	5730867	C	T	TGTGTGCCCA,CGAGCTCCTG	exonic	TRIM22	nonsynonymous SNV	[illegible]
11	5730868	C	T	GTGTGCCCAC,GAGCTCCTGA	exonic	TRIM22	nonsynonymous SNV	[illegible]
11	6645426	T	A	AGGGGGCAGG,CTTCAGTCAC	exonic	DCHS1	nonsynonymous SNV	[illegible]
11	7654158	G	A	GAAATCACTG,AAACCAGGTA	exonic	PPFIBP2	nonsynonymous SNV	[illegible]
11	7670033	G	C	AATTTCAGGA,CTAGGAATTA	exonic	PPFIBP2	nonsynonymous SNV	[illegible]
11	8640452	C	T	GGGTAGATCT,CTTCAACCAG	exonic	TRIM66	nonsynonymous SNV	[illegible]
11	12284042	C	A	TTCAGCCTTC,AGTGCTACAC	exonic	MICAL2	nonsynonymous SNV	[illegible]
11	17569044	G	C	GGGAGGTCGG,GGCTCGAGGA	exonic	OTOG	nonsynonymous SNV	[illegible]
11	30255210	G	T	GCCCGGCTGT,CTCACCATGC	exonic	FSHB	nonsynonymous SNV	[illegible]
11	34502319	C	T	ATTAACTTAC,TCAGGGCTCT	exonic	ELF5	nonsynonymous SNV	[illegible]
11	47377066	G	A	GGTCCAACAG,AACTGGTACA	exonic	SPI1	nonsynonymous SNV	[illegible]
11	49080570	T	G	GCAAAAGCTG,GCACACAGTC	exonic	TRIM64C	nonsynonymous SNV	[illegible]
11	55339830	C	T	ACTTCCATAA,CCCTAGAATG	exonic	OR4C16	nonsynonymous SNV	[illegible]
11	60501056	C	T	GCAATTAACC,TGTGCTGTAT	exonic	MS4A18	nonsynonymous SNV	[illegible]
11	61511320	G	A	CATCCCCGAG,AAAACCCATC	exonic	DAGLA	nonsynonymous SNV	[illegible]
11	62124474	C	T	TCATTGCTTT,TGACTGACCA	exonic	ASRGL1	nonsynonymous SNV	[illegible]
11	62437283	G	A	ATCCTGAATA,GGACAGCAGG	exonic	C11orf48	nonsynonymous SNV	[illegible]
11	64518928	C	A	CATGTGGTAC,CAGGTGCAGC	exonic	PYGM	nonsynonymous SNV	[illegible]
11	64884752	G	A	CGGCCGCCAT,GAGGCAGCAG	exonic	ZNHIT2	nonsynonymous SNV	[illegible]
11	65321842	G	T	GCAGGGGAGA,GGCACACCAC	exonic	LTBP3	nonsynonymous SNV	[illegible]
11	66192373	A	G	CTGGACTCCA,CCTGTCCCTG	exonic	NPAS4	nonsynonymous SNV	[illegible]
11	66468043	C	T	CCGCAGGAAT,CCTGGAAGCC	exonic	SPTBN2	nonsynonymous SNV	[illegible]
11	69625249	C	T	TGGTCCAGCA,GCGGGGCAGG	exonic	FGF3	nonsynonymous SNV	[illegible]
11	71819933	G	A	TGCCATCAAG,ATGGAATAGC	exonic	LRTOMT	nonsynonymous SNV	[illegible]
11	73020555	G	A	CGCTGGGGAG,GAGGCCCGGG	exonic	ARHGEF17	nonsynonymous SNV	[illegible]
11	73022490	G	A	GAGCTTCGGA,AGACGAGGGC	exonic	ARHGEF17	nonsynonymous SNV	[illegible]
11	76207268	C	G	GGTGTATCTA,ATCAGCAATC	exonic	C11orf30	nonsynonymous SNV	[illegible]
11	77885042	C	T	GCCACCCAGT,CAGAGTCAGG	exonic	KCTD21	nonsynonymous SNV	[illegible]
11	86518797	C	T	ATACCGCCTC,CTGTCGTCTT	exonic	PRSS23	nonsynonymous SNV	[illegible]
11	102495962	G	A	GGTCCTGGGG,AGGCTGCAAC	exonic	MMP20	nonsynonymous SNV	[illegible]
11	103908430	G	A	TAAAGGAGTG,GCACACAGAG	exonic	DDI1	nonsynonymous SNV	[illegible]
11	113679905	G	A	TTATCCTCCT,AATGTAATGC	exonic	USP28	stopgain	[illegible]
11	117242148	C	T	AAGAAGGCTT,TGCTCTGGAA	exonic	CEP164	nonsynonymous SNV	[illegible]
11	118764723	C	T	GCCGTCCATG,CTACCGCCAC	exonic	CXCR5	nonsynonymous SNV	[illegible]
11	123597070	G	A	TGGATTCTTT,GTGTGTTGTT	exonic	ZNF202	stopgain	[illegible]
11	124056258	C	A	TCATCTCCTA,AAAGACTGTG	exonic	OR10D3	stopgain	[illegible]
11	124135631	G	A	TCAGCTCCAT,GACCAGGGGA	exonic	OR8G1,OR8G	nonsynonymous SNV	[illegible]
11	125479445	T	C	CTGGTCCTCA,ACTATTTTGA	exonic	STT3A	nonsynonymous SNV	[illegible]
11	126870387	C	T	AAGAGCAGAT,GAGCTGGAAG	exonic	KIRREL3	nonsynonymous SNV	[illegible]
11	129746621	G	C	CCTACCAAGA,ACAGAATGGT	exonic	NFRKB	nonsynonymous SNV	[illegible]
11	130332560	G	A	AAGGCCAAGG,ACAGATGGTG	exonic	ADAMTS15	nonsynonymous SNV	[illegible]
12	1943822	C	T	CAAAAAGCGC,AGCCCCTGAT	exonic	LRTM2	stopgain	[illegible]
12	2061803	G	A	CCAGAGATGG,GAGTGTTTGT	exonic	DCP1B	nonsynonymous SNV	[illegible]
12	6085308	G	A	GGGCTTCTGG,AGCACTGGGC	exonic	VWF	nonsynonymous SNV	[illegible]
12	6105356	C	G	TCAAAGGTCA,GATGTGCCGA	exonic	VWF	nonsynonymous SNV	[illegible]
12	7016547	C	T	AGTGGAGCTT,GGGGGAACCA	exonic	LRRC23	nonsynonymous SNV	[illegible]
12	7044816	G	A	CAGGACAACC,AAGCACGTCC	exonic	ATN1	nonsynonymous SNV	[illegible]
12	7559198	C	T	GTCTGCAGTC,CAAAGAAAAG	exonic	CD163L1	stopgain	[illegible]
12	7559199	C	T	TCTGCAGTCC,AAAGAAAAGA	exonic	CD163L1	stopgain	[illegible]
12	10959464	T	A	GGAAATCTTT,TCTTCTTAAT	exonic	TAS2R8	nonsynonymous SNV	[illegible]
12	11546452	C	T	CTGGTTGCCT,CTTGTGGGGG	exonic	PRB2	nonsynonymous SNV	[illegible]
12	21998730	G	A	CATTACAGTG,ACATGTTATC	exonic	ABCC9	nonsynonymous SNV	[illegible]
12	26493219	G	A	TTGCTAACGA,GGACATGGCT	exonic	ITPR2	nonsynonymous SNV	[illegible]
12	26875437	C	A	TCCAGTAAAG,AGGTAATCTC	exonic	ITPR2	nonsynonymous SNV	[illegible]
12	32480548	C	T	CAGCAAGGAG,TCAAGGCTGA	exonic	BICD1	nonsynonymous SNV	[illegible]
12	40873001	T	A	AGATACAACA,CTGGACCATC	exonic	MUC19	nonsynonymous SNV	[illegible]
12	40873067	C	T	GGCATCTGAA,CATCAGTTGG	exonic	MUC19	nonsynonymous SNV	[illegible]
12	40873068	C	T	GCATCTGAAC,ATCAGTTGGA	exonic	MUC19	nonsynonymous SNV	[illegible]
12	40878492	C	A	GTCGGAGTGA,AGGGACAACT	exonic	MUC19	nonsynonymous SNV	[illegible]
12	41900462	C	T	GGCCAAGCTT,GTCCACCTAC	exonic	PDZRN4	nonsynonymous SNV	[illegible]
12	45173523	C	T	GAGGGCTTTT,TACTACCCTT	exonic	NELL2	nonsynonymous SNV	[illegible]
12	49434925	G	A	CCCAGCATCG,GGGCTGCGCA	exonic	KMT2D	nonsynonymous SNV	[illegible]
12	50028344	G	A	ATGCCTCATG,GAACAGGCCA	exonic	PRPF40B	stopgain	[illegible]
12	50473705	C	A	GGAGAAGGAC,AGGAGTACTG	exonic	ASIC1	nonsynonymous SNV	[illegible]
12	50749693	C	T	TTACTGAGAT,TCGTATTATC	exonic	FAM186A	nonsynonymous SNV	[illegible]
12	53683940	C	T	CCTGCTGACC,GGCTGGAAAA	exonic	ESPL1	nonsynonymous SNV	[illegible]
12	54912450	C	T	GTCTGTGTGT,CAGAGGAGGA	exonic	NCKAP1L	nonsynonymous SNV	[illegible]
12	55356400	C	T	AAAGTCTCAT,CTTGGCTGGA	exonic	TESPA1	nonsynonymous SNV	[illegible]
12	55863356	G	T	GTAAATGTGT,TCTGAGCAAG	exonic	OR6C70	nonsynonymous SNV	[illegible]
12	56981365	C	T	AGGGATGGAC,ATCCCATTTC	exonic	RBMS2	nonsynonymous SNV	[illegible]
12	57324221	G	A	CATTCGTTGG,ACCACTGGGC	exonic	SDR9C7	nonsynonymous SNV	[illegible]
12	57397683	T	C	CATGGCCTTA,TCTCCCGATT	exonic	ZBTB39	nonsynonymous SNV	[illegible]
12	57424072	G	A	TACCTGCTTC,GGGACAGCTG	exonic	MYO1A	nonsynonymous SNV	[illegible]
12	57424073	G	A	ACCTGCTTCG,GGACAGCTGA	exonic	MYO1A	nonsynonymous SNV	[illegible]
12	57689238	G	A	AACCTCTGTT,AAATTCTGTA	exonic	R3HDM2,RP1	stopgain	[illegible]
12	58024292	A	T	AGCTGCCTGA,GGCTCAGCCC	exonic	B4GALNT1	nonsynonymous SNV	[illegible]
12	70326323	C	T	ACAGGCGTGT,CTAATTGGGT	exonic	MYRFL	nonsynonymous SNV	[illegible]
12	70326324	C	T	CAGGCGTGTC,TAATTGGGTT	exonic	MYRFL	nonsynonymous SNV	[illegible]
12	81010018	C	T	TCCAAAGGTT,CGATGAGAAT	exonic	PTPRQ	nonsynonymous SNV	[illegible]
12	88490763	G	A	TTCTTTTAAG,AGATGTTTTC	exonic	CEP290	nonsynonymous SNV	[illegible]
12	96379852	C	T	CCTGCTATTT,TGATGGGTGG	exonic	HAL	nonsynonymous SNV	[illegible]
12	96728812	C	T	TCACCTACTC,GAATAATGCT	exonic	CDK17	nonsynonymous SNV	[illegible]
12	105446638	A	T	CATCGTCTGC,TCTGTGAACT	exonic	ALDH1L2	nonsynonymous SNV	[illegible]
12	108589740	C	T	GGCCAGCCCG,TGTCTCGGGG	exonic	WSCD2	nonsynonymous SNV	[illegible]
12	109182203	C	T	GTGGTCCAGG,GGTAGAAGAA	exonic	SSH1	nonsynonymous SNV	[illegible]
12	111701628	T	A	TTACAAGCAA,TAATTGAAGC	exonic	CUX2	nonsynonymous SNV	[illegible]
12	117595774	C	T	CTCCTTTTTG,GCTCAATGTG	exonic	FBXO21	nonsynonymous SNV	[illegible]
12	120584894	G	A	CCAATAATCT,GGCTGCCATC	exonic	GCN1L1	stopgain	[illegible]
12	121435340	C	T	AGCCTGACCA,CCTGCAGCCC	exonic	HNF1A	nonsynonymous SNV	[illegible]
12	121682955	C	T	GAGCAAGTTT,CAGGCGCTGA	exonic	CAMKK2	nonsynonymous SNV	[illegible]
12	121854065	G	A	GGAGCCACCG,TCCATTCAGA	exonic	RNF34	nonsynonymous SNV	[illegible]
12	122255483	C	T	TCACCCTCGT,CTCGGCCTCC	exonic	SETD1B	nonsynonymous SNV	[illegible]
12	123416795	G	A	ATGAGGACTG,GGGGTTCCGC	exonic	ABCB9	nonsynonymous SNV	[illegible]
12	124330245	A	C	AAAGCGCAAA,AGGGGAGAAG	exonic	DNAH10	nonsynonymous SNV	[illegible]
12	124366250	C	T	GCATTTAACC,GGGTGCACCG	exonic	DNAH10	nonsynonymous SNV	[illegible]
12	132445253	C	T	GAGCAGCCGG,CCACCCCAAC	exonic	EP400	nonsynonymous SNV	[illegible]
12	132905612	G	A	ATGGCCTCAC,GTCCCCCAGC	exonic	GALNT9	nonsynonymous SNV	[illegible]
13	21562363	G	A	CAGGTGCTTC,GGTAGGGCGG	exonic	LATS2	nonsynonymous SNV	[illegible]
13	21562364	G	A	AGGTGCTTCG,GTAGGGCGGA	exonic	LATS2	nonsynonymous SNV	[illegible]
13	23898558	T	G	GCAGGGGACG,GGGGTCCCTC	exonic	SGCG	nonsynonymous SNV	[illegible]
13	29041725	G	A	CTCAGTTCAG,ATCTTTTAAT	exonic	FLT1	nonsynonymous SNV	[illegible]
13	39263985	C	T	ATCCTCAACA,CGGCTTCACT	exonic	FREM2	nonsynonymous SNV	[illegible]
13	39266326	C	A	GTGAAGATAG,TTCTCCTTCA	exonic	FREM2	nonsynonymous SNV	[illegible]
13	46919676	G	A	GTCCTCAAGG,AGAACAGGTG	exonic	KIAA0226L	nonsynonymous SNV	[illegible]
13	51825699	G	A	GGAGGCCATC,ACAACGTCCT	exonic	FAM124A	nonsynonymous SNV	[illegible]
13	102030980	G	A	ACACACCAGC,ATCTTTCACA	exonic	NALCN	nonsynonymous SNV	[illegible]
13	111114696	G	T	AGGCCGCGAT,GGCTCGATGG	exonic	COL4A2	nonsynonymous SNV	[illegible]
14	23355934	G	C	ATCCGGCTGC,GCGGGGCCGA	exonic	REM2	nonsynonymous SNV	[illegible]
14	24659776	G	A	AGAAGCAGCA,GATGGTTGTG	exonic	TM9SF1	nonsynonymous SNV	[illegible]
14	24883851	C	T	GACCCTTCCT,CCAGCTCAGC	exonic	NYNRIN	nonsynonymous SNV	[illegible]
14	53251349	G	A	GTCAAACATA,GAGTTTCATC	exonic	GNPNAT1	nonsynonymous SNV	[illegible]
14	66096305	G	A	CTGGTTCAGC,GAGAATAACA	exonic	FUT8	nonsynonymous SNV	[illegible]
14	70512896	C	T	GAACTCCTGT,CCTGCAGAGC	exonic	SLC8A3	nonsynonymous SNV	[illegible]
14	71444165	T	A	CATGGACAGC,TGAGGAGCCT	exonic	PCNX	nonsynonymous SNV	[illegible]
14	73731034	C	T	GATCCAACTT,GCATCATAGG	exonic	PAPLN	nonsynonymous SNV	[illegible]
14	73743761	G	C	CACACCCACT,GCACAGGCTG	exonic	NUMB	nonsynonymous SNV	[illegible]
14	74179328	C	T	GCCTCAGCCT,CTCCTCCTCC	exonic	PNMA1	nonsynonymous SNV	[illegible]
14	76905893	G	A	GCAGGCGCCG,GCTGGGAGGC	exonic	ESRRB	nonsynonymous SNV	[illegible]
14	77492493	G	A	CGGGGGCTCC,GAGAGGCCTT	exonic	IRF2BPL	nonsynonymous SNV	[illegible]
14	77492494	G	A	GGGGGCTCCG,AGAGGCCTTT	exonic	IRF2BPL	nonsynonymous SNV	[illegible]
14	79175727	G	A	TGCCCAAGTG,AACACTAAAC	exonic	NRXN3	stopgain	[illegible]
14	81609488	G	C	AAGAGGATGA,ATCATTGGTT	exonic	TSHR	nonsynonymous SNV	[illegible]
14	88892832	C	T	TTCCCAAATT,CCACCGGTTT	exonic	SPATA7	nonsynonymous SNV	[illegible]
14	88971683	G	A	AGAAAGTCCT,GGATTCATAC	exonic	PTPN21	stopgain	[illegible]
14	89206888	G	A	TCGTTTTGGG,TCAGAGCATT	exonic	EML5	nonsynonymous SNV	[illegible]
14	92470707	C	A	AGAAGCTCCT,AAATTGATTA	exonic	TRIP11	stopgain	[illegible]
14	93408111	C	T	CACCAGGCCG,CCGCCGGCTC	exonic	ITPK1	nonsynonymous SNV	[illegible]
14	93408112	C	T	ACCAGGCCGC,CGCCGGCTCG	exonic	ITPK1	nonsynonymous SNV	[illegible]
14	96864502	G	A	TATGCTGGAA,CTTCCTCAAC	exonic	AK7	nonsynonymous SNV	[illegible]
14	99641674	G	A	GCTGGTGCCG,GCTCGGGGGA	exonic	BCL11B	nonsynonymous SNV	[illegible]
14	100602242	G	A	TCCATTTTAG,AAGATCCTAG	exonic	EVL	nonsynonymous SNV	[illegible]
14	100795187	C	T	TGTCCTGTGC,CCCAGCCTGC	exonic	SLC25A47	nonsynonymous SNV	[illegible]
14	102449915	G	A	GAACAGCTAA,AGCTGTTATC	exonic	DYNC1H1	nonsynonymous SNV	[illegible]
14	104381502	T	C	GGGATCCATA,GTTTTTAATA	exonic	C14orf2	nonsynonymous SNV	[illegible]
14	105206113	G	A	GGCTGTCGAC,GACTTCAGGA	exonic	ADSSL1	nonsynonymous SNV	[illegible]
14	105409562	G	A	TCCAGCTTGG,GCCCTTAACA	exonic	AHNAK2	nonsynonymous SNV	[illegible]
14	105517449	C	T	CTCGGTGTCC,TGCTGTGGGT	exonic	GPR132	nonsynonymous SNV	[illegible]
14	106967219	C	T	AGCCCTTGTC,AGGGGCCTGT	exonic	IGHV1-46	nonsynonymous SNV	[illegible]
15	22848300	G	A	GTAACTGTGA,AAACAATATT	exonic	TUBGCP5	nonsynonymous SNV	[illegible]
15	23889934	C	T	AGAGAGCTCC,TGGGCTTTCA	exonic	MAGEL2	nonsynonymous SNV	[illegible]
15	26825538	C	T	TCAATCCTTT,CACTCCGGTA	exonic	GABRB3	nonsynonymous SNV	[illegible]
15	30012673	G	C	TTCCAGAGGA,TCCTCTCTTA	exonic	TJP1	nonsynonymous SNV	[illegible]
15	34102825	C	T	GAGCTGATCT,CCTCGCAAAA	exonic	RYR3	nonsynonymous SNV	[illegible]
15	40656007	G	A	GGCACTGCAG,AATACCAGGG	exonic	DISP2	nonsynonymous SNV	[illegible]
15	41228857	C	G	GCTGCGGCTT,GACGGCCGGA	exonic	DLL4	nonsynonymous SNV	[illegible]
15	42853605	G	A	TCACAGGTTA,ACTGAAAAGT	exonic	HAUS2	nonsynonymous SNV	[illegible]
15	43020931	C	T	TGGGTCTCCC,CTTCCTCTCC	exonic	CDAN1	nonsynonymous SNV	[illegible]
15	43299420	G	A	CTCCTTTTCA,TAACAGATGG	exonic	UBR1	nonsynonymous SNV	[illegible]
15	45398339	G	A	ATACTCCTTA,GGATCTTGAG	exonic	DUOX2	nonsynonymous SNV	[illegible]
15	45707863	G	A	AGCTCGCTCA,CCAGCAAGAC	exonic	SPATA5L1	nonsynonymous SNV	[illegible]
15	52536625	C	T	GAATTTTTTC,TCTGGAGCCA	exonic	MYO5C	nonsynonymous SNV	[illegible]
15	52575052	C	T	AGCAGACTTC,AAACTTCTTC	exonic	MYO5C	stopgain	[illegible]
15	56134248	C	T	CAAACTCAAT,CACAGTCGAG	exonic	NEDD4	stopgain	[illegible]
15	59185133	G	A	CTTTCAAAAC,AACTAAATGT	exonic	SLTM	nonsynonymous SNV	[illegible]
15	65239645	G	A	TGATCCCTCT,GGAAGAGCTT	exonic	ANKDD1A	nonsynonymous SNV	[illegible]
15	65239646	G	A	GATCCCTCTG,GAAGAGCTTG	exonic	ANKDD1A	nonsynonymous SNV	[illegible]
15	65489359	C	T	AGCGCGATCT,CTTGGCCGTG	exonic	CILP	nonsynonymous SNV	[illegible]
15	65771244	C	T	ACCACTGGAT,GTTTATATTT	exonic	DPP8	nonsynonymous SNV	[illegible]
15	65917302	C	T	TCAGCCCATC,CTGGGGGTTA	exonic	SLC24A1	nonsynonymous SNV	[illegible]
15	68504005	G	A	AGCAGGAGGT,GCCTCACCAG	exonic	CLN6	nonsynonymous SNV	[illegible]
15	71229158	C	T	AACAATATAT,TAGGTAAGTG	exonic	LRRC49	nonsynonymous SNV	[illegible]
15	71507561	C	T	CCTGGCGCCC,TGCGCGCGCC	exonic	THSD4	nonsynonymous SNV	[illegible]
15	75798154	G	A	AACATGTACT,AGTCCCAGTC	exonic	PTPN9	nonsynonymous SNV	[illegible]
15	75968582	C	T	GCGGACCACG,GGCCATGCCG	exonic	CSPG4	nonsynonymous SNV	[illegible]
15	77906642	G	T	TCCCTCGCCC,GCTGGTTGGA	exonic	LINGO1	nonsynonymous SNV	[illegible]
15	78385103	G	A	TCACCCGCAA,TAAGGCTGCT	splicing	SH2D7(ENST	.	.
15	79750498	C	T	CACAGTGGCT,CCACGGACCC	exonic	KIAA1024	nonsynonymous SNV	[illegible]
15	81664993	C	T	GCCATGAGAT,TTTCTGGAAC	exonic	TMC3	nonsynonymous SNV	[illegible]
15	91456873	C	T	TGAAGAAGCT,CCCCTCCAGG	exonic	MAN2A2	nonsynonymous SNV	[illegible]
15	93015412	G	A	TGCTTCAAAG,AAGACTTGCA	exonic	C15orf32	nonsynonymous SNV	[illegible]
16	334730	G	A	GCGGCTGGAG,ACGAGGCGGC	exonic	PDIA2	nonsynonymous SNV	[illegible]
16	675469	C	T	AACCGCTGGT,CTTTGACGGC	exonic	RAB40C	nonsynonymous SNV	[illegible]
16	1634239	G	A	ACACTCCACT,ATGTGCATGT	exonic	IFT140	stopgain	[illegible]
16	2336761	G	A	GACCACAATG,AGGCGTGAGG	exonic	ABCA3	nonsynonymous SNV	[illegible]
16	2339457	G	A	CTTGACAGCG,TGCGCTCCTC	exonic	ABCA3	nonsynonymous SNV	[illegible]
16	3820629	G	A	TGACTGAGGG,TAGCCACAGA	exonic	CREBBP	nonsynonymous SNV	[illegible]
16	4833759	G	A	AATGTCCAGG,GCCGCAGGCT	exonic	SEPT12	nonsynonymous SNV	[illegible]
16	11495252	G	A	CAGGGAGGAG,AGACCAGCAC	exonic	CTD-3088G3.[illegible]	nonsynonymous SNV	[illegible]
16	23698820	G	A	GAGCGTCCCC,GGAAAAAGAA	exonic	PLK1	nonsynonymous SNV	[illegible]
16	27221806	G	A	GCAGCCCTGC,GGTCTGTGAC	exonic	KDM8	nonsynonymous SNV	[illegible]
16	27475713	C	A	GAACTGAACT,ACCGACACCC	exonic	GTF3C1	stopgain	[illegible]
16	28133019	A	T	TTCAATACTG,TGGCACAGCA	exonic	XPO6	nonsynonymous SNV	[illegible]
16	28167590	G	A	CTCCTGACCC,AGACACAGTT	exonic	XPO6	nonsynonymous SNV	[illegible]
16	28506529	G	A	GGAAAGACAG,GAAGATTGTA	exonic	APOBR	nonsynonymous SNV	[illegible]
16	28508043	G	A	CGTCACCAAA,GCCAAGGACC	exonic	APOBR	nonsynonymous SNV	[illegible]
16	28508044	G	A	GTCACCAAAG,CCAAGGACCT	exonic	APOBR	nonsynonymous SNV	[illegible]
16	30566885	G	A	GCAGGGGAAG,GGGTCTCGCC	exonic	ZNF764	nonsynonymous SNV	[illegible]
16	30673936	C	T	CTCCGTGGTC,CTAAAGTGTC	exonic	FBRS	nonsynonymous SNV	[illegible]
16	30939065	G	A	GTCCCGCCTA,GATTCAGGTG	splicing	FBXL19(ENST	.	.
16	30939066	G	A	TCCCGCCTAG,ATTCAGGTGA	exonic	FBXL19	nonsynonymous SNV	[illegible]
16	31473814	C	T	CCTGATTCGG,CTGCACTGGG	exonic	ARMC5	nonsynonymous SNV	[illegible]
16	47533697	C	T	TATCAAAGTC,AACTACCGGT	exonic	PHKB	nonsynonymous SNV	[illegible]
16	48395584	C	T	AGTCGCTATT,ATAATGGCTG	exonic	SIAH1	nonsynonymous SNV	[illegible]
16	49764709	C	T	GCCCGGTGGT,CGTCAGGTCT	exonic	ZNF423	nonsynonymous SNV	[illegible]
16	57415946	G	A	TTGTAGCTTG,AGACGAGACA	exonic	CX3CL1	stopgain	[illegible]
16	66583865	C	T	CGGCGCTGCA,CCTCCGCGGC	exonic	TK2	nonsynonymous SNV	[illegible]
16	67916872	C	T	CTGCAGGAGT,CATTTTAGCA	exonic	EDC4	nonsynonymous SNV	[illegible]
16	70508797	C	T	CAGGGCACGC,GCATCCCGGA	exonic	FUK	nonsynonymous SNV	[illegible]
16	71317837	G	A	AAACAACTGT,GAGTACAAAG	exonic	CMTR2	nonsynonymous SNV	[illegible]
16	75271104	G	A	TGGCTGGGAA,CAGCCCCCGA	exonic	BCAR1	nonsynonymous SNV	[illegible]
16	76486536	G	A	TTCGAACTTG,AATAAGGCAG	exonic	CNTNAP4	stopgain	[illegible]
16	83983109	G	A	GTGCAGACAA,AGGTACGTCG	exonic	OSGIN1	nonsynonymous SNV	[illegible]
16	87445532	C	T	GCCGCAGCTG,CGCTGCAGCC	exonic	ZCCHC14	nonsynonymous SNV	[illegible]
16	87445533	C	T	CCGCAGCTGC,GCTGCAGCCA	exonic	ZCCHC14	nonsynonymous SNV	[illegible]
16	88495029	C	T	CCCGAAAGAC,ACCTTCAGCC	exonic	ZNF469	nonsynonymous SNV	[illegible]
16	88501958	C	T	GCAGCCGCCT,GCTTGGCCAC	exonic	ZNF469	nonsynonymous SNV	[illegible]
16	89259928	G	A	GCAGTCTCGG,GCAAGGGGCT	exonic	CDH15	nonsynonymous SNV	[illegible]
16	89259929	G	A	CAGTCTCGGG,CAAGGGGCTG	exonic	CDH15	nonsynonymous SNV	[illegible]

17	422484	G	A	GGCGGTGCGG,GAGCCGCTGG	exonic	VPS53	nonsynonymous SNV	[illegible]
17	530468	G	A	CCACTCACGT,GAAACATGCG	exonic	VPS53	nonsynonymous SNV	[illegible]
17	722724	C	T	CGGGCCTCAT,CGTGTAGGGG	exonic	NXN	nonsynonymous SNV	[illegible]
17	1386328	G	A	CGCAGTGCTC,GTACACAGTG	exonic	MYO1C	stopgain	[illegible]
17	1555055	G	A	TTATCTGGTG,GCTCACCCCA	exonic	PRPF8	nonsynonymous SNV	[illegible]
17	1563866	C	T	TGAAAGCCTA,ATATACCTGC	exonic	PRPF8	nonsynonymous SNV	[illegible]
17	1637103	G	A	GTGGGTGGCG,GGGCCTGGGC	exonic	WDR81	nonsynonymous SNV	[illegible]
17	4619297	C	T	AGCGGGACTT,GTAGATCACC	exonic	ARRB2	nonsynonymous SNV	[illegible]
17	6900268	G	A	GCAGGGCCCT,GAGCCTGCGC	exonic	ALOX12	nonsynonymous SNV	[illegible]
17	7227199	C	T	GGTGCGGCCC,CGTTAGTGAT	exonic	NEURL4	nonsynonymous SNV	[illegible]
17	7227200	C	T	GTGCGGCCCC,GTTAGTGATG	exonic	NEURL4	nonsynonymous SNV	[illegible]
17	7292168	G	A	GGAGCCACTG,GGGAGATGTG	exonic	TNK1	nonsynonymous SNV	[illegible]
17	7578454	G	A	GATGGCCATG,CGCGGACGCG	exonic	TP53	nonsynonymous SNV	[illegible]
17	7578492	C	T	TGGAATCAAC,CACAGCTGCA	exonic	TP53	stopgain	[illegible]
17	7759179	G	T	AGCGTCAGTG,ACCCAGTGCT	exonic	TMEM88	nonsynonymous SNV	[illegible]
17	8926373	C	G	GGGCGGCCCT,GGCGCACGAC	exonic	NTN1	nonsynonymous SNV	[illegible]
17	10346844	C	T	GATTGTTCCT,CTGAGAACAG	exonic	MYH4	nonsynonymous SNV	[illegible]
17	13503902	C	T	GGGCTCGGCG,CCACGGCGCG	exonic	HS3ST3A1	nonsynonymous SNV	[illegible]
17	17968502	C	T	AGGTATCAGT,CCTCAATCTA	exonic	GID4	nonsynonymous SNV	[illegible]
17	18058708	G	A	AGGGTGGCCA,GAGGCCGGCG	exonic	MYO15A	nonsynonymous SNV	[illegible]
17	18058709	G	A	GGGTGGCCAG,AGGCCGGCGG	exonic	MYO15A	nonsynonymous SNV	[illegible]
17	19835233	A	G	AACCGAATGG,TGAGATCATT	exonic	AKAP10	nonsynonymous SNV	[illegible]
17	20135655	T	C	AATGCCCGGT,GCAGAAGGAG	exonic	SPECC1	nonsynonymous SNV	[illegible]
17	27015220	G	A	GATCGACACG,CCTCCCTGGG	exonic	SUPT6H	nonsynonymous SNV	[illegible]
17	30796119	C	T	AGCAACCTGC,GAAAGCCCGA	exonic	PSMD11	nonsynonymous SNV	[illegible]
17	32959879	C	T	CTCAGATGCC,GCCTCAGCCA	exonic	TMEM132E	nonsynonymous SNV	[illegible]
17	33520927	G	A	GAACCTTTGC,AACAGTGGCA	exonic	SLC35G3	nonsynonymous SNV	[illegible]
17	33520981	C	T	TAGGCACATC,AATGCTGAGG	exonic	SLC35G3	nonsynonymous SNV	[illegible]
17	36689611	G	A	GGGCTTTTCT,AGACTGGGCT	exonic	SRCIN1	nonsynonymous SNV	[illegible]
17	37619219	C	A	CAGTAGGCGA,AGAGATCTGT	exonic	CDK12	nonsynonymous SNV	[illegible]
17	37687337	C	T	GCGTTACACC,GGTGGTCGGG	exonic	CDK12	nonsynonymous SNV	[illegible]
17	39036435	C	T	TTCATGATGA,GCCAAGGTTC	exonic	KRT20	nonsynonymous SNV	[illegible]
17	40695259	G	A	AACTTTGGGG,AAACCATGGT	exonic	NAGLU	nonsynonymous SNV	[illegible]
17	42083279	G	A	CCCCATGCCA,GTGAGTGCCC	exonic	NAGS	nonsynonymous SNV	[illegible]
17	42083280	G	A	CCCATGCCAG,TGAGTGCCCG	splicing	NAGS(ENST0	.	[illegible]
17	42437425	C	T	AGTCCGGTTC,CAAACACATC	exonic	FAM171A2	nonsynonymous SNV	[illegible]
17	42437426	C	T	GTCCGGTTCC,AAACACATCC	exonic	FAM171A2	nonsynonymous SNV	[illegible]
17	42476087	C	T	AGCTTGGGCC,AGCTTTATTA	exonic	GPATCH8	nonsynonymous SNV	[illegible]
17	43190282	G	A	AAGTCATTGG,GGAGGTGGCG	exonic	PLCD3	nonsynonymous SNV	[illegible]
17	47583970	C	T	TGCGAGGACA,CGAGCGCCAG	exonic	NGFR	nonsynonymous SNV	[illegible]
17	56598997	T	C	ATGCTCAATC,CCTCCCGGAT	exonic	SEPT4	nonsynonymous SNV	[illegible]
17	56688666	A	G	CAGGTGCACA,TCACCTCCAT	exonic	TEX14	nonsynonymous SNV	[illegible]
17	57058190	C	A	AAGTTTTATT,ATTTCTCTCT	exonic	PPM1E	stopgain	[illegible]
17	66364691	C	G	TGTTTCAGCA,CAGCGGGAGG	exonic	ARSG	nonsynonymous SNV	[illegible]
17	67250002	T	A	ATCAAGTGCA,GTGTTATTCT	exonic	ABCA5	nonsynonymous SNV	[illegible]
17	72443099	C	T	CCAGAGCCAC,AGGCGGCCAC	exonic	GPRC5C	stopgain	[illegible]
17	73900630	C	T	CCTGTCTTCT,CCCGAAATAC	exonic	MRPL38	nonsynonymous SNV	[illegible]
17	74004146	A	T	TGCAGCTGCA,GTACTGGCCC	exonic	EVPL	nonsynonymous SNV	[illegible]
17	74943948	G	A	CCTACCAGAG,CCCACGCCCC	exonic	MGAT5B	nonsynonymous SNV	[illegible]
17	76888409	C	T	TGGCTCCATC,ATGTTTCTGT	exonic	DDC8	nonsynonymous SNV	[illegible]
17	78409932	G	A	GGAACGTCTA,CACTTTGTTG	splicing	ENDOV(ENST	.	[illegible]
17	79060351	C	T	CAGCAAGAAT,CTCAGAAGTA	exonic	BAIAP2	nonsynonymous SNV	[illegible]
17	79095328	G	A	TCCCTCCTGG,ATGGGGAGGG	exonic	AATK	nonsynonymous SNV	[illegible]
17	79781268	C	T	AGCCCTGCCC,CAGGTCAGCC	exonic	FAM195B	stopgain	[illegible]
18	8244102	G	A	CACCCGACAG,AGATGACTGT	exonic	PTPRM	nonsynonymous SNV	[illegible]
18	8818982	C	T	GAACAGTCCC,GGAGAGGTGG	exonic	SOGA2	nonsynonymous SNV	[illegible]
18	10736669	C	T	CTCCTCTTCA,TATCCGTTTC	exonic	PIEZO2	nonsynonymous SNV	[illegible]
18	10803971	C	T	TTGTCCTCTT,TTTGGTCCCC	exonic	PIEZO2	nonsynonymous SNV	[illegible]
18	12535504	A	G	TTGGTCAGAA,TGTATGGAGC	exonic	SPIRE1	nonsynonymous SNV	[illegible]
18	19263903	C	T	CCCCGCCACT,CTCTGTTGTT	exonic	ABHD3	nonsynonymous SNV	[illegible]
18	21228899	A	C	AGTTCACCAC,GTACCTTGGA	exonic	ANKRD29	nonsynonymous SNV	[illegible]
18	29126581	G	A	CACGGTGTCT,GAGCTGGAGT	exonic	DSG2	nonsynonymous SNV	[illegible]
18	43496048	C	T	CACAAGTGGT,CATGAGGAAG	exonic	EPG5	nonsynonymous SNV	[illegible]
18	46284715	G	A	ATCGGGGAGC,GCCCAAAATT	exonic	CTIF	nonsynonymous SNV	[illegible]
18	72343724	G	A	GATCTTTGTG,TTTTCAGTGT	exonic	ZNF407	nonsynonymous SNV	[illegible]
18	72346477	T	A	TTGTGAGACT,TCCAACAGGC	exonic	ZNF407	nonsynonymous SNV	[illegible]
18	77474782	C	T	GGACAGCGGC,TGCCCAGGGT	exonic	CTDP1	nonsynonymous SNV	[illegible]
18	77896102	G	A	CGTCCATTTG,TGCGCTGCAG	exonic	ADNP2	nonsynonymous SNV	[illegible]
19	581398	G	A	ACCTGAACAT,GAGGCCGACC	exonic	BSG	nonsynonymous SNV	[illegible]
19	581399	G	A	CCTGAACATG,AGGCCGACCC	exonic	BSG	nonsynonymous SNV	[illegible]
19	620456	G	A	CGTCTGCCAG,GTTCCTCCGC	exonic	POLRMT	nonsynonymous SNV	[illegible]
19	620457	G	A	GTCTGCCAGG,TTCCTCCGCG	exonic	POLRMT	nonsynonymous SNV	[illegible]
19	847863	G	A	TTCGTGATCT,GGGATGTGCC	exonic	PRTN3	stopgain	[illegible]
19	880044	C	T	ATGGCCGGCT,ATCCACAGGC	exonic	MED16	nonsynonymous SNV	[illegible]
19	984465	C	T	CCGCGGCGGC,AGGCGGGACC	exonic	WDR18	stopgain	[illegible]
19	3179635	C	T	AACCTCTGGG,CCAGGAGTAC	exonic	S1PR4	nonsynonymous SNV	[illegible]
19	5921239	C	T	GTGCCGTCAT,GGTGGACGCC	exonic	RANBP3	nonsynonymous SNV	[illegible]
19	6937382	G	A	AGCCTGCAGG,GGCCTTCATC	exonic	EMR1	nonsynonymous SNV	[illegible]
19	7591443	C	T	ACCTTCGCAG,CTACACGCGG	exonic	MCOLN1	nonsynonymous SNV	[illegible]
19	7677058	C	T	GCCACCAACT,CGAGGTGAAA	exonic	CAMSAP3	nonsynonymous SNV	[illegible]
19	7743241	G	A	AACAGATGCT,AGATGTCCAA	exonic	C19orf59	nonsynonymous SNV	[illegible]
19	8131038	C	T	GCCCTCTAGA,CCTCCAGGGC	exonic	FBN3	nonsynonymous SNV	[illegible]
19	8131039	C	T	CCCTCTAGAC,CTCCAGGGCC	exonic	FBN3	nonsynonymous SNV	[illegible]
19	9027576	C	T	TGGGTGAAAC,TGCACAGAGA	splicing	MUC16(ENST	.	[illegible]
19	9077142	C	T	GATGTCACTC,CAGATGTCTG	exonic	MUC16	nonsynonymous SNV	[illegible]
19	9077508	G	A	TAGGGAGACA,AGGACATTGT	exonic	MUC16	nonsynonymous SNV	[illegible]
19	9271424	G	A	ACGCAGTGCG,CAAAGCCTTC	exonic	ZNF317	nonsynonymous SNV	[illegible]
19	9967523	G	A	AGTCATCCAG,AGCCGAAGCG	exonic	OLFM2	nonsynonymous SNV	[illegible]
19	10084287	C	A	TCTCCAGCGT,CCCTCGCTCA	exonic	COL5A3	nonsynonymous SNV	[illegible]
19	10577840	C	T	GAGGCATTGA,TGCGCAGGGA	exonic	PDE4A	nonsynonymous SNV	[illegible]
19	10745846	G	A	TAGGGCTGTG,GATACGTCAT	exonic	SLC44A2	nonsynonymous SNV	[illegible]
19	10791947	G	A	AACGCCCAAT,GAGGAGGACG	exonic	ILF3	nonsynonymous SNV	[illegible]
19	11287408	C	T	GGAACCCTCT,CCTCGGCTCT	exonic	KANK2	nonsynonymous SNV	[illegible]
19	12858905	C	T	TACAAGCCCC,CAGTGCCCAG	exonic	ASNA1	nonsynonymous SNV	[illegible]
19	13029042	G	A	TCACCTAGCT,GCCCTCCACC	exonic	SYCE2	nonsynonymous SNV	[illegible]
19	13183923	G	A	ACTGCCCAAC,GTCAGTGCCC	exonic	NFIX	nonsynonymous SNV	[illegible]
19	13183924	G	A	CTGCCCAACG,TCAGTGCCCC	splicing	NFIX(ENST00	.	[illegible]
19	13882970	C	T	CGATGTCACC,CCCACGACCT	exonic	MRI1	nonsynonymous SNV	[illegible]
19	14000012	G	A	TGCTCAGGTG,GGCTTCGAAG	exonic	C19orf57	nonsynonymous SNV	[illegible]
19	16345235	C	T	TTACCAGCTC,GGACCCAGTG	exonic	AP1M1	nonsynonymous SNV	[illegible]
19	16643929	C	A	TGCTGCTCCA,CGCGTGGGCC	exonic	CHERP	nonsynonymous SNV	[illegible]
19	17306101	G	A	GGAAAAGCCC,ACAGCCCCGG	exonic	MYO9B	nonsynonymous SNV	[illegible]
19	17651335	G	A	TCAAGGCATG,ACCGACTGTC	exonic	FAM129C	nonsynonymous SNV	[illegible]
19	17932258	C	T	GGGCCCAACG,GAACACCAGG	exonic	INSL3	nonsynonymous SNV	[illegible]
19	18183003	A	G	GCCACGTTGT,GGCAGCACCC	exonic	IL12RB1	nonsynonymous SNV	[illegible]
19	18377602	G	A	AGACTCACTG,GCAGGGAAAT	exonic	KIAA1683	nonsynonymous SNV	[illegible]
19	18778743	C	T	AGCCTGGCCT,GCTGCGAGAG	exonic	KLHL26	nonsynonymous SNV	[illegible]
19	19749039	G	A	GAGGCCTGGG,GGCCGAGTCC	exonic	GMIP	nonsynonymous SNV	[illegible]
19	30020936	G	A	CCAGAGCAGC,GCCCGCGTCG	exonic	VSTM2B	nonsynonymous SNV	[illegible]
19	31039011	G	A	GTCAAGCAAA,CTTCTCTGTT	exonic	ZNF536	nonsynonymous SNV	[illegible]
19	33651395	C	T	CGGAAGCTCT,TTTGAAGGTA	exonic	WDR88	nonsynonymous SNV	[illegible]
19	38380461	C	T	CGACATAGAT,GTCCCTCCAT	exonic	WDR87	nonsynonymous SNV	[illegible]
19	38655126	C	T	CAGGGAGAAC,TCAATACTCA	exonic	SIPA1L3	nonsynonymous SNV	[illegible]
19	38713285	G	A	TGCGCTCGGC,CACAGGCGCG	exonic	DPF1	nonsynonymous SNV	[illegible]
19	38855728	C	T	GCTCTGCTTC,AGGAAACACA	exonic	CATSPERG	stopgain	[illegible]
19	39663841	C	T	CCCAAGTCTT,CAGGGAGGGC	exonic	PAK4	nonsynonymous SNV	[illegible]
19	39664244	G	A	GCCCCTAACG,GCCATCAGCG	exonic	PAK4	nonsynonymous SNV	[illegible]
19	40885535	C	T	CTGGTGGCCC,CCGGGGTGGA	exonic	HIPK4	nonsynonymous SNV	[illegible]
19	41095056	T	C	CAGCCAGCTC,TCGTGCGTCT	exonic	SHKBP1	nonsynonymous SNV	[illegible]
19	41206222	C	T	TACCTGGTTC,GCAGGTCCTG	exonic	ADCK4	nonsynonymous SNV	[illegible]
19	41351256	C	T	GGGCCAAACT,ATGGGGATCA	exonic	CYP2A6	nonsynonymous SNV	[illegible]
19	42409938	C	T	CTCAGCAGCT,TGAGAACGGC	exonic	ARHGEF1	nonsynonymous SNV	[illegible]
19	42489321	C	T	GTGGCCACCA,CACGCCCCGA	exonic	ATP1A3	nonsynonymous SNV	[illegible]
19	42626527	G	A	ATTTCTTTCG,TGTCTGCAAA	exonic	POU2F2	nonsynonymous SNV	[illegible]
19	43098941	G	A	CCCTGCCAGG,GATGCGCCAT	exonic	CEACAM8	nonsynonymous SNV	[illegible]
19	44278599	C	T	GCCCAGGAAT,CCGGCCAGGG	exonic	KCNN4	nonsynonymous SNV	[illegible]
19	44571348	C	A	GAAAATCCAT,CAAATGTGAA	exonic	ZNF223	nonsynonymous SNV	[illegible]
19	45116968	C	T	GAGAGCAAAA,CTCAGAGCCC	exonic	IGSF23	nonsynonymous SNV	[illegible]
19	45314577	G	A	GGGAACCCAC,ACCATTATAT	exonic	BCAM	nonsynonymous SNV	[illegible]
19	45716397	G	A	ACTGGGCCTG,CCAGCCGGGA	exonic	EXOC3L2	nonsynonymous SNV	[illegible]
19	47104220	C	T	GCTCTCATTT,CGCCATGGGC	exonic	PPP5D1	nonsynonymous SNV	[illegible]
19	47127307	G	A	CAGCAGGTCG,TGGCCGCCAG	exonic	PTGIR	nonsynonymous SNV	[illegible]
19	48024755	G	A	TCTCTGCATG,AAAAATGCCC	exonic	ZNF541	nonsynonymous SNV	[illegible]
19	48248938	C	T	CGGCGAGGCC,AAGAAATAAG	exonic	GLTSCR2	nonsynonymous SNV	[illegible]
19	48258678	C	T	GGGATCAAGG,CCAGGTGGCC	exonic	GLTSCR2	nonsynonymous SNV	[illegible]
19	48657195	G	A	AGGACTTCCT,AATGGTCCGT	exonic	LIG1	stopgain	[illegible]
19	49001852	G	A	GGGTGGCTCG,GGGGAGCCCG	exonic	LMTK3	nonsynonymous SNV	[illegible]
19	49142659	C	T	GGTGATGAAG,CGAAGGATTC	exonic	CA11	nonsynonymous SNV	[illegible]
19	49422320	G	A	GAATGAGGAG,ACGACATGCG	exonic	NUCB1	nonsynonymous SNV	[illegible]
19	49507467	C	T	TCACTAGCTT,CTGATGTGGT	exonic	RUVBL2	nonsynonymous SNV	[illegible]
19	49642992	C	A	GGAGACACCC,ACCACCCACT	exonic	PPFIA3	nonsynonymous SNV	[illegible]
19	49933886	C	T	GCCTCATCCT,CATTTCGCTG	exonic	SLC17A7	nonsynonymous SNV	[illegible]
19	50169132	C	T	TTTGGGTTTC,GGCCAGAGGC	exonic	BCL2L12	nonsynonymous SNV	[illegible]
19	50376204	C	T	AGCTGCTCCC,GGAGGTCTCT	exonic	AKT1S1	nonsynonymous SNV	[illegible]
19	50384681	G	A	TTCCACCGCG,GGGCACCCGC	exonic	TBC1D17	nonsynonymous SNV	[illegible]
19	50909668	T	G	ACCTAGGTGC,GCTGCGGGAG	exonic	POLD1	nonsynonymous SNV	[illegible]
19	51165511	C	T	CAAGGCTCCC,CGAGCCCCCC	exonic	SHANK1	nonsynonymous SNV	[illegible]
19	51919268	G	A	CAGGGGTCTA,GGCCCCAGGG	exonic	SIGLEC10	nonsynonymous SNV	[illegible]
19	52034755	C	T	CTGGAATCTC,GCTCCTGAGC	exonic	SIGLEC6	nonsynonymous SNV	[illegible]
19	54515342	G	A	CGTGGGGGCC,GCCTGATCCT	exonic	CACNG6	nonsynonymous SNV	[illegible]
19	54515343	G	A	GTGGGGGCCG,CCTGATCCTG	exonic	CACNG6	nonsynonymous SNV	[illegible]
19	54849295	G	T	ATGTACCCCT,TTGCTGAAGG	exonic	LILRA4	nonsynonymous SNV	[illegible]
19	55697253	C	T	AGTTCCCGCA,CGTCCAGTTC	exonic	PTPRH	nonsynonymous SNV	[illegible]
19	55816036	G	A	TGGGGGCGCC,GGGAGCAGCC	exonic	BRSK1	nonsynonymous SNV	[illegible]
19	55816037	G	A	GGGGGCGCCG,GGAGCAGCCC	exonic	BRSK1	nonsynonymous SNV	[illegible]
19	56041287	C	T	AGGGCGGTCC,GGGGCTGGCC	exonic	SBK2	nonsynonymous SNV	[illegible]
19	57326626	C	T	CCTTGAGACT,CTCGCCATGA	exonic	PEG3	nonsynonymous SNV	[illegible]
19	58453923	A	T	TTCTGGGGAG,AGGAAGGGCC	exonic	ZNF256	nonsynonymous SNV	[illegible]
19	58982676	C	T	CTCCGACCTC,TCAAGCACCT	exonic	ZNF324	nonsynonymous SNV	[illegible]
20	1162021	A	T	TGAGCTGCCC,GTCTCGTGTT	exonic	TMEM74B	nonsynonymous SNV	[illegible]
20	1234929	G	A	AACAAGATGG,AATGCAGTCC	exonic	RAD21L1	nonsynonymous SNV	[illegible]
20	1961247	G	A	TCCTCAGCGA,ATAGAGTGTG	exonic	PDYN	nonsynonymous SNV	[illegible]
20	3682253	C	T	GCCTGGGTCT,CAGGAAGGCT	exonic	SIGLEC1	nonsynonymous SNV	[illegible]
20	4713225	G	A	AGGTTTGCTA,AGAAGGGAAG	exonic	PRNT	nonsynonymous SNV	[illegible]
20	10036202	G	A	AGGGAACTAC,GCGAGAGAGG	exonic	ANKEF1	nonsynonymous SNV	[illegible]
20	17505503	C	T	CTTGTTAAGC,GTTCAAGCAT	exonic	BFSP1	nonsynonymous SNV	[illegible]
20	18407737	C	T	AGGGCCCCCT,ACAGGTGACA	exonic	DZANK1	nonsynonymous SNV	[illegible]
20	25062504	C	T	AGCGGTAGGG,CCCACGCGCC	exonic	VSX1	nonsynonymous SNV	[illegible]
20	31828131	A	C	GAGAGGATCC,CCTGGTCCTT	exonic	BPIFA1	nonsynonymous SNV	[illegible]
20	43353480	G	A	CGAGGACGGC,GCTTCACCTG	exonic	WISP2	nonsynonymous SNV	[illegible]
20	50407707	G	A	AACAGCTGGG,GTTTGCCTTC	exonic	SALL4	nonsynonymous SNV	[illegible]
20	51871015	T	C	CACAGGATCT,TTGCAGATTC	exonic	TSHZ2	nonsynonymous SNV	[illegible]
20	53226976	G	A	CCGAGACGGG,AGGCCATCTA	exonic	DOK5	nonsynonymous SNV	[illegible]
20	61542633	G	A	GCTGCCCTCG,AGGCTGTCTC	exonic	DIDO1	nonsynonymous SNV	[illegible]
20	61598723	G	A	GCTACTTGAT,GAGACCACGG	exonic	SLC17A9	nonsynonymous SNV	[illegible]
20	61909556	G	A	CCTATCAAGG,TAAGGACTTG	splicing	ARFGAP1(EN	.	[illegible]
20	62071009	C	T	AAGGAGCCTG,CGTTCCAGGT	exonic	KCNQ2	nonsynonymous SNV	[illegible]
20	62187333	G	A	ATCGACCCCT,GAAGTTCCAG	exonic	C20orf195	stopgain	[illegible]
21	27254063	G	A	GCGCTCCTCT,GGGTGACAGC	exonic	APP	nonsynonymous SNV	[illegible]
21	27254064	G	A	CGCTCCTCTG,GGTGACAGCG	exonic	APP	nonsynonymous SNV	[illegible]
21	30426498	G	A	TTTTATGAGA,AATACTGTAA	exonic	USP16	nonsynonymous SNV	[illegible]
21	34954266	G	T	CAGCTTGATA,AAAGTATGTC	exonic	DONSON	nonsynonymous SNV	[illegible]
21	35237625	A	C	CCAGACTTCA,GGAGTTCGTC	exonic	ITSN1	nonsynonymous SNV	[illegible]
21	35772031	C	T	ACCACCTACT,CCCTTGGAGG	exonic	SMIM11	nonsynonymous SNV	[illegible]
21	38997477	T	G	TTTGGATTCA,TCTCCAGGTT	exonic	KCNJ6	nonsynonymous SNV	[illegible]
21	46321471	A	C	GTTTCCGGAA,TCAGCTGCTT	exonic	ITGB2	nonsynonymous SNV	[illegible]
21	47685952	G	A	ACGAGGGACG,GGGGCAATGG	exonic	MCM3AP	nonsynonymous SNV	[illegible]
21	47975943	C	T	CCCCCCTCCC,CGATGTCCTC	exonic	DIP2A	nonsynonymous SNV	[illegible]
22	18022533	C	T	CCCAGGTGTG,CTTACCACCC	exonic	CECR2	nonsynonymous SNV	[illegible]
22	18029221	G	A	AGTGGAATAA,AGGACCTTTC	exonic	CECR2	nonsynonymous SNV	[illegible]
22	19711526	C	T	GACCGCCTTC,CTGTCGACAC	exonic	GP1BB	nonsynonymous SNV	[illegible]
22	19711527	C	T	ACCGCCTTCC,TGTCGACACA	exonic	GP1BB	nonsynonymous SNV	[illegible]
22	20126746	C	T	GCTGTGTCCC,AGCTGTTCCC	exonic	ZDHHC8	nonsynonymous SNV	[illegible]
22	20130939	G	A	GCTGTCCGAG,GCCCCCGAGG	exonic	ZDHHC8	nonsynonymous SNV	[illegible]
22	22057670	C	T	TATTTCCACC,GAGCGTGGTC	exonic	YPEL1	nonsynonymous SNV	[illegible]
22	22599475	A	G	TACAGCGTCT,CTGGTACCAG	exonic	VPREB1	nonsynonymous SNV	[illegible]
22	22681937	C	T	GTCACCATCT,CTGCACTGGG	exonic	IGLV1-50	nonsynonymous SNV	[illegible]
22	23401654	C	T	GGTTTGAACT,GATGACACTG	exonic	RTDR1	nonsynonymous SNV	[illegible]
22	32072847	G	A	CTCCAATATG,ATAGCTTCTC	exonic	PRR14L	nonsynonymous SNV	[illegible]
22	37603328	G	A	CACCACCACG,CTGAGGCCAC	exonic	SSTR3	nonsynonymous SNV	[illegible]
22	38111929	C	T	TGCTGCAGGC,AGAAAAAGGA	exonic	TRIOBP	stopgain	[illegible]
22	39813792	C	T	AGGGAAATTT,GGGAGGGGCC	exonic	TAB1	nonsynonymous SNV	[illegible]
22	40082367	G	A	CCGCTCCCCG,AGAGCTGGAG	exonic	CACNA1I	nonsynonymous SNV	[illegible]
22	41573434	C	T	CCAGGTGACC,CTCCAACCCC	exonic	EP300	nonsynonymous SNV	[illegible]
22	45132886	C	T	ACCTTCAGGT,CTCCCCGGCG	exonic	PRR5	nonsynonymous SNV	[illegible]
22	45283973	G	A	CTTGCAGGCG,CACAGTGCTC	exonic	PHF21B	nonsynonymous SNV	[illegible]
22	46653273	C	T	ATGATACAAC,CTCATCAACA	exonic	PKDREJ	nonsynonymous SNV	[illegible]
22	50656623	G	A	CTCACCTGAA,ACGGCCTTGT	exonic	TUBGCP6	nonsynonymous SNV	[illegible]
22	50752869	G	T	TGTGGGGCCA,TGCTGGAGTG	exonic	DENND6B	nonsynonymous SNV	[illegible]
22	50988331	C	T	GTCCTCAGTC,ATTCATCCTG	exonic	KLHDC7B	nonsynonymous SNV	[illegible]
X	3238084	G	A	GAAAGGCTTT,GTTTTCCTGT	exonic	MXRA5	nonsynonymous SNV	[illegible]
X	3238085	G	A	AAAGGCTTTG,TTTTCCTGTT	exonic	MXRA5	nonsynonymous SNV	[illegible]
X	3240691	G	A	TGATGTACTG,AGTCATTAAC	exonic	MXRA5	nonsynonymous SNV	[illegible]
X	10102556	G	A	CCGGGCCCGA,GGTCGCCTTT	exonic	WWC3	nonsynonymous SNV	[illegible]
X	12736172	T	G	AGTCAACACC,GAGCACTTTT	exonic	FRMPD4	nonsynonymous SNV	[illegible]
X	[illegible]	[illegible]	[illegible]	[illegible]	exonic	PIR	[illegible]	[illegible]
X	[illegible]	[illegible]	[illegible]	[illegible]	exonic	CA5B	[illegible]	[illegible]
X	[illegible]	[illegible]	[illegible]	[illegible]	exonic	RAI2	[illegible]	[illegible]
X	[illegible]	[illegible]	[illegible]	[illegible]	exonic	MAP7D2	[illegible]	[illegible]
X	[illegible]	[illegible]	[illegible]	[illegible]	exonic	KLHL34	[illegible]	[illegible]
X	[illegible]	[illegible]	[illegible]	[illegible]	exonic	KLHL34	[illegible]	[illegible]
X	[illegible]	[illegible]	[illegible]	[illegible]	exonic	ZNF645	[illegible]	[illegible]
X	[illegible]	[illegible]	[illegible]	[illegible]	exonic	MAGEB6	[illegible]	[illegible]
X	[illegible]	[illegible]	[illegible]	[illegible]	exonic	KRBOX4	[illegible]	[illegible]
X	[illegible]	[illegible]	[illegible]	[illegible]	exonic	ARAF	[illegible]	[illegible]
X	[illegible]	[illegible]	[illegible]	[illegible]	exonic	TBC1D25	[illegible]	[illegible]
X	[illegible]	[illegible]	[illegible]	[illegible]	exonic	CCDC22	[illegible]	[illegible]
X	[illegible]	[illegible]	[illegible]	[illegible]	exonic	TSPYL2	[illegible]	[illegible]
X	[illegible]	[illegible]	[illegible]	[illegible]	exonic	HUWE1	[illegible]	[illegible]
X	[illegible]	[illegible]	[illegible]	[illegible]	exonic	HUWE1	[illegible]	[illegible]

X	55513856	G	A	GTCTATGGTG,TAGAAACACT	exonic	USP51	nonsynonymous SNV	USP51:ENST00000500968.3:exon2:c.C1517T:p.T506I,	Xp11.21	.
X	65253538	G	A	ATGGTGACAG,GTCTGAGCCA	exonic	VSIG4	nonsynonymous SNV	VSIG4:ENST00000455586.2:exon2:c.C190T:p.P64S,VSIG4:ENST00000412866.2:exon2:c.C190T:p.P64S,VSIG4:ENST00000374737.4:exon2:c.C190T:p.P64S,	Xq12	VSIG4;basechange=c.190C>T;AAchange=p.P64S;tissue=prostate
X	68060435	C	T	CTACGGGCAC,CTGTCTACAT	exonic	EFNB1	nonsynonymous SNV	EFNB1:ENST00000204961.4:exon5:c.C979T:p.P327S,	Xq13.1	.
X	69455964	A	T	GTCTGGACCC,CATCAGGGAC	exonic	AWAT1	nonsynonymous SNV	AWAT1:ENST00000374521.3:exon3:c.A230T:p.H77L,	Xq13.1	.
X	69502410	T	C	ATCAGCCGCT,CATGTACAAC	exonic	RAB41	nonsynonymous SNV	RAB41:ENST00000276066.4:exon2:c.T149C:p.F50S,RAB41:ENST00000374473.2:exon2:c.T152C:p.F51S,	Xq13.1	.
X	70607132	C	T	CAACCTTTTT,GTGCTCCAAT	exonic	TAF1	nonsynonymous SNV	TAF1:ENST00000276072.3:exon15:c.C2308T:p.R770C,TAF1:ENST00000373790.4:exon15:c.C2245T:p.R749C,TAF1:ENST00000449580.1:exon15:c.C2245T:p.R749C,TAF1:ENST00000423759.1:exon15:c.C2308T:p.R770C,	Xq13.1	.
X	70777487	A	G	CAGGAAGGCT,TTGCTGAGAG	exonic	OGT	nonsynonymous SNV	OGT:ENST00000373701.3:exon12:c.A1537G:p.I513V,OGT:ENST00000373719.3:exon12:c.A1567G:p.I523V,	Xq13.1	.
X	71360399	C	T	AGGCAAGATT,CACCTCCCGT	exonic	NHSL2	nonsynonymous SNV	NHSL2:ENST00000510661.1:exon3:c.C2308T:p.P770S,NHSL2:ENST00000535692.1:exon3:c.C1903T:p.P635S,NHSL2:ENST00000373677.1:exon2:c.C1903T:p.P635S,NHSL2:ENST00000540800.1:exon6:c.C3001T:p.P1001S,	Xq13.1	.
X	71829504	G	A	AGGTTCTGGA,GAAGTCCTAC	exonic	PHKA1	nonsynonymous SNV	PHKA1:ENST00000541944.1:exon22:c.C2399T:p.P800L,PHKA1:ENST00000373542.4:exon23:c.C2576T:p.P859L,PHKA1:ENST00000373545.3:exon22:c.C2399T:p.P800L,PHKA1:ENST00000339490.3:exon23:c.C2576T:p.P859L,PHKA1:ENST00000373539.3:exon23:c.C2576T:p.P859L,	Xq13.2	.
X	71829505	G	A	GGTTCTGGAG,AAGTCCTACT	exonic	PHKA1	nonsynonymous SNV	PHKA1:ENST00000541944.1:exon22:c.C2398T:p.P800S,PHKA1:ENST00000373542.4:exon23:c.C2575T:p.P859S,PHKA1:ENST00000373545.3:exon22:c.C2398T:p.P800S,PHKA1:ENST00000339490.3:exon23:c.C2575T:p.P859S,PHKA1:ENST00000373539.3:exon23:c.C2575T:p.P859S,	Xq13.2	.
X	77284769	G	A	AGTATCTCCC,AACAGAAACG	exonic	ATP7A	nonsynonymous SNV	ATP7A:ENST00000341514.6:exon15:c.G2939A:p.R980Q,ATP7A:ENST00000343533.5:exon14:c.G2705A:p.R902Q,	Xq21.1	.
X	82763523	G	A	CTGAGCGACG,GGGCCCATGG	exonic	POU3F4	nonsynonymous SNV	POU3F4:ENST00000373200.2:exon1:c.G191A:p.G64E,	Xq21.1	POU3F4;basechange=c.191G>C;AAchange=p.G64A;tissue=lung&POU3F4_ENST00000373200;basechange=c.191G
X	99663114	A	G	CTGCACGCCA,AGCTTCCTGA	exonic	PCDH19	nonsynonymous SNV	PCDH19:ENST00000255531.7:exon1:c.T482C:p.F161S,PCDH19:ENST00000420881.2:exon1:c.T482C:p.F161S,PCDH19:ENST00000373034.4:exon1:c.T482C:p.F161S,	Xq22.1	.
X	100744066	G	A	GAAAGAAGCA,TGACACAGAC	exonic	ARMCX4	nonsynonymous SNV	ARMCX4:ENST00000354842.4:exon6:c.G766A:p.V256M,ARMCX4:ENST00000452188.1:exon6:c.G802A:p.V268M,ARMCX4:ENST00000433011.1:exon9:c.G802A:p.V268M,ARMCX4:ENST00000455331.1:exon6:c.G766A:p.V256M,ARMCX4:ENST00000423738.3:exon2:c.G490A:p.V164M,	Xq22.1	.
X	100880102	G	A	TGGATCTGGG,ATGTGGATGA	exonic	ARMCX3	nonsynonymous SNV	ARMCX3:ENST00000341189.4:exon5:c.G133A:p.D45N,ARMCX3:ENST00000471229.2:exon5:c.G133A:p.D45N,ARMCX3:ENST00000491568.2:exon5:c.G133A:p.D45N,ARMCX3:ENST00000537169.1:exon5:c.G133A:p.D45N,ARMCX3:ENST00000479298.1:exon4:c.G133A:p.D45N,	Xq22.1	.
X	107925050	C	T	GATGCTGGTC,TCCAGGAATC	exonic	COL4A5	nonsynonymous SNV	COL4A5:ENST00000328300.6:exon47:c.C4148T:p.P1383L,COL4A5:ENST00000361603.2:exon45:c.C4130T:p.P1377L,	Xq22.3	.
X	109694786	C	A	TTAATGTCAT,TCCAGGCTCT	exonic	RGAG1	nonsynonymous SNV	RGAG1:ENST00000465301.2:exon3:c.C941A:p.S314Y,RGAG1:ENST00000540313.1:exon1:c.C941A:p.S314Y,	Xq23	.
X	110951490	G	A	AATGCATAAA,GATGGAAAAA	exonic	ALG13	nonsynonymous SNV	ALG13:ENST00000495283.1:exon4:c.G307A:p.G103R,ALG13:ENST00000394780.3:exon4:c.G619A:p.G207R,ALG13:ENST00000486353.1:exon4:c.G619A:p.G207R,ALG13:ENST00000251943.4:exon4:c.G307A:p.G103R,	Xq23	.
X	114398339	T	C	AACTGTTCTT,CTCAAGTCTG	exonic	LRCH2	nonsynonymous SNV	LRCH2:ENST00000538422.1:exon11:c.A1363G:p.K455E,LRCH2:ENST00000317135.8:exon11:c.A1363G:p.K455E,	Xq23	.
X	125685608	G	T	GGAAAGAGAC,TGGGAATGGG	exonic	DCAF12L1	nonsynonymous SNV	DCAF12L1:ENST00000371126.1:exon1:c.C984A:p.H328Q,	Xq25	.
X	125686116	G	A	CGTCTTGGAG,GATTCAGCTC	exonic	DCAF12L1	nonsynonymous SNV	DCAF12L1:ENST00000371126.1:exon1:c.C476T:p.P159L,	Xq25	.
X	129201450	G	C	GGCCGAGCCC,ACCCCACGGG	exonic	ELF4	nonsynonymous SNV	ELF4:ENST00000335997.7:exon9:c.C1238G:p.S413W,ELF4:ENST00000308167.5:exon9:c.C1238G:p.S413W,	Xq26.1	.
X	131526200	G	A	ATCAGTTTTG,GCCAACAGCT	exonic	MBNL3	nonsynonymous SNV	MBNL3:ENST00000370849.3:exon3:c.C355T:p.P119S,MBNL3:ENST00000436215.1:exon3:c.C217T:p.P73S,MBNL3:ENST00000421707.1:exon3:c.C217T:p.P73S,MBNL3:ENST00000370857.3:exon3:c.C505T:p.P169S,MBNL3:ENST00000370844.1:exon4:c.C217T:p.P73S,MBNL3:ENST00000370839.3:exon3:c.C505T:p.P169S	Xq26.2	.
X	140271047	G	A	AACGTTTCGG,GAAGGGCACC	exonic	LDOC1	nonsynonymous SNV	LDOC1:ENST00000370526.2:exon1:c.C160T:p.P54S,	Xq27.1	.
X	149631056	A	T	GCCCTCGTGG,TGGAGGAAGA	exonic	MAMLD1	nonsynonymous SNV	MAMLD1:ENST00000358892.3:exon3:c.A115T:p.M39L,MAMLD1:ENST00000370401.2:exon3:c.A115T:p.M39L,MAMLD1:ENST00000262858.5:exon2:c.A115T:p.M39L,	Xq28	.
X	153041852	G	A	CCTATCGGAC,AAGACTTGAC	exonic	PLXNB3	nonsynonymous SNV	PLXNB3:ENST00000538776.1:exon27:c.G3730A:p.E1244K,PLXNB3:ENST00000538966.1:exon29:c.G4840A:p.E1614K,PLXNB3:ENST00000361971.5:exon28:c.G4771A:p.E1591K,	Xq28	.
X	153070192	G	A	CAGGCTTCCC,GGGTGTTGGT	exonic	PDZD4	nonsynonymous SNV	PDZD4:ENST00000164640.4:exon8:c.C926T:p.P309L,PDZD4:ENST00000544474.1:exon6:c.C599T:p.P200L,PDZD4:ENST00000393758.2:exon8:c.C701T:p.P234L,	Xq28	.
X	153135291	C	T	CTCCAGGTGA,CTCTGGTTGG	exonic	L1CAM	nonsynonymous SNV	L1CAM:ENST00000538883.1:exon9:c.G1096A:p.V366I,L1CAM:ENST00000361699.4:exon9:c.G1090A:p.V364I,L1CAM:ENST00000370055.1:exon9:c.G1075A:p.V359I,L1CAM:ENST00000370060.1:exon10:c.G1090A:p.V364I,L1CAM:ENST00000370057.3:exon9:c.G1090A:p.V364I,L1CAM:ENST00000361981.3:exon8:c.G1075A	Xq28	.
X	153217396	G	A	ACTGTCGGCA,GGGCCAGGGC	exonic	HCFC1	nonsynonymous SNV	HCFC1:ENST00000369984.4:exon20:c.C5291T:p.P1764L,HCFC1:ENST00000310441.7:exon20:c.C5156T:p.P1719L,HCFC1:ENST00000354233.3:exon21:c.C4949T:p.P1650L,	Xq28	.
X	153631736	G	A	CAAGGCCCAG,GACCGAGGGC	exonic	RPL10	nonsynonymous SNV	RPL10:ENST00000427682.1:exon3:c.G355A:p.G119R,	Xq28	.
X	153657528	C	T	AAGGTGCGCC,GCCCCAGCCC	exonic	ATP6AP1	nonsynonymous SNV	ATP6AP1:ENST00000439372.1:exon2:c.C296T:p.P99L,ATP6AP1:ENST00000446552.1:exon2:c.C296T:p.P99L,	Xq28	.

isechange=c.4940G>A;AAchange=p.R1647Q;tissue=large_intestine
;AAchange=p.Q2035E;tissue=lung
A;AAchange=p.P220T;tissue=lung&GREB1_ENST00000381486;basechange=c.658C>A;AAchange=p.P220T;tissue=lun
376G>T;AAchange=p.E126*;tissue=endometriur

E93*;tissue=lun

ange=c.1672C>T;AAchange=p.R558C;tissue=endometriur

)000537824;basechange=c.9715G>T;AAchange=p.V3239F;tissue=large_intestine

G>T;AAchange=p.C1381F;tissue=ovary

PRD_ENST00000346816;basechange=c.4105C>T;AAchange=p.R1369C;tissue=breast,prostate,endometrium&PTPRD;basechange=c.5692C>T;AAchange=p.R1898C;tissue=breast,prostate,endometrium&PTPRD_ENST00000381196;basechange=c.5692C>T;AAchange=p.R1898C;tissue=breast,prostate,endometriur

=c.2456G>T;AAchange=p.G819V;tissue=lun

ge=c.1209C>T;AAchange=p.D403D;tissue=large_intestine

p.R187W;tissue=large_intestine

metrium&TP53;basechange=c.476C>A;AAchange=p.A159D;tissue=lung,large_intestine,central_nervous_system&TP53_ENST00000414315;basechange=c.80C>T;AAchange=p.A27V;tissue=urinary_tract,lung,large_intestine,endometrium&TP53_ENST00000413465;basechange=c.476C>T;AAchange=p.A159V;tissue=urinary_tract,lung,large_intestine,endometrium&TP53;basechange=c.476C>T;AAchange=p.A159V;tissue=ovary,breast,haematopoietic_a
er,oesophagus,lung,upper_aerodigestive_tract,large_intestine,central_nervous_system,biliary_tract,endometrium&TP53;basechange=c.438G>T;AAchange=p.W146C;tissue=liver&TP53_ENST00000413465;basechange=c.438G>A;AAchange=p.W146*;tissue=liver,lung&TP53_ENST00000545858;basechange=c.159G>A;AAchange=p.W53*;tissue=liver,lung,endometrium&TP53_ENST00000414315;basechange=c.42G>A;AAchange=p.W14*;tissue=liver,lur

ge=c.86G>A;AAchange=p.R29Q;tissue=large_intestine

3>C;AAchange=p.G64A;tissue=lung&POU3F4;basechange=c.191G>A;AAchange=p.G64E;tissue=lur

nd_lymphoid_tissue,stomach,urinary_tract,pancreas,liver,oesophagus,skin,prostate,lung,large_intestine,endometrium&TP53_ENST00000545858;basechange=c.197C>T;AAchange=p.A66V;tissue=urinary_tract,lung,large_intestine,endometri
ıg,endometrium&TP53_ENST00000455263;basechange=c.438G>A;AAchange=p.W146*;tissue=lung&TP53_ENST00000269305;basechange=c.438G>A;AAchange=p.W146*;tissue=liver,lung,endometriur

C000-EXKBCS

Chromosome	Position (hg1	Reference all	Mutant allele	Sequence context	ANNOVAR fu	Gene	Mutation type	ANNOVAR output (alternative transcripts)	Cytoband	COSMIC
1	1115647	G	A	CCTGCTGGGG,GTGGGAAGCC	exonic	TTLL10	nonsynonymous SNV	[illegible]	[illegible]	[illegible]
1	1115648	G	A	CTGCTGGGGG,TGGGAAGCCA	exonic	TTLL10	nonsynonymous SNV	[illegible]	[illegible]	[illegible]
1	1133147	G	A	GTCGGGCACA,GCAACAGGCA	exonic	TTLL10	nonsynonymous SNV	[illegible]	[illegible]	[illegible]
1	1221410	G	A	CCTCGTTCCG,GAGCTGCTCA	exonic	SCNN1D	nonsynonymous SNV	[illegible]	[illegible]	[illegible]
1	1221411	G	A	CTCGTTCCGG,AGCTGCTCAC	exonic	SCNN1D	nonsynonymous SNV	[illegible]	[illegible]	[illegible]
1	1322876	G	A	AGCGCTGCGG,GGGCCTGTCT	exonic	CCNL2	nonsynonymous SNV	[illegible]	[illegible]	[illegible]
1	1374737	C	T	CGCACGCGGC,CGGTGAGGCA	exonic	VWA1	nonsynonymous SNV	[illegible]	[illegible]	[illegible]
1	3418486	C	T	ACCGGGCACT,TGCAGGAGAA	splicing	MEGF6(ENST	.	.	[illegible]	[illegible]
1	6305461	C	T	GGCTCGTCCG,CAGCGTCCCC	exonic	HES3	nonsynonymous SNV	[illegible]	[illegible]	[illegible]
1	9658663	G	A	GCGCCGGGAG,CCGACCAGAC	exonic	TMEM201	nonsynonymous SNV	[illegible]	[illegible]	[illegible]
1	9782163	C	T	GAGGCCCTCT,CCACCTGCAG	exonic	PIK3CD	nonsynonymous SNV	[illegible]	[illegible]	[illegible]
1	10699309	G	A	GCGCGGCCCG,GGGCGGCGGC	exonic	CASZ1	nonsynonymous SNV	[illegible]	[illegible]	[illegible]
1	11118895	G	A	TCCACGGAGG,GTGCTTCACC	exonic	SRM	nonsynonymous SNV	[illegible]	[illegible]	[illegible]
1	11118897	T	A	CACGGAGGGG,GCTTCACCAC	exonic	SRM	nonsynonymous SNV	[illegible]	[illegible]	[illegible]
1	11589645	C	T	CACAAGCCCC,CTTCCACGGG	exonic	PTCHD2	nonsynonymous SNV	[illegible]	[illegible]	[illegible]
1	11918782	C	T	GTTTCCAAGT,CGAGGCTGAA	exonic	NPPB	nonsynonymous SNV	[illegible]	[illegible]	[illegible]
1	12835203	C	T	CACCTGCCTT,CTCTAGGGTC	exonic	PRAMEF12	nonsynonymous SNV	[illegible]	[illegible]	[illegible]
1	15615904	C	T	GGTCTCTTTC,CATGGATGAG	exonic	FHAD1	nonsynonymous SNV	[illegible]	[illegible]	[illegible]
1	15821896	G	A	ACTGCCAGGG,ACTCGTCTTC	exonic	CASP9	nonsynonymous SNV	[illegible]	[illegible]	[illegible]
1	16044424	C	T	AACGAGAACT,CTTGGAGAGC	exonic	PLEKHM2	nonsynonymous SNV	[illegible]	[illegible]	[illegible]
1	16577866	G	A	GATCCTCGTC,GGGGGCCAAA	exonic	FBXO42	stopgain	[illegible]	[illegible]	[illegible]
1	17410304	C	T	TTCTTCACCT,TTTCAGGAAC	exonic	PADI2	nonsynonymous SNV	[illegible]	[illegible]	[illegible]
1	19166964	G	A	CTTGAAGCAG,AGGTCTCACT	exonic	TAS1R2	nonsynonymous SNV	[illegible]	[illegible]	[illegible]
1	19499462	A	T	CCATCTTAGA,AAATGCTCAT	exonic	UBR4	nonsynonymous SNV	[illegible]	[illegible]	[illegible]
1	20659370	C	T	CGCCAGCTCC,TCTTCCCTGG	exonic	VWA5B1	nonsynonymous SNV	[illegible]	[illegible]	[illegible]
1	21044019	C	T	GTGACGTGGT,CACGTGGTAG	exonic	KIF17	nonsynonymous SNV	[illegible]	[illegible]	[illegible]
1	21902293	G	A	CGGTGGAGAT,GACCGGGCCA	exonic	ALPL	nonsynonymous SNV	[illegible]	[illegible]	[illegible]
1	22205095	C	T	CCAGTGTAGC,TGGGGCACAG	exonic	HSPG2	nonsynonymous SNV	[illegible]	[illegible]	[illegible]
1	22965889	C	T	CCTCATCTTC,CATCTGCCTG	exonic	C1QA	nonsynonymous SNV	[illegible]	[illegible]	[illegible]
1	23519580	T	A	CGAATAAGAC,AGGAGGCCTT	exonic	HTR1D	stoploss	[illegible]	[illegible]	[illegible]
1	24202038	C	G	ATCATGTAAT,CTTCATAGGG	exonic	CNR2	nonsynonymous SNV	[illegible]	[illegible]	[illegible]
1	24402697	C	T	GACGGTTGCC,CAGACCAGCT	exonic	MYOM3	nonsynonymous SNV	[illegible]	[illegible]	[illegible]
1	27058088	C	T	CGCTTCCCTC,ACCGCAGGTA	exonic	ARID1A	nonsynonymous SNV	[illegible]	[illegible]	[illegible]
1	28206473	C	A	CTACAGGATC,AGCCCTGAAA	exonic	THEMIS2	nonsynonymous SNV	[illegible]	[illegible]	[illegible]
1	28800530	C	T	CAGCCCACTT,CCATGACTCC	exonic	PHACTR4	nonsynonymous SNV	[illegible]	[illegible]	[illegible]
1	29029076	T	C	TCAGAGGAAA,TTCAGGTATT	exonic	GMEB1	nonsynonymous SNV	[illegible]	[illegible]	[illegible]
1	31215312	G	A	CCGATCCTGA,GTAGCCCATC	exonic	LAPTM5	nonsynonymous SNV	[illegible]	[illegible]	[illegible]
1	32196681	C	T	ACCACCTCCA,CCCCACCGCC	exonic	BAI2	nonsynonymous SNV	[illegible]	[illegible]	[illegible]
1	32204477	G	A	GGCTCAGCTG,AGGCTGGGTA	exonic	BAI2	nonsynonymous SNV	[illegible]	[illegible]	[illegible]
1	32739920	C	T	TCTTCCATTC,CTCAGGGACC	exonic	LCK	nonsynonymous SNV	[illegible]	[illegible]	[illegible]
1	32768299	C	G	TAATTTGCTG,TCAACTATGG	exonic	HDAC1	nonsynonymous SNV	[illegible]	[illegible]	[illegible]
1	33236737	C	T	GATATTGACC,CCCTGGGTGA	exonic	KIAA1522	nonsynonymous SNV	[illegible]	[illegible]	[illegible]
1	34554723	G	C	GCGTAATTGG,GTAGCCATAT	exonic	CSMD2	nonsynonymous SNV	[illegible]	[illegible]	[illegible]
1	36479324	C	T	ACGCGTACTT,CAGCACCTAT	exonic	AGO3	nonsynonymous SNV	[illegible]	[illegible]	[illegible]
1	39906798	G	A	CATTGATGAG,TGTGGAGAAA	splicing	MACF1(ENST	.	.	[illegible]	[illegible]
1	40775983	G	A	CTGATGCCCT,GGGACCCTGT	exonic	COL9A2	stopgain	[illegible]	[illegible]	[illegible]
1	42045778	G	A	AGGTGCCAAG,AGGGGAGATC	exonic	HIVEP3	nonsynonymous SNV	[illegible]	[illegible]	[illegible]
1	43777426	G	T	TCGTTCTCTG,GGATGGACCC	exonic	TIE1	nonsynonymous SNV	[illegible]	[illegible]	[illegible]
1	44086508	G	A	ACCTTTCCCA,GATGGGCAGT	splicing	PTPRF(ENST	.	.	[illegible]	[illegible]
1	44415351	C	T	GCCAGTGGCT,CAAGATTGTA	exonic	IPO13	nonsynonymous SNV	[illegible]	[illegible]	[illegible]
1	45297676	G	A	TCAATAAGGG,AACTCCTGAC	exonic	PTCH2	nonsynonymous SNV	[illegible]	[illegible]	[illegible]
1	46383072	C	T	CACATCTGCT,CAGCCCTCTT	exonic	MAST2	nonsynonymous SNV	[illegible]	[illegible]	[illegible]
1	46500212	G	A	GTACAAAGTG,GAGGGAATTC	exonic	MAST2	nonsynonymous SNV	[illegible]	[illegible]	[illegible]
1	46500968	G	T	AGAGAGTGGG,AAGAGGATCC	exonic	MAST2	stopgain	[illegible]	[illegible]	[illegible]
1	46669109	C	T	ATGGAGGGGA,CGTGGAGTCC	exonic	LURAP1	nonsynonymous SNV	[illegible]	[illegible]	[illegible]
1	46669183	C	T	AGGGCTGCTC,GCGGGCTGCG	exonic	LURAP1	nonsynonymous SNV	[illegible]	[illegible]	[illegible]
1	47051617	G	A	ACTTTCCTTG,AAATGGAACT	exonic	MKNK1	nonsynonymous SNV	[illegible]	[illegible]	[illegible]
1	48688425	C	A	AAGGAGCTGG,AGCAATGGGG	exonic	SLC5A9	nonsynonymous SNV	[illegible]	[illegible]	[illegible]
1	53222242	G	A	CAGGAACCTG,AGTATTCCCA	exonic	ZYG11B	nonsynonymous SNV	[illegible]	[illegible]	[illegible]
1	53676338	C	T	GATGACTTCC,CATTAAGGAC	exonic	CPT2	nonsynonymous SNV	[illegible]	[illegible]	[illegible]
1	60370635	G	A	TGCACCTCAT,GATGACAGCA	exonic	CYP2J2	nonsynonymous SNV	[illegible]	[illegible]	[illegible]
1	62737180	T	G	CTGAAGGTTC,TTTTGGTCCC	exonic	KANK4	nonsynonymous SNV	[illegible]	[illegible]	[illegible]
1	67161077	C	T	TCTCCCTTTC,TTAACCCTTC	exonic	SGIP1	nonsynonymous SNV	[illegible]	[illegible]	[illegible]
1	67436622	C	T	TGAAGGATCT,ACATAAAAGA	exonic	MIER1	nonsynonymous SNV	[illegible]	[illegible]	[illegible]
1	74937556	C	T	TTCATGTAAT,TTCAAATTGC	exonic	LRRC53	nonsynonymous SNV	[illegible]	[illegible]	[illegible]
1	76397643	C	T	AGAAGCAATT,CACAAAGTCA	exonic	ASB17	nonsynonymous SNV	[illegible]	[illegible]	[illegible]
1	79385933	G	A	CTACAGCAAA,ATTTTTGTGA	exonic	ELTD1	nonsynonymous SNV	[illegible]	[illegible]	[illegible]
1	84971717	C	T	AGTCCGGCCT,CAGCCGGAGC	exonic	GNG5	nonsynonymous SNV	[illegible]	[illegible]	[illegible]
1	85560210	G	A	ATTCTTTTCT,GAGCTTCTCT	exonic	WDR63	stopgain	[illegible]	[illegible]	[illegible]
1	85592393	T	G	CCCTGGATCT,TTCTTGTATG	exonic	WDR63	nonsynonymous SNV	[illegible]	[illegible]	[illegible]
1	86374333	C	T	ACCTGGATAT,CCTATAATTT	exonic	COL24A1	nonsynonymous SNV	[illegible]	[illegible]	[illegible]
1	86939229	A	G	AAAACTTGAG,CCTACAAAAA	exonic	CLCA1	nonsynonymous SNV	[illegible]	[illegible]	[illegible]
1	90492976	G	A	AGATGAGGAG,ATGAAGAGAA	exonic	ZNF326	nonsynonymous SNV	[illegible]	[illegible]	[illegible]
1	90492978	T	G	ATGAGGAGGA,GAAGAGAAGA	exonic	ZNF326	nonsynonymous SNV	[illegible]	[illegible]	[illegible]
1	91182560	G	A	ACTGAGATAG,AGAAGAAGGC	exonic	BARHL2	nonsynonymous SNV	[illegible]	[illegible]	[illegible]
1	98060692	A	T	CAGGCCTTGG,AGATGGCATC	exonic	DPYD	nonsynonymous SNV	[illegible]	[illegible]	[illegible]
1	102290603	G	A	ATGCAGTCAC,AAGTCTTGTT	exonic	OLFM3	nonsynonymous SNV	[illegible]	[illegible]	[illegible]
1	103387091	C	T	CCATTGGGAC,TTGAGGGCCT	exonic	COL11A1	nonsynonymous SNV	[illegible]	[illegible]	[illegible]
1	108742601	G	A	TGGCCCAGAG,GATGCCCAGG	exonic	SLC25A24	nonsynonymous SNV	[illegible]	[illegible]	[illegible]
1	109814918	C	T	CTGCCCCAGC,CCTACGCAGA	exonic	CELSR2	nonsynonymous SNV	[illegible]	[illegible]	[illegible]
1	109814919	C	T	TGCCCCAGCC,CTACGCAGAT	exonic	CELSR2	nonsynonymous SNV	[illegible]	[illegible]	[illegible]
1	110051528	G	T	TCACGGTGGG,GTGCATAGTG	exonic	AMIGO1	nonsynonymous SNV	[illegible]	[illegible]	[illegible]
1	110293305	C	T	CTGACAGCCT,CAGCCGGGAC	exonic	EPS8L3	nonsynonymous SNV	[illegible]	[illegible]	[illegible]
1	111494899	G	C	TGCACTGAAG,AGGCAATGAT	exonic	LRIF1	nonsynonymous SNV	[illegible]	[illegible]	[illegible]
1	112026340	G	A	CATTTCCTTT,GAGTCAGGAC	exonic	ADORA3	nonsynonymous SNV	[illegible]	[illegible]	[illegible]
1	113119615	A	T	CACTTTTGGA,CATGAGGATT	exonic	ST7L	nonsynonymous SNV	[illegible]	[illegible]	[illegible]
1	114256056	T	C	TTTACTCTTT,AATCCTATTT	exonic	PHTF1	nonsynonymous SNV	[illegible]	[illegible]	[illegible]
1	115220579	C	A	TGTCTGTCTT,AAGTAGAGGT	exonic	AMPD1	nonsynonymous SNV	[illegible]	[illegible]	[illegible]
1	147142095	G	A	ACCCGCCGCT,GTGCAGGCAG	exonic	ACP6	stopgain	[illegible]	[illegible]	[illegible]
1	149901528	C	T	GACCTCAGGC,TTCCCCTCAG	exonic	MTMR11	nonsynonymous SNV	[illegible]	[illegible]	[illegible]
1	150772116	C	T	TTCTCATTCC,CTCGGGGATC	exonic	CTSK	nonsynonymous SNV	[illegible]	[illegible]	[illegible]
1	151824786	G	T	TGTGGTCTCT,TTGGACTTGA	exonic	THEM5	nonsynonymous SNV	[illegible]	[illegible]	[illegible]
1	152128737	C	T	CAGCCTAATT,CTGACCTAGC	exonic	RPTN	nonsynonymous SNV	[illegible]	[illegible]	[illegible]
1	152129382	G	C	TGTCCATCTC,GTCTTGATCT	exonic	RPTN	nonsynonymous SNV	[illegible]	[illegible]	[illegible]
1	152283065	C	T	CCAGAGCTTT,CCCTGACTGG	exonic	FLG	nonsynonymous SNV	[illegible]	[illegible]	[illegible]
1	152326972	C	T	AGATGTCTGT,CTGAATTTGA	exonic	FLG2	nonsynonymous SNV	[illegible]	[illegible]	[illegible]
1	152327341	A	G	ATGTTGTCCA,AGCCAGAGGA	exonic	FLG2	nonsynonymous SNV	[illegible]	[illegible]	[illegible]
1	154938902	G	A	CCCCCCAAGG,GGGTTCCTTC	exonic	SHC1	nonsynonymous SNV	[illegible]	[illegible]	[illegible]
1	155148708	G	A	CCGACCCAAG,TGAGCCAGCC	splicing	TRIM46(ENST	.	.	[illegible]	[illegible]
1	155148709	T	A	CGACCCAAGG,GAGCCAGCCC	splicing	TRIM46(ENST	.	.	[illegible]	[illegible]
1	155154649	G	A	GGAAGGGATG,GGTGTGGGGG	exonic	TRIM46	nonsynonymous SNV	[illegible]	[illegible]	[illegible]
1	155450730	G	A	GCTTATTCTT,GAATATCTAT	exonic	ASH1L	nonsynonymous SNV	[illegible]	[illegible]	[illegible]
1	155450731	G	A	CTTATTCTTG,AATATCTATA	exonic	ASH1L	nonsynonymous SNV	[illegible]	[illegible]	[illegible]
1	156354268	C	T	GCACCCTCCT,CATGGTGGGG	exonic	RHBG	nonsynonymous SNV	[illegible]	[illegible]	[illegible]
1	156641426	G	A	ATTTCTTCTG,AGTCCACAGT	exonic	NES	nonsynonymous SNV	[illegible]	[illegible]	[illegible]
1	157767595	G	A	CACAGCCACT,ATTGTTCTCT	exonic	FCRL1	nonsynonymous SNV	[illegible]	[illegible]	[illegible]
1	158064638	C	T	GCCCACACCC,CTGGCCCTGC	exonic	KIRREL	nonsynonymous SNV	[illegible]	[illegible]	[illegible]
1	158064653	C	T	CCCTGCTGCC,CAGCTGGCAC	exonic	KIRREL	nonsynonymous SNV	[illegible]	[illegible]	[illegible]
1	158908219	G	A	TCCAGTCAAA,GAATAATCCC	exonic	PYHIN1	nonsynonymous SNV	[illegible]	[illegible]	[illegible]
1	159778168	G	A	CAGCTGCTCT,GGCAGGTGAT	exonic	FCRL6	nonsynonymous SNV	[illegible]	[illegible]	[illegible]
1	159900590	G	A	GGCACTAGGA,GTGAGCAGCC	exonic	IGSF9	nonsynonymous SNV	[illegible]	[illegible]	[illegible]
1	159906209	G	A	CAGCTGGGTG,CGTGGGTGGC	exonic	IGSF9	nonsynonymous SNV	[illegible]	[illegible]	[illegible]
1	160100334	C	T	GATTGACCCT,CCCGGGCTGC	exonic	ATP1A2	nonsynonymous SNV	[illegible]	[illegible]	[illegible]
1	161017735	C	T	AGGCAGTAAT,CCCACCCTCA	exonic	ARHGAP30	nonsynonymous SNV	[illegible]	[illegible]	[illegible]
1	161192774	G	A	GTCAGTCACG,TCTGGAAGTA	exonic	APOA2	nonsynonymous SNV	[illegible]	[illegible]	[illegible]
1	161192815	C	A	CACATGGCTC,TTTGCCTGTC	exonic	APOA2	nonsynonymous SNV	[illegible]	[illegible]	[illegible]
1	162824601	C	T	ACACTCCTTT,CTGCCCTGGA	exonic	C1orf110	nonsynonymous SNV	[illegible]	[illegible]	[illegible]
1	166990959	G	T	GTTCGGGGAA,AGGAATTACC	exonic	MAEL	nonsynonymous SNV	[illegible]	[illegible]	[illegible]
1	167095138	A	C	ACCGTGCGTA,GAAGCGGGCC	exonic	DUSP27	nonsynonymous SNV	[illegible]	[illegible]	[illegible]
1	167873165	C	A	GGATCTCCAC,AACTGCTCAG	exonic	ADCY10	nonsynonymous SNV	[illegible]	[illegible]	[illegible]
1	169551736	A	T	CAATTTTCTT,AAGGAAGTTA	exonic	F5	nonsynonymous SNV	[illegible]	[illegible]	[illegible]
1	169697199	C	T	AAGACTGTAC,TTCACATGTG	exonic	SELE	nonsynonymous SNV	[illegible]	[illegible]	[illegible]
1	170688891	A	G	TCAGAAGAAA,AAAGAAGAGA	exonic	PRRX1	nonsynonymous SNV	[illegible]	[illegible]	[illegible]
1	171086544	C	T	CTTTGCAATT,CTATTCTGTT	exonic	FMO3	nonsynonymous SNV	[illegible]	[illegible]	[illegible]
1	176853572	C	T	CTGAGTGTTC,CACTCCAGGA	exonic	ASTN1	stopgain	[illegible]	[illegible]	[illegible]
1	176853573	C	T	TGAGTGTTCC,ACTCCAGGAC	exonic	ASTN1	stopgain	[illegible]	[illegible]	[illegible]
1	181724526	G	A	CACAGAGAAG,AGTGCATGTA	exonic	CACNA1E	nonsynonymous SNV	[illegible]	[illegible]	[illegible]
1	182025890	G	A	GCAGGGGAAG,GCCGCTCGCC	exonic	ZNF648	nonsynonymous SNV	[illegible]	[illegible]	[illegible]
1	183513620	C	T	CCCTCCTCTT,CCAGCAGGCC	exonic	SMG7	nonsynonymous SNV	[illegible]	[illegible]	[illegible]
1	196197403	C	T	ATTGCAGATG,TGTTTCTTCG	exonic	KCNT2	nonsynonymous SNV	[illegible]	[illegible]	[illegible]
1	197168526	T	A	CACTGCAATA,TTTTTTGTTG	exonic	ZBTB41	nonsynonymous SNV	[illegible]	[illegible]	[illegible]
1	200587797	G	A	TGGGAAGAAG,AATATCAAGA	exonic	KIF14	nonsynonymous SNV	[illegible]	[illegible]	[illegible]
1	201023649	C	T	CCCGCAGCCT,CACCCAGGCA	exonic	CACNA1S	nonsynonymous SNV	[illegible]	[illegible]	[illegible]
1	201181426	G	A	AGATCTTGGG,GCTATGGAAC	exonic	IGFN1	nonsynonymous SNV	[illegible]	[illegible]	[illegible]
1	201181427	G	A	GATCTTGGGG,CTATGGAACT	exonic	IGFN1	nonsynonymous SNV	[illegible]	[illegible]	[illegible]
1	201592458	G	A	AGCCCTTCTC,GACGAGCTTT	exonic	AC096677.1	nonsynonymous SNV	[illegible]	[illegible]	[illegible]
1	202990002	G	T	TCCAGACCAG,ACTGCTGCCT	exonic	TMEM183A	nonsynonymous SNV	[illegible]	[illegible]	[illegible]
1	203194944	C	T	GCGAGCCTCC,CCTGTCTGTA	exonic	CHIT1	nonsynonymous SNV	[illegible]	[illegible]	[illegible]
1	203316903	T	G	CGGGTCAGGT,GTTGTGGTCC	exonic	FMOD	nonsynonymous SNV	[illegible]	[illegible]	[illegible]
1	203316904	G	T	GGGTCAGGTT,TTGTGGTCCA	exonic	FMOD	nonsynonymous SNV	[illegible]	[illegible]	[illegible]
1	204938063	C	T	TGCCTGGCCT,CAACAAGATG	exonic	NFASC	nonsynonymous SNV	[illegible]	[illegible]	[illegible]
1	205156916	G	A	AGGTGGGAAG,AAATGCAGCT	exonic	DSTYK	nonsynonymous SNV	[illegible]	[illegible]	[illegible]
1	205312600	G	A	CAGTCCATGG,GACGCCGTTG	exonic	KLHDC8A	nonsynonymous SNV	[illegible]	[illegible]	[illegible]
1	206653438	G	T	CTAATGTTTC,GGGGCTGCAC	exonic	IKBKE	nonsynonymous SNV	[illegible]	[illegible]	[illegible]
1	207890869	G	A	ACTCCCCTTG,AGATATTCCC	exonic	CR1L	nonsynonymous SNV	[illegible]	[illegible]	[illegible]
1	209878309	C	T	GAAAAAATAT,TCCTCCCCAT	exonic	HSD11B1	nonsynonymous SNV	[illegible]	[illegible]	[illegible]
1	214819436	C	T	GCTAGTGATT,TTGATGCCGA	exonic	CENPF	nonsynonymous SNV	[illegible]	[illegible]	[illegible]
1	215848402	G	A	TGGGATCCAG,AAATCAGCAG	exonic	USH2A	nonsynonymous SNV	[illegible]	[illegible]	[illegible]
1	216595423	G	A	GGGCAATCCT,AATACAAAAC	exonic	USH2A	stopgain	[illegible]	[illegible]	[illegible]
1	222716994	G	T	TAGAACTTGC,ATTGTGGCGG	exonic	HHIPL2	nonsynonymous SNV	[illegible]	[illegible]	[illegible]
1	222717330	G	A	TCCTTGTCAG,AAGGTCCAGG	exonic	HHIPL2	nonsynonymous SNV	[illegible]	[illegible]	[illegible]
1	223177074	C	T	ACGTGTTCAC,ATGGCGAGGA	exonic	DISP1	nonsynonymous SNV	[illegible]	[illegible]	[illegible]
1	223983609	G	A	GGGTATGGTG,GGGTGGGTAT	exonic	TP53BP2	nonsynonymous SNV	[illegible]	[illegible]	[illegible]
1	223983620	G	A	GGGTGGGTAT,GAGGGTACTC	exonic	TP53BP2	nonsynonymous SNV	[illegible]	[illegible]	[illegible]
1	226552744	G	T	CCCTGGGACA,GATCCCAGCA	exonic	PARP1	nonsynonymous SNV	[illegible]	[illegible]	[illegible]
1	227335079	C	T	CATGATTTTT,CGTATGTCTC	exonic	CDC42BPA	nonsynonymous SNV	[illegible]	[illegible]	[illegible]
1	227335215	C	T	TGAAGGATTT,AGGAGAGATA	exonic	CDC42BPA	nonsynonymous SNV	[illegible]	[illegible]	[illegible]
1	228474524	G	A	CATCACAGAG,GTGAGGACTT	exonic	OBSCN	nonsynonymous SNV	[illegible]	[illegible]	[illegible]
1	230883338	G	T	TTGAGCAAAT,AGGCAGGAGT	exonic	CAPN9	nonsynonymous SNV	[illegible]	[illegible]	[illegible]
1	231557112	G	A	CCGCCGCCGG,GCTCAGCGCA	exonic	EGLN1	nonsynonymous SNV	[illegible]	[illegible]	[illegible]
1	234743107	C	T	TGGGTGTCCT,CAGCCGCTCG	exonic	IRF2BP2	nonsynonymous SNV	[illegible]	[illegible]	[illegible]
1	234744642	G	A	GAGCGCGGTG,GCAGCGGCGT	exonic	IRF2BP2	nonsynonymous SNV	[illegible]	[illegible]	[illegible]
1	236722311	G	A	CTTCTTCCAG,ATATATTTTG	exonic	HEATR1	nonsynonymous SNV	[illegible]	[illegible]	[illegible]
1	237895416	T	C	CAGCTGATCC,TCTGTTTAGT	exonic	RYR2	nonsynonymous SNV	[illegible]	[illegible]	[illegible]
1	237951371	G	A	CACAGATACG,AGAACCAGAA	exonic	RYR2	nonsynonymous SNV	[illegible]	[illegible]	[illegible]
1	241912920	G	A	AGACTGGAAG,AGGACGAGCA	exonic	WDR64	nonsynonymous SNV	[illegible]	[illegible]	[illegible]
1	242024790	C	T	TGACTACAAT,CAGACACTGC	exonic	EXO1	nonsynonymous SNV	[illegible]	[illegible]	[illegible]
1	243303309	G	A	TTGAGGTCTT,GATCACCAGA	exonic	CEP170	nonsynonymous SNV	[illegible]	[illegible]	[illegible]
1	243303310	G	A	TGAGGTCTTG,ATCACCAGAT	exonic	CEP170	nonsynonymous SNV	[illegible]	[illegible]	[illegible]
1	244528058	G	A	ATCCCGCCAA,GTATAGTTCT	exonic	C1orf100	nonsynonymous SNV	[illegible]	[illegible]	[illegible]
1	247200785	G	A	CTTTCGAAGG,AACTGGAACA	exonic	ZNF670	nonsynonymous SNV	[illegible]	[illegible]	[illegible]
1	247201026	G	A	GTCCTTTCAT,GACTCTGAGG	exonic	ZNF670	nonsynonymous SNV	[illegible]	[illegible]	[illegible]
1	248487750	C	T	ATGGAGTTTC,CATGAAGGCC	exonic	OR2M7	nonsynonymous SNV	[illegible]	[illegible]	[illegible]
1	248512656	G	A	CTTCAGCAAT,AGGTCATGAT	exonic	OR14C36	nonsynonymous SNV	[illegible]	[illegible]	[illegible]
2	10811780	G	A	GATTGCGAAT,AAATTCAATG	exonic	NOL10	nonsynonymous SNV	[illegible]	[illegible]	[illegible]
2	20113409	G	A	TGCACATCCA,AATTGATACT	exonic	WDR35	nonsynonymous SNV	[illegible]	[illegible]	[illegible]
2	21227461	C	T	CCATCTTCTT,ATATTCTGCA	exonic	APOB	nonsynonymous SNV	[illegible]	[illegible]	[illegible]
2	21230583	C	T	AAATTCCCTT,ATTGTTTGTG	exonic	APOB	nonsynonymous SNV	[illegible]	[illegible]	[illegible]
2	21241960	C	T	TCAATCTCTC,TGTAGGCCTC	exonic	APOB	nonsynonymous SNV	[illegible]	[illegible]	[illegible]
2	25355808	C	T	TACCAGCGCT,CGAGGTGATC	exonic	EFR3B	nonsynonymous SNV	[illegible]	[illegible]	[illegible]
2	25466799	C	T	AGACAGCACC,GGATGGGCTT	exonic	DNMT3A	nonsynonymous SNV	[illegible]	[illegible]	[illegible]
2	27715335	G	T	GTTGATAGAT,GTGACTTTTT	exonic	FNDC4	nonsynonymous SNV	[illegible]	[illegible]	[illegible]
2	27716868	G	A	GGGCCCTGGG,GACTCTCTCC	exonic	FNDC4	nonsynonymous SNV	[illegible]	[illegible]	[illegible]
2	28635221	C	T	CCCGCATCTC,CTCCGAATCC	exonic	FOSL2	nonsynonymous SNV	[illegible]	[illegible]	[illegible]
2	29420427	T	C	GGGCAGTTCT,GGGTGGGTCC	exonic	ALK	nonsynonymous SNV	[illegible]	[illegible]	[illegible]
2	38812952	C	A	AAATGTCACA,ATTCTTTGGC	exonic	HNRNPLL	nonsynonymous SNV	[illegible]	[illegible]	[illegible]
2	40657326	T	G	AGCAATTACA,GGTCCACATG	exonic	SLC8A1	nonsynonymous SNV	[illegible]	[illegible]	[illegible]
2	42180288	G	A	AAATCAAAGG,ACAGTGCAGA	exonic	C2orf91	nonsynonymous SNV	[illegible]	[illegible]	[illegible]
2	43520319	C	T	GATAATCCCT,TGACTTCCTC	exonic	THADA	nonsynonymous SNV	[illegible]	[illegible]	[illegible]
2	43801705	G	A	TCTAAACATG,TTTCCAAGAG	exonic	THADA	nonsynonymous SNV	[illegible]	[illegible]	[illegible]
2	45826824	C	T	TTGCTGGTTT,TTCTTCAACT	exonic	SRBD1	nonsynonymous SNV	[illegible]	[illegible]	[illegible]
2	46739745	C	T	TTAAACTCTT,CTCAGCCTTG	exonic	ATP6V1E2	nonsynonymous SNV	[illegible]	[illegible]	[illegible]
2	47635570	G	A	GTTGTGCTTA,TAAAATGAAT	exonic	MSH2	nonsynonymous SNV	[illegible]	[illegible]	[illegible]
2	48809208	G	A	GAAAAAAAGG,GATTGATATT	exonic	STON1,STON	nonsynonymous SNV	[illegible]	[illegible]	[illegible]
2	55080821	C	T	TAAAGGGATT,CTTGGGCCTC	exonic	EML6	nonsynonymous SNV	[illegible]	[illegible]	[illegible]
2	55561343	C	T	ATACCAATTT,TTTGGATAGG	exonic	CCDC88A	nonsynonymous SNV	[illegible]	[illegible]	[illegible]
2	71216065	G	A	CATGGTAGAG,GCAGGACGTT	exonic	AC007040.11	nonsynonymous SNV	[illegible]	[illegible]	[illegible]
2	74274215	C	T	CTTGTCACCT,CTGCCCCTTT	exonic	TET3	nonsynonymous SNV	[illegible]	[illegible]	[illegible]
2	74320116	C	T	CTATCAGAAC,AGGTAACGGG	exonic	TET3	stopgain	[illegible]	[illegible]	[illegible]
2	79971565	G	A	AAAAAGAAAG,GAGGTCAAAG	exonic	CTNNA2	nonsynonymous SNV	[illegible]	[illegible]	[illegible]
2	80620344	G	T	AGACTGGAAG,AAAGAAAAAG	exonic	CTNNA2	nonsynonymous SNV	[illegible]	[illegible]	[illegible]
2	80620345	A	G	GACTGGAAGG,AAGAAAAAGG	exonic	CTNNA2	nonsynonymous SNV	[illegible]	[illegible]	[illegible]
2	85531039	C	T	CCCCCTCACC,ATCCGAGCTG	exonic	TCF7L1	nonsynonymous SNV	[illegible]	[illegible]	[illegible]
2	85536253	C	T	GCTGGACTCC,CGGCCACTCC	exonic	TCF7L1	nonsynonymous SNV	[illegible]	[illegible]	[illegible]
2	90229258	G	A	ACATCCAGAT,ATCCAGTCTC	exonic	IGKV1D-42	nonsynonymous SNV	[illegible]	[illegible]	[illegible]
2	97462757	C	T	AGGGAAGTCC,ACCTGGCCAT	exonic	CNNM4	nonsynonymous SNV	[illegible]	[illegible]	[illegible]
2	100916212	C	A	GCGTTCAGGT,AGGTGCATCC	exonic	LONRF2	nonsynonymous SNV	[illegible]	[illegible]	[illegible]
2	101971749	C	T	AATTCTACTT,TTCTGGAGAC	exonic	CREG2	nonsynonymous SNV	[illegible]	[illegible]	[illegible]
2	108443485	G	A	TTGCAGCAAG,CCTACGGGGA	exonic	RGPD4	nonsynonymous SNV	[illegible]	[illegible]	[illegible]
2	108872049	C	T	GGCCAGAAAT,CCAAGGATTG	exonic	SULT1C3	nonsynonymous SNV	[illegible]	[illegible]	[illegible]
2	111850443	T	C	ACCCCTTAGT,TTGTCTGTTG	exonic	ACOXL	nonsynonymous SNV	[illegible]	[illegible]	[illegible]
2	112686876	C	A	CGTAGCCATT,CCCAGGTGAC	exonic	MERTK	nonsynonymous SNV	[illegible]	[illegible]	[illegible]
2	113943504	C	T	CACCTTGGCC,CCTGCCCCTG	exonic	PSD4	nonsynonymous SNV	[illegible]	[illegible]	[illegible]
2	121732644	G	A	CCACTGGGAA,ACTGCACCAA	exonic	GLI2	nonsynonymous SNV	[illegible]	[illegible]	[illegible]
2	122182873	C	T	GGAACTTGAC,GGCTGCCAGC	exonic	CLASP1	nonsynonymous SNV	[illegible]	[illegible]	[illegible]
2	128631541	G	A	TTAGGCCGGG,AAGAGGGCTC	exonic	AMMECR1L	nonsynonymous SNV	[illegible]	[illegible]	[illegible]
2	128932387	T	G	GCTTGGATCC,CAGACTTAGG	exonic	UGGT1	nonsynonymous SNV	[illegible]	[illegible]	[illegible]
2	135710995	G	A	TTCTGTTCAA,ACAGCCATAC	exonic	CCNT2	nonsynonymous SNV	[illegible]	[illegible]	[illegible]
2	137988680	C	T	TGTGAAATTC,CTGCCGAATG	exonic	THSD7B	nonsynonymous SNV	[illegible]	[illegible]	[illegible]
2	141812811	C	T	GGATCGACTT,ACATGCATGG	exonic	LRP1B	nonsynonymous SNV	[illegible]	[illegible]	[illegible]
2	145156038	G	A	AAAGTAGCAG,GGGAAATGCG	exonic	ZEB2	nonsynonymous SNV	[illegible]	[illegible]	[illegible]
2	148683611	G	A	ACCTGTAGAT,AATACATGTT	exonic	ACVR2A	nonsynonymous SNV	[illegible]	[illegible]	[illegible]
2	163046199	C	T	TTAATTTCCT,TTTAGGCAGC	exonic	FAP	nonsynonymous SNV	[illegible]	[illegible]	[illegible]
2	168102956	G	A	GATGAAAAAG,AGATATTAAC	exonic	XIRP2	nonsynonymous SNV	[illegible]	[illegible]	[illegible]
2	168108386	G	A	AAAGGCCTGG,ATATGCAACC	exonic	XIRP2	nonsynonymous SNV	[illegible]	[illegible]	[illegible]
2	168726203	T	G	ATGTCCGCAG,AAGTGGTATA	exonic	B3GALT1	nonsynonymous SNV	[illegible]	[illegible]	[illegible]
2	170027123	G	A	CCATCGCGAG,GAATGCACTG	exonic	LRP2	nonsynonymous SNV	[illegible]	[illegible]	[illegible]
2	170081839	G	A	CTGAGTTCTA,GATTGGTAGA	exonic	LRP2	nonsynonymous SNV	[illegible]	[illegible]	[illegible]
2	171806205	C	T	AGTGAGCATT,GTTTCTGCAG	exonic	GORASP2	nonsynonymous SNV	[illegible]	[illegible]	[illegible]
2	172216952	C	A	CAGAAGGACT,GCACAGCTGA	exonic	METTL8	nonsynonymous SNV	[illegible]	[illegible]	[illegible]
2	178415902	C	A	CATAAGAGAG,TGCTCTTCCT	exonic	TTC30B	nonsynonymous SNV	[illegible]	[illegible]	[illegible]
2	179444318	C	T	ACAGTGATTT,GCTTGGGGTT	exonic	TTN	nonsynonymous SNV	[illegible]	[illegible]	[illegible]
2	179475789	C	T	GTATAAATTC,GGCATCTGCA	exonic	TTN	nonsynonymous SNV	[illegible]	[illegible]	[illegible]
2	179634571	T	A	TTCAGCCACA,GGAAGGGACA	exonic	TTN	nonsynonymous SNV	[illegible]	[illegible]	[illegible]
2	179702284	C	T	CTCAGGGCTT,CTGGGAGAGG	exonic	CCDC141	nonsynonymous SNV	[illegible]	[illegible]	[illegible]
2	186671789	C	A	GTTGTTCACT,CTCTGTTTGT	exonic	FSIP2	nonsynonymous SNV	[illegible]	[illegible]	[illegible]
2	188223850	C	T	CAGAACTGAT,CAGCAACTAG	exonic	CALCRL	stopgain	[illegible]	[illegible]	[illegible]
2	189864605	G	A	GGTGTCCCAG,GAAAGATGGC	exonic	COL3A1	nonsynonymous SNV	[illegible]	[illegible]	[illegible]
2	197183515	G	A	AGTGCACACG,ATTCCTGCGA	exonic	HECW2	nonsynonymous SNV	[illegible]	[illegible]	[illegible]
2	198270156	G	A	CTTTCGAGCT,GAGTTCGAAT	exonic	SF3B1	nonsynonymous SNV	[illegible]	[illegible]	[illegible]
2	201492113	G	A	AAGCCAGAAA,GAAACTGGAA	exonic	AOX1	nonsynonymous SNV	[illegible]	[illegible]	[illegible]
2	201527657	G	A	TGCCTGTTCC,AGGTTGAAAT	exonic	AOX1	nonsynonymous SNV	[illegible]	[illegible]	[illegible]
2	202093791	G	A	TGCGCAGGTG,AGCAGCGTTT	exonic	CASP10	stopgain	[illegible]	[illegible]	[illegible]

2	220500457	G	A	GCTGCGGACG,GCTCGGTATT	exonic	SLC4A3	nonsynonymous SNV	SLC4A3:ENST00000273063.6:exon14:c.G2116A:p.G706S,SLC4A3:ENST00000373760.2:exon14:c.G2035A:p.G679S,SLC4A3:ENST00000317151.3:exon14:c.G2035A:p.G679S,SLC4A3:ENST00000358055.3:exon14:c.G2035A:p.G679S,SLC4A3:ENST00000373762.3:exon14:c.G2116A:p.G706S,	2q35	.
2	222365812	C	T	GTGGCTCCTT,CCAGACAGAG	exonic	EPHA4	nonsynonymous SNV	EPHA4:ENST00000409938.1:exon5:c.G904A:p.E302K,EPHA4:ENST00000281821.2:exon4:c.G904A:p.E302K,EPHA4:ENST00000409854.1:exon4:c.G904A:p.E302K,EPHA4:ENST00000392071.4:exon3:c.G751A:p.E251K,	2q36.1	.
2	222365813	C	T	TGGCTCCTTC,CAGACAGAGT	exonic	EPHA4	stopgain	EPHA4:ENST00000409938.1:exon5:c.G903A:p.W301X,EPHA4:ENST00000281821.2:exon4:c.G903A:p.W301X,EPHA4:ENST00000409854.1:exon4:c.G903A:p.W301X,EPHA4:ENST00000392071.4:exon3:c.G750A:p.W250X,	2q36.1	.
2	226378207	T	A	CCAGTGGCTT,TCTGTGAGAT	exonic	NYAP2	nonsynonymous SNV	NYAP2:ENST00000272907.6:exon3:c.T342A:p.F114L,	2q36.3	.
2	227958903	C	T	TCCTGGCTTC,CTGGTTTTCC	exonic	COL4A4	nonsynonymous SNV	COL4A4:ENST00000329662.7:exon20:c.G1307A:p.G436E,COL4A4:ENST00000396625.3:exon20:c.G1307A:p.G436E,	2q36.3	.
2	234113338	G	A	GGGCCACCAG,GCCTCTAGGC	exonic	INPP5D	nonsynonymous SNV	INPP5D:ENST00000359570.5:exon28:c.G3506A:p.G1169E,INPP5D:ENST00000415617.1:exon16:c.G2405A:p.G802E,INPP5D:ENST00000455936.2:exon21:c.G2798A:p.G933E,INPP5D:ENST00000450745.1:exon21:c.G2798A:p.G933E,INPP5D:ENST00000445964.1:exon16:c.G2405A:p.G802E,INPP5D:ENST000004	2q37.1	.
2	238283538	G	A	ACGGCCACGC,GACCCGGTCC	exonic	COL6A3	nonsynonymous SNV	COL6A3:ENST00000409809.1:exon6:c.C2578T:p.R860C,COL6A3:ENST00000472056.1:exon5:c.C1375T:p.R459C,COL6A3:ENST00000295550.4:exon8:c.C3196T:p.R1066C,COL6A3:ENST00000347401.3:exon7:c.C2593T:p.R865C,COL6A3:ENST00000392003.2:exon6:c.C1975T:p.R659C,COL6A3:ENST000003920(	2q37.3	COL6A3;basechange=c.3196C>A;AAchange=p.R1066S;tissue=lung&COL6A3_ENST00000392004;basechange=c.2578C>A;AAchange=p.R8
2	238494700	G	A	AGCCAAGCTG,ACTTACCCAC	exonic	RAB17	nonsynonymous SNV	RAB17:ENST00000414278.1:exon2:c.C98T:p.S33F,RAB17:ENST00000392001.2:exon2:c.C98T:p.S33F,RAB17:ENST00000264601.3:exon2:c.C98T:p.S33F,	2q37.3	.
2	239990215	G	A	CCCCCAAGAG,GGTGGGGTGG	exonic	HDAC4	nonsynonymous SNV	HDAC4:ENST00000543185.1:exon17:c.C1576T:p.P526S,HDAC4:ENST00000345617.3:exon23:c.C2824T:p.P942S,	2q37.3	.
2	240834665	C	T	ACAGACCATT,TGAGAACAGG	exonic	NDUFA10	nonsynonymous SNV	NDUFA10:ENST00000419408.1:exon5:c.G361A:p.E121K,	2q37.3	.
2	242035815	A	C	CACTCTTTAC,ATGTCTGGAT	exonic	MTERFD2	nonsynonymous SNV	MTERFD2:ENST00000414146.1:exon3:c.T255G:p.I85M,MTERFD2:ENST00000439144.1:exon4:c.T303G:p.I101M,MTERFD2:ENST00000406593.1:exon3:c.T180G:p.I60M,MTERFD2:ENST00000241527.6:exon4:c.T744G:p.I248M,MTERFD2:ENST00000391980.2:exon4:c.T744G:p.I248M,	2q37.3	.
3	407675	C	T	CCTCGTATCC,CAAATTGCAT	exonic	CHL1	nonsynonymous SNV	CHL1:ENST00000256509.2:exon15:c.C1628T:p.P543L,CHL1:ENST00000397491.2:exon14:c.C1580T:p.P527L,	3p26.3	.
3	7620373	C	T	GGCTGTGATT,CTGTCTTCCT	exonic	GRM7	nonsynonymous SNV	GRM7:ENST00000403881.1:exon8:c.C1780T:p.P594S,GRM7:ENST00000486284.1:exon8:c.C1780T:p.P594S,GRM7:ENST00000440923.3:exon8:c.C1780T:p.P594S,GRM7:ENST00000357716.4:exon8:c.C1780T:p.P594S,GRM7:ENST00000389336.4:exon8:c.C1780T:p.P594S,GRM7:ENST00000389335.3:exon8:c.C13	3p26.1	.
3	10320072	C	T	CTGGGAGGCC,GGGAAGCCTT	exonic	RP11-438J1.1	nonsynonymous SNV	TATDN2:ENST00000426850.1:exon3:c.C334T:p.R112W,TATDN2:ENST00000287652.4:exon6:c.C2071T:p.R691W,RP11-438J1.1:ENST00000437082.1:exon5:c.C1900T:p.R634W,TATDN2:ENST00000448281.2:exon6:c.C2071T:p.R691W,	3p25.3	TATDN2;basechange=c.2071C>T;AAchange=p.R691W;tissue=lung
3	14769981	G	A	CAAAAAGGAG,AGGAAGAATT	exonic	C3orf20	nonsynonymous SNV	C3orf20:ENST00000253697.3:exon12:c.G1726A:p.E576K,C3orf20:ENST00000435614.1:exon12:c.G1360A:p.E454K,C3orf20:ENST00000412910.1:exon12:c.G1360A:p.E454K,	3p25.1	.
3	15476009	C	T	GAAAGATAAC,CCTCACCTGA	exonic	EAF1	nonsynonymous SNV	EAF1:ENST00000396842.2:exon4:c.C490T:p.P164S,EAF1:ENST00000432764.2:exon3:c.C187T:p.P63S,	3p25.1	.
3	16254260	C	T	GAGGCCTTCT,CTTGAGCAAG	exonic	GALNT15	nonsynonymous SNV	GALNT15:ENST00000339732.5:exon6:c.C1382T:p.S461F,GALNT15:ENST00000437509.1:exon6:c.C1382T:p.S461F,	3p25.1	.
3	36873093	G	A	TAGAGGCCAC,GACTATGGAC	exonic	TRANK1	nonsynonymous SNV	TRANK1:ENST00000429976.2:exon21:c.C7849T:p.R2617C,TRANK1:ENST00000301807.6:exon21:c.C6199T:p.R2067C,TRANK1:ENST00000428977.2:exon11:c.C6199T:p.R2067C,	3p22.2	.
3	38889002	G	A	ATTCACTTTG,AAAACCAGTT	exonic	SCN11A	nonsynonymous SNV	SCN11A:ENST00000456224.3:exon25:c.C4445T:p.S1482F,SCN11A:ENST00000450244.1:exon26:c.C4559T:p.S1520F,SCN11A:ENST00000302328.3:exon26:c.C4559T:p.S1520F,	3p22.2	.
3	42728076	G	A	TCATCTTCAT,ATCAGTGAGG	exonic	KLHL40	nonsynonymous SNV	KLHL40:ENST00000287777.4:exon1:c.G966A:p.M322I,	3p22.1	.
3	46939926	C	T	TCCCTGGCGT,CCTCACCGTA	exonic	PTH1R	nonsynonymous SNV	PTH1R:ENST00000418619.1:exon8:c.C602T:p.S201F,PTH1R:ENST00000427125.2:exon7:c.C602T:p.S201F,PTH1R:ENST00000449590.1:exon8:c.C602T:p.S201F,PTH1R:ENST00000313049.5:exon6:c.C602T:p.S201F,PTH1R:ENST00000430002.2:exon7:c.C602T:p.S201F,PTH1R:ENST00000428220.1:exon6:c.C60	3p21.31	.
3	48719942	G	A	GAGGCTGAAG,GCCTCTGCGT	exonic	NCKIPSD	nonsynonymous SNV	NCKIPSD:ENST00000416649.2:exon3:c.C325T:p.P109S,NCKIPSD:ENST00000341520.4:exon3:c.C325T:p.P109S,NCKIPSD:ENST00000439518.1:exon3:c.C325T:p.P109S,NCKIPSD:ENST00000294129.2:exon3:c.C325T:p.P109S,NCKIPSD:ENST00000453349.1:exon3:c.C91T:p.P31S,	3p21.31	.
3	49933687	G	A	CTGGGTGGAT,GGGTGGGGGT	exonic	MST1R	nonsynonymous SNV	MST1R:ENST00000411578.2:exon10:c.C2590T:p.H864Y,MST1R:ENST00000344206.4:exon10:c.C2590T:p.H864Y,MST1R:ENST00000296474.3:exon10:c.C2590T:p.H864Y,	3p21.31	.
3	50129549	C	T	GCGTGAATCC,GAAGCAGGCG	exonic	RBM5	stopgain	RBM5:ENST00000417905.1:exon2:c.C91T:p.R31X,RBM5:ENST00000404526.2:exon3:c.C91T:p.R31X,RBM5:ENST00000437500.1:exon3:c.C91T:p.R31X,RBM5:ENST00000438369.1:exon3:c.C91T:p.R31X,RBM5:ENST00000441305.1:exon3:c.C91T:p.R31X,RBM5:ENST00000347869.3:exon3:c.C91T:p.R31X,RBM5:E	3p21.31	.
3	50385673	C	T	CTCTTGTGCC,TGTGGGTGCC	splicing	NPRL2(ENST	.	.	3p21.31	.
3	50395816	A	C	TCAGGGAAGA,AGTGGCGAAA	exonic	TMEM115	nonsynonymous SNV	TMEM115:ENST00000266025.3:exon1:c.T679G:p.F227V,	3p21.31	.
3	51198111	G	A	GGAAGAAAAG,ATTTTGTTCT	exonic	DOCK3	nonsynonymous SNV	DOCK3:ENST00000266037.9:exon12:c.G1015A:p.D339N,	3p21.2	.
3	51863691	C	T	AAAGGTGGTC,AGATGAAGAT	exonic	IQCF3	nonsynonymous SNV	IQCF3:ENST00000437810.2:exon6:c.C29T:p.P10L,IQCF3:ENST00000444293.1:exon2:c.C29T:p.P10L,IQCF3:ENST00000446775.1:exon6:c.C29T:p.P10L,IQCF3:ENST00000456080.1:exon7:c.C29T:p.P10L,IQCF3:ENST00000440739.2:exon4:c.C29T:p.P10L,	3p21.2	.
3	52090076	G	A	TTGCGCAGGC,GCGCAACATG	exonic	DUSP7	nonsynonymous SNV	DUSP7:ENST00000296483.6:exon1:c.C154T:p.R52C,DUSP7:ENST00000469623.1:exon1:c.C106T:p.R36C,DUSP7:ENST00000495880.1:exon1:c.C307T:p.R103C,	3p21.2	.
3	52835096	G	A	TGGAGAACAT,GCCCTGGAGA	exonic	ITIH3	nonsynonymous SNV	ITIH3:ENST00000449956.2:exon11:c.G1317A:p.M439I,ITIH3:ENST00000416872.2:exon11:c.G1317A:p.M439I,	3p21.1	.
3	54930861	C	T	TGAGCAGATT,CAGGGAGCTT	exonic	CACNA2D3	nonsynonymous SNV	CACNA2D3:ENST00000490478.1:exon25:c.C2050T:p.P684S,CACNA2D3:ENST00000415676.2:exon27:c.C2332T:p.P778S,CACNA2D3:ENST00000288197.5:exon26:c.C2332T:p.P778S,CACNA2D3:ENST00000474759.1:exon26:c.C2332T:p.P778S,	3p14.3	.
3	54930862	C	T	GAGCAGATTC,AGGGAGCTTC	exonic	CACNA2D3	nonsynonymous SNV	CACNA2D3:ENST00000490478.1:exon25:c.C2051T:p.P684L,CACNA2D3:ENST00000415676.2:exon27:c.C2333T:p.P778L,CACNA2D3:ENST00000288197.5:exon26:c.C2333T:p.P778L,CACNA2D3:ENST00000474759.1:exon26:c.C2333T:p.P778L,	3p14.3	.
3	58131653	C	T	CCCAGAGAGC,CACTCCAGTT	exonic	FLNB	nonsynonymous SNV	FLNB:ENST00000295956.4:exon33:c.C5431T:p.P1811S,FLNB:ENST00000357272.4:exon33:c.C5431T:p.P1811S,FLNB:ENST00000490882.1:exon34:c.C5524T:p.P1842S,FLNB:ENST00000419752.2:exon32:c.C4891T:p.P1631S,FLNB:ENST00000348383.5:exon33:c.C5431T:p.P1811S,FLNB:ENST00000358537.3:ex(	3p14.3	.
3	64085101	C	T	TCATAGTCCT,CCTGGCTCTC	exonic	PRICKLE2	nonsynonymous SNV	PRICKLE2:ENST00000564377.1:exon9:c.G2329A:p.E777K,PRICKLE2:ENST00000295902.6:exon8:c.G2161A:p.E721K,	3p14.1	.
3	64133204	G	T	GGAATCAGAG,AGTCAGAACC	exonic	PRICKLE2	nonsynonymous SNV	PRICKLE2:ENST00000564377.1:exon8:c.C1130A:p.S377Y,PRICKLE2:ENST00000295902.6:exon7:c.C962A:p.S321Y,	3p14.1	.
3	64641552	C	T	TAGAGGCTAC,TGCAACCGAG	splicing	ADAMTS9(EN	.	.	3p14.1	.
3	70000974	C	T	GTGTATTTTT,CCACAGAGTC	exonic	MITF	nonsynonymous SNV	MITF:ENST00000451708.1:exon7:c.C844T:p.P282S,MITF:ENST00000448226.2:exon7:c.C892T:p.P298S,MITF:ENST00000394351.3:exon6:c.C571T:p.P191S,	3p13	.
3	73004354	C	T	TAATTCCACC,AGCTTGCAGC	exonic	GXYLT2	stopgain	GXYLT2:ENST00000389617.4:exon4:c.C706T:p.Q236X,	3p13	.
3	74349056	C	T	GCTTCCGCCT,CTCCATTGAC	exonic	CNTN3	nonsynonymous SNV	CNTN3:ENST00000263665.6:exon16:c.G2129A:p.G710E,	3p12.3	.
3	98251835	T	G	CTTGTGCCCT,GCCTGAAAAA	exonic	GPR15	nonsynonymous SNV	GPR15:ENST00000284311.3:exon1:c.T958G:p.C320G,	3q11.2	.
3	98568347	C	T	AATCCGCGTC,AGAAACATGG	exonic	DCBLD2	nonsynonymous SNV	DCBLD2:ENST00000326840.6:exon3:c.G529A:p.G177R,DCBLD2:ENST00000326857.9:exon3:c.G529A:p.G177R,DCBLD2:ENST00000449482.1:exon4:c.G211A:p.G71R,	3q12.1	.
3	107096780	G	A	GAATGTTAAT,AAGACAAACA	exonic	CCDC54	nonsynonymous SNV	CCDC54:ENST00000261058.1:exon1:c.G346A:p.E116K,	3q13.12	.
3	108751625	C	T	AATTCTTTTT,CTGATAATTA	exonic	MORC1	nonsynonymous SNV	MORC1:ENST00000483760.1:exon16:c.G1507A:p.E503K,MORC1:ENST00000232603.5:exon16:c.G1507A:p.E503K,	3q13.13	.
3	108776194	A	T	ACTCACAAGG,CTTCAGTTTC	exonic	MORC1	nonsynonymous SNV	MORC1:ENST00000483760.1:exon13:c.T1171A:p.S391T,MORC1:ENST00000232603.5:exon13:c.T1171A:p.S391T,	3q13.13	.
3	112357275	G	A	CTTCTTTTTG,GAGGTTTCTC	exonic	CCDC80	nonsynonymous SNV	CCDC80:ENST00000439685.2:exon2:c.C1478T:p.P493L,CCDC80:ENST00000206423.3:exon2:c.C1478T:p.P493L,	3q13.2	.
3	112357276	G	A	TTCTTTTTGG,AGGTTTCTCC	exonic	CCDC80	nonsynonymous SNV	CCDC80:ENST00000439685.2:exon2:c.C1477T:p.P493S,CCDC80:ENST00000206423.3:exon2:c.C1477T:p.P493S,	3q13.2	.
3	113115460	C	T	ATATCAGCAT,CAAAATTTTC	exonic	WDR52	nonsynonymous SNV	WDR52:ENST00000393845.2:exon14:c.G1684A:p.D562N,WDR52:ENST00000295868.2:exon14:c.G1684A:p.D562N,	3q13.2	.
3	119301263	C	A	GTATTACCTC,TTGCTAAGCA	exonic	ADPRH	nonsynonymous SNV	ADPRH:ENST00000478399.1:exon2:c.C247A:p.L83I,ADPRH:ENST00000478927.1:exon2:c.C247A:p.L83I,ADPRH:ENST00000465513.1:exon1:c.C247A:p.L83I,ADPRH:ENST00000357003.3:exon3:c.C247A:p.L83I,ADPRH:ENST00000481816.1:exon3:c.C247A:p.L83I,	3q13.33	.
3	119305195	C	T	TGGAGGATTC,CTTCAACAGC	exonic	ADPRH	nonsynonymous SNV	ADPRH:ENST00000478399.1:exon3:c.C362T:p.P121L,ADPRH:ENST00000478927.1:exon3:c.C362T:p.P121L,ADPRH:ENST00000465513.1:exon2:c.C362T:p.P121L,ADPRH:ENST00000357003.3:exon4:c.C362T:p.P121L,ADPRH:ENST00000481816.1:exon4:c.C362T:p.P121L,	3q13.33	ADPRH;basechange=c.362C>T;AAchange=p.P121L;tissue=lung
3	119463041	G	A	GATATTTAAG,AGGCAAGTAG	exonic	MAATS1	nonsynonymous SNV	MAATS1:ENST00000273390.5:exon14:c.G1900A:p.E634K,	3q13.33	.
3	120428726	C	T	TAGGTCTTCT,TTCTGGGGTT	exonic	RABL3	nonsynonymous SNV	RABL3:ENST00000273375.3:exon3:c.G169A:p.E57K,RABL3:ENST00000483733.1:exon3:c.G169A:p.E57K,RABL3:ENST00000473654.1:exon3:c.G169A:p.E57K,RABL3:ENST00000465022.1:exon3:c.G169A:p.E57K,	3q13.33	.
3	121134770	G	A	CTTTCTCAAG,GACTGTTTGG	exonic	STXBP5L	nonsynonymous SNV	STXBP5L:ENST00000273666.6:exon26:c.G3253A:p.G1085R,STXBP5L:ENST00000471454.1:exon25:c.G3181A:p.G1061R,STXBP5L:ENST00000471262.1:exon24:c.G3082A:p.G1028R,	3q13.33	.
3	121342158	C	T	GAGGTATTCC,ATGGAGGCAC	exonic	FBXO40	nonsynonymous SNV	FBXO40:ENST00000338040.4:exon3:c.C1882T:p.H628Y,	3q13.33	.
3	121822533	C	T	AGTGTTCATT,CAAGTATATG	exonic	CD86	nonsynonymous SNV	CD86:ENST00000393627.2:exon3:c.C221T:p.S74F,CD86:ENST00000330540.2:exon3:c.C239T:p.S80F,CD86:ENST00000482356.1:exon3:c.C221T:p.S74F,	3q13.33	.
3	122630864	C	A	GGCTTCGGCT,TGGGCTCCGT	exonic	SEMA5B	nonsynonymous SNV	SEMA5B:ENST00000393583.2:exon20:c.G2949T:p.Q983H,SEMA5B:ENST00000357599.3:exon20:c.G2949T:p.Q983H,SEMA5B:ENST00000451055.2:exon20:c.G3111T:p.Q1037H,SEMA5B:ENST00000475244.1:exon20:c.G2949T:p.Q983H,SEMA5B:ENST00000195173.4:exon20:c.G2943T:p.Q981H,	3q21.1	.
3	122632135	A	G	CGGGTCTGCA,GGGGCGCGCG	exonic	SEMA5B	nonsynonymous SNV	SEMA5B:ENST00000393583.2:exon17:c.T2417C:p.L806P,SEMA5B:ENST00000357599.3:exon17:c.T2417C:p.L806P,SEMA5B:ENST00000451055.2:exon17:c.T2579C:p.L860P,SEMA5B:ENST00000475244.1:exon17:c.T2417C:p.L806P,SEMA5B:ENST00000195173.4:exon17:c.T2414C:p.L805P,	3q21.1	.
3	122825650	C	T	GGAGGAAGAT,CTGGAGCCAA	exonic	PDIA5	nonsynonymous SNV	PDIA5:ENST00000316218.7:exon6:c.C442T:p.P148S,PDIA5:ENST00000489923.1:exon6:c.C442T:p.P148S,PDIA5:ENST00000484644.1:exon6:c.C154T:p.P52S,	3q21.1	.
3	125855612	C	T	CTCACACTTC,ATCGGTGGGA	exonic	ALDH1L1	nonsynonymous SNV	ALDH1L1:ENST00000452905.2:exon9:c.G1036A:p.G346R,ALDH1L1:ENST00000393431.2:exon11:c.G1339A:p.G447R,ALDH1L1:ENST00000273450.3:exon11:c.G1369A:p.G457R,ALDH1L1:ENST00000393434.2:exon11:c.G1339A:p.G447R,ALDH1L1:ENST00000472186.1:exon11:c.G1339A:p.G447R,	3q21.3	.
3	127327301	C	T	CTGCCCCGCT,CAAGGACGCC	exonic	MCM2	nonsynonymous SNV	MCM2:ENST00000265056.7:exon7:c.C1178T:p.S393F,	3q21.3	.
3	127379815	C	T	ACCACAGCTC,CAGTGGGGCC	exonic	PODXL2	nonsynonymous SNV	PODXL2:ENST00000342480.6:exon3:c.C944T:p.P315L,	3q21.3	.
3	129130159	G	A	TTCTTGAGCG,ACCCTTCAGG	exonic	EFCAB12	nonsynonymous SNV	EFCAB12:ENST00000505956.1:exon5:c.C877T:p.P293S,EFCAB12:ENST00000326085.3:exon5:c.C877T:p.P293S,EFCAB12:ENST00000503957.1:exon4:c.C427T:p.P143S,	3q21.3	.
3	130285857	C	A	AGCAAAGAAG,AGCGAGGAAA	exonic	COL6A6	nonsynonymous SNV	COL6A6:ENST00000453409.2:exon4:c.C1594A:p.Q532K,COL6A6:ENST00000358511.6:exon4:c.C1594A:p.Q532K,	3q22.1	.
3	130308021	A	T	ACCACCAGGG,AAAGGGTGAT	exonic	COL6A6	stopgain	COL6A6:ENST00000453409.2:exon11:c.A4213T:p.K1405X,COL6A6:ENST00000358511.6:exon11:c.A4213T:p.K1405X,	3q22.1	.
3	130340710	G	A	AGGCCGTTTG,GAAGCCAAGG	exonic	COL6A6	nonsynonymous SNV	COL6A6:ENST00000453409.2:exon23:c.G4861A:p.G1621R,COL6A6:ENST00000358511.6:exon23:c.G4861A:p.G1621R,	3q22.1	.
3	130340711	G	A	GGCCGTTTGG,AAGCCAAGGA	exonic	COL6A6	nonsynonymous SNV	COL6A6:ENST00000453409.2:exon23:c.G4862A:p.G1621E,COL6A6:ENST00000358511.6:exon23:c.G4862A:p.G1621E,	3q22.1	.
3	130454760	G	A	ACTTGTTCAG,GAAAAAATGT	exonic	PIK3R4	nonsynonymous SNV	PIK3R4:ENST00000356763.3:exon3:c.C820T:p.P274S,	3q22.1	.
3	137484015	C	T	GCCTTCTTGC,GCCGGCCTCG	exonic	SOX14	nonsynonymous SNV	SOX14:ENST00000306087.1:exon1:c.C389T:p.P130L,	3q22.3	.
3	138224144	G	A	TACCTTTAAG,ATGTCAGTTG	exonic	CEP70	nonsynonymous SNV	CEP70:ENST00000264982.3:exon13:c.C1214T:p.S405F,CEP70:ENST00000481834.1:exon13:c.C1214T:p.S405F,CEP70:ENST00000484888.1:exon13:c.C1214T:p.S405F,CEP70:ENST00000474781.1:exon13:c.C1160T:p.S387F,CEP70:ENST00000542237.1:exon12:c.C1154T:p.S385F,CEP70:ENST00000489254.1:e	3q22.3	.
3	138739488	G	A	CTGGGGCTGC,GGGCCGGCTG	exonic	PRR23B	nonsynonymous SNV	PRR23B:ENST00000329447.5:exon1:c.C16T:p.R6C,	3q23	PRR23B;basechange=c.16C>T;AAchange=p.R6C;tissue=kidney
3	140406996	C	T	GAGCTCTTCC,CACAGGGCCC	exonic	TRIM42	nonsynonymous SNV	TRIM42:ENST00000286349.3:exon3:c.C1472T:p.P491L,	3q23	.
3	141011457	C	T	CGTGGATGTC,CCACCAAGCA	exonic	ACPL2	nonsynonymous SNV	ACPL2:ENST00000508812.1:exon5:c.C826T:p.P276S,ACPL2:ENST00000286353.4:exon6:c.C853T:p.P285S,ACPL2:ENST00000393007.1:exon5:c.C805T:p.P269S,ACPL2:ENST00000393010.2:exon8:c.C853T:p.P285S,ACPL2:ENST00000504264.1:exon5:c.C802T:p.P268S,ACPL2:ENST00000502783.1:exon7:c.C73	3q23	.
3	141011458	C	T	GTGGATGTCC,CACCAAGCAG	exonic	ACPL2	nonsynonymous SNV	ACPL2:ENST00000508812.1:exon5:c.C827T:p.P276L,ACPL2:ENST00000286353.4:exon6:c.C854T:p.P285L,ACPL2:ENST00000393007.1:exon5:c.C806T:p.P269L,ACPL2:ENST00000393010.2:exon8:c.C854T:p.P285L,ACPL2:ENST00000504264.1:exon5:c.C803T:p.P268L,ACPL2:ENST00000502783.1:exon7:c.C740	3q23	.
3	148459775	A	C	CAGCTTCTAA,ATATATTCCC	exonic	AGTR1	nonsynonymous SNV	AGTR1:ENST00000542281.1:exon4:c.A953C:p.K318T,AGTR1:ENST00000461609.1:exon3:c.A953C:p.K318T,AGTR1:ENST00000349243.3:exon3:c.A953C:p.K318T,AGTR1:ENST00000404754.2:exon2:c.A953C:p.K318T,AGTR1:ENST00000474935.1:exon3:c.A953C:p.K318T,AGTR1:ENST00000475347.1:exon2:c.A9	3q24	.
3	149220463	G	A	TACCTTCTAG,GAGATGGACC	exonic	TM4SF4	nonsynonymous SNV	TM4SF4:ENST00000305354.4:exon5:c.G592A:p.G198R,	3q25.1	.
3	149220464	G	A	ACCTTCTAGG,AGATGGACCC	exonic	TM4SF4	nonsynonymous SNV	TM4SF4:ENST00000305354.4:exon5:c.G593A:p.G198E,	3q25.1	.
3	150883255	C	T	ACAAAGAGGC,GTGCAGTGGA	exonic	MED12L	nonsynonymous SNV	MED12L:ENST00000273432.4:exon7:c.C886T:p.R296C,MED12L:ENST00000422248.2:exon9:c.C1306T:p.R436C,MED12L:ENST00000309237.4:exon9:c.C1306T:p.R436C,MED12L:ENST00000474524.1:exon9:c.C1306T:p.R436C,	3q25.1	.
3	151056288	C	T	GTTATCAGTC,CAGGAATGAA	exonic	P2RY12	nonsynonymous SNV	P2RY12:ENST00000302632.3:exon3:c.G346A:p.G116R,	3q25.1	.
3	151166154	G	A	TCAGCCATCT,GAGTTCCAAT	exonic	IGSF10	stopgain	IGSF10:ENST00000282466.3:exon4:c.C1615T:p.Q539X,	3q25.1	.
3	152058510	C	T	CTCAAATGTT,CTTTGGATTC	exonic	TMEM14E	nonsynonymous SNV	TMEM14E:ENST00000408960.3:exon1:c.G184A:p.E62K,	3q25.1	.
3	152165431	C	T	GCTGTACTTC,CCCATTACCA	exonic	MBNL1	nonsynonymous SNV	MBNL1:ENST00000357472.3:exon5:c.C830T:p.P277L,MBNL1:ENST00000282488.7:exon5:c.C626T:p.P209L,MBNL1:ENST00000545754.1:exon4:c.C626T:p.P209L,MBNL1:ENST00000485910.1:exon4:c.C626T:p.P209L,MBNL1:ENST00000324210.5:exon6:c.C830T:p.P277L,MBNL1:ENST00000463374.1:exon6:c.C88	3q25.2	.
3	155198996	G	A	TTGTTTCTAA,AACCACTCCT	exonic	PLCH1	nonsynonymous SNV	PLCH1:ENST00000334686.6:exon22:c.C4729T:p.L1577F,PLCH1:ENST00000340059.7:exon23:c.C4843T:p.L1615F,PLCH1:ENST00000414191.1:exon22:c.C4729T:p.L1577F,PLCH1:ENST00000460012.1:exon23:c.C4729T:p.L1577F,	3q25.31	.
3	160804148	G	A	ATCCTCTAAG,ACAATGCCAA	exonic	B3GALNT1	nonsynonymous SNV	B3GALNT1:ENST00000392779.2:exon4:c.C395T:p.S132F,B3GALNT1:ENST00000488170.1:exon5:c.C395T:p.S132F,B3GALNT1:ENST00000320474.4:exon5:c.C395T:p.S132F,B3GALNT1:ENST00000473285.1:exon5:c.C395T:p.S132F,B3GALNT1:ENST00000392781.2:exon8:c.C395T:p.S132F,B3GALNT1:ENST000(	3q26.1	.
3	167051659	G	A	GGTTTATGTT,AATTTTACTG	exonic	ZBBX	stopgain	ZBBX:ENST00000392767.2:exon7:c.C643T:p.Q215X,ZBBX:ENST00000307529.5:exon7:c.C643T:p.Q215X,ZBBX:ENST00000455345.2:exon9:c.C643T:p.Q215X,ZBBX:ENST00000392766.2:exon10:c.C643T:p.Q215X,ZBBX:ENST00000392764.1:exon10:c.C556T:p.Q186X,	3q26.1	.
3	167196693	A	T	TCACTTCACA,CTTTTTTCTT	exonic	WDR49	nonsynonymous SNV	WDR49:ENST00000308378.3:exon15:c.T2067A:p.S689R,WDR49:ENST00000476376.1:exon13:c.T1542A:p.S514R,WDR49:ENST00000453925.2:exon14:c.T1962A:p.S654R,	3q26.1	.
3	167344843	G	A	TTTACTGGGA,GAATTCAAGG	exonic	WDR49	nonsynonymous SNV	WDR49:ENST00000479765.1:exon3:c.C403T:p.L135F,	3q26.1	.
3	167728143	G	A	TGTTCCTCTC,GCCTTTGGGG	exonic	GOLIM4	stopgain	GOLIM4:ENST00000470487.1:exon16:c.C2005T:p.R669X,GOLIM4:ENST00000309027.4:exon15:c.C1921T:p.R641X,	3q26.2	.
3	169511587	C	T	TCTGTATTGT,TGGTTTTAGA	exonic	LRRC34	nonsynonymous SNV	LRRC34:ENST00000316515.7:exon10:c.G1096A:p.D366N,LRRC34:ENST00000446859.1:exon11:c.G1231A:p.D411N,LRRC34:ENST00000522830.1:exon11:c.G1048A:p.D350N,LRRC34:ENST00000522526.2:exon10:c.G1135A:p.D379N,	3q26.2	.
3	169998096	G	A	AATAGGAAGA,GAAGTTATGC	exonic	PRKCI	nonsynonymous SNV	PRKCI:ENST00000295797.4:exon9:c.G787A:p.G263R,	3q26.2	.
3	170244689	C	T	CCCCACTGCA,CCGCCGGGGG	exonic	SLC7A14	nonsynonymous SNV	SLC7A14:ENST00000231706.5:exon2:c.G37A:p.V13M,	3q26.2	.
3	171571491	C	T	TGCAAAATTC,CATTAGCCTG	exonic	TMEM212	nonsynonymous SNV	TMEM212:ENST00000334567.5:exon3:c.C394T:p.P132S,TMEM212:ENST00000450693.1:exon3:c.C262T:p.P88S,	3q26.31	.
3	179458102	C	T	CCGGAATTCT,CTCTAACAGG	exonic	USP13	nonsynonymous SNV	USP13:ENST00000497155.1:exon4:c.C260T:p.S87F,USP13:ENST00000496897.1:exon11:c.C1127T:p.S376F,USP13:ENST00000263966.3:exon11:c.C1322T:p.S441F,	3q26.33	.
3	180359791	C	T	CTTATGTTTT,CCGTTCTTGA	exonic	CCDC39	nonsynonymous SNV	CCDC39:ENST00000273654.4:exon19:c.G2116A:p.E706K,CCDC39:ENST00000442201.2:exon13:c.G1864A:p.E622K,CCDC39:ENST00000476379.1:exon19:c.G1864A:p.E622K,	3q26.33	.
3	183888436	C	T	CCCAGGAGCC,CTCCGGGCCG	exonic	DVL3	nonsynonymous SNV	DVL3:ENST00000313143.3:exon15:c.C2044T:p.P682S,DVL3:ENST00000431765.1:exon15:c.C1993T:p.P665S,	3q27.1	.
3	190573586	C	T	AGAGAGGTTT,TTTTGGCATT	exonic	GMNC	nonsynonymous SNV	GMNC:ENST00000442080.1:exon5:c.G503A:p.R168K,	3q28	.
3	193042694	C	T	AGAGGTCTC,CTGGATGGTC	exonic	ATP13A5	nonsynonymous SNV	ATP13A5:ENST00000342358.4:exon14:c.G1633A:p.G545R,	3q29	.
3	193182863	G	A	AGAGCCGGAG,AACCGCAATT	exonic	ATP13A4	nonsynonymous SNV	ATP13A4:ENST00000342695.4:exon12:c.C1327T:p.P443S,ATP13A4:ENST00000392443.3:exon12:c.C1270T:p.P424S,ATP13A4:ENST00000295548.3:exon12:c.C1327T:p.P443S,	3q29	.
3	193207584	A	G	GCAAAAGCAT,TTCCTTATAG	exonic	ATP13A4	nonsynonymous SNV	ATP13A4:ENST00000342695.4:exon7:c.T673C:p.Y225H,ATP13A4:ENST00000392443.3:exon7:c.T673C:p.Y225H,ATP13A4:ENST00000295548.3:exon7:c.T673C:p.Y225H,	3q29	.
3	194117721	G	A	CCCAGGAAGG,CCCCAGGCCA	exonic	GP5	nonsynonymous SNV	GP5:ENST00000401815.1:exon1:c.C1291T:p.P431S,GP5:ENST00000323007.3:exon2:c.C1291T:p.P431S,	3q29	.
3	195481127	C	A	GAGGGCAGGC,TCGCAGCCCT	exonic	MUC4	nonsynonymous SNV	MUC4:ENST00000475231.1:exon18:c.G15129T:p.E5043D,MUC4:ENST00000349607.4:exon17:c.G2424T:p.E808D,MUC4:ENST00000346145.4:exon18:c.G2577T:p.E859D,MUC4:ENST00000463781.3:exon19:c.G15285T:p.E5095D,	3q29	.
3	195517135	G	A	AGAACTGGGG,AGAGTGCTGT	exonic	MUC4	nonsynonymous SNV	MUC4:ENST00000477756.1:exon2:c.C1316T:p.S439F,MUC4:ENST00000475231.1:exon2:c.C1316T:p.S439F,MUC4:ENST00000480843.1:exon2:c.C1316T:p.S439F,MUC4:ENST00000479406.1:exon2:c.C1316T:p.S439F,MUC4:ENST00000478156.1:exon2:c.C1316T:p.S439F,MUC4:ENST00000477086.1:exon2:c.C13	3q29	.
3	195595025	G	A	GGTCTGTGCG,AGCTGGGCGG	exonic	TNK2	nonsynonymous SNV	TNK2:ENST00000392400.1:exon12:c.C2099T:p.S700F,TNK2:ENST00000428187.1:exon12:c.C2195T:p.S732F,TNK2:ENST00000333602.6:exon12:c.C2099T:p.S700F,TNK2:ENST00000416152.1:exon4:c.C800T:p.S267F,TNK2:ENST00000381916.2:exon13:c.C2333T:p.S778F,	3q29	.
3	196675122	G	A	ATGCCTCCAA,AAGCCAGCTG	exonic	PIGZ	nonsynonymous SNV	PIGZ:ENST00000412723.1:exon3:c.C646T:p.L216F,	3q29	PIGZ;basechange=c.646C>T;AAchange=p.L216F;tissue=lung
3	197557666	C	T	CTCAAGTCGC,CTTATGGAGC	exonic	LRCH3	nonsynonymous SNV	LRCH3:ENST00000425562.2:exon7:c.C913T:p.P305S,LRCH3:ENST00000438796.2:exon7:c.C913T:p.P305S,LRCH3:ENST00000334859.4:exon7:c.C913T:p.P305S,LRCH3:ENST00000441090.2:exon4:c.C535T:p.P179S,LRCH3:ENST00000414675.2:exon7:c.C913T:p.P305S,	3q29	.
4	997173	G	A	CCCGCCGGCG,CCGCCTGACC	exonic	IDUA	nonsynonymous SNV	IDUA:ENST00000247933.4:exon11:c.G1565A:p.G522D,IDUA:ENST00000453894.1:exon8:c.G1631A:p.G544D,IDUA:ENST00000514224.1:exon10:c.G1169A:p.G390D,	4p16.3	.
4	2062737	C	T	CTGTGCTTCG,CGTGAGCCGC	exonic	NAT8L	nonsynonymous SNV	NAT8L:ENST00000423729.2:exon2:c.C389T:p.A130V,	4p16.3	.
4	3445805	G	A	TGCCTCAATG,AGGCTCCTGC	exonic	HGFAC	nonsynonymous SNV	HGFAC:ENST00000382774.3:exon5:c.G515A:p.G172E,HGFAC:ENST00000511533.1:exon5:c.G515A:p.G172E,	4p16.3	.
4	4275396	A	G	ATCTGTTTCA,CTAAGTATTT	exonic	LYAR	nonsynonymous SNV	LYAR:ENST00000452476.1:exon8:c.T833C:p.V278A,LYAR:ENST00000343470.4:exon8:c.T833C:p.V278A,	4p16.3	.
4	6998060	C	T	AGAAATCTCC,AGCAAAACCA	exonic	TBC1D14	nonsynonymous SNV	TBC1D14:ENST00000448507.1:exon5:c.C971T:p.P324L,TBC1D14:ENST00000409757.4:exon5:c.C971T:p.P324L,TBC1D14:ENST00000410031.1:exon4:c.C287T:p.P96L,TBC1D14:ENST00000451522.2:exon3:c.C131T:p.P44L,	4p16.1	.
4	16193061	C	A	GAGTAGTCAA,ATAGTGCATC	exonic	TAPT1	nonsynonymous SNV	TAPT1:ENST00000304584.8:exon1:c.G13T:p.V5F,TAPT1:ENST00000405303.2:exon4:c.G535T:p.V179F,TAPT1:ENST00000399920.3:exon3:c.G202T:p.V68F,	4p15.32	.
4	20550133	A	G	CAACAGAATA,GCACGCTTTC	exonic	SLIT2	nonsynonymous SNV	SLIT2:ENST00000503837.1:exon23:c.A2356G:p.S786G,SLIT2:ENST00000273739.5:exon24:c.A2380G:p.S794G,SLIT2:ENST00000503823.1:exon22:c.A2344G:p.S782G,SLIT2:ENST00000504154.1:exon23:c.A2368G:p.S790G,	4p15.31	.
4	25759371	G	A	CCAGGCCAAG,AGCAGGGGCT	exonic	SEL1L3	nonsynonymous SNV	SEL1L3:ENST00000502949.1:exon22:c.C2666T:p.S889F,SEL1L3:ENST00000399878.3:exon22:c.C3125T:p.S1042F,SEL1L3:ENST00000264868.5:exon22:c.C3020T:p.S1007F,	4p15.2	.
4	26417172	T	G	TGGAACGCGA,GGTTGTTCTG	exonic	RBPJ	nonsynonymous SNV	RBPJ:ENST00000504938.1:exon6:c.T165G:p.D55E,RBPJ:ENST00000514675.1:exon5:c.T228G:p.D76E,RBPJ:ENST00000515573.1:exon4:c.T165G:p.D55E,RBPJ:ENST00000342295.1:exon5:c.T270G:p.D90E,RBPJ:ENST00000514730.1:exon5:c.T228G:p.D76E,RBPJ:ENST00000361572.6:exon4:c.T270G:p.D90E,RE	4p15.2	.
4	36295287	C	T	TTAAATTACC,TCCAGGAGTT	exonic	DTHD1	nonsynonymous SNV	DTHD1:ENST00000456874.2:exon3:c.C983T:p.P328L,DTHD1:ENST00000507598.1:exon3:c.C1103T:p.P368L,DTHD1:ENST00000357504.3:exon3:c.C488T:p.P163L,	4p14	.
4	40356430	G	A	CAATTCCAAG,GGAGTGAATG	exonic	CHRNA9	nonsynonymous SNV	CHRNA9:ENST00000310169.2:exon5:c.G1333A:p.G445R,	4p14	.
4	44685291	C	A	GGTTGAAAAC,AAATTGAGAA	exonic	GUF1	nonsynonymous SNV	GUF1:ENST00000281543.5:exon6:c.C625A:p.Q209K,	4p12	GUF1;basechange=c.625C>T;AAchange=p.Q209*;tissue=skin
4	47939174	T	A	ATCAACTGTT,TTTTGTTGGT	exonic	CNGA1	nonsynonymous SNV	CNGA1:ENST00000514170.1:exon11:c.A1337T:p.K446I,CNGA1:ENST00000358519.4:exon9:c.A1337T:p.K446I,CNGA1:ENST00000420489.2:exon11:c.A1337T:p.K446I,CNGA1:ENST00000402813.3:exon10:c.A1544T:p.K515I,CNGA1:ENST00000544810.1:exon10:c.A1337T:p.K446I,	4p12	.
4	48487027	G	A	GCTGGGCTTG,ATCAACACCC	exonic	SLC10A4	stopgain	SLC10A4:ENST00000273861.4:exon2:c.G669A:p.W223X,	4p11	.
4	55599285	G	A	ACTCATGGTC,GATCACAAAG	exonic	KIT	nonsynonymous SNV	KIT:ENST00000288135.5:exon17:c.G2411A:p.R804Q,KIT:ENST00000412167.2:exon17:c.G2399A:p.R800Q,	4q12	.
4	56290714	G	A	GGACCCCTAT,GTGTAGCCGT	exonic	TMEM165	nonsynonymous SNV	TMEM165:ENST00000542052.1:exon6:c.G613A:p.G205S,TMEM165:ENST00000506198.1:exon2:c.G217A:p.G73S,TMEM165:ENST00000381334.5:exon5:c.G802A:p.G268S,	4q12	.
4	62761499	G	A	GGATCCCCAA,GTCCAGATCT	exonic	LPHN3	nonsynonymous SNV	LPHN3:ENST00000514157.1:exon8:c.G1630A:p.G544S,LPHN3:ENST00000506700.1:exon8:c.G1630A:p.G544S,LPHN3:ENST00000514996.1:exon8:c.G1630A:p.G544S,LPHN3:ENST00000508693.1:exon9:c.G1834A:p.G612S,LPHN3:ENST00000511324.1:exon9:c.G1834A:p.G612S,LPHN3:ENST00000545650.1:exo	4q13.1	.
4	62903551	G	A	TCGAACTCCT,GACGCTACTC	exonic	LPHN3	nonsynonymous SNV	LPHN3:ENST00000514157.1:exon20:c.G3463A:p.G1155R,LPHN3:ENST00000506700.1:exon20:c.G3463A:p.G1155R,LPHN3:ENST00000514996.1:exon20:c.G3463A:p.G1155R,LPHN3:ENST00000508693.1:exon22:c.G3694A:p.G1232R,LPHN3:ENST00000511324.1:exon21:c.G3667A:p.G1223R,LPHN3:ENST00000	4q13.1	.
4	68378171	G	A	GAAATTCTTC,ACTTTTCGTG	exonic	CENPC	stopgain	CENPC:ENST00000273853.6:exon9:c.C1561T:p.R521X,CENPC:ENST00000506882.1:exon9:c.C1561T:p.R521X,CENPC:ENST00000513216.1:exon5:c.C1282T:p.R428X,	4q13.2	.
4	71255416	C	T	GGGACCATAT,CACCTGGACC	exonic	SMR3B	nonsynonymous SNV	SMR3B:ENST00000504825.1:exon4:c.C91T:p.P31S,SMR3B:ENST00000304915.3:exon3:c.C91T:p.P31S,	4q13.3	.
4	71346937	C	T	CCAGTGAATT,CCCAGCTCCA	exonic	MUC7	nonsynonymous SNV	MUC7:ENST00000456088.1:exon4:c.C476T:p.S159F,MUC7:ENST00000304887.5:exon3:c.C476T:p.S159F,MUC7:ENST00000413702.1:exon4:c.C476T:p.S159F,	4q13.3	.
4	71472224	G	A	AAGATGAAGG,ACTCCCCAGC	exonic	AMBN	nonsynonymous SNV	AMBN:ENST00000322937.6:exon13:c.G1121A:p.G374E,AMBN:ENST00000449493.2:exon13:c.G1076A:p.G359E,	4q13.3	.
4	73956389	G	C	CTCACCTGAG,GACTTGGAGC	exonic	ANKRD17	nonsynonymous SNV	ANKRD17:ENST00000358602.4:exon29:c.C6956G:p.P2319R,ANKRD17:ENST00000509867.2:exon29:c.C6617G:p.P2206R,ANKRD17:ENST00000330838.6:exon28:c.C6203G:p.P2068R,	4q13.3	.
4	74350088	C	T	AAGACGCTCC,AGAGTGTTCA	exonic	AFM	nonsynonymous SNV	AFM:ENST00000226355.3:exon3:c.C251T:p.P84L,	4q13.3	.
4	77255293	C	T	GTTGGATCTT,CTTCAGTGTG	exonic	CCDC158	nonsynonymous SNV	CCDC158:ENST00000388914.3:exon18:c.G2692A:p.E898K,	4q21.1	.
4	77317627	G	T	CACAAAAAAT,AACTTGAAGA	exonic	CCDC158	stopgain	CCDC158:ENST00000388914.3:exon3:c.C83A:p.S28X,CCDC158:ENST00000434846.2:exon3:c.C83A:p.S28X,	4q21.1	.
4	77651986	C	T	CGACCTCACT,GTGGCACACA	exonic	SHROOM3	nonsynonymous SNV	SHROOM3:ENST00000296043.6:exon4:c.C485T:p.S162L,	4q21.1	.
4	83996461	C	T	ACGAGAAGCC,TGCCAACGTG	exonic	COPS4	nonsynonymous SNV	COPS4:ENST00000511653.1:exon11:c.C1247T:p.P416L,	4q21.22	.
4	85626622	T	A	CCCATTGGCT,AGCCAGGTTT	exonic	WDFY3	stopgain	WDFY3:ENST00000295888.4:exon54:c.A8260T:p.K2754X,WDFY3:ENST00000322366.6:exon53:c.A8209T:p.K2737X,WDFY3:ENST00000514711.1:exon10:c.A1069T:p.K357X,	4q21.23	.
4	85717785	A	C	CACCCTGGTC,GGGGTACAGT	exonic	WDFY3	nonsynonymous SNV	WDFY3:ENST00000295888.4:exon19:c.T3056G:p.L1019R,WDFY3:ENST00000322366.6:exon19:c.T3056G:p.L1019R,	4q21.23	.
4	87967418	C	T	AAAGATTCCC,TTTTTGGAGA	exonic	AFF1	nonsynonymous SNV	AFF1:ENST00000507468.1:exon3:c.C139T:p.L47F,AFF1:ENST00000307808.6:exon2:c.C118T:p.L40F,AFF1:ENST00000395146.4:exon3:c.C139T:p.L47F,AFF1:ENST00000503477.1:exon3:c.C139T:p.L47F,	4q21.3	.
4	88534146	G	T	AGCAGGGAAT,GAAAAGACAG	exonic	DSPP	stopgain	DSPP:ENST00000282478.7:exon3:c.G808T:p.G270X,DSPP:ENST00000399271.1:exon4:c.G808T:p.G270X,	4q22.1	.
4	96073823	C	T	GCTTCCTTAT,ATGACCTAGT	exonic	BMPR1B	nonsynonymous SNV	BMPR1B:ENST00000394931.1:exon9:c.C1282T:p.H428Y,BMPR1B:ENST00000264568.4:exon10:c.C1282T:p.H428Y,BMPR1B:ENST00000515059.1:exon12:c.C1282T:p.H428Y,BMPR1B:ENST00000512312.1:exon10:c.C1282T:p.H428Y,BMPR1B:ENST00000509540.1:exon10:c.C1282T:p.H428Y,BMPR1B:ENST0000	4q22.3	.
4	102981520	A	C	GCAGATGGGA,AAAGTGGCCT	exonic	BANK1	nonsynonymous SNV	BANK1:ENST00000444316.2:exon12:c.A2032C:p.K678Q,BANK1:ENST00000428908.1:exon11:c.A1723C:p.K575Q,BANK1:ENST00000504592.1:exon16:c.A2077C:p.K693Q,BANK1:ENST00000508653.1:exon11:c.A1723C:p.K575Q,BANK1:ENST00000322953.4:exon12:c.A2122C:p.K708Q,	4q24	.
4	114276469	A	G	GAAGCTATA,GCATGAAGGC	exonic	ANK2	nonsynonymous SNV	ANK2:ENST00000357077.4:exon38:c.A6695G:p.K2232R,ANK2:ENST00000264366.6:exon37:c.A6596G:p.K2199R,	4q26	.
4	114278352	G	A	TGAAGGAAAA,AATTAGATGA	exonic	ANK2	nonsynonymous SNV	ANK2:ENST00000357077.4:exon38:c.G8578A:p.E2860K,ANK2:ENST00000264366.6:exon37:c.G8479A:p.E2827K,	4q26	.
4	114290912	A	G	TCTGCGATA,CCAGCCTGAG	exonic	ANK2	nonsynonymous SNV	ANK2:ENST00000394537.3:exon42:c.A5306G:p.N1769S,ANK2:ENST00000506722.1:exon44:c.A5279G:p.N1760S,ANK2:ENST00000509550.1:exon21:c.A2834G:p.N945S,ANK2:ENST00000357077.4:exon43:c.A11561G:p.N3854S,ANK2:ENST00000264366.6:exon42:c.A11462G:p.N3821S,ANK2:ENST00000505342.1	4q26	.
4	114438777	G	A	ATCCCAGAAG,GTGGATACCC	exonic	CAMK2D	nonsynonymous SNV	CAMK2D:ENST00000508738.1:exon9:c.C638T:p.P213L,CAMK2D:ENST00000514328.1:exon9:c.C638T:p.P213L,CAMK2D:ENST00000429180.1:exon9:c.C638T:p.P213L,CAMK2D:ENST00000342666.5:exon9:c.C638T:p.P213L,CAMK2D:ENST00000505990.1:exon9:c.C638T:p.P213L,CAMK2D:ENST00000511664.1:e	4q26	.
4	134084222	G	A	CCTTCTGATG,ACGCCAGGCT	exonic	PCDH10	nonsynonymous SNV	PCDH10:ENST00000264360.5:exon4:c.G2888A:p.G963E,	4q28.3	PCDH10;basechange=c.2888G>C;AAchange=p.G963A;tissue=lung
4	138450811	T	C	TGGCACGTGG,TTGATGAGAT	exonic	PCDH18	nonsynonymous SNV	PCDH18:ENST00000412923.2:exon1:c.A2432G:p.N811S,PCDH18:ENST00000344876.4:exon1:c.A2432G:p.N811S,PCDH18:ENST00000510305.1:exon2:c.A65G:p.N22S,PCDH18:ENST00000507846.1:exon2:c.A1772G:p.N591S,	4q28.3	.
4	141395974	A	G	TACATCTGCA,CCAAGACAAT	exonic	RP11-542P2.	nonsynonymous SNV	RP11-542P2.1:ENST00000511113.1:exon4:c.T518C:p.V173A,RP11-542P2.1:ENST00000515402.1:exon2:c.T221C:p.V74A,RP11-542P2.1:ENST00000503109.2:exon4:c.T518C:p.V173A,	4q31.1	.
4	144619884	C	T	TCCATTATGT,TCTCTGTAGC	exonic	FREM3	nonsynonymous SNV	FREM3:ENST00000329798.5:exon1:c.G1945A:p.D649N,	4q31.21	.
4	149075921	C	T	ACCGAGGGTT,TTTTGCAGGA	exonic	NR3C2	nonsynonymous SNV	NR3C2:ENST00000358102.3:exon5:c.G2146A:p.E716K,NR3C2:ENST00000511528.1:exon4:c.G2158A:p.E720K,NR3C2:ENST00000344721.4:exon5:c.G2146A:p.E716K,NR3C2:ENST00000355292.3:exon5:c.G2158A:p.E720K,	4q31.23	.
4	153897394	C	T	TTCAAGAAGC,CAGTGCCAAA	exonic	FHDC1	nonsynonymous SNV	FHDC1:ENST00000260008.3:exon11:c.C2951T:p.P984L,FHDC1:ENST00000511601.1:exon12:c.C2951T:p.P984L,	4q31.3	.
4	155157220	C	G	ACTAAGGAGG,AGTTGCATCA	exonic	DCHS2	nonsynonymous SNV	DCHS2:ENST00000357232.4:exon25:c.G7219C:p.A2407P,	4q31.3	.
4	155507032	C	T	AAGAAGGCAG,TTCATCAGGG	exonic	FGA	nonsynonymous SNV	FGA:ENST00000403106.3:exon5:c.G1549A:p.A517T,FGA:ENST00000302053.3:exon5:c.G1549A:p.A517T,	4q31.3	.
4	155665767	C	T	CAACAAGCGT,TCATCCTGGG	exonic	LRAT	nonsynonymous SNV	LRAT:ENST00000507827.1:exon2:c.C289T:p.L97F,LRAT:ENST00000336356.3:exon2:c.C289T:p.L97F,	4q32.1	.
4	158057815	T	A	TCCTCCTCTT,ATTTTTCGTG	exonic	GLRB	nonsynonymous SNV	GLRB:ENST00000509282.1:exon5:c.T492A:p.F164L,GLRB:ENST00000541722.1:exon5:c.T492A:p.F164L,GLRB:ENST00000264428.4:exon5:c.T492A:p.F164L,	4q32.1	.
4	159894434	T	C	TGATGAATTT,GGGTAAATAA	exonic	C4orf45	nonsynonymous SNV	C4orf45:ENST00000434826.2:exon2:c.A94G:p.K32E,	4q32.1	.
4	183664521	A	T	GATTTCAACT,TGTGCGGCGG	exonic	TENM3	nonsynonymous SNV	TENM3:ENST00000406950.2:exon18:c.A3578T:p.Y1193F,TENM3:ENST00000511685.1:exon19:c.A3578T:p.Y1193F,	4q35.1	.
4	183713613	G	A	GGACTACAAC,AGGAAGGGCT	exonic	TENM3	nonsynonymous SNV	TENM3:ENST00000406950.2:exon25:c.G5788A:p.E1930K,TENM3:ENST00000511685.1:exon26:c.G5788A:p.E1930K,	4q35.1	.
4	185045416	A	G	CAGAATCTCA,CCTCACAGCC	exonic	ENPP6	nonsynonymous SNV	ENPP6:ENST00000296741.2:exon3:c.T431C:p.V144A,ENPP6:ENST00000512353.1:exon4:c.T167C:p.V56A,	4q35.1	.
4	186244797	A	T	GTAGGAAATA,AGGTATTGAA	exonic	SNX25	nonsynonymous SNV	SNX25:ENST00000264694.8:exon9:c.A1100T:p.K367I,SNX25:ENST00000504273.1:exon9:c.A1100T:p.K367I,	4q35.1	.
4	186435893	C	T	CCGCAGCTGT,CGTGAAGCGC	exonic	PDLIM3	nonsynonymous SNV	PDLIM3:ENST00000284767.5:exon4:c.G482A:p.G161E,PDLIM3:ENST00000284771.6:exon4:c.G482A:p.G161E,	4q35.1	.
4	186544922	G	A	ATACTCAATG,AAAACCGCCT	exonic	SORBS2	nonsynonymous SNV	SORBS2:ENST00000284776.7:exon13:c.C1649T:p.S550F,SORBS2:ENST00000431808.1:exon14:c.C1649T:p.S550F,SORBS2:ENST00000418609.1:exon7:c.C1361T:p.S454F,SORBS2:ENST00000355634.5:exon16:c.C1949T:p.S650F,	4q35.1	.
4	186560171	G	A	ACAGGTGTCG,AGTTGTTGGG	exonic	SORBS2	nonsynonymous SNV	SORBS2:ENST00000319471.9:exon12:c.C961T:p.P321S,SORBS2:ENST00000437304.2:exon12:c.C1240T:p.P414S,SORBS2:ENST00000319454.6:exon11:c.C841T:p.P281S,	4q35.1	.
4	187003943	C	T	AATGATATTC,AGGCATAAAA	exonic	TLR3	nonsynonymous SNV	TLR3:ENST00000513189.1:exon5:c.C911T:p.P304L,TLR3:ENST00000504367.1:exon2:c.C272T:p.P91L,TLR3:ENST00000296795.3:exon4:c.C1103T:p.P368L,	4q35.1	.
4	188924040	C	T	TGGGGCTAAG,CCAGGCAAGG	exonic	ZFP42	nonsynonymous SNV	ZFP42:ENST00000509524.1:exon3:c.C79T:p.P27S,ZFP42:ENST00000326866.4:exon4:c.C79T:p.P27S,	4q35.2	.
4	189019424	G	A	CGAATTTCAA,ATCAAGGCAC	exonic	TRIML2	nonsynonymous SNV	TRIML2:ENST00000326754.3:exon5:c.C481T:p.L161F,	4q35.2	.
5	240484	C	T	AGATAAAGTC,CTCCAATTAA	exonic	SDHA	nonsynonymous SNV	SDHA:ENST00000264932.6:exon11:c.C1444T:p.P482S,SDHA:ENST00000510361.1:exon10:c.C1300T:p.P434S,SDHA:ENST00000504309.1:exon11:c.C1444T:p.P482S,	5p15.33	.
5	10564820	G	A	GGGCGTCCCG,AAAGCGTCCC	exonic	ANKRD33B	nonsynonymous SNV	ANKRD33B:ENST00000296657.5:exon1:c.G241A:p.E81K,ANKRD33B:ENST00000504806.1:exon1:c.G241A:p.E81K,	5p15.2	.
5	11565081	C	T	CTGCCAGTCT,GGATCCGAGC	exonic	CTNND2	nonsynonymous SNV	CTNND2:ENST00000359640.2:exon3:c.G262A:p.E88K,CTNND2:ENST00000304623.8:exon3:c.G262A:p.E88K,CTNND2:ENST00000508761.1:exon3:c.G220A:p.E74K,CTNND2:ENST00000504499.1:exon3:c.G262A:p.E88K,CTNND2:ENST00000502551.1:exon3:c.G220A:p.E74K,CTNND2:ENST00000513588.1:exon3:	5p15.2	.
5	13781008	C	T	CTCTGCTTTC,TGATCCGCCC	exonic	DNAH5	nonsynonymous SNV	DNAH5:ENST00000265104.4:exon53:c.G8881A:p.G2961R,	5p15.2	.
5	24509859	G	A	TAATAAAAAC,GGGATCTACA	exonic	CDH10	nonsynonymous SNV	CDH10:ENST00000264463.4:exon7:c.C1072T:p.R358C,CDH10:ENST00000510477.1:exon7:c.C1072T:p.R358C,	5p14.2	CDH10;basechange=c.1072C>A;AAchange=p.R358S;tissue=lung
5	32069747	C	T	CAATTCCTCT,CTGGCTTAGG	exonic	PDZD2	nonsynonymous SNV	PDZD2:ENST00000438447.1:exon15:c.C2524T:p.P842S,PDZD2:ENST00000282493.3:exon14:c.C2524T:p.P842S,	5p13.3	PDZD2;basechange=c.2524C>T;AAchange=p.P842S;tissue=skin
5	32174058	G	A	GCCGCCCGCC,CCCGCTCCTT	exonic	GOLPH3	nonsynonymous SNV	GOLPH3:ENST00000503610.1:exon1:c.C83T:p.A28V,GOLPH3:ENST00000265070.6:exon1:c.C83T:p.A28V,	5p13.3	.
5	33881579	A	G	GCCCTTGATA,AATGCTCTGA	exonic	ADAMTS12	nonsynonymous SNV	ADAMTS12:ENST00000515401.1:exon2:c.T134C:p.F45S,ADAMTS12:ENST00000352040.3:exon2:c.T134C:p.F45S,ADAMTS12:ENST00000504830.1:exon2:c.T134C:p.F45S,	5p13.2	.
5	34043179	G	A	AGGCAAAAAG,GAGGAAAAAC	exonic	C1QTNF3	nonsynonymous SNV	C1QTNF3:ENST00000231338.7:exon1:c.C52T:p.P18S,C1QTNF3:ENST00000382065.3:exon1:c.C52T:p.P18S,	5p13.2	.
5	35039475	C	T	GAAAAGAAAT,CAGGTATCTG	exonic	AGXT2	nonsynonymous SNV	AGXT2:ENST00000510428.1:exon3:c.G316A:p.D106N,AGXT2:ENST00000231420.6:exon3:c.G316A:p.D106N,	5p13.2	AGXT2;basechange=c.316G>T;AAchange=p.D106Y;tissue=breast
5	35860954	G	A	TCCTCCCCAG,AGACTTGGAA	exonic	IL7R	nonsynonymous SNV	IL7R:ENST00000508941.1:exon4:c.G83A:p.G28E,IL7R:ENST00000506850.1:exon2:c.G83A:p.G28E,IL7R:ENST00000303115.3:exon2:c.G83A:p.G28E,IL7R:ENST00000514217.1:exon2:c.G83A:p.G28E,IL7R:ENST00000515665.1:exon3:c.G83A:p.G28E,IL7R:ENST00000343305.4:exon2:c.G83A:p.G28E,IL7R:ENST00(	5p13.2	.
5	38350713	G	T	TCACCACTTT,TCCCAAGGTA	exonic	EGFLAM	nonsynonymous SNV	EGFLAM:ENST00000322350.5:exon4:c.G402T:p.L134F,EGFLAM:ENST00000354891.3:exon4:c.G402T:p.L134F,	5p13.2	.
5	38493731	C	T	AGTTTCAGTG,TGTTTGAGGG	exonic	LIFR	nonsynonymous SNV	LIFR:ENST00000263409.4:exon14:c.G2042A:p.S681N,LIFR:ENST00000453190.2:exon14:c.G2042A:p.S681N,	5p13.1	.
5	41009390	C	T	ACTGAAATTG,CTCAACCCTG	exonic	MROH2B	nonsynonymous SNV	MROH2B:ENST00000506092.2:exon22:c.G2077A:p.A693T,MROH2B:ENST00000399564.4:exon32:c.G3412A:p.A1138T,	5p13.1	.
5	41032884	G	A	CACCGAATAG,GCTAGCTAAG	exonic	MROH2B	nonsynonymous SNV	MROH2B:ENST00000506092.2:exon14:c.C1066T:p.P356S,MROH2B:ENST00000399564.4:exon24:c.C2401T:p.P801S,	5p13.1	HEATR7B2;basechange=c.2401C>A;AAchange=p.P801T;tissue=lung
5	55088523	G	A	TCGCATGTTG,ATATGGGTTT	exonic	DDX4	nonsynonymous SNV	DDX4:ENST00000354991.5:exon15:c.G1255A:p.D419N,DDX4:ENST00000353507.5:exon16:c.G1255A:p.D419N,DDX4:ENST00000514278.2:exon16:c.G1297A:p.D433N,DDX4:ENST00000511853.1:exon10:c.G910A:p.D304N,DDX4:ENST00000506511.1:exon16:c.G1297A:p.D433N,DDX4:ENST00000505374.1:exon1	5q11.2	.
5	55809699	C	T	TGCTCCTGGC,CTGTGCTCCC	exonic	AC022431.2	nonsynonymous SNV	AC022431.2:ENST00000438651.1:exon5:c.G259A:p.G87S,	5q11.2	.
5	58120934	G	A	AGTGTGACAT,GAAGACGAGC	exonic	RAB3C	nonsynonymous SNV	RAB3C:ENST00000282878.4:exon4:c.G441A:p.M147I,	5q11.2	.
5	78596667	C	T	GCCAAGAAAT,CTACCTCAGA	exonic	JMY	nonsynonymous SNV	JMY:ENST00000396137.4:exon6:c.C1862T:p.S621F,	5q14.1	.
5	82837401	C	T	TCTGTAATGT,CCCACAGGAT	exonic	VCAN	nonsynonymous SNV	VCAN:ENST00000265077.3:exon8:c.C8579T:p.S2860F,VCAN:ENST00000343200.5:exon7:c.C5618T:p.S1873F,	5q14.3	.
5	88025079	C	T	TCCTCCCATT,CTTGTCCTGG	exonic	MEF2C	nonsynonymous SNV	MEF2C:ENST00000539796.1:exon6:c.G752A:p.G251E,MEF2C:ENST00000506554.1:exon9:c.G920A:p.G307E,MEF2C:ENST00000514015.1:exon9:c.G920A:p.G307E,MEF2C:ENST00000340208.5:exon10:c.G950A:p.G317E,MEF2C:ENST00000510942.1:exon8:c.G896A:p.G299E,MEF2C:ENST00000508569.1:exon8:	5q14.3	.
5	101595925	C	A	ACCTTTAGAG,AGCTGGAAAA	exonic	SLCO4C1	nonsynonymous SNV	SLCO4C1:ENST00000310954.6:exon6:c.G1120T:p.A374S,	5q21.1	.
5	110409230	C	T	TACTGAAATC,AGAGCCTAAC	exonic	TSLP	stopgain	TSLP:ENST00000420978.2:exon4:c.C238T:p.Q80X,TSLP:ENST00000344895.3:exon3:c.C238T:p.Q80X,	5q22.1	TSLP;basechange=c.238C>T;AAchange=p.Q80*;tissue=lung
5	115298802	C	T	GGACCCCTTT,CCCGGGCACT	exonic	AQPEP	nonsynonymous SNV	AQPEP:ENST00000357872.4:exon1:c.C488T:p.S163F,AQPEP:ENST00000504467.1:exon1:c.C488T:p.S163F,	5q23.1	.
5	122131034	C	T	GGCCCAAAAC,CACAGAAGTT	exonic	SNX2	nonsynonymous SNV	SNX2:ENST00000379516.2:exon2:c.C182T:p.P61L,SNX2:ENST00000505934.1:exon2:c.C182T:p.P61L,SNX2:ENST00000512394.1:exon2:c.C182T:p.P61L,	5q23.2	.
5	125919688	C	T	TATTTCTCCT,GTTTTGGAGC	exonic	ALDH7A1	nonsynonymous SNV	ALDH7A1:ENST00000553117.1:exon4:c.G329A:p.R110Q,ALDH7A1:ENST00000447989.2:exon4:c.G410A:p.R137Q,ALDH7A1:ENST00000509270.1:exon2:c.G209A:p.R70Q,ALDH7A1:ENST00000409134.3:exon4:c.G329A:p.R110Q,	5q23.2	.
5	127653846	C	A	CTTACCAACA,AACCCACACC	exonic	FBN2	nonsynonymous SNV	FBN2:ENST00000262464.4:exon36:c.G4712T:p.C1571F,FBN2:ENST00000508053.1:exon42:c.G4712T:p.C1571F,	5q23.3	.
5	134232073	G	A	CATACAGATC,AACACTGGAA	exonic	TXNDC15	nonsynonymous SNV	TXNDC15:ENST00000546290.1:exon4:c.G776A:p.R259Q,TXNDC15:ENST00000358387.4:exon4:c.G845A:p.R282Q,	5q31.1	TXNDC15;basechange=c.845G>A;AAchange=p.R282Q;tissue=endometrium
5	138240097	G	T	TTGTTCGAAT,TCTGCAAGCC	exonic	CTNNA1	nonsynonymous SNV	CTNNA1:ENST00000519116.1:exon5:c.G246T:p.M82I,CTNNA1:ENST00000517533.1:exon4:c.G246T:p.M82I,CTNNA1:ENST00000518825.1:exon9:c.G1356T:p.M452I,CTNNA1:ENST00000521368.1:exon1:c.G10T:p.V4F,CTNNA1:ENST00000355078.5:exon9:c.G1047T:p.M349I,CTNNA1:ENST00000302763.7:exon10	5q31.2	.
5	140056971	C	A	GCCCTTCTCT,CCACCATCTC	exonic	HARS	nonsynonymous SNV	HARS:ENST00000504366.1:exon7:c.G557T:p.G186V,HARS:ENST00000448240.1:exon7:c.G179T:p.G60V,HARS:ENST00000504156.1:exon8:c.G764T:p.G255V,HARS:ENST00000457527.2:exon8:c.G704T:p.G235V,HARS:ENST00000431330.2:exon5:c.G422T:p.G141V,HARS:ENST00000438307.2:exon7:c.G644T:p.	5q31.3	.
5	140166233	G	A	TTTCATGTG,AGGTGAAGGT	exonic	PCDHA1	nonsynonymous SNV	PCDHA1:ENST00000394633.3:exon1:c.G358A:p.E120K,PCDHA1:ENST00000504120.2:exon1:c.G358A:p.E120K,PCDHA1:ENST00000378133.3:exon1:c.G358A:p.E120K,	5q31.3	.
5	140175016	G	A	CCTCTAGAGG,AGCATCTGAT	exonic	PCDHA2	nonsynonymous SNV	PCDHA2:ENST00000526136.1:exon1:c.G467A:p.G156E,PCDHA2:ENST00000520672.2:exon1:c.G467A:p.G156E,PCDHA2:ENST00000378132.1:exon1:c.G467A:p.G156E,	5q31.3	PCDHA2;basechange=c.467G>C;AAchange=p.G156A;tissue=prostate&PCDHA2_ENST00000526136;basechange=c.467G>C;AAchange=p.G
5	140183138	G	A	CCGGGATAGA,AGGAGAAACA	exonic	PCDHA3	nonsynonymous SNV	PCDHA3:ENST00000522353.2:exon1:c.G2356A:p.E786K,PCDHA3:ENST00000532566.2:exon1:c.G2356A:p.E786K,	5q31.3	.
5	140251020	G	A	TCTTCCTCTA,GTCTGAATAA	exonic	PCDHA11	nonsynonymous SNV	PCDHA11:ENST00000398640.2:exon1:c.G2332A:p.G778S,	5q31.3	.
5	140262133	G	A	CGACCGCGAG,AGCTGTGTGG	exonic	PCDHA13	nonsynonymous SNV	PCDHA13:ENST00000289272.2:exon1:c.G280A:p.E94K,PCDHA13:ENST00000409494.1:exon1:c.G280A:p.E94K,	5q31.3	.
5	140264033	C	T	TCGGCACCGC,CACCGAGGGC	exonic	PCDHA13	nonsynonymous SNV	PCDHA13:ENST00000289272.2:exon1:c.C2180T:p.P727L,PCDHA13:ENST00000409494.1:exon1:c.C2180T:p.P727L,	5q31.3	.
5	140347315	G	A	AGTAATTGGG,GGCTGGATTA	exonic	PCDHAC2	nonsynonymous SNV	PCDHAC2:ENST00000289269.5:exon1:c.G964A:p.G322R,	5q31.3	.
5	140503582	C	T	CTTCTCCCAG,CCTACCTGCC	exonic	PCDHB4	nonsynonymous SNV	PCDHB4:ENST00000194152.1:exon1:c.C2002T:p.P668S,	5q31.3	.
5	140718632	C	T	CGGGCAGATT,GCTATTCTGT	exonic	PCDHGA2	nonsynonymous SNV	PCDHGA2:ENST00000394576.2:exon1:c.C94T:p.R32C,	5q31.3	.
5	149546833	C	T	GAGGCCGACG,CCTACGAGTG	exonic	CDX1	nonsynonymous SNV	CDX1:ENST00000231656.8:exon1:c.C394T:p.P132S,	5q32	.
5	149546834	C	A	AGGCCGACGC,CTACGAGTGG	exonic	CDX1	nonsynonymous SNV	CDX1:ENST00000231656.8:exon1:c.C395A:p.P132H,	5q32	.
5	149618311	C	T	TCTGAGGATT,CTGCCAAAGA	exonic	CAMK2A	nonsynonymous SNV	CAMK2A:ENST00000398376.3:exon15:c.G1018A:p.E340K,CAMK2A:ENST00000348628.6:exon14:c.G985A:p.E329K,	5q32	.
5	150071401	G	A	CCGCATGAAA,GAGGGGGTGG	exonic	RBM22	nonsynonymous SNV	RBM22:ENST00000540000.1:exon10:c.C1028T:p.P343L,RBM22:ENST00000199814.4:exon11:c.C1175T:p.P392L,RBM22:ENST00000447771.2:exon10:c.C1028T:p.P343L,	5q33.1	.
5	150071402	G	A	CGCATGAAAG,AGGGGGTGGT	exonic	RBM22	nonsynonymous SNV	RBM22:ENST00000540000.1:exon10:c.C1027T:p.P343S,RBM22:ENST00000199814.4:exon11:c.C1174T:p.P392S,RBM22:ENST00000447771.2:exon10:c.C1027T:p.P343S,	5q33.1	.
5	150923493	G	A	TGGACTTTAA,AACCAGGTGT	exonic	FAT2	nonsynonymous SNV	FAT2:ENST00000261800.5:exon9:c.C7195T:p.L2399F,	5q33.1	.
5	150946583	G	A	AATCTTCAGG,AATAACTGGT	exonic	FAT2	nonsynonymous SNV	FAT2:ENST00000261800.5:exon1:c.C1910T:p.S637F,	5q33.1	.
5	151045943	A	T	GTGCTGGTCC,GCTGGCCGAA	exonic	SPARC	nonsynonymous SNV	SPARC:ENST00000538026.1:exon5:c.T440A:p.L147Q,SPARC:ENST00000231061.4:exon8:c.T713A:p.L238Q,	5q33.1	.
5	168112705	T	C	CAGGGAGATG,TGGCCTGGGG	exonic	SLIT3	nonsynonymous SNV	SLIT3:ENST00000332966.8:exon31:c.A3563G:p.N1188S,SLIT3:ENST00000404867.3:exon31:c.A3542G:p.N1181S,SLIT3:ENST00000519560.1:exon31:c.A3542G:p.N1181S,	5q34	.
5	169174500	C	T	AACTACCATC,TTTTGCAGGT	exonic	DOCK2	nonsynonymous SNV	DOCK2:ENST00000524185.1:exon23:c.C2368T:p.L790F,DOCK2:ENST00000256935.8:exon23:c.C2368T:p.L790F,DOCK2:ENST00000520908.1:exon9:c.C844T:p.L282F,	5q35.1	DOCK2;basechange=c.2368C>A;AAchange=p.L790I;tissue=endometrium
5	170736398	C	T	GCGCAGACCC,GCACCCGCAC	exonic	TLX3	nonsynonymous SNV	TLX3:ENST00000296921.5:exon1:c.C29T:p.P10L,	5q35.1	.
5	176072489	G	A	GAGGGAGGCG,AAAACCAGGC	exonic	EIF4E1B	nonsynonymous SNV	EIF4E1B:ENST00000504597.1:exon8:c.G586A:p.E196K,EIF4E1B:ENST00000318682.6:exon8:c.G586A:p.E196K,EIF4E1B:ENST00000505497.1:exon5:c.G370A:p.E124K,	5q35.2	.
5	178293308	C	T	AAAGCTGGCT,CTTCTCAGAG	exonic	ZNF354B	nonsynonymous SNV	ZNF354B:ENST00000520377.1:exon2:c.C97T:p.P33S,ZNF354B:ENST00000322434.3:exon3:c.C97T:p.P33S,	5q35.3	.
5	178293309	C	T	AAGCTGGCTC,TTCTCAGAGA	exonic	ZNF354B	nonsynonymous SNV	ZNF354B:ENST00000520377.1:exon2:c.C98T:p.P33L,ZNF354B:ENST00000322434.3:exon3:c.C98T:p.P33L,	5q35.3	.
5	178392670	G	A	AAAGCCTTTC,GGACCAATCA	exonic	ZNF454	nonsynonymous SNV	ZNF454:ENST00000320129.3:exon5:c.G1265A:p.R422Q,ZNF454:ENST00000519564.1:exon5:c.G1265A:p.R422Q,	5q35.3	.
5	178559862	C	T	CACTTGTCTT,CTGCTTGCTG	exonic	ADAMTS2	nonsynonymous SNV	ADAMTS2:ENST00000251582.7:exon14:c.G2125A:p.E709K,	5q35.3	.
5	180219241	T	C	CTCCTTGCCG,TGTCATTCCA	exonic	MGAT1	nonsynonymous SNV	MGAT1:ENST00000307826.4:exon2:c.A731G:p.N244S,MGAT1:ENST00000333055.3:exon3:c.A731G:p.N244S,MGAT1:ENST00000446023.2:exon3:c.A731G:p.N244S,MGAT1:ENST00000427865.2:exon2:c.A731G:p.N244S,MGAT1:ENST00000393340.3:exon4:c.A731G:p.N244S,	5q35.3	.
5	180474954	C	T	CCTGAGTATC,CATCCTGGCC	exonic	BTNL9	nonsynonymous SNV	BTNL9:ENST00000491209.1:exon3:c.C137T:p.P46L,BTNL9:ENST00000376841.2:exon3:c.C137T:p.P46L,BTNL9:ENST00000327705.9:exon3:c.C137T:p.P46L,BTNL9:ENST00000376842.3:exon3:c.C137T:p.P46L,	5q35.3	.
6	7584913	C	T	GACCCAGAAA,CAATAAAGAA	exonic	DSP	nonsynonymous SNV	DSP:ENST00000379802.3:exon24:c.C7418T:p.T2473I,DSP:ENST00000418664.2:exon24:c.C5621T:p.T1874I,	6p24.3	.
6	10956378	C	T	GAAGGAATTT,CACTTCATCC	exonic	SYCP2L	nonsynonymous SNV	SYCP2L:ENST00000283141.6:exon25:c.C2066T:p.S689F,	6p24.2	.
6	13642780	C	T	AAGGCCTCTG,TGTGGCACAG	exonic	RANBP9	nonsynonymous SNV	RANBP9:ENST00000539980.1:exon6:c.G469A:p.A157T,RANBP9:ENST00000011619.3:exon7:c.G1156A:p.A386T,	6p23	.
6	17130889	C	T	GCCAATCACT,AGATTCAGCT	exonic	STMND1	nonsynonymous SNV	STMND1:ENST00000536551.1:exon5:c.C608T:p.S203L,STMND1:ENST00000354384.5:exon5:c.C584T:p.S195L,	6p22.3	.
6	17805745	C	A	AAAGGTCTCT,ATGTCAATTA	exonic	KIF13A	nonsynonymous SNV	KIF13A:ENST00000378843.2:exon19:c.G2265T:p.M755I,KIF13A:ENST00000378814.5:exon19:c.G2265T:p.M755I,KIF13A:ENST00000378826.2:exon19:c.G2265T:p.M755I,KIF13A:ENST00000259711.6:exon19:c.G2265T:p.M755I,KIF13A:ENST00000378816.5:exon19:c.G2265T:p.M755I,	6p22.3	KIF13A_ENST00000259711;basechange=c.2265G>A;AAchange=p.M755I;tissue=endometrium&KIF13A;basechange=c.2265G>A;AAchange=
6	24134741	G	A	CAGTATTCTG,AAGGTAAGGA	exonic	NRSN1	stopgain	NRSN1:ENST00000378491.4:exon3:c.G186A:p.W62X,NRSN1:ENST00000378478.1:exon3:c.G186A:p.W62X,NRSN1:ENST00000378477.2:exon3:c.G186A:p.W62X,NRSN1:ENST00000378475.1:exon1:c.G186A:p.W62X,	6p22.3	.
6	25917285	C	A	CACTGTGAAC,ATAGGAGACA	exonic	SLC17A2	nonsynonymous SNV	SLC17A2:ENST00000360488.3:exon7:c.G680T:p.W227L,SLC17A2:ENST00000377850.3:exon7:c.G680T:p.W227L,SLC17A2:ENST00000265425.3:exon6:c.G680T:p.W227L,	6p22.2	.
6	28253448	C	T	GTGTGAACCT,CACAGCGTCC	exonic	PGBD1	nonsynonymous SNV	PGBD1:ENST00000259883.3:exon3:c.C517T:p.P173S,PGBD1:ENST00000405948.2:exon3:c.C517T:p.P173S,	6p22.1	.
6	29069530	C	T	TTCTCAAGAT,AAGGCAAGTT	exonic	OR2J1	stopgain	OR2J1:ENST00000377171.3:exon1:c.C811T:p.Q271X,	6p22.1	.
6	33273126	C	T	CCCAGTCTCA,TCGAGGGGCA	exonic	TAPBP	nonsynonymous SNV	TAPBP:ENST00000475304.1:exon5:c.G562A:p.V188M,TAPBP:ENST00000434618.2:exon4:c.G508A:p.V170M,TAPBP:ENST00000467025.1:exon3:c.G337A:p.V113M,TAPBP:ENST00000489157.1:exon3:c.G247A:p.V83M,TAPBP:ENST00000426633.2:exon4:c.G508A:p.V170M,TAPBP:ENST00000456592.2:exon4:c.G	6p21.32	.
6	33679352	C	T	TCCCGAAAGG,CTGTGCTACC	exonic	UQCC2	nonsynonymous SNV	UQCC2:ENST00000374214.3:exon1:c.G112A:p.A38T,UQCC2:ENST00000607484.1:exon1:c.G112A:p.A38T,	6p21.31	.
6	34123016	C	T	CCGGCAAAAT,CAAAGGACCT	exonic	GRM4	nonsynonymous SNV	GRM4:ENST00000374177.3:exon1:c.G152A:p.G51E,	6p21.31	.
6	35050570	G	C	ACTCATCTTC,AGTCCTGTGG	exonic	ANKS1A	nonsynonymous SNV	ANKS1A:ENST00000360359.3:exon18:c.G2812C:p.E938Q,	6p21.31	.
6	35088265	G	C	CCCTGACACA,CACCATTGTG	exonic	TCP11	nonsynonymous SNV	TCP11:ENST00000412155.2:exon6:c.C760G:p.L254V,TCP11:ENST00000311875.5:exon7:c.C913G:p.L305V,TCP11:ENST00000444780.2:exon7:c.C898G:p.L300V,TCP11:ENST00000486638.1:exon4:c.C451G:p.L151V,TCP11:ENST00000418521.2:exon6:c.C685G:p.L229V,TCP11:ENST00000373979.2:exon7:c.C688	6p21.31	.
6	35479436	T	C	GTCCTCCGCG,CTGGGGCACG	exonic	TULP1	nonsynonymous SNV	TULP1:ENST00000229771.6:exon4:c.A338G:p.D113G,	6p21.31	.
6	35716607	C	T	CGGCCCTCCT,CTGCCAGCCC	exonic	ARMC12	nonsynonymous SNV	ARMC12:ENST00000373869.3:exon6:c.C953T:p.S318F,ARMC12:ENST00000373866.3:exon6:c.C983T:p.S328F,ARMC12:ENST00000288065.2:exon6:c.C1064T:p.S355F,	6p21.31	.
6	35744465	C	T	GAGCGCGCTT,CGCAATATAA	exonic	CLPSL2	nonsynonymous SNV	CLPSL2:ENST00000360454.2:exon1:c.C70T:p.P24S,CLPSL2:ENST00000403376.3:exon1:c.C70T:p.P24S,	6p21.31	.
6	35744466	C	T	AGCGCGCTTC,GCAATATAAA	exonic	CLPSL2	nonsynonymous SNV	CLPSL2:ENST00000360454.2:exon1:c.C71T:p.P24L,CLPSL2:ENST00000403376.3:exon1:c.C71T:p.P24L,	6p21.31	.
6	36274119	C	T	GCCTCTGGTT,ATGTGAAGGA	exonic	PNPLA1	nonsynonymous SNV	PNPLA1:ENST00000388715.3:exon7:c.C1150T:p.H384Y,PNPLA1:ENST00000457797.1:exon7:c.C1438T:p.H480Y,PNPLA1:ENST00000394571.2:exon7:c.C1435T:p.H479Y,PNPLA1:ENST00000312917.5:exon7:c.C1177T:p.H393Y,	6p21.31	.
6	36291271	G	A	TGTGGGGCCG,GTTTTCTACA	exonic	C6orf222	nonsynonymous SNV	C6orf222:ENST00000437635.2:exon8:c.C1270T:p.P424S,	6p21.31	.
6	36926933	G	A	ATGGGACGAG,AGCTGGCCGC	exonic	PI16	nonsynonymous SNV	PI16:ENST00000373674.3:exon2:c.G184A:p.E62K,	6p21.2	.
6	36930908	G	A	GGAAACCCAG,CCCCAACTTC	exonic	PI16	nonsynonymous SNV	PI16:ENST00000373674.3:exon5:c.G790A:p.A264T,	6p21.2	.
6	38840510	G	A	TGACTGGACA,ATGGGATTTT	exonic	DNAH8	nonsynonymous SNV	DNAH8:ENST00000327475.6:exon49:c.G7153A:p.D2385N,DNAH8:ENST00000449981.2:exon50:c.G7189A:p.D2397N,DNAH8:ENST00000359357.3:exon48:c.G6538A:p.D2180N,DNAH8:ENST00000441566.1:exon45:c.G6430A:p.D2144N,	6p21.2	.
6	41121586	C	T	GCATCCTCTT,CTGCAGGGTA	exonic	TREML1	nonsynonymous SNV	TREML1:ENST00000373127.4:exon2:c.G286A:p.E96K,TREML1:ENST00000426005.2:exon2:c.G286A:p.E96K,	6p21.1	.
6	42200588	G	A	GTGCCCCCGT,GATGCGGGCA	exonic	TRERF1	nonsynonymous SNV	TRERF1:ENST00000541110.1:exon17:c.C3169T:p.H1057Y,TRERF1:ENST00000340840.2:exon15:c.C2896T:p.H966Y,TRERF1:ENST00000372922.4:exon17:c.C3109T:p.H1037Y,TRERF1:ENST00000354325.2:exon15:c.C2860T:p.H954Y,TRERF1:ENST00000372917.4:exon16:c.C2896T:p.H966Y,	6p21.1	.
6	42713337	G	A	ACTGCCGGGG,GATGCCAGGA	exonic	TBCC	nonsynonymous SNV	TBCC:ENST00000372876.1:exon1:c.C475T:p.P159S,TBCC:ENST00000244625.2:exon2:c.C475T:p.P159S,	6p21.1	.
6	43156300	C	T	CCTCGCAGCT,CCTGGATCAG	exonic	CUL9	nonsynonymous SNV	CUL9:ENST00000372647.2:exon8:c.C2027T:p.S676F,CUL9:ENST00000252050.4:exon8:c.C2027T:p.S676F,CUL9:ENST00000354495.3:exon6:c.C1697T:p.S566F,	6p21.1	.
[illegible]	[illegible]	[illegible]	[illegible]	[illegible]	[illegible]	[illegible]	[illegible]	[illegible]	[illegible]	[illegible]

6	45922908	C	T	GAACACGACT,CTTTCAGCCA	exonic	CLIC5	nonsynonymous SNV	[illegible]	6p21.1	.
6	46801056	G	A	CAATTCGGAG,GATATGGTTT	exonic	MEP1A	nonsynonymous SNV	[illegible]	6p12.3	.
6	47763282	G	A	CCATGTGCTG,AAATGAAACT	exonic	OPN5	nonsynonymous SNV	[illegible]	6p12.3	.
6	49704156	T	G	CAGTTCATTG,GCTTATTCAC	exonic	CRISP3	nonsynonymous SNV	[illegible]	6p12.3	.
6	49705055	G	A	TCATTTGCTG,AAAAGATGGA	exonic	CRISP3	nonsynonymous SNV	[illegible]	6p12.3	CRISP3;basechange=c.55C>T[illegible]
6	53135425	G	A	AGCAATCAGG,AAATCATGTA	exonic	ELOVL5	nonsynonymous SNV	[illegible]	6p12.1	.
6	53135477	G	A	AATGTGCACG,CCAGATGACC	exonic	ELOVL5	nonsynonymous SNV	[illegible]	6p12.1	.
6	56458795	G	A	TTCCTGCAGA,AGTGAGTTAA	exonic	DST	nonsynonymous SNV	[illegible]	6p12.1	.
6	56483652	G	A	CTCCTCTATG,TCTTTAAGTG	exonic	DST	nonsynonymous SNV	[illegible]	6p12.1	.
6	62611233	G	C	ATTTAAGTAA,ATAATTCACG	exonic	KHDRBS2	nonsynonymous SNV	[illegible]	6q11.1	.
6	63989958	C	T	TTATTTCTCT,TGCAGCTATT	exonic	LGSN	nonsynonymous SNV	[illegible]	6q12	.
6	64776351	T	G	AAGAAATAAA,GAAGAAACTG	exonic	EYS	nonsynonymous SNV	[illegible]	6q12	.
6	70965069	G	A	GCTTCTCCTG,AGGTCCTCGC	exonic	COL9A1	nonsynonymous SNV	[illegible]	6q13	.
6	70983763	G	A	AAATCCACGC,ATCCAGGCAC	exonic	COL9A1	nonsynonymous SNV	[illegible]	6q13	.
6	75884790	C	T	AGGGCGTCTC,AGCCCCAGAC	exonic	COL12A1	nonsynonymous SNV	[illegible]	6q13	.
6	84265883	C	T	TACAAGAAAT,CTTGATGTTA	exonic	SNAP91	nonsynonymous SNV	[illegible]	6q14.2	.
6	87969183	C	T	GAAATTTGCT,CCTTTAAATG	exonic	ZNF292	nonsynonymous SNV	[illegible]	6q14.3	.
6	88757753	G	A	CCTGGCCCAC,AAGGCGAGGG	exonic	SPACA1	nonsynonymous SNV	[illegible]	6q15	SPACA1;basechange=c.130G[illegible]
6	88854839	G	T	TCCCCTAAAG,AAGTTAAAGG	exonic	CNR1	nonsynonymous SNV	[illegible]	6q15	.
6	90422355	C	T	ATCTGTCCAT,TCTTCGGACT	exonic	MDN1	nonsynonymous SNV	[illegible]	6q15	.
6	90605698	C	T	TTTCTTTTTC,TTTCTTTCTT	exonic	GJA10	nonsynonymous SNV	[illegible]	6q15	.
6	99885152	C	T	GTGTTATGTT,CGATAATTTC	exonic	USP45	nonsynonymous SNV	[illegible]	6q16.2	.
6	102483342	G	A	TTTCCTAATG,AGTCAACAAC	exonic	GRIK2	nonsynonymous SNV	[illegible]	6q16.3	.
6	109283261	G	A	TGGAATCCTG,AGGCTGTGCG	exonic	ARMC2	nonsynonymous SNV	[illegible]	6q21	.
6	111912611	G	A	GGTCTGGGGA,GTCCTGGGGG	exonic	TRAF3IP2	nonsynonymous SNV	[illegible]	6q21	.
6	116953593	G	A	TGAGGAAGAT,ATTATGACTA	exonic	RSPH4A	nonsynonymous SNV	[illegible]	6q22.1	.
6	116953595	T	A	AGGAAGATGA,TATGACTAAT	exonic	RSPH4A	nonsynonymous SNV	[illegible]	6q22.1	.
6	117923381	G	A	CCCGCCAGGG,CCCCCACGGA	exonic	GOPC	nonsynonymous SNV	[illegible]	6q22.1	.
6	130370912	C	T	CCCGACCTCT,CCCCGAGCTC	exonic	L3MBTL3	nonsynonymous SNV	[illegible]	6q23.1	L3MBTL3;basechange=c.226C[illegible]
6	130370913	C	T	CCGACCTCTC,CCCGAGCTCC	exonic	L3MBTL3	nonsynonymous SNV	[illegible]	6q23.1	.
6	132004287	C	T	CAATATCATC,TTCTGGCTGA	exonic	ENPP3	nonsynonymous SNV	[illegible]	6q23.2	.
6	132891909	G	A	TCTGTGTCAG,AATTTGCATC	exonic	TAAR6	nonsynonymous SNV	[illegible]	6q23.2	.
6	133044905	C	A	AGCTGACTCC,ACTTAGAATG	exonic	VNN3	nonsynonymous SNV	[illegible]	6q23.2	.
6	133783513	A	C	AGCTCAAACA,TGTCTGCCTA	exonic	EYA4	nonsynonymous SNV	[illegible]	6q23.2	.
6	137219369	G	A	AGTCTTCAGA,CCCCACTCAG	exonic	PEX7	nonsynonymous SNV	[illegible]	6q23.3	.
6	144811289	C	T	CTGACCTCCC,AGAGAGTAGG	exonic	UTRN	nonsynonymous SNV	[illegible]	6q24.2	.
6	145956569	G	A	CAGTTTGATG,TTACATGTTC	exonic	EPM2A	nonsynonymous SNV	[illegible]	6q24.3	.
6	146870678	G	A	GATATATCAA,AAGTTCCACA	exonic	RAB32	nonsynonymous SNV	[illegible]	6q24.3	.
6	147109655	G	C	AAGGCTTGAG,GATGTGGCAA	exonic	ADGB	nonsynonymous SNV	[illegible]	6q24.3	.
6	149699485	C	T	ATGTCCAGTT,CTCTGGTGCT	exonic	TAB2	nonsynonymous SNV	[illegible]	6q25.1	.
6	149772453	C	T	CGCCCGGGCT,CTGCGGAGCC	exonic	ZC3H12D	nonsynonymous SNV	[illegible]	6q25.1	.
6	150005203	G	A	TCTCCCTGAT,GAAAGTTAAA	exonic	LATS1	nonsynonymous SNV	[illegible]	6q25.1	.
6	150158570	A	C	TCACAGAAGA,GTGGTAGCGT	exonic	LRP11	nonsynonymous SNV	[illegible]	6q25.1	.
6	150719350	G	A	CAAACCTCTG,ACCAGATCAT	exonic	IYD	nonsynonymous SNV	[illegible]	6q25.1	.
6	152684910	C	T	GAGCCTCACC,TGAGGGGCAG	splicing	SYNE1(ENST	.	.	6q25.2	.
6	152711433	G	A	GTGGACGGAG,AGCTCATGAT	exonic	SYNE1	nonsynonymous SNV	[illegible]	6q25.2	.
6	152770691	C	T	TTAACCTCTC,GTGGTTGGCA	exonic	SYNE1	nonsynonymous SNV	[illegible]	6q25.2	.
6	155743898	G	T	AGCAGCGAAG,GAGTGACTCC	exonic	NOX3	nonsynonymous SNV	[illegible]	6q25.3	.
6	160479104	C	T	AATCTTAATC,GCTAAACAGT	exonic	IGF2R	nonsynonymous SNV	[illegible]	6q25.3	.
6	165715288	C	T	CGGAGTTCTT,CCTCCTGCGC	exonic	C6orf118	nonsynonymous SNV	[illegible]	6q27	.
7	4874790	G	A	CGGGGGCTGA,AGGCTGATGC	exonic	RADIL	nonsynonymous SNV	[illegible]	7p22.1	.
7	6661616	G	A	CCTGGGGTCA,AGCAGGAGCT	exonic	ZNF853	nonsynonymous SNV	[illegible]	7p22.1	.
7	12394006	C	T	CCATAAAATC,TTCTGGACAT	exonic	VWDE	nonsynonymous SNV	[illegible]	7p21.3	.
7	14216508	G	A	ATTTGCATTG,CAGAGACTTG	exonic	DGKB	nonsynonymous SNV	[illegible]	7p21.2	.
7	15725804	T	G	GTGATGGTGG,GGTGGTGGTG	exonic	MEOX2	nonsynonymous SNV	[illegible]	7p21.2	.
7	17370479	G	A	TGTCGTCTAA,GTGTCTGCTG	exonic	AHR	nonsynonymous SNV	[illegible]	7p21.1	.
7	20768023	C	T	GGCAGGGTTT,GATTTGGAGC	exonic	ABCB5	stopgain	[illegible]	7p21.1	.
7	21751462	G	A	GAACCCACAA,ATCTGGGCTG	exonic	DNAH11	nonsynonymous SNV	[illegible]	7p15.3	.
7	21775345	G	A	AATGCTAGTA,GAAATGCAGG	exonic	DNAH11	nonsynonymous SNV	[illegible]	7p15.3	.
7	21778441	C	T	TGGCACCGTT,AGCCTCACAC	exonic	DNAH11	stopgain	[illegible]	7p15.3	.
7	25181865	C	T	CTTACCGGCT,TGGGTCAAAT	exonic	C7orf31	nonsynonymous SNV	[illegible]	7p15.3	.
7	25194647	G	T	CTTCAAAACT,TTGAATCATA	exonic	C7orf31	nonsynonymous SNV	[illegible]	7p15.3	.
7	30728826	C	T	TGGTGAGGGT,ATGATGGTGT	exonic	CRHR2	nonsynonymous SNV	[illegible]	7p14.3	.
7	31682464	G	A	GAATGCCTTG,AACAAAGGGC	exonic	CCDC129	nonsynonymous SNV	[illegible]	7p14.3	.
7	37890037	G	A	AGGCTTAACA,GTATAAGGAC	exonic	NME8	nonsynonymous SNV	[illegible]	7p14.1	.
7	37989928	C	T	AAAGACCCCT,GGTGTTTACC	exonic	EPDR1	nonsynonymous SNV	[illegible]	7p14.1	.
7	38857460	A	T	GGATCTCACG,AATGTGGGTG	exonic	VPS41	nonsynonymous SNV	[illegible]	7p14.1	.
7	44266243	G	A	GATCCTGGGG,CTGGGGCGGA	exonic	CAMK2B	nonsynonymous SNV	[illegible]	7p13	.
7	44363505	C	T	AGCCAGCCAC,CACTCAGGGA	exonic	CAMK2B	stopgain	[illegible]	7p13	.
7	44363506	C	T	GCCAGCCACC,ACTCAGGGAT	exonic	CAMK2B	stopgain	[illegible]	7p13	.
7	45010634	C	T	CCACCCTCCT,CGTCTCCACA	exonic	MYO1G	nonsynonymous SNV	[illegible]	7p13	.
7	45015126	C	T	GATCGGGTCC,CCTTGAAGTC	exonic	MYO1G	nonsynonymous SNV	[illegible]	7p13	.
7	45103557	C	T	TACCTGAATC,CTCCAGTAGG	exonic	CCM2	nonsynonymous SNV	[illegible]	7p13	.
7	50663139	A	T	CTTACAGGTA,GATCTGGAAA	exonic	GRB10	nonsynonymous SNV	[illegible]	7p12.1	.
7	50686932	C	T	CTCTCACTGG,CATGGTACTC	exonic	GRB10	nonsynonymous SNV	[illegible]	7p12.1	.
7	50742323	C	T	GCATTCATAT,GTTCACCAGG	exonic	GRB10	nonsynonymous SNV	[illegible]	7p12.1	.
7	55991305	C	T	AATAGGGTAT,AGCTTTGTAA	exonic	MRPS17,ZNF	stopgain	[illegible]	7p11.2	ZNF713;basechange=c.181C>G;AAchange=p.Q61E;tissue=urinary_[illegible]
7	70255798	C	T	CGCATCAGCC,CACCGCGGGC	exonic	AUTS2	nonsynonymous SNV	[illegible]	7q11.22	.
7	72891393	C	T	GCTTCCATTT,TTTCCGTTTC	exonic	BAZ1B	nonsynonymous SNV	[illegible]	7q11.23	BAZ1B;basechange=c.2398G>T;AAchange=p.E800*;tissue=large_in[illegible]
7	72891531	G	T	AGGATCCGGT,GCACAGTGCT	exonic	BAZ1B	nonsynonymous SNV	[illegible]	7q11.23	.
7	73008618	G	A	CCGGAGAGCG,GCAGAGAGCA	exonic	MLXIPL	nonsynonymous SNV	[illegible]	7q11.23	.
7	73111843	G	A	CACCAGACTC,GAGGTAAGCT	exonic	WBSCR22	nonsynonymous SNV	[illegible]	7q11.23	.
7	73477960	G	A	GGCCTAGTGG,AGCCGCTGGG	exonic	ELN	nonsynonymous SNV	[illegible]	7q11.23	.
7	73932588	T	A	GCCAGCCGAG,ATGACCCCAA	exonic	GTF2IRD1	nonsynonymous SNV	[illegible]	7q11.23	.
7	74015388	C	T	CAGCAGCATT,CCAAGCGCAA	exonic	GTF2IRD1	nonsynonymous SNV	[illegible]	7q11.23	.
7	80123948	C	T	AGTACTTTTC,CAGATTCTCC	exonic	GNAT3	nonsynonymous SNV	[illegible]	7q21.11	.
7	82545181	G	A	CGTGGAGTAT,GTGATCAATA	exonic	PCLO	nonsynonymous SNV	[illegible]	7q21.11	.
7	89909129	C	T	ATCAGTGGCT,CTTTATTAAT	exonic	C7orf63	nonsynonymous SNV	[illegible]	7q21.13	.
7	90225967	C	T	GAGCCGCAGC,GGCCGAGAAG	exonic	CDK14	nonsynonymous SNV	[illegible]	7q21.13	.
7	92098389	C	T	GCCCCAGGGT,CAGTGTTTCG	exonic	ERVW-1	nonsynonymous SNV	[illegible]	7q21.2	.
7	92760840	T	A	GTTTAGACCC,TCCTTTTGCC	exonic	SAMD9L	nonsynonymous SNV	[illegible]	7q21.2	.
7	92825165	A	G	AAACATCTGG,AAAGCAACAA	exonic	HEPACAM2	nonsynonymous SNV	[illegible]	7q21.3	.
7	92848492	C	T	ATGTAATTGC,TTCATCAGGG	exonic	HEPACAM2	nonsynonymous SNV	[illegible]	7q21.3	.
7	94050324	C	T	CATTTAGGGT,CAAATGGTCC	exonic	COL1A2	nonsynonymous SNV	[illegible]	7q21.3	COL1A2;basechange=c.2299C>A;AAchange=p.P767T;tissue=kidne[illegible]
7	96765716	T	G	TCCAGAAATC,TGATGCATGT	exonic	ACN9	nonsynonymous SNV	[illegible]	7q21.3	.
7	99474457	G	A	ATCCATGAGG,AGAGCTGGCT	exonic	OR2AE1	nonsynonymous SNV	[illegible]	7q22.1	.
7	100349698	C	T	CCCACCGTCC,CACAGAAGAG	exonic	ZAN	nonsynonymous SNV	[illegible]	7q22.1	.
7	100686920	C	T	CAGTACACCT,CAACAACCTC	exonic	MUC17	nonsynonymous SNV	[illegible]	7q22.1	.
7	100686921	C	T	AGTACACCTC,AACAACCTCT	exonic	MUC17	nonsynonymous SNV	[illegible]	7q22.1	.
7	100773756	G	A	ATGGGGCCAT,GAACAAGGAT	exonic	SERPINE1	stopgain	[illegible]	7q22.1	.
7	100883005	G	A	GATAGAGACG,GGGGCAGGGG	exonic	FIS1	nonsynonymous SNV	[illegible]	7q22.1	.
7	102760435	G	A	CGCATCTATT,GAGGGAGTTC	exonic	NAPEPLD	nonsynonymous SNV	[illegible]	7q22.1	.
7	104783626	G	A	GTCATTGGAA,GTGCAGCTGA	exonic	SRPK2	nonsynonymous SNV	[illegible]	7q22.3	.
7	107615816	C	A	ATAGCTGTCA,AAATTCCCTC	exonic	LAMB1	nonsynonymous SNV	[illegible]	7q31.1	.
7	107871510	C	T	TGGTGGTAAT,CAATTGGGGG	exonic	NRCAM	nonsynonymous SNV	[illegible]	7q31.1	.
7	110303696	G	A	CGCTCTGGAG,AAGAACAGAT	exonic	IMMP2L	nonsynonymous SNV	[illegible]	7q31.1	.
7	113520098	G	T	TGCTTTATTT,GAAAATTGAC	exonic	PPP1R3A	nonsynonymous SNV	[illegible]	7q31.1	.
7	122261614	T	G	AAATGCAGAG,TCTGTGAACG	exonic	CADPS2	nonsynonymous SNV	[illegible]	7q31.32	.
7	122808616	C	G	TTAACACTTT,TGCAAAACAT	splicing	SLC13A1(EN	.	.	7q31.32	.
7	123257740	G	A	ATTAGAAAAG,GAGTGTGGCC	exonic	ASB15	nonsynonymous SNV	[illegible]	7q31.32	.
7	127251701	G	A	GGGCAGGCAG,GCTGCTGTGG	exonic	PAX4	nonsynonymous SNV	[illegible]	7q32.1	.
7	128315862	C	T	CCCCAGGGTC,ATTTACCACC	exonic	FAM71F2	nonsynonymous SNV	[illegible]	7q32.1	.
7	130025062	C	T	GAGGTCAAGT,CATTGTAGAC	exonic	CPA1	nonsynonymous SNV	[illegible]	7q32.2	.
7	133933810	G	A	CCTCCATTTG,ACCATATCCT	exonic	LRGUK	nonsynonymous SNV	[illegible]	7q33	.
7	134871064	C	T	TCATCGCCAC,CAGCTGCAAG	exonic	WDR91	nonsynonymous SNV	[illegible]	7q33	.
7	139706975	G	A	GTACCCGCCA,CTTTCAGGTG	exonic	TBXAS1	nonsynonymous SNV	[illegible]	7q34	.
7	140156454	T	C	AGTCTCACTT,ATGATCTTGC	exonic	MKRN1	nonsynonymous SNV	[illegible]	7q34	.
7	140301266	G	A	GGAAGGTGGG,GAGAGGAGGG	exonic	DENND2A	nonsynonymous SNV	[illegible]	7q34	.
7	140301998	C	T	TCCGTCTGCT,TCCTGCTGGG	exonic	DENND2A	nonsynonymous SNV	[illegible]	7q34	.
7	142111834	A	G	ACAAAGCAGC,CCCAGCAGAG	exonic	TRBV5-7	nonsynonymous SNV	[illegible]	7q34	.
7	142353747	C	T	GGATTGTACC,CCGAAAAAGG	exonic	TRBV23-1	nonsynonymous SNV	[illegible]	7q34	.
7	143141189	T	C	TGCATGATTT,GCTCATCACA	exonic	TAS2R60	nonsynonymous SNV	[illegible]	7q35	.
7	143573208	C	T	GAACAGAACC,TTTCATCCTC	exonic	FAM115A	nonsynonymous SNV	[illegible]	7q35	.
7	150644491	G	A	GCTGGAGAGG,GGATGTTGAG	exonic	KCNH2	nonsynonymous SNV	[illegible]	7q36.1	.
7	150654506	G	A	GGTGATTTGG,GAATCTTGCT	exonic	KCNH2	nonsynonymous SNV	[illegible]	7q36.1	.
7	150654507	G	A	GTGATTTGGG,AATCTTGCTA	exonic	KCNH2	nonsynonymous SNV	[illegible]	7q36.1	.
7	150733710	C	T	TCATGTCCTT,CTGGTGGCCT	exonic	ABCB8	nonsynonymous SNV	[illegible]	7q36.1	.
7	151133396	G	A	ATTTCTAGGC,GAAATGTTCT	exonic	CRYGN	nonsynonymous SNV	[illegible]	7q36.1	.
7	152550640	G	A	GTGTGGTTCG,AGGCTCCATG	exonic	ACTR3B	nonsynonymous SNV	[illegible]	7q36.1	.
7	154767582	G	A	ATGTTACCTG,GACGGGTGGG	exonic	PAXIP1	nonsynonymous SNV	[illegible]	7q36.2	.
7	157414113	G	A	GGCCACGAAC,AGCTGTTGGG	exonic	PTPRN2	nonsynonymous SNV	[illegible]	7q36.3	.
8	12956035	T	G	GATACAGAGT,GAGGCTTGGC	exonic	DLC1	nonsynonymous SNV	[illegible]	8p22	.
8	16885164	G	A	GGCTGCCAAG,AGACGGTGAG	exonic	MICU3	nonsynonymous SNV	[illegible]	8p22	.
8	18413790	G	A	TTGTCGGGGG,ATATGAGCGG	exonic	PSD3	nonsynonymous SNV	[illegible]	8p22	.
8	22138962	T	A	TCTCCCACTT,TTGGGATCCA	exonic	PIWIL2	nonsynonymous SNV	[illegible]	8p21.3	.
8	22275191	G	A	CTCAACGCTG,GATGAGCATC	exonic	SLC39A14	nonsynonymous SNV	[illegible]	8p21.3	.
8	22862898	C	T	CTGGAACGCT,CCGAGACGTG	exonic	RHOBTB2	nonsynonymous SNV	[illegible]	8p21.3	.
8	23117833	G	A	CTTATCAGAG,GAGGTATGGA	exonic	CHMP7	nonsynonymous SNV	[illegible]	8p21.3	.
8	23117834	G	A	TTATCAGAGG,AGGTATGGAG	exonic	CHMP7	nonsynonymous SNV	[illegible]	8p21.3	.
8	25898467	G	A	TTGCTGTAGA,GAGCTGTAAC	exonic	EBF2	nonsynonymous SNV	[illegible]	8p21.2	.
8	27292040	G	A	AAACCTGGAG,CCCGGCGGTC	exonic	PTK2B	nonsynonymous SNV	[illegible]	8p21.2	.
8	28929346	G	A	GGCCCCCGGG,AGCAGCCGGG	exonic	KIF13B	nonsynonymous SNV	[illegible]	8p12	.
8	30695338	C	T	GTTCACATTT,TTTTCTTCAT	exonic	TEX15	nonsynonymous SNV	[illegible]	8p12	TEX15;basechange=c.7313G>C;AAchange=p.R2438T;tissue=breas[illegible]
8	36663896	T	C	AGCAATTGGC,AGGTAAGTGT	exonic	KCNU1	nonsynonymous SNV	[illegible]	8p11.23	.
8	38176446	G	A	GTAGCCTGAG,AACTGTTTCA	exonic	WHSC1L1	nonsynonymous SNV	[illegible]	8p11.23	.
8	39646247	C	T	GTATCAGACC,CATATGATTA	exonic	ADAM2	nonsynonymous SNV	[illegible]	8p11.22	.
8	41655046	G	A	GAAGCCCACA,AATAGGGCAT	exonic	ANK1	nonsynonymous SNV	[illegible]	8p11.21	.
8	42256236	T	C	CCAGGAATTT,CTACTTCTGG	exonic	VDAC3	nonsynonymous SNV	[illegible]	8p11.21	.
8	42552726	G	A	CATCGTCCTT,GCATCCCTTC	exonic	CHRNB3	nonsynonymous SNV	[illegible]	8p11.21	.
8	42552727	G	A	ATCGTCCTTG,CATCCCTTCC	exonic	CHRNB3	nonsynonymous SNV	[illegible]	8p11.21	.
8	48752643	G	A	AGAAGGATGG,AAACGAATTC	exonic	PRKDC	nonsynonymous SNV	[illegible]	8q11.21	.
8	52567297	C	T	TGGAATTTCT,TTATTCTGTT	exonic	PXDNL	nonsynonymous SNV	[illegible]	8q11.22	.
8	54792080	G	A	CGACCCTCGG,GGGTCGTCCG	exonic	RGS20	nonsynonymous SNV	[illegible]	8q11.23	.
8	55540283	T	G	TGACACTTTT,TTCCTAGTGA	exonic	RP1	nonsynonymous SNV	[illegible]	8q12.1	.
8	56436473	G	A	AGCACCCTAC,GTCCATCTCC	exonic	XKR4	nonsynonymous SNV	[illegible]	8q12.1	.
8	61763638	G	A	AAACCAACCA,AACACCGTTC	exonic	CHD7	nonsynonymous SNV	[illegible]	8q12.2	.
8	68942782	G	T	CAGCAGTGAT,GAAGCCCTCC	exonic	PREX2	nonsynonymous SNV	[illegible]	8q13.2	.
8	73848248	G	A	GGAGCTGCAG,AAACGGACGA	exonic	KCNB2	nonsynonymous SNV	[illegible]	8q13.3	.
8	97299316	C	A	CAGGTTAAAT,TCTAATGTAT	exonic	PTDSS1	nonsynonymous SNV	[illegible]	8q22.1	.
8	102555613	T	G	TGAGCATTAA,GGTGATGAGG	exonic	GRHL2	nonsynonymous SNV	[illegible]	8q22.3	.
8	104390369	G	A	CACATTCAAT,GAGCTGAATG	exonic	CTHRC1	nonsynonymous SNV	[illegible]	8q22.3	.
8	105106947	G	A	ATTCTCACAA,ATGATACCAG	exonic	RIMS2	nonsynonymous SNV	[illegible]	8q22.3	.
8	105479076	C	A	TCCACCAGCA,GTCGGCCACC	exonic	DPYS	nonsynonymous SNV	[illegible]	8q22.3	.
8	107782248	C	G	GAGGTGAGTC,TGGGTCCCTC	exonic	ABRA	nonsynonymous SNV	[illegible]	8q23.1	.
8	110492861	C	A	GGTCTTGAAC,TGTACCAGCT	exonic	PKHD1L1	nonsynonymous SNV	[illegible]	8q23.1	.
8	110520058	C	T	CTACAGAATT,CTAAGGCGAT	exonic	PKHD1L1	nonsynonymous SNV	[illegible]	8q23.2	.
8	113237052	C	T	TACCGCCTTC,CTTCCACTGA	exonic	CSMD3	nonsynonymous SNV	[illegible]	8q23.3	.
8	113246706	C	T	TTTATATACC,CTGTAAAATG	exonic	CSMD3	nonsynonymous SNV	[illegible]	8q23.3	CSMD3;basechange=c.10628G>T;AAchange=p.G3543V;tissue=lun[illegible]
8	113259274	C	T	GAATCCCACT,CACGTAAGAT	exonic	CSMD3	stopgain	[illegible]	8q23.3	.
8	113259275	C	T	AATCCCACTC,ACGTAAGATC	exonic	CSMD3	stopgain	[illegible]	8q23.3	.
8	114449043	G	C	CCAGGGTTTG,ATTCCTTTGC	exonic	CSMD3	nonsynonymous SNV	[illegible]	8q23.3	.
8	143960520	G	A	CATCCTGTGG,GATGCAGGCT	exonic	CYP11B1	nonsynonymous SNV	[illegible]	8q24.3	.
8	145165117	G	A	CCCGGGCCCG,TTGTCGCCAT	exonic	KIAA1875	nonsynonymous SNV	[illegible]	8q24.3	.
9	8331661	C	A	TCAATAAATC,TTCTCCGGAC	exonic	PTPRD	stopgain	[illegible]	9p24.1	PTPRD;basechange=c.5455G>A;AAchange=p.G1819R;tissue=large[illegible]
9	21141380	C	T	TTTACCATCT,CTGGGGGAAC	exonic	IFNW1	nonsynonymous SNV	[illegible]	9p21.3	.
9	27209118	G	A	TTTCTTCCCA,AATATGCCTC	splicing	TEK(ENST00	.	.	9p21.2	.
9	39109157	G	A	CACTACTTAC,ATCTCCGCGG	exonic	CNTNAP3	stopgain	[illegible]	9p13.1	.
9	73151978	A	T	ACCGAATAGA,AGAATGGCTT	exonic	TRPM3	nonsynonymous SNV	[illegible]	9q21.12	.
9	73213472	G	A	TCTTGGAGAC,AAGGATCATT	exonic	TRPM3	nonsynonymous SNV	[illegible]	9q21.12	TRPM3;basechange=c.2491C>T;AAchange=p.R831C;tissue=large_[illegible]
9	78774059	G	A	CTCGCCCTCT,GAACTAGGTC	exonic	PCSK5	nonsynonymous SNV	[illegible]	9q21.13	.
9	90582487	G	A	TGGGCCTTGG,GGCAGGTCCC	exonic	CDK20	nonsynonymous SNV	[illegible]	9q22.1	.
9	96051723	G	A	GCTCTACCAG,AGCACGTGCC	exonic	WNK2	nonsynonymous SNV	[illegible]	9q22.31	.
9	96870209	C	T	AAAAGATGGC,CTAAGCCTGG	exonic	PTPDC1	nonsynonymous SNV	[illegible]	9q22.32	.
9	100963843	C	T	CACGAGGAAC,AGTTGAAGGT	exonic	TBC1D2	stopgain	[illegible]	9q22.33	.
9	104239080	G	A	AGCCAGGGCC,GGGGGTGGCC	exonic	TMEM246	nonsynonymous SNV	[illegible]	9q31.1	.
9	105767096	A	T	AGCCTCTCAA,CCAACTCGTA	exonic	CYLC2	nonsynonymous SNV	[illegible]	9q31.1	CYLC2;basechange=c.300A>C;AAchange=p.K100N;tissue=endome[illegible]
9	112686110	C	T	ACAGAAAAAT,CTTCAAGGAC	exonic	AKAP2,PALM	nonsynonymous SNV	[illegible]	9q31.3	.
9	113703958	C	A	GGGTATAGCA,CCATAACGAT	exonic	LPAR1	nonsynonymous SNV	[illegible]	9q31.3	.
9	114138770	C	T	TTGATTGTTT,CATCATTGGA	exonic	KIAA0368	nonsynonymous SNV	[illegible]	9q31.3	.
9	114138771	C	T	TGATTGTTTC,ATCATTGGAG	exonic	KIAA0368	nonsynonymous SNV	[illegible]	9q31.3	.
9	115038228	G	A	TTTCGAAGAT,GAGAACACGG	exonic	PTBP3	nonsynonymous SNV	[illegible]	9q32	.
9	116823285	G	A	GCCCCCGTAG,GGAAGAGGAC	exonic	AMBP	nonsynonymous SNV	[illegible]	9q32	.
9	117086082	C	T	GAGTACCAGA,CCGGTGAGAG	exonic	ORM1	nonsynonymous SNV	[illegible]	9q32	ORM1;basechange=c.254C>T;AAchange=p.T85I;tissue=endometriu[illegible]
9	118997501	C	T	CACGGTTCCT,CAGCCTGCCC	exonic	PAPPA	nonsynonymous SNV	[illegible]	9q33.1	.
9	121929510	C	T	CTGGGGTTTC,CCGGGGCCAC	exonic	BRINP1	nonsynonymous SNV	[illegible]	9q33.1	.
9	130223518	C	T	GACCCAGCTC,AGACTCTCAA	exonic	LRSAM1	stopgain	[illegible]	9q33.3	.
9	130504101	A	T	GACCGCCGCG,AGCTGAACAT	exonic	SH2D3C	nonsynonymous SNV	[illegible]	9q34.11	.
9	131804628	C	T	GCGGAAAGTC,TCTTTGCCAC	exonic	FAM73B	nonsynonymous SNV	[illegible]	9q34.11	.
9	133370364	C	T	GGTGTACATC,TCGGCCGGGA	exonic	ASS1	nonsynonymous SNV	[illegible]	9q34.11	.
9	135073471	C	T	CAGAGCATCA,CTGGAGCCGC	exonic	NTNG2	nonsynonymous SNV	[illegible]	9q34.13	.
9	139311650	C	T	GCCCAGTACA,TGGGGGGATT	exonic	PMPCA	nonsynonymous SNV	[illegible]	9q34.3	PMPCA;basechange=c.881C>T;AAchange=p.T294I;tissue=lung
9	139847350	G	A	GTAGTTCCAG,GGGAATGGTT	exonic	LCN12	nonsynonymous SNV	[illegible]	9q34.3	.
9	140327508	C	A	AGTTGGCAAA,AGGCTCCTCT	exonic	NOXA1	nonsynonymous SNV	[illegible]	9q34.3	.
10	95141	C	T	GATCTGATTC,CGCACTGCCC	exonic	TUBB8	nonsynonymous SNV	[illegible]	10p15.3	.
10	3185605	G	A	GTCCGTGTAG,GGACAGTTCG	exonic	PITRM1	nonsynonymous SNV	[illegible]	10p15.2	.
10	7747089	C	T	CTATGAAGAT,TTGTGGAACT	exonic	ITIH2	nonsynonymous SNV	[illegible]	10p14	.
10	11997416	T	A	GTTCTGAAAA,AACAGCTGAT	exonic	UPF2	nonsynonymous SNV	[illegible]	10p14	.
10	15649744	G	A	GGGAAGACGC,ATGAGCCTGA	exonic	ITGA8	nonsynonymous SNV	[illegible]	10p13	ITGA8;basechange=c.1696C>T;AAchange=p.R566C;tissue=endome[illegible]
10	25839989	G	A	TACTGTTTAC,GAACTGTCAC	exonic	GPR158	nonsynonymous SNV	[illegible]	10p12.1	.
10	29169217	C	T	TGGCTCATTC,CTGGGAGTGC	exonic	C10orf126	nonsynonymous SNV	[illegible]	10p12.1	.
10	45941007	G	A	GCCTGTGAAG,AAGCCATGGC	exonic	ALOX5	nonsynonymous SNV	[illegible]	10q11.21	.
10	45959179	C	T	GCAAAACTTT,TGAAGTATCC	exonic	MARCH8	nonsynonymous SNV	[illegible]	10q11.21	.
10	46135859	G	A	GTCATCCTGA,GAGATTCTCA	exonic	ZFAND4	nonsynonymous SNV	[illegible]	10q11.22	.
10	55569161	A	T	AACTTTCTTA,AAATCATGGG	exonic	PCDH15	nonsynonymous SNV	[illegible]	10q21.1	.
10	60553377	T	C	CCAAGCAAGG,TGGTTCAGAG	splicing	BICC1(ENST	.	.	10q21.1	.
10	69648774	C	T	TATTAATATC,TTTCAGAACC	exonic	SIRT1	nonsynonymous SNV	[illegible]	10q21.3	.
10	70525797	G	T	GTGTCCTATT,GACTACAGAT	exonic	CCAR1	nonsynonymous SNV	[illegible]	10q21.3	.
10	72195499	G	A	CAAGGAAAAG,TGACCTGGGA	exonic	AC022532.1,	nonsynonymous SNV	[illegible]	10q22.1	.
10	72326472	C	T	GCCCTCTGCC,CCGAGGACTT	exonic	PALD1	nonsynonymous SNV	[illegible]	10q22.1	.
10	74660221	C	T	TGTGCTAGGC,CTGACAGGCA	exonic	OIT3	nonsynonymous SNV	[illegible]	10q22.1	.
10	75412972	G	A	CCCACCATAG,GATGAGATGA	exonic	SYNPO2L	nonsynonymous SNV	[illegible]	10q22.2	.
10	82266999	C	T	GCTGTCCGAC,TCACCAAAGT	exonic	TSPAN14	nonsynonymous SNV	[illegible]	10q23.1	.
10	89487205	C	T	ACGCTGTTCC,GTGTTTGGGG	exonic	PAPSS2	nonsynonymous SNV	[illegible]	10q23.2	.
10	91404780	G	A	GGCGCCTGGG,ATGGCGAACC	exonic	PANK1	nonsynonymous SNV	[illegible]	10q23.31	.
10	94372874	G	C	GTTAAAATCG,AAAGTTGAAC	exonic	KIF11	nonsynonymous SNV	[illegible]	10q23.33	.
10	95445060	C	T	AAATTAACTT,CCAACTCTTT	exonic	FRA10AC1	nonsynonymous SNV	[illegible]	10q23.33	.
10	96731905	A	C	AAAGCTTGGA,AACACTGCAG	exonic	CYP2C9	nonsynonymous SNV	[illegible]	10q23.33	.
10	96802808	C	T	CTGCCAATTA,ATGATCAATC	exonic	CYP2C8	nonsynonymous SNV	[illegible]	10q23.33	.
10	98157015	C	T	AGCCGGCTGT,CGTGGAGACG	exonic	TLL2	nonsynonymous SNV	[illegible]	10q24.1	.
10	101496042	C	T	TGTGTTGATT,AGTGGAATCA	exonic	CUTC	nonsynonymous SNV	[illegible]	10q24.2	CUTC;basechange=c.101C>T;AAchange=p.S34L;tissue=lung
10	105038001	G	A	GGAGCTGGAG,AGCGGCACAG	exonic	INA	nonsynonymous SNV	[illegible]	10q24.33	.
10	106937892	G	A	TTTGTTGACG,GGCTCTGGTG	exonic	SORCS3	nonsynonymous SNV	[illegible]	10q25.1	.
10	106982903	C	T	TCTCCGAGTT,CATTTGTTGC	exonic	SORCS3	nonsynonymous SNV	[illegible]	10q25.1	.
10	107022096	C	T	TAGGAAAATC,CTTGGATTAA	exonic	SORCS3	nonsynonymous SNV	[illegible]	10q25.1	.
10	112541203	C	T	GCTGCCTTTT,CAAAGATTTT	exonic	RBM20	nonsynonymous SNV	[illegible]	10q25.2	.
10	118138859	G	A	AGATCTTCAG,AAAGCAAATA	exonic	CCDC172	nonsynonymous SNV	[illegible]	10q25.3	.
10	118313330	G	A	AGAACCAATG,GACCATTGGA	exonic	PNLIP	nonsynonymous SNV	[illegible]	10q25.3	.
10	123239496	G	A	GGGTAACTAG,TGAATACTGT	exonic	FGFR2	nonsynonymous SNV	[illegible]	10q26.13	.
10	123848148	C	T	ATGATGCCTT,CTTAGATACA	exonic	TACC2	nonsynonymous SNV	[illegible]	10q26.13	.
10	123970587	C	T	GTTGTCCCCC,GGCTTCTGGA	exonic	TACC2	nonsynonymous SNV	[illegible]	10q26.13	.
10	124248996	G	A	TATTGTGTCG,AAGATGGACT	exonic	HTRA1	nonsynonymous SNV	[illegible]	10q26.13	.
10	124335915	G	T	CTTCCTCTAG,ATCTGATTCT	exonic	DMBT1	nonsynonymous SNV	[illegible]	10q26.13	.
10	124358569	C	T	GGCTGGCTCT,CCACAACTGT	exonic	DMBT1	nonsynonymous SNV	[illegible]	10q26.13	.
10	124671186	G	A	CGAAGATCAT,ATCGGCATCG	exonic	FAM24A	nonsynonymous SNV	[illegible]	10q26.13	.
10	124895798	G	A	GGGGGCCCTG,AGGGCGCCGC	exonic	HMX3	nonsynonymous SNV	[illegible]	10q26.13	.
10	125804302	G	A	GTCGAAGGTG,AGCGGAAGCG	exonic	CHST15	nonsynonymous SNV	[illegible]	10q26.13	.
10	127737919	C	T	AATCCGGCCT,CTTGCTGCAG	exonic	ADAM12	nonsynonymous SNV	[illegible]	10q26.2	.
10	129535754	C	T	GGCTCCCGGC,CGCTCCTCGG	exonic	FOXI2	nonsynonymous SNV	[illegible]	10q26.2	.
10	129913393	G	A	ACATCTGTAG,AATACTTCCT	exonic	MKI67	nonsynonymous SNV	[illegible]	10q26.2	.

10	134886431	C	A	TTTATTTGTT,CCGACTTTCC	exonic	GPR123	nonsynonymous SNV	GPR123:ENST00000607359.1:exon3:c.C465A:p.F155L,	10q26.3	.
10	134942518	G	A	GACGGCGGAC,AGGCGCACGT	exonic	GPR123	nonsynonymous SNV	GPR123:ENST00000392607.3:exon7:c.G1186A:p.E396K,GPR123:ENST00000392606.2:exon4:c.G895A:p.E299K,GPR123:ENST00000607359.1:exon16:c.G3343A:p.E1115K,	10q26.3	.
11	209911	C	T	ACCCACGCTC,TTCCTTCCCA	exonic	RIC8A	nonsynonymous SNV	RIC8A:ENST00000526104.1:exon3:c.C637T:p.L213F,RIC8A:ENST00000527696.1:exon1:c.C619T:p.L207F,RIC8A:ENST00000325207.5:exon3:c.C637T:p.L213F,	11p15.5	.
11	379981	G	A	GCGAGGATAT,GACGTGGAGC	exonic	B4GALNT4	nonsynonymous SNV	B4GALNT4:ENST00000329962.6:exon16:c.G2604A:p.M868I,	11p15.5	.
11	379982	G	A	CGAGGATATG,ACGTGGAGCG	exonic	B4GALNT4	nonsynonymous SNV	B4GALNT4:ENST00000526584.1:exon1:c.G1A:p.D1N,B4GALNT4:ENST00000329962.6:exon16:c.G2605A:p.D869N,	11p15.5	.
11	607909	C	T	CCCCTCTTCT,CATCAAGAAG	exonic	PHRF1	nonsynonymous SNV	PHRF1:ENST00000534320.1:exon14:c.C2453T:p.S818F,PHRF1:ENST00000413872.2:exon14:c.C2447T:p.S816F,PHRF1:ENST00000533464.1:exon14:c.C2441T:p.S814F,PHRF1:ENST00000416188.2:exon14:c.C2450T:p.S817F,PHRF1:ENST00000264555.5:exon14:c.C2453T:p.S818F,	11p15.5	.
11	1270931	G	A	TCCACCCCGG,AACAGCTCCC	exonic	MUC5B	nonsynonymous SNV	MUC5B:ENST00000529681.1:exon31:c.G12821A:p.G4274E,MUC5B:ENST00000447027.1:exon31:c.G12830A:p.G4277E,	11p15.5	.
11	1606220	C	T	GCCACAGCCC,CCTTGCAGCC	exonic	KRTAP5-1	nonsynonymous SNV	KRTAP5-1:ENST00000382171.2:exon1:c.G260A:p.G87E,	11p15.5	.
11	1651422	G	A	GTCCAAGGGG,GCTGTGGCTC	exonic	KRTAP5-5	nonsynonymous SNV	KRTAP5-5:ENST00000399676.2:exon1:c.G352A:p.G118S,	11p15.5	KRTAP5-5;basechange=c.352G>A;AAchange=p.G118S;tissue=lung
11	1769271	G	A	CCGTCCAGGG,GACTCGGGCT	exonic	IFITM10,RP1	nonsynonymous SNV	IFITM10:ENST00000382123.1:exon3:c.C275T:p.P92L,IFITM10:ENST00000340134.4:exon2:c.C163T:p.P55S,	11p15.5	.
11	1769272	G	A	CGTCCAGGGG,ACTCGGGCTT	exonic	IFITM10,RP1	nonsynonymous SNV	IFITM10:ENST00000382123.1:exon3:c.C274T:p.P92S,	11p15.5	.
11	3249932	C	T	GAGGCCGAGC,CCTCGGTGAG	exonic	MRGPRE	nonsynonymous SNV	MRGPRE:ENST00000389832.5:exon2:c.G98A:p.G33E,MRGPRE:ENST00000436689.2:exon2:c.G95A:p.G32E,	11p15.4	.
11	3819140	C	T	AGACTCCGCC,CCTTCCTTGG	exonic	PGAP2	nonsynonymous SNV	PGAP2:ENST00000396986.2:exon1:c.C67T:p.P23S,PGAP2:ENST00000300730.6:exon1:c.C67T:p.P23S,PGAP2:ENST00000532535.1:exon1:c.C13T:p.P5S,	11p15.4	.
11	3819141	C	T	GACTCCGCCC,CTTCCTTGGA	exonic	PGAP2	nonsynonymous SNV	PGAP2:ENST00000396986.2:exon1:c.C68T:p.P23L,PGAP2:ENST00000300730.6:exon1:c.C68T:p.P23L,PGAP2:ENST00000532535.1:exon1:c.C14T:p.P5L,	11p15.4	.
11	4409594	G	A	CTCCTGTAGG,CCTGGCTCTG	exonic	TRIM21	nonsynonymous SNV	TRIM21:ENST00000254436.7:exon4:c.C671T:p.A224V,TRIM21:ENST00000543625.1:exon4:c.C671T:p.A224V,	11p15.4	.
11	4881028	G	A	ACCCATAAAA,GAACATAGAA	exonic	OR51H1P	nonsynonymous SNV	OR51H1P:ENST00000322059.1:exon1:c.C767T:p.P256L,	11p15.4	.
11	5424133	C	T	TTGTCTTTTT,CAGATGTTCT	exonic	OR51J1	nonsynonymous SNV	OR51J1:ENST00000332043.1:exon1:c.C307T:p.P103S,	11p15.4	.
11	5878392	G	A	TAAGTATGAG,GATGATACGA	exonic	OR52E8	nonsynonymous SNV	OR52E8:ENST00000537935.1:exon1:c.C541T:p.P181S,	11p15.4	.
11	6265616	G	A	GGCCAGCCAG,AGGGACCCCC	exonic	CNGA4	nonsynonymous SNV	CNGA4:ENST00000379936.2:exon6:c.G1705A:p.E569K,	11p15.4	.
11	6891362	G	A	ATGAACCAAA,GACTCGTGCC	exonic	OR10A2	nonsynonymous SNV	OR10A2:ENST00000307322.4:exon1:c.G377A:p.R126K,	11p15.4	OR10A2;basechange=c.377G>T;AAchange=p.R126M;tissue=large_intestine
11	8640376	C	T	AGTGGAACAT,CACTCTCACT	exonic	TRIM66	nonsynonymous SNV	TRIM66:ENST00000299550.6:exon19:c.G3512A:p.G1171E,TRIM66:ENST00000402157.2:exon21:c.G3599A:p.G1200E,	11p15.4	.
11	15197504	G	A	GCGCAGGATC,GGCAGAAGCT	exonic	INSC	nonsynonymous SNV	INSC:ENST00000528567.1:exon2:c.G274A:p.G92R,INSC:ENST00000525218.1:exon2:c.G274A:p.G92R,INSC:ENST00000379554.3:exon3:c.G415A:p.G139R,INSC:ENST00000530161.1:exon2:c.G274A:p.G92R,INSC:ENST00000424273.1:exon3:c.G274A:p.G92R,INSC:ENST00000379556.3:exon3:c.G274A:p.G92R,	11p15.2	.
11	17140255	G	A	TTGCACTAAT,AACTATTCAA	exonic	PIK3C2A	nonsynonymous SNV	PIK3C2A:ENST00000540361.1:exon17:c.C1832T:p.S611L,PIK3C2A:ENST00000265970.7:exon17:c.C2972T:p.S991L,	11p15.1	.
11	17631924	C	T	TGCTTCAAGT,CCAGCACCCC	exonic	OTOG	nonsynonymous SNV	OTOG:ENST00000399391.2:exon35:c.C5113T:p.P1705S,OTOG:ENST00000342528.2:exon12:c.C2131T:p.P711S,OTOG:ENST00000399397.1:exon35:c.C4894T:p.P1632S,	11p15.1	.
11	18763942	C	T	ATCTTCTCCT,CATCCACTCT	exonic	PTPN5	nonsynonymous SNV	PTPN5:ENST00000358540.2:exon7:c.G592A:p.E198K,PTPN5:ENST00000477854.1:exon3:c.G4A:p.E2K,PTPN5:ENST00000396167.2:exon7:c.G496A:p.E166K,PTPN5:ENST00000396171.4:exon7:c.G592A:p.E198K,PTPN5:ENST00000396168.1:exon6:c.G520A:p.E174K,PTPN5:ENST00000396170.1:exon7:c.G496A:p	11p15.1	.
11	19077187	C	A	CAAAATAAGA,ATCAGAATCC	exonic	MRGPRX2	nonsynonymous SNV	MRGPRX2:ENST00000329773.2:exon2:c.G763T:p.V255F,	11p15.1	.
11	20673909	G	A	ACTGGTCCAT,GTGCTCGGAT	exonic	SLC6A5	nonsynonymous SNV	SLC6A5:ENST00000525748.1:exon15:c.G2145A:p.M715I,	11p15.1	.
11	20673910	G	A	CTGGTCCATG,TGCTCGGATG	exonic	SLC6A5	nonsynonymous SNV	SLC6A5:ENST00000525748.1:exon15:c.G2146A:p.V716M,	11p15.1	.
11	32605464	G	A	CGACATCAGT,AAGAAGATCA	exonic	EIF3M	nonsynonymous SNV	EIF3M:ENST00000525782.1:exon1:c.G31A:p.E11K,EIF3M:ENST00000524711.1:exon1:c.G31A:p.E11K,EIF3M:ENST00000524896.1:exon1:c.G31A:p.E11K,EIF3M:ENST00000531120.1:exon1:c.G31A:p.E11K,EIF3M:ENST00000532444.1:exon1:c.G31A:p.E11K,EIF3M:ENST00000531921.1:exon1:c.G31A:p.E11K,	11p13	.
11	33054307	T	C	TCTGGTACTC,TTTTAATGGA	exonic	DEPDC7	nonsynonymous SNV	DEPDC7:ENST00000241051.3:exon7:c.T1231C:p.F411L,DEPDC7:ENST00000311388.3:exon7:c.T1204C:p.F402L,	11p13	.
11	40137148	G	A	CCTGATGGCA,ATAAATGATT	exonic	LRRC4C	nonsynonymous SNV	LRRC4C:ENST00000528697.1:exon7:c.C695T:p.S232F,LRRC4C:ENST00000278198.2:exon2:c.C695T:p.S232F,LRRC4C:ENST00000527150.1:exon3:c.C695T:p.S232F,LRRC4C:ENST00000530763.1:exon5:c.C695T:p.S232F,	11p12	.
11	44072895	G	A	CCTGCGGGAA,AAGTGGCCCG	exonic	ACCSL	nonsynonymous SNV	ACCSL:ENST00000378832.1:exon4:c.G646A:p.E216K,	11p11.2	.
11	44074244	G	A	CTTCTATGGT,GCTTTGCCTT	exonic	ACCSL	nonsynonymous SNV	ACCSL:ENST00000378832.1:exon6:c.G805A:p.G269S,	11p11.2	.
11	46404316	G	A	AAAGGAGAAG,GGGTGGGGCT	exonic	MDK	nonsynonymous SNV	MDK:ENST00000489525.1:exon4:c.G424A:p.G142R,	11p11.2	.
11	47354841	C	T	CATTAAGACC,CAGGCGTCAG	exonic	MYBPC3	stopgain	MYBPC3:ENST00000545968.1:exon30:c.G3234A:p.W1078X,MYBPC3:ENST00000256993.4:exon28:c.G3231A:p.W1077X,MYBPC3:ENST00000399249.2:exon29:c.G3234A:p.W1078X,	11p11.2	.
11	47354842	C	T	ATTAAGACCC,AGGCGTCAGT	exonic	MYBPC3	stopgain	MYBPC3:ENST00000545968.1:exon30:c.G3233A:p.W1078X,MYBPC3:ENST00000256993.4:exon28:c.G3230A:p.W1077X,MYBPC3:ENST00000399249.2:exon29:c.G3233A:p.W1078X,	11p11.2	.
11	47707525	C	T	TTATTACAGC,ATACAGGAAG	exonic	AGBL2	nonsynonymous SNV	AGBL2:ENST00000298861.4:exon10:c.G1708A:p.G570S,AGBL2:ENST00000525123.1:exon11:c.G1708A:p.G570S,AGBL2:ENST00000528244.1:exon10:c.G1594A:p.G532S,AGBL2:ENST00000357610.3:exon10:c.G1708A:p.G570S,	11p11.2	.
11	47711923	G	A	GCCTCTTGAG,GGTCTGGGAT	exonic	AGBL2	nonsynonymous SNV	AGBL2:ENST00000298861.4:exon9:c.C1336T:p.P446S,AGBL2:ENST00000525123.1:exon10:c.C1336T:p.P446S,AGBL2:ENST00000528244.1:exon9:c.C1222T:p.P408S,AGBL2:ENST00000357610.3:exon9:c.C1336T:p.P446S,	11p11.2	.
11	48238521	G	T	CAAGAGTCTG,ATTCTCCCAT	exonic	OR4B1	nonsynonymous SNV	OR4B1:ENST00000309562.2:exon1:c.G160T:p.D54Y,	11p11.2	.
11	48238806	G	A	TTCCTGGCTG,GGGGCTTTTG	exonic	OR4B1	nonsynonymous SNV	OR4B1:ENST00000309562.2:exon1:c.G445A:p.G149R,	11p11.2	.
11	48267322	G	A	CTGGAGCTCT,AAGGGTGGTG	exonic	OR4X2	nonsynonymous SNV	OR4X2:ENST00000302329.3:exon1:c.G667A:p.E223K,	11p11.2	.
11	48286317	G	A	TTTATTGGGG,AAAAGTAATT	exonic	OR4X1	nonsynonymous SNV	OR4X1:ENST00000320048.1:exon1:c.G905A:p.G302E,	11p11.2	OR4X1;basechange=c.905G>A;AAchange=p.G302E;tissue=skin
11	49078819	C	T	TGGAAAAGCT,TTTTGCTTCA	exonic	TRIM64C	nonsynonymous SNV	TRIM64C:ENST00000530230.1:exon4:c.G610A:p.E204K,	11p11.12	.
11	55541409	C	T	ATATTTTCTT,TTGACTTATC	exonic	OR5D13	nonsynonymous SNV	OR5D13:ENST00000361760.1:exon1:c.C496T:p.L166F,	11q11	.
11	55944571	A	T	ATTAATCCAC,CAATCAGCTT	exonic	OR5J2	nonsynonymous SNV	OR5J2:ENST00000312298.1:exon1:c.A478T:p.T160S,	11q12.1	.
11	56043311	G	A	GGGGATTTCT,GCTTCACAGC	exonic	OR5T1	stopgain	OR5T1:ENST00000313033.2:exon1:c.G197A:p.W66X,	11q12.1	.
11	56086657	G	A	TATAGTTTAC,AAACAAAGAT	exonic	OR8K3	nonsynonymous SNV	OR8K3:ENST00000312711.1:exon1:c.G875A:p.R292Q,	11q12.1	OR8K3;basechange=c.875G>A;AAchange=p.R292Q;tissue=skin
11	56113999	C	G	ACGTTTGTGT,ACTATTTCTC	exonic	OR8K1	stopgain	OR8K1:ENST00000279783.2:exon1:c.C485G:p.S162X,	11q12.1	.
11	56128522	C	T	AGTAACCATT,ACTGGATACT	exonic	OR8J1	nonsynonymous SNV	OR8J1:ENST00000303039.3:exon1:c.C800T:p.S267L,	11q12.1	.
11	56258246	C	A	CAGCCAGCCA,AATAAACATT	exonic	OR5M8	nonsynonymous SNV	OR5M8:ENST00000327216.2:exon1:c.G601T:p.V201L,	11q12.1	.
11	57075917	G	A	AGGGTCTGCA,GGTGTGGCTC	exonic	TNKS1BP1	nonsynonymous SNV	TNKS1BP1:ENST00000532437.1:exon5:c.C4268T:p.P1423L,TNKS1BP1:ENST00000358252.3:exon6:c.C4268T:p.P1423L,	11q12.1	.
11	57075918	G	A	GGGTCTGCAG,GTGTGGCTCC	exonic	TNKS1BP1	nonsynonymous SNV	TNKS1BP1:ENST00000532437.1:exon5:c.C4267T:p.P1423S,TNKS1BP1:ENST00000358252.3:exon6:c.C4267T:p.P1423S,	11q12.1	.
11	58125702	G	A	CTGAGCATGG,GATGATCATA	exonic	OR5B17	nonsynonymous SNV	OR5B17:ENST00000357377.3:exon1:c.C841T:p.P281S,	11q12.1	.
11	58207512	A	G	CAGGTTCCCA,CCAGAGTGAT	exonic	OR5B12	nonsynonymous SNV	OR5B12:ENST00000302572.2:exon1:c.T113C:p.V38A,	11q12.1	.
11	58920606	C	T	TGCTGTGATC,CTCAGGGTCA	exonic	FAM111A	nonsynonymous SNV	FAM111A:ENST00000528737.1:exon5:c.C1465T:p.P489S,FAM111A:ENST00000420244.1:exon5:c.C1465T:p.P489S,FAM111A:ENST00000531147.1:exon2:c.C1465T:p.P489S,FAM111A:ENST00000361723.3:exon4:c.C1465T:p.P489S,FAM111A:ENST00000533703.1:exon3:c.C1465T:p.P489S,	11q12.1	.
11	58979209	G	A	CCCACCGAAA,AGAAGTTGGT	exonic	MPEG1	nonsynonymous SNV	MPEG1:ENST00000361050.3:exon1:c.C1130T:p.S377F,	11q12.1	.
11	60105281	C	T	ATTCTCCTGT,TGTCAACCCG	exonic	MS4A6E	nonsynonymous SNV	MS4A6E:ENST00000300182.4:exon2:c.C215T:p.S72F,MS4A6E:ENST00000532756.1:exon2:c.C140T:p.S47F,	11q12.2	.
11	60170494	C	T	CAGATGCCAT,CTTTGAAGAA	exonic	MS4A14	nonsynonymous SNV	MS4A14:ENST00000527841.1:exon3:c.C377T:p.S126F,MS4A14:ENST00000395001.1:exon4:c.C92T:p.S31F,MS4A14:ENST00000395005.2:exon3:c.C377T:p.S126F,MS4A14:ENST00000526375.1:exon3:c.C377T:p.S126F,MS4A14:ENST00000530662.1:exon3:c.C377T:p.S126F,MS4A14:ENST00000525397.1:exon4:c	11q12.2	.
11	61252187	G	A	CGACACCCAG,AGCACGGGCC	exonic	PPP1R32	nonsynonymous SNV	PPP1R32:ENST00000432063.2:exon5:c.G409A:p.E137K,PPP1R32:ENST00000338608.2:exon5:c.G409A:p.E137K,	11q12.2	.
11	61546871	C	T	AGCCTCAAGT,CACCGGCAGC	exonic	MYRF	nonsynonymous SNV	MYRF:ENST00000278836.5:exon15:c.C2150T:p.S717F,MYRF:ENST00000327797.1:exon9:c.C1082T:p.S361F,MYRF:ENST00000265460.5:exon15:c.C2123T:p.S708F,MYRF:ENST00000389602.4:exon3:c.C323T:p.S108F,	11q12.2	.
11	62010865	G	A	TGCCCCTCCG,AAGCTGTTGC	exonic	SCGB1D2	nonsynonymous SNV	SCGB1D2:ENST00000244926.3:exon2:c.G160A:p.E54K,	11q12.3	.
11	62382223	G	A	AAAACAGCAT,GCTACAGGGA	exonic	ROM1	stopgain	ROM1:ENST00000278833.3:exon3:c.G968A:p.W323X,	11q12.3	.
11	62462086	G	A	GGTGTAGCAG,AAATGGTGAC	exonic	BSCL2	nonsynonymous SNV	BSCL2:ENST00000405837.1:exon5:c.C584T:p.S195F,BSCL2:ENST00000524862.1:exon5:c.C584T:p.S195F,BSCL2:ENST00000301781.6:exon4:c.C584T:p.S195F,BSCL2:ENST00000448568.2:exon4:c.C392T:p.S131F,BSCL2:ENST00000421906.1:exon4:c.C392T:p.S131F,BSCL2:ENST00000407022.3:exon4:c.C392	11q12.3	.
11	62520112	G	A	CTCTGGCTGG,AGGGCTGTGC	exonic	ZBTB3	nonsynonymous SNV	ZBTB3:ENST00000394807.3:exon2:c.C1175T:p.S392F,	11q12.3	.
11	62554999	G	C	CTCGGACCCA,GTGCGGCTGC	exonic	TMEM179B	nonsynonymous SNV	TMEM179B:ENST00000333449.4:exon1:c.G96C:p.Q32H,TMEM179B:ENST00000533861.1:exon1:c.G96C:p.Q32H,	11q12.3	.
11	62848491	G	A	GACTCCATAG,AAATCCAGGG	exonic	SLC22A24	nonsynonymous SNV	SLC22A24:ENST00000417740.1:exon9:c.C1499T:p.S500F,	11q12.3	.
11	63885738	C	T	CGGCGGCATC,CCGACATAGA	exonic	FLRT1	nonsynonymous SNV	FLRT1:ENST00000246841.3:exon2:c.C1999T:p.P667S,	11q13.1	.
11	64418076	G	A	ATCAAGGACG,GCCATTCCCC	exonic	NRXN2	nonsynonymous SNV	NRXN2:ENST00000265459.6:exon15:c.C2953T:p.P985S,NRXN2:ENST00000409571.1:exon14:c.C2932T:p.P978S,NRXN2:ENST00000377559.3:exon13:c.C2833T:p.P945S,NRXN2:ENST00000377551.1:exon14:c.C2953T:p.P985S,	11q13.1	.
11	64419096	C	T	CATATGGGCT,CTGCCATCTG	exonic	NRXN2	nonsynonymous SNV	NRXN2:ENST00000265459.6:exon14:c.G2549A:p.G850E,NRXN2:ENST00000409571.1:exon13:c.G2528A:p.G843E,NRXN2:ENST00000377559.3:exon12:c.G2429A:p.G810E,NRXN2:ENST00000377551.1:exon13:c.G2549A:p.G850E,	11q13.1	.
11	64503016	C	T	GAGCTCACCT,CACCATCTTC	exonic	RASGRP2	nonsynonymous SNV	RASGRP2:ENST00000354024.3:exon11:c.G1294A:p.E432K,RASGRP2:ENST00000377497.3:exon11:c.G1294A:p.E432K,RASGRP2:ENST00000394432.3:exon11:c.G1294A:p.E432K,RASGRP2:ENST00000377494.1:exon10:c.G1294A:p.E432K,	11q13.1	RASGRP2;basechange=c.1480G>C;AAchange=p.E494Q;tissue=lung
11	64594777	G	A	CTGCTCCTCC,ACACGCGGAA	exonic	CDC42BPG	nonsynonymous SNV	CDC42BPG:ENST00000342711.5:exon33:c.C4244T:p.S1415L,	11q13.1	.
11	64854020	G	A	CCGTGGCCTC,GCACTGAGAG	exonic	ZFPL1	nonsynonymous SNV	ZFPL1:ENST00000531761.1:exon3:c.C236A:p.P79Q,	11q13.1	.
11	65767559	G	A	GTATTTGGAG,GCATGCTCAC	exonic	EIF1AD	nonsynonymous SNV	EIF1AD:ENST00000530462.1:exon3:c.C161T:p.P54L,EIF1AD:ENST00000526451.1:exon3:c.C161T:p.P54L,EIF1AD:ENST00000525767.1:exon2:c.C5T:p.P2L,EIF1AD:ENST00000312234.2:exon3:c.C161T:p.P54L,EIF1AD:ENST00000527249.1:exon3:c.C161T:p.P54L,EIF1AD:ENST00000533544.1:exon3:c.C161T:p.P5	11q13.1	.
11	65810334	G	A	ACGCACGCGC,GCCCGCGCGC	exonic	GAL3ST3	nonsynonymous SNV	GAL3ST3:ENST00000527878.1:exon2:c.C940T:p.R314C,GAL3ST3:ENST00000312006.4:exon3:c.C940T:p.R314C,	11q13.1	.
11	66062465	G	A	CGAGGGCCTG,ACGACTATCT	exonic	TMEM151A	nonsynonymous SNV	TMEM151A:ENST00000327259.4:exon2:c.G748A:p.D250N,	11q13.2	.
11	66135001	G	A	TTATTGGCCA,GTAGTAGCGG	exonic	SLC29A2	nonsynonymous SNV	SLC29A2:ENST00000541567.1:exon7:c.C599T:p.P200L,	11q13.2	.
11	66617523	C	T	GGCCTCTGCC,GGCTCAATCC	exonic	PC	nonsynonymous SNV	PC:ENST00000393960.1:exon20:c.G2783A:p.R928Q,PC:ENST00000393958.2:exon19:c.G2783A:p.R928Q,PC:ENST00000529047.1:exon2:c.G143A:p.R48Q,PC:ENST00000393955.2:exon18:c.G2783A:p.R928Q,	11q13.2	.
11	67120533	G	T	CTCGTCGCGG,GCTGGGGCTC	exonic	POLD4	nonsynonymous SNV	POLD4:ENST00000312419.3:exon2:c.C113A:p.P38H,POLD4:ENST00000539074.1:exon2:c.C113A:p.P38H,POLD4:ENST00000531239.1:exon2:c.C52A:p.P18T,	11q13.2	.
11	67173101	G	A	CACAGTGGAT,GAGACCATTG	exonic	TBC1D10C	nonsynonymous SNV	TBC1D10C:ENST00000526387.1:exon5:c.G396A:p.M132I,TBC1D10C:ENST00000542590.1:exon4:c.G396A:p.M132I,TBC1D10C:ENST00000312390.5:exon5:c.G396A:p.M132I,TBC1D10C:ENST00000529635.1:exon4:c.G396A:p.M132I,	11q13.2	.
11	67173102	G	A	ACAGTGGATG,AGACCATTGG	exonic	TBC1D10C	nonsynonymous SNV	TBC1D10C:ENST00000526387.1:exon5:c.G397A:p.E133K,TBC1D10C:ENST00000542590.1:exon4:c.G397A:p.E133K,TBC1D10C:ENST00000312390.5:exon5:c.G397A:p.E133K,TBC1D10C:ENST00000529635.1:exon4:c.G397A:p.E133K,	11q13.2	.
11	67223114	C	T	CAATAACCCT,CCAGCACTGG	exonic	CABP4	nonsynonymous SNV	CABP4:ENST00000325656.5:exon1:c.C220T:p.P74S,CABP4:ENST00000545777.1:exon1:c.C220T:p.P74S,	11q13.2	CABP4;basechange=c.220C>A;AAchange=p.P74T;tissue=lung
11	67223115	C	T	AATAACCCTC,CAGCACTGGA	exonic	CABP4	nonsynonymous SNV	CABP4:ENST00000325656.5:exon1:c.C221T:p.P74L,CABP4:ENST00000545777.1:exon1:c.C221T:p.P74L,	11q13.2	.
11	67808785	T	A	GTCCAGCTCT,TCTGCCCACA	exonic	TCIRG1	nonsynonymous SNV	TCIRG1:ENST00000265686.3:exon2:c.T47A:p.F16Y,TCIRG1:ENST00000529657.1:exon3:c.T47A:p.F16Y,TCIRG1:ENST00000524598.1:exon2:c.T47A:p.F16Y,TCIRG1:ENST00000534673.1:exon2:c.T47A:p.F16Y,	11q13.2	.
11	68772778	C	T	CACTGCATCT,CATGGTGACT	exonic	MRGPRF	nonsynonymous SNV	MRGPRF:ENST00000309099.6:exon3:c.G1000A:p.E334K,MRGPRF:ENST00000441623.1:exon3:c.G1000A:p.E334K,	11q13.3	.
11	68772779	C	T	ACTGCATCTC,ATGGTGACTG	exonic	MRGPRF	nonsynonymous SNV	MRGPRF:ENST00000309099.6:exon3:c.G999A:p.M333I,MRGPRF:ENST00000441623.1:exon3:c.G999A:p.M333I,	11q13.3	.
11	69062934	T	G	CTCCGTGGTG,GTCCTTCCTG	exonic	MYEOV	nonsynonymous SNV	MYEOV:ENST00000308946.3:exon2:c.T113G:p.V38G,MYEOV:ENST00000441339.2:exon2:c.T113G:p.V38G,	11q13.3	.
11	69466039	G	T	CGTGCGGGAC,TGGACATCTG	exonic	CCND1	nonsynonymous SNV	CCND1:ENST00000227507.2:exon5:c.G877T:p.V293L,	11q13.3	.
11	70319282	G	A	TCCCCAGAAG,GGGCTGGCTT	exonic	SHANK2	nonsynonymous SNV	SHANK2:ENST00000294018.4:exon15:c.C4114T:p.P1372S,SHANK2:ENST00000424924.1:exon7:c.C3079T:p.P1027S,SHANK2:ENST00000449833.2:exon10:c.C3457T:p.P1153S,SHANK2:ENST00000338508.4:exon33:c.C5245T:p.P1749S,SHANK2:ENST00000409161.1:exon10:c.C3454T:p.P1152S,SHANK2:ENST0	11q13.3	.
11	70803497	C	T	CCGTTCTCAT,CTTTGCAGCAC	exonic	SHANK2	nonsynonymous SNV	SHANK2:ENST00000457074.1:exon6:c.G883A:p.D295N,SHANK2:ENST00000413503.1:exon9:c.G883A:p.D295N,SHANK2:ENST00000338508.4:exon6:c.G883A:p.D295N,	11q13.4	.
11	71849882	G	T	TCCCCAGGTG,GAGCTCCTCA	exonic	FOLR3	stopgain	FOLR3:ENST00000456237.1:exon3:c.G172T:p.G58X,FOLR3:ENST00000445078.2:exon3:c.G166T:p.G56X,	11q13.4	.
11	71946480	C	T	CCTGGGCACC,GTGAGCGGCT	exonic	INPPL1	nonsynonymous SNV	INPPL1:ENST00000538751.1:exon22:c.C1918T:p.R640C,INPPL1:ENST00000298229.2:exon23:c.C2644T:p.R882C,INPPL1:ENST00000541756.1:exon23:c.C1918T:p.R640C,	11q13.4	.
11	72368990	C	T	GGGGCATGTC,CTCCTGATGT	exonic	PDE2A	nonsynonymous SNV	PDE2A:ENST00000536458.1:exon1:c.G110A:p.G37E,	11q13.4	.
11	72406132	G	A	AGCAGTCATG,ATGCTGGGAC	exonic	ARAP1	nonsynonymous SNV	ARAP1:ENST00000426523.1:exon25:c.C2852T:p.S951F,ARAP1:ENST00000334211.8:exon25:c.C2852T:p.S951F,ARAP1:ENST00000455638.2:exon25:c.C3587T:p.S1196F,ARAP1:ENST00000393605.3:exon22:c.C2867T:p.S956F,ARAP1:ENST00000393609.3:exon27:c.C3587T:p.S1196F,ARAP1:ENST000004296	11q13.4	.
11	72613632	G	T	TGGGGTTTGT,AAATACAGCG	exonic	FCHSD2	nonsynonymous SNV	FCHSD2:ENST00000409314.1:exon10:c.C880A:p.H294N,FCHSD2:ENST00000458644.2:exon9:c.C400A:p.H134N,FCHSD2:ENST00000409853.1:exon10:c.C712A:p.H238N,FCHSD2:ENST00000409418.4:exon10:c.C880A:p.H294N,FCHSD2:ENST00000311172.7:exon9:c.C712A:p.H238N,	11q13.4	.
11	73021176	G	A	GGGGACCGTG,AAGCAACCCC	exonic	ARHGEF17	nonsynonymous SNV	ARHGEF17:ENST00000263674.3:exon1:c.G1493A:p.G498E,	11q13.4	.
11	73670617	C	T	GGATCCAGA,CCCATGGACA	exonic	DNAJB13	nonsynonymous SNV	DNAJB13:ENST00000339764.1:exon3:c.C251T:p.T84I,	11q13.4	.
11	73714930	G	A	CAGTCGAGGG,GCTGAAGTAC	exonic	UCP3	nonsynonymous SNV	UCP3:ENST00000314032.4:exon6:c.C766T:p.P256S,UCP3:ENST00000348534.4:exon4:c.C460T:p.P154S,UCP3:ENST00000426995.2:exon6:c.C766T:p.P256S,	11q13.4	.
11	74876836	G	A	TTGCAGGTGG,GAACACAGCC	exonic	SLCO2B1	nonsynonymous SNV	SLCO2B1:ENST00000428359.2:exon4:c.G224A:p.G75E,SLCO2B1:ENST00000289575.5:exon4:c.G290A:p.G97E,SLCO2B1:ENST00000534186.1:exon4:c.G224A:p.G75E,SLCO2B1:ENST00000527180.1:exon4:c.G224A:p.G75E,SLCO2B1:ENST00000531713.1:exon4:c.G224A:p.G75E,	11q13.4	.
11	74987890	C	T	TACTCAGCCT,TTCCATGGCA	exonic	ARRB1	nonsynonymous SNV	ARRB1:ENST00000360025.3:exon10:c.G769A:p.E257K,ARRB1:ENST00000393505.4:exon10:c.G769A:p.E257K,ARRB1:ENST00000420843.2:exon10:c.G769A:p.E257K,ARRB1:ENST00000532525.1:exon10:c.G754A:p.E252K,	11q13.4	.
11	75280056	C	T	CACAAGCTCT,CAGCCTCATC	exonic	SERPINH1	nonsynonymous SNV	SERPINH1:ENST00000532356.1:exon5:c.C794T:p.S265F,SERPINH1:ENST00000358171.3:exon4:c.C794T:p.S265F,SERPINH1:ENST00000530284.1:exon5:c.C794T:p.S265F,SERPINH1:ENST00000524558.1:exon4:c.C794T:p.S265F,SERPINH1:ENST00000533603.1:exon5:c.C794T:p.S265F,SERPINH1:ENST0000	11q13.5	.
11	75316854	C	T	GAAATTTACT,TTTCGCCTCA	exonic	MAP6	nonsynonymous SNV	MAP6:ENST00000434603.2:exon3:c.G1315A:p.E439K,MAP6:ENST00000526740.1:exon3:c.G328A:p.E110K,MAP6:ENST00000304771.3:exon3:c.G1315A:p.E439K,	11q13.5	.
11	75438540	G	A	CCACCCCCAT,GAGTCCTGGC	exonic	MOGAT2	nonsynonymous SNV	MOGAT2:ENST00000198801.5:exon3:c.G331A:p.G111R,MOGAT2:ENST00000526712.1:exon2:c.G85A:p.G29R,MOGAT2:ENST00000525093.1:exon3:c.G331A:p.G111R,	11q13.5	.
11	76371618	G	A	GAAGCACAGG,AGGTCAGGTG	exonic	LRRC32	nonsynonymous SNV	LRRC32:ENST00000404995.1:exon3:c.C1019T:p.S340F,LRRC32:ENST00000407242.2:exon3:c.C1019T:p.S340F,LRRC32:ENST00000260061.5:exon3:c.C1019T:p.S340F,	11q13.5	.
11	77386128	G	A	ATCATCATCG,AATATTTTTT	exonic	RSF1	nonsynonymous SNV	RSF1:ENST00000308488.6:exon14:c.C3515T:p.S1172F,RSF1:ENST00000480887.1:exon9:c.C2759T:p.S920F,RSF1:ENST00000360355.2:exon13:c.C3422T:p.S1141F,	11q14.1	.
11	78574019	C	T	TCTGTGGTTC,TTTGCCTTTC	exonic	TENM4	nonsynonymous SNV	TENM4:ENST00000278550.7:exon10:c.G1243A:p.G415R,	11q14.1	.
11	83673979	C	T	TGAGTACCTT,CTGGAGAGAT	exonic	DLG2	nonsynonymous SNV	DLG2:ENST00000398301.2:exon9:c.G1091A:p.G364E,DLG2:ENST00000524982.1:exon9:c.G974A:p.G325E,DLG2:ENST00000418306.2:exon7:c.G821A:p.G274E,DLG2:ENST00000280241.8:exon8:c.G1091A:p.G364E,DLG2:ENST00000330014.6:exon8:c.G791A:p.G264E,DLG2:ENST00000398309.2:exon9:c.G974A:	11q14.1	.
11	84245714	C	T	CCTCTTACTT,GTGGGTTAGT	exonic	DLG2	nonsynonymous SNV	DLG2:ENST00000524982.1:exon2:c.G103A:p.E35K,DLG2:ENST00000527088.1:exon3:c.G166A:p.E56K,DLG2:ENST00000398309.2:exon2:c.G103A:p.E35K,DLG2:ENST00000543673.1:exon5:c.G418A:p.E140K,DLG2:ENST00000532653.1:exon2:c.G103A:p.E35K,DLG2:ENST00000376104.2:exon7:c.G418A:p.E140K,	11q14.1	.
11	92532054	G	A	TAAGGTGTCT,ATGGAAAGTT	exonic	FAT3	nonsynonymous SNV	FAT3:ENST00000298047.6:exon9:c.G5875A:p.D1959N,FAT3:ENST00000409404.2:exon9:c.G5875A:p.D1959N,FAT3:ENST00000525166.1:exon9:c.G5425A:p.D1809N,	11q14.3	.
11	92532306	G	A	AGTCCCCCTT,ACCGTGAAGA	exonic	FAT3	nonsynonymous SNV	FAT3:ENST00000298047.6:exon9:c.G6127A:p.D2043N,FAT3:ENST00000409404.2:exon9:c.G6127A:p.D2043N,FAT3:ENST00000525166.1:exon9:c.G5677A:p.D1893N,	11q14.3	.
11	92715218	A	T	TGTGGCCATC,ACCCCCAAGA	exonic	MTNR1B	nonsynonymous SNV	MTNR1B:ENST00000257068.2:exon2:c.A829T:p.N277Y,	11q14.3	.
11	93911708	G	T	ACCTTGACAT,AGAGATGGAG	exonic	PANX1	nonsynonymous SNV	PANX1:ENST00000436171.2:exon3:c.G495T:p.M165I,PANX1:ENST00000227638.3:exon3:c.G495T:p.M165I,	11q21	.
11	94758743	C	G	GCACTCCAGT,CCCAGAACAC	exonic	KDM4E	nonsynonymous SNV	KDM4E:ENST00000450979.2:exon1:c.C22G:p.P8A,	11q21	.
11	94758744	C	T	CACTCCAGTC,CCAGAACACG	exonic	KDM4E	nonsynonymous SNV	KDM4E:ENST00000450979.2:exon1:c.C23T:p.P8L,	11q21	.
11	100061935	G	A	GTTTGCCGAG,GGAAAACGTC	exonic	CNTN5	nonsynonymous SNV	CNTN5:ENST00000524871.1:exon14:c.G1658A:p.G553E,CNTN5:ENST00000418526.2:exon11:c.G1436A:p.G479E,CNTN5:ENST00000528682.1:exon13:c.G1658A:p.G553E,CNTN5:ENST00000527185.1:exon14:c.G1658A:p.G553E,CNTN5:ENST00000279463.3:exon12:c.G1658A:p.G553E,	11q22.1	.
11	101984989	G	T	GACCCCCACT,GAGTAGTCTC	exonic	YAP1	stopgain	YAP1:ENST00000531439.1:exon2:c.G436T:p.G146X,YAP1:ENST00000537274.1:exon2:c.G436T:p.G146X,YAP1:ENST00000345877.2:exon2:c.G436T:p.G146X,YAP1:ENST00000526343.1:exon2:c.G436T:p.G146X,YAP1:ENST00000282441.5:exon2:c.G436T:p.G146X,	11q22.1	.
11	102665977	T	A	GTCACACGCT,TTGGGGTTTG	exonic	MMP1	nonsynonymous SNV	MMP1:ENST00000315274.6:exon6:c.A827T:p.K276I,	11q22.2	.
11	103866942	C	T	GTACTGGTTT,GGATATATCT	exonic	PDGFD	nonsynonymous SNV	PDGFD:ENST00000393158.2:exon3:c.G361A:p.E121K,PDGFD:ENST00000529268.1:exon3:c.G430A:p.E144K,PDGFD:ENST00000302251.5:exon3:c.G343A:p.E115K,	11q22.3	.
11	105929725	G	A	TTTTGTCCAT,ATCTTCTGAT	exonic	KBTBD3	nonsynonymous SNV	KBTBD3:ENST00000526793.1:exon2:c.C100T:p.H34Y,KBTBD3:ENST00000531482.1:exon1:c.C100T:p.H34Y,KBTBD3:ENST00000531837.1:exon3:c.C100T:p.H34Y,	11q22.3	.
11	110451086	C	T	GAATAAATTC,CCTGAGATAG	exonic	ARHGAP20	nonsynonymous SNV	ARHGAP20:ENST00000533353.1:exon16:c.G2506A:p.G836R,ARHGAP20:ENST00000529591.1:exon5:c.G1213A:p.G405R,ARHGAP20:ENST00000524756.1:exon15:c.G2515A:p.G839R,ARHGAP20:ENST00000357139.3:exon15:c.G2506A:p.G836R,ARHGAP20:ENST00000260283.4:exon16:c.G2584A:p.G862R,ARH	11q23.1	.
11	111228315	G	A	CTCGGTGTAA,GTGTCCATGG	exonic	POU2AF1	nonsynonymous SNV	POU2AF1:ENST00000393067.3:exon4:c.C311T:p.P104L,POU2AF1:ENST00000531398.1:exon5:c.C311T:p.P104L,	11q23.1	.
11	111368741	G	A	TACTTCAAAG,GATCTACCCA	exonic	BTG4	nonsynonymous SNV	BTG4:ENST00000525791.1:exon3:c.C293T:p.P98L,BTG4:ENST00000356018.2:exon3:c.C293T:p.P98L,BTG4:ENST00000456861.2:exon4:c.C293T:p.P98L,	11q23.1	.
11	114569133	G	A	TTCAGGAAAG,TGACTGACTT	exonic	NXPE2	nonsynonymous SNV	NXPE2:ENST00000375475.5:exon3:c.G499A:p.V167M,NXPE2:ENST00000389586.4:exon3:c.G499A:p.V167M,	11q23.2	.
11	116643592	G	A	CTTCGCGACG,ACTCAGATCC	exonic	BUD13	nonsynonymous SNV	BUD13:ENST00000375445.3:exon1:c.C89T:p.S30F,BUD13:ENST00000260210.4:exon1:c.C89T:p.S30F,	11q23.3	.
11	116767031	G	A	CAGATTCTGC,GTGTGCTTCC	exonic	SIK3	nonsynonymous SNV	SIK3:ENST00000292055.4:exon6:c.C629A:p.L210Q,SIK3:ENST00000542607.1:exon6:c.T629A:p.L210Q,SIK3:ENST00000434315.2:exon7:c.T326A:p.L109Q,SIK3:ENST00000446921.2:exon6:c.T803A:p.L268Q,SIK3:ENST00000375300.1:exon6:c.T803A:p.L268Q,	11q23.3	.
11	117109790	G	A	GATCAAGATA,CTCTTCCCTG	exonic	RNF214	nonsynonymous SNV	RNF214:ENST00000531452.1:exon3:c.G581A:p.S194N,RNF214:ENST00000300650.4:exon2:c.G581A:p.S194N,	11q23.3	.
11	117267818	A	T	CTGTCCTCCC,CAATGTCTGG	exonic	CEP164	nonsynonymous SNV	CEP164:ENST00000278935.3:exon27:c.A3290T:p.H1097L,	11q23.3	.
11	118498572	A	G	GGAGGCCCGG,GAGCACTGGA	exonic	PHLDB1	nonsynonymous SNV	PHLDB1:ENST00000361417.2:exon7:c.A1033G:p.R345G,PHLDB1:ENST00000528594.1:exon6:c.A1033G:p.R345G,PHLDB1:ENST00000530994.1:exon7:c.A1033G:p.R345G,PHLDB1:ENST00000356063.5:exon6:c.A1033G:p.R345G,	11q23.3	.
11	121028820	C	T	CATCTCCTTC,AGCTTATCAT	exonic	TECTA	stopgain	TECTA:ENST00000392793.1:exon14:c.C4576T:p.Q1526X,TECTA:ENST00000264037.2:exon13:c.C4576T:p.Q1526X,	11q23.3	.
11	123464874	G	A	CCTCATTGTT,GTGAGTTGGG	exonic	GRAMD1B	nonsynonymous SNV	GRAMD1B:ENST00000534764.1:exon4:c.G328A:p.D110N,GRAMD1B:ENST00000456860.2:exon5:c.G361A:p.D121N,GRAMD1B:ENST00000529750.1:exon4:c.G340A:p.D114N,GRAMD1B:ENST00000529432.1:exon4:c.G220A:p.D74N,GRAMD1B:ENST00000322282.7:exon4:c.G340A:p.D114N,	11q24.1	.
11	124487356	C	T	GAGTTCCGAC,CCTATGTGTT	exonic	PANX3	nonsynonymous SNV	PANX3:ENST00000284288.2:exon3:c.C511T:p.P171S,	11q24.2	.
11	124487357	C	T	AGTTCCGACC,CTATGTGTTC	exonic	PANX3	nonsynonymous SNV	PANX3:ENST00000284288.2:exon3:c.C512T:p.P171L,	11q24.2	.
11	132180045	C	T	ACAGGTGTCC,CGTGGGACAA	exonic	NTM	nonsynonymous SNV	NTM:ENST00000427481.2:exon6:c.C674T:p.P225L,NTM:ENST00000374791.3:exon6:c.C701T:p.P234L,NTM:ENST00000539799.1:exon6:c.C701T:p.P234L,NTM:ENST00000425719.2:exon5:c.C701T:p.P234L,NTM:ENST00000374786.1:exon5:c.C701T:p.P234L,NTM:ENST00000374784.1:exon5:c.C701T:p.P234L,	11q25	.
11	134251849	G	A	TCCACCGAGG,GTCAGTGAAG	exonic	B3GAT1	nonsynonymous SNV	B3GAT1:ENST00000312527.4:exon5:c.C988T:p.P330S,B3GAT1:ENST00000392580.1:exon6:c.C988T:p.P330S,B3GAT1:ENST00000537389.1:exon5:c.C1027T:p.P343S,B3GAT1:ENST00000524765.1:exon5:c.C988T:p.P330S,	11q25	.
12	971422	C	T	TGGGGTATAT,CACCCTCAAG	exonic	WNK1	nonsynonymous SNV	WNK1:ENST00000315939.6:exon8:c.C2125T:p.P709S,WNK1:ENST00000530271.2:exon8:c.C2125T:p.P709S,WNK1:ENST00000535572.1:exon8:c.C2125T:p.P709S,WNK1:ENST00000535698.1:exon1:c.C22T:p.P8S,WNK1:ENST00000537687.1:exon8:c.C2125T:p.P709S,WNK1:ENST00000340908.4:exon5:c.C904T:	12p13.33	.
12	2927481	G	A	CAGGAGAGTA,GAGTCAGTGG	exonic	ITFG2	nonsynonymous SNV	ITFG2:ENST00000542548.1:exon4:c.G32A:p.R11K,	12p13.33	.
12	3649784	C	T	GAACAGCCCC,CCTCCCAGCC	exonic	PRMT8	nonsynonymous SNV	PRMT8:ENST00000452611.2:exon2:c.C61T:p.P21S,PRMT8:ENST00000382622.3:exon2:c.C88T:p.P30S,	12p13.32	.
12	3750957	C	T	ATCTCTTTTT,CAACTTGCCC	exonic	EFCAB4B	stopgain	EFCAB4B:ENST00000444507.1:exon13:c.G1212A:p.W404X,EFCAB4B:ENST00000440314.2:exon13:c.G1212A:p.W404X,	12p13.32	.
12	6103351	C	T	GTCATTGGCT,CGTTCTCATC	exonic	VWF	nonsynonymous SNV	VWF:ENST00000261405.5:exon37:c.G6275A:p.G2092E,	12p13.31	.
12	6753407	C	T	TCTCCATTAT,ATAGGAGTAG	exonic	ACRBP	nonsynonymous SNV	ACRBP:ENST00000229243.2:exon5:c.G840A:p.M280I,ACRBP:ENST00000536350.1:exon5:c.G840A:p.M280I,ACRBP:ENST00000414226.2:exon5:c.G741A:p.M247I,	12p13.31	.
12	6779936	G	A	CTGCAGATTG,AGAGTTGTGT	exonic	ZNF384	nonsynonymous SNV	ZNF384:ENST00000396799.2:exon8:c.C980T:p.S327F,ZNF384:ENST00000396795.1:exon7:c.C980T:p.S327F,ZNF384:ENST00000355772.4:exon7:c.C815T:p.S272F,ZNF384:ENST00000319770.3:exon7:c.C932T:p.S311F,ZNF384:ENST00000361959.3:exon9:c.C1163T:p.S388F,ZNF384:ENST00000396801.3:exon9:	12p13.31	.
12	7091898	A	T	TCGAAGGATG,GGAACTGAAG	exonic	LPCAT3	nonsynonymous SNV	LPCAT3:ENST00000535479.1:exon3:c.T305A:p.L102H,LPCAT3:ENST00000261407.4:exon3:c.T305A:p.L102H,LPCAT3:ENST00000543794.1:exon3:c.T305A:p.L102H,LPCAT3:ENST00000536797.1:exon3:c.T305A:p.L102H,	12p13.31	.
12	7361668	C	T	CTCCATTGAC,CTGATGTGCA	exonic	PEX5	nonsynonymous SNV	PEX5:ENST00000420616.2:exon14:c.C1462T:p.P488S,PEX5:ENST00000412720.2:exon14:c.C1525T:p.P509S,PEX5:ENST00000266564.3:exon13:c.C1438T:p.P480S,PEX5:ENST00000434354.2:exon14:c.C1507T:p.P503S,PEX5:ENST00000266563.5:exon13:c.C1351T:p.P451S,PEX5:ENST00000455147.2:exon15:c	12p13.31	.
12	7640656	C	T	GGGAATGTCC,CTCCAACCAG	exonic	CD163	nonsynonymous SNV	CD163:ENST00000541972.1:exon7:c.G1412A:p.G471E,CD163:ENST00000359156.4:exon7:c.G1448A:p.G483E,CD163:ENST00000432237.2:exon7:c.G1448A:p.G483E,CD163:ENST00000396620.3:exon7:c.G1448A:p.G483E,	12p13.31	.
12	7890143	G	T	CTGAAATGAA,TCCAAGGGGT	exonic	CLEC4C	nonsynonymous SNV	CLEC4C:ENST00000542353.1:exon5:c.C263A:p.T88N,CLEC4C:ENST00000360345.3:exon4:c.C263A:p.T88N,CLEC4C:ENST00000537530.1:exon1:c.C29A:p.T10N,CLEC4C:ENST00000354629.5:exon5:c.C170A:p.T57N,CLEC4C:ENST00000543765.1:exon3:c.C143A:p.T48N,CLEC4C:ENST00000540085.1:exon4:c	12p13.31	.
12	8200738	C	T	CTATGGGCCT,CCCCTGTAAT	exonic	FOXJ2	nonsynonymous SNV	FOXJ2:ENST00000428177.2:exon7:c.C1078T:p.R360S,FOXJ2:ENST00000162391.3:exon7:c.C1078T:p.R360S,	12p13.31	.
12	8200739	C	T	TATGGGCCTC,CCCTGTAATG	exonic	FOXJ2	nonsynonymous SNV	FOXJ2:ENST00000428177.2:exon7:c.C1079T:p.P360L,FOXJ2:ENST00000162391.3:exon7:c.C1079T:p.P360L,	12p13.31	.
12	8211821	C	T	GTGAATTGGC,TAAATTGTAA	exonic	C3AR1	nonsynonymous SNV	C3AR1:ENST00000307637.4:exon2:c.G961A:p.V321S,	12p13.31	.
12	8212598	G	A	GTGAGGTGGA,GAACCAAATT	exonic	C3AR1	nonsynonymous SNV	C3AR1:ENST00000307637.4:exon2:c.C184T:p.L62F,C3AR1:ENST00000546241.1:exon2:c.C184T:p.L62F,	12p13.31	.
12	9161650	G	A	TGGGAAGATG,ATCACCTCTA	exonic	KLRG1	nonsynonymous SNV	KLRG1:ENST00000539240.1:exon4:c.G200A:p.G67E,KLRG1:ENST00000266551.4:exon4:c.G437A:p.G146E,KLRG1:ENST00000543895.1:exon3:c.G200A:p.G67E,KLRG1:ENST00000356986.3:exon4:c.G437A:p.G146E,	12p13.31	.
12	9262543	G	A	GCCCTGGAAG,GCTCTGATGA	exonic	A2M	nonsynonymous SNV	A2M:ENST00000318602.7:exon6:c.C593T:p.P198L,	12p13.31	.
12	10217364	G	T	GAAGATTAAA,GAAGCTATGA	exonic	CLEC9A	stopgain	CLEC9A:ENST00000355819.1:exon8:c.G505T:p.G169X,	12p13.2	.
12	11546848	C	T	TTGTGGCTTT,GGAGGAGAGA	exonic	PRB2	nonsynonymous SNV	PRB2:ENST00000389362.4:exon3:c.G164A:p.G55E,	12p13.2	.
12	12976240	C	T	ATTTTTATTC,CTCTAAATTC	exonic	DDX47	nonsynonymous SNV	DDX47:ENST00000358007.3:exon7:c.C737T:p.P246L,DDX47:ENST00000352940.4:exon7:c.C737T:p.P246L,DDX47:ENST00000544400.1:exon6:c.C548T:p.P183L,	12p13.1	.
12	18872445	A	T	AAATAAAATA,TCATTTAATG	exonic	PLCZ1	nonsynonymous SNV	PLCZ1:ENST00000541695.1:exon1:c.T78A:p.D26E,PLCZ1:ENST00000266505.7:exon5:c.T489A:p.D163E,PLCZ1:ENST00000447925.2:exon5:c.T483A:p.D161E,PLCZ1:ENST00000541966.1:exon5:c.T177A:p.D59E,	12p12.3	.
12	19511355	C	T	AGGGATAATC,ATTTAGGACT	exonic	PLEKHA5	nonsynonymous SNV	PLEKHA5:ENST00000424268.1:exon22:c.C2801T:p.P934L,PLEKHA5:ENST00000359180.3:exon19:c.C2666T:p.P889L,PLEKHA5:ENST00000538714.1:exon23:c.C3008T:p.P1003L,PLEKHA5:ENST00000538972.1:exon5:c.C677T:p.P226L,PLEKHA5:ENST00000317589.4:exon22:c.C3023T:p.P1008L,PLEKHA5:ENST	12p12.3	.
12	24989476	C	T	AATGCACCGC,TTGTCACTCC	exonic	BCAT1	nonsynonymous SNV	BCAT1:ENST00000261192.7:exon8:c.G872A:p.R291K,BCAT1:ENST00000538118.1:exon8:c.G869A:p.R290K,BCAT1:ENST00000539282.1:exon8:c.G908A:p.R303K,BCAT1:ENST00000342945.5:exon6:c.G689A:p.R230K,BCAT1:ENST00000539780.1:exon7:c.G761A:p.R254K,	12p12.1	.
12	25368495	C	T	CCTCCACTCT,TGCATTGTAA	splicing	KRAS(ENST0	.		12p12.1	.
12	27829403	C	T	GACCCTTCCT,CCAGGCCCCC	exonic	PPFIBP1	nonsynonymous SNV	PPFIBP1:ENST00000540114.1:exon15:c.C997T:p.P333S,PPFIBP1:ENST00000537261.1:exon7:c.C529T:p.P177S,PPFIBP1:ENST00000228425.6:exon18:c.C1453T:p.P485S,PPFIBP1:ENST00000318304.8:exon18:c.C1504T:p.P502S,PPFIBP1:ENST00000542629.1:exon17:c.C1411T:p.P471S,PPFIBP1:ENST0000053	12p11.22	.
12	29659856	G	C	TCCTTCAGCA,TTTGCTGTCT	exonic	TMTC1	nonsynonymous SNV	TMTC1:ENST00000539277.1:exon18:c.C2572G:p.L858V,TMTC1:ENST00000551659.1:exon20:c.C2758G:p.L920V,TMTC1:ENST00000552618.1:exon19:c.C2644G:p.L882V,TMTC1:ENST00000256062.5:exon18:c.C2248G:p.L750V,	12p11.22	.
12	31135623	G	A	CTATAAGGTG,AGGTGAGCAC	exonic	TSPAN11	nonsynonymous SNV	TSPAN11:ENST00000546076.1:exon6:c.G613A:p.E205K,TSPAN11:ENST00000535215.1:exon5:c.G400A:p.E134K,TSPAN11:ENST00000261177.9:exon6:c.G613A:p.E205K,TSPAN11:ENST00000544427.1:exon5:c.G583A:p.E195K,	12p11.21	.
12	31135624	A	G	TATAAGGTGG,GGTGAGCACC	exonic	TSPAN11	nonsynonymous SNV	TSPAN11:ENST00000546076.1:exon6:c.A614G:p.E205G,TSPAN11:ENST00000535215.1:exon5:c.A401G:p.E134G,TSPAN11:ENST00000261177.9:exon6:c.A614G:p.E205G,TSPAN11:ENST00000544427.1:exon5:c.A584G:p.E195G,	12p11.21	.
12	33559999	C	A	ACATAAGGGT,AGAAGTTCCT	exonic	SYT10	nonsynonymous SNV	SYT10:ENST00000228567.3:exon3:c.G802T:p.D268Y,SYT10:ENST00000535526.1:exon5:c.G259T:p.D87Y,	12p11.1	SYT10;basechange=c.802G>C;AAchange=p.D268H;tissue=lung
12	33579155	C	T	GTTTGGACTT,TGCTGGGATG	exonic	SYT10	nonsynonymous SNV	SYT10:ENST00000228567.3:exon2:c.G427A:p.E143K,SYT10:ENST00000539102.1:exon2:c.G427A:p.E143K,	12p11.1	.
12	40078026	T	G	CTTCCAGGCT,TTCTGAATAA	exonic	C12orf40	nonsynonymous SNV	C12orf40:ENST00000468200.2:exon9:c.T1159G:p.F387V,C12orf40:ENST00000398716.1:exon10:c.T1159G:p.F387V,	12q12	.
12	40877463	C	T	ACTGGACTAT,AGTTGGAGTG	exonic	MUC19	nonsynonymous SNV	MUC19:ENST00000454784.4:exon49:c.C9008T:p.S3003L,	12q12	.
12	43944720	C	T	ACCAGGCCTC,GCATAAGCTG	exonic	ADAMTS20	nonsynonymous SNV	ADAMTS20:ENST00000389420.3:exon2:c.G445A:p.G149R,ADAMTS20:ENST00000553158.1:exon2:c.G445A:p.G149R,	12q12	.
12	46205299	C	T	GGTGCAATTC,ATCTTCCTAC	exonic	ARID2	nonsynonymous SNV	ARID2:ENST00000334344.6:exon4:c.C383T:p.P128L,	12q12	.
12	48369298	C	T	GGGCCCTTCT,TCTCGGGCCT	exonic	COL2A1	nonsynonymous SNV	COL2A1:ENST00000380518.3:exon51:c.G3688A:p.E1230K,COL2A1:ENST00000337299.6:exon50:c.G3481A:p.E1161K,	12q13.11	.
12	48374699	G	C	GATACTCACA,GAGGCCCAGC	exonic	COL2A1	nonsynonymous SNV	COL2A1:ENST00000380518.3:exon38:c.C2516G:p.P839R,COL2A1:ENST00000337299.6:exon37:c.C2309G:p.P770R,	12q13.11	.
12	48545039	G	A	CACCATACAG,CACAGTGCAA	exonic	ASB8	nonsynonymous SNV	ASB8:ENST00000540782.1:exon3:c.C179T:p.A60V,ASB8:ENST00000317697.3:exon3:c.C179T:p.A60V,ASB8:ENST00000536549.1:exon3:c.C179T:p.A60V,ASB8:ENST00000540212.1:exon4:c.C179T:p.A60V,ASB8:ENST00000539503.1:exon4:c.C179T:p.A60V,ASB8:ENST00000535988.1:exon3:c.C179T:p.A60V,ASE	12q13.11	.
12	49426307	C	A	TCTCCTGGTT,AGTGGCCATT	exonic	KMT2D	stopgain	KMT2D:ENST00000301067.7:exon39:c.G12181T:p.E4061X,	12q13.12	.
12	49436867	G	T	ACCTTCTGTG,AAATCCTGTC	exonic	KMT2D	nonsynonymous SNV	KMT2D:ENST00000301067.7:exon25:c.C5636A:p.S1879Y,	12q13.12	.
12	49444241	G	A	AGGGCAGGAG,AGAGCACTGG	exonic	KMT2D	nonsynonymous SNV	KMT2D:ENST00000301067.7:exon11:c.C3130T:p.P1044S,	12q13.12	.
12	50367258	T	G	CCCCGTGCTG,GGCCTATGTG	exonic	AQP6	nonsynonymous SNV	AQP6:ENST00000315520.5:exon1:c.T302G:p.V101G,	12q13.12	.
12	51584355	G	T	ACTCTTGGGT,TGATGTCTAG	exonic	POU6F1	nonsynonymous SNV	POU6F1:ENST00000389243.4:exon11:c.C581A:p.T194K,POU6F1:ENST00000333640.10:exon6:c.C581A:p.T194K,POU6F1:ENST00000550824.1:exon5:c.C581A:p.T194K,	12q13.13	.
12	52629056	C	T	GATTGCTGGC,TTCGGGGTCA	exonic	KRT7	nonsynonymous SNV	KRT7:ENST00000331817.5:exon2:c.C442T:p.L148F,	12q13.13	.
12	53498960	G	A	GGCCATGCGG,AGGCTATACA	exonic	SOAT2	nonsynonymous SNV	SOAT2:ENST00000542365.1:exon3:c.G208A:p.E70K,SOAT2:ENST00000301466.3:exon3:c.G208A:p.E70K,SOAT2:ENST00000551896.1:exon3:c.G208A:p.E70K,	12q13.13	.
12	54403369	G	A	GTCCCTTTAT,GGGCTCAGCA	exonic	HOXC8	nonsynonymous SNV	HOXC8:ENST00000040584.4:exon1:c.G301A:p.G101R,	12q13.13	.
12	54677688	G	T	AGGAAATTTT,GAGGCAGAAG	exonic	HNRNPA1	stopgain	HNRNPA1:ENST00000547566.1:exon8:c.G844T:p.G282X,HNRNPA1:ENST00000340913.6:exon9:c.G1000T:p.G334X,HNRNPA1:ENST00000550482.1:exon5:c.G457T:p.G153X,HNRNPA1:ENST00000330752.8:exon8:c.G805T:p.G269X,HNRNPA1:ENST00000547276.1:exon7:c.G685T:p.G229X,HNRNPA1:ENST0000	12q13.13	.
12	54905628	G	A	TGGAGCGCTG,ATTATCAGTA	exonic	NCKAP1L	stopgain	NCKAP1L:ENST00000545638.2:exon8:c.G627A:p.W209X,NCKAP1L:ENST00000293373.6:exon8:c.G777A:p.W259X,NCKAP1L:ENST00000548221.1:exon8:c.G777A:p.W259X,	12q13.2	.
12	55615214	A	T	TTCAGTCATA,TGAACAGGTC	exonic	OR10A7	nonsynonymous SNV	OR10A7:ENST00000326258.1:exon1:c.A406T:p.M136L,	12q13.2	.
12	55714516	A	C	GACCCTTCTC,CAATTACCCT	exonic	OR6C1	nonsynonymous SNV	OR6C1:ENST00000379668.2:exon1:c.A133C:p.T45P,	12q13.2	.
12	57009025	G	A	GTCAGGAAAG,AATCATACAG	exonic	BAZ2A	nonsynonymous SNV	BAZ2A:ENST00000179765.5:exon3:c.C503T:p.S168F,BAZ2A:ENST00000549884.1:exon3:c.C503T:p.S168F,BAZ2A:ENST00000379441.3:exon3:c.C509T:p.S170F,BAZ2A:ENST00000551812.1:exon3:c.C509T:p.S170F,	12q13.3	.
12	57114367	G	A	AGACCAAAA,AACTCTGATG	exonic	NACA	nonsynonymous SNV	NACA:ENST00000550952.1:exon3:c.C947T:p.S316F,NACA:ENST00000454682.1:exon3:c.C947T:p.S316F,	12q13.3	NACA_ENST00000550952;basechange=c.947C>A;AAchange=p.S316Y;tissue=endometrium&NACA_ENST00000454682;basechange=c.947(
12	57406467	G	A	AAGCTGATTG,GATGAAGGGG	exonic	TAC3	nonsynonymous SNV	TAC3:ENST00000393867.1:exon6:c.C347T:p.P116L,TAC3:ENST00000438756.1:exon6:c.C347T:p.P116L,TAC3:ENST00000357616.3:exon6:c.C347T:p.P116L,	12q13.3	.
12	57440852	G	A	CACAAATCGG,AGGAATTGTT	exonic	MYO1A	nonsynonymous SNV	MYO1A:ENST00000300119.3:exon6:c.C470T:p.S157F,MYO1A:ENST00000442789.2:exon7:c.C470T:p.S157F,	12q13.3	.
12	63087730	C	A	TTGCCTTCTC,ATATATAAGG	exonic	PPM1H	stopgain	PPM1H:ENST00000228705.6:exon7:c.G1123T:p.G375X,	12q14.1	.
12	68646321	G	A	GGCCAGGAAG,GCACCACCTC	exonic	IL22	nonsynonymous SNV	IL22:ENST00000328087.4:exon3:c.C359T:p.P120L,IL22:ENST00000538666.1:exon4:c.C359T:p.P120L,	12q15	.
12	70249066	G	A	GCAGCTCAGG,AGCCAATGGT	exonic	MYRFL	nonsynonymous SNV	MYRFL:ENST00000547771.2:exon2:c.G53A:p.G18E,MYRFL:ENST00000552032.2:exon2:c.G53A:p.G18E,	12q15	.
12	70953320	G	T	GTTGTAGTCT,TCCAGTCTGG	exonic	PTPRB	nonsynonymous SNV	PTPRB:ENST00000261266.5:exon16:c.C3863A:p.T1288K,PTPRB:ENST00000550358.1:exon17:c.C4253A:p.T1418K,PTPRB:ENST00000538708.1:exon15:c.C3593A:p.T1198K,PTPRB:ENST00000551525.1:exon18:c.C4514A:p.T1505K,PTPRB:ENST00000334414.6:exon18:c.C4517A:p.T1506K,PTPRB:ENST0000055	12q15	.
12	71139761	C	T	GTCTTTGCCT,GGACAGTGCT	exonic	PTPRR	nonsynonymous SNV	PTPRR:ENST00000283228.2:exon6:c.G844A:p.E282K,PTPRR:ENST00000549308.1:exon3:c.G109A:p.E37K,PTPRR:ENST00000550661.1:exon3:c.G109A:p.E37K,PTPRR:ENST00000342084.4:exon5:c.G508A:p.E170K,PTPRR:ENST00000378778.1:exon3:c.G226A:p.E76K,PTPRR:ENST00000440835.2:exon2:c.G10	12q15	.
12	73050775	G	A	AATACTGAAG,TGAACTCAAA	exonic	TRHDE	nonsynonymous SNV	TRHDE:ENST00000261180.4:exon18:c.G2918A:p.G973D,	12q21.1	.
12	76741306	A	T	AGATAGACAA,AAGTGTCTAC	exonic	BBS10	nonsynonymous SNV	BBS10:ENST00000393262.3:exon2:c.T459A:p.F153L,	12q21.2	.
12	78604259	G	A	CAGCCACCAT,AAGACATTTT	exonic	NAV3	nonsynonymous SNV	NAV3:ENST00000228327.6:exon39:c.G7054A:p.E2352K,NAV3:ENST00000536525.2:exon39:c.G7054A:p.E2352K,NAV3:ENST00000397909.2:exon40:c.G7120A:p.E2374K,NAV3:ENST00000541270.1:exon5:c.G610A:p.E204K,NAV3:ENST00000266692.7:exon37:c.G6523A:p.E2175K,	12q21.2	.
12	82147808	C	T	TAGATGACAT,CTGAAGTCTT	exonic	PPFIA2	nonsynonymous SNV	PPFIA2:ENST00000552948.1:exon2:c.G193A:p.D65N,PPFIA2:ENST00000549396.1:exon3:c.G193A:p.D65N,PPFIA2:ENST00000547623.1:exon3:c.G193A:p.D65N,PPFIA2:ENST00000549325.1:exon2:c.G193A:p.D65N,PPFIA2:ENST00000548586.1:exon2:c.G193A:p.D65N,PPFIA2:ENST00000550584.2:exon2:c.G19	12q21.31	PPFIA2;basechange=c.193G>T;AAchange=p.D65Y;tissue=lung
12	82792860	C	T	GAAAAGATGC,TACCTCAGCT	exonic	METTL25	nonsynonymous SNV	METTL25:ENST00000248306.3:exon4:c.C818T:p.P273L,	12q21.31	.
12	89885749	G	A	TCGATACAAG,AGTAACAGAA	exonic	POC1B	nonsynonymous SNV	POC1B:ENST00000313546.3:exon4:c.C416T:p.S139F,POC1B:ENST00000378528.2:exon2:c.C26T:p.S9F,POC1B:ENST00000549504.1:exon2:c.C26T:p.S9F,POC1B:ENST00000393179.4:exon2:c.C26T:p.S9F,POC1B:ENST00000549035.1:exon3:c.C290T:p.S97F,POC1B:ENST00000541909.1:exon3:c.C26T:p.S9F,	12q21.33	.
12	93100748	G	A	GAACCTGAAA,GGCCCTGAAT	exonic	C12orf74	nonsynonymous SNV	C12orf74:ENST00000544406.2:exon2:c.G341A:p.R114K,C12orf74:ENST00000397833.3:exon2:c.G341A:p.R114K,	12q22	.
12	101576586	C	T	CTTACTCATT,CTTGGGAATT	exonic	SLC5A8	nonsynonymous SNV	SLC5A8:ENST00000536262.2:exon9:c.G1160A:p.G387E,	12q23.1	SLC5A8;basechange=c.1160G>C;AAchange=p.G387A;tissue=oesophagus
12	103699824	C	T	ATGCCCTGAG,CTCCAGGTGA	exonic	C12orf42	nonsynonymous SNV	C12orf42:ENST00000552578.1:exon5:c.G559A:p.A187T,C12orf42:ENST00000548883.1:exon5:c.G559A:p.A187T,C12orf42:ENST00000548048.1:exon8:c.G358A:p.A120T,C12orf42:ENST00000378113.2:exon5:c.G559A:p.A187T,	12q23.2	.
12	103984723	C	G	GACCGAGTGC,GATCCTGCGC	exonic	STAB2	nonsynonymous SNV	STAB2:ENST00000388887.2:exon2:c.C130G:p.R44G,	12q23.3	.
12	106889922	C	T	ACACGGCTTC,CATCACGAAT	exonic	POLR3B	nonsynonymous SNV	POLR3B:ENST00000228347.4:exon24:c.C2803T:p.P935S,POLR3B:ENST00000539066.1:exon24:c.C2629T:p.P877S,	12q23.3	.
12	108685649	G	A	CTCATTCATA,AAGTCCTCTC	exonic	CMKLR1	nonsynonymous SNV	CMKLR1:ENST00000552995.1:exon3:c.C1085T:p.S362F,CMKLR1:ENST00000550402.1:exon4:c.C1091T:p.S364F,CMKLR1:ENST00000312143.7:exon3:c.C1091T:p.S364F,CMKLR1:ENST00000412676.1:exon3:c.C1091T:p.S364F,CMKLR1:ENST00000397688.2:exon3:c.C1085T:p.S362F,	12q23.3	.
12	108985354	G	A	GCTTTCGGGG,GACCCACTGG	exonic	TMEM119	nonsynonymous SNV	TMEM119:ENST00000392806.3:exon2:c.C806T:p.P269L,	12q23.3	.
12	109577260	T	C	TGTCTCGACT,TTCCTGGTTA	exonic	ACACB	nonsynonymous SNV	ACACB:ENST00000377854.5:exon1:c.T50C:p.F17S,ACACB:ENST00000338432.7:exon2:c.T50C:p.F17S,ACACB:ENST00000377848.3:exon1:c.T50C:p.F17S,	12q24.11	.
12	111884991	C	T	ACGTCCAGCT,CCCAAGGGGC	exonic	SH2B3	nonsynonymous SNV	SH2B3:ENST00000341259.2:exon2:c.C989T:p.S330F,SH2B3:ENST00000538307.1:exon4:c.C383T:p.S128F,	12q24.12	.
12	112654098	G	A	GCACTCACCG,GGGAGAGAGT	exonic	HECTD4	nonsynonymous SNV	HECTD4:ENST00000377560.5:exon47:c.C6947T:p.P2316L,HECTD4:ENST00000550722.1:exon48:c.C7025T:p.P2342L,HECTD4:ENST00000430131.2:exon47:c.C6197T:p.P2066L,	12q24.13	.
12	113382392	T	G	CGGAGGAACT,TGTGAACATT	exonic	OAS3	nonsynonymous SNV	OAS3:ENST00000548514.1:exon3:c.T572G:p.F191C,OAS3:ENST00000228928.7:exon3:c.T572G:p.F191C,	12q24.13	.
12	116413434	A	C	CACGAAGAGC,CATTGTATTT	exonic	MED13L	nonsynonymous SNV	MED13L:ENST00000281928.3:exon24:c.T5474G:p.V1825G,	12q24.21	.
12	117977611	G	T	GGGAGGGGGG,TGCTGGCGAG	exonic	KSR2	nonsynonymous SNV	KSR2:ENST00000339824.5:exon10:c.C1600A:p.P534T,KSR2:ENST00000302438.5:exon8:c.C691A:p.P231T,KSR2:ENST00000425217.1:exon10:c.C1513A:p.P505T,	12q24.22	.
12	118520108	A	G	CCACCATTCA,CCACGGGTGG	exonic	VSIG10	nonsynonymous SNV	VSIG10:ENST00000359236.5:exon3:c.T488C:p.V163A,	12q24.23	.
12	119961552	G	T	GTAACACCAT,AGGGCCAAGT	exonic	CCDC60	nonsynonymous SNV	CCDC60:ENST00000327554.2:exon11:c.G1158T:p.M386I,	12q24.23	.
12	119978449	C	T	CCATATGCCT,AAGAGGATTA	exonic	CCDC60	stopgain	CCDC60:ENST00000327554.2:exon14:c.C1582T:p.Q528X,	12q24.23	.
12	120622033	G	A	AAGGCCCTGC,GGAGGCTGCA	exonic	GCN1L1	nonsynonymous SNV	GCN1L1:ENST00000300648.6:exon4:c.C199T:p.R67C,	12q24.23	.
12	120650260	G	C	GTGATGCAGC,GCCGGTGATG	exonic	PXN	nonsynonymous SNV	PXN:ENST00000536957.1:exon12:c.C1627G:p.R543G,PXN:ENST00000267257.7:exon11:c.C1675G:p.R559G,PXN:ENST00000228307.7:exon12:c.C1633G:p.R545G,PXN:ENST00000397506.3:exon9:c.C1069G:p.R357G,PXN:ENST00000424649.2:exon11:c.C1531G:p.R511G,PXN:ENST00000458477.2:exon11:c.C11	12q24.23	.
12	121229358	G	A	CATATTAAGG,ACCTGTGGAA	exonic	SPPL3	nonsynonymous SNV	SPPL3:ENST00000353487.2:exon3:c.C104T:p.S35F,	12q24.31	UNQ1887;basechange=c.104C>A;AAchange=p.S35Y;tissue=lung
12	122257367	C	T	AGTGAGAGTT,CGAGTCTTCT	exonic	SETD1B	nonsynonymous SNV	SETD1B:ENST00000267197.5:exon11:c.C3347T:p.S1116F,SETD1B:ENST00000542440.1:exon12:c.C3347T:p.S1116F,SETD1B:ENST00000604567.1:exon11:c.C3476T:p.S1159F,	12q24.31	.
12	123687337	C	T	ATGGTATATA,TGACGGAAAC	exonic	MPHOSPH9	nonsynonymous SNV	MPHOSPH9:ENST00000541076.2:exon8:c.G1525A:p.V509I,MPHOSPH9:ENST00000302349.5:exon6:c.G1159A:p.V387I,MPHOSPH9:ENST00000302373.4:exon5:c.G1093A:p.V365I,MPHOSPH9:ENST00000606320.1:exon10:c.G1615A:p.V539I,MPHOSPH9:ENST00000392425.3:exon6:c.G1159A:p.V387I,MPHOSPH	12q24.31	.
12	123798212	G	A	GATACCATAG,AGAATCCAAG	exonic	SBNO1	nonsynonymous SNV	SBNO1:ENST00000602398.1:exon24:c.C3175T:p.P1059S,SBNO1:ENST00000267176.4:exon23:c.C3172T:p.P1058S,SBNO1:ENST00000602750.1:exon24:c.C3172T:p.P1058S,SBNO1:ENST00000420886.2:exon23:c.C3175T:p.P1059S,	12q24.31	.
12	125435290	G	A	ACAAACTCGG,GAGCTCTTTG	exonic	DHX37	nonsynonymous SNV	DHX37:ENST00000308736.2:exon22:c.C2929T:p.P977S,DHX37:ENST00000544745.1:exon19:c.C2290T:p.P764S,	12q24.31	.
12	125834405	G	A	TGACCTTACG,AAGATCTACC	exonic	TMEM132B	nonsynonymous SNV	TMEM132B:ENST00000299308.3:exon2:c.G460A:p.E154K,	12q24.31	.
12	129190541	C	A	GGAGAGCAAC,ATCTCCTGCT	exonic	TMEM132C	nonsynonymous SNV	TMEM132C:ENST00000315208.8:exon7:c.C1876A:p.H626N,TMEM132C:ENST00000537538.1:exon4:c.C1183A:p.H395N,TMEM132C:ENST00000435159.2:exon9:c.C3028A:p.H1010N,	12q24.32	.
12	130926806	C	T	AGTTCTGCTC,CCAGCGTGAG	exonic	RIMBP2	nonsynonymous SNV	RIMBP2:ENST00000261655.4:exon8:c.G1040A:p.G347E,RIMBP2:ENST00000536002.1:exon12:c.G764A:p.G255E,RIMBP2:ENST00000535703.1:exon10:c.G764A:p.G255E,	12q24.33	.
12	131456053	G	A	AGAGGAAAAG,GAGTCACGCT	exonic	GPR133	nonsynonymous SNV	GPR133:ENST00000535015.1:exon5:c.G334A:p.G112R,GPR133:ENST00000261654.5:exon4:c.G238A:p.G80R,GPR133:ENST00000542091.1:exon4:c.G238A:p.G80R,	12q24.33	.
12	133249230	G	A	AACCGGCAAG,GATATCGCTG	exonic	POLE	nonsynonymous SNV	POLE:ENST00000535270.1:exon14:c.C1588T:p.P530S,POLE:ENST00000320574.5:exon15:c.C1669T:p.P557S,POLE:ENST00000539006.1:exon9:c.C1009T:p.P337S,	12q24.33	.
12	133423647	C	T	AGAAACACTC,CCGCTGGAGA	exonic	CHFR	nonsynonymous SNV	CHFR:ENST00000315585.7:exon15:c.G1630A:p.G544R,CHFR:ENST00000432561.2:exon15:c.G1753A:p.G585R,CHFR:ENST00000541341.1:exon2:c.G34A:p.G12R,CHFR:ENST00000537522.1:exon7:c.G619A:p.G207R,CHFR:ENST00000443047.2:exon13:c.G1477A:p.G493R,CHFR:ENST00000266880.7:exon16:c.	12q24.33	.
12	133721098	C	T	AAGTCACTAA,TGCCTGGTCC	exonic	CTD-2140B24	nonsynonymous SNV	ZNF10:ENST00000538918.1:exon3:c.C20T:p.T7I,ZNF10:ENST00000540096.2:exon2:c.C20T:p.T7I,ZNF10:ENST00000426665.2:exon2:c.C20T:p.T7I,ZNF268:ENST00000416488.1:exon2:c.C20T:p.T7I,ZNF10:ENST00000540609.1:exon4:c.C20T:p.T7I,ZNF10:ENST00000543271.1:exon2:c.C20T:p.T7I,ZNF10:	12q24.33	.
12	133803715	G	C	CAGGGGTCAA,CTCGTCTCCA	exonic	ANHX	nonsynonymous SNV	ANHX:ENST00000545940.1:exon4:c.C525G:p.S175R,ANHX:ENST00000419717.1:exon5:c.C525G:p.S175R,	12q24.33	.
13	20567619	C	T	AAAAATTCCT,CAATTTTATT	exonic	ZMYM2	nonsynonymous SNV	ZMYM2:ENST00000382874.2:exon4:c.C407T:p.S136F,ZMYM2:ENST00000382869.3:exon4:c.C407T:p.S136F,ZMYM2:ENST00000382881.3:exon3:c.C407T:p.S136F,ZMYM2:ENST00000382871.2:exon4:c.C407T:p.S136F,	13q12.11	.
13	27256917	C	T	CCTCACCCAC,CTCCACCGGG	exonic	WASF3	nonsynonymous SNV	WASF3:ENST00000335327.5:exon9:c.C1157T:p.P386L,WASF3:ENST00000361042.4:exon9:c.C1148T:p.P383L,	13q12.13	.
13	28519436	C	T	CAGCTACATC,GATACCTCCA	exonic	ATP5EP2	stopgain	ATP5EP2:ENST00000381026.3:exon1:c.C40T:p.R14X,	13q12.2	.
13	28578233	C	T	AGCCCCAAAT,CATCTCTCTG	exonic	FLT3	nonsynonymous SNV	FLT3:ENST00000241453.7:exon24:c.G2938A:p.D980N,FLT3:ENST00000537084.1:exon23:c.G2815A:p.D939N,FLT3:ENST00000380982.4:exon24:c.G2947A:p.D983N,	13q12.2	.
13	28913395	C	T	TCATCTGGGT,CATTATAATT	exonic	FLT1	nonsynonymous SNV	FLT1:ENST00000282397.4:exon17:c.G2398A:p.D800N,FLT1:ENST00000540678.1:exon4:c.G52A:p.D18N,	13q12.3	.
13	32705849	G	A	GGAGCTAAAA,AATTACGGCA	exonic	FRY	nonsynonymous SNV	FRY:ENST00000380250.3:exon8:c.G757A:p.E253K,	13q13.1	FRY;basechange=c.757G>T;AAchange=p.E253*;tissue=large_intestine&FRY;basechange=c.757G>C;AAchange=p.E253Q;tissue=lung
13	46801719	G	A	AAAATGGATA,AACTCGTTTT	exonic	LRRC63	nonsynonymous SNV	LRRC63:ENST00000595396.1:exon2:c.G158A:p.R53K,LRRC63:ENST00000446175.1:exon3:c.G158A:p.R53K,LRRC63:ENST00000378805.3:exon3:c.G158A:p.R53K,	13q14.13	.
13	77847627	G	A	TGGCAAGCAT,GCTTCCATTG	exonic	MYCBP2	nonsynonymous SNV	MYCBP2:ENST00000357337.6:exon6:c.C811T:p.H271Y,MYCBP2:ENST00000544440.2:exon5:c.C811T:p.H271Y,MYCBP2:ENST00000407578.2:exon5:c.C925T:p.H309Y,	13q22.3	.
13	101714385	C	T	ACCTCCTCCT,TATGGTGTAC	exonic	NALCN	nonsynonymous SNV	NALCN:ENST00000251127.6:exon41:c.G4690A:p.E1564K,	13q33.1	.
13	101726878	C	T	TTAATATTCT,CCCATATTTC	exonic	NALCN	nonsynonymous SNV	NALCN:ENST00000251127.6:exon36:c.G4090A:p.E1364K,	13q33.1	.
13	102235614	G	A	GATCCGACTG,GGACTGGGGA	exonic	ITGBL1	nonsynonymous SNV	ITGBL1:ENST00000545560.2:exon5:c.G353A:p.G118E,ITGBL1:ENST00000376180.3:exon6:c.G776A:p.G259E,ITGBL1:ENST00000376162.3:exon5:c.G497A:p.G166E,	13q33.1	.
13	110831711	G	A	GGTGTGCCAG,AATGCCGGGA	exonic	COL4A1	nonsynonymous SNV	COL4A1:ENST00000375820.4:exon30:c.C2251T:p.P751S,	13q34	.
13	111077210	G	A	ACCCAAGGGC,ACGTGGTACG	exonic	COL4A2	nonsynonymous SNV	COL4A2:ENST00000360467.5:exon5:c.G310A:p.D104N,COL4A2:ENST00000400163.2:exon5:c.G310A:p.D104N,	13q34	.
14	20897249	G	A	CCCCACATGG,GGGAGGGTAG	exonic	KLHL33	nonsynonymous SNV	KLHL33:ENST00000344581.4:exon4:c.C1361T:p.P454L,	14q11.2	.
14	21559261	C	T	GACTGGGTGC,CGCGTAGTCG	exonic	ZNF219	nonsynonymous SNV	ZNF219:ENST00000421093.2:exon5:c.G1603A:p.G535S,ZNF219:ENST00000360947.3:exon5:c.G1603A:p.G535S,ZNF219:ENST00000451119.2:exon5:c.G1603A:p.G535S,	14q11.2	.
14	22409688	G	A	CCAATATCCT,GAGAAGGTCT	exonic	TRAV9-2	nonsynonymous SNV	TRAV9-2:ENST00000390441.2:exon2:c.G178A:p.G60R,	14q11.2	.
14	22409689	G	A	CAATATCCTG,AGAAGGTCTA	exonic	TRAV9-2	nonsynonymous SNV	TRAV9-2:ENST00000390441.2:exon2:c.G179A:p.G60E,	14q11.2	.
14	22471811	A	T	CGGACAGCAG,GGTTTCAGG	exonic	TRAV18	nonsynonymous SNV	TRAV18:ENST00000390446.3:exon2:c.A234T:p.R78S,	14q11.2	.
14	22471812	G	A	GGACAGCAGA,GTTTTCAGGC	exonic	TRAV18	nonsynonymous SNV	TRAV18:ENST00000390446.3:exon2:c.G235A:p.G79S,	14q11.2	.
14	23902316	G	A	GAGCCGTAGC,ATCCTTGAGG	exonic	MYH7	nonsynonymous SNV	MYH7:ENST00000355349.3:exon4:c.C322T:p.R108C,	14q11.2	.
14	24727515	C	A	TGGGGTGTGG,ATCCACCACC	exonic	TGM1	nonsynonymous SNV	TGM1:ENST00000206765.6:exon9:c.G1378T:p.A460S,TGM1:ENST00000544573.1:exon3:c.G52T:p.A18S,TGM1:ENST00000559136.1:exon5:c.G451T:p.A151S,	14q12	.

14	24845503	T	A	TTCCCAGTGA,CTGCAAAGAG	exonic	NFATC4	nonsynonymous SNV	NFATC4:ENST00000555590.1:exon10:c.T2099A:p.I700N,NFATC4:ENST00000250373.4:exon9:c.T2060A:p.I687N,NFATC4:ENST00000422617.3:exon9:c.T2024A:p.I675N,NFATC4:ENST00000554344.1:exon8:c.T1850A:p.I617N,NFATC4:ENST00000539237.2:exon10:c.T2156A:p.I719N,NFATC4:ENST00000413692.2	14q12	.
14	38678843	C	G	TCTACGTGAT,CTGCGCTATG	exonic	SSTR1	nonsynonymous SNV	SSTR1:ENST00000267377.2:exon3:c.C249G:p.I83M,	14q21.1	.
14	42356247	C	T	TTAATTTCCT,TACAGCGTTT	exonic	LRFN5	nonsynonymous SNV	LRFN5:ENST00000554120.1:exon3:c.C419T:p.S140F,LRFN5:ENST00000298119.4:exon3:c.C419T:p.S140F,LRFN5:ENST00000554171.1:exon5:c.C419T:p.S140F,	14q21.1	.
14	44975395	G	A	ATTTTAGCTG,GAATTTTTCT	exonic	FSCB	nonsynonymous SNV	FSCB:ENST00000340446.4:exon1:c.C796T:p.P266S,	14q21.2	.
14	55424317	G	A	TACCTTAAAG,GATTTACTCG	exonic	WDHD1	nonsynonymous SNV	WDHD1:ENST00000420358.2:exon21:c.C2369T:p.P790L,WDHD1:ENST00000360586.3:exon22:c.C2738T:p.P913L,WDHD1:ENST00000359167.4:exon10:c.C1292T:p.P431L,WDHD1:ENST00000421192.1:exon22:c.C2369T:p.P790L,	14q22.2	.
14	55819000	C	T	AGAGAGGATC,TTGCAAACAG	exonic	FBXO34	nonsynonymous SNV	FBXO34:ENST00000440021.1:exon2:c.C1892T:p.P631L,FBXO34:ENST00000313833.4:exon2:c.C1892T:p.P631L,FBXO34:ENST00000555280.1:exon1:c.C1490T:p.P497L,	14q22.3	.
14	59970688	G	T	AGCAAGATTT,CCCCTTTTGG	exonic	JKAMP	nonsynonymous SNV	JKAMP:ENST00000356057.5:exon7:c.G858T:p.L286F,JKAMP:ENST00000261247.9:exon7:c.G834T:p.L278F,JKAMP:ENST00000554271.1:exon7:c.G876T:p.L292F,JKAMP:ENST00000425728.2:exon7:c.G816T:p.L272F,	14q23.1	JKAMP_ENST00000356057;basechange=c.858G>T;AAchange=p.L286F;tissue=lung&JKAMP;basechange=c.864G>T;AAchange=p.L288F;tiss
14	62462960	G	T	ACAAGACAAT,ATTGGAGTCA	exonic	SYT16	nonsynonymous SNV	SYT16:ENST00000446982.2:exon2:c.G223T:p.D75Y,SYT16:ENST00000555409.1:exon1:c.G223T:p.D75Y,SYT16:ENST00000430451.2:exon1:c.G223T:p.D75Y,	14q23.2	.
14	63174329	G	A	TGGCATTTGG,ATTTGGGAGA	exonic	KCNH5	nonsynonymous SNV	KCNH5:ENST00000322893.7:exon11:c.C2864T:p.S955F,	14q23.2	.
14	65210179	G	A	CACCCTGGAG,ACAACCGGCG	exonic	PLEKHG3	nonsynonymous SNV	PLEKHG3:ENST00000484731.2:exon4:c.G1933A:p.D645N,PLEKHG3:ENST00000247226.7:exon15:c.G3250A:p.D1084N,PLEKHG3:ENST00000394691.1:exon17:c.G3418A:p.D1140N,PLEKHG3:ENST00000471182.2:exon4:c.G2017A:p.D673N,	14q23.3	.
14	74943172	G	A	GCAGGTGAGG,GAGCCACACT	exonic	NPC2	nonsynonymous SNV	NPC2:ENST00000434013.2:exon5:c.C565T:p.P189S,	14q24.3	.
14	77844329	C	T	GGAGACTGAA,CGGGGGTTCC	exonic	SAMD15	nonsynonymous SNV	SAMD15:ENST00000216471.4:exon1:c.C568T:p.P190S,	14q24.3	.
14	77950834	C	A	GTAGCTCTGC,TGCAGGTGGG	exonic	ISM2	nonsynonymous SNV	ISM2:ENST00000493585.1:exon3:c.G459T:p.Q153H,ISM2:ENST00000393684.3:exon4:c.G195T:p.Q65H,ISM2:ENST00000429906.1:exon2:c.G216T:p.Q72H,ISM2:ENST00000342219.4:exon3:c.G459T:p.Q153H,	14q24.3	.
14	77984455	G	A	GGGGTGGCAG,AAATCCAACC	exonic	SPTLC2	nonsynonymous SNV	SPTLC2:ENST00000216484.2:exon11:c.C1495T:p.P499S,	14q24.3	.
14	86088168	C	T	GGACGAATTC,CCATGAACCT	exonic	FLRT2	nonsynonymous SNV	FLRT2:ENST00000330753.4:exon2:c.C310T:p.P104S,FLRT2:ENST00000554746.1:exon2:c.C310T:p.P104S,	14q31.3	FLRT2;basechange=c.310C>T;AAchange=p.P104S;tissue=skin
14	92170952	C	A	GGCACAGGGG,ATTTTGTCAC	exonic	CATSPERB	nonsynonymous SNV	CATSPERB:ENST00000553329.1:exon6:c.G419T:p.C140F,CATSPERB:ENST00000256343.3:exon7:c.G560T:p.C187F,	14q32.12	.
14	94044270	G	A	AGTCCGTTTC,GAGTCCTTTG	exonic	UNC79	nonsynonymous SNV	UNC79:ENST00000553484.1:exon18:c.G2294A:p.R765Q,UNC79:ENST00000393151.2:exon18:c.G2294A:p.R765Q,UNC79:ENST00000256339.4:exon18:c.G1763A:p.R588Q,UNC79:ENST00000555664.1:exon18:c.G2294A:p.R765Q,	14q32.12	KIAA1409;basechange=c.1763G>A;AAchange=p.R588Q;tissue=haematopoietic_and_lymphoid_tissue&UNC79;basechange=c.2294G>A;AAch
14	94849489	C	T	GGCAGCATCT,CCTGGGGATC	exonic	SERPINA1	nonsynonymous SNV	SERPINA1:ENST00000557492.1:exon4:c.G86A:p.G29E,SERPINA1:ENST00000404814.4:exon3:c.G86A:p.G29E,SERPINA1:ENST00000393088.4:exon4:c.G86A:p.G29E,SERPINA1:ENST00000402629.1:exon2:c.G86A:p.G29E,SERPINA1:ENST00000556955.1:exon2:c.G86A:p.G29E,SERPINA1:ENST00000449399.3	14q32.13	.
14	94849490	C	T	GCAGCATCTC,CTGGGGATCC	exonic	SERPINA1	nonsynonymous SNV	SERPINA1:ENST00000557492.1:exon4:c.G85A:p.G29R,SERPINA1:ENST00000404814.4:exon3:c.G85A:p.G29R,SERPINA1:ENST00000393088.4:exon4:c.G85A:p.G29R,SERPINA1:ENST00000402629.1:exon2:c.G85A:p.G29R,SERPINA1:ENST00000556955.1:exon2:c.G85A:p.G29R,SERPINA1:ENST00000449399.3	14q32.13	SERPINA1;basechange=c.85G>A;AAchange=p.G29R;tissue=skin
14	96953250	G	C	ACGACTGGAG,AAGTGAAAAG	exonic	AK7	nonsynonymous SNV	AK7:ENST00000267584.4:exon17:c.G1990C:p.E664Q,	14q32.2	.
14	100406403	C	T	CACCTCATCT,CACGGGCGGG	exonic	EML1	nonsynonymous SNV	EML1:ENST00000334192.4:exon23:c.C2459T:p.S820F,EML1:ENST00000327921.9:exon23:c.C2366T:p.S789F,EML1:ENST00000262233.6:exon22:c.C2402T:p.S801F,	14q32.2	.
14	102798122	C	T	GCTCAGCTAC,TGGTGGCCCT	exonic	ZNF839	nonsynonymous SNV	ZNF839:ENST00000559098.1:exon3:c.C1163T:p.P388L,ZNF839:ENST00000442396.2:exon3:c.C1355T:p.P452L,ZNF839:ENST00000262236.5:exon3:c.C1007T:p.P336L,ZNF839:ENST00000558850.1:exon3:c.C1007T:p.P336L,ZNF839:ENST00000559185.1:exon3:c.C1007T:p.P336L,	14q32.31	.
14	103412002	C	T	CACTCACCAT,TCGGGTGACC	exonic	CDC42BPB	nonsynonymous SNV	CDC42BPB:ENST00000361246.2:exon29:c.G3808A:p.D1270N,	14q32.32	.
14	105397184	C	T	GGCTGTCCTC,CCAAAACCTG	exonic	PLD4	nonsynonymous SNV	PLD4:ENST00000540372.1:exon7:c.C844T:p.P282S,PLD4:ENST00000392593.4:exon7:c.C823T:p.P275S,	14q32.33	.
14	105407585	G	A	GATGATAAAG,AATCGTGGAA	exonic	AHNAK2	nonsynonymous SNV	AHNAK2:ENST00000333244.5:exon7:c.C14203T:p.P4735S,	14q32.33	.
14	106963340	G	T	CTGCCACCAA,AAGAGGATTC	exonic	IGHV1-45	nonsynonymous SNV	IGHV1-45:ENST00000390621.2:exon1:c.C27A:p.F9L,	14q32.33	.
15	22845825	C	T	ACAGCCAGGC,CTCCCAGTTT	exonic	TUBGCP5	nonsynonymous SNV	TUBGCP5:ENST00000283645.4:exon7:c.C686T:p.P229L,TUBGCP5:ENST00000453949.2:exon7:c.C686T:p.P229L,	15q11.2	.
15	35083461	T	A	ATGATGCTAT,GTAAGTTGTT	exonic	ACTC1	nonsynonymous SNV	ACTC1:ENST00000290378.4:exon6:c.A844T:p.N282Y,	15q14	.
15	35083462	G	T	TGATGCTATT,TAAGTTGTTT	exonic	ACTC1	stopgain	ACTC1:ENST00000290378.4:exon6:c.C843A:p.Y281X,	15q14	.
15	35086924	G	T	GACAGCGCGG,GCGCGTCATC	exonic	ACTC1	nonsynonymous SNV	ACTC1:ENST00000290378.4:exon2:c.C86A:p.P29H,	15q14	.
15	35149102	G	A	AGCAGGGATG,CTTCTGGAGT	exonic	AQR	nonsynonymous SNV	AQR:ENST00000156471.5:exon35:c.C4349T:p.A1450V,	15q14	.
15	40573893	C	T	GGCGCTCCCT,CAGCACCTCC	exonic	ANKRD63	nonsynonymous SNV	ANKRD63:ENST00000434396.1:exon1:c.G895A:p.E299K,	15q15.1	.
15	41857337	C	T	CTGTACAGTT,AGGTAGGCTC	exonic	TYRO3	stopgain	TYRO3:ENST00000559066.1:exon6:c.C646T:p.Q216X,TYRO3:ENST00000263798.3:exon6:c.C781T:p.Q261X,	15q15.1	.
15	42164573	G	A	TGCTGCTCAG,GACTTCGGGG	exonic	SPTBN5	nonsynonymous SNV	SPTBN5:ENST00000320955.6:exon27:c.C5092T:p.P1698S,	15q15.1	.
15	42743127	G	A	CACCTCGAAG,AAGCAGCGGA	exonic	ZNF106	nonsynonymous SNV	ZNF106:ENST00000263805.4:exon2:c.C1274T:p.S425F,ZNF106:ENST00000565948.1:exon2:c.C623T:p.S208F,	15q15.1	.
15	45396399	C	A	CATTGCCATC,TTGTCAGCCA	exonic	DUOX2	nonsynonymous SNV	DUOX2:ENST00000389039.6:exon19:c.G2499T:p.K833N,DUOX2:ENST00000603300.1:exon19:c.G2499T:p.K833N,	15q21.1	.
15	48703281	T	G	TTGGTTAAGT,CTTTCTTTTT	exonic	FBN1	nonsynonymous SNV	FBN1:ENST00000316623.5:exon66:c.A8522C:p.E2841A,	15q21.1	.
15	48788297	C	T	TATGGTTTAC,TTCACATGTT	exonic	FBN1	nonsynonymous SNV	FBN1:ENST00000316623.5:exon20:c.G2419A:p.D807N,	15q21.1	.
15	48807722	C	T	ACTGGCAGCA,CCCTAGAAGA	exonic	FBN1	nonsynonymous SNV	FBN1:ENST00000316623.5:exon12:c.G1330A:p.V444M,	15q21.1	.
15	50929721	G	A	AATATGAAAT,GGAATGCAGA	exonic	TRPM7	nonsynonymous SNV	TRPM7:ENST00000560955.1:exon7:c.C730T:p.H244Y,TRPM7:ENST00000313478.7:exon7:c.C730T:p.H244Y,	15q21.2	.
15	54542603	G	A	GAAATGCCAC,AAAAGTGTCA	exonic	UNC13C	nonsynonymous SNV	UNC13C:ENST00000260323.11:exon7:c.G3409A:p.E1137K,UNC13C:ENST00000545554.1:exon7:c.G3409A:p.E1137K,UNC13C:ENST00000537900.1:exon6:c.G3403A:p.E1135K,	15q21.3	UNC13C_ENST00000260323;basechange=c.3409G>A;AAchange=p.E1137K;tissue=endometrium&UNC13C;basechange=c.3409G>A;AAcha
15	57731611	G	A	GAAAGTTCTG,AAACCGAAGG	exonic	CGNL1	nonsynonymous SNV	CGNL1:ENST00000281282.5:exon2:c.G1414A:p.E472K,	15q21.3	.
15	58861013	G	A	ACATCAAAGC,AAAGATCAGA	exonic	LIPC	nonsynonymous SNV	LIPC:ENST00000299022.5:exon9:c.G1487A:p.R496Q,LIPC:ENST00000356113.6:exon11:c.G1487A:p.R496Q,LIPC:ENST00000433326.2:exon8:c.G1304A:p.R435Q,	15q21.3	.
15	65262509	A	C	GGCCAGATGA,AAATTTCCAA	exonic	SPG21	nonsynonymous SNV	SPG21:ENST00000416889.2:exon5:c.T423G:p.F141L,SPG21:ENST00000561078.1:exon5:c.T358G:p.F120V,SPG21:ENST00000204566.2:exon6:c.T504G:p.F168L,SPG21:ENST00000559199.1:exon4:c.T42G:p.F14L,SPG21:ENST00000433215.2:exon6:c.T504G:p.F168L,	15q22.31	.
15	65968839	C	T	TCTCTTAGTC,CTGAAGTAGC	exonic	DENND4A	nonsynonymous SNV	DENND4A:ENST00000431932.2:exon23:c.G4181A:p.G1394E,DENND4A:ENST00000443035.3:exon24:c.G4310A:p.G1437E,	15q22.31	.
15	66789879	G	T	GTGAGTAGCG,AGAACTGGCC	exonic	SNAPC5	nonsynonymous SNV	SNAPC5:ENST00000566658.1:exon1:c.C191A:p.S64Y,SNAPC5:ENST00000565465.1:exon1:c.C191A:p.S64Y,	15q22.31	.
15	67073869	G	A	AACAACCCCA,ATAGTGGCGG	exonic	SMAD6	nonsynonymous SNV	SMAD6:ENST00000338426.4:exon4:c.G704A:p.R235K,SMAD6:ENST00000288840.5:exon4:c.G1487A:p.R496K,	15q22.31	.
15	68120262	C	T	GGACCCCCTT,CGCCACCTCC	exonic	SKOR1	nonsynonymous SNV	SKOR1:ENST00000389002.1:exon3:c.C1964T:p.S655F,SKOR1:ENST00000554054.1:exon2:c.C2012T:p.S671F,SKOR1:ENST00000554240.1:exon2:c.C1979T:p.S660F,SKOR1:ENST00000380035.2:exon2:c.C2096T:p.S699F,	15q23	.
15	72338483	G	A	TTGAGGCTGT,GAAGAAAACC	exonic	MYO9A	nonsynonymous SNV	MYO9A:ENST00000424560.1:exon2:c.C422T:p.P141L,MYO9A:ENST00000564571.1:exon2:c.C422T:p.P141L,MYO9A:ENST00000444904.1:exon2:c.C422T:p.P141L,MYO9A:ENST00000356056.5:exon2:c.C422T:p.P141L,	15q23	.
15	74328028	A	C	CCTCCCATTT,TCCCAGTGGC	exonic	PML	nonsynonymous SNV	PML:ENST00000354026.6:exon7:c.A2082C:p.L694F,PML:ENST00000268059.6:exon8:c.A2226C:p.L742F,	15q24.1	.
15	74425739	C	T	GCCTCGCCTC,CGCGCTGCAG	exonic	ISLR2	nonsynonymous SNV	ISLR2:ENST00000445793.1:exon4:c.C644T:p.P215L,ISLR2:ENST00000435464.1:exon4:c.C644T:p.P215L,ISLR2:ENST00000565540.1:exon2:c.C644T:p.P215L,ISLR2:ENST00000565159.1:exon4:c.C644T:p.P215L,ISLR2:ENST00000453268.2:exon3:c.C644T:p.P215L,ISLR2:ENST00000361742.3:exon4:c.C644T:p.P2	15q24.1	.
15	74467780	G	A	CTCAAGACAT,GGCCCTGACC	exonic	ISLR	stopgain	ISLR:ENST00000395118.1:exon2:c.G581A:p.W194X,ISLR:ENST00000249842.3:exon2:c.G581A:p.W194X,	15q24.1	.
15	74559106	C	T	GTCTTCCACC,CTACCACTTT	exonic	CCDC33	nonsynonymous SNV	CCDC33:ENST00000398814.3:exon4:c.C407T:p.P136L,CCDC33:ENST00000321288.5:exon6:c.C1016T:p.P339L,	15q24.1	.
15	75305077	C	T	ATGTTTCTAC,AAGACTTCGA	exonic	SCAMP5	stopgain	SCAMP5:ENST00000564491.1:exon4:c.C67T:p.Q23X,SCAMP5:ENST00000361900.6:exon4:c.C67T:p.Q23X,SCAMP5:ENST00000566872.1:exon5:c.C67T:p.Q23X,SCAMP5:ENST00000568018.1:exon4:c.C67T:p.Q23X,SCAMP5:ENST00000562212.1:exon3:c.C67T:p.Q23X,SCAMP5:ENST00000425597.3:exon3:c.C67	15q24.2	.
15	79132943	G	A	GATGCTCTTG,AAGATACTCC	exonic	MORF4L1	nonsynonymous SNV	MORF4L1:ENST00000379535.4:exon2:c.G41A:p.G14E,	15q25.1	.
15	84694102	C	T	GGTGGTGTCT,CAAGAGCATG	exonic	ADAMTSL3	nonsynonymous SNV	ADAMTSL3:ENST00000286744.5:exon27:c.C4570T:p.P1524S,ADAMTSL3:ENST00000567476.1:exon27:c.C4570T:p.P1524S,	15q25.2	.
15	88679745	C	T	CAGTCCACAT,AGGAAGGGGT	exonic	NTRK3	nonsynonymous SNV	NTRK3:ENST00000542733.2:exon6:c.G424A:p.D142N,NTRK3:ENST00000558676.1:exon6:c.G718A:p.D240N,NTRK3:ENST00000557856.1:exon6:c.G718A:p.D240N,NTRK3:ENST00000394480.2:exon8:c.G718A:p.D240N,NTRK3:ENST00000355254.2:exon7:c.G718A:p.D240N,NTRK3:ENST00000357724.2:exon7:c.G	15q25.3	NTRK3_ENST00000317501;basechange=c.718G>C;AAchange=p.D240H;tissue=lung&NTRK3_ENST00000360948;basechange=c.718G>C;A
15	89401820	C	T	ACCAGGTACT,CATATTTTAG	exonic	ACAN	nonsynonymous SNV	ACAN:ENST00000439576.2:exon12:c.C6004T:p.P2002S,ACAN:ENST00000559004.1:exon12:c.C6004T:p.P2002S,ACAN:ENST00000352105.7:exon12:c.C6004T:p.P2002S,ACAN:ENST00000561243.1:exon11:c.C6004T:p.P2002S,	15q26.1	.
15	89402009	C	A	ACACACACCC,AGCTTTTTGA	exonic	ACAN	nonsynonymous SNV	ACAN:ENST00000439576.2:exon12:c.C6193A:p.Q2065K,ACAN:ENST00000559004.1:exon12:c.C6193A:p.Q2065K,ACAN:ENST00000352105.7:exon12:c.C6193A:p.Q2065K,ACAN:ENST00000561243.1:exon11:c.C6193A:p.Q2065K,	15q26.1	.
15	89421368	G	A	TCCAGGCCAT,GAACTTCCAG	exonic	HAPLN3	nonsynonymous SNV	HAPLN3:ENST00000562889.1:exon6:c.C1102T:p.H368Y,HAPLN3:ENST00000359595.3:exon5:c.C916T:p.H306Y,	15q26.1	.
15	89442906	G	A	GCCAGTCCTG,GGTCCTGGTA	exonic	MFGE8	nonsynonymous SNV	MFGE8:ENST00000542878.1:exon6:c.C875T:p.P292L,MFGE8:ENST00000566497.1:exon7:c.C1007T:p.P336L,MFGE8:ENST00000539437.1:exon8:c.C983T:p.P328L,MFGE8:ENST00000268150.8:exon7:c.C1007T:p.P336L,	15q26.1	.
15	90176952	C	T	ATTTCTGCCT,CAGGCGCCGC	exonic	KIF7	nonsynonymous SNV	KIF7:ENST00000394412.3:exon12:c.G2557A:p.E853K,	15q26.1	.
15	90190129	G	T	CCCAGCACAT,GGCGTGGGCA	exonic	KIF7	nonsynonymous SNV	KIF7:ENST00000394412.3:exon7:c.C1720A:p.H574N,	15q26.1	.
15	90349217	C	T	ACATTGCCCT,CATGTACTCG	exonic	ANPEP	nonsynonymous SNV	ANPEP:ENST00000300060.6:exon2:c.G598A:p.E200K,	15q26.1	.
15	91519989	C	G	CGATTTGGAT,TGAAGCTTTT	exonic	PRC1	nonsynonymous SNV	PRC1:ENST00000394249.3:exon9:c.G1120C:p.D374H,PRC1:ENST00000361188.5:exon9:c.G1120C:p.D374H,PRC1:ENST00000442656.2:exon8:c.G997C:p.D333H,PRC1:ENST00000361919.3:exon9:c.G1120C:p.D374H,	15q26.1	.
15	91769602	G	A	TGATGTCACC,AAGGCCATGA	exonic	SV2B	nonsynonymous SNV	SV2B:ENST00000557410.1:exon3:c.G109A:p.E37K,SV2B:ENST00000394232.1:exon2:c.G109A:p.E37K,SV2B:ENST00000330276.4:exon1:c.G109A:p.E37K,	15q26.1	.
15	101905175	G	A	ACAGACCAAT,GAACAGAATC	exonic	PCSK6	nonsynonymous SNV	PCSK6:ENST00000331826.7:exon12:c.C1435T:p.H479Y,	15q26.3	.
15	102284705	G	A	CAGGCCCCGG,GAGAGGGATC	exonic	RP11-89K11.	nonsynonymous SNV	RP11-89K11.1:ENST00000560292.2:exon1:c.C71T:p.P24L,RP11-89K11.1:ENST00000558592.1:exon2:c.C71T:p.P24L,	15q26.3	.
16	112887	G	A	CATCCCTAAC,GAGCGGCCCT	exonic	RHBDF1	nonsynonymous SNV	RHBDF1:ENST00000428730.1:exon5:c.C562T:p.R188C,	16p13.3	.
16	396431	G	A	AGGAAGGAGG,ATAGGTGTTT	exonic	AXIN1	nonsynonymous SNV	AXIN1:ENST00000354866.3:exon2:c.C595T:p.P199S,AXIN1:ENST00000262320.3:exon2:c.C595T:p.P199S,	16p13.3	.
16	815218	C	T	GCTGCTACCC,GGCTGGTGAG	exonic	MSLN	nonsynonymous SNV	MSLN:ENST00000563651.1:exon8:c.C619T:p.R207W,MSLN:ENST00000566549.1:exon8:c.C619T:p.R207W,MSLN:ENST00000382862.3:exon8:c.C619T:p.R207W,MSLN:ENST00000563941.1:exon9:c.C619T:p.R207W,MSLN:ENST00000545450.2:exon9:c.C619T:p.R207W,	16p13.3	.
16	1272654	C	T	CTCCTCACCT,CCTCCCGCGT	exonic	TPSG1	nonsynonymous SNV	TPSG1:ENST00000234798.4:exon4:c.G509A:p.G170E,	16p13.3	.
16	1272655	C	T	TCCTCACCTC,CTCCCGCGTA	exonic	TPSG1	nonsynonymous SNV	TPSG1:ENST00000234798.4:exon4:c.G508A:p.G170R,	16p13.3	.
16	1536040	T	C	CGGGAACTCT,TCCCGATGGC	exonic	PTX4	nonsynonymous SNV	PTX4:ENST00000447419.2:exon3:c.A1337G:p.K446R,PTX4:ENST00000293922.1:exon3:c.A1322G:p.K441R,	16p13.3	.
16	2043015	C	T	TACCAGAGCC,GCCCTTCACC	exonic	SYNGR3	nonsynonymous SNV	SYNGR3:ENST00000248121.2:exon4:c.C632T:p.P211L,	16p13.3	.
16	2215888	G	A	AGACCAGAAT,GAAACGACCT	exonic	TRAF7	nonsynonymous SNV	TRAF7:ENST00000564067.1:exon3:c.G112A:p.G38R,TRAF7:ENST00000567645.1:exon3:c.G90A:p.M30I,TRAF7:ENST00000326181.6:exon3:c.G90A:p.M30I,	16p13.3	.
16	2816993	C	T	GCCGGCGGCT,CATGATGGAT	exonic	SRRM2	nonsynonymous SNV	SRRM2:ENST00000301740.8:exon11:c.C6464T:p.S2155F,	16p13.3	.
16	3100427	G	A	CTTCCACCAG,ACAGCTACCC	exonic	MMP25	nonsynonymous SNV	MMP25:ENST00000336577.4:exon4:c.G541A:p.D181N,	16p13.3	.
16	3434891	G	A	GAACTACTAA,AATAGCCAGG	exonic	ZSCAN32	nonsynonymous SNV	ZSCAN32:ENST00000396846.3:exon5:c.C802T:p.L268F,ZSCAN32:ENST00000396852.4:exon6:c.C802T:p.L268F,ZSCAN32:ENST00000573327.1:exon6:c.C802T:p.L268F,ZSCAN32:ENST00000304926.3:exon5:c.C166T:p.L56F,ZSCAN32:ENST00000574940.1:exon5:c.C802T:p.L268F,ZSCAN32:ENST00000422427.	16p13.3	.
16	3640211	G	A	GTTCAGTTTG,ATGAAGATTT	exonic	SLX4	nonsynonymous SNV	SLX4:ENST00000294008.3:exon12:c.C3428T:p.S1143F,	16p13.3	SLX4;basechange=c.3428C>T;AAchange=p.S1143F;tissue=urinary_tract
16	3641069	A	T	AGTAGCTGCA,ATTCATAAAT	exonic	SLX4	nonsynonymous SNV	SLX4:ENST00000294008.3:exon12:c.T2570A:p.F857Y,	16p13.3	.
16	4256768	C	T	CCCTGATGTC,CTCCTCGGGC	exonic	SRL	nonsynonymous SNV	SRL:ENST00000572111.1:exon2:c.G1399A:p.G467R,	16p13.3	.
16	4391585	C	T	TGGCTCACCC,TGCCACCAGC	exonic;splicing	PAM16;COR	nonsynonymous SNV	PAM16:ENST00000575636.1:exon3:c.G187A:p.G63R,	16p13.3	.
16	4911059	G	A	TCTCCCCGAA,GCCTGCCAGC	exonic	UBN1	nonsynonymous SNV	UBN1:ENST00000545171.1:exon7:c.G1066A:p.G356S,UBN1:ENST00000396658.4:exon6:c.G1066A:p.G356S,UBN1:ENST00000590769.1:exon7:c.G1066A:p.G356S,UBN1:ENST00000262376.6:exon7:c.G1066A:p.G356S,	16p13.3	.
16	4911060	G	A	CTCCCCGAAG,CCTGCCAGCA	exonic	UBN1	nonsynonymous SNV	UBN1:ENST00000545171.1:exon7:c.G1067A:p.G356D,UBN1:ENST00000396658.4:exon6:c.G1067A:p.G356D,UBN1:ENST00000590769.1:exon7:c.G1067A:p.G356D,UBN1:ENST00000262376.6:exon7:c.G1067A:p.G356D,	16p13.3	.
16	9858558	G	A	CCCCTGAAAG,ACCTGTTGTC	exonic	GRIN2A	nonsynonymous SNV	GRIN2A:ENST00000330684.3:exon13:c.C2843T:p.S948F,GRIN2A:ENST00000396575.2:exon12:c.C2843T:p.S948F,GRIN2A:ENST00000404927.2:exon13:c.C2843T:p.S948F,GRIN2A:ENST00000535259.1:exon12:c.C2372T:p.S791F,GRIN2A:ENST00000396573.2:exon14:c.C2843T:p.S948F,GRIN2A:ENST00000562	16p13.2	.
16	16228250	C	T	TCGTCCCTGA,CCTGGGCTTA	exonic	ABCC1	nonsynonymous SNV	ABCC1:ENST00000399410.3:exon28:c.C4010T:p.T1337I,ABCC1:ENST00000346370.5:exon27:c.C3842T:p.T1281I,ABCC1:ENST00000349029.5:exon26:c.C3665T:p.T1222I,ABCC1:ENST00000351154.5:exon27:c.C3833T:p.T1278I,ABCC1:ENST00000399408.2:exon29:c.C4040T:p.T1347I,ABCC1:ENST00000345148	16p13.11	.
16	20497945	G	A	AAAATTCAAC,AGCCAAGCTT	exonic	ACSM2A	nonsynonymous SNV	ACSM2A:ENST00000575690.1:exon15:c.G1679A:p.R560Q,ACSM2A:ENST00000417235.2:exon13:c.G1442A:p.R481Q,ACSM2A:ENST00000536134.1:exon15:c.G995A:p.R332Q,ACSM2A:ENST00000573854.1:exon14:c.G1679A:p.R560Q,ACSM2A:ENST00000396104.2:exon13:c.G1679A:p.R560Q,ACSM2A:ENST00	16p12.3	ACSM2A;basechange=c.1679G>A;AAchange=p.R560Q;tissue=skin
16	20753066	T	A	CGCCATGGTG,GCGCAAACTG	exonic	THUMPD1	nonsynonymous SNV	THUMPD1:ENST00000431224.2:exon1:c.A254T:p.H85L,	16p12.3	.
16	21065803	C	A	CAGCTTCCCT,GCACCAGCTG	exonic	DNAH3	nonsynonymous SNV	DNAH3:ENST00000415178.1:exon28:c.G3977T:p.R1326L,DNAH3:ENST00000261383.3:exon28:c.G3977T:p.R1326L,	16p12.3	DNAH3;basechange=c.3977G>A;AAchange=p.R1326Q;tissue=endometrium&DNAH3_ENST00000261383;basechange=c.3977G>A;AAchange
16	21147682	C	A	AGTCATTCTC,TTCTCCTGCA	exonic	DNAH3	nonsynonymous SNV	DNAH3:ENST00000415178.1:exon6:c.G849T:p.K283N,DNAH3:ENST00000261383.3:exon6:c.G849T:p.K283N,	16p12.3	.
16	21728264	G	A	AGGCATCGTG,AGATACAAGG	exonic	OTOA	nonsynonymous SNV	OTOA:ENST00000388957.3:exon5:c.G553A:p.E185K,OTOA:ENST00000286149.4:exon14:c.G1567A:p.E523K,OTOA:ENST00000388956.4:exon10:c.G1288A:p.E430K,OTOA:ENST00000388958.3:exon14:c.G1525A:p.E509K,	16p12.2	.
16	23536653	C	A	CTTCAGTTCT,CATTCAGCAT	exonic	EARS2	nonsynonymous SNV	EARS2:ENST00000563232.1:exon8:c.G1400T:p.G467V,EARS2:ENST00000563459.1:exon8:c.G1400T:p.G467V,EARS2:ENST00000564501.1:exon8:c.G1400T:p.G467V,EARS2:ENST00000449606.1:exon8:c.G1400T:p.G467V,	16p12.2	.
16	28332016	C	T	GCGCCTGGGC,GCTCAAGCGG	exonic	SBK1	nonsynonymous SNV	SBK1:ENST00000341901.4:exon4:c.C1049T:p.P350L,	16p11.2	.
16	28846438	C	T	GCAGCCATCC,CAGGCCATCG	exonic	ATXN2L	nonsynonymous SNV	ATXN2L:ENST00000569318.1:exon1:c.C19T:p.P7S,	16p11.2	.
16	28997939	C	T	GTCTGGGGTC,GTCCTGGACT	exonic	LAT	nonsynonymous SNV	LAT:ENST00000354453.4:exon7:c.C359T:p.P120L,LAT:ENST00000566177.1:exon6:c.C386T:p.P129L,LAT:ENST00000360872.5:exon6:c.C389T:p.P130L,	16p11.2	.
16	29811297	G	A	AAGAGAGCCC,AGGCCCTGAG	exonic	KIF22	nonsynonymous SNV	KIF22:ENST00000160827.4:exon8:c.G1208A:p.R403Q,KIF22:ENST00000400751.5:exon8:c.G1004A:p.R335Q,KIF22:ENST00000569382.2:exon7:c.G1004A:p.R335Q,KIF22:ENST00000561482.1:exon8:c.G1004A:p.R335Q,	16p11.2	.
16	29937347	G	A	CGAGGCCTCC,CCGACATGCC	exonic	KCTD13	nonsynonymous SNV	KCTD13:ENST00000568000.1:exon1:c.C8T:p.A3V,KCTD13:ENST00000561540.1:exon1:c.C8T:p.A3V,KCTD13:ENST00000308768.5:exon1:c.C8T:p.A3V,	16p11.2	.
16	30020806	C	T	CACCTTGGCC,TGAGGATGCT	exonic	DOC2A	nonsynonymous SNV	DOC2A:ENST00000350119.4:exon3:c.G335A:p.R112K,DOC2A:ENST00000564944.1:exon4:c.G335A:p.R112K,DOC2A:ENST00000565273.1:exon3:c.G335A:p.R112K,DOC2A:ENST00000563378.1:exon4:c.G335A:p.R112K,DOC2A:ENST00000564979.1:exon3:c.G335A:p.R112K,	16p11.2	.
16	31150508	G	A	GACTGCATGC,GGGATCCCGA	exonic	PRSS36	nonsynonymous SNV	PRSS36:ENST00000418068.2:exon14:c.C2210T:p.P737L,PRSS36:ENST00000268281.4:exon15:c.C2519T:p.P840L,PRSS36:ENST00000569305.1:exon15:c.C2504T:p.P835L,	16p11.2	.
16	47462739	C	G	TCAGTCAGAT,AATAAATGCA	exonic	ITFG1	nonsynonymous SNV	ITFG1:ENST00000544001.2:exon6:c.G292C:p.D98H,ITFG1:ENST00000320640.6:exon6:c.G631C:p.D211H,	16q12.1	.
16	51171120	G	A	GCCGGCCAGG,GAGCATTGGG	exonic	SALL1	nonsynonymous SNV	SALL1:ENST00000251020.4:exon3:c.C3878T:p.P1293L,SALL1:ENST00000541611.1:exon2:c.C347T:p.P116L,SALL1:ENST00000440970.1:exon3:c.C3587T:p.P1196L,	16q12.1	.
16	56623868	C	T	GGACCCTTCC,CTCTGCCGCC	exonic	MT3	nonsynonymous SNV	MT3:ENST00000570176.1:exon2:c.C118T:p.P40S,	16q12.2	.
16	57161774	C	T	TAAGATGTTT,CAGCTCTGGG	exonic	CPNE2	nonsynonymous SNV	CPNE2:ENST00000565874.1:exon11:c.C1075T:p.P359S,CPNE2:ENST00000290776.8:exon12:c.C1075T:p.P359S,CPNE2:ENST00000537605.1:exon12:c.C769T:p.P257S,CPNE2:ENST00000535318.2:exon13:c.C1075T:p.P359S,	16q13	.
16	57685294	G	T	ATCCTTCCCT,ACCCCAGGGG	exonic	GPR56	nonsynonymous SNV	GPR56:ENST00000569101.1:exon3:c.G247T:p.D83Y,GPR56:ENST00000538815.1:exon4:c.G247T:p.D83Y,GPR56:ENST00000388812.4:exon3:c.G247T:p.D83Y,GPR56:ENST00000568909.1:exon3:c.G247T:p.D83Y,GPR56:ENST00000566123.1:exon4:c.G247T:p.D83Y,GPR56:ENST00000567702.1:exon4:c.G247T:p	16q21	GPR56;basechange=c.247G>T;AAchange=p.D83Y;tissue=urinary_tract
16	64981666	C	T	GCCTTCATAA,CGTAGATTTG	exonic	CDH11	nonsynonymous SNV	CDH11:ENST00000566827.1:exon12:c.G1853A:p.G618D,CDH11:ENST00000268603.4:exon13:c.G2231A:p.G744D,	16q21	.
16	64981667	C	T	CCTTCATAAC,GTAGATTTGA	exonic	CDH11	nonsynonymous SNV	CDH11:ENST00000566827.1:exon12:c.G1852A:p.G618S,CDH11:ENST00000268603.4:exon13:c.G2230A:p.G744S,	16q21	.
16	67228767	C	T	CTGCCCAAGC,TGCCCTAGCC	exonic	E2F4	nonsynonymous SNV	E2F4:ENST00000379378.3:exon6:c.C692T:p.P231L,	16q22.1	.
16	67327522	T	C	GGCTTGAAGG,TGGCTCTGGC	exonic	KCTD19	nonsynonymous SNV	KCTD19:ENST00000562841.1:exon1:c.A205G:p.T69A,KCTD19:ENST00000304372.5:exon12:c.A2143G:p.T715A,	16q22.1	.
16	67328904	C	T	GCAAGGGCTT,TGAGAGGGAT	exonic	KCTD19	nonsynonymous SNV	KCTD19:ENST00000304372.5:exon10:c.G1447A:p.E483K,	16q22.1	.
16	67338392	T	C	ACGCCAGTAG,TCAGAGATGC	exonic	KCTD19	nonsynonymous SNV	KCTD19:ENST00000567976.1:exon3:c.A368G:p.N123S,KCTD19:ENST00000304372.5:exon3:c.A383G:p.N128S,	16q22.1	.
16	67981997	G	A	CCGCCAGTTG,GGGTCCTCAG	exonic	SLC12A4	nonsynonymous SNV	SLC12A4:ENST00000316341.3:exon14:c.C1814T:p.P605L,SLC12A4:ENST00000572037.1:exon13:c.C1670T:p.P557L,SLC12A4:ENST00000338335.3:exon14:c.C1814T:p.P605L,SLC12A4:ENST00000422611.2:exon13:c.C1820T:p.P607L,SLC12A4:ENST00000537830.2:exon14:c.C1796T:p.P599L,SLC12A4:ENST000	16q22.1	.
16	68405316	C	T	TCGTCAGCTT,AGGTGGCGGG	exonic	SMPD3	nonsynonymous SNV	SMPD3:ENST00000563226.1:exon1:c.G769A:p.E257K,SMPD3:ENST00000568373.1:exon1:c.G769A:p.E257K,SMPD3:ENST00000219334.5:exon3:c.G769A:p.E257K,	16q22.1	.
16	69383547	C	T	CCCCACTGTC,GCTGAACCTG	exonic	RP11-343C2.	nonsynonymous SNV	TMED6:ENST00000288025.3:exon2:c.G221A:p.R74Q,RP11-343C2.7:ENST00000570293.1:exon2:c.G197A:p.R66Q,	16q22.1	.
16	69788780	C	T	AGGAAAGCCC,AGCATCCGCC	exonic	NOB1	nonsynonymous SNV	NOB1:ENST00000564620.1:exon1:c.G34A:p.G12R,NOB1:ENST00000569871.1:exon1:c.G34A:p.G12R,NOB1:ENST00000268802.5:exon1:c.G34A:p.G12R,	16q22.1	.
16	70778470	C	T	ACCTCCAGGT,CTTCAGGATC	exonic	VAC14	nonsynonymous SNV	VAC14:ENST00000261776.5:exon13:c.G1384A:p.D462N,	16q22.1	.
16	71718383	G	A	TTTCACCTTG,ATGCTTGCCG	exonic	PHLPP2	nonsynonymous SNV	PHLPP2:ENST00000360429.3:exon4:c.C731T:p.S244F,PHLPP2:ENST00000567016.1:exon5:c.C836T:p.S279F,PHLPP2:ENST00000393524.2:exon4:c.C731T:p.S244F,PHLPP2:ENST00000568954.1:exon5:c.C731T:p.S244F,PHLPP2:ENST00000356272.3:exon4:c.C731T:p.S244F,	16q22.2	.
16	71808431	G	C	CAGCAGCATT,CCCCTAAATA	exonic	AP1G1	nonsynonymous SNV	AP1G1:ENST00000567583.1:exon3:c.C266G:p.A89G,AP1G1:ENST00000450149.2:exon3:c.C266G:p.A89G,AP1G1:ENST00000433195.2:exon6:c.C335G:p.A112G,AP1G1:ENST00000565009.1:exon3:c.C266G:p.A89G,AP1G1:ENST00000563259.1:exon3:c.C266G:p.A89G,AP1G1:ENST00000423132.2:exon3:c.C266	16q22.2	.
16	72831821	G	A	GCTGCTGGTG,GTTCTGGCTG	exonic	ZFHX3	nonsynonymous SNV	ZFHX3:ENST00000397992.5:exon8:c.C2018T:p.P673L,ZFHX3:ENST00000268489.5:exon9:c.C4760T:p.P1587L,	16q22.2	.
16	72831822	G	A	CTGCTGGTGG,TTCTGGCTGA	exonic	ZFHX3	nonsynonymous SNV	ZFHX3:ENST00000397992.5:exon8:c.C2017T:p.P673S,ZFHX3:ENST00000268489.5:exon9:c.C4759T:p.P1587S,	16q22.2	.
16	72832314	G	A	CTGATCACAT,GTACTGAGAA	exonic	ZFHX3	nonsynonymous SNV	ZFHX3:ENST00000397992.5:exon8:c.C1525T:p.H509Y,ZFHX3:ENST00000268489.5:exon9:c.C4267T:p.H1423Y,	16q22.2	.
16	80666936	T	A	AGGGCATTGT,ATCCGAGGTC	exonic	CDYL2	nonsynonymous SNV	CDYL2:ENST00000566173.1:exon4:c.A817T:p.N273Y,CDYL2:ENST00000562812.1:exon4:c.A817T:p.N273Y,CDYL2:ENST00000563890.1:exon4:c.A817T:p.N273Y,CDYL2:ENST00000561616.1:exon2:c.A265T:p.N89Y,CDYL2:ENST00000570137.2:exon3:c.A814T:p.N272Y,	16q23.2	.
16	83999058	G	A	CATTGGCGCG,GGCTGTCAGC	exonic	OSGIN1	nonsynonymous SNV	OSGIN1:ENST00000393306.1:exon6:c.G880A:p.G294R,OSGIN1:ENST00000343939.2:exon7:c.G1129A:p.G377R,OSGIN1:ENST00000361711.3:exon6:c.G880A:p.G294R,	16q23.3	.
16	84189286	C	A	GCTTTGTGTC,TTGACCTTTC	exonic	DNAAF1	nonsynonymous SNV	DNAAF1:ENST00000378553.5:exon5:c.C673A:p.L225I,DNAAF1:ENST00000563093.1:exon5:c.C673A:p.L225I,DNAAF1:ENST00000567918.1:exon5:c.C673A:p.L225I,DNAAF1:ENST00000334315.5:exon5:c.C673A:p.L225I,	16q23.3	.
16	84203896	C	T	GGATGGAGAT,GAGAGCCAGA	exonic	DNAAF1	stopgain	DNAAF1:ENST00000378553.5:exon8:c.C1462T:p.R488X,	16q24.1	.
16	85116186	G	T	TGCTGGAGAG,AGGAGGAGAA	exonic	KIAA0513	stopgain	KIAA0513:ENST00000258180.3:exon10:c.G997T:p.E333X,KIAA0513:ENST00000566428.1:exon10:c.G997T:p.E333X,	16q24.1	.
16	88495605	C	T	CACGGGACAC,CAGCCTGCCC	exonic	ZNF469	nonsynonymous SNV	ZNF469:ENST00000437464.1:exon1:c.C1727T:p.P576L,ZNF469:ENST00000565624.1:exon1:c.C1727T:p.P576L,	16q24.2	.
16	88498067	G	A	TTTCCTGGAA,AACTGCACCC	exonic	ZNF469	nonsynonymous SNV	ZNF469:ENST00000437464.1:exon2:c.G4105A:p.E1369K,ZNF469:ENST00000565624.1:exon1:c.G4189A:p.E1397K,	16q24.2	.
16	88501179	C	T	CACCAGACTC,CCAGGGGGAC	exonic	ZNF469	nonsynonymous SNV	ZNF469:ENST00000437464.1:exon2:c.C7217T:p.P2406L,ZNF469:ENST00000565624.1:exon1:c.C7301T:p.P2434L,	16q24.2	.
16	88600820	G	C	ACTACCACGA,TGCACGGCCT	exonic	ZFPM1	nonsynonymous SNV	ZFPM1:ENST00000319555.3:exon10:c.G2454C:p.E818D,	16q24.2	.
16	88689665	C	T	GGCTCCGGCT,CTCCCGGTCG	exonic	ZC3H18	nonsynonymous SNV	ZC3H18:ENST00000301011.5:exon10:c.C1706T:p.S569F,ZC3H18:ENST00000452588.2:exon11:c.C1778T:p.S593F,	16q24.2	.
16	89650482	C	T	GTGGAGCTCT,CTTCCGGGCC	exonic	CPNE7	nonsynonymous SNV	CPNE7:ENST00000268720.5:exon6:c.C704T:p.S235F,CPNE7:ENST00000319518.8:exon4:c.C479T:p.S160F,	16q24.3	.
16	89916908	C	T	GAGGGCGTCC,CCGCAGCGTG	exonic	SPIRE2	nonsynonymous SNV	SPIRE2:ENST00000393062.2:exon3:c.C485T:p.P162L,SPIRE2:ENST00000378247.3:exon3:c.C485T:p.P162L,	16q24.3	.
17	1945086	C	T	CGCGCGTCCC,CTTCGGCCGT	exonic	DPH1	nonsynonymous SNV	DPH1:ENST00000263084.6:exon2:c.C94T:p.P32S,DPH1:ENST00000263083.6:exon12:c.C1267T:p.P423S,DPH1:ENST00000575162.1:exon1:c.C259T:p.P87S,DPH1:ENST00000570477.1:exon11:c.C1027T:p.P343S,	17p13.3	.
17	3336511	A	T	CCCATGATAA,TATCACCCAT	exonic	OR1E2	nonsynonymous SNV	OR1E2:ENST00000248384.1:exon1:c.T625A:p.F209I,	17p13.2	.
17	4540502	T	C	AGAATGACGT,GGCCTTGATC	exonic	ALOX15	nonsynonymous SNV	ALOX15:ENST00000545513.1:exon8:c.A925G:p.N309D,ALOX15:ENST00000574640.1:exon7:c.A742G:p.N248D,ALOX15:ENST00000570836.1:exon8:c.A859G:p.N287D,ALOX15:ENST00000293761.3:exon7:c.A859G:p.N287D,	17p13.2	.
17	4925700	C	T	GAGGCCCTGG,CGCCCTCAAG	exonic	KIF1C	nonsynonymous SNV	KIF1C:ENST00000320785.5:exon22:c.C2324T:p.A775V,	17p13.2	.
17	5291179	C	A	TGCTGAGGAG,TGAAGGCATT	exonic	NUP88	nonsynonymous SNV	NUP88:ENST00000573584.1:exon12:c.G1689T:p.Q563H,	17p13.2	.
17	7578263	G	A	CCTTCCACTC,GATAAGATGC	exonic	TP53	stopgain	TP53:ENST00000445888.2:exon6:c.C586T:p.R196X,TP53:ENST00000359597.4:exon5:c.C586T:p.R196X,TP53:ENST00000420246.2:exon6:c.C586T:p.R196X,TP53:ENST00000413465.2:exon5:c.C586T:p.R196X,TP53:ENST00000514944.1:exon5:c.C307T:p.R103X,TP53:ENST00000455263.2:exon6:c.C586T:p.R196	17p13.1	TP53;basechange=c.586C>A;AAchange=p.R196R;tissue=oesophagus,central_nervous_system&TP53_ENST00000545858;basechange=c.30
17	7849299	C	T	CTCACTTCAT,CACAGGTAAC	exonic	CNTROB	nonsynonymous SNV	CNTROB:ENST00000563694.1:exon13:c.C1988T:p.S663F,CNTROB:ENST00000380262.3:exon13:c.C1988T:p.S663F,CNTROB:ENST00000565740.1:exon13:c.C1988T:p.S663F,	17p13.1	.
17	8133655	C	T	ACCCGGGCTC,TGGAAGTAGT	exonic	CTC1	nonsynonymous SNV	CTC1:ENST00000580299.1:exon1:c.G61A:p.G21R,CTC1:ENST00000315684.8:exon17:c.G2890A:p.G964R,CTC1:ENST00000449476.2:exon17:c.G2785A:p.G929R,	17p13.1	.
17	10303758	C	A	CAGTCTCCAT,TTCAGCTCAC	exonic	MYH8	nonsynonymous SNV	MYH8:ENST00000403437.2:exon27:c.G3684T:p.K1228N,	17p13.1	.
17	10398521	C	A	CAGTGATGGC,TTCTTGGCCT	exonic	MYH1	nonsynonymous SNV	MYH1:ENST00000226207.5:exon36:c.G5283T:p.K1761N,	17p13.1	.
17	10408730	G	A	TTTATACTGG,TGTGGTCAAT	exonic	MYH1	nonsynonymous SNV	MYH1:ENST00000226207.5:exon20:c.C2273T:p.T758I,	17p13.1	MYH1;basechange=c.2273C>A;AAchange=p.T758N;tissue=lung
17	10432765	G	A	AGGTCCATGC,AAGTTTCTTT	exonic	MYH2	nonsynonymous SNV	MYH2:ENST00000245503.5:exon25:c.C3151T:p.R1051C,MYH2:ENST00000397183.2:exon25:c.C3151T:p.R1051C,	17p13.1	MYH2;basechange=c.3151C>T;AAchange=p.R1051C;tissue=prostate,endometrium
17	12859221	C	T	GTCAGAATAT,AAGATGTAAA	exonic	ARHGAP44	stopgain	ARHGAP44:ENST00000544416.2:exon14:c.C1174T:p.Q392X,ARHGAP44:ENST00000262444.9:exon14:c.C1174T:p.Q392X,ARHGAP44:ENST00000340825.3:exon14:c.C1174T:p.Q392X,ARHGAP44:ENST00000379672.5:exon14:c.C1174T:p.Q392X,	17p12	.
17	16001797	C	T	GGCTTTGAGT,CATAGGAAAC	exonic	NCOR1	nonsynonymous SNV	NCOR1:ENST00000395851.1:exon20:c.G2752A:p.D918N,NCOR1:ENST00000395848.1:exon18:c.G2425A:p.D809N,NCOR1:ENST00000268712.3:exon21:c.G2704A:p.D902N,	17p11.2	.
17	17700343	C	T	GAGCCCATCC,TTTCCGACAA	exonic	RAI1	nonsynonymous SNV	RAI1:ENST00000353383.1:exon3:c.C4081T:p.L1361F,RAI1:ENST00000261641.6:exon1:c.C4081T:p.L1361F,	17p11.2	.
17	17772735	G	A	GGACACGCGG,AGATGAGCTC	exonic	TOM1L2	nonsynonymous SNV	TOM1L2:ENST00000581396.1:exon7:c.C680T:p.S227F,TOM1L2:ENST00000318094.10:exon7:c.C695T:p.S232F,TOM1L2:ENST00000540946.1:exon6:c.C536T:p.S179F,TOM1L2:ENST00000395739.4:exon7:c.C695T:p.S232F,TOM1L2:ENST00000379504.3:exon8:c.C830T:p.S277F,TOM1L2:ENST00000535933.1:exo	17p11.2	.
17	19290194	C	T	CTTCCCAGCT,CAGAGATCCC	exonic	MFAP4	stopgain	MFAP4:ENST00000497081.2:exon1:c.G39A:p.W13X,	17p11.2	.
17	27896099	C	T	GCCTCTGCCT,CGCAGGTAGG	exonic	TP53I13	nonsynonymous SNV	TP53I13:ENST00000578749.1:exon2:c.C61T:p.P21S,TP53I13:ENST00000581411.2:exon1:c.C61T:p.P21S,TP53I13:ENST00000301057.7:exon2:c.C136T:p.P46S,TP53I13:ENST00000582829.2:exon3:c.C61T:p.P21S,TP53I13:ENST00000580183.2:exon3:c.C61T:p.P21S,TP53I13:ENST00000583940.1:exon3:c.C61T:p.	17q11.2	.
17	27903104	G	A	ACCCGGTAAA,GCCAGCAGGG	exonic	GIT1	nonsynonymous SNV	GIT1:ENST00000394869.3:exon16:c.C1684T:p.L562F,GIT1:ENST00000579937.1:exon15:c.C1657T:p.L553F,GIT1:ENST00000225394.3:exon15:c.C1657T:p.L553F,GIT1:ENST00000578266.1:exon1:c.C46T:p.L16F,GIT1:ENST00000581348.1:exon16:c.C1684T:p.L562F,	17q11.2	.
17	32906157	C	T	AGCGTGCATC,TTTGAAAAGG	exonic	C17orf102	nonsynonymous SNV	C17orf102:ENST00000357754.1:exon1:c.G143A:p.R48K,	17q12	.
17	33497155	G	A	TATGTCCATA,ACACTCGGAC	exonic	UNC45B	nonsynonymous SNV	UNC45B:ENST00000433649.1:exon12:c.G1570A:p.D524N,UNC45B:ENST00000268876.5:exon12:c.G1570A:p.D524N,UNC45B:ENST00000394570.2:exon12:c.G1570A:p.D524N,	17q12	.
17	36891496	G	A	GGGGGCACGG,AGCGCCGTTG	exonic	PCGF2	nonsynonymous SNV	PCGF2:ENST00000360797.2:exon11:c.C1015T:p.P339S,PCGF2:ENST00000581345.1:exon10:c.C1015T:p.P339S,PCGF2:ENST00000580830.1:exon12:c.C1015T:p.P339S,	17q12	.
17	38233171	C	T	GGCCAATGTT,CCTGAAAACC	exonic	THRA	nonsynonymous SNV	THRA:ENST00000578218.1:exon3:c.C101T:p.S34F,THRA:ENST00000585047.1:exon3:c.C101T:p.S34F,THRA:ENST00000264637.4:exon3:c.C101T:p.S34F,THRA:ENST00000546243.1:exon3:c.C101T:p.S34F,THRA:ENST00000584985.1:exon3:c.C101T:p.S34F,THRA:ENST00000450525.2:exon3:c.C101T:p.S34F,TH	17q21.1	.
17	40948184	G	A	ACAGAAAAAA,AAATTGAAGA	exonic	WNK4	nonsynonymous SNV	WNK4:ENST00000246914.5:exon17:c.G3475A:p.E1159K,	17q21.31	.
17	42266784	C	T	CCAAGGCCTG,CCAAAAGACA	exonic	TMUB2	nonsynonymous SNV	TMUB2:ENST00000589785.1:exon2:c.C370T:p.P124S,TMUB2:ENST00000587989.1:exon3:c.C430T:p.P144S,TMUB2:ENST00000538716.2:exon3:c.C430T:p.P144S,TMUB2:ENST00000446571.3:exon5:c.C259T:p.P87S,TMUB2:ENST00000589856.1:exon2:c.C370T:p.P124S,TMUB2:ENST00000319511.6:exon2:c.C3	17q21.31	.
17	42266785	C	T	CAAGGCCTGC,CAAAAGACAA	exonic	TMUB2	nonsynonymous SNV	TMUB2:ENST00000589785.1:exon2:c.C371T:p.P124L,TMUB2:ENST00000587989.1:exon3:c.C431T:p.P144L,TMUB2:ENST00000538716.2:exon3:c.C431T:p.P144L,TMUB2:ENST00000446571.3:exon5:c.C260T:p.P87L,TMUB2:ENST00000589856.1:exon2:c.C371T:p.P124L,TMUB2:ENST00000319511.6:exon2:c.C37	17q21.31	TMUB2;basechange=c.371C>A;AAchange=p.P124H;tissue=large_intestine&TMUB2_ENST00000538716;basechange=c.431C>A;AAchange=
17	45412629	C	A	TCATCAGGAA,TATTAACCAC	exonic	EFCAB13	nonsynonymous SNV	EFCAB13:ENST00000523285.1:exon4:c.C98A:p.T33N,EFCAB13:ENST00000331493.2:exon5:c.C98A:p.T33N,EFCAB13:ENST00000517484.1:exon5:c.C98A:p.T33N,EFCAB13:ENST00000519772.1:exon5:c.C98A:p.T33N,	17q21.32	.
17	45994167	A	T	GTCTAAGACT,ACAAGAAAGC	exonic	SP2	nonsynonymous SNV	SP2:ENST00000376741.4:exon3:c.A730T:p.N244Y,	17q21.32	.
17	46846539	G	A	TGCAAGAGGA,GGAGGACACA	exonic	TTLL6	nonsynonymous SNV	TTLL6:ENST00000433608.2:exon8:c.C1567T:p.L523F,TTLL6:ENST00000393382.3:exon15:c.C2488T:p.L830F,	17q21.32	.
17	47123306	G	A	TGGTCCCAAG,AGGAAGTGAA	exonic	IGF2BP1	nonsynonymous SNV	IGF2BP1:ENST00000431824.2:exon11:c.G1033A:p.E345K,IGF2BP1:ENST00000290341.3:exon13:c.G1450A:p.E484K,	17q21.32	.
17	48154342	C	T	TCTCAGGCTC,AGTACAGCAG	exonic	ITGA3	stopgain	ITGA3:ENST00000007722.7:exon15:c.C1927T:p.Q643X,ITGA3:ENST00000320031.8:exon15:c.C1927T:p.Q643X,	17q21.33	.
17	48191582	G	A	CGTGTGGTAG,GGTAAGAACA	exonic	SAMD14	nonsynonymous SNV	SAMD14:ENST00000503131.1:exon9:c.C995T:p.P332L,SAMD14:ENST00000285206.8:exon7:c.C947T:p.P316L,SAMD14:ENST00000330175.4:exon8:c.C911T:p.P304L,	17q21.33	.
17	48191583	G	A	GTGTGGTAGG,GTAAGAACAT	exonic	SAMD14	nonsynonymous SNV	SAMD14:ENST00000503131.1:exon9:c.C994T:p.P332S,SAMD14:ENST00000285206.8:exon7:c.C946T:p.P316S,SAMD14:ENST00000330175.4:exon8:c.C910T:p.P304S,	17q21.33	.
17	48452673	G	A	TCAACAAAGA,GAGACAGCCT	exonic	EME1	nonsynonymous SNV	EME1:ENST00000511711.1:exon2:c.G104A:p.R35K,EME1:ENST00000338165.4:exon2:c.G104A:p.R35K,EME1:ENST00000511519.2:exon2:c.G104A:p.R35K,EME1:ENST00000511648.2:exon1:c.G104A:p.R35K,EME1:ENST00000393271.2:exon2:c.G104A:p.R35K,	17q21.33	.
17	48603452	C	T	GAACGAGGCC,TCAGGGAATC	exonic	MYCBPAP	nonsynonymous SNV	MYCBPAP:ENST00000436259.2:exon14:c.C2011T:p.L671F,MYCBPAP:ENST00000323776.5:exon14:c.C2122T:p.L708F,	17q21.33	.
17	48755438	A	T	TTTCCTCCTC,TCTGATCCCC	exonic	ABCC3	nonsynonymous SNV	ABCC3:ENST00000505699.1:exon24:c.A3712T:p.I1238F,	17q21.33	.
17	56582908	A	C	GATGTTGGCC,TTCCCATGAA	exonic	MTMR4	nonsynonymous SNV	MTMR4:ENST00000579925.1:exon11:c.T1028G:p.M343R,MTMR4:ENST00000323456.5:exon11:c.T1028G:p.M343R,	17q22	.
17	56619240	C	T	CCTCCCATCC,CCAAGCTCCC	exonic	C17orf47	nonsynonymous SNV	C17orf47:ENST00000321691.3:exon2:c.G1649A:p.G550E,	17q22	.
17	57290448	G	T	CATTCAAATT,CCCTAAAGGT	exonic	SMG8	nonsynonymous SNV	SMG8:ENST00000300917.5:exon3:c.G2264T:p.C755F,SMG8:ENST00000543872.2:exon4:c.G2264T:p.C755F,	17q22	.
17	58133498	G	A	AAGCTGAATG,ATGGATCTGC	exonic	HEATR6	nonsynonymous SNV	HEATR6:ENST00000184956.6:exon13:c.C2180T:p.S727F,	17q23.1	.
17	61601499	G	A	TCCCACCCCA,GTCGGAAGTT	splicing	KCNH6(ENST		.	17q23.3	.
17	61601500	G	A	CCCACCCCAG,TCGGAAGTTC	exonic	KCNH6	nonsynonymous SNV	KCNH6:ENST00000580652.1:exon2:c.G77A:p.S26N,KCNH6:ENST00000314672.5:exon2:c.G77A:p.S26N,KCNH6:ENST00000583023.1:exon2:c.G77A:p.S26N,KCNH6:ENST00000456941.2:exon2:c.G77A:p.S26N,KCNH6:ENST00000581784.1:exon2:c.G77A:p.S26N,KCNH6:ENST00000583465.1:exon2:c.G77A:p.S26	17q23.3	.
17	62022743	C	T	GAGAGGTAGC,CAGACCCACG	exonic	SCN4A	nonsynonymous SNV	SCN4A:ENST00000578147.1:exon19:c.G3697A:p.G1233S,SCN4A:ENST00000435607.1:exon19:c.G3697A:p.G1233S,	17q23.3	SCN4A;basechange=c.3697G>T;AAchange=p.G1233C;tissue=lung
17	62265742	G	A	GTGCCCGGAC,GACTGTTGTG	exonic	TEX2	nonsynonymous SNV	TEX2:ENST00000258991.3:exon5:c.C2231T:p.P744L,TEX2:ENST00000584379.1:exon5:c.C2210T:p.P737L,TEX2:ENST00000583097.1:exon5:c.C2210T:p.P737L,	17q23.3	.
17	64125873	C	T	CCCCAAGATT,CAACTATTAA	exonic	CEP112	stopgain	CEP112:ENST00000583358.1:exon6:c.G633A:p.W211X,CEP112:ENST00000535342.2:exon6:c.G633A:p.W211X,CEP112:ENST00000537949.1:exon6:c.G633A:p.W211X,CEP112:ENST00000392769.2:exon6:c.G633A:p.W211X,	17q24.1	.
17	64224201	C	T	TTTCTCATCC,TCCTCGGGAC	exonic	APOH	nonsynonymous SNV	APOH:ENST00000577982.1:exon3:c.G178A:p.G60R,APOH:ENST00000205948.6:exon2:c.G178A:p.G60R,	17q24.2	.
17	66878852	G	A	ACTACTTCTT,GAGAAATTCT	exonic	ABCA8	nonsynonymous SNV	ABCA8:ENST00000586539.1:exon30:c.C3716T:p.P1239L,ABCA8:ENST00000430352.2:exon28:c.C3716T:p.P1239L,ABCA8:ENST00000269080.2:exon28:c.C3596T:p.P1199L,	17q24.2	.
17	67515535	C	T	GGTGGACTGT,CATTCACTGT	exonic	MAP2K6	nonsynonymous SNV	MAP2K6:ENST00000589647.1:exon5:c.C160T:p.P54S,MAP2K6:ENST00000588110.1:exon6:c.C337T:p.P113S,MAP2K6:ENST00000590474.1:exon5:c.C328T:p.P110S,MAP2K6:ENST00000589295.1:exon5:c.C160T:p.P54S,	17q24.3	.
17	72357930	C	T	GCCCCAGGAC,CAGGCTTGGA	exonic	BTBD17	nonsynonymous SNV	BTBD17:ENST00000375366.3:exon1:c.G29A:p.G10E,	17q25.1	.
17	72786345	G	A	CCAGGAGGAG,AAGATGACGA	exonic	TMEM104	nonsynonymous SNV	TMEM104:ENST00000335464.5:exon5:c.G256A:p.E86K,TMEM104:ENST00000417024.2:exon5:c.G295A:p.E99K,TMEM104:ENST00000582773.1:exon5:c.G256A:p.E86K,TMEM104:ENST00000578764.1:exon3:c.G52A:p.E18K,TMEM104:ENST00000582330.1:exon5:c.G256A:p.E86K,	17q25.1	.
17	73484096	T	G	ATCCTGGTCC,TGCACTGGGC	exonic	KIAA0195	nonsynonymous SNV	KIAA0195:ENST00000314256.7:exon6:c.T493G:p.L165V,KIAA0195:ENST00000375248.5:exon5:c.T523G:p.L175V,	17q25.1	.
17	73775164	G	A	GCCGGTAGAG,GAGCGCTTTT	exonic	H3F3B	nonsynonymous SNV	H3F3B:ENST00000591890.1:exon2:c.C92T:p.P31L,H3F3B:ENST00000592643.1:exon2:c.C92T:p.P31L,H3F3B:ENST00000586607.1:exon2:c.C92T:p.P31L,H3F3B:ENST00000587560.1:exon1:c.C92T:p.P31L,H3F3B:ENST00000586270.1:exon2:c.C92T:p.P31L,H3F3B:ENST00000254810.4:exon2:c.C92T:p.P31L,H3F3	17q25.1	.
17	73775165	G	A	CCGGTAGAGG,AGCGCTTTTC	exonic	H3F3B	nonsynonymous SNV	H3F3B:ENST00000591890.1:exon2:c.C91T:p.P31S,H3F3B:ENST00000592643.1:exon2:c.C91T:p.P31S,H3F3B:ENST00000586607.1:exon2:c.C91T:p.P31S,H3F3B:ENST00000587560.1:exon1:c.C91T:p.P31S,H3F3B:ENST00000586270.1:exon2:c.C91T:p.P31S,H3F3B:ENST00000254810.4:exon2:c.C91T:p.P31S,H3F	17q25.1	.
17	74004869	C	T	TTCTCCAGGT,CGGGTCCTTC	exonic	EVPL	nonsynonymous SNV	EVPL:ENST00000301607.3:exon22:c.G4417A:p.D1473N,EVPL:ENST00000586740.1:exon22:c.G4483A:p.D1495N,	17q25.1	EVPL;basechange=c.4417G>T;AAchange=p.D1473Y;tissue=large_intestine
17	74266463	G	A	AGGCGCCCTG,ATCCCGCCCT	exonic	UBALD2	stopgain	UBALD2:ENST00000327490.6:exon3:c.G372A:p.W124X,UBALD2:ENST00000587913.1:exon3:c.G192A:p.W64X,UBALD2:ENST00000589240.1:exon2:c.G192A:p.W64X,	17q25.1	.
17	74394996	G	A	TCGTTGGAGC,GATGTTGCAT	exonic	UBE2O	nonsynonymous SNV	UBE2O:ENST00000319380.7:exon10:c.C1705T:p.R569C,	17q25.1	.
17	75276860	G	T	GAGAACAAAT,ATCATCTGTC	exonic	SEPT9	nonsynonymous SNV	SEPT9:ENST00000589070.1:exon1:c.G3T:p.M1I,	17q25.2	.
17	76490902	C	T	TCGTAATGAT,CTGCGGGCAG	exonic	DNAH17	nonsynonymous SNV	DNAH17:ENST00000585328.1:exon40:c.G6028A:p.D2010N,DNAH17:ENST00000389840.5:exon40:c.G6001A:p.D2001N,	17q25.3	.
17	78897415	C	T	GCGCAGTACA,CCCTCACTCC	exonic	RPTOR	nonsynonymous SNV	RPTOR:ENST00000306801.3:exon23:c.C2750T:p.T917I,RPTOR:ENST00000576366.1:exon2:c.C230T:p.T77I,RPTOR:ENST00000544334.2:exon19:c.C2276T:p.T759I,	17q25.3	.
17	80047599	A	T	CTCCCAGGAC,AGCCTATGGC	exonic	FASN	stopgain	FASN:ENST00000306749.2:exon12:c.T1874A:p.L625X,	17q25.3	.
17	80159423	C	T	CCTGTGGACG,GCTCCAGCTC	exonic	CCDC57	nonsynonymous SNV	CCDC57:ENST00000392347.1:exon2:c.G398A:p.R133H,CCDC57:ENST00000389641.4:exon2:c.G398A:p.R133H,CCDC57:ENST00000392343.3:exon3:c.G398A:p.R133H,	17q25.3	.
18	908363	G	A	CCGGGGCGTG,GGTAAGAGTT	exonic	ADCYAP1	nonsynonymous SNV	ADCYAP1:ENST00000450565.3:exon4:c.G340A:p.G114S,ADCYAP1:ENST00000579794.1:exon3:c.G340A:p.G114S,	18p11.32	.
18	3879858	G	A	TGCCTGCGGG,GAAGGTGCTG	exonic	DLGAP1	nonsynonymous SNV	DLGAP1:ENST00000584874.1:exon1:c.C211T:p.P71S,DLGAP1:ENST00000315677.3:exon4:c.C211T:p.P71S,DLGAP1:ENST00000515196.2:exon3:c.C211T:p.P71S,DLGAP1:ENST00000581527.1:exon4:c.C211T:p.P71S,	18p11.31	.
18	10697879	C	T	CTTGTGCTGC,TAGAAATAAA	splicing	PIEZO2(ENS		.	18p11.22	.
18	10770244	C	T	TGAAAATACT,CAGGAATTTT	exonic	PIEZO2	nonsynonymous SNV	PIEZO2:ENST00000503781.3:exon19:c.G2773A:p.E925K,PIEZO2:ENST00000580640.1:exon21:c.G2848A:p.E950K,PIEZO2:ENST00000582913.1:exon20:c.G2815A:p.E939K,PIEZO2:ENST00000383408.2:exon6:c.G637A:p.E213K,PIEZO2:ENST00000302079.6:exon19:c.G2773A:p.E925K,	18p11.22	.
18	14763904	C	T	AAAGAAACAT,TGAGAAATTT	exonic	ANKRD30B	nonsynonymous SNV	ANKRD30B:ENST00000447268.2:exon7:c.C1040T:p.S347F,ANKRD30B:ENST00000580206.1:exon7:c.C1040T:p.S347F,ANKRD30B:ENST00000358984.4:exon7:c.C1040T:p.S347F,	18p11.21	.
18	18624092	C	T	GTACCAAAAT,TGCTAACTTC	exonic	ROCK1	nonsynonymous SNV	ROCK1:ENST00000399799.2:exon6:c.G646A:p.D216N,	18q11.1	ROCK1_ENST00000399799;basechange=c.646G>T;AAchange=p.D216Y;tissue=lung
18	19239192	G	A	CAGTTCAGTG,TTTTTCCAAT	exonic	ABHD3	nonsynonymous SNV	ABHD3:ENST00000289119.2:exon6:c.C781T:p.P261S,ABHD3:ENST00000578270.1:exon5:c.C196T:p.P66S,ABHD3:ENST00000580981.1:exon4:c.C622T:p.P208S,	18q11.2	.
18	21660827	C	T	GCTGGGTTTT,CTGGAGACCG	exonic	TTC39C	nonsynonymous SNV	TTC39C:ENST00000304621.6:exon5:c.C556T:p.P186S,TTC39C:ENST00000317571.3:exon5:c.C739T:p.P247S,	18q11.2	.
18	22805087	C	A	GCACACGTTG,ACTTGTAATT	exonic	ZNF521	nonsynonymous SNV	ZNF521:ENST00000538137.2:exon4:c.G2795T:p.C932F,ZNF521:ENST00000361524.3:exon4:c.G2795T:p.C932F,ZNF521:ENST00000584787.1:exon3:c.G2135T:p.C712F,ZNF521:ENST00000399425.2:exon4:c.G2795T:p.C932F,	18q11.2	.
18	31324323	C	A	CAGGGCAGAC,AGTGAGGACA	exonic	ASXL3	nonsynonymous SNV	ASXL3:ENST00000269197.5:exon12:c.C4511A:p.P1504Q,	18q12.1	.
18	32470367	C	T	GGGTTTCTTT,CTTGGCTCTC	exonic	DTNA	nonsynonymous SNV	DTNA:ENST00000598334.1:exon20:c.C2138T:p.S713F,	18q12.1	.
18	32706987	C	T	AGGGCTTCTT,CAGTGGCTCA	exonic	MAPRE2	nonsynonymous SNV	MAPRE2:ENST00000538170.2:exon4:c.C527T:p.S176F,MAPRE2:ENST00000589699.1:exon5:c.C557T:p.S186F,MAPRE2:ENST00000588910.1:exon5:c.C686T:p.S229F,MAPRE2:ENST00000300249.5:exon5:c.C686T:p.S229F,MAPRE2:ENST00000436190.2:exon6:c.C650T:p.S217F,MAPRE2:ENST00000591734.1:e	18q12.2	.
18	32707008	C	T	GCATCCAAAT,CGATAAAGAT	exonic	MAPRE2	nonsynonymous SNV	MAPRE2:ENST00000538170.2:exon4:c.C548T:p.S183F,MAPRE2:ENST00000589699.1:exon5:c.C578T:p.S193F,MAPRE2:ENST00000588910.1:exon5:c.C707T:p.S236F,MAPRE2:ENST00000300249.5:exon5:c.C707T:p.S236F,MAPRE2:ENST00000436190.2:exon6:c.C671T:p.S224F,MAPRE2:ENST00000591734.1:e	18q12.2	.
18	32707010	G	A	ATCCAAATCC,ATAAAGATTT	exonic	MAPRE2	nonsynonymous SNV	MAPRE2:ENST00000538170.2:exon4:c.G550A:p.D184N,MAPRE2:ENST00000589699.1:exon5:c.G580A:p.D194N,MAPRE2:ENST00000588910.1:exon5:c.G709A:p.D237N,MAPRE2:ENST00000300249.5:exon5:c.G709A:p.D237N,MAPRE2:ENST00000436190.2:exon6:c.G673A:p.D225N,MAPRE2:ENST00000591734.	18q12.2	.
18	42531514	C	T	AGAGCTGCCA,CCCCATCCGA	exonic	SETBP1	nonsynonymous SNV	SETBP1:ENST00000282030.5:exon4:c.C2209T:p.P737S,	18q12.3	.
18	42532162	T	A	TGACCTCCAG,TTCTGGCAGA	exonic	SETBP1	nonsynonymous SNV	SETBP1:ENST00000282030.5:exon4:c.T2857A:p.F953I,	18q12.3	.
18	43418839	A	T	CGTGAGGGTC,CGGCCACCTA	exonic	SIGLEC15	nonsynonymous SNV	SIGLEC15:ENST00000389474.3:exon4:c.A653T:p.H218L,SIGLEC15:ENST00000546268.1:exon4:c.A191T:p.H64L,	18q12.3	.
18	43418840	C	A	GTGAGGGTCA,GGCCACCTAG	exonic	SIGLEC15	nonsynonymous SNV	SIGLEC15:ENST00000389474.3:exon4:c.C654A:p.H218Q,SIGLEC15:ENST00000546268.1:exon4:c.C192A:p.H64Q,	18q12.3	.
18	43418841	G	A	TGAGGGTCAC,GCCACCTAGT	exonic	SIGLEC15	nonsynonymous SNV	SIGLEC15:ENST00000389474.3:exon4:c.G655A:p.G219S,SIGLEC15:ENST00000546268.1:exon4:c.G193A:p.G65S,	18q12.3	.
18	44100289	A	T	GACCTGGTCC,AGAAGCCCAT	exonic	LOXHD1	stopgain	LOXHD1:ENST00000452425.1:exon1:c.T11A:p.L4X,	18q21.1	.
18	47463700	C	T	CGAAGATGAC,CCTTCCCAGG	exonic	MYO5B	nonsynonymous SNV	MYO5B:ENST00000285039.7:exon15:c.G1820A:p.G607E,	18q21.1	.
18	48333153	C	A	GTCCCGAGCT,TTCTCGCAAA	exonic	MRO	stopgain	MRO:ENST00000590988.1:exon3:c.G103T:p.E35X,	18q21.2	.
18	53070893	G	A	GCAAGAGAGG,AATAACACTT	exonic	TCF4	nonsynonymous SNV	TCF4:ENST00000564228.1:exon1:c.C53T:p.S18F,TCF4:ENST00000544241.2:exon1:c.C53T:p.S18F,	18q21.2	.
18	59221707	A	G	CGTGCTGGCC,AGCTCTACGA	exonic	CDH20	nonsynonymous SNV	CDH20:ENST00000538374.1:exon12:c.A2185G:p.K729E,CDH20:ENST00000262717.4:exon12:c.A2185G:p.K729E,CDH20:ENST00000536675.2:exon11:c.A2185G:p.K729E,	18q21.33	.
18	61602447	C	T	CAACACCATT,TTTTTTATGG	exonic	SERPINB10	nonsynonymous SNV	SERPINB10:ENST00000238508.3:exon8:c.C1165T:p.L389F,	18q22.1	.
18	72245521	C	T	ATTTTCAATC,GTCTAGTCCC	exonic	CNDP1	nonsynonymous SNV	CNDP1:ENST00000358821.3:exon9:c.C1126T:p.R376C,CNDP1:ENST00000582365.1:exon8:c.C997T:p.R333C,	18q22.3	.
18	74637502	C	T	CTCCCTGCCT,TGTGTCAGGT	exonic	ZNF236	nonsynonymous SNV	ZNF236:ENST00000543926.2:exon22:c.C4013T:p.S1338F,ZNF236:ENST00000320610.9:exon22:c.C4019T:p.S1340F,ZNF236:ENST00000253159.8:exon22:c.C4013T:p.S1338F,	18q23	.
18	76756931	G	A	GCCCCCGCGA,ACGCGGCCGC	exonic	SALL3	nonsynonymous SNV	SALL3:ENST00000536229.3:exon3:c.G2897A:p.R966K,SALL3:ENST00000537592.2:exon3:c.G3512A:p.R1171K,SALL3:ENST00000575389.2:exon4:c.G3296A:p.R1099K,	18q23	.
18	77246368	C	T	GCGATGCCAC,CGACCCCAGC	exonic	NFATC1	nonsynonymous SNV	NFATC1:ENST00000545796.1:exon8:c.C797T:p.P266L,NFATC1:ENST00000427363.2:exon9:c.C2213T:p.P738L,NFATC1:ENST00000318065.5:exon9:c.C2174T:p.P725L,NFATC1:ENST00000329101.4:exon9:c.C2174T:p.P725L,NFATC1:ENST00000253506.5:exon9:c.C2213T:p.P738L,NFATC1:ENST00000397790.2:	18q23	.
18	77895176	C	T	CTGCCGGTTC,CCCTGGAGGC	exonic	ADNP2	nonsynonymous SNV	ADNP2:ENST00000262198.4:exon4:c.C1880T:p.P627L,	18q23	.
19	367152	C	A	GTGGCTGGGG,CTTCGCTTT	exonic	THEG	nonsynonymous SNV	THEG:ENST00000346878.2:exon6:c.G754T:p.A252S,THEG:ENST00000342640.4:exon7:c.G826T:p.A276S,	19p13.3	.
19	419020	G	T	TCGATCAGGT,GCTGATGCTC	exonic	SHC2	nonsynonymous SNV	SHC2:ENST00000264554.6:exon12:c.C1657A:p.H553N,	19p13.3	.
19	579603	G	T	GCTGGCTGAA,GGGGGCGTGG	exonic	BSG	nonsynonymous SNV	BSG:ENST00000353555.4:exon2:c.G171T:p.K57N,BSG:ENST00000573216.1:exon2:c.G147T:p.K49N,BSG:ENST00000333511.3:exon3:c.G519T:p.K173N,	19p13.3	.
19	831876	A	T	GGGCCAGCCT,GGGGGGCCTG	exonic	AZU1	stoploss	AZU1:ENST00000233997.2:exon5:c.A755T:p.X252L,	19p13.3	.
19	1020928	G	A	CCCGCGCGCG,GGGCCGGGCC	exonic	TMEM259	nonsynonymous SNV	TMEM259:ENST00000333175.5:exon1:c.C68T:p.P23L,TMEM259:ENST00000356663.3:exon1:c.C68T:p.P23L,	19p13.3	.
19	1229710	G	A	CCAGGCGCGC,GGCTGCGGGG	exonic	C19orf26	nonsynonymous SNV	C19orf26:ENST00000382477.2:exon10:c.C1646T:p.P549L,	19p13.3	.
19	1529430	C	T	AAAGAGGTTC,CTGCCTGGAA	exonic	PLK5	nonsynonymous SNV	PLK5:ENST00000334770.4:exon9:c.C431T:p.P144L,PLK5:ENST00000454744.2:exon10:c.C431T:p.P144L,PLK5:ENST00000588430.1:exon4:c.C431T:p.P144L,	19p13.3	.
19	1823639	G	A	AATGTGGACG,AGGGCGACTT	exonic	REXO1	nonsynonymous SNV	REXO1:ENST00000170168.4:exon4:c.G2162T:p.S721F,	19p13.3	.
19	2987920	C	T	GAGGCCTCCT,CAGTCCCCCT	exonic	TLE6	nonsynonymous SNV	TLE6:ENST00000452088.1:exon9:c.C281T:p.S94F,TLE6:ENST00000246112.4:exon10:c.C650T:p.S217F,	19p13.3	.
19	3527649	C	T	CTCCGGTCCC,CCGGAAACCC	exonic	FZR1	nonsynonymous SNV	FZR1:ENST00000441788.2:exon7:c.C491T:p.P164L,FZR1:ENST00000395095.3:exon6:c.C491T:p.P164L,FZR1:ENST00000591290.1:exon6:c.C491T:p.P164L,	19p13.3	.
19	3527726	C	T	CGACTTCTAC,TCAATCTGGT	exonic	FZR1	nonsynonymous SNV	FZR1:ENST00000441788.2:exon7:c.C568T:p.L190F,FZR1:ENST00000395095.3:exon6:c.C568T:p.L190F,FZR1:ENST00000591290.1:exon6:c.C568T:p.L190F,	19p13.3	.
19	5243965	G	A	GTCGCCGACG,AGGTGAAGGC	exonic	PTPRS	nonsynonymous SNV	PTPRS:ENST00000353284.2:exon8:c.C1478T:p.S493F,PTPRS:ENST00000348075.2:exon8:c.C1478T:p.S493F,PTPRS:ENST00000592099.1:exon7:c.C1478T:p.S493F,PTPRS:ENST00000372412.4:exon11:c.C1520T:p.S507F,PTPRS:ENST00000588012.1:exon7:c.C1478T:p.S493F,PTPRS:ENST00000357368.4:exon	19p13.3	.
19	5594188	C	T	GCTCGCGCTC,CCTGCGGGGA	exonic	SAFB2	nonsynonymous SNV	SAFB2:ENST00000252542.4:exon15:c.G1921A:p.E641K,	19p13.3	.
19	5790236	G	A	CTTAGCCTCA,GCTCAGCCAG	exonic	DUS3L	nonsynonymous SNV	DUS3L:ENST00000592491.1:exon1:c.C41T:p.P14L,DUS3L:ENST00000590110.1:exon2:c.C209T:p.P70L,DUS3L:ENST00000309061.7:exon2:c.C209T:p.P70L,	19p13.3	.
19	7584924	C	T	CCACAAGTGC,CGGTGTGCGC	exonic	ZNF358	nonsynonymous SNV	ZNF358:ENST00000597229.1:exon2:c.C796T:p.P266S,ZNF358:ENST00000394341.2:exon1:c.C796T:p.P266S,	19p13.2	.
19	7589886	C	T	GGGTATGGGA,CCAGGCGGGG	exonic	MCOLN1	nonsynonymous SNV	MCOLN1:ENST00000264079.6:exon2:c.C71T:p.T24I,MCOLN1:ENST00000601003.1:exon2:c.C71T:p.T24I,	19p13.2	.
19	7975127	C	T	GACCCTCCAA,CCTCCCTCTC	exonic	MAP2K7	nonsynonymous SNV	MAP2K7:ENST00000545011.1:exon4:c.C442T:p.P148S,	19p13.2	.
19	8153003	C	T	TTCGAAGCCT,CGATGACATT	exonic	FBN3	nonsynonymous SNV	FBN3:ENST00000270509.2:exon51:c.G6437A:p.G2146E,FBN3:ENST00000600128.1:exon52:c.G6437A:p.G2146E,FBN3:ENST00000601739.1:exon52:c.G6437A:p.G2146E,	19p13.2	.
19	8619594	G	A	TGCTTGAAGG,GTTTACAGAG	exonic	MYO1F	nonsynonymous SNV	MYO1F:ENST00000598321.1:exon3:c.C197T:p.P66L,MYO1F:ENST00000338257.8:exon3:c.C175T:p.P59S,	19p13.2	.
19	8841653	G	A	TTTCTGCGGG,AGAAGGTGCC	exonic	OR2Z1	nonsynonymous SNV	OR2Z1:ENST00000324060.2:exon1:c.G263A:p.G88E,	19p13.2	.
19	9049388	C	T	AGTCATATTT,TGTCTGTCCC	exonic	MUC16	nonsynonymous SNV	MUC16:ENST00000397910.4:exon5:c.G32243A:p.R10748K,	19p13.2	.
19	9058574	G	T	TGGATGTTCT,CTAGAGGGCA	exonic	MUC16	nonsynonymous SNV	MUC16:ENST00000397910.4:exon3:c.C28872A:p.S9624R,	19p13.2	.
19	9064972	G	A	ACAGAGGAAG,GGATTCATGT	exonic	MUC16	nonsynonymous SNV	MUC16:ENST00000397910.4:exon3:c.C22474T:p.P7492S,	19p13.2	.
19	9065917	G	A	TTTGTGGAAG,ATGCACAGCT	exonic	MUC16	nonsynonymous SNV	MUC16:ENST00000397910.4:exon3:c.C21529T:p.P7177S,	19p13.2	.
19	9066157	G	A	AGCAGGGAAG,GAGAGAGCTG	exonic	MUC16	nonsynonymous SNV	MUC16:ENST00000397910.4:exon3:c.C21289T:p.P7097S,	19p13.2	MUC16_ENST00000331986;basechange=c.8188C>T;AAchange=p.P2730S;tissue=kidney

19	9074029	C	T	GTATGTTGCC,TTGTAACGTG	exonic	MUC16	nonsynonymous SNV	MUC16:ENST00000397910.4:exon3:c.G13417A:p.G4473R,	19p13.2	MUC16_ENST00000397910;basechange=c.13417G>A;AAchange=p.G4473R;tissue=lung&MUC16_ENST00000331986;basechange=c.316G>
19	9267332	C	T	CTCAGAATTT,CTGTTTCTTC	exonic	ZNF317	nonsynonymous SNV	ZNF317:ENST00000360385.3:exon3:c.C70T:p.P24S,ZNF317:ENST00000591278.1:exon3:c.C70T:p.P24S,ZNF317:ENST00000247956.6:exon3:c.C70T:p.P24S,	19p13.2	.
19	9325251	C	T	GTCTTTGCTC,GTGCCTGGAT	exonic	OR7D4	nonsynonymous SNV	OR7D4:ENST00000308682.2:exon1:c.G263A:p.R88Q,	19p13.2	.
19	10502307	C	T	TCCTCTCCCT,CTTGGCCTCG	exonic	CDC37	nonsynonymous SNV	CDC37:ENST00000222005.2:exon8:c.G1057A:p.E353K,	19p13.2	.
19	10799337	C	T	GGCGGCTCCT,ATACCAAGGC	exonic	ILF3	nonsynonymous SNV	ILF3:ENST00000588657.1:exon18:c.C2546T:p.S849L,ILF3:ENST00000590261.1:exon18:c.C2534T:p.S845L,ILF3:ENST00000590869.1:exon3:c.C200T:p.S67L,ILF3:ENST00000318511.3:exon19:c.C2534T:p.S845L,ILF3:ENST00000449870.1:exon19:c.C2546T:p.S849L,	19p13.2	.
19	11132621	C	T	TTTAACGCAC,CTTTGCCATG	exonic	SMARCA4	nonsynonymous SNV	SMARCA4:ENST00000541122.2:exon20:c.C2837T:p.P946L,SMARCA4:ENST00000450717.3:exon18:c.C2837T:p.P946L,SMARCA4:ENST00000444061.3:exon20:c.C2837T:p.P946L,SMARCA4:ENST00000429416.3:exon20:c.C2837T:p.P946L,SMARCA4:ENST00000344626.4:exon19:c.C2837T:p.P946L,SMARCA4:EI	19p13.2	SMARCA4;basechange=c.2837C>T;AAchange=p.P946L;tissue=central_nervous_system
19	11462739	A	T	ACAGAAGCGC,GGAGGATGAG	exonic	CCDC159	nonsynonymous SNV	CCDC159:ENST00000458408.1:exon7:c.A497T:p.Q166L,CCDC159:ENST00000591260.1:exon4:c.A203T:p.Q68L,CCDC159:ENST00000588790.1:exon9:c.A497T:p.Q166L,	19p13.2	.
19	11464143	A	C	ACCAAGATGA,GCAGCAGGGT	exonic	CCDC159	nonsynonymous SNV	CCDC159:ENST00000458408.1:exon8:c.A584C:p.K195T,CCDC159:ENST00000591260.1:exon5:c.A290C:p.K97T,CCDC159:ENST00000588790.1:exon10:c.A584C:p.K195T,	19p13.2	.
19	11510960	G	A	TCAATGACCC,GGAGAGCCGG	exonic	RGL3	nonsynonymous SNV	RGL3:ENST00000380456.3:exon14:c.C1498T:p.R500W,RGL3:ENST00000393423.3:exon14:c.C1498T:p.R500W,	19p13.2	RGL3_ENST00000380456;basechange=c.1498C>T;AAchange=p.R500W;tissue=large_intestine&RGL3;basechange=c.790C>T;AAchange=p.I
19	11529199	C	T	GCCCCCAGTC,CCTTTCTACC	exonic	RGL3	nonsynonymous SNV	RGL3:ENST00000567431.1:exon1:c.G23A:p.G8E,	19p13.2	.
19	11725406	C	T	TTGCTGGATC,TTCCCAGAAG	exonic	ZNF627	nonsynonymous SNV	ZNF627:ENST00000361113.5:exon2:c.C68T:p.P23L,ZNF627:ENST00000588174.1:exon2:c.C68T:p.P23L,	19p13.2	.
19	11728345	G	A	GTGTGGGAAA,CCTTTGATTT	exonic	ZNF627	nonsynonymous SNV	ZNF627:ENST00000361113.5:exon4:c.G1027A:p.A343T,	19p13.2	.
19	12058104	A	T	GGAAACTTTC,GGAACCTGAC	exonic	ZNF700	nonsynonymous SNV	ZNF700:ENST00000482090.1:exon1:c.A118T:p.R40W,ZNF700:ENST00000254321.5:exon2:c.A172T:p.R58W,	19p13.2	.
19	12429854	T	C	GTGTGCCTTT,CATGTGTATC	exonic	ZNF563	nonsynonymous SNV	ZNF563:ENST00000293725.5:exon4:c.A985G:p.K329E,	19p13.2	.
19	12429855	C	A	TGTGCCTTTT,ATGTGTATCT	exonic	ZNF563	nonsynonymous SNV	ZNF563:ENST00000293725.5:exon4:c.G984T:p.M328I,	19p13.2	.
19	13015454	T	G	CGACTTCCCT,CCAGGACCGT	exonic	SYCE2	nonsynonymous SNV	SYCE2:ENST00000293695.7:exon3:c.A158C:p.E53A,	19p13.2	.
19	13246437	C	A	GAGGAGGCCC,ATCGTCGGAG	exonic	NACC1	nonsynonymous SNV	NACC1:ENST00000586171.1:exon2:c.C416A:p.P139Q,NACC1:ENST00000292431.4:exon2:c.C416A:p.P139Q,	19p13.2	.
19	14047188	G	A	ACCTTCGGAG,AGATGAGGTT	exonic	PODNL1	nonsynonymous SNV	PODNL1:ENST00000254320.3:exon2:c.C151T:p.P51S,PODNL1:ENST00000586075.1:exon2:c.C151T:p.P51S,PODNL1:ENST00000585607.1:exon3:c.C326T:p.S109F,PODNL1:ENST00000538371.2:exon3:c.C326T:p.S109F,PODNL1:ENST00000538517.2:exon3:c.C326T:p.S109F,PODNL1:ENST00000339560.5:exon	19p13.12	.
19	14167609	G	C	TTCCCGGAGA,ACTTCCTCTG	exonic	PALM3	nonsynonymous SNV	PALM3:ENST00000340790.4:exon3:c.C134G:p.S45C,	19p13.12	.
19	14863302	C	T	ACGCTCAGCC,CATGTAGGTG	exonic	EMR2	nonsynonymous SNV	EMR2:ENST00000594076.1:exon12:c.G1348A:p.G450R,EMR2:ENST00000392967.2:exon15:c.G1594A:p.G532R,EMR2:ENST00000596991.2:exon14:c.G1594A:p.G532R,EMR2:ENST00000353005.1:exon12:c.G1201A:p.G401R,EMR2:ENST00000595839.1:exon11:c.G1201A:p.G401R,EMR2:ENST00000594294.1:exc	19p13.12	.
19	14910315	T	G	AATATTCCAG,GAAGGAAATC	exonic	OR7C1	nonsynonymous SNV	OR7C1:ENST00000248073.2:exon1:c.A634C:p.T212P,	19p13.12	.
19	15296349	G	A	ATGGGCACAG,GATGGCTCGG	exonic	NOTCH3	nonsynonymous SNV	NOTCH3:ENST00000263388.2:exon13:c.C2093T:p.P698L,NOTCH3:ENST00000601011.1:exon13:c.C2090T:p.P697L,	19p13.12	NOTCH3;basechange=c.2093C>T;AAchange=p.P698L;tissue=lung&NOTCH3_ENST00000263388;basechange=c.2093C>T;AAchange=p.P69
19	15535084	G	A	CCGGACTCGG,GTGGGGGTTG	exonic	WIZ	nonsynonymous SNV	WIZ:ENST00000389282.4:exon9:c.C4706T:p.P1569L,WIZ:ENST00000599910.2:exon8:c.C2657T:p.P886L,WIZ:ENST00000599686.3:exon7:c.C2258T:p.P753L,WIZ:ENST00000545156.1:exon7:c.C2648T:p.P883L,WIZ:ENST00000263381.7:exon7:c.C2135T:p.P712L,	19p13.12	.
19	15904866	G	A	GCCATGCAGG,GCTAAACCAC	exonic	OR10H5	nonsynonymous SNV	OR10H5:ENST00000308940.8:exon1:c.G8A:p.G3E,	19p13.12	.
19	16590073	C	T	GCATCCATCT,CCTTTCTGGA	exonic	CALR3	nonsynonymous SNV	CALR3:ENST00000269881.3:exon9:c.G1024A:p.E342K,	19p13.11	.
19	16918543	C	T	ATGCATTCCC,CTCCCGAGGC	exonic	NWD1	nonsynonymous SNV	NWD1:ENST00000379808.3:exon16:c.C3883T:p.P1295S,NWD1:ENST00000524140.2:exon18:c.C3883T:p.P1295S,NWD1:ENST00000552788.1:exon16:c.C3883T:p.P1295S,NWD1:ENST00000523826.1:exon17:c.C3265T:p.P1089S,NWD1:ENST00000339803.6:exon14:c.C3478T:p.P1160S,NWD1:ENST00000549814	19p13.11	.
19	17291815	C	T	TCTCCAGTCC,TCAGTCGGCT	exonic	MYO9B	nonsynonymous SNV	MYO9B:ENST00000595618.1:exon15:c.C2299T:p.L767F,MYO9B:ENST00000397274.2:exon14:c.C2299T:p.L767F,MYO9B:ENST00000595641.1:exon14:c.C2299T:p.L767F,MYO9B:ENST00000594824.1:exon15:c.C2299T:p.L767F,	19p13.11	.
19	17831260	C	T	GGGATGGACC,AAAGCGAGTG	exonic	MAP1S	nonsynonymous SNV	MAP1S:ENST00000596637.1:exon1:c.C95T:p.P32L,MAP1S:ENST00000593593.1:exon1:c.C95T:p.P32L,MAP1S:ENST00000594365.1:exon1:c.C95T:p.P32L,	19p13.11	.
19	19030150	G	A	GGGGGCCGGA,GCGCCATTTC	exonic	COPE	nonsynonymous SNV	COPE:ENST00000593827.1:exon1:c.C8T:p.P3L,COPE:ENST00000262812.4:exon1:c.C8T:p.P3L,COPE:ENST00000349893.4:exon1:c.C8T:p.P3L,COPE:ENST00000600932.1:exon1:c.C8T:p.P3L,COPE:ENST00000351079.4:exon1:c.C8T:p.P3L,	19p13.11	.
19	19030151	G	A	GGGGCCGGAG,CGCCATTTCG	exonic	COPE	nonsynonymous SNV	COPE:ENST00000593827.1:exon1:c.C7T:p.P3S,COPE:ENST00000262812.4:exon1:c.C7T:p.P3S,COPE:ENST00000349893.4:exon1:c.C7T:p.P3S,COPE:ENST00000600932.1:exon1:c.C7T:p.P3S,COPE:ENST00000351079.4:exon1:c.C7T:p.P3S,	19p13.11	.
19	19335186	G	A	CGGAGCTATG,GAGGCGCAAC	exonic	NCAN	nonsynonymous SNV	NCAN:ENST00000252575.6:exon5:c.G722A:p.G241E,	19p13.11	.
19	19339381	G	A	AGAGCTTCTG,GAGGAGGTGG	exonic	NCAN	stopgain	NCAN:ENST00000538881.1:exon2:c.G1305A:p.W435X,NCAN:ENST00000252575.6:exon8:c.G2952A:p.W984X,	19p13.11	.
19	19339382	G	A	GAGCTTCTGG,AGGAGGTGGC	exonic	NCAN	nonsynonymous SNV	NCAN:ENST00000538881.1:exon2:c.G1306A:p.E436K,NCAN:ENST00000252575.6:exon8:c.G2953A:p.E985K,	19p13.11	.
19	19651996	G	A	AGCCCCGCTG,GGATGCGTGT	exonic	CILP2	nonsynonymous SNV	CILP2:ENST00000291495.5:exon4:c.G533A:p.G178E,CILP2:ENST00000586018.1:exon4:c.G551A:p.G184E,	19p13.11	.
19	20727507	C	T	AGTATGAATT,TCTTATGTCT	exonic	ZNF737	nonsynonymous SNV	ZNF737:ENST00000427401.4:exon4:c.G1502A:p.R501K,	19p12	ZNF737;basechange=c.1499G>T;AAchange=p.R500I;tissue=endometrium
19	21991115	A	G	GGATTGGGTA,AAGCTTTGCC	exonic	ZNF43	nonsynonymous SNV	ZNF43:ENST00000594012.1:exon7:c.T1706C:p.F569S,ZNF43:ENST00000595461.1:exon7:c.T1706C:p.F569S,ZNF43:ENST00000354959.4:exon4:c.T1724C:p.F575S,ZNF43:ENST00000598381.1:exon6:c.T1706C:p.F569S,	19p12	.
19	22940952	G	A	TCCCCAGTAT,AATTGCTTTA	exonic	ZNF99	nonsynonymous SNV	ZNF99:ENST00000397104.3:exon5:c.C1486T:p.H496Y,ZNF99:ENST00000596209.1:exon4:c.C1759T:p.H587Y,	19p12	.
19	31768538	G	A	GCGCTCAAAG,GTTAACAAAA	exonic	TSHZ3	nonsynonymous SNV	TSHZ3:ENST00000240587.4:exon2:c.C2161T:p.P721S,	19q12	.
19	32973073	C	T	AACCTGCCTC,CTACGTGGCC	exonic	DPY19L3	nonsynonymous SNV	DPY19L3:ENST00000392250.2:exon19:c.C2078T:p.P693L,DPY19L3:ENST00000342179.5:exon19:c.C2078T:p.P693L,	19q13.11	.
19	36224574	G	A	GCCCGCTGGG,AAGAAAGTCC	exonic	KMT2B	nonsynonymous SNV	KMT2B:ENST00000222270.7:exon29:c.G7036A:p.E2346K,KMT2B:ENST00000420124.1:exon29:c.G7036A:p.E2346K,	19q13.12	.
19	36277668	C	T	ACCCCCCGCC,CTGCCTCTGC	exonic	ARHGAP33	nonsynonymous SNV	ARHGAP33:ENST00000007510.4:exon20:c.C2296T:p.P766S,ARHGAP33:ENST00000378944.5:exon19:c.C1888T:p.P630S,	19q13.12	.
19	36594727	G	A	TAGCCCTGTG,AAGAGAGCGC	exonic	WDR62	nonsynonymous SNV	WDR62:ENST00000270301.7:exon30:c.G3982A:p.E1328K,WDR62:ENST00000401500.2:exon30:c.G3997A:p.E1333K,	19q13.12	.
19	37726899	C	T	ACTCCTAAGC,TCAAGTGATC	exonic	ZNF383	nonsynonymous SNV	ZNF383:ENST00000588250.1:exon3:c.C238T:p.L80F,ZNF383:ENST00000352998.3:exon4:c.C155T:p.P52L,ZNF383:ENST00000590503.1:exon5:c.C155T:p.P52L,ZNF383:ENST00000589413.1:exon7:c.C155T:p.P52L,ZNF383:ENST00000588371.1:exon4:c.C155T:p.P52L,	19q13.12	.
19	37880068	C	T	TTATGCCTTC,TTGTTGAACA	exonic	ZNF527	nonsynonymous SNV	ZNF527:ENST00000436120.2:exon5:c.C1117T:p.L373F,	19q13.12	.
19	37904557	A	G	ATTCGAGGGA,GGCTTTACCA	exonic	ZNF569	nonsynonymous SNV	ZNF569:ENST00000316950.6:exon6:c.T1003C:p.F335L,ZNF569:ENST00000392149.2:exon5:c.T1003C:p.F335L,ZNF569:ENST00000392150.2:exon2:c.T526C:p.F176L,	19q13.12	.
19	38379552	C	T	GACACTTCTT,CTCCAAAGTC	exonic	WDR87	nonsynonymous SNV	WDR87:ENST00000447313.2:exon6:c.G4759A:p.E1587K,WDR87:ENST00000303868.5:exon6:c.G4642A:p.E1548K,	19q13.13	.
19	38379939	C	T	TTGGTCATTT,CCTCTTTATT	exonic	WDR87	nonsynonymous SNV	WDR87:ENST00000447313.2:exon6:c.G4372A:p.E1458K,WDR87:ENST00000303868.5:exon6:c.G4255A:p.E1419K,	19q13.13	.
19	38609978	C	T	GACGCTCCTC,TTTCGGCCCC	exonic	SIPA1L3	nonsynonymous SNV	SIPA1L3:ENST00000222345.6:exon9:c.C2324T:p.P775L,	19q13.13	.
19	38865345	C	T	GCCTTGGGCT,CACCTCTCGG	exonic	PSMD8	nonsynonymous SNV	PSMD8:ENST00000215071.4:exon1:c.C104T:p.S35F,	19q13.2	.
19	38876339	G	C	TGCGGGTCTT,ATGGGCGCAG	exonic	GGN	nonsynonymous SNV	GGN:ENST00000334928.6:exon3:c.C1563G:p.I521M,GGN:ENST00000585737.1:exon3:c.C1314G:p.I438M,	19q13.2	.
19	38903662	C	T	GTTCCTCGGC,TTCAGGGTCA	exonic	RASGRP4	nonsynonymous SNV	RASGRP4:ENST00000589358.1:exon12:c.G1444A:p.G482S,RASGRP4:ENST00000293062.9:exon10:c.G1153A:p.G385S,RASGRP4:ENST00000589474.1:exon12:c.G1402A:p.G468S,RASGRP4:ENST00000587738.1:exon12:c.G1444A:p.G482S,RASGRP4:ENST00000587753.1:exon12:c.G1237A:p.G413S,RASGRP4	19q13.2	.
19	38943488	G	A	GGGAAGCCAC,GGGCTCGGGG	exonic	RYR1	nonsynonymous SNV	RYR1:ENST00000359596.3:exon13:c.G1274A:p.R425Q,RYR1:ENST00000355481.4:exon13:c.G1274A:p.R425Q,RYR1:ENST00000360985.3:exon13:c.G1274A:p.R425Q,	19q13.2	.
19	38946335	G	A	CATCATCCAG,AGAATCACAT	exonic	RYR1	nonsynonymous SNV	RYR1:ENST00000359596.3:exon16:c.G1735A:p.E579K,RYR1:ENST00000355481.4:exon16:c.G1735A:p.E579K,RYR1:ENST00000360985.3:exon16:c.G1735A:p.E579K,	19q13.2	.
19	38976402	C	T	CGCGCACCTG,CAGGCCCACT	exonic	RYR1	nonsynonymous SNV	RYR1:ENST00000359596.3:exon34:c.C5107T:p.P1703S,RYR1:ENST00000355481.4:exon34:c.C5107T:p.P1703S,RYR1:ENST00000360985.3:exon34:c.C5107T:p.P1703S,	19q13.2	.
19	38976403	C	T	GCGCACCTGC,AGGCCCACTG	exonic	RYR1	nonsynonymous SNV	RYR1:ENST00000359596.3:exon34:c.C5108T:p.P1703L,RYR1:ENST00000355481.4:exon34:c.C5108T:p.P1703L,RYR1:ENST00000360985.3:exon34:c.C5108T:p.P1703L,	19q13.2	.
19	38976798	G	A	GGGCTCCGTG,AGTTCCAGTT	exonic	RYR1	nonsynonymous SNV	RYR1:ENST00000359596.3:exon34:c.G5503A:p.E1835K,RYR1:ENST00000355481.4:exon34:c.G5503A:p.E1835K,RYR1:ENST00000360985.3:exon34:c.G5503A:p.E1835K,	19q13.2	RYR1;basechange=c.5503G>C;AAchange=p.E1835Q;tissue=lung
19	39068666	C	T	CAAGCCCAAC,CGCCGCCAGG	exonic	RYR1	nonsynonymous SNV	RYR1:ENST00000359596.3:exon98:c.C14281T:p.P4761S,RYR1:ENST00000355481.4:exon97:c.C14266T:p.P4756S,RYR1:ENST00000360985.3:exon97:c.C14266T:p.P4756S,	19q13.2	.
19	39068667	C	T	AAGCCCAACC,GCCGCCAGGG	exonic	RYR1	nonsynonymous SNV	RYR1:ENST00000359596.3:exon98:c.C14282T:p.P4761L,RYR1:ENST00000355481.4:exon97:c.C14267T:p.P4756L,RYR1:ENST00000360985.3:exon97:c.C14267T:p.P4756L,	19q13.2	.
19	39303513	G	A	GCCCGGTGCG,GGACATAGGC	exonic	LGALS4	nonsynonymous SNV	LGALS4:ENST00000307751.4:exon1:c.C14T:p.P5L,	19q13.2	.
19	39591829	G	A	CGGGTTGGAG,ATCTTTTCTA	exonic	PAPL	nonsynonymous SNV	PAPL:ENST00000331256.5:exon9:c.G955A:p.D319N,	19q13.2	.
19	40928849	G	A	CTCCTTGCCC,GCCCATCCTC	exonic	SERTAD1	nonsynonymous SNV	SERTAD1:ENST00000357949.4:exon2:c.C605T:p.P202L,	19q13.2	.
19	40928850	G	A	TCCTTGCCCG,CCCATCCTCA	exonic	SERTAD1	nonsynonymous SNV	SERTAD1:ENST00000357949.4:exon2:c.C604T:p.P202S,	19q13.2	.
19	41008059	G	A	ATTGGAGGTG,GGAAGATCAT	exonic	SPTBN4	nonsynonymous SNV	SPTBN4:ENST00000352632.3:exon9:c.G922A:p.G308R,SPTBN4:ENST00000338932.3:exon9:c.G922A:p.G308R,SPTBN4:ENST00000595535.1:exon9:c.G922A:p.G308R,SPTBN4:ENST00000344104.3:exon9:c.G922A:p.G308R,SPTBN4:ENST00000598249.1:exon9:c.G922A:p.G308R,	19q13.2	.
19	41231300	C	T	TTTGTGGTCT,CTTCCGAAAA	exonic	ITPKC	nonsynonymous SNV	ITPKC:ENST00000263370.2:exon2:c.C1211T:p.S404F,	19q13.2	.
19	41263330	T	C	GGCCAGGCCT,TGTCATCTTC	exonic	SNRPA	nonsynonymous SNV	SNRPA:ENST00000601545.1:exon2:c.T17C:p.F6S,SNRPA:ENST00000597353.1:exon3:c.T167C:p.F56S,SNRPA:ENST00000600456.1:exon2:c.T167C:p.F56S,SNRPA:ENST00000601253.1:exon3:c.T167C:p.F56S,SNRPA:ENST00000599362.1:exon3:c.T167C:p.F56S,SNRPA:ENST00000243563.3:exon2:c.T167C:p.F	19q13.2	.
19	41383898	C	T	TTCTTCTCCT,CTGCAGGGAG	exonic	CYP2A7	nonsynonymous SNV	CYP2A7:ENST00000291764.3:exon5:c.G679A:p.E227K,CYP2A7:ENST00000301146.4:exon6:c.G832A:p.E278K,	19q13.2	.
19	41622403	C	T	CACCTGGGAC,CAGGCGGGTG	exonic	CYP2F1	nonsynonymous SNV	CYP2F1:ENST00000532164.2:exon3:c.C215T:p.P72L,CYP2F1:ENST00000331105.2:exon3:c.C215T:p.P72L,	19q13.2	.
19	41798363	C	T	GCACCTTCCC,TTAGTGAGCG	exonic	HNRNPUL1	nonsynonymous SNV	HNRNPUL1:ENST00000263367.3:exon8:c.C946T:p.L316F,HNRNPUL1:ENST00000378215.4:exon7:c.C871T:p.L291F,HNRNPUL1:ENST00000595018.1:exon8:c.C913T:p.L305F,HNRNPUL1:ENST00000392006.3:exon8:c.C1213T:p.L405F,HNRNPUL1:ENST00000352456.3:exon8:c.C913T:p.L305F,HNRNPUL1:ENST(	19q13.2	.
19	42224868	C	T	CATCATTTCC,CCCCAGACTC	exonic	CEACAM5	nonsynonymous SNV	CEACAM5:ENST00000221992.6:exon8:c.C1798T:p.P600S,CEACAM5:ENST00000398599.4:exon8:c.C1795T:p.P599S,CEACAM5:ENST00000405816.1:exon8:c.C1798T:p.P600S,	19q13.2	.
19	42818423	G	A	CCCTTTTTCA,GACTGGGTGT	splicing	TMEM145(EN	.	.	19q13.2	.
19	42858840	G	A	GGCCAGCGAC,GGACAGGCCC	exonic	MEGF8	nonsynonymous SNV	MEGF8:ENST00000334370.4:exon22:c.G3911A:p.R1304Q,MEGF8:ENST00000251268.6:exon23:c.G4112A:p.R1371Q,	19q13.2	.
19	43093869	G	A	GCTGGAGATG,AGGGCTTGGG	exonic	CEACAM8	nonsynonymous SNV	CEACAM8:ENST00000244336.5:exon3:c.C443T:p.S148F,	19q13.2	.
19	43237004	G	A	ACATTCATAG,GTCCTGCAGT	exonic	PSG3	nonsynonymous SNV	PSG3:ENST00000595140.1:exon3:c.C641T:p.P214L,PSG3:ENST00000327495.5:exon3:c.C641T:p.P214L,	19q13.2	.
19	44471335	C	T	GGGAGAGCGA,CCTATAATTG	exonic	ZNF221	nonsynonymous SNV	ZNF221:ENST00000251269.5:exon6:c.C1681T:p.P561S,ZNF221:ENST00000587682.1:exon5:c.C1681T:p.P561S,ZNF221:ENST00000592350.1:exon6:c.C1681T:p.P561S,	19q13.31	.
19	44611004	C	T	TGGAGAGAAA,CGTTCAAATG	exonic	ZNF224	nonsynonymous SNV	ZNF224:ENST00000336976.6:exon6:c.C691T:p.P231S,	19q13.31	.
19	44611005	C	T	GGAGAGAAAC,GTTCAAATGT	exonic	ZNF224	nonsynonymous SNV	ZNF224:ENST00000336976.6:exon6:c.C692T:p.P231L,	19q13.31	.
19	45252100	C	T	GACCTGCGCA,CCGGCCCAAG	exonic	BCL3	nonsynonymous SNV	BCL3:ENST00000164227.5:exon1:c.C53T:p.T18I,	19q13.32	.
19	45262727	C	T	TCCCAGTCTC,CCCCAGGGAC	exonic	BCL3	nonsynonymous SNV	BCL3:ENST00000164227.5:exon9:c.C1220T:p.P407L,	19q13.32	.
19	45805684	C	T	GCTCCGATTC,CCTGGAGTGT	exonic	MARK4	nonsynonymous SNV	MARK4:ENST00000262891.4:exon17:c.C1975T:p.P659S,	19q13.32	.
19	45926535	G	A	TACCACTCCA,GAGGGACCTC	exonic	ERCC1	nonsynonymous SNV	ERCC1:ENST00000589165.1:exon2:c.C98T:p.P33L,ERCC1:ENST00000589214.1:exon2:c.C98T:p.P33L,ERCC1:ENST00000592023.1:exon2:c.C98T:p.P33L,ERCC1:ENST00000589381.1:exon1:c.C98T:p.P33L,ERCC1:ENST00000592083.1:exon3:c.C98T:p.P33L,ERCC1:ENST00000013807.5:exon1:c.C98T:p.P33L,EI	19q13.32	.
19	46271429	T	A	CGGCGTGGGG,AGCGGTTGCC	exonic	SIX5	nonsynonymous SNV	SIX5:ENST00000317578.6:exon1:c.A674T:p.Y225F,	19q13.32	.
19	46299289	G	A	CTTGTTGAAT,GGGGCTGGCA	exonic	RSPH6A	nonsynonymous SNV	RSPH6A:ENST00000597055.1:exon6:c.C1988T:p.P663L,	19q13.32	.
19	46387723	G	T	AGGGTCCTTG,GTGAGTGGCC	exonic	IRF2BP1	nonsynonymous SNV	IRF2BP1:ENST00000302165.3:exon1:c.C1310A:p.P437H,	19q13.32	.
19	47152748	G	A	GCCGGGGGAC,GAGACGCGTC	exonic	DACT3	nonsynonymous SNV	DACT3:ENST00000391916.2:exon4:c.C881T:p.P294L,DACT3:ENST00000300875.4:exon4:c.C206T:p.P69L,	19q13.32	.
19	47823566	C	T	CCGGGTGGTC,GGGAGGAGTA	exonic	C5AR1	nonsynonymous SNV	C5AR1:ENST00000355085.3:exon2:c.C532T:p.R178W,	19q13.32	.
19	48715140	G	A	TTCTCAGTAA,AACCTCATTG	exonic	CARD8	nonsynonymous SNV	CARD8:ENST00000519940.1:exon14:c.C1441T:p.L481F,CARD8:ENST00000447740.2:exon11:c.C1291T:p.L431F,CARD8:ENST00000520153.1:exon12:c.C1291T:p.L431F,CARD8:ENST00000391898.3:exon11:c.C1441T:p.L481F,CARD8:ENST00000521613.1:exon12:c.C1291T:p.L431F,CARD8:ENST00000359009.4:	19q13.33	.
19	48785688	G	A	GACCCTGCTG,ACCCAGCTCA	exonic	ZNF114	nonsynonymous SNV	ZNF114:ENST00000595408.1:exon5:c.G208A:p.D70N,ZNF114:ENST00000595607.1:exon5:c.G70A:p.D24N,ZNF114:ENST00000600687.1:exon5:c.G70A:p.D24N,ZNF114:ENST00000315849.1:exon4:c.G70A:p.D24N,ZNF114:ENST00000594024.1:exon5:c.G208A:p.D70N,	19q13.33	.
19	48806025	T	G	CGAGTGCACC,TGTCCATGCG	exonic	CCDC114	nonsynonymous SNV	CCDC114:ENST00000315396.7:exon10:c.A1055C:p.K352T,	19q13.33	.
19	49642950	G	A	GAGGAAGCTG,AGCTCCACGA	exonic	PPFIA3	nonsynonymous SNV	PPFIA3:ENST00000602655.1:exon16:c.G1879A:p.E627K,PPFIA3:ENST00000334186.4:exon17:c.G2102A:p.G701E,PPFIA3:ENST00000602351.1:exon16:c.G2102A:p.G701E,	19q13.33	.
19	49878199	G	T	CGTCCTAGAA,AGGGGACCGA	exonic	DKKL1	stopgain	DKKL1:ENST00000597546.1:exon4:c.G550T:p.E184X,DKKL1:ENST00000594268.1:exon2:c.G217T:p.E73X,DKKL1:ENST00000221498.2:exon5:c.G643T:p.E215X,DKKL1:ENST00000597873.1:exon5:c.G418T:p.E140X,	19q13.33	.
19	50215102	G	A	CGTGGCAGTG,ACAAGCACCA	exonic	CPT1C	nonsynonymous SNV	CPT1C:ENST00000598293.1:exon17:c.G1903A:p.D635N,CPT1C:ENST00000405931.2:exon17:c.G1870A:p.D624N,CPT1C:ENST00000323446.5:exon16:c.G1903A:p.D635N,CPT1C:ENST00000392518.4:exon17:c.G1903A:p.D635N,	19q13.33	.
19	50666447	G	A	CAAAGCCAGA,GCATGGCGGG	exonic	IZUMO2	nonsynonymous SNV	IZUMO2:ENST00000293405.3:exon1:c.C5T:p.P2L,	19q13.33	.
19	50666451	T	G	GCCAGAGGCA,GGCGGGGCCT	exonic	IZUMO2	nonsynonymous SNV	IZUMO2:ENST00000293405.3:exon1:c.A1C:p.M1L,	19q13.33	.
19	50983467	C	T	CTCCTTTGCC,CTGCGGTGAG	exonic	EMC10	nonsynonymous SNV	EMC10:ENST00000376918.3:exon4:c.C397T:p.P133S,EMC10:ENST00000598585.1:exon4:c.C397T:p.P133S,EMC10:ENST00000334976.6:exon4:c.C397T:p.P133S,	19q13.33	.
19	51451975	C	T	ATCCTCGCAC,TTTTCTGACT	exonic	KLK5	nonsynonymous SNV	KLK5:ENST00000391809.2:exon6:c.G647A:p.R216K,KLK5:ENST00000336334.3:exon5:c.G647A:p.R216K,KLK5:ENST00000593428.1:exon5:c.G647A:p.R216K,	19q13.33	.
19	51837080	G	A	TCCTCCAGAG,GACTTTGAGG	exonic	VSIG10L	nonsynonymous SNV	VSIG10L:ENST00000335624.4:exon9:c.C2539T:p.P847S,	19q13.41	.
19	52380537	G	A	TCACTTACCT,GACAGATTTG	exonic	ZNF577	nonsynonymous SNV	ZNF577:ENST00000412216.1:exon5:c.C281T:p.P94L,ZNF577:ENST00000301399.5:exon6:c.C281T:p.P94L,ZNF577:ENST00000420592.1:exon5:c.C281T:p.P94L,ZNF577:ENST00000451628.2:exon6:c.C281T:p.P94L,ZNF577:ENST00000458390.1:exon5:c.C281T:p.P94L,	19q13.41	.
19	52448168	T	A	AGAAACCCTA,GAATGCACTG	exonic	ZNF613	stopgain	ZNF613:ENST00000293471.6:exon6:c.T1032A:p.Y344X,ZNF613:ENST00000391794.4:exon6:c.T924A:p.Y308X,	19q13.41	.
19	52570279	C	T	TAAGGTTTCT,TCTGTAGTATGT	exonic	ZNF841	nonsynonymous SNV	ZNF841:ENST00000359973.2:exon1:c.G508A:p.E170K,ZNF841:ENST00000389534.4:exon7:c.G856A:p.E286K,ZNF841:ENST00000594295.1:exon6:c.G856A:p.E286K,ZNF841:ENST00000426391.2:exon5:c.G508A:p.E170K,	19q13.41	.
19	53652619	C	T	TGTCACCTGT,CCTAAAATGA	exonic	ZNF347	nonsynonymous SNV	ZNF347:ENST00000601469.2:exon3:c.G17A:p.G6E,ZNF347:ENST00000334197.7:exon3:c.G17A:p.G6E,ZNF347:ENST00000452676.2:exon3:c.G17A:p.G6E,ZNF347:ENST00000599096.1:exon3:c.G17A:p.G6E,	19q13.42	.
19	53652620	C	T	GTCACCTGTC,CTAAAATGAA	exonic	ZNF347	nonsynonymous SNV	ZNF347:ENST00000601469.2:exon3:c.G16A:p.G6R,ZNF347:ENST00000334197.7:exon3:c.G16A:p.G6R,ZNF347:ENST00000452676.2:exon3:c.G16A:p.G6R,ZNF347:ENST00000599096.1:exon3:c.G16A:p.G6R,	19q13.42	.
19	53855477	C	T	TAGGATAATT,ATACTGGAGA	exonic	ZNF845	nonsynonymous SNV	ZNF845:ENST00000595091.1:exon5:c.C1549T:p.H517Y,ZNF845:ENST00000458035.1:exon4:c.C1549T:p.H517Y,	19q13.42	.
19	54485975	G	A	CACGGTCACG,TCACCGGGCC	exonic	CACNG8	nonsynonymous SNV	CACNG8:ENST00000270458.2:exon4:c.G1150A:p.V384I,	19q13.42	.
19	54696029	C	T	ATCTGCTCTC,TTGTCCAGCT	exonic	TSEN34	nonsynonymous SNV	TSEN34:ENST00000302937.4:exon4:c.C550T:p.L184F,TSEN34:ENST00000456872.1:exon4:c.C559T:p.L187F,TSEN34:ENST00000429671.2:exon4:c.C550T:p.L184F,TSEN34:ENST00000396388.2:exon3:c.C550T:p.L184F,TSEN34:ENST00000396383.1:exon4:c.C550T:p.L184F,TSEN34:ENST00000455798.1:exon4:(	19q13.42	.
19	54754824	G	A	GAGAGGTAAG,AACGTGGTGG	exonic	LILRB5	nonsynonymous SNV	LILRB5:ENST00000450632.1:exon13:c.C1811T:p.S604F,	19q13.42	.
19	54875905	C	T	GACTTACCCT,CTGCGTGTGG	exonic	LAIR1	nonsynonymous SNV	LAIR1:ENST00000438193.1:exon2:c.G49A:p.E17K,LAIR1:ENST00000436513.1:exon2:c.G67A:p.E23K,LAIR1:ENST00000434277.2:exon2:c.G67A:p.E23K,LAIR1:ENST00000313038.6:exon2:c.G49A:p.E17K,LAIR1:ENST00000474878.1:exon2:c.G67A:p.E23K,LAIR1:ENST00000418556.1:exon2:c.G67A:p.E23K,LAIR1:	19q13.42	.
19	54927241	G	T	ATCAGAGCAG,TGAATATGTC	splicing	TTYH1(ENST	.	.	19q13.42	.
19	55085358	C	T	GACCCCCATC,TCACGGTCCT	exonic	LILRA2,LILRE	nonsynonymous SNV	LILRA2:ENST00000251376.3:exon1:c.C13T:p.L5F,LILRB1:ENST00000396321.2:exon1:c.C13T:p.L5F,LILRB1:ENST00000418536.2:exon1:c.C13T:p.L5F,LILRA2:ENST00000251377.3:exon2:c.C13T:p.L5F,LILRA2:ENST00000391737.1:exon1:c.C13T:p.L5F,LILRA2:ENST00000391738.3:exon1:c.C13T:p.L5F,LILRB1:EN	19q13.42	.
19	55331311	C	T	CCCCTCACGC,TCGTTGGACA	exonic	KIR3DL1	nonsynonymous SNV	KIR3DL1:ENST00000402254.2:exon4:c.C499T:p.L167F,KIR3DL1:ENST00000541392.1:exon4:c.C499T:p.L167F,KIR3DL1:ENST00000358178.4:exon3:c.C214T:p.L72F,KIR3DL1:ENST00000391728.4:exon4:c.C499T:p.L167F,KIR3DL1:ENST00000326542.7:exon4:c.C499T:p.L167F,KIR3DL1:ENST00000538269.1:exon	19q13.42	.
19	55445995	C	T	GAATTCAGCC,ACTGCTCCGG	exonic	NLRP7	stopgain	NLRP7:ENST00000328092.5:exon6:c.G2249A:p.W750X,NLRP7:ENST00000340844.2:exon7:c.G2333A:p.W778X,NLRP7:ENST00000590030.1:exon6:c.G2333A:p.W778X,NLRP7:ENST00000446217.1:exon9:c.G2417A:p.W806X,NLRP7:ENST00000588756.1:exon9:c.G2333A:p.W778X,NLRP7:ENST00000586379.1:ex	19q13.42	.
19	55828329	C	T	GCCGCTGGTT,TTTTCCTGGG	exonic	TMEM150B	nonsynonymous SNV	TMEM150B:ENST00000592731.1:exon5:c.G202A:p.E68K,	19q13.42	TMEM150B;basechange=c.330G>T;AAchange=p.K110N;tissue=endometrium
19	56718572	G	A	TCCGGAGGAA,GCCAGGCCAG	exonic	ZSCAN5C	nonsynonymous SNV	ZSCAN5C:ENST00000534327.1:exon3:c.G529A:p.G177S,ZSCAN5C:ENST00000376267.1:exon2:c.G529A:p.G177S,	19q13.43	.
19	57327631	G	A	TCAGTGAATG,CCCACTATGA	exonic	PEG3	nonsynonymous SNV	PEG3:ENST00000593695.1:exon9:c.C1801T:p.P601S,PEG3:ENST00000599577.1:exon9:c.C2179T:p.P727S,PEG3:ENST00000599534.1:exon7:c.C2179T:p.P727S,PEG3:ENST00000423103.2:exon9:c.C2179T:p.P727S,PEG3:ENST00000598410.1:exon9:c.C1807T:p.P603S,PEG3:ENST00000326441.9:exon10:c.C21	19q13.43	.
19	57904217	C	T	GGGGCCAGAC,ATTGGTGGCT	exonic	ZNF548	nonsynonymous SNV	ZNF548:ENST00000602086.1:exon2:c.C64T:p.H22Y,	19q13.43	.
19	58048770	C	T	AAACCTTATA,GTCTGTGGCC	exonic	ZNF549	nonsynonymous SNV	ZNF549:ENST00000376233.3:exon4:c.C398T:p.T133M,ZNF549:ENST00000240719.3:exon3:c.C359T:p.T120M,	19q13.43	ZNF549;basechange=c.359C>T;AAchange=p.T120M;tissue=endometrium&ZNF549_ENST00000376233;basechange=c.398C>T;AAchange=p
19	58102288	G	A	AATCAACACC,AAGAATTCAC	exonic	ZIK1	nonsynonymous SNV	ZIK1:ENST00000599456.1:exon3:c.G944A:p.R315Q,ZIK1:ENST00000536878.2:exon3:c.G1070A:p.R357Q,ZIK1:ENST00000597850.1:exon4:c.G1109A:p.R370Q,	19q13.43	.
19	58199475	G	A	GGTCACACTG,AGAAAGACCT	exonic	ZNF551	nonsynonymous SNV	ZNF551:ENST00000356715.4:exon3:c.G1784A:p.G595E,ZNF551:ENST00000282296.5:exon3:c.G1832A:p.G611E,	19q13.43	.
19	58216295	G	A	ATCAAGGAGA,CCCATTCCTC	exonic	ZNF154	nonsynonymous SNV	ZNF154:ENST00000451275.1:exon2:c.G86A:p.G29D,ZNF154:ENST00000426889.1:exon2:c.G86A:p.G29D,ZNF154:ENST00000512439.2:exon2:c.G86A:p.G29D,	19q13.43	.
19	58578256	G	A	CACTGGGAAT,GAGTTGTGAG	exonic	ZNF135	stopgain	ZNF135:ENST00000511556.1:exon5:c.G440A:p.W147X,ZNF135:ENST00000401053.4:exon4:c.G476A:p.W159X,ZNF135:ENST00000359978.6:exon4:c.G440A:p.W147X,ZNF135:ENST00000313434.5:exon5:c.G404A:p.W135X,ZNF135:ENST00000439855.2:exon5:c.G404A:p.W135X,ZNF135:ENST00000506786.1:exc	19q13.43	.
19	58868754	C	T	GGGCCCAGCC,CGTCCCGCCC	exonic	ZNF497	nonsynonymous SNV	ZNF497:ENST00000311044.3:exon3:c.G248A:p.G83E,ZNF497:ENST00000425453.3:exon2:c.G248A:p.G83E,	19q13.43	.
20	2312923	G	A	AGTGTGGAAG,ACTCTGCCAC	exonic	TGM3	nonsynonymous SNV	TGM3:ENST00000381458.5:exon10:c.G1609A:p.D537N,	20p13	.
20	2539224	C	T	CCCGGGGTCT,CCCGGAGGAA	exonic	TMC2	nonsynonymous SNV	TMC2:ENST00000358864.1:exon3:c.C205T:p.P69S,	20p13	.
20	2740538	G	A	GGCAGAAGAG,GGGTGCCAGG	exonic	EBF4	nonsynonymous SNV	EBF4:ENST00000463145.2:exon6:c.G457A:p.G153R,	20p13	.
20	2797675	C	T	CAACCCCATC,CACTGCCTGT	exonic	TMEM239	nonsynonymous SNV	TMEM239:ENST00000380593.4:exon3:c.C746T:p.P249L,TMEM239:ENST00000554164.1:exon3:c.C746T:p.P249L,	20p13	.
20	3212128	G	A	AGCTTCTGGC,GAAGGCGATA	exonic	SLC4A11	nonsynonymous SNV	SLC4A11:ENST00000380056.3:exon7:c.C844T:p.R282C,SLC4A11:ENST00000380059.3:exon8:c.C925T:p.R309C,SLC4A11:ENST00000474451.1:exon8:c.C739T:p.R247C,SLC4A11:ENST00000539553.2:exon8:c.C796T:p.R266C,	20p13	.
20	3652850	C	T	TAGCCACTGC,CCTGGCACAG	exonic	ADAM33	nonsynonymous SNV	ADAM33:ENST00000356518.2:exon14:c.G1528A:p.G510S,ADAM33:ENST00000379861.4:exon14:c.G1528A:p.G510S,ADAM33:ENST00000350009.2:exon14:c.G1528A:p.G510S,	20p13	.
20	3728981	G	A	GAGTGGCCGG,CCCCAGGTGG	exonic	HSPA12B	nonsynonymous SNV	HSPA12B:ENST00000254963.2:exon8:c.G793A:p.A265T,HSPA12B:ENST00000542646.1:exon6:c.G295A:p.A99T,HSPA12B:ENST00000399701.1:exon7:c.G535A:p.A179T,	20p13	.
20	4713232	G	A	CTAGAGAAGG,AAGTCTGAAA	exonic	PRNT	nonsynonymous SNV	PRNT:ENST00000423718.2:exon2:c.G91T:p.P31S,PRNT:ENST00000326539.2:exon2:c.G91T:p.P31S,PRNT:ENST00000418528.1:exon2:c.G91T:p.P31S,	20p13	.
20	5283110	G	A	GGAGATCCTG,CATAGCACAG	exonic	PROKR2	nonsynonymous SNV	PROKR2:ENST00000217270.3:exon2:c.C731T:p.A244V,PROKR2:ENST00000546004.1:exon3:c.C731T:p.A244V,	20p12.3	.
20	10273583	G	A	AGACATGAAG,AGGCTGAGAA	exonic	SNAP25	nonsynonymous SNV	SNAP25:ENST00000304886.2:exon5:c.G217A:p.E73K,	20p12.2	.
20	17948948	G	A	GACGCGCAAG,GAAAGAATGC	exonic	SNX5	nonsynonymous SNV	SNX5:ENST00000606557.1:exon1:c.C121T:p.P41S,	20p11.23	.
20	19677485	G	A	ACGTCATCAT,GGGATCACCT	exonic	SLC24A3	nonsynonymous SNV	SLC24A3:ENST00000328041.6:exon14:c.G1536A:p.M512I,	20p11.23	.
20	20033165	C	T	GGCACCGTTT,CATGGTGACC	exonic	CRNKL1	nonsynonymous SNV	CRNKL1:ENST00000496549.1:exon2:c.G175A:p.E59K,CRNKL1:ENST00000377327.4:exon2:c.G269A:p.G90E,CRNKL1:ENST00000377340.2:exon2:c.G305A:p.G102E,CRNKL1:ENST00000490910.1:exon2:c.G269A:p.G90E,	20p11.23	CRNKL1;basechange=c.305G>A;AAchange=p.G102E;tissue=skin
20	21689300	G	A	GAATGGGCTA,AGAAACCTGC	exonic	PAX1	nonsynonymous SNV	PAX1:ENST00000398485.2:exon3:c.G1021A:p.E341K,PAX1:ENST00000444366.2:exon2:c.G949A:p.E317K,	20p11.22	.
20	23017214	C	T	CCCCCACTCC,CTGCCAGCAG	exonic	SSTR4	nonsynonymous SNV	SSTR4:ENST00000255008.3:exon1:c.C1094T:p.P365L,	20p11.21	SSTR4;basechange=c.1094C>T;AAchange=p.P365L;tissue=lung
20	23065407	C	T	GATGGTGGTC,CAGAGACACA	exonic	CD93	nonsynonymous SNV	CD93:ENST00000246006.4:exon1:c.G1423A:p.G475R,	20p11.21	.
20	31572970	C	T	AACACCTCCT,CTTGGGGGAG	exonic	SUN5	nonsynonymous SNV	SUN5:ENST00000375523.3:exon11:c.G844A:p.E282K,SUN5:ENST00000356173.3:exon12:c.G919A:p.E307K,	20q11.21	.
20	31622086	A	C	CATGACCGTC,CTGGCAAGAG	exonic	BPIFB6	nonsynonymous SNV	BPIFB6:ENST00000349552.1:exon3:c.A292C:p.T98P,	20q11.21	.
20	31624305	C	T	ATGTCCGCAC,AGCCACCACA	exonic	BPIFB6	nonsynonymous SNV	BPIFB6:ENST00000349552.1:exon7:c.C632T:p.P211L,	20q11.21	.
20	32369173	C	T	TCACAACTTC,CCTGCCCACA	exonic	ZNF341	nonsynonymous SNV	ZNF341:ENST00000375200.1:exon11:c.C1699T:p.P567S,ZNF341:ENST00000342427.2:exon11:c.C1678T:p.P560S,	20q11.22	.
20	33345301	G	A	CTGCTGCAAG,GAGTTGGGAC	exonic	NCOA6	nonsynonymous SNV	NCOA6:ENST00000374796.2:exon8:c.C1250T:p.P417L,NCOA6:ENST00000359003.2:exon7:c.C1250T:p.P417L,	20q11.22	.
20	33572663	C	T	CCAGGGCACC,TTGAGGATCA	exonic	MYH7B	nonsynonymous SNV	MYH7B:ENST00000262873.7:exon10:c.C781T:p.L261F,	20q11.22	.
20	33578863	G	A	GGTCCTGGAG,GGATCCGGAT	exonic	MYH7B	nonsynonymous SNV	MYH7B:ENST00000262873.7:exon22:c.G2248A:p.G750R,	20q11.22	.
20	34091031	C	T	CAGCCAGATC,ATGACCTGGA	exonic	CEP250	nonsynonymous SNV	CEP250:ENST00000342580.4:exon29:c.C4666T:p.H1556Y,CEP250:ENST00000422671.1:exon1:c.C298T:p.H100Y,CEP250:ENST00000397527.1:exon30:c.C4834T:p.H1612Y,	20q11.22	.
20	35444049	G	A	CTGGAGGTTG,AGAGGAGCAC	exonic	SOGA1	nonsynonymous SNV	SOGA1:ENST00000279034.6:exon5:c.C1082T:p.S361F,SOGA1:ENST00000456801.2:exon1:c.C605T:p.S202F,SOGA1:ENST00000357779.3:exon5:c.C1082T:p.S361F,SOGA1:ENST00000237536.4:exon5:c.C1796T:p.S599F,	20q11.23	.
20	36869581	G	A	AGTATCTTTT,AAATAAGGGA	exonic	KIAA1755	stopgain	KIAA1755:ENST00000279024.4:exon3:c.C952T:p.Q318X,	20q11.23	.
20	40043875	C	T	GAGGTCCATC,CTCCTTCAAC	exonic	CHD6	nonsynonymous SNV	CHD6:ENST00000373233.3:exon34:c.G6890A:p.G2297E,	20q12	.
20	44485900	G	A	TCCCCAGGGG,ATCGCCGCGG	exonic	ACOT8	nonsynonymous SNV	ACOT8:ENST00000483141.1:exon1:c.C55T:p.P19S,ACOT8:ENST00000461272.1:exon1:c.C55T:p.P19S,ACOT8:ENST00000481938.1:exon1:c.C55T:p.P19S,ACOT8:ENST00000488679.1:exon1:c.C55T:p.P19S,ACOT8:ENST00000426915.1:exon1:c.C49T:p.P17S,ACOT8:ENST00000217455.4:exon1:c.C55T:p.P19S,A	20q13.12	ACOT8;basechange=c.55C>T;AAchange=p.P19S;tissue=skin
20	44505609	C	T	CAAAAAAGAC,TTGACACTGC	exonic	ZSWIM3	nonsynonymous SNV	ZSWIM3:ENST00000454862.2:exon3:c.C394T:p.L132F,ZSWIM3:ENST00000255152.2:exon2:c.C412T:p.L138F,	20q13.12	.
20	44569148	C	A	ACCCCACTGC,CCAAGACTCA	exonic	PCIF1	nonsynonymous SNV	PCIF1:ENST00000372409.3:exon5:c.C284A:p.P95H,	20q13.12	.
20	45354861	C	T	CTCTGCCCTC,CTGGGCCCCC	exonic	SLC2A10	nonsynonymous SNV	SLC2A10:ENST00000359271.2:exon2:c.C1186T:p.P396S,	20q13.12	.
20	45717980	G	A	CGGTCTAAGA,GAGCAGTGAC	exonic	EYA2	nonsynonymous SNV	EYA2:ENST00000497062.2:exon8:c.G692A:p.R231K,EYA2:ENST00000458636.1:exon4:c.G377A:p.R126K,EYA2:ENST00000357410.3:exon8:c.G764A:p.R255K,EYA2:ENST00000327619.5:exon8:c.G764A:p.R255K,EYA2:ENST00000317304.6:exon7:c.G764A:p.R255K,	20q13.12	.
20	47251229	C	T	ACATAGTTCT,GTCCAGCTGC	exonic	PREX1	nonsynonymous SNV	PREX1:ENST00000371941.3:exon33:c.G4252A:p.E1418K,PREX1:ENST00000396220.1:exon33:c.G4252A:p.E1418K,	20q13.13	PREX1_ENST00000396220;basechange=c.4252G>A;AAchange=p.E1418K;tissue=endometrium&PREX1;basechange=c.4252G>A;AAchange
20	47990041	G	A	CCTAGAACGG,GAGGAGTGGA	exonic	KCNB1	nonsynonymous SNV	KCNB1:ENST00000371741.4:exon2:c.C2056T:p.P686S,	20q13.13	KCNB1;basechange=c.2056C>G;AAchange=p.P686A;tissue=lung
20	50408892	C	T	ACTGGAGCAC,TGTAACAAGA	splicing	SALL4(ENST(	.	.	20q13.2	.
20	51870985	C	T	TGTCAATCGG,CGTGTTCCCC	exonic	TSHZ2	nonsynonymous SNV	TSHZ2:ENST00000329613.6:exon2:c.C979T:p.P327S,TSHZ2:ENST00000371497.5:exon2:c.C988T:p.P330S,TSHZ2:ENST00000603338.2:exon2:c.C979T:p.P327S,	20q13.2	.
20	52775569	C	T	ACCTGATTCT,AGGTAATACA	exonic	CYP24A1	nonsynonymous SNV	CYP24A1:ENST00000216862.3:exon8:c.G1084A:p.E362K,CYP24A1:ENST00000395954.3:exon6:c.G658A:p.E220K,CYP24A1:ENST00000395955.3:exon8:c.G1084A:p.E362K,	20q13.2	.
20	56139489	G	A	CGGGCTGCAG,GACTGCCTGT	exonic	PCK1	nonsynonymous SNV	PCK1:ENST00000535860.1:exon5:c.G830A:p.G277E,	20q13.31	.
20	56227245	G	A	GTGCTGGAAG,AGGACCCCGG	exonic	PMEPA1	nonsynonymous SNV	PMEPA1:ENST00000395814.1:exon4:c.C578T:p.S193F,PMEPA1:ENST00000265626.4:exon4:c.C578T:p.S193F,PMEPA1:ENST00000341744.3:exon4:c.C728T:p.S243F,PMEPA1:ENST00000347215.4:exon4:c.C623T:p.S208F,PMEPA1:ENST00000395816.3:exon4:c.C578T:p.S193F,	20q13.31	.
20	60578787	C	T	AGGCCCTCAC,TGGCTGCAGT	exonic	TAF4	nonsynonymous SNV	TAF4:ENST00000252996.4:exon8:c.G2371A:p.V791I,TAF4:ENST00000488539.1:exon3:c.G376A:p.V126I,	20q13.33	.
20	60791471	G	A	GCCCCTCGAG,AGCTGCCGGA	exonic	HRH3	nonsynonymous SNV	HRH3:ENST00000317393.6:exon3:c.C929T:p.S310F,HRH3:ENST00000340177.5:exon3:c.C929T:p.S310F,	20q13.33	.
20	61940730	C	T	CTGGCGTCCC,GCCGAGGGAC	exonic	COL20A1	nonsynonymous SNV	COL20A1:ENST00000326996.6:exon9:c.C986T:p.P329L,COL20A1:ENST00000435874.1:exon9:c.C1007T:p.P336L,COL20A1:ENST00000422202.1:exon8:c.C1007T:p.P336L,COL20A1:ENST00000358894.6:exon9:c.C986T:p.P329L,	20q13.33	.
21	15954569	G	T	ATCAAGACAT,TCCAGAGAGG	exonic	SAMSN1	nonsynonymous SNV	SAMSN1:ENST00000285670.2:exon2:c.C149A:p.T50K,	21q11.2	.
21	19642327	C	T	AACCTTGAGA,CCTGGCATAC	exonic	TMPRSS15	nonsynonymous SNV	TMPRSS15:ENST00000284885.3:exon25:c.G3019A:p.V1007I,	21q21.1	TMPRSS15;basechange=c.3019G>T;AAchange=p.V1007F;tissue=lung
21	26973715	T	A	ATTGACCATA,ATTCATCTTT	exonic	MRPL39	nonsynonymous SNV	MRPL39:ENST00000352957.4:exon4:c.A485T:p.Y162F,MRPL39:ENST00000419219.1:exon4:c.A485T:p.Y162F,MRPL39:ENST00000307301.7:exon4:c.A485T:p.Y162F,	21q21.3	.
21	30365209	G	A	GGCGGCGGAA,AGGACACCCT	exonic	LTN1	nonsynonymous SNV	LTN1:ENST00000389195.2:exon1:c.C56T:p.S19F,LTN1:ENST00000389194.2:exon1:c.C56T:p.S19F,	21q21.3	.
21	32253501	C	T	GAGATGCCTC,CAGGGGCTGA	exonic	KRTAP11-1	nonsynonymous SNV	KRTAP11-1:ENST00000332378.4:exon1:c.G343A:p.G115R,	21q22.11	.
21	33723203	G	A	AAGGGCAGGG,GCGAGGGGCT	exonic	URB1	nonsynonymous SNV	URB1:ENST00000382751.3:exon20:c.C2642T:p.P881L,	21q22.11	.
21	33723204	G	A	AGGGCAGGGG,CGAGGGGCTG	exonic	URB1	nonsynonymous SNV	URB1:ENST00000382751.3:exon20:c.C2641T:p.P881S,	21q22.11	.
21	35467894	C	T	GTCTCTGATT,TCTATATTTT	exonic	SLC5A3	nonsynonymous SNV	SLC5A3:ENST00000608209.1:exon2:c.C397T:p.L133F,SLC5A3:ENST00000381151.3:exon2:c.C397T:p.L133F,	21q22.11	SLC5A3;basechange=c.397C>G;AAchange=p.L133V;tissue=breast
21	35467931	C	T	GATCTGTATT,GGGTGCCCTT	exonic	SLC5A3	nonsynonymous SNV	SLC5A3:ENST00000608209.1:exon2:c.C434T:p.S145L,SLC5A3:ENST00000381151.3:exon2:c.C434T:p.S145L,	21q22.11	.
21	40568598	G	A	TCCAATTCAG,ATGCGATCTC	exonic	BRWD1	nonsynonymous SNV	BRWD1:ENST00000333229.2:exon41:c.C6397T:p.P2133S,BRWD1:ENST00000380800.3:exon41:c.C6397T:p.P2133S,BRWD1:ENST00000342449.3:exon41:c.C6397T:p.P2133S,	21q22.2	.
21	42813675	G	A	GGTGGACAAA,GAACTGAAGA	exonic	MX1	nonsynonymous SNV	MX1:ENST00000288383.6:exon6:c.G694A:p.G232R,MX1:ENST00000398598.3:exon10:c.G763A:p.G255R,MX1:ENST00000455164.2:exon8:c.G763A:p.G255R,MX1:ENST00000398600.2:exon12:c.G763A:p.G255R,	21q22.3	.
21	43524101	G	A	GCAGGACCCC,GGTTTCCCAT	exonic	C21orf128,UM	nonsynonymous SNV	UMODL1:ENST00000408910.2:exon9:c.G1423A:p.G475R,UMODL1:ENST00000400424.2:exon9:c.G1207A:p.G403R,UMODL1:ENST00000408989.2:exon9:c.G1423A:p.G475R,UMODL1:ENST00000400427.1:exon9:c.G1207A:p.G403R,	21q22.3	.
21	44106363	G	A	ATCGCCACCG,CCTGCCTCGG	exonic	PDE9A	nonsynonymous SNV	PDE9A:ENST00000539837.1:exon2:c.G26A:p.G9D,PDE9A:ENST00000398236.3:exon2:c.G131A:p.G44D,PDE9A:ENST00000328862.6:exon2:c.G131A:p.G44D,PDE9A:ENST00000380328.2:exon2:c.G131A:p.G44D,PDE9A:ENST00000335512.4:exon2:c.G131A:p.G44D,PDE9A:ENST00000291539.6:exon2:c.G131A:p	21q22.3	.
21	44492171	G	A	GCATCGGGCC,GATCCACAGG	exonic	CBS	nonsynonymous SNV	CBS:ENST00000398165.3:exon3:c.C133T:p.R45W,CBS:ENST00000352178.5:exon3:c.C133T:p.R45W,CBS:ENST00000398158.1:exon3:c.C133T:p.R45W,CBS:ENST00000398168.1:exon3:c.C133T:p.R45W,CBS:ENST00000359624.3:exon3:c.C133T:p.R45W,CBS:ENST00000441030.1:exon3:c.C133T:p.R45W,	21q22.3	.
21	45681054	G	A	TGTCCCGGGT,AAACGGAGCT	exonic	DNMT3L	nonsynonymous SNV	DNMT3L:ENST00000431166.1:exon2:c.C89T:p.S30L,DNMT3L:ENST00000270172.3:exon2:c.C89T:p.S30L,DNMT3L:ENST00000418993.1:exon2:c.C89T:p.S30L,	21q22.3	.
21	45706903	C	T	AAGCCCCCGG,CGTCCCCAAG	exonic	AIRE	nonsynonymous SNV	AIRE:ENST00000291582.5:exon3:c.C350T:p.A117V,	21q22.3	.
21	46074389	G	A	CCCACACAGG,GCCGGCACAG	exonic	KRTAP12-4	nonsynonymous SNV	KRTAP12-4:ENST00000391618.1:exon1:c.C143T:p.P48L,	21q22.3	.
21	46951749	G	A	GCCCAGCACG,AGCTGGTGAA	exonic	SLC19A1	nonsynonymous SNV	SLC19A1:ENST00000567670.1:exon3:c.C503T:p.S168F,SLC19A1:ENST00000427839.1:exon3:c.C503T:p.S168F,SLC19A1:ENST00000485649.2:exon2:c.C383T:p.S128F,SLC19A1:ENST00000380010.4:exon3:c.C503T:p.S168F,SLC19A1:ENST00000311124.4:exon3:c.C503T:p.S168F,	21q22.3	.
21	47687043	G	A	CGCCCGGAGA,CATCCTTGCG	exonic	MCM3AP	nonsynonymous SNV	MCM3AP:ENST00000291688.1:exon10:c.C2642T:p.A881V,MCM3AP:ENST00000397708.1:exon11:c.C2642T:p.A881V,	21q22.3	.
22	17585674	C	T	GCGACTGTTT,CTGCCCAGAA	exonic	IL17RA	nonsynonymous SNV	IL17RA:ENST00000319363.6:exon9:c.C905T:p.S302F,	22q11.1	.
22	18222162	C	T	AGGCCGTTCA,CAGGCCCGGA	exonic	BID	stopgain	BID:ENST00000342111.5:exon5:c.G411A:p.W137X,	22q11.21	.
22	19711697	G	T	CCCCGAGCGT,CGCCCTACCG	exonic	GP1BB	nonsynonymous SNV	GP1BB:ENST00000366425.3:exon2:c.G331T:p.A111S,	22q11.21	.
22	20082274	A	G	TGACTTTGTT,AACAGACCTC	exonic	DGCR8	nonsynonymous SNV	DGCR8:ENST00000383024.2:exon8:c.A1645G:p.K549E,DGCR8:ENST00000407755.1:exon7:c.A1645G:p.K549E,DGCR8:ENST00000351989.3:exon9:c.A1744G:p.K582E,	22q11.21	.
22	20779997	G	A	GGGGACCGCC,GCGTCCGTGG	exonic	SCARF2	nonsynonymous SNV	SCARF2:ENST00000266214.5:exon11:c.C2281T:p.R761W,SCARF2:ENST00000405555.3:exon11:c.C2266T:p.R756W,	22q11.21	.
22	20936979	C	T	GCCCCCTCCC,CCCAGCCGTC	exonic	MED15	nonsynonymous SNV	MED15:ENST00000263205.7:exon10:c.C1354T:p.P452S,MED15:ENST00000406969.1:exon9:c.C1156T:p.P386S,MED15:ENST00000541476.1:exon9:c.C1156T:p.P386S,MED15:ENST00000382974.2:exon8:c.C1021T:p.P341S,MED15:ENST00000292733.7:exon9:c.C1234T:p.P412S,MED15:ENST00000425759.2:exo	22q11.21	.
22	21235374	G	A	AGAACAGGAA,CAAAGTACCA	exonic	SNAP29	nonsynonymous SNV	SNAP29:ENST00000215730.7:exon3:c.G472A:p.A158T,SNAP29:ENST00000439214.1:exon3:c.G193A:p.A65T,	22q11.21	.
22	21344766	G	A	GGGCAAAGCG,AGCCAAAATA	exonic	LZTR1	nonsynonymous SNV	LZTR1:ENST00000215739.8:exon8:c.G743A:p.G248E,LZTR1:ENST00000389355.3:exon7:c.G686A:p.G229E,	22q11.21	.
22	21385436	C	T	CGTCAGCGCT,CAGTTGTGAG	exonic	SLC7A4	stopgain	SLC7A4:ENST00000403586.1:exon2:c.G666A:p.W222X,SLC7A4:ENST00000382932.2:exon2:c.G666A:p.W222X,	22q11.21	.
22	23135185	G	C	CTCTTTCCAG,ATCCTGGGCT	exonic	IGLV2-11	nonsynonymous SNV	IGLV2-11:ENST00000390314.2:exon2:c.G47C:p.G16A,	22q11.22	.
22	24584026	A	T	GACAACGGCT,CAGCCTGGCC	exonic	SUSD2	nonsynonymous SNV	SUSD2:ENST00000358321.3:exon13:c.A2264T:p.Y755F,	22q11.23	.
22	25131767	A	C	TGCTCCTGCT,TAGAGTATGA	exonic	PIWIL3	stopgain	PIWIL3:ENST00000527701.1:exon14:c.T1215G:p.Y405X,PIWIL3:ENST00000533313.1:exon14:c.T1215G:p.Y405X,PIWIL3:ENST00000332271.5:exon13:c.T1542G:p.Y514X,	22q11.23	.
22	29279833	C	T	CCGCGGCCTC,GGTGCAGCCG	exonic	ZNRF3	nonsynonymous SNV	ZNRF3:ENST00000544604.2:exon1:c.C79T:p.R27W,	22q12.1	.
22	31330808	G	A	CTTCACCACT,GAGTCTTGAT	exonic	MORC2	nonsynonymous SNV	MORC2:ENST00000397641.3:exon19:c.C2153T:p.P718L,MORC2:ENST00000215862.4:exon20:c.C1967T:p.P656L,	22q12.2	.
22	31679097	C	T	CGGCCTGGCC,ATAAGGGGGG	exonic	PIK3IP1	nonsynonymous SNV	PIK3IP1:ENST00000215912.5:exon6:c.G765A:p.M255I,PIK3IP1:ENST00000487265.2:exon4:c.G528A:p.M176I,	22q12.2	.
22	31942873	G	A	CGATTACTAC,GAAGGTCTTC	exonic	SFI1	nonsynonymous SNV	SFI1:ENST00000400289.1:exon3:c.G119A:p.R40Q,SFI1:ENST00000432498.1:exon5:c.G365A:p.R122Q,SFI1:ENST00000540643.1:exon4:c.G293A:p.R98Q,SFI1:ENST00000414585.1:exon3:c.G119A:p.R40Q,SFI1:ENST00000524296.1:exon4:c.G365A:p.R122Q,SFI1:ENST00000400288.2:exon5:c.G365A:p.R122Q,SF	22q12.2	.
22	32795595	G	A	GTTACCTGAG,AAGGCCTCTT	exonic	RTCB	nonsynonymous SNV	RTCB:ENST00000216038.5:exon6:c.C649T:p.P217S,	22q12.3	.
22	37524291	G	A	CCAGCGTCAG,GACCTCCTCC	exonic	IL2RB	nonsynonymous SNV	IL2RB:ENST00000216223.5:exon10:c.C1501T:p.P501S,	22q12.3	.
22	38119354	G	A	CCTGCCCAAA,GGACACTGCT	exonic	TRIOBP	nonsynonymous SNV	TRIOBP:ENST00000406386.3:exon7:c.G791A:p.R264K,	22q13.1	.
22	38201649	T	G	GACATGATCG,GGCTGCCATC	exonic	H1F0	nonsynonymous SNV	H1F0:ENST00000340857.2:exon1:c.T98G:p.V33G,	22q13.1	.
22	40036981	C	T	CCATGAGATC,CCCCGCTCAA	exonic	CACNA1I	nonsynonymous SNV	CACNA1I:ENST00000336649.4:exon8:c.C850T:p.P284S,CACNA1I:ENST00000401624.1:exon6:c.C850T:p.P284S,CACNA1I:ENST00000400164.3:exon8:c.C850T:p.P284S,CACNA1I:ENST00000407673.1:exon6:c.C850T:p.P284S,CACNA1I:ENST00000402142.3:exon6:c.C850T:p.P284S,CACNA1I:ENST00000404898	22q13.1	.
22	41513781	C	T	GGGCTCTCCC,AGATGGGAGG	exonic	EP300	stopgain	EP300:ENST00000263253.7:exon2:c.C685T:p.Q229X,	22q13.2	.
22	41536160	C	T	AGCCATATTT,CTACGCCGGA	exonic	EP300	nonsynonymous SNV	EP300:ENST00000263253.7:exon9:c.C1777T:p.P593S,	22q13.2	.
22	42607020	G	C	TGAAGACCTT,GAAGAGGTTT	exonic	TCF20	nonsynonymous SNV	TCF20:ENST00000359486.3:exon1:c.C4292G:p.P1431R,TCF20:ENST00000335626.4:exon1:c.C4292G:p.P1431R,	22q13.2	.
22	43024257	G	A	CCTCCAGCGG,AAACTTGGGA	exonic	CYB5R3	nonsynonymous SNV	CYB5R3:ENST00000407623.3:exon5:c.C295T:p.P99S,CYB5R3:ENST00000438270.1:exon5:c.C295T:p.P99S,CYB5R3:ENST00000361740.4:exon5:c.C463T:p.P155S,CYB5R3:ENST00000402438.1:exon6:c.C295T:p.P99S,CYB5R3:ENST00000352397.5:exon5:c.C364T:p.P122S,CYB5R3:ENST00000396303.3:exon5:c	22q13.2	.
22	46658205	C	T	CCAAGCATCA,CGCCTGCAGG	exonic	PKDREJ	nonsynonymous SNV	PKDREJ:ENST00000253255.5:exon1:c.G1015A:p.V339M,	22q13.31	.
22	47287188	G	A	ATGTAGACCG,AGACCAGCCA	exonic	TBC1D22A	nonsynonymous SNV	TBC1D22A:ENST00000394449.2:exon5:c.G664A:p.E222K,	22q13.31	.
22	50217178	T	G	GGCCGCGCAC,GCAGGCAGTG	exonic	BRD1	nonsynonymous SNV	BRD1:ENST00000404034.1:exon2:c.A788C:p.Q263P,BRD1:ENST00000216267.8:exon1:c.A788C:p.Q263P,BRD1:ENST00000457780.2:exon1:c.A788C:p.Q263P,BRD1:ENST00000404760.1:exon2:c.A788C:p.Q263P,	22q13.33	.
22	50217179	G	T	GCCGCGCACT,CAGGCAGTGG	exonic	BRD1	nonsynonymous SNV	BRD1:ENST00000404034.1:exon2:c.C787A:p.Q263K,BRD1:ENST00000216267.8:exon1:c.C787A:p.Q263K,BRD1:ENST00000457780.2:exon1:c.C787A:p.Q263K,BRD1:ENST00000404760.1:exon2:c.C787A:p.Q263K,	22q13.33	.
22	50488577	C	T	TCCGGAATGA,CTTGGGCAAA	exonic	TTLL8	nonsynonymous SNV	TTLL8:ENST00000400475.1:exon2:c.G151A:p.V51I,TTLL8:ENST00000266182.6:exon2:c.G151A:p.V51I,TTLL8:ENST00000433387.1:exon3:c.G259A:p.V87I,	22q13.33	.
22	50537887	G	A	AGCTGTCTCT,ATAAGTGGGA	exonic	MOV10L1	nonsynonymous SNV	MOV10L1:ENST00000395858.3:exon3:c.G298A:p.D100N,MOV10L1:ENST00000545383.1:exon4:c.G298A:p.D100N,MOV10L1:ENST00000540615.1:exon3:c.G238A:p.D80N,MOV10L1:ENST00000262794.5:exon3:c.G298A:p.D100N,	22q13.33	.
22	51010527	G	A	TAGCCCAGGT,GAAGCTGTCT	exonic	CPT1B	nonsynonymous SNV	CPT1B:ENST00000405237.3:exon12:c.C1483T:p.H495Y,CPT1B:ENST00000440709.1:exon13:c.C1240T:p.H414Y,CPT1B:ENST00000434492.2:exon15:c.C874T:p.H292Y,CPT1B:ENST00000395650.2:exon13:c.C1483T:p.H495Y,CPT1B:ENST00000360719.2:exon13:c.C1483T:p.H495Y,CPT1B:ENST00000457250.1:e	22q13.33	.
22	51064410	G	A	GCCATCCAAG,TGACATTGGG	exonic	ARSA	nonsynonymous SNV	ARSA:ENST00000547805.1:exon7:c.C1055T:p.T352I,ARSA:ENST00000395621.3:exon7:c.C1061T:p.T354I,ARSA:ENST00000395619.3:exon7:c.C1061T:p.T354I,ARSA:ENST00000216124.5:exon6:c.C1061T:p.T354I,ARSA:ENST00000547307.1:exon6:c.C1055T:p.T352I,ARSA:ENST00000453344.2:exon6:c.C803T:p.`	22q13.33	.
22	51065440	G	A	ACCGTCGCAA,GAGTGGCCGG	exonic	ARSA	nonsynonymous SNV	ARSA:ENST00000547805.1:exon4:c.C500T:p.P167L,ARSA:ENST00000395621.3:exon4:c.C506T:p.P169L,ARSA:ENST00000395619.3:exon4:c.C506T:p.P169L,ARSA:ENST00000216124.5:exon3:c.C506T:p.P169L,ARSA:ENST00000547307.1:exon3:c.C500T:p.P167L,ARSA:ENST00000453344.2:exon3:c.C248T:p.P8	22q13.33	.
22	51159802	C	T	AGAGAAGGTC,CCCGGGAGGA	exonic	SHANK3	nonsynonymous SNV	SHANK3:ENST00000445220.2:exon21:c.C3547T:p.P1183S,SHANK3:ENST00000414786.2:exon21:c.C3499T:p.P1167S,SHANK3:ENST00000262795.3:exon22:c.C3589T:p.P1197S,	22q13.33	.
X	2609562	G	A	GCGCCCCGCG,GGACACCCGG	exonic	CD99	nonsynonymous SNV	CD99:ENST00000449611.1:exon1:c.G22A:p.G8R,	Xp22.33	.
X	14748528	C	T	GCTGCCTTCC,ATTGGCCTTC	exonic	GLRA2	nonsynonymous SNV	GLRA2:ENST00000355020.4:exon9:c.C1280T:p.P427L,GLRA2:ENST00000218075.4:exon9:c.C1280T:p.P427L,GLRA2:ENST00000443437.2:exon11:c.C1013T:p.P338L,	Xp22.2	.
X	17744175	C	T	GAAACCATCC,TCCTGCAGCT	exonic	NHS	nonsynonymous SNV	NHS:ENST00000398097.3:exon7:c.C1418T:p.P473L,NHS:ENST00000380060.3:exon6:c.C1886T:p.P629L,	Xp22.13	.
X	22117181	A	C	CCCCCATCTG,AAGACATCAG	exonic	PHEX	nonsynonymous SNV	PHEX:ENST00000537599.1:exon9:c.A991C:p.K331Q,PHEX:ENST00000418858.3:exon2:c.A100C:p.K34Q,PHEX:ENST00000535894.1:exon8:c.A700C:p.K234Q,PHEX:ENST00000379374.4:exon9:c.A991C:p.K331Q,	Xp22.11	.
X	22291322	C	T	GCGAATAATT,CGTGCAAGCA	exonic	ZNF645	nonsynonymous SNV	ZNF645:ENST00000323684.1:exon1:c.C214T:p.P72S,	Xp22.11	ZNF645;basechange=c.214C>T;AAchange=p.P72S;tissue=skin
X	24593318	G	A	TCCCACTTTT,AATTAGATCA	exonic	PCYT1B	stopgain	PCYT1B:ENST00000379144.2:exon7:c.C826T:p.Q276X,PCYT1B:ENST00000379145.1:exon7:c.C772T:p.Q258X,PCYT1B:ENST00000356768.4:exon7:c.C826T:p.Q276X,	Xp22.11	.
X	32716048	G	A	CTGTGTGTAG,CATAGCTCTT	exonic	DMD	nonsynonymous SNV	DMD:ENST00000357033.4:exon9:c.C899T:p.A300V,DMD:ENST00000378677.2:exon9:c.C887T:p.A296V,DMD:ENST00000288447.4:exon9:c.C875T:p.A292V,	Xp21.1	.
X	34148085	G	A	CGTTGAATAG,AGTCTTAACA	exonic	FAM47A	nonsynonymous SNV	FAM47A:ENST00000346193.3:exon1:c.C2311T:p.P771S,	Xp21.1	.
X	50054102	C	T	CCAAATGTGT,TAGCACTGCC	exonic	CCNB3	nonsynonymous SNV	CCNB3:ENST00000376042.1:exon6:c.C2933T:p.S978F,CCNB3:ENST00000276014.7:exon5:c.C2933T:p.S978F,	Xp11.22	.
X	71358887	C	T	ATTCCGGGAG,GGTCACTGTC	exonic	NHSL2	nonsynonymous SNV	NHSL2:ENST00000373677.1:exon2:c.C391T:p.R131W,NHSL2:ENST00000540800.1:exon6:c.C1489T:p.R497W,NHSL2:ENST00000510661.1:exon3:c.C796T:p.R266W,NHSL2:ENST00000535692.1:exon3:c.C391T:p.R131W,	Xq13.1	.
X	83129491	G	A	AATGAAAAAG,GGAAAAAGCA	exonic	CYLC1	nonsynonymous SNV	CYLC1:ENST00000329312.4:exon4:c.G1775A:p.G592E,	Xq21.1	.
X	100548008	G	A	TGAAGAAATG,GGAGCTGTCC	exonic	TAF7L	nonsynonymous SNV	TAF7L:ENST00000372907.3:exon1:c.C26T:p.P9L,	Xq22.1	.
X	101409198	C	T	TGCACTGGGG,CTTCTCCACA	exonic	BEX5	nonsynonymous SNV	BEX5:ENST00000333643.3:exon3:c.G40A:p.A14T,BEX5:ENST00000543160.1:exon3:c.G40A:p.A14T,	Xq22.1	.
X	105066872	G	A	GGACAGGGAG,TCACGGATCT	exonic	NRK	nonsynonymous SNV	NRK:ENST00000243300.9:exon1:c.G34A:p.V12I,NRK:ENST00000428173.2:exon1:c.G34A:p.V12I,NRK:ENST00000536164.1:exon1:c.G34A:p.V12I,	Xq22.3	.
X	114425175	G	A	CTGCTACGAG,AGTACAGAGG	exonic	RBMXL3	nonsynonymous SNV	RBMXL3:ENST00000424776.3:exon1:c.G1171A:p.E391K,	Xq23	.
X	119438226	C	T	GTAATAACCT,CTACAGTCAC	exonic	TMEM255A	nonsynonymous SNV	TMEM255A:ENST00000440464.1:exon2:c.G179A:p.G60E,TMEM255A:ENST00000519908.1:exon2:c.G179A:p.G60E,TMEM255A:ENST00000309720.5:exon2:c.G179A:p.G60E,TMEM255A:ENST00000371369.4:exon2:c.G179A:p.G60E,	Xq24	.
X	130222658	G	T	TGACGATGAG,AAGGAGCGGG	exonic	ARHGAP36	stopgain	ARHGAP36:ENST00000370921.1:exon9:c.G1135T:p.E379X,ARHGAP36:ENST00000412432.2:exon12:c.G1450T:p.E484X,ARHGAP36:ENST00000276211.5:exon12:c.G1543T:p.E515X,ARHGAP36:ENST00000370922.1:exon12:c.G1507T:p.E503X,	Xq26.1	.
X	130415841	G	A	TGAGATACTC,GCACTGAAGG	exonic	IGSF1	stopgain	IGSF1:ENST00000370904.1:exon14:c.C1297T:p.R433X,IGSF1:ENST00000370910.1:exon7:c.C1297T:p.R433X,IGSF1:ENST00000370903.3:exon8:c.C1324T:p.R442X,IGSF1:ENST00000361420.3:exon8:c.C1324T:p.R442X,	Xq26.2	.
X	134421701	C	T	TTTGATACTT,GCATCCTGAT	exonic	ZNF75D	nonsynonymous SNV	ZNF75D:ENST00000370766.3:exon7:c.G901A:p.E301K,ZNF75D:ENST00000370764.1:exon4:c.G616A:p.E206K,	Xq26.3	ZNF75D;basechange=c.901G>A;AAchange=p.E301K;tissue=breast
X	142717117	G	A	GGGACCCAAA,GAGTGGTGAA	exonic	SLITRK4	nonsynonymous SNV	SLITRK4:ENST00000338017.4:exon2:c.C1808T:p.P603L,SLITRK4:ENST00000356928.1:exon2:c.C1808T:p.P603L,SLITRK4:ENST00000381779.4:exon2:c.C1808T:p.P603L,	Xq27.3	.
X	142718159	T	A	TGTTTGTTGG,TTCTTTTAAA	exonic	SLITRK4	nonsynonymous SNV	SLITRK4:ENST00000338017.4:exon2:c.A766T:p.T256S,SLITRK4:ENST00000356928.1:exon2:c.A766T:p.T256S,SLITRK4:ENST00000381779.4:exon2:c.A766T:p.T256S,	Xq27.3	.
X	147088357	G	A	GCTCCATTTA,AGATGGTAAG	exonic	FMR1NB	nonsynonymous SNV	FMR1NB:ENST00000370467.3:exon3:c.G533A:p.R178K,	Xq27.3	.
X	149680447	G	A	ACCAACTCTG,GGAAGCGGCA	exonic	MAMLD1	nonsynonymous SNV	MAMLD1:ENST00000432680.2:exon5:c.G2101A:p.G701R,	Xq28	.
X	149680448	G	A	CCAACTCTGG,GAAGCGGCAA	exonic	MAMLD1	nonsynonymous SNV	MAMLD1:ENST00000432680.2:exon5:c.G2102A:p.G701E,	Xq28	.
X	150817120	G	A	ACACTAGCAT,AAAGCCGTGT	exonic	PASD1	nonsynonymous SNV	PASD1:ENST00000370357.4:exon9:c.G663A:p.M221I,	Xq28	.
X	152139056	T	G	CACCATTCAC,GTGGGAAATG	exonic	ZNF185	nonsynonymous SNV	ZNF185:ENST00000535861.1:exon23:c.T2137G:p.C713G,ZNF185:ENST00000318504.7:exon20:c.T1864G:p.C622G,ZNF185:ENST00000324823.6:exon19:c.T1345G:p.C449G,ZNF185:ENST00000449285.2:exon22:c.T2044G:p.C682G,ZNF185:ENST00000370268.4:exon22:c.T2041G:p.C681G,ZNF185:ENST0000053	Xq28	.
X	153175532	G	A	CTCTCCAGCT,GGCGTCCCTG	exonic	ARHGAP4	stopgain	ARHGAP4:ENST00000370016.1:exon17:c.C2101T:p.Q701X,ARHGAP4:ENST00000393721.1:exon15:c.C1630T:p.Q544X,ARHGAP4:ENST00000350060.5:exon18:c.C2164T:p.Q722X,ARHGAP4:ENST00000370028.3:exon19:c.C2284T:p.Q762X,ARHGAP4:ENST00000537206.1:exon18:c.C2095T:p.Q699X,	Xq28	.
X	153296101	G	A	GGAGCTCTCG,GCTCAGGTGG	exonic	MECP2	nonsynonymous SNV	MECP2:ENST00000303391.6:exon4:c.C1178T:p.P393L,MECP2:ENST00000453960.2:exon3:c.C1214T:p.P405L,	Xq28	.
X	153599481	G	A	GTGTTCTGCT,GATCTTCTTC	exonic	FLNA	stopgain	FLNA:ENST00000420627.1:exon1:c.C91T:p.Q31X,FLNA:ENST00000422373.1:exon2:c.C133T:p.Q45X,FLNA:ENST00000360319.4:exon1:c.C133T:p.Q45X,FLNA:ENST00000344736.4:exon1:c.C133T:p.Q45X,FLNA:ENST00000369850.3:exon2:c.C133T:p.Q45X,	Xq28	.
X	154741371	G	A	TCAGTGTGCC,ATCCAGAGCT	exonic	TMLHE	nonsynonymous SNV	TMLHE:ENST00000334398.3:exon5:c.C721T:p.R241W,TMLHE:ENST00000369439.4:exon5:c.C721T:p.R241W,	Xq28	.

.R211C;tissue=large_intestine

.P659S;tissue=large_intestine

hange=p.R635Q;tissue=haematopoietic_and_lymphoid_tissu

60S;tissue=lung

156A;tissue=prostate

p.M755I;tissue=endometrium&KIF13A_ENST00000378814;basechange=c.2265G>A;AAchange=p.M755I;tissue=endometriu

3503V;tissue=lung

ge=p.G1413R;tissue=large_intestine&PTPRD_ENST00000346816;basechange=c.3868G>A;AAchange=p.G1290R;tissue=large_intestine&PTPRD_ENST00000381196;basechange=c.5455G>A;AAchange=p.G1819R;tissue=large_intestine

C>A;AAchange=p.S316Y;tissue=endometriur

sue=lung

ange=p.R765Q;tissue=haematopoietic_and_lymphoid_tissu

nge=p.E1137K;tissue=endometrium

Achange=p.D240H;tissue=lung&NTRK3;basechange=c.718G>C;AAchange=p.D240H;tissue=lung

e=p.R1326Q;tissue=endometrium

7C>T;AAchange=p.R103*;tissue=breast,haematopoietic_and_lymphoid_tissue,stomach,oesophagus,lung,upper_aerodigestive_tract,large_intestine,central_nervous_system,biliary_tract&TP53;basechange=c.586C>T;AAchange=p.R196*;tissue=ovary,breast,stomach,haematopoietic_and_lymphoid_tissue,urinary_tract,kidney,pancreas,liver,oesophagus,skin,lung,upper_aerodigestive_tract,large_intestine,central_nervous_system,biliary_tract&TP53_ENST00000269305;ba

p.P144H;tissue=large_intestine

>A;AAchange=p.G106R;tissue=lung&MUC16;basechange=c.13417G>A;AAchange=p.G4473R;tissue=lung

R264W;tissue=large_intestine

8L;tissue=lung

.T133M;tissue=endometrium

=p.E1418K;tissue=endometrium

asechange=c.586C>T;AAchange=p.R196*;tissue=breast,stomach,haematopoietic_and_lymphoid_tissue,oesophagus,lung,upper_aerodigestive_tract,large_intestine,central_nervous_system,biliary_tract&TP53_ENST00000455263;basechange=c.586C>T;AAchange=p.R196*;tissue=stomach,lung,large_intestine&TP53_ENST00000413465;basechange=c.586C>T;AAchange=p.R196*;tissue=breast,haematopoietic_and_lymphoid_tissue,stomach,oesophagus,lung,upper_ae

rodigestive_tract,large_intestine,central_nerv

C000-HZGGV2

Chromosom	Position (hg1	Reference all	Mutant allele	Sequence context	ANNOVAR fu	Gene	Mutation type	ANNOVAR output (alternative transcripts)	Cytoband	C
1	1500147	G	A	CAACCCGCTG,GATACAGTTC	exonic	SSU72	nonsynonymous SNV	SSU72:ENST00000359060.4:exon2:c.C230T:p.P77L,	1p36.33	.
1	3348577	C	T	GAGCCGATGC,GACTTTTGGG	exonic	PRDM16	nonsynonymous SNV	PRDM16:ENST00000441472.2:exon16:c.C3566T:p.P1189L,PRDM16:ENST00000270722.5:exon16:c.C3569T:p.P1190L,PRDM16:ENST00000509860.1:exon12:c.C29[illegible]	1p36.32	.
1	4772642	G	A	GGGGTTCACC,AGTCCTTGGA	exonic	AJAP1	nonsynonymous SNV	AJAP1:ENST00000378191.4:exon2:c.G712A:p.E238K,AJAP1:ENST00000378190.3:exon2:c.G712A:p.E238K,	1p36.32	.
1	7724103	G	A	CAGACGTACG,GGGTGGAGGC	exonic	CAMTA1	nonsynonymous SNV	CAMTA1:ENST00000303635.7:exon9:c.G1496A:p.G499E,CAMTA1:ENST00000439411.2:exon9:c.G1496A:p.G499E,	1p36.23	.
1	7724220	C	T	GAGGACACCT,CTTCGAGCAG	exonic	CAMTA1	nonsynonymous SNV	CAMTA1:ENST00000303635.7:exon9:c.C1613T:p.S538F,CAMTA1:ENST00000439411.2:exon9:c.C1613T:p.S538F,	1p36.23	.
1	7887210	G	A	GCGGTCGGCC,GCTGCAGGAA	exonic	PER3	nonsynonymous SNV	PER3:ENST00000361923.2:exon17:c.G2197A:p.G733S,PER3:ENST00000377532.3:exon17:c.G2221A:p.G741S,	1p36.23	.
1	8420607	G	A	GAGTTGCAGG,GTGGGGGGTG	exonic	RERE	nonsynonymous SNV	RERE:ENST00000476556.1:exon8:c.C1298T:p.P433L,RERE:ENST00000337907.3:exon19:c.C2960T:p.P987L,RERE:ENST00000400908.2:exon18:c.C2960T:p.P987L[illegible]	1p36.23	.
1	8420608	G	A	AGTTGCAGGG,TGGGGGGTGA	exonic	RERE	nonsynonymous SNV	RERE:ENST00000476556.1:exon8:c.C1297T:p.P433S,RERE:ENST00000337907.3:exon19:c.C2959T:p.P987S,RERE:ENST00000400908.2:exon18:c.C2959T:p.P987S[illegible]	1p36.23	.
1	9165675	G	A	GTCCGCCATG,AGGAGTGGCG	exonic	GPR157	nonsynonymous SNV	GPR157:ENST00000377411.4:exon3:c.C662T:p.S221F,	1p36.23	.
1	10715809	G	A	GTGCTGCAGG,AGTTGTCGCG	exonic	CASZ1	nonsynonymous SNV	CASZ1:ENST00000344008.5:exon9:c.C1562T:p.S521F,CASZ1:ENST00000377022.3:exon9:c.C1562T:p.S521F,	1p36.22	.
1	15420777	C	T	GAGGACCACC,CTATGTCCCA	exonic	KAZN	nonsynonymous SNV	KAZN:ENST00000376030.2:exon9:c.C1324T:p.P442S,	1p36.21	.
1	17422406	T	G	CTAGGTACCT,CTTTGGGTTG	exonic	PADI2	nonsynonymous SNV	PADI2:ENST00000375481.1:exon4:c.A409C:p.K137Q,PADI2:ENST00000375486.4:exon4:c.A409C:p.K137Q,PADI2:ENST00000444885.2:exon4:c.A409C:p.K137Q,	1p36.13	.
1	22178167	G	A	GGCTCGATGC,GATGGGCTGG	exonic	HSPG2	nonsynonymous SNV	HSPG2:ENST00000374695.3:exon55:c.C7030T:p.R2344C,	1p36.12	.
1	26356121	C	T	TCTCAGCCCC,GCTGGGAGCT	exonic	EXTL1	nonsynonymous SNV	EXTL1:ENST00000374280.3:exon3:c.C904T:p.R302C,	1p36.11	.
1	27874056	G	A	TGGGCCAGGG,GCCGGGCCAT	exonic	AHDC1	nonsynonymous SNV	AHDC1:ENST00000247087.5:exon5:c.C4571T:p.P1524L,AHDC1:ENST00000374011.2:exon6:c.C4571T:p.P1524L,	1p36.11	.
1	31437562	G	A	AGAGAGGGAA,GAGGTGGTGT	exonic	PUM1	nonsynonymous SNV	PUM1:ENST00000440538.2:exon14:c.C2204T:p.P735L,PUM1:ENST00000373742.2:exon12:c.C2105T:p.P702L,PUM1:ENST00000424085.2:exon10:c.C1556T:p.P519[illegible]	1p35.2	P[illegible]
1	34330055	G	A	CGGAGAAAGC,GGATCCCCAG	exonic	HMGB4	nonsynonymous SNV	HMGB4:ENST00000519684.1:exon2:c.G263A:p.R88Q,HMGB4:ENST00000522796.1:exon4:c.G263A:p.R88Q,	1p35.1	.
1	36563303	G	C	GGCCCCACCA,ATGCCTGGTC	exonic	COL8A2	nonsynonymous SNV	COL8A2:ENST00000397799.1:exon4:c.C1979G:p.S660C,COL8A2:ENST00000303143.4:exon2:c.C1979G:p.S660C,COL8A2:ENST00000481785.1:exon2:c.C1784G:p.[illegible]	1p34.3	.
1	36563313	C	T	GATGCCTGGT,CAGGTAGCCC	exonic	COL8A2	nonsynonymous SNV	COL8A2:ENST00000397799.1:exon4:c.G1969A:p.D657N,COL8A2:ENST00000303143.4:exon2:c.G1969A:p.D657N,COL8A2:ENST00000481785.1:exon2:c.G1774A:p.[illegible]	1p34.3	.
1	36564717	C	T	GGCCCTGGTT,CCCCTGGAAT	exonic	COL8A2	nonsynonymous SNV	COL8A2:ENST00000397799.1:exon4:c.G565A:p.E189K,COL8A2:ENST00000303143.4:exon2:c.G565A:p.E189K,COL8A2:ENST00000481785.1:exon2:c.G370A:p.E12[illegible]	1p34.3	.
1	38095269	C	T	CTTGATCCCC,GGCTGCTGAT	exonic	RSPO1	nonsynonymous SNV	RSPO1:ENST00000401071.2:exon3:c.G65A:p.R22Q,RSPO1:ENST00000401070.1:exon3:c.G65A:p.R22Q,RSPO1:ENST00000401068.1:exon4:c.G65A:p.R22Q,RSPO[illegible]	1p34.3	.
1	39799170	C	A	ACAAACAGGT,AAAAGCTCTT	exonic	MACF1	nonsynonymous SNV	MACF1:ENST00000567887.1:exon37:c.C7021A:p.Q2341K,MACF1:ENST00000289893.4:exon1:c.C2230A:p.Q744K,MACF1:ENST00000564288.1:exon37:c.C6910A:p.[illegible]	1p34.3	.
1	40126861	C	T	GCGTCCCCAT,GAAGGCCACG	exonic	NT5C1A	nonsynonymous SNV	NT5C1A:ENST00000235628.1:exon5:c.G631A:p.D211N,	1p34.2	.
1	43687070	G	A	AGAGAAGCAG,ATGTTTATCA	exonic	WDR65	nonsynonymous SNV	WDR65:ENST00000372492.4:exon14:c.G2275A:p.D759N,	1p34.2	.
1	44056724	C	T	TCTGGGAACT,GGAGCCTGTA	exonic	PTPRF	nonsynonymous SNV	PTPRF:ENST00000372413.3:exon7:c.C1031T:p.S344L,PTPRF:ENST00000359947.4:exon9:c.C1031T:p.S344L,PTPRF:ENST00000438120.1:exon9:c.C1031T:p.S344[illegible]	1p34.2	.
1	44128655	C	T	TGTGAACACC,CATACCTGTA	exonic	KDM4A	nonsynonymous SNV	KDM4A:ENST00000372396.3:exon5:c.C520T:p.P174S,	1p34.1	K[illegible]
1	44137244	G	A	GGAGGATGAG,AGGAAGAACA	exonic	KDM4A	nonsynonymous SNV	KDM4A:ENST00000372396.3:exon11:c.G1432A:p.E478K,	1p34.1	.
1	50884924	C	T	AGCACCTGCT,GATGGCCTGC	exonic	DMRTA2	nonsynonymous SNV	DMRTA2:ENST00000404795.3:exon3:c.G1042A:p.E348K,DMRTA2:ENST00000418121.1:exon2:c.G1042A:p.E348K,	1p32.3	.
1	53072387	G	A	GCCAGCGAGT,CGGCTTCACA	exonic	GPX7	nonsynonymous SNV	GPX7:ENST00000361314.4:exon2:c.G170A:p.C57Y,	1p32.3	.
1	53556309	C	T	TGGCCAAAGT,CAGCTCGTAG	exonic	SLC1A7	nonsynonymous SNV	SLC1A7:ENST00000371494.4:exon8:c.G1201A:p.D401N,	1p32.3	.
1	57171786	C	T	ATACCATCTT,GTGTAAGAAG	exonic	PRKAA2	nonsynonymous SNV	PRKAA2:ENST00000371244.4:exon8:c.C1315T:p.R439C,	1p32.2	P[illegible]
1	57425773	C	T	AGGGTAACAT,CACACTCCGC	exonic	C8B	nonsynonymous SNV	C8B:ENST00000371237.4:exon2:c.G169A:p.D57N,C8B:ENST00000543257.1:exon3:c.G13A:p.D5N,	1p32.2	.
1	59147638	A	T	ACCATTTGGC,AGAATGAAAA	exonic	MYSM1	nonsynonymous SNV	MYSM1:ENST00000472487.1:exon8:c.T1078A:p.C360S,	1p32.1	.
1	66088628	G	C	CGAACCCCAA,AATTGTTCCT	exonic	LEPR	nonsynonymous SNV	LEPR:ENST00000371060.3:exon19:c.G2637C:p.K879N,LEPR:ENST00000371058.1:exon17:c.G2637C:p.K879N,LEPR:ENST00000371059.3:exon19:c.G2637C:p.K87[illegible]	1p31.3	.
1	66828947	C	T	TGCTATATTC,AGGTGAGTGA	exonic	PDE4B	stopgain	PDE4B:ENST00000329654.4:exon11:c.C1117T:p.Q373X,PDE4B:ENST00000423207.2:exon9:c.C1072T:p.Q358X,PDE4B:ENST00000341517.4:exon11:c.C1117T:p.Q[illegible]	1p31.3	.
1	67147645	C	T	TCCCCCAAGT,TGTTGCTGTT	exonic	SGIP1	nonsynonymous SNV	SGIP1:ENST00000371037.4:exon15:c.C908T:p.S303F,SGIP1:ENST00000237247.6:exon16:c.C920T:p.S307F,	1p31.3	.
1	67147960	G	A	TTGGGACAAA,AGCAACTCCA	exonic	SGIP1	nonsynonymous SNV	SGIP1:ENST00000371037.4:exon15:c.G1223A:p.R408K,SGIP1:ENST00000237247.6:exon16:c.G1235A:p.R412K,	1p31.3	.
1	72748081	G	A	TGTCCAGCCG,GAGGCAGGAG	exonic	NEGR1	nonsynonymous SNV	NEGR1:ENST00000357731.5:exon1:c.C97T:p.P33S,NEGR1:ENST00000434200.1:exon1:c.C91T:p.P31S,	1p31.1	.
1	75072512	C	T	CAGTTCCTCT,CTTTCTCAGT	exonic	C1orf173	nonsynonymous SNV	C1orf173:ENST00000420661.2:exon5:c.G671A:p.G224E,C1orf173:ENST00000326665.5:exon10:c.G1262A:p.G421E,	1p31.1	.
1	75602794	G	A	GGTGAGCCCC,AGGGAGCGGG	exonic	LHX8	nonsynonymous SNV	LHX8:ENST00000294638.5:exon4:c.G115A:p.E39K,LHX8:ENST00000356261.3:exon3:c.G85A:p.E29K,	1p31.1	L[illegible]
1	84791327	G	A	CAGTTCCATG,AAAATGGGAG	exonic	SAMD13	nonsynonymous SNV	SAMD13:ENST00000394834.3:exon3:c.G61A:p.E21K,SAMD13:ENST00000370673.3:exon3:c.G103A:p.E35K,SAMD13:ENST00000370669.1:exon3:c.G61A:p.E21K,S[illegible]	1p31.1	.
1	90180431	C	T	TGAAATCCTC,CTCCTGAACT	exonic	LRRC8C	nonsynonymous SNV	LRRC8C:ENST00000370454.4:exon3:c.C2302T:p.P768S,	1p22.2	L[illegible]
1	99771334	C	T	CAATCAAGAT,CCAACCGACT	exonic	LPPR4	nonsynonymous SNV	LPPR4:ENST00000370185.3:exon7:c.C1060T:p.P354S,LPPR4:ENST00000370184.1:exon4:c.C586T:p.P196S,LPPR4:ENST00000457765.1:exon6:c.C886T:p.P296S,	1p21.2	.
1	100696327	C	T	TAATGGCTTC,CCACATAGGC	exonic	DBT	nonsynonymous SNV	DBT:ENST00000370131.3:exon4:c.G395A:p.G132E,DBT:ENST00000370132.4:exon4:c.G395A:p.G132E,	1p21.2	.
1	100696328	C	T	AATGGCTTCC,CACATAGGCA	exonic	DBT	nonsynonymous SNV	DBT:ENST00000370131.3:exon4:c.G394A:p.G132R,DBT:ENST00000370132.4:exon4:c.G394A:p.G132R,	1p21.2	.
1	109730875	C	G	CTGCCTGCCT,TGGTGTGAAG	exonic	KIAA1324	nonsynonymous SNV	KIAA1324:ENST00000457623.2:exon8:c.C968G:p.S323C,KIAA1324:ENST00000529753.1:exon7:c.C857G:p.S286C,KIAA1324:ENST00000369939.3:exon9:c.C1118G:[illegible]	1p13.3	.
1	110086743	G	A	TCCAGAGGAG,AGCTGAGGCT	exonic	GPR61	nonsynonymous SNV	GPR61:ENST00000404129.2:exon2:c.G1099A:p.E367K,GPR61:ENST00000527748.1:exon2:c.G1099A:p.E367K,GPR61:ENST00000469383.2:exon2:c.G1099A:p.E36[illegible]	1p13.3	.
1	110148600	C	T	ACCACTTCGT,ATCTTCCACC	exonic	GNAT2	nonsynonymous SNV	GNAT2:ENST00000351050.3:exon6:c.G712A:p.D238N,	1p13.3	.
1	111861775	G	A	AGCCACTCAG,GATGGGATGC	exonic	CHIA	nonsynonymous SNV	CHIA:ENST00000451398.2:exon9:c.G466A:p.G156R,CHIA:ENST00000369740.1:exon10:c.G949A:p.G317R,CHIA:ENST00000489524.1:exon7:c.G466A:p.G156R,CHI[illegible]	1p13.2	.
1	113464673	C	G	AAGCTGCAAT,AAGCCACAGC	exonic	SLC16A1	nonsynonymous SNV	SLC16A1:ENST00000369626.3:exon3:c.G300C:p.L100F,SLC16A1:ENST00000443580.1:exon3:c.G300C:p.L100F,SLC16A1:ENST00000433570.4:exon4:c.G300C:p.L[illegible]	1p13.2	.
1	114695295	G	A	CGCCGCCAGG,CGCGACACGC	exonic	SYT6	nonsynonymous SNV	SYT6:ENST00000608879.1:exon1:c.C14T:p.A5V,	1p13.2	.
1	114945458	A	G	ATCATATTCA,CTTCTGGCTT	exonic	TRIM33	nonsynonymous SNV	TRIM33:ENST00000450349.2:exon15:c.T1712C:p.V571A,TRIM33:ENST00000369543.2:exon16:c.T2816C:p.V939A,TRIM33:ENST00000358465.2:exon16:c.T2816C:p.[illegible]	1p13.2	T[illegible]
1	118526470	C	T	ACAGCTGTTT,GTTTGCATCT	exonic	SPAG17	nonsynonymous SNV	SPAG17:ENST00000336338.5:exon42:c.G5836A:p.E1946K,SPAG17:ENST00000437255.1:exon12:c.G1276A:p.E426K,	1p12	.
1	120506379	G	A	ATGGTGGCAA,GATCGGGGTC	exonic	NOTCH2	nonsynonymous SNV	NOTCH2:ENST00000256646.2:exon11:c.C1733T:p.P578L,	1p12	.
1	146727559	C	T	GTCACGTTTT,ATGTGCTACT	exonic	CHD1L	nonsynonymous SNV	CHD1L:ENST00000431239.1:exon4:c.C439T:p.H147Y,CHD1L:ENST00000369258.4:exon4:c.C439T:p.H147Y,	1q21.1	.
1	150131377	C	T	ACCCCCAACC,CTGCCCTCCC	exonic	PLEKHO1	nonsynonymous SNV	PLEKHO1:ENST00000025469.6:exon5:c.C787T:p.P263S,PLEKHO1:ENST00000369124.4:exon6:c.C889T:p.P297S,PLEKHO1:ENST00000369126.1:exon5:c.C340T:p.[illegible]	1q21.2	.
1	150483540	C	G	GGTATATGGT,CCTGGAACCT	exonic	ECM1	nonsynonymous SNV	ECM1:ENST00000369047.4:exon6:c.C574G:p.P192A,ECM1:ENST00000369049.4:exon6:c.C655G:p.P219A,ECM1:ENST00000346569.6:exon6:c.C574G:p.P192A,	1q21.3	.
1	150483541	C	T	GTATATGGTC,CTGGAACCTA	exonic	ECM1	nonsynonymous SNV	ECM1:ENST00000369047.4:exon6:c.C575T:p.P192L,ECM1:ENST00000369049.4:exon6:c.C656T:p.P219L,ECM1:ENST00000346569.6:exon6:c.C575T:p.P192L,	1q21.3	.
1	150799069	C	G	TGACAGAATT,TACAATATTC	exonic	ARNT	nonsynonymous SNV	ARNT:ENST00000515192.1:exon14:c.G1150C:p.E384Q,ARNT:ENST00000358595.5:exon13:c.G1192C:p.E398Q,ARNT:ENST00000354396.2:exon13:c.G1192C:p.E3[illegible]	1q21.3	.
1	151338136	G	A	GTCGTCCAGG,AGAGCAGGAT	exonic	SELENBP1	nonsynonymous SNV	SELENBP1:ENST00000435071.1:exon9:c.C755T:p.S252F,SELENBP1:ENST00000447402.3:exon8:c.C761T:p.S254F,SELENBP1:ENST00000368868.5:exon9:c.C947[illegible]	1q21.3	.
1	152058158	G	A	TATTTCCAGG,ACTTTCTATT	exonic	TCHHL1	nonsynonymous SNV	TCHHL1:ENST00000368806.1:exon3:c.C2000T:p.S667F,	1q21.3	.
1	152058489	C	T	CTATTGCCTT,CTCTGAAGCC	exonic	TCHHL1	nonsynonymous SNV	TCHHL1:ENST00000368806.1:exon3:c.G1669A:p.E557K,	1q21.3	.
1	152276254	C	T	ACGTCCAGAC,TTCCTGCTGA	exonic	FLG	nonsynonymous SNV	FLG:ENST00000368799.1:exon3:c.G11108A:p.R3703K,	1q21.3	.
1	152323252	C	T	TGATCCATGC,TTTGCCTTTC	exonic	FLG2	nonsynonymous SNV	FLG2:ENST00000388718.5:exon3:c.G7010A:p.R2337K,	1q21.3	.
1	152329342	C	T	AAATTGATTT,CTTGCCCTCC	exonic	FLG2	nonsynonymous SNV	FLG2:ENST00000388718.5:exon3:c.G920A:p.G307E,	1q21.3	.
1	153907418	G	A	TGTGCCAGAC,GCCACTTGCT	exonic	DENND4B	nonsynonymous SNV	DENND4B:ENST00000361217.4:exon18:c.C2591T:p.P864L,DENND4B:ENST00000368646.2:exon18:c.C2624T:p.P875L,	1q21.3	.
1	153955735	G	A	ATTCTCGAAA,ATTTCTCATC	exonic	RAB13	nonsynonymous SNV	RAB13:ENST00000368575.3:exon4:c.C284T:p.S95F,	1q21.3	.
1	154234074	C	T	TTAGGTGGCT,CTACCTCTTC	exonic	UBAP2L	nonsynonymous SNV	UBAP2L:ENST00000271877.7:exon24:c.C2836T:p.P946S,UBAP2L:ENST00000428931.1:exon24:c.C2803T:p.P935S,UBAP2L:ENST00000343815.6:exon24:c.C2803T[illegible]	1q21.3	.
1	155029728	G	A	CCTCCTGGAT,GAATGGGCAG	exonic	ADAM15	nonsynonymous SNV	[illegible]	1q22	.
1	155168290	C	T	TTGTCATCAT,CCCATCACAC	exonic	THBS3	nonsynonymous SNV	[illegible]	1q22	.
1	155223717	G	A	CCCCGACTCT,GACAGGGCCG	exonic	FAM189B	nonsynonymous SNV	[illegible]	1q22	.
1	155223718	G	A	CCCGACTCTG,ACAGGGCCGG	exonic	FAM189B	nonsynonymous SNV	[illegible]	1q22	.
1	155247487	C	T	GGCCTCAGGT,CGATCCCCAA	exonic	HCN3	nonsynonymous SNV	[illegible]	1q22	.
1	155261625	G	A	TAAAGCAAGG,GAAGACTCCT	exonic	PKLR	nonsynonymous SNV	[illegible]	1q22	.
1	156347189	C	T	TCACCTTCCT,CTGGCCGCCT	exonic	RHBG	nonsynonymous SNV	[illegible]	1q22	R[illegible]
1	156779559	G	C	TCCAAAGTTT,ATTCTTCGGT	exonic	SH2D2A	stopgain	[illegible]	1q23.1	.
1	156849818	C	T	GCTGCCCATT,GCTGGATGCC	exonic	NTRK1	nonsynonymous SNV	[illegible]	1q23.1	N[illegible]
1	157665406	G	A	AACTGAAGGA,ACAAAAGGGC	exonic	FCRL3	nonsynonymous SNV	[illegible]	1q23.1	.
1	158262105	G	A	ACTTGCCCCC,ATTTCTCTTG	exonic	CD1C	nonsynonymous SNV	[illegible]	1q23.1	C[illegible]
1	158532857	C	T	GGGGAAATAT,ACAGAAAAAG	exonic	OR6P1	nonsynonymous SNV	[illegible]	1q23.1	.
1	158669797	A	C	TAGGACATGA,GATGAGCATC	exonic	OR6K2	nonsynonymous SNV	[illegible]	1q23.1	.
1	158746540	C	T	TTAATGATTT,CTTGTTACGA	exonic	OR6N2	nonsynonymous SNV	[illegible]	1q23.1	.
1	159779306	C	T	ATCCTGTATT,CTTCTACCTT	exonic	FCRL6	nonsynonymous SNV	[illegible]	1q23.2	F[illegible]
1	159907601	G	A	AATCTGGAGA,AGGCCCCCTT	exonic	IGSF9	nonsynonymous SNV	[illegible]	1q23.2	.
1	160105251	G	A	GACGGGTGAC,GGGTGAACGA	exonic	ATP1A2	nonsynonymous SNV	[illegible]	1q23.2	.
1	160105252	G	A	ACGGGTGACG,GGTGAACGAC	exonic	ATP1A2	nonsynonymous SNV	[illegible]	1q23.2	.
1	161141846	C	G	CATCTTGCGT,CAGGAATTCT	exonic	B4GALT3	nonsynonymous SNV	[illegible]	1q23.3	.
1	162326814	C	T	TCTTCTTCCT,GAAGCCTCCA	exonic	NOS1AP	nonsynonymous SNV	[illegible]	1q23.3	.
1	162743361	C	T	GAAAATGCTC,GAGCAGATGC	exonic	DDR2	stopgain	[illegible]	1q23.3	.
1	163043359	C	G	TAAACTAAGT,CCAAGGCCAA	exonic	RGS4	nonsynonymous SNV	[illegible]	1q23.3	.
1	167096307	G	A	GGCAAGCTGG,AGGCGGACAG	exonic	DUSP27	nonsynonymous SNV	[illegible]	1q24.1	.
1	171168490	G	C	TGAAGGTATG,AGAGGTTCAA	exonic	FMO2	nonsynonymous SNV	[illegible]	1q24.3	.
1	172501880	G	A	ACGAATTCTG,AAGGCAAACT	exonic	SUCO	nonsynonymous SNV	[illegible]	1q24.3	.
1	180885448	C	T	CCCCAGCCTC,CAGGGTATGG	exonic	KIAA1614	nonsynonymous SNV	[illegible]	1q25.3	.
1	181762838	C	T	GTGCAGCCCT,TAACCATGGC	exonic	CACNA1E	nonsynonymous SNV	[illegible]	1q25.3	.
1	183094583	C	T	AGCTGTAACC,CGTGACGGGG	exonic	LAMC1	nonsynonymous SNV	[illegible]	1q25.3	.
1	196309636	G	A	ATGTATCGAG,ACCTGGATTC	exonic	KCNT2	nonsynonymous SNV	[illegible]	1q31.3	.
1	196459061	C	T	CAGGCGTATC,TTAGACCTTT	exonic	KCNT2	nonsynonymous SNV	[illegible]	1q31.3	.
1	196642275	C	T	TGCTCTTAAT,CATTAAGGAA	exonic	CFH	nonsynonymous SNV	[illegible]	1q31.3	.
1	197091345	C	T	GCAGGTATTC,ACCAAGGTCT	exonic	ASPM	nonsynonymous SNV	[illegible]	1q31.3	.
1	200378275	G	T	TGGGCTGGTT,CTGCTGGACA	exonic	ZNF281	nonsynonymous SNV	[illegible]	1q32.1	.
1	200946373	G	A	GGCATTGCCC,AGGCGGCGTA	exonic	KIF21B	nonsynonymous SNV	[illegible]	1q32.1	.
1	201178258	G	A	TCATAGGAAT,GGATTGGAGG	exonic	IGFN1	nonsynonymous SNV	[illegible]	1q32.1	.
1	201180045	A	G	CTGAGGGAAT,GGTTCAGGGA	exonic	IGFN1	nonsynonymous SNV	[illegible]	1q32.1	.
1	201182392	C	T	GGTCCAGGGT,CTGGGGCCCT	exonic	IGFN1	nonsynonymous SNV	[illegible]	1q32.1	.
1	201779231	A	G	GTCTCCCTCA,AGGTCAGTCT	exonic	NAV1	nonsynonymous SNV	[illegible]	1q32.1	.
1	204125434	A	G	AAGGTGGATG,CCCCACAGAC	exonic	REN	nonsynonymous SNV	[illegible]	1q32.1	.
1	214170675	C	T	AGCACTGATT,GGAAAATGAT	exonic	PROX1	nonsynonymous SNV	[illegible]	1q32.3	.
1	215814047	C	T	AAATTGCTGT,CACCGAAAAT	exonic	USH2A	nonsynonymous SNV	[illegible]	1q41	.
1	216219791	C	T	ATGTAGTTCT,CTCACTGCCT	exonic	USH2A	nonsynonymous SNV	[illegible]	1q41	.
1	216246248	C	T	TGTTGTACGT,CTCGACTCCA	exonic	USH2A	nonsynonymous SNV	[illegible]	1q41	.
1	225226482	C	T	CATGCTATTT,TGTTCAAAAG	exonic	DNAH14	nonsynonymous SNV	[illegible]	1q42.12	.
1	225305630	G	C	TATTGATATG,AAACAAGACA	exonic	DNAH14	nonsynonymous SNV	[illegible]	1q42.12	.
1	225541558	G	A	TTTTCCCTGG,AGAAACTCAC	exonic	DNAH14	nonsynonymous SNV	[illegible]	1q42.12	.
1	226736688	C	T	GCGTCCTCGT,CAGCGGGGTG	exonic	C1orf95	nonsynonymous SNV	[illegible]	1q42.12	.
1	227335080	C	T	ATGATTTTTC,GTATGTCTCC	exonic	CDC42BPA	nonsynonymous SNV	[illegible]	1q42.13	.
1	230846479	G	A	AGGTGGAAGG,GTGTATGTAC	exonic	AGT	nonsynonymous SNV	[illegible]	1q42.2	.
1	231003991	C	T	CGCGTGAGCT,CTCCGAGTCC	exonic	C1orf198	nonsynonymous SNV	[illegible]	1q42.2	.
1	231829706	C	T	GTTCCGGTTC,CAGGAGGGGT	exonic	DISC1	nonsynonymous SNV	[illegible]	1q42.2	.
1	237052553	G	A	TACATTGACT,GAAGCCTTTC	exonic	MTR	stopgain	[illegible]	1q43	.
1	237052554	G	A	ACATTGACTG,AAGCCTTTCT	exonic	MTR	stopgain	[illegible]	1q43	.
1	237850796	C	T	CATAGGATTT,ACTATTTGGT	exonic	RYR2	nonsynonymous SNV	[illegible]	1q43	.
1	237893594	G	A	TCAGGGATAT,AAAAGTCTTG	exonic	RYR2	nonsynonymous SNV	[illegible]	1q43	.
1	237895370	G	A	ACCTCCAGAA,AAGATGAAGG	exonic	RYR2	nonsynonymous SNV	[illegible]	1q43	.
1	237957249	C	T	GAACAGCCTT,AGAAGATGAT	exonic	RYR2	nonsynonymous SNV	[illegible]	1q43	.
1	238053456	C	T	TTCTGCAGCT,GTGCAGCAGC	exonic	ZP4	nonsynonymous SNV	[illegible]	1q43	.
1	240256734	G	A	CAGAGCCCCA,GATCAAGAGG	exonic	FMN2	nonsynonymous SNV	[illegible]	1q43	.
1	241714342	C	T	GACAAAGTCT,AGGTAGGTTT	exonic	KMO	stopgain	[illegible]	1q43	K[illegible]
1	241797328	C	G	AGGCCACAGT,AGGCCCAGAG	exonic	CHML	nonsynonymous SNV	[illegible]	1q43	.
1	241834427	G	T	CCAGTTGGAT,AAGAAAATTT	exonic	WDR64	stopgain	[illegible]	1q43	.
1	241834428	A	G	CAGTTGGATG,AGAAAATTTG	exonic	WDR64	nonsynonymous SNV	[illegible]	1q43	.
1	245020994	C	T	TTTTCCAGCT,CTGGCAAGCC	exonic	HNRNPU	nonsynonymous SNV	[illegible]	1q44	.
1	247039401	C	T	GGCTAATTTT,GATGGACTCT	exonic	AHCTF1	nonsynonymous SNV	[illegible]	1q44	.
1	247921510	C	T	TCAACAAAGG,CAAGTTACTA	exonic	OR1C1	nonsynonymous SNV	[illegible]	1q44	.
1	248028101	T	C	CTGAGGCGGC,GGAGGCGGAG	exonic	TRIM58	nonsynonymous SNV	[illegible]	1q44	.
1	248059237	G	T	GCTTCTGGCT,TCATGGCCTA	exonic	OR2W3	nonsynonymous SNV	[illegible]	1q44	.
1	248263470	C	T	CAGGAATCTC,GCTCACCAGC	exonic	OR2L13	nonsynonymous SNV	[illegible]	1q44	O[illegible]
1	248651929	T	A	CACTGGAAAG,TGGATTTCAT	exonic	OR2T5	nonsynonymous SNV	[illegible]	1q44	.
2	1079234	G	A	GTTGTATGAT,AAGAGTCCGA	exonic	SNTG2	nonsynonymous SNV	[illegible]	2p25.3	.
2	1481077	G	A	GACCAGTGCC,AAGGGCTGCT	exonic	TPO	nonsynonymous SNV	[illegible]00345913.4:exon8:c.G1039A:p.E347K,TPO:ENST00000337415.3:exon8:c.G1039A:p.E[illegible]	2p25.3	.
2	1652070	C	T	GTCCCGGCCC,GCTGGATGTT	exonic	PXDN	nonsynonymous SNV	[illegible]	2p25.3	P[illegible]
2	5832878	G	A	GGAGAGCTTG,AAGCGGAGAG	exonic	SOX11	nonsynonymous SNV	[illegible]	2p25.2	.
2	11361406	C	T	GTTTCTACAT,TCCTTTGTCA	exonic	ROCK2	nonsynonymous SNV	[illegible]	2p25.1	.
2	21230175	G	A	ACAGCCAAAG,ATTTGTGATG	exonic	APOB	nonsynonymous SNV	[illegible]	2p24.1	.
2	23785114	G	A	GGGTGGGCTG,GACCGCCGCG	exonic	KLHL29	stopgain	[illegible]	2p24.1	.
2	24964827	C	T	CCCCCGTCTT,CTCAGGGTGC	exonic	NCOA1	nonsynonymous SNV	[illegible]P1160S,NCOA1:ENST00000538539.1:exon18:c.C3478T:p.P1160S,NCOA1:ENST0000[illegible]	2p23.3	.
2	25656854	G	A	CAAGTCAGTG,GAGGACGAGT	exonic	DTNB	nonsynonymous SNV	[illegible]:ENST00000405222.1:exon12:c.C1178T:p.P393L,DTNB:ENST00000406818.3:exon13[illegible]	2p23.3	.
2	25656855	G	A	AAGTCAGTGG,AGGACGAGTC	exonic	DTNB	nonsynonymous SNV	[illegible]B:ENST00000405222.1:exon12:c.C1177T:p.P393S,DTNB:ENST00000406818.3:exon1[illegible]	2p23.3	.
2	25967335	G	A	GGAGATTCTG,AGACCGGGAT	exonic	ASXL2	nonsynonymous SNV	[illegible]	2p23.3	.
2	25990469	G	T	GAGTCTCTCA,ATCTCTGGAA	exonic	ASXL2	nonsynonymous SNV	[illegible]	2p23.3	.
2	26690349	C	T	TTCTCCTTGC,CTTCATTGAC	exonic	OTOF	nonsynonymous SNV	[illegible]S,OTOF:ENST00000402415.3:exon16:c.G2041A:p.G681S,	2p23.3	.
2	27157521	C	T	CAGGACTGGT,CCACGCGGCT	exonic	DPYSL5	nonsynonymous SNV	[illegible]	2p23.3	.
2	27597016	G	A	TTCTTAGTTC,AGAAAAAGAG	exonic	SNX17	nonsynonymous SNV	[illegible]	2p23.3	.
2	27663772	C	T	TGTCAAGACT,CGACACCTGA	exonic	NRBP1	nonsynonymous SNV	[illegible]	2p23.3	.
2	27822481	G	C	AGGGGAAAAG,AAACCCAGGC	exonic	ZNF512	nonsynonymous SNV	[illegible]12:ENST00000556601.1:exon3:c.G41C:p.G14A,	2p23.3	.
2	27824259	C	T	GAAACAACTT,GTTCACTGGC	exonic	ZNF512	nonsynonymous SNV	[illegible]512:ENST00000556601.1:exon5:c.C226T:p.R76C,	2p23.3	.
2	29296871	C	T	CATATCTTTC,TTTTGCCTGA	exonic	C2orf71	nonsynonymous SNV	[illegible]	2p23.2	C[illegible]
2	33745717	G	A	ACTAGGATAT,AAAAACACGT	exonic	RASGRP3	nonsynonymous SNV	[illegible]112K,RASGRP3:ENST00000403687.3:exon6:c.G334A:p.E112K,RASGRP3:ENST0000[illegible]	2p22.3	.
2	37374868	G	A	GGCAGTTCTT,ATATTTAAGT	exonic	EIF2AK2	stopgain	[illegible]NST00000395127.2:exon3:c.C82T:p.Q28X,	2p22.2	.
2	39089456	C	T	TCATCTTCCT,CTCCCCAGAA	exonic	DHX57	nonsynonymous SNV	[illegible]	2p22.1	.
2	39440602	C	T	TTTGATCACT,CATCAGCAAC	exonic	CDKL4	nonsynonymous SNV	[illegible]	2p22.1	.
2	40656766	A	C	AAAATAATGT,AAGCCAGGTG	exonic	SLC8A1	nonsynonymous SNV	[illegible]C8A1:ENST00000406391.2:exon1:c.T655G:p.Y219D,SLC8A1:ENST00000407929.2:ex[illegible]	2p22.1	.
2	42924928	G	C	ACGTCTAAAA,CAGCAGAAGC	exonic	MTA3	nonsynonymous SNV	[illegible]ENST00000405094.1:exon11:c.G979C:p.A327P,MTA3:ENST00000454356.1:exon11:c.[illegible]	2p21	.
2	43452515	C	T	GCCGCCGCCC,CCTTCTGCTG	exonic	ZFP36L2	nonsynonymous SNV	[illegible]	2p21	.
2	48035283	C	G	GTATTAGATT,TATAGGTGGA	exonic	FBXO11	nonsynonymous SNV	[illegible]p.E344Q,FBXO11:ENST00000434234.1:exon3:c.G220C:p.E74Q,	2p16.3	.
2	49195877	G	A	CAATGGCTGG,ATAGGTGAGG	exonic	FSHR	nonsynonymous SNV	[illegible]	2p16.3	.
2	51254771	C	T	GCACGGGCTT,CCCCGCCGCT	exonic	NRXN1	nonsynonymous SNV	[illegible]N1:ENST00000405472.3:exon2:c.G641A:p.G214E,NRXN1:ENST00000402717.3:exon1[illegible]	2p16.3	.
2	54609049	G	A	GGAAGAAAAG,AACAGAGAGA	exonic	C2orf73	nonsynonymous SNV	[illegible]	2p16.2	.
2	55179825	G	C	CCACCAGCCA,ATAGGTAGGA	exonic	EML6	nonsynonymous SNV	[illegible]	2p16.1	.
2	55826000	C	T	CTTTTCCCTA,GGATAGGTGA	exonic	SMEK2	nonsynonymous SNV	[illegible]	2p16.1	.
2	56420458	G	A	TCACAAACAC,GAGGAGGCAG	exonic	CCDC85A	nonsynonymous SNV	[illegible]	2p16.1	C[illegible]
2	56420459	G	A	CACAAACACG,AGGAGGCAGT	exonic	CCDC85A	nonsynonymous SNV	[illegible]	2p16.1	.
2	61194725	C	T	TATATCATCT,TTCCCAGCGA	exonic	PUS10	nonsynonymous SNV	[illegible]	2p16.1	.
2	61319723	G	A	ATGTTATCAG,TAAGATTTTT	splicing	KIAA1841(EN		.	2p15	.
2	68882609	G	C	TGCTCCACTG,AAGGCTTCTT	exonic	PROKR1	nonsynonymous SNV	[illegible]	2p13.3	.
2	70046451	A	T	TTTGCTGAAA,GCTCTATAAA	exonic	ANXA4	nonsynonymous SNV	[illegible]	2p13.3	.
2	71576751	C	T	CACAGAAGAT,CACTTGAAGT	exonic	ZNF638	nonsynonymous SNV	[illegible]	2p13.2	.
2	71654152	C	T	GTAAGGGATT,CATTGGCTTC	exonic	ZNF638	nonsynonymous SNV	[illegible]	2p13.2	.
2	73717163	G	A	GCCACCTAAA,AAGTGGATTT	exonic	ALMS1	nonsynonymous SNV	[illegible]	2p13.1	A[illegible]
2	74141810	A	G	CTTCCAGCTG,GAGAGAAATT	exonic	ACTG2	nonsynonymous SNV	[illegible]	2p13.1	.
2	74320708	G	A	AAGGAAGGAC,GCCCTTCGCG	exonic	TET3	nonsynonymous SNV	[illegible]	2p13.1	.
2	84852068	C	T	GCAGCTTGAC,TTGGGGGTGC	exonic	DNAH6	nonsynonymous SNV	[illegible]	2p11.2	.
2	85014391	G	A	TGTAAAGAAG,AAAGATTCCC	exonic	DNAH6	nonsynonymous SNV	[illegible]	2p11.2	.
2	88828927	C	T	TCCCTGTCTT,ATTGCCATAT	exonic	TEX37	nonsynonymous SNV	[illegible]	2p11.2	C[illegible]
2	96948957	C	T	AAGAGCTGCT,CACCAGGCGT	exonic	SNRNP200	nonsynonymous SNV	[illegible]	2q11.2	.
2	98280351	C	T	CGTAGGACTC,ATGGCCGGGC	exonic	ACTR1B	nonsynonymous SNV	[illegible]	2q11.2	.
2	99870708	C	T	CCCCAAAACA,CACGGAGGAT	exonic	LYG2	nonsynonymous SNV	[illegible]1:exon1:c.G16A:p.V6M,	2q11.2	.
2	100368763	C	G	CAGGTCATCT,CTGAAGGACG	exonic	AFF3	nonsynonymous SNV	[illegible]	2q11.2	.
2	107459497	C	T	CTACCTATTT,CTCGCCCAAG	exonic	ST6GAL2	nonsynonymous SNV	[illegible]	2q12.2	.
2	107459979	G	A	CTCCCCGGGG,AAGGGAATCC	exonic	ST6GAL2	nonsynonymous SNV	[illegible]	2q12.2	.
2	113890327	C	T	AGTTTTGAGT,TGCCGCCTGC	exonic	IL1RN	nonsynonymous SNV	[illegible]00000409930.3:exon4:c.C413T:p.S138F,	2q13	.
2	120003277	C	T	CAGGCTGTTT,CCTCAGCGGC	exonic	STEAP3	nonsynonymous SNV	[illegible]:ENST00000425223.2:exon2:c.C205T:p.P69S,STEAP3:ENST00000393108.2:exon2:c.[illegible]	2q14.2	.
2	120385290	C	G	CCAGCCGGTT,TCTGTGTCGC	exonic	PCDP1	nonsynonymous SNV	[illegible]	2q14.2	.
2	120720235	C	G	GGCAGTTCAT,TTATGGATGC	exonic	PTPN4	nonsynonymous SNV	[illegible]	2q14.2	.
2	125504829	A	T	TACCTGGTGG,TTGGGCGGTC	exonic	CNTNAP5	nonsynonymous SNV	[illegible]	2q14.3	.
2	128477291	G	A	TGAGACACAG,ACCTTGGATC	exonic	WDR33	nonsynonymous SNV	[illegible]	2q14.3	.
2	128935485	C	T	TTTTGGGATT,CTTCAAATGG	exonic	UGGT1	nonsynonymous SNV	[illegible]	2q14.3	.
2	131128305	G	C	CAGTCTCTTT,ATGTGGTCCT	exonic	PTPN18	nonsynonymous SNV	[illegible]	2q21.1	P[illegible]
2	133539940	C	T	TCCACATTTT,CTGAATGCAC	exonic	NCKAP5	nonsynonymous SNV	[illegible]	2q21.2	.
2	136566366	G	A	TGGCAGGCGG,AGGTGGCTAA	exonic	LCT	nonsynonymous SNV	[illegible]	2q21.3	.
2	141215031	C	T	AGAATAATTA,CATCAGGTTG	splicing	LRP1B(ENST		.	2q22.1	.
2	142012102	C	T	TTTACAGCTT,TCCCATCTTC	exonic	LRP1B	nonsynonymous SNV	[illegible]	2q22.1	.
2	153525653	G	A	TCTTTTACCT,GAAAGATTCC	exonic	PRPF40A	stopgain	[illegible]	2q23.3	.
2	162224352	G	A	GATGCTTGGA,AATTTGTTGA	exonic	PSMD14	nonsynonymous SNV	[illegible]	2q24.2	.
2	164466975	G	A	CTCGTCCACA,AGTGGTTGGA	exonic	FIGN	nonsynonymous SNV	[illegible]	2q24.3	.
2	167056085	C	T	ACAGGCAAAT,ATACTGTTGC	exonic	SCN9A	nonsynonymous SNV	[illegible]	2q24.3	.
2	167085261	C	T	TCAGGTTTTT,CATCGCACAT	exonic	SCN9A	stopgain	[illegible]	2q24.3	.
2	167159672	G	A	AGTGAATTTC,AAAACATTTA	exonic	SCN9A	stopgain	[illegible]	2q24.3	.
2	168103073	C	T	TTGTCTTCCA,ATCAAACAAT	exonic	XIRP2	nonsynonymous SNV	[illegible]	2q24.3	.

2	170034408	C	T	ATATTTGTTT,CCTTTTCCAC	exonic	LRP2	nonsynonymous SNV	LRP2:ENST00000263816.3:exon53:c.G10298A:p.G3433E,	2q31.1	.
2	170058204	G	C	ATAAACTCAC,AGGTATGCAC	exonic	LRP2	nonsynonymous SNV	LRP2:ENST00000263816.3:exon44:c.C8386G:p.R2796G,	2q31.1	.
2	170136918	C	T	GAAAGCCACA,CCACGGCCAC	exonic	LRP2	nonsynonymous SNV	LRP2:ENST00000263816.3:exon11:c.G1283A:p.G428D,LRP2:ENST00000443831.1:exon11:c.G1283A:p.G428D,	2q31.1	.
2	171243677	G	T	GAAGCCTTTG,GAACTCATGC	exonic	MYO3B	nonsynonymous SNV	MYO3B:ENST00000409044.3:exon14:c.G1436T:p.G479V,MYO3B:ENST00000408978.4:exon14:c.G1436T:p.G479V,MYO3B:ENST00000484338.2:exon14:c.G1463T:p.G488V,MYO3B:ENST00000317935.6:exon14:c.G1436T:p.G479V,MYO3B:ENST00000334231.6:exon14:c.G1463T:p.G488V,	2q31.1	.
2	171687469	C	T	GATCTGCTTC,GGCTAAGAAC	exonic	GAD1	nonsynonymous SNV	GAD1:ENST00000456864.1:exon5:c.C314T:p.P105L,GAD1:ENST00000358196.3:exon5:c.C314T:p.P105L,GAD1:ENST00000414527.2:exon5:c.C314T:p.P105L,GAD1:ENST00000493875.1:exon4:c.C314T:p.P105L,GAD1:ENST00000375272.1:exon5:c.C314T:p.P105L,GAD1:ENST00000344257.5:exon5:c.C314T:p.P	2q31.1	.
2	179413990	C	T	ATACTCATTG,CTTCTGTCAG	exonic	TTN	nonsynonymous SNV	TTN:ENST00000359218.5:exon168:c.G65543A:p.G21848D,TTN:ENST00000589042.1:exon339:c.G92363A:p.G30788D,TTN:ENST00000591111.1:exon289:c.G87440A:p.G29147D,TTN:ENST00000460472.2:exon167:c.G65168A:p.G21723D,TTN:ENST00000342175.6:exon168:c.G65744A:p.G21915D,TTN:ENST000	2q31.2	.
2	179424554	C	T	AATGAGGCAC,AGCCTTGACA	exonic	TTN	nonsynonymous SNV	TTN:ENST00000359218.5:exon155:c.G59485A:p.G19829S,TTN:ENST00000589042.1:exon326:c.G86305A:p.G28769S,TTN:ENST00000591111.1:exon276:c.G81382A:p.G27128S,TTN:ENST00000460472.2:exon154:c.G59110A:p.G19704S,TTN:ENST00000342175.6:exon155:c.G59686A:p.G19896S,TTN:ENST000	2q31.2	.
2	179478493	T	C	TGTTGTGTCC,TTACTGGCAT	exonic	TTN	nonsynonymous SNV	TTN:ENST00000359218.5:exon92:c.A22697G:p.K7566R,TTN:ENST00000589042.1:exon263:c.A49517G:p.K16506R,TTN:ENST00000591111.1:exon213:c.A44594G:p.K14865R,TTN:ENST00000460472.2:exon91:c.A22322G:p.K7441R,TTN:ENST00000342175.6:exon92:c.A22898G:p.K7633R,TTN:ENST0000034299	2q31.2	.
2	179516849	G	A	TTCCGGTTTG,GAGGAATAGC	exonic	TTN	nonsynonymous SNV	TTN:ENST00000589042.1:exon204:c.C39269T:p.P13090L,TTN:ENST00000591111.1:exon159:c.C34748T:p.P11583L,TTN:ENST00000342992.6:exon158:c.C31967T:p.P10656L,	2q31.2	.
2	179565886	T	C	TCTTCGGGTG,TGGTAGCAAA	exonic	TTN	nonsynonymous SNV	TTN:ENST00000414766.1:exon5:c.A277G:p.T93A,TTN:ENST00000589042.1:exon111:c.A30643G:p.T10215A,TTN:ENST00000591111.1:exon109:c.A29692G:p.T9898A,TTN:ENST00000342992.6:exon108:c.A26911G:p.T8971A,	2q31.2	.
2	182542584	G	C	GCTATCGAAG,ACATAATATT	exonic	NEUROD1	nonsynonymous SNV	NEUROD1:ENST00000295108.3:exon2:c.C1004G:p.S335C,	2q31.3	.
2	186667578	G	A	ATATTAGGAT,AAAGTTTCCC	exonic	FSIP2	nonsynonymous SNV	FSIP2:ENST00000343098.5:exon17:c.G13812A:p.M4604I,FSIP2:ENST00000424728.1:exon17:c.G13545A:p.M4515I,	2q32.1	.
2	187360029	C	T	TGTCACACAT,AAGTTAAATT	exonic	ZC3H15	stopgain	ZC3H15:ENST00000337859.6:exon2:c.C145T:p.Q49X,ZC3H15:ENST00000437396.1:exon2:c.C145T:p.Q49X,ZC3H15:ENST00000421536.1:exon2:c.C145T:p.Q49X,	2q32.1	.
2	187627164	C	T	CAAGAGAACC,AGAGCAATGA	exonic	FAM171B	stopgain	FAM171B:ENST00000304698.5:exon8:c.C2095T:p.Q699X,	2q32.1	.
2	189917689	C	T	ACCAGCATCT,CCTTCTGTCC	exonic	COL5A2	nonsynonymous SNV	COL5A2:ENST00000374866.3:exon39:c.G2609A:p.G870E,	2q32.2	.
2	190332278	C	T	GAAGATGGTT,TTTACTAGCA	exonic	WDR75	nonsynonymous SNV	WDR75:ENST00000314761.4:exon14:c.C1532T:p.S511F,	2q32.2	W
2	190719778	C	T	CATAGAAAAT,CTAAGACTAG	exonic	PMS1	nonsynonymous SNV	PMS1:ENST00000409823.3:exon8:c.C1663T:p.P555S,PMS1:ENST00000447232.2:exon9:c.C1780T:p.P594S,PMS1:ENST00000418224.3:exon8:c.C1252T:p.P418S,PMS1:ENST00000450931.1:exon9:c.C1663T:p.P555S,PMS1:ENST00000424307.1:exon7:c.C1597T:p.P533S,PMS1:ENST00000432292.3:exon7:c.C1	2q32.2	.
2	191765297	C	T	CAGATGTGTT,AGAGCAACAT	exonic	GLS	stopgain	GLS:ENST00000338435.4:exon4:c.C613T:p.Q205X,GLS:ENST00000320717.3:exon4:c.C613T:p.Q205X,	2q32.2	.
2	196661454	C	T	TTTTGATCCC,CATATCCCTG	exonic	DNAH7	nonsynonymous SNV	DNAH7:ENST00000312428.6:exon56:c.G10361A:p.G3454E,	2q32.3	.
2	196791250	C	T	CCAGTTAATT,GTTCATATTT	exonic	DNAH7	nonsynonymous SNV	DNAH7:ENST00000312428.6:exon22:c.G3512A:p.R1171Q,	2q32.3	DI
2	197540918	G	A	CTTATTGCTG,ATGCAGCCCA	exonic	CCDC150	nonsynonymous SNV	CCDC150:ENST00000389175.4:exon11:c.G1189A:p.D397N,CCDC150:ENST00000423093.2:exon5:c.G193A:p.D65N,CCDC150:ENST00000272831.7:exon7:c.G193A:p.D65N,	2q33.1	.
2	198950762	C	T	GCACGTAACC,TTTTTGTCCA	exonic	PLCL1	nonsynonymous SNV	PLCL1:ENST00000428675.1:exon2:c.C2521T:p.L841F,PLCL1:ENST00000487695.2:exon2:c.C2299T:p.L767F,PLCL1:ENST00000437704.2:exon1:c.C2227T:p.L743F,	2q33.1	.
2	202957827	C	T	ACAACAAGAT,TTTCAGGTGT	exonic	AC079354.1	nonsynonymous SNV	AC079354.1:ENST00000295844.3:exon5:c.C772T:p.L258F,AC079354.1:ENST00000541917.1:exon4:c.C604T:p.L202F,	2q33.1	.
2	202957828	T	A	CAACAAGATC,TTCAGGTGTA	exonic	AC079354.1	nonsynonymous SNV	AC079354.1:ENST00000295844.3:exon5:c.T773A:p.L258H,AC079354.1:ENST00000541917.1:exon4:c.T605A:p.L202H,	2q33.1	.
2	207175689	T	A	AAAAGAAATT,CCAGCTAACA	exonic	ZDBF2	nonsynonymous SNV	ZDBF2:ENST00000374423.3:exon5:c.T6437A:p.F2146Y,	2q33.3	.
2	210704125	C	T	CGCAGAATTT,CCTCCGAAAG	exonic	UNC80	nonsynonymous SNV	UNC80:ENST00000439458.1:exon19:c.C3221T:p.S1074F,UNC80:ENST00000272845.6:exon19:c.C3206T:p.S1069F,	2q34	.
2	210791584	G	A	ACATACCACT,AACACACGCC	exonic	UNC80	nonsynonymous SNV	UNC80:ENST00000439458.1:exon35:c.G5482A:p.E1828K,UNC80:ENST00000272845.6:exon35:c.G5467A:p.E1823K,	2q34	.
2	211464199	C	T	CTTCCCATCA,CCCTCAGTTT	exonic	CPS1	nonsynonymous SNV	CPS1:ENST00000233072.5:exon14:c.C1463T:p.T488I,CPS1:ENST00000430249.2:exon15:c.C1481T:p.T494I,CPS1:ENST00000451903.2:exon4:c.C110T:p.T37I,	2q34	.
2	216279672	C	T	TTCGACAGGA,CACTTGAGCC	exonic	FN1	nonsynonymous SNV	FN1:ENST00000426059.1:exon13:c.G1829A:p.G610D,FN1:ENST00000336916.4:exon13:c.G1829A:p.G610D,FN1:ENST00000432072.2:exon13:c.G1829A:p.G610D,FN1:ENST00000346544.3:exon13:c.G1829A:p.G610D,FN1:ENST00000356005.4:exon13:c.G1829A:p.G610D,FN1:ENST00000443816.1:exon13:c.G1	2q35	.
2	217026765	G	A	GGCCAGCTTT,AGGAAGGTGA	exonic	XRCC5	nonsynonymous SNV	XRCC5:ENST00000392132.2:exon16:c.G1828A:p.E610K,XRCC5:ENST00000392133.3:exon18:c.G1828A:p.E610K,	2q35	.
2	219000234	C	T	ACCCTGCGTA,GCTGTTTAAG	exonic	CXCR2	nonsynonymous SNV	CXCR2:ENST00000318507.2:exon3:c.C710T:p.T237M,	2q35	C
2	219507149	G	A	AGTCGGCTAG,GAAGGCCTCC	exonic	ZNF142	nonsynonymous SNV	ZNF142:ENST00000449707.1:exon8:c.C4090T:p.P1364S,ZNF142:ENST00000411696.2:exon7:c.C4090T:p.P1364S,	2q35	.
2	219892328	C	T	ACTCAGGCTC,TAGCAGAGAC	exonic	CCDC108	nonsynonymous SNV	CCDC108:ENST00000410037.1:exon12:c.G2060A:p.R687K,CCDC108:ENST00000409865.3:exon13:c.G2222A:p.R741K,	2q35	.
2	220313955	G	A	CGCAGCCAGG,CAAAGGTCGC	exonic	SPEG	nonsynonymous SNV	SPEG:ENST00000396698.1:exon5:c.G1763A:p.G588D,SPEG:ENST00000312358.7:exon4:c.G2075A:p.G692D,	2q35	.
2	220370718	C	T	CCCAACGTCT,CATCGGGAAG	exonic	GMPPA	nonsynonymous SNV	GMPPA:ENST00000358215.3:exon11:c.C917T:p.S306F,GMPPA:ENST00000313597.5:exon11:c.C917T:p.S306F,GMPPA:ENST00000341142.3:exon11:c.C917T:p.S306F,GMPPA:ENST00000373908.1:exon11:c.C917T:p.S306F,GMPPA:ENST00000373917.3:exon10:c.C1076T:p.S359F,	2q35	.
2	220505608	C	T	CTCGCCTTTC,CTTCCTGCTG	exonic	SLC4A3	nonsynonymous SNV	SLC4A3:ENST00000373760.2:exon22:c.C3545T:p.P1182L,SLC4A3:ENST00000358055.3:exon22:c.C3545T:p.P1182L,SLC4A3:ENST00000373762.3:exon22:c.C3626T:p.P1209L,SLC4A3:ENST00000317151.3:exon22:c.C3545T:p.P1182L,SLC4A3:ENST00000273063.6:exon22:c.C3626T:p.P1209L,	2q35	.
2	227660258	G	A	CATGCCCCCA,GTCCTTGTGG	exonic	IRS1	nonsynonymous SNV	IRS1:ENST00000305123.5:exon1:c.C3197T:p.P1066L,	2q36.3	.
2	227660259	G	A	ATGCCCCCAG,TCCTTGTGGG	exonic	IRS1	nonsynonymous SNV	IRS1:ENST00000305123.5:exon1:c.C3196T:p.P1066S,	2q36.3	.
2	228159778	G	A	GGGAGAAACG,GTACAACTTG	exonic	COL4A3	nonsynonymous SNV	COL4A3:ENST00000396578.3:exon40:c.G3517A:p.G1173S,	2q36.3	.
2	231906116	C	T	TGCAGAAGCT,AGCTCAGGGT	exonic	C2orf72	nonsynonymous SNV	C2orf72:ENST00000373640.4:exon2:c.C740T:p.S247L,	2q37.1	.
2	234102533	G	A	ACCCACCATG,GGAGTTGACA	exonic	INPP5D	nonsynonymous SNV	INPP5D:ENST00000450745.1:exon18:c.G1778A:p.G593E,INPP5D:ENST00000445964.1:exon13:c.G1385A:p.G462E,INPP5D:ENST00000455936.2:exon18:c.G1778A:p.G593E,INPP5D:ENST00000415617.1:exon13:c.G1385A:p.G462E,INPP5D:ENST00000435188.1:exon13:c.G1385A:p.G462E,INPP5D:ENST0000	2q37.1	.
2	234602173	C	T	CAGGGGTTTT,CGTGTTCCCT	exonic	UGT1A6	nonsynonymous SNV	UGT1A6:ENST00000305139.6:exon1:c.C523T:p.P175S,UGT1A6:ENST00000441351.1:exon2:c.C523T:p.P175S,	2q37.1	.
2	241569917	C	T	TTCTACCTGC,CCTGGCCGTG	exonic	GPR35	nonsynonymous SNV	GPR35:ENST00000430267.1:exon1:c.C548T:p.P183L,GPR35:ENST00000319838.5:exon6:c.C548T:p.P183L,GPR35:ENST00000407714.1:exon2:c.C548T:p.P183L,GPR35:ENST00000403859.1:exon6:c.C548T:p.P183L,GPR35:ENST00000438013.2:exon6:c.C641T:p.P214L,	2q37.3	.
2	241682373	G	A	ATGACCCGAG,GAAGTGGCGG	exonic	KIF1A	nonsynonymous SNV	KIF1A:ENST00000320389.7:exon32:c.C3310T:p.P1104S,KIF1A:ENST00000498729.2:exon34:c.C3613T:p.P1205S,KIF1A:ENST00000404283.3:exon33:c.C3613T:p.P1205S,	2q37.3	.
2	241808343	C	T	CCTCTCCATC,CCAACCAGCT	exonic	AGXT	nonsynonymous SNV	AGXT:ENST00000307503.3:exon1:c.C61T:p.P21S,	2q37.3	.
3	5251884	C	T	CTTCTACCTC,ATGTAGGAAT	exonic	EDEM1	nonsynonymous SNV	EDEM1:ENST00000256497.4:exon9:c.C1534T:p.H512Y,EDEM1:ENST00000445686.1:exon9:c.C949T:p.H317Y,	3p26.1	.
3	9032455	G	A	CAGGCCCCGG,GCGGGCTGTG	exonic	SRGAP3	nonsynonymous SNV	SRGAP3:ENST00000383836.3:exon21:c.C2627T:p.P876L,SRGAP3:ENST00000360413.3:exon21:c.C2555T:p.P852L,	3p25.3	.
3	9883728	C	G	TGGCTCAGCT,TGGCAGCACT	exonic	RPUSD3	nonsynonymous SNV	RPUSD3:ENST00000424438.1:exon4:c.G250C:p.E84Q,RPUSD3:ENST00000433972.1:exon4:c.G391C:p.E131Q,RPUSD3:ENST00000383820.5:exon4:c.G346C:p.E116Q,RPUSD3:ENST00000418713.1:exon4:c.G334C:p.E112Q,RPUSD3:ENST00000433535.2:exon3:c.G301C:p.E101Q,	3p25.3	.
3	10280468	C	T	AGAGCGGCTC,GAGGTCGGGA	exonic	IRAK2	stopgain	IRAK2:ENST00000256458.4:exon12:c.C1510T:p.R504X,	3p25.3	.
3	14731600	G	A	CTCTCTGCTG,AAAAGAAGCC	exonic	C3orf20	nonsynonymous SNV	C3orf20:ENST00000412910.1:exon5:c.G356A:p.G119E,C3orf20:ENST00000435614.1:exon5:c.G356A:p.G119E,C3orf20:ENST00000253697.3:exon5:c.G722A:p.G241E,	3p25.1	.
3	17053614	C	T	TGCCCAGCCC,CTATACGGGA	exonic	PLCL2	nonsynonymous SNV	PLCL2:ENST00000418129.2:exon2:c.C2398T:p.P800S,PLCL2:ENST00000432376.1:exon2:c.C2398T:p.P800S,PLCL2:ENST00000396755.2:exon2:c.C2398T:p.P800S,	3p24.3	.
3	18393590	A	C	TGGCTGAGGA,GACTGAGGAA	exonic	SATB1	nonsynonymous SNV	SATB1:ENST00000454909.2:exon10:c.T1673G:p.L558R,SATB1:ENST00000417717.2:exon10:c.T1673G:p.L558R,SATB1:ENST00000338745.6:exon10:c.T1673G:p.L558R,	3p24.3	.
3	21478668	G	C	TGTCGTCGTT,ATATTGCTGG	exonic	ZNF385D	stopgain	ZNF385D:ENST00000281523.2:exon5:c.C467G:p.S156X,	3p24.3	.
3	30885754	C	T	TATCATTTTA,CTCTAAGGGA	exonic	GADL1	nonsynonymous SNV	GADL1:ENST00000282538.5:exon8:c.G734A:p.G245D,GADL1:ENST00000454381.3:exon8:c.G734A:p.G245D,	3p24.1	.
3	30885882	C	A	CCATTACCTT,CATCTGTTTC	exonic	GADL1	nonsynonymous SNV	GADL1:ENST00000282538.5:exon7:c.G728T:p.G243V,GADL1:ENST00000454381.3:exon7:c.G728T:p.G243V,	3p24.1	.
3	32995848	C	T	GGAGAAATTT,GCAAGTACAT	exonic	CCR4	nonsynonymous SNV	CCR4:ENST00000330953.5:exon2:c.C934T:p.R312C,	3p22.3	C
3	33406139	C	T	GAAAATATCT,GAAGCGATGC	exonic	FBXL2	stopgain	FBXL2:ENST00000422741.1:exon3:c.C88T:p.R30X,	3p22.3	F
3	37388713	C	T	GGATGTCTCA,TCTTTGGAGA	exonic	GOLGA4	nonsynonymous SNV	GOLGA4:ENST00000356847.4:exon21:c.C6547T:p.L2183F,GOLGA4:ENST00000437131.1:exon19:c.C6115T:p.L2039F,GOLGA4:ENST00000361924.2:exon21:c.C6502T:p.L2168F,	3p22.2	.
3	37670702	C	T	CGTCATCAGC,CGATCGTGTT	exonic	ITGA9	nonsynonymous SNV	ITGA9:ENST00000264741.5:exon16:c.C1714T:p.P572S,ITGA9:ENST00000422441.1:exon16:c.C1714T:p.P572S,	3p22.2	.
3	38595923	G	A	ATTTTCTCAG,ACTTTGGTCA	exonic	SCN5A	nonsynonymous SNV	SCN5A:ENST00000333535.4:exon27:c.C4660T:p.P1554S,SCN5A:ENST00000413689.1:exon27:c.C4660T:p.P1554S,SCN5A:ENST00000414099.2:exon25:c.C4606T:p.P1536S,SCN5A:ENST00000451551.2:exon26:c.C4498T:p.P1500S,SCN5A:ENST00000449557.2:exon25:c.C4498T:p.P1500S,SCN5A:ENST0000	3p22.2	.
3	38648257	G	A	GCCGTGGTCG,GGTTCTCGCC	exonic	SCN5A	nonsynonymous SNV	SCN5A:ENST00000333535.4:exon9:c.C1043T:p.P348L,SCN5A:ENST00000413689.1:exon9:c.C1043T:p.P348L,SCN5A:ENST00000414099.2:exon8:c.C1043T:p.P348L,SCN5A:ENST00000451551.2:exon9:c.C1043T:p.P348L,SCN5A:ENST00000449557.2:exon8:c.C1043T:p.P348L,SCN5A:ENST00000443581.1:exon	3p22.2	.
3	38889013	C	T	AAAACCAGTT,ATACCCAGAA	exonic	SCN11A	nonsynonymous SNV	SCN11A:ENST00000456224.3:exon25:c.G4434A:p.M1478I,SCN11A:ENST00000302328.3:exon26:c.G4548A:p.M1516I,SCN11A:ENST00000450244.1:exon26:c.G4548A:p.M1516I,	3p22.2	.
3	40192597	C	T	CCGCTTCAAG,GCTTTGGCAG	exonic	MYRIP	nonsynonymous SNV	MYRIP:ENST00000458292.1:exon4:c.C391T:p.R131C,MYRIP:ENST00000302541.6:exon4:c.C391T:p.R131C,MYRIP:ENST00000425621.1:exon4:c.C391T:p.R131C,MYRIP:ENST00000444716.1:exon4:c.C391T:p.R131C,	3p22.1	.
3	42739719	G	A	GTCAGCTAAG,AGTAGTGGCG	exonic	HHATL	nonsynonymous SNV	HHATL:ENST00000455195.1:exon6:c.C608T:p.S203F,HHATL:ENST00000441594.1:exon6:c.C608T:p.S203F,HHATL:ENST00000416756.1:exon6:c.C608T:p.S203F,HHATL:ENST00000457462.1:exon5:c.C413T:p.S138F,HHATL:ENST00000310417.5:exon7:c.C608T:p.S203F,	3p22.1	.
3	44700246	G	A	GATATGTGAG,AAGCTGAAAA	exonic	ZNF35	nonsynonymous SNV	ZNF35:ENST00000415571.2:exon4:c.G391A:p.E131K,ZNF35:ENST00000396056.2:exon4:c.G391A:p.E131K,	3p21.31	.
3	46490500	C	T	GCAGCCACTT,CTCCTCACCT	exonic	LTF	nonsynonymous SNV	LTF:ENST00000231751.4:exon9:c.G1066A:p.E356K,LTF:ENST00000426532.2:exon9:c.G934A:p.E312K,LTF:ENST00000443496.1:exon12:c.G1027A:p.E343K,LTF:ENST00000417439.1:exon9:c.G1066A:p.E356K,	3p21.31	.
3	47882491	C	T	CCCCCTACTT,CTGGCGGCAG	exonic	DHX30	nonsynonymous SNV	DHX30:ENST00000457607.1:exon4:c.C575T:p.S192F,DHX30:ENST00000348968.4:exon8:c.C407T:p.S136F,DHX30:ENST00000445061.1:exon7:c.C491T:p.S164F,DHX30:ENST00000446256.2:exon8:c.C374T:p.S125F,	3p21.31	.
3	48465902	C	T	GGTGGGGTCC,TTGCCAGGTG	exonic	PLXNB1	nonsynonymous SNV	PLXNB1:ENST00000449094.1:exon1:c.G119A:p.R40K,PLXNB1:ENST00000296440.6:exon3:c.G119A:p.R40K,PLXNB1:ENST00000456774.1:exon1:c.G119A:p.R40K,PLXNB1:ENST00000358459.4:exon3:c.G119A:p.R40K,PLXNB1:ENST00000358536.4:exon3:c.G119A:p.R40K,	3p21.31	.
3	49136368	G	A	AAGGGAACCT,ATGGAAGCCT	exonic	QARS	stopgain	QARS:ENST00000306125.6:exon19:c.C1813T:p.Q605X,QARS:ENST00000453392.1:exon4:c.C373T:p.Q125X,QARS:ENST00000414533.1:exon19:c.C1780T:p.Q594X,	3p21.31	.
3	49662646	C	T	CCCCACATCA,CCTACTCCGT	exonic	BSN	nonsynonymous SNV	BSN:ENST00000296452.4:exon2:c.C463T:p.P155S,	3p21.31	.
3	49898410	C	T	GCCCAGCAGT,CACAGGGCGT	exonic	CAMKV	nonsynonymous SNV	CAMKV:ENST00000467248.1:exon7:c.G379A:p.D127N,CAMKV:ENST00000466940.1:exon7:c.G475A:p.D159N,CAMKV:ENST00000477224.1:exon7:c.G604A:p.D202N,CAMKV:ENST00000480398.2:exon5:c.G335A:p.G112E,CAMKV:ENST00000488336.1:exon7:c.G604A:p.D202N,CAMKV:ENST00000463537.1:exo	3p21.31	.
3	51737946	G	A	GCCCTTAGGG,AGTCACGGCT	exonic	TEX264	nonsynonymous SNV	TEX264:ENST00000416589.1:exon5:c.G856A:p.E286K,TEX264:ENST00000457573.1:exon6:c.G856A:p.E286K,TEX264:ENST00000415259.1:exon5:c.G856A:p.E286K,TEX264:ENST00000341333.5:exon5:c.G856A:p.E286K,TEX264:ENST00000395057.1:exon5:c.G856A:p.E286K,	3p21.2	.
3	52427492	A	C	ACGAGGGCAA,ATCAACCAGG	exonic	DNAH1	nonsynonymous SNV	DNAH1:ENST00000420323.2:exon66:c.A10617C:p.K3539N,DNAH1:ENST00000490713.1:exon9:c.A1317C:p.K439N,	3p21.1	.
3	52433570	G	C	GGTGATGTTT,AGGCACCATC	exonic	DNAH1	nonsynonymous SNV	DNAH1:ENST00000420323.2:exon77:c.G12451C:p.E4151Q,	3p21.1	.
3	52740108	C	T	TCGGAGACTT,CGCTTCCGGG	exonic	SPCS1	nonsynonymous SNV	SPCS1:ENST00000233025.7:exon1:c.C47T:p.S16F,	3p21.1	.
3	52836397	G	A	GTGTGGAGAT,GAGTACCCCG	exonic	ITIH3	nonsynonymous SNV	ITIH3:ENST00000449956.2:exon12:c.G1431A:p.M477I,ITIH3:ENST00000416872.2:exon12:c.G1431A:p.M477I,	3p21.1	.
3	52836398	G	A	TGTGGAGATG,AGTACCCCGA	exonic	ITIH3	nonsynonymous SNV	ITIH3:ENST00000449956.2:exon12:c.G1432A:p.E478K,ITIH3:ENST00000416872.2:exon12:c.G1432A:p.E478K,	3p21.1	.
3	53212515	C	T	GCGAACCAGC,CTTCTGTGCC	exonic	PRKCD	nonsynonymous SNV	PRKCD:ENST00000394729.2:exon2:c.C77T:p.P26L,PRKCD:ENST00000487897.1:exon3:c.C77T:p.P26L,PRKCD:ENST00000464818.1:exon2:c.C77T:p.P26L,PRKCD:ENST00000478843.1:exon3:c.C77T:p.P26L,PRKCD:ENST00000330452.3:exon3:c.C77T:p.P26L,	3p21.1	.
3	53265431	G	C	AATGTCCACT,AGGGTGCGTC	exonic	TKT	stopgain	TKT:ENST00000462138.1:exon7:c.C884G:p.S295X,TKT:ENST00000423516.1:exon8:c.C908G:p.S303X,TKT:ENST00000423525.2:exon7:c.C884G:p.S295X,TKT:ENST00000296289.6:exon8:c.C743G:p.S248X,TKT:ENST00000469678.1:exon7:c.C884G:p.S295X,	3p21.1	.
3	56468804	C	T	GTCATAGTGC,CTTTGGGTAG	exonic	ERC2	nonsynonymous SNV	ERC2:ENST00000460849.1:exon2:c.G232A:p.G78S,ERC2:ENST00000288221.6:exon2:c.G232A:p.G78S,	3p14.3	.
3	62118288	G	A	CAGTCCGAGG,ACAATTCTGC	exonic	PTPRG	nonsynonymous SNV	PTPRG:ENST00000295874.10:exon6:c.G628A:p.D210N,PTPRG:ENST00000474889.1:exon6:c.G628A:p.D210N,	3p14.2	.
3	62189429	G	A	CGTCCCCACA,ACCAGACGGG	exonic	PTPRG	nonsynonymous SNV	PTPRG:ENST00000295874.10:exon12:c.G1960A:p.D654N,PTPRG:ENST00000474889.1:exon12:c.G1960A:p.D654N,	3p14.2	.
3	71739281	C	T	TCTTCTCGGT,CCGAACACTG	exonic	EIF4E3	nonsynonymous SNV	EIF4E3:ENST00000448225.1:exon7:c.G190A:p.D64N,EIF4E3:ENST00000421769.2:exon7:c.G190A:p.D64N,EIF4E3:ENST00000295612.3:exon7:c.G190A:p.D64N,EIF4E3:ENST00000425534.3:exon6:c.G508A:p.D170N,EIF4E3:ENST00000497838.1:exon7:c.G190A:p.D64N,EIF4E3:ENST00000389826.3:exon7:c.G1	3p13	.
3	78666980	C	T	ACAGAGCTCT,CAGGTCCCCA	exonic	ROBO1	nonsynonymous SNV	ROBO1:ENST00000467549.1:exon25:c.G3787A:p.E1263K,ROBO1:ENST00000436010.2:exon25:c.G3970A:p.E1324K,ROBO1:ENST00000495273.1:exon25:c.G3952A:p.E1318K,ROBO1:ENST00000464233.1:exon27:c.G4087A:p.E1363K,	3p12.3	.
3	87017773	C	T	ACTATGTCTA,TGTTCCATGA	exonic	VGLL3	nonsynonymous SNV	VGLL3:ENST00000398399.2:exon3:c.G904A:p.V302I,VGLL3:ENST00000383698.3:exon3:c.G904A:p.V302I,	3p12.1	.
3	87310472	C	T	CCCACTTTTT,ATTGTACAAA	exonic	POU1F1	nonsynonymous SNV	POU1F1:ENST00000561167.1:exon4:c.G391A:p.E131K,POU1F1:ENST00000344265.3:exon5:c.G694A:p.E232K,POU1F1:ENST00000350375.2:exon5:c.G616A:p.E206K,	3p11.2	.
3	97311525	T	A	TTAAATAGCA,CCAGGCCCCG	exonic	EPHA6	nonsynonymous SNV	EPHA6:ENST00000389672.5:exon12:c.T2456A:p.I819N,EPHA6:ENST00000514100.1:exon9:c.T632A:p.I211N,EPHA6:ENST00000442602.2:exon6:c.T554A:p.I185N,EPHA6:ENST00000502694.1:exon9:c.T632A:p.I211N,EPHA6:ENST00000508345.1:exon7:c.T554A:p.I185N,	3q11.2	.
3	97868502	G	A	AGAGTAAGAT,ATATCTCTCT	exonic	OR5H14	nonsynonymous SNV	OR5H14:ENST00000437310.1:exon1:c.G273A:p.M91I,	3q11.2	.
3	99514202	G	A	GGAGAGCCAG,AATCCCAGGG	exonic	COL8A1	nonsynonymous SNV	COL8A1:ENST00000261037.3:exon5:c.G1457A:p.G486E,COL8A1:ENST00000273342.4:exon4:c.G1457A:p.G486E,	3q12.1	C
3	107497309	C	T	TGTACCTGTC,CAAGAAAAAA	exonic	BBX	nonsynonymous SNV	BBX:ENST00000406780.1:exon12:c.C2146T:p.P716S,BBX:ENST00000415149.2:exon13:c.C2146T:p.P716S,BBX:ENST00000402543.1:exon14:c.C2146T:p.P716S,BBX:ENST00000325805.8:exon13:c.C2146T:p.P716S,	3q13.12	.
3	108549543	T	A	CTTTTTCCTC,GGGGACTTCT	exonic	TRAT1	nonsynonymous SNV	TRAT1:ENST00000295756.6:exon2:c.T34A:p.W12R,	3q13.13	.
3	112710168	C	T	CGCCCCGGAC,TTCCGCGGCC	exonic	GTPBP8	nonsynonymous SNV	GTPBP8:ENST00000485330.1:exon1:c.C322T:p.L108F,GTPBP8:ENST00000295864.5:exon1:c.C322T:p.L108F,GTPBP8:ENST00000488781.1:exon1:c.C277T:p.L93F,GTPBP8:ENST00000383678.2:exon1:c.C322T:p.L108F,GTPBP8:ENST00000383677.3:exon1:c.C322T:p.L108F,	3q13.2	.
3	113049370	G	A	AGGAATTTTG,GAATGGGTAT	exonic	WDR52	nonsynonymous SNV	WDR52:ENST00000393845.2:exon26:c.C3761T:p.P1254L,	3q13.2	.
3	114070064	C	T	TGCGTGTGAT,CAGCTGGGGT	exonic	ZBTB20	stopgain	ZBTB20:ENST00000462705.1:exon11:c.G642A:p.W214X,ZBTB20:ENST00000471418.1:exon6:c.G642A:p.W214X,ZBTB20:ENST00000357258.3:exon9:c.G642A:p.W214X,ZBTB20:ENST00000481632.1:exon5:c.G642A:p.W214X,ZBTB20:ENST00000393785.2:exon5:c.G642A:p.W214X,ZBTB20:ENST00000464560.1	3q13.31	.
3	119121121	C	T	CAACAGCACG,CGTGCAGAAC	exonic	ARHGAP31	nonsynonymous SNV	ARHGAP31:ENST00000264245.4:exon10:c.C1522T:p.P508S,	3q13.33	.
3	119153625	G	C	GCGGGCATGA,ATACATCTGA	exonic	TMEM39A	nonsynonymous SNV	TMEM39A:ENST00000319172.5:exon8:c.C1217G:p.S406C,	3q13.33	.
3	124114166	C	T	GCGCCTCCCT,CCTCGGGGAG	exonic	KALRN	nonsynonymous SNV	KALRN:ENST00000460856.1:exon12:c.C2141T:p.S714F,KALRN:ENST00000439170.2:exon4:c.C569T:p.S190F,KALRN:ENST00000240874.3:exon12:c.C2141T:p.S714F,KALRN:ENST00000360013.3:exon12:c.C2141T:p.S714F,	3q21.2	.
3	128664538	G	A	TGTCTCTCTG,GAGAGATGAG	exonic	RP11-723O4.	nonsynonymous SNV	RP11-723O4.6:ENST00000508239.1:exon3:c.C272T:p.P91L,RP11-723O4.6:ENST00000511204.1:exon2:c.C272T:p.P91L,	3q21.3	.
3	129140298	G	A	CTCTGAAGGC,TGATGCTGGG	exonic	EFCAB12	nonsynonymous SNV	EFCAB12:ENST00000505956.1:exon2:c.C398T:p.T133M,EFCAB12:ENST00000326085.3:exon2:c.C398T:p.T133M,	3q21.3	.
3	129140299	T	A	TCTGAAGGCG,GATGCTGGGC	exonic	EFCAB12	nonsynonymous SNV	EFCAB12:ENST00000505956.1:exon2:c.A397T:p.T133S,EFCAB12:ENST00000326085.3:exon2:c.A397T:p.T133S,	3q21.3	.
3	129305099	A	T	GCAGGCGGCG,CCTTCACCCT	exonic	PLXND1	nonsynonymous SNV	PLXND1:ENST00000324093.4:exon4:c.T1637A:p.V546D,PLXND1:ENST00000393239.1:exon4:c.T1637A:p.V546D,PLXND1:ENST00000505237.2:exon3:c.T326A:p.V109D,	3q22.1	.
3	130113808	C	T	GATTTTGTAA,CATGACAACT	exonic	COL6A5	nonsynonymous SNV	COL6A5:ENST00000265379.6:exon8:c.C3068T:p.T1023I,COL6A5:ENST00000312481.7:exon8:c.C3068T:p.T1023I,COL6A5:ENST00000432398.2:exon8:c.C3068T:p.T1023I,	3q22.1	.
3	130134549	C	T	GAAAGGACCT,AGGTGAGTGA	exonic	COL6A5	stopgain	COL6A5:ENST00000265379.6:exon23:c.C4822T:p.Q1608X,COL6A5:ENST00000312481.7:exon23:c.C4822T:p.Q1608X,COL6A5:ENST00000432398.2:exon23:c.C4822T:p.Q1608X,	3q22.1	.
3	130308013	C	A	GATCCTGGAC,ACCAGGGAAA	exonic	COL6A6	nonsynonymous SNV	COL6A6:ENST00000453409.2:exon11:c.C4205A:p.P1402Q,COL6A6:ENST00000358511.6:exon11:c.C4205A:p.P1402Q,	3q22.1	.
3	130889665	C	T	TCAGCCTATT,CTTCCATGGA	exonic	NEK11	nonsynonymous SNV	NEK11:ENST00000383366.4:exon14:c.C1333T:p.P445S,NEK11:ENST00000507910.1:exon13:c.C1333T:p.P445S,NEK11:ENST00000510688.1:exon13:c.C1333T:p.P445S,NEK11:ENST00000510769.1:exon10:c.C1018T:p.P340S,NEK11:ENST00000508196.1:exon12:c.C1333T:p.P445S,NEK11:ENST00000511262.1	3q22.1	.
3	130889666	C	T	CAGCCTATTC,TTCCATGGAC	exonic	NEK11	nonsynonymous SNV	NEK11:ENST00000383366.4:exon14:c.C1334T:p.P445L,NEK11:ENST00000507910.1:exon13:c.C1334T:p.P445L,NEK11:ENST00000510688.1:exon13:c.C1334T:p.P445L,NEK11:ENST00000510769.1:exon10:c.C1019T:p.P340L,NEK11:ENST00000508196.1:exon12:c.C1334T:p.P445L,NEK11:ENST00000511262.1	3q22.1	.
3	136046483	G	A	TAGGCCTATG,AGGTGCCTAT	exonic	PCCB	stopgain	PCCB:ENST00000484181.1:exon12:c.G1206A:p.W402X,	3q22.3	.
3	138022426	C	T	AGCATCTTCT,TGTCCCGGCT	exonic	NME9	nonsynonymous SNV	NME9:ENST00000536478.1:exon9:c.G668A:p.R223K,NME9:ENST00000341790.5:exon8:c.G662A:p.R221K,NME9:ENST00000317876.4:exon8:c.G668A:p.R223K,NME9:ENST00000383180.2:exon11:c.G668A:p.R223K,NME9:ENST00000484930.1:exon9:c.G662A:p.R221K,NME9:ENST00000333911.3:exon10:c.G6	3q22.3	.
3	140997315	C	T	CTGCAACATC,CCAGCGTGGC	exonic	ACPL2	nonsynonymous SNV	ACPL2:ENST00000393010.2:exon5:c.C211T:p.P71S,ACPL2:ENST00000508812.1:exon2:c.C184T:p.P62S,ACPL2:ENST00000504264.1:exon2:c.C160T:p.P54S,ACPL2:ENST00000393007.1:exon2:c.C163T:p.P55S,ACPL2:ENST00000502783.1:exon4:c.C97T:p.P33S,ACPL2:ENST00000505013.1:exon3:c.C211T:p.P	3q23	.
3	148459396	C	T	TTCAACCCTC,CGATAGGCT	exonic	AGTR1	nonsynonymous SNV	AGTR1:ENST00000461609.1:exon3:c.C574T:p.P192S,AGTR1:ENST00000402260.1:exon2:c.C574T:p.P192S,AGTR1:ENST00000349243.3:exon3:c.C574T:p.P192S,AGTR1:ENST00000418473.2:exon3:c.C574T:p.P192S,AGTR1:ENST00000497524.1:exon2:c.C574T:p.P192S,AGTR1:ENST00000404754.2:exon2:c.C	3q24	.
3	150128950	C	T	ACCTCAACCA,CACTTTCTCT	exonic	TSC22D2	nonsynonymous SNV	TSC22D2:ENST00000361875.3:exon1:c.C1813T:p.P605S,TSC22D2:ENST00000361136.2:exon1:c.C1813T:p.P605S,	3q25.1	.
3	150421562	C	T	TCCTCCTCCT,CACCTCTTCC	exonic	FAM194A	nonsynonymous SNV	FAM194A:ENST00000295910.6:exon1:c.G124A:p.E42K,FAM194A:ENST00000474463.1:exon1:c.G124A:p.E42K,	3q25.1	.
3	151165566	G	A	CTAAAACGTC,ATGTGTTGAA	exonic	IGSF10	stopgain	IGSF10:ENST00000282466.3:exon4:c.C2203T:p.R735X,	3q25.1	.
3	151171505	G	A	AAGCTCCTGA,GCCATAAAAA	exonic	IGSF10	nonsynonymous SNV	IGSF10:ENST00000282466.3:exon3:c.C382T:p.L128F,	3q25.1	.
3	155222361	T	A	TTTCCAAAGT,GGTCATGAGG	exonic	PLCH1	nonsynonymous SNV	PLCH1:ENST00000447496.2:exon12:c.A1651T:p.N551Y,PLCH1:ENST00000414191.1:exon12:c.A1597T:p.N533Y,PLCH1:ENST00000334686.6:exon12:c.A1597T:p.N533Y,PLCH1:ENST00000460012.1:exon13:c.A1597T:p.N533Y,PLCH1:ENST00000494598.1:exon12:c.A1651T:p.N551Y,PLCH1:ENST00000340059.	3q25.31	.
3	161221356	C	T	AAAGCCATTT,CTCCTCCTAA	exonic	OTOL1	nonsynonymous SNV	OTOL1:ENST00000327928.4:exon4:c.C1060T:p.P354S,	3q26.1	.
3	161221371	C	T	TCCTAACATC,CCATCAAATT	exonic	OTOL1	nonsynonymous SNV	OTOL1:ENST00000327928.4:exon4:c.C1075T:p.P359S,	3q26.1	.
3	170585956	G	A	CAAAACTAGG,AAGAGAACCC	exonic	RPL22L1	nonsynonymous SNV	RPL22L1:ENST00000475836.1:exon3:c.C137T:p.S46F,RPL22L1:ENST00000494771.1:exon3:c.C134T:p.S45F,	3q26.2	.
3	184039330	C	T	ACCCACTCCT,TCGCCGAACC	exonic	EIF4G1	nonsynonymous SNV	EIF4G1:ENST00000450424.1:exon9:c.C958T:p.L320F,EIF4G1:ENST00000428387.1:exon4:c.C370T:p.L124F,EIF4G1:ENST00000441154.1:exon4:c.C466T:p.L156F,EIF4G1:ENST00000427607.1:exon4:c.C370T:p.L124F,EIF4G1:ENST00000457456.1:exon4:c.C370T:p.L124F,EIF4G1:ENST00000346169.2:exon10:c.	3q27.1	.
3	184870595	G	C	CGACGCCGGC,AGGCCGCCGC	exonic	C3orf70	nonsynonymous SNV	C3orf70:ENST00000335012.2:exon1:c.C17G:p.S6W,	3q27.2	C
3	194167705	G	A	TATTCTTTGA,GACTGATACA	exonic	ATP13A3	nonsynonymous SNV	ATP13A3:ENST00000439040.1:exon14:c.C1448T:p.P483L,ATP13A3:ENST00000256031.4:exon13:c.C1448T:p.P483L,	3q29	.
3	194167706	G	A	ATTCTTTGAG,ACTGATACAG	exonic	ATP13A3	nonsynonymous SNV	ATP13A3:ENST00000439040.1:exon14:c.C1447T:p.P483S,ATP13A3:ENST00000256031.4:exon13:c.C1447T:p.P483S,	3q29	.
3	194175074	C	T	CATCAGTGCT,CACAGTATAA	exonic	ATP13A3	stopgain	ATP13A3:ENST00000439040.1:exon9:c.G678A:p.W226X,ATP13A3:ENST00000256031.4:exon8:c.G678A:p.W226X,	3q29	.
3	195452646	G	A	TCTTCCGACG,CCCCCATCCA	exonic	MUC20	nonsynonymous SNV	MUC20:ENST00000436408.1:exon2:c.G1172A:p.G391D,MUC20:ENST00000447234.2:exon2:c.G1172A:p.G391D,MUC20:ENST00000320736.6:exon3:c.G659A:p.G220D,MUC20:ENST00000445522.2:exon1:c.G1067A:p.G356D,	3q29	.
4	437583	C	T	TTCCCACATT,TTTACATTTG	exonic	ZNF721	nonsynonymous SNV	ZNF721:ENST00000338977.5:exon2:c.G637A:p.E213K,ZNF721:ENST00000511833.2:exon3:c.G673A:p.E225K,	4p16.3	.
4	1803742	C	T	CCCTACGTTA,CGTGCTCAAG	exonic	FGFR3	nonsynonymous SNV	FGFR3:ENST00000481110.2:exon7:c.C920T:p.T307I,FGFR3:ENST00000412135.2:exon7:c.C920T:p.T307I,FGFR3:ENST00000440486.2:exon7:c.C920T:p.T307I,FGFR3:ENST00000352904.1:exon6:c.C920T:p.T307I,FGFR3:ENST00000260795.2:exon6:c.C920T:p.T307I,FGFR3:ENST00000340107.4:exon7:c.C920T	4p16.3	.
4	2824763	C	T	TGGCTATAAC,GGTAAGTGCC	exonic	SH3BP2	nonsynonymous SNV	SH3BP2:ENST00000515737.1:exon3:c.C238T:p.R80W,SH3BP2:ENST00000508385.1:exon3:c.C238T:p.R80W,SH3BP2:ENST00000511747.1:exon3:c.C238T:p.R80W,SH3BP2:ENST00000504294.1:exon3:c.C238T:p.R80W,SH3BP2:ENST00000502260.1:exon4:c.C238T:p.R80W,SH3BP2:ENST00000452765.2:exon	4p16.3	.
4	20852276	C	T	ATCTCCAGTT,ATCTTCCACG	exonic	KCNIP4	nonsynonymous SNV	KCNIP4:ENST00000382148.3:exon2:c.G103A:p.E35K,KCNIP4:ENST00000382150.4:exon2:c.G115A:p.E39K,KCNIP4:ENST00000447367.2:exon2:c.G76A:p.E26K,KCNIP4:ENST00000382152.2:exon3:c.G178A:p.E60K,	4p15.31	.
4	30725980	G	A	AGCATGGGGC,ATACAGGTCC	exonic	PCDH7	nonsynonymous SNV	PCDH7:ENST00000543491.1:exon1:c.G2936A:p.R979Q,PCDH7:ENST00000361762.2:exon1:c.G2936A:p.R979Q,	4p15.1	P
4	46060559	A	T	GCAAACTGAT,TAATCTCCAG	exonic	GABRG1	nonsynonymous SNV	GABRG1:ENST00000295452.4:exon6:c.T706A:p.Y236N,	4p12	.
4	55575606	C	T	AAGTGAACTT,ATCTAACGAG	exonic	KIT	nonsynonymous SNV	KIT:ENST00000288135.5:exon7:c.C1132T:p.H378Y,KIT:ENST00000412167.2:exon7:c.C1132T:p.H378Y,	4q12	.
4	68385068	G	A	GCATCCAAAA,AACAGAAGGT	exonic	CENPC	nonsynonymous SNV	CENPC:ENST00000273853.6:exon6:c.C484T:p.L162F,CENPC:ENST00000513216.1:exon2:c.C205T:p.L69F,CENPC:ENST00000506882.1:exon6:c.C484T:p.L162F,	4q13.2	.
4	68688123	C	T	CCTGGCTTAT,CGGCAGGCCA	exonic	TMPRSS11D	nonsynonymous SNV	TMPRSS11D:ENST00000545541.1:exon9:c.G838A:p.D280N,TMPRSS11D:ENST00000283916.6:exon10:c.G1189A:p.D397N,	4q13.2	.
4	71099851	C	T	ACGTAATTTT,CTATTCCAAT	exonic	FDCSP	nonsynonymous SNV	FDCSP:ENST00000317987.5:exon4:c.C205T:p.P69S,	4q13.3	.
4	77177353	G	A	GCACAAGAAC,GGCTGAAGTT	exonic	FAM47E	nonsynonymous SNV	FAM47E:ENST00000515604.1:exon2:c.G97A:p.G33R,FAM47E:ENST00000424749.2:exon2:c.G97A:p.G33R,	4q21.1	.
4	77253585	C	T	TTCTTCTTAC,TATGGCACAT	exonic	CCDC158	nonsynonymous SNV	CCDC158:ENST00000388914.3:exon19:c.G2870A:p.R957K,	4q21.1	C
4	77675919	C	T	TGGCCACCTC,TTCTCGAGCA	exonic	SHROOM3	nonsynonymous SNV	SHROOM3:ENST00000296043.6:exon7:c.C4283T:p.P1428L,	4q21.1	.
4	79321928	G	A	TCGATGGTGT,GGTTCCAGAA	exonic	FRAS1	stopgain	FRAS1:ENST00000325942.6:exon30:c.G4016A:p.W1339X,FRAS1:ENST00000264895.6:exon30:c.G4016A:p.W1339X,	4q21.21	.
4	79321929	G	A	CGATGGTGTG,GTTCCAGAAG	exonic	FRAS1	stopgain	FRAS1:ENST00000325942.6:exon30:c.G4017A:p.W1339X,FRAS1:ENST00000264895.6:exon30:c.G4017A:p.W1339X,	4q21.21	.
4	79460452	G	A	TCCTAGGACC,CAATCAGCCA	exonic	FRAS1	nonsynonymous SNV	FRAS1:ENST00000264895.6:exon73:c.G11303A:p.R3768H,	4q21.21	.
4	87622590	C	G	CTTACCAGTT,AAAACTAGTG	exonic	PTPN13	nonsynonymous SNV	PTPN13:ENST00000427191.2:exon7:c.C831G:p.F277L,PTPN13:ENST00000411767.2:exon7:c.C831G:p.F277L,PTPN13:ENST00000511467.1:exon6:c.C831G:p.F277L,PTPN13:ENST00000436978.1:exon7:c.C831G:p.F277L,PTPN13:ENST00000316707.6:exon7:c.C831G:p.F277L,	4q21.3	P
4	89415368	C	T	TGTGGACTTT,CCTGTTCAAT	exonic	HERC5	nonsynonymous SNV	HERC5:ENST00000264350.3:exon18:c.C2330T:p.S777F,HERC5:ENST00000508159.1:exon12:c.C1244T:p.S415F,	4q22.1	.
4	90756815	C	T	ATGAATACAT,CATGGCTAAT	exonic	SNCA	nonsynonymous SNV	SNCA:ENST00000505199.1:exon1:c.G4A:p.D2N,SNCA:ENST00000502987.1:exon1:c.G4A:p.D2N,SNCA:ENST00000508895.1:exon2:c.G4A:p.D2N,SNCA:ENST00000394986.1:exon2:c.G4A:p.D2N,SNCA:ENST00000394991.3:exon2:c.G4A:p.D2N,SNCA:ENST00000420646.2:exon2:c.G4A:p.D2N,SNCA:ENST0000	4q22.1	S
4	96762263	G	A	CTCCAAGATA,AATGGTAAAC	exonic	PDHA2	nonsynonymous SNV	PDHA2:ENST00000295266.4:exon1:c.G962A:p.R321K,	4q22.3	P
4	118006496	G	C	TCTGCAGGAT,AATTCCTGGC	exonic	TRAM1L1	nonsynonymous SNV	TRAM1L1:ENST00000310754.4:exon1:c.C54G:p.F18L,	4q26	.
4	121706237	C	T	GGTCTCTCCT,AGAGTGGGTC	exonic	PRDM5	nonsynonymous SNV	PRDM5:ENST00000264808.3:exon11:c.G1198A:p.E400K,PRDM5:ENST00000428209.2:exon10:c.G1105A:p.E369K,PRDM5:ENST00000515109.1:exon10:c.G1105A:p.E369K,	4q27	.
4	123246856	G	A	GAAATTAGAA,GAAGAAGATC	exonic	KIAA1109	nonsynonymous SNV	KIAA1109:ENST00000455637.1:exon64:c.G11012A:p.R3671K,	4q27	.
4	123797524	G	A	TCTATCAAAG,AGTGTGTGCT	exonic	FGF2	nonsynonymous SNV	FGF2:ENST00000608478.1:exon2:c.G227A:p.G76E,FGF2:ENST00000264498.3:exon2:c.G626A:p.G209E,	4q27	.
4	126336140	C	T	AGCTTTGGAT,GGGAAAGTCA	exonic	FAT4	nonsynonymous SNV	FAT4:ENST00000394329.3:exon5:c.C6022T:p.R2008W,FAT4:ENST00000335110.5:exon5:c.C916T:p.R306W,	4q28.1	F
4	126336906	C	T	GAGCTATCTC,ATATTCTGTA	exonic	FAT4	nonsynonymous SNV	FAT4:ENST00000394329.3:exon5:c.C6788T:p.P2263L,FAT4:ENST00000335110.5:exon5:c.C1682T:p.P561L,	4q28.1	.
4	134072409	G	A	TGAGGGCGCG,CGCCCGGCAC	exonic	PCDH10	nonsynonymous SNV	PCDH10:ENST00000264360.5:exon1:c.G1114A:p.A372T,	4q28.3	.
4	145636478	C	T	CAGCAAAGTC,TGTGACAAAG	exonic	HHIP	nonsynonymous SNV	HHIP:ENST00000296575.3:exon10:c.C1574T:p.P525L,	4q31.21	H
4	149075780	C	T	AGCAGATTTT,GGCTGTATCT	exonic	NR3C2	nonsynonymous SNV	NR3C2:ENST00000344721.4:exon5:c.G2287A:p.E763K,NR3C2:ENST00000355292.3:exon5:c.G2299A:p.E767K,NR3C2:ENST00000511528.1:exon4:c.G2299A:p.E767K,NR3C2:ENST00000358102.3:exon5:c.G2287A:p.E763K,	4q31.23	.
4	151023750	C	A	GCTGCTGCCT,CTCTGTGAAA	exonic	DCLK2	nonsynonymous SNV	DCLK2:ENST00000506325.1:exon2:c.C542A:p.S181Y,DCLK2:ENST00000302176.8:exon2:c.C542A:p.S181Y,DCLK2:ENST00000296550.7:exon2:c.C542A:p.S181Y,DCLK2:ENST00000411937.2:exon2:c.C542A:p.S181Y,	4q31.23	.
4	154217117	G	A	GCTGTGAAAA,ACCCGCAAGC	exonic	TRIM2	nonsynonymous SNV	TRIM2:ENST00000437508.2:exon6:c.G1358A:p.R453K,TRIM2:ENST00000338700.5:exon6:c.G1439A:p.R480K,	4q31.3	.
4	155157337	C	T	AGGAAGAGAT,ATGATCATAA	exonic	DCHS2	nonsynonymous SNV	DCHS2:ENST00000357232.4:exon25:c.G7102A:p.D2368N,	4q31.3	.
4	159789595	G	A	TGAGTGCCCA,AGGGCACTGA	exonic	FNIP2	nonsynonymous SNV	FNIP2:ENST00000512986.1:exon13:c.G1876A:p.E626K,FNIP2:ENST00000264433.6:exon13:c.G1807A:p.E603K,FNIP2:ENST00000379346.3:exon13:c.G1876A:p.E626K,	4q32.1	.
4	160189326	C	T	ACTAGCAATC,CAGCTAACCA	exonic	RAPGEF2	nonsynonymous SNV	RAPGEF2:ENST00000264431.4:exon1:c.C19T:p.P7S,	4q32.1	.
4	162307164	G	C	TTGTGTGCTT,AACTACCGTA	exonic	FSTL5	stopgain	FSTL5:ENST00000536695.1:exon15:c.C2276G:p.S759X,FSTL5:ENST00000379164.4:exon16:c.C2276G:p.S759X,FSTL5:ENST00000306100.5:exon16:c.C2279G:p.S760X,FSTL5:ENST00000427802.2:exon15:c.C2249G:p.S750X,	4q32.2	.
4	165961438	C	T	CCCCCAGCTC,GTAATTTGAC	exonic	TRIM60	nonsynonymous SNV	TRIM60:ENST00000508504.1:exon3:c.C214T:p.R72C,TRIM60:ENST00000341062.5:exon2:c.C214T:p.R72C,TRIM60:ENST00000512596.1:exon3:c.C214T:p.R72C,TRIM60:ENST00000507119.1:exon2:c.C214T:p.R72C,	4q32.3	T
4	175897940	G	A	TTGTGCAAAA,ATCCCTGCTG	exonic	ADAM29	nonsynonymous SNV	ADAM29:ENST00000359240.3:exon5:c.G1264A:p.D422N,ADAM29:ENST00000445694.1:exon4:c.G1264A:p.D422N,ADAM29:ENST00000404450.4:exon4:c.G1264A:p.D422N,ADAM29:ENST00000514159.1:exon3:c.G1264A:p.D422N,	4q34.1	.
4	183650321	C	T	TGGAGAAAGT,CTTTCAATAA	exonic	TENM3	nonsynonymous SNV	TENM3:ENST00000511685.1:exon14:c.C2572T:p.P858S,TENM3:ENST00000406950.2:exon13:c.C2572T:p.P858S,	4q35.1	.
4	183650322	C	T	GGAGAAAGTC,TTTCAATAAG	exonic	TENM3	nonsynonymous SNV	TENM3:ENST00000511685.1:exon14:c.C2573T:p.P858L,TENM3:ENST00000406950.2:exon13:c.C2573T:p.P858L,	4q35.1	.
4	186339831	G	A	TATATACACT,AACTGTGGCA	exonic	UFSP2	nonsynonymous SNV	UFSP2:ENST00000264689.6:exon3:c.C176T:p.S59L,UFSP2:ENST00000505357.1:exon3:c.C158T:p.S53L,UFSP2:ENST00000514247.1:exon3:c.C176T:p.S59L,UFSP2:ENST00000510755.1:exon3:c.C176T:p.S59L,UFSP2:ENST00000502428.1:exon3:c.C176T:p.S59L,	4q35.1	.
4	187208952	G	A	GCCGGCTACA,GGAAGGAGGG	exonic	F11	nonsynonymous SNV	F11:ENST00000264692.4:exon14:c.G1535A:p.R512K,F11:ENST00000403665.2:exon14:c.G1691A:p.R564K,	4q35.2	.
5	434914	C	T	CACGCTGGTC,CGCCCCAAGC	exonic	AHRR	nonsynonymous SNV	AHRR:ENST00000316418.5:exon12:c.C2125T:p.P709S,AHRR:ENST00000505113.1:exon11:c.C2071T:p.P691S,AHRR:ENST00000512529.1:exon9:c.C1609T:p.P537S,AHRR:ENST00000506456.1:exon7:c.C1639T:p.P547S,	5p15.33	.
5	3600771	C	T	CAGTTAAAGT,GCCCTTCCAG	exonic	IRX1	nonsynonymous SNV	IRX1:ENST00000302006.3:exon3:c.C1361T:p.S454L,	5p15.33	.
5	13830729	C	T	TCCAAGTCCT,GGAAATCCAT	exonic	DNAH5	nonsynonymous SNV	DNAH5:ENST00000265104.4:exon36:c.G6038A:p.R2013Q,	5p15.2	.
5	15937090	C	T	GAGTGCCTGG,CCTGAACTGC	exonic	FBXL7	nonsynonymous SNV	FBXL7:ENST00000329673.7:exon3:c.C1235T:p.A412V,FBXL7:ENST00000510662.1:exon4:c.C1130T:p.A377V,FBXL7:ENST00000504595.1:exon4:c.C1271T:p.A424V,	5p15.1	.
5	34823827	C	A	AGTGATGAAG,TGAGGACATG	exonic	RAI14	nonsynonymous SNV	RAI14:ENST00000512629.1:exon14:c.C1793A:p.A598D,RAI14:ENST00000428746.2:exon15:c.C1880A:p.A627D,RAI14:ENST00000265109.3:exon15:c.C1880A:p.A627D,RAI14:ENST00000506376.1:exon14:c.C1856A:p.A619D,RAI14:ENST00000515799.1:exon17:c.C1889A:p.A630D,RAI14:ENST00000397449.1:ex	5p13.2	.
5	37342692	G	C	CAGTGATTCA,AATTTTCAAT	exonic	NUP155	nonsynonymous SNV	NUP155:ENST00000381843.2:exon10:c.C875G:p.S292C,NUP155:ENST00000231498.3:exon10:c.C1052G:p.S351C,NUP155:ENST00000513532.1:exon10:c.C1052G:p.S351C,	5p13.2	.
5	40998720	T	G	TCACGTGTGG,CAGTTGTTCT	exonic	MROH2B	nonsynonymous SNV	MROH2B:ENST00000399564.4:exon41:c.A4645C:p.T1549P,MROH2B:ENST00000506092.2:exon31:c.A3310C:p.T1104P,	5p13.1	.
5	52204843	G	A	GAGGAGCAAG,AAAAGTGTAT	exonic	ITGA1	nonsynonymous SNV	ITGA1:ENST00000282588.6:exon13:c.G1571A:p.G524E,	5q11.2	.
5	53813939	G	A	CCTCTTCCCG,CCTCCTATGT	exonic	SNX18	nonsynonymous SNV	SNX18:ENST00000381410.4:exon1:c.G157A:p.A53T,SNX18:ENST00000343017.6:exon1:c.G157A:p.A53T,SNX18:ENST00000326277.3:exon1:c.G157A:p.A53T,	5q11.2	.
5	53813940	C	A	CTCTTCCCGG,CTCCTATGTG	exonic	SNX18	nonsynonymous SNV	SNX18:ENST00000381410.4:exon1:c.C158A:p.A53D,SNX18:ENST00000343017.6:exon1:c.C158A:p.A53D,SNX18:ENST00000326277.3:exon1:c.C158A:p.A53D,	5q11.2	.
5	54329680	G	A	CACAAAGCCT,GAATCTACAC	exonic	GZMK	nonsynonymous SNV	GZMK:ENST00000231009.2:exon5:c.G721A:p.G241R,	5q11.2	.
5	61747729	G	A	GCATCTAAAC,ACTTGCTGCT	exonic	IPO11	nonsynonymous SNV	IPO11:ENST00000325324.6:exon5:c.G485A:p.R162Q,IPO11:ENST00000424533.1:exon5:c.G485A:p.R162Q,IPO11:ENST00000506200.1:exon5:c.G485A:p.R162Q,IPO11:ENST00000409296.3:exon5:c.G605A:p.R202Q,	5q12.1	.
5	64483947	C	T	TCCAGCGTCT,CTCCTCAGAA	exonic	ADAMTS6	nonsynonymous SNV	ADAMTS6:ENST00000381055.3:exon22:c.G2806A:p.E936K,	5q12.3	.
5	64483948	C	A	CCAGCGTCTC,TCCTCAGAAG	exonic	ADAMTS6	nonsynonymous SNV	ADAMTS6:ENST00000381055.3:exon22:c.G2805T:p.E935D,	5q12.3	.
5	64769472	C	T	GCTCAAAATC,AGGTCAACGT	exonic	ADAMTS6	stopgain	ADAMTS6:ENST00000536360.1:exon2:c.G29A:p.W10X,ADAMTS6:ENST00000381052.4:exon2:c.G29A:p.W10X,ADAMTS6:ENST00000464680.2:exon1:c.G29A:p.W10X,ADAMTS6:ENST00000381055.3:exon2:c.G29A:p.W10X,	5q12.3	.
5	64824825	C	T	TGTTGCTGTT,ATCCAACCAC	exonic	CENPK	nonsynonymous SNV	CENPK:ENST00000502997.1:exon6:c.G391A:p.E131K,CENPK:ENST00000242872.3:exon6:c.G391A:p.E131K,CENPK:ENST00000508421.1:exon8:c.G301A:p.E101K,CENPK:ENST00000515497.1:exon8:c.G397A:p.E133K,CENPK:ENST00000396679.1:exon8:c.G391A:p.E131K,CENPK:ENST00000514814.1:exon8	5q12.3	.
5	66400333	C	T	TTGGATAAAT,CCACCAGGGC	exonic	MAST4	nonsynonymous SNV	MAST4:ENST00000403625.2:exon10:c.C1286T:p.S429F,MAST4:ENST00000405643.1:exon9:c.C749T:p.S250F,MAST4:ENST00000261569.7:exon7:c.C704T:p.S235F,MAST4:ENST00000490016.2:exon9:c.C719T:p.S240F,MAST4:ENST00000403666.1:exon9:c.C719T:p.S240F,MAST4:ENST00000404260.3:exon10	5q12.3	.
5	67589271	T	C	AATCCCAAAT,GGATGTGAAA	exonic	PIK3R1	nonsynonymous SNV	PIK3R1:ENST00000521409.1:exon3:c.T170C:p.L57S,PIK3R1:ENST00000320694.8:exon4:c.T359C:p.L120S,PIK3R1:ENST00000336483.5:exon4:c.T449C:p.L150S,PIK3R1:ENST00000521381.1:exon10:c.T1259C:p.L420S,PIK3R1:ENST00000274335.5:exon9:c.T1259C:p.L420S,PIK3R1:ENST00000521657.1:exon1	5q13.1	.
5	68505579	C	T	GGATCCTGCC,TTAAGGAAAT	exonic	CENPH	nonsynonymous SNV	CENPH:ENST00000283006.2:exon9:c.C697T:p.L233F,CENPH:ENST00000515001.1:exon8:c.C640T:p.L214F,	5q13.2	.
5	71491823	G	A	CATCCAGAAG,AGAGAGAAGT	exonic	MAP1B	nonsynonymous SNV	MAP1B:ENST00000296755.7:exon5:c.G2641A:p.E881K,	5q13.2	.
5	71654133	C	T	ACCACTGATT,TTTGGATGCT	exonic	PTCD2	nonsynonymous SNV	PTCD2:ENST00000380639.5:exon10:c.C1046T:p.S349F,PTCD2:ENST00000536805.1:exon8:c.C530T:p.S177F,PTCD2:ENST00000503868.1:exon7:c.C719T:p.S240F,PTCD2:ENST00000510676.2:exon9:c.C533T:p.S178F,PTCD2:ENST00000308077.5:exon10:c.C1046T:p.S349F,	5q13.2	.
5	71756986	G	A	GAACAGCAAA,GGAGGTTGGG	exonic	ZNF366	nonsynonymous SNV	ZNF366:ENST00000318442.5:exon2:c.C338T:p.P113L,	5q13.2	.
5	76355541	G	A	ACTTTCCAAA,AGATGATGCT	exonic	AGGF1	nonsynonymous SNV	AGGF1:ENST00000312916.7:exon12:c.G1817A:p.R606K,	5q13.3	.
5	78326738	A	T	AAATGGTGAT,GGTCAGTTAC	exonic	DMGDH	nonsynonymous SNV	DMGDH:ENST00000255189.3:exon10:c.T1601A:p.L534Q,DMGDH:ENST00000523732.1:exon7:c.T1118A:p.L373Q,DMGDH:ENST00000540686.1:exon5:c.T461A:p.L154Q,DMGDH:ENST00000380311.4:exon7:c.T998A:p.L333Q,	5q14.1	.
5	79026431	C	A	AGAAGGTGAG,CAGTTCCCCC	exonic	CMYA5	nonsynonymous SNV	CMYA5:ENST00000446378.2:exon2:c.C1843A:p.P615T,	5q14.1	.
5	82815651	C	T	AAGCACATTC,TTCCAAGGAA	exonic	VCAN	nonsynonymous SNV	VCAN:ENST00000512590.2:exon7:c.C1382T:p.P461L,VCAN:ENST00000342785.4:exon7:c.C1526T:p.P509L,VCAN:ENST00000265077.3:exon7:c.C1526T:p.P509L,	5q14.3	.
5	82834539	T	C	TCCAGGGAAA,AGTGATTTCA	exonic	VCAN	nonsynonymous SNV	VCAN:ENST00000513960.1:exon7:c.T2756C:p.I919T,VCAN:ENST00000343200.5:exon7:c.T2756C:p.I919T,VCAN:ENST00000265077.3:exon8:c.T5717C:p.I1906T,	5q14.3	V
5	89992960	G	A	CATAGGTCAT,AAGGTGGGTT	exonic	GPR98	nonsynonymous SNV	GPR98:ENST00000405460.2:exon34:c.G8152A:p.E2718K,	5q14.3	.
5	90024714	G	A	TTCAGCCAAT,ATATTTACCT	exonic	GPR98	nonsynonymous SNV	GPR98:ENST00000405460.2:exon49:c.G10390A:p.D3464N,	5q14.3	.
5	118485181	C	T	GTTTCTTTAT,GTGGGTCCGG	exonic	DMXL1	nonsynonymous SNV	DMXL1:ENST00000311085.8:exon18:c.C3659T:p.S1220L,DMXL1:ENST00000539542.1:exon18:c.C3659T:p.S1220L,	5q23.1	DI
5	118965512	G	A	AGAGTCCCAG,AAACCGCTGA	exonic	FAM170A	nonsynonymous SNV	FAM170A:ENST00000509264.1:exon1:c.G49A:p.E17K,FAM170A:ENST00000515256.1:exon1:c.G49A:p.E17K,FAM170A:ENST00000335286.6:exon1:c.G49A:p.E17K,FAM170A:ENST00000504819.1:exon1:c.G49A:p.E17K,FAM170A:ENST00000379555.3:exon1:c.G49A:p.E17K,	5q23.1	.
5	118969732	C	T	AGAGACTCCT,CCCGCTCACA	exonic	FAM170A	nonsynonymous SNV	FAM170A:ENST00000509264.1:exon3:c.C289T:p.P97S,FAM170A:ENST00000515256.1:exon3:c.C289T:p.P97S,FAM170A:ENST00000335286.6:exon3:c.C289T:p.P97S,FAM170A:ENST00000504819.1:exon2:c.C148T:p.P50S,FAM170A:ENST00000379555.3:exon2:c.C148T:p.P50S,	5q23.1	.
5	127597456	C	T	ATGAATACTT,TCTTCTGCCT	exonic	FBN2	nonsynonymous SNV	FBN2:ENST00000508053.1:exon70:c.G8336A:p.R2779K,FBN2:ENST00000262464.4:exon64:c.G8336A:p.R2779K,	5q23.3	.
5	131007754	G	A	TTAGTTTCTT,ATGCTGATCA	exonic	FNIP1	stopgain	FNIP1:ENST00000510461.1:exon14:c.C2383T:p.Q795X,FNIP1:ENST00000307968.7:exon13:c.C2299T:p.Q767X,FNIP1:ENST00000307954.8:exon13:c.C2248T:p.Q750X,	5q23.3	.
5	131304891	T	A	TCGGCTTGCT,ACGCTTTGCT	exonic	ACSL6	stopgain	ACSL6:ENST00000379244.1:exon13:c.A1189T:p.K397X,ACSL6:ENST00000379272.2:exon14:c.A1234T:p.K412X,ACSL6:ENST00000296869.4:exon13:c.A1264T:p.K422X,ACSL6:ENST00000413683.1:exon12:c.A1189T:p.K397X,ACSL6:ENST00000357096.1:exon11:c.A964T:p.K322X,ACSL6:ENST00000544770.1:e	5q31.1	.
5	133861669	C	T	TGTTGATGTC,CAGCCATGCT	exonic	IRF1	stopgain	IRF1:ENST00000377654.1:exon3:c.G138A:p.W46X,IRF1:ENST00000458069.1:exon3:c.G138A:p.W46X,IRF1:ENST00000405885.2:exon3:c.G138A:p.W46X,IRF1:ENST00000476613.1:exon2:c.G138A:p.W46X,IRF1:ENST00000245414.4:exon3:c.G138A:p.W46X,	5q31.1	.
5	133901806	T	C	TTCCCCTCAG,GTTCCATGCC	exonic	PHF15	nonsynonymous SNV	PHF15:ENST00000361895.2:exon9:c.T970C:p.C324R,PHF15:ENST00000402835.1:exon9:c.T970C:p.C324R,PHF15:ENST00000395003.1:exon9:c.T970C:p.C324R,PHF15:ENST00000282605.4:exon9:c.T970C:p.C324R,	5q31.1	.
5	136974794	T	A	CAGTGAGCCA,TAAACCCTCC	exonic	KLHL3	nonsynonymous SNV	KLHL3:ENST00000506491.1:exon8:c.A821T:p.N274I,KLHL3:ENST00000309755.4:exon10:c.A1067T:p.N356I,KLHL3:ENST00000508657.1:exon10:c.A977T:p.N326I,	5q31.2	.
5	138720803	C	T	GGCGGGCCCG,GAGACGAGGG	exonic	PROB1	nonsynonymous SNV	PROB1:ENST00000434752.2:exon1:c.G2768T:p.P923L,	5q31.2	.
5	139189303	C	T	CTGGAAGATT,AGCGGAGTCC	exonic	PSD2	nonsynonymous SNV	PSD2:ENST00000274710.3:exon2:c.C278T:p.S93L,	5q31.2	.
5	139229079	C	T	GGGCCCGGGT,CGGGTCCCGG	exonic	NRG2	nonsynonymous SNV	NRG2:ENST00000289422.7:exon11:c.G2000A:p.G667E,NRG2:ENST00000545385.1:exon11:c.G1982A:p.G661E,NRG2:ENST00000358522.3:exon11:c.G1982A:p.G661E,NRG2:ENST00000340391.3:exon10:c.G1367A:p.G456E,NRG2:ENST00000361474.1:exon10:c.G1976A:p.G659E,NRG2:ENST00000289409.4:e	5q31.2	.
5	139838812	C	A	GCTTGCTGTG,AGGATTTTCT	exonic	ANKHD1,ANK	nonsynonymous SNV	ANKHD1:ENST00000394722.3:exon9:c.G1511A:p.G504E,ANKHD1:ENST00000360839.2:exon9:c.G1544A:p.G515E,ANKHD1:ENST00000297183.6:exon9:c.G1544A:p.G515E,ANKHD1:ENST00000421134.1:exon9:c.G1544A:p.G515E,ANKHD1-EIF4EBP3:ENST00000532219.1:exon9:c.G1544A:p.G515E,ANKHD1:E	5q31.3	.
5	139931637	G	A	CACAGCCTCA,ACTCGACTGG	exonic	SRA1	nonsynonymous SNV	SRA1:ENST00000336283.6:exon3:c.C320T:p.S107F,	5q31.3	.
5	139946900	G	A	GTGCCCTTCC,GCCCTCTCCA	exonic	SLC35A4	nonsynonymous SNV	SLC35A4:ENST00000514199.1:exon4:c.G146A:p.R49Q,SLC35A4:ENST00000323146.3:exon3:c.G146A:p.R49Q,	5q31.3	.
5	140181908	G	A	CAGCGTGTCC,ACCGCGACTC	exonic	PCDHA3	nonsynonymous SNV	PCDHA3:ENST00000532566.2:exon1:c.G1126A:p.D376N,PCDHA3:ENST00000522353.2:exon1:c.G1126A:p.D376N,	5q31.3	.
5	140228159	G	A	GTGGGTGGTG,GGAGCGGCCA	exonic	PCDHA9	nonsynonymous SNV	PCDHA9:ENST00000378122.3:exon1:c.G79A:p.G27R,PCDHA9:ENST00000532602.1:exon1:c.G79A:p.G27R,	5q31.3	.
5	140501812	C	T	GCAGCTGGAT,GTCAGCTGG	exonic	PCDHB4	nonsynonymous SNV	PCDHB4:ENST00000194152.1:exon1:c.C232T:p.R78C,	5q31.3	.
5	140740864	G	A	CCAAGTGTTG,GAAATGCCAA	exonic	PCDHGB2	nonsynonymous SNV	PCDHGB2:ENST00000522605.1:exon1:c.G1162A:p.G388R,	5q31.3	.

5	140744792	G	A	TTCCAACCTG,GGGAAATCTC	exonic	PCDHGA5	nonsynonymous SNV	PCDHGA5:ENST00000518069.1:exon1:c.G895A:p.G299R,	5q31.3	P
5	140752003	C	T	CGCCCCACTC,CTCTGACCCT	exonic	PCDHGB3	nonsynonymous SNV	PCDHGB3:ENST00000576222.1:exon1:c.C2042T:p.P681L,	5q31.3	.
5	140803166	C	T	AAAAGCGAGC,ACTCTTGATA	exonic	PCDHGA11	nonsynonymous SNV	PCDHGA11:ENST00000398587.2:exon1:c.C2372T:p.P791L,	5q31.3	.
5	140957799	C	T	TATTCCATCT,CATGCGAATG	exonic	DIAPH1	nonsynonymous SNV	DIAPH1:ENST00000398566.3:exon10:c.G1129A:p.E377K,DIAPH1:ENST00000389057.5:exon10:c.G1129A:p.E377K,DIAPH1:ENST00000253811.6:exon11:c.G1156A:p.E386K,DIAPH1:ENST00000520569.1:exon9:c.G994A:p.E332K,DIAPH1:ENST00000398562.2:exon10:c.G1129A:p.E377K,DIAPH1:ENST000005181	5q31.3	.
5	140957800	C	T	ATTCCATCTC,ATGCGAATGT	exonic	DIAPH1	nonsynonymous SNV	DIAPH1:ENST00000398566.3:exon10:c.G1128A:p.M376I,DIAPH1:ENST00000389057.5:exon10:c.G1128A:p.M376I,DIAPH1:ENST00000253811.6:exon11:c.G1155A:p.M385I,DIAPH1:ENST00000520569.1:exon9:c.G993A:p.M331I,DIAPH1:ENST00000398562.2:exon10:c.G1128A:p.M376I,DIAPH1:ENST0000051804	5q31.3	.
5	141027561	G	A	AGTTCCTCCT,GTAGTAATGG	exonic	FCHSD1	stopgain	FCHSD1:ENST00000522783.1:exon8:c.C673T:p.Q225X,FCHSD1:ENST00000435817.2:exon8:c.C679T:p.Q227X,FCHSD1:ENST00000519800.1:exon8:c.C673T:p.Q225X,FCHSD1:ENST00000522126.1:exon7:c.C451T:p.Q151X,	5q31.3	.
5	143586432	G	A	TCCCTCCTGT,GAAAATGTTT	exonic	KCTD16	stopgain	KCTD16:ENST00000512467.1:exon3:c.G155A:p.W52X,KCTD16:ENST00000507359.3:exon2:c.G155A:p.W52X,	5q31.3	.
5	146798148	C	T	CCATTGGCTT,AATGGTCTTC	exonic	DPYSL3	nonsynonymous SNV	DPYSL3:ENST00000343218.5:exon3:c.G517A:p.E173K,DPYSL3:ENST00000512722.1:exon4:c.G175A:p.E59K,DPYSL3:ENST00000398514.3:exon3:c.G175A:p.E59K,	5q32	.
5	147023743	G	A	AGGGGTGGAT,GGATGTTATT	exonic	JAKMIP2	nonsynonymous SNV	JAKMIP2:ENST00000333010.6:exon6:c.C976T:p.H326Y,JAKMIP2:ENST00000507386.1:exon7:c.C1102T:p.H368Y,JAKMIP2:ENST00000265272.5:exon7:c.C1102T:p.H368Y,	5q32	.
5	147491386	G	A	CCCTGTACCA,AGAGAATGAT	exonic	SPINK5	nonsynonymous SNV	SPINK5:ENST00000508733.1:exon19:c.G1691A:p.R564K,SPINK5:ENST00000256084.7:exon19:c.G1748A:p.R583K,SPINK5:ENST00000359874.3:exon19:c.G1748A:p.R583K,SPINK5:ENST00000398454.1:exon19:c.G1748A:p.R583K,	5q32	.
5	149242766	C	T	GCCTTCCACT,CTTGCGATTG	exonic	PDE6A	nonsynonymous SNV	PDE6A:ENST00000255266.5:exon21:c.G2422A:p.E808K,	5q32	.
5	149374623	G	A	TGTGTCCTGA,AGATAATGAG	exonic	TIGD6	nonsynonymous SNV	TIGD6:ENST00000515406.2:exon2:c.C1289T:p.S430F,TIGD6:ENST00000296736.3:exon2:c.C1289T:p.S430F,	5q32	.
5	149514559	G	A	GCATCATTAG,GAGGAAGCCC	exonic	PDGFRB	nonsynonymous SNV	PDGFRB:ENST00000517488.1:exon3:c.C193T:p.P65S,PDGFRB:ENST00000261799.4:exon4:c.C385T:p.P129S,PDGFRB:ENST00000517957.1:exon4:c.C385T:p.P129S,PDGFRB:ENST00000520579.1:exon4:c.C385T:p.P129S,	5q32	.
5	149546698	C	T	TGCCGCCAGC,CAGCTTCGCT	exonic	CDX1	nonsynonymous SNV	CDX1:ENST00000231656.8:exon1:c.C259T:p.P87S,	5q32	.
5	149755774	C	T	TGCGACTTCT,CAGCAGGCTC	exonic	TCOF1	nonsynonymous SNV	TCOF1:ENST00000394269.3:exon13:c.C2023T:p.P675S,TCOF1:ENST00000377797.3:exon13:c.C2023T:p.P675S,TCOF1:ENST00000427724.2:exon13:c.C2023T:p.P675S,TCOF1:ENST00000445265.2:exon12:c.C1792T:p.P598S,TCOF1:ENST00000323668.7:exon12:c.C1792T:p.P598S,TCOF1:ENST00000439160	5q32	.
5	149755775	C	T	GCGACTTCTC,AGCAGGCTCA	exonic	TCOF1	nonsynonymous SNV	TCOF1:ENST00000394269.3:exon13:c.C2024T:p.P675L,TCOF1:ENST00000377797.3:exon13:c.C2024T:p.P675L,TCOF1:ENST00000427724.2:exon13:c.C2024T:p.P675L,TCOF1:ENST00000445265.2:exon12:c.C1793T:p.P598L,TCOF1:ENST00000323668.7:exon12:c.C1793T:p.P598L,TCOF1:ENST00000439160.	5q32	.
5	149772977	A	G	AACCCCAGCC,ATTCCCAGGC	exonic	TCOF1	nonsynonymous SNV	TCOF1:ENST00000377797.3:exon23:c.A3646G:p.N1216D,TCOF1:ENST00000427724.2:exon22:c.A3529G:p.N1177D,TCOF1:ENST00000445265.2:exon22:c.A3415G:p.N1139D,TCOF1:ENST00000323668.7:exon22:c.A3412G:p.N1138D,TCOF1:ENST00000439160.2:exon22:c.A3532G:p.N1178D,TCOF1:ENST0000	5q32	.
5	150696607	G	A	GCCCACCAGG,AGATGACCAG	exonic	SLC36A2	nonsynonymous SNV	SLC36A2:ENST00000450886.1:exon4:c.C395T:p.S132F,SLC36A2:ENST00000335244.4:exon10:c.C1223T:p.S408F,	5q33.1	.
5	150921899	G	A	AGCATCCAGG,TCTTTAGAGT	exonic	FAT2	nonsynonymous SNV	FAT2:ENST00000261800.5:exon9:c.C8789T:p.T2930I,	5q33.1	.
5	153056575	G	A	GCTCACCTAC,ATGGGGTGAA	exonic	GRIA1	nonsynonymous SNV	GRIA1:ENST00000518142.1:exon6:c.G643A:p.D215N,GRIA1:ENST00000448073.4:exon7:c.G913A:p.D305N,GRIA1:ENST00000285900.5:exon7:c.G883A:p.D295N,GRIA1:ENST00000521843.2:exon7:c.G676A:p.D226N,GRIA1:ENST00000340592.5:exon7:c.G883A:p.D295N,GRIA1:ENST00000518783.1:exon7:c.G5	5q33.2	.
5	157214816	C	T	TGCTCTGGTT,ATCATCGGAG	exonic	CLINT1	nonsynonymous SNV	CLINT1:ENST00000530742.1:exon12:c.G1716A:p.M572I,CLINT1:ENST00000411809.2:exon12:c.G1716A:p.M572I,CLINT1:ENST00000523908.1:exon12:c.G1770A:p.M590I,CLINT1:ENST00000523094.1:exon12:c.G1716A:p.M572I,CLINT1:ENST00000296951.5:exon12:c.G1716A:p.M572I,	5q33.3	.
5	159707789	C	T	AGGGCTGCCT,CTGAGAGTCT	exonic	CCNJL	nonsynonymous SNV	CCNJL:ENST00000520748.1:exon3:c.G128A:p.G43E,	5q33.3	.
5	168310319	G	A	GGGATCCCCT,GATCTGGTTT	exonic	SLIT3	stopgain	SLIT3:ENST00000519560.1:exon5:c.C436T:p.Q146X,SLIT3:ENST00000332966.8:exon5:c.C436T:p.Q146X,SLIT3:ENST00000404867.3:exon5:c.C436T:p.Q146X,	5q34	.
5	169144401	G	A	TACATAATAG,ACTCATTGCA	exonic	DOCK2	nonsynonymous SNV	DOCK2:ENST00000520908.1:exon7:c.G521A:p.G174E,DOCK2:ENST00000524185.1:exon21:c.G2045A:p.G682E,DOCK2:ENST00000256935.8:exon21:c.G2045A:p.G682E,	5q35.1	.
5	169533370	C	A	CATGGCCATC,ACGGGGCACC	exonic	FOXI1	nonsynonymous SNV	FOXI1:ENST00000306268.6:exon1:c.C409A:p.H137N,FOXI1:ENST00000449804.2:exon1:c.C409A:p.H137N,	5q35.1	.
5	172517466	G	A	GATACATACT,TGAAGACCTA	exonic	CREBRF	nonsynonymous SNV	CREBRF:ENST00000522692.1:exon4:c.G284A:p.C95Y,CREBRF:ENST00000540014.1:exon4:c.G284A:p.C95Y,CREBRF:ENST00000296953.2:exon4:c.G284A:p.C95Y,CREBRF:ENST00000520420.1:exon4:c.G284A:p.C95Y,	5q35.1	.
5	172755126	C	T	GGCGTCGGTC,CCCGCGCCGG	exonic	STC2	nonsynonymous SNV	STC2:ENST00000265087.4:exon1:c.G71A:p.G24E,	5q35.1	.
5	172755127	C	T	GCGTCGGTCC,CCGCGCCGGG	exonic	STC2	nonsynonymous SNV	STC2:ENST00000265087.4:exon1:c.G70A:p.G24R,	5q35.1	S
5	176314219	G	T	CCAGAACCCT,GGCCACAGTC	exonic	HK3	nonsynonymous SNV	HK3:ENST00000292432.5:exon12:c.C1720A:p.Q574K,	5q35.2	.
5	178559288	G	A	GCTCCTGCAG,GATCTCAAAC	exonic	ADAMTS2	nonsynonymous SNV	ADAMTS2:ENST00000251582.7:exon15:c.C2233T:p.P745S,	5q35.3	.
5	180480308	G	A	AAGCAGCGGA,AAGCCGAGGT	exonic	BTNL9	nonsynonymous SNV	BTNL9:ENST00000327705.9:exon5:c.G845A:p.R282K,BTNL9:ENST00000515271.1:exon4:c.G638A:p.R213K,BTNL9:ENST00000491209.1:exon5:c.G845A:p.R282K,BTNL9:ENST00000376841.2:exon5:c.G845A:p.R282K,BTNL9:ENST00000376842.3:exon5:c.G845A:p.R282K,	5q35.3	.
5	180632079	C	T	GGCGCCGGTT,CGGGGCCGGT	exonic	TRIM7	nonsynonymous SNV	TRIM7:ENST00000334421.5:exon1:c.G32A:p.G11E,TRIM7:ENST00000274773.7:exon1:c.G32A:p.G11E,	5q35.3	.
6	619502	C	T	AGCACTCATT,CATGGAATAG	exonic	EXOC2	nonsynonymous SNV	EXOC2:ENST00000230449.4:exon5:c.G464A:p.G155E,	6p25.3	.
6	3298453	C	T	GGGGAATATC,TTTAAAGACA	splicing	SLC22A23(EN		.	6p25.2	.
6	7231321	C	T	GGCCCCTGCT,CGGCGGCCAC	exonic	RREB1	nonsynonymous SNV	RREB1:ENST00000349384.6:exon10:c.C2989T:p.P997S,RREB1:ENST00000379938.2:exon10:c.C2989T:p.P997S,RREB1:ENST00000379933.3:exon10:c.C2989T:p.P997S,RREB1:ENST00000334984.6:exon10:c.C2989T:p.P997S,	6p24.3	.
6	7231322	C	T	GCCCCTGCTC,GGCGGCCACC	exonic	RREB1	nonsynonymous SNV	RREB1:ENST00000349384.6:exon10:c.C2990T:p.P997L,RREB1:ENST00000379938.2:exon10:c.C2990T:p.P997L,RREB1:ENST00000379933.3:exon10:c.C2990T:p.P997L,RREB1:ENST00000334984.6:exon10:c.C2990T:p.P997L,	6p24.3	.
6	10756712	C	T	TGCTGATGGC,GCCAAAGTTG	exonic	TMEM14B	nonsynonymous SNV	TMEM14B:ENST00000473276.1:exon4:c.C127T:p.R43C,	6p24.2	TI
6	17541330	C	T	TCTTATCCTT,TCAAAAACAT	exonic	CAP2	nonsynonymous SNV	CAP2:ENST00000465994.1:exon7:c.C761T:p.S254F,CAP2:ENST00000378990.2:exon8:c.C875T:p.S292F,CAP2:ENST00000229922.2:exon9:c.C953T:p.S318F,CAP2:ENST00000489374.1:exon6:c.C617T:p.S206F,	6p22.3	.
6	25917001	C	T	GCTGAAAAAA,CCAGGAAAAT	exonic	SLC17A2	nonsynonymous SNV	SLC17A2:ENST00000377850.3:exon8:c.G842A:p.G281D,SLC17A2:ENST00000360488.3:exon8:c.G842A:p.G281D,SLC17A2:ENST00000265425.3:exon7:c.G842A:p.G281D,	6p22.2	SI
6	25983652	C	G	GCAAGAGCCT,AGTCTGGATT	exonic	TRIM38	nonsynonymous SNV	TRIM38:ENST00000357085.3:exon8:c.C1135G:p.Q379E,TRIM38:ENST00000349458.3:exon7:c.C1135G:p.Q379E,	6p22.2	.
6	27216966	G	A	CCTGCTGGAG,CCTGGAAATG	exonic	PRSS16	nonsynonymous SNV	PRSS16:ENST00000230582.3:exon4:c.G425A:p.G142D,PRSS16:ENST00000470870.1:exon4:c.G425A:p.G142D,	6p22.2	.
6	27925880	C	T	GCTGAATCCC,TTATATATAC	exonic	OR2B6	nonsynonymous SNV	OR2B6:ENST00000244623.1:exon1:c.C862T:p.L288F,	6p22.1	.
6	28542640	A	T	ATATACAACT,TTAACACCTG	exonic	SCAND3	nonsynonymous SNV	SCAND3:ENST00000452236.2:exon3:c.T1842A:p.N614K,	6p22.1	.
6	28963410	C	T	TTGGTGAGTT,TGCCTTGATG	exonic	ZNF311	nonsynonymous SNV	ZNF311:ENST00000377179.3:exon7:c.G1369A:p.E457K,	6p22.1	.
6	29395189	G	A	CATCACTGCG,AGGTGTAGAG	exonic	OR11A1	nonsynonymous SNV	OR11A1:ENST00000377147.2:exon1:c.C230T:p.S77F,OR11A1:ENST00000377149.1:exon5:c.C230T:p.S77F,OR11A1:ENST00000377148.1:exon2:c.C230T:p.S77F,	6p22.1	.
6	29429857	C	T	ATCTTCCTGT,CCTGGGGACC	exonic	OR2H1	nonsynonymous SNV	OR2H1:ENST00000377132.1:exon2:c.C311T:p.S104F,OR2H1:ENST00000377133.1:exon4:c.C311T:p.S104F,OR2H1:ENST00000377136.1:exon4:c.C311T:p.S104F,OR2H1:ENST00000396792.2:exon1:c.C311T:p.S104F,OR2H1:ENST00000442615.1:exon2:c.C311T:p.S104F,	6p22.1	.
6	29638955	C	T	CTAGGAAATC,CTTCTGAGTG	exonic	MOG	nonsynonymous SNV	MOG:ENST00000490427.1:exon7:c.C320T:p.P107L,MOG:ENST00000416766.2:exon7:c.C623T:p.P208L,MOG:ENST00000376888.2:exon7:c.C389T:p.P130L,MOG:ENST00000376917.3:exon8:c.C737T:p.P246L,MOG:ENST00000431798.2:exon7:c.C620T:p.P207L,MOG:ENST00000396704.3:exon8:c.C668T:p.P223	6p22.1	.
6	30080285	C	T	TGGAACATCT,CTGGTGCCTC	exonic	TRIM31	nonsynonymous SNV	TRIM31:ENST00000540829.1:exon2:c.G298A:p.E100K,TRIM31:ENST00000376734.3:exon2:c.G298A:p.E100K,	6p22.1	.
6	30618769	A	G	TCTTCCACCT,GCTTCCCAAG	splicing	C6orf136(ENS		.	6p21.33	.
6	30680703	G	A	TGGGGTTGCT,GGATCCTCTC	exonic	MDC1	nonsynonymous SNV	MDC1:ENST00000376405.2:exon5:c.C1016T:p.P339L,MDC1:ENST00000376406.3:exon5:c.C1016T:p.P339L,	6p21.33	.
6	30995010	C	T	GTCTCTACCA,AGGCTCTGAG	exonic	MUC22	nonsynonymous SNV	MUC22:ENST00000561890.1:exon2:c.C1802T:p.T601I,	6p21.33	.
6	31602684	C	T	AATGCTGTTC,TTGTGAGGGT	exonic	PRRC2A	nonsynonymous SNV	PRRC2A:ENST00000376007.4:exon21:c.C5090T:p.P1697L,PRRC2A:ENST00000376033.2:exon21:c.C5090T:p.P1697L,	6p21.33	PI
6	31617074	G	A	CCCCCACCAT,AGTGGCTGAG	exonic	BAG6	nonsynonymous SNV	BAG6:ENST00000439687.2:exon4:c.C325T:p.H109Y,BAG6:ENST00000434444.1:exon4:c.C325T:p.H109Y,BAG6:ENST00000362049.6:exon4:c.C325T:p.H109Y,BAG6:ENST00000424176.1:exon4:c.C325T:p.H109Y,BAG6:ENST00000456286.1:exon3:c.C325T:p.H109Y,BAG6:ENST00000424480.1:exon4:c.C325T:p.	6p21.33	.
6	31750063	G	A	CCTACACACC,GATGTTGTCC	exonic	VARS	nonsynonymous SNV	VARS:ENST00000375663.3:exon17:c.C2149T:p.R717W,	6p21.33	.
6	31940093	G	A	GACGCCCGGG,AGACCGTACG	exonic	STK19	nonsynonymous SNV	STK19:ENST00000460018.1:exon3:c.G235A:p.E79K,STK19:ENST00000375333.2:exon2:c.G235A:p.E79K,STK19:ENST00000375331.2:exon2:c.G235A:p.E79K,	6p21.33	.
6	32023927	G	A	GAGTTCCGTG,GGCTGGGGGT	exonic	TNXB	nonsynonymous SNV	TNXB:ENST00000375244.3:exon24:c.C8168T:p.P2723L,TNXB:ENST00000375247.2:exon24:c.C8168T:p.P2723L,	6p21.33	.
6	34664356	C	T	AGCTCCGGGT,CAGGTCTACG	exonic	C6orf106	nonsynonymous SNV	C6orf106:ENST00000374023.3:exon1:c.G25A:p.D9N,C6orf106:ENST00000374026.3:exon1:c.G25A:p.D9N,	6p21.31	.
6	35027986	G	A	GCAGCAGTAT,AGAGCAAGTT	exonic	ANKS1A	nonsynonymous SNV	ANKS1A:ENST00000360359.3:exon13:c.G2140A:p.E714K,	6p21.31	.
6	35420519	C	T	TTGCTCGAGG,CCTGTGCCGG	exonic	FANCE	nonsynonymous SNV	FANCE:ENST00000229769.2:exon1:c.C197T:p.A66V,	6p21.31	.
6	36181711	C	T	CCTGCACCTT,CTTGTCTGAG	exonic	BRPF3	nonsynonymous SNV	BRPF3:ENST00000534400.1:exon7:c.C2537T:p.S846F,BRPF3:ENST00000357641.6:exon8:c.C2537T:p.S846F,	6p21.31	.
6	38862535	C	T	CGAGATCTTT,CAGAATTTGG	exonic	DNAH8	nonsynonymous SNV	DNAH8:ENST00000327475.6:exon58:c.C8606T:p.S2869F,DNAH8:ENST00000449981.2:exon59:c.C8642T:p.S2881F,DNAH8:ENST00000441566.1:exon54:c.C7883T:p.S2628F,DNAH8:ENST00000359357.3:exon57:c.C7991T:p.S2664F,	6p21.2	.
6	39507930	C	T	CACGTCTATC,ATGCCAGCCA	exonic	KIF6	nonsynonymous SNV	KIF6:ENST00000373213.4:exon9:c.G1011A:p.M337I,KIF6:ENST00000373215.3:exon13:c.G1494A:p.M498I,KIF6:ENST00000373216.3:exon13:c.G1494A:p.M498I,KIF6:ENST00000287152.7:exon13:c.G1494A:p.M498I,	6p21.2	KI
6	39869132	G	A	GCAGCCAGAC,AATTCTTTGG	exonic	DAAM2	nonsynonymous SNV	DAAM2:ENST00000398904.2:exon24:c.G2866A:p.E956K,DAAM2:ENST00000538976.1:exon24:c.G2863A:p.E955K,DAAM2:ENST00000274867.4:exon24:c.G2866A:p.E956K,	6p21.2	.
6	42933802	C	T	ATTCTCCTCA,TTTGGCCCAC	exonic	PEX6	nonsynonymous SNV	PEX6:ENST00000304611.8:exon12:c.G2342A:p.S781N,	6p21.1	.
6	43007922	C	G	AGTATCTGTT,AAAGTGCCCC	exonic	CUL7	nonsynonymous SNV	CUL7:ENST00000535468.1:exon22:c.G4518C:p.L1506F,CUL7:ENST00000265348.3:exon22:c.G4266C:p.L1422F,	6p21.1	.
6	43042360	G	A	TCTGTTTCAG,TCTCCCGGGG	exonic	KLC4	nonsynonymous SNV	KLC4:ENST00000479388.1:exon16:c.G1810A:p.V604I,KLC4:ENST00000347162.5:exon16:c.G1810A:p.V604I,KLC4:ENST00000259708.3:exon16:c.G1864A:p.V622I,KLC4:ENST00000453940.2:exon15:c.G1579A:p.V527I,KLC4:ENST00000394056.2:exon17:c.G1810A:p.V604I,KLC4:ENST00000394058.1:exon16:c.G	6p21.1	.
6	43250948	G	A	CTCCATGGCC,ATGGGGACCT	exonic	TTBK1	nonsynonymous SNV	TTBK1:ENST00000259750.4:exon14:c.G2470A:p.D824N,	6p21.1	.
6	43600975	C	T	AATGGCATGA,CTCAGCTCAC	exonic	MAD2L1BP	nonsynonymous SNV	MAD2L1BP:ENST00000451025.2:exon2:c.C65T:p.T22I,	6p21.1	.
6	44233362	G	A	CGCCTCACGC,GATGGCCCCG	exonic	NFKBIE	nonsynonymous SNV	NFKBIE:ENST00000275015.5:exon1:c.C139T:p.R47C,	6p21.1	.
6	47846320	C	A	GTCCCATGGT,TTGCAAGGAG	exonic	PTCHD4	nonsynonymous SNV	PTCHD4:ENST00000339488.4:exon3:c.G2260T:p.D754Y,	6p12.3	.
6	49705063	G	A	TGGAAAAGAT,GAAGCAGCCC	exonic	CRISP3	nonsynonymous SNV	CRISP3:ENST00000393666.1:exon1:c.C47T:p.P16L,CRISP3:ENST00000354620.4:exon2:c.C116T:p.P39L,CRISP3:ENST00000433368.2:exon2:c.C116T:p.P39L,CRISP3:ENST00000263045.4:exon2:c.C86T:p.P29L,CRISP3:ENST00000371159.4:exon2:c.C140T:p.P47L,	6p12.3	.
6	51890355	C	A	AACTGACCTC,TTCTAGAGTT	exonic	PKHD1	nonsynonymous SNV	PKHD1:ENST00000340994.4:exon32:c.G4253T:p.R1418M,PKHD1:ENST00000371117.3:exon32:c.G4253T:p.R1418M,	6p12.2	.
6	54214603	G	A	GATGGGCGAG,AAAACGGCAT	exonic	TINAG	nonsynonymous SNV	TINAG:ENST00000259782.4:exon7:c.G989A:p.G330E,	6p12.1	.
6	56006608	C	T	CCCTGGTTCT,CTTTGTAACC	exonic	COL21A1	nonsynonymous SNV	COL21A1:ENST00000244728.5:exon12:c.G1517A:p.G506E,COL21A1:ENST00000535941.1:exon12:c.G1517A:p.G506E,COL21A1:ENST00000370819.1:exon11:c.G1508A:p.G503E,	6p12.1	.
6	56006609	C	G	CCTGGTTCTC,TTTGTAACCT	exonic	COL21A1	nonsynonymous SNV	COL21A1:ENST00000244728.5:exon12:c.G1516C:p.G506R,COL21A1:ENST00000535941.1:exon12:c.G1516C:p.G506R,COL21A1:ENST00000370819.1:exon11:c.G1507C:p.G503R,	6p12.1	.
6	70916934	C	T	AGAAGATTGC,TCTATCCTGT	exonic	COL19A1	nonsynonymous SNV	COL19A1:ENST00000393344.1:exon38:c.C2251T:p.L751F,COL19A1:ENST00000322773.4:exon51:c.C3385T:p.L1129F,	6q13	.
6	74073314	C	T	TGTCGCCACT,AGAAGGCCGA	exonic	KHDC3L	stopgain	KHDC3L:ENST00000370367.3:exon3:c.C385T:p.Q129X,	6q13	.
6	74517887	C	T	TACTCTAGCC,TTATAACTTA	exonic	CD109	nonsynonymous SNV	CD109:ENST00000422508.2:exon25:c.C3040T:p.L1014F,CD109:ENST00000437994.2:exon26:c.C3271T:p.L1091F,CD109:ENST00000287097.5:exon26:c.C3271T:p.L1091F,	6q13	.
6	76022532	G	A	ATCTGAATAG,GGATGTGGGC	exonic	FILIP1	nonsynonymous SNV	FILIP1:ENST00000393004.2:exon5:c.C3016T:p.P1006S,FILIP1:ENST00000370020.1:exon3:c.C2719T:p.P907S,FILIP1:ENST00000237172.7:exon5:c.C3016T:p.P1006S,	6q14.1	.
6	76072476	T	G	AATCGGTTTC,CATAGACATC	exonic	FILIP1	nonsynonymous SNV	FILIP1:ENST00000393004.2:exon3:c.A434C:p.E145A,FILIP1:ENST00000370020.1:exon1:c.A137C:p.E46A,FILIP1:ENST00000237172.7:exon3:c.A434C:p.E145A,	6q14.1	.
6	76660497	G	A	TATTCGCTGA,CTCTGGTACC	exonic	IMPG1	nonsynonymous SNV	IMPG1:ENST00000369950.3:exon13:c.C1606T:p.L536F,	6q14.1	.
6	76712677	G	A	TCAAGCTGGG,TTCAACAGGA	exonic	IMPG1	nonsynonymous SNV	IMPG1:ENST00000369950.3:exon12:c.C1249T:p.P417S,	6q14.1	.
6	82459412	T	G	TAAATGATTC,TAATTGCAGG	exonic	FAM46A	stoploss	FAM46A:ENST00000369756.3:exon3:c.A1572C:p.X524Y,FAM46A:ENST00000320172.6:exon3:c.A1329C:p.X443Y,FAM46A:ENST00000369754.3:exon3:c.A1386C:p.X462Y,	6q14.1	.
6	83834885	C	T	CATTTTGAAG,CTACTAGAAG	exonic	DOPEY1	nonsynonymous SNV	DOPEY1:ENST00000349129.2:exon14:c.C1456T:p.P486S,DOPEY1:ENST00000369739.3:exon13:c.C1429T:p.P477S,	6q14.1	.
6	85469973	C	A	ATCACTGTAC,TGTATCTTTT	exonic	TBX18	nonsynonymous SNV	TBX18:ENST00000606784.1:exon3:c.G125T:p.R42M,TBX18:ENST00000369663.5:exon3:c.G599T:p.R200M,TBX18:ENST00000606325.1:exon3:c.G125T:p.R42M,	6q14.3	.
6	85472302	G	A	CCTATCTCAT,AAAGCGCTTC	exonic	TBX18	nonsynonymous SNV	TBX18:ENST00000369663.5:exon2:c.C457T:p.H153Y,	6q14.3	.
6	88387662	G	A	TTTTTTAATG,TGAGGATGCT	exonic	AKIRIN2	nonsynonymous SNV	AKIRIN2:ENST00000257787.5:exon3:c.C403T:p.P135S,	6q15	.
6	89808568	C	T	GCCAAAATTC,TTCTTGGAGT	exonic	SRSF12	nonsynonymous SNV	SRSF12:ENST00000452027.2:exon5:c.G515A:p.R172K,	6q15	.
6	90605632	G	A	TCAATGGTAA,ACAGGCAGCC	exonic	GJA10	nonsynonymous SNV	GJA10:ENST00000369352.1:exon1:c.G1445A:p.R482K,	6q15	.
6	105406115	G	A	ATGATAAACC,AGAGGGAAGC	exonic	LIN28B	nonsynonymous SNV	LIN28B:ENST00000345080.4:exon2:c.G152A:p.R51Q,	6q16.3	.
6	108204329	C	T	ACTTCTTCTT,ATCTTCCTTT	exonic	SEC63	nonsynonymous SNV	SEC63:ENST00000369002.4:exon17:c.G1696A:p.E566K,	6q21	.
6	109835540	G	T	TTTTTCTTTT,TTCTTTTACT	exonic	AK9	nonsynonymous SNV	AK9:ENST00000424296.2:exon32:c.C4166A:p.T1389K,	6q21	.
6	110056408	C	T	CTTGCGAATG,CCCTGGAGAT	exonic	FIG4	nonsynonymous SNV	FIG4:ENST00000454215.1:exon6:c.C490T:p.P164S,FIG4:ENST00000230124.3:exon6:c.C553T:p.P185S,FIG4:ENST00000441478.2:exon4:c.C115T:p.P39S,FIG4:ENST00000368941.1:exon4:c.C322T:p.P108S,	6q21	.
6	110081452	G	A	TTTTGATAAA,GAACGAGAGA	splicing	FIG4(ENST0C		.	6q21	.
6	111696619	A	T	TCTATTAATA,TAATATACTT	exonic	REV3L	nonsynonymous SNV	REV3L:ENST00000435970.1:exon15:c.T2705A:p.I902N,REV3L:ENST00000368802.3:exon13:c.T2939A:p.I980N,REV3L:ENST00000358835.3:exon14:c.T2939A:p.I980N,REV3L:ENST00000368805.1:exon14:c.T2939A:p.I980N,	6q21	.
6	116265466	G	A	TCACCAACGA,GACATTTCTG	exonic	FRK	nonsynonymous SNV	FRK:ENST00000538210.1:exon5:c.C655T:p.L219F,FRK:ENST00000606080.1:exon6:c.C1081T:p.L361F,	6q22.1	.
6	117240336	G	A	CTAAAAATTG,GAACAGTGGG	exonic	RFX6	stopgain	RFX6:ENST00000332958.2:exon11:c.G1059A:p.W353X,	6q22.1	.
6	117679064	C	T	AGATCAACAT,AAATACTTGC	exonic	ROS1	nonsynonymous SNV	ROS1:ENST00000368508.3:exon24:c.G3757A:p.D1253N,ROS1:ENST00000368507.3:exon25:c.G3742A:p.D1248N,	6q22.1	.
6	117683852	C	T	ATCCAGTCTT,ACATGTTTTG	exonic	ROS1	nonsynonymous SNV	ROS1:ENST00000368508.3:exon21:c.G3295A:p.E1099K,ROS1:ENST00000368507.3:exon22:c.G3280A:p.E1094K,	6q22.1	.
6	118786694	G	T	TTTCTGTGCT,TGGTCATTCT	exonic	CEP85L	nonsynonymous SNV	CEP85L:ENST00000368491.3:exon13:c.C2292A:p.H764Q,CEP85L:ENST00000368488.5:exon14:c.C2301A:p.H767Q,	6q22.31	.
6	119243217	G	A	AACCTTCATA,ATCGTGGAAT	exonic	MCM9	nonsynonymous SNV	MCM9:ENST00000316316.6:exon4:c.C656T:p.S219F,MCM9:ENST00000316068.3:exon4:c.C656T:p.S219F,	6q22.31	.
6	119338045	C	T	TTCCAATCTA,TTTGCAGGTT	exonic	FAM184A	nonsynonymous SNV	FAM184A:ENST00000522284.1:exon5:c.G1037A:p.S346N,FAM184A:ENST00000352896.5:exon5:c.G1037A:p.S346N,FAM184A:ENST00000338891.7:exon5:c.G1397A:p.S466N,FAM184A:ENST00000521531.1:exon5:c.G1397A:p.S466N,FAM184A:ENST00000368475.4:exon5:c.G1037A:p.S346N,	6q22.31	.
6	123022645	G	A	GTCACACCAG,GTATAGTTAA	exonic	PKIB	nonsynonymous SNV	PKIB:ENST00000258014.3:exon3:c.G13A:p.D5N,	6q22.31	.
6	128135068	C	T	CGGATTATAT,TTTTCGAACT	exonic	THEMIS	nonsynonymous SNV	THEMIS:ENST00000368248.2:exon4:c.G718A:p.D240N,THEMIS:ENST00000434358.1:exon3:c.G22A:p.D8N,THEMIS:ENST00000543064.1:exon4:c.G718A:p.D240N,THEMIS:ENST00000368250.1:exon5:c.G481A:p.D161N,THEMIS:ENST00000537166.1:exon4:c.G613A:p.D205N,	6q22.33	.
6	131190945	C	T	TCCTCCCTCT,TACTACCTTG	exonic	EPB41L2	nonsynonymous SNV	EPB41L2:ENST00000368128.2:exon15:c.G2365A:p.E789K,EPB41L2:ENST00000527411.1:exon13:c.G2155A:p.E719K,EPB41L2:ENST00000527423.1:exon5:c.G562A:p.E188K,EPB41L2:ENST00000337057.3:exon15:c.G2365A:p.E789K,EPB41L2:ENST00000529208.1:exon13:c.G2155A:p.E719K,EPB41L2:ENST0	6q23.1	.
6	134491512	G	A	CTTGCCAATG,AGTTGGGGAC	exonic	SGK1	nonsynonymous SNV	SGK1:ENST00000367857.5:exon11:c.C1160T:p.S387F,SGK1:ENST00000475719.2:exon11:c.C1058T:p.S353F,SGK1:ENST00000413996.3:exon12:c.C1232T:p.S411F,SGK1:ENST00000528577.1:exon12:c.C1274T:p.S425F,SGK1:ENST00000237305.7:exon12:c.C1190T:p.S397F,SGK1:ENST00000367858.5:exon1	6q23.2	.
6	135776925	G	A	AGCAAATAGG,AAAATTTTCA	exonic	AHI1	nonsynonymous SNV	AHI1:ENST00000531788.1:exon9:c.C1291T:p.P431S,AHI1:ENST00000367800.4:exon8:c.C1291T:p.P431S,AHI1:ENST00000327035.6:exon9:c.C1291T:p.P431S,AHI1:ENST00000457866.2:exon9:c.C1291T:p.P431S,AHI1:ENST00000265602.6:exon10:c.C1291T:p.P431S,	6q23.3	.
6	137325888	G	A	CTTGAACTCT,ATGATTGATC	exonic	IL20RA	nonsynonymous SNV	IL20RA:ENST00000541547.1:exon6:c.C587T:p.S196L,IL20RA:ENST00000316649.5:exon6:c.C734T:p.S245L,IL20RA:ENST00000367748.1:exon5:c.C401T:p.S134L,	6q23.3	.
6	138428270	C	T	TCACCGTACT,CATGAGGCTC	exonic	PERP	nonsynonymous SNV	PERP:ENST00000421351.3:exon1:c.G208A:p.E70K,	6q23.3	.
6	139094917	G	A	TAAACAAACG,AGCTGCGGAG	exonic	CCDC28A	nonsynonymous SNV	CCDC28A:ENST00000332797.6:exon1:c.G106A:p.E36K,	6q24.1	C
6	147122980	C	T	AGATACAGAT,CTCTGCTGCA	exonic	ADGB	nonsynonymous SNV	ADGB:ENST00000367490.1:exon11:c.C1525T:p.P509S,ADGB:ENST00000397944.3:exon35:c.C4651T:p.P1551S,	6q24.3	.
6	147588235	G	C	GATTGGCTTT,AATCTGGAAC	exonic	STXBP5	nonsynonymous SNV	STXBP5:ENST00000367480.3:exon7:c.G646C:p.E216Q,STXBP5:ENST00000546097.1:exon7:c.G646C:p.E216Q,STXBP5:ENST00000321680.6:exon7:c.G646C:p.E216Q,STXBP5:ENST00000367481.3:exon7:c.G646C:p.E216Q,	6q24.3	.
6	151204021	G	A	AGATGTGGAT,GGTAAGAAAA	exonic	MTHFD1L	nonsynonymous SNV	MTHFD1L:ENST00000367307.4:exon5:c.G541A:p.G181R,MTHFD1L:ENST00000367321.3:exon5:c.G541A:p.G181R,MTHFD1L:ENST00000443074.1:exon1:c.G76A:p.G26R,MTHFD1L:ENST00000425276.1:exon1:c.G73A:p.G25R,MTHFD1L:ENST00000423867.1:exon5:c.G211A:p.G71R,	6q25.1	.
6	152382154	G	A	AGAGGGCATG,TGGAGATCTT	exonic	ESR1	nonsynonymous SNV	ESR1:ENST00000443427.1:exon7:c.G1264A:p.V422M,ESR1:ENST00000440973.1:exon8:c.G1264A:p.V422M,ESR1:ENST00000456483.2:exon6:c.G928A:p.V310M,ESR1:ENST00000415488.1:exon3:c.G283A:p.V95M,ESR1:ENST00000427531.2:exon6:c.G745A:p.V249M,ESR1:ENST00000206249.3:exon6:c.G126	6q25.1	.
6	157502202	T	C	ATACCTCACC,TCATGGAAGA	exonic	ARID1B	nonsynonymous SNV	ARID1B:ENST00000275248.4:exon12:c.T3181C:p.F1061L,ARID1B:ENST00000350026.5:exon11:c.T3196C:p.F1066L,ARID1B:ENST00000414678.2:exon11:c.T1762C:p.F588L,ARID1B:ENST00000400790.3:exon4:c.T397C:p.F133L,ARID1B:ENST00000319584.6:exon10:c.T1621C:p.F541L,ARID1B:ENST00000346	6q25.3	.
6	159044602	C	T	TTACATGATC,AATGTACCAG	exonic	TMEM181	nonsynonymous SNV	TMEM181:ENST00000367090.3:exon11:c.C1328T:p.P443L,	6q25.3	.
6	159462505	C	T	CTGAATATCC,TTCCGTTGAA	exonic	TAGAP	nonsynonymous SNV	TAGAP:ENST00000338313.5:exon6:c.G358A:p.G120R,TAGAP:ENST00000367066.3:exon6:c.G358A:p.G120R,	6q25.3	.
6	160445697	C	T	TGACTCCGAT,CGGACACTTC	exonic	IGF2R	nonsynonymous SNV	IGF2R:ENST00000356956.1:exon5:c.C607T:p.P203S,	6q25.3	.
6	160494437	C	T	ATGCTCATCT,CCTGGACAAG	exonic	IGF2R	nonsynonymous SNV	IGF2R:ENST00000356956.1:exon34:c.C4883T:p.S1628F,	6q25.3	.
6	163483278	G	A	TGCTCGGCAA,GAATCCACGA	exonic	PACRG	nonsynonymous SNV	PACRG:ENST00000366888.2:exon3:c.G388A:p.G130R,PACRG:ENST00000337019.3:exon4:c.G388A:p.G130R,PACRG:ENST00000366889.2:exon4:c.G388A:p.G130R,	6q26	.
6	163876449	C	T	AAAGAATACC,AGATGTAAGT	exonic	QKI	nonsynonymous SNV	QKI:ENST00000392127.2:exon2:c.C281T:p.P94L,QKI:ENST00000537041.1:exon2:c.C116T:p.P39L,QKI:ENST00000453779.2:exon2:c.C281T:p.P94L,QKI:ENST00000361758.4:exon2:c.C281T:p.P94L,QKI:ENST00000537124.1:exon2:c.C116T:p.P39L,QKI:ENST00000361752.3:exon2:c.C281T:p.P94L,QKI:ENST000	6q26	.
6	165756906	C	T	GCATATATAT,ATTTGCCGTC	exonic	PDE10A	nonsynonymous SNV	PDE10A:ENST00000354448.4:exon19:c.G2041A:p.D681N,PDE10A:ENST00000539869.2:exon19:c.G2071A:p.D691N,PDE10A:ENST00000366882.1:exon20:c.G2041A:p.D681N,	6q27	.
6	165808749	G	A	ATGTCAAAGT,GAATCTGAAA	exonic	PDE10A	nonsynonymous SNV	PDE10A:ENST00000354448.4:exon15:c.C1396T:p.H466Y,PDE10A:ENST00000539869.2:exon15:c.C1426T:p.H476Y,PDE10A:ENST00000366882.1:exon16:c.C1396T:p.H466Y,	6q27	.
6	167754736	C	T	GCTTGATGCT,CTGACTGTCT	exonic	TTLL2	nonsynonymous SNV	TTLL2:ENST00000515138.1:exon3:c.C1348T:p.P450S,TTLL2:ENST00000239587.5:exon3:c.C1348T:p.P450S,	6q27	.
6	169642033	C	T	TCTGTGTTCT,ACTGATGGCG	exonic	THBS2	nonsynonymous SNV	THBS2:ENST00000366787.3:exon6:c.G715A:p.E239K,	6q27	.
7	934976	C	T	TCCAGGGAAC,TTCTGACCAG	exonic	GET4	nonsynonymous SNV	GET4:ENST00000265857.3:exon9:c.C901T:p.L301F,GET4:ENST00000407192.1:exon8:c.C742T:p.L248F,	7p22.3	.
7	2685390	C	T	GGGCCTCTTC,ATGTTCACCC	exonic	TTYH3	nonsynonymous SNV	TTYH3:ENST00000400376.2:exon1:c.C14T:p.P5L,	7p22.3	.
7	3658827	G	A	AGTTCAAGTG,ATGCGCGATG	exonic	SDK1	stopgain	SDK1:ENST00000404826.2:exon2:c.G414A:p.W138X,SDK1:ENST00000389531.3:exon2:c.G414A:p.W138X,	7p22.2	SI
7	5239248	C	T	AAATTTTTCT,CCTTTCTTCT	exonic	WIPI2	nonsynonymous SNV	WIPI2:ENST00000288828.4:exon3:c.C170T:p.S57F,WIPI2:ENST00000382384.2:exon2:c.C116T:p.S39F,WIPI2:ENST00000401525.3:exon2:c.C116T:p.S39F,WIPI2:ENST00000404704.3:exon3:c.C170T:p.S57F,	7p22.1	.
7	5267842	G	A	AAGCAGCACC,GTGAGTCTGC	exonic	WIPI2	nonsynonymous SNV	WIPI2:ENST00000288828.4:exon11:c.G1121A:p.R374Q,WIPI2:ENST00000382384.2:exon10:c.G1067A:p.R356Q,WIPI2:ENST00000401525.3:exon10:c.G1067A:p.R356Q,WIPI2:ENST00000484262.1:exon8:c.G944A:p.R315Q,WIPI2:ENST00000404704.3:exon11:c.G1121A:p.R374Q,	7p22.1	.
7	5267843	G	A	AGCAGCACCG,TGAGTCTGCT	splicing	WIPI2(ENST0		.	7p22.1	.
7	17873770	G	A	TCTGAAGGAA,GATAAAATCT	exonic	SNX13	nonsynonymous SNV	SNX13:ENST00000428135.3:exon15:c.C1502T:p.P501L,SNX13:ENST00000409389.1:exon15:c.C1502T:p.P501L,	7p21.1	.
7	17873771	G	A	CTGAAGGAAG,ATAAAATCTT	exonic	SNX13	nonsynonymous SNV	SNX13:ENST00000428135.3:exon15:c.C1501T:p.P501S,SNX13:ENST00000409389.1:exon15:c.C1501T:p.P501S,	7p21.1	.
7	18633565	C	T	TCAAAGCTCT,CACCCCTTAG	exonic	HDAC9	nonsynonymous SNV	HDAC9:ENST00000406451.4:exon6:c.C568T:p.P190S,HDAC9:ENST00000441542.2:exon5:c.C577T:p.P193S,HDAC9:ENST00000406072.1:exon5:c.C661T:p.P221S,HDAC9:ENST00000456174.2:exon5:c.C484T:p.P162S,HDAC9:ENST00000417496.2:exon8:c.C694T:p.P232S,HDAC9:ENST00000405010.3:exon6:c.	7p21.1	.
7	20683212	C	T	GTGACTCTAT,CACGTCTCCT	exonic	ABCB5	nonsynonymous SNV	ABCB5:ENST00000404938.2:exon7:c.C635T:p.S212F,	7p21.1	.
7	24901342	G	A	TGACAAATTA,GATTGGAGGA	exonic	OSBPL3	nonsynonymous SNV	OSBPL3:ENST00000313367.2:exon10:c.C917T:p.P306L,OSBPL3:ENST00000352860.1:exon9:c.C824T:p.P275L,OSBPL3:ENST00000396429.1:exon9:c.C917T:p.P306L,OSBPL3:ENST00000409555.1:exon8:c.C824T:p.P275L,OSBPL3:ENST00000409069.1:exon8:c.C824T:p.P275L,OSBPL3:ENST00000353930.1:e	7p15.3	.
7	25266263	G	A	TTTTTGATCG,GATTCTGGAT	exonic	NPVF	nonsynonymous SNV	NPVF:ENST00000222674.2:exon2:c.C521T:p.P174L,	7p15.3	.
7	30830914	C	T	AGCAACAGCT,CCCCTCCAGG	exonic	FAM188B	nonsynonymous SNV	FAM188B:ENST00000265299.6:exon5:c.C797T:p.S266F,	7p14.3	.
7	37382228	C	T	TGATCAATTT,CATGAGTTTG	exonic	ELMO1	nonsynonymous SNV	ELMO1:ENST00000455119.1:exon1:c.G67A:p.E23K,ELMO1:ENST00000453399.1:exon3:c.G67A:p.E23K,ELMO1:ENST00000442504.1:exon2:c.G67A:p.E23K,ELMO1:ENST00000310758.4:exon2:c.G67A:p.E23K,ELMO1:ENST00000455879.1:exon2:c.G67A:p.E23K,ELMO1:ENST00000445322.1:exon3:c.G67A:p.E2	7p14.1	.
7	43351468	C	T	AGCTACAACC,CGACCAGTTC	exonic	HECW1	nonsynonymous SNV	HECW1:ENST00000453890.1:exon3:c.C134T:p.P45L,HECW1:ENST00000395891.2:exon4:c.C134T:p.P45L,	7p13	.
7	44144464	G	A	CCGCGGGTCC,AAAAGCCCAG	exonic	AEBP1	nonsynonymous SNV	AEBP1:ENST00000223357.3:exon1:c.G200A:p.R67Q,	7p13	.
7	44147260	G	A	ACCCACACTG,ACTACAATGA	exonic	AEBP1	nonsynonymous SNV	AEBP1:ENST00000223357.3:exon4:c.G700A:p.D234N,AEBP1:ENST00000449162.1:exon4:c.G448A:p.D150N,	7p13	.
7	44179411	G	A	TTTGCCGCTG,GGTCAAACAT	exonic	MYL7	nonsynonymous SNV	MYL7:ENST00000447951.1:exon4:c.C434T:p.P145L,MYL7:ENST00000446581.1:exon2:c.C128T:p.P43L,MYL7:ENST00000223364.3:exon5:c.C347T:p.P116L,MYL7:ENST00000457314.1:exon4:c.C413T:p.P138L,MYL7:ENST00000458240.1:exon4:c.C266T:p.P89L,	7p13	.
7	44179412	G	A	TTGCCGCTGG,GTCAAACATG	exonic	MYL7	nonsynonymous SNV	MYL7:ENST00000447951.1:exon4:c.C433T:p.P145S,MYL7:ENST00000446581.1:exon2:c.C127T:p.P43S,MYL7:ENST00000223364.3:exon5:c.C346T:p.P116S,MYL7:ENST00000457314.1:exon4:c.C412T:p.P138S,MYL7:ENST00000458240.1:exon4:c.C265T:p.P89S,	7p13	.
7	45649982	G	A	GTCCAGGAGC,GCTCCTCATG	exonic	ADCY1	nonsynonymous SNV	ADCY1:ENST00000432715.1:exon4:c.G119A:p.R40Q,ADCY1:ENST00000297323.7:exon3:c.G794A:p.R265Q,	7p12.3	AI
7	45650020	G	A	CGTTGCCATG,AGATGAAGGA	exonic	ADCY1	nonsynonymous SNV	ADCY1:ENST00000432715.1:exon4:c.G157A:p.E53K,ADCY1:ENST00000297323.7:exon3:c.G832A:p.E278K,	7p12.3	.
7	53103759	G	A	AACCCAGGAC,GACCTGGAGC	exonic	POM121L12	nonsynonymous SNV	POM121L12:ENST00000408890.4:exon1:c.G395A:p.R132Q,	7p12.1	P
7	63727047	C	T	TAAGATAATT,ATACTGGAGA	exonic	ZNF679	nonsynonymous SNV	ZNF679:ENST00000255746.4:exon4:c.C1036T:p.H346Y,ZNF679:ENST00000421025.1:exon5:c.C1036T:p.H346Y,	7q11.21	.
7	72891305	G	A	ATCTACTTGG,GCTCAAACTT	exonic	BAZ1B	nonsynonymous SNV	BAZ1B:ENST00000404251.1:exon7:c.C2486T:p.P829L,BAZ1B:ENST00000339594.4:exon7:c.C2486T:p.P829L,	7q11.23	.
7	73021345	G	A	CTGGGCTTAC,GAGCTGCAGG	exonic	MLXIPL	nonsynonymous SNV	MLXIPL:ENST00000456640.1:exon4:c.C463T:p.R155C,MLXIPL:ENST00000313375.3:exon5:c.C577T:p.R193C,MLXIPL:ENST00000345114.5:exon5:c.C577T:p.R193C,MLXIPL:ENST00000414749.2:exon5:c.C577T:p.R193C,MLXIPL:ENST00000354613.1:exon5:c.C577T:p.R193C,MLXIPL:ENST00000429400.2:exon	7q11.23	.
7	73971990	C	T	GCGAGGCTCT,ACGGATCGAC	exonic	GTF2IRD1	nonsynonymous SNV	GTF2IRD1:ENST00000424337.2:exon20:c.C2045T:p.S682L,GTF2IRD1:ENST00000265755.3:exon20:c.C2090T:p.S697L,GTF2IRD1:ENST00000476977.1:exon20:c.C2045T:p.S682L,GTF2IRD1:ENST00000455841.2:exon20:c.C2141T:p.S714L,	7q11.23	.
7	78636499	G	A	AGGTAGTGAC,AAGGTCTTTA	exonic	MAGI2	nonsynonymous SNV	MAGI2:ENST00000354212.4:exon2:c.C325T:p.R109C,MAGI2:ENST00000522391.1:exon2:c.C325T:p.R109C,MAGI2:ENST00000419488.1:exon2:c.C325T:p.R109C,	7q21.11	.
7	86522373	C	T	CAAAGAAATT,CTTTAATGCA	exonic	KIAA1324L	nonsynonymous SNV	KIAA1324L:ENST00000450689.2:exon20:c.G2729A:p.G910E,KIAA1324L:ENST00000444627.1:exon20:c.G2516A:p.G839E,KIAA1324L:ENST00000416314.1:exon19:c.G2228A:p.G743E,KIAA1324L:ENST00000297222.6:exon16:c.G2009A:p.G670E,	7q21.12	.
7	87175237	C	T	ATCATGATTT,CTTTCTCCAC	exonic	ABCB1	nonsynonymous SNV	ABCB1:ENST00000543898.1:exon13:c.G1637A:p.G546E,ABCB1:ENST00000265724.3:exon16:c.G1829A:p.G610E,	7q21.12	.
7	89854787	G	A	ATCCAATGCT,AATATTTGGC	exonic	STEAP2	nonsynonymous SNV	STEAP2:ENST00000394629.2:exon3:c.G391A:p.E131K,STEAP2:ENST00000402625.2:exon2:c.G391A:p.E131K,STEAP2:ENST00000394621.2:exon3:c.G391A:p.E131K,STEAP2:ENST00000428074.1:exon2:c.G391A:p.E131K,STEAP2:ENST00000394622.2:exon3:c.G391A:p.E131K,STEAP2:ENST00000287908.3:e	7q21.13	.
7	91863877	A	G	GCTTCGGTGG,GAGGAAAATC	exonic	KRIT1	nonsynonymous SNV	KRIT1:ENST00000412043.2:exon10:c.T875C:p.L292P,KRIT1:ENST00000340022.2:exon10:c.T875C:p.L292P,KRIT1:ENST00000455116.1:exon3:c.T161C:p.L54P,KRIT1:ENST00000394507.1:exon11:c.T875C:p.L292P,KRIT1:ENST00000394505.2:exon10:c.T875C:p.L292P,	7q21.2	.
7	92761232	G	T	TAGCATCTTT,TAGTTTGGAT	exonic	SAMD9L	stopgain	SAMD9L:ENST00000318238.4:exon5:c.C4053A:p.Y1351X,SAMD9L:ENST00000411955.1:exon6:c.C4053A:p.Y1351X,SAMD9L:ENST00000437805.1:exon6:c.C4053A:p.Y1351X,	7q21.2	.
7	92855681	C	T	AGGGCCTTAC,GAAGAGAAGG	exonic	HEPACAM2	nonsynonymous SNV	HEPACAM2:ENST00000394468.2:exon1:c.G79A:p.G27S,HEPACAM2:ENST00000453812.2:exon1:c.G79A:p.D27N,	7q21.3	.
7	92953022	C	T	GCAATATATA,CTTTTTTGGT	exonic	CCDC132	nonsynonymous SNV	CCDC132:ENST00000535481.1:exon11:c.C1115T:p.T372I,CCDC132:ENST00000544910.1:exon22:c.C1865T:p.T622I,CCDC132:ENST00000541136.1:exon20:c.C1388T:p.T463I,CCDC132:ENST00000305866.5:exon21:c.C1955T:p.T652I,	7q21.3	.
7	97488654	G	A	TAGTGTCCAG,AAGAAAAGGC	exonic	ASNS	nonsynonymous SNV	ASNS:ENST00000394308.3:exon5:c.C544T:p.P182S,ASNS:ENST00000454046.1:exon4:c.C544T:p.P182S,ASNS:ENST00000437628.1:exon3:c.C295T:p.P99S,ASNS:ENST00000442734.1:exon5:c.C544T:p.P182S,ASNS:ENST00000444334.1:exon5:c.C481T:p.P161S,ASNS:ENST00000175506.4:exon6:c.C544T:p.F	7q21.3	.
7	99817943	G	A	GGCCCGCTGG,AAACCCAGAG	exonic	PVRIG	nonsynonymous SNV	PVRIG:ENST00000317271.2:exon3:c.G325A:p.E109K,	7q22.1	.
7	100088164	G	A	GCCCGGGCCT,GAATGGCAGT	exonic	NYAP1	stopgain	NYAP1:ENST00000454988.1:exon3:c.G1802A:p.W601X,NYAP1:ENST00000300179.2:exon5:c.G1970A:p.W657X,NYAP1:ENST00000423930.1:exon5:c.G1973A:p.W658X,	7q22.1	.
7	100088165	G	A	CCCGGGCCTG,AATGGCAGTG	exonic	NYAP1	stopgain	NYAP1:ENST00000454988.1:exon3:c.G1803A:p.W601X,NYAP1:ENST00000300179.2:exon5:c.G1971A:p.W657X,NYAP1:ENST00000423930.1:exon5:c.G1974A:p.W658X,	7q22.1	.
7	100175181	C	T	GGTCGCTGCT,CTAAGGAGAG	exonic	LRCH4	nonsynonymous SNV	LRCH4:ENST00000310300.6:exon10:c.G1117A:p.E373K,	7q22.1	.
7	100175182	C	T	GTCGCTGCTC,TAAGGAGAGA	splicing	LRCH4(ENST		.	7q22.1	.
7	100175358	C	T	ACAGGGTCTC,GTCCGCTGGG	exonic	LRCH4	nonsynonymous SNV	LRCH4:ENST00000310300.6:exon9:c.G1045A:p.G349R,	7q22.1	.
7	100334193	G	A	GACTGGGTTC,AGCCAGTGGG	exonic	ZAN	nonsynonymous SNV	ZAN:ENST00000538115.1:exon3:c.G194A:p.R65Q,ZAN:ENST00000546292.1:exon3:c.G194A:p.R65Q,ZAN:ENST00000542585.1:exon3:c.G194A:p.R65Q,	7q22.1	Z
7	100456784	G	T	CTTGTGACAG,TGTCTGGGGA	splicing	SLC12A9(EN		.	7q22.1	.
7	100635288	G	C	AGCGTTACCC,GCAGTACCAC	exonic	MUC12	nonsynonymous SNV	MUC12:ENST00000536621.1:exon2:c.G1444C:p.G482R,MUC12:ENST00000379442.3:exon5:c.G1873C:p.G625R,	7q22.1	.
7	100657276	G	A	TGGGATCGTG,GGGCTGTGAT	exonic	MUC12	nonsynonymous SNV	MUC12:ENST00000536621.1:exon9:c.G15721A:p.G5241R,MUC12:ENST00000379442.3:exon12:c.G16150A:p.G5384R,MUC12:ENST00000305119.3:exon2:c.G172A:p.G58R,	7q22.1	.
7	100681667	G	A	TCCCACTGCT,AAGGTACCAG	exonic	MUC17	nonsynonymous SNV	MUC17:ENST00000306151.4:exon3:c.G6970A:p.E2324K,MUC17:ENST00000379439.3:exon3:c.G6970A:p.E2324K,	7q22.1	.
7	102087396	C	T	TTCTACCGCT,CCTGGTGCGC	exonic	ORAI2	nonsynonymous SNV	ORAI2:ENST00000473939.1:exon3:c.C662T:p.S221F,ORAI2:ENST00000478730.2:exon4:c.C662T:p.S221F,ORAI2:ENST00000403646.3:exon3:c.C662T:p.S221F,ORAI2:ENST00000356387.2:exon4:c.C662T:p.S221F,ORAI2:ENST00000495936.1:exon4:c.C662T:p.S221F,	7q22.1	O
7	103113290	C	G	CCTTCGTCTT,TGTTGTAGAA	exonic	RELN	nonsynonymous SNV	RELN:ENST00000343529.5:exon64:c.G10346C:p.R3449T,RELN:ENST00000424685.2:exon65:c.G10352C:p.R3451T,RELN:ENST00000428762.1:exon65:c.G10352C:p.R3451T,	7q22.1	.
7	105206038	G	A	GATATGACTC,GAATCTTTTC	exonic	EFCAB10,RIN	nonsynonymous SNV	RINT1:ENST00000257700.2:exon14:c.G2129A:p.R710Q,	7q22.3	.
7	111398805	C	T	TGCAGGACCT,CTGGCTCTCT	exonic	DOCK4	nonsynonymous SNV	DOCK4:ENST00000437633.1:exon40:c.G4177A:p.E1393K,DOCK4:ENST00000494651.2:exon10:c.G826A:p.E276K,DOCK4:ENST00000428084.1:exon41:c.G4204A:p.E1402K,	7q31.1	.
7	111982627	C	T	TATTATTTTC,AACCTATGAA	exonic	ZNF277	nonsynonymous SNV	ZNF277:ENST00000421864.2:exon4:c.C200T:p.P67L,ZNF277:ENST00000361822.3:exon12:c.C1196T:p.P399L,	7q31.1	.
7	113519990	C	T	GTAAAAATCT,CCTTTACGGA	exonic	PPP1R3A	nonsynonymous SNV	PPP1R3A:ENST00000284601.3:exon4:c.G1157A:p.G386E,PPP1R3A:ENST00000449795.1:exon4:c.G194A:p.G65E,	7q31.1	.
7	114619749	C	T	TAGAAAAATT,AGTCCAGCTT	exonic	MDFIC	stopgain	MDFIC:ENST00000393486.1:exon4:c.C406T:p.Q136X,MDFIC:ENST00000431629.1:exon3:c.C376T:p.Q126X,MDFIC:ENST00000498196.1:exon3:c.C241T:p.Q81X,MDFIC:ENST00000427207.1:exon3:c.C364T:p.Q122X,MDFIC:ENST00000257724.3:exon4:c.C733T:p.Q245X,	7q31.2	.
7	115594655	C	T	TTGTGGTTGT,CTTTTTTTGT	exonic	TFEC	nonsynonymous SNV	TFEC:ENST00000393485.1:exon4:c.G337A:p.D113N,TFEC:ENST00000320239.7:exon4:c.G337A:p.D113N,TFEC:ENST00000484212.1:exon7:c.G694A:p.D232N,TFEC:ENST00000457268.1:exon3:c.G223A:p.D75N,TFEC:ENST00000265440.7:exon5:c.G424A:p.D142N,	7q31.2	.
7	117180272	G	A	ACTAATCAAA,GAATCATCCT	exonic	CFTR	nonsynonymous SNV	CFTR:ENST00000426809.1:exon7:c.G898A:p.G300R,CFTR:ENST00000003084.6:exon8:c.G988A:p.G330R,CFTR:ENST00000454343.1:exon8:c.G988A:p.G330R,	7q31.2	.
7	121678932	G	A	AAACCTCGTG,AGAAAGGAAG	exonic	PTPRZ1	nonsynonymous SNV	PTPRZ1:ENST00000393386.2:exon19:c.G5491A:p.E1831K,PTPRZ1:ENST00000449182.1:exon18:c.G2890A:p.E964K,	7q31.32	.
7	121694092	G	A	GCCAAAACAT,GTAAGTCCCT	exonic	PTPRZ1	nonsynonymous SNV	PTPRZ1:ENST00000393386.2:exon26:c.G6381A:p.M2127I,PTPRZ1:ENST00000449182.1:exon25:c.G3780A:p.M1260I,	7q31.32	.
7	124386628	G	A	ACGGCGTATG,TACTGAAAGG	exonic	GPR37	nonsynonymous SNV	GPR37:ENST00000303921.2:exon2:c.C1793T:p.T598I,	7q31.33	.
7	128477300	G	T	CGACTGGCTT,GGGTGCCCCA	exonic	FLNC	nonsynonymous SNV	FLNC:ENST00000346177.6:exon3:c.G688T:p.G230W,FLNC:ENST00000325888.8:exon3:c.G688T:p.G230W,	7q32.1	.
7	130008390	G	A	AGGAGACGTG,ATGGCGCTTC	exonic	CPA5	stopgain	CPA5:ENST00000461828.1:exon12:c.G1263A:p.W421X,CPA5:ENST00000466363.2:exon13:c.G1263A:p.W421X,CPA5:ENST00000355388.3:exon14:c.G1263A:p.W421X,CPA5:ENST00000474905.1:exon13:c.G1263A:p.W421X,CPA5:ENST00000393213.3:exon11:c.G1263A:p.W421X,CPA5:ENST00000485477.1:ex	7q32.2	.
7	130417980	G	A	GTCGATGCGG,GAGTTCGACG	exonic	KLF14	nonsynonymous SNV	KLF14:ENST00000310992.4:exon1:c.C881T:p.P294L,	7q32.3	.
7	130417981	G	A	TCGATGCGGG,AGTTCGACGA	exonic	KLF14	nonsynonymous SNV	KLF14:ENST00000310992.4:exon1:c.C880T:p.P294S,	7q32.3	.
7	135304216	G	A	TGTGGTCGCC,GGGCAGTGTT	exonic	NUP205	nonsynonymous SNV	NUP205:ENST00000285968.6:exon29:c.G4009A:p.G1337R,	7q33	.
7	138223508	C	T	TGGGCAGTTT,CAGTGGCAGC	exonic	TRIM24	nonsynonymous SNV	TRIM24:ENST00000415680.2:exon7:c.C1103T:p.S368F,TRIM24:ENST00000343526.4:exon7:c.C1103T:p.S368F,	7q34	.
7	138391373	T	G	CTCAGCCTAC,CCTCGGCTGT	exonic	ATP6V0A4	nonsynonymous SNV	ATP6V0A4:ENST00000353492.4:exon21:c.A2519C:p.E840A,ATP6V0A4:ENST00000310018.2:exon22:c.A2519C:p.E840A,ATP6V0A4:ENST00000393054.1:exon21:c.A2519C:p.E840A,	7q34	.
7	138601805	G	A	CAGAGGGAAG,GGGTTGCTTC	exonic	KIAA1549	nonsynonymous SNV	KIAA1549:ENST00000440172.1:exon2:c.C2567T:p.P856L,KIAA1549:ENST00000242365.4:exon1:c.C2417T:p.P806L,KIAA1549:ENST00000422774.1:exon2:c.C2567T:p.P856L,	7q34	.
7	141781933	G	T	TGGCTTTGGG,AGACTGAGCA	exonic	MGAM	stopgain	MGAM:ENST00000475668.2:exon51:c.G6094T:p.E2032X,	7q34	.
7	141843653	G	A	TAAATTTCAT,ATCATCTGGA	exonic	RP11-1220K2	nonsynonymous SNV	RP11-1220K2.2:ENST00000550469.2:exon13:c.G1402A:p.D468N,RP11-1220K2.2:ENST00000477922.3:exon13:c.G1402A:p.D468N,	7q34	.
7	141919736	G	A	TACTTGGATT,ATGGTGGTCC	exonic	RP11-1220K2	nonsynonymous SNV	RP11-1220K2.2:ENST00000477922.3:exon48:c.G5425A:p.D1809N,	7q34	.
7	142045626	T	C	TAACGCTATG,ATTGGTACAA	exonic	TRBV4-2	nonsynonymous SNV	TRBV4-2:ENST00000390392.3:exon2:c.T154C:p.Y52H,	7q34	.
7	142119908	C	G	TCAGGCCTCT,TGCAGAGAAC	exonic	TRBV7-7	nonsynonymous SNV	TRBV7-7:ENST00000390377.1:exon2:c.G259C:p.E87Q,	7q34	.
7	143036350	G	A	CTCCAGTTCT,GATGTCCATC	exonic	CLCN1	stopgain	CLCN1:ENST00000343257.2:exon13:c.G1406A:p.W469X,	7q34	.
7	148984698	G	C	GCCCAGATCT,ATCTGCAGAG	exonic	ZNF783	stoploss	ZNF783:ENST00000476295.1:exon6:c.G845C:p.X282S,ZNF783:ENST00000378052.1:exon6:c.G845C:p.X282S,	7q36.1	.
7	150737648	C	T	CCCCTGCATC,CACTGTCTGG	exonic	ABCB8	nonsynonymous SNV	ABCB8:ENST00000358849.4:exon11:c.C1315T:p.P439S,ABCB8:ENST00000542328.1:exon10:c.C1051T:p.P351S,ABCB8:ENST00000498578.1:exon11:c.C1315T:p.P439S,ABCB8:ENST00000297504.6:exon12:c.C1366T:p.P456S,	7q36.1	.
7	150784102	C	T	CCTGATCGAG,GCATCGAGGA	exonic	AGAP3	nonsynonymous SNV	AGAP3:ENST00000397238.2:exon1:c.C274T:p.R92C,AGAP3:ENST00000479901.1:exon1:c.C274T:p.R92C,AGAP3:ENST00000473312.1:exon1:c.C274T:p.R92C,	7q36.1	.
7	150884091	G	A	ATGGGTCCCG,GCCAGACTTG	exonic	ASB10	nonsynonymous SNV	ASB10:ENST00000434669.1:exon1:c.C262T:p.P88S,ASB10:ENST00000275838.1:exon1:c.C127T:p.P43S,ASB10:ENST00000422024.1:exon1:c.C262T:p.P88S,ASB10:ENST00000420175.2:exon1:c.C127T:p.P43S,	7q36.1	.
7	154677424	C	T	CGGCTCTGCT,TCTCTCCAAT	exonic	DPP6	nonsynonymous SNV	DPP6:ENST00000404039.1:exon22:c.C2023T:p.L675F,DPP6:ENST00000377770.3:exon22:c.C2215T:p.L739F,DPP6:ENST00000332007.3:exon22:c.C2029T:p.L677F,DPP6:ENST00000427557.1:exon20:c.C1894T:p.L632F,	7q36.2	.
8	2876151	G	A	TGGGGGAAAG,AAAGGCTTCC	exonic	CSMD1	nonsynonymous SNV	CSMD1:ENST00000520002.1:exon53:c.C7880T:p.S2627F,CSMD1:ENST00000537824.1:exon52:c.C7877T:p.S2626F,CSMD1:ENST00000602557.1:exon53:c.C7880T:p.S2627F,	8p23.2	C
8	3000053	C	T	ATGAGGGTTT,ATGCGTTGTG	exonic	CSMD1	nonsynonymous SNV	CSMD1:ENST00000400186.3:exon42:c.G6178A:p.E2060K,CSMD1:ENST00000602723.1:exon42:c.G6178A:p.E2060K,CSMD1:ENST00000539096.1:exon41:c.G6175A:p.E2059K,CSMD1:ENST00000542608.1:exon41:c.G6175A:p.E2059K,CSMD1:ENST00000520002.1:exon42:c.G6178A:p.E2060K,CSMD1:ENST00	8p23.2	.
8	6299659	C	T	GAACATTTCA,GTGATACTTT	exonic	MCPH1	nonsynonymous SNV	MCPH1:ENST00000344683.5:exon7:c.C652T:p.R218C,MCPH1:ENST00000522905.1:exon6:c.C508T:p.R170C,MCPH1:ENST00000519480.1:exon7:c.C652T:p.R218C,	8p23.1	.
8	8176298	G	A	CCCTTCCGGG,AGGCGGGGCC	exonic	SGK223	nonsynonymous SNV	SGK223:ENST00000330777.4:exon5:c.C3587T:p.S1196F,SGK223:ENST00000520004.1:exon6:c.C3587T:p.S1196F,	8p23.1	.
8	8750447	G	A	CCCGGCCCCG,GGCAGGCCCC	exonic	MFHAS1	nonsynonymous SNV	MFHAS1:ENST00000276282.6:exon1:c.C122T:p.P41L,	8p23.1	.
8	10755763	G	A	TCTGGTGGGC,TAACCTGGGT	exonic	XKR6	nonsynonymous SNV	XKR6:ENST00000304437.2:exon3:c.C788T:p.T263M,XKR6:ENST00000416569.2:exon3:c.C1625T:p.T542M,	8p23.1	.
8	11412302	A	C	GGAGCTGATC,AGCACTATAA	exonic	BLK	nonsynonymous SNV	BLK:ENST00000259089.4:exon7:c.A523C:p.K175Q,BLK:ENST00000529894.1:exon6:c.A310C:p.K104Q,	8p23.1	.
8	16026295	C	T	GCTGTCATTT,CTTTTCCCGT	exonic	MSR1	nonsynonymous SNV	MSR1:ENST00000355282.2:exon3:c.G302A:p.G101E,MSR1:ENST00000381998.4:exon4:c.G302A:p.G101E,MSR1:ENST00000262101.5:exon4:c.G302A:p.G101E,MSR1:ENST00000445506.2:exon4:c.G356A:p.G119E,MSR1:ENST00000350896.3:exon4:c.G302A:p.G101E,	8p22	.
8	17401128	C	T	GGCCCGTGTT,CCAAGACGGG	exonic	SLC7A2	nonsynonymous SNV	SLC7A2:ENST00000522656.1:exon2:c.C280T:p.P94S,SLC7A2:ENST00000004531.10:exon2:c.C400T:p.P134S,SLC7A2:ENST00000398090.3:exon2:c.C400T:p.P134S,SLC7A2:ENST00000494857.1:exon3:c.C280T:p.P94S,SLC7A2:ENST00000470360.1:exon4:c.C400T:p.P134S,	8p22	.
8	19363329	C	T	AAGCAGCCCC,GGCGAACCAT	exonic	CSGALNACT	nonsynonymous SNV	CSGALNACT1:ENST00000517494.1:exon2:c.G17A:p.R6Q,CSGALNACT1:ENST00000332246.6:exon4:c.G17A:p.R6Q,CSGALNACT1:ENST00000519222.1:exon3:c.G17A:p.R6Q,CSGALNACT1:ENST00000544602.1:exon3:c.G17A:p.R6Q,CSGALNACT1:ENST00000520003.1:exon3:c.G17A:p.R6Q,CSGALNACT1:E	8p21.3	.
8	22005804	G	A	GCCAGCTCCT,GAACCGTACA	exonic	LGI3	stopgain	LGI3:ENST00000306317.2:exon8:c.C1516T:p.Q506X,LGI3:ENST00000424267.2:exon7:c.C1444T:p.Q482X,	8p21.3	.

8	23186039	C	T	ACGCGGCCCT,CCCGATGTAG	exonic	LOXL2	nonsynonymous SNV	LOXL2:ENST00000389131.3:exon6:c.G1006A:p.E336K,	8p21.3	.
8	26212141	C	G	TTTTTATTGT,TACCAATGGT	exonic	PPP2R2A	nonsynonymous SNV	PPP2R2A:ENST00000518397.1:exon4:c.C338G:p.S113C,PPP2R2A:ENST00000315985.7:exon4:c.C368G:p.S123C,PPP2R2A:ENST00000380737.3:exon4:c.C338G:p.S113C,	8p21.2	.
8	28207585	G	A	ACTCACCGGA,AGAGAGAGCA	exonic	ZNF395	nonsynonymous SNV	ZNF395:ENST00000344423.5:exon8:c.C1322T:p.S441F,ZNF395:ENST00000523202.1:exon8:c.C1322T:p.S441F,ZNF395:ENST00000523095.1:exon8:c.C1322T:p.S441F,	8p21.1	.
8	28929560	G	A	TCGGCCTCAG,GGCGGTGGGC	exonic	KIF13B	nonsynonymous SNV	KIF13B:ENST00000404075.3:exon3:c.C352T:p.P118S,KIF13B:ENST00000523130.1:exon4:c.C571T:p.P191S,KIF13B:ENST00000524189.1:exon39:c.C4795T:p.P1599S,	8p12	.
8	28976461	C	T	CGCATCCCAT,CACCAGCTTC	exonic	KIF13B	nonsynonymous SNV	KIF13B:ENST00000524189.1:exon30:c.G3584A:p.G1195E,	8p12	.
8	29043991	T	A	TTCCAGAGCC,AAAAAAATGG	splicing	KIF13B(ENST.		.	8p12	.
8	30704270	G	A	TTGTGTAGAG,AATAAAAATA	exonic	TEX15	nonsynonymous SNV	TEX15:ENST00000256246.2:exon1:c.C2264T:p.S755F,	8p12	.
8	37641818	G	A	GGAGGAAAAG,GAAGGTGCCC	exonic	GPR124	nonsynonymous SNV	GPR124:ENST00000428068.1:exon1:c.G110A:p.G37E,	8p11.23	.
8	38874919	A	G	TCCCAGCATG,CTCAGCTACT	exonic	ADAM9	nonsynonymous SNV	ADAM9:ENST00000481873.3:exon6:c.A592G:p.T198A,ADAM9:ENST00000379917.3:exon6:c.A592G:p.T198A,ADAM9:ENST00000487273.2:exon6:c.A592G:p.T198A,ADAM9:ENST00000468065.1:exon6:c.A592G:p.T198A,	8p11.22	.
8	41552698	C	T	CCCCTACCTT,GTCCATCCCG	exonic	ANK1	nonsynonymous SNV	ANK1:ENST00000379758.2:exon27:c.G3112A:p.E1038K,ANK1:ENST00000396942.1:exon27:c.G3112A:p.E1038K,ANK1:ENST00000352337.4:exon27:c.G3112A:p.E1038K,ANK1:ENST00000289734.7:exon27:c.G3112A:p.E1038K,ANK1:ENST00000347528.4:exon27:c.G3112A:p.E1038K,ANK1:ENST00000265709.	8p11.21	.
8	42294913	G	A	TTGCTTTCCT,GGCTGGCTTC	exonic	SLC20A2	stopgain	SLC20A2:ENST00000520179.1:exon8:c.C1117T:p.Q373X,SLC20A2:ENST00000342228.3:exon8:c.C1117T:p.Q373X,SLC20A2:ENST00000520262.1:exon8:c.C1117T:p.Q373X,	8p11.21	.
8	48887508	C	T	GTGGACATAT,TATTCTTACT	exonic	MCM4	nonsynonymous SNV	MCM4:ENST00000523944.1:exon15:c.C2351T:p.S784F,MCM4:ENST00000262105.2:exon14:c.C2351T:p.S784F,	8q11.21	.
8	49642384	C	T	ACATTTCCTC,TTTGAAATGA	exonic;splicing	EFCAB1;EFC	synonymous SNV	EFCAB1:ENST00000262103.3:exon4:c.G366A:p.K122K,EFCAB1:ENST00000523092.1:exon3:c.G210A:p.K70K,EFCAB1:ENST00000433756.1:exon3:c.G210A:p.K70K,	8q11.21	.
8	57078875	G	C	TGCTGACAGT,AGTGCAGAGA	exonic	PLAG1	stopgain	PLAG1:ENST00000429357.2:exon4:c.C1430G:p.S477X,PLAG1:ENST00000316981.3:exon5:c.C1430G:p.S477X,PLAG1:ENST00000423799.2:exon3:c.C1184G:p.S395X,	8q12.1	.
8	61693560	A	T	GTCTCTCAGC,TTCCCCGTCG	exonic	CHD7	nonsynonymous SNV	CHD7:ENST00000527900.1:exon2:c.A68T:p.H23L,CHD7:ENST00000525508.1:exon2:c.A1667T:p.H556L,CHD7:ENST00000524602.1:exon2:c.A1667T:p.H556L,CHD7:ENST00000423902.2:exon3:c.A1667T:p.H556L,	8q12.2	Cl
8	61757925	C	T	TGCCCTGTTC,AGGAGGACAG	exonic	CHD7	stopgain	CHD7:ENST00000423902.2:exon23:c.C5167T:p.Q1723X,	8q12.2	.
8	67592009	G	C	TGGAGAAGCA,AAAAGGCAAA	exonic	C8orf44	nonsynonymous SNV	C8orf44:ENST00000390159.3:exon4:c.G300C:p.Q100H,C8orf44:ENST00000519561.1:exon3:c.G300C:p.Q100H,	8q13.1	.
8	72184051	G	A	CCGTCCACGT,ATTTCCCATC	exonic	EYA1	nonsynonymous SNV	EYA1:ENST00000388740.3:exon8:c.C809T:p.S270L,EYA1:ENST00000388743.2:exon9:c.C905T:p.S302L,EYA1:ENST00000303824.7:exon10:c.C890T:p.S297L,EYA1:ENST00000340726.3:exon10:c.C908T:p.S303L,EYA1:ENST00000388741.2:exon9:c.C806T:p.S269L,EYA1:ENST00000419131.1:exon9:c.C893T:p.	8q13.3	.
8	73850048	G	A	AGAGCTCCCA,GGGCAAGGGA	exonic	KCNB2	nonsynonymous SNV	KCNB2:ENST00000523207.1:exon3:c.G2458A:p.G820R,	8q13.3	.
8	77617549	G	A	GTGAATCTGG,GGGGCTGTCT	exonic	ZFHX4	nonsynonymous SNV	ZFHX4:ENST00000521891.2:exon2:c.G1226A:p.G409E,ZFHX4:ENST00000518282.1:exon1:c.G1226A:p.G409E,ZFHX4:ENST00000050961.6:exon2:c.G1226A:p.G409E,ZFHX4:ENST00000455469.2:exon2:c.G1226A:p.G409E,	8q21.11	.
8	77766685	G	A	ACTCTGGCAG,AACACCAGCA	exonic	ZFHX4	nonsynonymous SNV	ZFHX4:ENST00000521891.2:exon10:c.G7528A:p.E2510K,ZFHX4:ENST00000518282.1:exon9:c.G7450A:p.E2484K,ZFHX4:ENST00000050961.6:exon10:c.G7393A:p.E2465K,ZFHX4:ENST00000455469.2:exon10:c.G7393A:p.E2465K,	8q21.11	.
8	81431632	C	A	TACAGAATTC,CTCGGGATGA	exonic	ZBTB10	nonsynonymous SNV	ZBTB10:ENST00000379091.4:exon6:c.C1609A:p.P537T,ZBTB10:ENST00000455036.3:exon6:c.C2485A:p.P829T,ZBTB10:ENST00000430430.1:exon7:c.C2485A:p.P829T,ZBTB10:ENST00000426744.2:exon7:c.C2413A:p.P805T,	8q21.13	.
8	87076604	G	A	GCAATGAGTC,ATCAAAGAGC	exonic	PSKH2	stopgain	PSKH2:ENST00000276616.2:exon2:c.C442T:p.R148X,	8q21.3	.
8	87165087	G	A	GTTCCACTAC,GTGTGTTTTA	exonic	ATP6V0D2	nonsynonymous SNV	ATP6V0D2:ENST00000285393.3:exon8:c.G934A:p.G312S,	8q21.3	.
8	87165088	G	A	TTCCACTACG,TGTGTTTTAT	exonic	ATP6V0D2	nonsynonymous SNV	ATP6V0D2:ENST00000285393.3:exon8:c.G935A:p.G312D,	8q21.3	.
8	92052877	G	A	CTTACCTCTG,GGCTGCTTTC	exonic	TMEM55A	nonsynonymous SNV	TMEM55A:ENST00000285419.3:exon1:c.C101T:p.P34L,TMEM55A:ENST00000518359.1:exon1:c.C101T:p.P34L,TMEM55A:ENST00000518869.1:exon1:c.C101T:p.P34L,TMEM55A:ENST00000520709.1:exon1:c.C101T:p.P34L,TMEM55A:ENST00000520014.1:exon1:c.C101T:p.P34L,	8q21.3	.
8	94935779	G	C	TGTTGATCAT,AGCGCCTCTC	exonic	PDP1	nonsynonymous SNV	PDP1:ENST00000396200.3:exon3:c.G1567C:p.E523Q,PDP1:ENST00000297598.4:exon2:c.G1492C:p.E498Q,PDP1:ENST00000517764.1:exon2:c.G1492C:p.E498Q,PDP1:ENST00000520728.1:exon3:c.G1492C:p.E498Q,	8q22.1	.
8	95777540	C	T	TAAGTGCCTT,AGGTAACTAG	exonic	DPY19L4	stopgain	DPY19L4:ENST00000414645.2:exon9:c.C1000T:p.Q334X,	8q22.1	.
8	95795990	C	T	ACAAGTTTGC,TCTTTACAAT	exonic	DPY19L4	nonsynonymous SNV	DPY19L4:ENST00000414645.2:exon17:c.C1808T:p.P603L,	8q22.1	.
8	103324027	G	A	GCTGCTTGTA,GAAAATTATT	exonic	UBR5	nonsynonymous SNV	UBR5:ENST00000220959.4:exon19:c.C2354T:p.P785L,UBR5:ENST00000521922.1:exon19:c.C2336T:p.P779L,UBR5:ENST00000520539.1:exon19:c.C2354T:p.P785L,	8q22.3	.
8	103324028	G	A	CTGCTTGTAG,AAAATTATTT	exonic	UBR5	nonsynonymous SNV	UBR5:ENST00000220959.4:exon19:c.C2353T:p.P785S,UBR5:ENST00000521922.1:exon19:c.C2335T:p.P779S,UBR5:ENST00000520539.1:exon19:c.C2353T:p.P785S,	8q22.3	.
8	104427594	G	A	GGGAAAAGCG,AAGTCGCCTG	exonic	DCAF13	nonsynonymous SNV	DCAF13:ENST00000297579.5:exon1:c.G376A:p.E126K,	8q22.3	.
8	104897636	C	T	CCTAGATCTC,ATCAGATTAT	exonic	RIMS2	nonsynonymous SNV	RIMS2:ENST00000515551.1:exon2:c.C233T:p.P78L,RIMS2:ENST00000504942.2:exon4:c.C809T:p.P270L,RIMS2:ENST00000436393.2:exon2:c.C143T:p.P48L,RIMS2:ENST00000406091.3:exon4:c.C809T:p.P270L,RIMS2:ENST00000408894.2:exon2:c.C233T:p.P78L,RIMS2:ENST00000507740.1:exon2:c.C233T:p.	8q22.3	.
8	110592088	G	A	GCTTGGGGAA,AAGGTGCATA	exonic	SYBU	nonsynonymous SNV	SYBU:ENST00000533065.1:exon4:c.C317T:p.S106F,SYBU:ENST00000408889.3:exon4:c.C317T:p.S106F,SYBU:ENST00000433638.1:exon7:c.C674T:p.S225F,SYBU:ENST00000532779.1:exon4:c.C470T:p.S157F,SYBU:ENST00000424158.2:exon7:c.C689T:p.S230F,SYBU:ENST00000446070.2:exon6:c.C671T:p.	8q23.2	.
8	119945482	G	A	TCGTCATAAT,AAGGTACTTT	exonic	TNFRSF11B	nonsynonymous SNV	TNFRSF11B:ENST00000297350.4:exon2:c.C88T:p.H30Y,TNFRSF11B:ENST00000517352.1:exon2:c.C88T:p.H30Y,	8q24.12	.
8	120118056	G	A	AGGGATTAGG,AAACTGAAGA	exonic	COLEC10	nonsynonymous SNV	COLEC10:ENST00000332843.2:exon6:c.G460A:p.E154K,	8q24.12	.
8	121644724	C	T	CTCTCGGCTC,CAGCAATGCC	exonic	SNTB1	nonsynonymous SNV	SNTB1:ENST00000517992.1:exon3:c.G956A:p.G319E,SNTB1:ENST00000395601.3:exon4:c.G956A:p.G319E,	8q24.12	.
8	132966129	C	T	CGATGAACTT,GGGCCACCAT	exonic	EFR3A	nonsynonymous SNV	EFR3A:ENST00000519656.1:exon6:c.C445T:p.R149W,EFR3A:ENST00000334503.4:exon6:c.C553T:p.R185W,EFR3A:ENST00000254624.5:exon6:c.C553T:p.R185W,	8q24.22	.
8	133945856	C	T	CACGACGGAG,CAGAGATTTC	exonic	TG	nonsynonymous SNV	TG:ENST00000542445.1:exon4:c.C169T:p.P57S,TG:ENST00000377869.1:exon23:c.C4696T:p.P1566S,TG:ENST00000220616.4:exon24:c.C4867T:p.P1623S,	8q24.22	.
8	139603742	C	T	GCTCCTCGCT,ACCTGGGAAG	exonic	COL22A1	nonsynonymous SNV	COL22A1:ENST00000303045.6:exon64:c.G4618A:p.E1540K,COL22A1:ENST00000435777.1:exon63:c.G4558A:p.E1520K,	8q24.23	.
8	139609161	C	T	AAGCTGCTTC,CCAGCTCTTC	exonic	COL22A1	nonsynonymous SNV	COL22A1:ENST00000303045.6:exon62:c.G4418A:p.G1473E,COL22A1:ENST00000435777.1:exon61:c.G4358A:p.G1453E,	8q24.23	.
8	142228708	G	A	GGTGCTGCGG,GGGTGGCGGG	exonic	SLC45A4	nonsynonymous SNV	SLC45A4:ENST00000517878.1:exon4:c.C1031T:p.P344L,SLC45A4:ENST00000433583.2:exon4:c.C857T:p.P286L,SLC45A4:ENST00000519067.1:exon4:c.C878T:p.P293L,SLC45A4:ENST00000024061.3:exon4:c.C878T:p.P293L,	8q24.3	.
8	142228709	G	A	GTGCTGCGGG,GGTGGCGGGG	exonic	SLC45A4	nonsynonymous SNV	SLC45A4:ENST00000517878.1:exon4:c.C1030T:p.P344S,SLC45A4:ENST00000433583.2:exon4:c.C856T:p.P286S,SLC45A4:ENST00000519067.1:exon4:c.C877T:p.P293S,SLC45A4:ENST00000024061.3:exon4:c.C877T:p.P293S,	8q24.3	.
8	143425335	G	A	ACCTCTGGGC,GCTCCAGACT	exonic	TSNARE1	nonsynonymous SNV	TSNARE1:ENST00000307180.3:exon4:c.C737T:p.P246L,TSNARE1:ENST00000520166.1:exon3:c.C737T:p.P246L,TSNARE1:ENST00000524325.1:exon4:c.C737T:p.P246L,	8q24.3	.
8	143425336	G	A	CCTCTGGGCG,CTCCAGACTG	exonic	TSNARE1	nonsynonymous SNV	TSNARE1:ENST00000307180.3:exon4:c.C736T:p.P246S,TSNARE1:ENST00000520166.1:exon3:c.C736T:p.P246S,TSNARE1:ENST00000524325.1:exon4:c.C736T:p.P246S,	8q24.3	.
8	143602238	C	G	TGTCCATCAT,TCCTCCAATG	exonic	BAI1	nonsynonymous SNV	BAI1:ENST00000323289.5:exon19:c.C2976G:p.I992M,BAI1:ENST00000521208.1:exon20:c.C2976G:p.I992M,BAI1:ENST00000517894.1:exon20:c.C2976G:p.I992M,	8q24.3	.
8	144942464	G	A	ATAGGGGTCG,TGTAGCCGGT	exonic	EPPK1	nonsynonymous SNV	EPPK1:ENST00000525985.1:exon2:c.C4958T:p.T1653I,	8q24.3	.
8	144990623	C	T	GTGCCCTCCT,CACCATGCTG	exonic	PLEC	nonsynonymous SNV	PLEC:ENST00000357649.2:exon32:c.G13378A:p.E4460K,PLEC:ENST00000356346.3:exon32:c.G13324A:p.E4442K,PLEC:ENST00000398774.2:exon32:c.G13270A:p.E4424K,PLEC:ENST00000322810.4:exon32:c.G13777A:p.E4593K,PLEC:ENST00000436759.2:exon33:c.G13447A:p.E4483K,PLEC:ENST0000052	8q24.3	.
8	145533212	C	T	CACGGAGTTC,AGCACCCATG	exonic	HSF1	stopgain	HSF1:ENST00000533240.1:exon3:c.C103T:p.Q35X,HSF1:ENST00000528838.1:exon3:c.C298T:p.Q100X,HSF1:ENST00000400780.4:exon2:c.C103T:p.Q35X,	8q24.3	.
8	145725507	G	A	GGTCAACACC,ACGGGAACAT	exonic	PPP1R16A	nonsynonymous SNV	PPP1R16A:ENST00000435887.1:exon4:c.G505A:p.D169N,PPP1R16A:ENST00000292539.4:exon5:c.G505A:p.D169N,	8q24.3	.
8	146032579	C	T	CTCTCAGATT,CAGGATGGAG	exonic	ZNF517	nonsynonymous SNV	ZNF517:ENST00000359971.3:exon5:c.C278T:p.S93F,ZNF517:ENST00000528012.1:exon5:c.C257T:p.S86F,ZNF517:ENST00000533965.1:exon5:c.C278T:p.S93F,ZNF517:ENST00000531720.1:exon4:c.C278T:p.S93F,	8q24.3	.
9	2639882	G	C	TGCTGAATCT,ACTTCGTGTG	exonic	VLDLR	nonsynonymous SNV	VLDLR:ENST00000382100.3:exon3:c.G226C:p.D76H,VLDLR:ENST00000382096.1:exon3:c.G226C:p.D76H,VLDLR:ENST00000382099.2:exon3:c.G226C:p.D76H,	9p24.2	.
9	3937120	C	T	TACGGCTTCT,GCCTGTGTGG	exonic	GLIS3	nonsynonymous SNV	GLIS3:ENST00000381971.3:exon5:c.G1780A:p.E594K,GLIS3:ENST00000324333.10:exon4:c.G1315A:p.E439K,	9p24.2	.
9	8317917	G	A	CTCTAGTGCG,CACGATAGGA	exonic	PTPRD	nonsynonymous SNV	PTPRD:ENST00000356435.5:exon35:c.C5696T:p.A1899V,PTPRD:ENST00000540109.1:exon35:c.C5696T:p.A1899V,PTPRD:ENST00000397611.3:exon30:c.C4466T:p.A1489V,PTPRD:ENST00000397617.3:exon29:c.C4475T:p.A1492V,PTPRD:ENST00000397606.3:exon29:c.C4475T:p.A1492V,PTPRD:ENST0000	9p24.1	.
9	8389280	T	G	TGGCACTCCG,TGTTCCCATA	exonic	PTPRD	nonsynonymous SNV	PTPRD:ENST00000356435.5:exon26:c.A4338C:p.Q1446H,PTPRD:ENST00000540109.1:exon26:c.A4338C:p.Q1446H,PTPRD:ENST00000397611.3:exon21:c.A3108C:p.Q1036H,PTPRD:ENST00000397617.3:exon20:c.A3117C:p.Q1039H,PTPRD:ENST00000397606.3:exon20:c.A3117C:p.Q1039H,PTPRD:ENST0000	9p24.1	.
9	18662059	C	T	AACTTGGATC,TTGTCCAGCC	exonic	ADAMTSL1	nonsynonymous SNV	ADAMTSL1:ENST00000276935.6:exon9:c.C1073T:p.P358L,ADAMTSL1:ENST00000380548.4:exon9:c.C1073T:p.P358L,ADAMTSL1:ENST00000327883.7:exon9:c.C1073T:p.P358L,ADAMTSL1:ENST00000380566.4:exon9:c.C1073T:p.P358L,	9p22.1	.
9	21304868	C	T	GGAGTGTCTT,CACTCCAACC	exonic	IFNA5	nonsynonymous SNV	IFNA5:ENST00000259555.4:exon1:c.G388A:p.E130K,	9p21.3	.
9	27192565	G	A	GTCCGTCGTG,AGAGGGTGGG	exonic	TEK	nonsynonymous SNV	TEK:ENST00000519080.1:exon8:c.G998A:p.G333E,TEK:ENST00000519097.1:exon9:c.G1127A:p.G376E,TEK:ENST00000406359.4:exon10:c.G1439A:p.G480E,TEK:ENST00000380036.4:exon11:c.G1568A:p.G523E,	9p21.2	.
9	32631392	G	A	TCTGTGCGGC,TCGGTGGATG	exonic	TAF1L	nonsynonymous SNV	TAF1L:ENST00000242310.4:exon1:c.C4186T:p.R1396C,	9p21.1	T/
9	32635090	C	T	CTTCATTGGT,CCGGGGGTGG	exonic	TAF1L	nonsynonymous SNV	TAF1L:ENST00000242310.4:exon1:c.G488A:p.G163E,	9p21.1	.
9	33386489	C	G	CTCCAGGGCA,GCGGCCCAGC	exonic	AQP7	nonsynonymous SNV	AQP7:ENST00000541274.1:exon4:c.G43C:p.V15L,AQP7:ENST00000539936.1:exon5:c.G319C:p.V107L,AQP7:ENST00000379507.3:exon3:c.G316C:p.V106L,AQP7:ENST00000379503.4:exon2:c.G127C:p.V43L,AQP7:ENST00000377425.4:exon4:c.G148C:p.V50L,AQP7:ENST00000537089.1:exon4:c.G43C:p.V15L,	9p13.3	.
9	35376104	G	A	TTTGAGGTCT,GACGGCCACT	exonic	UNC13B	stopgain	UNC13B:ENST00000378495.3:exon14:c.G1448A:p.W483X,UNC13B:ENST00000378496.4:exon14:c.G1448A:p.W483X,UNC13B:ENST00000396787.1:exon15:c.G1484A:p.W495X,	9p13.3	.
9	35401992	C	T	TGAGGACATT,GTCCGGAAAA	exonic	UNC13B	nonsynonymous SNV	UNC13B:ENST00000378496.4:exon37:c.C4282T:p.R1428C,UNC13B:ENST00000396787.1:exon38:c.C4318T:p.R1440C,	9p13.3	.
9	35751283	C	T	TGTCCGGTTT,CCCAGGATGA	exonic	RGP1	nonsynonymous SNV	RGP1:ENST00000378078.4:exon6:c.C508T:p.P170S,RGP1:ENST00000456972.2:exon7:c.C628T:p.P210S,	9p13.3	.
9	71687546	C	G	AGCGTTATGA,TGGACTGGGA	exonic	FXN	nonsynonymous SNV	FXN:ENST00000498653.1:exon5:c.C276G:p.D92E,FXN:ENST00000377270.3:exon5:c.C501G:p.D167E,FXN:ENST00000396366.2:exon5:c.C509G:p.T170S,	9q21.11	.
9	72000768	C	G	ACTCCCAGTT,AACCCTGGTG	exonic	FAM189A2	stopgain	FAM189A2:ENST00000257515.8:exon9:c.C761G:p.S254X,FAM189A2:ENST00000303068.7:exon7:c.C266G:p.S89X,FAM189A2:ENST00000455972.1:exon9:c.C761G:p.S254X,	9q21.11	.
9	72131759	C	T	GGCCTCGGGC,GGTACTGCAC	exonic	APBA1	nonsynonymous SNV	APBA1:ENST00000265381.4:exon2:c.G368A:p.R123Q,	9q21.11	Al
9	72471547	C	T	AAGAGCAAGG,TTTATTGAAT	exonic	C9orf135	nonsynonymous SNV	C9orf135:ENST00000527647.1:exon3:c.C338T:p.A113V,C9orf135:ENST00000377197.3:exon3:c.C338T:p.A113V,	9q21.12	.
9	79323649	C	T	TGATTTGCTT,CTGATACTCC	exonic	PRUNE2	nonsynonymous SNV	PRUNE2:ENST00000428286.1:exon8:c.G2464A:p.E822K,PRUNE2:ENST00000376718.3:exon8:c.G3541A:p.E1181K,	9q21.2	.
9	79834907	G	A	AACTTGTGAT,AATCGCCGAT	exonic	VPS13A	nonsynonymous SNV	VPS13A:ENST00000357409.5:exon11:c.G792A:p.M264I,VPS13A:ENST00000360280.3:exon11:c.G792A:p.M264I,VPS13A:ENST00000376636.3:exon11:c.G792A:p.M264I,VPS13A:ENST00000376634.4:exon11:c.G792A:p.M264I,	9q21.2	.
9	79959943	G	C	TATTATAGCA,AAGTGAATTT	exonic	VPS13A	nonsynonymous SNV	VPS13A:ENST00000357409.5:exon52:c.G7174C:p.E2392Q,VPS13A:ENST00000360280.3:exon52:c.G7174C:p.E2392Q,VPS13A:ENST00000376636.3:exon51:c.G7057C:p.E2353Q,VPS13A:ENST00000376634.4:exon52:c.G7174C:p.E2392Q,	9q21.2	.
9	86571186	G	A	CGACCAAAAG,AGAAGTTGAG	exonic	C9orf64	nonsynonymous SNV	C9orf64:ENST00000376344.3:exon1:c.C230T:p.S77F,	9q21.32	.
9	86585186	C	T	ATCGAAGCTC,CGACTCATGA	exonic	HNRNPK	nonsynonymous SNV	HNRNPK:ENST00000376263.3:exon16:c.G1252A:p.G418R,HNRNPK:ENST00000376264.2:exon16:c.G1252A:p.G418R,HNRNPK:ENST00000360384.5:exon16:c.G1252A:p.G418R,HNRNPK:ENST00000351839.3:exon15:c.G1252A:p.G418R,HNRNPK:ENST00000376281.4:exon16:c.G1252A:p.G418R,	9q21.32	.
9	91991883	G	A	GGGGTCCAGG,AGAGGAAAGC	exonic	SEMA4D	nonsynonymous SNV	SEMA4D:ENST00000455551.2:exon17:c.C1747T:p.P583S,SEMA4D:ENST00000339861.4:exon16:c.C1747T:p.P583S,SEMA4D:ENST00000343780.4:exon18:c.C1747T:p.P583S,SEMA4D:ENST00000420987.1:exon17:c.C1747T:p.P583S,	9q22.2	.
9	95086377	C	G	TTTTGTAGGT,TGCCTCAGAA	exonic	NOL8	nonsynonymous SNV	NOL8:ENST00000432670.2:exon2:c.G67C:p.D23H,NOL8:ENST00000535387.1:exon1:c.G67C:p.D23H,NOL8:ENST00000536624.1:exon2:c.G67C:p.D23H,NOL8:ENST00000544867.1:exon2:c.G67C:p.D23H,NOL8:ENST00000542573.1:exon2:c.G67C:p.D23H,NOL8:ENST00000360868.3:exon1:c.G67C:p.D23H,NOL	9q22.31	.
9	95272236	T	A	CTTTAAGTTC,TCTAATGTAG	exonic	ECM2	nonsynonymous SNV	ECM2:ENST00000444490.2:exon6:c.A1185T:p.E395D,ECM2:ENST00000344604.5:exon6:c.A1251T:p.E417D,	9q22.31	.
9	101156457	C	T	CCTGCCTTAC,TTGGAACCTG	exonic	GABBR2	nonsynonymous SNV	GABBR2:ENST00000259455.2:exon9:c.G1378A:p.G460R,	9q22.33	.
9	104130596	G	A	CCTGGGAAGA,ACCCTCTCCT	exonic	BAAT	nonsynonymous SNV	BAAT:ENST00000395051.3:exon2:c.C475T:p.L159F,BAAT:ENST00000259407.2:exon3:c.C475T:p.L159F,	9q31.1	.
9	104433364	C	A	CCACTGAGGC,TCTGAAAGTG	exonic	GRIN3A	nonsynonymous SNV	GRIN3A:ENST00000361820.3:exon3:c.G1330T:p.G444C,	9q31.1	.
9	107267084	A	T	CACTTGTGAA,TTCTGGCCAT	exonic	OR13F1	nonsynonymous SNV	OR13F1:ENST00000334726.2:exon1:c.A541T:p.I181F,	9q31.1	.
9	111710491	G	A	TAAATCCTGG,AAGTTTTCAG	exonic	CTNNAL1	nonsynonymous SNV	CTNNAL1:ENST00000325551.4:exon15:c.C1859T:p.S620F,CTNNAL1:ENST00000374594.1:exon4:c.C62T:p.S21F,CTNNAL1:ENST00000325580.6:exon13:c.C1607T:p.S536F,CTNNAL1:ENST00000374595.4:exon15:c.C1859T:p.S620F,	9q31.3	.
9	112918626	C	T	AAACCTCCTC,ATCCCCCACC	exonic	AKAP2;PALM	nonsynonymous SNV	AKAP2:ENST00000510514.5:exon9:c.C3023T:p.P1008L,AKAP2:ENST00000259318.7:exon3:c.C2330T:p.P777L,AKAP2:ENST00000374525.1:exon3:c.C2597T:p.P866L,PALM2-AKAP2:ENST00000302798.7:exon9:c.C3023T:p.P1008L,AKAP2:ENST00000434623.2:exon3:c.C2597T:p.P866L,AKAP2:ENST00000552	9q31.3	.
9	115421846	G	A	TCTGGGGGAT,ACAGCCCTAT	exonic	KIAA1958	nonsynonymous SNV	KIAA1958:ENST00000337530.6:exon4:c.G1648A:p.D550N,KIAA1958:ENST00000536272.1:exon5:c.G1732A:p.D578N,	9q32	.
9	115933930	G	C	CTAGTTCCTT,ATGTGCTGTT	exonic	FKBP15	nonsynonymous SNV	FKBP15:ENST00000446284.1:exon24:c.C2664G:p.I888M,FKBP15:ENST00000238256.3:exon24:c.C2589G:p.I863M,	9q32	.
9	116135944	G	A	AGAGAGGGGA,GATGTGTTCT	exonic	HDHD3	nonsynonymous SNV	HDHD3:ENST00000374180.3:exon3:c.C691T:p.L231F,HDHD3:ENST00000238379.5:exon2:c.C691T:p.L231F,	9q32	.
9	116356248	G	A	GCTTCCTGCC,GGGCGGCCCC	exonic	RGS3	nonsynonymous SNV	RGS3:ENST00000462403.1:exon1:c.G49A:p.G17R,	9q32	.
9	116356249	G	A	CTTCCTGCCG,GGCGGCCCCA	exonic	RGS3	nonsynonymous SNV	RGS3:ENST00000462403.1:exon1:c.G50A:p.G17E,	9q32	.
9	123201999	G	C	GAGTGCTTTT,AAGCTGAAAT	exonic	CDK5RAP2	nonsynonymous SNV	CDK5RAP2:ENST00000360822.3:exon23:c.C3304G:p.Q1102E,CDK5RAP2:ENST00000425647.1:exon2:c.C430G:p.Q144E,CDK5RAP2:ENST00000359309.3:exon23:c.C3277G:p.Q1093E,CDK5RAP2:ENST00000349780.4:exon24:c.C3400G:p.Q1134E,CDK5RAP2:ENST00000416449.1:exon9:c.C1582G:p.Q528E,Cl	9q33.2	.
9	123725026	C	T	TATCCGGAAT,GTACACAAAG	exonic	C5	nonsynonymous SNV	C5:ENST00000223642.1:exon36:c.G4427A:p.R1476Q,	9q33.2	.
9	123808489	G	A	GCAGAGTTTT,GAATTTATTC	exonic	C5	stopgain	C5:ENST00000223642.1:exon2:c.C238T:p.Q80X,	9q33.2	.
9	124921909	A	C	GCCTCACCTC,TCTACTTTCA	exonic	NDUFA8	nonsynonymous SNV	NDUFA8:ENST00000537618.1:exon1:c.T48G:p.D16E,NDUFA8:ENST00000373768.3:exon1:c.T48G:p.D16E,	9q33.2	.
9	125424478	C	G	GCACATTCTT,TGACTAATCA	exonic	OR1L1	nonsynonymous SNV	OR1L1:ENST00000373686.1:exon1:c.C634G:p.L212V,OR1L1:ENST00000309623.1:exon1:c.C484G:p.L162V,	9q33.2	.
9	130269465	G	A	GCCTCAAAGG,CAGCCCCACC	exonic	FAM129B	nonsynonymous SNV	FAM129B:ENST00000373314.3:exon14:c.C1861T:p.P621S,FAM129B:ENST00000373312.3:exon14:c.C1900T:p.P634S,	9q33.3	.
9	130885360	G	A	CACATTGGGG,AGATCAGGTG	exonic	PTGES2	nonsynonymous SNV	PTGES2:ENST00000449878.1:exon5:c.C635T:p.S212F,PTGES2:ENST00000277462.5:exon6:c.C167T:p.S56F,PTGES2:ENST00000338961.6:exon5:c.C740T:p.S247F,	9q34.11	.
9	130948047	G	A	CATAGCCTCT,GAGGTTACCT	exonic	CIZ1	stopgain	CIZ1:ENST00000538431.1:exon5:c.C367T:p.R123X,CIZ1:ENST00000357558.5:exon5:c.C367T:p.R123X,CIZ1:ENST00000372954.1:exon4:c.C295T:p.R99X,CIZ1:ENST00000372948.3:exon5:c.C367T:p.R123X,CIZ1:ENST00000420484.1:exon5:c.C367T:p.R123X,CIZ1:ENST00000415526.1:exon3:c.C148T:p.R50X,C	9q34.11	.
9	131755859	C	T	CTGATTGATT,CCAACAGCAA	exonic	NUP188	nonsynonymous SNV	NUP188:ENST00000372577.2:exon27:c.C2903T:p.S968F,	9q34.11	.
9	133962963	T	G	TCCCAGCAGG,GCTGGCGTCT	exonic	LAMC3	nonsynonymous SNV	LAMC3:ENST00000361069.4:exon26:c.T4331G:p.V1444G,	9q34.12	.
9	134074318	G	A	TCCTGGCTTT,GACAGGGAGG	exonic	NUP214	nonsynonymous SNV	NUP214:ENST00000359428.5:exon29:c.G5437A:p.G1813R,NUP214:ENST00000411637.2:exon29:c.G5407A:p.G1803R,NUP214:ENST00000451030.1:exon29:c.G5440A:p.G1814R,NUP214:ENST00000483497.2:exon4:c.G1915A:p.G639R,	9q34.13	.
9	135538016	C	T	AATGAAGTTT,GTTCCTCCGT	exonic	DDX31	nonsynonymous SNV	DDX31:ENST00000544003.1:exon3:c.G169A:p.E57K,DDX31:ENST00000438527.3:exon2:c.G70A:p.E24K,DDX31:ENST00000310532.2:exon2:c.G457A:p.E153K,DDX31:ENST00000372159.3:exon2:c.G457A:p.E153K,DDX31:ENST00000372153.1:exon2:c.G457A:p.E153K,	9q34.13	.
9	135545422	C	T	GCCACCGCCC,TTCCGGGTCC	exonic	DDX31	nonsynonymous SNV	DDX31:ENST00000310532.2:exon1:c.G215A:p.R72K,DDX31:ENST00000372159.3:exon1:c.G215A:p.R72K,DDX31:ENST00000372153.1:exon1:c.G215A:p.R72K,	9q34.13	.
9	136234263	G	A	CTCCAGGAAG,TGCTGATCAG	exonic	SURF4	nonsynonymous SNV	SURF4:ENST00000371997.3:exon2:c.C107T:p.T36I,SURF4:ENST00000371989.3:exon2:c.C107T:p.T36I,SURF4:ENST00000485435.2:exon2:c.C107T:p.T36I,SURF4:ENST00000545297.1:exon2:c.C107T:p.T36I,	9q34.2	.
9	136308529	C	T	CCATGCAGCG,CTCTGTGGG	exonic	ADAMTS13	nonsynonymous SNV	ADAMTS13:ENST00000355699.2:exon19:c.C2267T:p.A756V,ADAMTS13:ENST00000356589.2:exon19:c.C2174T:p.A725V,ADAMTS13:ENST00000371929.3:exon19:c.C2267T:p.A756V,	9q34.2	.
9	137681010	G	A	GGGCTCTATT,GATTCCCTGG	exonic	COL5A1	nonsynonymous SNV	COL5A1:ENST00000371817.3:exon32:c.G2656A:p.G886R,	9q34.3	.
9	139273650	G	A	AGGGACGCCT,GGGTAGGGTG	exonic	SNAPC4	stopgain	SNAPC4:ENST00000298532.2:exon21:c.C2629T:p.Q877X,	9q34.3	.
9	139275797	G	A	GTAAACACAG,GGTGCTGGCC	exonic	SNAPC4	nonsynonymous SNV	SNAPC4:ENST00000298532.2:exon18:c.C2365T:p.P789S,	9q34.3	.
9	139835459	G	A	CATGGTGCGT,GGGAGCGCCA	exonic	FBXW5	nonsynonymous SNV	FBXW5:ENST00000325285.3:exon9:c.C1622T:p.P541L,	9q34.3	.
9	139902873	C	T	CTCACCCGCT,CTCCTCGAAG	exonic	ABCA2	nonsynonymous SNV	ABCA2:ENST00000448336.1:exon4:c.G517A:p.E173K,ABCA2:ENST00000490486.1:exon5:c.G547A:p.E183K,ABCA2:ENST00000265662.5:exon48:c.G7270A:p.E2424K,ABCA2:ENST00000371605.3:exon47:c.G7267A:p.E2423K,ABCA2:ENST00000341511.6:exon48:c.G7270A:p.E2424K,	9q34.3	.
9	140245832	C	T	GGCCTGGGCT,CCTGCCCTGT	exonic	EXD3	nonsynonymous SNV	EXD3:ENST00000340951.4:exon13:c.G1322A:p.G441E,EXD3:ENST00000342129.4:exon13:c.G362A:p.G121E,	9q34.3	.
9	140358635	G	A	GCGAAATAAG,AATCCACAGG	exonic	PNPLA7	nonsynonymous SNV	PNPLA7:ENST00000406427.1:exon28:c.C3169T:p.P1057S,PNPLA7:ENST00000371457.1:exon17:c.C1912T:p.P638S,PNPLA7:ENST00000277531.4:exon27:c.C3094T:p.P1032S,PNPLA7:ENST00000434090.1:exon27:c.C3067T:p.P1023S,	9q34.3	.
10	93697	G	A	TAGGGTCTTG,AACATATGTC	exonic	TUBB8	nonsynonymous SNV	TUBB8:ENST00000309812.4:exon4:c.C635T:p.S212F,TUBB8:ENST00000447903.2:exon4:c.C419T:p.S140F,	10p15.3	.
10	5248256	G	A	GAAGTGTAAG,ATGCAGGATT	exonic	AKR1C4	nonsynonymous SNV	AKR1C4:ENST00000263126.1:exon5:c.G466A:p.D156N,AKR1C4:ENST00000380448.1:exon7:c.G466A:p.D156N,	10p15.1	.
10	6470206	G	A	GTTCATGAAG,AAAAGTTCCT	exonic	PRKCQ	nonsynonymous SNV	PRKCQ:ENST00000263125.5:exon18:c.C2084T:p.S695F,PRKCQ:ENST00000397176.2:exon17:c.C1895T:p.S632F,PRKCQ:ENST00000539722.1:exon17:c.C1709T:p.S570F,	10p15.1	.
10	11330437	G	A	TGCAGCTGCG,CCCAGACCTC	exonic	CELF2	nonsynonymous SNV	CELF2:ENST00000354897.3:exon8:c.G805A:p.A269T,CELF2:ENST00000609692.1:exon8:c.G805A:p.A269T,CELF2:ENST00000537122.1:exon7:c.G544A:p.A182T,CELF2:ENST00000399850.3:exon9:c.G805A:p.A269T,CELF2:ENST00000416382.2:exon9:c.G877A:p.A293T,CELF2:ENST00000315874.4:exon9:c.G8	10p14	.
10	14572386	T	C	CGGGAGGTTT,TACAGGATTG	exonic	FAM107B	nonsynonymous SNV	FAM107B:ENST00000488576.1:exon4:c.A73G:p.K25E,FAM107B:ENST00000181796.2:exon3:c.A598G:p.K200E,FAM107B:ENST00000494865.1:exon3:c.A73G:p.K25E,FAM107B:ENST00000468747.1:exon2:c.A73G:p.K25E,FAM107B:ENST00000475786.1:exon3:c.A73G:p.K25E,FAM107B:ENST00000378470.1:ex	10p13	.
10	19498548	G	A	CAGATATTTG,GAATCAAGGC	exonic	C10orf112	nonsynonymous SNV	C10orf112:ENST00000377266.3:exon4:c.G857A:p.G286E,C10orf112:ENST00000454679.2:exon3:c.G818A:p.G273E,	10p12.31	.
10	19856483	G	A	TCAGTGATTG,AAATAAAAGA	exonic	C10orf112	nonsynonymous SNV	C10orf112:ENST00000377266.3:exon20:c.G3668A:p.G1223E,C10orf112:ENST00000454679.2:exon19:c.G3629A:p.G1210E,C10orf112:ENST00000455457.2:exon5:c.G113A:p.G38E,	10p12.31	.
10	28491168	G	A	GGCTGCAGCT,GGCTGGCAGA	exonic	MPP7	stopgain	MPP7:ENST00000441595.2:exon4:c.C70T:p.Q24X,MPP7:ENST00000375732.1:exon3:c.C70T:p.Q24X,MPP7:ENST00000540098.1:exon2:c.C70T:p.Q24X,MPP7:ENST00000337532.5:exon4:c.C70T:p.Q24X,MPP7:ENST00000496637.2:exon2:c.C70T:p.Q24X,MPP7:ENST00000375719.3:exon5:c.C70T:p.Q24X,	10p12.1	.
10	29818739	G	A	GCGTCGCTTT,GAACATTTTG	exonic	SVIL	nonsynonymous SNV	SVIL:ENST00000355867.4:exon12:c.C2141T:p.P714L,SVIL:ENST00000375398.2:exon14:c.C2141T:p.P714L,SVIL:ENST00000375400.3:exon11:c.C959T:p.P320L,	10p11.23	.
10	29818773	C	T	TTTTCATCT,CTAAGATTAG	exonic	SVIL	nonsynonymous SNV	SVIL:ENST00000355867.4:exon12:c.G2107A:p.E703K,SVIL:ENST00000375398.2:exon14:c.G2107A:p.E703K,SVIL:ENST00000375400.3:exon11:c.G925A:p.E309K,	10p11.23	.
10	29818774	C	T	TTTCCATCTC,TAAGATTAGA	splicing	SVIL(ENST00.		.	10p11.23	.
10	30653799	G	A	CACCTGAAGG,AGGGGTGCTC	exonic	MTPAP	nonsynonymous SNV	MTPAP:ENST00000358107.4:exon2:c.C383T:p.S128F,	10p11.23	.
10	32327750	G	A	TTCAAAATAT,AAACCTAAAG	exonic	KIF5B	nonsynonymous SNV	KIF5B:ENST00000302418.4:exon5:c.C398T:p.S133L,	10p11.22	.
10	38242677	C	G	ATGAATGCTC,ATAGGTCTTC	exonic	ZNF25	nonsynonymous SNV	ZNF25:ENST00000302609.7:exon5:c.G248C:p.W83S,	10p11.1	.
10	48438697	G	A	GGTCCGAGCG,GGACATGAGC	exonic	GDF10	nonsynonymous SNV	GDF10:ENST00000224605.2:exon1:c.C14T:p.P5L,	10q11.22	.
10	49634040	T	G	GACCTAAATA,GCTGGATATA	exonic	MAPK8	stopgain	MAPK8:ENST00000360332.3:exon7:c.T798G:p.Y266X,MAPK8:ENST00000374189.1:exon8:c.T798G:p.Y266X,MAPK8:ENST00000374179.3:exon7:c.T798G:p.Y266X,MAPK8:ENST00000374176.4:exon7:c.T798G:p.Y266X,MAPK8:ENST00000374182.3:exon7:c.T798G:p.Y266X,	10q11.22	.
10	49937557	G	A	ACAACCTGTG,GAGGTCAGAG	exonic	WDFY4	nonsynonymous SNV	WDFY4:ENST00000413659.2:exon7:c.G1052A:p.G351E,WDFY4:ENST00000325239.5:exon7:c.G1052A:p.G351E,WDFY4:ENST00000360890.2:exon8:c.G1052A:p.G351E,	10q11.23	.
10	50028463	C	T	CAGTGGCTGC,CCTGGAGGTG	exonic	WDFY4	nonsynonymous SNV	WDFY4:ENST00000325239.5:exon32:c.C5690T:p.P1897L,	10q11.23	.
10	55945018	G	A	GTGAAGCTCT,GGTCTTTTGT	exonic	PCDH15	nonsynonymous SNV	PCDH15:ENST00000414778.1:exon14:c.C1331T:p.P444L,PCDH15:ENST00000373965.2:exon13:c.C1337T:p.P446L,PCDH15:ENST00000395438.1:exon12:c.C1316T:p.P439L,PCDH15:ENST00000409834.1:exon5:c.C149T:p.P50L,PCDH15:ENST00000320301.6:exon12:c.C1316T:p.P439L,PCDH15:ENST0000039	10q21.1	.
10	61846521	G	A	CGGGGGCACC,GAATGGTCAT	exonic	ANK3	nonsynonymous SNV	ANK3:ENST00000355288.2:exon8:c.C1064T:p.P355L,ANK3:ENST00000503366.1:exon31:c.C3665T:p.P1222L,ANK3:ENST00000280772.2:exon30:c.C3662T:p.P1221L,ANK3:ENST00000373827.2:exon31:c.C3644T:p.P1215L,	10q21.2	.
10	61846522	G	A	GGGGGCACCG,AATGGTCATT	exonic	ANK3	nonsynonymous SNV	ANK3:ENST00000355288.2:exon8:c.C1063T:p.P355S,ANK3:ENST00000503366.1:exon31:c.C3664T:p.P1222S,ANK3:ENST00000280772.2:exon30:c.C3661T:p.P1221S,ANK3:ENST00000373827.2:exon31:c.C3643T:p.P1215S,	10q21.2	.
10	64573361	C	T	CCGGTCGCAG,CTTCTGCTGG	exonic	EGR2	nonsynonymous SNV	EGR2:ENST00000439032.1:exon4:c.G1037A:p.G346D,EGR2:ENST00000242480.3:exon2:c.G1037A:p.G346D,EGR2:ENST00000411732.1:exon3:c.G887A:p.G296D,	10q21.3	.
10	64573362	C	T	CGGTCGCAGC,TTCTGCTGGG	exonic	EGR2	nonsynonymous SNV	EGR2:ENST00000439032.1:exon4:c.G1036A:p.G346S,EGR2:ENST00000242480.3:exon2:c.G1036A:p.G346S,EGR2:ENST00000411732.1:exon3:c.G886A:p.G296S,	10q21.3	.
10	68687206	C	T	GCGAATATTC,AAGACTGCCG	exonic	LRRTM3	stopgain	LRRTM3:ENST00000361320.4:exon2:c.C532T:p.Q178X,	10q21.3	.
10	70123764	G	C	ATGATTTGTT,AGTCTCATTT	exonic	RUFY2	nonsynonymous SNV	RUFY2:ENST00000602465.1:exon14:c.C1432G:p.Q478E,RUFY2:ENST00000388768.2:exon14:c.C1537G:p.Q513E,RUFY2:ENST00000265865.3:exon2:c.C202G:p.Q68E,	10q21.3	.
10	71689823	C	T	GCCAGGCCCT,CAGGACACGA	exonic	COL13A1	nonsynonymous SNV	COL13A1:ENST00000520133.1:exon25:c.C1324T:p.P442S,COL13A1:ENST00000479733.1:exon29:c.C1504T:p.P502S,COL13A1:ENST00000357811.3:exon27:c.C1411T:p.P471S,COL13A1:ENST00000354547.3:exon27:c.C1411T:p.P471S,COL13A1:ENST00000398964.3:exon25:c.C1390T:p.P464S,COL13A1:ENS	10q22.1	.
10	72292431	G	A	CCATAACACC,AGGACCTGTG	exonic	PALD1	nonsynonymous SNV	PALD1:ENST00000263563.6:exon6:c.G688A:p.E230K,	10q22.1	.
10	73439160	C	T	GATGAAGACT,CCCTCCCAAC	exonic	CDH23	nonsynonymous SNV	CDH23:ENST00000224721.6:exon16:c.C1784T:p.S595F,CDH23:ENST00000299366.7:exon17:c.C1904T:p.S635F,	10q22.1	.
10	75408208	C	A	TTCCTGGGTG,TGGAGTCTGC	exonic	SYNPO2L	nonsynonymous SNV	SYNPO2L:ENST00000372873.4:exon2:c.G530T:p.S177I,SYNPO2L:ENST00000394810.2:exon4:c.G1202T:p.S401I,	10q22.2	.
10	76735471	C	T	ATAGACTTTT,AAAGCACTAT	exonic	KAT6B	nonsynonymous SNV	KAT6B:ENST00000372711.1:exon7:c.C1376T:p.S459L,KAT6B:ENST00000287239.4:exon8:c.C1376T:p.S459L,	10q22.2	.
10	78709121	G	A	GCTTCACTTC,AGTCTACAAC	exonic	KCNMA1	stopgain	KCNMA1:ENST00000372437.1:exon22:c.C2293T:p.R765X,KCNMA1:ENST00000372408.2:exon21:c.C2125T:p.R709X,KCNMA1:ENST00000372440.1:exon21:c.C2314T:p.R772X,KCNMA1:ENST00000354353.5:exon23:c.C2497T:p.R833X,KCNMA1:ENST00000406533.3:exon23:c.C2500T:p.R834X,KCNMA1:ENST0	10q22.3	.
10	79814332	C	T	GGAAGAATTT,CCAGGAAGAG	exonic	RPS24	nonsynonymous SNV	RPS24:ENST00000440692.1:exon5:c.C434T:p.S145F,	10q22.3	.
10	81067249	C	T	CACGGGCCCC,CCAGCTCTCC	exonic	ZMIZ1	nonsynonymous SNV	ZMIZ1:ENST00000334512.5:exon23:c.C2756T:p.P919L,ZMIZ1:ENST00000446377.2:exon2:c.C215T:p.P72L,	10q22.3	.
10	85964322	C	T	GGATGGAGAC,GGGGCAAACC	exonic	CDHR1	nonsynonymous SNV	CDHR1:ENST00000332904.3:exon9:c.C823T:p.R275W,CDHR1:ENST00000372117.3:exon9:c.C823T:p.R275W,CDHR1:ENST00000440770.2:exon3:c.C100T:p.R34W,	10q23.1	.
10	89574314	C	T	CCAACAACTT,ATTCCGACTC	exonic	ATAD1	nonsynonymous SNV	ATAD1:ENST00000541004.1:exon2:c.G43A:p.E15K,ATAD1:ENST00000328142.3:exon1:c.G43A:p.E15K,ATAD1:ENST00000308448.7:exon2:c.G43A:p.E15K,	10q23.31	.
10	91099869	C	T	CTCTCTAACT,AGAGCAACTG	exonic	IFIT3	nonsynonymous SNV	IFIT3:ENST00000371818.4:exon2:c.C1457T:p.S486L,IFIT3:ENST00000371811.4:exon2:c.C1457T:p.S486L,	10q23.31	.
10	96541671	G	A	TGATATTTTG,AGAAAGTAAA	exonic	CYP2C19	nonsynonymous SNV	CYP2C19:ENST00000371321.3:exon5:c.G736A:p.E246K,	10q23.33	.
10	96954402	G	A	TTTTGCAAAG,ATGTGTTGGA	exonic	C10orf129	nonsynonymous SNV	C10orf129:ENST00000404473.2:exon1:c.G160A:p.D54N,C10orf129:ENST00000327739.3:exon2:c.G160A:p.D54N,C10orf129:ENST00000341686.3:exon2:c.G160A:p.D54N,C10orf129:ENST00000394005.3:exon1:c.G160A:p.D54N,	10q23.33	.
10	97731872	C	T	CTATATATAC,TTTACCTAAC	exonic	RP11-248J23	nonsynonymous SNV	RP11-248J23.6:ENST00000472454.2:exon8:c.C686T:p.P229L,	10q24.1	.
10	98743393	C	T	AAAGTGACTT,AGAAAGGAAA	exonic	C10orf12	nonsynonymous SNV	C10orf12:ENST00000286067.2:exon1:c.C2246T:p.S749L,	10q24.1	.
10	100013448	G	A	CGCAGACTTG,AGAGGCAGTT	exonic	LOXL4	nonsynonymous SNV	LOXL4:ENST00000260702.3:exon11:c.C1697T:p.S566F,	10q24.2	.
10	101603622	G	A	TGTGGCTGTT,AGCGAATAAC	exonic	ABCC2	nonsynonymous SNV	ABCC2:ENST00000370449.4:exon27:c.G3808A:p.E1270K,	10q24.2	.
10	102265159	G	A	CATTTGGGGG,TTTTTTCAGG	exonic	SEC31B	nonsynonymous SNV	SEC31B:ENST00000451524.1:exon10:c.C1138T:p.P380S,SEC31B:ENST00000370345.3:exon10:c.C1138T:p.P380S,	10q24.31	.
10	102732948	G	A	GCTGCTGGAG,AGGCCTCAGC	exonic	SEMA4G	nonsynonymous SNV	SEMA4G:ENST00000370250.4:exon2:c.G187A:p.E63K,SEMA4G:ENST00000519649.1:exon3:c.G187A:p.E63K,SEMA4G:ENST00000518124.1:exon3:c.G187A:p.E63K,SEMA4G:ENST00000517724.1:exon2:c.G187A:p.E63K,SEMA4G:ENST00000210633.3:exon2:c.G187A:p.E63K,SEMA4G:ENST00000521006.1:ex	10q24.31	.
10	102782000	C	T	AACTCCTTGC,CCCACGGATG	exonic	PDZD7	nonsynonymous SNV	PDZD7:ENST00000370215.3:exon5:c.G685A:p.G229S,	10q24.31	.
10	112838602	C	T	GAGGCCGAAC,GCTGCCCACC	exonic	ADRA2A	nonsynonymous SNV	ADRA2A:ENST00000280155.2:exon1:c.C848T:p.P283L,	10q25.2	.
10	121616881	G	A	CCCAGGCACC,GAACACAATA	exonic	MCMBP	nonsynonymous SNV	MCMBP:ENST00000360003.3:exon5:c.C398T:p.P133L,MCMBP:ENST00000369077.3:exon5:c.C398T:p.P133L,	10q26.11	.
10	122650273	C	T	TGTGACCTTT,GTATATTGGA	exonic	WDR11	nonsynonymous SNV	WDR11:ENST00000263461.6:exon19:c.C2389T:p.R797C,	10q26.12	.
10	125506471	C	T	GTGAAACCTT,GGCCTTTGCT	exonic	CPXM2	nonsynonymous SNV	CPXM2:ENST00000241305.3:exon14:c.G2080A:p.E694K,	10q26.13	.
10	125528203	C	T	ACCTCATTGC,GTGGGCCCCC	exonic	CPXM2	nonsynonymous SNV	CPXM2:ENST00000241305.3:exon9:c.G1138A:p.G380S,	10q26.13	.
10	126370591	C	T	GCTGGAACTC,TCTGCATGGT	exonic	FAM53B	nonsynonymous SNV	FAM53B:ENST00000337318.3:exon4:c.G491A:p.R164K,FAM53B:ENST00000280780.6:exon4:c.G491A:p.R164K,FAM53B:ENST00000392754.3:exon4:c.G491A:p.R164K,	10q26.13	.
10	134674344	G	A	GGTCAGAGTG,AATTGTACCA	exonic	TTC40	nonsynonymous SNV	TTC40:ENST00000368586.5:exon36:c.C5033T:p.S1678F,	10q26.3	.
10	134884437	G	A	CGGCAGATGC,AGGCCACGGG	exonic	GPR123	nonsynonymous SNV	GPR123:ENST00000607359.1:exon1:c.G5A:p.R2Q,	10q26.3	.
11	1276611	A	C	TTTGCCCCAC,GGGTCTTTGC	splicing	MUC5B(ENS'.		.	11p15.5	.
11	1466639	G	A	GCACTCACTG,GCTGCTTCCG	exonic	BRSK2	nonsynonymous SNV	BRSK2:ENST00000308219.9:exon10:c.G928A:p.G310S,BRSK2:ENST00000528710.1:exon10:c.G748A:p.G250S,BRSK2:ENST00000528841.1:exon10:c.G928A:p.G310S,BRSK2:ENST00000544817.1:exon5:c.G13A:p.G5S,BRSK2:ENST00000529433.1:exon10:c.G928A:p.G310S,BRSK2:ENST00000531197.1:exon1	11p15.5	.
11	3048019	G	A	AGACCCATTG,AGACATAGCT	exonic	CARS	nonsynonymous SNV	CARS:ENST00000401769.3:exon10:c.C830T:p.S277F,CARS:ENST00000380525.4:exon10:c.C1040T:p.S347F,CARS:ENST00000278224.9:exon9:c.C791T:p.S264F,CARS:ENST00000397111.5:exon9:c.C791T:p.S264F,CARS:ENST00000397114.3:exon10:c.C761T:p.S254F,	11p15.4	.
11	3048020	A	C	GACCCATTGG,GACATAGCTG	exonic	CARS	nonsynonymous SNV	CARS:ENST00000401769.3:exon10:c.T829G:p.S277A,CARS:ENST00000380525.4:exon10:c.T1039G:p.S347A,CARS:ENST00000278224.9:exon9:c.T790G:p.S264A,CARS:ENST00000397111.5:exon9:c.T790G:p.S264A,CARS:ENST00000397114.3:exon10:c.T760G:p.S254A,	11p15.4	.
11	3141658	G	A	CACCTTCACG,CCCAGACGGA	exonic	OSBPL5	nonsynonymous SNV	OSBPL5:ENST00000263650.7:exon6:c.C599T:p.A200V,	11p15.4	.
11	4615433	G	A	CAATCTGGAT,GATTCCACTC	exonic	OR52I1	nonsynonymous SNV	OR52I1:ENST00000530443.2:exon1:c.G165A:p.M55I,OR52I1:ENST00000450052.2:exon2:c.G237A:p.M79I,	11p15.4	.
11	6236030	G	A	GCGGAGCAGG,GCTGGGGGTG	exonic	FAM160A2	nonsynonymous SNV	FAM160A2:ENST00000449352.2:exon10:c.C2327T:p.P776L,FAM160A2:ENST00000265978.4:exon10:c.C2369T:p.P790L,	11p15.4	.
11	6432313	G	A	GTCACATTGC,ATTCTGGTCA	exonic	APBB1	nonsynonymous SNV	APBB1:ENST00000311051.3:exon2:c.C265T:p.R89C,APBB1:ENST00000608435.1:exon2:c.C265T:p.R89C,APBB1:ENST00000299402.6:exon2:c.C265T:p.R89C,APBB1:ENST00000389906.2:exon2:c.C265T:p.R89C,APBB1:ENST00000609360.1:exon2:c.C265T:p.R89C,	11p15.4	.
11	6643024	C	T	ATGTGCAGCT,CGTGTCATCA	exonic	DCHS1	nonsynonymous SNV	DCHS1:ENST00000299441.3:exon21:c.G9883A:p.E3295K,	11p15.4	.
11	7059945	G	A	CCTGAGCATG,CCTGACACCC	exonic	NLRP14	nonsynonymous SNV	NLRP14:ENST00000299481.4:exon2:c.G128A:p.G43D,	11p15.4	.
11	8008857	G	A	TGATATTCAT,AATGCTGTCA	exonic	EIF3F	nonsynonymous SNV	EIF3F:ENST00000537635.1:exon1:c.G3A:p.M1I,	11p15.4	.
11	10050094	G	A	TGCACCCAAA,AAAACAGCTT	exonic	SBF2	nonsynonymous SNV	SBF2:ENST00000256190.8:exon6:c.C524T:p.S175F,	11p15.4	.
11	10515214	C	G	TCTCTTCTAT,TGACTGAGAG	exonic	AMPD3	nonsynonymous SNV	AMPD3:ENST00000533116.1:exon1:c.C301G:p.L101V,	11p15.4	.
11	12262627	C	T	GTCCTGTGCT,TTCCTCCGGC	exonic	MICAL2	nonsynonymous SNV	MICAL2:ENST00000342902.5:exon17:c.C2255T:p.S752F,MICAL2:ENST00000256194.4:exon18:c.C2255T:p.S752F,	11p15.3	.
11	15257208	G	A	CATCATTCAG,AAAATGGTAT	exonic	INSC	nonsynonymous SNV	INSC:ENST00000530161.1:exon9:c.G1231A:p.E411K,INSC:ENST00000525218.1:exon8:c.G1105A:p.E369K,INSC:ENST00000379556.3:exon10:c.G1231A:p.E411K,INSC:ENST00000379554.3:exon10:c.G1372A:p.E458K,INSC:ENST00000424273.1:exon9:c.G1105A:p.E369K,INSC:ENST00000528567.1:exon9:c.G1	11p15.2	.
11	15260572	C	T	GACCCTGGCT,GTCTCAGCCG	exonic	INSC	nonsynonymous SNV	INSC:ENST00000530161.1:exon10:c.C1345T:p.R449C,INSC:ENST00000525218.1:exon9:c.C1219T:p.R407C,INSC:ENST00000379556.3:exon11:c.C1345T:p.R449C,INSC:ENST00000379554.3:exon11:c.C1486T:p.R496C,INSC:ENST00000424273.1:exon10:c.C1219T:p.R407C,INSC:ENST00000528567.1:exon10:c	11p15.2	.
11	17531252	G	A	GGGAGTCTTG,GGGGATAGTA	exonic	USH1C	nonsynonymous SNV	USH1C:ENST00000005226.7:exon18:c.C1664T:p.P555L,	11p15.1	.
11	17546005	C	T	CACCTCCAAT,CCACCTCAGC	exonic	USH1C	nonsynonymous SNV	USH1C:ENST00000318024.4:exon9:c.G752A:p.G251E,USH1C:ENST00000526181.1:exon9:c.G785A:p.G262E,USH1C:ENST00000527720.1:exon8:c.G659A:p.G220E,USH1C:ENST00000526313.1:exon9:c.G752A:p.G251E,USH1C:ENST00000527020.1:exon9:c.G752A:p.G251E,USH1C:ENST00000005226.7:exon9	11p15.1	.
11	17608098	G	A	TATTTCCCTG,GGACCAGGTG	exonic	OTOG	stopgain	OTOG:ENST00000342528.2:exon2:c.G57A:p.W19X,	11p15.1	.
11	18194916	C	T	TGCATCATTT,CCTTGTCGGA	exonic	MRGPRX4	nonsynonymous SNV	MRGPRX4:ENST00000314254.3:exon1:c.C113T:p.S38F,	11p15.1	M
11	22364802	T	A	GAAAGCCAAA,TCAACTGGGA	exonic	SLC17A6	nonsynonymous SNV	SLC17A6:ENST00000263160.3:exon3:c.T349A:p.F117I,	11p14.3	.
11	26692720	C	T	GGAACATGTA,CAGGCTTTCT	exonic	SLC5A12	nonsynonymous SNV	SLC5A12:ENST00000396005.3:exon15:c.G1786A:p.V596I,	11p14.2	.
11	46407402	C	T	GCTTTCTTCT,CTTCGGGCCC	exonic	CHRM4	nonsynonymous SNV	CHRM4:ENST00000433765.2:exon1:c.G706A:p.E236K,	11p11.2	.
11	46726478	C	T	GCGGCCCTTC,CCTGTCCACA	exonic	ZNF408	nonsynonymous SNV	ZNF408:ENST00000311764.2:exon5:c.C1228T:p.P410S,	11p11.2	.
11	46726479	C	T	CGGCCCTTCC,CTGTCCACAA	exonic	ZNF408	nonsynonymous SNV	ZNF408:ENST00000311764.2:exon5:c.C1229T:p.P410L,	11p11.2	.
11	47827755	C	G	CAGGCCAATT,AATCCAGTTT	exonic	NUP160	nonsynonymous SNV	NUP160:ENST00000378460.2:exon20:c.G2556C:p.L852F,NUP160:ENST00000530326.1:exon20:c.G2214C:p.L738F,NUP160:ENST00000528071.1:exon18:c.G2214C:p.L738F,	11p11.2	.
11	55340166	C	G	CAAGCCTGTT,AGAAACCTAT	exonic	OR4C16	stopgain	OR4C16:ENST00000314634.3:exon1:c.C563G:p.S188X,	11q11	.
11	55702967	C	T	AGAACTTTCT,AGCTGCATCC	exonic	OR5I1	nonsynonymous SNV	OR5I1:ENST00000301532.3:exon1:c.G910A:p.E304K,	11q12.1	.
11	55762082	G	T	AGTCAGTGAG,TATAATTTTT	exonic	OR5F1	nonsynonymous SNV	OR5F1:ENST00000278409.1:exon1:c.C20A:p.T7N,	11q12.1	.
11	55798033	A	T	ACTCTTAATA,TTCTAGTTAA	exonic	OR5AS1	nonsynonymous SNV	OR5AS1:ENST00000313555.1:exon1:c.A139T:p.I47F,	11q12.1	.
11	56085928	C	T	ATTGTCCTCA,CAAGTTGGAC	exonic	OR8K3	nonsynonymous SNV	OR8K3:ENST00000312711.1:exon1:c.C146T:p.T49I,	11q12.1	.
11	56113740	G	A	GTCAATCACT,ATCTTGGTTA	exonic	OR8K1	nonsynonymous SNV	OR8K1:ENST00000279783.2:exon1:c.G226A:p.D76N,	11q12.1	.
11	56258650	G	A	ATCCACGAAG,ATAAGTGGCT	exonic	OR5M8	nonsynonymous SNV	OR5M8:ENST00000327216.2:exon1:c.C197T:p.S66F,	11q12.1	.
11	56310061	G	A	TTGATCCGGA,GATGGCAGCA	exonic	OR5M11	nonsynonymous SNV	OR5M11:ENST00000528616.2:exon1:c.C673T:p.L225F,	11q12.1	.
11	60231791	A	G	CCTGGCAGCA,CGGAGAAAAA	exonic	MS4A1	nonsynonymous SNV	MS4A1:ENST00000534668.1:exon4:c.A310G:p.T104A,MS4A1:ENST00000533306.1:exon6:c.A319G:p.T107A,MS4A1:ENST00000389939.2:exon3:c.A310G:p.T104A,MS4A1:ENST00000345732.4:exon4:c.A310G:p.T104A,MS4A1:ENST00000532073.1:exon4:c.A310G:p.T104A,	11q12.2	.
11	62362037	G	C	CCGGTTCTGG,ACAGCTGGTT	exonic	MTA2	nonsynonymous SNV	MTA2:ENST00000278823.2:exon16:c.C1643G:p.S548C,MTA2:ENST00000524902.1:exon14:c.C1124G:p.S375C,MTA2:ENST00000527204.1:exon16:c.C1124G:p.S375C,	11q12.3	.
11	62416137	G	A	ATCTAAAAAG,GCACCAAGCG	exonic	INTS5	nonsynonymous SNV	INTS5:ENST00000330574.2:exon2:c.C1415T:p.P472L,	11q12.3	.
11	64000208	C	T	CAGTACTGGT,CCAGTTTCAC	exonic	DNAJC4	nonsynonymous SNV	DNAJC4:ENST00000321685.3:exon5:c.C398T:p.S133F,DNAJC4:ENST00000321460.5:exon4:c.C401T:p.S134F,	11q13.1	.
11	64032493	C	T	TCCTCAAACC,CACCCCCAGC	exonic	PLCB3	nonsynonymous SNV	PLCB3:ENST00000325234.5:exon21:c.C2522T:p.P841L,PLCB3:ENST00000540288.1:exon23:c.C2723T:p.P908L,PLCB3:ENST00000279230.6:exon23:c.C2723T:p.P908L,	11q13.1	.
11	64522792	G	A	GCCAGGTTTC,GTCCAACACA	exonic	PYGM	stopgain	PYGM:ENST00000377432.3:exon5:c.C544T:p.R182X,PYGM:ENST00000164139.3:exon7:c.C808T:p.R270X,	11q13.1	.
11	65422358	G	A	ACTTGGGGAG,GGCCGGGGCC	exonic	RELA	nonsynonymous SNV	RELA:ENST00000308639.9:exon11:c.C1138T:p.P380S,RELA:ENST00000532999.1:exon11:c.C1180T:p.P394S,RELA:ENST00000406246.3:exon11:c.C1147T:p.P383S,	11q13.1	.
11	66463828	C	G	TGCAGGCTCT,CAGCCAGCTC	exonic	SPTBN2	nonsynonymous SNV	SPTBN2:ENST00000309996.2:exon20:c.G4198C:p.E1400Q,SPTBN2:ENST00000529997.1:exon19:c.G4198C:p.E1400Q,SPTBN2:ENST00000533211.1:exon21:c.G4198C:p.E1400Q,	11q13.2	.
11	66475718	G	A	GATGAGCTCG,TGCGCAGGGC	exonic	SPTBN2	nonsynonymous SNV	SPTBN2:ENST00000309996.2:exon11:c.C1244T:p.T415I,SPTBN2:ENST00000529997.1:exon10:c.C1244T:p.T415I,SPTBN2:ENST00000533211.1:exon12:c.C1244T:p.T415I,	11q13.2	.
11	67258448	G	A	CGGTTCCGGG,GATCTTCTCC	exonic	AIP	nonsynonymous SNV	AIP:ENST00000279146.3:exon6:c.G977A:p.G326E,	11q13.2	.
11	68821501	G	A	CTGCCTGCAG,TGCCGCGGCC	exonic	TPCN2	nonsynonymous SNV	TPCN2:ENST00000542467.1:exon2:c.G110A:p.G37D,TPCN2:ENST00000294309.3:exon2:c.G110A:p.G37D,	11q13.3	.
11	70007364	A	G	GTGCGGTCCA,CATCCGGGTC	exonic	ANO1	nonsynonymous SNV	ANO1:ENST00000355303.5:exon17:c.A1676G:p.N559S,ANO1:ENST00000531300.1:exon6:c.A329G:p.N110S,ANO1:ENST00000538023.1:exon17:c.A1676G:p.N559S,ANO1:ENST00000530676.1:exon15:c.A1238G:p.N413S,ANO1:ENST00000316296.5:exon15:c.A1502G:p.N501S,ANO1:ENST00000531349.1:exon1	11q13.3	.
11	70331908	C	T	GCTGCTACTC,GGCTGTCCAC	exonic	SHANK2	nonsynonymous SNV	SHANK2:ENST00000423696.2:exon15:c.G3353A:p.R1118Q,SHANK2:ENST00000424924.1:exon6:c.G2327A:p.R776Q,SHANK2:ENST00000338508.4:exon32:c.G4493A:p.R1498Q,SHANK2:ENST00000409161.1:exon9:c.G2702A:p.R901Q,SHANK2:ENST00000294018.4:exon14:c.G3362A:p.R1121Q,SHANK2:ENST1	11q13.3	.
11	70644647	C	T	AAGCCCCCTT,ACCGATGCTC	exonic	SHANK2	nonsynonymous SNV	SHANK2:ENST00000423696.2:exon4:c.G541A:p.E181K,SHANK2:ENST00000338508.4:exon20:c.G1681A:p.E561K,SHANK2:ENST00000294018.4:exon4:c.G571A:p.E191K,	11q13.4	.
11	71276847	G	A	GGGCTCCAAG,GGGACTGTGG	exonic	KRTAP5-10	nonsynonymous SNV	KRTAP5-10:ENST00000376536.4:exon1:c.G214A:p.G72R,KRTAP5-10:ENST00000398531.1:exon1:c.G214A:p.G72R,	11q13.4	.
11	71712325	C	T	GGCAATGGTT,CTTCATTGAG	exonic	IL18BP	nonsynonymous SNV	IL18BP:ENST00000393703.4:exon4:c.C314T:p.S105F,IL18BP:ENST00000343898.5:exon4:c.C314T:p.S105F,IL18BP:ENST00000497194.2:exon3:c.C314T:p.S105F,IL18BP:ENST00000337131.5:exon6:c.C314T:p.S105F,IL18BP:ENST00000260049.5:exon4:c.C314T:p.S105F,IL18BP:ENST00000531053.1:exon4:c.C	11q13.4	.
11	72295361	G	A	ATGACCTCTG,GGAAGGGAGA	exonic	PDE2A	nonsynonymous SNV	PDE2A:ENST00000441209.2:exon1:c.C157T:p.P53S,	11q13.4	.

11	74617347	C	G	TCTGAGTCCT,AAGCATCCTT	exonic	XRRA1	nonsynonymous SNV	XRRA1:ENST00000531849.1:exon8:c.G217C:p.E73Q,XRRA1:ENST00000340360.6:exon10:c.G916C:p.E306Q,XRRA1:ENST00000321448.8:exon8:c.G217C:p.E73Q,XRRA1:ENST00000527087.1:exon10:c.G916C:p.E306Q,	11q13.4	.
11	74716515	C	T	GACCATGAAC,CCTGTCCTGT	exonic	NEU3	nonsynonymous SNV	NEU3:ENST00000531509.1:exon4:c.C364T:p.P122S,NEU3:ENST00000545272.1:exon2:c.C37T:p.P13S,NEU3:ENST00000532963.1:exon2:c.C152T:p.P51L,NEU3:ENST00000294064.4:exon3:c.C364T:p.P122S,NEU3:ENST00000544263.1:exon4:c.C265T:p.P89S,NEU3:ENST00000529024.1:exon3:c.C152T:p.P51L,	11q13.4	NI
11	76507052	C	T	TTCACCAGCT,ACCCCTGAGC	exonic	TSKU	nonsynonymous SNV	TSKU:ENST00000533752.1:exon2:c.C392T:p.S131L,TSKU:ENST00000527881.1:exon2:c.C392T:p.S131L,TSKU:ENST00000333090.4:exon2:c.C392T:p.S131L,	11q13.5	.
11	85375207	C	T	GGCTGCCAGA,CCCGGACTCG	exonic	CREBZF	nonsynonymous SNV	CREBZF:ENST00000398294.2:exon1:c.G467A:p.G156D,CREBZF:ENST00000490820.2:exon1:c.G713A:p.G238D,CREBZF:ENST00000527447.1:exon1:c.G713A:p.G238D,CREBZF:ENST00000260058.5:exon1:c.G491A:p.G164D,CREBZF:ENST00000525639.1:exon1:c.G713A:p.G238D,	11q14.1	.
11	85375208	C	T	GCTGCCAGAC,CCGGACTCGA	exonic	CREBZF	nonsynonymous SNV	CREBZF:ENST00000398294.2:exon1:c.G466A:p.G156S,CREBZF:ENST00000490820.2:exon1:c.G712A:p.G238S,CREBZF:ENST00000527447.1:exon1:c.G712A:p.G238S,CREBZF:ENST00000260058.5:exon1:c.G490A:p.G164S,CREBZF:ENST00000525639.1:exon1:c.G712A:p.G238S,	11q14.1	.
11	85375223	C	T	ACTCGACTCT,CAGCCCCATC	exonic	CREBZF	nonsynonymous SNV	CREBZF:ENST00000398294.2:exon1:c.G451A:p.E151K,CREBZF:ENST00000490820.2:exon1:c.G697A:p.E233K,CREBZF:ENST00000527447.1:exon1:c.G697A:p.E233K,CREBZF:ENST00000260058.5:exon1:c.G475A:p.E159K,CREBZF:ENST00000525639.1:exon1:c.G697A:p.E233K,	11q14.1	.
11	88924452	C	G	CGGCGTAATC,TGGAAACCAT	exonic	TYR	nonsynonymous SNV	TYR:ENST00000263321.5:exon2:c.C902G:p.P301R,	11q14.3	.
11	93458911	C	T	ATGCATTCTT,TCTTAACACA	exonic	KIAA1731	nonsynonymous SNV	KIAA1731:ENST00000325212.6:exon23:c.C6413T:p.S2138F,KIAA1731:ENST00000529185.1:exon2:c.C11T:p.S4F,KIAA1731:ENST00000531700.1:exon10:c.C953T:p.S318F,KIAA1731:ENST00000411936.1:exon23:c.C6413T:p.S2138F,KIAA1731:ENST00000344196.4:exon22:c.C953T:p.S318F,KIAA1731:ENST0000	11q21	.
11	93527178	G	A	CTCTTCAAAC,ACCTTTGCCC	exonic	MED17	nonsynonymous SNV	MED17:ENST00000251871.3:exon5:c.G836A:p.R279Q,	11q21	.
11	94169022	G	A	TGAAGTGGTA,GAAAAATGTC	exonic	MRE11A	nonsynonymous SNV	MRE11A:ENST00000407439.3:exon18:c.C1979T:p.P660L,MRE11A:ENST00000393241.4:exon18:c.C1967T:p.P656L,MRE11A:ENST00000323977.3:exon17:c.C1886T:p.P629L,MRE11A:ENST00000323929.3:exon18:c.C1970T:p.P657L,	11q21	.
11	94592790	C	T	GCCCTGGAGG,CGACATGACA	exonic	AMOTL1	nonsynonymous SNV	AMOTL1:ENST00000317829.8:exon8:c.C1895T:p.A632V,AMOTL1:ENST00000433060.2:exon9:c.C2045T:p.A682V,	11q21	.
11	94910889	C	T	ATACCTGATT,CAAACATACA	exonic	SESN3	nonsynonymous SNV	SESN3:ENST00000278499.2:exon7:c.G824A:p.G275E,SESN3:ENST00000536441.1:exon8:c.G1241A:p.G414E,	11q21	.
11	100126495	C	T	CCTATAGGAC,CCCAGGCCCA	exonic	CNTN5	nonsynonymous SNV	CNTN5:ENST00000527185.1:exon17:c.C2009T:p.P670L,CNTN5:ENST00000418526.2:exon14:c.C1787T:p.P596L,CNTN5:ENST00000524871.1:exon17:c.C2009T:p.P670L,CNTN5:ENST00000279463.3:exon15:c.C2009T:p.P670L,CNTN5:ENST00000528682.1:exon16:c.C2009T:p.P670L,	11q22.1	.
11	100999182	C	T	CACTGGGGCC,CGGACCAGTG	exonic	PGR	nonsynonymous SNV	PGR:ENST00000263463.5:exon1:c.G620A:p.G207E,PGR:ENST00000528960.1:exon1:c.G620A:p.G207E,PGR:ENST00000325455.5:exon1:c.G620A:p.G207E,PGR:ENST00000534780.1:exon1:c.G620A:p.G207E,PGR:ENST00000526300.1:exon1:c.G620A:p.G207E,	11q22.1	.
11	113650977	C	T	TGACAGCATC,CTGACATCAT	exonic	CLDN25	nonsynonymous SNV	CLDN25:ENST00000453129.2:exon1:c.C460T:p.P154S,	11q23.2	.
11	114401156	G	A	GCCCTCCAGA,AGCCGACGCC	exonic	NXPE1	nonsynonymous SNV	NXPE1:ENST00000251921.2:exon3:c.C148T:p.L50F,NXPE1:ENST00000536312.1:exon6:c.C574T:p.L192F,NXPE1:ENST00000424269.1:exon2:c.C574T:p.L192F,	11q23.2	F/
11	117389424	C	T	GAGAACTGCT,CCCGGGGTTG	exonic	DSCAML1	nonsynonymous SNV	DSCAML1:ENST00000527706.1:exon5:c.G637A:p.E213K,DSCAML1:ENST00000321322.6:exon7:c.G1447A:p.E483K,	11q23.3	.
11	118239432	C	T	TGAGATTAGC,GCTCATTCCG	exonic	UBE4A	nonsynonymous SNV	UBE4A:ENST00000431736.2:exon3:c.C208T:p.R70C,UBE4A:ENST00000252108.3:exon3:c.C208T:p.R70C,	11q23.3	.
11	118242304	C	T	AACGCTAAAT,TCTCTGCTGA	exonic	UBE4A	nonsynonymous SNV	UBE4A:ENST00000431736.2:exon5:c.C484T:p.L162F,UBE4A:ENST00000252108.3:exon5:c.C484T:p.L162F,	11q23.3	.
11	120837909	G	A	CGCAGGCTCG,TTTTCCGGGA	exonic	GRIK4	nonsynonymous SNV	GRIK4:ENST00000527524.2:exon19:c.G2272A:p.V758I,GRIK4:ENST00000438375.2:exon18:c.G2272A:p.V758I,	11q23.3	.
11	120996293	G	A	GTACCACGCA,ACTGGAAGTG	exonic	TECTA	nonsynonymous SNV	TECTA:ENST00000264037.2:exon7:c.G1486A:p.D496N,TECTA:ENST00000392793.1:exon8:c.G1486A:p.D496N,	11q23.3	.
11	122665514	C	A	GAGACGACTC,TCTTACTATC	exonic	UBASH3B	nonsynonymous SNV	UBASH3B:ENST00000284273.5:exon7:c.C1085A:p.P362H,	11q24.1	.
11	122774967	C	T	CAGCCTTTCT,CGTACGTGAA	exonic	C11orf63	nonsynonymous SNV	C11orf63:ENST00000531316.1:exon2:c.C679T:p.P227S,C11orf63:ENST00000227349.2:exon3:c.C679T:p.P227S,C11orf63:ENST00000307257.6:exon3:c.C679T:p.P227S,	11q24.1	.
11	123676348	G	A	AGAAGCACAG,TAGAAAAAGC	exonic	OR6M1	nonsynonymous SNV	OR6M1:ENST00000309154.2:exon1:c.C710T:p.T237I,	11q24.1	.
11	123894302	G	A	CTCAGCCAAC,AGATGGTCAT	exonic	OR10G9	nonsynonymous SNV	OR10G9:ENST00000375024.1:exon1:c.G583A:p.E195K,	11q24.1	.
11	123909348	G	A	GCCAGGTAGC,ATCATAGGAC	exonic	OR10G7	nonsynonymous SNV	OR10G7:ENST00000330487.5:exon1:c.C361T:p.R121C,	11q24.2	.
11	124120852	C	A	TTGCTTTTCT,TGATTTTAGG	exonic	OR8G1	nonsynonymous SNV	OR8G1:ENST00000341493.2:exon1:c.C430A:p.L144M,	11q24.2	.
11	132180051	G	A	GTCCCCGTGG,ACAAAAGGGG	exonic	NTM	nonsynonymous SNV	NTM:ENST00000427481.2:exon6:c.G680A:p.G227E,NTM:ENST00000539799.1:exon6:c.G707A:p.G236E,NTM:ENST00000374786.1:exon5:c.G707A:p.G236E,NTM:ENST00000374791.3:exon6:c.G707A:p.G236E,NTM:ENST00000374784.1:exon5:c.G707A:p.G236E,NTM:ENST00000425719.2:exon5:c.G707A:p.G23	11q25	.
12	1895131	C	T	TCAGCTGTTT,ATATCTTTGT	exonic	ADIPOR2	nonsynonymous SNV	ADIPOR2:ENST00000357103.4:exon8:c.C1054T:p.H352Y,	12p13.33	.
12	2939956	C	T	GTCCTGCATC,GGGGAGGCTC	exonic	NRIP2	nonsynonymous SNV	NRIP2:ENST00000542990.1:exon3:c.G263A:p.R88Q,NRIP2:ENST00000542386.1:exon3:c.G263A:p.R88Q,NRIP2:ENST00000337508.4:exon2:c.G413A:p.R138Q,	12p13.33	.
12	4920510	G	A	GACTGTGGGG,GAAAGATCGT	exonic	KCNA6	nonsynonymous SNV	KCNA6:ENST00000433855.1:exon1:c.G1303A:p.G435R,KCNA6:ENST00000280684.3:exon1:c.G1303A:p.G435R,	12p13.32	.
12	6091023	G	A	TACCCAAGGG,ACAGCTCACT	exonic	VWF	nonsynonymous SNV	VWF:ENST00000261405.5:exon42:c.C7216T:p.P2406S,	12p13.31	.
12	6091032	C	T	GGACAGCTCA,TGTGGAGTTG	exonic	VWF	nonsynonymous SNV	VWF:ENST00000261405.5:exon42:c.G7207A:p.V2403M,	12p13.31	.
12	6126007	G	A	CCATCCAGGA,AAGGATCACG	exonic	VWF	nonsynonymous SNV	VWF:ENST00000261405.5:exon29:c.C5083T:p.L1695F,	12p13.31	.
12	7053822	C	G	GAAGGTGGGT,AGACGCGGGA	exonic	C12orf57	stopgain	C12orf57:ENST00000544681.1:exon2:c.C236G:p.S79X,	12p13.31	.
12	7254437	C	T	TTGAAGATAC,AGGGTCATGG	exonic	C1RL	nonsynonymous SNV	C1RL:ENST00000545337.1:exon3:c.G547A:p.G183S,	12p13.31	.
12	10603136	C	T	ACAGATAATT,CCAGGATCCC	exonic	KLRC1	nonsynonymous SNV	KLRC1:ENST00000347831.5:exon3:c.G230A:p.G77E,KLRC1:ENST00000359151.3:exon3:c.G230A:p.G77E,KLRC1:ENST00000536188.1:exon4:c.G230A:p.G77E,KLRC1:ENST00000544822.1:exon4:c.G230A:p.G77E,KLRC1:ENST00000408006.3:exon4:c.G230A:p.G77E,	12p13.2	.
12	11139354	C	T	TTTCTCTTCA,CCAGTCAATG	exonic	TAS2R50	nonsynonymous SNV	TAS2R50:ENST00000506868.1:exon1:c.G106A:p.V36M,	12p13.2	.
12	11546678	G	A	GGAGGAGATC,GGGACTTCGG	exonic	PRB2	stopgain	PRB2:ENST00000389362.4:exon3:c.C334T:p.R112X,	12p13.2	.
12	14634132	T	A	AAGAGATTTT,CTTGTATATG	exonic	ATF7IP	nonsynonymous SNV	ATF7IP:ENST00000543189.1:exon13:c.T3290A:p.F1097Y,	12p13.1	.
12	15679137	C	T	TATAACCTCT,AGTAACTGCT	exonic	PTPRO	nonsynonymous SNV	PTPRO:ENST00000281171.4:exon12:c.C2105T:p.S702L,PTPRO:ENST00000348962.2:exon12:c.C2105T:p.S702L,	12p12.3	.
12	27950684	C	T	TGCCCCTCTT,CGACATGTGG	exonic	KLHL42	nonsynonymous SNV	KLHL42:ENST00000381271.2:exon3:c.C1103T:p.S368F,	12p11.22	.
12	31854886	G	A	CAGGAGAGCA,CATCTGATAT	exonic	AMN1	nonsynonymous SNV	AMN1:ENST00000537562.1:exon6:c.C173T:p.A58V,AMN1:ENST00000506446.2:exon4:c.C233T:p.A78V,AMN1:ENST00000281471.6:exon3:c.C227T:p.A76V,AMN1:ENST00000536761.1:exon5:c.C173T:p.A58V,AMN1:ENST00000535408.1:exon3:c.C173T:p.A58V,AMN1:ENST00000537960.1:exon4:c.C173T:p.A58V	12p11.21	.
12	32832376	C	G	CCAGCTGCCT,AAATCGTCGT	exonic	DNM1L	nonsynonymous SNV	DNM1L:ENST00000266481.6:exon1:c.C79G:p.Q27E,DNM1L:ENST00000546649.1:exon1:c.C79G:p.Q27E,DNM1L:ENST00000551476.1:exon1:c.C79G:p.Q27E,DNM1L:ENST00000413295.2:exon1:c.C79G:p.Q27E,DNM1L:ENST00000452533.2:exon1:c.C79G:p.Q27E,DNM1L:ENST00000549701.1:exon1:c.C79G:p.(	12p11.21	.
12	39047728	G	C	GTGTATGGGG,AGGCGCAGGT	exonic	CPNE8	nonsynonymous SNV	CPNE8:ENST00000538596.2:exon8:c.C658G:p.P220A,CPNE8:ENST00000360449.3:exon20:c.C1615G:p.P539A,CPNE8:ENST00000331366.5:exon20:c.C1651G:p.P551A,	12q12	.
12	40814194	G	A	TGACAATAAC,ACGTCTCTAT	exonic	MUC19	nonsynonymous SNV	MUC19:ENST00000425730.2:exon7:c.G553A:p.D185N,	12q12	.
12	40829983	C	T	AACCCTAAAC,CTATCATGAG	exonic	MUC19	nonsynonymous SNV	MUC19:ENST00000454784.4:exon19:c.C1331T:p.P444L,	12q12	.
12	40874387	G	A	ATTAGATATA,GATCAGGGAC	exonic	MUC19	nonsynonymous SNV	MUC19:ENST00000454784.4:exon49:c.G5932A:p.G1978R,	12q12	.
12	40874388	G	A	TTAGATATAG,ATCAGGGACA	exonic	MUC19	nonsynonymous SNV	MUC19:ENST00000454784.4:exon49:c.G5933A:p.G1978E,	12q12	.
12	40878765	T	C	GCAACAGGGA,AACTGGACTA	exonic	MUC19	nonsynonymous SNV	MUC19:ENST00000454784.4:exon49:c.T10310C:p.I3437T,	12q12	.
12	46589932	G	A	ACTAAATCCA,AATCCAGAGA	exonic	SLC38A1	nonsynonymous SNV	SLC38A1:ENST00000552197.1:exon17:c.C1490T:p.S497F,	12q13.11	.
12	48132488	G	C	TCTCAAAGTT,ATGAGATTCT	exonic	RAPGEF3	nonsynonymous SNV	RAPGEF3:ENST00000171000.4:exon25:c.C2385G:p.I795M,RAPGEF3:ENST00000405493.2:exon25:c.C2385G:p.I795M,RAPGEF3:ENST00000549151.1:exon25:c.C2385G:p.I795M,RAPGEF3:ENST00000548919.1:exon24:c.C2184G:p.I728M,RAPGEF3:ENST00000449771.2:exon25:c.C2511G:p.I837M,RAPGEF3:E	12q13.11	.
12	48723402	C	T	CACGCTCCTC,GGGTCAGCGG	exonic	H1FNT	nonsynonymous SNV	H1FNT:ENST00000335017.1:exon1:c.C328T:p.R110W,	12q13.11	.
12	49958630	G	A	GTGAGTCCAG,GGCTGGGGCT	exonic	MCRS1	nonsynonymous SNV	MCRS1:ENST00000550165.1:exon6:c.C337T:p.P113S,MCRS1:ENST00000548334.1:exon5:c.C337T:p.P113S,MCRS1:ENST00000343810.4:exon5:c.C337T:p.P113S,MCRS1:ENST00000357123.4:exon4:c.C376T:p.P126S,MCRS1:ENST00000549528.1:exon5:c.C349T:p.P117S,MCRS1:ENST00000553173.1:exon4:	12q13.12	.
12	49958631	G	A	TGAGTCCAGG,GCTGGGGCTG	exonic	MCRS1	nonsynonymous SNV	MCRS1:ENST00000548602.1:exon3:c.C164T:p.P55L,	12q13.12	.
12	50195723	T	C	ACTCTCTTCT,CAGCTTGATG	exonic	NCKAP5L	nonsynonymous SNV	NCKAP5L:ENST00000335999.6:exon6:c.A259G:p.K87E,	12q13.12	.
12	50236666	G	C	TCGAGCCCCA,AATCGGCCCG	exonic	BCDIN3D	nonsynonymous SNV	BCDIN3D:ENST00000333924.4:exon1:c.C205G:p.L69V,	12q13.12	BC
12	51382172	C	A	TACCGTATGC,CACAGTCCAG	exonic	SLC11A2	nonsynonymous SNV	SLC11A2:ENST00000547688.1:exon17:c.G1709T:p.G570V,SLC11A2:ENST00000541174.2:exon16:c.G1622T:p.G541V,SLC11A2:ENST00000262052.5:exon16:c.G1622T:p.G541V,SLC11A2:ENST00000545993.2:exon15:c.G1610T:p.G537V,SLC11A2:ENST00000546743.1:exon16:c.G1385T:p.G462V,SLC11A2:ENS	12q13.12	.
12	51739592	G	A	AGAGGGCCAG,AATTCCTCCC	exonic	CELA1	nonsynonymous SNV	CELA1:ENST00000293636.1:exon2:c.C86T:p.S29F,	12q13.13	.
12	53345412	A	C	GGAGCTAGAC,AGTACTGGTC	exonic	KRT18	nonsynonymous SNV	KRT18:ENST00000550600.1:exon5:c.A805C:p.K269Q,KRT18:ENST00000388837.2:exon5:c.A805C:p.K269Q,KRT18:ENST00000388835.3:exon4:c.A805C:p.K269Q,	12q13.13	.
12	54930003	C	G	ACTGACCCTT,TTCCTTTTAT	exonic	NCKAP1L	nonsynonymous SNV	NCKAP1L:ENST00000293373.6:exon28:c.C3047G:p.S1016C,NCKAP1L:ENST00000545638.2:exon28:c.C2897G:p.S966C,	12q13.2	N(
12	55714973	G	A	GAGGTGATGG,ATTTTCTTGT	exonic	OR6C1	nonsynonymous SNV	OR6C1:ENST00000379668.2:exon1:c.G590A:p.G197E,	12q13.2	O
12	55846809	G	A	ATAAATAAAG,AGTTTCAGTT	exonic	OR6C2	nonsynonymous SNV	OR6C2:ENST00000322678.1:exon1:c.G812A:p.G271E,	12q13.2	.
12	56345529	C	T	TGACCCAGTC,CCTTTCAAAT	exonic	DGKA	nonsynonymous SNV	DGKA:ENST00000394147.1:exon18:c.C1549T:p.P517S,DGKA:ENST00000551156.1:exon18:c.C1549T:p.P517S,DGKA:ENST00000552903.2:exon5:c.C379T:p.P127S,DGKA:ENST00000331886.5:exon18:c.C1549T:p.P517S,DGKA:ENST00000555218.1:exon17:c.C1306T:p.P436S,	12q13.2	.
12	56418876	G	A	GTTCCTCGGG,CCCCAGTGGG	exonic	IKZF4	nonsynonymous SNV	IKZF4:ENST00000547791.1:exon2:c.G85A:p.A29T,IKZF4:ENST00000547167.1:exon3:c.G220A:p.A74T,IKZF4:ENST00000262032.5:exon7:c.G220A:p.A74T,	12q13.2	.
12	57112370	G	A	GCCCCTTTGG,GGATGGAGTA	exonic	NACA	nonsynonymous SNV	NACA:ENST00000454682.1:exon3:c.C2944T:p.P982S,	12q13.3	.
12	57572328	G	C	CCGTGGTACA,AGGACCAACA	exonic	LRP1	nonsynonymous SNV	LRP1:ENST00000243077.3:exon27:c.G4548C:p.Q1516H,	12q13.3	.
12	57865177	C	T	GACTTTGATT,CCCCACCCAT	exonic	GLI1	nonsynonymous SNV	GLI1:ENST00000543426.1:exon10:c.C2270T:p.S757F,GLI1:ENST00000228682.2:exon12:c.C2654T:p.S885F,GLI1:ENST00000546141.1:exon11:c.C2531T:p.S844F,GLI1:ENST00000528467.1:exon10:c.C2531T:p.S844F,	12q13.3	.
12	58177000	G	A	AGCTGCGGCG,AAAACAGGCT	exonic	RP11-571M6.	nonsynonymous SNV	RP11-571M6.15:ENST00000546504.1:exon2:c.G184A:p.E62K,	12q14.1	.
12	65116805	G	A	AGGACTCAGG,AAGGGCATGT	exonic	GNS	nonsynonymous SNV	GNS:ENST00000543646.1:exon12:c.C1385T:p.S462F,GNS:ENST00000258145.3:exon11:c.C1289T:p.S430F,GNS:ENST00000418919.2:exon10:c.C1121T:p.S374F,GNS:ENST00000542058.1:exon10:c.C1229T:p.S410F,	12q14.3	G
12	65152900	G	A	TCGTCCGTGA,GAGCAGCACC	exonic	GNS	nonsynonymous SNV	GNS:ENST00000543646.1:exon1:c.C157T:p.L53F,GNS:ENST00000258145.3:exon1:c.C157T:p.L53F,GNS:ENST00000542058.1:exon1:c.C157T:p.L53F,	12q14.3	.
12	69208398	C	T	CTTGGGCATC,CTGGATCCCA	exonic	MDM2	nonsynonymous SNV	MDM2:ENST00000393417.3:exon4:c.C172T:p.P58S,	12q15	.
12	70946612	C	T	TGAGTGGGAT,GCATTTTCCA	exonic	PTPRB	nonsynonymous SNV	PTPRB:ENST00000550857.1:exon18:c.G4408A:p.D1470N,PTPRB:ENST00000261266.5:exon19:c.G4678A:p.D1560N,PTPRB:ENST00000334414.6:exon21:c.G5332A:p.D1778N,PTPRB:ENST00000451516.2:exon18:c.G4408A:p.D1470N,PTPRB:ENST00000538708.1:exon18:c.G4408A:p.D1470N,PTPRB:ENST000(	12q15	.
12	81239637	C	T	ACATTAAAAC,AAGGCCTTCA	exonic	LIN7A	nonsynonymous SNV	LIN7A:ENST00000549417.1:exon4:c.G337A:p.G113S,LIN7A:ENST00000552864.1:exon4:c.G355A:p.G119S,	12q21.31	.
12	97306594	G	A	CTTGCTGAAG,GGTAAGTGAT	exonic	NEDD1	nonsynonymous SNV	NEDD1:ENST00000554226.1:exon4:c.G251A:p.G84E,NEDD1:ENST00000557478.1:exon3:c.G230A:p.G77E,NEDD1:ENST00000429527.2:exon3:c.G230A:p.G77E,NEDD1:ENST00000557092.1:exon5:c.G230A:p.G77E,NEDD1:ENST00000557644.1:exon3:c.G251A:p.G84E,NEDD1:ENST00000266742.4:exon4:c.G2:	12q23.1	.
12	100774486	G	A	TAGAAAAATC,ATGGGACAAC	exonic	SLC17A8	nonsynonymous SNV	SLC17A8:ENST00000323346.5:exon2:c.G109A:p.D37N,SLC17A8:ENST00000392989.3:exon2:c.G109A:p.D37N,	12q23.1	SI
12	100774630	G	A	GAGTGGGCTG,GATTCTGCAT	exonic	SLC17A8	nonsynonymous SNV	SLC17A8:ENST00000323346.5:exon2:c.G253A:p.G85R,SLC17A8:ENST00000392989.3:exon2:c.G253A:p.G85R,	12q23.1	.
12	102161907	G	A	GCAGCCCTCG,CACAGTTTGG	exonic	GNPTAB	nonsynonymous SNV	GNPTAB:ENST00000299314.7:exon11:c.C1316T:p.A439V,GNPTAB:ENST00000549940.1:exon11:c.C1316T:p.A439V,	12q23.2	.
12	104149197	A	G	CTATGGACCT,GACCCAACAA	exonic	STAB2	nonsynonymous SNV	STAB2:ENST00000388887.2:exon62:c.A6832G:p.R2278G,	12q23.3	.
12	106827548	G	A	AATACATTTC,ACTCATGAGA	exonic	POLR3B	nonsynonymous SNV	POLR3B:ENST00000228347.4:exon16:c.G1679A:p.R560Q,POLR3B:ENST00000539066.1:exon16:c.G1505A:p.R502Q,	12q23.3	P(
12	107937726	C	T	CCTGGTGTCC,GTGCAATGCA	exonic	BTBD11	nonsynonymous SNV	BTBD11:ENST00000420571.2:exon3:c.C1300T:p.R434C,BTBD11:ENST00000490090.2:exon3:c.C1300T:p.R434C,BTBD11:ENST00000550706.1:exon2:c.C193T:p.R65C,BTBD11:ENST00000280758.5:exon3:c.C1300T:p.R434C,BTBD11:ENST00000415943.2:exon3:c.C202T:p.R68C,	12q23.3	.
12	108102701	A	T	TACTCTTTCT,GGCCAGTACA	splicing	PWP1(ENST(		.	12q23.3	.
12	112513577	C	G	GACTCTGAAT,TCACTTGTCA	exonic	NAA25	nonsynonymous SNV	NAA25:ENST00000261745.4:exon8:c.G681C:p.E227D,	12q24.13	.
12	112632854	C	T	AGCGCAGCCT,GCAGCGCAGC	exonic	HECTD4	nonsynonymous SNV	HECTD4:ENST00000430131.2:exon55:c.G7318A:p.E2440K,HECTD4:ENST00000550722.1:exon56:c.G8146A:p.E2716K,HECTD4:ENST00000377560.5:exon55:c.G8068A:p.E2690K,	12q24.13	.
12	115109953	G	A	GATGGAGTAG,GGCTGTAGCG	exonic	TBX3	nonsynonymous SNV	TBX3:ENST00000349155.2:exon7:c.C1865T:p.P622L,TBX3:ENST00000257566.3:exon8:c.C1925T:p.P642L,	12q24.21	.
12	118198864	G	A	GAACTCGTGG,ATTTGCTCCG	exonic	KSR2	nonsynonymous SNV	KSR2:ENST00000339824.5:exon4:c.C938T:p.S313F,KSR2:ENST00000425217.1:exon4:c.C851T:p.S284F,	12q24.23	.
12	118481045	C	T	GAGCTTCCTG,TGGGTGGGGA	exonic	WSB2	nonsynonymous SNV	WSB2:ENST00000535496.1:exon2:c.G326A:p.S109N,WSB2:ENST00000315436.3:exon3:c.G320A:p.S107N,WSB2:ENST00000441406.2:exon3:c.G371A:p.S124N,WSB2:ENST00000537945.1:exon3:c.G326A:p.S109N,	12q24.23	.
12	120569817	G	A	GAGGAGATGC,AGTGTTGTCC	exonic	GCN1L1	nonsynonymous SNV	GCN1L1:ENST00000300648.6:exon54:c.C7327T:p.R2443C,	12q24.23	.
12	120574506	C	T	AGGACTCCTT,CCGCAACACT	exonic	GCN1L1	nonsynonymous SNV	GCN1L1:ENST00000300648.6:exon51:c.G6808A:p.E2270K,	12q24.23	.
12	120741544	G	C	TCCTTGTGAT,ACTGGGGCAG	exonic	SIRT4	nonsynonymous SNV	SIRT4:ENST00000202967.4:exon2:c.G180C:p.M60I,SIRT4:ENST00000536460.1:exon2:c.G3C:p.M1I,	12q24.31	.
12	122361820	C	T	GCAGGGATCT,CCGGGAGTCA	exonic	WDR66	nonsynonymous SNV	WDR66:ENST00000288912.4:exon3:c.C671T:p.S224F,WDR66:ENST00000397454.2:exon3:c.C671T:p.S224F,	12q24.31	.
12	122399936	T	G	GTCCTGGACA,TCACCACACC	exonic	WDR66	nonsynonymous SNV	WDR66:ENST00000288912.4:exon15:c.T2360G:p.I787S,WDR66:ENST00000397454.2:exon15:c.T2360G:p.I787S,	12q24.31	.
12	122958193	G	A	TCAGGTATAG,GCTATGAATT	exonic	ZCCHC8	nonsynonymous SNV	ZCCHC8:ENST00000543897.1:exon12:c.C1261T:p.P421S,ZCCHC8:ENST00000536306.1:exon12:c.C1261T:p.P421S,ZCCHC8:ENST00000336229.4:exon14:c.C1975T:p.P659S,ZCCHC8:ENST00000538116.1:exon3:c.C808T:p.P270S,	12q24.31	.
12	123065212	T	G	GGTTGCTAGC,TACAGGTAAA	exonic	KNTC1	nonsynonymous SNV	KNTC1:ENST00000333479.7:exon33:c.T2977G:p.L993V,	12q24.31	.
12	123068964	C	G	GATACATGAT,TAGCAAGCCA	exonic	KNTC1	nonsynonymous SNV	KNTC1:ENST00000333479.7:exon35:c.C3403G:p.L1135V,	12q24.31	.
12	123444346	G	A	TGGCTCCAGG,CCTGGGTGCC	exonic	ABCB9	nonsynonymous SNV	ABCB9:ENST00000542678.1:exon2:c.C437T:p.A146V,ABCB9:ENST00000346530.5:exon2:c.C437T:p.A146V,ABCB9:ENST00000540285.1:exon2:c.C437T:p.A146V,ABCB9:ENST00000442833.2:exon2:c.C437T:p.A146V,ABCB9:ENST00000537276.1:exon2:c.C437T:p.A146V,ABCB9:ENST00000392439.3:exon2:c.C	12q24.31	.
12	124270360	G	A	AGTGCCCTGC,GATGGTGTGG	exonic	DNAH10	nonsynonymous SNV	DNAH10:ENST00000409039.3:exon9:c.G1115A:p.R372Q,	12q24.31	.
12	128899435	G	A	GGCGAGGGTG,AGTCCTTCTT	exonic	TMEM132C	nonsynonymous SNV	TMEM132C:ENST00000435159.2:exon2:c.G244A:p.E82K,	12q24.32	.
12	132281833	C	T	CCCGCGGCCC,CGCGGCCCAC	exonic	SFSWAP	nonsynonymous SNV	SFSWAP:ENST00000541286.1:exon17:c.C2801T:p.P934L,SFSWAP:ENST00000261674.4:exon16:c.C2645T:p.P882L,	12q24.33	.
12	132529230	A	T	ATGTCTACGA,GATGTCGATG	exonic	EP400	nonsynonymous SNV	EP400:ENST00000332482.4:exon35:c.A6489T:p.E2163D,EP400:ENST00000389561.2:exon36:c.A6600T:p.E2200D,EP400:ENST00000333577.4:exon37:c.A6708T:p.E2236D,EP400:ENST00000330386.6:exon35:c.A6357T:p.E2119D,EP400:ENST00000389562.2:exon36:c.A6597T:p.E2199D,	12q24.33	.
12	132683723	G	A	TCCTACCTGG,AGGACATCCC	exonic	GALNT9	nonsynonymous SNV	GALNT9:ENST00000397325.2:exon5:c.C395T:p.S132F,GALNT9:ENST00000328957.8:exon9:c.C1493T:p.S498F,GALNT9:ENST00000535228.1:exon6:c.C746T:p.S249F,GALNT9:ENST00000541995.1:exon4:c.C395T:p.S132F,	12q24.33	.
12	133149173	C	T	ACACCCCGGC,CCTTCGGGTC	exonic	FBRSL1	nonsynonymous SNV	FBRSL1:ENST00000261673.6:exon12:c.C1330T:p.P444S,FBRSL1:ENST00000434748.2:exon11:c.C1549T:p.P517S,	12q24.33	.
12	133732125	C	T	TCATCAGTTT,CAGCAGGAGC	exonic	CTD-2140B24	nonsynonymous SNV	ZNF10:ENST00000426665.2:exon5:c.C293T:p.S98F,ZNF10:ENST00000402932.2:exon4:c.C293T:p.S98F,ZNF10:ENST00000537119.1:exon3:c.C167T:p.S56F,ZNF10:ENST00000248211.6:exon5:c.C293T:p.S98F,ZNF268:ENST00000416488.1:exon5:c.C293T:p.S98F,CTD-2140B24.4:ENST00000540096.2:exon5:c.(	12q24.33	.
13	24465918	G	A	CTCACCCTGG,GCCCCGTGGG	exonic	C1QTNF9B	nonsynonymous SNV	C1QTNF9B:ENST00000382140.2:exon5:c.C512T:p.P171L,C1QTNF9B:ENST00000382137.3:exon3:c.C512T:p.P171L,	13q12.12	.
13	25367472	G	A	AATTATTGAA,ACAACGTGGA	exonic	RNF17	nonsynonymous SNV	RNF17:ENST00000255324.5:exon10:c.G1228A:p.D410N,RNF17:ENST00000381921.1:exon10:c.G1228A:p.D410N,RNF17:ENST00000255325.6:exon10:c.G1228A:p.D410N,	13q12.12	.
13	25671298	C	G	GGTACAATCA,TAGTGCAAAG	exonic	PABPC3	nonsynonymous SNV	PABPC3:ENST00000281589.3:exon1:c.C962G:p.T321S,	13q12.13	.
13	25671309	G	T	TAGTGCAAAG,TTATGATGGA	exonic	PABPC3	nonsynonymous SNV	PABPC3:ENST00000281589.3:exon1:c.G973T:p.V325F,	13q12.13	.
13	43930154	G	A	AGCTTGCGCC,GTGCCGCTCC	exonic	ENOX1	nonsynonymous SNV	ENOX1:ENST00000261488.6:exon8:c.C724T:p.R242W,ENOX1:ENST00000540032.1:exon3:c.C163T:p.R55W,ENOX1:ENST00000412891.1:exon7:c.C724T:p.R242W,	13q14.11	EI
13	46658361	G	C	TACCTGCATG,AATTCCGCTC	exonic	CPB2	nonsynonymous SNV	CPB2:ENST00000181383.4:exon3:c.C268G:p.P90A,CPB2:ENST00000439329.3:exon3:c.C268G:p.P90A,	13q14.13	.
13	58207623	G	A	TGAGGAAAAC,GGATGCTGGA	exonic	PCDH17	nonsynonymous SNV	PCDH17:ENST00000377918.3:exon1:c.G943A:p.G315R,PCDH17:ENST00000484979.1:exon1:c.G943A:p.G315R,	13q21.1	.
13	60545242	C	T	GCCAGTTCCA,CTTCTTTAGA	exonic	DIAPH3	nonsynonymous SNV	DIAPH3:ENST00000377908.2:exon15:c.G1670A:p.G557D,DIAPH3:ENST00000400324.4:exon16:c.G1703A:p.G568D,DIAPH3:ENST00000267215.4:exon16:c.G1703A:p.G568D,DIAPH3:ENST00000400320.1:exon14:c.G1565A:p.G522D,DIAPH3:ENST00000400330.1:exon16:c.G1703A:p.G568D,DIAPH3:ENST00000	13q21.2	.
13	95121248	G	A	CTCGCAGTTG,GACCGGTCCA	exonic	DCT	nonsynonymous SNV	DCT:ENST00000446125.1:exon2:c.C347T:p.P116L,DCT:ENST00000377028.5:exon2:c.C347T:p.P116L,	13q32.1	.
13	98829229	C	T	TCGAGGCTCT,AGGCGCCGCC	exonic	RNF113B	nonsynonymous SNV	RNF113B:ENST00000267291.6:exon1:c.G262A:p.E88K,	13q32.2	.
13	99064230	C	T	GAATTTTGAA,CTTTGCACAA	exonic	FARP1	nonsynonymous SNV	FARP1:ENST00000319562.6:exon16:c.C1768T:p.P590S,FARP1:ENST00000595437.1:exon16:c.C1768T:p.P590S,FARP1:ENST00000376586.2:exon16:c.C1768T:p.P590S,	13q32.2	.
13	99449395	C	T	TCAGAAAGCT,CTTCGCCATT	exonic	DOCK9	nonsynonymous SNV	DOCK9:ENST00000376460.1:exon55:c.G6172A:p.E2058K,DOCK9:ENST00000339416.2:exon53:c.G6133A:p.E2045K,	13q32.3	.
13	103384533	G	A	TGGAATTTAG,AAGACAGATT	exonic	CCDC168	nonsynonymous SNV	CCDC168:ENST00000322527.2:exon1:c.C4627T:p.P1543S,	13q33.1	.
13	110839523	C	T	GGATGGCCAT,TCTTCCAGGA	exonic	COL4A1	nonsynonymous SNV	COL4A1:ENST00000375820.4:exon25:c.G1690A:p.D564N,	13q34	.
13	110845249	C	T	AGGCAACTCT,TCCTTTTTGA	exonic	COL4A1	nonsynonymous SNV	COL4A1:ENST00000543140.1:exon23:c.G1393A:p.E465K,COL4A1:ENST00000375820.4:exon23:c.G1393A:p.E465K,	13q34	.
13	111532159	G	A	TTCCACCCAG,AAGGCCTACT	exonic	ANKRD10	nonsynonymous SNV	ANKRD10:ENST00000267339.2:exon6:c.C1088T:p.S363F,	13q34	.
13	111927972	C	G	CATTAAAACT,TGGGCAACGT	exonic	ARHGEF7	nonsynonymous SNV	ARHGEF7:ENST00000375739.2:exon11:c.C1279G:p.L427V,ARHGEF7:ENST00000375723.1:exon10:c.C895G:p.L299V,ARHGEF7:ENST00000375741.2:exon13:c.C1429G:p.L477V,ARHGEF7:ENST00000544132.1:exon10:c.C397G:p.L133V,ARHGEF7:ENST00000218789.5:exon11:c.C895G:p.L299V,ARHGEF7:EN	13q34	.
13	112722391	G	A	CTGGCCGGCG,GCTCCTGGCG	exonic	SOX1	nonsynonymous SNV	SOX1:ENST00000330949.1:exon1:c.G419A:p.G140E,	13q34	.
13	113731520	C	T	CCCTGCCCCT,CCCAGGTCTG	exonic	MCF2L	nonsynonymous SNV	MCF2L:ENST00000434480.2:exon13:c.C1673T:p.S558F,MCF2L:ENST00000442652.2:exon13:c.C1745T:p.S582F,MCF2L:ENST00000535094.2:exon13:c.C1655T:p.S552F,MCF2L:ENST00000397030.1:exon13:c.C1754T:p.S585F,MCF2L:ENST00000375601.3:exon13:c.C1667T:p.S556F,MCF2L:ENST00000421756.	13q34	.
14	20863724	G	A	CGCCTGGGAC,GTTCTTTTCA	exonic	TEP1	nonsynonymous SNV	TEP1:ENST00000556935.1:exon10:c.C1489T:p.R497C,TEP1:ENST00000262715.5:exon12:c.C1813T:p.R605C,TEP1:ENST00000555727.1:exon12:c.C1813T:p.R605C,	14q11.2	TE
14	21025105	C	T	TCAAATTCTT,TTTTTTATCT	exonic	RNASE9	nonsynonymous SNV	RNASE9:ENST00000429244.2:exon3:c.G124A:p.E42K,RNASE9:ENST00000553706.1:exon5:c.G139A:p.E47K,RNASE9:ENST00000554964.1:exon3:c.G124A:p.E42K,RNASE9:ENST00000553541.1:exon4:c.G124A:p.E42K,RNASE9:ENST00000338904.3:exon2:c.G124A:p.E42K,RNASE9:ENST00000556208.1:exon	14q11.2	.
14	21936902	C	T	CGGAAGGATT,TTGCCCAGCC	exonic	RAB2B	nonsynonymous SNV	RAB2B:ENST00000397762.1:exon4:c.G196A:p.E66K,	14q11.2	.
14	22038020	G	A	TAGATAAGGG,GTTGAGGAAA	exonic	OR10G3	nonsynonymous SNV	OR10G3:ENST00000303532.1:exon1:c.C856T:p.P286S,	14q11.2	.
14	22251223	G	A	GAGAAGATGC,GAGACCTGTC	exonic	TRAV7	nonsynonymous SNV	TRAV7:ENST00000390429.3:exon1:c.G14A:p.R5Q,	14q11.2	.
14	22476351	C	T	TCCACCAGTT,CTTCAACTTC	exonic	TRAV19	nonsynonymous SNV	TRAV19:ENST00000390447.3:exon2:c.C281T:p.S94F,	14q11.2	.
14	23588246	C	G	CCTGAGAACT,GAGTGGCTGC	exonic	CEBPE	nonsynonymous SNV	CEBPE:ENST00000206513.5:exon1:c.G55C:p.E19Q,	14q11.2	.
14	23828143	G	A	GGCCTGATCC,GGGGCCTAGA	exonic	EFS	nonsynonymous SNV	EFS:ENST00000429593.2:exon5:c.C686T:p.P229L,EFS:ENST00000351354.3:exon4:c.C914T:p.P305L,EFS:ENST00000216733.3:exon5:c.C1193T:p.P398L,	14q11.2	.
14	23828144	G	A	GCCTGATCCG,GGGCCTAGAT	exonic	EFS	nonsynonymous SNV	EFS:ENST00000429593.2:exon5:c.C685T:p.P229S,EFS:ENST00000351354.3:exon4:c.C913T:p.P305S,EFS:ENST00000216733.3:exon5:c.C1192T:p.P398S,	14q11.2	.
14	23829128	G	A	ACATCATAGG,AGCATCATCC	exonic	EFS	nonsynonymous SNV	EFS:ENST00000351354.3:exon3:c.C280T:p.P94S,EFS:ENST00000216733.3:exon4:c.C559T:p.P187S,	14q11.2	.
14	23862616	C	T	TCTTCCTCAA,CTGAAGGTCA	exonic	MYH6	nonsynonymous SNV	MYH6:ENST00000356287.3:exon22:c.G3040A:p.V1014I,MYH6:ENST00000405093.3:exon23:c.G3040A:p.V1014I,	14q11.2	.
14	23886203	T	G	AGATCTCCTC,GTGTGGGGAA	splicing	MYH7(ENST(		.	14q11.2	.
14	23886518	C	T	TGCTTCCACT,GGCCAGGATC	exonic	MYH7	nonsynonymous SNV	MYH7:ENST00000355349.3:exon32:c.G4363A:p.E1455K,	14q11.2	M
14	23992051	C	T	GGGCATGGGT,TCTGAGGTAA	exonic	ZFHX2	nonsynonymous SNV	ZFHX2:ENST00000419474.3:exon10:c.G6839A:p.R2280K,	14q11.2	.
14	24802184	G	A	GCTCGGGTCT,AGGTCAGCTC	exonic	ADCY4,RP11	nonsynonymous SNV	ADCY4:ENST00000310677.4:exon3:c.C170T:p.S57L,ADCY4:ENST00000554781.1:exon2:c.C170T:p.S57L,ADCY4:ENST00000561138.1:exon3:c.C170T:p.S57L,ADCY4:ENST00000554068.2:exon3:c.C170T:p.S57L,ADCY4:ENST00000418030.2:exon2:c.C170T:p.S57L,	14q12	.
14	33290899	G	C	CCTCCACAAT,TTGAACTCTA	exonic	AKAP6	nonsynonymous SNV	AKAP6:ENST00000280979.4:exon13:c.G3880C:p.V1294L,	14q12	.
14	33291816	G	A	TAGAAAGTTG,TTGACTTCCT	exonic	AKAP6	stopgain	AKAP6:ENST00000280979.4:exon13:c.G4797A:p.W1599X,	14q12	.
14	45658543	G	A	AAAGACTGTA,AAAATTTCCA	exonic	FANCM	nonsynonymous SNV	FANCM:ENST00000542564.2:exon19:c.G5240A:p.R1747K,FANCM:ENST00000267430.5:exon20:c.G5318A:p.R1773K,FANCM:ENST00000556250.1:exon13:c.G3866A:p.R1289K,	14q21.2	.
14	55227182	C	T	GGGGGACCTC,CCGGGCAGTT	exonic	SAMD4A	nonsynonymous SNV	SAMD4A:ENST00000555192.1:exon2:c.C253T:p.P85S,SAMD4A:ENST00000251091.5:exon5:c.C1216T:p.P406S,SAMD4A:ENST00000357634.3:exon6:c.C1477T:p.P493S,SAMD4A:ENST00000554335.1:exon7:c.C1480T:p.P494S,SAMD4A:ENST00000392067.3:exon6:c.C1480T:p.P494S,	14q22.2	.
14	58604806	G	A	TTCCTTGGTG,ATTCCGTGAA	exonic	C14orf37	nonsynonymous SNV	C14orf37:ENST00000267485.7:exon2:c.C1271T:p.S424F,	14q23.1	.
14	58714471	G	C	CCTTAAGTAT,ACCTGTCAGC	exonic	PSMA3	nonsynonymous SNV	PSMA3:ENST00000555931.1:exon2:c.G25C:p.D9H,PSMA3:ENST00000216455.4:exon2:c.G25C:p.D9H,PSMA3:ENST00000412908.2:exon2:c.G25C:p.D9H,PSMA3:ENST00000557087.1:exon2:c.G25C:p.D9H,	14q23.1	.
14	60470375	G	A	ACATCTTTTT,AAATCACAAA	exonic	LRRC9	nonsynonymous SNV	LRRC9:ENST00000445360.1:exon21:c.G2701A:p.E901K,LRRC9:ENST00000254271.4:exon21:c.G2701A:p.E901K,	14q23.1	.
14	64464092	C	T	ATCAGATAAT,AGCCATCAAC	exonic	SYNE2	stopgain	SYNE2:ENST00000554584.1:exon24:c.C3226T:p.Q1076X,SYNE2:ENST00000344113.4:exon25:c.C3226T:p.Q1076X,SYNE2:ENST00000358025.3:exon25:c.C3226T:p.Q1076X,	14q23.2	.
14	67646374	C	T	GAACCACTAC,TTGGGCACAG	exonic	GPHN	nonsynonymous SNV	GPHN:ENST00000305960.9:exon20:c.C1967T:p.P656L,GPHN:ENST00000478722.1:exon22:c.C2159T:p.P720L,GPHN:ENST00000543237.1:exon24:c.C2198T:p.P733L,GPHN:ENST00000315266.5:exon21:c.C2060T:p.P687L,	14q23.3	.
14	68157131	G	A	GGGTTAGGAG,AAGTGACCTG	exonic	RDH11	nonsynonymous SNV	RDH11:ENST00000554731.1:exon2:c.C82T:p.P28S,	14q24.1	.
14	70245071	C	T	TTGAATTTCT,ATAGCACCAA	exonic	SLC10A1	nonsynonymous SNV	SLC10A1:ENST00000216540.4:exon4:c.G922A:p.E308K,	14q24.2	SI
14	70634248	C	T	AGGTTCCCAT,TAGAAAATGG	exonic	SLC8A3	nonsynonymous SNV	SLC8A3:ENST00000494208.1:exon2:c.G892A:p.D298N,SLC8A3:ENST00000528359.1:exon1:c.G892A:p.D298N,SLC8A3:ENST00000381269.2:exon2:c.G892A:p.D298N,SLC8A3:ENST00000357887.3:exon2:c.G892A:p.D298N,SLC8A3:ENST00000534137.1:exon1:c.G892A:p.D298N,SLC8A3:ENST00000356921.2:e	14q24.2	.
14	72190584	G	A	AAACTCCGTG,GATTTATGGA	exonic	SIPA1L1	nonsynonymous SNV	SIPA1L1:ENST00000358550.2:exon15:c.G4429A:p.G1477R,SIPA1L1:ENST00000381232.3:exon15:c.G4429A:p.G1477R,SIPA1L1:ENST00000537413.1:exon14:c.G2854A:p.G952R,SIPA1L1:ENST00000555818.1:exon16:c.G4492A:p.G1498R,	14q24.2	.
14	72190585	G	A	AACTCCGTGG,ATTTATGGAC	exonic	SIPA1L1	nonsynonymous SNV	SIPA1L1:ENST00000358550.2:exon15:c.G4430A:p.G1477E,SIPA1L1:ENST00000381232.3:exon15:c.G4430A:p.G1477E,SIPA1L1:ENST00000537413.1:exon14:c.G2855A:p.G952E,SIPA1L1:ENST00000555818.1:exon16:c.G4493A:p.G1498E,	14q24.2	.
14	73441568	G	A	GGCACTGGCC,AGTCTTTGAT	exonic	ZFYVE1	nonsynonymous SNV	ZFYVE1:ENST00000394207.2:exon7:c.C661T:p.R221W,ZFYVE1:ENST00000318876.5:exon10:c.C1864T:p.R622W,ZFYVE1:ENST00000556143.1:exon10:c.C1906T:p.R636W,ZFYVE1:ENST00000553891.1:exon10:c.C1906T:p.R636W,ZFYVE1:ENST00000555072.1:exon7:c.C661T:p.R221W,	14q24.2	.
14	74824966	C	T	CCCTCCCGCC,CCGGGGAGCT	exonic	VRTN	nonsynonymous SNV	VRTN:ENST00000256362.4:exon2:c.C1480T:p.P494S,	14q24.3	.
14	75643068	C	T	CTTGAGGTGG,TGCGCAGGCC	exonic	TMED10	nonsynonymous SNV	TMED10:ENST00000303575.4:exon1:c.G215A:p.S72N,TMED10:ENST00000555873.1:exon1:c.G215A:p.S72N,	14q24.3	.
14	76101251	A	C	TGGTTTCCCC,GGGGGAAGAA	splicing	FLVCR2(ENS		.	14q24.3	.
14	76639940	G	A	CGATGCCTCA,AAAGGGGCGA	exonic	GPATCH2L	nonsynonymous SNV	GPATCH2L:ENST00000557263.1:exon5:c.G956A:p.R319K,GPATCH2L:ENST00000261530.7:exon5:c.G956A:p.R319K,GPATCH2L:ENST00000554375.1:exon5:c.G956A:p.R319K,GPATCH2L:ENST00000556663.1:exon4:c.G956A:p.R319K,GPATCH2L:ENST00000312858.5:exon5:c.G956A:p.R319K,	14q24.3	.
14	77492569	C	T	GCCCCGGTCC,CGGGGGTGCG	exonic	IRF2BPL	nonsynonymous SNV	IRF2BPL:ENST00000238647.3:exon1:c.G1567A:p.G523R,	14q24.3	.
14	89878765	G	A	CAGTCCACTG,AGACACTAAT	exonic	FOXN3	nonsynonymous SNV	FOXN3:ENST00000555353.1:exon2:c.C56T:p.S19F,FOXN3:ENST00000557258.1:exon2:c.C56T:p.S19F,FOXN3:ENST00000261302.5:exon1:c.C56T:p.S19F,FOXN3:ENST00000555855.1:exon3:c.C56T:p.S19F,FOXN3:ENST00000553904.1:exon3:c.C56T:p.S19F,FOXN3:ENST00000345097.4:exon2:c.C56T:p.S19F,	14q32.11	.
14	92264187	C	T	TGTAAGTATT,CTTTTATAGA	exonic	TC2N	nonsynonymous SNV	TC2N:ENST00000435962.2:exon8:c.G797A:p.G266E,TC2N:ENST00000340892.5:exon8:c.G797A:p.G266E,TC2N:ENST00000556018.1:exon8:c.G797A:p.G266E,TC2N:ENST00000360594.5:exon8:c.G797A:p.G266E,	14q32.12	.
14	100141514	C	T	CACGGCCGCT,CCCCCGCGCC	exonic	HHIPL1	nonsynonymous SNV	HHIPL1:ENST00000330710.5:exon9:c.C1900T:p.P634S,	14q32.2	.
14	102807819	C	T	GCTGCTCTCT,ATCCCGGGAT	exonic	ZNF839	nonsynonymous SNV	ZNF839:ENST00000558850.1:exon8:c.C1739T:p.S580L,ZNF839:ENST00000442396.2:exon8:c.C2087T:p.S696L,ZNF839:ENST00000561251.1:exon6:c.C731T:p.S244L,ZNF839:ENST00000559185.1:exon8:c.C1739T:p.S580L,ZNF839:ENST00000262236.5:exon8:c.C1745T:p.S582L,	14q32.31	.
14	105353166	C	T	CCCAGCCTTT,TCCGGCAAGA	exonic	CEP170B	nonsynonymous SNV	CEP170B:ENST00000418279.1:exon11:c.C2380T:p.L794F,CEP170B:ENST00000453495.1:exon12:c.C2593T:p.L865F,CEP170B:ENST00000556508.1:exon11:c.C2380T:p.L794F,CEP170B:ENST00000414716.3:exon12:c.C2590T:p.L864F,	14q32.33	.
14	105393477	G	C	GCCCTGTGCA,ATGCTGAAGC	exonic;splicing	PLD4;PLD4	nonsynonymous SNV	PLD4:ENST00000540372.1:exon2:c.G21C:p.Q7H,	14q32.33	.
14	105409199	C	T	TTCACGTCCA,TTGGCCAGCC	exonic	AHNAK2	nonsynonymous SNV	AHNAK2:ENST00000333244.5:exon7:c.G12589A:p.V4197M,	14q32.33	.
14	105410651	C	T	CCGGCTCCCT,GGGCACCTGG	exonic	AHNAK2	nonsynonymous SNV	AHNAK2:ENST00000333244.5:exon7:c.G11137A:p.E3713K,	14q32.33	.
14	105410657	C	G	CCCTCGGGCA,CTGGCCCTCC	exonic	AHNAK2	nonsynonymous SNV	AHNAK2:ENST00000333244.5:exon7:c.G11131C:p.V3711L,	14q32.33	.
14	105410658	C	G	CCTCGGGCAC,TGGCCCTCCG	exonic	AHNAK2	nonsynonymous SNV	AHNAK2:ENST00000333244.5:exon7:c.G11130C:p.Q3710H,	14q32.33	.
14	105413946	C	T	CGTCCACCTC,ATGCTGGACA	exonic	AHNAK2	nonsynonymous SNV	AHNAK2:ENST00000333244.5:exon7:c.G7842A:p.M2614I,	14q32.33	.
14	105416680	G	A	GAGGTCAGTG,TCTTCAGGTC	exonic	AHNAK2	nonsynonymous SNV	AHNAK2:ENST00000333244.5:exon7:c.C5108T:p.T1703I,	14q32.33	.
14	105517438	C	T	GACTGCAGCT,CTCGGTGTCC	exonic	GPR132	nonsynonymous SNV	GPR132:ENST00000539291.2:exon5:c.G1036A:p.E346K,GPR132:ENST00000392585.2:exon3:c.G1009A:p.E337K,GPR132:ENST00000329797.3:exon4:c.G1036A:p.E346K,	14q32.33	.
14	106586378	G	A	GAGTCTCAGG,ACCCCCCAGG	exonic	IGHV3-13	nonsynonymous SNV	IGHV3-13:ENST00000390602.2:exon2:c.C107T:p.S36F,	14q32.33	.
14	106963178	G	A	CTTCACTGAG,ACCCAGTCTT	exonic	IGHV1-45	nonsynonymous SNV	IGHV1-45:ENST00000390621.2:exon2:c.C104T:p.S35F,	14q32.33	.
15	22841080	G	A	CTTGATGGAA,ATGAAGAAAT	exonic	TUBGCP5	nonsynonymous SNV	TUBGCP5:ENST00000283645.4:exon5:c.G448A:p.D150N,TUBGCP5:ENST00000453949.2:exon5:c.G448A:p.D150N,	15q11.2	.
15	22851077	T	A	CATTCTTGAA,ATGACAATGT	exonic	TUBGCP5	nonsynonymous SNV	TUBGCP5:ENST00000283645.4:exon11:c.T1339A:p.Y447N,TUBGCP5:ENST00000453949.2:exon11:c.T1339A:p.Y447N,	15q11.2	.
15	23685853	C	T	ATTCTCTCCT,CTTCTCCCGC	exonic	GOLGA6L2	nonsynonymous SNV	GOLGA6L2:ENST00000567107.1:exon8:c.G1769A:p.G590E,	15q11.2	.
15	24921274	C	T	GTCCTGCCGG,TGTGGGTTGG	exonic	NPAP1	nonsynonymous SNV	NPAP1:ENST00000329468.2:exon1:c.C260T:p.A87V,	15q11.2	.
15	25926052	C	T	ACGTTCGAGT,ATAGTAGGCC	exonic	ATP10A	nonsynonymous SNV	ATP10A:ENST00000356865.6:exon19:c.G3583A:p.D1195N,	15q12	.
15	27193172	C	T	GAACAGACCC,AGCAGGGACG	exonic	GABRA5	nonsynonymous SNV	GABRA5:ENST00000400081.3:exon11:c.C1181T:p.P394L,GABRA5:ENST00000335625.5:exon11:c.C1181T:p.P394L,GABRA5:ENST00000355395.5:exon10:c.C1181T:p.P394L,	15q12	.
15	27772753	C	T	AGAAAACCAA,CACCACGAAG	exonic	GABRG3	nonsynonymous SNV	GABRG3:ENST00000333743.6:exon8:c.C1040T:p.T347I,	15q12	.
15	33261219	G	A	CTCACAGGTG,CATTGGAGGT	exonic	FMN1	nonsynonymous SNV	FMN1:ENST00000561249.1:exon3:c.C2389T:p.P797S,FMN1:ENST00000334528.9:exon4:c.C2014T:p.P672S,FMN1:ENST00000559047.1:exon5:c.C2683T:p.P895S,	15q13.3	.
15	34649232	G	A	AGGGGAACCA,GAATGCCATA	exonic	NUTM1	nonsynonymous SNV	NUTM1:ENST00000438749.3:exon7:c.G2993A:p.R998K,NUTM1:ENST00000333756.4:exon7:c.G2939A:p.R980K,NUTM1:ENST00000537011.1:exon8:c.G3023A:p.R1008K,	15q14	.
15	40564525	C	T	ACCTTCAGCC,TCTGACCACT	exonic	PAK6	nonsynonymous SNV	PAK6:ENST00000560346.1:exon6:c.C959T:p.P320L,PAK6:ENST00000260404.4:exon6:c.C959T:p.P320L,PAK6:ENST00000455577.2:exon6:c.C959T:p.P320L,PAK6:ENST00000441369.1:exon6:c.C959T:p.P320L,PAK6:ENST00000453867.1:exon5:c.C959T:p.P320L,PAK6:ENST00000542403.2:exon4:c.C959T:p.P3:	15q15.1	.
15	40590492	C	T	GGGGGTTTCC,CTTCCAGCAG	exonic	PLCB2	nonsynonymous SNV	PLCB2:ENST00000557821.1:exon11:c.G1087A:p.G363R,PLCB2:ENST00000558588.1:exon11:c.G1087A:p.G363R,PLCB2:ENST00000260402.3:exon11:c.G1087A:p.G363R,PLCB2:ENST00000456256.2:exon11:c.G1087A:p.G363R,	15q15.1	.
15	41275129	C	T	GCTTTGGCTC,TGCCATTGCA	exonic	INO80	nonsynonymous SNV	INO80:ENST00000361937.3:exon35:c.G4384A:p.G1462R,INO80:ENST00000401393.3:exon35:c.G4384A:p.G1462R,	15q15.1	.
15	41991104	C	T	ACAGAAGAAT,TTCTAGTCTC	exonic	MGA	nonsynonymous SNV	MGA:ENST00000389936.4:exon4:c.C2057T:p.S686F,MGA:ENST00000566586.1:exon3:c.C2057T:p.S686F,MGA:ENST00000570161.1:exon3:c.C2057T:p.S686F,MGA:ENST00000566718.1:exon4:c.C2057T:p.S686F,MGA:ENST00000545763.1:exon4:c.C2057T:p.S686F,MGA:ENST00000219905.7:exon4:c.C2057T:p	15q15.1	.
15	42103404	C	T	TGCCTTCTCC,CTGATGGCAA	exonic	MAPKBP1	nonsynonymous SNV	MAPKBP1:ENST00000457542.2:exon5:c.C298T:p.P100S,MAPKBP1:ENST00000456763.2:exon5:c.C298T:p.P100S,MAPKBP1:ENST00000512970.1:exon5:c.C298T:p.P100S,MAPKBP1:ENST00000221214.6:exon5:c.C298T:p.P100S,MAPKBP1:ENST00000510535.1:exon5:c.C298T:p.P100S,MAPKBP1:ENST00000	15q15.1	.
15	42134398	C	T	CAGGATGCCC,CGAGGAGCAA	exonic	JMJD7-PLA2(	nonsynonymous SNV	PLA2G4B:ENST00000458483.1:exon9:c.C629T:p.P210L,PLA2G4B:ENST00000542534.2:exon14:c.C1322T:p.P441L,JMJD7-PLA2G4B:ENST00000342159.4:exon14:c.C1322T:p.P441L,JMJD7-PLA2G4B:ENST00000382448.4:exon14:c.C1322T:p.P441L,PLA2G4B:ENST00000452633.1:exon10:c.C629T:p.P210L,	15q15.1	.
15	42139604	G	A	GCCCAGCCCC,AAGAGCAGCT	exonic	JMJD7-PLA2(	nonsynonymous SNV	PLA2G4B:ENST00000458483.1:exon19:c.G2017A:p.E673K,PLA2G4B:ENST00000542534.2:exon24:c.G2710A:p.E904K,JMJD7-PLA2G4B:ENST00000382448.4:exon24:c.G2710A:p.E904K,PLA2G4B:ENST00000452633.1:exon20:c.G2017A:p.E673K,	15q15.1	.
15	42147148	G	C	CTTCCTTTCT,AAAGCATCAA	exonic	SPTBN5	nonsynonymous SNV	SPTBN5:ENST00000320955.6:exon56:c.C9450G:p.F3150L,	15q15.1	.
15	42967897	C	T	ACAGAAGTTC,GATTCAACCA	exonic	STARD9	stopgain	STARD9:ENST00000569419.1:exon5:c.C349T:p.R117X,STARD9:ENST00000290607.7:exon19:c.C1723T:p.R575X,	15q15.2	.
15	42980209	C	T	TCCCCAGGTC,AGAAAATCAC	exonic	STARD9	stopgain	STARD9:ENST00000290607.7:exon23:c.C6433T:p.Q2145X,	15q15.2	.
15	43360184	G	A	GTGGTCATAT,AATGGTGTTC	exonic	UBR1	nonsynonymous SNV	UBR1:ENST00000382177.2:exon6:c.C710T:p.S237L,UBR1:ENST00000290650.4:exon6:c.C710T:p.S237L,	15q15.2	.
15	43738647	C	T	CAGTTTCATT,TTAGACATAA	exonic	TP53BP1	nonsynonymous SNV	TP53BP1:ENST00000413546.1:exon13:c.G2858A:p.R953K,TP53BP1:ENST00000450115.2:exon14:c.G2978A:p.R993K,TP53BP1:ENST00000382039.3:exon14:c.G2978A:p.R993K,TP53BP1:ENST00000263801.3:exon14:c.G2963A:p.R988K,TP53BP1:ENST00000382044.4:exon14:c.G2978A:p.R993K,	15q15.3	.
15	45387702	G	A	GGAGGCAAAG,GGGTGACCCC	exonic	DUOX2	nonsynonymous SNV	DUOX2:ENST00000389039.6:exon31:c.C4172T:p.P1391L,DUOX2:ENST00000603300.1:exon31:c.C4172T:p.P1391L,	15q21.1	.
15	48054475	C	A	GGTGATGGAT,TGCCCTTCGC	exonic	SEMA6D	nonsynonymous SNV	SEMA6D:ENST00000354744.4:exon8:c.C617A:p.S206Y,SEMA6D:ENST00000316364.5:exon8:c.C617A:p.S206Y,SEMA6D:ENST00000358066.4:exon8:c.C617A:p.S206Y,SEMA6D:ENST00000389425.3:exon8:c.C617A:p.S206Y,SEMA6D:ENST00000389428.3:exon8:c.C617A:p.S206Y,SEMA6D:ENST00000559196.	15q21.1	.
15	48063011	A	T	GCTACCACCC,ATGGAGATAC	exonic	SEMA6D	nonsynonymous SNV	SEMA6D:ENST00000354744.4:exon18:c.A2083T:p.N695Y,SEMA6D:ENST00000316364.5:exon19:c.A2251T:p.N751Y,SEMA6D:ENST00000358066.4:exon17:c.A2065T:p.N689Y,SEMA6D:ENST00000389428.3:exon17:c.A2026T:p.N676Y,SEMA6D:ENST00000389433.2:exon18:c.A2194T:p.N732Y,SEMA6D:ENST00	15q21.1	.
15	48063013	T	A	TACCACCCAA,GGAGATACTA	exonic	SEMA6D	nonsynonymous SNV	SEMA6D:ENST00000354744.4:exon18:c.T2085A:p.N695K,SEMA6D:ENST00000316364.5:exon19:c.T2253A:p.N751K,SEMA6D:ENST00000358066.4:exon17:c.T2067A:p.N689K,SEMA6D:ENST00000389428.3:exon17:c.T2028A:p.N676K,SEMA6D:ENST00000389433.2:exon18:c.T2196A:p.N732K,SEMA6D:ENST00	15q21.1	.
15	53957929	C	T	AAGAATAATT,GTGCTCTTTC	exonic	WDR72	nonsynonymous SNV	WDR72:ENST00000360509.5:exon14:c.G1802A:p.R601Q,WDR72:ENST00000557913.1:exon14:c.G1793A:p.R598Q,WDR72:ENST00000559418.1:exon13:c.G1832A:p.R611Q,WDR72:ENST00000396328.1:exon14:c.G1802A:p.R601Q,WDR72:ENST00000560036.1:exon15:c.G1802A:p.R601Q,	15q21.3	W
15	56207648	C	T	TGGATAAGCT,CTCCCAATGA	exonic	NEDD4	nonsynonymous SNV	NEDD4:ENST00000506154.1:exon1:c.G1382A:p.G461E,NEDD4:ENST00000338963.2:exon1:c.G1382A:p.G461E,NEDD4:ENST00000508342.1:exon1:c.G1382A:p.G461E,	15q21.3	.
15	63054504	G	A	CCTGGTCTCA,CCAAGACCAT	exonic	TLN2	nonsynonymous SNV	TLN2:ENST00000306829.6:exon36:c.G4813A:p.A1605T,TLN2:ENST00000561311.1:exon38:c.G4813A:p.A1605T,TLN2:ENST00000494733.1:exon12:c.G1555A:p.A519T,	15q22.2	TI
15	63866279	G	A	GGCTATATTC,GAGGCATTCT	exonic	USP3	nonsynonymous SNV	USP3:ENST00000380324.3:exon10:c.G943A:p.G315R,USP3:ENST00000559711.1:exon8:c.G676A:p.G226R,USP3:ENST00000540797.1:exon9:c.G811A:p.G271R,USP3:ENST00000539772.1:exon4:c.G196A:p.G66R,USP3:ENST00000268049.7:exon11:c.G877A:p.G293R,USP3:ENST00000558285.1:exon9:c.G892/	15q22.31	.
15	63866280	G	A	GCTATATTCG,AGGCATTCTC	exonic	USP3	nonsynonymous SNV	USP3:ENST00000380324.3:exon10:c.G944A:p.G315E,USP3:ENST00000559711.1:exon8:c.G677A:p.G226E,USP3:ENST00000540797.1:exon9:c.G812A:p.G271E,USP3:ENST00000539772.1:exon4:c.G197A:p.G66E,USP3:ENST00000268049.7:exon11:c.G878A:p.G293E,USP3:ENST00000558285.1:exon9:c.G893A	15q22.31	.
15	64419436	G	A	GAAAAACGGA,GGCCGCTTTA	exonic	SNX1	nonsynonymous SNV	SNX1:ENST00000353874.4:exon7:c.G716A:p.R239K,SNX1:ENST00000561026.1:exon5:c.G521A:p.R174K,SNX1:ENST00000261889.5:exon7:c.G716A:p.R239K,SNX1:ENST00000559844.1:exon7:c.G716A:p.R239K,SNX1:ENST00000560829.1:exon6:c.G62A:p.R21K,	15q22.31	.
15	64480609	G	C	GCAGCCAAGT,AGACGTTAGG	exonic	CSNK1G1	nonsynonymous SNV	CSNK1G1:ENST00000607537.1:exon11:c.C1183G:p.H395D,	15q22.31	.
15	65110144	C	T	CCGGTGCTCA,TTGGCTCTTG	splicing	PIF1(ENST00.		.	15q22.31	.
15	65625738	C	T	GCCCTCCACC,CGATAGGGCG	exonic	IGDCC3	nonsynonymous SNV	IGDCC3:ENST00000558354.1:exon5:c.G428A:p.G143E,IGDCC3:ENST00000327987.4:exon6:c.G839A:p.G280E,	15q22.31	.
15	66820209	G	A	AAATAAGCTG,ATGGATTTGG	exonic	ZWILCH	nonsynonymous SNV	ZWILCH:ENST00000565627.1:exon9:c.G586A:p.D196N,ZWILCH:ENST00000307897.5:exon10:c.G928A:p.D310N,ZWILCH:ENST00000446801.2:exon10:c.G586A:p.D196N,ZWILCH:ENST00000535141.2:exon9:c.G586A:p.D196N,	15q22.31	.
15	70976640	T	C	TTCAACAAGG,TAGAATGTCC	exonic	UACA	nonsynonymous SNV	UACA:ENST00000379983.2:exon8:c.A709G:p.T237A,UACA:ENST00000558758.1:exon8:c.A709G:p.T237A,UACA:ENST00000560441.1:exon8:c.A709G:p.T237A,UACA:ENST00000322954.6:exon8:c.A748G:p.T250A,	15q23	.
15	72057532	G	A	GCACCGAATG,AGCATGGTAA	exonic	THSD4	stopgain	THSD4:ENST00000357769.4:exon10:c.G1683A:p.W561X,THSD4:ENST00000355327.3:exon16:c.G2763A:p.W921X,THSD4:ENST00000261862.6:exon15:c.G2763A:p.W921X,	15q23	.
15	72190790	C	T	TGCAAGTCTC,TTCATCTGAA	exonic	MYO9A	nonsynonymous SNV	MYO9A:ENST00000356056.5:exon25:c.G4054A:p.G1352R,MYO9A:ENST00000566885.1:exon23:c.G2914A:p.G972R,MYO9A:ENST00000424560.1:exon25:c.G4054A:p.G1352R,MYO9A:ENST00000444904.1:exon24:c.G3997A:p.G1333R,MYO9A:ENST00000564571.1:exon25:c.G4054A:p.G1352R,	15q23	.
15	73052858	C	A	AACTGGACCG,AAAGAAGAAC	exonic	ADPGK	nonsynonymous SNV	ADPGK:ENST00000569534.1:exon2:c.G244T:p.A82S,ADPGK:ENST00000565814.1:exon4:c.G167T:p.C56F,ADPGK:ENST00000311669.8:exon4:c.G533T:p.C178F,	15q24.1	.
15	74426248	G	A	GCCTGGCGCC,GGGGAGAACC	exonic	ISLR2	nonsynonymous SNV	ISLR2:ENST00000453268.2:exon3:c.G1153A:p.G385R,ISLR2:ENST00000361742.3:exon4:c.G1153A:p.G385R,ISLR2:ENST00000565540.1:exon2:c.G1153A:p.G385R,ISLR2:ENST00000435464.1:exon4:c.G1153A:p.G385R,ISLR2:ENST00000419208.1:exon2:c.G1153A:p.G385R,ISLR2:ENST00000445793.1:exon4:(	15q24.1	.
15	75015057	C	T	CACACTGGTC,AGAGTCTGGG	exonic	CYP1A1	nonsynonymous SNV	CYP1A1:ENST00000567032.1:exon2:c.G382A:p.G128R,CYP1A1:ENST00000395049.4:exon2:c.G382A:p.G128R,CYP1A1:ENST00000379727.3:exon2:c.G382A:p.G128R,CYP1A1:ENST00000562201.1:exon2:c.G382A:p.G128R,CYP1A1:ENST00000569630.1:exon2:c.G382A:p.G128R,CYP1A1:ENST00000395048.2	15q24.1	.

15	79749696	C	T	TAGCCAGACC,CCAATTTCCC	exonic	KIAA1024	nonsynonymous SNV	KIAA1024:ENST00000559272.1:exon1:c.C1207T:p.P403S,KIAA1024:ENST00000305428.3:exon2:c.C1207T:p.P403S, [illegible]
15	85230934	C	T	TTCTCCCACT,GTATGGGATC	exonic	SEC11A	nonsynonymous SNV	SEC11A:ENST00000558217.1:exon3:c.G221A:p.R74Q,SEC11A:ENST00000560266.1:exon3:c.G233A:p.R78Q,SEC11A:ENST00000560409.1:[illegible]
15	89424842	G	A	ACGCCGCGGG,AGACCAGGGC	exonic	HAPLN3	nonsynonymous SNV	HAPLN3:ENST00000359595.3:exon3:c.C239T:p.S80F,HAPLN3:ENST00000562281.1:exon3:c.C239T:p.S80F,HAPLN3:ENST00000562889.1:[illegible]
15	90347126	C	T	TTACCAAGTT,CAGGTGGGCT	exonic	ANPEP	stopgain	ANPEP:ENST00000560030.1:exon1:c.G75A:p.W25X,ANPEP:ENST00000300060.6:exon7:c.G1287A:p.W429X, [illegible]
15	91433170	G	A	GAATGAAGAG,AGAACACCCA	exonic	FES	nonsynonymous SNV	FES:ENST00000394300.3:exon6:c.G853A:p.E285K,FES:ENST00000328850.3:exon8:c.G1027A:p.E343K,FES:ENST00000394302.1:exon7:[illegible]
15	91493400	C	T	ATCCCGCTGG,CCTGGAAGGC	exonic	UNC45A	nonsynonymous SNV	UNC45A:ENST00000394275.2:exon19:c.C2045T:p.A682V,UNC45A:ENST00000418476.2:exon16:c.C2090T:p.A697V, [illegible]
15	91545331	G	A	ACGGCTGTGA,GGTGTCCCCG	exonic	VPS33B	nonsynonymous SNV	VPS33B:ENST00000535843.1:exon17:c.C1081T:p.L361F,VPS33B:ENST00000535906.1:exon17:c.C1273T:p.L425F,VPS33B:[illegible]
15	94841745	C	T	AGTCTGTCCA,TGCAGGGATC	exonic	MCTP2	nonsynonymous SNV	MCTP2:ENST00000456504.1:exon2:c.C251T:p.T84I,MCTP2:ENST00000357742.4:exon1:c.C251T:p.T84I,MCTP2:ENST00000451018.3:[illegible]
16	684730	G	A	ATCCTCTCCA,GAGCAGGCCA	exonic	C16orf13	nonsynonymous SNV	C16orf13:ENST00000397665.2:exon3:c.C428T:p.P143L,C16orf13:ENST00000397666.2:exon4:c.C488T:p.P163L, [illegible]
16	735721	C	G	TCGTCCTCCT,ACCTTCCACG	exonic	WDR24	nonsynonymous SNV	WDR24:ENST00000248142.6:exon10:c.G2026C:p.E676Q,WDR24:ENST00000293883.4:exon6:c.G1636C:p.E546Q, [illegible]
16	815290	G	A	GCAGGGCGGG,GACCCCCCTA	exonic	MSLN	nonsynonymous SNV	MSLN:ENST00000545450.2:exon9:c.G691A:p.G231R,MSLN:ENST00000382862.3:exon8:c.G691A:p.G231R,MSLN:ENST00000566549.1:[illegible]
16	1035270	C	T	CCCCTGCTTC,ACTCGCCGCG	exonic	SOX8	nonsynonymous SNV	SOX8:ENST00000293894.3:exon3:c.C1225T:p.H409Y, [illegible]
16	1307018	G	A	CTTCCCCCCG,GGATGCCGTG	exonic	TPSD1	nonsynonymous SNV	TPSD1:ENST00000211076.3:exon3:c.G475A:p.G159R,TPSD1:ENST00000397534.2:exon4:c.G454A:p.G152R, [illegible]
16	1393494	C	T	GGAAGAGGCT,CTTCACTGCC	exonic	BAIAP3	nonsynonymous SNV	BAIAP3:ENST00000421665.2:exon14:c.C1258T:p.P420S,BAIAP3:ENST00000426824.3:exon15:c.C1366T:p.P456S,BAIAP3:[illegible]
16	1836762	G	A	TTGCAGGTGG,AATCCTGGAT	exonic	NUBP2	nonsynonymous SNV	NUBP2:ENST00000262302.9:exon3:c.G140A:p.G47E,NUBP2:ENST00000568287.1:exon4:c.G404A:p.G135E,NUBP2:ENST00000565134.1:[illegible]
16	1840659	G	A	CACGACCTCG,GCGGGCTGGC	exonic	IGFALS	nonsynonymous SNV	IGFALS:ENST00000415638.3:exon2:c.C1874T:p.P625L,IGFALS:ENST00000215539.3:exon2:c.C1760T:p.P587L, [illegible]
16	2294443	C	A	TGGACTGGTA,AACCGCAGCC	exonic	ECI1	nonsynonymous SNV	ECI1:ENST00000570258.1:exon4:c.G228T:p.L76F,ECI1:ENST00000562238.1:exon4:c.G405T:p.L135F,ECI1:ENST00000566379.1:[illegible]
16	3634801	C	T	ACCGGCGTCT,CATAATGGAA	exonic	SLX4	nonsynonymous SNV	SLX4:ENST00000294008.3:exon13:c.G4708A:p.E1570K, [illegible]
16	4555550	G	C	GGAAACCTCA,AGGGGGTAGA	exonic	HMOX2	nonsynonymous SNV	HMOX2:ENST00000575051.1:exon2:c.G25C:p.E9Q,HMOX2:ENST00000414777.1:exon2:c.G25C:p.E9Q,HMOX2:ENST00000570445.1:[illegible]
16	9923416	C	T	GCTGGTGGTC,CTTTAGGATT	exonic	GRIN2A	nonsynonymous SNV	GRIN2A:ENST00000562109.1:exon8:c.G1871A:p.G624E,GRIN2A:ENST00000535259.1:exon8:c.G1400A:p.G467E,GRIN2A:[illegible]
16	11525054	G	A	GCTGTGTTCT,GTCCAGCAGT	exonic	CTD-3088G3.	stopgain	CTD-3088G3.8:ENST00000598234.1:exon37:c.C5332T:p.Q1778X, [illegible]
16	14041558	G	A	ATCCATCGTC,GGGCATTGAC	exonic	ERCC4	nonsynonymous SNV	ERCC4:ENST00000311895.7:exon11:c.G2105A:p.R702Q, [illegible]
16	14341053	G	T	CTCACTCCCT,ACTGCTCCAG	exonic	MKL2	nonsynonymous SNV	MKL2:ENST00000318282.5:exon11:c.G1936T:p.D646Y,MKL2:ENST00000571589.1:exon12:c.G1936T:p.D646Y,MKL2:ENST00000341243.5:[illegible]
16	15092232	C	T	AAGCTCATAT,CGGAGATATT	exonic	PDXDC1	nonsynonymous SNV	PDXDC1:ENST00000563679.1:exon3:c.C185T:p.S62F,PDXDC1:ENST00000565362.1:exon3:c.C131T:p.S44F,PDXDC1:ENST00000567306.1:[illegible]
16	19068001	C	T	TTTGCAGTTC,TTTCTTCATG	exonic	TMC7	nonsynonymous SNV	TMC7:ENST00000569532.1:exon14:c.C2009T:p.P670L,TMC7:ENST00000421369.3:exon14:c.C1679T:p.P560L,TMC7:ENST00000304381.5:[illegible]
16	19195279	C	T	ACCCAGAAGC,CGTGTTTGAG	exonic	SYT17	nonsynonymous SNV	SYT17:ENST00000568115.1:exon4:c.C578T:p.P193L,SYT17:ENST00000355377.2:exon5:c.C761T:p.P254L,SYT17:ENST00000562711.2:[illegible]
16	20370859	C	T	ACTTCCTCTT,TATCACTTCA	exonic	PDILT	nonsynonymous SNV	PDILT:ENST00000302451.4:exon12:c.G1537A:p.E513K, [illegible]
16	20838768	G	A	AGTTATTTTG,GGTAGGTTTG	exonic	AC004381.6	nonsynonymous SNV	AC004381.6:ENST00000564274.1:exon10:c.G1054A:p.G352R,AC004381.6:ENST00000348433.6:exon10:c.G1054A:p.G352R,AC004381.6:[illegible]
16	20838769	G	A	GTTATTTTGG,GTAGGTTTGC	exonic	AC004381.6	nonsynonymous SNV	AC004381.6:ENST00000564274.1:exon10:c.G1055A:p.G352E,AC004381.6:ENST00000348433.6:exon10:c.G1055A:p.G352E,AC004381.6:[illegible]
16	21080894	G	A	TCTTGTATGC,AATTAGCTTT	exonic	DNAH3	nonsynonymous SNV	DNAH3:ENST00000415178.1:exon23:c.C3223T:p.R1075C,DNAH3:ENST00000261383.3:exon23:c.C3223T:p.R1075C, [illegible]
16	21261183	A	T	AATGACTTAC,GACTCCACTG	exonic	ANKS4B	nonsynonymous SNV	ANKS4B:ENST00000311620.5:exon2:c.A296T:p.Q99L, [illegible]
16	23080152	G	A	GGTTGGGCAG,GGCAGGGGAT	exonic	USP31	nonsynonymous SNV	USP31:ENST00000567975.1:exon2:c.C1153T:p.P385S,USP31:ENST00000219689.7:exon16:c.C3274T:p.P1092S, [illegible]
16	23536554	C	T	CAACCCGTGT,CCTGCCTCAC	exonic	EARS2	nonsynonymous SNV	EARS2:ENST00000563232.1:exon8:c.G1499A:p.G500E, [illegible]
16	24580185	G	A	TCACATTCTC,TTCCTATTCA	exonic	RBBP6	nonsynonymous SNV	RBBP6:ENST00000564314.1:exon15:c.G1775A:p.R592H,RBBP6:ENST00000319715.4:exon17:c.G2174A:p.R725H,RBBP6:[illegible]
16	24909387	C	T	TGCCCCTCTT,ATAATGGTGT	exonic	SLC5A11	nonsynonymous SNV	SLC5A11:ENST00000569071.1:exon8:c.C565T:p.H189Y,SLC5A11:ENST00000449109.2:exon9:c.C565T:p.H189Y, [illegible]
16	25263293	G	A	CTTTGGGCAG,AATCTGATGC	exonic	ZKSCAN2	nonsynonymous SNV	ZKSCAN2:ENST00000328086.7:exon4:c.C742T:p.P248S, [illegible]
16	28331721	C	T	CGGCAACTTC,CGTGGGAGGC	exonic	SBK1	nonsynonymous SNV	SBK1:ENST00000341901.4:exon4:c.C754T:p.P252S, [illegible]
16	28905557	G	T	CGCTCCAGAG,GAGAGGTGTA	exonic	ATP2A1	stopgain	ATP2A1:ENST00000536376.1:exon8:c.G802T:p.G268X,ATP2A1:ENST00000395503.4:exon10:c.G1177T:p.G393X,ATP2A1:[illegible]
16	29676017	G	A	GGAGGGTCCG,GGGCGACAAG	exonic	SPN	nonsynonymous SNV	SPN:ENST00000360121.3:exon2:c.G968A:p.G323E,SPN:ENST00000436527.1:exon2:c.G968A:p.G323E,SPN:ENST00000563039.1:[illegible]
16	29891359	G	A	GCCAGGAAGG,GGCGAAGCAG	exonic	SEZ6L2	nonsynonymous SNV	SEZ6L2:ENST00000350527.3:exon9:c.C1189T:p.P397S,SEZ6L2:ENST00000308713.5:exon9:c.C1399T:p.P467S,SEZ6L2:[illegible]
16	30532867	C	T	CAGAGGTGTC,CGAATGGAAT	exonic	ITGAL	nonsynonymous SNV	ITGAL:ENST00000356798.6:exon31:c.C3394T:p.P1132S,ITGAL:ENST00000433423.2:exon13:c.C1096T:p.P366S,ITGAL:[illegible]
16	30532868	C	T	AGAGGTGTCC,GAATGGAATC	exonic	ITGAL	nonsynonymous SNV	ITGAL:ENST00000356798.6:exon31:c.C3395T:p.P1132L,ITGAL:ENST00000433423.2:exon13:c.C1097T:p.P366L,ITGAL:[illegible]
16	30910754	C	T	CAGACTGATT,CTCAGTCTCA	exonic	CTF1	nonsynonymous SNV	CTF1:ENST00000395019.3:exon2:c.C41T:p.S14F,CTF1:ENST00000279804.2:exon2:c.C44T:p.S15F, [illegible]
16	31090376	G	A	TGGCATGACT,AGGGCTCAGA	exonic	ZNF646	nonsynonymous SNV	ZNF646:ENST00000394979.2:exon1:c.G2731A:p.E911K,ZNF646:ENST00000300850.5:exon2:c.G2731A:p.E911K, [illegible]
16	47581456	C	T	GAGCTACATT,GAGGTAATTT	exonic	PHKB	nonsynonymous SNV	PHKB:ENST00000299167.8:exon7:c.C707T:p.S236L,PHKB:ENST00000566037.2:exon8:c.C686T:p.S229L,PHKB:ENST00000566044.1:[illegible]
16	50707866	C	A	CGTCTTCGAT,TCCTCCCTGA	exonic	SNX20	nonsynonymous SNV	SNX20:ENST00000330943.4:exon4:c.G402T:p.E134D, [illegible]
16	53496493	G	A	TCGTTTTGCG,AGATGCTTTA	exonic	RBL2	nonsynonymous SNV	RBL2:ENST00000544405.2:exon11:c.G1264A:p.E422K,RBL2:ENST00000262133.6:exon11:c.G1486A:p.E496K,RBL2:ENST00000544545.1:[illegible]
16	56534901	C	T	TGTAAAAATT,CTTCTGCAAA	exonic	BBS2	nonsynonymous SNV	BBS2:ENST00000568104.1:exon11:c.G1262A:p.G421E,BBS2:ENST00000245157.5:exon11:c.G1262A:p.G421E, [illegible]
16	61891029	C	T	AAACCTGTTT,AGGCTCAATG	exonic	CDH8	nonsynonymous SNV	CDH8:ENST00000583382.1:exon4:c.G661A:p.E221K,CDH8:ENST00000299345.6:exon4:c.G661A:p.E221K,CDH8:ENST00000577390.1:[illegible]
16	66947175	G	A	GCCCGCACCT,GAGCAGGTAC	exonic	CDH16	stopgain	CDH16:ENST00000570262.1:exon7:c.C673T:p.Q225X,CDH16:ENST00000565796.1:exon9:c.C913T:p.Q305X,CDH16:ENST00000394055.3:[illegible]
16	70515729	G	A	CTAGTCATGA,GCCGGTTAGG	exonic	COG4	nonsynonymous SNV	COG4:ENST00000323786.5:exon17:c.C2044T:p.L682F,COG4:ENST00000565715.1:exon2:c.C106T:p.L36F, [illegible]
16	70698783	G	A	GGGCCTCCTG,GACTGACCTG	exonic	FLJ00418	nonsynonymous SNV	FLJ00418:ENST00000597002.1:exon1:c.G92A:p.G31E, [illegible]
16	71406097	C	T	AAACTTTTTC,AAGAGCTGGA	exonic	CALB2	stopgain	CALB2:ENST00000302628.4:exon2:c.C136T:p.Q46X,CALB2:ENST00000349553.5:exon2:c.C136T:p.Q46X, [illegible]
16	72057490	C	T	TGGGGGCCAC,CGTTGTGGGC	exonic	DHODH	nonsynonymous SNV	DHODH:ENST00000572887.1:exon8:c.C1085T:p.P362L,DHODH:ENST00000219240.4:exon8:c.C1091T:p.P364L, [illegible]
16	84432166	C	T	GCCCTGCCCC,CAAGGAAGCG	exonic	ATP2C2	nonsynonymous SNV	ATP2C2:ENST00000416219.2:exon2:c.C161T:p.P54L,ATP2C2:ENST00000262429.4:exon2:c.C161T:p.P54L, [illegible]
16	86575791	G	A	CCAGATCGGC,TAGCCTTCTC	exonic	MTHFSD	nonsynonymous SNV	MTHFSD:ENST00000567539.1:exon3:c.C149T:p.T50M, [illegible]
16	86612775	C	T	AAGCCGGGCC,CGGGGCCCCG	exonic	FOXL1	nonsynonymous SNV	FOXL1:ENST00000320241.3:exon1:c.C446T:p.P149L, [illegible]
16	88497482	C	T	CCCCAAGGAT,CCCTGCAGGT	exonic	ZNF469	nonsynonymous SNV	ZNF469:ENST00000437464.1:exon2:c.C3520T:p.P1174S,ZNF469:ENST00000565624.1:exon1:c.C3604T:p.P1202S, [illegible]
16	88500702	G	A	CCTCTGGCAG,GGCCGTCTCC	exonic	ZNF469	nonsynonymous SNV	ZNF469:ENST00000437464.1:exon2:c.G6740A:p.G2247E,ZNF469:ENST00000565624.1:exon1:c.G6824A:p.G2275E, [illegible]
17	1960880	C	T	GGACCCGAGC,CCCCGGCCGC	exonic	HIC1	nonsynonymous SNV	HIC1:ENST00000322941.3:exon2:c.C953T:p.P318L,HIC1:ENST00000399849.3:exon2:c.C896T:p.P299L, [illegible]
17	3195576	G	A	AGCTGGGTAA,GCAGGCCCCA	exonic	OR3A1	nonsynonymous SNV	OR3A1:ENST00000323404.1:exon1:c.C301T:p.L101F, [illegible]
17	3853987	C	T	GGGGCCTCAC,CGTCAGGATG	exonic	ATP2A3	nonsynonymous SNV	ATP2A3:ENST00000397041.3:exon6:c.G544A:p.G182S,ATP2A3:ENST00000352011.3:exon6:c.G544A:p.G182S,ATP2A3:[illegible]
17	4536188	G	A	CCCGATTTCA,TGATCTCTCG	exonic	ALOX15	nonsynonymous SNV	ALOX15:ENST00000574640.1:exon11:c.C1391T:p.T464I,ALOX15:ENST00000293761.3:exon11:c.C1508T:p.T503I,ALOX15:[illegible]
17	4576145	G	A	CACTAGGCCT,GGACAGAAAG	exonic	PELP1	nonsynonymous SNV	PELP1:ENST00000269230.7:exon20:c.C1871T:p.P624L,PELP1:ENST00000436683.2:exon17:c.C1700T:p.P567L,PELP1:[illegible]
17	7489336	T	C	TCTGTAATGC,GGAGCTAGTG	exonic	MPDU1	nonsynonymous SNV	MPDU1:ENST00000584378.1:exon3:c.T242C:p.L81P,MPDU1:ENST00000584479.1:exon3:c.T242C:p.L81P,MPDU1:ENST00000423172.2:[illegible]
17	7574026	C	A	GCGCTCACGC,CACGGATCTG	exonic	TP53	nonsynonymous SNV	TP53:ENST00000269305.4:exon10:c.G1001T:p.G334V,TP53:ENST00000445888.2:exon10:c.G1001T:p.G334V, [illegible]
17	8701721	C	T	CGCAACGCCT,CAAGGACAGG	exonic	MFSD6L	nonsynonymous SNV	MFSD6L:ENST00000329805.4:exon1:c.G718A:p.E240K, [illegible]
17	10235466	G	A	ATGGAGTTGA,GAGCTTCTCT	exonic	MYH13	nonsynonymous SNV	MYH13:ENST00000252172.4:exon20:c.C2248T:p.L750F,MYH13:ENST00000418404.3:exon19:c.C2248T:p.L750F, [illegible]
17	10399401	C	T	GCTCTGCGCT,CACCATAGCC	exonic	MYH1	nonsynonymous SNV	MYH1:ENST00000226207.5:exon35:c.G5035A:p.E1679K, [illegible]
17	18022308	C	T	GGCCTCCACA,CGGCCCCCAG	exonic	MYO15A	nonsynonymous SNV	MYO15A:ENST00000205890.5:exon2:c.C194T:p.T65I, [illegible]
17	18167014	C	T	CCCGCCCCTT,ACTCTGCAGA	exonic	MIEF2	nonsynonymous SNV	MIEF2:ENST00000578621.1:exon4:c.C338T:p.S113L, [illegible]
17	18923026	G	A	TCCTCCCCAG,GGGCCTTCTG	exonic	SLC5A10	nonsynonymous SNV	SLC5A10:ENST00000395647.2:exon13:c.G1459A:p.G487R,SLC5A10:ENST00000417251.2:exon12:c.G1303A:p.G435R,SLC5A10:[illegible]
17	20108732	C	T	GAAGAGCCCA,CACTCAGGAA	exonic	SPECC1	nonsynonymous SNV	SPECC1:ENST00000395525.3:exon2:c.C1127T:p.T376I,SPECC1:ENST00000395522.2:exon2:c.C1127T:p.T376I,SPECC1:[illegible]
17	21454586	G	A	CGCGAAGAGG,GGTGAAACTC	exonic	C17orf51	nonsynonymous SNV	C17orf51:ENST00000412778.3:exon1:c.C100T:p.P34S,C17orf51:ENST00000391411.5:exon1:c.C100T:p.P34S, [illegible]
17	27379960	G	A	ACTGCTGCTG,GAATGAAAGA	exonic	PIPOX	nonsynonymous SNV	PIPOX:ENST00000323372.4:exon3:c.G286A:p.G96R,PIPOX:ENST00000466889.1:exon3:c.G88A:p.G30R,PIPOX:ENST00000469082.1:[illegible]
17	29645844	G	A	AGGAGTTATA,GGTTTGTGTT	exonic	CTD-2370N5.	nonsynonymous SNV	EVI2A:ENST00000247270.3:exon3:c.C257T:p.P86L,EVI2A:ENST00000462804.2:exon2:c.C188T:p.P63L,EVI2A:ENST00000461237.1:[illegible]
17	30186140	C	T	GGGGGCGCCC,CCGCGCGCTA	exonic	COPRS	nonsynonymous SNV	COPRS:ENST00000302362.6:exon1:c.G79A:p.G27R,COPRS:ENST00000496655.1:exon1:c.G79A:p.G27R,COPRS:ENST00000579984.1:[illegible]
17	33881672	G	A	TCGTGCAGAT,AAGCTGATGA	exonic	SLFN14	nonsynonymous SNV	SLFN14:ENST00000415846.3:exon2:c.C1112T:p.S371L, [illegible]
17	34324841	G	A	ATGCAATCCT,AACTCCCGGA	exonic	CCL15,CCL1[illegible]	stopgain	CCL15:ENST00000354059.4:exon4:c.C304T:p.Q102X,CCL15-CCL14:ENST00000481427.2:exon4:c.C304T:p.Q102X,CCL15-CCL14:[illegible]
17	36486507	G	A	GCTTTGTGGG,ATACTGGGAC	exonic	GPR179	nonsynonymous SNV	GPR179:ENST00000342292.4:exon11:c.C2945T:p.S982F, [illegible]
17	38645149	G	C	CGGGGTGACA,AGGGGCTGGA	exonic	TNS4	nonsynonymous SNV	TNS4:ENST00000254051.6:exon3:c.C512G:p.S171C, [illegible]
17	40870534	G	A	GGAGTGCAGA,ATTGCTCCCG	exonic	EZH1	nonsynonymous SNV	EZH1:ENST00000586382.1:exon8:c.C722T:p.S241F,EZH1:ENST00000415827.2:exon9:c.C842T:p.S281F,EZH1:ENST00000592743.1:[illegible]
17	40997391	C	T	GGCCCTGGAC,CTGCCCACTG	exonic	AOC2	nonsynonymous SNV	AOC2:ENST00000253799.3:exon1:c.C748T:p.P250S,AOC2:ENST00000452774.2:exon1:c.C748T:p.P250S, [illegible]
17	41170653	C	T	CTGTAGGTTG,CGAAGTCAAA	exonic	VAT1	nonsynonymous SNV	VAT1:ENST00000355653.3:exon2:c.G551A:p.G184D,VAT1:ENST00000420567.3:exon2:c.G149A:p.G50D,VAT1:ENST00000589709.1:[illegible]
17	41170654	C	T	TGTAGGTTGC,GAAGTCAAAG	exonic	VAT1	nonsynonymous SNV	VAT1:ENST00000355653.3:exon2:c.G550A:p.G184S,VAT1:ENST00000420567.3:exon2:c.G148A:p.G50S,VAT1:ENST00000589709.1:[illegible]
17	45914376	C	A	CAGGGGCCAC,AAAGCTCTTT	exonic	LRRC46	nonsynonymous SNV	LRRC46:ENST00000269025.4:exon8:c.C856A:p.Q286K, [illegible]
17	56651594	C	T	GATGTCTCTT,TTGTTCATCT	exonic	TEX14	nonsynonymous SNV	TEX14:ENST00000349033.5:exon22:c.G3337A:p.E1113K,TEX14:ENST00000389934.3:exon23:c.G3457A:p.E1153K,TEX14:[illegible]
17	58040524	G	A	ACACACTGAG,GGTTGAGGCA	exonic	RNFT1	nonsynonymous SNV	RNFT1:ENST00000442346.2:exon2:c.C67T:p.P23S,RNFT1:ENST00000466544.1:exon2:c.C178T:p.P60S,RNFT1:ENST00000305783.8:[illegible]
17	61628063	G	A	CAAACGGAAG,AGATCTACAA	exonic	DCAF7	nonsynonymous SNV	DCAF7:ENST00000415273.2:exon1:c.G25A:p.E9K,DCAF7:ENST00000431926.1:exon1:c.G25A:p.E9K,DCAF7:ENST00000310827.4:[illegible]
17	62081172	A	G	GCCTTCCCGA,GGTCTCATAG	exonic	ICAM2	nonsynonymous SNV	ICAM2:ENST00000418105.1:exon4:c.T481C:p.F161L,ICAM2:ENST00000579687.1:exon5:c.T481C:p.F161L,ICAM2:ENST00000578379.1:[illegible]
17	67031440	C	T	AAAGTCCATT,CAAAAATGCA	exonic	ABCA9	nonsynonymous SNV	ABCA9:ENST00000340001.4:exon8:c.G1075A:p.E359K,ABCA9:ENST00000453985.2:exon8:c.G1075A:p.E359K,ABCA9:ENST00000370732.2:[illegible]
17	67039800	C	T	GTAATATCTT,ATATGTACAC	exonic	ABCA9	nonsynonymous SNV	ABCA9:ENST00000340001.4:exon6:c.G630A:p.M210I,ABCA9:ENST00000453985.2:exon6:c.G630A:p.M210I,ABCA9:ENST00000495634.1:[illegible]
17	73236104	G	C	GGAGGGGGCT,AGGGCTGGAG	exonic	GGA3	stopgain	GGA3:ENST00000245541.6:exon13:c.C1349G:p.S450X,GGA3:ENST00000582717.1:exon13:c.C1133G:p.S378X,GGA3:ENST00000582486.1:[illegible]
17	76210851	G	A	GGAAGGCTGG,AGCCAGATGA	exonic	AC087645.1,[illegible]	nonsynonymous SNV	BIRC5:ENST00000592734.1:exon2:c.G202A:p.E68K,BIRC5:ENST00000590925.2:exon2:c.G202A:p.E68K,BIRC5:ENST00000301633.4:[illegible]
17	76212078	A	C	CGCCTGTAAT,CCAGCACTTT	exonic	BIRC5	nonsynonymous SNV	BIRC5:ENST00000301633.4:exon3:c.A253C:p.T85P, [illegible]
17	78831645	G	A	AGCTCGGCCC,AGAGCTGCGG	exonic	RPTOR	nonsynonymous SNV	RPTOR:ENST00000537330.1:exon12:c.G899A:p.R300Q,RPTOR:ENST00000544334.2:exon13:c.G1454A:p.R485Q,RPTOR:[illegible]
17	80788640	G	A	TGATTTCTTG,AGAGGTTGAG	exonic	ZNF750	nonsynonymous SNV	ZNF750:ENST00000269394.3:exon3:c.C1550T:p.S517F,ZNF750:ENST00000572562.1:exon3:c.C353T:p.S118F, [illegible]
17	80790167	A	T	CTCTGATACT,AAGTAATCGA	exonic	ZNF750	stopgain	ZNF750:ENST00000269394.3:exon2:c.T164A:p.L55X, [illegible]
18	756764	G	C	ACAGGCTCTG,AGTATTTTCA	exonic	YES1	nonsynonymous SNV	YES1:ENST00000314574.4:exon2:c.C64G:p.P22A,YES1:ENST00000577961.1:exon2:c.C79G:p.P27A,YES1:ENST00000584307.1:[illegible]
18	5891263	C	T	CCCGGAGCCA,CGCAGCCCCC	exonic	TMEM200C	nonsynonymous SNV	TMEM200C:ENST00000383490.2:exon1:c.G800A:p.G267D,TMEM200C:ENST00000581347.2:exon3:c.G800A:p.G267D, [illegible]
18	8069989	G	A	GACAGATGAA,ACCGTGAGTA	exonic	PTPRM	nonsynonymous SNV	PTPRM:ENST00000580170.1:exon8:c.G1438A:p.D480N,PTPRM:ENST00000400053.4:exon8:c.G1252A:p.D418N,PTPRM:[illegible]
18	20716551	C	G	CCAGCGCCTT,GAGCAGCTGC	exonic	CABLES1	nonsynonymous SNV	CABLES1:ENST00000256925.7:exon1:c.C825G:p.F275L, [illegible]
18	23619363	C	T	GTAATGCTGT,CGCCTCCCTG	exonic	SS18	nonsynonymous SNV	SS18:ENST00000545952.1:exon7:c.G509A:p.G170E,SS18:ENST00000415083.2:exon6:c.G665A:p.G222E,SS18:ENST00000269137.7:[illegible]
18	23619364	C	T	TAATGCTGTC,GCCTCCCTGT	exonic	SS18	nonsynonymous SNV	SS18:ENST00000545952.1:exon7:c.G508A:p.G170R,SS18:ENST00000415083.2:exon6:c.G664A:p.G222R,SS18:ENST00000269137.7:[illegible]
18	23670475	G	A	CTTCTGAATC,CAGCGGGAGT	exonic	SS18	nonsynonymous SNV	SS18:ENST00000585121.1:exon1:c.C59T:p.A20V,SS18:ENST00000578954.1:exon1:c.C59T:p.A20V,SS18:ENST00000582792.1:[illegible]
18	23724572	C	T	GAAAAAAAAT,TGTTGCCAAG	exonic	PSMA8	nonsynonymous SNV	PSMA8:ENST00000308268.6:exon2:c.C152T:p.S51F,PSMA8:ENST00000415576.2:exon2:c.C152T:p.S51F, [illegible]
18	25570262	T	A	TGGCACTTGA,TTTCTGCAGC	exonic	CDH2	nonsynonymous SNV	CDH2:ENST00000399380.3:exon9:c.A1304T:p.N435I,CDH2:ENST00000269141.3:exon10:c.A1397T:p.N466I, [illegible]
18	25583020	A	G	TTGATTGTAA,CATGTTGGGT	exonic	CDH2	nonsynonymous SNV	CDH2:ENST00000399380.3:exon6:c.T868C:p.F290L,CDH2:ENST00000269141.3:exon7:c.T961C:p.F321L, [illegible]
18	28935251	C	T	GGGTCCGCCT,CCCTAGCACA	exonic	DSG1	nonsynonymous SNV	DSG1:ENST00000257192.4:exon15:c.C3092T:p.S1031F,DSG1:ENST00000462981.2:exon4:c.C1169T:p.S390F, [illegible]
18	29122798	A	G	AGAATTCTTA,GAAATTATTT	exonic	DSG2	nonsynonymous SNV	DSG2:ENST00000261590.8:exon14:c.A2317G:p.R773G, [illegible]
18	29797758	G	A	GTACATGGGA,AAAGGTGTGA	exonic	MEP1B	nonsynonymous SNV	MEP1B:ENST00000581447.1:exon14:c.G1921A:p.E641K,MEP1B:ENST00000269202.6:exon14:c.G1921A:p.E641K, [illegible]
18	31226245	G	A	TGGCACGTTG,ATTTAGTCTG	exonic	ASXL3	nonsynonymous SNV	ASXL3:ENST00000269197.5:exon4:c.G283A:p.D95N,ASXL3:ENST00000590189.1:exon4:c.G274A:p.D92N,ASXL3:ENST00000592288.1:[illegible]
18	31311944	G	A	GGGAAGTGAT,GAATTTTACG	exonic	ASXL3	nonsynonymous SNV	ASXL3:ENST00000269197.5:exon9:c.G892A:p.G298R, [illegible]
18	43420871	C	T	TGTCCTGTGT,CCTGTTTGCT	exonic	SIGLEC15	nonsynonymous SNV	SIGLEC15:ENST00000587418.1:exon4:c.C235T:p.P79S, [illegible]
18	50734065	G	A	GAGGTTGATG,ACTATCTTAT	exonic	DCC	nonsynonymous SNV	DCC:ENST00000442544.2:exon11:c.G1739A:p.G580E,DCC:ENST00000581580.1:exon8:c.G704A:p.G235E,DCC:ENST00000412726.1:[illegible]
18	56586570	C	T	CCCGTCCTCT,CCGCAGGGTC	exonic	ZNF532	nonsynonymous SNV	ZNF532:ENST00000336078.4:exon4:c.C1051T:p.P351S,ZNF532:ENST00000589288.1:exon3:c.C1051T:p.P351S,ZNF532:[illegible]
18	56892775	G	A	GTTTCTGAGA,AGGGAGCCTG	exonic	GRP	nonsynonymous SNV	GRP:ENST00000256857.2:exon2:c.G191A:p.R64K,GRP:ENST00000529320.2:exon2:c.G191A:p.R64K,GRP:ENST00000420468.2:[illegible]
18	58039535	C	T	TGCTGCGGTT,CAGAGGTGCA	exonic	MC4R	stopgain	MC4R:ENST00000299766.3:exon1:c.G48A:p.W16X, [illegible]
18	59221887	G	A	GAAGCTGGCC,AGCTCTACGG	exonic	CDH20	nonsynonymous SNV	CDH20:ENST00000538374.1:exon12:c.G2365A:p.E789K,CDH20:ENST00000262717.4:exon12:c.G2365A:p.E789K,CDH20:[illegible]
18	63477210	G	C	ATACACGGCA,GAGTTCCCGA	exonic	CDH7	nonsynonymous SNV	CDH7:ENST00000397968.2:exon3:c.G481C:p.G161R,CDH7:ENST00000323011.3:exon3:c.G481C:p.G161R,CDH7:ENST00000536984.2:[illegible]
18	64235727	G	A	ATTGATATCC,AAACTTTGAT	exonic	CDH19	nonsynonymous SNV	CDH19:ENST00000262150.2:exon3:c.C416T:p.S139L,CDH19:ENST00000540086.1:exon3:c.C416T:p.S139L,CDH19:ENST00000579658.1:[illegible]
18	67833401	G	A	TTGTAGTAAT,GACTGGCCTG	exonic	RTTN	nonsynonymous SNV	RTTN:ENST00000437017.1:exon14:c.C1826T:p.P609L,RTTN:ENST00000255674.6:exon14:c.C1826T:p.P609L,RTTN:ENST00000583043.1:[illegible]
18	72114487	C	T	GCAGAGGTCC,CGGCCAGCGT	exonic	FAM69C	nonsynonymous SNV	FAM69C:ENST00000343998.6:exon2:c.G230A:p.G77E, [illegible]
18	72114488	C	T	CAGAGGTCCC,GGCCAGCGTG	exonic	FAM69C	nonsynonymous SNV	FAM69C:ENST00000343998.6:exon2:c.G229A:p.G77R, [illegible]
19	608148	C	T	CTCATCCAGT,GCTGGACTCC	exonic	HCN2	nonsynonymous SNV	HCN2:ENST00000251287.2:exon4:c.C1403T:p.S468L, [illegible]
19	1430334	C	T	GGGCTTCCCT,AGGGCTACGG	exonic	DAZAP1	stopgain	DAZAP1:ENST00000336761.6:exon10:c.C844T:p.Q282X,DAZAP1:ENST00000233078.4:exon10:c.C844T:p.Q282X, [illegible]
19	1816719	C	T	TCCACGATCT,GTTGTCAGGC	exonic	REXO1	nonsynonymous SNV	REXO1:ENST00000170168.4:exon13:c.G3295A:p.E1099K, [illegible]
19	1821679	G	A	GCACCTGTGG,GGCTGGCGGG	exonic	REXO1	nonsynonymous SNV	REXO1:ENST00000170168.4:exon5:c.C2233T:p.P745S, [illegible]
19	3449071	C	T	CAGCCCGTCC,CCCAGGACTC	exonic	NFIC	nonsynonymous SNV	NFIC:ENST00000589123.1:exon7:c.C991T:p.P331S,NFIC:ENST00000341919.3:exon7:c.C1018T:p.P340S,NFIC:ENST00000586919.1:[illegible]
19	3543899	C	G	CTGTCGGCAC,CCAGCGCCCG	exonic	C19orf71	nonsynonymous SNV	C19orf71:ENST00000329493.5:exon4:c.C521G:p.P174R, [illegible]
19	3653392	G	A	TTGTAGGTGG,GAAGCTCTTC	exonic	PIP5K1C	nonsynonymous SNV	PIP5K1C:ENST00000537021.1:exon7:c.C817T:p.P273S,PIP5K1C:ENST00000539785.1:exon7:c.C817T:p.P273S,PIP5K1C:[illegible]
19	4207861	C	T	CCGGGCATCA,CGATGCGCTG	exonic	ANKRD24	nonsynonymous SNV	ANKRD24:ENST00000600132.1:exon10:c.C728T:p.T243I,ANKRD24:ENST00000262970.5:exon8:c.C998T:p.T333I,ANKRD24:[illegible]
19	5707751	C	T	GGACTCGGCC,CGGTGAGCGC	exonic	LONP1	nonsynonymous SNV	LONP1:ENST00000540670.2:exon6:c.G431A:p.G144E,LONP1:ENST00000590729.1:exon6:c.G629A:p.G210E,LONP1:ENST00000593119.1:[illegible]
19	7676646	G	A	CGTCATGGGA,ACCCTGTGCT	exonic	CAMSAP3	nonsynonymous SNV	CAMSAP3:ENST00000160298.4:exon11:c.G1267A:p.D423N,CAMSAP3:ENST00000446248.2:exon13:c.G1348A:p.D450N, [illegible]
19	7935401	G	A	GGGCGTGGCC,AAGGAGACAC	exonic	CTD-3193O1[illegible]	nonsynonymous SNV	CTD-3193O13.9:ENST00000539422.1:exon5:c.C2729T:p.S910L, [illegible]
19	8841652	G	A	CTTTCTGCGG,GAGAAGGTGC	exonic	OR2Z1	nonsynonymous SNV	OR2Z1:ENST00000324060.2:exon1:c.G262A:p.G88R, [illegible]
19	8995694	C	T	TGTGGGGGTC,CAGGAGCTGA	exonic	MUC16	nonsynonymous SNV	MUC16:ENST00000397910.4:exon63:c.G41294A:p.G13765E,MUC16:ENST00000380951.5:exon15:c.G1217A:p.G406E, [illegible]
19	9021053	C	T	GGGGCTCTCA,CAGACCCTGC	splicing	MUC16(ENST.	.	
19	9049955	G	A	ATTGAAAACA,ATGGGGTTGT	exonic	MUC16	nonsynonymous SNV	MUC16:ENST00000397910.4:exon5:c.C31676T:p.S10559F, [illegible]
19	9065696	C	A	AACTGCCTGT,TCCCTCAATT	exonic	MUC16	nonsynonymous SNV	MUC16:ENST00000397910.4:exon3:c.G21750T:p.E7250D, [illegible]
19	9452909	G	A	CAACATTTAA,AACTCATAGT	exonic	ZNF559	nonsynonymous SNV	ZNF559:ENST00000587557.1:exon6:c.G974A:p.R325K,ZNF559:ENST00000603380.1:exon6:c.G782A:p.R261K,ZNF559:[illegible]
19	9645907	G	A	TCTTCCTGCT,GTGTCTTTTC	exonic	ZNF426	nonsynonymous SNV	ZNF426:ENST00000253115.2:exon4:c.C74T:p.P25L,ZNF426:ENST00000589289.1:exon3:c.C74T:p.P25L,ZNF426:ENST00000535489.1:[illegible]
19	10400762	C	T	GCTACTCGGC,TCTGGGCTGC	exonic	ICAM5	nonsynonymous SNV	ICAM5:ENST00000221980.4:exon1:c.C43T:p.L15F, [illegible]
19	11040010	G	A	GGCGCCCTTC,ACGCCCAGGC	exonic	C19orf52	nonsynonymous SNV	C19orf52:ENST00000270502.6:exon2:c.G415A:p.D139N, [illegible]
19	11333451	G	A	GGGCGAGGCT,GAGGTGACAG	exonic	DOCK6	nonsynonymous SNV	DOCK6:ENST00000294618.7:exon26:c.C3200T:p.P1067L,DOCK6:ENST00000319867.7:exon12:c.C1217T:p.P406L, [illegible]
19	12386857	T	C	CCACTCCTCA,GGGTGAAGTT	exonic	ZNF44	nonsynonymous SNV	ZNF44:ENST00000393337.3:exon3:c.A188G:p.H63R,ZNF44:ENST00000397742.3:exon2:c.A44G:p.H15R,ZNF44:ENST00000356109.5:[illegible]
19	12769105	C	A	GTAACCGGTC,AGAACTGGTG	exonic	MAN2B1	nonsynonymous SNV	MAN2B1:ENST00000221363.4:exon9:c.G1160T:p.W387L,MAN2B1:ENST00000456935.2:exon9:c.G1163T:p.W388L, [illegible]
19	13322931	G	A	TTCTCTGCAG,GAGGCGGGGC	exonic	CACNA1A	nonsynonymous SNV	CACNA1A:ENST00000573710.2:exon43:c.C6292T:p.P2098S,CACNA1A:ENST00000360228.5:exon43:c.C6289T:p.P2097S,CACNA1A:[illegible]
19	14041067	G	C	TGCTGCAAAG,AGGCGCTCTA	exonic	CC2D1A	nonsynonymous SNV	CC2D1A:ENST00000318003.7:exon28:c.G2800C:p.E934Q,CC2D1A:ENST00000589606.1:exon28:c.G2797C:p.E933Q, [illegible]
19	14042759	C	T	TCCACCCCTC,GGTCACCCTC	exonic	PODNL1	nonsynonymous SNV	PODNL1:ENST00000587954.1:exon2:c.G314A:p.R105Q, [illegible]
19	14162744	C	T	GGAGAAAGTT,CTGATCCTGC	exonic	IL27RA	nonsynonymous SNV	IL27RA:ENST00000263379.2:exon13:c.C1675T:p.P559S, [illegible]
19	14165969	G	A	CCTGGGCTCA,AGGGGGACTC	exonic	PALM3	nonsynonymous SNV	PALM3:ENST00000589048.1:exon5:c.C317T:p.S106F,PALM3:ENST00000340790.4:exon6:c.C470T:p.S157F, [illegible]
19	16552786	G	A	GTGTATGCCG,ATCGACCTGA	exonic	EPS15L1	nonsynonymous SNV	EPS15L1:ENST00000596037.1:exon3:c.C82T:p.P28S,EPS15L1:ENST00000597937.1:exon3:c.C82T:p.P28S,EPS15L1:[illegible]
19	17286549	C	T	AAAACTTTAC,GGTGAGCAAG	exonic	MYO9B	nonsynonymous SNV	MYO9B:ENST00000595618.1:exon14:c.C2218T:p.R740C,MYO9B:ENST00000594824.1:exon14:c.C2218T:p.R740C,MYO9B:[illegible]
19	17305415	A	G	AAGGAGAGGG,AGCCCTGGAA	exonic	MYO9B	nonsynonymous SNV	MYO9B:ENST00000595618.1:exon22:c.A3179G:p.E1060G,MYO9B:ENST00000594824.1:exon22:c.A3179G:p.E1060G,MYO9B:[illegible]
19	17305418	C	T	GAGAGGGAAG,CCTGGAAGCC	exonic	MYO9B	nonsynonymous SNV	MYO9B:ENST00000595618.1:exon22:c.C3182T:p.A1061V,MYO9B:ENST00000594824.1:exon22:c.C3182T:p.A1061V,MYO9B:[illegible]
19	17533252	G	A	ACAGTGCCTG,ATACCTTCGA	exonic	MVB12A	nonsynonymous SNV	MVB12A:ENST00000317040.7:exon4:c.G398A:p.G133E,MVB12A:ENST00000529939.1:exon4:c.G398A:p.G133E,MVB12A:[illegible]
19	17766186	C	T	CTCTCTCGGC,TGAAACTGAG	exonic	UNC13A	nonsynonymous SNV	UNC13A:ENST00000550896.1:exon11:c.G1289A:p.R430K,UNC13A:ENST00000551649.1:exon11:c.G1289A:p.R430K,UNC13A:[illegible]
19	18092527	G	A	GCTGTTCATG,TGGACAACGG	exonic	KCNN1	nonsynonymous SNV	KCNN1:ENST00000222249.9:exon5:c.G508A:p.V170M, [illegible]
19	19337405	G	A	GCCCACCCTG,AGGAGGAAGA	exonic	NCAN	nonsynonymous SNV	NCAN:ENST00000252575.6:exon7:c.G1183A:p.E395K, [illegible]
19	19349139	G	A	GAGGGCATGG,AAGATGCCGA	exonic	NCAN	nonsynonymous SNV	NCAN:ENST00000252575.6:exon11:c.G3328A:p.E1110K,NCAN:ENST00000538881.1:exon5:c.G1681A:p.E561K, [illegible]
19	19368843	G	A	GTTCACGATG,GGTAGCGCGC	exonic	HAPLN4	nonsynonymous SNV	HAPLN4:ENST00000291481.7:exon5:c.C992T:p.P331L, [illegible]
19	19368981	G	A	TCCGGAGAAG,GTACAGGTCG	exonic	HAPLN4	nonsynonymous SNV	HAPLN4:ENST00000291481.7:exon5:c.C854T:p.P285L, [illegible]
19	19737675	C	T	GGGCAGGCGG,TGTGCAGCTG	exonic	LPAR2	nonsynonymous SNV	LPAR2:ENST00000542587.1:exon5:c.G419A:p.S140N,LPAR2:ENST00000586703.1:exon2:c.G419A:p.S140N,LPAR2:ENST00000407877.3:[illegible]
19	19902495	T	C	GGGGTTTCAC,ATGTTGGCCA	exonic	ZNF506	nonsynonymous SNV	ZNF506:ENST00000586260.1:exon5:c.A346G:p.S116G, [illegible]
19	20807532	A	C	ATGCGTAGTA,GGTCTGAAGA	exonic	ZNF626	nonsynonymous SNV	ZNF626:ENST00000601440.1:exon4:c.T1151G:p.L384R, [illegible]
19	20989992	A	G	AGACATAAGA,AATTCATACT	exonic	ZNF66	nonsynonymous SNV	ZNF66:ENST00000344519.8:exon4:c.A1586G:p.K529R, [illegible]
19	22363413	C	T	TTTGCCACAT,CTTCACATTT	exonic	ZNF676	nonsynonymous SNV	ZNF676:ENST00000397121.2:exon3:c.G1106A:p.G369E, [illegible]
19	22497243	C	T	TAAGATAATT,ATACTGGAAA	exonic	ZNF729	nonsynonymous SNV	ZNF729:ENST00000357491.6:exon4:c.C1024T:p.H342Y,ZNF729:ENST00000601693.1:exon4:c.C1024T:p.H342Y, [illegible]
19	30934929	G	A	CACGCACACG,GCGAGAAGCC	exonic	ZNF536	nonsynonymous SNV	ZNF536:ENST00000355537.3:exon2:c.G460A:p.G154S,ZNF536:ENST00000585628.1:exon4:c.G460A:p.G154S, [illegible]
19	34263543	G	A	CCCGGTCTTC,GCAAGGCCAT	exonic	CHST8	nonsynonymous SNV	CHST8:ENST00000438847.3:exon4:c.G850A:p.G284S,CHST8:ENST00000434302.1:exon5:c.G850A:p.G284S,CHST8:ENST00000262622.4:[illegible]
19	35512768	G	A	TCACACCTCG,GTACGTTCCG	exonic	GRAMD1A	nonsynonymous SNV	GRAMD1A:ENST00000504615.2:exon14:c.G1051A:p.G351S,GRAMD1A:ENST00000317991.5:exon15:c.G1753A:p.G585S,GRAMD1A:[illegible]
19	36223703	C	T	GGGCACGCCT,CTTCGGGGCC	exonic	KMT2B	nonsynonymous SNV	KMT2B:ENST00000222270.7:exon28:c.C6253T:p.P2085S,KMT2B:ENST00000420124.1:exon28:c.C6253T:p.P2085S, [illegible]
19	36223704	C	T	GGCACGCCTC,TTCGGGGCCA	exonic	KMT2B	nonsynonymous SNV	KMT2B:ENST00000222270.7:exon28:c.C6254T:p.P2085L,KMT2B:ENST00000420124.1:exon28:c.C6254T:p.P2085L, [illegible]
19	37037959	T	A	TTTTCTCCAG,ATGAATTCTC	exonic	ZNF529	nonsynonymous SNV	ZNF529:ENST00000591340.1:exon5:c.A1501T:p.T501S,ZNF529:ENST00000334116.7:exon6:c.A1186T:p.T396S, [illegible]
19	37309394	C	T	TCTTTAAATT,ATAGGATTTC	exonic	ZNF790	nonsynonymous SNV	ZNF790:ENST00000356725.4:exon5:c.G1852A:p.E618K, [illegible]
19	38378982	C	T	TTCCCCTCTT,CTGATTCAGT	exonic	WDR87	nonsynonymous SNV	WDR87:ENST00000303868.5:exon6:c.G5212A:p.E1738K,WDR87:ENST00000447313.2:exon6:c.G5329A:p.E1777K, [illegible]
19	38939337	C	T	CATGGGCCCC,CTGAGATCAA	exonic	RYR1	nonsynonymous SNV	RYR1:ENST00000359596.3:exon11:c.C1006T:p.P336S,RYR1:ENST00000360985.3:exon11:c.C1006T:p.P336S,RYR1:ENST00000355481.4:[illegible]
19	39002717	G	A	CCCAGGGACA,ACGCCCCAGC	exonic	RYR1	nonsynonymous SNV	RYR1:ENST00000359596.3:exon62:c.G9178A:p.D3060N,RYR1:ENST00000360985.3:exon62:c.G9178A:p.D3060N,RYR1:ENST00000355481.4:[illegible]
19	39297124	C	T	GGCTGGCCTC,GATGAAGTTG	exonic	LGALS4	nonsynonymous SNV	LGALS4:ENST00000307751.4:exon4:c.G451A:p.G151R, [illegible]
19	39971458	G	A	GGCGAAGAGG,AGCGAGTGGG	exonic	TIMM50	nonsynonymous SNV	TIMM50:ENST00000314349.4:exon1:c.G274A:p.E92K, [illegible]
19	40895365	G	A	AAGGGGCAGC,GGTCTGGTCC	exonic	HIPK4	nonsynonymous SNV	HIPK4:ENST00000291823.2:exon1:c.C445T:p.R149C, [illegible]
19	41223559	G	A	ATGGGCCCTG,ACAGAGCTGG	exonic	ITPKC	stopgain	ITPKC:ENST00000263370.2:exon1:c.G519A:p.W173X, [illegible]
19	42082655	G	A	TGTCCCCACA,AGAATGCATC	exonic	CEACAM21	nonsynonymous SNV	CEACAM21:ENST00000457737.1:exon1:c.G29A:p.R10K,CEACAM21:ENST00000187608.9:exon1:c.G29A:p.R10K,CEACAM21:[illegible]
19	42187818	C	T	CTGAGTAGAA,GAGGGTCCTG	exonic	CEACAM7	nonsynonymous SNV	CEACAM7:ENST00000401731.1:exon3:c.G604A:p.V202I,CEACAM7:ENST00000006724.3:exon3:c.G604A:p.V202I, [illegible]
19	42354495	G	A	GCCTGGGGAA,GAAAAAGCAG	exonic	DMRTC2	nonsynonymous SNV	DMRTC2:ENST00000596827.1:exon7:c.G871A:p.G291R, [illegible]
19	42471350	G	A	CCAGCTGCCT,AGACCCTGCT	exonic	ATP1A3	stopgain	ATP1A3:ENST00000441343.1:exon22:c.C3064T:p.Q1022X, [illegible]
19	42492203	T	G	CCGGCAAAAC,TGACCCACTC	exonic	ATP1A3	nonsynonymous SNV	ATP1A3:ENST00000473086.2:exon4:c.A152C:p.K51T,ATP1A3:ENST00000302102.5:exon4:c.A242C:p.K81T,ATP1A3:ENST00000543770.1:[illegible]
19	42583905	C	T	CCTGGCCCAC,GGCGAGCCCA	exonic	ZNF574	nonsynonymous SNV	ZNF574:ENST00000600245.1:exon2:c.C1147T:p.R383W,ZNF574:ENST00000359044.4:exon2:c.C1147T:p.R383W,ZNF574:[illegible]
19	42794410	C	T	GTGTTTTCAC,TGTGATCCGT	exonic	CIC	nonsynonymous SNV	CIC:ENST00000575354.2:exon10:c.C1490T:p.P497L,CIC:ENST00000160740.3:exon10:c.C1490T:p.P497L,CIC:ENST00000572681.2:[illegible]
19	42906092	C	G	TGGCGCGTCT,GCGGCACAGC	exonic	LIPE	nonsynonymous SNV	LIPE:ENST00000244289.4:exon10:c.G3103C:p.E1035Q, [illegible]
19	43031351	G	T	CCCTGGGGTA,CTTGTTGAGT	exonic	CEACAM1	nonsynonymous SNV	CEACAM1:ENST00000161559.6:exon2:c.C266A:p.A89D,CEACAM1:ENST00000471298.2:exon2:c.C146A:p.A49D,CEACAM1:[illegible]
19	44251998	G	A	TTCTCCAGAG,GGCTGGAGCA	exonic	SMG9	nonsynonymous SNV	SMG9:ENST00000602222.1:exon4:c.C277T:p.P93S,SMG9:ENST00000601170.1:exon4:c.C277T:p.P93S,SMG9:ENST00000270066.6:[illegible]
19	44590487	C	T	TGGGGAGAAG,CATTCAAATG	exonic	ZNF284	nonsynonymous SNV	ZNF284:ENST00000421176.3:exon5:c.C856T:p.P286S, [illegible]
19	44590488	C	T	GGGGAGAAGC,ATTCAAATGT	exonic	ZNF284	nonsynonymous SNV	ZNF284:ENST00000421176.3:exon5:c.C857T:p.P286L, [illegible]
19	45261578	C	T	CGGGCTCCTC,CGCTGGTGCG	exonic	BCL3	nonsynonymous SNV	BCL3:ENST00000164227.5:exon7:c.C967T:p.P323S, [illegible]
19	45261579	C	T	GGGCTCCTCC,GCTGGTGCGC	exonic	BCL3	nonsynonymous SNV	BCL3:ENST00000164227.5:exon7:c.C968T:p.P323L, [illegible]
19	45389406	C	T	CAGCTCTTCA,TCTGGGGGCC	exonic	PVRL2	nonsynonymous SNV	PVRL2:ENST00000252483.5:exon8:c.C1277T:p.T426I, [illegible]
19	45412495	C	G	CTGTGCCCAG,GACAATCACT	exonic	APOE	nonsynonymous SNV	APOE:ENST00000252486.4:exon4:c.C942G:p.S314R, [illegible]
19	45528945	G	A	GACCAGCAGG,ACAGATGCGC	exonic	RELB	nonsynonymous SNV	RELB:ENST00000505236.1:exon6:c.G827A:p.G276E,RELB:ENST00000540120.1:exon7:c.G836A:p.G279E,RELB:ENST00000221452.8:[illegible]
19	45535848	C	A	TGACTTCTCC,AGGCCGACGT	exonic	RELB	nonsynonymous SNV	RELB:ENST00000505236.1:exon8:c.C1039A:p.Q347K,RELB:ENST00000540120.1:exon9:c.C1048A:p.Q350K,RELB:ENST00000221452.8:[illegible]
19	47240098	C	T	AAGCTGCCGC,CCTCCTTCCA	exonic	STRN4	nonsynonymous SNV	STRN4:ENST00000593979.2:exon4:c.G164A:p.G55E,STRN4:ENST00000596012.2:exon5:c.G164A:p.G55E,STRN4:ENST00000539396.1:[illegible]
19	47918147	G	A	GGGGTACTGG,GGGGGCCACC	exonic	MEIS3	nonsynonymous SNV	MEIS3:ENST00000560245.1:exon1:c.C49T:p.P17S,MEIS3:ENST00000557833.1:exon5:c.C608T:p.P203L, [illegible]
19	48480090	G	A	GTACCAGAAG,GAAATACACA	exonic	BSPH1	nonsynonymous SNV	BSPH1:ENST00000344839.3:exon5:c.C278T:p.P93L, [illegible]
19	48733883	G	A	GGCTCCACCC,GGCTGGATGC	exonic	CARD8	nonsynonymous SNV	CARD8:ENST00000520153.1:exon8:c.C697T:p.R233W,CARD8:ENST00000519940.1:exon10:c.C847T:p.R283W,CARD8:ENST00000520015.1:[illegible]
19	49494593	C	T	ATCCAGTGTC,TCTTCAGGGC	exonic	GYS1	nonsynonymous SNV	GYS1:ENST00000323798.3:exon2:c.G266A:p.R89K,GYS1:ENST00000263276.6:exon2:c.G266A:p.R89K, [illegible]
19	49930183	C	T	CCCAGAATTT,CTGAGACCCC	exonic	CTD-3148I10.	nonsynonymous SNV	CTD-3148I10.1:ENST00000576655.1:exon3:c.C496T:p.P166S, [illegible]
19	50154214	C	T	ACCCCCTGCC,CCTCCTCTGC	exonic	SCAF1	nonsynonymous SNV	SCAF1:ENST00000598359.1:exon7:c.C568T:p.P190S,SCAF1:ENST00000360565.3:exon7:c.C568T:p.P190S, [illegible]
19	50154235	C	T	ATCCTCCTCC,CTTCCCCTTC	exonic	SCAF1	nonsynonymous SNV	SCAF1:ENST00000598359.1:exon7:c.C589T:p.P197S,SCAF1:ENST00000360565.3:exon7:c.C589T:p.P197S, [illegible]
19	50154311	C	T	GCCCCACCTG,CCCCCGATTC	exonic	SCAF1	nonsynonymous SNV	SCAF1:ENST00000360565.3:exon7:c.C665T:p.A222V, [illegible]
19	50341901	C	G	GAGCCTATTT,TTTGTGGAAG	exonic	MED25	nonsynonymous SNV	MED25:ENST00000593767.1:exon5:c.C657G:p.F219L, [illegible]
19	50735219	C	T	CTCCCACTAC,GGTTCCTGAC	exonic	MYH14	nonsynonymous SNV	MYH14:ENST00000598205.1:exon10:c.C1006T:p.R336W,MYH14:ENST00000440075.2:exon10:c.C1006T:p.R336W,MYH14:[illegible]
19	51830444	G	A	GTGGTACAAG,ATGACAGACT	exonic	IGLON5	nonsynonymous SNV	IGLON5:ENST00000270642.8:exon6:c.G757A:p.D253N, [illegible]

19	52570038	A	T	TCCAGTATGA,TTCTCTGATG	exonic	ZNF841	nonsynonymous SNV	ZNF841:ENST00000359973.2:exon1:c.T749A:p.I250N,ZNF841:ENST00000389534.4:exon7:c.T1097A:p.I366N,ZNF841:ENST00000594295.1:exon6:c.T1097A:p.I366N,ZNF841:ENST00000426391.2:exon5:c.T749A:p.I250N,	19q13.41	.
19	53303954	G	A	CATTCATAAG,TTTCTCTCCA	exonic	ZNF28	nonsynonymous SNV	ZNF28:ENST00000457749.2:exon4:c.C1144T:p.P382S,ZNF28:ENST00000438150.2:exon2:c.C985T:p.P329S,ZNF28:ENST00000360272.4:exon3:c.C985T:p.P329S,ZNF28:ENST00000391783.2:exon3:c.C985T:p.P329S,ZNF28:ENST00000414252.2:exon3:c.C985T:p.P329S,	19q13.41	.
19	53770660	G	A	CCCACTCTAT,GAGATAGAAA	exonic	VN1R4	nonsynonymous SNV	VN1R4:ENST00000311170.4:exon1:c.C259T:p.H87Y,	19q13.42	.
19	54782255	C	T	TTAGGATATT,GTGTATTGAT	exonic	LILRB2	nonsynonymous SNV	LILRB2:ENST00000434421.1:exon6:c.G769A:p.E257K,LILRB2:ENST00000391749.4:exon7:c.G1117A:p.E373K,LILRB2:ENST00000391748.1:exon7:c.G1117A:p.E373K,LILRB2:ENST00000314446.5:exon7:c.G1117A:p.E373K,LILRB2:ENST00000391746.1:exon6:c.G1117A:p.E373K,	19q13.42	LI
19	55014156	C	T	CCTCACTGCT,TCCTGGGCCT	exonic	LAIR2	nonsynonymous SNV	LAIR2:ENST00000351841.2:exon1:c.C22T:p.L8F,LAIR2:ENST00000301202.2:exon1:c.C22T:p.L8F,	19q13.42	.
19	55087393	G	A	TCTGACCAAG,AGGGGGCAGG	exonic	LILRA2	nonsynonymous SNV	LILRA2:ENST00000391737.1:exon5:c.G1036A:p.E346K,LILRA2:ENST00000251377.3:exon7:c.G1072A:p.E358K,LILRA2:ENST00000391738.3:exon6:c.G1072A:p.E358K,LILRA2:ENST00000251376.3:exon6:c.G1072A:p.E358K,	19q13.42	.
19	55690467	T	G	AGTCCCCCAT,CCCACCCCAG	exonic	SYT5	nonsynonymous SNV	SYT5:ENST00000590851.1:exon1:c.A107C:p.E36A,	19q13.42	.
19	55690468	C	T	GTCCCCCATT,CCACCCCAGA	exonic	SYT5	nonsynonymous SNV	SYT5:ENST00000590851.1:exon1:c.G106A:p.E36K,	19q13.42	.
19	55697687	G	A	CTGTGGCAGG,GACCCTGGGT	exonic	PTPRH	nonsynonymous SNV	PTPRH:ENST00000263434.5:exon14:c.C2150T:p.P717L,PTPRH:ENST00000376350.3:exon16:c.C2684T:p.P895L,	19q13.42	.
19	55697688	G	A	TGTGGCAGGG,ACCCTGGGTT	exonic	PTPRH	nonsynonymous SNV	PTPRH:ENST00000263434.5:exon14:c.C2149T:p.P717S,PTPRH:ENST00000376350.3:exon16:c.C2683T:p.P895S,	19q13.42	.
19	55993879	C	T	GCCCCCGTCC,GCCGCCACCC	exonic	ZNF628	nonsynonymous SNV	ZNF628:ENST00000391718.2:exon3:c.C1307T:p.P436L,ZNF628:ENST00000598519.1:exon3:c.C1319T:p.P440L,	19q13.42	.
19	56329471	G	A	TTAAAACTCT,AAATTCCTTG	exonic	NLRP11	stopgain	NLRP11:ENST00000589093.1:exon2:c.C70T:p.Q24X,NLRP11:ENST00000360133.3:exon4:c.C70T:p.Q24X,NLRP11:ENST00000589824.2:exon1:c.C70T:p.Q24X,NLRP11:ENST00000593244.1:exon4:c.C70T:p.Q24X,NLRP11:ENST00000443188.1:exon4:c.C70T:p.Q24X,	19q13.43	.
19	56369493	C	T	GAACCCGATT,GGATCTGTGT	exonic	NLRP4	nonsynonymous SNV	NLRP4:ENST00000301295.6:exon3:c.C734T:p.S245L,NLRP4:ENST00000346986.5:exon3:c.C734T:p.S245L,NLRP4:ENST00000587891.1:exon1:c.C509T:p.S170L,	19q13.43	.
19	57335936	C	T	ACATCCGGCT,CTTAGTCAAG	exonic	PEG3	nonsynonymous SNV	PEG3:ENST00000594389.1:exon3:c.G88A:p.E30K,PEG3:ENST00000599577.1:exon3:c.G88A:p.E30K,PEG3:ENST00000326441.9:exon4:c.G88A:p.E30K,PEG3:ENST00000423103.2:exon3:c.G88A:p.E30K,PEG3:ENST00000599534.1:exon1:c.G88A:p.E30K,	19q13.43	.
19	57640425	G	A	TATGCTGAAG,AAATTGACAA	exonic	USP29	nonsynonymous SNV	USP29:ENST00000254181.4:exon4:c.G382A:p.E128K,USP29:ENST00000598197.1:exon1:c.G382A:p.E128K,	19q13.43	U
19	57765333	G	C	CCTTCTGTGA,AGCGCAGCGC	exonic	ZNF805	nonsynonymous SNV	ZNF805:ENST00000535550.1:exon4:c.G747C:p.E249D,ZNF805:ENST00000354309.4:exon3:c.G747C:p.E249D,ZNF805:ENST00000414468.2:exon4:c.G1146C:p.E382D,	19q13.43	.
19	58083626	G	A	ACACTCATAT,GCCTTTCTCC	exonic	ZNF416	nonsynonymous SNV	ZNF416:ENST00000196489.3:exon4:c.C1646T:p.P549L,	19q13.43	.
19	58101460	C	T	AAGGCAGGTT,ATCCACACAG	exonic	ZIK1	nonsynonymous SNV	ZIK1:ENST00000598689.1:exon3:c.C200T:p.S67L,ZIK1:ENST00000597850.1:exon4:c.C281T:p.S94L,ZIK1:ENST00000536878.2:exon3:c.C242T:p.S81L,ZIK1:ENST00000600053.1:exon4:c.C224T:p.S75L,ZIK1:ENST00000599456.1:exon3:c.C116T:p.S39L,	19q13.43	.
19	58196630	G	A	GCTGTGACAG,GTATGACCTT	exonic	ZNF551	nonsynonymous SNV	ZNF551:ENST00000356715.4:exon2:c.G34A:p.G12S,ZNF551:ENST00000282296.5:exon2:c.G82A:p.G28S,ZNF551:ENST00000596085.1:exon2:c.G34A:p.G12S,	19q13.43	.
19	58196631	G	A	CTGTGACAGG,TATGACCTTT	exonic	ZNF551	nonsynonymous SNV	ZNF551:ENST00000356715.4:exon2:c.G35A:p.G12D,ZNF551:ENST00000282296.5:exon2:c.G83A:p.G28D,ZNF551:ENST00000596085.1:exon2:c.G35A:p.G12D,	19q13.43	.
19	58258443	T	A	GCGGCCGCGC,GAGGCCCCCG	exonic	ZNF776	nonsynonymous SNV	ZNF776:ENST00000431353.1:exon1:c.T17A:p.L6Q,ZNF776:ENST00000317178.5:exon1:c.T17A:p.L6Q,	19q13.43	.
19	58379853	A	G	AGTGGCTCAC,CCTGTAATCC	exonic;splicin	ZNF814;ZNF8	synonymous SNV	ZNF814:ENST00000595894.1:exon3:c.T258C:p.G86G,ZNF814:ENST00000600634.1:exon3:c.T258C:p.G86G,	19q13.43	.
19	58438312	G	A	TGGTTCCTAA,GTGTCCCTTT	exonic	ZNF418	nonsynonymous SNV	ZNF418:ENST00000425570.3:exon5:c.C1300T:p.L434F,ZNF418:ENST00000595830.1:exon3:c.C1237T:p.L413F,ZNF418:ENST00000599852.1:exon2:c.C982T:p.L328F,ZNF418:ENST00000396147.1:exon4:c.C1237T:p.L413F,	19q13.43	Z
19	58551901	G	A	GTCCCAGAAA,AACCATCGCA	exonic	ZSCAN1	nonsynonymous SNV	ZSCAN1:ENST00000282326.1:exon4:c.G454A:p.E152K,	19q13.43	.
19	58551908	C	T	AAAGAACCAT,GCAGGTGAGC	exonic	ZSCAN1	nonsynonymous SNV	ZSCAN1:ENST00000282326.1:exon4:c.C461T:p.S154L,	19q13.43	Z
19	58596705	C	T	GGGGCGGCCT,CTCGCAGGCG	exonic	ZSCAN18	nonsynonymous SNV	ZSCAN18:ENST00000600404.1:exon7:c.G1048A:p.E350K,ZSCAN18:ENST00000240727.6:exon7:c.G880A:p.E294K,ZSCAN18:ENST00000601144.1:exon7:c.G880A:p.E294K,ZSCAN18:ENST00000433686.2:exon6:c.G571A:p.E191K,ZSCAN18:ENST00000421612.2:exon6:c.G472A:p.E158K,	19q13.43	Z
19	58722950	G	A	CCAGGATGTG,CTGTGGACTT	exonic	ZNF274	nonsynonymous SNV	ZNF274:ENST00000345813.3:exon7:c.G778A:p.A260T,ZNF274:ENST00000326804.4:exon8:c.G874A:p.A292T,ZNF274:ENST00000424679.2:exon5:c.G559A:p.A187T,	19q13.43	.
19	58868704	G	T	TCCTCTGGGA,AGGCGAAGGG	exonic	ZNF497	nonsynonymous SNV	ZNF497:ENST00000311044.3:exon3:c.C298A:p.L100I,ZNF497:ENST00000425453.3:exon2:c.C298A:p.L100I,	19q13.43	.
20	2945678	C	T	GTCAATTCTT,AGACTCTGAC	exonic	PTPRA	nonsynonymous SNV	PTPRA:ENST00000430705.1:exon5:c.C245T:p.S82L,PTPRA:ENST00000318266.5:exon6:c.C245T:p.S82L,PTPRA:ENST00000380393.3:exon9:c.C245T:p.S82L,PTPRA:ENST00000356147.3:exon5:c.C245T:p.S82L,PTPRA:ENST00000399903.2:exon5:c.C245T:p.S82L,PTPRA:ENST00000455631.1:exon3:c.C245T:p	20p13	.
20	3103028	G	C	AGATGAGGCA,ATGTGTACAT	exonic	UBOX5	nonsynonymous SNV	UBOX5:ENST00000217173.2:exon3:c.C257G:p.S86C,UBOX5:ENST00000348031.2:exon3:c.C257G:p.S86C,UBOX5:ENST00000449731.1:exon4:c.C257G:p.S86C,	20p13	.
20	3146669	C	A	GCCCGACCCC,CACCGCTGCT	exonic	LZTS3	nonsynonymous SNV	LZTS3:ENST00000337576.5:exon2:c.G797T:p.G266V,LZTS3:ENST00000329152.3:exon2:c.G797T:p.G266V,LZTS3:ENST00000360342.3:exon4:c.G797T:p.G266V,	20p13	.
20	5843889	G	C	ACTCAACTAA,AGGCCAGGAG	exonic	C20orf196	nonsynonymous SNV	C20orf196:ENST00000303142.6:exon3:c.G398C:p.R133T,C20orf196:ENST00000442185.1:exon4:c.G539C:p.R180T,	20p12.3	.
20	8665579	G	A	TTTCCTCCAG,ACAAATATCA	exonic	PLCB1	nonsynonymous SNV	PLCB1:ENST00000378641.3:exon10:c.G863A:p.G288E,PLCB1:ENST00000338037.6:exon10:c.G863A:p.G288E,PLCB1:ENST00000378637.2:exon10:c.G863A:p.G288E,	20p12.3	.
20	8689378	C	T	ATTCTCCTTT,GTTTGAGAAC	exonic	PLCB1	nonsynonymous SNV	PLCB1:ENST00000378641.3:exon12:c.C1229T:p.S410L,PLCB1:ENST00000338037.6:exon12:c.C1229T:p.S410L,PLCB1:ENST00000378637.2:exon12:c.C1229T:p.S410L,	20p12.3	PI
20	8703055	C	T	TGTAAAAAAT,TTCAATGGAT	exonic	PLCB1	nonsynonymous SNV	PLCB1:ENST00000378641.3:exon15:c.C1568T:p.S523F,PLCB1:ENST00000338037.6:exon15:c.C1568T:p.S523F,PLCB1:ENST00000378637.2:exon15:c.C1568T:p.S523F,	20p12.3	.
20	8745828	C	T	CAACAGAAAT,GTTTGTGAAA	exonic	PLCB1	nonsynonymous SNV	PLCB1:ENST00000378641.3:exon26:c.C2753T:p.S918L,PLCB1:ENST00000338037.6:exon26:c.C2753T:p.S918L,PLCB1:ENST00000378637.2:exon26:c.C2753T:p.S918L,	20p12.3	PI
20	10025085	T	A	AGAGAAGGGG,AGTGGAAATA	exonic	ANKEF1	nonsynonymous SNV	ANKEF1:ENST00000378380.3:exon4:c.T590A:p.V197E,ANKEF1:ENST00000378392.1:exon5:c.T590A:p.V197E,	20p12.2	.
20	13753237	C	G	TTCATCCTCT,CTTTCCAAAT	exonic	ESF1	nonsynonymous SNV	ESF1:ENST00000202816.1:exon5:c.G1174C:p.E392Q,	20p12.1	.
20	22562934	G	A	TCCTGGCACG,GGAGGCGCTC	exonic	FOXA2	nonsynonymous SNV	FOXA2:ENST00000377115.4:exon3:c.C928T:p.P310S,FOXA2:ENST00000419308.2:exon2:c.C946T:p.P316S,	20p11.21	.
20	25205864	C	T	GGAGACACAG,CGCAGAGCAG	exonic	ENTPD6	nonsynonymous SNV	ENTPD6:ENST00000376652.4:exon14:c.C1267T:p.P423S,ENTPD6:ENST00000433259.2:exon13:c.C1208T:p.A403V,ENTPD6:ENST00000354989.5:exon13:c.C1216T:p.P406S,ENTPD6:ENST00000360031.2:exon14:c.C1264T:p.P422S,	20p11.21	.
20	25457019	C	T	TCAGCCTGTC,CCTGCACGAA	exonic	NINL	nonsynonymous SNV	NINL:ENST00000278886.6:exon17:c.G2908A:p.G970R,	20p11.21	.
20	25491340	C	T	TTCTGAGCTT,TTTAGTGCTC	exonic	NINL	nonsynonymous SNV	NINL:ENST00000278886.6:exon5:c.G487A:p.E163K,NINL:ENST00000422516.1:exon5:c.G487A:p.E163K,	20p11.21	.
20	30142584	G	C	GAAAGGCCTC,AAGCAAACAA	exonic	HM13	nonsynonymous SNV	HM13:ENST00000398174.3:exon8:c.G760C:p.E254Q,HM13:ENST00000340852.5:exon8:c.G760C:p.E254Q,HM13:ENST00000344042.5:exon7:c.G634C:p.E212Q,HM13:ENST00000335574.5:exon8:c.G760C:p.E254Q,HM13:ENST00000376127.3:exon7:c.G634C:p.E212Q,	20q11.21	.
20	30662469	G	A	CACCCGGAAG,AGGGCTACAT	exonic	HCK	nonsynonymous SNV	HCK:ENST00000520553.1:exon5:c.G310A:p.E104K,HCK:ENST00000534862.1:exon6:c.G313A:p.E105K,HCK:ENST00000518730.1:exon5:c.G307A:p.E103K,HCK:ENST00000375862.2:exon5:c.G370A:p.E124K,HCK:ENST00000538448.1:exon6:c.G310A:p.E104K,HCK:ENST00000375852.2:exon5:c.G373A:p.E125K	20q11.21	H
20	31017823	C	T	GGACCCTGCC,CGCTCCTGAG	exonic	ASXL1	nonsynonymous SNV	ASXL1:ENST00000306058.5:exon7:c.C670T:p.P224S,ASXL1:ENST00000375687.4:exon8:c.C685T:p.P229S,	20q11.21	.
20	31812219	G	A	ATTCCATAAG,AGTGGTTCTC	exonic	BPIFA3	nonsynonymous SNV	BPIFA3:ENST00000375454.3:exon3:c.G337A:p.E113K,	20q11.21	.
20	31873886	G	A	GAAGATGGCC,GCCCGTGGAC	exonic	BPIFB1	nonsynonymous SNV	BPIFB1:ENST00000253354.1:exon2:c.G7A:p.G3S,BPIFB1:ENST00000423645.1:exon2:c.G7A:p.G3S,	20q11.21	.
20	32267663	G	A	CAGCACCTCG,CAGCCCAGTT	exonic	E2F1	nonsynonymous SNV	E2F1:ENST00000343380.5:exon3:c.C470T:p.A157V,	20q11.22	.
20	33450616	C	T	CTGCTCCTTA,CCGCCCAGGC	splicing	GGT7(ENST0	.	.	20q11.22	.
20	33875576	G	A	GTGGGGCTTG,GACATCAGGC	exonic	FAM83C	nonsynonymous SNV	FAM83C:ENST00000374408.3:exon4:c.C1006T:p.P336S,	20q11.22	.
20	34785929	C	T	CCCGCCTCCC,CTCCCCCAAG	exonic	EPB41L1	nonsynonymous SNV	EPB41L1:ENST00000338074.2:exon14:c.C1634T:p.P545L,EPB41L1:ENST00000373941.1:exon13:c.C1634T:p.P545L,EPB41L1:ENST00000373950.2:exon13:c.C1307T:p.P436L,EPB41L1:ENST00000454226.1:exon1:c.C128T:p.P43L,EPB41L1:ENST00000202028.5:exon13:c.C1412T:p.P471L,EPB41L1:ENST0000	20q11.23	.
20	36997744	G	C	CCCCTATATG,AGATAGATGC	exonic	LBP	nonsynonymous SNV	LBP:ENST00000217407.2:exon10:c.G1087C:p.E363Q,	20q11.23	LE
20	40049838	G	A	TTCAAGGAAG,GGAAGCTAAA	exonic	CHD6	nonsynonymous SNV	CHD6:ENST00000373233.3:exon31:c.C5437T:p.P1813S,	20q12	.
20	42788523	C	T	ACGCCGAAGC,CGAGCGTTTG	exonic	JPH2	nonsynonymous SNV	JPH2:ENST00000372980.3:exon2:c.G904A:p.G302S,	20q13.12	.
20	43254269	C	T	GTCTCGCTCC,CCTCCTGCAG	exonic	ADA	nonsynonymous SNV	ADA:ENST00000537820.1:exon5:c.G419A:p.G140E,ADA:ENST00000372874.4:exon5:c.G419A:p.G140E,ADA:ENST00000536532.1:exon5:c.G419A:p.G140E,	20q13.12	.
20	43804687	C	T	GTTGAATCCC,CTAACCGCTG	exonic	PI3	nonsynonymous SNV	PI3:ENST00000243924.3:exon2:c.C265T:p.P89S,	20q13.12	.
20	45644852	C	T	GAACCATTCC,CTGGCCAGAG	exonic	EYA2	nonsynonymous SNV	EYA2:ENST00000458636.1:exon3:c.C190T:p.P64S,EYA2:ENST00000327619.5:exon5:c.C331T:p.P111S,EYA2:ENST00000357410.3:exon5:c.C331T:p.P111S,EYA2:ENST00000317304.6:exon4:c.C331T:p.P111S,	20q13.12	.
20	47282875	C	T	ACTGCCTCCT,CCGAGTCTGG	exonic	PREX1	nonsynonymous SNV	PREX1:ENST00000371941.3:exon15:c.G1684A:p.E562K,PREX1:ENST00000396220.1:exon15:c.G1684A:p.E562K,	20q13.13	.
20	49575912	A	C	GTGGTGGCTG,CTGCTCGGAC	exonic	MOCS3	nonsynonymous SNV	MOCS3:ENST00000244051.1:exon1:c.A533C:p.D178A,	20q13.13	.
20	50408460	C	T	ACCTTGGTGC,CCGTAGTGCC	exonic	SALL4	nonsynonymous SNV	SALL4:ENST00000395997.3:exon2:c.G562A:p.G188S,SALL4:ENST00000217086.4:exon2:c.G562A:p.G188S,	20q13.2	.
20	52645001	G	A	TTGATGCTCT,CCCTCTTGGC	exonic	BCAS1	nonsynonymous SNV	BCAS1:ENST00000411563.1:exon2:c.C362T:p.A121V,BCAS1:ENST00000371435.2:exon4:c.C653T:p.A218V,BCAS1:ENST00000448484.1:exon1:c.C239T:p.A80V,BCAS1:ENST00000395961.3:exon4:c.C653T:p.A218V,BCAS1:ENST00000371440.3:exon4:c.C653T:p.A218V,	20q13.2	.
20	57428897	G	A	AGGTGCTGCA,GGGTCCCCGG	exonic	GNAS	nonsynonymous SNV	GNAS:ENST00000371100.4:exon1:c.G577A:p.G193R,GNAS:ENST00000371102.4:exon1:c.G577A:p.G193R,GNAS:ENST00000371099.2:exon1:c.G577A:p.G193R,	20q13.32	.
20	57428898	G	A	GGTGCTGCAG,GGTCCCCGGA	exonic	GNAS	nonsynonymous SNV	GNAS:ENST00000371100.4:exon1:c.G578A:p.G193E,GNAS:ENST00000306120.3:exon1:c.G388A:p.G130R,GNAS:ENST00000371102.4:exon1:c.G578A:p.G193E,GNAS:ENST00000371099.2:exon1:c.G578A:p.G193E,	20q13.32	.
20	62194195	G	A	TTCTCCTCGA,GAAGGCGGCC	exonic	HELZ2	nonsynonymous SNV	HELZ2:ENST00000427522.2:exon3:c.C4273T:p.L1425F,HELZ2:ENST00000467148.1:exon8:c.C5980T:p.L1994F,	20q13.33	.
20	62366096	C	T	ACCTGCAGCT,TGCCTTTGCT	exonic	RP4-583P15.	nonsynonymous SNV	ZGPAT:ENST00000357119.4:exon5:c.C884T:p.S295F,ZGPAT:ENST00000328969.5:exon5:c.C971T:p.S324F,ZGPAT:ENST00000369967.3:exon5:c.C911T:p.S304F,ZGPAT:ENST00000355969.6:exon5:c.C911T:p.S304F,ZGPAT:ENST00000448100.2:exon5:c.C911T:p.S304F,	20q13.33	Z
20	62660744	G	C	CCTGTGGTCT,AGGCCATCTT	exonic	PRPF6	nonsynonymous SNV	PRPF6:ENST00000535781.1:exon18:c.G2326C:p.E776Q,PRPF6:ENST00000266079.4:exon19:c.G2446C:p.E816Q,	20q13.33	.
21	16340039	C	T	TATCCTTGCT,CTTGAGGCTC	exonic	NRIP1	nonsynonymous SNV	NRIP1:ENST00000400202.1:exon3:c.G475A:p.E159K,NRIP1:ENST00000318948.4:exon4:c.G475A:p.E159K,NRIP1:ENST00000400199.1:exon3:c.G475A:p.E159K,	21q11.2	.
21	30338844	G	A	TGCACCGCAG,ATTTTTTTGT	exonic	LTN1	nonsynonymous SNV	LTN1:ENST00000361371.5:exon10:c.C1969T:p.P657S,LTN1:ENST00000389194.2:exon10:c.C2107T:p.P703S,LTN1:ENST00000389195.2:exon10:c.C2107T:p.P703S,	21q21.3	.
21	31965045	G	T	TTCTGTGGCT,TGGCTATAGG	exonic	KRTAP6-3	nonsynonymous SNV	KRTAP6-3:ENST00000391624.1:exon1:c.G260T:p.C87F,	21q22.11	.
21	34166213	G	A	CTGTTGAAAA,AGCCATATCT	exonic	C21orf62	nonsynonymous SNV	C21orf62:ENST00000382373.4:exon3:c.C661T:p.L221F,C21orf62:ENST00000487113.1:exon2:c.C520T:p.L174F,C21orf62:ENST00000490358.1:exon3:c.C520T:p.L174F,C21orf62:ENST00000536776.1:exon2:c.C520T:p.L174F,C21orf62:ENST00000479548.1:exon4:c.C520T:p.L174F,	21q22.11	.
21	36164625	G	A	GCCCACCATG,AGAACTGGTA	exonic	RUNX1	nonsynonymous SNV	RUNX1:ENST00000325074.5:exon7:c.C1214T:p.S405F,RUNX1:ENST00000300305.3:exon8:c.C1250T:p.S417F,RUNX1:ENST00000344691.4:exon6:c.C1169T:p.S390F,RUNX1:ENST00000399240.1:exon5:c.C977T:p.S326F,RUNX1:ENST00000437180.1:exon9:c.C1250T:p.S417F,	21q22.12	.
21	43413939	G	A	TAGAGAGGAA,AATAAATGTA	exonic	ZBTB21	nonsynonymous SNV	ZBTB21:ENST00000398505.3:exon3:c.C266T:p.S89F,ZBTB21:ENST00000398511.3:exon2:c.C266T:p.S89F,ZBTB21:ENST00000310826.5:exon3:c.C266T:p.S89F,ZBTB21:ENST00000425521.1:exon2:c.C266T:p.S89F,ZBTB21:ENST00000398499.1:exon4:c.C266T:p.S89F,	21q22.3	.
21	43640092	C	T	ATGCGCATTT,ACTTCCCCGA	exonic	ABCG1	nonsynonymous SNV	ABCG1:ENST00000343687.3:exon1:c.C11T:p.S4L,	21q22.3	.
21	43967237	C	T	AGGTGCTCCT,CACCCTGGTG	exonic	SLC37A1	nonsynonymous SNV	SLC37A1:ENST00000398341.3:exon10:c.C755T:p.S252F,SLC37A1:ENST00000352133.2:exon9:c.C755T:p.S252F,	21q22.3	.
21	44480572	G	A	CACTGAGTCG,GCAGAATGAC	exonic	CBS	nonsynonymous SNV	CBS:ENST00000398158.1:exon12:c.C1124T:p.P375L,CBS:ENST00000398168.1:exon12:c.C1124T:p.P375L,CBS:ENST00000359624.3:exon12:c.C1124T:p.P375L,CBS:ENST00000398165.3:exon12:c.C1124T:p.P375L,CBS:ENST00000544202.1:exon10:c.C860T:p.P287L,CBS:ENST00000352178.5:exon12:c.C1124	21q22.3	.
21	44480573	G	A	ACTGAGTCGG,CAGAATGACC	exonic	CBS	nonsynonymous SNV	CBS:ENST00000398158.1:exon12:c.C1123T:p.P375S,CBS:ENST00000398168.1:exon12:c.C1123T:p.P375S,CBS:ENST00000359624.3:exon12:c.C1123T:p.P375S,CBS:ENST00000398165.3:exon12:c.C1123T:p.P375S,CBS:ENST00000544202.1:exon10:c.C859T:p.P287S,CBS:ENST00000352178.5:exon12:c.C112	21q22.3	.
21	45877101	G	A	TCTGGATGCG,GTGCCAGCCT	exonic	LRRC3	nonsynonymous SNV	LRRC3:ENST00000291592.4:exon2:c.G574A:p.G192S,	21q22.3	.
21	45919734	C	T	AGGTAGGCAC,AGCCGTGGTG	exonic	TSPEAR	nonsynonymous SNV	TSPEAR:ENST00000323084.4:exon12:c.G1942A:p.G648S,	21q22.3	.
21	45978132	G	A	GGACACGGAG,AGGAGGGTCT	exonic	KRTAP10-3	nonsynonymous SNV	KRTAP10-3:ENST00000391620.1:exon1:c.C467T:p.S156F,	21q22.3	.
21	46078042	C	A	GTGGCTCCCT,CTGCCAGCCC	exonic	KRTAP12-3	nonsynonymous SNV	KRTAP12-3:ENST00000397907.1:exon1:c.C146A:p.S49Y,	21q22.3	KI
21	46888617	C	T	GCCGCTTGCT,GGTCCTCACG	exonic	COL18A1	nonsynonymous SNV	COL18A1:ENST00000359759.4:exon2:c.C1813T:p.R605W,COL18A1:ENST00000400337.2:exon3:c.C568T:p.R190W,COL18A1:ENST00000355480.5:exon2:c.C1108T:p.R370W,	21q22.3	.
21	47423345	C	A	TCCTGCTGGA,GGCTCCGCCA	exonic	COL6A1	nonsynonymous SNV	COL6A1:ENST00000361866.3:exon35:c.C2505A:p.D835E,	21q22.3	.
21	47538576	G	A	AGAAATCGGG,CCAAGGGAAG	exonic	COL6A2	nonsynonymous SNV	COL6A2:ENST00000310645.5:exon13:c.G1165A:p.A389T,COL6A2:ENST00000357838.4:exon13:c.G1165A:p.A389T,COL6A2:ENST00000300527.4:exon13:c.G1165A:p.A389T,COL6A2:ENST00000409416.1:exon12:c.G1165A:p.A389T,COL6A2:ENST00000397763.1:exon12:c.G1165A:p.A389T,	21q22.3	.
21	47602589	G	A	CTGGGTGGGA,CAGGTCGTGG	exonic	SPATC1L	nonsynonymous SNV	SPATC1L:ENST00000291672.5:exon2:c.C142T:p.L48F,	21q22.3	.
21	47845811	C	T	CCTTGCACCC,CAAGCGGCGA	exonic	PCNT	nonsynonymous SNV	PCNT:ENST00000359568.5:exon33:c.C7246T:p.P2416S,	21q22.3	.
22	18029232	C	T	AGGACCTTTC,AGGAAATGTA	exonic	CECR2	stopgain	CECR2:ENST00000400585.2:exon17:c.C3763T:p.Q1255X,CECR2:ENST00000400573.5:exon17:c.C4189T:p.Q1397X,CECR2:ENST00000262608.8:exon16:c.C4192T:p.Q1398X,CECR2:ENST00000355219.3:exon1:c.C544T:p.Q182X,	22q11.21	.
22	18304206	C	T	TGTTGAGGGG,TGTGGACTGG	exonic	MICAL3	nonsynonymous SNV	MICAL3:ENST00000441493.2:exon25:c.G3518A:p.S1173N,	22q11.21	.
22	22869098	C	T	CTGCCCATCT,CTTTATGCTG	exonic	ZNF280A	nonsynonymous SNV	ZNF280A:ENST00000302097.3:exon2:c.G857A:p.G286E,	22q11.22	.
22	25120860	G	A	CTACTTGTTA,GAGAGATGGT	exonic	PIWIL3	nonsynonymous SNV	PIWIL3:ENST00000527701.1:exon19:c.C1898T:p.P633L,PIWIL3:ENST00000332271.5:exon18:c.C2252T:p.P751L,PIWIL3:ENST00000533313.1:exon19:c.C1898T:p.P633L,	22q11.23	.
22	25120861	G	A	TACTTGTTAG,AGAGATGGTT	exonic	PIWIL3	nonsynonymous SNV	PIWIL3:ENST00000527701.1:exon19:c.C1897T:p.P633S,PIWIL3:ENST00000332271.5:exon18:c.C2251T:p.P751S,PIWIL3:ENST00000533313.1:exon19:c.C1897T:p.P633S,	22q11.23	.
22	26243614	G	A	CGTCTGCATA,GACAGGTAAG	exonic	MYO18B	nonsynonymous SNV	MYO18B:ENST00000407587.2:exon20:c.G3773A:p.R1258K,MYO18B:ENST00000335473.7:exon20:c.G3770A:p.R1257K,MYO18B:ENST00000536101.1:exon20:c.G3770A:p.R1257K,	22q12.1	.
22	26423481	C	T	TCCTCCGGCT,CATCGTGTCC	exonic	MYO18B	nonsynonymous SNV	MYO18B:ENST00000407587.2:exon43:c.C7544T:p.S2515F,MYO18B:ENST00000335473.7:exon43:c.C7541T:p.S2514F,MYO18B:ENST00000540454.1:exon1:c.C467T:p.S156F,MYO18B:ENST00000536101.1:exon43:c.C7541T:p.S2514F,	22q12.1	.
22	30682282	G	A	CATGACAATG,AGATATAACC	exonic	GATSL3	nonsynonymous SNV	GATSL3:ENST00000407689.3:exon6:c.C713T:p.S238F,	22q12.2	.
22	32112197	G	A	GCTGACTAAG,AGTTACAGTC	exonic	PRR14L	nonsynonymous SNV	PRR14L:ENST00000434485.1:exon4:c.C1628T:p.S543F,PRR14L:ENST00000327423.6:exon4:c.C1628T:p.S543F,PRR14L:ENST00000397493.2:exon4:c.C1628T:p.S543F,	22q12.2	.
22	37425295	C	T	CGTGAACATC,CCTTCACAGA	exonic	MPST	nonsynonymous SNV	MPST:ENST00000397225.2:exon3:c.C634T:p.P212S,MPST:ENST00000401419.3:exon3:c.C634T:p.P212S,MPST:ENST00000341116.3:exon4:c.C634T:p.P212S,MPST:ENST00000397129.1:exon3:c.C694T:p.P232S,MPST:ENST00000429360.2:exon3:c.C634T:p.P212S,MPST:ENST00000404802.3:exon4:c.C634T:p	22q12.3	.
22	38318107	C	T	AGGCCTGGGC,CTTCTCACAG	exonic	MICALL1	nonsynonymous SNV	MICALL1:ENST00000445494.1:exon6:c.C446T:p.P149L,MICALL1:ENST00000215957.6:exon6:c.C698T:p.P233L,	22q13.1	.
22	39530529	G	A	TTGGGCCCGC,GGGCGGGAAC	exonic	CBX7	nonsynonymous SNV	CBX7:ENST00000216133.5:exon5:c.C475T:p.R159C,	22q13.1	.
22	40080402	C	T	CCAGAAAGGC,CAGAAAAGGG	exonic	CACNA1I	nonsynonymous SNV	CACNA1I:ENST00000404898.1:exon35:c.C5821T:p.P1941S,CACNA1I:ENST00000336649.4:exon39:c.C5944T:p.P1982S,CACNA1I:ENST00000407673.1:exon35:c.C5821T:p.P1941S,CACNA1I:ENST00000402142.3:exon36:c.C5926T:p.P1976S,CACNA1I:ENST00000401624.1:exon36:c.C5926T:p.P1976S,CACNA1I	22q13.1	.
22	42915753	C	T	ATACGGTCCT,CGGGTCCCGC	exonic	RRP7A	nonsynonymous SNV	RRP7A:ENST00000323013.6:exon1:c.G40A:p.E14K,RRP7A:ENST00000416699.1:exon1:c.G40A:p.E14K,	22q13.2	.
22	45937171	G	C	CATCAACACA,AGGGCTCCTA	exonic	FBLN1	nonsynonymous SNV	FBLN1:ENST00000262722.7:exon9:c.G985C:p.E329Q,FBLN1:ENST00000442170.2:exon9:c.G985C:p.E329Q,FBLN1:ENST00000348697.2:exon9:c.G985C:p.E329Q,FBLN1:ENST00000402984.3:exon10:c.G1099C:p.E367Q,FBLN1:ENST00000327858.6:exon9:c.G985C:p.E329Q,FBLN1:ENST00000340923.5:exon9:	22q13.31	.
22	47185264	C	T	CCTCGTGATC,GCCCGCCTCG	exonic	TBC1D22A	nonsynonymous SNV	TBC1D22A:ENST00000441936.1:exon2:c.C118T:p.R40C,	22q13.31	.
22	50898421	G	A	GGAGAAATGC,GAAGGGCTCA	exonic	SBF1	nonsynonymous SNV	SBF1:ENST00000348911.6:exon26:c.C3454T:p.R1152C,SBF1:ENST00000380817.3:exon26:c.C3451T:p.R1151C,SBF1:ENST00000390679.3:exon26:c.C3451T:p.R1151C,	22q13.33	.
X	228294	C	T	CGTGGTCTTT,CTAGGAGGGC	exonic	GTPBP6	nonsynonymous SNV	GTPBP6:ENST00000326153.4:exon3:c.G230A:p.G77E,	Xp22.33	.
X	1424536	C	T	ACCGCAGCGT,ACCACCGGTG	exonic	CSF2RA	nonsynonymous SNV	CSF2RA:ENST00000381509.3:exon13:c.C1141T:p.H381Y,	Xp22.33	.
X	19444423	G	A	ATTAATTCCC,AATAGAGGGA	exonic	MAP3K15	nonsynonymous SNV	MAP3K15:ENST00000338883.4:exon8:c.C1205T:p.S402L,MAP3K15:ENST00000469203.2:exon6:c.C701T:p.S234L,	Xp22.12	.
X	41437760	C	T	TGTGCCACTA,GTCGTGAGTC	exonic	CASK	nonsynonymous SNV	CASK:ENST00000378154.1:exon15:c.G1336A:p.V446I,CASK:ENST00000318588.9:exon15:c.G1336A:p.V446I,CASK:ENST00000378163.1:exon15:c.G1336A:p.V446I,CASK:ENST00000361962.4:exon15:c.G1336A:p.V446I,CASK:ENST00000378166.4:exon15:c.G1336A:p.V446I,CASK:ENST00000421587.2:exon14:	Xp11.4	.
X	47065778	C	T	CCAGGACCCA,CTGAGAAGTC	exonic	UBA1	nonsynonymous SNV	UBA1:ENST00000377351.4:exon16:c.C1873T:p.P625S,UBA1:ENST00000335972.6:exon16:c.C1873T:p.P625S,	Xp11.23	.
X	49455951	C	T	TGAACCAGTT,CTGGCTATCA	exonic	PAGE1	nonsynonymous SNV	PAGE1:ENST00000376150.3:exon4:c.G193A:p.E65K,	Xp11.23	.
X	53285109	G	A	CTGAGCCCAG,GAAGGCCCAC	exonic	IQSEC2	nonsynonymous SNV	IQSEC2:ENST00000396435.3:exon3:c.C872T:p.P291L,IQSEC2:ENST00000375368.5:exon2:c.C842T:p.P281L,IQSEC2:ENST00000375365.2:exon3:c.C257T:p.P86L,	Xp11.22	.
X	65408329	C	T	CTGCTCAGAT,CTGCTTCAGT	exonic	HEPH	nonsynonymous SNV	HEPH:ENST00000425114.1:exon4:c.C754T:p.P252S,HEPH:ENST00000343002.2:exon4:c.C754T:p.P252S,HEPH:ENST00000374727.3:exon5:c.C763T:p.P255S,HEPH:ENST00000419594.1:exon5:c.C763T:p.P255S,HEPH:ENST00000441993.2:exon5:c.C763T:p.P255S,HEPH:ENST00000519389.1:exon5:c.C916T:p	Xq12	.
X	70354310	C	T	GACATGCAGT,CAACAAGTAA	exonic	MED12	nonsynonymous SNV	MED12:ENST00000333646.6:exon34:c.C4721T:p.S1574F,MED12:ENST00000374080.3:exon34:c.C4721T:p.S1574F,MED12:ENST00000374102.1:exon34:c.C4721T:p.S1574F,	Xq13.1	.
X	70617204	C	T	CAAGATTTAT,GCACGTTTCG	exonic	TAF1	nonsynonymous SNV	TAF1:ENST00000449580.1:exon23:c.C3505T:p.R1169C,TAF1:ENST00000373790.4:exon23:c.C3505T:p.R1169C,TAF1:ENST00000423759.1:exon23:c.C3568T:p.R1190C,TAF1:ENST00000276072.3:exon23:c.C3568T:p.R1190C,	Xq13.1	T
X	83724567	T	C	CTTTCTTCTC,TATTGCCAAC	exonic	HDX	nonsynonymous SNV	HDX:ENST00000297977.5:exon3:c.A164G:p.K55R,HDX:ENST00000373177.2:exon4:c.A164G:p.K55R,	Xq21.1	.
X	90691547	C	T	GAATTTTCTT,CTTTGGGTCA	exonic	PABPC5	nonsynonymous SNV	PABPC5:ENST00000312600.3:exon2:c.C971T:p.S324F,PABPC5:ENST00000373105.1:exon2:c.C479T:p.S160F,	Xq21.31	.
X	101857428	C	T	AGTACTGTAA,CAAGTCTGAA	exonic	ARMCX5	nonsynonymous SNV	ARMCX5:ENST00000604957.1:exon1:c.C359T:p.T120I,ARMCX5:ENST00000537008.1:exon6:c.C359T:p.T120I,ARMCX5:ENST00000541409.1:exon5:c.C359T:p.T120I,ARMCX5:ENST00000246174.2:exon6:c.C359T:p.T120I,ARMCX5:ENST00000536530.1:exon3:c.C359T:p.T120I,ARMCX5:ENST00000372742.1:exo	Xq22.1	.
X	107976574	G	A	GCACTGAGGG,AAGGGGAGGA	exonic	IRS4	nonsynonymous SNV	IRS4:ENST00000372129.2:exon1:c.C3001T:p.P1001S,	Xq22.3	.
X	125954750	T	A	AGCCAGAGGA,GACGCAGAGC	exonic	CXorf64	nonsynonymous SNV	CXorf64:ENST00000371125.3:exon2:c.T129A:p.D43E,	Xq25	.
X	138687074	C	T	AAGAGAATGT,CTTTTTATTT	exonic	MCF2	nonsynonymous SNV	MCF2:ENST00000519895.1:exon18:c.G1855A:p.D619N,MCF2:ENST00000414978.1:exon17:c.G1807A:p.D603N,MCF2:ENST00000536274.1:exon13:c.G1510A:p.D504N,MCF2:ENST00000370576.4:exon14:c.G1627A:p.D543N,MCF2:ENST00000446225.1:exon5:c.G436A:p.D146N,MCF2:ENST00000370573.4:exon	Xq27.1	.
X	139865880	G	A	CCAAATCCAG,TCTTCCAGTC	exonic	CDR1	nonsynonymous SNV	CDR1:ENST00000370532.2:exon1:c.C652T:p.P218S,	Xq27.1	.
X	151821178	G	A	GGCCCCCCTG,CCACTGGAGA	exonic	GABRQ	nonsynonymous SNV	GABRQ:ENST00000370306.2:exon9:c.G1333A:p.A445T,	Xq28	.
X	152770197	G	A	CATTCATGAT,AACGATGAGG	exonic	BGN	nonsynonymous SNV	BGN:ENST00000331595.4:exon2:c.G108A:p.M36I,BGN:ENST00000431891.1:exon2:c.G108A:p.M36I,	Xq28	.
Y	2655453	C	G	TGAATGCGTT,ATGGGTCGCT	exonic	SRY	nonsynonymous SNV	SRY:ENST00000534739.2:exon1:c.G192C:p.M64I,SRY:ENST00000525526.2:exon1:c.G192C:p.M64I,SRY:ENST00000383070.1:exon1:c.G192C:p.M64I,	Yp11.31	.

arge_intestine

ng&RHBG;basechange=c.285C>T;AAchange=p.L95L;tissue=ovary,lung&RHBG_ENST00000368246;basechange=c.285C>T;AAchange=p.L95L;tissue=ovary,lung

ge_intestine&NTRK1;basechange=c.2074C>T;AAchange=p.R692C;tissue=large_intestine

ng&OR2L13;basechange=c.793C>T;AAchange=p.R265C;tissue=lung

5;basechange=c.1532C>T;AAchange=p.S511F;tissue=large_intestine

ımphoid_tissue

dometrium&FAT4;basechange=c.6022C>T;AAchange=p.R2008W;tissue=endometrium

lung

ous_system

KIF6;basechange=c.1494G>T;AAchange=p.M498I;tissue=lung

t

ge=c.662C>T;AAchange=p.S221F;tissue=endometrium

ung&CSMD1_ENST00000537824;basechange=c.7877C>T;AAchange=p.S2626F;tissue=lung

e

change=c.113C>A;AAchange=p.S38Y;tissue=pancreas

IST00000294064;basechange=c.364C>T;AAchange=p.P122S;tissue=large_intestine

ct

ivary_gland&ENOX1;basechange=c.724C>A;AAchange=p.R242R;tissue=lung&ENOX1;basechange=c.724C>T;AAchange=p.R242W;tissue=salivary_gland&ENOX1_ENST00000261488;basechange=c.724C>A;AAchange=p.R242R;tissue=lung

arge_intestine,endometrium&DNAH3;basechange=c.3223C>T;AAchange=p.R1075C;tissue=large_intestine,endometrium

_intestine&PHKB_ENST00000323584;basechange=c.707C>T;AAchange=p.S236L;tissue=large_intestine&PHKB;basechange=c.707C>T;AAchange=p.S236L;tissue=large_intestine

_tract&TP53_ENST00000269305;basechange=c.1001G>T;AAchange=p.G334V;tissue=lung&TP53;basechange=c.1001G>C;AAchange=p.G334A;tissue=lung&TP53_ENST00000269305;basechange=c.1001G>C;AAchange=p.G334A;tissue=lung

trium

east&MYH14;basechange=c.982C>T;AAchange=p.R328W;tissue=breast

:kidney&ZSCAN18;basechange=c.880G>A;AAchange=p.E294K;tissue=kidney

dometrium&PLCB1;basechange=c.1229C>T;AAchange=p.S410L;tissue=endometrium

00378641;basechange=c.2753C>T;AAchange=p.S918L;tissue=lung

ICK;basechange=c.310G>T;AAchange=p.E104*;tissue=lung

em

dometrium&TAF1;basechange=c.3505C>T;AAchange=p.R1169C;tissue=endometrium

C000-JMUDMJ

Chromosome	Position (hg19)	Reference allele	Mutant allele	Sequence context	ANNOVAR function	Gene	Mutation type	ANNOVAR output (alternative transcripts)
1	2938260	C	T	CATGTTTAAT,CGCACGCTTT	exonic	ACTRT2	[illegible]	[illegible]
1	6614369	C	A	CTGGCGGGCT,CCTCCCTCCA	exonic	NOL9	[illegible]	[illegible]
1	23340484	C	T	AGCTCATCAG,CAAGCTGCCA	exonic	C1orf234	[illegible]	[illegible]
1	29644321	G	T	CTGTTGCCCC,GAACCGCGAC	exonic	PTPRU	[illegible]	[illegible]
1	34035054	G	A	TGTGTATCCG,AATTGCAGGA	exonic	CSMD2	[illegible]	[illegible]
1	35370709	C	T	GGCCAGGGTA,ATCCTGGGGA	exonic	DLGAP3	[illegible]	[illegible]
1	55074778	C	T	AGCCTCCAGA,CCTCTAGATG	exonic	ACOT11	[illegible]	[illegible]
1	76276418	A	T	ACCTTTGGAA,TAATAATGTC	exonic	MSH4	[illegible]	[illegible]
1	100378068	G	C	ACAGTAAAAA,ACATGGTAAG	exonic	AGL	[illegible]	[illegible]
1	102270177	C	T	CCGATTTCAT,AGCCATTAGG	exonic	OLFM3	[illegible]	[illegible]
1	117156651	C	T	ACGGGCTTCT,GCCAACTTTC	exonic	IGSF3	[illegible]	[illegible]
1	150902588	G	A	CCTCAGTATT,ATTCAGGTAA	exonic	SETDB1	[illegible]	[illegible]
1	152191626	C	T	TGACTATAGC,CTGTCCTGAG	exonic	HRNR	[illegible]	[illegible]
1	153982447	C	T	GTGTAACTTA,CTTTCTCCAT	splicing	NUP210L(ENST00000368559.3:exon36:c.4930+1G>A,ENST00000368553.1:exon14:c.1729+1G>A,ENST0000[illegible]	[illegible]	[illegible]
1	157771734	C	T	CGCCTCACTG,GCTGGGCCCC	exonic	FCRL1	[illegible]	[illegible]
1	158576701	G	A	TTTGGACTGG,AATGACACTA	exonic	OR10Z1	[illegible]	[illegible]
1	203767480	C	T	TACACTGATC,TCAGCACATC	exonic	ZBED6	[illegible]	[illegible]
1	205052814	T	C	AGGCTTGGAG,AGCTGTCCAG	exonic	TMEM81	[illegible]	[illegible]
1	212977713	G	C	ACAGAAGGAA,AGCAAAGACA	exonic	TATDN3	[illegible]	[illegible]
1	227073324	G	A	CATGATCAGC,TCATCGTGGT	exonic	PSEN2	[illegible]	[illegible]
1	229772834	C	T	ATTCTGTGTT,TGGTGCCCAG	exonic	URB2	[illegible]	[illegible]
2	11728854	G	A	GTGCCAAGTC,CTGCTGTTCC	exonic	GREB1	[illegible]	[illegible]
2	20192876	C	G	AGCAATTTAA,GATGTATTTG	exonic	MATN3	[illegible]	[illegible]
2	48958420	G	A	CACTTTGACA,GGAGGTAGGC	exonic	LHCGR	[illegible]	[illegible]
2	49190357	G	A	TTGAGCACAA,GAGGGACATG	exonic	FSHR	[illegible]	[illegible]
2	71365806	T	G	GCTTTTTCCA,GGTTCCATTT	exonic	MPHOSPH10	[illegible]	[illegible]
2	74759771	G	A	ACAGCTTCGA,AACCAAGCTT	exonic	HTRA2	[illegible]	[illegible]
2	80101412	G	A	GCCCACTGAC,AAGCCAAGGG	exonic	CTNNA2	[illegible]	[illegible]
2	100055324	G	A	GAAGGCCCCT,AACAGTGGAG	exonic	REV1	[illegible]	[illegible]
2	128255753	C	T	GAATCTGAAT,TTCTGCTTCT	exonic	IWS1	[illegible]	[illegible]
2	128750799	G	A	ACTGGTGATT,GGGTGTAGGT	exonic	SAP130	[illegible]	[illegible]
2	160639971	G	A	CCAAGTAGAT,AAGGACAGTC	exonic	CD302,LY75,LY75-CD302	[illegible]	[illegible]
2	166915089	A	G	TGAATGTACC,AAATCTTAAT	exonic	SCN1A	[illegible]	[illegible]
2	168101389	C	T	TGGGGATGTT,GTACAGCATG	exonic	XIRP2	[illegible]	[illegible]
2	172016869	T	C	GAGCCCGGGG,TGGAGACGTG	exonic	TLK1	[illegible]	[illegible]
2	179574406	T	C	CACTAACGTG,TGTTCTTGAA	exonic	TTN	[illegible]	[illegible]
2	186666977	G	A	ATTGTTGCCA,ACTTTTGTCA	exonic	FSIP2	[illegible]	[illegible]
2	203500121	G	A	TGGCTGCTGT,GTGGCGCCTC	exonic	FAM117B	[illegible]	[illegible]
2	206480227	G	A	GCACCTCGGG,GCTCTACAAG	exonic	PARD3B	[illegible]	[illegible]
2	220251040	C	A	AGAGTCAGGT,ACGGTCAAAC	exonic	DNPEP	[illegible]	[illegible]
2	233349723	C	T	TGCAGGATCT,TTGGGCCTTC	exonic	ECEL1	[illegible]	[illegible]
2	239149213	C	T	AGGCAGGGTC,ATTGGGCAGC	exonic	HES6	[illegible]	[illegible]
2	242077439	C	T	TGCCCAGCCA,GTCCTCCCCA	exonic	PASK	[illegible]	[illegible]
3	10320126	C	T	AACGGATGCT,CCTATTTCCT	exonic	RP11-438J1.1,TATDN2	[illegible]	[illegible]
3	44286066	C	T	TTTTAAAATT,CTTTACTTAA	exonic	TOPAZ1	[illegible]	[illegible]
3	52442078	A	T	TGAGTTGCAC,AGAGTTGGGT	exonic	BAP1	[illegible]	[illegible]
3	125876320	A	G	AAGATGGAAA,CCCCCCTTTC	exonic	ALDH1L1	[illegible]	[illegible]
3	126916061	C	T	AGAGGAGCTC,CTACTTGAAG	exonic	C3orf56	[illegible]	[illegible]
3	140277573	G	A	CACCCTCCGG,GCACAGACCA	exonic	CLSTN2	[illegible]	[illegible]
3	142720496	C	A	CCAGGCGGAT,TCAGAAGGCC	exonic	U2SURP	[illegible]	[illegible]
3	150377858	G	A	TGAAACAGGC,ACGGATCTTT	exonic	FAM194A	[illegible]	[illegible]
3	157131725	G	A	AGTGAGGTAG,GGAATCTCTC	exonic	VEPH1	[illegible]	[illegible]
3	181430246	A	G	GCCGGCGGCA,CCAGAAAAAC	exonic	SOX2	[illegible]	[illegible]
3	185823650	C	T	ATCATAAAAC,CGTCCATGGT	exonic	ETV5	[illegible]	[illegible]
4	15602875	C	T	TGGATTTCCT,TTCACATGCC	exonic	CC2D2A	[illegible]	[illegible]
4	39352966	A	G	CAGAAAACTA,CCTTGATTTC	splicing	RFC1(ENST00000349703.2:exon3:c.132+2T>C,ENST00000381897.1:exon3:c.132+2T>C,ENST00000512275[illegible]	[illegible]	[illegible]
4	46967034	G	A	GGAGCAGCGG,AACTTCCTGA	exonic	GABRA4	[illegible]	[illegible]
4	71629299	C	T	CAAAATAAAT,CTTCTGGGGG	exonic	RUFY3	[illegible]	[illegible]
4	77700112	G	A	CCAGCACTTC,TGAAAATGAA	exonic	SHROOM3	[illegible]	[illegible]
4	78663299	C	A	ACTTACTTTT,TGTTTTGAAG	exonic	CNOT6L	[illegible]	[illegible]
4	79362317	T	C	CTTTCTTAGG,TGATGAGGGA	exonic	FRAS1	[illegible]	[illegible]
4	100570462	C	G	GTGGTGCCAT,AAAGGAAGGG	exonic	RP11-766F14.2	[illegible]	[illegible]
4	104012432	G	A	ACAGTTCCAT,ATGGACAAAA	exonic	BDH2	[illegible]	[illegible]
4	123663967	C	T	TGTACAAAAC,ATTTATGTTT	exonic	BBS12	[illegible]	[illegible]
4	156634346	G	A	AGATTTTACA,GACGAGGGCT	exonic	GUCY1A3	[illegible]	[illegible]
4	169433338	G	A	GAAGACCAAG,GGAGATGGAA	exonic	PALLD	[illegible]	[illegible]
4	183609364	G	A	TGCATGGGGG,GACGTGTCGC	exonic	TENM3	[illegible]	[illegible]
5	26915810	C	T	TCATTGATAT,ATGTATTTTA	exonic	CDH9	[illegible]	[illegible]
5	34824067	C	T	TCTCAGTATT,AAAAGTGTTG	exonic	RAI14	[illegible]	[illegible]
5	40854535	G	A	CAGAAGAGGG,AGGGAAGCAT	exonic	CARD6	[illegible]	[illegible]
5	168114019	G	C	TGTAGCCATT,ATTGTGTCCA	exonic	SLIT3	[illegible]	[illegible]
5	168187939	C	T	GTCATTCAGT,GCCTGTGTAG	exonic	SLIT3	[illegible]	[illegible]
5	171765683	G	A	CAAAGAGTTG,AGAGAAAAGG	exonic	SH3PXD2B	[illegible]	[illegible]
5	171765684	A	G	AAAGAGTTGG,GAGAAAAGGT	exonic	SH3PXD2B	[illegible]	[illegible]
5	179731848	G	A	CATGATGACG,GCATCTGCTT	exonic	GFPT2	[illegible]	[illegible]
6	407574	C	G	ACAGATCTAT,CGCCATTCCT	exonic	IRF4	[illegible]	[illegible]
6	7559515	G	A	AGTGTCCCTC,AGTCCGCAGG	exonic	DSP	[illegible]	[illegible]
6	31594940	C	T	TCACCGGGGC,CCGCCGGGAA	exonic	PRRC2A	[illegible]	[illegible]
6	31730916	G	C	AAGCCGCCAA,ACCCAGGGGC	exonic	SAPCD1	[illegible]	[illegible]
6	32118434	G	A	AGCAGGGCCG,GAGGGGCGTG	exonic	PRRT1	[illegible]	[illegible]
6	32118435	G	A	GCAGGGCCGG,AGGGGCGTGG	exonic	PRRT1	[illegible]	[illegible]
6	33287871	G	C	CTCCTCTTCA,AATCTGTGGC	exonic	DAXX	[illegible]	[illegible]
6	72892433	C	G	GCCAGGGCCT,GCCGCCGGAC	exonic	RIMS1	[illegible]	[illegible]
6	88853663	C	T	TTCTGCGGCC,TGTGAACACT	exonic	CNR1	[illegible]	[illegible]
6	94120509	C	T	AAGATAGAAT,CCTTTTTGGA	exonic	EPHA7	[illegible]	[illegible]
6	97344645	G	A	AGGATCTTTG,AATCAACATA	exonic	NDUFAF4	[illegible]	[illegible]
6	111208713	A	C	GATAGATCTG,GTGGGACATA	exonic	AMD1	[illegible]	[illegible]
6	131602722	G	T	GGCTCAGTAA,AGGCTGGTGG	exonic	AKAP7	[illegible]	[illegible]
6	162622218	C	A	CCTGAGTTTT,CCGGCTGCAC	exonic	PARK2	[illegible]	[illegible]
6	167357260	G	T	AGTTGAGGGG,AAGGGAGTGA	exonic	RNASET2	[illegible]	[illegible]
7	893142	C	T	TGAGAATCTC,GAGAGCTGAC	exonic	SUN1	[illegible]	[illegible]
7	8009070	C	T	GCCGCGGGGT,CCCGCCCGCC	exonic	GLCCI1	[illegible]	[illegible]
7	37890324	T	A	GACGAAATTC,GCATTTTGCT	exonic	NME8	[illegible]	[illegible]
7	44796669	G	A	GTTTGCTGAA,GCGGCGCCAA	exonic	ZMIZ2	[illegible]	[illegible]
7	48142919	C	G	CCTTCTATCT,AATCATGTTG	exonic	UPP1	[illegible]	[illegible]
7	75608872	C	G	CGAGGCATGT,AGCGGACCCT	exonic	POR	[illegible]	[illegible]
7	75614122	C	T	CACCCATTCC,GTGCCCTACG	exonic	POR	[illegible]	[illegible]
7	77694907	C	T	TTTAGGGTGG,CCAATAAGTA	exonic	MAGI2	[illegible]	[illegible]
7	100382245	C	G	TCACCCACAG,TCTGGAATGC	exonic	ZAN	[illegible]	[illegible]
7	145814045	G	A	TGCAGAGCCT,GACGGCTCCC	exonic	CNTNAP2	[illegible]	[illegible]
7	148876723	C	T	GGACCACCTC,GTTCAGGCCA	exonic	ZNF398	[illegible]	[illegible]
8	38027369	C	T	AAAAATCCCT,GAGGAATATC	exonic	LSM1	[illegible]	[illegible]
8	55372417	C	G	ATCTGCACTT,GTGTGCAAGC	exonic	SOX17	[illegible]	[illegible]
8	55540286	C	T	CACTTTTTTT,CTAGTGATGG	exonic	RP1	[illegible]	[illegible]
8	75227709	G	A	GCGTCGTGGA,CACGCTGCCA	exonic	JPH1	[illegible]	[illegible]
8	91937878	G	A	TAATGTCAGC,GTCCAGGTAA	exonic	NECAB1	[illegible]	[illegible]
8	135669905	G	A	GTGCTTCTCT,AAACGTGGGA	exonic	ZFAT	[illegible]	[illegible]
8	139165241	C	T	GAGCACATGT,CATATGATTT	exonic	FAM135B	[illegible]	[illegible]
8	139788247	G	T	CGTTCTCCTT,GAGACCCTGG	exonic	COL22A1	[illegible]	[illegible]
8	144998006	C	T	CGCGCCGACT,CTGCTCCGCT	exonic	PLEC	[illegible]	[illegible]
8	144999738	C	G	CCACGCGGAT,TCCTCCTCGA	exonic	PLEC	[illegible]	[illegible]
8	145758552	T	A	CGCCAGCACC,CCTCAGAGAG	exonic	ARHGAP39	[illegible]	[illegible]
9	6620259	G	A	GCCAATATAC,ATCTCCAGAT	exonic	GLDC	[illegible]	[illegible]
9	78803520	C	T	TGCTCTGTCT,CTGTGAAGAT	exonic	PCSK5	[illegible]	[illegible]
9	109688285	G	A	CAGGATTGAC,AGATAGCAAG	exonic	ZNF462	[illegible]	[illegible]
9	113169985	C	T	CTCAAAATAT,CCTGGTCATC	exonic	SVEP1	[illegible]	[illegible]
9	115336902	C	T	GTCCCATCTT,CCTCCATTCA	exonic	KIAA1958	[illegible]	[illegible]
9	119495735	C	T	CACTGCTCCT,CACCGGCAGC	exonic	ASTN2	[illegible]	[illegible]
9	140611149	G	A	TGCGGACGGT,AGACCAATGG	exonic	EHMT1	[illegible]	[illegible]
10	17113884	G	C	TTTTAAACCT,ATCCAGACAC	exonic	CUBN	[illegible]	[illegible]
10	58120994	C	A	TCCGCTGCCT,CATCTTTCCA	exonic	ZWINT	[illegible]	[illegible]
10	68857541	A	T	GACCATAAAC,GCAGCTAGCT	exonic	LRRTM3	[illegible]	[illegible]
10	70204838	A	T	AGCTTTAATA,TTCTATGAAA	exonic	DNA2	[illegible]	[illegible]
10	75279565	G	A	CTTGTTGGCT,AGAGAGAGTC	exonic	USP54	[illegible]	[illegible]
10	99144995	G	A	ATTCTCACTG,GAACATAGTC	exonic	RRP12	[illegible]	[illegible]
10	103909017	G	A	GCGGCAGGCA,ATGAGCAGCC	exonic	PPRC1	[illegible]	[illegible]
10	105758958	C	G	CCCTGGGTAT,ACTTTAATAG	exonic	SLK	[illegible]	[illegible]
10	106074441	G	C	TCGTGCAGAC,AGCGTCCAGC	exonic	ITPRIP	[illegible]	[illegible]
10	121663758	C	T	CGATTTATTC,CTATACTGAG	exonic	SEC23IP	[illegible]	[illegible]
11	723320	C	T	CCGGGGGATG,CCTACCACCA	exonic	EPS8L2	[illegible]	[illegible]
11	4406648	G	A	TTCAGAGAAG,AGTAGATGAG	exonic	TRIM21	[illegible]	[illegible]
11	5862851	C	T	AAAGATATTT,CTTGATATTG	exonic	OR52E6	[illegible]	[illegible]
11	6232243	C	G	GCTGCAGGGT,TATCCTCAGA	exonic	C11orf42	[illegible]	[illegible]
11	17448601	C	T	ATTACCTGCT,CAGAAGACAG	exonic	ABCC8	[illegible]	[illegible]
11	22296268	G	A	TGCACCTTCG,AAAAACGAGA	exonic	ANO5	[illegible]	[illegible]
11	46760624	G	A	AGGGAAACGA,GGGATGCCTG	exonic	F2	[illegible]	[illegible]
11	55861448	C	T	ATCATCATCT,AGCCATCCTG	exonic	OR8I2	[illegible]	[illegible]
11	56043719	C	T	ATTTCTTGTT,TGACACTCAC	exonic	OR5T1	[illegible]	[illegible]
11	61068380	C	G	GTTCTGTCTT,CGCTCGGTGT	exonic	DDB1	[illegible]	[illegible]
11	64802367	A	T	GGGGCAGAGG,CCTGCTTCGC	exonic	SNX15	[illegible]	[illegible]
11	65427643	C	G	ATAGCCTGCT,CAGGTCCCGC	exonic	RELA	[illegible]	[illegible]
11	70218446	G	C	TGGACCACTT,ACCAAGAAAG	exonic	PPFIA1	[illegible]	[illegible]
11	120322404	C	T	GCCTCTTTTT,CCAGGAAGGA	exonic	ARHGEF12	[illegible]	[illegible]
11	123600392	G	A	CCCAGATCTG,GCTCTGTGTG	exonic	ZNF202	[illegible]	[illegible]
11	123848358	G	A	GGGGTTCTCC,TTGTCATGGT	exonic	OR10S1	[illegible]	[illegible]
11	124756579	G	A	GGCCGAGGTG,GCACAGCAAG	exonic	ROBO4	[illegible]	[illegible]
11	124756580	G	T	GCCGAGGTGG,CACAGCAAGA	exonic	ROBO4	[illegible]	[illegible]
12	977851	C	T	AATTTTACCT,AGCGTGTTTA	exonic	WNK1	[illegible]	[illegible]
12	2794921	G	A	CCAGGATGAC,AAAATCGGCA	exonic	CACNA1C	[illegible]	[illegible]
12	3765493	G	A	CTGCTTCTGG,TGAGCTGCTC	exonic	EFCAB4B	[illegible]	[illegible]
12	6425571	G	A	CTTCCTGACG,TGAGGCCTGT	splicing	PLEKHG6(ENST00000011684.7:exon7:c.756+1G>A,ENST00000536531.1:exon7:c.756+1G>A,ENST000003[illegible]	[illegible]	[illegible]
12	9225259	A	G	CTGGAGGTAG,CACATCCTTC	exonic	A2M	[illegible]	[illegible]
12	22778372	A	T	GCCATGGCCA,TTACATCCAC	exonic	ETNK1	[illegible]	[illegible]
12	27064153	G	A	ATTTCAACAA,GGGTTTTTTG	exonic	ASUN	[illegible]	[illegible]
12	40877507	G	A	TGTAGTGACA,AGACAACTGG	exonic	MUC19	[illegible]	[illegible]
12	48369379	C	T	CCAGGATTTC,AGGAGGACCC	exonic	COL2A1	[illegible]	[illegible]
12	50030610	G	A	CGCCAACAAC,CAAGAATCGG	exonic	PRPF40B	[illegible]	[illegible]
12	55968758	G	A	GAACTCTCAT,TTCAGACACA	exonic	OR2AP1	[illegible]	[illegible]
12	56197508	G	A	CTTTAGTTCG,CAAGCTGAGG	exonic	RP11-762I7.5,SARNP	[illegible]	[illegible]
12	96691416	A	G	AGATACTCAA,CACCAAAGTC	exonic	CDK17	[illegible]	[illegible]
12	96691425	T	G	AACACCAAAG,CAAGGATTTA	exonic	CDK17	[illegible]	[illegible]
12	112471120	C	G	ATATGATCCA,AACATTGGAA	exonic	NAA25	[illegible]	[illegible]
12	112471132	T	A	ACATTGGAAA,TAAAGTCTGA	exonic	NAA25	[illegible]	[illegible]
12	112696348	G	A	ACCACACGGG,AACAGCCCAC	exonic	HECTD4	[illegible]	[illegible]
12	123270306	C	G	GTGGAACTTT,TGCCCAGGTA	exonic	CCDC62	[illegible]	[illegible]
12	123285688	C	G	ATGTGTTTAT,AGACCTTGAA	exonic	CCDC62	[illegible]	[illegible]
12	125834705	C	T	GCAAGCGTTT,CAGCCCGAGA	exonic	TMEM132B	[illegible]	[illegible]
12	129154038	A	T	TCTGTGGAGG,GAACAGTGCC	exonic	TMEM132C	[illegible]	[illegible]
13	23929747	G	A	ACTCTCACTC,AAGTCACTCT	exonic	SACS	[illegible]	[illegible]
13	25671222	C	T	GGTTGTTAAT,TTTATGTGAA	exonic	PABPC3	[illegible]	[illegible]
13	37598479	G	C	TGTAATACCT,AGGAACTATT	exonic	SUPT20H	[illegible]	[illegible]
13	92346062	T	G	GGACACGTGC,GCTGAACTTT	exonic	GPC5	[illegible]	[illegible]
13	112722308	G	A	CGCTGCACAT,AAGGAGCACC	exonic	SOX1	[illegible]	[illegible]
14	44973931	C	T	GACACATTCT,TACCAGAGCC	exonic	FSCB	[illegible]	[illegible]
14	50081177	T	A	TTCTGTTCTC,AGGCTGTTAT	exonic	LRR1	[illegible]	[illegible]
14	50769715	A	G	TCCGCCACCA,CGATGACTAT	exonic	L2HGDH	[illegible]	[illegible]
14	64935542	A	T	TCATTCCAAA,TTATAGAAGA	exonic	AKAP5	[illegible]	[illegible]
14	102457924	G	C	ACCAATTGAA,AGTGCAGATA	exonic	DYNC1H1	[illegible]	[illegible]
14	104173426	C	T	CGAGCTGCGT,CGGCCAGCTC	exonic	XRCC3	[illegible]	[illegible]
15	50288937	G	A	AGTGCATGGC,GACTTTTAGG	exonic	ATP8B4	[illegible]	[illegible]
15	53994552	C	T	GGGGGAGAAT,TGAAAAACAA	splicing	WDR72(ENST00000396328.1:exon13:c.1349-1G>A,ENST00000557913.1:exon13:c.1340-1G>A,ENST000003[illegible]	[illegible]	[illegible]
15	92987871	A	G	GGCAGGTGCA,CCTGGCCCCA	exonic	ST8SIA2	[illegible]	[illegible]
15	94901831	C	T	GCATGAGGAA,GTCTGGGCAC	exonic	MCTP2	[illegible]	[illegible]
15	101814817	C	T	TTTGCATTTC,TTTGTAACTT	exonic	VIMP	[illegible]	[illegible]
16	1260883	A	G	CCTGGTGGAC,TTGTCGTGGC	exonic	CACNA1H	[illegible]	[illegible]
16	2283940	G	C	TCAGTGCAGT,AGACCTTCCC	exonic	E4F1	[illegible]	[illegible]
16	2285487	G	C	GGTGTCATCA,AGGACATCGA	exonic	E4F1	[illegible]	[illegible]
16	23672527	C	T	GAGACCATGT,TTTATTGAGG	exonic	DCTN5	[illegible]	[illegible]
16	30124666	C	T	CTCACCTCCT,GGTGGGCCCC	exonic	GDPD3	[illegible]	[illegible]
16	88793964	C	T	GGGGCACTCA,TGATGAGGTT	splicing	PIEZO1(ENST00000301015.9:exon24:c.3301+1G>A)	[illegible]	[illegible]
17	5323552	C	G	GTTGAGGTCT,CGCGCCGCTC	exonic	RPAIN	[illegible]	[illegible]
17	5462613	G	A	CTCCAAAGAA,GAATGAGGTT	exonic	NLRP1	[illegible]	[illegible]
17	5462614	G	A	TCCAAAGAAG,AATGAGGTTC	exonic	NLRP1	[illegible]	[illegible]
17	16527131	C	T	TAAGGTTTCT,TTTAGTGTGA	exonic	ZNF624	[illegible]	[illegible]
17	30503198	C	T	TCGATGGATT,CTCTCATAAA	exonic	RHOT1	[illegible]	[illegible]
17	38172805	G	A	GCCCTGGAAG,GATCTCCCCC	exonic	CSF3	[illegible]	[illegible]
17	54535241	A	T	GTATGTGGGA,GATATAAGGT	exonic	ANKFN1	[illegible]	[illegible]
17	72920993	G	A	CGCTGCCCCT,CGCGGTACCC	exonic	OTOP2	[illegible]	[illegible]
17	74076878	G	A	CAGTGGCTCG,CCCCTTGCAG	exonic	ZACN	[illegible]	[illegible]
17	79226921	C	T	TCCTTGCCAT,TTCCCGTCCA	exonic	SLC38A10	[illegible]	[illegible]
18	756655	G	A	TGGTGTCATG,AAAGACTGCT	exonic	YES1	[illegible]	[illegible]
18	3193871	C	T	TTCTCTTCTC,AGACAGTAGG	exonic	MYOM1	[illegible]	[illegible]
19	2269490	C	T	GGCGCAGCAT,TATAAAGGCG	exonic	JSRP1	[illegible]	[illegible]
19	4048234	T	G	CACCTTCAGC,TGTCCTGCCT	exonic	ZBTB7A	[illegible]	[illegible]
19	8595395	C	T	GCCGCCAGGC,TTCTGGATGG	exonic	MYO1F	[illegible]	[illegible]
19	12383844	T	C	TTCATGTCTT,GAAATACACT	exonic	ZNF44	[illegible]	[illegible]
19	15760883	G	A	CACAGATGCC,TCATCCAGGA	exonic	CYP4F3	[illegible]	[illegible]
19	17932134	C	T	ACGGTCGCCT,CGGTCGCAGG	exonic	INSL3	[illegible]	[illegible]
19	18420646	G	A	GTAGCACTCG,GCATCCGCCA	exonic	LSM4	[illegible]	[illegible]
19	21719328	C	T	TATGCATTTT,AAATGCAGAT	exonic	ZNF429	[illegible]	[illegible]
19	33392302	C	T	TCTTTCTCTT,TTCCTTCTGT	exonic	CEP89	[illegible]	[illegible]
19	39664289	C	T	TCCTCCTCCT,CTCCCGGCCT	exonic	PAK4	[illegible]	[illegible]
19	41630767	C	T	CTTGCCGCAC,GCGTCACTAG	exonic	CYP2F1	[illegible]	[illegible]
19	45515283	C	T	CCTGGTGTCT,GCGGGGCTGC	exonic	RELB	[illegible]	[illegible]
19	48722140	C	G	GGCTTCACCT,AGTATCCCAC	exonic	CARD8	[illegible]	[illegible]
19	49002809	G	A	GTTGGCGTGG,GGGCCGGGGG	exonic	LMTK3	[illegible]	[illegible]
19	51016640	C	T	AAGTTCTGGT,CCTGAGCCAA	exonic	ASPDH	[illegible]	[illegible]
19	51205883	C	T	CCCCCCGTGC,CCCGGCTGGC	exonic	SHANK1	[illegible]	[illegible]
19	53344960	G	A	AGAATGGAGG,AATTATTTCC	exonic	ZNF468	[illegible]	[illegible]
19	55044990	C	T	TGGCCGAGCC,TGTGGTTCCC	exonic	KIR3DX1	[illegible]	[illegible]
19	55044992	G	T	GCCGAGCCCT,TGGTTCCCCT	exonic	KIR3DX1	[illegible]	[illegible]
19	57932052	C	T	CTTTAGATAC,GTTCCACACT	exonic	ZNF17	[illegible]	[illegible]
20	941100	G	A	CTTCTGGCCG,GGCTCCTCTC	exonic	RSPO4	[illegible]	[illegible]
20	941101	G	A	TTCTGGCCGG,GCTCCTCTCT	exonic	RSPO4	[illegible]	[illegible]
20	1559211	C	G	TCCAGCTCCT,TAAACCACAT	exonic	RP4-576H24.4,SIRPB1	[illegible]	[illegible]
20	13098347	C	T	TTTGGAGCTT,AGGAGGTTAC	exonic	SPTLC3	[illegible]	[illegible]
20	31044182	C	G	CGCTGCCACT,TCAGAGGAGG	exonic	C20orf112	[illegible]	[illegible]
20	34282804	C	T	GAGTGAGACT,AGTCTAAAAA	exonic	NFS1	[illegible]	[illegible]
20	36962852	C	G	TGCAGGATAT,ATGAACTACA	exonic	BPI	[illegible]	[illegible]
20	48098747	C	T	GAGGTGAAGG,GCCCGGGTGG	exonic	KCNB1	[illegible]	[illegible]
20	50405468	G	C	TCTCCAGTGT,AGTCCGCTCG	exonic	SALL4	[illegible]	[illegible]
20	62073835	G	A	CACCAGGAAC,AGGCCAGGAT	exonic	KCNQ2	[illegible]	[illegible]
21	16015443	C	T	ATAAGCTATC,ATAGAGCCCT	exonic	AF165138.7	[illegible]	[illegible]
21	41741116	C	T	AATCCATCTT,ATTCTGTACA	exonic	DSCAM	[illegible]	[illegible]
21	45537810	G	A	CCATCTTCAT,AGCTGCCAGA	exonic	PWP2	[illegible]	[illegible]
22	26114317	G	A	TTTCCAAATA,ACTTGAATGG	exonic	ADRBK2	[illegible]	[illegible]
22	31672964	G	C	CAGAACTAGA,ATTGACGTGG	exonic	LIMK2	[illegible]	[illegible]
22	32937578	C	T	GTAGTTGGAT,CAATTTTCTG	exonic	SYN3	[illegible]	[illegible]
X	1467412	C	T	CCACCATTCT,CACGTGGATC	exonic	IL3RA	[illegible]	[illegible]
X	2945371	G	A	AGGAATGGGA,GATGGGAAGG	exonic	ARSH	[illegible]	[illegible]
X	2945372	G	A	GGAATGGGAG,ATGGGAAGGA	exonic	ARSH	[illegible]	[illegible]
X	22292345	C	T	CCTACATGGT,GATCACATCA	exonic	ZNF645	[illegible]	[illegible]
X	49854869	C	T	GCAGCCATGA,AAGCAAGTGG	exonic	CLCN5	[illegible]	[illegible]
X	53630423	G	C	AGACCCAATT,AGAGCCCCAC	exonic	HUWE1	[illegible]	[illegible]
X	69497959	C	G	TAGACAAGTA,ACCAAGACTG	exonic	ARR3	[illegible]	[illegible]
X	70149573	G	A	ACCAGAACGG,GAACCCGGGC	exonic	SLC7A3	[illegible]	[illegible]
X	129201492	G	A	GCTGGGCACT,AAGATGCACT	exonic	ELF4	[illegible]	[illegible]

G>A;AAchange=p.E1900K;tissue=kidney,endon

C000-RCWM1J

Chromosom	Position (hg1	Reference all	Mutant allele	Sequence context	ANNOVAR fu	Gene	Mutation type	ANNOVAR output (alternative transcripts)	Cytoband	COSMIC
1	1114711	C	G	GCTCGAGTCT,AGGTGAATAG	exonic	TTLL10	stopgain	TTLL10:ENST00000379289.1:exon4:c.C116G:p.S39X,TTLL10:ENST00000379290.1:exon4:c.C116G:p.S39X,	1p36.33	.
1	1115590	G	A	TGCAGACTCG,ATGACACTAA	exonic	TTLL10	nonsynonymous SNV	TTLL10:ENST00000379289.1:exon6:c.G376A:p.D126N,TTLL10:ENST00000379288.3:exon2:c.G157A:p.D53N,TTLL10:ENST00000379290.1:exon6:c.G376A:p.D126N,	1p36.33	.
1	3342626	C	T	GAGCCAGCAC,CCGGGGTCCT	exonic	PRDM16	nonsynonymous SNV	PRDM16:ENST00000514189.1:exon14:c.C3121T:p.P1041S,PRDM16:ENST00000270722.5:exon14:c.C3121T:p.P1041S,PRDM16:ENST00000509860.1:exon10:c.C2545T:p.P849S,PRDM16:ENST00000378391.2:exon14:c.C3121T:p.P1041S,PRDM16:ENST00000442529.2:exon14:c.C3118T:p.P1040S,PRDM16:ENST	1p36.32	.
1	9416090	C	T	CCCCCTGTGT,CTATGATGTC	exonic	SPSB1	nonsynonymous SNV	SPSB1:ENST00000450402.1:exon2:c.C140T:p.S47F,SPSB1:ENST00000328089.6:exon2:c.C140T:p.S47F,SPSB1:ENST00000377399.2:exon1:c.C140T:p.S47F,SPSB1:ENST00000357898.3:exon2:c.C140T:p.S47F,	1p36.22	.
1	11007746	C	T	TCAGAAAGCC,CTCCACACTG	exonic	C1orf127	nonsynonymous SNV	C1orf127:ENST00000377004.4:exon12:c.G2446A:p.G816R,C1orf127:ENST00000377008.4:exon11:c.G1945A:p.G649R,	1p36.22	.
1	11009805	C	T	GGTCTCTGGT,CTGGGGATGG	exonic	C1orf127	nonsynonymous SNV	C1orf127:ENST00000377004.4:exon11:c.G1166A:p.G389E,C1orf127:ENST00000377008.4:exon10:c.G665A:p.G222E,	1p36.22	.
1	11009806	C	T	GTCTCTGGTC,TGGGGATGGT	exonic	C1orf127	nonsynonymous SNV	C1orf127:ENST00000377004.4:exon11:c.G1165A:p.G389R,C1orf127:ENST00000377008.4:exon10:c.G664A:p.G222R,	1p36.22	.
1	11710670	C	A	GCGCCGTCCA,CAGCTCCTTC	exonic	FBXO2	nonsynonymous SNV	FBXO2:ENST00000465901.1:exon2:c.G295T:p.V99L,FBXO2:ENST00000354287.4:exon2:c.G244T:p.V82L,	1p36.22	.
1	11769359	C	T	GGTCCCAGCT,CCTGATGAAG	exonic	DRAXIN	nonsynonymous SNV	DRAXIN:ENST00000294485.5:exon3:c.C479T:p.S160F,	1p36.22	.
1	11983252	A	C	ACCCACCTTC,CCAGCCCCTT	exonic	KIAA2013	nonsynonymous SNV	KIAA2013:ENST00000376576.3:exon2:c.T1328G:p.V443G,KIAA2013:ENST00000376572.3:exon2:c.T1328G:p.V443G,	1p36.22	.
1	12460290	C	T	TATGTGACTC,CCTGAGCAAT	exonic	VPS13D	nonsynonymous SNV	VPS13D:ENST00000356315.4:exon60:c.C11612T:p.P3871L,VPS13D:ENST00000358136.3:exon61:c.C11687T:p.P3896L,	1p36.22	.
1	16374411	C	T	TTCTGGAATC,CGGAGGTGAA	exonic	CLCNKB	nonsynonymous SNV	CLCNKB:ENST00000375679.4:exon5:c.C370T:p.P124S,	1p36.13	.
1	16456025	G	A	CAGCCACTCG,ACACCGTGCG	exonic	EPHA2	nonsynonymous SNV	EPHA2:ENST00000358432.5:exon16:c.C2729T:p.S910F,	1p36.13	.
1	17685895	C	T	AGCTTTTTTC,CCAACATGGT	exonic	PADI4	nonsynonymous SNV	PADI4:ENST00000375448.4:exon15:c.C1750T:p.P584S,	1p36.13	.
1	19412756	C	G	TTCCCGGCCT,GAGCCAACCT	exonic	UBR4	nonsynonymous SNV	UBR4:ENST00000375267.2:exon101:c.G14696C:p.R4899P,UBR4:ENST00000375226.2:exon101:c.G14624C:p.R4875P,UBR4:ENST00000375217.2:exon101:c.G14675C:p.R4892P,UBR4:ENST00000375254.3:exon101:c.G14696C:p.R4899P,UBR4:ENST00000429347.2:exon11:c.G1265C:p.R422P,UBR4:ENST00000	1p36.13	.
1	20680460	G	A	CTTCTCCCTG,AGCCTCTGGC	exonic	VWA5B1	nonsynonymous SNV	VWA5B1:ENST00000375079.2:exon22:c.G3367A:p.E1123K,VWA5B1:ENST00000289815.8:exon21:c.G3274A:p.E1092K,	1p36.12	.
1	22168743	C	T	GTAGGAAGAC,CCTCACTGGG	exonic	HSPG2	nonsynonymous SNV	HSPG2:ENST00000374695.3:exon68:c.G9041A:p.G3014E,	1p36.12	.
1	22174190	G	A	ACCTGGTGTC,GGAGGGAAGG	exonic	HSPG2	stopgain	HSPG2:ENST00000453796.1:exon1:c.C262T:p.R88X,HSPG2:ENST00000374695.3:exon61:c.C8017T:p.R2673X,	1p36.12	.
1	22974269	C	T	CTGCTCTTCC,CGACTAGGGC	exonic	C1QC	nonsynonymous SNV	C1QC:ENST00000374637.1:exon3:c.C731T:p.P244L,C1QC:ENST00000374639.3:exon3:c.C731T:p.P244L,C1QC:ENST00000374640.4:exon3:c.C731T:p.P244L,	1p36.12	.
1	23419259	C	T	TCCCTTCAGT,CGCTCTCGGT	exonic	LUZP1	nonsynonymous SNV	LUZP1:ENST00000418342.1:exon3:c.G1496A:p.G499E,LUZP1:ENST00000374623.3:exon2:c.G1496A:p.G499E,LUZP1:ENST00000302291.4:exon4:c.G1496A:p.G499E,LUZP1:ENST00000314174.5:exon2:c.G1496A:p.G499E,	1p36.12	.
1	23782644	G	A	TGCCCGCACA,CCTTCTTTAT	exonic	ASAP3	nonsynonymous SNV	ASAP3:ENST00000336689.3:exon2:c.C173T:p.A58V,ASAP3:ENST00000608765.1:exon3:c.C173T:p.A58V,ASAP3:ENST00000437606.2:exon2:c.C173T:p.A58V,	1p36.12	.
1	24201157	C	T	CACACTTCTT,CAGTGAGCCA	exonic	CNR2	stopgain	CNR2:ENST00000536471.1:exon3:c.G951A:p.W317X,CNR2:ENST00000374472.4:exon2:c.G951A:p.W317X,	1p36.11	.
1	26670582	G	A	GTCCCTGGAG,GGCTGGGCCC	exonic	AIM1L	nonsynonymous SNV	AIM1L:ENST00000374207.4:exon4:c.C602T:p.P201L,	1p36.11	.
1	26691142	G	A	GAACTCAATG,GACCCGCTTT	exonic	ZNF683	nonsynonymous SNV	ZNF683:ENST00000436292.1:exon4:c.C895T:p.P299S,ZNF683:ENST00000349618.3:exon4:c.C895T:p.P299S,ZNF683:ENST00000374204.1:exon4:c.C895T:p.P299S,ZNF683:ENST00000403843.1:exon4:c.C895T:p.P299S,	1p36.11	.
1	29495137	C	T	TGTGTGGGTT,AGAACATAAC	exonic	SRSF4	nonsynonymous SNV	SRSF4:ENST00000605204.1:exon2:c.G121A:p.E41K,	1p35.3	.
1	32222205	G	A	CAGGTAGAGG,AGTACTTGGT	exonic	BAI2	nonsynonymous SNV	BAI2:ENST00000398547.1:exon2:c.C197T:p.S66F,BAI2:ENST00000398538.1:exon1:c.C197T:p.S66F,BAI2:ENST00000373658.3:exon4:c.C233T:p.S78F,BAI2:ENST00000527361.1:exon2:c.C233T:p.S78F,BAI2:ENST00000420125.1:exon1:c.C212T:p.S71F,BAI2:ENST00000398556.3:exon2:c.C242T:p.S81F,BAI2:ENS	1p35.2	.
1	32676898	C	T	AGGGAAGGAC,CAGGTGGGAA	exonic	DCDC2B	nonsynonymous SNV	DCDC2B:ENST00000409358.1:exon2:c.C292T:p.P98S,	1p35.1	.
1	33354728	G	A	CACCAACAGC,ATGGCACCAT	exonic	HPCA	nonsynonymous SNV	HPCA:ENST00000373467.3:exon2:c.G229A:p.D77N,	1p35.1	.
1	33583663	C	T	ATGGGTTCCC,CTTTTGGGGG	exonic	ADC	nonsynonymous SNV	ADC:ENST00000373443.3:exon10:c.C1190T:p.P397L,ADC:ENST00000294517.6:exon11:c.C1190T:p.P397L,ADC:ENST00000373441.1:exon8:c.C1250T:p.P417L,ADC:ENST00000398167.1:exon10:c.C1250T:p.P417L,	1p35.1	.
1	35365777	C	T	CTCCTCATCC,CCATGGCTTT	exonic	DLGAP3	nonsynonymous SNV	DLGAP3:ENST00000373347.1:exon4:c.G1205A:p.G402E,DLGAP3:ENST00000235180.4:exon2:c.G1205A:p.G402E,	1p34.3	.
1	36748184	C	T	ACAAACAAAT,CAAGTCTGGA	exonic	THRAP3	nonsynonymous SNV	THRAP3:ENST00000354618.5:exon3:c.C20T:p.S7F,THRAP3:ENST00000478853.1:exon4:c.C20T:p.S7F,THRAP3:ENST00000469141.2:exon4:c.C20T:p.S7F,	1p34.3	.
1	38227502	G	A	GCCTAGGCGG,GACGCCCACG	exonic	EPHA10	nonsynonymous SNV	EPHA10:ENST00000427468.2:exon3:c.C425T:p.P142L,EPHA10:ENST00000373048.4:exon3:c.C425T:p.P142L,EPHA10:ENST00000319637.6:exon3:c.C425T:p.P142L,	1p34.3	.
1	38397399	C	T	CTGCCTGCTT,CTCAAGCTCC	exonic	INPP5B	nonsynonymous SNV	INPP5B:ENST00000373026.1:exon6:c.G718A:p.E240K,INPP5B:ENST00000373021.1:exon6:c.G718A:p.E240K,INPP5B:ENST00000373023.2:exon7:c.G718A:p.E240K,	1p34.3	.
1	39571314	G	A	CTACGTGCCG,GTCAGCAGCC	exonic	MACF1	nonsynonymous SNV	MACF1:ENST00000524432.1:exon1:c.G19A:p.G7S,	1p34.3	.
1	40218603	C	G	ATGGCCAACT,TGGCCCAAAC	exonic	PPIE	nonsynonymous SNV	PPIE:ENST00000372835.5:exon6:c.C563G:p.S188C,PPIE:ENST00000497370.1:exon9:c.C518G:p.S173C,PPIE:ENST00000324379.5:exon9:c.C716G:p.S239C,PPIE:ENST00000372830.1:exon9:c.C716G:p.S239C,PPIE:ENST00000470213.1:exon8:c.C592G:p.L198V,PPIE:ENST00000356511.2:exon9:c.C716G:p.S239	1p34.2	.
1	40771434	G	A	TGCGGGCCCG,CTCACCCTGC	exonic	COL9A2	nonsynonymous SNV	COL9A2:ENST00000372748.3:exon21:c.C1060T:p.P354S,	1p34.2	.
1	41092372	C	T	ACTTGTGGTC,ATGCGGCCAT	exonic	RIMS3	nonsynonymous SNV	RIMS3:ENST00000372684.3:exon8:c.G744A:p.M248I,RIMS3:ENST00000372683.1:exon8:c.G744A:p.M248I,	1p34.2	.
1	42629179	G	A	GGTTCACCAG,GATGGGTGCA	exonic	GUCA2A	nonsynonymous SNV	GUCA2A:ENST00000357001.2:exon2:c.C178T:p.P60S,	1p34.2	.
1	42657046	G	A	TGGTTCGGAT,GTGCTGTATG	exonic	FOXJ3	nonsynonymous SNV	FOXJ3:ENST00000361346.1:exon9:c.C1279T:p.H427Y,FOXJ3:ENST00000372572.1:exon11:c.C1279T:p.H427Y,FOXJ3:ENST00000545068.1:exon9:c.C1279T:p.H427Y,FOXJ3:ENST00000361776.1:exon8:c.C1177T:p.H393Y,FOXJ3:ENST00000372573.1:exon9:c.C1279T:p.H427Y,	1p34.2	.
1	43233184	C	T	TCGCAGGAGC,CTGGTGGAGC	exonic	C1orf50	nonsynonymous SNV	C1orf50:ENST00000603943.1:exon1:c.C202T:p.P68S,	1p34.2	.
1	44202042	G	A	CCAGTGGGAG,AGGACTCCAG	exonic	ST3GAL3	nonsynonymous SNV	ST3GAL3:ENST00000528371.1:exon1:c.G109A:p.E37K,ST3GAL3:ENST00000372362.2:exon1:c.G109A:p.E37K,ST3GAL3:ENST00000353126.3:exon2:c.G109A:p.E37K,ST3GAL3:ENST00000531816.1:exon1:c.G109A:p.E37K,ST3GAL3:ENST00000545417.1:exon2:c.G109A:p.E37K,ST3GAL3:ENST00000372375.2:ex	1p34.1	.
1	45205641	C	T	CCGCCTGTTT,CCGGTCTCGC	exonic	KIF2C	nonsynonymous SNV	KIF2C:ENST00000372224.4:exon1:c.C37T:p.P13S,KIF2C:ENST00000452259.1:exon1:c.C37T:p.P13S,KIF2C:ENST00000372218.4:exon1:c.C37T:p.P13S,	1p34.1	.
1	49056539	T	C	GCAGAGGAGC,TGGGAAAAAT	exonic	AGBL4	nonsynonymous SNV	AGBL4:ENST00000334103.7:exon5:c.A269G:p.K90R,AGBL4:ENST00000371839.1:exon10:c.A1070G:p.K357R,	1p33	.
1	53544397	C	G	CCACCTACTT,CTGGAGGAGC	exonic	PODN	nonsynonymous SNV	PODN:ENST00000395871.2:exon4:c.C933G:p.F311L,PODN:ENST00000312553.5:exon8:c.C1359G:p.F453L,PODN:ENST00000371500.3:exon10:c.C1302G:p.F434L,	1p32.3	.
1	53925422	C	T	CGCCCGCTGT,CCCGGGGACT	exonic	DMRTB1	nonsynonymous SNV	DMRTB1:ENST00000371445.3:exon1:c.C296T:p.S99F,	1p32.3	.
1	54562106	C	T	AAGAGCGGCT,GCTGCCAGTC	exonic	TCEANC2	nonsynonymous SNV	TCEANC2:ENST00000371331.1:exon4:c.C677T:p.S226L,TCEANC2:ENST00000234827.1:exon5:c.C587T:p.S196L,	1p32.3	.
1	55277780	G	A	TATGTGGCAC,ATCCGTCGGC	exonic	C1orf177	nonsynonymous SNV	C1orf177:ENST00000358193.3:exon6:c.G680A:p.R227Q,C1orf177:ENST00000371273.3:exon6:c.G680A:p.R227Q,	1p32.3	.
1	55457663	C	T	CGCCTGGCCT,CACCCAGCTA	exonic	TMEM61	nonsynonymous SNV	TMEM61:ENST00000371268.3:exon3:c.C520T:p.P174S,	1p32.3	.
1	63070447	A	G	AAAATCAACC,AAATGTTGAT	exonic	ANGPTL3	nonsynonymous SNV	ANGPTL3:ENST00000371129.3:exon7:c.A1342G:p.K448E,	1p31.3	.
1	65858311	C	T	GGATGTGGAC,TTTTGGCCCT	exonic	DNAJC6	nonsynonymous SNV	DNAJC6:ENST00000263441.7:exon13:c.C1456T:p.L486F,DNAJC6:ENST00000371069.4:exon12:c.C1666T:p.L556F,DNAJC6:ENST00000395325.3:exon12:c.C1495T:p.L499F,	1p31.3	.
1	67101697	C	T	AAAAAAGTTT,GGTAAGGAAA	exonic	SGIP1	nonsynonymous SNV	SGIP1:ENST00000371037.4:exon4:c.C170T:p.S57L,SGIP1:ENST00000424320.1:exon5:c.C170T:p.S57L,	1p31.3	.
1	67147654	T	G	TCTGTTGCTG,TAATGCTGAA	exonic	SGIP1	nonsynonymous SNV	SGIP1:ENST00000371037.4:exon15:c.T917G:p.V306G,SGIP1:ENST00000237247.6:exon16:c.T929G:p.V310G,	1p31.3	.
1	67880922	C	T	GCCGCCACGT,CTGGGCGGCC	exonic	SERBP1	nonsynonymous SNV	SERBP1:ENST00000370990.5:exon7:c.G1079A:p.G360E,SERBP1:ENST00000370994.4:exon7:c.G1034A:p.G345E,SERBP1:ENST00000361219.6:exon7:c.G1052A:p.G351E,SERBP1:ENST00000370995.2:exon7:c.G1097A:p.G366E,	1p31.3	.
1	70504826	G	A	CATCGCCAAG,ATTTGATTAG	exonic	LRRC7	nonsynonymous SNV	LRRC7:ENST00000310961.5:exon22:c.G3220A:p.D1074N,LRRC7:ENST00000415775.2:exon15:c.G1057A:p.D353N,LRRC7:ENST00000035383.5:exon19:c.G3205A:p.D1069N,	1p31.1	.
1	74937655	A	T	GGATCATCAT,TTTTGATCTA	exonic	LRRC53	nonsynonymous SNV	LRRC53:ENST00000294635.4:exon5:c.T1651A:p.Y551N,	1p31.1	.
1	75005997	G	A	TGACAAATAT,GTAAGTAGGC	exonic	FPGT-TNNI3I	nonsynonymous SNV	TNNI3K:ENST00000326637.3:exon24:c.G2431A:p.G811S,FPGT-TNNI3K:ENST00000557284.2:exon26:c.G2773A:p.G925S,TNNI3K:ENST00000370891.2:exon26:c.G2734A:p.G912S,	1p31.1	.
1	76345796	G	A	ATGAATGAAA,ATGCCAAGAA	exonic	MSH4	nonsynonymous SNV	MSH4:ENST00000263187.3:exon13:c.G1739A:p.R580K,	1p31.1	.
1	78408439	G	T	ATGGAGGAGA,TATATGTGTA	exonic	NEXN	nonsynonymous SNV	NEXN:ENST00000330010.8:exon12:c.G1761T:p.E587D,NEXN:ENST00000334785.7:exon13:c.G1953T:p.E651D,NEXN:ENST00000457030.1:exon12:c.G1911T:p.E637D,	1p31.1	.
1	79102724	C	T	CAGTTTAATT,CCGTAAACCA	exonic	IFI44L	nonsynonymous SNV	IFI44L:ENST00000342282.3:exon5:c.C110T:p.S37F,IFI44L:ENST00000370751.5:exon6:c.C884T:p.S295F,	1p31.1	.
1	79383617	C	T	GTGCAAAAAT,CCTTGTTGTA	exonic	ELTD1	nonsynonymous SNV	ELTD1:ENST00000370742.3:exon11:c.G1580A:p.G527E,	1p31.1	.
1	86315043	C	T	TACCTTTTTT,CTGGACGACC	exonic	COL24A1	nonsynonymous SNV	COL24A1:ENST00000370571.2:exon38:c.G3347A:p.G1116E,COL24A1:ENST00000436319.1:exon38:c.G3347A:p.G1116E,	1p22.3	.
1	86437043	G	A	TTGAGACCAG,AGGTCCAGGA	exonic	COL24A1	nonsynonymous SNV	COL24A1:ENST00000370571.2:exon21:c.C2398T:p.P800S,COL24A1:ENST00000436319.1:exon21:c.C2398T:p.P800S,COL24A1:ENST00000426639.1:exon21:c.C2398T:p.P800S,	1p22.3	.
1	89599054	C	T	TCTTGAGCCT,CATCATTTGC	exonic	GBP7	nonsynonymous SNV	GBP7:ENST00000294671.2:exon10:c.G1549A:p.E517K,	1p22.2	.
1	91182601	G	A	CTCCGAACAG,GAGATGGGGT	exonic	BARHL2	nonsynonymous SNV	BARHL2:ENST00000370445.4:exon1:c.C152T:p.P51L,	1p22.2	.
1	91404020	G	A	GTAAGGACAC,ATTTCTTCTC	exonic	ZNF644	nonsynonymous SNV	ZNF644:ENST00000370440.1:exon3:c.C2891T:p.S964L,ZNF644:ENST00000337393.5:exon3:c.C2891T:p.S964L,	1p22.2	ZNF644;basechange=c.2891C>T;AAchange=p.S964L;tissue=endometrium
1	94502296	C	T	AGCACCATAC,CGCAAACAGA	exonic	ABCA4	nonsynonymous SNV	ABCA4:ENST00000370225.3:exon26:c.G3862A:p.G1288S,	1p22.1	.
1	101705613	C	T	TCGGACAATT,CTCCCACCCC	exonic	S1PR1	nonsynonymous SNV	S1PR1:ENST00000305352.6:exon2:c.C1073T:p.S358F,	1p21.2	.
1	103388908	C	T	ACCAACATCC,CATTTTCACC	exonic	COL11A1	nonsynonymous SNV	COL11A1:ENST00000358392.2:exon47:c.G3674A:p.G1225E,COL11A1:ENST00000353414.4:exon46:c.G3521A:p.G1174E,COL11A1:ENST00000512756.1:exon45:c.G3290A:p.G1097E,COL11A1:ENST00000370096.3:exon47:c.G3638A:p.G1213E,	1p21.1	.
1	103470023	G	A	AGACCTCTTG,TCCAATTTCT	exonic	COL11A1	nonsynonymous SNV	COL11A1:ENST00000358392.2:exon20:c.C1957T:p.P653S,COL11A1:ENST00000353414.4:exon19:c.C1804T:p.P602S,COL11A1:ENST00000512756.1:exon18:c.C1573T:p.P525S,COL11A1:ENST00000370096.3:exon20:c.C1921T:p.P641S,	1p21.1	.
1	103491867	C	T	TAGTCATATT,GATTATATCC	exonic	COL11A1	nonsynonymous SNV	COL11A1:ENST00000512756.1:exon6:c.G802A:p.E268K,COL11A1:ENST00000370096.3:exon6:c.G802A:p.E268K,	1p21.1	.
1	109779138	C	T	GCTTCGAATC,GATATGGGCA	exonic	SARS	stopgain	SARS:ENST00000369923.4:exon9:c.C1225T:p.R409X,SARS:ENST00000234677.2:exon9:c.C1225T:p.R409X,	1p13.3	.
1	111147359	G	A	GGGTGCCCAG,GAGGGCAGCA	exonic	KCNA2	nonsynonymous SNV	KCNA2:ENST00000485317.1:exon3:c.C46T:p.P16S,KCNA2:ENST00000440270.1:exon2:c.C46T:p.P16S,KCNA2:ENST00000316361.4:exon3:c.C46T:p.P16S,KCNA2:ENST00000369770.3:exon3:c.C46T:p.P16S,	1p13.3	.
1	111216419	G	A	GATAAAATAA,GAATGATGGC	exonic	KCNA3	nonsynonymous SNV	KCNA3:ENST00000369769.2:exon1:c.C1013T:p.P338L,	1p13.3	.
1	111216420	G	A	ATAAAATAAG,AATGATGGCC	exonic	KCNA3	nonsynonymous SNV	KCNA3:ENST00000369769.2:exon1:c.C1012T:p.P338S,	1p13.3	.
1	114454578	C	T	GGTTTAGGGT,CCCCTTGGTA	exonic	DCLRE1B	nonsynonymous SNV	DCLRE1B:ENST00000369563.3:exon4:c.C1364T:p.S455F,	1p13.2	.
1	115220095	G	A	GCGGGGCTCA,CATGCTGGTA	exonic	AMPD1	nonsynonymous SNV	AMPD1:ENST00000520113.2:exon10:c.C1364T:p.A455V,AMPD1:ENST00000369538.3:exon9:c.C1352T:p.A451V,AMPD1:ENST00000353928.6:exon10:c.C1265T:p.A422V,	1p13.2	.
1	120166295	G	A	CAGGCCGAAG,GCTCCAGGCT	exonic	ZNF697	nonsynonymous SNV	ZNF697:ENST00000421812.2:exon3:c.C671T:p.P224L,	1p12	.
1	120166296	G	A	AGGCCGAAGG,CTCCAGGCTG	exonic	ZNF697	nonsynonymous SNV	ZNF697:ENST00000421812.2:exon3:c.C670T:p.P224S,	1p12	.
1	120345546	C	T	AACACACAAC,CTCTTCTCTC	exonic	REG4	nonsynonymous SNV	REG4:ENST00000369401.4:exon3:c.G310A:p.G104S,	1p12	.
1	120466566	C	T	CCCCTGGTCA,AGTGGTTGTC	exonic	NOTCH2	nonsynonymous SNV	NOTCH2:ENST00000256646.2:exon26:c.G4553A:p.C1518Y,	1p12	.
1	145562524	G	A	CACCCTGGTG,ATCGGCACCG	exonic	ANKRD35	nonsynonymous SNV	ANKRD35:ENST00000355594.4:exon10:c.G2212A:p.D738N,	1q21.1	.
1	150131075	C	T	GAAGACCCTT,CCCTGAGGAA	exonic	PLEKHO1	nonsynonymous SNV	PLEKHO1:ENST00000369124.4:exon6:c.C587T:p.S196F,PLEKHO1:ENST00000369126.1:exon5:c.C38T:p.S13F,PLEKHO1:ENST00000441340.1:exon3:c.C227T:p.S76F,PLEKHO1:ENST00000025469.6:exon5:c.C485T:p.S162F,	1q21.2	.
1	150445008	C	T	CATCATCTTC,CCCATCCCCC	exonic	RPRD2	nonsynonymous SNV	RPRD2:ENST00000369068.4:exon11:c.C3584T:p.P1195L,RPRD2:ENST00000401000.4:exon10:c.C3506T:p.P1169L,	1q21.3	.
1	150771720	C	T	TTCAGATTAT,GCTATTGCAG	exonic	CTSK	nonsynonymous SNV	CTSK:ENST00000271651.3:exon7:c.G814A:p.D272N,	1q21.3	.
1	151108622	C	T	TCCCCCTACT,CTGCACAGGA	exonic	SEMA6C	nonsynonymous SNV	SEMA6C:ENST00000341697.3:exon13:c.G1124A:p.G375E,SEMA6C:ENST00000368913.3:exon13:c.G1124A:p.G375E,SEMA6C:ENST00000368912.3:exon12:c.G1004A:p.G335E,SEMA6C:ENST00000368914.3:exon13:c.G1124A:p.G375E,	1q21.3	.
1	151773430	C	T	CCGGTTACCC,CAGAGTTTTT	exonic	LINGO4	nonsynonymous SNV	LINGO4:ENST00000368820.3:exon2:c.G1751A:p.G584E,	1q21.3	.
1	151810771	C	T	CCGCAGCCGC,CGGGCCCGGC	exonic	C2CD4D	nonsynonymous SNV	C2CD4D:ENST00000454109.1:exon2:c.G695A:p.G232E,	1q21.3	.
1	151810772	C	T	CGCAGCCGCC,GGGCCCGGCC	exonic	C2CD4D	nonsynonymous SNV	C2CD4D:ENST00000454109.1:exon2:c.G694A:p.G232R,	1q21.3	.
1	152193774	C	T	TCTTCTTGTT,CTCTTGGTGC	exonic	HRNR	nonsynonymous SNV	HRNR:ENST00000368801.2:exon3:c.G331A:p.E111K,	1q21.3	.
1	152278724	C	T	CTCCTGGACC,CTCTGATTGT	exonic	FLG	nonsynonymous SNV	FLG:ENST00000368799.1:exon3:c.G8638A:p.G2880R,	1q21.3	.
1	152280052	C	T	TCCTTGTCTT,CTCCAGTGCT	exonic	FLG	nonsynonymous SNV	FLG:ENST00000368799.1:exon3:c.G7310A:p.G2437E,	1q21.3	.
1	152281478	C	T	GCTCTGTCTT,GTGATGGGAC	exonic	FLG	nonsynonymous SNV	FLG:ENST00000368799.1:exon3:c.G5884A:p.E1962K,	1q21.3	.
1	153276470	G	A	TAAGGCAGCA,GGCTGGGACT	exonic	PGLYRP3	nonsynonymous SNV	PGLYRP3:ENST00000290722.1:exon4:c.C392T:p.P131L,	1q21.3	.
1	153390680	C	T	GAGAACTTCC,CAATTTCCTC	exonic	S100A7A	nonsynonymous SNV	S100A7A:ENST00000368728.2:exon2:c.C122T:p.P41L,S100A7A:ENST00000368729.4:exon2:c.C122T:p.P41L,S100A7A:ENST00000329256.2:exon1:c.C122T:p.P41L,	1q21.3	.
1	153945326	C	T	ACATGCTTCC,CTAAGGGTAT	exonic	CREB3L4	nonsynonymous SNV	CREB3L4:ENST00000368601.1:exon5:c.C650T:p.P217L,	1q21.3	.
1	153946117	C	T	TTCCCTGGCT,TCATCATCCT	exonic	CREB3L4	nonsynonymous SNV	CREB3L4:ENST00000368607.3:exon9:c.C919T:p.L307F,CREB3L4:ENST00000271889.4:exon9:c.C919T:p.L307F,CREB3L4:ENST00000405694.3:exon8:c.C478T:p.L160F,CREB3L4:ENST00000368600.3:exon9:c.C859T:p.L287F,CREB3L4:ENST00000368603.1:exon9:c.C919T:p.L307F,	1q21.3	.
1	154229908	C	G	ACATGCCTAC,CGGTAAGTGG	exonic	UBAP2L	nonsynonymous SNV	UBAP2L:ENST00000343815.6:exon20:c.C2440G:p.P814A,UBAP2L:ENST00000361546.2:exon19:c.C2440G:p.P814A,UBAP2L:ENST00000271877.7:exon20:c.C2473G:p.P825A,UBAP2L:ENST00000428931.1:exon20:c.C2440G:p.P814A,	1q21.3	.
1	154243375	G	A	CAATTTCGTC,ATGACCAGCT	exonic	UBAP2L	nonsynonymous SNV	UBAP2L:ENST00000271877.7:exon27:c.G3217A:p.D1073N,	1q21.3	.
1	154319147	G	A	CTTCATCTGG,GAAGCCTTGC	exonic	ATP8B2	nonsynonymous SNV	ATP8B2:ENST00000368489.3:exon26:c.G3175A:p.G1059R,	1q21.3	.
1	154987671	G	A	CAATGGTGAA,ACAGTCCTCC	exonic	ZBTB7B	nonsynonymous SNV	ZBTB7B:ENST00000417934.2:exon4:c.G637A:p.D213N,ZBTB7B:ENST00000368426.3:exon3:c.G535A:p.D179N,ZBTB7B:ENST00000535420.1:exon4:c.G535A:p.D179N,ZBTB7B:ENST00000292176.2:exon1:c.G535A:p.D179N,	1q21.3	.
1	155920741	G	A	GCCCAGGGCG,CCAGCTGCCT	exonic	ARHGEF2	nonsynonymous SNV	ARHGEF2:ENST00000313695.7:exon20:c.C2498T:p.A833V,ARHGEF2:ENST00000368315.4:exon20:c.C2585T:p.A862V,ARHGEF2:ENST00000313667.4:exon20:c.C2579T:p.A860V,ARHGEF2:ENST00000368316.1:exon24:c.C2498T:p.A833V,ARHGEF2:ENST00000361247.4:exon20:c.C2582T:p.A861V,ARHGEF2:EN	1q22	.
1	156306966	T	C	GAGGCCGAGG,GGGCGGATCA	exonic	CCT3	nonsynonymous SNV	CCT3:ENST00000368262.3:exon2:c.A97G:p.T33A,	1q22	.
1	156377728	C	T	GTGGAGATTC,ACAATGGCTT	exonic	C1orf61	nonsynonymous SNV	C1orf61:ENST00000368243.1:exon5:c.G211A:p.G71R,C1orf61:ENST00000310027.5:exon4:c.G253A:p.G85R,C1orf61:ENST00000357975.4:exon4:c.G250A:p.G84R,	1q22	.
1	156823573	C	T	GGTCCAGCAT,TGTAGTCAGT	exonic	INSRR	nonsynonymous SNV	INSRR:ENST00000368195.3:exon2:c.G608A:p.R203K,	1q23.1	.
1	157665845	G	A	ACGGTGACTC,AATCCACGTG	exonic	FCRL3	stopgain	FCRL3:ENST00000477837.1:exon6:c.C832T:p.R278X,FCRL3:ENST00000368184.3:exon7:c.C1117T:p.R373X,FCRL3:ENST00000492769.1:exon7:c.C1117T:p.R373X,FCRL3:ENST00000485028.1:exon7:c.C1117T:p.R373X,FCRL3:ENST00000368186.5:exon7:c.C1117T:p.R373X,	1q23.1	FCRL3;basechange=c.1117C>T;AAchange=p.R373*;tissue=large_intestine
1	158325784	G	A	TAATGCTGAC,AGACATGGTA	exonic	CD1E	nonsynonymous SNV	CD1E:ENST00000434258.1:exon4:c.G787A:p.E263K,CD1E:ENST00000368167.3:exon4:c.G793A:p.E265K,CD1E:ENST00000368165.3:exon3:c.G523A:p.E175K,CD1E:ENST00000452291.2:exon2:c.G226A:p.E76K,CD1E:ENST00000368161.3:exon4:c.G793A:p.E265K,CD1E:ENST00000368160.3:exon4:c.G793A:p.E2	1q23.1	CD1E;basechange=c.793G>A;AAchange=p.E265K;tissue=large_intestine
1	158908228	C	T	AGGAATAATC,CATCTAAAAA	exonic	PYHIN1	nonsynonymous SNV	PYHIN1:ENST00000368140.1:exon3:c.C307T:p.P103S,PYHIN1:ENST00000368138.3:exon3:c.C280T:p.P94S,PYHIN1:ENST00000368135.4:exon3:c.C307T:p.P103S,PYHIN1:ENST00000392252.3:exon3:c.C280T:p.P94S,PYHIN1:ENST00000458222.1:exon4:c.C307T:p.P103S,PYHIN1:ENST00000392254.2:exon3:c.C	1q23.1	.
1	158909027	C	T	CCCAACACTT,CTCAACTGAG	exonic	PYHIN1	nonsynonymous SNV	PYHIN1:ENST00000368140.1:exon4:c.C569T:p.S190F,PYHIN1:ENST00000368138.3:exon4:c.C542T:p.S181F,PYHIN1:ENST00000368135.4:exon4:c.C569T:p.S190F,PYHIN1:ENST00000392252.3:exon4:c.C542T:p.S181F,PYHIN1:ENST00000392254.2:exon4:c.C569T:p.S190F,	1q23.1	.
1	159283503	C	T	TTCACAACCT,TCTTCATTAA	exonic	OR10J3	nonsynonymous SNV	OR10J3:ENST00000332217.5:exon1:c.G947A:p.R316K,	1q23.2	OR10J3;basechange=c.947G>A;AAchange=p.R316K;tissue=skin
1	159857723	C	T	GCCACCTCCT,CAGGTCACTG	exonic	CCDC19	nonsynonymous SNV	CCDC19:ENST00000426543.2:exon5:c.G241A:p.E81K,CCDC19:ENST00000368099.4:exon5:c.G496A:p.E166K,	1q23.2	.
1	160209735	G	A	TCCCGCTCCC,AAGGGCAGGG	exonic	DCAF8	nonsynonymous SNV	DCAF8:ENST00000326837.2:exon4:c.C475T:p.R159W,DCAF8:ENST00000610139.1:exon4:c.C475T:p.R159W,DCAF8:ENST00000461888.1:exon4:c.C475T:p.R159W,DCAF8:ENST00000608310.1:exon8:c.C937T:p.R313W,DCAF8:ENST00000368074.1:exon4:c.C475T:p.R159W,DCAF8:ENST00000447377.1:exon3:c.	1q23.2	.
1	160917820	C	T	GCAACAAATT,CCCTGAAAAA	exonic	ITLN2	nonsynonymous SNV	ITLN2:ENST00000368029.3:exon7:c.G724A:p.E242K,	1q23.3	.
1	161035849	C	T	GAGACGGAAT,TCACCATGTT	exonic	AL591806.1	nonsynonymous SNV	AL591806.1:ENST00000600454.1:exon2:c.C56T:p.S19F,	1q23.3	.
1	162353346	G	A	GAGAGGAATG,CTTCAAACTC	exonic	C1orf226	nonsynonymous SNV	C1orf226:ENST00000458626.2:exon2:c.G692A:p.G231D,C1orf226:ENST00000426197.2:exon3:c.G821A:p.G274D,	1q23.3	.
1	165175157	G	A	CTGTCGGAAG,GATCTGAGCT	exonic	LMX1A	nonsynonymous SNV	LMX1A:ENST00000294816.2:exon8:c.C932T:p.P311L,LMX1A:ENST00000342310.3:exon8:c.C932T:p.P311L,LMX1A:ENST00000367893.4:exon7:c.C932T:p.P311L,	1q23.3	.
1	167064195	C	T	CTCCCCTAGC,AGTAAGTGAC	exonic	DUSP27	stopgain	DUSP27:ENST00000361200.2:exon2:c.C109T:p.Q37X,DUSP27:ENST00000271385.5:exon2:c.C109T:p.Q37X,DUSP27:ENST00000443333.1:exon1:c.C109T:p.Q37X,	1q24.1	.
1	169521929	C	T	TAATGTTTTC,AATTTGGTTT	exonic	F5	nonsynonymous SNV	F5:ENST00000367796.3:exon8:c.G1162A:p.G388R,F5:ENST00000367797.3:exon8:c.G1162A:p.G388R,F5:ENST00000546081.1:exon7:c.G751A:p.G251R,	1q24.2	.
1	171605528	G	A	CACTGTCTCG,TATTCAGCTC	exonic	MYOC	nonsynonymous SNV	MYOC:ENST00000037502.6:exon3:c.C1052T:p.T351I,	1q24.3	.
1	171753372	C	T	CAGGCCAGTC,CTGGCTCTGC	exonic	METTL13	nonsynonymous SNV	METTL13:ENST00000458517.1:exon3:c.C643T:p.P215S,METTL13:ENST00000362019.3:exon2:c.C388T:p.P130S,METTL13:ENST00000361735.3:exon2:c.C646T:p.P216S,	1q24.3	.
1	175048468	G	A	CTCCCTCCCA,ATCTAAGCCG	splicing	TNN(ENST00	.	.	1q25.1	.
1	178489812	C	T	CCCCAGTCCC,TTAGAAGAGC	exonic	TEX35	nonsynonymous SNV	TEX35:ENST00000367641.3:exon7:c.C346T:p.L116F,TEX35:ENST00000319416.2:exon7:c.C346T:p.L116F,TEX35:ENST00000258298.2:exon7:c.C118T:p.L40F,TEX35:ENST00000367639.1:exon7:c.C370T:p.L124F,TEX35:ENST00000367643.3:exon7:c.C346T:p.L116F,	1q25.2	.
1	181480613	G	A	CGCAATGGCT,GAATGTCATG	exonic	CACNA1E	stopgain	CACNA1E:ENST00000367573.2:exon3:c.G479A:p.W160X,CACNA1E:ENST00000524607.1:exon4:c.G479A:p.W160X,CACNA1E:ENST00000367570.1:exon3:c.G479A:p.W160X,CACNA1E:ENST00000526775.1:exon3:c.G479A:p.W160X,CACNA1E:ENST00000360108.3:exon3:c.G479A:p.W160X,CACNA1E:ENST0000	1q25.3	.
1	181701866	C	T	GCGGGAGCCA,CATGGCTGGC	exonic	CACNA1E	nonsynonymous SNV	CACNA1E:ENST00000367573.2:exon20:c.C2644T:p.P882S,CACNA1E:ENST00000367570.1:exon20:c.C2644T:p.P882S,CACNA1E:ENST00000526775.1:exon19:c.C2587T:p.P863S,CACNA1E:ENST00000360108.3:exon19:c.C2587T:p.P863S,CACNA1E:ENST00000357570.5:exon20:c.C2497T:p.P833S,CACNA1E:EN	1q25.3	.
1	181701891	G	A	CCCTGTCATG,AAACTGTGAC	exonic	CACNA1E	nonsynonymous SNV	CACNA1E:ENST00000367573.2:exon20:c.G2669A:p.G890E,CACNA1E:ENST00000367570.1:exon20:c.G2669A:p.G890E,CACNA1E:ENST00000526775.1:exon19:c.G2612A:p.G871E,CACNA1E:ENST00000360108.3:exon19:c.G2612A:p.G871E,CACNA1E:ENST00000357570.5:exon20:c.G2522A:p.G841E,CACNA1E:	1q25.3	.
1	182442824	A	G	TGGCTTCCCA,CTTTTACACC	exonic	RGSL1	nonsynonymous SNV	RGSL1:ENST00000542961.1:exon7:c.A683G:p.N228S,RGSL1:ENST00000294854.8:exon6:c.A578G:p.N193S,RGSL1:ENST00000443996.2:exon7:c.A683G:p.N228S,	1q25.3	.
1	183502933	G	C	CAGACCCAGG,TCTTTCAGGA	exonic	SMG7	nonsynonymous SNV	SMG7:ENST00000456731.2:exon10:c.G1003C:p.V335L,SMG7:ENST00000515829.2:exon10:c.G1129C:p.V377L,SMG7:ENST00000419169.1:exon9:c.G1003C:p.V335L,SMG7:ENST00000347615.2:exon10:c.G1129C:p.V377L,SMG7:ENST00000508461.1:exon9:c.G1003C:p.V335L,SMG7:ENST00000367537.3:exon11	1q25.3	.
1	183909849	T	G	AATAGTCGGC,TCGACCAGGT	exonic	COLGALT2	nonsynonymous SNV	COLGALT2:ENST00000546159.1:exon11:c.A1470C:p.E490D,COLGALT2:ENST00000367520.3:exon6:c.A681C:p.E227D,COLGALT2:ENST00000367521.1:exon3:c.A294C:p.E98D,COLGALT2:ENST00000361927.4:exon11:c.A1470C:p.E490D,	1q25.3	.
1	186266027	C	T	AAAACACTTC,CATTTACCTG	exonic	PRG4	nonsynonymous SNV	PRG4:ENST00000367484.3:exon2:c.C20T:p.P7L,PRG4:ENST00000367482.4:exon2:c.C20T:p.P7L,PRG4:ENST00000445192.2:exon2:c.C20T:p.P7L,PRG4:ENST00000367486.3:exon2:c.C20T:p.P7L,PRG4:ENST00000533951.1:exon2:c.C20T:p.P7L,PRG4:ENST00000367483.4:exon2:c.C20T:p.P7L,PRG4:ENST0000	1q31.1	.
1	186276602	C	T	CCCAAGGAGC,TGCACCCACC	exonic	PRG4	nonsynonymous SNV	PRG4:ENST00000367482.4:exon6:c.C1349T:p.P450L,PRG4:ENST00000445192.2:exon7:c.C1751T:p.P584L,PRG4:ENST00000367486.3:exon6:c.C1622T:p.P541L,PRG4:ENST00000367483.4:exon6:c.C1628T:p.P543L,PRG4:ENST00000367485.4:exon5:c.C1472T:p.P491L,	1q31.1	.
1	196871590	T	G	CATGGAGGTC,ATATTATAAG	exonic	CFHR4	nonsynonymous SNV	CFHR4:ENST00000251424.4:exon2:c.T101G:p.L34R,CFHR4:ENST00000367418.2:exon2:c.T101G:p.L34R,CFHR4:ENST00000367416.2:exon2:c.T98G:p.L33R,	1q31.3	.
1	197404034	G	A	CAATTGCAAA,TGGCAACAGC	exonic	CRB1	nonsynonymous SNV	CRB1:ENST00000367397.1:exon5:c.G1184A:p.S395N,CRB1:ENST00000535699.1:exon12:c.G2969A:p.S990N,CRB1:ENST00000484075.1:exon9:c.G3041A:p.S1014N,CRB1:ENST00000544212.1:exon5:c.G1484A:p.S495N,CRB1:ENST00000367399.2:exon7:c.G2705A:p.S902N,CRB1:ENST00000367400.3:exon9:c.G	1q31.3	.
1	200534674	G	A	GTCTGCTATA,TTCTTTCTTC	exonic	KIF14	nonsynonymous SNV	KIF14:ENST00000367350.4:exon24:c.C3785T:p.T1262I,	1q32.1	.
1	200974494	G	A	GATGGCGTGG,AGCGTGAGCT	exonic	KIF21B	nonsynonymous SNV	KIF21B:ENST00000332129.2:exon5:c.C674T:p.S225F,KIF21B:ENST00000422435.2:exon5:c.C674T:p.S225F,KIF21B:ENST00000461742.2:exon5:c.C674T:p.S225F,KIF21B:ENST00000360529.5:exon5:c.C674T:p.S225F,	1q32.1	.
1	201081318	C	T	AAGGATACTT,CATTCTACAA	exonic	CACNA1S	stopgain	CACNA1S:ENST00000362061.3:exon1:c.G150A:p.W50X,CACNA1S:ENST00000367338.3:exon1:c.G150A:p.W50X,	1q32.1	.
1	201081319	C	T	AGGATACTTC,ATTCTACAAT	exonic	CACNA1S	stopgain	CACNA1S:ENST00000362061.3:exon1:c.G149A:p.W50X,CACNA1S:ENST00000367338.3:exon1:c.G149A:p.W50X,	1q32.1	.
1	202735565	C	T	ATCTAGGAGG,GGAGGTTGCA	exonic	KDM5B	nonsynonymous SNV	KDM5B:ENST00000367264.2:exon6:c.G779A:p.R260H,	1q32.1	.
1	204106339	C	T	AGCTGGGTCT,CCGCGCCGGG	exonic	ETNK2	nonsynonymous SNV	ETNK2:ENST00000367199.2:exon5:c.G700A:p.E234K,ETNK2:ENST00000452983.1:exon5:c.G478A:p.E160K,ETNK2:ENST00000367201.3:exon6:c.G907A:p.E303K,ETNK2:ENST00000367202.4:exon6:c.G907A:p.E303K,ETNK2:ENST00000367198.2:exon5:c.G373A:p.E125K,ETNK2:ENST00000422699.1:exon5:c.G5	1q32.1	.
1	204981969	G	A	AGACCCCAAG,AAGAGGATGG	exonic	NFASC	nonsynonymous SNV	NFASC:ENST00000339876.6:exon29:c.G3466A:p.E1156K,NFASC:ENST00000401399.1:exon28:c.G3466A:p.E1156K,NFASC:ENST00000513543.1:exon24:c.G3253A:p.E1085K,NFASC:ENST00000447819.1:exon3:c.G400A:p.E134K,NFASC:ENST00000367171.4:exon29:c.G3742A:p.E1248K,NFASC:ENST00000539	1q32.1	.
1	205035011	C	T	GACAGCGCGT,CAAGGAGGCC	exonic	CNTN2	nonsynonymous SNV	CNTN2:ENST00000331830.4:exon14:c.C1790T:p.S597F,	1q32.1	.
1	205588176	G	A	GGAGAGCAAG,GGCTTGGAGT	exonic	ELK4	nonsynonymous SNV	ELK4:ENST00000357992.4:exon4:c.C1106T:p.P369L,	1q32.1	.
1	208072523	C	T	GTTTGTGTTT,CATAAACTGA	exonic	CD34	nonsynonymous SNV	CD34:ENST00000310833.7:exon3:c.G311A:p.G104E,CD34:ENST00000356522.4:exon3:c.G311A:p.G104E,	1q32.2	.
1	220089235	C	T	TTACAAGTTC,CAGATGTGCA	exonic	SLC30A10	stopgain	SLC30A10:ENST00000536446.1:exon4:c.G279A:p.W93X,SLC30A10:ENST00000366926.3:exon4:c.G1014A:p.W338X,	1q41	.
1	220275744	C	T	GTTTTGGTCT,CGTCATCTAG	exonic	IARS2	nonsynonymous SNV	IARS2:ENST00000302637.5:exon5:c.C739T:p.P247S,IARS2:ENST00000366922.1:exon5:c.C523T:p.P175S,	1q41	.
1	220863826	G	A	GCGAACCGAG,GGGACGAGGA	exonic	C1orf115	nonsynonymous SNV	C1orf115:ENST00000294889.5:exon1:c.G82A:p.G28R,	1q41	.
1	223178638	C	T	CAGTGCTCTC,TACCACTAGC	exonic	DISP1	nonsynonymous SNV	DISP1:ENST00000284476.6:exon8:c.C3899T:p.P1300L,	1q41	.
1	225237926	C	T	AATTACTCCT,TTTGCCAAGA	exonic	DNAH14	nonsynonymous SNV	DNAH14:ENST00000430092.1:exon16:c.C1927T:p.L643F,DNAH14:ENST00000439375.2:exon15:c.C1927T:p.L643F,DNAH14:ENST00000445597.2:exon12:c.C1840T:p.L614F,	1q42.12	.
1	227152773	C	T	CTTCTCAGTC,CGCATGCAGC	exonic	ADCK3	nonsynonymous SNV	ADCK3:ENST00000366777.3:exon3:c.C250T:p.P84S,ADCK3:ENST00000366779.1:exon8:c.C250T:p.P84S,ADCK3:ENST00000366778.1:exon3:c.C94T:p.P32S,	1q42.13	.
1	228295708	G	A	GGGGCAGGAT,AGCCTCGGGG	exonic	MRPL55	nonsynonymous SNV	MRPL55:ENST00000366731.5:exon4:c.C104T:p.S35L,MRPL55:ENST00000430433.1:exon4:c.C104T:p.S35L,MRPL55:ENST00000366738.1:exon3:c.C104T:p.S35L,	1q42.13	.
1	230314051	C	T	GGAGACCCTC,CTCCAGGTAC	exonic	GALNT2	nonsynonymous SNV	GALNT2:ENST00000543760.1:exon2:c.C100T:p.P34S,GALNT2:ENST00000366672.4:exon2:c.C214T:p.P72S,	1q42.13	GALNT2;basechange=c.214C>T;AAchange=p.P72S;tissue=skin
1	231298836	G	A	GACCCCGGAC,GTGAGCAGCA	exonic	TRIM67	nonsynonymous SNV	TRIM67:ENST00000366652.2:exon1:c.G121A:p.G41S,TRIM67:ENST00000449018.3:exon1:c.G121A:p.G41S,TRIM67:ENST00000444294.3:exon1:c.G121A:p.G41S,TRIM67:ENST00000366653.5:exon1:c.G121A:p.G41S,	1q42.2	TRIM67;basechange=c.121G>A;AAchange=p.G41S;tissue=large_intestine&TRIM67_ENST00000366653;basechange=c.121G>A;AAchange=p.G41S;tissue=large_intestine
1	236212039	G	A	GGGGGCCACG,ATTCCCAAGT	exonic	NID1	nonsynonymous SNV	NID1:ENST00000264187.6:exon2:c.C476T:p.S159F,NID1:ENST00000366595.3:exon2:c.C476T:p.S159F,	1q42.3	.
1	240072012	C	T	AGGCAGTTTT,CAAAAAGCTT	exonic	CHRM3	nonsynonymous SNV	CHRM3:ENST00000255380.4:exon5:c.C1261T:p.P421S,	1q43	CHRM3;basechange=c.1261C>T;AAchange=p.P421S;tissue=skin
1	240072013	C	T	GGCAGTTTTC,AAAAAGCTTC	exonic	CHRM3	nonsynonymous SNV	CHRM3:ENST00000255380.4:exon5:c.C1262T:p.P421L,	1q43	CHRM3;basechange=c.1262C>T;AAchange=p.P421L;tissue=skin
1	240370393	G	A	GCCTCCTGTG,ATGGGCTGCC	exonic	FMN2	nonsynonymous SNV	FMN2:ENST00000447095.1:exon7:c.G592A:p.D198N,FMN2:ENST00000319653.9:exon5:c.G2281A:p.D761N,	1q43	.
1	243668610	C	T	CTCTCATTGT,CATGCAGTCC	exonic	AKT3	nonsynonymous SNV	AKT3:ENST00000366539.1:exon14:c.G1381A:p.D461N,AKT3:ENST00000263826.5:exon13:c.G1381A:p.D461N,	1q43	.
1	243668611	C	T	TCTCATTGTC,ATGCAGTCCA	exonic	AKT3	nonsynonymous SNV	AKT3:ENST00000366539.1:exon14:c.G1380A:p.M460I,AKT3:ENST00000263826.5:exon13:c.G1380A:p.M460I,	1q43	.
1	245851915	G	A	GTGCTGAGCG,GGAGCTCCCG	exonic	KIF26B	nonsynonymous SNV	KIF26B:ENST00000407071.2:exon12:c.G5630A:p.G1877E,KIF26B:ENST00000366518.4:exon9:c.G4487A:p.G1496E,	1q44	.
1	247712498	G	A	GAAAAGATGG,AAATTATCTC	exonic	GCSAML	nonsynonymous SNV	GCSAML:ENST00000366490.3:exon4:c.G367A:p.E123K,GCSAML:ENST00000366491.2:exon4:c.G5A:p.G2E,GCSAML:ENST00000536561.1:exon1:c.G5A:p.G2E,GCSAML:ENST00000526896.1:exon3:c.G5A:p.G2E,GCSAML:ENST00000366488.4:exon1:c.G5A:p.G2E,GCSAML:ENST00000366489.1:exon3:c.G5A:p.G	1q44	.
1	248525923	G	A	AAAAAGCTAT,GAGTAGACCA	exonic	OR2T4	nonsynonymous SNV	OR2T4:ENST00000366475.1:exon1:c.G1041A:p.M347I,	1q44	.
2	1079279	G	A	GCTGACGAAA,AGGTGCTGAC	exonic	SNTG2	nonsynonymous SNV	SNTG2:ENST00000450962.1:exon2:c.G148A:p.E50K,SNTG2:ENST00000407292.1:exon2:c.G148A:p.E50K,SNTG2:ENST00000308624.5:exon2:c.G148A:p.E50K,SNTG2:ENST00000452177.1:exon2:c.G148A:p.E50K,	2p25.3	SNTG2;basechange=c.148G>A;AAchange=p.E50K;tissue=oesophagus
2	3624161	C	T	AATCCAAGTC,GGCTAGTACG	exonic	RPS7	nonsynonymous SNV	RPS7:ENST00000403564.1:exon4:c.C232T:p.R78W,RPS7:ENST00000407445.3:exon4:c.C232T:p.R78W,RPS7:ENST00000304921.5:exon4:c.C232T:p.R78W,RPS7:ENST00000406376.1:exon4:c.C232T:p.R78W,	2p25.3	.
2	7179887	C	T	CCGTTACCCA,CTAGAGGAAG	exonic	RNF144A	nonsynonymous SNV	RNF144A:ENST00000320892.6:exon9:c.C875T:p.T292I,	2p25.1	.
2	9531310	C	T	GCCCCCTCTT,GCGTGACATC	exonic	ASAP2	nonsynonymous SNV	ASAP2:ENST00000281419.3:exon23:c.C2503T:p.R835C,	2p25.1	.
2	9994521	C	T	TTGTAAGAAC,CAGTTTATAC	exonic	TAF1B	nonsynonymous SNV	TAF1B:ENST00000434858.1:exon5:c.C370T:p.P124S,TAF1B:ENST00000263663.5:exon5:c.C370T:p.P124S,	2p25.1	.
2	10712268	C	T	CTTTTCCTTT,TTGTCGATGC	exonic	NOL10	nonsynonymous SNV	NOL10:ENST00000542668.1:exon20:c.G1846A:p.E616K,NOL10:ENST00000345985.3:exon20:c.G1846A:p.E616K,NOL10:ENST00000538384.1:exon20:c.G1918A:p.E640K,NOL10:ENST00000381685.5:exon21:c.G1996A:p.E666K,	2p25.1	.
2	11742628	G	A	GCTCAGATAC,ATAGCTCCTT	exonic	GREB1	nonsynonymous SNV	GREB1:ENST00000432985.1:exon8:c.G1528A:p.D510N,GREB1:ENST00000234142.5:exon16:c.G2626A:p.D876N,GREB1:ENST00000381486.2:exon17:c.G2626A:p.D876N,	2p25.1	.
2	11758678	C	T	TCCTCCTCCT,GGGCTCATCC	exonic	GREB1	nonsynonymous SNV	GREB1:ENST00000396123.1:exon5:c.C671T:p.S224L,GREB1:ENST00000234142.5:exon21:c.C3677T:p.S1226L,GREB1:ENST00000381486.2:exon22:c.C3677T:p.S1226L,	2p25.1	.
2	11767127	G	A	CTTTATGTGC,GCGTCAGACG	exonic	GREB1	nonsynonymous SNV	GREB1:ENST00000396123.1:exon8:c.G1340A:p.R447Q,GREB1:ENST00000234142.5:exon24:c.G4346A:p.R1449Q,GREB1:ENST00000381486.2:exon25:c.G4346A:p.R1449Q,	2p25.1	.
2	11770205	G	C	GCCAGCTGGA,AGCATGCGAC	exonic	GREB1	nonsynonymous SNV	GREB1:ENST00000396123.1:exon9:c.G1575C:p.E525D,GREB1:ENST00000234142.5:exon25:c.G4581C:p.E1527D,GREB1:ENST00000381486.2:exon26:c.G4581C:p.E1527D,	2p25.1	.
2	17699637	G	A	GAGGAGGTAG,CTTTCTGAGC	exonic	RAD51AP2	nonsynonymous SNV	RAD51AP2:ENST00000399080.2:exon1:c.C46T:p.P16S,	2p24.2	.
2	21227229	G	A	GGAGGCTGCT,AGGTGGAGAT	exonic	APOB	nonsynonymous SNV	APOB:ENST00000233242.1:exon28:c.C11999T:p.S4000L,	2p24.1	.
2	24253989	G	A	AGGTTAACGG,ATAAAGCCCT	exonic	C2orf44	nonsynonymous SNV	C2orf44:ENST00000406895.3:exon3:c.C1862T:p.S621F,	2p23.3	.
2	25387533	G	A	TCCGTGGTGA,GTCCTGACAC	exonic	POMC	nonsynonymous SNV	POMC:ENST00000405623.1:exon2:c.C109T:p.L37F,POMC:ENST00000380794.1:exon3:c.C109T:p.L37F,POMC:ENST00000449220.1:exon3:c.C109T:p.L37F,POMC:ENST00000264708.3:exon3:c.C109T:p.L37F,POMC:ENST00000395826.2:exon2:c.C109T:p.L37F,	2p23.3	.
2	26251397	C	T	ATACGGACCT,GGTGGCCTGG	exonic	AC013449.1	nonsynonymous SNV	AC013449.1:ENST00000599234.1:exon1:c.G85A:p.E29K,	2p23.3	.
2	26785551	C	T	GGACTCATGC,CGGGTAAGGC	exonic	C2orf70	nonsynonymous SNV	C2orf70:ENST00000329615.3:exon1:c.C71T:p.P24L,	2p23.3	.
2	27303050	G	A	GACCCGGACA,TGAGCTGTGT	exonic	EMILIN1	nonsynonymous SNV	EMILIN1:ENST00000380320.4:exon2:c.G202A:p.V68M,	2p23.3	.
2	27712508	G	A	GTCCTCAGGT,CTTCAAGTGC	exonic	IFT172	nonsynonymous SNV	IFT172:ENST00000359466.6:exon1:c.C13T:p.H5Y,IFT172:ENST00000260570.3:exon1:c.C13T:p.H5Y,	2p23.3	.
2	28775831	G	A	TCCTGACAAA,ACCCCTCCGA	exonic	PLB1	nonsynonymous SNV	PLB1:ENST00000422425.2:exon17:c.G1153A:p.D385N,PLB1:ENST00000327757.5:exon17:c.G1120A:p.D374N,PLB1:ENST00000329020.6:exon4:c.G184A:p.D62N,PLB1:ENST00000436544.1:exon4:c.G250A:p.D84N,	2p23.2	.
2	28812563	T	G	TGACAACTCT,TCTTCGCTCC	exonic	PLB1	nonsynonymous SNV	PLB1:ENST00000422425.2:exon27:c.T1909G:p.F637V,PLB1:ENST00000327757.5:exon28:c.T1942G:p.F648V,PLB1:ENST00000329020.6:exon15:c.T1006G:p.F336V,PLB1:ENST00000436544.1:exon15:c.T1072G:p.F358V,	2p23.2	.
2	29473997	C	T	CTGGCACCTT,CAGATCTGGA	exonic	ALK	stopgain	ALK:ENST00000389048.3:exon12:c.G2178A:p.W726X,	2p23.2	ALK;basechange=c.2178G>T;AAchange=p.W726C;tissue=endometrium
2	33812341	G	A	CTTCAGTAAA,GCTTTCCCAC	exonic	FAM98A	nonsynonymous SNV	FAM98A:ENST00000403368.1:exon5:c.C569T:p.P190L,FAM98A:ENST00000238823.8:exon5:c.C569T:p.P190L,	2p22.3	.
2	33813514	C	T	ACTACCGCCT,CTTCTTGAGC	exonic	FAM98A	nonsynonymous SNV	FAM98A:ENST00000403368.1:exon4:c.G410A:p.G137E,FAM98A:ENST00000238823.8:exon4:c.G410A:p.G137E,	2p22.3	.
2	37441018	T	A	AATCAGCTCT,CAAATTCTTC	exonic	CEBPZ	nonsynonymous SNV	CEBPZ:ENST00000234170.5:exon10:c.A2534T:p.E845V,	2p22.2	.
2	43793792	G	A	AGTATGTTTT,AAAGAAAACA	exonic	THADA	stopgain	THADA:ENST00000403856.1:exon16:c.C2356T:p.Q786X,	2p21	.

2	155711304	C	T	TCGTTTTTTT,CTGTAATTTC	exonic	KCNJ3	nonsynonymous SNV	KCNJ3:ENST00000295101.2:exon3:c.C985T:p.P329S,	2q24.1	.
2	164466892	C	T	TCATTCCAGT,CACTGGAGGT	exonic	FIGN	nonsynonymous SNV	FIGN:ENST00000333129.3:exon3:c.G1450A:p.D484N,	2q24.3	.
2	169721364	G	A	GATTATACAC,AGAAAAAAGA	exonic	NOSTRIN	nonsynonymous SNV	NOSTRIN:ENST00000444448.2:exon19:c.G1576A:p.E526K,NOSTRIN:ENST00000397209.2:exon15:c.G1321A:p.E441K,NOSTRIN:ENST00000421711.2:exon15:c.G1321A:p.E441K,NOSTRIN:ENST00000445023.2:exon15:c.G1171A:p.E391K,NOSTRIN:ENST00000458381.2:exon21:c.G1576A:p.E526K,NOSTRIN:ENS	2q31.1	.
2	170487449	C	T	CGGAAAAATT,CCGAAAACAC	exonic	PPIG	nonsynonymous SNV	PPIG:ENST00000433207.1:exon7:c.C701T:p.S234F,PPIG:ENST00000448752.2:exon10:c.C713T:p.S238F,PPIG:ENST00000260970.3:exon10:c.C713T:p.S238F,PPIG:ENST00000462903.1:exon10:c.C713T:p.S238F,PPIG:ENST00000414307.1:exon10:c.C713T:p.S238F,PPIG:ENST00000409714.3:exon9:c.C668T:p.S	2q31.1	.
2	176802174	G	A	CTAAACTCAG,AAGTCTTGGA	exonic	KIAA1715	nonsynonymous SNV	KIAA1715:ENST00000535310.1:exon13:c.C727T:p.P243S,KIAA1715:ENST00000272748.4:exon12:c.C952T:p.P318S,KIAA1715:ENST00000544803.1:exon13:c.C1045T:p.P349S,KIAA1715:ENST00000431754.1:exon3:c.C206T:p.S69F,KIAA1715:ENST00000409660.1:exon10:c.C583T:p.P195S,	2q31.1	.
2	178096208	C	T	ACTTCAGAAT,ACTGAGGCCA	exonic	NFE2L2	nonsynonymous SNV	NFE2L2:ENST00000449627.1:exon3:c.G307A:p.D103N,NFE2L2:ENST00000397062.3:exon5:c.G1123A:p.D375N,NFE2L2:ENST00000446151.2:exon5:c.G1054A:p.D352N,NFE2L2:ENST00000397063.4:exon5:c.G1075A:p.D359N,NFE2L2:ENST00000464747.1:exon8:c.G1075A:p.D359N,	2q31.2	.
2	179431523	G	T	GTCACTCTTA,ACGCAAATCT	exonic	TTN	nonsynonymous SNV	TTN:ENST00000342175.6:exon155:c.C52717A:p.L17573I,TTN:ENST00000591111.1:exon276:c.C74413A:p.L24805I,TTN:ENST00000359218.5:exon155:c.C52516A:p.L17506I,TTN:ENST00000589042.1:exon326:c.C79336A:p.L26446I,TTN:ENST00000342992.6:exon275:c.C71632A:p.L23878I,TTN:ENST00000460472	2q31.2	TTN;basechange=c.52141C>A;AAchange=p.L17381I;tissue=large_intestine&TTN_ENST00000356127;basechange=c.71626C>A;AAchange=p.L23876I;tissue=large_intestine&TTN_ENST00000359218;basechange=c.52516C>A;AAchange=p.L17506I;tissue=large_intestine&TTN_ENST00000342175;basechange
2	179456201	A	G	ACCTTCAATT,ATTTCACATC	exonic	TTN	nonsynonymous SNV	TTN:ENST00000342175.6:exon133:c.T33632C:p.L11211S,TTN:ENST00000591111.1:exon254:c.T55328C:p.L18443S,TTN:ENST00000359218.5:exon133:c.T33431C:p.L11144S,TTN:ENST00000589042.1:exon304:c.T60251C:p.L20084S,TTN:ENST00000342992.6:exon253:c.T52547C:p.L17516S,TTN:ENST00000460	2q31.2	.
2	179579944	C	T	ACATCAGCTC,TTTCAGTGTC	exonic	TTN	nonsynonymous SNV	TTN:ENST00000591111.1:exon88:c.G25018A:p.G8340R,TTN:ENST00000589042.1:exon90:c.G25969A:p.G8657R,TTN:ENST00000342992.6:exon87:c.G22237A:p.G7413R,	2q31.2	.
2	179587550	C	T	TTTGGTATCT,CTTTGTACCA	exonic	TTN	nonsynonymous SNV	TTN:ENST00000591111.1:exon74:c.G21125A:p.G7042E,TTN:ENST00000589042.1:exon76:c.G22076A:p.G7359E,TTN:ENST00000342992.6:exon73:c.G18344A:p.G6115E,	2q31.2	.
2	179595397	C	T	TCTTGGTCAT,TTTGAACCAC	exonic	TTN	nonsynonymous SNV	TTN:ENST00000591111.1:exon59:c.G16912A:p.D5638N,TTN:ENST00000589042.1:exon61:c.G17863A:p.D5955N,TTN:ENST00000342992.6:exon58:c.G14131A:p.D4711N,	2q31.2	.
2	179612317	G	A	CACCTCCATT,AAGTGATTGA	exonic	TTN	nonsynonymous SNV	TTN:ENST00000360870.5:exon46:c.C14810T:p.S4937L,	2q31.2	TTN_ENST00000360870;basechange=c.14810C>T;AAchange=p.S4937L;tissue=large_intestine
2	179613674	G	A	TTTTGAGGAG,ATTAACAATT	exonic	TTN	nonsynonymous SNV	TTN:ENST00000360870.5:exon46:c.C13453T:p.P4485S,	2q31.2	.
2	179654713	G	A	ACCTTCTCCT,AGTTATTTGC	exonic	TTN	stopgain	TTN:ENST00000342175.6:exon11:c.C1792T:p.Q598X,TTN:ENST00000591111.1:exon12:c.C1930T:p.Q644X,TTN:ENST00000359218.5:exon11:c.C1792T:p.Q598X,TTN:ENST00000589042.1:exon12:c.C1930T:p.Q644X,TTN:ENST00000360870.5:exon12:c.C1930T:p.Q644X,TTN:ENST00000342992.6:exon12:c.C1930	2q31.2	.
2	179749782	A	G	TTTCTCCATC,TGGTTTTTGC	exonic	CCDC141	nonsynonymous SNV	CCDC141:ENST00000420890.2:exon11:c.T1565C:p.M522T,CCDC141:ENST00000409284.1:exon11:c.T1565C:p.M522T,CCDC141:ENST00000446116.1:exon10:c.T1370C:p.M457T,CCDC141:ENST00000443758.1:exon11:c.T1565C:p.M522T,	2q31.2	.
2	186669016	G	A	AATTGTTCTG,AGTTCACCAC	exonic	FSIP2	nonsynonymous SNV	FSIP2:ENST00000424728.1:exon17:c.G14983A:p.E4995K,FSIP2:ENST00000343098.5:exon17:c.G15250A:p.E5084K,	2q32.1	.
2	190647303	G	A	ACTTCACTGT,GGCAACTCCA	exonic	ORMDL1	nonsynonymous SNV	ORMDL1:ENST00000392350.3:exon2:c.C19T:p.H7Y,ORMDL1:ENST00000325795.3:exon1:c.C19T:p.H7Y,ORMDL1:ENST00000392349.4:exon3:c.C19T:p.H7Y,ORMDL1:ENST00000409519.1:exon2:c.C19T:p.H7Y,ORMDL1:ENST00000442547.1:exon2:c.C19T:p.H7Y,ORMDL1:ENST00000458355.1:exon2:c.C19T:p.H	2q32.2	.
2	196912137	T	G	GATTTGCCCT,TGATTTGGAA	exonic	DNAH7	nonsynonymous SNV	DNAH7:ENST00000410072.1:exon5:c.A337C:p.K113Q,DNAH7:ENST00000427816.1:exon5:c.A262C:p.K88Q,DNAH7:ENST00000312428.6:exon5:c.A337C:p.K113Q,	2q32.3	.
2	197986269	C	T	TTTCATCAAT,TGCAAAAGAG	splicing	ANKRD44(EN	.	.	2q33.1	.
2	201800565	C	T	TCATCATCCT,TGAGTTGGAA	exonic	ORC2	nonsynonymous SNV	ORC2:ENST00000234296.2:exon9:c.G565A:p.E189K,	2q33.1	.
2	202899656	C	T	GCAGTGTTCT,CCGAACTCCG	exonic	FZD7	nonsynonymous SNV	FZD7:ENST00000286201.1:exon1:c.C286T:p.P96S,	2q33.1	.
2	206480385	C	T	AGGACCCTTT,GACAAGACGT	exonic	PARD3B	stopgain	PARD3B:ENST00000358768.2:exon22:c.C3280T:p.R1094X,PARD3B:ENST00000406610.2:exon23:c.C3466T:p.R1156X,PARD3B:ENST00000349953.3:exon22:c.C3163T:p.R1055X,PARD3B:ENST00000351153.1:exon22:c.C3259T:p.R1087X,	2q33.3	.
2	206608010	C	T	CCAGGAATAC,TCTGGAGCCC	exonic	NRP2	nonsynonymous SNV	NRP2:ENST00000357118.4:exon9:c.C1375T:p.L459F,NRP2:ENST00000412873.2:exon9:c.C1375T:p.L459F,NRP2:ENST00000357785.5:exon9:c.C1375T:p.L459F,NRP2:ENST00000272849.3:exon9:c.C1375T:p.L459F,NRP2:ENST00000540841.1:exon9:c.C1375T:p.L459F,NRP2:ENST00000355117.4:exon9:c.C1375T	2q33.3	.
2	210570356	C	T	AGACCTTCCT,CATTCTCCCT	exonic	MAP2	nonsynonymous SNV	MAP2:ENST00000360351.4:exon11:c.C4637T:p.S1546F,MAP2:ENST00000361559.4:exon8:c.C569T:p.S190F,MAP2:ENST00000447185.1:exon8:c.C4625T:p.S1542F,MAP2:ENST00000452717.1:exon7:c.C566T:p.S189F,MAP2:ENST00000392194.1:exon7:c.C569T:p.S190F,MAP2:ENST00000199940.6:exon10:c.C74	2q34	.
2	211504771	C	T	AGGCTGTGGT,CATATCACAT	exonic	CPS1	nonsynonymous SNV	CPS1:ENST00000233072.5:exon24:c.C2947T:p.P983S,CPS1:ENST00000451903.2:exon14:c.C1594T:p.P532S,CPS1:ENST00000430249.2:exon25:c.C2965T:p.P989S,	2q34	.
2	217285042	C	T	CCAGAGCCTC,CCACGGTCAA	exonic	SMARCAL1	nonsynonymous SNV	SMARCAL1:ENST00000357276.4:exon5:c.C883T:p.P295S,SMARCAL1:ENST00000358207.5:exon5:c.C883T:p.P295S,SMARCAL1:ENST00000392128.2:exon3:c.C475T:p.P159S,SMARCAL1:ENST00000427645.1:exon3:c.C580T:p.P194S,SMARCAL1:ENST00000412913.1:exon3:c.C43T:p.P15S,	2q35	.
2	226446839	G	A	CAGCCCCGCG,GAGACCCCGA	exonic	NYAP2	nonsynonymous SNV	NYAP2:ENST00000272907.6:exon4:c.G706A:p.G236R,	2q36.3	.
2	233712097	C	T	GTAGAATCTC,TTATGAGGTC	exonic	GIGYF2	nonsynonymous SNV	GIGYF2:ENST00000409451.3:exon29:c.C3563T:p.P1188L,GIGYF2:ENST00000373563.4:exon27:c.C3500T:p.P1167L,GIGYF2:ENST00000409196.3:exon26:c.C3482T:p.P1161L,GIGYF2:ENST00000409547.1:exon29:c.C3500T:p.P1167L,GIGYF2:ENST00000409480.1:exon29:c.C3566T:p.P1189L,GIGYF2:ENST0000	2q37.1	.
2	236708101	C	T	GATCGATGTT,CTCCCACTGC	exonic	AGAP1	nonsynonymous SNV	AGAP1:ENST00000336665.5:exon8:c.C892T:p.P298S,AGAP1:ENST00000304032.8:exon8:c.C892T:p.P298S,AGAP1:ENST00000428334.2:exon4:c.C409T:p.P137S,AGAP1:ENST00000409457.1:exon8:c.C892T:p.P298S,AGAP1:ENST00000409538.1:exon8:c.C1687T:p.P563S,	2q37.2	.
2	238253426	C	T	TTGGTTGACT,CCTCAGAGGT	exonic	COL6A3	nonsynonymous SNV	COL6A3:ENST00000472056.1:exon33:c.G5414A:p.G1805E,COL6A3:ENST00000295550.4:exon36:c.G7235A:p.G2412E,COL6A3:ENST00000347401.3:exon35:c.G6632A:p.G2211E,COL6A3:ENST00000346358.4:exon35:c.G6635A:p.G2212E,COL6A3:ENST00000353578.4:exon35:c.G6617A:p.G2206E,COL6A3:ENST	2q37.3	.
2	238270417	C	T	CCGCGGTCTC,CCTCTGCCCA	exonic	COL6A3	nonsynonymous SNV	COL6A3:ENST00000472056.1:exon12:c.G4300A:p.G1434R,COL6A3:ENST00000295550.4:exon15:c.G6121A:p.G2041R,COL6A3:ENST00000347401.3:exon14:c.G5518A:p.G1840R,COL6A3:ENST00000346358.4:exon14:c.G5521A:p.G1841R,COL6A3:ENST00000353578.4:exon14:c.G5503A:p.G1835R,COL6A3:ENST	2q37.3	.
2	238285841	C	T	TTGACATCAT,GCTGTACTGA	exonic	COL6A3	nonsynonymous SNV	COL6A3:ENST00000433762.1:exon6:c.G2044A:p.D682N,COL6A3:ENST00000472056.1:exon4:c.G823A:p.D275N,COL6A3:ENST00000295550.4:exon7:c.G2644A:p.D882N,COL6A3:ENST00000392003.2:exon5:c.G1423A:p.D475N,COL6A3:ENST00000347401.3:exon6:c.G2041A:p.D681N,COL6A3:ENST0000034635	2q37.3	.
3	1415653	G	A	ACAGTGGTTG,TTTGAGTCCT	exonic	CNTN6	nonsynonymous SNV	CNTN6:ENST00000446702.2:exon16:c.G1991A:p.G664D,CNTN6:ENST00000539053.1:exon15:c.G1775A:p.G592D,CNTN6:ENST00000350110.2:exon16:c.G1991A:p.G664D,	3p26.3	CNTN6;basechange=c.1991G>T;AAchange=p.G664V;tissue=haematopoietic_and_lymphoid_tissue
3	1444077	C	T	GCTTCTGGTT,CATTTGAAGA	exonic	CNTN6	nonsynonymous SNV	CNTN6:ENST00000446702.2:exon22:c.C2893T:p.P965S,CNTN6:ENST00000539053.1:exon21:c.C2677T:p.P893S,CNTN6:ENST00000350110.2:exon22:c.C2893T:p.P965S,	3p26.3	.
3	3116538	C	T	ACAACTTGAT,CATAAATGAC	exonic	IL5RA	stopgain	IL5RA:ENST00000256452.3:exon12:c.G1104A:p.W368X,IL5RA:ENST00000446632.2:exon11:c.G1104A:p.W368X,IL5RA:ENST00000418488.2:exon7:c.G819A:p.W273X,IL5RA:ENST00000445864.2:exon5:c.G477A:p.W159X,	3p26.2	IL5RA;basechange=c.1104G>A;AAchange=p.W368*;tissue=endometrium
3	3116539	C	T	CAACTTGATC,ATAAATGACA	exonic	IL5RA	stopgain	IL5RA:ENST00000256452.3:exon12:c.G1103A:p.W368X,IL5RA:ENST00000446632.2:exon11:c.G1103A:p.W368X,IL5RA:ENST00000418488.2:exon7:c.G818A:p.W273X,IL5RA:ENST00000445864.2:exon5:c.G476A:p.W159X,	3p26.2	.
3	10885969	C	T	GGCCTTGTGT,TCTTGGCAGC	exonic	SLC6A11	nonsynonymous SNV	SLC6A11:ENST00000254488.2:exon5:c.C694T:p.L232F,	3p25.3	.
3	19975426	C	T	ATCCCCAACT,CATGGGAAAT	exonic	EFHB	nonsynonymous SNV	EFHB:ENST00000389256.4:exon1:c.G85A:p.E29K,EFHB:ENST00000295824.9:exon1:c.G85A:p.E29K,	3p24.3	.
3	27216212	C	T	TTCGTCTTTA,CATAGGAGGC	exonic	NEK10	nonsynonymous SNV	NEK10:ENST00000295720.6:exon6:c.G554A:p.G185D,NEK10:ENST00000429845.2:exon28:c.G2618A:p.G873D,NEK10:ENST00000383770.3:exon6:c.G554A:p.G185D,NEK10:ENST00000357467.2:exon9:c.G809A:p.G270D,NEK10:ENST00000383771.4:exon6:c.G554A:p.G185D,	3p24.1	.
3	39544180	C	T	ACGGTCCCCT,CGAGGTCTGA	exonic	MOBP	nonsynonymous SNV	MOBP:ENST00000441980.2:exon4:c.C361T:p.P121S,MOBP:ENST00000311042.6:exon4:c.C433T:p.P145S,MOBP:ENST00000442631.1:exon2:c.C361T:p.P121S,MOBP:ENST00000420739.1:exon4:c.C361T:p.P121S,MOBP:ENST00000424090.1:exon2:c.C361T:p.P121S,MOBP:ENST00000354668.4:exon4:c.C361T:p	3p22.1	.
3	42299705	G	A	CTTAACGATG,ACATTCGTCC	exonic	CCK	nonsynonymous SNV	CCK:ENST00000396169.2:exon5:c.C233T:p.S78F,CCK:ENST00000334681.5:exon3:c.C233T:p.S78F,CCK:ENST00000434608.1:exon3:c.C233T:p.S78F,	3p22.1	.
3	43122864	C	T	GCACATGCTT,CACAGGACCG	exonic	POMGNT2	stopgain	POMGNT2:ENST00000441964.1:exon3:c.G60A:p.W20X,POMGNT2:ENST00000344697.2:exon2:c.G60A:p.W20X,	3p22.1	.
3	45067895	C	T	TGCCTCTTCT,CCTCCTGACC	exonic	CLEC3B	nonsynonymous SNV	CLEC3B:ENST00000296130.4:exon1:c.C41T:p.S14F,	3p21.31	.
3	48309617	C	T	AGAAGATCAA,GAGTGGAAGG	exonic	ZNF589	stopgain	ZNF589:ENST00000440261.2:exon4:c.C433T:p.R145X,ZNF589:ENST00000448461.1:exon4:c.C436T:p.R146X,ZNF589:ENST00000354698.3:exon4:c.C436T:p.R146X,	3p21.31	.
3	48622542	G	A	ATCTGCTCCA,GGAAGCCCTG	exonic	COL7A1	nonsynonymous SNV	COL7A1:ENST00000328333.8:exon32:c.C3902T:p.P1301L,COL7A1:ENST00000454817.1:exon32:c.C3902T:p.P1301L,	3p21.31	.
3	49679770	C	T	CACTGCACCT,GCTCCAAGAG	exonic	BSN	nonsynonymous SNV	BSN:ENST00000296452.4:exon3:c.C703T:p.R235C,	3p21.31	.
3	52257352	G	A	GAAGGCCTTG,TTTTAGTGAT	exonic	TLR9	nonsynonymous SNV	TLR9:ENST00000360658.2:exon2:c.C980T:p.T327I,TLR9:ENST00000597542.1:exon9:c.C1052T:p.T351I,	3p21.2	.
3	52429402	C	T	CGGCACAGAC,CTGCTGCCGA	exonic	DNAH1	nonsynonymous SNV	DNAH1:ENST00000490713.1:exon12:c.C1747T:p.P583S,DNAH1:ENST00000420323.2:exon69:c.C11047T:p.P3683S,	3p21.1	.
3	52858582	C	T	CTTCCCGGGT,TGGTCAGGGA	splicing	ITIH4(ENST0	.	.	3p21.1	.
3	53857989	C	T	CAGGGCTCCC,GTGCCAGGGC	exonic	CHDH	nonsynonymous SNV	CHDH:ENST00000467802.1:exon3:c.G47A:p.R16Q,CHDH:ENST00000481668.1:exon3:c.G47A:p.R16Q,CHDH:ENST00000315251.6:exon3:c.G47A:p.R16Q,	3p21.1	.
3	57389175	C	T	CAAAGCATTT,CACCAGATTG	exonic	DNAH12	nonsynonymous SNV	DNAH12:ENST00000351747.2:exon43:c.G6752A:p.G2251E,DNAH12:ENST00000495027.1:exon43:c.G6809A:p.G2270E,	3p14.3	.
3	57843866	C	T	TATGGGAAAC,AATTACAGGC	exonic	SLMAP	stopgain	SLMAP:ENST00000383718.3:exon7:c.C667T:p.Q223X,SLMAP:ENST00000428312.1:exon7:c.C667T:p.Q223X,SLMAP:ENST00000295951.3:exon8:c.C667T:p.Q223X,SLMAP:ENST00000449503.2:exon7:c.C667T:p.Q223X,SLMAP:ENST00000295952.3:exon7:c.C667T:p.Q223X,	3p14.3	.
3	58519783	C	T	GAGTGGGTCC,ATTTGGCAAT	exonic	ACOX2	stopgain	ACOX2:ENST00000302819.5:exon4:c.G413A:p.W138X,ACOX2:ENST00000459701.2:exon4:c.G413A:p.W138X,ACOX2:ENST00000474098.1:exon4:c.G413A:p.W138X,	3p14.3	.
3	63264374	C	T	GGGCACCTTC,GGGTGCTGAA	exonic	SYNPR	nonsynonymous SNV	SYNPR:ENST00000450542.2:exon2:c.C40T:p.R14W,SYNPR:ENST00000468110.1:exon2:c.C40T:p.R14W,SYNPR:ENST00000478300.1:exon2:c.C40T:p.R14W,	3p14.2	SYNPR;basechange=c.40C>T;AAchange=p.R14W;tissue=large_intestine
3	64636796	C	T	CTCTTAACTC,TTCTTCTTTA	exonic	ADAMTS9	nonsynonymous SNV	ADAMTS9:ENST00000498707.1:exon9:c.G1360A:p.G454R,ADAMTS9:ENST00000459780.1:exon9:c.G1360A:p.G454R,ADAMTS9:ENST00000295903.4:exon8:c.G1276A:p.G426R,	3p14.1	.
3	65342691	G	A	GATGATTTGT,AAGATCGGGT	exonic	MAGI1	nonsynonymous SNV	MAGI1:ENST00000402939.2:exon23:c.C3751T:p.H1251Y,	3p14.1	.
3	65342724	G	A	TCCAATGACG,ATGCTGCCGG	exonic	MAGI1	nonsynonymous SNV	MAGI1:ENST00000402939.2:exon23:c.C3718T:p.P1240S,	3p14.1	.
3	69111021	G	A	TCTCTCTAGG,ATTTTTGGAA	exonic	UBA3	nonsynonymous SNV	UBA3:ENST00000349511.4:exon10:c.C812T:p.S271F,UBA3:ENST00000540295.1:exon6:c.C323T:p.S108F,UBA3:ENST00000361055.4:exon11:c.C854T:p.S285F,UBA3:ENST00000415609.2:exon9:c.C731T:p.S244F,	3p14.1	.
3	74315640	C	T	ACTGGTTATT,GTGGAATCCT	exonic	CNTN3	nonsynonymous SNV	CNTN3:ENST00000263665.6:exon21:c.G2978A:p.R993Q,	3p12.3	.
3	74334632	C	T	ACCCCCATTC,AGTACCGCAC	exonic	CNTN3	stopgain	CNTN3:ENST00000263665.6:exon19:c.G2528A:p.W843X,	3p12.3	.
3	74420454	G	A	CTCCACCTTA,ATATGTAGAG	exonic	CNTN3	nonsynonymous SNV	CNTN3:ENST00000263665.6:exon5:c.C551T:p.S184F,	3p12.3	.
3	78710285	G	A	CTGAGATCAG,GATTACCACA	exonic	ROBO1	nonsynonymous SNV	ROBO1:ENST00000467549.1:exon15:c.C2107T:p.P703S,ROBO1:ENST00000436010.2:exon14:c.C2098T:p.P700S,ROBO1:ENST00000464233.1:exon16:c.C2215T:p.P739S,ROBO1:ENST00000495273.1:exon15:c.C2107T:p.P703S,	3p12.3	ROBO1_ENST00000495273;basechange=c.2107C>T;AAchange=p.P703S;tissue=autonomic_ganglia&ROBO1_ENST00000464233;basechange=c.2215C>T;AAchange=p.P739S;tissue=autonomic_ganglia&ROBO1_ENST00000305299;basechange=c.2215C>T;AAchange=p.P739S;tissue=autonomic_ganglia&R
3	89391081	C	T	TGTCCGCTTC,TCCCTCGACA	exonic	EPHA3	nonsynonymous SNV	EPHA3:ENST00000494014.1:exon5:c.C1147T:p.L383F,EPHA3:ENST00000336596.2:exon5:c.C1147T:p.L383F,EPHA3:ENST00000452448.2:exon5:c.C1147T:p.L383F,	3p11.1	.
3	89528576	T	A	GTCACCGTGG,TGGGCCACAG	exonic	EPHA3	nonsynonymous SNV	EPHA3:ENST00000336596.2:exon17:c.T2876A:p.V959D,	3p11.1	.
3	97806407	C	T	CATATGTAAT,CCTTGCTTTA	exonic	OR5AC2	nonsynonymous SNV	OR5AC2:ENST00000358642.2:exon1:c.C391T:p.P131S,	3q11.2	OR5AC2;basechange=c.391C>T;AAchange=p.P131S;tissue=lung
3	98109967	A	G	CTAGCTGGCA,CCTGCATCCC	exonic	OR5K3	nonsynonymous SNV	OR5K3:ENST00000383695.1:exon1:c.A458G:p.N153S,	3q11.2	.
3	98216685	G	C	ACACACTGTC,GCTTCACACA	exonic	OR5K2	nonsynonymous SNV	OR5K2:ENST00000427338.1:exon1:c.G161C:p.R54P,	3q11.2	OR5K2;basechange=c.161G>A;AAchange=p.R54Q;tissue=lung
3	100535573	G	A	TGTAGTTTTG,GTCTGGGACG	exonic	ABI3BP	nonsynonymous SNV	ABI3BP:ENST00000495063.1:exon39:c.C2948T:p.P983L,ABI3BP:ENST00000383691.4:exon22:c.C1040T:p.P347L,ABI3BP:ENST00000471714.1:exon43:c.C3209T:p.P1070L,	3q12.2	.
3	110830926	G	A	CAGCAGTATG,GGAAAGAATG	exonic	PVRL3	stopgain	PVRL3:ENST00000481766.1:exon3:c.G165A:p.W55X,PVRL3:ENST00000319792.3:exon2:c.G210A:p.W70X,PVRL3:ENST00000461477.1:exon2:c.G69A:p.W23X,PVRL3:ENST00000493615.1:exon2:c.G141A:p.W47X,PVRL3:ENST00000485303.1:exon2:c.G210A:p.W70X,	3q13.13	.
3	126158571	C	T	AGGGGTCATC,TGGGAAGGCA	splicing	ZXDC(ENSTC	.	.	3q21.3	.
3	126181039	G	A	AAGAGAACTC,GAGCTTCTAG	exonic	ZXDC	nonsynonymous SNV	ZXDC:ENST00000389709.3:exon6:c.C1466T:p.P489L,ZXDC:ENST00000336332.5:exon6:c.C1466T:p.P489L,	3q21.3	.
3	126272190	C	T	CTGGATCCTC,ACTTCTTACT	exonic	C3orf22	stopgain	C3orf22:ENST00000505070.1:exon2:c.G44A:p.W15X,C3orf22:ENST00000318225.2:exon2:c.G44A:p.W15X,	3q21.3	.
3	126916024	C	T	CAGCTCAGCC,CCAGGGCTGC	exonic	C3orf56	nonsynonymous SNV	C3orf56:ENST00000398112.1:exon2:c.C496T:p.P166S,	3q21.3	.
3	128664631	C	T	CTTGCGGTCC,GGTCGTGAAC	exonic	RP11-723O4.	nonsynonymous SNV	RP11-723O4.6:ENST00000508239.1:exon3:c.G179A:p.R60Q,RP11-723O4.6:ENST00000511204.1:exon2:c.G179A:p.R60Q,	3q21.3	.
3	130187846	G	A	TCCACCTTAG,AGACAGGGTT	exonic	COL6A5	nonsynonymous SNV	COL6A5:ENST00000512482.1:exon3:c.G503A:p.G168E,COL6A5:ENST00000265379.6:exon38:c.G6998A:p.G2333E,COL6A5:ENST00000373157.4:exon4:c.G827A:p.G276E,COL6A5:ENST00000312481.7:exon38:c.G6998A:p.G2333E,COL6A5:ENST00000432398.2:exon38:c.G6998A:p.G2333E,	3q22.1	.
3	133099681	C	T	CCAGGGGATT,CTCAGGGAGC	exonic	TMEM108	nonsynonymous SNV	TMEM108:ENST00000515826.1:exon3:c.C1126T:p.P376S,TMEM108:ENST00000321871.6:exon4:c.C1126T:p.P376S,TMEM108:ENST00000393130.3:exon4:c.C1126T:p.P376S,	3q22.1	.
3	133099682	C	T	CAGGGGATTC,TCAGGGAGCA	exonic	TMEM108	nonsynonymous SNV	TMEM108:ENST00000515826.1:exon3:c.C1127T:p.P376L,TMEM108:ENST00000321871.6:exon4:c.C1127T:p.P376L,TMEM108:ENST00000393130.3:exon4:c.C1127T:p.P376L,	3q22.1	.
3	133670079	G	T	TTGCTCGAGG,AAGAAAAAAA	exonic	SLCO2A1	nonsynonymous SNV	SLCO2A1:ENST00000310926.4:exon6:c.C834A:p.F278L,SLCO2A1:ENST00000481359.3:exon6:c.C834A:p.F278L,SLCO2A1:ENST00000493729.1:exon5:c.C606A:p.F202L,	3q22.1	.
3	138724404	G	A	GCGCGGAGAG,GAGGTAGAGG	exonic	PRR23A	nonsynonymous SNV	PRR23A:ENST00000383163.2:exon1:c.C707T:p.P236L,	3q23	.
3	142408604	C	T	TAACACATAC,CGTGCCTGCA	exonic	PLS1	nonsynonymous SNV	PLS1:ENST00000337777.3:exon10:c.C1126T:p.P376S,PLS1:ENST00000497002.1:exon10:c.C1126T:p.P376S,PLS1:ENST00000457734.2:exon10:c.C1126T:p.P376S,	3q23	.
3	148614438	G	A	AACGTGCAGA,AGACCATGCT	exonic	CPA3	nonsynonymous SNV	CPA3:ENST00000296046.3:exon11:c.G1198A:p.E400K,	3q24	.
3	151162713	G	A	ATACCTGATG,GACTCTGGTC	exonic	IGSF10	nonsynonymous SNV	IGSF10:ENST00000282466.3:exon4:c.C5056T:p.P1686S,	3q25.1	.
3	157160454	G	A	GAATTCAGAG,AAGGGCTCAC	exonic	PTX3	nonsynonymous SNV	PTX3:ENST00000295927.3:exon3:c.G832A:p.E278K,	3q25.32	.
3	164906488	C	T	CCTCCACCAT,CTCAAACAGC	exonic	SLITRK3	nonsynonymous SNV	SLITRK3:ENST00000241274.3:exon2:c.G2131A:p.D711N,SLITRK3:ENST00000475390.1:exon2:c.G2131A:p.D711N,	3q26.1	.
3	167196724	C	T	CTTGGCTTTT,GTTCATGATG	exonic	WDR49	nonsynonymous SNV	WDR49:ENST00000476376.1:exon13:c.G1511A:p.R504Q,WDR49:ENST00000308378.3:exon15:c.G2036A:p.R679Q,WDR49:ENST00000453925.2:exon14:c.G1931A:p.R644Q,	3q26.1	.
3	170843778	T	C	AGAGGAGGAT,TTCCGAGGTG	exonic	TNIK	nonsynonymous SNV	TNIK:ENST00000460047.1:exon16:c.A1771G:p.N591D,TNIK:ENST00000357327.5:exon16:c.A1849G:p.N617D,TNIK:ENST00000284483.8:exon17:c.A1936G:p.N646D,TNIK:ENST00000470834.1:exon16:c.A1849G:p.N617D,TNIK:ENST00000341852.6:exon15:c.A1684G:p.N562D,TNIK:ENST00000488470.1:exon16:c.A	3q26.2	.
3	179533713	C	T	TGGCAGATCC,CTTCCTTCAG	exonic	PEX5L	nonsynonymous SNV	PEX5L:ENST00000468741.1:exon9:c.G443A:p.G148E,PEX5L:ENST00000476138.1:exon10:c.G890A:p.G297E,PEX5L:ENST00000467460.1:exon10:c.G1019A:p.G340E,PEX5L:ENST00000485199.1:exon9:c.G914A:p.G305E,PEX5L:ENST00000465751.1:exon9:c.G947A:p.G316E,PEX5L:ENST00000472994.1:exon8:c	3q26.33	.
3	183951401	G	A	CCTTCAGGAG,AAGGGCTGGC	exonic	VWA5B2	nonsynonymous SNV	VWA5B2:ENST00000426955.2:exon4:c.G568A:p.E190K,	3q27.1	.
3	185146485	C	T	AACCACCCTT,TCCCAAGCTG	exonic	MAP3K13	nonsynonymous SNV	MAP3K13:ENST00000438053.1:exon2:c.C116T:p.S39F,MAP3K13:ENST00000447637.1:exon3:c.C116T:p.S39F,MAP3K13:ENST00000439882.1:exon1:c.C116T:p.S39F,MAP3K13:ENST00000428617.1:exon2:c.C116T:p.S39F,MAP3K13:ENST00000424227.1:exon3:c.C116T:p.S39F,MAP3K13:ENST00000265026.3:ex	3q27.2	.
3	186760774	G	A	CTCCTTCCAG,TGTGGAACAA	exonic	ST6GAL1	nonsynonymous SNV	ST6GAL1:ENST00000417392.1:exon4:c.G283A:p.V95M,ST6GAL1:ENST00000448044.1:exon3:c.G283A:p.V95M,ST6GAL1:ENST00000438590.1:exon3:c.G283A:p.V95M,ST6GAL1:ENST00000169298.3:exon4:c.G283A:p.V95M,ST6GAL1:ENST00000448408.1:exon4:c.G283A:p.V95M,ST6GAL1:ENST00000423451.1:	3q27.3	.
3	187088599	G	A	TCCTGCCAGC,AAGTTGGGCT	exonic	RTP4	nonsynonymous SNV	RTP4:ENST00000259030.2:exon2:c.G179A:p.R60Q,	3q27.3	.
3	188327594	C	T	TATCACAGAT,CTGTTTCAGC	exonic	LPP	nonsynonymous SNV	LPP:ENST00000415906.1:exon1:c.C586T:p.P196S,LPP:ENST00000543006.1:exon6:c.C1075T:p.P359S,LPP:ENST00000312675.4:exon6:c.C1075T:p.P359S,LPP:ENST00000448637.1:exon6:c.C1075T:p.P359S,	3q28	.
3	189706707	G	A	TCGATAGTAG,CAAACTGTAG	exonic	LEPREL1	nonsynonymous SNV	LEPREL1:ENST00000426003.1:exon4:c.C401T:p.A134V,LEPREL1:ENST00000319332.5:exon4:c.C944T:p.A315V,LEPREL1:ENST00000444866.1:exon4:c.C401T:p.A134V,LEPREL1:ENST00000427335.2:exon4:c.C401T:p.A134V,	3q28	.
3	193125113	G	A	TCGGCCACAA,GGACACAATG	exonic	ATP13A4	nonsynonymous SNV	ATP13A4:ENST00000342695.4:exon29:c.C3367T:p.L1123F,ATP13A4:ENST00000392443.3:exon29:c.C3310T:p.L1104F,ATP13A4:ENST00000400270.2:exon6:c.C415T:p.L139F,	3q29	.
3	194063338	C	T	CAGAACACCT,CTGGACGAAG	exonic	CPN2	nonsynonymous SNV	CPN2:ENST00000429275.1:exon2:c.G94A:p.E32K,CPN2:ENST00000323830.3:exon2:c.G94A:p.E32K,	3q29	.
4	2453080	G	A	AGCTGCAGAG,TGAAGGGCGG	splicing	RP11-503N1	.	.	4p16.3	.
4	3263129	C	T	GCGCGCCTTC,AGTTGCTCGG	exonic	MSANTD1	nonsynonymous SNV	MSANTD1:ENST00000510580.1:exon4:c.C836T:p.P279L,	4p16.3	.
4	4199770	G	A	GATCCCGTGG,AGATGGCAGT	exonic	OTOP1	nonsynonymous SNV	OTOP1:ENST00000296358.4:exon5:c.C791T:p.S264F,	4p16.3	.
4	4322463	C	T	AAGTGCTTTA,CCGCTCTGCG	exonic	ZBTB49	nonsynonymous SNV	ZBTB49:ENST00000337872.4:exon8:c.C1718T:p.T573I,ZBTB49:ENST00000355834.3:exon4:c.C1352T:p.T451I,ZBTB49:ENST00000538529.1:exon6:c.C167T:p.T56I,	4p16.3	.
4	5733362	C	T	GAACGCCTTC,CTGAAGTGCT	exonic	EVC	nonsynonymous SNV	EVC:ENST00000509451.1:exon4:c.C595T:p.P199S,EVC:ENST00000264956.6:exon4:c.C595T:p.P199S,EVC:ENST00000382674.2:exon4:c.C595T:p.P199S,	4p16.2	.
4	5827371	C	T	CTGGAAACCC,TTGCAATCCA	exonic	CRMP1	nonsynonymous SNV	CRMP1:ENST00000324989.7:exon13:c.G1819A:p.G607R,CRMP1:ENST00000397890.2:exon13:c.G1477A:p.G493R,CRMP1:ENST00000512574.1:exon13:c.G1471A:p.G491R,	4p16.2	.
4	6864639	G	C	AGTAGAAATA,AAAGAACTGA	exonic	KIAA0232	nonsynonymous SNV	KIAA0232:ENST00000307659.5:exon7:c.G2530C:p.E844Q,KIAA0232:ENST00000425103.1:exon6:c.G2530C:p.E844Q,	4p16.1	.
4	8621039	A	G	CCACATTGTC,TTGCCCAAGC	exonic	CPZ	nonsynonymous SNV	CPZ:ENST00000315782.6:exon10:c.A1621G:p.I541V,CPZ:ENST00000382480.2:exon11:c.A1243G:p.I415V,CPZ:ENST00000429646.2:exon9:c.A478G:p.I160V,CPZ:ENST00000360986.4:exon11:c.A1654G:p.I552V,	4p16.1	.
4	13485719	C	T	GGCGCCGTCC,CCAGCACGAC	exonic	RAB28	nonsynonymous SNV	RAB28:ENST00000330852.5:exon1:c.G56A:p.G19E,RAB28:ENST00000508274.1:exon1:c.G56A:p.G19E,RAB28:ENST00000288723.4:exon1:c.G56A:p.G19E,RAB28:ENST00000338176.4:exon1:c.G56A:p.G19E,	4p15.33	.
4	25157679	A	G	CATGGATTTA,AGCAGGACTT	exonic	SEPSECS	nonsynonymous SNV	SEPSECS:ENST00000382103.2:exon4:c.T527C:p.F176S,SEPSECS:ENST00000302922.3:exon3:c.T290C:p.F97S,	4p15.2	.
4	26483616	C	T	ATGCGGATCA,CCTTTTCTTG	exonic	CCKAR	nonsynonymous SNV	CCKAR:ENST00000295589.3:exon5:c.G931A:p.V311M,	4p15.2	.
4	42583712	C	T	AACTGAGCTC,TCGAAGAAGA	exonic	ATP8A1	nonsynonymous SNV	ATP8A1:ENST00000264449.10:exon10:c.G760A:p.G254R,ATP8A1:ENST00000381668.5:exon10:c.G760A:p.G254R,	4p13	.
4	46060338	C	T	AGTGAAATAT,CCATTCTTCT	exonic	GABRG1	nonsynonymous SNV	GABRG1:ENST00000295452.4:exon7:c.G812A:p.G271E,	4p12	.
4	46930666	G	A	AACAACTGTG,AAGATTTGCT	exonic	GABRA4	nonsynonymous SNV	GABRA4:ENST00000264318.3:exon9:c.C1241T:p.S414F,	4p12	.
4	47163376	G	A	ACCAACTCTG,GTACCAGACA	exonic	GABRB1	stopgain	GABRB1:ENST00000538619.1:exon3:c.G141A:p.W47X,GABRB1:ENST00000295454.3:exon4:c.G351A:p.W117X,GABRB1:ENST00000513567.1:exon4:c.G252A:p.W84X,	4p12	.
4	47655678	C	T	GGTGAGCATT,TATTAAAAAC	splicing	CORIN(ENST	.	.	4p12	.
4	47685790	C	T	TCATCATATC,ATCACACACA	exonic	CORIN	nonsynonymous SNV	CORIN:ENST00000504584.1:exon7:c.G979A:p.G327R,CORIN:ENST00000273857.4:exon7:c.G979A:p.G327R,CORIN:ENST00000508498.1:exon6:c.G562A:p.G188R,CORIN:ENST00000502252.1:exon6:c.G778A:p.G260R,CORIN:ENST00000505909.1:exon7:c.G979A:p.G327R,	4p12	.
4	57180564	G	A	GAAGCGCCAG,TTGGGAGGAC	exonic	KIAA1211	nonsynonymous SNV	KIAA1211:ENST00000264229.6:exon8:c.G896A:p.G299D,KIAA1211:ENST00000541073.1:exon7:c.G875A:p.G292D,KIAA1211:ENST00000504228.1:exon6:c.G896A:p.G299D,	4q12	.
4	69519944	T	C	GGTTCCACCA,GAGTTATAAA	exonic	UGT2B15	nonsynonymous SNV	UGT2B15:ENST00000338206.5:exon5:c.A1124G:p.H375R,	4q13.2	.
4	71201459	G	C	AACTGCCCTT,AAGAAGAGAA	exonic	CABS1	nonsynonymous SNV	CABS1:ENST00000273936.5:exon1:c.G703C:p.E235Q,	4q13.3	.
4	71510523	G	A	CTTCAAAGAG,GACCAATGTA	exonic	ENAM	nonsynonymous SNV	ENAM:ENST00000396073.3:exon9:c.G3380A:p.G1127E,	4q13.3	.
4	72313412	G	A	GTCCTACCAC,AGATTGGCAG	exonic	SLC4A4	nonsynonymous SNV	SLC4A4:ENST00000425175.1:exon9:c.G1015A:p.E339K,SLC4A4:ENST00000264485.5:exon9:c.G1015A:p.E339K,SLC4A4:ENST00000351898.6:exon9:c.G1015A:p.E339K,SLC4A4:ENST00000340595.3:exon6:c.G883A:p.E295K,SLC4A4:ENST00000512686.1:exon6:c.G883A:p.E295K,	4q13.3	.
4	72423516	C	T	GCGCAGAGTC,ACCTGTTCAC	exonic	SLC4A4	nonsynonymous SNV	SLC4A4:ENST00000425175.1:exon22:c.C2851T:p.H951Y,SLC4A4:ENST00000264485.5:exon22:c.C2851T:p.H951Y,SLC4A4:ENST00000351898.6:exon20:c.C2599T:p.H867Y,SLC4A4:ENST00000340595.3:exon19:c.C2719T:p.H907Y,	4q13.3	.
4	74010543	C	T	CCTTCAGATT,ATGTTCCTGT	exonic	ANKRD17	nonsynonymous SNV	ANKRD17:ENST00000509867.2:exon11:c.G1537A:p.E513K,ANKRD17:ENST00000330838.6:exon11:c.G1876A:p.E626K,ANKRD17:ENST00000358602.4:exon11:c.G1876A:p.E626K,	4q13.3	.
4	76489547	C	T	GGTGTAACCA,CGTTTTCCAT	exonic	C4orf26	nonsynonymous SNV	C4orf26:ENST00000435974.2:exon3:c.C335T:p.T112I,	4q21.1	.
4	76797819	G	A	CCTCATGGTG,AAACTATCTA	exonic	PPEF2	nonsynonymous SNV	PPEF2:ENST00000286719.7:exon11:c.C941T:p.S314F,	4q21.1	.
4	111397877	G	A	AGTCCACTAC,ACCTGCACGT	exonic	ENPEP	nonsynonymous SNV	ENPEP:ENST00000265162.5:exon1:c.G307A:p.D103N,	4q25	.
4	114280158	G	A	CCCCACAAAA,AAAGTAAGGA	exonic	ANK2	nonsynonymous SNV	ANK2:ENST00000505342.1:exon1:c.G1414A:p.E472K,ANK2:ENST00000357077.4:exon38:c.G10384A:p.E3462K,ANK2:ENST00000264366.6:exon37:c.G10285A:p.E3429K,	4q26	.
4	122589645	A	C	TCCACAGAGC,GCAGAAGAGC	exonic	ANXA5	nonsynonymous SNV	ANXA5:ENST00000515017.1:exon10:c.T641G:p.L214R,ANXA5:ENST00000501272.2:exon11:c.T761G:p.L254R,ANXA5:ENST00000296511.5:exon13:c.T941G:p.L314R,	4q27	.
4	126367600	C	T	CTTTCCAGTT,CACCAGTGTG	exonic	FAT4	nonsynonymous SNV	FAT4:ENST00000394329.3:exon8:c.C7346T:p.S2449F,FAT4:ENST00000335110.5:exon8:c.C2240T:p.S747F,	4q28.1	.
4	128694638	C	T	GGCGCCTTCT,CAATGTTCTT	exonic	SLC25A31	nonsynonymous SNV	SLC25A31:ENST00000281154.4:exon6:c.C857T:p.S286F,	4q28.1	.
4	134072773	G	A	CGGGATGAGG,CGCCAACGCC	exonic	PCDH10	nonsynonymous SNV	PCDH10:ENST00000264360.5:exon1:c.G1478A:p.G493D,	4q28.3	.
4	144545282	C	T	TCTACAATTT,AACCGTTGCC	exonic	FREM3	nonsynonymous SNV	FREM3:ENST00000329798.5:exon4:c.G5632A:p.E1878K,	4q31.21	.
4	170641151	C	T	TCAACTGAAT,TCACAGATGA	exonic	CLCN3	nonsynonymous SNV	CLCN3:ENST00000347613.4:exon14:c.C2536T:p.L846F,	4q33	.
4	170912940	A	T	CCTCTGGAGT,TGCCTCACAA	exonic	MFAP3L	nonsynonymous SNV	MFAP3L:ENST00000361618.3:exon3:c.T819A:p.H273Q,MFAP3L:ENST00000393704.3:exon2:c.T510A:p.H170Q,	4q33	.
4	187026633	G	A	GGGCCCCGGC,GGGTCTGGGC	exonic	FAM149A	nonsynonymous SNV	FAM149A:ENST00000356371.5:exon1:c.G403A:p.G135R,	4q35.1	.
4	189060927	G	A	GAGCGTCTGG,GAGGCTGGCC	exonic	TRIML1	nonsynonymous SNV	TRIML1:ENST00000332517.3:exon1:c.G215A:p.G72E,	4q35.2	TRIML1;basechange=c.215G>A;AAchange=p.G72E;tissue=NS
5	163615	C	T	CTGTCCTCCC,CTCGGGGCTC	exonic	PLEKHG4B	nonsynonymous SNV	PLEKHG4B:ENST00000283426.6:exon11:c.C2360T:p.P787L,	5p15.33	.
5	1057727	G	A	TGCCGCCGCG,TGTGGGACGC	exonic	SLC12A7	nonsynonymous SNV	SLC12A7:ENST00000264930.5:exon22:c.C2885T:p.T962I,	5p15.33	.
5	1878436	G	A	ACAGCCGCAG,GGCCGCGGGA	exonic	IRX4	nonsynonymous SNV	IRX4:ENST00000505790.1:exon6:c.C1207T:p.P403S,IRX4:ENST00000231357.2:exon5:c.C1207T:p.P403S,IRX4:ENST00000513692.1:exon6:c.C1207T:p.P403S,	5p15.33	.
5	3600126	C	T	CTGCAACACC,CGCCTTCCTG	exonic	IRX1	nonsynonymous SNV	IRX1:ENST00000302006.3:exon2:c.C1064T:p.P355L,	5p15.33	.
5	5222935	G	A	CCATCGTATT,GAAGGAAATG	exonic	ADAMTS16	nonsynonymous SNV	ADAMTS16:ENST00000274181.7:exon11:c.G1639A:p.G547R,ADAMTS16:ENST00000511368.1:exon11:c.G1639A:p.G547R,	5p15.32	.
5	10680974	C	A	CAACCAGACT,CCCATAAACA	exonic	DAP	stopgain	DAP:ENST00000432074.2:exon3:c.G460T:p.E154X,	5p15.2	.
5	13817798	C	T	TGTGGTTTTC,ACAATCTATA	exonic	DNAH5	nonsynonymous SNV	DNAH5:ENST00000265104.4:exon42:c.G6847A:p.G2283R,	5p15.2	.
5	14488233	C	T	CGACCCGGCT,CTTCACCTTC	exonic	TRIO	nonsynonymous SNV	TRIO:ENST00000513206.1:exon44:c.C6695T:p.S2232F,TRIO:ENST00000344204.4:exon48:c.C7496T:p.S2499F,	5p15.2	.
5	21842356	C	T	AAGCTGTCCT,CCATATCCTT	exonic	CDH12	nonsynonymous SNV	CDH12:ENST00000522262.1:exon5:c.G608A:p.G203E,CDH12:ENST00000504376.2:exon7:c.G728A:p.G243E,CDH12:ENST00000382254.1:exon8:c.G728A:p.G243E,	5p14.3	.
5	23521238	C	T	AAACCAGTGT,CCCTTCTGGA	exonic	PRDM9	nonsynonymous SNV	PRDM9:ENST00000296682.3:exon6:c.C458T:p.S153F,	5p14.2	.
5	37329319	G	A	CCCAAGATGG,ACCAACATTA	exonic	NUP155	nonsynonymous SNV	NUP155:ENST00000513532.1:exon16:c.C1786T:p.P596S,NUP155:ENST00000231498.3:exon16:c.C1786T:p.P596S,NUP155:ENST00000381843.2:exon16:c.C1609T:p.P537S,	5p13.2	.
5	38407110	G	A	GAATGGTGTG,CCATAATGTC	exonic	EGFLAM	nonsynonymous SNV	EGFLAM:ENST00000336740.6:exon3:c.G307A:p.A103T,EGFLAM:ENST00000354891.3:exon8:c.G1009A:p.A337T,EGFLAM:ENST00000322350.5:exon8:c.G1009A:p.A337T,	5p13.1	.
5	40681351	C	T	GGGCCAATGG,CCGGGGGCCA	exonic	PTGER4	nonsynonymous SNV	PTGER4:ENST00000302472.3:exon2:c.C256T:p.P86S,	5p13.1	.
5	41159199	G	A	TTCCATGATT,AAAATGTGCA	exonic	C6	nonsynonymous SNV	C6:ENST00000263413.3:exon12:c.C1841T:p.S614L,C6:ENST00000337836.5:exon12:c.C1841T:p.S614L,	5p13.1	.
5	41160321	C	T	ACATTCAGTC,CTGAGAGGGT	exonic	C6	nonsynonymous SNV	C6:ENST00000263413.3:exon11:c.G1607A:p.G536E,C6:ENST00000337836.5:exon11:c.G1607A:p.G536E,	5p13.1	.
5	41186287	G	A	TTCTCCTCTG,GCTCTCCTGC	exonic	C6	nonsynonymous SNV	C6:ENST00000263413.3:exon6:c.C611T:p.P204L,C6:ENST00000337836.5:exon6:c.C611T:p.P204L,	5p13.1	C6;basechange=c.611C>T;AAchange=p.P204L;tissue=endometrium&C6;basechange=c.611C>A;AAchange=p.P204H;tissue=lung
5	45695862	G	A	CCAAACATGC,GAGGGAGAAT	exonic	HCN1	nonsynonymous SNV	HCN1:ENST00000303230.4:exon1:c.C334T:p.R112C,	5p12	.
5	54518110	G	T	CAGCGGTGGT,ACTGCAGGGC	exonic	MCIDAS	stopgain	MCIDAS:ENST00000513312.1:exon5:c.C500A:p.S167X,	5q11.2	.
5	55256257	C	T	CCACTTGCTT,TTCACTCCAG	exonic	IL6ST	nonsynonymous SNV	IL6ST:ENST00000502326.3:exon7:c.G946A:p.E316K,IL6ST:ENST00000381294.3:exon6:c.G946A:p.E316K,IL6ST:ENST00000381287.4:exon6:c.G946A:p.E316K,IL6ST:ENST00000381298.2:exon8:c.G946A:p.E316K,IL6ST:ENST00000336909.5:exon6:c.G946A:p.E316K,IL6ST:ENST00000381293.2:exon4:c.G448A:p.E	5q11.2	.
5	59189406	C	T	GCTGCCCTCT,CGCTGCCCGC	exonic	PDE4D	nonsynonymous SNV	PDE4D:ENST00000340635.6:exon1:c.G44A:p.G15E,	5q12.1	.
5	63871683	A	T	TACCACAGAG,TGCTAAAATC	exonic	RGS7BP	nonsynonymous SNV	RGS7BP:ENST00000334025.2:exon3:c.A415T:p.M139L,	5q12.3	.
5	64838669	G	A	TTCAGTCAAG,GAATTGCTAC	exonic	CENPK	nonsynonymous SNV	CENPK:ENST00000396679.1:exon6:c.C248T:p.P83L,CENPK:ENST00000242872.3:exon4:c.C248T:p.P83L,CENPK:ENST00000510693.1:exon6:c.C158T:p.P53L,CENPK:ENST00000510768.1:exon6:c.C248T:p.P83L,CENPK:ENST00000515497.1:exon6:c.C254T:p.P85L,CENPK:ENST00000502997.1:exon4:c.C248T:p.	5q12.3	.
5	64838670	G	A	TCAGTCAAGG,AATTGCTACA	exonic	CENPK	nonsynonymous SNV	CENPK:ENST00000396679.1:exon6:c.C247T:p.P83S,CENPK:ENST00000242872.3:exon4:c.C247T:p.P83S,CENPK:ENST00000510693.1:exon6:c.C157T:p.P53S,CENPK:ENST00000510768.1:exon6:c.C247T:p.P83S,CENPK:ENST00000515497.1:exon6:c.C253T:p.P85S,CENPK:ENST00000502997.1:exon4:c.C247T:p	5q12.3	.
5	70307202	C	T	TCCTGTTTAC,TATATATGAA	splicing	NAIP(ENST0	.	.	5q13.2	.
5	70806623	C	T	GAAGAAATTT,CCAAAGGGAA	exonic	BDP1	nonsynonymous SNV	BDP1:ENST00000358731.4:exon17:c.C3704T:p.S1235F,	5q13.2	.
5	72744099	T	C	CTCTTCCTCG,CTTCTTCGTC	exonic	FOXD1	nonsynonymous SNV	FOXD1:ENST00000499003.3:exon1:c.A89G:p.D30G,	5q13.2	.
5	82833515	G	A	CTTTGCAAGG,CTACAGAAGT	exonic	VCAN	nonsynonymous SNV	VCAN:ENST00000513960.1:exon7:c.G1732A:p.A578T,VCAN:ENST00000343200.5:exon7:c.G1732A:p.A578T,VCAN:ENST00000265077.3:exon8:c.G4693A:p.A1565T,	5q14.3	.
5	90144546	G	C	CAAAGCGCAA,GACACTAGGG	exonic	GPR98	nonsynonymous SNV	GPR98:ENST00000425867.2:exon15:c.G4095C:p.K1365N,GPR98:ENST00000405460.2:exon79:c.G17112C:p.K5704N,	5q14.3	.
5	95728838	G	A	AAGCTGGGAA,GTTTGTTCAG	exonic	PCSK1	nonsynonymous SNV	PCSK1:ENST00000311106.3:exon14:c.C2129T:p.P710L,PCSK1:ENST00000508626.1:exon14:c.C1988T:p.P663L,	5q15	.
5	114607005	C	T	CTCAAAAATT,TTCCTGTTCT	exonic	CCDC112	nonsynonymous SNV	CCDC112:ENST00000395557.4:exon7:c.G988A:p.E330K,CCDC112:ENST00000506442.1:exon7:c.G988A:p.E330K,CCDC112:ENST00000379611.5:exon7:c.G1237A:p.E413K,CCDC112:ENST00000512261.1:exon8:c.G988A:p.E330K,	5q22.3	.
5	127648419	C	T	ACCCCGATCT,GGTGTTGCAA	exonic	FBN2	nonsynonymous SNV	FBN2:ENST00000262464.4:exon37:c.G4786A:p.E1596K,FBN2:ENST00000508053.1:exon43:c.G4786A:p.E1596K,	5q23.3	FBN2;basechange=c.4786G>A;AAchange=p.E1596K;tissue=skin&FBN2;basechange=c.4786G>T;AAchange=p.E1596*;tissue=upper_aerodigestive_tract&FBN2_ENST00000508053;basechange=c.4786G>A;AAchange=p.E1596K;tissue=skin&FBN2_ENST00000508053;basechange=c.4786G>T;AAchange=p.E1
5	139260465	G	A	AGCCGGCCCC,GACGGTGTCC	exonic	NRG2	nonsynonymous SNV	NRG2:ENST00000289422.7:exon3:c.C967T:p.R323W,NRG2:ENST00000289409.4:exon3:c.C967T:p.R323W,NRG2:ENST00000378238.4:exon3:c.C967T:p.R323W,NRG2:ENST00000394770.1:exon3:c.C967T:p.R323W,NRG2:ENST00000541337.1:exon3:c.C967T:p.R323W,NRG2:ENST00000361474.1:exon3:c.C967T	5q31.2	NRG2;basechange=c.967C>T;AAchange=p.R323W;tissue=pancreas
5	140181962	C	T	GCCCCACGTC,CCTTCAAGCT	exonic	PCDHA3	nonsynonymous SNV	PCDHA3:ENST00000522353.2:exon1:c.C1180T:p.P394S,PCDHA3:ENST00000532566.2:exon1:c.C1180T:p.P394S,	5q31.3	.
5	140202640	G	A	GTGACCGCGC,GGACGGGGGC	exonic	PCDHA5	nonsynonymous SNV	PCDHA5:ENST00000378126.3:exon1:c.G1280A:p.R427Q,PCDHA5:ENST00000529859.1:exon1:c.G1280A:p.R427Q,PCDHA5:ENST00000529619.1:exon1:c.G1280A:p.R427Q,	5q31.3	PCDHA5;basechange=c.1280G>A;AAchange=p.R427Q;tissue=endometrium&PCDHA5_ENST00000529859;basechange=c.1280G>A;AAchange=p.R427Q;tissue=endometrium
5	140474745	G	A	GCGGAGCTAC,GATTAGGGAC	exonic	PCDHB2	nonsynonymous SNV	PCDHB2:ENST00000194155.4:exon1:c.G371A:p.R124Q,	5q31.3	PCDHB2;basechange=c.371G>A;AAchange=p.R124Q;tissue=endometrium
5	140580192	C	T	TATACCTTTT,CCATGCCTCA	exonic	PCDHB11	nonsynonymous SNV	PCDHB11:ENST00000354757.3:exon1:c.C845T:p.S282F,	5q31.3	.
5	140744933	G	A	CAATGCCCCC,AAGTGATCCT	exonic	PCDHGA5	nonsynonymous SNV	PCDHGA5:ENST00000518069.1:exon1:c.G1036A:p.E346K,	5q31.3	PCDHGA5_ENST00000518069;basechange=c.1036G>A;AAchange=p.E346K;tissue=large_intestine
5	140797521	C	T	CTGTGTGAGC,GATCCGCTAC	exonic	PCDHGB7	nonsynonymous SNV	PCDHGB7:ENST00000398594.2:exon1:c.C95T:p.P32L,	5q31.3	.
5	140866254	C	T	GTATCAGCTT,CTCTTTCATC	exonic	PCDHGC4	nonsynonymous SNV	PCDHGC4:ENST00000306593.1:exon1:c.C1514T:p.S505F,	5q31.3	.
5	148742286	G	A	CGACGTGTAC,AGAAGGGAAC	exonic	PCYOX1L	nonsynonymous SNV	PCYOX1L:ENST00000510990.2:exon2:c.G175A:p.E59K,PCYOX1L:ENST00000505669.1:exon2:c.G191A:p.R64Q,PCYOX1L:ENST00000511945.1:exon2:c.G191A:p.R64Q,PCYOX1L:ENST00000274569.4:exon2:c.G175A:p.E59K,	5q32	.
5	149357604	C	T	ATTGCTTATT,CCTGCTGGCT	exonic	SLC26A2	nonsynonymous SNV	SLC26A2:ENST00000286298.4:exon2:c.C389T:p.S130F,SLC26A2:ENST00000503336.1:exon1:c.C62T:p.S21F,	5q32	.
5	150028136	C	T	AATCAGAACC,ACCGGCAACT	exonic	SYNPO	nonsynonymous SNV	SYNPO:ENST00000394243.1:exon3:c.C1031T:p.P344L,SYNPO:ENST00000522122.1:exon3:c.C1031T:p.P344L,SYNPO:ENST00000519664.1:exon2:c.C299T:p.P100L,SYNPO:ENST00000307662.4:exon2:c.C299T:p.P100L,	5q33.1	.
5	156381548	C	T	GGAGACATCA,CTCTCGGGAT	exonic	TIMD4	nonsynonymous SNV	TIMD4:ENST00000274532.2:exon2:c.G278A:p.G93D,TIMD4:ENST00000407087.3:exon2:c.G278A:p.G93D,	5q33.3	.
5	156476133	G	A	TCTGCTGGCT,AGGTGAAGAT	exonic	HAVCR1	stopgain	HAVCR1:ENST00000339252.3:exon4:c.C697T:p.Q233X,HAVCR1:ENST00000425854.1:exon4:c.C697T:p.Q233X,HAVCR1:ENST00000522693.1:exon5:c.C697T:p.Q233X,HAVCR1:ENST00000523175.1:exon5:c.C697T:p.Q233X,HAVCR1:ENST00000544197.1:exon5:c.C697T:p.Q233X,	5q33.3	HAVCR1;basechange=c.697C>T;AAchange=p.Q233*;tissue=lung
5	158139237	C	T	GCGGCAGAGC,GTATCCGTTC	exonic	EBF1	nonsynonymous SNV	EBF1:ENST00000313708.6:exon14:c.G1474A:p.G492S,EBF1:ENST00000380654.4:exon13:c.G1381A:p.G461S,	5q33.3	EBF1;basechange=c.1474G>T;AAchange=p.G492C;tissue=lung
5	162886981	C	T	AACACCTCCT,CAAGGTCTGG	exonic	NUDCD2	nonsynonymous SNV	NUDCD2:ENST00000302764.4:exon1:c.G76A:p.E26K,NUDCD2:ENST00000517501.1:exon1:c.G76A:p.E26K,	5q34	.
5	176759252	G	A	CGTTGTCTGG,GGAGAAGAAA	exonic	LMAN2	nonsynonymous SNV	LMAN2:ENST00000515209.1:exon8:c.C929T:p.P310L,	5q35.3	.
5	179744032	C	T	TCGAAGATCT,CTTCTGCATA	exonic	GFPT2	nonsynonymous SNV	GFPT2:ENST00000253778.8:exon11:c.G985A:p.E329K,	5q35.3	.
6	11778910	C	T	CTCGTCTTTT,CTTCCTGTGA	exonic	ADTRP	nonsynonymous SNV	ADTRP:ENST00000414691.3:exon1:c.G83A:p.G28E,ADTRP:ENST00000379415.2:exon3:c.G83A:p.G28E,ADTRP:ENST00000485323.2:exon1:c.G83A:p.G28E,ADTRP:ENST00000229583.5:exon1:c.G83A:p.G28E,ADTRP:ENST00000379413.2:exon1:c.G83A:p.G28E,ADTRP:ENST00000506810.1:exon2:c.G83A:p.G28	6p24.1	.
6	13230558	T	C	CCGAGGCAGG,GGATCACATG	exonic	PHACTR1	nonsynonymous SNV	PHACTR1:ENST00000434977.1:exon5:c.T682C:p.W228R,PHACTR1:ENST00000379345.2:exon6:c.T589C:p.W197R,	6p24.1	.
6	24515441	C	T	AATGTTATTC,CTGTTCTCGA	exonic	ALDH5A1	nonsynonymous SNV	ALDH5A1:ENST00000348925.2:exon6:c.C812T:p.P271L,ALDH5A1:ENST00000491546.1:exon4:c.C689T:p.P230L,ALDH5A1:ENST00000357578.3:exon5:c.C773T:p.P258L,ALDH5A1:ENST00000546278.1:exon6:c.C509T:p.P170L,	6p22.3	.
6	24581180	C	T	ATTGACAAAT,CTTCTCCAAA	exonic	KIAA0319	nonsynonymous SNV	KIAA0319:ENST00000535378.1:exon8:c.G1226A:p.G409E,KIAA0319:ENST00000543707.1:exon7:c.G1253A:p.G418E,KIAA0319:ENST00000378214.3:exon7:c.G1253A:p.G418E,KIAA0319:ENST00000537886.1:exon7:c.G1253A:p.G418E,KIAA0319:ENST00000430948.2:exon6:c.G1118A:p.G373E,	6p22.3	.
6	25778228	G	A	ACTGCTGCTG,ATTTTTCATC	exonic	SLC17A4	nonsynonymous SNV	SLC17A4:ENST00000377905.4:exon11:c.G1343A:p.G448E,SLC17A4:ENST00000439485.2:exon6:c.G653A:p.G218E,	6p22.2	SLC17A4;basechange=c.1343G>T;AAchange=p.G448V;tissue=lung
6	26385450	C	T	GGAAGAATCA,CTTTGTGAGC	exonic	BTN2A2	nonsynonymous SNV	BTN2A2:ENST00000432533.2:exon3:c.C302T:p.T101I,BTN2A2:ENST00000356709.4:exon3:c.C302T:p.T101I,BTN2A2:ENST00000469230.1:exon3:c.C302T:p.T101I,BTN2A2:ENST00000493275.1:exon3:c.C302T:p.T101I,BTN2A2:ENST00000416795.2:exon3:c.C302T:p.T101I,	6p22.2	.
6	26452376	G	T	TCTATATCCT,TTTTCAGAAT	exonic	BTN3A3	nonsynonymous SNV	BTN3A3:ENST00000244519.2:exon11:c.G1492T:p.V498F,BTN3A3:ENST00000339789.4:exon11:c.G1366T:p.V456F,BTN3A3:ENST00000361232.3:exon10:c.G1345T:p.V449F,	6p22.2	.

6	78173118	C	T	CCGGTTCCTC,ATGGCTCTCC	exonic	HTR1B	nonsynonymous SNV	HTR1B:ENST00000369947.2:exon1:c.G3A:p.M1I,	6q14.1	.
6	87965964	C	T	ATCTATGCTT,CTTCAGAAAA	exonic	ZNF292	nonsynonymous SNV	ZNF292:ENST00000369577.3:exon8:c.C2617T:p.P873S,ZNF292:ENST00000339907.4:exon8:c.C2602T:p.P868S,	6q14.3	.
6	100901651	G	A	GAGCAGATGG,AGCCCAGTTC	exonic	SIM1	nonsynonymous SNV	SIM1:ENST00000369208.3:exon3:c.C245T:p.S82F,SIM1:ENST00000262901.4:exon2:c.C245T:p.S82F,	6q16.3	.
6	106553434	C	T	CACTTCTCTC,CGAGCTCGCT	exonic	PRDM1	nonsynonymous SNV	PRDM1:ENST00000369091.2:exon5:c.C1291T:p.P431S,PRDM1:ENST00000369096.4:exon5:c.C1399T:p.P467S,PRDM1:ENST00000369089.3:exon3:c.C997T:p.P333S,	6q21	.
6	111587201	C	T	GGCTTTGGGT,CGACAGCGTC	exonic	KIAA1919	nonsynonymous SNV	KIAA1919:ENST00000368847.4:exon4:c.C436T:p.P146S,	6q21	.
6	116442149	C	T	ACCCCTTTCA,CCTTAGCCCC	exonic	COL10A1	nonsynonymous SNV	COL10A1:ENST00000327673.4:exon2:c.G1130A:p.G377D,COL10A1:ENST00000243222.4:exon3:c.G1130A:p.G377D,	6q22.1	.
6	116720676	C	T	TACCTCCCTC,CTGGGATCCC	exonic	DSE	nonsynonymous SNV	DSE:ENST00000331677.3:exon3:c.C263T:p.P88L,DSE:ENST00000452085.3:exon2:c.C263T:p.P88L,DSE:ENST00000430252.2:exon3:c.C263T:p.P88L,DSE:ENST00000537543.1:exon2:c.C320T:p.P107L,DSE:ENST00000359564.2:exon2:c.C263T:p.P88L,	6q22.1	.
6	117841113	G	A	CGACCATCCA,GTATAACGGA	exonic	DCBLD1	nonsynonymous SNV	DCBLD1:ENST00000368503.4:exon3:c.G460A:p.D154N,DCBLD1:ENST00000338728.5:exon3:c.G460A:p.D154N,DCBLD1:ENST00000296955.8:exon3:c.G460A:p.D154N,	6q22.1	.
6	126073205	G	A	TCTGAGTTGA,AAGACTTGTG	exonic	HEY2	nonsynonymous SNV	HEY2:ENST00000368364.3:exon3:c.G215A:p.R72K,HEY2:ENST00000368365.1:exon3:c.G77A:p.R26K,	6q22.31	.
6	130762202	C	G	ACGATCGCCT,TTTCTCGGGT	exonic	TMEM200A	nonsynonymous SNV	TMEM200A:ENST00000545622.1:exon2:c.C635G:p.S212C,TMEM200A:ENST00000392429.1:exon2:c.C635G:p.S212C,TMEM200A:ENST00000296978.3:exon3:c.C635G:p.S212C,	6q23.1	KIAA1913;basechange=c.635C>A;AAchange=p.S212Y;tissue=lung&TMEM200A;basechange=c.635C>A;AAchange=p.S212Y;tissue=lung
6	132006554	C	T	TGATTATTTT,CCAGAATAAA	exonic	ENPP3	nonsynonymous SNV	ENPP3:ENST00000357639.3:exon13:c.C1171T:p.P391S,ENPP3:ENST00000358229.5:exon13:c.C1171T:p.P391S,ENPP3:ENST00000414305.1:exon14:c.C1171T:p.P391S,	6q23.2	.
6	132892175	G	A	TAGCAAGACA,AATCATCCTC	exonic	TAAR6	nonsynonymous SNV	TAAR6:ENST00000275198.1:exon1:c.G715A:p.E239K,	6q23.2	.
6	134350566	G	A	CCCACTATAA,AACCGTGTAG	exonic	SLC2A12	nonsynonymous SNV	SLC2A12:ENST00000275230.5:exon2:c.C399T:p.L133F,	6q23.2	.
6	137527354	G	A	CTGACCCAAA,AGAATTTGAT	exonic	IFNGR1	nonsynonymous SNV	IFNGR1:ENST00000414770.1:exon4:c.C262T:p.L88F,IFNGR1:ENST00000543628.1:exon3:c.C208T:p.L70F,IFNGR1:ENST00000367739.4:exon3:c.C292T:p.L98F,IFNGR1:ENST00000367735.2:exon4:c.C262T:p.L88F,	6q23.3	.
6	143093564	G	A	TGACACAAGA,ATGGACTTCC	exonic	HIVEP2	nonsynonymous SNV	HIVEP2:ENST00000367604.1:exon2:c.C2312T:p.S771F,HIVEP2:ENST00000367603.2:exon5:c.C2312T:p.S771F,HIVEP2:ENST00000012134.2:exon3:c.C2312T:p.S771F,	6q24.2	.
6	144898346	T	G	CCACAGTGAA,GTGCTTGTGG	exonic	UTRN	nonsynonymous SNV	UTRN:ENST00000367545.3:exon50:c.T7401G:p.N2467K,	6q24.2	.
6	147042602	G	A	TGAACTACTG,TTTGCTTTTC	exonic	ADGB	nonsynonymous SNV	ADGB:ENST00000367493.3:exon17:c.G313A:p.V105I,ADGB:ENST00000397944.3:exon17:c.G2056A:p.V686I,	6q24.3	.
6	148792616	G	A	AACCAGAAAG,AATAATGAGA	exonic	SASH1	nonsynonymous SNV	SASH1:ENST00000367467.3:exon6:c.G491A:p.G164E,	6q24.3	.
6	149983275	G	A	TTGCCTAAGC,ATCTTCGGGT	exonic	LATS1	nonsynonymous SNV	LATS1:ENST00000253339.5:exon7:c.C2983T:p.R995C,LATS1:ENST00000543571.1:exon8:c.C2983T:p.R995C,	6q25.1	LATS1_ENST00000253339;basechange=c.2983C>T;AAchange=p.R995C;tissue=breast
6	154489144	C	T	TTTCTCAACT,CTTTTTAGTC	exonic	IPCEF1	nonsynonymous SNV	IPCEF1:ENST00000265198.4:exon11:c.G1012A:p.E338K,IPCEF1:ENST00000519344.1:exon8:c.G928A:p.E310K,IPCEF1:ENST00000367220.4:exon11:c.G1015A:p.E339K,IPCEF1:ENST00000422970.2:exon12:c.G1015A:p.E339K,	6q25.2	.
6	154521105	T	C	TTCACTGTAT,TTCCAGGGAA	exonic	IPCEF1	nonsynonymous SNV	IPCEF1:ENST00000265198.4:exon10:c.A604G:p.N202D,IPCEF1:ENST00000519344.1:exon7:c.A520G:p.N174D,IPCEF1:ENST00000367220.4:exon10:c.A607G:p.N203D,IPCEF1:ENST00000422970.2:exon11:c.A607G:p.N203D,	6q25.2	.
6	155153532	C	T	CCAAGATTCC,TTTAATACAG	exonic	SCAF8	nonsynonymous SNV	SCAF8:ENST00000417268.1:exon21:c.C2819T:p.P940L,SCAF8:ENST00000367186.4:exon22:c.C3017T:p.P1006L,SCAF8:ENST00000367178.3:exon20:c.C2819T:p.P940L,	6q25.2	.
6	155450857	G	A	AAAACGCTGG,GAAGCTGGAT	exonic	TIAM2	nonsynonymous SNV	TIAM2:ENST00000461783.5:exon6:c.G500A:p.G167E,TIAM2:ENST00000360366.4:exon1:c.G500A:p.G167E,TIAM2:ENST00000528535.1:exon4:c.G500A:p.G167E,TIAM2:ENST00000535583.1:exon3:c.G500A:p.G167E,TIAM2:ENST00000456144.1:exon3:c.G500 (cut)	6q25.2	.
6	161470409	C	T	AGCACTTTAT,CATCATTGCA	exonic	MAP3K4	nonsynonymous SNV	MAP3K4:ENST00000392142.4:exon3:c.C1105T:p.P369S,MAP3K4:ENST00000490904.2:exon3:c.C1105T:p.P369S,MAP3K4:ENST00000366919.2:exon3:c.C1105T:p.P369S,MAP3K4:ENST00000348824.7:exon3:c.C1105T:p.P369S,MAP3K4:ENST00000544041.1:exon3:c.C1105T:p.P369S,MAP3K4:ENST0000036692 (cut)	6q26	.
6	161990397	C	T	CTGCTCTTCT,CCAGAATCCT	exonic	PARK2	nonsynonymous SNV	PARK2:ENST00000366892.1:exon6:c.G923A:p.G308E,PARK2:ENST00000338468.3:exon7:c.G350A:p.G117E,PARK2:ENST00000366897.1:exon7:c.G839A:p.G280E,PARK2:ENST00000366896.1:exon5:c.G476A:p.G159E,PARK2:ENST00000366894.1:exon7:c.G350A:p.G117E,PARK2:ENST00000366898.1:exon8:c.G (cut)	6q26	.
6	161990398	C	T	TGCTCTTCTC,CAGAATCCTG	exonic	PARK2	nonsynonymous SNV	PARK2:ENST00000366892.1:exon6:c.G922A:p.G308R,PARK2:ENST00000338468.3:exon7:c.G349A:p.G117R,PARK2:ENST00000366897.1:exon7:c.G838A:p.G280R,PARK2:ENST00000366896.1:exon5:c.G475A:p.G159R,PARK2:ENST00000366894.1:exon7:c.G349A:p.G117R,PARK2:ENST00000366898.1:exon8:c. (cut)	6q26	.
6	165715363	C	T	CTGACAGCCT,CACTGGAAGG	exonic	C6orf118	nonsynonymous SNV	C6orf118:ENST00000230301.8:exon2:c.G448A:p.E150K,C6orf118:ENST00000543069.1:exon2:c.G136A:p.E46K,	6q27	.
6	165715528	C	T	GGCGGCTCTC,CACCTCAGAG	exonic	C6orf118	nonsynonymous SNV	C6orf118:ENST00000230301.8:exon2:c.G283A:p.G95R,	6q27	.
6	165715772	C	T	CGCAGTGCTT,CACTTCAGGT	exonic	C6orf118	stopgain	C6orf118:ENST00000230301.8:exon2:c.G39A:p.W13X,	6q27	.
6	165832192	T	A	AAGGTCTGAA,ATAACTCCTT	exonic	PDE10A	nonsynonymous SNV	PDE10A:ENST00000354446.4:exon11:c.A899T:p.Y300F,PDE10A:ENST00000366882.1:exon12:c.A899T:p.Y300F,PDE10A:ENST00000539869.2:exon11:c.A929T:p.Y310F,	6q27	.
6	166576037	G	A	GGGAGGGAGA,GGCACCTCCA	exonic	T	nonsynonymous SNV	T:ENST00000366876.2:exon6:c.C805T:p.L269F,T:ENST00000296946.2:exon7:c.C802T:p.L268F,	6q27	.
6	167571207	G	A	GAACAGGAAG,TCCACAGCGC	exonic	GPR31	nonsynonymous SNV	GPR31:ENST00000366834.1:exon1:c.C113T:p.T38I,	6q27	.
6	167585750	G	A	GGTCCCCAGG,AGGACAGCAA	exonic	TCP10L2	nonsynonymous SNV	TCP10L2:ENST00000366832.2:exon2:c.G118A:p.E40K,TCP10L2:ENST00000283507.6:exon2:c.G118A:p.E40K,TCP10L2:ENST00000486697.2:exon4:c.G118A:p.E40K,	6q27	.
6	167753762	G	A	AACCTGTACT,GAGGACATCC	exonic	TTLL2	stopgain	TTLL2:ENST00000515138.1:exon3:c.C374A:p.W125X,TTLL2:ENST00000239587.5:exon3:c.C374A:p.W125X,	6q27	.
6	168366449	G	A	GCGACAGGAA,AAGGGTATTA	exonic	MLLT4	nonsynonymous SNV	MLLT4:ENST00000366806.2:exon32:c.G4960A:p.E1654K,MLLT4:ENST00000351017.4:exon32:c.G4981A:p.E1661K,MLLT4:ENST00000447894.2:exon31:c.G4960A:p.E1654K,MLLT4:ENST00000400822.3:exon32:c.G4990A:p.E1664K,MLLT4:ENST00000392112.1:exon31:c.G4909A:p.E1637K,	6q27	.
6	170034526	G	A	AAGGATACGG,AATAAAAGTC	exonic	WDR27	nonsynonymous SNV	WDR27:ENST00000423258.1:exon17:c.C1718T:p.S573F,WDR27:ENST00000333572.6:exon20:c.C2099T:p.S700F,WDR27:ENST00000448612.1:exon20:c.C2099T:p.S700F,	6q27	WDR27_ENST00000333572;basechange=c.2099C>T;AAchange=p.S700F;tissue=upper_aerodigestive_tract
6	170657361	G	A	GCAGGCTGTC,AAAGCCCATT	exonic	FAM120B	nonsynonymous SNV	FAM120B:ENST00000476760.1:exon6:c.G2209A:p.E737K,FAM120B:ENST00000544080.1:exon5:c.G2178A:p.E726K,FAM120B:ENST00000252510.9:exon3:c.G136A:p.E46K,FAM120B:ENST00000476287.1:exon5:c.G2140A:p.E714K,	6q27	.
7	5922243	G	A	GTACCTGGAT,AAGAAGAGCT	exonic	OCM	nonsynonymous SNV	OCM:ENST00000242104.5:exon2:c.G181A:p.E61K,OCM:ENST00000416608.1:exon3:c.G181A:p.E61K,	7p22.1	.
7	11521441	G	A	GGCCAGAATG,ATCGTGCTCG	exonic	THSD7A	nonsynonymous SNV	THSD7A:ENST00000423059.4:exon7:c.C1991T:p.S664F,	7p21.3	.
7	12373119	G	A	TTTGACTCCA,AGTATTCAGT	exonic	VWDE	nonsynonymous SNV	VWDE:ENST00000275358.3:exon28:c.C4730T:p.S1577F,	7p21.3	.
7	12409926	G	A	AATATATTCG,AGGTGACATC	exonic	VWDE	nonsynonymous SNV	VWDE:ENST00000452576.2:exon12:c.C2006T:p.S669F,VWDE:ENST00000275358.3:exon12:c.C2006T:p.S669F,	7p21.3	.
7	16655376	G	A	TACCTTGACA,GATGAAGATT	exonic	ANKMY2	nonsynonymous SNV	ANKMY2:ENST00000447802.1:exon5:c.C524T:p.P175L,ANKMY2:ENST00000306999.2:exon5:c.C524T:p.P175L,	7p21.1	.
7	18067374	G	A	GTCCTGGTGG,AGCTGCCGCT	exonic	PRPS1L1	nonsynonymous SNV	PRPS1L1:ENST00000506618.2:exon1:c.C32T:p.S11F,	7p21.1	.
7	23226765	G	A	TAATATTTCA,GTAACTGAAA	exonic	NUPL2	nonsynonymous SNV	NUPL2:ENST00000410002.3:exon3:c.G445A:p.V149I,NUPL2:ENST00000413919.1:exon3:c.G445A:p.G149S,NUPL2:ENST00000438012.1:exon3:c.G445A:p.G149S,NUPL2:ENST00000258742.5:exon4:c.G445A:p.G149S,	7p15.3	.
7	25218793	C	T	TTTTGTTTTA,CTGTCCTCTG	splicing	C7orf31(ENS	.	.	7p15.3	.
7	28848856	A	G	CAGCCACCCC,GCCCACAGGG	exonic	CREB5	nonsynonymous SNV	CREB5:ENST00000396299.2:exon5:c.A662G:p.Q221R,CREB5:ENST00000409603.1:exon6:c.A980G:p.Q327R,CREB5:ENST00000396300.2:exon9:c.A1058G:p.Q353R,CREB5:ENST00000426500.1:exon5:c.A557G:p.Q186R,CREB5:ENST00000357727.2:exon9: (cut)	7p14.3	.
7	30793466	G	A	CCGCAACCGG,AGGAGCTGGA	exonic	INMT,INMT-F	nonsynonymous SNV	INMT:ENST00000013222.5:exon2:c.G274A:p.E92K,INMT:ENST00000409539.1:exon2:c.G271A:p.E91K,INMT-FAM188B:ENST00000458257.1:exon2:c.G271A:p.E91K,	7p14.3	.
7	31614287	G	A	AGCAGCCAGA,GAATCAACAT	exonic	CCDC129	nonsynonymous SNV	CCDC129:ENST00000409210.1:exon5:c.G253A:p.G85R,CCDC129:ENST00000407970.3:exon7:c.G529A:p.G177R,CCDC129:ENST00000319386.3:exon7:c.G529A:p.G177R,CCDC129:ENST00000451887.2:exon7:c.G607A:p.G203R,	7p14.3	.
7	37953878	G	A	CACTTGCACC,ATCTAAAAAA	exonic	SFRP4	nonsynonymous SNV	SFRP4:ENST00000436072.2:exon2:c.C529T:p.R177W,SFRP4:ENST00000447200.2:exon4:c.C127T:p.R43W,	7p14.1	.
7	38393397	G	T	TCTTCCTTGT,CTTCCGTAAG	exonic	TRGV4	nonsynonymous SNV	TRGV4:ENST00000390345.2:exon2:c.C273A:p.S91R,	7p14.1	.
7	39990272	G	A	GCGCTGGGGG,AGGCGGGGGC	exonic	CDK13	nonsynonymous SNV	CDK13:ENST00000181839.4:exon1:c.G32A:p.G11E,CDK13:ENST00000340829.5:exon1:c.G32A:p.G11E,	7p14.1	.
7	41729952	C	T	CCCACTTCCT,GGCCTCTTCC	exonic	INHBA	nonsynonymous SNV	INHBA:ENST00000442711.1:exon2:c.G577A:p.E193K,INHBA:ENST00000242208.4:exon3:c.G577A:p.E193K,	7p14.1	.
7	43908602	G	C	GGGCGATGTA,TGCGGCGCGT	exonic	MRPS24,URG	nonsynonymous SNV	URGCP-MRPS24:ENST00000603700.1:exon6:c.C316G:p.L106V,MRPS24:ENST00000317534.5:exon3:c.C180G:p.H60Q,	7p13	.
7	44146156	A	T	GCCTCCGGAA,AGACCAAAGA	exonic	AEBP1	stopgain	AEBP1:ENST00000223357.3:exon2:c.A265T:p.K89X,AEBP1:ENST00000449162.1:exon2:c.A13T:p.K5X,	7p13	.
7	44259774	C	T	CGGGGCCGGC,CTGCCCGTCA	exonic	CAMK2B	nonsynonymous SNV	CAMK2B:ENST00000347193.4:exon19:c.G1366A:p.G456S,CAMK2B:ENST00000258682.6:exon19:c.G1441A:p.G481S,CAMK2B:ENST00000457475.1:exon19:c.G1444A:p.G482S,CAMK2B:ENST00000346990.4:exon15:c.G1237A:p.G413S,CAMK2B:ENST00000358707.3:exon18:c.G1399A:p.G467S,CAMK2B:ENST0 (cut)	7p13	.
7	44274055	C	T	TGCTATTCGT,TGGGGCTGTG	exonic	CAMK2B	nonsynonymous SNV	CAMK2B:ENST00000353185.3:exon13:c.G947A:p.R316K,	7p13	.
7	47944111	G	A	GCTGAGGAGG,GGACGTGAGC	exonic	PKD1L1	nonsynonymous SNV	PKD1L1:ENST00000289672.2:exon12:c.C1795T:p.P599S,	7p12.3	.
7	48887759	G	C	CGCCGCGCGG,CTCTCAGGAC	exonic	AC004899.1	nonsynonymous SNV	AC004899.1:ENST00000596947.1:exon1:c.C259G:p.A87P,	7p12.3	.
7	50367248	C	T	GGAAAGCCCC,CTGTAAGCGA	exonic	IKZF1	nonsynonymous SNV	IKZF1:ENST00000357364.4:exon2:c.C55T:p.P19S,IKZF1:ENST00000349824.4:exon2:c.C55T:p.P19S,IKZF1:ENST00000359197.5:exon2:c.C55T:p.P19S,IKZF1:ENST00000331340.3:exon2:c.C55T:p.P19S,IKZF1:ENST00000413698.1:exon3:c.C55T:p.P19S,IKZF1:ENST00000346667.4:exon2:c.C55T:p.P19S,IKZF1:EN (cut)	7p12.2	.
7	50605626	C	T	AGCATCTTCC,GAGCCAGTCC	exonic	DDC	nonsynonymous SNV	DDC:ENST00000444124.2:exon4:c.G367A:p.G123R,DDC:ENST00000444733.1:exon3:c.G253A:p.G85R,DDC:ENST00000380984.4:exon4:c.G367A:p.G123R,DDC:ENST00000357936.5:exon4:c.G367A:p.G123R,DDC:ENST00000431062.1:exon3:c.G367A:p.G123R,	7p12.1	.
7	53103866	G	A	CGCCGCCCAG,AGCTCCTGGA	exonic	POM121L12	nonsynonymous SNV	POM121L12:ENST00000408890.4:exon1:c.G502A:p.E168K,	7p12.1	.
7	54612347	G	A	GAACGTGACG,CGACCGAGGG	exonic	VSTM2A	nonsynonymous SNV	VSTM2A:ENST00000402026.2:exon2:c.G109A:p.A37T,VSTM2A:ENST00000407838.3:exon2:c.G112A:p.A38T,VSTM2A:ENST00000402613.3:exon2:c.G112A:p.A38T,VSTM2A:ENST00000302287.3:exon2:c.G112A:p.A38T,VSTM2A:ENST00000404951.1:exon2:c.G112A:p.A38T,	7p11.2	.
7	63726765	T	C	TTTTACCTGG,CCTCAACCCT	exonic	ZNF679	nonsynonymous SNV	ZNF679:ENST00000421025.1:exon5:c.T754C:p.S252P,ZNF679:ENST00000255746.4:exon4:c.T754C:p.S252P,	7q11.21	.
7	70800548	C	T	TTATCCAAAT,CCTTGGGCTC	exonic	WBSCR17	nonsynonymous SNV	WBSCR17:ENST00000447516.1:exon2:c.C185T:p.S62F,WBSCR17:ENST00000333538.5:exon2:c.C251T:p.S84F,	7q11.22	.
7	75211410	C	T	ACGTCACTTT,TCCAGCCTCG	exonic	HIP1	nonsynonymous SNV	HIP1:ENST00000434438.2:exon6:c.G523A:p.E175K,HIP1:ENST00000420909.1:exon6:c.G436A:p.E146K,HIP1:ENST00000336926.6:exon6:c.G523A:p.E175K,	7q11.23	HIP1;basechange=c.523G>T;AAchange=p.E175*;tissue=large_intestine
7	82455913	G	A	ACCTCCCCAA,GAAGTCGTTG	exonic	PCLO	nonsynonymous SNV	PCLO:ENST00000423517.2:exon18:c.C14407T:p.L4803F,PCLO:ENST00000437081.1:exon18:c.C14407T:p.L4803F,	7q21.11	.
7	82544398	C	T	ATTGATAAAT,AAGCCCATAG	exonic	PCLO	nonsynonymous SNV	PCLO:ENST00000437081.1:exon2:c.G3064A:p.D1022N,PCLO:ENST00000423517.2:exon2:c.G12904A:p.D4302N,PCLO:ENST00000333891.9:exon2:c.G12904A:p.D4302N,	7q21.11	.
7	82582174	G	A	GGCTCTGGAG,AATTGTTATG	exonic	PCLO	nonsynonymous SNV	PCLO:ENST00000423517.2:exon5:c.C8095T:p.P2699S,PCLO:ENST00000333891.9:exon5:c.C8095T:p.P2699S,	7q21.11	.
7	83037717	G	A	TGCTCAGTGC,GATATGGGCC	exonic	SEMA3E	nonsynonymous SNV	SEMA3E:ENST00000442159.3:exon6:c.C457T:p.R153C,SEMA3E:ENST00000427262.1:exon6:c.C457T:p.R153C,SEMA3E:ENST00000307792.3:exon6:c.C637T:p.R213C,	7q21.11	.
7	86567478	A	T	GGATCTGGAA,TAGTCTTTTG	exonic	KIAA1324L	stopgain	KIAA1324L:ENST00000416314.1:exon7:c.T552A:p.Y184X,KIAA1324L:ENST00000297222.6:exon4:c.T333A:p.Y111X,KIAA1324L:ENST00000444627.1:exon8:c.T1053A:p.Y351X,KIAA1324L:ENST00000450689.2:exon8:c.T1053A:p.Y351X,	7q21.12	.
7	87037472	C	T	AGGCTCAGCC,CTGAAGCACT	exonic	ABCB4	nonsynonymous SNV	ABCB4:ENST00000545634.1:exon24:c.G3160A:p.G1054R,ABCB4:ENST00000453593.1:exon23:c.G3019A:p.G1007R,ABCB4:ENST00000265723.4:exon25:c.G3160A:p.G1054R,ABCB4:ENST00000358400.3:exon24:c.G3019A:p.G1007R,ABCB4:ENST00000359206.3:exon25:c.G3160A:p.G1054R,	7q21.12	.
7	87507401	C	T	AAAAACAGAC,ATCTCTGAAA	exonic	DBF4	nonsynonymous SNV	DBF4:ENST00000413643.1:exon2:c.C80T:p.P27L,DBF4:ENST00000431138.1:exon2:c.C80T:p.P27L,DBF4:ENST00000430279.1:exon2:c.C80T:p.P27L,DBF4:ENST00000265728.1:exon2:c.C80T:p.P27L,	7q21.12	.
7	92735038	G	A	ATTGCAGACG,ATTAGCCATA	exonic	SAMD9	nonsynonymous SNV	SAMD9:ENST00000379958.2:exon3:c.C373T:p.P125S,SAMD9:ENST00000446617.1:exon2:c.C373T:p.P125S,	7q21.2	.
7	92844969	G	A	GAGGGAGGAT,AATCTGCACC	exonic	HEPACAM2	nonsynonymous SNV	HEPACAM2:ENST00000394468.2:exon3:c.C460T:p.H154Y,HEPACAM2:ENST00000440868.1:exon2:c.C424T:p.H142Y,HEPACAM2:ENST00000341723.4:exon2:c.C424T:p.H142Y,HEPACAM2:ENST00000453812.2:exon4:c.C529T:p.H177Y,	7q21.3	HEPACAM2;basechange=c.424C>G;AAchange=p.H142D;tissue=ovary
7	93055831	G	A	GCGGTTGGAG,GGCGCCTCCC	exonic	CALCR	nonsynonymous SNV	CALCR:ENST00000359558.2:exon16:c.C1364T:p.P455L,CALCR:ENST00000394441.1:exon14:c.C1262T:p.P421L,CALCR:ENST00000426151.1:exon14:c.C1271T:p.P424L,CALCR:ENST00000421592.1:exon15:c.C1310T:p.P437L,CALCR:ENST00000360249.4:exon15:c.C1310T:p.P437L,	7q21.3	.
7	93055832	G	A	CGGTTGGAGG,GCGCCTCCCC	exonic	CALCR	nonsynonymous SNV	CALCR:ENST00000359558.2:exon16:c.C1363T:p.P455S,CALCR:ENST00000426151.1:exon14:c.C1261T:p.P421S,CALCR:ENST00000394441.1:exon13:c.C1261T:p.P421S,CALCR:ENST00000421592.1:exon15:c.C1309T:p.P437S,CALCR:ENST00000360249.4:exon15:c.C1309T:p.P437S,	7q21.3	.
7	94049597	C	T	CGGGGAAGCC,TGTAAGTAAG	exonic	COL1A2	nonsynonymous SNV	COL1A2:ENST00000297268.6:exon35:c.C2132T:p.P711L,	7q21.3	.
7	94540195	C	T	GATTCTAATT,CTGGCCTCCT	exonic	PPP1R9A	nonsynonymous SNV	PPP1R9A:ENST00000456331.2:exon1:c.C770T:p.S257F,PPP1R9A:ENST00000340694.4:exon2:c.C770T:p.S257F,PPP1R9A:ENST00000433360.1:exon2:c.C770T:p.S257F,PPP1R9A:ENST00000424654.1:exon2:c.C770T:p.S257F,PPP1R9A:ENST00000433881.1:exon2:c.C770T:p.S257F,PPP1R9A:ENST0000028949 (cut)	7q21.3	.
7	95001588	C	T	CATTCAGATC,ATCAAGAAGA	exonic	PON3	nonsynonymous SNV	PON3:ENST00000427422.1:exon4:c.G264A:p.M88I,PON3:ENST00000265627.5:exon4:c.G264A:p.M88I,PON3:ENST00000451904.1:exon4:c.G264A:p.M88I,	7q21.3	.
7	95115384	A	G	GGAAAATTGA,GGCTATTTTG	exonic	ASB4	nonsynonymous SNV	ASB4:ENST00000428113.1:exon1:c.A101G:p.K34R,ASB4:ENST00000325885.5:exon1:c.A101G:p.K34R,	7q21.3	.
7	97832995	C	T	TGCATCAACT,CGAAAGCTCC	exonic	LMTK2	nonsynonymous SNV	LMTK2:ENST00000297293.5:exon12:c.C4217T:p.S1406F,	7q21.3	.
7	99693454	G	A	GTCAAACCGG,AGAGCAGTGC	exonic	MCM7	nonsynonymous SNV	MCM7:ENST00000303887.5:exon11:c.C1538T:p.S513F,MCM7:ENST00000354230.3:exon10:c.C1010T:p.S337F,	7q22.1	.
7	100075460	G	A	CGGGAGGAAG,GGCCCCAGGT	exonic	TSC22D4	nonsynonymous SNV	TSC22D4:ENST00000300181.2:exon2:c.C202T:p.P68S,	7q22.1	.
7	100282188	G	A	CAGTGACATG,AAAAGTAGCC	exonic	GIGYF1	nonsynonymous SNV	GIGYF1:ENST00000275732.5:exon13:c.C1514T:p.S505F,	7q22.1	.
7	100349760	C	T	GCCTGTCATC,CTACAGAAAA	exonic	ZAN	nonsynonymous SNV	ZAN:ENST00000538115.1:exon13:c.C2032T:p.P678S,ZAN:ENST00000542585.1:exon13:c.C2032T:p.P678S,ZAN:ENST00000546292.1:exon13:c.C2032T:p.P678S,	7q22.1	.
7	100490079	G	A	TAGCCGTGGG,CACCCCCATC	exonic	ACHE	nonsynonymous SNV	ACHE:ENST00000428317.1:exon3:c.C1429T:p.P477S,ACHE:ENST00000412389.1:exon2:c.C1429T:p.P477S,ACHE:ENST00000419336.2:exon3:c.C1165T:p.P389S,ACHE:ENST00000302913.4:exon3:c.C1429T:p.P477S,ACHE:ENST00000411582.1:exon3:c.C1429T:p.P477S,ACHE:ENST00000241069.5:exon3:c.C14 (cut)	7q22.1	.
7	100634824	G	A	TTGGGCCATA,TGAGGAATCG	exonic	MUC12	nonsynonymous SNV	MUC12:ENST00000536621.1:exon2:c.G980A:p.S327N,MUC12:ENST00000379458.4:exon5:c.G1409A:p.S470N,	7q22.1	.
7	100677378	C	A	GACACCAGCA,ACCTGTGACC	exonic	MUC17	nonsynonymous SNV	MUC17:ENST00000379439.3:exon3:c.C2681A:p.T894K,MUC17:ENST00000306151.4:exon3:c.C2681A:p.T894K,	7q22.1	MUC17;basechange=c.2681C>A;AAchange=p.T894K;tissue=haematopoietic_and_lymphoid_tissue
7	101844918	C	T	TCTGCCGAAC,CCCCGGCCCT	exonic	CUX1	nonsynonymous SNV	CUX1:ENST00000549414.2:exon17:c.C2275T:p.P759S,CUX1:ENST00000556210.1:exon16:c.C1867T:p.P623S,CUX1:ENST00000292535.7:exon18:c.C2341T:p.P781S,CUX1:ENST00000360264.3:exon18:c.C2374T:p.P792S,CUX1:ENST00000550008.2:exon16:c.C2173T:p.P725S,CUX1:ENST00000546411.2:exon18: (cut)	7q22.1	.
7	103163848	C	T	ACGCAGTTTC,CTGGATGCAT	exonic	RELN	nonsynonymous SNV	RELN:ENST00000343529.5:exon47:c.G7480A:p.G2494R,RELN:ENST00000424685.2:exon47:c.G7480A:p.G2494R,RELN:ENST00000428762.1:exon47:c.G7480A:p.G2494R,	7q22.1	.
7	103197520	G	A	GTTGTTTGAG,AAAGTAAAAT	exonic	RELN	nonsynonymous SNV	RELN:ENST00000343529.5:exon38:c.C5701T:p.P1901S,RELN:ENST00000424685.2:exon38:c.C5701T:p.P1901S,RELN:ENST00000428762.1:exon38:c.C5701T:p.P1901S,	7q22.1	.
7	104703850	C	T	TCCCCTCCTC,ATCAGTCCTT	exonic	KMT2E	nonsynonymous SNV	KMT2E:ENST00000311117.3:exon3:c.C239T:p.P80L,KMT2E:ENST00000257745.4:exon4:c.C239T:p.P80L,KMT2E:ENST00000334877.4:exon5:c.C239T:p.P80L,KMT2E:ENST00000482560.2:exon4:c.C239T:p.P80L,KMT2E:ENST00000495267.1:exon4:c.C239T:p.F (cut)	7q22.3	.
7	105254946	G	A	GGATGGGGAA,GGATGACGGC	exonic	ATXN7L1	nonsynonymous SNV	ATXN7L1:ENST00000484475.1:exon4:c.C938T:p.P313L,ATXN7L1:ENST00000388807.4:exon4:c.C815T:p.P272L,ATXN7L1:ENST00000472195.1:exon8:c.C1463T:p.P488L,ATXN7L1:ENST00000419735.3:exon10:c.C1835T:p.P612L,ATXN7L1:ENST00000477775.1:exon6:c.C1463T:p.P488L,	7q22.3	.
7	105254947	G	A	GATGGGGAAG,GATGACGGCT	exonic	ATXN7L1	nonsynonymous SNV	ATXN7L1:ENST00000484475.1:exon4:c.C937T:p.P313S,ATXN7L1:ENST00000388807.4:exon4:c.C814T:p.P272S,ATXN7L1:ENST00000472195.1:exon8:c.C1462T:p.P488S,ATXN7L1:ENST00000419735.3:exon10:c.C1834T:p.P612S,ATXN7L1:ENST00000477775.1:exon6:c.C1462T:p.P488S,	7q22.3	.
7	107399084	C	T	ACCTCCTGCC,CAGCACCTGC	exonic	CBLL1	nonsynonymous SNV	CBLL1:ENST00000420796.1:exon6:c.C787T:p.P263S,CBLL1:ENST00000222597.2:exon6:c.C934T:p.P312S,CBLL1:ENST00000440859.3:exon6:c.C937T:p.P313S,	7q22.3	.
7	113519715	C	T	TCTTTTACTT,AATATTTTTA	exonic	PPP1R3A	nonsynonymous SNV	PPP1R3A:ENST00000449795.1:exon4:c.G469A:p.E157K,PPP1R3A:ENST00000284601.3:exon4:c.G1432A:p.E478K,	7q31.1	.
7	121691554	C	T	GGAAAAGAAT,GAACTTCTTC	exonic	PTPRZ1	stopgain	PTPRZ1:ENST00000449182.1:exon23:c.C3556T:p.R1186X,PTPRZ1:ENST00000393386.2:exon24:c.C6157T:p.R2053X,	7q31.32	.
7	127255489	C	T	GGGCCGCATT,CACTGACTGC	exonic	PAX4	nonsynonymous SNV	PAX4:ENST00000338516.3:exon2:c.G110A:p.G37E,PAX4:ENST00000378740.2:exon2:c.G86A:p.G29E,PAX4:ENST00000341640.2:exon1:c.G86A:p.G29E,	7q32.1	.
7	127361428	C	T	CATCCGACCA,CGAGGCTGGA	exonic	SND1	nonsynonymous SNV	SND1:ENST00000354725.3:exon10:c.C1126T:p.P376S,	7q32.1	.
7	127965884	G	T	ATTCTCTGGA,AAGCAGCTAG	exonic	RBM28	nonsynonymous SNV	RBM28:ENST00000223073.2:exon11:c.C1190A:p.S397Y,RBM28:ENST00000415472.2:exon7:c.C767A:p.S256Y,	7q32.1	.
7	128355661	G	A	AGGTGTGGAG,AGGGACTGCT	exonic	FAM71F1	nonsynonymous SNV	FAM71F1:ENST00000471558.1:exon6:c.G166A:p.E56K,FAM71F1:ENST00000315184.5:exon6:c.G166A:p.E56K,	7q32.1	.
7	128478726	C	T	GTGGAGGTGG,CCTGGAGGAC	exonic	FLNC	nonsynonymous SNV	FLNC:ENST00000325888.8:exon6:c.C1280T:p.A427V,FLNC:ENST00000346177.6:exon6:c.C1280T:p.A427V,	7q32.1	.
7	128480637	G	A	GCGGGAGGCT,GGGATGGTGT	exonic	FLNC	nonsynonymous SNV	FLNC:ENST00000325888.8:exon10:c.G1585A:p.G529R,FLNC:ENST00000346177.6:exon10:c.G1585A:p.G529R,	7q32.1	.
7	129367107	C	T	GCTGTCGCCA,CCTGGCTGAG	exonic	NRF1	nonsynonymous SNV	NRF1:ENST00000393232.1:exon10:c.C1250T:p.T417I,NRF1:ENST00000393230.2:exon10:c.C1250T:p.T417I,NRF1:ENST00000539636.1:exon9:c.C767T:p.T256I,NRF1:ENST00000353868.4:exon9:c.C1052T:p.T351I,NRF1:ENST00000311967.2:exon10:c.C1250T:p.T417I,NRF1:ENST00000393231.3:exon9:c.C1250T: (cut)	7q32.2	.
7	129962364	G	C	CTGGGCATAT,ACAACGGCAT	exonic	CPA4	nonsynonymous SNV	CPA4:ENST00000493259.1:exon9:c.G802C:p.D268H,CPA4:ENST00000222482.4:exon11:c.G1114C:p.D372H,CPA4:ENST00000445470.2:exon10:c.G1015C:p.D339H,	7q32.2	.
7	131195021	A	G	TTGGGAGGCC,AGGTGGGCAG	exonic	PODXL	nonsynonymous SNV	PODXL:ENST00000378555.3:exon3:c.T776C:p.L259S,PODXL:ENST00000541194.1:exon3:c.T782C:p.L261S,	7q32.3	.
7	131887508	C	T	CTGGCCTGGG,CCTGGCACCA	exonic	PLXNA4	nonsynonymous SNV	PLXNA4:ENST00000321063.4:exon12:c.G2483A:p.G828D,PLXNA4:ENST00000359827.3:exon12:c.G2483A:p.G828D,	7q32.3	.
7	141734523	C	T	ACCCGTTCTA,CTTTGCGGGC	exonic	MGAM	nonsynonymous SNV	MGAM:ENST00000475668.2:exon16:c.C1841T:p.T614I,MGAM:ENST00000549489.2:exon16:c.C1841T:p.T614I,	7q34	.
7	141752612	G	T	GTCCCTTTTT,CTATTTTGTC	exonic	MGAM	nonsynonymous SNV	MGAM:ENST00000475668.2:exon26:c.G2987T:p.C996F,MGAM:ENST00000549489.2:exon26:c.G2987T:p.C996F,	7q34	.
7	141759362	C	T	TCTGATCAAT,GCATGAAGGC	exonic	MGAM	nonsynonymous SNV	MGAM:ENST00000475668.2:exon32:c.C3910T:p.R1304C,MGAM:ENST00000549489.2:exon32:c.C3910T:p.R1304C,	7q34	.
7	141785723	C	T	TCTGATCAAT,GCATGAAGGC	exonic	MGAM	nonsynonymous SNV	MGAM:ENST00000475668.2:exon55:c.C6598T:p.R2200C,	7q34	.
7	142379003	G	A	AATAAGGACG,AGCATTTTCC	exonic	TRBV25-1	nonsynonymous SNV	TRBV25-1:ENST00000390398.3:exon2:c.G271A:p.E91K,	7q34	.
7	143039049	C	T	GGTGCCGTTT,CCACACAGTC	exonic	CLCN1	nonsynonymous SNV	CLCN1:ENST00000343257.2:exon15:c.C1610T:p.S537F,	7q34	.
7	143053831	C	T	TCAGCAGGGC,CCTTGCCAGG	exonic	FAM131B	nonsynonymous SNV	FAM131B:ENST00000409222.3:exon7:c.G811A:p.G271S,FAM131B:ENST00000409578.1:exon6:c.G859A:p.G287S,FAM131B:ENST00000409346.1:exon7:c.G811A:p.G271S,FAM131B:ENST00000409408.1:exon6:c.G811A:p.G271S,FAM131B:ENST00000443739.2:exon7:c.G895A:p.G299S,	7q34	.
7	146741098	G	A	GGATTGGAAT,GAGAAGGTCG	exonic	CNTNAP2	nonsynonymous SNV	CNTNAP2:ENST00000361727.3:exon4:c.G502A:p.G168R,	7q35	.
7	149133780	C	A	TCTGGCTGTT,CCCACAGGGG	exonic	ZNF777	stopgain	ZNF777:ENST00000247930.4:exon2:c.G1225T:p.E409X,	7q36.1	.
7	149461997	G	A	GCCTGGTGGC,GACTAGGTTG	exonic	ZNF467	nonsynonymous SNV	ZNF467:ENST00000302017.3:exon5:c.C1594T:p.R532C,	7q36.1	.
7	149544892	C	T	TGGACAGTCC,TCCCGCCTCA	exonic	ZNF862	nonsynonymous SNV	ZNF862:ENST00000460379.1:exon4:c.C58T:p.L20F,ZNF862:ENST00000223210.4:exon4:c.C310T:p.L104F,	7q36.1	.
7	150558040	G	A	GGACCTGGTG,CCTGGGTGAC	exonic	AOC1	nonsynonymous SNV	AOC1:ENST00000467291.1:exon7:c.G1999A:p.A667T,AOC1:ENST00000416793.2:exon5:c.G2056A:p.A686T,AOC1:ENST00000360937.4:exon5:c.G1999A:p.A667T,AOC1:ENST00000493429.1:exon7:c.G1999A:p.A667T,	7q36.1	.
7	151372567	G	A	GCTCTGGAAG,AAGACGGGCA	exonic	PRKAG2	nonsynonymous SNV	PRKAG2:ENST00000418337.2:exon6:c.C623T:p.S208F,PRKAG2:ENST00000392801.2:exon4:c.C491T:p.S164F,PRKAG2:ENST00000287878.4:exon4:c.C623T:p.S208F,PRKAG2:ENST00000433631.2:exon2:c.C251T:p.S84F,PRKAG2:ENST00000492843.1:exon2:c.C251T:p.S84F,	7q36.1	.
7	154379625	G	A	AGAGTCCCAG,AGGCAGGACT	exonic	DPP6	nonsynonymous SNV	DPP6:ENST00000406326.1:exon6:c.G893A:p.G298E,	7q36.2	.
7	156742658	C	T	AGCTTTCGCC,GGGAGGGAGA	exonic	NOM1	nonsynonymous SNV	NOM1:ENST00000275820.3:exon1:c.C227T:p.P76L,	7q36.3	.
7	158889762	G	A	TGGCACCCTG,GAGGCTGGTG	exonic	VIPR2	nonsynonymous SNV	VIPR2:ENST00000377633.3:exon1:c.C73T:p.P25S,	7q36.3	.
8	1626517	C	T	CACTCCGAGC,CAGCACCCCC	exonic	DLGAP2	nonsynonymous SNV	DLGAP2:ENST00000421627.2:exon9:c.C2186T:p.P729L,	8p23.3	.
8	3889600	C	T	TCCAGGATTT,CACAAGTGTG	exonic	CSMD1	nonsynonymous SNV	CSMD1:ENST00000602557.1:exon4:c.G437A:p.G146E,CSMD1:ENST00000520002.1:exon4:c.G437A:p.G146E,CSMD1:ENST00000537824.1:exon4:c.G437A:p.G146E,CSMD1:ENST00000602723.1:exon4:c.G437A:p.G146E,CSMD1:ENST00000400186.3:exon4:c.G437A:p.G146E,CSMD1:ENST00000542608.1:exon4: (cut)	8p23.2	.
8	6366554	G	A	TATTTTGCCG,CTGTCCCTGT	exonic	ANGPT2	nonsynonymous SNV	ANGPT2:ENST00000325203.5:exon8:c.C1226T:p.A409V,ANGPT2:ENST00000415216.1:exon8:c.C1226T:p.A409V,ANGPT2:ENST00000629816.1:exon8:c.C1229T:p.A410V,ANGPT2:ENST00000338312.6:exon7:c.C1073T:p.A358V,	8p23.1	.
8	10470538	G	A	CCCCAGAACG,GGTCTTCCCC	exonic	RP1L1	nonsynonymous SNV	RP1L1:ENST00000382483.3:exon4:c.C1070T:p.P357L,	8p23.1	.
8	19221738	C	T	AAGACCTCCC,TTCCACCCAA	exonic	SH2D4A	nonsynonymous SNV	SH2D4A:ENST00000265807.3:exon7:c.C862T:p.L288F,SH2D4A:ENST00000519207.1:exon7:c.C862T:p.L288F,SH2D4A:ENST00000518040.1:exon6:c.C727T:p.L243F,	8p21.3	.
8	20008210	T	C	ACCAAAGAGG,TGGTGCCAAT	exonic	SLC18A1	nonsynonymous SNV	SLC18A1:ENST00000519026.1:exon10:c.A965G:p.N322S,SLC18A1:ENST00000440926.1:exon12:c.A1061G:p.N354S,SLC18A1:ENST00000517776.1:exon11:c.A1061G:p.N354S,SLC18A1:ENST00000381608.4:exon10:c.A1061G:p.N354S,SLC18A1:ENST00000276373.5:exon11:c.A1061G:p.N354S,SLC18A1:ENST0 (cut)	8p21.3	.
8	22428573	C	T	CCCCCAGCTC,CCACGTCTCC	exonic	SORBS3	nonsynonymous SNV	SORBS3:ENST00000523348.1:exon3:c.C415T:p.P139S,SORBS3:ENST00000240123.7:exon4:c.C1582T:p.P528S,SORBS3:ENST00000428103.1:exon7:c.C556T:p.P186S,SORBS3:ENST00000523965.1:exon7:c.C556T:p.P186S,	8p21.3	.
8	22926394	C	T	GGCGTTCTGT,CCCGTTGTTC	exonic	TNFRSF10B	nonsynonymous SNV	TNFRSF10B:ENST00000276431.4:exon1:c.G14A:p.G5E,TNFRSF10B:ENST00000347739.3:exon1:c.G14A:p.G5E,TNFRSF10B:ENST00000523504.1:exon1:c.G14A:p.G5E,	8p21.3	.
8	22926395	C	A	GCGTTCTGTC,CCGTTGTTCC	exonic	TNFRSF10B	stopgain	TNFRSF10B:ENST00000276431.4:exon1:c.G13T:p.G5X,TNFRSF10B:ENST00000347739.3:exon1:c.G13T:p.G5X,TNFRSF10B:ENST00000523504.1:exon1:c.G13T:p.G5X,	8p21.3	.
8	24188697	C	A	TTCCTGCAGC,GTCTCAGCTA	exonic	ADAM28	nonsynonymous SNV	ADAM28:ENST00000540823.1:exon11:c.C439A:p.R147S,ADAM28:ENST00000265769.4:exon12:c.C1138A:p.R380S,ADAM28:ENST00000437154.2:exon12:c.C1138A:p.R380S,ADAM28:ENST00000397649.3:exon12:c.C379A:p.R127S,	8p21.2	.
8	25315760	C	T	TCACCCGCCT,ATCGCGCTGC	exonic	KCTD9	nonsynonymous SNV	KCTD9:ENST00000519665.1:exon1:c.G3A:p.M1I,KCTD9:ENST00000221200.4:exon1:c.G3A:p.M1I,KCTD9:ENST00000523140.1:exon1:c.G3A:p.M1I,	8p21.2	.
8	28997989	C	T	ACCAACCTCT,CTTTCTGGTT	exonic	KIF13B	nonsynonymous SNV	KIF13B:ENST00000524189.1:exon20:c.G2480A:p.G827E,	8p12	.
8	38839233	C	T	TTTGCAATTC,TTCAGATCGA	exonic	HTRA4	nonsynonymous SNV	HTRA4:ENST00000302495.4:exon6:c.C1061T:p.P354L,	8p11.22	.
8	39634562	A	T	TGGATTCATA,TGCAGACAGC	exonic	ADAM2	nonsynonymous SNV	ADAM2:ENST00000521880.1:exon11:c.T1010A:p.I337N,ADAM2:ENST00000379853.2:exon11:c.T953A:p.I318N,ADAM2:ENST00000347580.4:exon10:c.T953A:p.I318N,ADAM2:ENST00000265708.4:exon11:c.T1010A:p.I337N,	8p11.22	.
8	39691493	C	T	ATATGGTTTC,CTTCAATTAC	exonic	ADAM2	nonsynonymous SNV	ADAM2:ENST00000521880.1:exon3:c.G158A:p.G53E,ADAM2:ENST00000379853.2:exon3:c.G158A:p.G53E,ADAM2:ENST00000347580.4:exon3:c.G158A:p.G53E,ADAM2:ENST00000265708.4:exon3:c.G158A:p.G53E,	8p11.22	ADAM2;basechange=c.158G>T;AAchange=p.G53V;tissue=lung
8	41519019	C	T	GCCCGCTTCA,TATCTGCGCC	exonic	ANK1	nonsynonymous SNV	ANK1:ENST00000396942.1:exon42:c.G5683A:p.V1895M,ANK1:ENST00000289734.7:exon42:c.G5608A:p.V1870M,ANK1:ENST00000314214.8:exon4:c.G433A:p.V145M,	8p11.21	.
8	49985397	C	T	GCCATGGCAT,CGTACCTTCC	exonic	C8orf22	nonsynonymous SNV	C8orf22:ENST00000399653.4:exon2:c.C8T:p.S3F,C8orf22:ENST00000303202.8:exon2:c.C8T:p.S3F,C8orf22:ENST00000522267.1:exon2:c.C8T:p.S3F,C8orf22:ENST00000517663.1:exon2:c.C8T:p.S3F,	8q11.21	.
8	52320918	G	A	GATTATTCTG,ACGGTGAAAA	exonic	PXDNL	nonsynonymous SNV	PXDNL:ENST00000522628.1:exon11:c.C1064T:p.S355F,PXDNL:ENST00000356297.4:exon17:c.C3266T:p.S1089F,PXDNL:ENST00000543296.1:exon17:c.C3266T:p.S1089F,	8q11.22	PXDNL_ENST00000356297;basechange=c.3266C>T;AAchange=p.S1089F;tissue=endometrium&PXDNL;basechange=c.863C>T;AAchange=p.S288F;tissue=endometrium
8	52321905	C	T	CGCGCGGATG,CGTCCCGGTA	exonic	PXDNL	nonsynonymous SNV	PXDNL:ENST00000522628.1:exon11:c.G77A:p.G26D,PXDNL:ENST00000356297.4:exon17:c.G2279A:p.G760D,PXDNL:ENST00000543296.1:exon17:c.G2279A:p.G760D,	8q11.22	.
8	52321906	C	T	GCGCGGATGC,GTCCCGGTAG	exonic	PXDNL	nonsynonymous SNV	PXDNL:ENST00000522628.1:exon11:c.G76A:p.G26S,PXDNL:ENST00000356297.4:exon17:c.G2278A:p.G760S,PXDNL:ENST00000543296.1:exon17:c.G2278A:p.G760S,	8q11.22	.
8	53062413	G	A	TGTTTTGAAT,GGGAAGAGGA	exonic	ST18	nonsynonymous SNV	ST18:ENST00000276480.7:exon18:c.C1931T:p.P644L,	8q11.23	.
8	54792077	C	T	GGCCGACCCT,GGGGGGTCGT	exonic	RGS20	nonsynonymous SNV	RGS20:ENST00000297313.3:exon2:c.C425T:p.S142L,	8q11.23	.
8	57890669	C	A	TTTACAGCCA,ACACACCATA	exonic	IMPAD1	nonsynonymous SNV	IMPAD1:ENST00000262644.4:exon3:c.G586T:p.V196L,	8q12.1	.
8	62370966	G	A	TATGACATGG,AACTTGGGCC	exonic	CLVS1	nonsynonymous SNV	CLVS1:ENST00000519846.1:exon6:c.G842A:p.G281E,CLVS1:ENST00000325897.4:exon6:c.G842A:p.G281E,CLVS1:ENST00000520712.1:exon4:c.G5A:p.G2E,CLVS1:ENST00000518592.1:exon4:c.G5A:p.G2E,CLVS1:ENST00000524095.1:exon4:c.G5A:p.G2E,	8q12.3	.
8	70585549	G	A	GTAGATTGGA,TAGGAATGTA	exonic	SLCO5A1	nonsynonymous SNV	SLCO5A1:ENST00000260126.4:exon10:c.C2102T:p.T701I,SLCO5A1:ENST00000530307.1:exon8:c.C1937T:p.T646I,	8q13.3	.
8	77765920	G	A	AAAAGATGAT,AAATAGAACA	exonic	ZFHX4	nonsynonymous SNV	ZFHX4:ENST00000518282.1:exon9:c.G6685A:p.E2229K,ZFHX4:ENST00000521891.2:exon10:c.G6763A:p.E2255K,ZFHX4:ENST00000050961.6:exon10:c.G6628A:p.E2210K,ZFHX4:ENST00000455469.2:exon10:c.G6628A:p.E2210K,	8q21.11	.
8	88885642	C	T	AGGCACAGGA,CAGGCATCAG	exonic	DCAF4L2	stopgain	DCAF4L2:ENST00000319675.3:exon1:c.G558A:p.W186X,	8q21.3	.
8	93015589	A	T	AATACTGGGA,AGAGCTGGTG	exonic	RUNX1T1	nonsynonymous SNV	RUNX1T1:ENST00000518361.1:exon2:c.T65A:p.F22Y,	8q21.3	.
8	94746454	C	T	CGGAAATGCT,CTGAGGTGGC	exonic	RBM12B	nonsynonymous SNV	RBM12B:ENST00000399300.2:exon3:c.G2185A:p.E729K,	8q22.1	.
8	95183126	C	T	ACGTAAATAT,TCCTTCCTGG	exonic	CDH17	nonsynonymous SNV	CDH17:ENST00000027335.3:exon8:c.G871A:p.D291N,CDH17:ENST00000450165.2:exon8:c.G871A:p.D291N,	8q22.1	.
8	98735147	C	T	ACCGAGCCAT,TGTAATCTTA	exonic	MTDH	nonsynonymous SNV	MTDH:ENST00000519934.1:exon10:c.C1394T:p.S465F,MTDH:ENST00000336273.3:exon11:c.C1562T:p.S521F,MTDH:ENST00000521933.1:exon5:c.C431T:p.S144F,	8q22.1	MTDH;basechange=c.1562C>T;AAchange=p.S521F;tissue=skin
8	98827607	T	A	ATATGTGCTA,GGCTACTTAC	exonic	LAPTM4B	nonsynonymous SNV	LAPTM4B:ENST00000445593.2:exon3:c.T536A:p.M179K,LAPTM4B:ENST00000521545.2:exon3:c.T263A:p.M88K,	8q22.1	.
8	104195603	C	T	AGCCCATTAC,CTCTTGCCTT	exonic	BAALC	nonsynonymous SNV	BAALC:ENST00000309391.6:exon2:c.C169T:p.P57S,	8q22.3	.
8	110467081	G	A	GGATCTGCAC,GTACTGTGGC	exonic	PKHD1L1	nonsynonymous SNV	PKHD1L1:ENST00000378402.5:exon45:c.G6874A:p.G2292S,	8q23.1	.
8	110477367	G	A	AGATGTGGGG,TTGGAGTGCA	exonic	PKHD1L1	nonsynonymous SNV	PKHD1L1:ENST00000378402.5:exon49:c.G8306A:p.G2769D,	8q23.1	.
8	110489463	G	A	GTGTCAGGAA,AAATGACCTT	exonic	PKHD1L1	nonsynonymous SNV	PKHD1L1:ENST00000378402.5:exon53:c.G8927A:p.R2976K,	8q23.1	.
8	113303802	C	T	ACTGAAGATC,AAATAAAAAA	exonic	CSMD3	nonsynonymous SNV	CSMD3:ENST00000297405.5:exon56:c.G8911A:p.G2971R,CSMD3:ENST00000352409.3:exon54:c.G8701A:p.G2901R,CSMD3:ENST00000343508.3:exon57:c.G8791A:p.G2931R,CSMD3:ENST00000455883.2:exon54:c.G8404A:p.G2802R,CSMD3:ENST00000339701.3:exon41:c.G6721A:p.G2241R,	8q23.3	.
8	120816152	G	A	GAGAAAACAT,AGCACCTCTC	exonic	TAF2	nonsynonymous SNV	TAF2:ENST00000378164.2:exon6:c.C526T:p.H176Y,	8q24.12	.
8	125498438	C	T	TTTACTTCTT,CTTGATTCTC	exonic	RNF139	nonsynonymous SNV	RNF139:ENST00000303545.3:exon2:c.C548T:p.S183F,RNF139:ENST00000517684.2:exon2:c.C167T:p.S56F,	8q24.13	.
8	125498587	C	T	GAAGAGGATT,GTTTCCCAGA	exonic	RNF139	nonsynonymous SNV	RNF139:ENST00000303545.3:exon2:c.C697T:p.R233C,	8q24.13	.
8	125534420	C	T	GACAGCTCTC,CTATTACATC	exonic	TATDN1	nonsynonymous SNV	TATDN1:ENST00000605953.1:exon3:c.G110A:p.G37E,TATDN1:ENST00000520921.1:exon2:c.G44A:p.G15E,TATDN1:ENST00000520938.1:exon3:c.G62A:p.G21E,TATDN1:ENST00000523214.1:exon3:c.G110A:p.G37E,TATDN1:ENST00000276692.6:exon3:c.G110A:p.G37E,TATDN1:ENST00000522927.1:exon2:c.G (cut)	8q24.13	.
8	125555480	C	T	TTCCCTGACT,TCCTGGGGGC	exonic	NDUFB9	nonsynonymous SNV	NDUFB9:ENST00000522532.1:exon2:c.C74T:p.S25F,NDUFB9:ENST00000276689.3:exon2:c.C254T:p.S85F,NDUFB9:ENST00000517367.1:exon2:c.C221T:p.S74F,NDUFB9:ENST00000518008.1:exon2:c.C254T:p.S85F,	8q24.13	.
8	139155261	C	T	TTACCTAATT,GGGATATGGA	exonic	FAM135B	nonsynonymous SNV	FAM135B:ENST00000395297.1:exon16:c.G3632A:p.R1211Q,	8q24.23	LOC51059;basechange=c.3632G>A;AAchange=p.R1211Q;tissue=lung,large_intestine&FAM135B;basechange=c.3632G>A;AAchange=p.R1211Q;tissue=lung,large_intestine
8	139158223	G	A	CAGACATTAG,AAGTCCAGTT	exonic	FAM135B	nonsynonymous SNV	FAM135B:ENST00000276425.6:exon15:c.C3415T:p.P1139S,FAM135B:ENST00000467365.1:exon13:c.C1345T:p.P449S,	8q24.23	.
8	139164562	C	T	GGCATGTCTT,GAACAAACGG	exonic	FAM135B	nonsynonymous SNV	FAM135B:ENST00000276425.6:exon14:c.G2156A:p.R719Q,FAM135B:ENST00000395297.1:exon13:c.G2156A:p.R719Q,	8q24.23	.
8	139628262	T	G	CTGGTAACCC,TCCTGACCAG	exonic	COL22A1	nonsynonymous SNV	COL22A1:ENST00000435777.1:exon64:c.A3846C:p.E1282D,COL22A1:ENST00000303045.6:exon55:c.A3906C:p.E1302D,	8q24.23	.
8	139661993	G	A	GAGGCCTGGG,GGCCAGGAGG	exonic	COL22A1	nonsynonymous SNV	COL22A1:ENST00000435777.1:exon45:c.C3302T:p.T1101I,COL22A1:ENST00000303045.6:exon46:c.C3362T:p.T1121I,	8q24.23	.
8	142264814	G	A	CCCCGAGCAA,CCGTGTGCCC	exonic	RP11-10J21.3	nonsynonymous SNV	RP11-10J21.3:ENST00000518520.1:exon1:c.G91A:p.A31T,	8q24.3	.
8	144397919	G	A	CCATCCCCTT,GGCTTTGTGC	exonic	TOP1MT	stopgain	TOP1MT:ENST00000329245.4:exon12:c.C1231T:p.Q411X,TOP1MT:ENST00000519148.1:exon12:c.C1237T:p.Q413X,TOP1MT:ENST00000523676.1:exon13:c.C1237T:p.Q413X,TOP1MT:ENST00000521193.1:exon13:c.C1237T:p.Q413X,	8q24.3	.
8	145659626	T	A	GCCCAAGCCC,GGAGCTCCAC	exonic	TONSL	nonsynonymous SNV	TONSL:ENST00000409379.3:exon21:c.A3122T:p.Q1041L,	8q24.3	.
8	145677808	G	A	ATCTCCTTGC,GTGGCTCTGG	exonic	CYHR1	nonsynonymous SNV	CYHR1:ENST00000526887.1:exon4:c.C478T:p.R160C,CYHR1:ENST00000438911.2:exon4:c.C631T:p.R211C,CYHR1:ENST00000530374.1:exon3:c.C757T:p.R253C,	8q24.3	.
8	145694941	C	T	GGAGCCTGGC,CAGGGGGCAC	exonic	KIFC2	nonsynonymous SNV	KIFC2:ENST00000301331.5:exon4:c.C535T:p.P179S,KIFC2:ENST00000301332.2:exon12:c.C1291T:p.P431S,	8q24.3	.
8	146032954	C	T	AAGCAAAGCT,CATCCTGCTG	exonic	ZNF517	nonsynonymous SNV	ZNF517:ENST00000359971.3:exon5:c.C653T:p.S218F,ZNF517:ENST00000531720.1:exon4:c.C653T:p.S218F,ZNF517:ENST00000533965.1:exon5:c.C653T:p.S218F,	8q24.3	.
9	2077744	C	T	ACAGTCTGCC,TCCTAATTAA	exonic	SMARCA2	nonsynonymous SNV	SMARCA2:ENST00000382203.1:exon14:c.C2152T:p.L718F,SMARCA2:ENST00000382194.1:exon14:c.C2152T:p.L718F,SMARCA2:ENST00000357248.2:exon14:c.C2152T:p.L718F,SMARCA2:ENST00000349721.2:exon14:c.C2152T:p.L718F,	9p24.3	.
9	2643444	C	T	CAGCGAAATC,AGTGCGGCTC	exonic	VLDLR	stopgain	VLDLR:ENST00000382100.3:exon5:c.C733T:p.Q245X,VLDLR:ENST00000382099.2:exon5:c.C733T:p.Q245X,	9p24.2	.
9	2717929	G	A	CGGCGAGGAG,AGGACCAGTG	exonic	KCNV2	nonsynonymous SNV	KCNV2:ENST00000382082.3:exon1:c.G190A:p.E64K,	9p24.2	.
9	5773004	T	C	TACACAGTCT,CAGAGGTAGA	exonic	KIAA1432	nonsynonymous SNV	KIAA1432:ENST00000414202.2:exon23:c.T3907C:p.S1303P,KIAA1432:ENST00000449720.2:exon23:c.T3559C:p.S1187P,KIAA1432:ENST00000418622.3:exon24:c.T3670C:p.S1224P,KIAA1432:ENST00000490816.1:exon13:c.T364C:p.S122P,	9p24.1	.
9	6328899	G	A	GTCCACCATG,GCACTCTCAT	exonic	TPD52L3	nonsynonymous SNV	TPD52L3:ENST00000381428.1:exon1:c.G304A:p.G102S,TPD52L3:ENST00000344545.5:exon1:c.G304A:p.G102S,TPD52L3:ENST00000314556.3:exon1:c.G304A:p.G102S,	9p24.1	.
9	8518246	C	T	GGGACTTAGT,CAGCGACACT	exonic	PTPRD	nonsynonymous SNV	PTPRD:ENST00000540109.1:exon10:c.G1145T:p.G382V,PTPRD:ENST00000397606.3:exon8:c.G1145T:p.G382V,PTPRD:ENST00000537002.1:exon8:c.G1136T:p.G379V,PTPRD:ENST00000486161.1:exon10:c.G1145T:p.G382V,PTPRD:ENST00000358503. (cut)	9p24.1	PTPRD;basechange=c.1145G>T;AAchange=p.G382V;tissue=lung&PTPRD_ENST00000346816;basechange=c.1145G>T;AAchange=p.G382V;tissue=lung
9	13224491	G	A	TAATAAAAAC,ATTCATTTTG	exonic	MPDZ	nonsynonymous SNV	MPDZ:ENST00000536827.1:exon3:c.C275T:p.S92L,MPDZ:ENST00000447879.1:exon3:c.C275T:p.S92L,MPDZ:ENST00000381015.4:exon3:c.C275T:p.S92L,MPDZ:ENST00000541718.1:exon4:c.C275T:p.S92L,MPDZ:ENST00000381022.2:exon3:c.C275T:p.S92L,MPDZ:ENST00000319217.7:exon4:c.C275T:p.S92L,M (cut)	9p23	.
9	14792797	C	T	TAAATCTTCT,CCTGGGTGAG	exonic	FREM1	nonsynonymous SNV	FREM1:ENST00000380880.3:exon22:c.G3925A:p.E1309K,FREM1:ENST00000380881.4:exon23:c.G3928A:p.E1310K,	9p22.3	.
9	19121144	C	T	AGTCACAGCG,CTTTGGCATT	exonic	PLIN2	nonsynonymous SNV	PLIN2:ENST00000411567.1:exon6:c.G329A:p.G110D,PLIN2:ENST00000276914.2:exon5:c.G329A:p.G110D,PLIN2:ENST00000434144.1:exon6:c.G329A:p.G110D,	9p22.1	.
9	21350654	G	A	ATGGAGGACA,AGATGGCTTC	exonic	IFNA6	nonsynonymous SNV	IFNA6:ENST00000380210.1:exon1:c.C233T:p.S78F,	9p21.3	IFNA6;basechange=c.233C>T;AAchange=p.S78F;tissue=lung
9	21385136	C	T	TTGCCAAACT,CTCCTGGGGA	exonic	IFNA2	nonsynonymous SNV	IFNA2:ENST00000380206.2:exon1:c.G193A:p.E65K,	9p21.3	.

9	134340360	G	A	GTGTAAGCAG,CACGAAAGGC	exonic	PRRC2B	nonsynonymous SNV	PRRC2B:ENST00000357304.4:exon11:c.G1615A:p.A539T,PRRC2B:ENST00000422467.1:exon2:c.G235A:p.A79T,PRRC2B:ENST00000405995.1:exon12:c.G1615A:p.A539T,PRRC2B:ENST00000458550.1:exon11:c.G1615A:p.A539T,	9q34.13	.
9	135781194	G	A	ACTCTCGTCG,AGGTGGAATT	exonic	TSC1	nonsynonymous SNV	TSC1:ENST00000545250.1:exon12:c.C1618T:p.P540S,TSC1:ENST00000298552.3:exon15:c.C1771T:p.P591S,TSC1:ENST00000440111.2:exon13:c.C1771T:p.P591S,	9q34.13	.
9	136199568	G	A	TTTCTCCAAG,CGGCAGGGGA	exonic	SURF6	nonsynonymous SNV	SURF6:ENST00000372022.4:exon4:c.C422T:p.A141V,	9q34.2	.
9	136578226	T	C	ATCAGGGGCT,GTGGTCGGGC	exonic	SARDH	nonsynonymous SNV	SARDH:ENST00000439388.1:exon9:c.A1171G:p.K391E,SARDH:ENST00000422262.2:exon8:c.A667G:p.K223E,SARDH:ENST00000371872.4:exon9:c.A1171G:p.K391E,SARDH:ENST00000427237.2:exon9:c.A1171G:p.K391E,	9q34.2	.
9	137686953	G	A	CCAGGACCGC,GGGGCAGCGA	exonic	COL5A1	nonsynonymous SNV	COL5A1:ENST00000371817.3:exon33:c.G2726A:p.R909Q,	9q34.3	.
9	139008666	G	A	AGGCGCGCAC,GGCCCGCAGG	exonic	C9orf69	nonsynonymous SNV	C9orf69:ENST00000561457.1:exon2:c.C155T:p.P52L,	9q34.3	C9orf69;basechange=c.81C>T;AAchange=p.P27P;tissue=endometrium&C9orf69_ENST00000418388;basechange=c.81C>T;AAchange=p.P27P;tissue=endometrium
9	140379053	G	A	GGCGCTGAGG,CATGCTCCAG	exonic	PNPLA7	nonsynonymous SNV	PNPLA7:ENST00000371457.1:exon10:c.C1076T:p.A359V,PNPLA7:ENST00000434090.1:exon20:c.C2231T:p.A744V,PNPLA7:ENST00000277531.4:exon20:c.C2258T:p.A753V,PNPLA7:ENST00000406427.1:exon21:c.C2333T:p.A778V,	9q34.3	.
10	7214024	C	T	GGCACCTCCG,CGACGAGGTG	exonic	SFMBT2	nonsynonymous SNV	SFMBT2:ENST00000397167.1:exon19:c.G2248A:p.A750T,SFMBT2:ENST00000361972.4:exon19:c.G2248A:p.A750T,	10p14	.
10	8006161	C	T	AATGCTTTCT,CAGTCCATGT	exonic	TAF3	nonsynonymous SNV	TAF3:ENST00000344293.5:exon3:c.C688T:p.P230S,	10p14	.
10	8006162	C	T	ATGCTTTCTC,AGTCCATGTA	exonic	TAF3	nonsynonymous SNV	TAF3:ENST00000344293.5:exon3:c.C689T:p.P230L,	10p14	.
10	8007707	T	G	CATGAAAAAG,AAGCAGTTTC	splicing	TAF3(ENST0	.	.	10p14	.
10	13522976	A	G	CCCATTGGGT,AGGAGTTAGC	exonic	BEND7	nonsynonymous SNV	BEND7:ENST00000341083.3:exon6:c.T830C:p.L277S,BEND7:ENST00000378605.3:exon5:c.T869C:p.L290S,BEND7:ENST00000440282.1:exon1:c.T98C:p.L33S,BEND7:ENST00000396900.2:exon6:c.T986C:p.L329S,BEND7:ENST00000396898.2:exon6:c.T1025C:p.L342S,	10p13	.
10	15559216	G	A	TCCTCCTGAG,AGGTCTGGCT	exonic	ITGA8	nonsynonymous SNV	ITGA8:ENST00000378076.3:exon30:c.C3133T:p.P1045S,	10p13	.
10	15614218	C	T	ACCTTTATAT,CTGTGGATTG	exonic	ITGA8	nonsynonymous SNV	ITGA8:ENST00000378076.3:exon25:c.G2629A:p.D877N,	10p13	.
10	25887442	C	A	AAACTCAAAT,CTGCGGAGGA	exonic	GPR158	nonsynonymous SNV	GPR158:ENST00000376351.3:exon11:c.C2887A:p.P963T,	10p12.1	GPR158;basechange=c.2887C>T;AAchange=p.P963S;tissue=large_intestine
10	26434423	G	A	AGACCCAAAA,AATGGAACTT	exonic	MYO3A	nonsynonymous SNV	MYO3A:ENST00000265944.5:exon22:c.G2465A:p.R822K,	10p12.1	.
10	34673142	T	A	TGTTCAGCTT,ACCACCTTTC	exonic	PARD3	stopgain	PARD3:ENST00000374773.1:exon8:c.A931T:p.K311X,PARD3:ENST00000350537.4:exon8:c.A931T:p.K311X,PARD3:ENST00000374788.3:exon8:c.A931T:p.K311X,PARD3:ENST00000374789.3:exon8:c.A931T:p.K311X,PARD3:ENST00000346874.4:exon8:c.A931T:p.K311X,PARD3:ENST00000340077.5:exon8:c.A93	10p11.21	.
10	43088156	G	A	CATTCATAGG,CTTTTCCCCT	exonic	ZNF33B	nonsynonymous SNV	ZNF33B:ENST00000359467.3:exon5:c.C2242T:p.P748S,	10q11.21	.
10	44104101	C	T	GGCCTTTACC,GGATTGAGTG	exonic	ZNF485	nonsynonymous SNV	ZNF485:ENST00000361807.3:exon3:c.C64T:p.R22W,ZNF485:ENST00000374435.3:exon3:c.C64T:p.R22W,ZNF485:ENST00000430885.1:exon3:c.C64T:p.R22W,	10q11.21	.
10	45799011	C	T	CTTGCTCTTC,CTGCGCTGTA	exonic	OR13A1	nonsynonymous SNV	OR13A1:ENST00000553795.1:exon4:c.G860A:p.G287E,OR13A1:ENST00000374401.2:exon2:c.G860A:p.G287E,OR13A1:ENST00000536058.1:exon3:c.G860A:p.G287E,	10q11.21	.
10	49400782	C	T	TTGAAGTTAT,CATTGATGTA	exonic	FRMPD2	nonsynonymous SNV	FRMPD2:ENST00000305531.3:exon14:c.G2035A:p.D679N,FRMPD2:ENST00000407470.4:exon15:c.G2014A:p.D672N,FRMPD2:ENST00000374201.3:exon16:c.G2110A:p.D704N,	10q11.22	FRMPD2;basechange=c.2110G>T;AAchange=p.D704Y;tissue=large_intestine
10	50081648	C	T	CCCTGTTCCC,ATGGGAACTC	exonic	WDFY4	nonsynonymous SNV	WDFY4:ENST00000325239.5:exon40:c.C6854T:p.P2285L,	10q11.23	.
10	52610471	C	T	AGCATTATTT,CTTTTTTGAG	exonic	A1CF	nonsynonymous SNV	A1CF:ENST00000395489.2:exon5:c.G32A:p.G11E,A1CF:ENST00000373995.3:exon3:c.G77A:p.G26E,	10q11.23	.
10	54530540	C	T	GCCTCTGAGC,CTTGGCCTGT	exonic	MBL2	nonsynonymous SNV	MBL2:ENST00000373968.3:exon2:c.G194A:p.G65E,	10q21.1	.
10	54530541	C	T	CCTCTGAGCC,TTGGCCTGTT	exonic	MBL2	nonsynonymous SNV	MBL2:ENST00000373968.3:exon2:c.G193A:p.G65R,	10q21.1	MBL2;basechange=c.193G>T;AAchange=p.G65W;tissue=haematopoietic_and_lymphoid_tissue
10	55569266	C	T	ACTAGGCTCT,TAATTTCAAC	exonic	PCDH15	nonsynonymous SNV	PCDH15:ENST00000409834.1:exon29:c.G3427A:p.E1143K,PCDH15:ENST00000395438.1:exon36:c.G4594A:p.E1532K,PCDH15:ENST00000395440.1:exon12:c.G1346A:p.R449K,PCDH15:ENST00000395445.1:exon36:c.G4544A:p.R1515K,PCDH15:ENST00000395442.1:exon11:c.G1139A:p.R380K,PCDH15:ENST0	10q21.1	.
10	55591154	C	T	AACAAGGCCC,TTCTGTGTAT	exonic	PCDH15	nonsynonymous SNV	PCDH15:ENST00000409834.1:exon23:c.G2956A:p.G986R,PCDH15:ENST00000373965.2:exon31:c.G4144A:p.G1382R,PCDH15:ENST00000395433.1:exon29:c.G4057A:p.G1353R,PCDH15:ENST00000395438.1:exon30:c.G4123A:p.G1375R,PCDH15:ENST00000395445.1:exon31:c.G4144A:p.G1382R,PCDH15:ENS	10q21.1	.
10	70940136	C	T	GCCCTTCGTC,CCACTTTGGG	exonic	SUPV3L1	nonsynonymous SNV	SUPV3L1:ENST00000359655.4:exon1:c.C89T:p.P30L,SUPV3L1:ENST00000422378.1:exon1:c.C89T:p.P30L,	10q22.1	.
10	71654452	G	A	ACAAAAAGGA,AAAAGGTAAG	exonic	COL13A1	nonsynonymous SNV	COL13A1:ENST00000522165.1:exon11:c.G625A:p.E209K,COL13A1:ENST00000354547.3:exon11:c.G625A:p.E209K,COL13A1:ENST00000398974.3:exon9:c.G589A:p.E197K,COL13A1:ENST00000357811.3:exon11:c.G625A:p.E209K,COL13A1:ENST00000479733.1:exon12:c.G652A:p.E218K,COL13A1:ENST000003	10q22.1	.
10	71851565	C	T	AGAATTCACC,CGAACTGCTG	exonic	H2AFY2	nonsynonymous SNV	H2AFY2:ENST00000373255.4:exon4:c.C332T:p.P111L,	10q22.1	.
10	72513662	G	A	AACCCACAAG,TCATGCCGGC	exonic	ADAMTS14	nonsynonymous SNV	ADAMTS14:ENST00000373207.1:exon19:c.G2836A:p.V946I,ADAMTS14:ENST00000373208.1:exon19:c.G2845A:p.V949I,	10q22.1	.
10	73485264	G	A	GGCCCCATGC,GAGCTCCGTC	exonic	CDH23	nonsynonymous SNV	CDH23:ENST00000224721.6:exon29:c.G3581A:p.R1194Q,	10q22.1	.
10	75408158	C	T	GACTGCAGGC,TTCCCCGTTG	exonic	SYNPO2L	nonsynonymous SNV	SYNPO2L:ENST00000372873.4:exon2:c.G580A:p.G194S,SYNPO2L:ENST00000394810.2:exon4:c.G1252A:p.G418S,	10q22.2	.
10	75520600	C	T	GATGCCATCC,AAGCCCTGTA	exonic	SEC24C	nonsynonymous SNV	SEC24C:ENST00000411652.2:exon5:c.C554T:p.P185L,SEC24C:ENST00000339365.2:exon7:c.C980T:p.P327L,SEC24C:ENST00000546025.1:exon4:c.C554T:p.P185L,SEC24C:ENST00000465076.1:exon6:c.C980T:p.P327L,SEC24C:ENST00000345254.4:exon6:c.C980T:p.P327L,	10q22.2	.
10	75552204	C	T	GAGGCCAGCA,CTTCGGGGGA	exonic	ZSWIM8	nonsynonymous SNV	ZSWIM8:ENST00000492395.1:exon2:c.C764T:p.T255I,ZSWIM8:ENST00000603114.1:exon10:c.C1907T:p.T636I,ZSWIM8:ENST00000398706.2:exon10:c.C1907T:p.T636I,ZSWIM8:ENST00000605216.1:exon10:c.C1907T:p.T636I,ZSWIM8:ENST00000433366.2:exon10:c.C1907T:p.T636I,ZSWIM8:ENST00000604729.1	10q22.2	.
10	81372130	C	T	GCCTGGAGCC,CTGGTATCCC	exonic	SFTPA1	nonsynonymous SNV	SFTPA1:ENST00000372313.5:exon2:c.C58T:p.P20S,SFTPA1:ENST00000372308.3:exon4:c.C235T:p.P79S,SFTPA1:ENST00000398636.3:exon4:c.C235T:p.P79S,SFTPA1:ENST00000419470.2:exon4:c.C280T:p.P94S,SFTPA1:ENST00000439264.1:exon4:c.C235T:p.P79S,SFTPA1:ENST00000428376.2:exon3:c.C23	10q22.3	.
10	86131766	C	T	AATGGTTCAT,CCTCTCTACT	exonic	CCSER2	nonsynonymous SNV	CCSER2:ENST00000372088.2:exon2:c.C958T:p.P320S,CCSER2:ENST00000359979.4:exon2:c.C958T:p.P320S,CCSER2:ENST00000224756.8:exon2:c.C958T:p.P320S,	10q23.1	.
10	87614297	G	A	GTAGTGCCAA,GATTTCAAAC	exonic	GRID1	nonsynonymous SNV	GRID1:ENST00000464741.1:exon8:c.C1189T:p.L397F,GRID1:ENST00000327946.7:exon8:c.C1189T:p.L397F,	10q23.1	.
10	95079727	G	A	GATCTGTAAT,GACATCTGTT	exonic	MYOF	nonsynonymous SNV	MYOF:ENST00000371502.4:exon49:c.C5470T:p.H1824Y,MYOF:ENST00000358334.5:exon48:c.C5461T:p.H1821Y,MYOF:ENST00000371501.4:exon49:c.C5500T:p.H1834Y,MYOF:ENST00000359263.4:exon49:c.C5500T:p.H1834Y,	10q23.33	.
10	96443734	C	T	ATGAGCAAAT,CTTAACCAAT	exonic	CYP2C18	nonsynonymous SNV	CYP2C18:ENST00000285979.6:exon1:c.C158T:p.S53F,CYP2C18:ENST00000339022.5:exon1:c.C158T:p.S53F,	10q23.33	.
10	96493082	C	T	TCCCTGACTT,TGTGCTGCAC	exonic	CYP2C18	nonsynonymous SNV	CYP2C18:ENST00000285979.6:exon8:c.C1178T:p.S393F,CYP2C18:ENST00000339022.5:exon7:c.C1001T:p.S334F,	10q23.33	.
10	96698527	C	T	AGGAAAACTC,CTCCTGGCCC	exonic	CYP2C9	nonsynonymous SNV	CYP2C9:ENST00000260682.6:exon1:c.C88T:p.P30S,	10q23.33	.
10	96741038	G	A	TGTGGTGCAC,AGGTCCAGAG	exonic	CYP2C9	nonsynonymous SNV	CYP2C9:ENST00000260682.6:exon7:c.G1060A:p.E354K,	10q23.33	.
10	96745844	C	T	CAAAGAATTT,CCAACCCAGA	exonic	CYP2C9	nonsynonymous SNV	CYP2C9:ENST00000260682.6:exon8:c.C1204T:p.P402S,	10q23.33	CYP2C9;basechange=c.1204C>T;AAchange=p.P402S;tissue=lung
10	98820505	G	A	GGAGCCGGAG,ACAGGGTGCA	exonic	SLIT1	nonsynonymous SNV	SLIT1:ENST00000314867.5:exon9:c.C782T:p.S261F,SLIT1:ENST00000371041.3:exon9:c.C833T:p.S278F,SLIT1:ENST00000266058.4:exon9:c.C833T:p.S278F,SLIT1:ENST00000371070.4:exon9:c.C833T:p.S278F,	10q24.1	.
10	100157116	G	A	CTTCAGCAGG,GCTTGAGGGT	exonic	PYROXD2	nonsynonymous SNV	PYROXD2:ENST00000370575.4:exon6:c.C611T:p.P204L,	10q24.2	.
10	104230472	C	T	CGGGTGCAGG,CCTGGGCTGG	exonic	TMEM180	nonsynonymous SNV	TMEM180:ENST00000238936.4:exon5:c.C302T:p.A101V,	10q24.32	.
10	104934631	G	A	TAGGCTTCTC,ACGATACTTT	exonic	NT5C2	stopgain	NT5C2:ENST00000452156.1:exon2:c.C85T:p.R29X,NT5C2:ENST00000470299.1:exon2:c.C85T:p.R29X,NT5C2:ENST00000343289.5:exon2:c.C85T:p.R29X,NT5C2:ENST00000461461.1:exon2:c.C85T:p.R29X,NT5C2:ENST00000404739.3:exon1:c.C85T:p.R29X,	10q24.33	.
10	105762533	G	A	GAAATTGGGG,AAGACGACAA	exonic	SLK	nonsynonymous SNV	SLK:ENST00000369755.3:exon9:c.G1597A:p.E533K,SLK:ENST00000335753.4:exon9:c.G1597A:p.E533K,	10q24.33	.
10	108589424	C	T	GTTCCATAAT,GGTTGACCTA	exonic	SORCS1	nonsynonymous SNV	SORCS1:ENST00000344440.6:exon3:c.G634A:p.D212N,SORCS1:ENST00000263054.6:exon3:c.G634A:p.D212N,	10q25.1	.
10	112572208	G	A	TGCCAGGAGG,AGGAAGAGCG	exonic	RBM20	nonsynonymous SNV	RBM20:ENST00000369519.3:exon9:c.G2053A:p.E685K,	10q25.2	.
10	115341718	G	A	TGAGATAGCA,AGAGGAAGAT	exonic	HABP2	nonsynonymous SNV	HABP2:ENST00000351270.3:exon9:c.G922A:p.E308K,HABP2:ENST00000542051.1:exon9:c.G844A:p.E282K,	10q25.3	.
10	115349436	G	A	TAAGCTCCCT,GAGACCCAGG	exonic	NRAP	stopgain	NRAP:ENST00000369358.4:exon41:c.C5101T:p.Q1701X,NRAP:ENST00000359988.3:exon41:c.C5077T:p.Q1693X,NRAP:ENST00000360478.3:exon40:c.C4972T:p.Q1658X,NRAP:ENST00000369360.3:exon40:c.C4996T:p.Q1666X,	10q25.3	.
10	118383474	G	A	GTCTGCTACG,ACAACTTGGC	exonic	PNLIPRP2	nonsynonymous SNV	PNLIPRP2:ENST00000537242.1:exon4:c.G68A:p.G23E,	10q25.3	.
10	124600760	T	G	GGTGCAGTTC,CACTGGGATT	exonic	CUZD1	nonsynonymous SNV	CUZD1:ENST00000392790.1:exon2:c.A167C:p.E56A,CUZD1:ENST00000545804.1:exon4:c.A167C:p.E56A,CUZD1:ENST00000368904.1:exon4:c.A167C:p.E56A,	10q26.13	.
10	126138464	T	C	AAAGAAATCT,GTGGTGGGAG	exonic	NKX1-2	nonsynonymous SNV	NKX1-2:ENST00000440536.2:exon1:c.A49G:p.K17E,NKX1-2:ENST00000451024.3:exon1:c.A49G:p.K17E,	10q26.13	.
10	126138465	G	T	AAGAAATCTT,TGGTGGGAGG	exonic	NKX1-2	nonsynonymous SNV	NKX1-2:ENST00000440536.2:exon1:c.C48A:p.H16Q,NKX1-2:ENST00000451024.3:exon1:c.C48A:p.H16Q,	10q26.13	.
10	128192855	C	T	GCCATCTACT,CATCTGCTGT	exonic	C10orf90	nonsynonymous SNV	C10orf90:ENST00000454341.1:exon3:c.G914A:p.G305E,C10orf90:ENST00000544758.1:exon4:c.G1205A:p.G402E,C10orf90:ENST00000356858.3:exon3:c.G773A:p.G258E,C10orf90:ENST00000392694.1:exon3:c.G773A:p.G258E,C10orf90:ENST00000432642.1:exon3:c.G914A:p.G305E,C10orf90:ENST00000284694	10q26.2	.
10	134218584	C	T	CAGCCCCGGA,CCCCAGCCGC	exonic	PWWP2B	nonsynonymous SNV	PWWP2B:ENST00000305233.5:exon2:c.C580T:p.P194S,PWWP2B:ENST00000368609.4:exon2:c.C580T:p.P194S,	10q26.3	.
10	134648807	C	T	GAGGGCCTCA,CGAGCCTATG	splicing	TTC40(ENST	.	.	10q26.3	.
10	134886456	C	T	CAACAAATTA,CAGAGGCCGC	exonic	GPR123	nonsynonymous SNV	GPR123:ENST00000607359.1:exon3:c.C490T:p.P164S,	10q26.3	.
10	135439000	C	T	CCTGGAACGT,CCCTATGGTG	exonic	FRG2B	nonsynonymous SNV	FRG2B:ENST00000443774.1:exon4:c.G443A:p.G148E,FRG2B:ENST00000425520.1:exon4:c.G440A:p.G147E,	10q26.3	.
11	500632	C	T	CACCGTGCTT,CGTGAGGCCA	exonic	RNH1	nonsynonymous SNV	RNH1:ENST00000397604.3:exon3:c.G124A:p.E42K,RNH1:ENST00000524464.1:exon3:c.G124A:p.E42K,RNH1:ENST00000529368.1:exon3:c.G124A:p.E42K,RNH1:ENST00000397614.1:exon3:c.G124A:p.E42K,RNH1:ENST00000531149.1:exon3:c.G124A:p.E42K,RNH1:ENST00000356187.5:exon3:c.G124A:p.E42K,R	11p15.5	.
11	654637	A	G	GACTTTGGGC,GCCGAGGCGG	exonic	DEAF1	nonsynonymous SNV	DEAF1:ENST00000338675.6:exon9:c.T1256C:p.L419P,	11p15.5	.
11	793551	G	A	AGCTGATGTC,GAAGAAGTCG	exonic	SLC25A22	stopgain	SLC25A22:ENST00000531437.1:exon5:c.C259T:p.R87X,SLC25A22:ENST00000531214.1:exon5:c.C271T:p.R91X,SLC25A22:ENST00000528936.1:exon5:c.C271T:p.R91X,SLC25A22:ENST00000320230.5:exon5:c.C271T:p.R91X,SLC25A22:ENST00000481290.1:exon4:c.C346T:p.R116X,SLC25A22:ENST000005286	11p15.5	.
11	819816	C	G	CGCGAGCACG,GCCCTTCCTG	exonic	PNPLA2	nonsynonymous SNV	PNPLA2:ENST00000336615.4:exon2:c.C98G:p.A33G,	11p15.5	.
11	1606221	C	T	CCACAGCCCC,CTTGCAGCCT	exonic	KRTAP5-1	nonsynonymous SNV	KRTAP5-1:ENST00000382171.2:exon1:c.G259A:p.G87R,	11p15.5	.
11	1629300	C	T	CCCTTGGAAC,CCCACAGGAG	exonic	KRTAP5-3	nonsynonymous SNV	KRTAP5-3:ENST00000399685.1:exon1:c.G316A:p.G106S,	11p15.5	.
11	3797138	G	A	GTCCCAGTAG,AGCAGCTGTA	exonic	NUP98	nonsynonymous SNV	NUP98:ENST00000397007.4:exon5:c.C469T:p.P157S,NUP98:ENST00000397004.4:exon5:c.C469T:p.P157S,NUP98:ENST00000359171.4:exon5:c.C469T:p.P157S,NUP98:ENST00000532475.1:exon5:c.C358T:p.P120S,NUP98:ENST00000355260.3:exon5:c.C469T:p.P157S,NUP98:ENST00000324932.7:exon5:c.C46	11p15.4	.
11	4076824	G	A	TCAGTATGAG,AGACCTTCCG	exonic	STIM1	nonsynonymous SNV	STIM1:ENST00000530554.1:exon4:c.G232A:p.E78K,STIM1:ENST00000532610.1:exon4:c.G232A:p.E78K,STIM1:ENST00000525055.1:exon5:c.G232A:p.E78K,STIM1:ENST00000525403.1:exon4:c.G232A:p.E78K,STIM1:ENST00000532919.1:exon4:c.G232A:p.E78K,STIM1:ENST00000528656.1:exon4:c.G232A:p.E7	11p15.4	.
11	5173350	G	C	AATATTCCAA,CATCTTTGGC	exonic	OR52A1	nonsynonymous SNV	OR52A1:ENST00000328942.1:exon1:c.C250G:p.L84V,OR52A1:ENST00000380367.1:exon2:c.C250G:p.L84V,	11p15.4	.
11	5274521	A	C	TGGTATCTGG,GGACAGGGCA	exonic	HBG2	nonsynonymous SNV	HBG2:ENST00000336906.4:exon3:c.T430G:p.S144A,HBG2:ENST00000380259.2:exon8:c.T430G:p.S144A,HBG2:ENST00000380252.1:exon3:c.T400G:p.S134A,	11p15.4	.
11	5373215	C	T	AATAGTTGTT,GCCTACACTG	exonic	OR51B6	nonsynonymous SNV	OR51B6:ENST00000380219.1:exon1:c.C478T:p.R160C,	11p15.4	.
11	5424280	C	T	GAGCTATTAT,GTACTGAAAA	exonic	OR51J1	nonsynonymous SNV	OR51J1:ENST00000332043.1:exon1:c.C454T:p.R152C,	11p15.4	.
11	6472247	G	A	CCGGTCTACG,CCACTCCCTT	exonic	TRIM3	nonsynonymous SNV	TRIM3:ENST00000359518.3:exon10:c.C1745T:p.A582V,TRIM3:ENST00000537602.1:exon9:c.C1511T:p.A504V,TRIM3:ENST00000525074.1:exon9:c.C1745T:p.A582V,TRIM3:ENST00000345851.3:exon9:c.C1745T:p.A582V,TRIM3:ENST00000536344.1:exon8:c.C1388T:p.A463V,	11p15.4	.
11	6622760	G	A	TGTATGAGGG,GTTTAGGGGA	exonic	RRP8	nonsynonymous SNV	RRP8:ENST00000254605.6:exon3:c.C536T:p.P179L,RRP8:ENST00000533907.1:exon3:c.C536T:p.P179L,	11p15.4	.
11	6661991	C	T	CCCATTGACA,CAGCATCGGC	exonic	DCHS1	nonsynonymous SNV	DCHS1:ENST00000299441.3:exon2:c.G854A:p.G285D,	11p15.4	DCHS1;basechange=c.854G>T;AAchange=p.G285V;tissue=lung
11	7064168	T	C	GCATCCTTAT,GGTGACAACA	exonic	NLRP14	nonsynonymous SNV	NLRP14:ENST00000299481.4:exon4:c.T911C:p.L304S,	11p15.4	.
11	7949705	A	T	TTAAGGCCAC,AAAGGGAAAA	exonic	OR10A6	nonsynonymous SNV	OR10A6:ENST00000309838.2:exon1:c.T505A:p.C169S,	11p15.4	.
11	8015989	C	T	AATGGGAGTC,CTGGGAGGAC	exonic	EIF3F	nonsynonymous SNV	EIF3F:ENST00000537635.1:exon5:c.C715T:p.P239S,EIF3F:ENST00000533626.1:exon7:c.C670T:p.P224S,EIF3F:ENST00000309828.4:exon5:c.C670T:p.P224S,EIF3F:ENST00000449102.2:exon4:c.C223T:p.P75S,	11p15.4	.
11	10649302	C	T	CGGCCTCATC,CCCTTCTGCT	exonic	MRVI1	nonsynonymous SNV	MRVI1:ENST00000533631.1:exon5:c.G349A:p.G117R,	11p15.4	.
11	17408810	G	A	TCCTGGTGGT,GTGCAGGTCG	exonic	KCNJ11	nonsynonymous SNV	KCNJ11:ENST00000339994.4:exon1:c.C829T:p.H277Y,KCNJ11:ENST00000528731.1:exon2:c.C568T:p.H190Y,	11p15.1	.
11	17575047	G	A	GAGCCCGAGG,ACAGAGCTTC	exonic	OTOG	nonsynonymous SNV	OTOG:ENST00000399397.1:exon5:c.G338A:p.G113E,OTOG:ENST00000399391.2:exon5:c.G557A:p.G186E,	11p15.1	.
11	17793999	C	A	CAGAAACTAC,AAAGAAAAAA	exonic	KCNC1	nonsynonymous SNV	KCNC1:ENST00000379472.3:exon2:c.C1358A:p.P453Q,KCNC1:ENST00000265969.6:exon2:c.C1358A:p.P453Q,	11p15.1	.
11	18195117	C	T	ATGACCTTTC,CTACTTTACA	exonic	MRGPRX4	nonsynonymous SNV	MRGPRX4:ENST00000314254.3:exon1:c.C314T:p.P105L,	11p15.1	.
11	18303651	G	A	ACATTGATGG,GGAAGGCCCA	exonic	HPS5	nonsynonymous SNV	HPS5:ENST00000438420.2:exon21:c.C2833T:p.P945S,HPS5:ENST00000396253.3:exon21:c.C2833T:p.P945S,HPS5:ENST00000537258.1:exon4:c.C496T:p.P166S,HPS5:ENST00000349215.3:exon22:c.C3175T:p.P1059S,	11p15.1	.
11	22646763	C	A	GGAAGTTGTT,TGAGGCAAGC	exonic	FANCF	nonsynonymous SNV	FANCF:ENST00000327470.3:exon1:c.G594T:p.Q198H,	11p14.3	.
11	32955037	C	T	AAGTAATTCC,GTCTTGAAGA	exonic	QSER1	nonsynonymous SNV	QSER1:ENST00000399302.2:exon4:c.C1846T:p.R616C,QSER1:ENST00000527788.1:exon5:c.C1129T:p.R377C,	11p13	.
11	33569349	C	T	ATCAGTGAAT,CTTGAAGTTC	exonic	KIAA1549L	nonsynonymous SNV	KIAA1549L:ENST00000389726.3:exon3:c.C2552T:p.S851F,KIAA1549L:ENST00000265654.5:exon3:c.C2552T:p.S851F,KIAA1549L:ENST00000321505.4:exon3:c.C2534T:p.S845F,	11p13	.
11	35327665	A	G	CTTCTTGATA,CCATCTTAGT	exonic	SLC1A2	nonsynonymous SNV	SLC1A2:ENST00000278379.3:exon5:c.T686C:p.V229A,SLC1A2:ENST00000395750.1:exon6:c.T659C:p.V220A,SLC1A2:ENST00000395753.1:exon6:c.T659C:p.V220A,SLC1A2:ENST00000606205.1:exon5:c.T686C:p.V229A,	11p13	.
11	45671375	C	T	GCCACGATGT,GTAGGAGAGG	exonic	CHST1	nonsynonymous SNV	CHST1:ENST00000308064.2:exon4:c.G1099A:p.D367N,	11p11.2	.
11	45947803	C	T	CAAGGAGCAC,CGGGGCTAGT	exonic	GYLTL1B	nonsynonymous SNV	GYLTL1B:ENST00000401752.1:exon8:c.C913T:p.P305S,GYLTL1B:ENST00000389968.3:exon8:c.C94T:p.P32S,GYLTL1B:ENST00000325468.5:exon7:c.C913T:p.P305S,GYLTL1B:ENST00000531526.1:exon8:c.C913T:p.P305S,GYLTL1B:ENST00000529052.1:exon9:c.C820T:p.P274S,GYLTL1B:ENST00000536139.1:	11p11.2	.
11	45947804	C	T	AAGGAGCACC,GGGGCTAGTG	exonic	GYLTL1B	nonsynonymous SNV	GYLTL1B:ENST00000401752.1:exon8:c.C914T:p.P305L,GYLTL1B:ENST00000389968.3:exon8:c.C95T:p.P32L,GYLTL1B:ENST00000325468.5:exon7:c.C914T:p.P305L,GYLTL1B:ENST00000531526.1:exon8:c.C914T:p.P305L,GYLTL1B:ENST00000529052.1:exon9:c.C821T:p.P274L,GYLTL1B:ENST00000536139.1:e	11p11.2	.
11	46341929	C	T	CCCGGGGGGC,GGCAGAGCAG	exonic	CREB3L1	nonsynonymous SNV	CREB3L1:ENST00000529193.1:exon11:c.C1373T:p.P458L,CREB3L1:ENST00000288400.3:exon11:c.C1373T:p.P458L,	11p11.2	.
11	46916187	T	A	GTTGCTGCCG,TGAGGTTGGC	exonic	LRP4	nonsynonymous SNV	LRP4:ENST00000378623.1:exon12:c.A1493T:p.N498I,	11p11.2	.
11	48286247	C	T	GGTCACACCT,TCTTAAACCC	exonic	OR4X1	nonsynonymous SNV	OR4X1:ENST00000320048.1:exon1:c.C835T:p.L279F,	11p11.2	.
11	55322114	C	T	CTGGTGTCTC,TATGTACTTC	exonic	OR4C15	nonsynonymous SNV	OR4C15:ENST00000314644.2:exon1:c.C332T:p.P111L,	11q11	.
11	55944802	G	A	CAGACAGCAA,CCTTCTCCAC	exonic	OR5J2	nonsynonymous SNV	OR5J2:ENST00000312298.1:exon1:c.G709A:p.A237T,	11q12.1	.
11	56127885	C	A	TGACTCTCGA,TTCAAACCCC	exonic	OR8J1	nonsynonymous SNV	OR8J1:ENST00000303039.3:exon1:c.C163A:p.L55I,	11q12.1	.
11	56345197	T	A	GGGGACAACA,CTTCTTATTT	exonic	OR5M10	nonsynonymous SNV	OR5M10:ENST00000526812.2:exon1:c.A1T:p.M1L,	11q12.1	.
11	56409572	C	T	GCACTCAGTC,CCAGGAAGGA	exonic	OR5AP2	nonsynonymous SNV	OR5AP2:ENST00000544374.1:exon1:c.G347A:p.G116E,OR5AP2:ENST00000302981.1:exon1:c.G344A:p.G115E,	11q12.1	.
11	57147059	C	T	ATGTCTTCTT,CCTGGGGCAC	exonic	PRG3	nonsynonymous SNV	PRG3:ENST00000287143.2:exon3:c.G283A:p.E95K,	11q12.1	.
11	57971136	G	A	GAGCAGAAGG,TGTGTGTAAG	exonic	OR1S2	nonsynonymous SNV	OR1S2:ENST00000302592.6:exon1:c.C518T:p.T173I,	11q12.1	.
11	58317456	G	A	ATCCAGGATG,GAGCAGCGCA	exonic	LPXN	nonsynonymous SNV	LPXN:ENST00000528954.1:exon6:c.C665T:p.P222L,LPXN:ENST00000528489.1:exon5:c.C590T:p.P197L,LPXN:ENST00000395074.2:exon6:c.C650T:p.P217L,	11q12.1	.
11	59211300	C	T	CTCCTTATCT,CTATGGTTAC	exonic	OR5A1	nonsynonymous SNV	OR5A1:ENST00000302030.2:exon1:c.C659T:p.S220F,	11q12.1	.
11	60183543	C	T	CACATCATCT,AAGATATGCT	exonic	MS4A14	stopgain	MS4A14:ENST00000531787.1:exon3:c.C766T:p.Q256X,MS4A14:ENST00000300187.6:exon5:c.C1102T:p.Q368X,MS4A14:ENST00000395005.2:exon4:c.C1051T:p.Q351X,MS4A14:ENST00000531783.1:exon6:c.C1201T:p.Q401X,	11q12.2	.
11	60184323	G	A	TCAAGCCGAA,GACAGCAAGC	exonic	MS4A14	nonsynonymous SNV	MS4A14:ENST00000531787.1:exon3:c.G1546A:p.G516R,MS4A14:ENST00000300187.6:exon5:c.G1882A:p.G628R,MS4A14:ENST00000395005.2:exon4:c.G1831A:p.G611R,MS4A14:ENST00000531783.1:exon6:c.G1981A:p.G661R,	11q12.2	.
11	60504497	G	A	AAGGACCCCA,TCCTTGTGTG	exonic	MS4A18	nonsynonymous SNV	MS4A18:ENST00000529108.1:exon2:c.G161A:p.S54N,MS4A18:ENST00000398983.2:exon3:c.G632A:p.S211N,	11q12.2	.
11	60899339	G	A	AACCCATAGG,AGGGGCGGGC	exonic	VPS37C	nonsynonymous SNV	VPS37C:ENST00000301765.5:exon5:c.C1021T:p.P341S,	11q12.2	.
11	61493543	G	A	GCGGACGAAG,ACTCCCAGTC	exonic	DAGLA	nonsynonymous SNV	DAGLA:ENST00000257215.5:exon6:c.G625A:p.D209N,	11q12.2	.
11	61730253	G	A	TAACCTGACG,ATATGCCAGA	exonic	BEST1	nonsynonymous SNV	BEST1:ENST00000301774.9:exon5:c.G511A:p.D171N,BEST1:ENST00000449131.2:exon9:c.G1447A:p.D483N,BEST1:ENST00000378043.4:exon10:c.G1627A:p.D543N,BEST1:ENST00000378042.3:exon9:c.G1366A:p.D456N,	11q12.3	.
11	62407171	C	T	CAGGCAGACC,CTAAAAAAGC	exonic	GANAB	nonsynonymous SNV	GANAB:ENST00000346178.4:exon2:c.G71A:p.G24E,GANAB:ENST00000532402.1:exon2:c.G71A:p.G24E,GANAB:ENST00000529737.1:exon2:c.G71A:p.G24E,GANAB:ENST00000526210.1:exon2:c.G71A:p.G24E,GANAB:ENST00000534613.1:exon2:c.G71A:p.G24E,GANAB:ENST00000356638.3:exon2:c.G71A:p.G2	11q12.3	.
11	62521094	G	A	CGCTGCTCCC,GAGGCTCTGC	exonic	ZBTB3	nonsynonymous SNV	ZBTB3:ENST00000527994.1:exon2:c.C43T:p.R15W,ZBTB3:ENST00000394807.3:exon2:c.C193T:p.R65W,	11q12.3	.
11	63411690	T	C	TCCATAGCTA,ATTCATAAGG	exonic	ATL3	nonsynonymous SNV	ATL3:ENST00000332645.4:exon9:c.A743G:p.Y248C,ATL3:ENST00000398868.3:exon7:c.A662G:p.Y221C,ATL3:ENST00000538786.1:exon7:c.A608G:p.Y203C,	11q13.1	.
11	63753619	G	A	CCAGGGAAAA,AAAACGGAAT	exonic	AP000721.4	nonsynonymous SNV	AP000721.4:ENST00000535431.1:exon2:c.G121A:p.E41K,	11q13.1	.
11	64522780	C	T	GAGATGTTCT,CGCCAGGTTT	exonic	PYGM	nonsynonymous SNV	PYGM:ENST00000164139.3:exon7:c.G820A:p.E274K,PYGM:ENST00000377432.3:exon5:c.G556A:p.E186K,	11q13.1	.
11	64825403	C	T	TCTGGCCCCC,TTGCTCCCCG	exonic	NAALADL1	nonsynonymous SNV	NAALADL1:ENST00000356632.3:exon3:c.G424A:p.G142R,NAALADL1:ENST00000340252.4:exon3:c.G424A:p.G142R,NAALADL1:ENST00000355369.2:exon3:c.G424A:p.G142R,NAALADL1:ENST00000339885.2:exon3:c.G424A:p.G142R,NAALADL1:ENST00000355721.3:exon3:c.G424A:p.G142R,NAALADL1:ENST0	11q13.1	.
11	66472068	C	T	GAGGGGCCTA,CTCTGCTGCA	splicing	SPTBN2(ENS	.	.	11q13.2	.
11	67353974	C	T	CCTCAGTGCC,GGCCCAAGCT	exonic	GSTP1	nonsynonymous SNV	GSTP1:ENST00000398606.3:exon7:c.C559T:p.R187W,GSTP1:ENST00000398603.1:exon6:c.C451T:p.R151W,	11q13.2	.
11	67432986	G	A	GGTCTGGCCG,CATTGAAGTA	exonic	ALDH3B2	nonsynonymous SNV	ALDH3B2:ENST00000349015.3:exon7:c.C476T:p.A159V,ALDH3B2:ENST00000530069.1:exon7:c.C476T:p.A159V,	11q13.2	.
11	72533411	T	G	GCCTCGCCGG,GTCTGGAAGG	exonic	ATG16L2	nonsynonymous SNV	ATG16L2:ENST00000541367.1:exon1:c.T26G:p.V9G,ATG16L2:ENST00000538973.1:exon1:c.T26G:p.V9G,	11q13.4	.
11	73076849	G	A	ACTTCGCCCG,TACTGTCAGC	exonic	ARHGEF17	nonsynonymous SNV	ARHGEF17:ENST00000263674.3:exon20:c.G5852A:p.G1951D,	11q13.4	.
11	77091019	C	T	TCTGGCCGCT,TTTCTCTTTC	exonic	PAK1	nonsynonymous SNV	PAK1:ENST00000528592.1:exon4:c.G211A:p.E71K,PAK1:ENST00000530617.1:exon3:c.G211A:p.E71K,PAK1:ENST00000524847.1:exon4:c.G211A:p.E71K,PAK1:ENST00000356341.3:exon3:c.G211A:p.E71K,PAK1:ENST00000278568.4:exon3:c.G211A:p.E71K,PAK1:ENST00000529248.1:exon4:c.G211A:p.E71K,	11q13.5	.
11	78419488	C	T	GGAGATGATC,CATTCTGATC	exonic	TENM4	nonsynonymous SNV	TENM4:ENST00000278550.7:exon27:c.G4127A:p.G1376E,	11q14.1	.
11	78419512	A	G	GCGTCTGATC,TGGTGCCATC	exonic	TENM4	nonsynonymous SNV	TENM4:ENST00000278550.7:exon27:c.T4103C:p.M1368T,	11q14.1	.
11	85422202	G	A	ATTGGTTAAA,AGCTCGGGCT	exonic	SYTL2	nonsynonymous SNV	SYTL2:ENST00000389958.3:exon2:c.C77T:p.S26F,SYTL2:ENST00000525423.1:exon5:c.C2750T:p.S917F,SYTL2:ENST00000524911.1:exon5:c.C110T:p.S37F,SYTL2:ENST00000528231.1:exon11:c.C1784T:p.S595F,SYTL2:ENST00000316356.4:exon12:c.C1787T:p.S596F,SYTL2:ENST00000525702.1:exon2:c.C11	11q14.1	.
11	92616244	C	T	CAATGGCATC,CGTTCCGGAA	exonic	FAT3	nonsynonymous SNV	FAT3:ENST00000533797.1:exon6:c.C1627T:p.P543S,FAT3:ENST00000298047.6:exon23:c.C12622T:p.P4208S,FAT3:ENST00000525166.1:exon23:c.C12172T:p.P4058S,FAT3:ENST00000409404.2:exon23:c.C12622T:p.P4208S,	11q14.3	.
11	94554875	C	T	GGTCCAGATG,CTTTGCGATT	exonic	AMOTL1	nonsynonymous SNV	AMOTL1:ENST00000433060.2:exon4:c.C1301T:p.A434V,AMOTL1:ENST00000317829.8:exon3:c.C1151T:p.A384V,	11q21	.
11	94730798	C	T	GGTGTTTACT,AATACCATAA	exonic	KDM4D	stopgain	KDM4D:ENST00000335080.5:exon3:c.C262T:p.Q88X,KDM4D:ENST00000536741.1:exon2:c.C262T:p.Q88X,	11q21	.
11	101929646	G	T	TTCCCAATTT,AAGTTACTTT	exonic	C11orf70	nonsynonymous SNV	C11orf70:ENST00000434758.2:exon3:c.G228T:p.L76F,C11orf70:ENST00000526781.1:exon3:c.G228T:p.L76F,C11orf70:ENST00000534360.1:exon3:c.G228T:p.L76F,	11q22.1	.
11	102666213	C	T	TCAATGTCAT,CTGAGCTAGC	exonic	MMP1	nonsynonymous SNV	MMP1:ENST00000315274.6:exon5:c.G751A:p.D251N,	11q22.2	.
11	113856753	C	G	ACATCAACAT,TCTTTGTGGC	exonic	HTR3A	nonsynonymous SNV	HTR3A:ENST00000375498.2:exon6:c.C579G:p.I193M,HTR3A:ENST00000299961.5:exon6:c.C516G:p.I172M,HTR3A:ENST00000355556.2:exon6:c.C579G:p.I193M,HTR3A:ENST00000504030.2:exon6:c.C561G:p.I187M,HTR3A:ENST00000506841.2:exon6:c.C561G:p.I187M,	11q23.2	.
11	113935263	C	T	GTCGAGCTTC,TGATAACGAG	exonic	ZBTB16	nonsynonymous SNV	ZBTB16:ENST00000392996.2:exon2:c.C1241T:p.P414L,ZBTB16:ENST00000335953.4:exon2:c.C1241T:p.P414L,	11q23.2	.
11	118211218	C	T	GAGCAGTGTT,CCACCGTTCC	exonic	CD3D	nonsynonymous SNV	CD3D:ENST00000300692.4:exon2:c.G146A:p.G49E,CD3D:ENST00000392884.2:exon2:c.G146A:p.G49E,	11q23.3	.
11	118402823	C	T	ACAGTACCAA,CGTGCCTGAG	exonic	TMEM25	nonsynonymous SNV	TMEM25:ENST00000525118.1:exon3:c.C82T:p.P28S,	11q23.3	.
11	118627020	G	A	TGAAATACAC,ATTTCGATGT	exonic	DDX6	nonsynonymous SNV	DDX6:ENST00000534980.1:exon11:c.C1123T:p.R375C,DDX6:ENST00000264018.4:exon11:c.C1123T:p.R375C,DDX6:ENST00000526070.2:exon11:c.C1123T:p.R375C,	11q23.3	.
11	123508907	C	T	TTTTCTTGGG,TGCCTCTTCG	exonic	SCN3B	nonsynonymous SNV	SCN3B:ENST00000299333.3:exon5:c.G571A:p.A191T,SCN3B:ENST00000392770.2:exon4:c.G571A:p.A191T,SCN3B:ENST00000530277.1:exon5:c.G571A:p.A191T,	11q24.1	.
11	123624835	G	A	CATGATGGTG,GGTGGTGAAG	exonic	OR6X1	nonsynonymous SNV	OR6X1:ENST00000327930.2:exon1:c.C392T:p.P131L,	11q24.1	.
11	124746176	C	T	CACAGCGTCC,CGCCGGACCT	exonic	ROBO3	nonsynonymous SNV	ROBO3:ENST00000397801.1:exon17:c.C2599T:p.P867S,ROBO3:ENST00000538940.1:exon16:c.C2533T:p.P845S,	11q24.2	.
11	125301065	C	T	CTCGCAGGTT,CATCAACTTG	exonic	PKNOX2	nonsynonymous SNV	PKNOX2:ENST00000542175.1:exon12:c.C1004T:p.S335F,PKNOX2:ENST00000298282.9:exon13:c.C1196T:p.S399F,	11q24.2	.
11	126074195	C	A	ATCCAAAAGA,AAGTGAACTC	exonic	RPUSD4	nonsynonymous SNV	RPUSD4:ENST00000533628.1:exon6:c.G732T:p.L244F,RPUSD4:ENST00000298317.4:exon6:c.G825T:p.L275F,	11q24.2	.
11	132200074	G	A	CTGACCCCTT,GAAAGGTTTG	exonic	NTM	stopgain	NTM:ENST00000427481.2:exon8:c.G935A:p.W312X,NTM:ENST00000425719.2:exon7:c.G962A:p.W321X,NTM:ENST00000539799.1:exon8:c.G962A:p.W321X,	11q25	.
11	133794748	C	T	TTGCTGGGCT,GCTGATCAGA	exonic	IGSF9B	nonsynonymous SNV	IGSF9B:ENST00000533871.2:exon15:c.G2086A:p.E696K,IGSF9B:ENST00000321016.8:exon15:c.G2086A:p.E696K,	11q25	.
12	3686118	C	T	ATCGTGGATC,AAAGCAAGTG	exonic	PRMT8	nonsynonymous SNV	PRMT8:ENST00000452611.2:exon7:c.C767T:p.P256L,PRMT8:ENST00000382622.3:exon7:c.C794T:p.P265L,	12p13.32	.
12	6806693	C	T	GGCGGCCCCT,CTCAAAGCCT	exonic	PIANP	nonsynonymous SNV	PIANP:ENST00000320591.5:exon3:c.G283A:p.E95K,PIANP:ENST00000542740.1:exon2:c.G283A:p.E95K,PIANP:ENST00000545917.1:exon3:c.G283A:p.E95K,PIANP:ENST00000534837.1:exon3:c.G283A:p.E95K,PIANP:ENST00000540656.1:exon3:c.G283A:p.E95K,	12p13.31	.
12	7475054	G	C	GACCGGAGGG,AGCCACTCAA	exonic	ACSM4	nonsynonymous SNV	ACSM4:ENST00000399422.4:exon7:c.G1042C:p.E348Q,	12p13.31	.
12	7526152	C	T	GCCGACGCTG,CCCAGGTCCC	exonic	CD163L1	nonsynonymous SNV	CD163L1:ENST00000313599.3:exon14:c.G3494A:p.G1165D,CD163L1:ENST00000396630.1:exon14:c.G3494A:p.G1165D,CD163L1:ENST00000416109.2:exon14:c.G3524A:p.G1175D,	12p13.31	.
12	10954244	C	T	GAATTTTCTT,CTTTTAGAAT	exonic	TAS2R7	nonsynonymous SNV	TAS2R7:ENST00000240687.2:exon1:c.G926A:p.G309E,	12p13.2	.
12	10954245	C	T	AATTTTCTTC,TTTTAGAATA	exonic	TAS2R7	nonsynonymous SNV	TAS2R7:ENST00000240687.2:exon1:c.G925A:p.G309R,	12p13.2	.
12	11035674	G	A	TATTACGAGG,GAACATCTTC	exonic	PRH1	nonsynonymous SNV	PRH1:ENST00000428168.2:exon2:c.C86T:p.P29L,	12p13.2	.
12	16430342	G	A	AAACACAGGG,TAAGTATTGA	exonic	SLC15A5	nonsynonymous SNV	SLC15A5:ENST00000344941.3:exon1:c.C278T:p.T93I,	12p12.3	.
12	20766527	C	T	GCTCACCTTC,AGGCCATTCA	exonic	PDE3A	stopgain	PDE3A:ENST00000359062.3:exon3:c.C1162T:p.Q388X,	12p12.2	.
12	20807032	G	T	CCTGGAAAAT,GGTGGAAGAC	exonic	PDE3A	nonsynonymous SNV	PDE3A:ENST00000359062.3:exon15:c.G3077T:p.W1026L,	12p12.2	.
12	20854291	G	T	CTTTGCCAAA,CATTGGCAGA	exonic	SLCO1C1	nonsynonymous SNV	SLCO1C1:ENST00000540354.1:exon4:c.G169T:p.A57S,SLCO1C1:ENST00000545604.1:exon4:c.G169T:p.A57S,SLCO1C1:ENST00000266509.2:exon3:c.G169T:p.A57S,SLCO1C1:ENST00000381552.1:exon3:c.G169T:p.A57S,	12p12.2	SLCO1C1_ENST00000381552;basechange=c.169G>A;AAchange=p.A57T;tissue=endometrium&SLCO1C1;basechange=c.169G>A;AAchange=p.A57T;tissue=endometrium
12	21355521	C	T	GCCAAATTCT,ATGTTTTACT	exonic	SLCO1B1	nonsynonymous SNV	SLCO1B1:ENST00000256958.2:exon10:c.C1232T:p.S411L,	12p12.1	.
12	21958170	C	A	AAATTTGGGA,AGTATCACAC	exonic	ABCC9	nonsynonymous SNV	ABCC9:ENST00000544039.1:exon31:c.G3469T:p.V1157F,ABCC9:ENST00000261201.4:exon38:c.G4588T:p.V1530F,ABCC9:ENST00000345162.2:exon37:c.G4480T:p.V1494F,	12p12.1	.
12	26714720	T	C	AATATTTCTG,AATCCCAAGC	exonic	ITPR2	nonsynonymous SNV	ITPR2:ENST00000381340.3:exon35:c.A4796G:p.Y1599C,	12p11.23	.
12	28122369	A	T	GCAGGAGGGC,CCGCGTAGCT	exonic	PTHLH	nonsynonymous SNV	PTHLH:ENST00000534890.1:exon2:c.T83A:p.V28E,PTHLH:ENST00000354417.3:exon3:c.T59A:p.V20E,PTHLH:ENST00000545234.1:exon4:c.T59A:p.V20E,PTHLH:ENST00000539239.1:exon1:c.T59A:p.V20E,PTHLH:ENST00000395868.3:exon2:c.T59A:p.V20E,PTHLH:ENST00000538310.1:exon1:c.T59A:p.V20E,PT	12p11.22	.
12	29460659	C	T	AATATTTATA,CTACACCAAG	exonic	FAR2	nonsynonymous SNV	FAR2:ENST00000182377.4:exon5:c.C614T:p.T205I,FAR2:ENST00000536681.3:exon5:c.C614T:p.T205I,FAR2:ENST00000547116.1:exon4:c.C323T:p.T108I,	12p11.22	.
12	29629166	C	G	TCAGAATCAC,GTAAATCACA	exonic	OVCH1	nonsynonymous SNV	OVCH1:ENST00000318184.5:exon13:c.G1444C:p.G482R,	12p11.22	OVCH1;basechange=c.1444G>A;AAchange=p.G482S;tissue=kidney
12	31632961	C	T	GCCAATGAGT,CATACTGCAG	exonic	DENND5B	nonsynonymous SNV	DENND5B:ENST00000389082.5:exon3:c.G466A:p.D156N,DENND5B:ENST00000306833.6:exon4:c.G571A:p.D191N,DENND5B:ENST00000546299.1:exon2:c.G322A:p.D108N,DENND5B:ENST00000354285.4:exon4:c.G532A:p.D178N,DENND5B:ENST00000536562.1:exon5:c.G571A:p.D191N,	12p11.21	.
12	40114682	G	A	TTTTTATGTA,AAAGGATGGC	exonic	C12orf40	nonsynonymous SNV	C12orf40:ENST00000324616.5:exon13:c.G1588A:p.E530K,	12q12	.
12	40814120	G	A	GAATCTGGAG,GGATTTCAAT	exonic	MUC19	nonsynonymous SNV	MUC19:ENST00000425730.2:exon7:c.G479A:p.G160E,	12q12	.
12	40878486	G	A	CAATCTGTCG,AGTGACAGGG	exonic	MUC19	nonsynonymous SNV	MUC19:ENST00000454784.4:exon49:c.G10031A:p.G3344E,	12q12	.
12	41900404	G	A	TGCAGCTTAT,AATGCCAGCA	exonic	PDZRN4	nonsynonymous SNV	PDZRN4:ENST00000402685.2:exon4:c.G990A:p.M330I,PDZRN4:ENST00000539469.2:exon2:c.G216A:p.M72I,PDZRN4:ENST00000298919.7:exon4:c.G210A:p.M70I,	12q12	.
12	44913873	G	A	GTCCAGGCAA,TCTTGGTGAT	exonic	NELL2	nonsynonymous SNV	NELL2:ENST00000395487.2:exon19:c.C2312T:p.T771I,NELL2:ENST00000437801.2:exon20:c.C2465T:p.T822I,NELL2:ENST00000551601.1:exon20:c.C2171T:p.T724I,NELL2:ENST00000549027.1:exon21:c.C2312T:p.T771I,NELL2:ENST00000333837.4:exon20:c.C2384T:p.T795I,NELL2:ENST00000429094.2:exon19	12q12	.
12	46589996	G	A	AGCGCTGCAG,GAGAAGGATG	exonic	SLC38A1	nonsynonymous SNV	SLC38A1:ENST00000552197.1:exon17:c.C1426T:p.P476S,	12q13.11	.
12	48737079	T	A	AGATGGGAGT,GCATCGGAAA	exonic	ZNF641	nonsynonymous SNV	ZNF641:ENST00000448928.3:exon5:c.A925T:p.N309Y,ZNF641:ENST00000544117.2:exon6:c.A994T:p.N332Y,ZNF641:ENST00000547026.1:exon6:c.A952T:p.N318Y,ZNF641:ENST00000301042.3:exon7:c.A994T:p.N332Y,	12q13.11	.
12	49221458	C	T	CGAGAGCTCC,GCCAAGCCTG	exonic	CACNB3	nonsynonymous SNV	CACNB3:ENST00000547392.1:exon12:c.C1150T:p.R384C,CACNB3:ENST00000547230.1:exon12:c.C1108T:p.R370C,CACNB3:ENST00000540990.1:exon13:c.C1192T:p.R398C,CACNB3:ENST00000301050.2:exon13:c.C1231T:p.R411C,CACNB3:ENST00000536187.2:exon13:c.C1228T:p.R410C,	12q13.12	.
12	49297660	G	A	CCCGGGAGGC,GAGGTTGCAG	exonic	RP11-302B13	nonsynonymous SNV	RP11-302B13.5:ENST00000398092.4:exon5:c.C416T:p.P139L,	12q13.12	.
12	49433632	G	A	TTTTCGACAG,AGAGGTGATG	exonic	KMT2D	nonsynonymous SNV	KMT2D:ENST00000301067.7:exon31:c.C7921T:p.P2641S,	12q13.12	.
12	49462954	C	T	TCTGTCCTTT,GGACTGTGGC	exonic	RHEBL1	nonsynonymous SNV	RHEBL1:ENST00000550797.1:exon2:c.G56A:p.R19Q,	12q13.12	.
12	49719931	G	A	GGAGACAGCT,GCAGCAGCCG	exonic	TROAP	nonsynonymous SNV	TROAP:ENST00000257909.3:exon6:c.G706A:p.G236S,TROAP:ENST00000547807.1:exon6:c.G706A:p.G236S,TROAP:ENST00000550346.1:exon4:c.G355A:p.G119S,TROAP:ENST00000551245.1:exon6:c.G706A:p.G236S,	12q13.12	.
12	49719932	G	A	GAGACAGCTG,CAGCAGCCGG	exonic	TROAP	nonsynonymous SNV	TROAP:ENST00000257909.3:exon6:c.G707A:p.G236D,TROAP:ENST00000547807.1:exon6:c.G707A:p.G236D,TROAP:ENST00000550346.1:exon4:c.G356A:p.G119D,TROAP:ENST00000551245.1:exon6:c.G707A:p.G236D,	12q13.12	.
12	50190802	G	A	TGGGCCTGAG,AGGTCCCCTG	exonic	NCKAP5L	nonsynonymous SNV	NCKAP5L:ENST00000335999.6:exon8:c.C841T:p.P281S,	12q13.12	.
12	50745322	C	T	TTGAGGGGAA,CCTTGATATC	exonic	FAM186A	nonsynonymous SNV	FAM186A:ENST00000543111.1:exon4:c.G5293A:p.V1765I,FAM186A:ENST00000327337.5:exon4:c.G5293A:p.V1765I,	12q13.12	.
12	52080874	G	A	TTCTTTGACA,GTACACGTTC	splicing	SCN8A(ENST	.	.	12q13.13	.
12	52408690	C	T	GCCGGCGCTG,CGCCCCCGCC	exonic	GRASP	nonsynonymous SNV	GRASP:ENST00000380039.2:exon5:c.C466T:p.P156S,GRASP:ENST00000546756.1:exon7:c.C505T:p.P169S,GRASP:ENST00000552049.1:exon7:c.C466T:p.P156S,GRASP:ENST00000293662.4:exon8:c.C895T:p.P299S,	12q13.13	.
12	52567518	C	T	ACCGACACAT,CTTCACCTGT	exonic	KRT80	nonsynonymous SNV	KRT80:ENST00000394815.2:exon5:c.G697A:p.D233N,KRT80:ENST00000313234.5:exon5:c.G697A:p.D233N,	12q13.13	.
12	52772035	G	A	GGGGCCACAG,AAGCCAGGAC	exonic	KRT84	nonsynonymous SNV	KRT84:ENST00000257951.3:exon9:c.C1586T:p.S529F,	12q13.13	.
12	52913935	A	G	CGCAAGGCTG,CCCTGCCGAA	exonic	KRT5	nonsynonymous SNV	KRT5:ENST00000546577.1:exon2:c.T146C:p.V49A,KRT5:ENST00000551275.1:exon1:c.T146C:p.V49A,KRT5:ENST00000252242.4:exon1:c.T146C:p.V49A,	12q13.13	.
12	53045556	C	T	CCCAAAACCT,CAAAGCGGCC	exonic	KRT2	nonsynonymous SNV	KRT2:ENST00000309680.3:exon1:c.G371A:p.G124E,	12q13.13	.
12	53045557	C	T	CCAAAACCTC,AAAGCGGCCT	exonic	KRT2	nonsynonymous SNV	KRT2:ENST00000309680.3:exon1:c.G370A:p.G124R,	12q13.13	.
12	53225244	C	T	TGAGTCCAGC,TCCCCCGCTC	exonic	KRT79	nonsynonymous SNV	KRT79:ENST00000330553.5:exon2:c.G644A:p.R215K,	12q13.13	.
12	53555124	C	T	GTCAAAGGCC,CTAGCACAGT	exonic	CSAD	nonsynonymous SNV	CSAD:ENST00000548698.1:exon5:c.G311A:p.G104E,CSAD:ENST00000379846.1:exon6:c.G311A:p.G104E,CSAD:ENST00000267085.4:exon11:c.G833A:p.G278E,CSAD:ENST00000444623.1:exon11:c.G752A:p.G251E,CSAD:ENST00000453446.2:exon10:c.G752A:p.G251E,CSAD:ENST00000379843.3:exon7:c.G31	12q13.13	.
12	53591704	C	T	GGCGCAGCGC,GCGCCTCCGC	exonic	ITGB7	nonsynonymous SNV	ITGB7:ENST00000267082.5:exon4:c.G233A:p.R78Q,ITGB7:ENST00000422257.3:exon4:c.G233A:p.R78Q,ITGB7:ENST00000338737.4:exon4:c.G233A:p.R78Q,ITGB7:ENST00000550743.2:exon2:c.G233A:p.R78Q,ITGB7:ENST00000542497.1:exon3:c.G233A:p.R78Q,ITGB7:ENST00000552935.1:exon3:c.G233A:p.R7	12q13.13	.
12	53722937	G	A	GGGAATGAGT,GGAAAAGGGA	exonic	SP7	nonsynonymous SNV	SP7:ENST00000547755.1:exon2:c.C235T:p.H79Y,SP7:ENST00000536324.2:exon3:c.C289T:p.H97Y,SP7:ENST00000303846.3:exon2:c.C289T:p.H97Y,SP7:ENST00000537210.2:exon2:c.C235T:p.H79Y,	12q13.13	.

12	114384210 G	A	GTAGGACGAC,ATCCCAGGGC	exonic	RBM19	nonsynonymous SNV	RBM19:ENST00000392561.3:exon12:c.C1478T:p.S493L,RBM19:ENST00000545145.2:exon12:c.C1478T:p.S493L,RBM19:ENST00000261741.5:exon12:c.C1478T:p.S493L,	12q24.21	.
12	117615406 A	G	CGCAGGTAGT,AAGAATTTTT	exonic	FBXO21	nonsynonymous SNV	FBXO21:ENST00000330622.5:exon4:c.T511C:p.Y171H,FBXO21:ENST00000427718.2:exon4:c.T511C:p.Y171H,	12q24.22	.
12	120214180 T	A	GTTTCGCATA,TCTCCCACTT	exonic	CIT	nonsynonymous SNV	CIT:ENST00000261833.7:exon15:c.A1869T:p.E623D,CIT:ENST00000392521.2:exon15:c.A1869T:p.E623D,	12q24.23	.
12	120637117 C	T	ACTGACTTCT,CAGAGCTGGG	exonic	RPLP0	nonsynonymous SNV	RPLP0:ENST00000547191.1:exon4:c.G184A:p.E62K,RPLP0:ENST00000546989.1:exon3:c.G226A:p.E76K,RPLP0:ENST00000546990.1:exon2:c.G226A:p.E76K,RPLP0:ENST00000392514.4:exon3:c.G226A:p.E76K,RPLP0:ENST00000550423.1:exon3:c.G184A:p.E62K,RPLP0:ENST00000550856.1:exon3:c.G226A:p.E	12q24.23	.
12	121416467 G	A	TGGGTGCAAG,AGTTTGGTTT	exonic	HNF1A	nonsynonymous SNV	HNF1A:ENST00000535955.1:exon1:c.G14A:p.G5E,	12q24.31	.
12	121698268 G	A	TTGCCAACAG,AGGGCTGCAT	exonic	CAMKK2	nonsynonymous SNV	CAMKK2:ENST00000545538.1:exon1:c.C11T:p.S4F,	12q24.31	.
12	122257697 C	T	AGGGAGCCGC,TGAGGAACCA	exonic	SETD1B	nonsynonymous SNV	SETD1B:ENST00000267197.5:exon11:c.C3677T:p.P1226L,SETD1B:ENST00000604567.1:exon11:c.C3806T:p.P1269L,SETD1B:ENST00000542440.1:exon12:c.C3677T:p.P1226L,	12q24.31	.
12	124819139 G	A	GGGTGGTGCC,GGTGTAGTCC	exonic	NCOR2	nonsynonymous SNV	NCOR2:ENST00000397355.1:exon41:c.C6409T:p.R2137W,NCOR2:ENST00000404621.1:exon41:c.C6406T:p.R2136W,NCOR2:ENST00000405201.1:exon41:c.C6436T:p.R2146W,NCOR2:ENST00000356219.3:exon42:c.C6457T:p.R2153W,NCOR2:ENST00000404121.2:exon41:c.C5119T:p.R1707W,NCOR2:ENST00	12q24.31	.
12	124979719 G	A	ATCTGCACTG,GTAGGAAAGG	exonic	NCOR2	nonsynonymous SNV	NCOR2:ENST00000397355.1:exon2:c.C79T:p.P27S,NCOR2:ENST00000404621.1:exon2:c.C79T:p.P27S,NCOR2:ENST00000458234.1:exon3:c.C79T:p.P27S,NCOR2:ENST00000405201.1:exon1:c.C79T:p.P27S,NCOR2:ENST00000356219.3:exon2:c.C79T:p.P27S,NCOR2:ENST00000420698.1:exon2:c.C79T:p.P27S	12q24.31	.
12	125834264 G	A	GACATCTACA,CCTTTGGAAA	exonic	TMEM132B	nonsynonymous SNV	TMEM132B:ENST00000299308.3:exon2:c.G319A:p.A107T,	12q24.31	.
12	126128796 G	A	CCAGATCAAG,GCTGGAGGAT	exonic	TMEM132B	nonsynonymous SNV	TMEM132B:ENST00000299308.3:exon6:c.G1597A:p.G533S,TMEM132B:ENST00000535886.1:exon2:c.G133A:p.G45S,	12q24.32	TMEM132B;basechange=c.1597G>A;AAchange=p.G533S;tissue=skin
12	126137071 G	A	GGAGCTGGGA,TGCAGCTCGT	exonic	TMEM132B	nonsynonymous SNV	TMEM132B:ENST00000299308.3:exon8:c.G1984A:p.V662M,TMEM132B:ENST00000535886.1:exon4:c.G520A:p.V174M,	12q24.32	.
12	128900101 G	A	AGTCAAGCAG,GAGAGGTGGT	exonic	TMEM132C	nonsynonymous SNV	TMEM132C:ENST00000435159.2:exon2:c.G910A:p.G304R,	12q24.32	.
12	129178515 G	C	CCTGCAGATC,AGGTCTCTGA	exonic	TMEM132C	nonsynonymous SNV	TMEM132C:ENST00000315208.8:exon4:c.G439C:p.E147Q,TMEM132C:ENST00000435159.2:exon6:c.G1591C:p.E531Q,	12q24.32	.
12	130921694 G	A	CAGGAGCTCG,GGGGAACGGC	exonic	RIMBP2	nonsynonymous SNV	RIMBP2:ENST00000261655.4:exon10:c.C1748T:p.P583L,RIMBP2:ENST00000536002.1:exon14:c.C1472T:p.P491L,RIMBP2:ENST00000535703.1:exon12:c.C1472T:p.P491L,	12q24.33	.
12	130941052 G	A	TTTGCCGAGG,AGGTGGCTAG	exonic	RIMBP2	nonsynonymous SNV	RIMBP2:ENST00000261655.4:exon4:c.C296T:p.S99F,RIMBP2:ENST00000536002.1:exon8:c.C20T:p.S7F,RIMBP2:ENST00000535703.1:exon6:c.C20T:p.S7F,	12q24.33	.
12	132195844 G	A	GGCCGGGCCA,GCGGTGCCGG	exonic	SFSWAP	nonsynonymous SNV	SFSWAP:ENST00000541286.1:exon1:c.G70A:p.G24S,SFSWAP:ENST00000261674.4:exon1:c.G70A:p.G24S,SFSWAP:ENST00000538548.1:exon1:c.G70A:p.G24S,	12q24.33	.
13	19753574 C	T	GAGTCGTCCC,ACCACCAATG	exonic	TUBA3C	nonsynonymous SNV	TUBA3C:ENST00000400113.3:exon2:c.G133A:p.G45R,	13q12.11	TUBA3C;basechange=c.133G>T;AAchange=p.G45W;tissue=lung
13	23912517 G	A	CTCACTCAGG,AAAACTTCAG	exonic	SACS	nonsynonymous SNV	SACS:ENST00000382292.3:exon9:c.C5498T:p.S1833F,SACS:ENST00000382298.3:exon10:c.C5498T:p.S1833F,SACS:ENST00000402364.1:exon8:c.C3248T:p.S1083F,	13q12.12	.
13	28877345 C	T	GAGTTGTAGT,TGGGGGCGGG	exonic	FLT1	nonsynonymous SNV	FLT1:ENST00000543394.1:exon10:c.G1045A:p.D349N,FLT1:ENST00000282397.4:exon30:c.G3976A:p.D1326N,FLT1:ENST00000540678.1:exon17:c.G1630A:p.D544N,	13q12.2	.
13	29008229 A	C	TCTTATACAA,TGCCCATTGA	exonic	FLT1	nonsynonymous SNV	FLT1:ENST00000541932.1:exon5:c.T642G:p.H214Q,FLT1:ENST00000282397.4:exon5:c.T642G:p.H214Q,FLT1:ENST00000539099.1:exon5:c.T642G:p.H214Q,	13q12.3	.
13	35806681 G	A	TCTTCTTCGT,AAATTTTTGT	exonic	NBEA	nonsynonymous SNV	NBEA:ENST00000400445.3:exon34:c.G5701A:p.E1901K,NBEA:ENST00000379939.2:exon33:c.G5692A:p.E1898K,NBEA:ENST00000540320.1:exon34:c.G5701A:p.E1901K,NBEA:ENST00000310336.4:exon35:c.G5701A:p.E1901K,	13q13.3	.
13	38164633 C	T	TCCCACGATG,CCAGAGTGCC	exonic	POSTN	nonsynonymous SNV	POSTN:ENST00000379747.4:exon4:c.G317A:p.G106D,POSTN:ENST00000541179.1:exon4:c.G317A:p.G106D,POSTN:ENST00000379742.4:exon4:c.G317A:p.G106D,POSTN:ENST00000379749.4:exon4:c.G317A:p.G106D,POSTN:ENST00000541481.1:exon4:c.G317A:p.G106D,POSTN:ENST00000379743.4:exon4:	13q13.3	.
13	52663450 C	T	CTCAGCAGGC,TTAGAAAATA	splicing	NEK5(ENST0	.	.	13q14.3	.
13	58299063 C	T	ATCAAGCAGC,CAACCAAGGC	exonic	PCDH17	nonsynonymous SNV	PCDH17:ENST00000377918.3:exon4:c.C3115T:p.P1039S,	13q21.1	.
13	70293595 C	T	GTGGCCACTC,GACACCCCCT	exonic	KLHL1	nonsynonymous SNV	KLHL1:ENST00000545028.1:exon8:c.G1342A:p.G448R,KLHL1:ENST00000377844.4:exon9:c.G1921A:p.G641R,	13q21.33	KLHL1;basechange=c.1921G>T;AAchange=p.G641*;tissue=lung
13	74420075 G	A	ACCACCACGG,GATGCGGTGA	exonic	KLF12	nonsynonymous SNV	KLF12:ENST00000377669.2:exon3:c.C559T:p.P187S,KLF12:ENST00000377666.4:exon4:c.C559T:p.P187S,	13q22.1	.
13	77531327 G	A	GCCAAGCATG,GATAGAAGCT	exonic	IRG1	nonsynonymous SNV	IRG1:ENST00000449753.1:exon4:c.G653A:p.G218E,IRG1:ENST00000377462.1:exon5:c.G653A:p.G218E,	13q22.3	.
13	77532022 G	A	AGACCTAGAA,ACTGTTCTGT	exonic	IRG1	nonsynonymous SNV	IRG1:ENST00000449753.1:exon4:c.G1348A:p.D450N,IRG1:ENST00000377462.1:exon5:c.G1348A:p.D450N,	13q22.3	.
13	77635365 C	T	GTGCCATCAA,CAGAACAATT	exonic	MYCBP2	stopgain	MYCBP2:ENST00000544440.2:exon76:c.G12861A:p.W4287X,MYCBP2:ENST00000357337.6:exon77:c.G12861A:p.W4287X,MYCBP2:ENST00000407578.2:exon76:c.G12975A:p.W4325X,	13q22.3	.
13	88328230 T	A	CCCAACAATC,TTTCCGTTTT	exonic	SLITRK5	nonsynonymous SNV	SLITRK5:ENST00000325089.6:exon2:c.T587A:p.L196H,	13q31.2	.
13	88330284 C	T	TCCCAAATTC,CGTGCAGCCC	exonic	SLITRK5	nonsynonymous SNV	SLITRK5:ENST00000400028.3:exon2:c.C1918T:p.P640S,SLITRK5:ENST00000325089.6:exon2:c.C2641T:p.P881S,	13q31.2	.
13	92345833 G	A	CAACACCACA,ACTATCTGCA	exonic	GPC5	nonsynonymous SNV	GPC5:ENST00000377067.3:exon3:c.G718A:p.D240N,	13q31.3	.
13	92560258 G	A	CAAAGTCAAA,GAATTGATCC	exonic	GPC5	nonsynonymous SNV	GPC5:ENST00000377067.3:exon6:c.G1348A:p.G450R,	13q31.3	.
13	95055385 G	A	GAGAGAGGTG,ACTCTTCTGC	exonic	GPC6	nonsynonymous SNV	GPC6:ENST00000377047.4:exon9:c.G1582A:p.D528N,	13q32.1	.
13	102527574 C	T	ATCGAGAGCT,CATCGGGGTG	exonic	FGF14	nonsynonymous SNV	FGF14:ENST00000376143.4:exon2:c.G266A:p.G89E,FGF14:ENST00000376131.4:exon2:c.G281A:p.G94E,	13q33.1	.
13	109779671 C	T	CACCCCAGCT,CATGTCAGTC	exonic	MYO16	nonsynonymous SNV	MYO16:ENST00000457511.2:exon19:c.C2294T:p.S765F,MYO16:ENST00000357550.2:exon30:c.C3758T:p.S1253F,MYO16:ENST00000356711.2:exon31:c.C3758T:p.S1253F,	13q33.3	.
13	111138148 G	A	TGGCCCCCCT,GAATTACGGG	exonic	COL4A2	nonsynonymous SNV	COL4A2:ENST00000360467.5:exon34:c.G3172A:p.G1058R,	13q34	.
13	113720388 G	A	CTGAAAGAGG,GCACAGTGTC	exonic	MCF2L	nonsynonymous SNV	MCF2L:ENST00000535094.2:exon8:c.G785A:p.G262E,MCF2L:ENST00000375597.4:exon8:c.G779A:p.G260E,MCF2L:ENST00000421756.1:exon8:c.G797A:p.G266E,MCF2L:ENST00000397030.1:exon8:c.G884A:p.G295E,MCF2L:ENST00000375604.2:exon8:c.G956A:p.G319E,MCF2L:ENST00000442652.2:exon8:c.C	13q34	.
14	20344805 T	A	TATTGCAATA,GCAAGCCCCT	exonic	OR4K2	nonsynonymous SNV	OR4K2:ENST00000298642.2:exon1:c.T379A:p.C127S,	14q11.2	.
14	21526203 A	G	CAAGCATGCA,CTCAGCCATG	exonic	RNASE8	nonsynonymous SNV	RNASE8:ENST00000308227.2:exon1:c.A152G:p.N51S,	14q11.2	.
14	21570563 G	A	CTCACTGGCA,GTGCGGGCCA	splicing	TMEM253(EN	.	.	14q11.2	.
14	22133790 G	C	TTGACTATTC,TCTACCTTAC	exonic	OR4E2	nonsynonymous SNV	OR4E2:ENST00000408935.1:exon1:c.G494C:p.R165P,	14q11.2	.
14	22891825 G	A	TCCATGAAAG,AGAAGCGATC	exonic	TRDV2	nonsynonymous SNV	TRDV2:ENST00000390469.2:exon2:c.G137A:p.G46E,	14q11.2	.
14	23235791 C	T	GCTCCGTTCT,TTTTACCGCC	exonic	OXA1L	nonsynonymous SNV	OXA1L:ENST00000285848.5:exon1:c.C61T:p.L21F,	14q11.2	.
14	23842338 G	A	ATGAGGGAGC,ACCCAGATTA	exonic	IL25	nonsynonymous SNV	IL25:ENST00000329715.2:exon1:c.G11A:p.R4Q,	14q11.2	.
14	23898190 C	T	CCAGCGATGT,CAGGACTCCT	exonic	MYH7	nonsynonymous SNV	MYH7:ENST00000355349.3:exon14:c.G1381A:p.D461N,	14q11.2	.
14	24040478 G	A	CTGGAGAAGG,ACCCTGGTCC	exonic	JPH4	nonsynonymous SNV	JPH4:ENST00000397118.3:exon6:c.C1462T:p.P488S,JPH4:ENST00000544177.1:exon3:c.C457T:p.P153S,JPH4:ENST00000356300.4:exon5:c.C1462T:p.P488S,	14q11.2	.
14	24567866 G	A	GCACTCCGTG,GCCAGCCCCT	exonic	PCK2	nonsynonymous SNV	PCK2:ENST00000545054.2:exon4:c.G241A:p.G81S,PCK2:ENST00000561286.1:exon4:c.G241A:p.G81S,PCK2:ENST00000558096.1:exon4:c.G241A:p.G81S,PCK2:ENST00000559250.1:exon4:c.G679A:p.G227S,PCK2:ENST00000216780.4:exon4:c.G643A:p.G215S,PCK2:ENST00000559837.1:exon4:c.G241A:p.G81	14q11.2	.
14	24706480 G	A	GAAGAAAACT,GAGTGGGGTA	exonic	GMPR2	nonsynonymous SNV	GMPR2:ENST00000355299.4:exon7:c.G577A:p.G193R,GMPR2:ENST00000420554.2:exon6:c.G631A:p.G211R,GMPR2:ENST00000558865.1:exon5:c.G115A:p.G39R,GMPR2:ENST00000557854.1:exon6:c.G631A:p.G211R,GMPR2:ENST00000559836.1:exon7:c.G577A:p.G193R,GMPR2:ENST00000559104.1:exon6:	14q12	.
14	24801056 A	C	GCCTCCCGGA,CGTGGCCCGC	exonic	ADCY4	nonsynonymous SNV	ADCY4:ENST00000554068.2:exon5:c.T607G:p.F203V,ADCY4:ENST00000310677.4:exon5:c.T607G:p.F203V,ADCY4:ENST00000557056.1:exon4:c.T211G:p.F71V,ADCY4:ENST00000554781.1:exon4:c.T607G:p.F203V,ADCY4:ENST00000418030.2:exon4:c.T607G:p.F203V,	14q12	.
14	29236837 G	A	CGCCCTGGAC,GGGCTAAAGC	exonic	FOXG1	nonsynonymous SNV	FOXG1:ENST00000382535.3:exon2:c.G352A:p.G118R,FOXG1:ENST00000313071.4:exon1:c.G352A:p.G118R,	14q12	.
14	29236838 G	C	GCCCTGGACG,GGCTAAAGCG	exonic	FOXG1	nonsynonymous SNV	FOXG1:ENST00000382535.3:exon2:c.G353C:p.G118A,FOXG1:ENST00000313071.4:exon1:c.G353C:p.G118A,	14q12	.
14	34400358 C	T	CGACCATCAC,GTTGGGGTTG	exonic	EGLN3	nonsynonymous SNV	EGLN3:ENST00000553215.1:exon2:c.G139A:p.G47S,EGLN3:ENST00000250457.3:exon2:c.G421A:p.G141S,EGLN3:ENST00000487915.2:exon5:c.G67A:p.G23S,	14q13.1	.
14	34561526 C	T	CAGACTCTTA,CATCTTTCTG	splicing	EGLN3(ENST	.	.	14q13.1	.
14	34561527 C	T	AGACTCTTAC,ATCTTTCTGC	exonic	EGLN3	nonsynonymous SNV	EGLN3:ENST00000487915.2:exon4:c.G3A:p.M1I,	14q13.1	.
14	35483027 C	T	CACAAAAAGT,CGATTATTTT	exonic	SRP54	nonsynonymous SNV	SRP54:ENST00000216774.6:exon10:c.C805T:p.P269S,SRP54:ENST00000555557.1:exon9:c.C613T:p.P205S,SRP54:ENST00000546080.1:exon9:c.C658T:p.P220S,SRP54:ENST00000556994.1:exon11:c.C805T:p.P269S,	14q13.2	.
14	37739703 G	A	GAAAGAAACA,AAGCTAAAAT	exonic	MIPOL1	nonsynonymous SNV	MIPOL1:ENST00000537471.1:exon6:c.G466A:p.E156K,MIPOL1:ENST00000539062.2:exon7:c.G373A:p.E125K,MIPOL1:ENST00000396294.2:exon8:c.G466A:p.E156K,MIPOL1:ENST00000555870.1:exon7:c.G373A:p.E125K,MIPOL1:ENST00000556451.1:exon8:c.G373A:p.E125K,MIPOL1:ENST00000545536.1:exon6	14q13.3	.
14	38091422 C	T	AAAGCCCAGT,CACAGCCTCC	exonic	TTC6	nonsynonymous SNV	TTC6:ENST00000556845.1:exon3:c.C14T:p.S5F,	14q21.1	.
14	44974375 C	T	TCAGCAGGAG,CTCTTCTGCA	exonic	FSCB	nonsynonymous SNV	FSCB:ENST00000340446.4:exon1:c.G1816A:p.A606T,	14q21.2	.
14	53360146 C	T	TGTCTGATAT,TGTTAATAAA	splicing	FERMT2(ENS	.	.	14q22.1	.
14	56763399 G	A	AACAGCAGAT,GGCTTTTTCA	exonic	PELI2	nonsynonymous SNV	PELI2:ENST00000267460.4:exon6:c.G778A:p.G260R,	14q22.3	.
14	58943881 T	C	CTCTGGGCTC,TTCCAGTCCA	exonic	KIAA0586	nonsynonymous SNV	KIAA0586:ENST00000261244.5:exon19:c.T2638C:p.F880L,KIAA0586:ENST00000556134.1:exon21:c.T2821C:p.F941L,KIAA0586:ENST00000423743.3:exon21:c.T2734C:p.F912L,KIAA0586:ENST00000354386.6:exon22:c.T3025C:p.F1009L,	14q23.1	.
14	59977505 C	T	TGCAAGGTCT,TTCACCATTG	exonic	CCDC175	nonsynonymous SNV	CCDC175:ENST00000281581.4:exon19:c.G2164A:p.E722K,CCDC175:ENST00000537690.2:exon19:c.G2164A:p.E722K,	14q23.1	.
14	64682025 C	T	CCTGAAAATC,TGAGGCATAT	exonic	SYNE2	nonsynonymous SNV	SYNE2:ENST00000358025.3:exon107:c.C19355T:p.P6452L,SYNE2:ENST00000555002.1:exon58:c.C9257T:p.P3086L,SYNE2:ENST00000458046.2:exon2:c.C257T:p.P86L,	14q23.2	.
14	69077786 C	T	GACTCACATC,CAGCCCTACC	exonic	RAD51B	nonsynonymous SNV	RAD51B:ENST00000487861.1:exon11:c.C1100T:p.P367L,	14q24.1	.
14	69791483 C	T	GAGGCCCGTT,CACCCTGCTG	exonic	GALNT16	nonsynonymous SNV	GALNT16:ENST00000337827.4:exon3:c.C410T:p.S137F,GALNT16:ENST00000448469.3:exon3:c.C410T:p.S137F,GALNT16:ENST00000553471.2:exon3:c.C410T:p.S137F,GALNT16:ENST00000553669.1:exon3:c.C410T:p.S137F,	14q24.1	.
14	69994013 G	A	TAAGGAGAAG,AGGAGAGGAT	exonic	PLEKHD1	nonsynonymous SNV	PLEKHD1:ENST00000322564.7:exon11:c.G1165A:p.E389K,	14q24.1	.
14	71201115 G	A	ATCTCCATAA,GCTGGTGAAC	exonic	MAP3K9	nonsynonymous SNV	MAP3K9:ENST00000555993.2:exon10:c.C2014T:p.L672F,MAP3K9:ENST00000554146.1:exon9:c.C1156T:p.L386F,MAP3K9:ENST00000381250.4:exon9:c.C1945T:p.L649F,MAP3K9:ENST00000553414.1:exon8:c.C1171T:p.L391F,MAP3K9:ENST00000554752.2:exon10:c.C2014T:p.L672F,	14q24.2	.
14	73989439 T	C	TTGGCAGAGC,TTCTGTCTTC	exonic	HEATR4	nonsynonymous SNV	HEATR4:ENST00000556455.1:exon2:c.A418G:p.S140G,HEATR4:ENST00000553558.1:exon3:c.A418G:p.S140G,HEATR4:ENST00000334988.2:exon2:c.A418G:p.S140G,HEATR4:ENST00000560393.1:exon2:c.A277G:p.S93G,	14q24.3	.
14	74876377 G	A	CTCCGGGTAG,GATAGGGGCC	exonic	SYNDIG1L	nonsynonymous SNV	SYNDIG1L:ENST00000554953.1:exon2:c.C71T:p.P24L,SYNDIG1L:ENST00000331628.3:exon2:c.C71T:p.P24L,SYNDIG1L:ENST00000554823.1:exon1:c.C71T:p.P24L,	14q24.3	.
14	75329304 G	A	ACCGAGGGAA,AAGGGGTAGA	exonic	PROX2	nonsynonymous SNV	PROX2:ENST00000556489.2:exon1:c.C1234T:p.L412F,PROX2:ENST00000445876.1:exon1:c.C1234T:p.L412F,	14q24.3	.
14	75376357 C	T	TTCACCTGCT,CTCTCTCACA	exonic	RPS6KL1	nonsynonymous SNV	RPS6KL1:ENST00000354625.2:exon6:c.G1066A:p.E356K,RPS6KL1:ENST00000358328.4:exon7:c.G1159A:p.E387K,RPS6KL1:ENST00000557413.1:exon8:c.G1159A:p.E387K,RPS6KL1:ENST00000555647.1:exon8:c.G1159A:p.E387K,RPS6KL1:ENST00000555009.1:exon6:c.G1066A:p.E356K,	14q24.3	.
14	75568374 G	A	AATAGCAGCT,TGTGAGTCTT	exonic	NEK9	nonsynonymous SNV	NEK9:ENST00000238616.5:exon15:c.C1826T:p.T609I,	14q24.3	.
14	76437944 G	A	GCTGGGGTTG,GCACCCGCAA	exonic	TGFB3	nonsynonymous SNV	TGFB3:ENST00000556285.1:exon2:c.C470T:p.P157L,TGFB3:ENST00000238682.3:exon2:c.C470T:p.P157L,	14q24.3	.
14	77872445 C	G	ATAGAACACT,CCTCCAACCA	exonic	NOXRED1	nonsynonymous SNV	NOXRED1:ENST00000380835.2:exon5:c.G716C:p.G239A,	14q24.3	.
14	77872446 C	T	TAGAACACTC,CTCCAACCAC	exonic	NOXRED1	nonsynonymous SNV	NOXRED1:ENST00000380835.2:exon5:c.G715A:p.G239R,	14q24.3	.
14	79433601 G	A	GTGATCAAAG,CAACAGTGAC	exonic	NRXN3	nonsynonymous SNV	NRXN3:ENST00000554719.1:exon10:c.G1709A:p.G570D,NRXN3:ENST00000554738.1:exon11:c.G2795A:p.G932D,NRXN3:ENST00000335750.5:exon10:c.G1709A:p.G570D,	14q31.1	.
14	80327787 G	T	GAAACTAGTA,GACTACTACC	exonic	NRXN3	nonsynonymous SNV	NRXN3:ENST00000557594.1:exon6:c.G1394T:p.R465M,	14q31.1	.
14	92908448 G	A	CCATCTGAGC,AAGATGTGGC	exonic	SLC24A4	nonsynonymous SNV	SLC24A4:ENST00000298877.1:exon5:c.G358A:p.E120K,SLC24A4:ENST00000393265.2:exon5:c.G217A:p.E73K,SLC24A4:ENST00000532405.1:exon5:c.G409A:p.E137K,SLC24A4:ENST00000554461.1:exon4:c.G82A:p.E28K,SLC24A4:ENST00000531433.1:exon6:c.G409A:p.E137K,SLC24A4:ENST00000351924.5:e	14q32.12	.
14	94931184 C	T	ATGAACACCT,TATCCACCTG	exonic	SERPINA9	nonsynonymous SNV	SERPINA9:ENST00000337425.5:exon4:c.G964A:p.E322K,SERPINA9:ENST00000380365.3:exon4:c.G910A:p.E304K,SERPINA9:ENST00000298845.7:exon5:c.G664A:p.E222K,SERPINA9:ENST00000448305.2:exon4:c.G670A:p.E224K,SERPINA9:ENST00000546329.1:exon4:c.G856A:p.E286K,SERPINA9:ENST0000	14q32.13	.
14	94962858 C	T	GAGAGCTTAT,GTCATAGCCA	exonic	SERPINA12	nonsynonymous SNV	SERPINA12:ENST00000556881.1:exon3:c.G757A:p.D253N,SERPINA12:ENST00000341228.2:exon4:c.G757A:p.D253N,	14q32.13	SERPINA12;basechange=c.757G>T;AAchange=p.D253Y;tissue=lung
14	95030331 A	G	TTCCACACCA,CTTCTACGAC	exonic	SERPINA4	nonsynonymous SNV	SERPINA4:ENST00000298841.5:exon2:c.A512G:p.N171S,SERPINA4:ENST00000557004.1:exon2:c.A512G:p.N171S,SERPINA4:ENST00000555095.1:exon2:c.A512G:p.N171S,	14q32.13	.
14	95080903 G	A	CAAGACCGAG,GACACACGTG	exonic	SERPINA3	nonsynonymous SNV	SERPINA3:ENST00000393080.4:exon2:c.G125A:p.G42E,SERPINA3:ENST00000393078.3:exon2:c.G125A:p.G42E,SERPINA3:ENST00000556968.1:exon2:c.G125A:p.G42E,SERPINA3:ENST00000467132.1:exon2:c.G125A:p.G42E,	14q32.13	.
14	100069812 C	T	GCTTGCGGCC,CCGTCGCCGC	exonic	CCDC85C	nonsynonymous SNV	CCDC85C:ENST00000380243.4:exon1:c.G485A:p.G162E,	14q32.2	.
14	100182494 G	A	AGCCCAGGAC,ACGAGGGTCT	exonic	CYP46A1	nonsynonymous SNV	CYP46A1:ENST00000554176.1:exon4:c.G406A:p.D136N,CYP46A1:ENST00000423126.2:exon9:c.G574A:p.D192N,CYP46A1:ENST00000556313.1:exon2:c.G124A:p.D42N,CYP46A1:ENST00000261835.3:exon9:c.G865A:p.D289N,	14q32.2	CYP46A1;basechange=c.865G>A;AAchange=p.D289N;tissue=large_intestine
14	100344859 C	T	ACGAGTGTCT,CTGGGGGTCG	exonic	EML1	nonsynonymous SNV	EML1:ENST00000334192.4:exon5:c.C478T:p.P160S,EML1:ENST00000327921.9:exon5:c.C385T:p.P129S,EML1:ENST00000262233.6:exon4:c.C421T:p.P141S,EML1:ENST00000554479.1:exon4:c.C382T:p.P128S,EML1:ENST00000556714.1:exon4:c.C328T:p.P110S,EML1:ENST00000555145.1:exon5:c.C439T:p.P1	14q32.2	.
14	100595927 C	T	AGGCTCCAGT,CCAGTGGGAC	exonic	EVL	nonsynonymous SNV	EVL:ENST00000554695.1:exon2:c.C190T:p.P64S,EVL:ENST00000557384.1:exon6:c.C427T:p.P143S,EVL:ENST00000402714.2:exon7:c.C739T:p.P247S,EVL:ENST00000544450.2:exon7:c.C757T:p.P253S,EVL:ENST00000392920.3:exon7:c.C745T:p.P249S,	14q32.2	.
14	100595928 C	T	GGCTCCAGTC,CAGTGGGACC	exonic	EVL	nonsynonymous SNV	EVL:ENST00000554695.1:exon2:c.C191T:p.P64L,EVL:ENST00000557384.1:exon6:c.C428T:p.P143L,EVL:ENST00000402714.2:exon7:c.C740T:p.P247L,EVL:ENST00000544450.2:exon7:c.C758T:p.P253L,EVL:ENST00000392920.3:exon7:c.C746T:p.P249L,	14q32.2	EVL;basechange=c.746C>T;AAchange=p.P249L;tissue=large_intestine
14	101347339 C	T	TCCTGCACGT,GTTGTCCTGC	exonic	RTL1	nonsynonymous SNV	RTL1:ENST00000534062.1:exon1:c.G3787A:p.D1263N,	14q32.2	.
14	101347510 G	A	CCCTCCTGGG,GGTGACTCTG	exonic	RTL1	nonsynonymous SNV	RTL1:ENST00000534062.1:exon1:c.C3616T:p.P1206S,	14q32.2	.
14	101349045 A	C	CATCTCTTCA,GCTCCAAACC	exonic	RTL1	nonsynonymous SNV	RTL1:ENST00000534062.1:exon1:c.T2081G:p.L694R,	14q32.2	.
14	102469176 C	T	TCCAAGTCTC,CCTTGTTATG	exonic	DYNC1H1	nonsynonymous SNV	DYNC1H1:ENST00000360184.4:exon23:c.C4757T:p.P1586L,	14q32.31	.
14	102729886 G	A	TACGTACCTC,TATTAGCTCA	exonic	MOK	stopgain	MOK:ENST00000517537.1:exon4:c.C280T:p.R94X,MOK:ENST00000522874.1:exon4:c.C280T:p.R94X,MOK:ENST00000361847.2:exon4:c.C280T:p.R94X,MOK:ENST00000524214.1:exon3:c.C190T:p.R64X,	14q32.31	.
14	105173895 C	T	ACCCCTGCTC,CTGGTTCCAG	exonic	INF2	nonsynonymous SNV	INF2:ENST00000392634.4:exon8:c.C1291T:p.P431S,INF2:ENST00000330634.7:exon8:c.C1291T:p.P431S,	14q32.33	.
14	105413188 G	A	CAGTTGGGCG,AGGGGGGCTG	exonic	AHNAK2	nonsynonymous SNV	AHNAK2:ENST00000333244.5:exon7:c.C8600T:p.S2867F,	14q32.33	.
14	105413945 C	T	ACGTCCACCT,CATGCTGGAC	exonic	AHNAK2	nonsynonymous SNV	AHNAK2:ENST00000333244.5:exon7:c.G7843A:p.E2615K,	14q32.33	.
14	106110435 T	A	ACACAACATT,GCGCTCTGCA	exonic	IGHG2	stopgain	IGHG2:ENST00000390545.2:exon2:c.A301T:p.K101X,	14q32.33	.
14	106815885 C	T	CCACTCCAGC,CCTTGCCTGG	exonic	IGHV3-33	nonsynonymous SNV	IGHV3-33:ENST00000390615.2:exon2:c.G188A:p.G63E,	14q32.33	.
14	106845493 C	A	AGCCCCTTTC,TGGAGCCTGA	exonic	IGHV3-35	stopgain	IGHV3-35:ENST00000390617.2:exon2:c.G181T:p.G61X,	14q32.33	.
14	107211243 C	T	CACTGGACAC,TGCAAACACA	splicing	IGHV3-73(EN	.	.	14q32.33	.
15	22382797 G	A	CTTCCTTGGA,GAGGGGAGGG	exonic	OR4N4	nonsynonymous SNV	OR4N4:ENST00000328795.4:exon1:c.G325A:p.G109R,	15q11.2	.
15	22382798 G	A	TTCCTTGGAG,AGGGGAGGGA	exonic	OR4N4	nonsynonymous SNV	OR4N4:ENST00000328795.4:exon1:c.G326A:p.G109E,	15q11.2	.
15	22473000 C	T	GACTTGTCTA,TGATATGGTG	exonic	IGHV4OR15-	nonsynonymous SNV	IGHV4OR15-8:ENST00000557788.2:exon2:c.G271A:p.V91I,	15q11.2	.
15	22962544 T	G	CAGAGGCATG,GCAGGTGAGC	exonic	CYFIP1	nonsynonymous SNV	CYFIP1:ENST00000560848.1:exon21:c.T2264G:p.V755G,CYFIP1:ENST00000435939.2:exon5:c.T971G:p.V324G,CYFIP1:ENST00000313077.7:exon20:c.T2264G:p.V755G,	15q11.2	.
15	23052678 C	T	CACGACGGAG,CTGCACAGCT	exonic	NIPA1	nonsynonymous SNV	NIPA1:ENST00000437912.2:exon4:c.G170A:p.G57D,NIPA1:ENST00000337435.4:exon4:c.G395A:p.G132D,NIPA1:ENST00000561183.1:exon4:c.G170A:p.G57D,	15q11.2	.
15	23889921 G	A	CACAACTACG,GCAGAGAGCT	exonic	MAGEL2	nonsynonymous SNV	MAGEL2:ENST00000532292.1:exon1:c.C1160T:p.P387L,	15q11.2	.
15	23890249 G	C	TTGCCGGAGC,GCGTGGCGGC	exonic	MAGEL2	nonsynonymous SNV	MAGEL2:ENST00000532292.1:exon1:c.C832G:p.R278G,	15q11.2	.
15	23890347 G	A	CACTGCCTGC,ATGCCTTTGA	exonic	MAGEL2	nonsynonymous SNV	MAGEL2:ENST00000532292.1:exon1:c.C734T:p.S245L,	15q11.2	MAGEL2_ENST00000532292;basechange=c.734C>T;AAchange=p.S245L;tissue=endometrium
15	23890770 G	A	GGAGCCCAGG,GAAAATTTGC	exonic	MAGEL2	nonsynonymous SNV	MAGEL2:ENST00000532292.1:exon1:c.C311T:p.P104L,	15q11.2	.
15	28000563 C	T	ACATGAGCCA,AAGGAGATAA	exonic	OCA2	nonsynonymous SNV	OCA2:ENST00000353809.5:exon23:c.G2416A:p.V806M,OCA2:ENST00000354638.3:exon24:c.G2488A:p.V830M,	15q12	.
15	29390738 A	C	GGACCTCTTC,TTTCCACCCA	exonic	APBA2	nonsynonymous SNV	APBA2:ENST00000561069.1:exon9:c.A1297C:p.I433L,APBA2:ENST00000558259.1:exon8:c.A1297C:p.I433L,APBA2:ENST00000411764.1:exon7:c.A1261C:p.I421L,APBA2:ENST00000558330.1:exon9:c.A1261C:p.I421L,APBA2:ENST00000558402.1:exon10:c.A1297C:p.I433L,	15q13.1	.
15	33944947 T	G	CTACATGGTC,GGGGTGGAGA	exonic	RYR3	nonsynonymous SNV	RYR3:ENST00000389232.4:exon32:c.T4171G:p.W1391G,RYR3:ENST00000415757.3:exon32:c.T4171G:p.W1391G,	15q14	.
15	33944948 G	T	TACATGGTCT,GGGTGGAGAC	exonic	RYR3	nonsynonymous SNV	RYR3:ENST00000389232.4:exon32:c.G4172T:p.W1391L,RYR3:ENST00000415757.3:exon32:c.G4172T:p.W1391L,	15q14	.
15	34331012 G	A	CCAGCCTCCC,GCTCCCGGCG	exonic	AVEN	nonsynonymous SNV	AVEN:ENST00000306730.3:exon1:c.C236T:p.P79L,	15q14	.
15	40331130 C	T	CTGGAAAAGT,TGGTCAGCTC	exonic	SRP14	nonsynonymous SNV	SRP14:ENST00000267884.6:exon2:c.G44A:p.R15K,SRP14:ENST00000559081.1:exon2:c.G44A:p.R15K,	15q15.1	.
15	42371829 T	G	CTCCCGGCGG,AGCTCGCCAG	exonic	PLA2G4D	nonsynonymous SNV	PLA2G4D:ENST00000290472.3:exon13:c.A1223C:p.Y408S,	15q15.1	.
15	43525532 G	A	TGGGGTTTGA,GACTCCAAGG	exonic	TGM5	nonsynonymous SNV	TGM5:ENST00000220420.5:exon13:c.C2020T:p.L674F,TGM5:ENST00000349114.4:exon12:c.C1774T:p.L592F,	15q15.2	.
15	43627938 G	A	CTCATACCAT,AAGAAATTAA	exonic	ADAL	nonsynonymous SNV	ADAL:ENST00000422466.2:exon5:c.G108A:p.M36I,ADAL:ENST00000389651.4:exon4:c.G108A:p.M36I,ADAL:ENST00000562188.1:exon2:c.G108A:p.M36I,ADAL:ENST00000428046.3:exon5:c.G108A:p.M36I,ADAL:ENST00000563551.1:exon3:c.G3A:p.M1I,	15q15.3	.
15	45814437 C	T	CCGAGAAAGC,CCTCGTCCCC	exonic	SLC30A4	nonsynonymous SNV	SLC30A4:ENST00000261867.4:exon2:c.G116A:p.G39E,	15q21.1	.
15	50211057 C	T	CCTGTTAGGA,CCAGATCTTA	exonic	ATP8B4	nonsynonymous SNV	ATP8B4:ENST00000559829.1:exon19:c.G2014A:p.V672I,ATP8B4:ENST00000284509.6:exon19:c.G2014A:p.V672I,ATP8B4:ENST00000557955.1:exon19:c.G2014A:p.V672I,	15q21.2	.
15	51634083 T	G	CCTGCGCTCC,TCCTGGCCGA	exonic	GLDN	nonsynonymous SNV	GLDN:ENST00000335449.6:exon1:c.T202G:p.F68V,	15q21.2	.
15	52901802 G	A	GTGTCTTCTG,GGAACTAAAC	exonic	FAM214A	nonsynonymous SNV	FAM214A:ENST00000546305.2:exon5:c.C1330T:p.P444S,FAM214A:ENST00000534964.2:exon6:c.C1309T:p.P437S,FAM214A:ENST00000261844.7:exon6:c.C1309T:p.P437S,FAM214A:ENST00000399202.4:exon5:c.C1045T:p.P349S,	15q21.3	.
15	53081320 G	A	CGTTCAGGTG,GCGTGGGGAT	exonic	ONECUT1	nonsynonymous SNV	ONECUT1:ENST00000570208.2:exon1:c.C274T:p.P92S,	15q21.3	.
15	54859996 A	G	TTTTCTTTGT,GCAATACTTT	splicing	UNC13C(ENS	.	.	15q21.3	.
15	54914587 G	A	TGCCACCCCA,GAACGGGAGA	exonic	UNC13C	nonsynonymous SNV	UNC13C:ENST00000260323.11:exon30:c.G6169A:p.G2057R,UNC13C:ENST00000537900.1:exon29:c.G6163A:p.G2055R,UNC13C:ENST00000545554.1:exon30:c.G6169A:p.G2057R,	15q21.3	.
15	57744370 G	A	GAGAGAGAGA,GATGAGAGCA	exonic	CGNL1	nonsynonymous SNV	CGNL1:ENST00000281282.5:exon6:c.G1937A:p.R646K,	15q21.3	.
15	62174835 G	C	TCTCTGGTGA,AAGACTGCTT	exonic	VPS13C	nonsynonymous SNV	VPS13C:ENST00000261517.5:exon69:c.C9584G:p.S3195C,VPS13C:ENST00000395896.4:exon69:c.C9584G:p.S3195C,VPS13C:ENST00000395898.3:exon67:c.C9455G:p.S3152C,VPS13C:ENST00000249837.3:exon67:c.C9455G:p.S3152C,	15q22.2	.
15	66824595 G	A	CTCCATAGTG,AAGTAACAGT	exonic	ZWILCH	nonsynonymous SNV	ZWILCH:ENST00000565627.1:exon12:c.G824A:p.G275E,ZWILCH:ENST00000446801.2:exon13:c.G824A:p.G275E,ZWILCH:ENST00000307897.5:exon13:c.G1166A:p.G389E,ZWILCH:ENST00000535141.2:exon12:c.G824A:p.G275E,	15q22.31	.
15	72581581 G	A	ATGGGCGCCG,AAGACCTGGG	exonic	CELF6	nonsynonymous SNV	CELF6:ENST00000567083.1:exon8:c.C928T:p.P310S,CELF6:ENST00000539635.1:exon7:c.C511T:p.P171S,CELF6:ENST00000543764.2:exon7:c.C580T:p.P194S,CELF6:ENST00000287202.5:exon8:c.C928T:p.P310S,CELF6:ENST00000569547.1:exon8:c.C928T:p.P310S,CELF6:ENST00000395258.2:exon6:c.C589	15q23	.
15	74426699 C	T	CTGTGTCCAG,GGGGGGCGGC	exonic	ISLR2	nonsynonymous SNV	ISLR2:ENST00000435464.1:exon4:c.C1604T:p.A535V,ISLR2:ENST00000419208.1:exon2:c.C1604T:p.A535V,ISLR2:ENST00000361742.3:exon4:c.C1604T:p.A535V,ISLR2:ENST00000565159.1:exon4:c.C1604T:p.A535V,ISLR2:ENST00000445793.1:exon4:c.C1604T:p.A535V,ISLR2:ENST00000565540.1:exon2:c.C16	15q24.1	.
15	74427006 G	A	CTGACCCTAT,GAGAAGCGCA	exonic	ISLR2	nonsynonymous SNV	ISLR2:ENST00000435464.1:exon4:c.G1911A:p.M637I,ISLR2:ENST00000419208.1:exon2:c.G1911A:p.M637I,ISLR2:ENST00000361742.3:exon4:c.G1911A:p.M637I,ISLR2:ENST00000565159.1:exon4:c.G1911A:p.M637I,ISLR2:ENST00000445793.1:exon4:c.G1911A:p.M637I,ISLR2:ENST00000565540.1:exon2:c.G191	15q24.1	.
15	74427007 G	A	TGACCCTATG,AGAAGCGCAT	exonic	ISLR2	nonsynonymous SNV	ISLR2:ENST00000435464.1:exon4:c.G1912A:p.E638K,ISLR2:ENST00000419208.1:exon2:c.G1912A:p.E638K,ISLR2:ENST00000361742.3:exon4:c.G1912A:p.E638K,ISLR2:ENST00000565159.1:exon4:c.G1912A:p.E638K,ISLR2:ENST00000445793.1:exon4:c.G1912A:p.E638K,ISLR2:ENST00000565540.1:exon2:c.G1	15q24.1	.
15	74473713 G	A	GTAGAGGGCA,AGAGGAGCAC	exonic	STRA6	nonsynonymous SNV	STRA6:ENST00000323940.5:exon17:c.C1607T:p.S536F,STRA6:ENST00000563965.1:exon17:c.C1724T:p.S575F,STRA6:ENST00000395105.4:exon17:c.C1607T:p.S536F,STRA6:ENST00000535552.1:exon17:c.C1718T:p.S573F,STRA6:ENST00000423167.2:exon17:c.C1580T:p.S527F,STRA6:ENST00000416286.3:e	15q24.1	.
15	74907961 G	C	CGTGCGCCGC,ACACGGCGGG	exonic	CLK3	nonsynonymous SNV	CLK3:ENST00000395066.3:exon1:c.G166C:p.D56H,	15q24.1	.
15	75014883 G	A	TACCTGTAGG,GTTAAAGTGC	exonic	CYP1A1	nonsynonymous SNV	CYP1A1:ENST00000562201.1:exon2:c.C556T:p.P186S,CYP1A1:ENST00000395048.2:exon2:c.C556T:p.P186S,CYP1A1:ENST00000567032.1:exon2:c.C556T:p.P186S,CYP1A1:ENST00000379727.3:exon2:c.C556T:p.P186S,CYP1A1:ENST00000395049.4:exon2:c.C556T:p.P186S,CYP1A1:ENST00000569630.1:exon	15q24.1	.
15	77908220 C	T	CCTCACGCCC,CCGCCAGCAT	exonic	LINGO1	nonsynonymous SNV	LINGO1:ENST00000570216.1:exon6:c.G11A:p.G4E,LINGO1:ENST00000564472.1:exon4:c.G11A:p.G4E,LINGO1:ENST00000559893.1:exon4:c.G11A:p.G4E,LINGO1:ENST00000566711.1:exon4:c.G11A:p.G4E,LINGO1:ENST00000355300.6:exon2:c.G29A:p.G10E,LINGO1:ENST00000567726.1:exon3:c.G11A:p.G4E,	15q24.3	.
15	78582378 C	T	GTTCACTTTC,CGACATGAGT	exonic	WDR61	nonsynonymous SNV	WDR61:ENST00000560569.1:exon6:c.G383A:p.G128E,WDR61:ENST00000559848.1:exon6:c.G383A:p.G128E,WDR61:ENST00000558459.1:exon4:c.G104A:p.G35E,WDR61:ENST00000267973.2:exon6:c.G383A:p.G128E,WDR61:ENST00000558311.1:exon6:c.G383A:p.G128E,	15q25.1	.
15	81592026 C	T	TCCTGTGGCT,CCAAGCCAGC	exonic	IL16	nonsynonymous SNV	IL16:ENST00000302987.4:exon13:c.C2359T:p.P787S,IL16:ENST00000559388.1:exon16:c.C2221T:p.P741S,IL16:ENST00000394660.2:exon14:c.C2359T:p.P787S,IL16:ENST00000394652.2:exon2:c.C256T:p.P86S,	15q25.1	.
15	83455283 C	T	ATGAAAACCT,CTCCAAATGA	exonic	FSD2	nonsynonymous SNV	FSD2:ENST00000334574.8:exon3:c.G715A:p.E239K,FSD2:ENST00000541889.1:exon3:c.G715A:p.E239K,	15q25.2	.
15	84286973 G	C	ATGAAGGAAT,ATACACGGAG	exonic	SH3GL3	nonsynonymous SNV	SH3GL3:ENST00000434347.1:exon9:c.G1002C:p.M334I,SH3GL3:ENST00000324537.5:exon12:c.G1002C:p.M334I,SH3GL3:ENST00000427482.2:exon9:c.G978C:p.M326I,	15q25.2	.
15	85401339 C	T	GCAGGCAGCC,CTGGACAGGG	exonic	ALPK3	nonsynonymous SNV	ALPK3:ENST00000258888.5:exon6:c.C3976T:p.P1326S,	15q25.3	.
15	89398533 C	T	GGTCTTACTT,CACAGTGGGC	exonic	ACAN	nonsynonymous SNV	ACAN:ENST00000439576.2:exon12:c.C2717T:p.S906F,ACAN:ENST00000559004.1:exon12:c.C2717T:p.S906F,ACAN:ENST00000561243.1:exon11:c.C2717T:p.S906F,ACAN:ENST00000352105.7:exon12:c.C2717T:p.S906F,	15q26.1	.
15	90784294 G	A	TGGCATCCCA,ATGCTCTGCC	exonic	GDPGP1	nonsynonymous SNV	GDPGP1:ENST00000329600.6:exon1:c.G154A:p.D52N,GDPGP1:ENST00000558017.1:exon4:c.G154A:p.D52N,GDPGP1:ENST00000559204.1:exon4:c.G154A:p.D52N,	15q26.1	.
16	318778 C	T	CTTCCAGGCT,ACACTGGGCG	exonic	ITFG3	nonsynonymous SNV	ITFG3:ENST00000301679.2:exon14:c.C1549T:p.H517Y,ITFG3:ENST00000442458.2:exon13:c.C1451T:p.S484L,ITFG3:ENST00000450082.2:exon14:c.C1549T:p.H517Y,ITFG3:ENST00000600536.1:exon12:c.C1451T:p.S484L,	16p13.3	.
16	341241 G	A	GTGCAGCACC,GCGCGCACGC	exonic	AXIN1	nonsynonymous SNV	AXIN1:ENST00000262320.3:exon9:c.C2243T:p.P748L,	16p13.3	.
16	622590 C	T	TCTGAGACCT,CAGGCATCCT	exonic	PIGQ	nonsynonymous SNV	PIGQ:ENST00000443147.1:exon1:c.C22T:p.P8S,	16p13.3	.
16	723526 G	C	CTGGCTCCGG,GGCTGCTGGG	exonic	RHOT2	nonsynonymous SNV	RHOT2:ENST00000315082.4:exon19:c.G1777C:p.G593R,	16p13.3	.
16	1250437 G	A	GCAGGCCGAG,GGGTGGGCGC	exonic	CACNA1H	nonsynonymous SNV	CACNA1H:ENST00000565831.1:exon6:c.G985A:p.G329R,CACNA1H:ENST00000358590.4:exon7:c.G985A:p.G329R,CACNA1H:ENST00000348261.5:exon7:c.G985A:p.G329R,	16p13.3	.
16	1257416 G	A	CCTCGTGGAG,GCTTCCAGGC	exonic	CACNA1H	nonsynonymous SNV	CACNA1H:ENST00000565831.1:exon13:c.G3049A:p.G1017S,CACNA1H:ENST00000358590.4:exon14:c.G3049A:p.G1017S,CACNA1H:ENST00000348261.5:exon14:c.G3049A:p.G1017S,	16p13.3	.
16	1272213 G	A	GCATCCCCGG,GCCCCGGGCA	exonic	TPSG1	nonsynonymous SNV	TPSG1:ENST00000234798.4:exon5:c.C640T:p.P214S,	16p13.3	.
16	1384676 G	A	AGACCCCGGG,AGCAGCGTTT	exonic	BAIAP3	nonsynonymous SNV	BAIAP3:ENST00000324385.5:exon1:c.G14A:p.G5E,	16p13.3	.
16	2812874 C	T	CTTTCAGGGT,TTCCCCATGC	exonic	SRRM2	nonsynonymous SNV	SRRM2:ENST00000571378.1:exon10:c.C2057T:p.S686F,SRRM2:ENST00000301740.8:exon11:c.C2345T:p.S782F,SRRM2:ENST00000576924.1:exon11:c.C2345T:p.S782F,	16p13.3	.
16	3016700 C	T	CGACCTGGCC,GGGGACGCCT	exonic	KREMEN2	nonsynonymous SNV	KREMEN2:ENST00000572045.1:exon5:c.C544T:p.R182W,KREMEN2:ENST00000575769.1:exon5:c.C544T:p.R182W,KREMEN2:ENST00000319500.6:exon5:c.C544T:p.R182W,KREMEN2:ENST00000303746.5:exon5:c.C544T:p.R182W,	16p13.3	.
16	3790551 C	T	GTCTGCAGCC,TAGGAAGTCC	splicing	CREBBP(EN:	.	.	16p13.3	.
16	4606795 C	T	CAGGACCTGC,CTCCACTGCC	exonic	C16orf96	nonsynonymous SNV	C16orf96:ENST00000444310.4:exon1:c.C305T:p.P102L,	16p13.3	.
16	4625632 G	A	CCACCACTGG,AGACTGGCCT	exonic	C16orf96	nonsynonymous SNV	C16orf96:ENST00000444310.4:exon5:c.G1151A:p.G384E,	16p13.3	.
16	8870282 G	A	CATCAAGCGG,AGGACCTGCT	exonic	ABAT	nonsynonymous SNV	ABAT:ENST00000425191.2:exon14:c.G1204A:p.E402K,ABAT:ENST00000569156.1:exon14:c.G1204A:p.E402K,ABAT:ENST00000268251.8:exon14:c.G1204A:p.E402K,ABAT:ENST00000567812.1:exon14:c.G1249A:p.E417K,ABAT:ENST00000396600.2:exon14:c.G1204A:p.E402K,	16p13.2	.
16	10769847 A	C	CTTATTCTTC,CATCCGTCAC	exonic	TEKT5	nonsynonymous SNV	TEKT5:ENST00000283025.2:exon5:c.T1055G:p.V352G,TEKT5:ENST00000576638.1:exon4:c.T374G:p.V125G,	16p13.13	.
16	11369974 C	T	ATGCCTCCTC,GGTGCCTGCA	exonic	PRM2	nonsynonymous SNV	PRM2:ENST00000435245.2:exon1:c.G254A:p.R85Q,PRM2:ENST00000241808.4:exon1:c.G254A:p.R85Q,	16p13.13	.
16	11570641 G	A	CAGCTCCACG,CCTCCTCAGG	exonic	CTD-3088G3.	nonsynonymous SNV	CTD-3088G3.8:ENST00000598234.1:exon18:c.C2606T:p.A869V,	16p13.13	.
16	14354967 C	T	TTGGATGGAA,TTTACCCTCA	exonic	MKL2	nonsynonymous SNV	MKL2:ENST00000574045.1:exon17:c.C2816T:p.T939I,MKL2:ENST00000341243.5:exon15:c.C2933T:p.T978I,MKL2:ENST00000318282.5:exon16:c.C2816T:p.T939I,MKL2:ENST00000571589.1:exon17:c.C2966T:p.T989I,	16p13.12	.
16	15977949 G	A	AGGTTTTCAT,AGACAATGAT	exonic	FOPNL	nonsynonymous SNV	FOPNL:ENST00000573396.1:exon2:c.C142T:p.H48Y,FOPNL:ENST00000573968.1:exon2:c.C142T:p.H48Y,FOPNL:ENST00000255759.6:exon2:c.C142T:p.H48Y,FOPNL:ENST00000575938.1:exon2:c.C61T:p.H21Y,FOPNL:ENST00000572415.1:exon2:c.C142T:p.H48Y,FOPNL:ENST00000573087.1:exon2:c.C142T:p.	16p13.11	.
16	17211812 C	T	AATAGCCTCT,CTTGGCATCC	exonic	XYLT1	nonsynonymous SNV	XYLT1:ENST00000261381.6:exon11:c.G2248A:p.E750K,	16p12.3	XYLT1;basechange=c.2248G>T;AAchange=p.E750*;tissue=lung
16	21608601 G	A	CACGAGCTGG,ATACCAAGTT	exonic	METTL9	nonsynonymous SNV	METTL9:ENST00000567404.1:exon1:c.G25A:p.D9N,	16p12.2	.
16	27689203 C	T	TGACCGCCCT,CTTCCAGTGG	exonic	KIAA0556	nonsynonymous SNV	KIAA0556:ENST00000261588.4:exon7:c.C694T:p.P232S,	16p12.1	.
16	28331958 A	G	CCGCGCGCGC,AGCCCCCCGG	exonic	SBK1	nonsynonymous SNV	SBK1:ENST00000341901.4:exon4:c.A991G:p.K331E,	16p11.2	.
16	29824626 C	T	ACAGCCCAGG,CACAGACCTC	exonic	PRRT2	nonsynonymous SNV	PRRT2:ENST00000358758.7:exon2:c.C251T:p.A84V,PRRT2:ENST00000567659.1:exon2:c.C251T:p.A84V,PRRT2:ENST00000300797.6:exon2:c.C251T:p.A84V,PRRT2:ENST00000562148.1:exon2:c.C251T:p.A84V,	16p11.2	.
16	30991072 C	T	GAGCCAGTGC,CGCACCCGCC	exonic	SETD1A	nonsynonymous SNV	SETD1A:ENST00000262519.8:exon14:c.C3965T:p.P1322L,	16p11.2	.
16	31422524 C	T	TCCGTGTGTC,CTTGCCTAGG	exonic	ITGAD	nonsynonymous SNV	ITGAD:ENST00000389202.2:exon13:c.C1484T:p.P495L,	16p11.2	.
16	47730340 C	T	TTTCTCTCTC,TTGTTGAAGA	exonic	PHKB	nonsynonymous SNV	PHKB:ENST00000299167.8:exon29:c.C2944T:p.L982F,PHKB:ENST00000455779.1:exon30:c.C2923T:p.L975F,PHKB:ENST00000566044.1:exon30:c.C2923T:p.L975F,PHKB:ENST00000323584.5:exon29:c.C2944T:p.L982F,	16q12.1	.
16	55893490 T	A	CTCGTGGTTA,TGACTCCGAT	exonic	CES5A	nonsynonymous SNV	CES5A:ENST00000521992.1:exon9:c.A1112T:p.N371I,CES5A:ENST00000520435.1:exon8:c.A935T:p.N312I,CES5A:ENST00000518005.1:exon8:c.A707T:p.N236I,CES5A:ENST00000319165.9:exon8:c.A1025T:p.N342I,CES5A:ENST00000290567.9:exon8:c.A1025T:p.N342I,	16q12.2	.
16	56686910 G	A	TGCCCCGTGG,CTGTGCCAAG	exonic	MT1B	nonsynonymous SNV	MT1B:ENST00000334346.2:exon3:c.G119A:p.G40D,	16q12.2	.
16	57085514 C	T	ACCTCAACTC,CTCAGCTGAG	exonic	NLRC5	nonsynonymous SNV	NLRC5:ENST00000436936.1:exon24:c.C3487T:p.P1163S,NLRC5:ENST00000308149.7:exon24:c.C3487T:p.P1163S,NLRC5:ENST00000538110.1:exon18:c.C1918T:p.P640S,NLRC5:ENST00000539144.1:exon22:c.C3487T:p.P1163S,NLRC5:ENST00000543030.1:exon16:c.C1294T:p.P432S,NLRC5:ENST0000053845	16q13	.
16	57685197 C	T	ACTACAAACC,ACACCAGACC	exonic	GPR56	nonsynonymous SNV	GPR56:ENST00000569154.1:exon3:c.C191T:p.P64L,GPR56:ENST00000565770.1:exon4:c.C191T:p.P64L,GPR56:ENST00000563374.1:exon4:c.C154T:p.H52Y,GPR56:ENST00000564907.1:exon3:c.C107T:p.P36L,GPR56:ENST00000566508.1:exon3:c.C107T:p.P36L,GPR56:ENST00000565539.1:exon3:c.C107T:p.P	16q21	.
16	66436860 G	A	GAAGAAGGAC,AGGCGGACCA	exonic	CDH5	nonsynonymous SNV	CDH5:ENST00000341529.3:exon12:c.G2143A:p.E715K,CDH5:ENST00000539168.1:exon6:c.G460A:p.E154K,	16q21	.
16	67396111 A	G	TGTCGCCCAG,CTGGAGTGCT	exonic	LRRC36	nonsynonymous SNV	LRRC36:ENST00000567723.1:exon3:c.A239G:p.D80G,	16q22.1	.
16	68024791 G	A	AGCCGAGTGG,AGAAGATCAC	exonic	DPEP2	nonsynonymous SNV	DPEP2:ENST00000572888.1:exon6:c.C842T:p.S281F,DPEP2:ENST00000412757.2:exon8:c.C842T:p.S281F,DPEP2:ENST00000393847.1:exon7:c.C842T:p.S281F,DPEP2:ENST00000575510.1:exon3:c.C218T:p.S73F,	16q22.1	.
16	68389630 C	T	TCGCAGCGTG,CCTGGACTTC	exonic	PRMT7	nonsynonymous SNV	PRMT7:ENST00000339507.5:exon17:c.C1655T:p.A552V,PRMT7:ENST00000449359.3:exon15:c.C1505T:p.A502V,PRMT7:ENST00000441236.1:exon16:c.C1505T:p.A502V,PRMT7:ENST00000348497.4:exon12:c.C1211T:p.A404V,	16q22.1	.
16	72831852 G	A	GATTCTTGAA,GGCTCTCTTT	exonic	ZFHX3	nonsynonymous SNV	ZFHX3:ENST00000397992.5:exon8:c.C1987T:p.L663F,ZFHX3:ENST00000268489.5:exon9:c.C4729T:p.L1577F,	16q22.2	.
16	72831854 G	C	TTCTTGAAGG,CTCTCTTTAA	exonic	ZFHX3	nonsynonymous SNV	ZFHX3:ENST00000397992.5:exon8:c.C1985G:p.A662G,ZFHX3:ENST00000268489.5:exon9:c.C4727G:p.A1576G,	16q22.2	.
16	75512738 G	T	CCAGGCCTGG,AGACGTTGAG	exonic	CHST6	nonsynonymous SNV	CHST6:ENST00000390664.2:exon4:c.C989A:p.S330Y,CHST6:ENST00000332272.4:exon3:c.C989A:p.S330Y,	16q23.1	.
16	78011569 G	A	CGAGAACAAG,AGCGGATGCC	exonic	VAT1L	nonsynonymous SNV	VAT1L:ENST00000302536.2:exon9:c.G1237A:p.E413K,	16q23.1	.
16	86565748 G	A	CTGCGCCGCG,GCCCTGCCAG	exonic	MTHFSD	nonsynonymous SNV	MTHFSD:ENST00000543303.2:exon8:c.C1018T:p.P340S,MTHFSD:ENST00000381214.5:exon8:c.C1021T:p.P341S,MTHFSD:ENST00000360900.6:exon8:c.C1021T:p.P341S,MTHFSD:ENST00000546093.1:exon9:c.C532T:p.P178S,MTHFSD:ENST00000322911.6:exon8:c.C1018T:p.P340S,	16q24.1	.
16	87969962 C	T	AGAACACCAT,GCCCTGGCCT	exonic	CA5A	nonsynonymous SNV	CA5A:ENST00000309893.2:exon1:c.G95A:p.R32Q,	16q24.2	.
16	88500191 C	T	TAGCCCAGCA,CCTCTGTCGG	exonic	ZNF469	nonsynonymous SNV	ZNF469:ENST00000437464.1:exon2:c.C6229T:p.P2077S,ZNF469:ENST00000565624.1:exon1:c.C6313T:p.P2105S,	16q24.2	.
16	88500192 C	T	AGCCCAGCAC,CTCTGTCGGG	exonic	ZNF469	nonsynonymous SNV	ZNF469:ENST00000437464.1:exon2:c.C6230T:p.P2077L,ZNF469:ENST00000565624.1:exon1:c.C6314T:p.P2105L,	16q24.2	.

17	54672251	C	T	CATCCAGTAC,CCATCATTTC	exonic	NOG	nonsynonymous SNV	NOG:ENST00000332822.4:exon1:c.C667T:p.P223S,	17q22	.
17	57046967	C	T	GCTATTTATG,CTCCATTCAC	exonic	PPM1E	nonsynonymous SNV	PPM1E:ENST00000308249.2:exon4:c.C851T:p.A284V,	17q22	.
17	59560638	G	A	CCAGCCACCA,GAAATGCCCA	exonic	TBX4	nonsynonymous SNV	TBX4:ENST00000240335.1:exon8:c.G1399A:p.G467R,TBX4:ENST00000393853.4:exon9:c.G1402A:p.G468R,	17q23.2	.
17	59560639	G	A	CAGCCACCAG,AAATGCCCAC	exonic	TBX4	nonsynonymous SNV	TBX4:ENST00000240335.1:exon8:c.G1400A:p.G467E,TBX4:ENST00000393853.4:exon9:c.G1403A:p.G468E,	17q23.2	.
17	60472440	G	A	TCCAGAAAAG,AGACCTGTTT	exonic	EFCAB3	nonsynonymous SNV	EFCAB3:ENST00000518576.1:exon6:c.G379A:p.E127K,EFCAB3:ENST00000520404.1:exon6:c.G379A:p.E127K,EFCAB3:ENST00000450662.2:exon8:c.G535A:p.E179K,EFCAB3:ENST00000305286.3:exon6:c.G379A:p.E127K,	17q23.2	.
17	62144122	G	A	GTCAGATAGC,CAGGGTCTCC	exonic	ERN1	nonsynonymous SNV	ERN1:ENST00000433197.3:exon8:c.C751T:p.R251C,	17q23.3	.
17	63221145	C	T	GCTCCCAGCC,CCATCTGACC	exonic	RGS9	nonsynonymous SNV	RGS9:ENST00000443584.3:exon18:c.C1424T:p.P475L,RGS9:ENST00000262406.9:exon18:c.C1433T:p.P478L,RGS9:ENST00000449996.3:exon18:c.C1424T:p.P475L,	17q24.1	.
17	63221164	C	T	CCGTGTACAC,GGGACCTGCA	exonic	RGS9	nonsynonymous SNV	RGS9:ENST00000584234.1:exon18:c.C1456T:p.R486W,	17q24.1	.
17	66872623	G	A	TGCCCCTCGG,GTCCATCCCG	exonic	ABCA8	nonsynonymous SNV	ABCA8:ENST00000586539.1:exon35:c.C4348T:p.P1450S,ABCA8:ENST00000269080.2:exon33:c.C4228T:p.P1410S,ABCA8:ENST00000430352.2:exon33:c.C4348T:p.P1450S,	17q24.2	.
17	68093060	G	T	CTTCAGAAGT,CAGAAAGGTA	exonic	KCNJ16	nonsynonymous SNV	KCNJ16:ENST00000586462.1:exon1:c.G17T:p.C6F,	17q24.3	.
17	73488702	C	T	CCTCACCTCC,TCAAACCCCT	exonic	KIAA0195	nonsynonymous SNV	KIAA0195:ENST00000314256.7:exon15:c.C1744T:p.L582F,KIAA0195:ENST00000580918.1:exon4:c.C628T:p.L210F,KIAA0195:ENST00000579208.1:exon7:c.C697T:p.L233F,KIAA0195:ENST00000375248.5:exon14:c.C1774T:p.L592F,	17q25.1	.
17	74729233	G	A	ATATATCTTG,GATCTTCTGG	exonic	METTL23,RP	stopgain	METTL23:ENST00000589977.1:exon3:c.G258A:p.W86X,METTL23:ENST00000591571.1:exon2:c.G57A:p.W19X,METTL23:ENST00000341249.6:exon3:c.G258A:p.W86X,METTL23:ENST00000592849.1:exon3:c.G258A:p.W86X,METTL23:ENST00000588783.1:exon3:c.G258A:p.W86X,METTL23:ENST00000588822.1:	17q25.1	.
17	74878370	C	T	CCTGCACTTT,CCGCAGACAG	exonic	MGAT5B	nonsynonymous SNV	MGAT5B:ENST00000565043.1:exon2:c.C352T:p.P118S,MGAT5B:ENST00000569840.2:exon3:c.C319T:p.P107S,MGAT5B:ENST00000301618.4:exon3:c.C319T:p.P107S,MGAT5B:ENST00000565675.1:exon3:c.C319T:p.P107S,MGAT5B:ENST00000428789.2:exon2:c.C352T:p.P118S,	17q25.2	.
17	74928783	G	A	CAACGAGACG,AGAAGCGGCT	exonic	MGAT5B	nonsynonymous SNV	MGAT5B:ENST00000569840.2:exon11:c.G1348A:p.E450K,MGAT5B:ENST00000301618.4:exon11:c.G1348A:p.E450K,MGAT5B:ENST00000428789.2:exon10:c.G1381A:p.E461K,	17q25.2	.
17	76128190	C	T	AGAGTGGCAT,ATCCCACCTG	exonic	TMC8	nonsynonymous SNV	TMC8:ENST00000590799.1:exon3:c.C377T:p.S126L,	17q25.3	.
17	76499003	G	A	TGCTCCCTCG,CTTCTCTTCG	exonic	DNAH17	nonsynonymous SNV	DNAH17:ENST00000585328.1:exon32:c.C5038T:p.P1680S,DNAH17:ENST00000389840.5:exon32:c.C5035T:p.P1679S,	17q25.3	DNAH17;basechange=c.5038C>G;AAchange=p.P1680A;tissue=lung
17	76832403	G	A	GAGTCCTTGC,GCCGGGTTTC	exonic	USP36	nonsynonymous SNV	USP36:ENST00000592275.1:exon3:c.C43T:p.R15C,USP36:ENST00000589225.1:exon3:c.C43T:p.R15C,USP36:ENST00000586896.2:exon2:c.C43T:p.R15C,USP36:ENST00000591942.2:exon3:c.C43T:p.R15C,USP36:ENST00000589424.1:exon3:c.C43T:p.R15C,USP36:ENST00000588365.1:exon3:c.C43T:p.R15C,U	17q25.3	.
17	79782278	G	A	TGGAGACGGG,GAGCTGGGGG	exonic	FAM195B	nonsynonymous SNV	FAM195B:ENST00000576679.1:exon1:c.C23T:p.P8L,	17q25.3	.
17	79975224	C	T	GGTGCCCACT,CGCCAGCCAC	exonic	ASPSCR1	nonsynonymous SNV	ASPSCR1:ENST00000583744.1:exon7:c.C484T:p.P162S,	17q25.3	.
17	80046174	G	A	AGGAGACTCG,AGCTGGTGTC	exonic	FASN	nonsynonymous SNV	FASN:ENST00000306749.2:exon17:c.C2603T:p.S868F,	17q25.3	.
17	81009639	C	T	GCCTGGCTCT,CTCGGACGCC	exonic	B3GNTL1	nonsynonymous SNV	B3GNTL1:ENST00000571218.1:exon1:c.G34A:p.E12K,B3GNTL1:ENST00000320865.3:exon1:c.G34A:p.E12K,	17q25.3	.
18	2707619	G	A	GCCTAATGAG,TTAGGCCTGC	exonic	SMCHD1	nonsynonymous SNV	SMCHD1:ENST00000577880.1:exon5:c.G535A:p.V179I,SMCHD1:ENST00000320876.6:exon16:c.G2122A:p.V708I,SMCHD1:ENST00000261598.8:exon16:c.G2122A:p.V708I,	18p11.32	.
18	2885077	C	T	TCCCAAAGAC,CCGTGAAGAC	exonic	EMILIN2	nonsynonymous SNV	EMILIN2:ENST00000254528.3:exon3:c.C373T:p.P125S,	18p11.32	.
18	3079302	C	T	TCCAGTGACC,CCGGTCTTAAC	exonic	MYOM1	nonsynonymous SNV	MYOM1:ENST00000261606.7:exon33:c.G4235A:p.G1412E,MYOM1:ENST00000400569.3:exon34:c.G4523A:p.G1508E,MYOM1:ENST00000356443.4:exon34:c.G4523A:p.G1508E,	18p11.31	MYOM1;basechange=c.4523G>A;AAchange=p.G1508E;tissue=liver
18	7080433	G	A	TTGCTGGCAA,ATTGAGAATG	exonic	LAMA1	nonsynonymous SNV	LAMA1:ENST00000389658.3:exon2:c.C85T:p.L29F,	18p11.31	.
18	7080439	G	A	GCAAGATTGA,AATGGCAGGA	exonic	LAMA1	nonsynonymous SNV	LAMA1:ENST00000389658.3:exon2:c.C79T:p.L27F,	18p11.31	.
18	9887812	G	A	GGAGTTCCCG,AGGGGGACAA	exonic	TXNDC2	nonsynonymous SNV	TXNDC2:ENST00000357775.5:exon2:c.G1135A:p.E379K,TXNDC2:ENST00000306084.6:exon2:c.G1336A:p.E446K,	18p11.22	.
18	12420333	G	A	GCCACCCGTG,GACACGGTCA	exonic	SLMO1	stopgain	SLMO1:ENST00000336990.4:exon2:c.G42A:p.W14X,SLMO1:ENST00000587862.1:exon2:c.G105A:p.W35X,SLMO1:ENST00000588729.1:exon3:c.G183A:p.W61X,SLMO1:ENST00000440960.1:exon2:c.G42A:p.W14X,	18p11.21	.
18	19057152	C	T	TGTCACATAG,GAACCCAGTC	exonic	GREB1L	nonsynonymous SNV	GREB1L:ENST00000400483.4:exon16:c.C2210T:p.A737V,	18q11.2	.
18	19075742	C	T	AGAAACCTTT,CCAGGTACAG	exonic	GREB1L	nonsynonymous SNV	GREB1L:ENST00000424526.1:exon20:c.C3142T:p.P1048S,GREB1L:ENST00000269218.6:exon19:c.C2815T:p.P939S,GREB1L:ENST00000580732.2:exon20:c.C3142T:p.P1048S,	18q11.2	.
18	20602172	C	T	ACATTCCACC,AACACACCAG	exonic	RBBP8	nonsynonymous SNV	RBBP8:ENST00000399725.2:exon17:c.C2438T:p.P813L,RBBP8:ENST00000583057.1:exon7:c.C809T:p.P270L,	18q11.2	.
18	32398181	C	T	GATGGGATTT,GCTACCGATG	exonic	DTNA	nonsynonymous SNV	DTNA:ENST00000590727.1:exon1:c.C13T:p.R5C,DTNA:ENST00000554864.3:exon9:c.C763T:p.R255C,DTNA:ENST00000283365.9:exon9:c.C763T:p.R255C,DTNA:ENST00000595022.1:exon9:c.C763T:p.R255C,DTNA:ENST00000598334.1:exon9:c.C763T:p.R255C,DTNA:ENST00000444659.1:exon7:c.C763T:p.R25	18q12.1	DTNA_ENST00000315456;basechange=c.763C>T;AAchange=p.R255C;tissue=oesophagus&DTNA_ENST00000269190;basechange=c.763C>T;AAchange=p.R255C;tissue=oesophagus&AC022601.1_ENST00000554864;basechange=c.763C>T;AAchange=p.R255C;tissue=oesophagus&AC022601.1;basechang
18	44260190	C	T	AAGAGGTGCA,CTCCTCACAG	exonic	ST8SIA5	nonsynonymous SNV	ST8SIA5:ENST00000536490.1:exon6:c.G853A:p.V285M,ST8SIA5:ENST00000315087.7:exon7:c.G946A:p.V316M,ST8SIA5:ENST00000538168.1:exon8:c.G1054A:p.V352M,	18q21.1	.
18	44773804	C	T	GGCCCCTGCC,CGGCAGCTGC	exonic	SKOR2	nonsynonymous SNV	SKOR2:ENST00000425639.1:exon1:c.G1751A:p.G584E,	18q21.1	.
18	45566692	C	T	GCACCGAAGT,CCCTGGCCAG	exonic	ZBTB7C	nonsynonymous SNV	ZBTB7C:ENST00000586438.1:exon2:c.G787A:p.D263N,ZBTB7C:ENST00000535628.2:exon2:c.G787A:p.D263N,ZBTB7C:ENST00000590800.1:exon4:c.G787A:p.D263N,ZBTB7C:ENST00000332053.2:exon1:c.G787A:p.D263N,ZBTB7C:ENST00000588982.1:exon3:c.G787A:p.D263N,	18q21.1	.
18	48702836	G	A	GTGGCCACAT,GAACTAGGGC	exonic	MEX3C	nonsynonymous SNV	MEX3C:ENST00000406189.3:exon2:c.C1865T:p.P622L,	18q21.2	.
18	65180300	C	T	AGTTGACCTT,CCAGGGCTTA	exonic	DSEL	nonsynonymous SNV	DSEL:ENST00000310045.7:exon2:c.G1576A:p.E526K,	18q22.1	.
18	74625824	G	A	TTCCTCTGGG,TCCTCAAGTC	exonic	ZNF236	nonsynonymous SNV	ZNF236:ENST00000543926.2:exon18:c.G3025A:p.V1009I,ZNF236:ENST00000320610.9:exon18:c.G3031A:p.V1011I,ZNF236:ENST00000253159.8:exon18:c.G3025A:p.V1009I,	18q23	.
19	548959	C	T	ACCATCCGGC,GTTGGCCCTG	exonic	GZMM	nonsynonymous SNV	GZMM:ENST00000264553.3:exon4:c.C386T:p.P129L,	19p13.3	.
19	808728	C	T	GAACATATTC,CGCCCTCGGC	exonic	PTBP1	nonsynonymous SNV	PTBP1:ENST00000356948.6:exon13:c.C1429T:p.P477S,PTBP1:ENST00000394601.4:exon13:c.C1408T:p.P470S,PTBP1:ENST00000349038.4:exon12:c.C1351T:p.P451S,PTBP1:ENST00000350092.4:exon5:c.C349T:p.P117S,	19p13.3	.
19	966601	C	T	CATCACAGTC,CTGGCCGCCT	exonic	ARID3A	nonsynonymous SNV	ARID3A:ENST00000263620.3:exon7:c.C1228T:p.P410S,	19p13.3	.
19	1005001	G	A	GTACCTCGTG,GTGACGGCAA	exonic	GRIN3B	nonsynonymous SNV	GRIN3B:ENST00000234389.3:exon3:c.G1501A:p.G501S,	19p13.3	.
19	1556277	G	A	GTCCGGCACG,AGGGGCCGCC	exonic	MEX3D	nonsynonymous SNV	MEX3D:ENST00000402693.4:exon2:c.C1241T:p.S414F,MEX3D:ENST00000388824.6:exon2:c.C1241T:p.S414F,MEX3D:ENST00000605173.1:exon2:c.C713T:p.S238F,	19p13.3	.
19	1806633	C	T	TCACCTCTTC,CCATCAGAAT	exonic	ATP8B3	nonsynonymous SNV	ATP8B3:ENST00000539485.1:exon7:c.G671A:p.G224E,ATP8B3:ENST00000525591.1:exon7:c.G512A:p.G171E,ATP8B3:ENST00000526092.2:exon7:c.G512A:p.G171E,ATP8B3:ENST00000310127.6:exon7:c.G671A:p.G224E,	19p13.3	.
19	1912834	C	T	AGACCCTTCC,CGCCTGCTCC	exonic	ADAT3	nonsynonymous SNV	ADAT3:ENST00000329478.2:exon2:c.C788T:p.P263L,ADAT3:ENST00000602400.1:exon2:c.C740T:p.P247L,	19p13.3	.
19	2269437	C	T	CGGCTCTGCC,CTCGTGCGGT	exonic	JSRP1	nonsynonymous SNV	JSRP1:ENST00000593238.2:exon1:c.G262A:p.G88R,	19p13.3	.
19	2877790	G	A	GAGTCCATAT,ATCATGCACG	exonic	ZNF556	nonsynonymous SNV	ZNF556:ENST00000307635.2:exon4:c.G834A:p.M278I,ZNF556:ENST00000586426.1:exon4:c.G831A:p.M277I,	19p13.3	.
19	3019413	G	A	GCTGGGCGGG,GGTGAGGGGC	exonic	TLE2	nonsynonymous SNV	TLE2:ENST00000592584.1:exon8:c.C421T:p.P141S,TLE2:ENST00000426948.2:exon7:c.C460T:p.P154S,TLE2:ENST00000262953.6:exon7:c.C418T:p.P140S,TLE2:ENST00000590536.1:exon7:c.C421T:p.P141S,TLE2:ENST00000591529.1:exon8:c.C460T:p.P154S,TLE2:ENST00000586422.1:exon5:c.C253T:p.P85S,	19p13.3	.
19	3611935	G	A	CGCCGATAGC,GTAGCGCTTG	exonic	CACTIN	nonsynonymous SNV	CACTIN:ENST00000221899.3:exon10:c.C2059T:p.R687C,CACTIN:ENST00000429344.2:exon10:c.C2263T:p.R755C,CACTIN:ENST00000585942.1:exon10:c.C2263T:p.R755C,CACTIN:ENST00000591726.1:exon1:c.C340T:p.R114C,CACTIN:ENST00000248420.5:exon10:c.C2263T:p.R755C,	19p13.3	.
19	3982878	C	T	CTCCACGATG,GCTGGAAAGT	exonic	EEF2	nonsynonymous SNV	EEF2:ENST00000309311.6:exon4:c.G539A:p.R180H,	19p13.3	.
19	4054459	G	A	ACCGGCGGCG,AAAGAGACCC	exonic	ZBTB7A	nonsynonymous SNV	ZBTB7A:ENST00000601588.1:exon2:c.C772T:p.P258S,ZBTB7A:ENST00000322357.4:exon2:c.C772T:p.P258S,	19p13.3	.
19	7584729	C	T	CCGTGGCATC,ACACTGGGGC	exonic	ZNF358	nonsynonymous SNV	ZNF358:ENST00000597229.1:exon2:c.C601T:p.H201Y,ZNF358:ENST00000394341.2:exon1:c.C601T:p.H201Y,	19p13.2	.
19	8152712	G	A	GGCCTACCTC,ACACATGGCC	exonic	FBN3	stopgain	FBN3:ENST00000600128.1:exon53:c.C6616T:p.R2206X,FBN3:ENST00000601739.1:exon53:c.C6616T:p.R2206X,FBN3:ENST00000270509.2:exon52:c.C6616T:p.R2206X,	19p13.2	.
19	8155018	C	T	GCAGGGGTCT,CCCAGCCCTC	exonic	FBN3	nonsynonymous SNV	FBN3:ENST00000600128.1:exon49:c.G6149A:p.G2050E,FBN3:ENST00000601739.1:exon49:c.G6149A:p.G2050E,FBN3:ENST00000270509.2:exon48:c.G6149A:p.G2050E,	19p13.2	.
19	8486860	C	T	AAGGGATGGC,GGCTCCTCTC	exonic	MARCH2	nonsynonymous SNV	MARCH2:ENST00000393944.1:exon3:c.C136T:p.R46W,MARCH2:ENST00000595142.1:exon3:c.C136T:p.R46W,MARCH2:ENST00000381035.4:exon2:c.C136T:p.R46W,MARCH2:ENST00000601724.1:exon3:c.C136T:p.R46W,MARCH2:ENST00000595213.1:exon3:c.C136T:p.R46W,MARCH2:ENST00000215555.2:e	19p13.2	.
19	8651605	G	A	TCCCTTCAGG,CTGGGGGACG	exonic	ADAMTS10	nonsynonymous SNV	ADAMTS10:ENST00000597188.1:exon20:c.C2240T:p.A747V,ADAMTS10:ENST00000270328.4:exon19:c.C2240T:p.A747V,ADAMTS10:ENST00000595838.1:exon7:c.C701T:p.A234V,	19p13.2	.
19	8668620	C	T	ACCTCTCACT,CACAGGCTGT	exonic	ADAMTS10	nonsynonymous SNV	ADAMTS10:ENST00000593913.1:exon3:c.G584A:p.G195E,ADAMTS10:ENST00000597188.1:exon5:c.G584A:p.G195E,ADAMTS10:ENST00000270328.4:exon4:c.G584A:p.G195E,ADAMTS10:ENST00000596851.1:exon4:c.G584A:p.G195E,	19p13.2	.
19	8807916	G	A	GATCCATACG,AGAAATTCCT	exonic	ACTL9	nonsynonymous SNV	ACTL9:ENST00000324436.3:exon1:c.C1136T:p.S379F,	19p13.2	ACTL9;basechange=c.1136C>T;AAchange=p.S379F;tissue=NS
19	8976335	C	T	TGACTCAGCT,CCAGTAAAGC	exonic	MUC16	nonsynonymous SNV	MUC16:ENST00000397910.4:exon75:c.G42493A:p.E14165K,MUC16:ENST00000380951.5:exon27:c.G2416A:p.E806K,	19p13.2	.
19	9011423	C	T	TCTGTTGAGT,CAGGGCTTTT	exonic	MUC16	nonsynonymous SNV	MUC16:ENST00000397910.4:exon36:c.G38810A:p.G12937E,	19p13.2	.
19	9076809	C	T	TGAGTTGGTT,CCATGGTGGT	exonic	MUC16	nonsynonymous SNV	MUC16:ENST00000397910.4:exon3:c.G10637A:p.G3546E,	19p13.2	.
19	9362575	C	T	GTTACCATCC,CCAGGAAGAG	exonic	OR7E24	nonsynonymous SNV	OR7E24:ENST00000456448.1:exon1:c.C856T:p.P286S,	19p13.2	.
19	9406375	G	A	GAAGAATGAC,AAATGCTTTC	exonic	ZNF699	nonsynonymous SNV	ZNF699:ENST00000308650.3:exon5:c.C1705T:p.R569C,ZNF699:ENST00000591998.1:exon6:c.C1705T:p.R569C,	19p13.2	.
19	9770108	C	T	TTTGTCTTTT,TTCAAAAGGA	exonic	ZNF562	nonsynonymous SNV	ZNF562:ENST00000585688.1:exon3:c.G61A:p.E21K,ZNF562:ENST00000453372.2:exon3:c.G61A:p.E21K,ZNF562:ENST00000448622.1:exon3:c.G61A:p.E21K,ZNF562:ENST00000590155.1:exon3:c.G61A:p.E21K,ZNF562:ENST00000589542.1:exon3:c.G61A:p.E21K,ZNF562:ENST00000587392.1:exon3:c.G61A:p.E2	19p13.2	.
19	10197608	G	A	ATCCCAGCTG,AGAAGAGCGT	exonic	C19orf66	nonsynonymous SNV	C19orf66:ENST00000253110.11:exon2:c.G25A:p.E9K,C19orf66:ENST00000591813.1:exon2:c.G25A:p.E9K,C19orf66:ENST00000587710.1:exon2:c.G25A:p.E9K,	19p13.2	.
19	11350480	C	T	TACAGGACCA,GGAGGGACGG	exonic	C19orf80	nonsynonymous SNV	C19orf80:ENST00000252453.8:exon1:c.C167T:p.T56M,	19p13.2	.
19	12224421	C	T	CCTTCAAAAT,ATGGAAGGAC	exonic	ZNF788	nonsynonymous SNV	ZNF788:ENST00000397759.3:exon3:c.C667T:p.H223Y,	19p13.2	.
19	13566011	C	T	TGGCTAAAAT,ATATATTCAA	exonic	CACNA1A	nonsynonymous SNV	CACNA1A:ENST00000573710.2:exon2:c.G309A:p.M103I,CACNA1A:ENST00000360228.5:exon2:c.G309A:p.M103I,	19p13.2	CACNA1A;basechange=c.309G>A;AAchange=p.M103I;tissue=lung&CACNA1A_ENST00000357018;basechange=c.309G>A;AAchange=p.M103I;tissue=lung&CACNA1A_ENST00000360228;basechange=c.309G>A;AAchange=p.M103I;tissue=lung&CACNA1A_ENST00000325084;basechange=c.309G>A;AAcha
19	14707699	C	T	AACTCCAGTC,CCTGGCTCAA	exonic	CLEC17A	nonsynonymous SNV	CLEC17A:ENST00000551730.1:exon9:c.C457T:p.P153S,CLEC17A:ENST00000339847.5:exon9:c.C457T:p.P153S,CLEC17A:ENST00000417570.1:exon9:c.C457T:p.P153S,CLEC17A:ENST00000547437.1:exon9:c.C457T:p.P153S,CLEC17A:ENST00000397439.2:exon8:c.C406T:p.P136S,	19p13.12	.
19	14707700	C	T	ACTCCAGTCC,CTGGCTCAAT	exonic	CLEC17A	nonsynonymous SNV	CLEC17A:ENST00000551730.1:exon9:c.C458T:p.P153L,CLEC17A:ENST00000339847.5:exon9:c.C458T:p.P153L,CLEC17A:ENST00000417570.1:exon9:c.C458T:p.P153L,CLEC17A:ENST00000547437.1:exon9:c.C458T:p.P153L,CLEC17A:ENST00000397439.2:exon8:c.C407T:p.P136L,	19p13.12	.
19	15224376	C	A	TTCAGATCCC,GCCTGCCCCG	exonic	SYDE1	nonsynonymous SNV	SYDE1:ENST00000342784.2:exon8:c.C1810A:p.R604S,SYDE1:ENST00000600440.1:exon8:c.C1609A:p.R537S,SYDE1:ENST00000600252.1:exon5:c.C781A:p.R261S,	19p13.12	.
19	15281506	G	A	AGTGGGTACG,GAAGTCCAGG	exonic	NOTCH3	nonsynonymous SNV	NOTCH3:ENST00000263388.2:exon26:c.C4867T:p.P1623S,	19p13.12	.
19	17036106	C	T	CAAAGTGGAT,ATGTTCTGCT	exonic	CPAMD8	nonsynonymous SNV	CPAMD8:ENST00000443236.1:exon26:c.C3588A:p.M1196I,	19p13.11	.
19	17137551	G	A	CCGCCGCCGG,GGCCCTTTGT	exonic	CPAMD8	nonsynonymous SNV	CPAMD8:ENST00000291440.3:exon1:c.C43T:p.P15S,CPAMD8:ENST00000443236.1:exon1:c.C43T:p.P15S,	19p13.11	.
19	17444307	G	A	ATGAGCTCGG,AATGCCCACG	exonic	ANO8	nonsynonymous SNV	ANO8:ENST00000159087.4:exon3:c.C274T:p.P92S,ANO8:ENST00000597643.1:exon3:c.C274T:p.P92S,	19p13.11	.
19	17651302	G	A	AGCCTCGCTC,AGGCGGTGCG	exonic	FAM129C	nonsynonymous SNV	FAM129C:ENST00000449408.2:exon8:c.G352A:p.E118K,FAM129C:ENST00000599124.1:exon9:c.G1081A:p.E361K,FAM129C:ENST00000595684.1:exon10:c.G1174A:p.E392K,FAM129C:ENST00000600871.1:exon9:c.G1012A:p.E338K,FAM129C:ENST00000332386.5:exon10:c.G1174A:p.E392K,FAM129C:ENST00	19p13.11	.
19	18084773	C	T	ACGAGACCCT,CGGACCCTGA	exonic	KCNN1	nonsynonymous SNV	KCNN1:ENST00000609922.1:exon2:c.C325T:p.P109S,KCNN1:ENST00000222249.9:exon3:c.C76T:p.P26S,	19p13.11	.
19	18895743	G	A	AGCACGGAAG,GGTTCGCCTG	exonic	COMP	nonsynonymous SNV	COMP:ENST00000222271.2:exon16:c.C1877T:p.P626L,COMP:ENST00000425807.1:exon15:c.C1718T:p.P573L,COMP:ENST00000542601.2:exon15:c.C1778T:p.P593L,	19p13.11	.
19	19603180	C	T	AACGAGCAGC,CTCGAGCCCG	exonic	GATAD2A	nonsynonymous SNV	GATAD2A:ENST00000417582.2:exon3:c.C335T:p.P112L,GATAD2A:ENST00000457895.2:exon3:c.C335T:p.P112L,GATAD2A:ENST00000429242.3:exon3:c.C335T:p.P112L,GATAD2A:ENST00000360315.3:exon3:c.C335T:p.P112L,GATAD2A:ENST00000252577.5:exon3:c.C335T:p.P112L,GATAD2A:ENST0000404	19p13.11	.
19	19654650	C	G	GCTGCGGGGA,GCCAGCTCCC	exonic	CILP2	nonsynonymous SNV	CILP2:ENST00000291495.5:exon8:c.C1296G:p.D432E,CILP2:ENST00000586018.1:exon8:c.C1314G:p.D438E,	19p13.11	CILP2;basechange=c.1296C>T;AAchange=p.D432D;tissue=large_intestine
19	19654765	C	T	GTGTCTGCCC,CTCGGGGGCT	exonic	CILP2	nonsynonymous SNV	CILP2:ENST00000291495.5:exon8:c.C1411T:p.P471S,CILP2:ENST00000586018.1:exon8:c.C1429T:p.P477S,	19p13.11	.
19	19757128	G	A	GTTGGGCAGG,GCCGTTCTCG	exonic	ATP13A1	nonsynonymous SNV	ATP13A1:ENST00000357324.6:exon23:c.C3134T:p.P1045L,ATP13A1:ENST00000291503.5:exon23:c.C2780T:p.P927L,	19p13.11	.
19	20733984	T	C	GTAATTCCAG,ACTTTGGGAG	exonic	ZNF737	nonsynonymous SNV	ZNF737:ENST00000596797.1:exon4:c.A307G:p.T103A,	19p12	.
19	20989241	C	T	TAAGAGAATT,ATACTGGAGA	exonic	ZNF66	nonsynonymous SNV	ZNF66:ENST00000425625.1:exon4:c.C973T:p.H325Y,ZNF66:ENST00000344519.8:exon4:c.C835T:p.H279Y,	19p12	.
19	21128371	G	A	GCTCACTGCA,CCTCCGCCTC	exonic	ZNF85	nonsynonymous SNV	ZNF85:ENST00000595854.1:exon4:c.G296A:p.S99N,	19p12	.
19	22156602	G	T	TGTTTAGTAA,GATTGAGAAC	exonic	ZNF208	nonsynonymous SNV	ZNF208:ENST00000397126.4:exon4:c.C1234A:p.L412I,	19p12	.
19	22939574	C	A	AAAAGCTTTG,CACATTCTTC	exonic	ZNF99	nonsynonymous SNV	ZNF99:ENST00000397104.3:exon7:c.G2597T:p.G866V,	19p12	.
19	23543729	T	A	ATTTGTAGGG,TTCTCTTCAG	exonic	ZNF91	nonsynonymous SNV	ZNF91:ENST00000397082.2:exon3:c.A1956T:p.K652N,ZNF91:ENST00000300619.7:exon4:c.A2052T:p.K684N,	19p12	.
19	30935764	C	T	AGGTACCTCT,CTGCCTGCAG	exonic	ZNF536	nonsynonymous SNV	ZNF536:ENST00000355537.3:exon2:c.C1295T:p.S432F,ZNF536:ENST00000585628.1:exon4:c.C1295T:p.S432F,	19q12	.
19	35758572	C	T	GGCCTACTAC,CGCCCGCGCC	exonic	LSR	nonsynonymous SNV	LSR:ENST00000354900.3:exon8:c.C1792T:p.P598S,LSR:ENST00000605618.1:exon6:c.C1402T:p.P468S,LSR:ENST00000427250.1:exon6:c.C1381T:p.P461S,LSR:ENST00000602122.1:exon8:c.C1789T:p.P597S,LSR:ENST00000361790.3:exon9:c.C1849T:p.P617S,LSR:ENST00000347609.4:exon8:c.C1675T:p.P55	19q13.12	.
19	35832695	A	G	AGCGACGCCA,CCCTCCCGTC	exonic	CD22	nonsynonymous SNV	CD22:ENST00000270311.6:exon9:c.A1502G:p.N501S,CD22:ENST00000085219.5:exon9:c.A1862G:p.N621S,CD22:ENST00000536635.2:exon8:c.A1598G:p.N533S,CD22:ENST00000544992.2:exon9:c.A1862G:p.N621S,CD22:ENST00000419549.2:exon8:c.A1346G:p.N449S,CD22:ENST00000341773.6:exon7:c.A133	19q13.12	.
19	36051468	T	G	CACCACCAGT,GGTCAGCGTT	exonic	ATP4A	nonsynonymous SNV	ATP4A:ENST00000262623.3:exon6:c.A584C:p.Q195P,	19q13.12	.
19	36051469	G	T	ACCACCAGTT,GTCAGCGTTG	exonic	ATP4A	nonsynonymous SNV	ATP4A:ENST00000262623.3:exon6:c.C583A:p.Q195K,	19q13.12	.
19	36214864	C	T	GACTCGGAGC,CGGGGGCCCC	exonic	KMT2B	nonsynonymous SNV	KMT2B:ENST00000222270.7:exon8:c.C3290T:p.P1097L,KMT2B:ENST00000420124.1:exon8:c.C3290T:p.P1097L,	19q13.12	.
19	36673573	G	A	AGTCAGTTGT,AAACACGAAT	exonic	ZNF565	nonsynonymous SNV	ZNF565:ENST00000392173.2:exon5:c.C1295T:p.S432L,ZNF565:ENST00000355114.5:exon5:c.C1415T:p.S472L,ZNF565:ENST00000304116.5:exon5:c.C1295T:p.S432L,	19q13.12	.
19	37130739	C	T	GGCATGTTTT,ATGGTTAATC	exonic	ZNF461	nonsynonymous SNV	ZNF461:ENST00000588268.1:exon6:c.G508A:p.E170K,ZNF461:ENST00000360357.4:exon5:c.G439A:p.E147K,ZNF461:ENST00000591370.1:exon4:c.G244A:p.E82K,	19q13.12	.
19	37676893	G	A	TCCCCAGTAT,GATTCTCTGA	exonic	ZNF585B	nonsynonymous SNV	ZNF585B:ENST00000532828.2:exon5:c.C1546T:p.H516Y,ZNF585B:ENST00000312908.5:exon2:c.C310T:p.H104Y,ZNF585B:ENST00000531805.1:exon3:c.C1381T:p.H461Y,	19q13.12	.
19	37686869	G	A	CAGAGGTTGC,GTGAGCCAAG	exonic	ZNF585B	nonsynonymous SNV	ZNF585B:ENST00000591273.1:exon3:c.C125T:p.P42L,	19q13.12	.
19	38755545	T	G	GGCGCAGCTG,GCGGGCTGAG	exonic	SPINT2	nonsynonymous SNV	SPINT2:ENST00000301244.7:exon1:c.T13G:p.C5G,SPINT2:ENST00000587516.1:exon1:c.T13G:p.C5G,SPINT2:ENST00000454580.3:exon1:c.T13G:p.C5G,	19q13.2	.
19	38954078	C	T	CCTGCCGCCC,ATCTGGAGCG	exonic	RYR1	nonsynonymous SNV	RYR1:ENST00000360985.3:exon21:c.C2593T:p.H865Y,RYR1:ENST00000355481.4:exon21:c.C2593T:p.H865Y,RYR1:ENST00000359596.3:exon21:c.C2593T:p.H865Y,	19q13.2	.
19	38964021	C	T	CCTCAGGTAT,CCGAGTGGAC	exonic	RYR1	nonsynonymous SNV	RYR1:ENST00000360985.3:exon28:c.C3770T:p.S1257F,RYR1:ENST00000355481.4:exon28:c.C3770T:p.S1257F,RYR1:ENST00000359596.3:exon28:c.C3770T:p.S1257F,	19q13.2	.
19	38964291	G	A	GCAGAGAACG,CAAAGAAGGG	exonic	RYR1	nonsynonymous SNV	RYR1:ENST00000360985.3:exon28:c.G4040A:p.G1347D,RYR1:ENST00000355481.4:exon28:c.G4040A:p.G1347D,RYR1:ENST00000359596.3:exon28:c.G4040A:p.G1347D,	19q13.2	.
19	38996502	G	A	TGATTGCCTG,GAATGGACGA	exonic	RYR1	stopgain	RYR1:ENST00000360985.3:exon54:c.G8457A:p.W2819X,RYR1:ENST00000355481.4:exon54:c.G8457A:p.W2819X,RYR1:ENST00000359596.3:exon54:c.G8457A:p.W2819X,	19q13.2	.
19	40225701	C	T	GAGGCAGCCT,TGTGTATGGC	exonic	CLC	nonsynonymous SNV	CLC:ENST00000221804.4:exon2:c.G28A:p.E10K,	19q13.2	.
19	40331072	C	T	CGATGCGGCT,CACCATCACA	exonic	FBL	nonsynonymous SNV	FBL:ENST00000221801.3:exon3:c.G265A:p.E89K,FBL:ENST00000595545.1:exon1:c.G10A:p.E4K,FBL:ENST00000594309.1:exon3:c.G262A:p.E88K,FBL:ENST00000594443.1:exon2:c.G94A:p.E32K,FBL:ENST00000599134.1:exon3:c.G226A:p.E76K,FBL:ENST00000597634.1:exon3:c.G154A:p.E52K,FBL:ENST0000	19q13.2	.
19	40902108	C	G	CAGGGACTTT,ATTTCACAGA	exonic	PRX	nonsynonymous SNV	PRX:ENST00000324001.7:exon7:c.G2151C:p.M717I,	19q13.2	.
19	41060425	G	A	CCCCCAGGAC,AACAGAGCAC	exonic	SPTBN4	nonsynonymous SNV	SPTBN4:ENST00000352632.3:exon24:c.G4957A:p.E1653K,SPTBN4:ENST00000392025.1:exon8:c.G1186A:p.E396K,SPTBN4:ENST00000595535.1:exon24:c.G4957A:p.E1653K,SPTBN4:ENST00000598249.1:exon24:c.G4957A:p.E1653K,SPTBN4:ENST00000338932.3:exon24:c.G4957A:p.E1653K,SPTBN4:ENST00	19q13.2	.
19	41518386	C	T	TACATCATCC,CAAGGTAAGA	exonic	CYP2B6	nonsynonymous SNV	CYP2B6:ENST00000330446.5:exon3:c.C548T:p.P183L,CYP2B6:ENST00000593831.1:exon3:c.C440T:p.P147L,CYP2B6:ENST00000324071.4:exon7:c.C1148T:p.P383L,	19q13.2	.
19	42353281	C	T	ACTGACAGCT,CTCTTTCTGG	exonic	DMRTC2	nonsynonymous SNV	DMRTC2:ENST00000596827.1:exon6:c.C712T:p.P238S,DMRTC2:ENST00000269945.3:exon6:c.C712T:p.P238S,	19q13.2	.
19	42471275	G	A	CCGGAGAGAT,GGAAGAGAGA	exonic	ATP1A3	nonsynonymous SNV	ATP1A3:ENST00000441343.1:exon22:c.C3139T:p.H1047Y,	19q13.2	.
19	42506500	C	T	CATATTTGTA,GTTGGTGGCC	exonic	GRIK5	nonsynonymous SNV	GRIK5:ENST00000301218.4:exon19:c.G2713A:p.V905I,	19q13.2	.
19	42506589	A	G	TGAGAAGGCA,CCTGGACACG	exonic	GRIK5	nonsynonymous SNV	GRIK5:ENST00000301218.4:exon19:c.T2624C:p.V875A,	19q13.2	.
19	42825822	A	C	GGATTACAGG,GTGAGCCACT	exonic	TMEM145	nonsynonymous SNV	TMEM145:ENST00000601644.1:exon3:c.A233C:p.E78A,	19q13.2	.
19	42830489	G	A	GGACTGCAAG,GGCAGCGGCA	exonic	MEGF8	nonsynonymous SNV	MEGF8:ENST00000251268.6:exon1:c.G94A:p.G32R,MEGF8:ENST00000334370.4:exon1:c.G94A:p.G32R,	19q13.2	.
19	42880359	C	T	GTCTTCTTCT,CTGCTTCTTC	exonic	MEGF8	nonsynonymous SNV	MEGF8:ENST00000251268.6:exon42:c.C7970T:p.S2657F,MEGF8:ENST00000334370.4:exon41:c.C7769T:p.S2590F,MEGF8:ENST00000378073.4:exon4:c.C752T:p.S251F,	19q13.2	.
19	43023274	G	A	AAGAACCAAC,GATGGAGATT	exonic	CEACAM1	nonsynonymous SNV	CEACAM1:ENST00000161559.6:exon5:c.C1072T:p.R358C,CEACAM1:ENST00000403444.3:exon5:c.C1072T:p.R358C,	19q13.2	.
19	43575888	C	T	GAGCTTTCCT,GCCAGTGGCT	exonic	PSG2	nonsynonymous SNV	PSG2:ENST00000406487.1:exon4:c.G928A:p.E310K,	19q13.31	.
19	44680640	C	T	TCTTCAATCC,ATCAAAGAGT	exonic	ZNF226	nonsynonymous SNV	ZNF226:ENST00000590089.1:exon7:c.C1225T:p.H409Y,ZNF226:ENST00000337433.5:exon6:c.C1225T:p.H409Y,ZNF226:ENST00000454662.2:exon6:c.C1225T:p.H409Y,	19q13.31	.
19	44793065	G	A	TTCCCAGTTG,AAATTCTTGA	exonic	ZNF235	nonsynonymous SNV	ZNF235:ENST00000291182.4:exon5:c.C523T:p.P175S,ZNF235:ENST00000591609.1:exon5:c.C778T:p.P260S,	19q13.31	.
19	44891053	C	T	GGTTTCTCCC,TGTGTGGACT	exonic	ZNF285	nonsynonymous SNV	ZNF285:ENST00000330997.4:exon4:c.G1354A:p.G452R,ZNF285:ENST00000544719.2:exon4:c.G1354A:p.G452R,ZNF285:ENST00000591679.1:exon5:c.G1375A:p.G459R,	19q13.31	.
19	44936415	C	T	TTCCTCTCAC,CACTGAGAGT	exonic	ZNF229	nonsynonymous SNV	ZNF229:ENST00000591604.1:exon5:c.G220A:p.G74S,ZNF229:ENST00000588931.1:exon5:c.G220A:p.G74S,ZNF229:ENST00000291187.4:exon5:c.G220A:p.G74R,	19q13.31	.
19	45495921	C	T	GCGTCGACCC,ACCCGAGTCA	exonic	CLPTM1	nonsynonymous SNV	CLPTM1:ENST00000586915.1:exon2:c.C244T:p.H82Y,	19q13.32	.
19	45645682	C	G	CCCTGCAGAT,CTGGACCTCC	exonic	PPP1R37	nonsynonymous SNV	PPP1R37:ENST00000221462.4:exon7:c.C840G:p.I280M,PPP1R37:ENST00000421905.1:exon7:c.C840G:p.I280M,	19q13.32	.
19	46248581	T	A	CTCACAAAGG,ACAGGGACTA	splicing	AC074212.3(I	.	.	19q13.32	.
19	46262238	G	A	AGCTCCCCAG,GAAACTGCTG	exonic	AC074212.3	nonsynonymous SNV	AC074212.3:ENST00000457052.2:exon10:c.G1115A:p.G372E,	19q13.32	.
19	46663919	G	A	CCCAGGTGTG,AGACAAGATC	exonic	IGFL2	nonsynonymous SNV	IGFL2:ENST00000377693.4:exon3:c.G122A:p.G41E,IGFL2:ENST00000434646.2:exon4:c.G155A:p.G52E,IGFL2:ENST00000601052.1:exon1:c.G20A:p.G7E,	19q13.32	.
19	46915085	C	T	AGCCTCTGCC,CCTGGTCAGC	exonic	CCDC8	nonsynonymous SNV	CCDC8:ENST00000307522.3:exon1:c.G983A:p.G328E,	19q13.32	CCDC8;basechange=c.983G>A;AAchange=p.G328E;tissue=lung
19	47152748	G	A	GCCGGGGGAC,GAGACGCGTC	exonic	DACT3	nonsynonymous SNV	DACT3:ENST00000391916.2:exon4:c.C881T:p.P294L,DACT3:ENST00000300875.4:exon4:c.C206T:p.P69L,	19q13.32	.
19	47774729	G	T	CCCCTGCAGT,AGCAGGCCCA	exonic	CCDC9	stopgain	CCDC9:ENST00000221922.6:exon12:c.G1390T:p.E464X,	19q13.32	.
19	48041398	C	T	GCCTCACTCA,CGAGCATGGA	splicing	ZNF541(ENS	.	.	19q13.33	.
19	48041399	C	T	CCTCACTCAC,GAGCATGGAG	exonic	ZNF541	nonsynonymous SNV	ZNF541:ENST00000391901.3:exon7:c.G3094A:p.D1032N,ZNF541:ENST00000314121.4:exon7:c.G3151A:p.D1051N,	19q13.33	.
19	48305555	G	A	GCCTGGGATC,GGCCTGGGTT	exonic	TPRX1	nonsynonymous SNV	TPRX1:ENST00000322175.3:exon2:c.C713T:p.P238L,TPRX1:ENST00000535759.1:exon4:c.C1004T:p.P335L,TPRX1:ENST00000543508.1:exon3:c.C683T:p.P228L,	19q13.33	.
19	48342830	C	T	GCCTCAGAGT,CCCTTTGCCT	exonic	CRX	nonsynonymous SNV	CRX:ENST00000221996.7:exon4:c.C506T:p.S169F,CRX:ENST00000539067.1:exon4:c.C506T:p.S169F,	19q13.33	.
19	48698756	G	A	CGACTGGGGA,GGGCTCCGAA	exonic	C19orf68	nonsynonymous SNV	C19orf68:ENST00000328759.7:exon4:c.G1435A:p.G479R,	19q13.33	.
19	48698757	G	A	GACTGGGGAG,GGCTCCGAAA	exonic	C19orf68	nonsynonymous SNV	C19orf68:ENST00000328759.7:exon4:c.G1436A:p.G479E,	19q13.33	.
19	49398749	G	A	CGGAGGAAAG,GTTCCCAAGG	exonic	TULP2	nonsynonymous SNV	TULP2:ENST00000520977.1:exon3:c.C166T:p.P56S,TULP2:ENST00000221399.3:exon5:c.C223T:p.P75S,TULP2:ENST00000522945.1:exon5:c.C214T:p.P72S,	19q13.33	.
19	49651940	G	A	CTACATTATG,GATTATGTGC	exonic	PPFIA3	nonsynonymous SNV	PPFIA3:ENST00000602351.1:exon23:c.G3002A:p.G1001E,PPFIA3:ENST00000334186.4:exon25:c.G3029A:p.G1010E,PPFIA3:ENST00000602897.1:exon2:c.G176A:p.G59E,PPFIA3:ENST00000602848.1:exon4:c.G278A:p.G93E,	19q13.33	.
19	49669416	C	G	CACGGAGAAG,CCACCGATGC	exonic	TRPM4	nonsynonymous SNV	TRPM4:ENST00000252826.5:exon3:c.C211G:p.P71A,TRPM4:ENST00000598691.1:exon3:c.C211G:p.P71A,TRPM4:ENST00000598502.1:exon3:c.C211G:p.P71A,TRPM4:ENST00000427978.2:exon3:c.C211G:p.P71A,	19q13.33	.
19	49793911	A	G	ACAAAGATGA,CCATCATGGT	exonic	SLC6A16	nonsynonymous SNV	SLC6A16:ENST00000454748.3:exon11:c.T1892C:p.V631A,SLC6A16:ENST00000335875.4:exon11:c.T1892C:p.V631A,	19q13.33	.
19	49900940	G	A	AGCCAACCTG,AGAGCTTCGG	exonic	CCDC155	nonsynonymous SNV	CCDC155:ENST00000447857.3:exon6:c.G433A:p.E145K,CCDC155:ENST00000595828.1:exon7:c.G391A:p.E131K,CCDC155:ENST00000594043.1:exon6:c.G433A:p.E145K,	19q13.33	.
19	50099217	C	T	CCAGCGCTCT,GGGCCATGGG	exonic	PRR12	nonsynonymous SNV	PRR12:ENST00000418929.2:exon4:c.C1625T:p.S542L,	19q13.33	.
19	50865475	G	A	GTCCCCAGGG,ATGGGGCCCC	exonic	NAPSA	nonsynonymous SNV	NAPSA:ENST00000253719.2:exon2:c.C179T:p.S60F,NAPSA:ENST00000599233.1:exon2:c.C179T:p.S60F,NAPSA:ENST00000598915.1:exon2:c.C179T:p.S60F,	19q13.33	.
19	51051896	G	A	CACCCGGCTG,CCTGGTTGCT	exonic	LRRC4B	nonsynonymous SNV	LRRC4B:ENST00000389201.3:exon2:c.C200T:p.A67V,LRRC4B:ENST00000599957.1:exon2:c.C200T:p.A67V,LRRC4B:ENST00000600381.1:exon2:c.C200T:p.A67V,	19q13.33	.
19	51535134	C	T	CCCCTTACTC,GTGGGTGGTT	exonic	KLK12	nonsynonymous SNV	KLK12:ENST00000525263.1:exon3:c.G455A:p.R152Q,KLK12:ENST00000250351.4:exon4:c.G455A:p.R152Q,KLK12:ENST00000531374.1:exon4:c.G455A:p.R152Q,KLK12:ENST00000319590.4:exon4:c.G455A:p.R152Q,	19q13.41	.
19	51993848	C	T	TAGGACAAAT,GGGCTCCATG	exonic	CEACAM18	nonsynonymous SNV	CEACAM18:ENST00000396477.4:exon6:c.C1097T:p.S366L,	19q13.41	.
19	52954647	G	A	CGAGGCCACG,GACTCTCAGG	exonic	ZNF534	nonsynonymous SNV	ZNF534:ENST00000432303.2:exon3:c.G350A:p.G117E,ZNF534:ENST00000301085.4:exon5:c.G479A:p.G160E,	19q13.41	.
19	53075505	T	A	ATGGGGTTTC,CCATGTTGGT	exonic	ZNF701	nonsynonymous SNV	ZNF701:ENST00000593941.1:exon2:c.T11A:p.L4H,ZNF701:ENST00000540331.1:exon2:c.T11A:p.L4H,ZNF701:ENST00000301093.2:exon2:c.T11A:p.L4H,	19q13.41	.
19	53518686	C	T	GCTGTGAAGT,TGCCCTCCCC	exonic	ERVV-1	nonsynonymous SNV	ERVV-1:ENST00000602168.1:exon1:c.C1343T:p.S448F,	19q13.41	.
19	53612755	A	C	TGGTAGAAGA,ATTATTTGGG	exonic	ZNF415	nonsynonymous SNV	ZNF415:ENST00000440291.1:exon5:c.T504G:p.I168M,ZNF415:ENST00000448501.1:exon6:c.T687G:p.I229M,ZNF415:ENST00000243643.4:exon4:c.T543G:p.I181M,ZNF415:ENST00000421033.1:exon5:c.T579G:p.I193M,ZNF415:ENST00000455735.2:exon7:c.T687G:p.I229M,ZNF415:ENST00000500065.4:exon4:c.T	19q13.42	.
19	54578294	C	T	AGTCCAACAT,GCAGCGTCAC	exonic	TARM1	nonsynonymous SNV	TARM1:ENST00000432826.1:exon3:c.G143A:p.R48Q,TARM1:ENST00000446034.2:exon3:c.G167A:p.R56Q,	19q13.42	.
19	54675802	G	A	TCTCGGTACC,AAGGGTGGCA	exonic	TMC4	nonsynonymous SNV	TMC4:ENST00000301187.4:exon2:c.C130T:p.R44W,TMC4:ENST00000376591.4:exon2:c.C148T:p.R50W,	19q13.42	TMC4;basechange=c.130C>T;AAchange=p.R44W;tissue=lung
19	55143005	G	A	ATCACCCAGG,GAGTCCTGTG	exonic	LILRB1	nonsynonymous SNV	LILRB1:ENST00000421584.1:exon3:c.G125A:p.G42E,LILRB1:ENST00000396321.2:exon3:c.G125A:p.G42E,LILRB1:ENST00000324602.7:exon4:c.G125A:p.G42E,LILRB1:ENST00000396315.1:exon3:c.G125A:p.G42E,LILRB1:ENST00000448689.1:exon3:c.G125A:p.G42E,LILRB1:ENST00000396317.1:exon3:c.G125A	19q13.42	.
19	55713575	G	T	CATCTCCAGT,TACTCAACCC	exonic	PTPRH	stopgain	PTPRH:ENST00000376350.3:exon6:c.C1002A:p.Y334X,PTPRH:ENST00000263434.5:exon4:c.C468A:p.Y156X,	19q13.42	.
19	56041641	G	A	CTCCAGGGCG,AGGCCAGCTG	exonic	SBK2	nonsynonymous SNV	SBK2:ENST00000413299.1:exon4:c.C506T:p.S169F,SBK2:ENST00000344158.3:exon3:c.C506T:p.S169F,	19q13.42	.
19	56126275	G	A	GGCCCACGCG,GGGCGGGCGC	exonic	ZNF865	nonsynonymous SNV	ZNF865:ENST00000568956.1:exon2:c.G1291A:p.G431R,	19q13.42	.
19	56170695	G	A	CGCCTCCCGG,ACAGGCGACG	exonic	U2AF2	nonsynonymous SNV	U2AF2:ENST00000308924.4:exon2:c.G169A:p.D57N,U2AF2:ENST00000450554.2:exon2:c.G169A:p.D57N,	19q13.42	.
19	56297125	C	T	ACACCAATTT,TCTGCCTTCC	exonic	NLRP11	nonsynonymous SNV	NLRP11:ENST00000360133.3:exon11:c.G2806A:p.E936K,NLRP11:ENST00000443188.1:exon12:c.G2968A:p.E990K,NLRP11:ENST00000589093.1:exon10:c.G2968A:p.E990K,NLRP11:ENST00000592953.1:exon9:c.G2671A:p.E891K,NLRP11:ENST00000589824.2:exon8:c.G2806A:p.E936K,	19q13.42	NLRP11;basechange=c.2968G>T;AAchange=p.E990*;tissue=endometrium
19	56370005	G	A	TGAGTTTTGT,AAGACGACCT	exonic	NLRP4	nonsynonymous SNV	NLRP4:ENST00000301295.6:exon3:c.G1246A:p.E416K,NLRP4:ENST00000346986.5:exon3:c.G1246A:p.E416K,NLRP4:ENST00000587891.1:exon1:c.G1021A:p.E341K,	19q13.43	.
19	56720549	C	T	GAAAACACAT,GAGAAGCCAC	exonic	ZSCAN5C	stopgain	ZSCAN5C:ENST00000376267.1:exon4:c.C1471T:p.R491X,ZSCAN5C:ENST00000534327.1:exon5:c.C1471T:p.R491X,	19q13.43	ZSCAN5C;basechange=c.1471C>T;AAchange=p.R491*;tissue=endometrium
19	57954733	A	G	ACAGGTCACA,TTCCAAAGCC	exonic	ZNF749	nonsynonymous SNV	ZNF749:ENST00000334181.4:exon3:c.A217G:p.I73V,	19q13.43	.
19	58083603	T	C	GTAAAGGACT,CCCACATTTG	exonic	ZNF416	nonsynonymous SNV	ZNF416:ENST00000196489.3:exon4:c.A1669G:p.K557E,	19q13.43	.
19	58152618	G	A	ACTAGAGAAG,ACTTTTTGAG	exonic	ZNF211	nonsynonymous SNV	ZNF211:ENST00000299871.5:exon5:c.G959A:p.G320E,ZNF211:ENST00000541801.1:exon4:c.G737A:p.G246E,ZNF211:ENST00000240731.4:exon4:c.G803A:p.G268E,ZNF211:ENST00000420680.1:exon3:c.G776A:p.G259E,ZNF211:ENST00000254182.7:exon5:c.G737A:p.G246E,ZNF211:ENST00000391703.3:exon	19q13.43	.
19	58434560	C	T	TCCCACCTAC,CGGGAGGCTG	exonic	ZNF418	nonsynonymous SNV	ZNF418:ENST00000600989.1:exon5:c.G302A:p.G101E,	19q13.43	.
19	58490946	C	A	GCTTTCTCTA,AGTACCAATT	exonic	ZNF606	nonsynonymous SNV	ZNF606:ENST00000341164.4:exon7:c.G1102T:p.V368L,ZNF606:ENST00000551380.1:exon7:c.G1102T:p.V368L,ZNF606:ENST00000536132.1:exon6:c.G832T:p.V278L,	19q13.43	.
19	58596464	G	A	CCCATCCTCG,GGTGCGGCCT	exonic	ZSCAN18	nonsynonymous SNV	ZSCAN18:ENST00000421612.2:exon6:c.C713T:p.P238L,ZSCAN18:ENST00000601144.1:exon7:c.C1121T:p.P374L,ZSCAN18:ENST00000240727.6:exon7:c.C1121T:p.P374L,ZSCAN18:ENST00000433686.2:exon6:c.C812T:p.P271L,ZSCAN18:ENST00000600404.1:exon7:c.C1289T:p.P430L,	19q13.43	.
19	58596615	C	T	TCTTCCTCCT,CTCAGTGGTG	exonic	ZSCAN18	nonsynonymous SNV	ZSCAN18:ENST00000421612.2:exon6:c.G562A:p.E188K,ZSCAN18:ENST00000601144.1:exon7:c.G970A:p.E324K,ZSCAN18:ENST00000240727.6:exon7:c.G970A:p.E324K,ZSCAN18:ENST00000433686.2:exon6:c.G661A:p.E221K,ZSCAN18:ENST00000600404.1:exon7:c.G1138A:p.E380K,	19q13.43	.
19	59080896	G	A	GGCCTCCTCA,GCAGGAGGGT	exonic	MZF1	nonsynonymous SNV	MZF1:ENST00000215057.2:exon4:c.C638T:p.P213L,MZF1:ENST00000599369.1:exon4:c.C638T:p.P213L,MZF1:ENST00000594234.1:exon4:c.C638T:p.P213L,MZF1:ENST00000594108.1:exon4:c.C638T:p.P213L,	19q13.43	.
20	3684069	G	A	TCCAGGATGG,ACCTGCTGGG	exonic	SIGLEC1	nonsynonymous SNV	SIGLEC1:ENST00000202578.4:exon5:c.C1003T:p.P335S,SIGLEC1:ENST00000344754.4:exon5:c.C1003T:p.P335S,	20p13	.
20	4866538	G	T	AAAAGCACTG,CCTGAAACAG	exonic	SLC23A2	nonsynonymous SNV	SLC23A2:ENST00000379333.1:exon7:c.C500A:p.A167D,SLC23A2:ENST00000338244.1:exon7:c.C500A:p.A167D,	20p13	.
20	9561361	A	T	TCGGTCGTGT,GTCAGCAGTA	exonic	PAK7	nonsynonymous SNV	PAK7:ENST00000353224.5:exon4:c.T421A:p.Y141N,PAK7:ENST00000378429.3:exon5:c.T421A:p.Y141N,PAK7:ENST00000378423.1:exon5:c.T421A:p.Y141N,	20p12.2	.
20	13260329	C	T	AGATAAAGAT,AGCATCCGGA	exonic	ISM1	stopgain	ISM1:ENST00000262487.4:exon3:c.C427T:p.Q143X,	20p12.1	.
20	17462543	G	A	CCGCAGAAGG,GGTGCTGAAG	exonic	PCSK2	nonsynonymous SNV	PCSK2:ENST00000377899.1:exon13:c.G1688A:p.G563E,PCSK2:ENST00000262545.2:exon12:c.G1745A:p.G582E,PCSK2:ENST00000536609.1:exon11:c.G1640A:p.G547E,	20p12.1	.
20	17474877	G	A	AAAGCCTTGG,AGGGCCTTTT	exonic	BFSP1	nonsynonymous SNV	BFSP1:ENST00000536626.1:exon10:c.C1423T:p.P475S,BFSP1:ENST00000544874.1:exon9:c.C1423T:p.P475S,BFSP1:ENST00000377873.3:exon8:c.C1840T:p.P614S,BFSP1:ENST00000377868.2:exon8:c.C1465T:p.P489S,	20p12.1	.
20	18037489	C	T	CGGCAGTCCT,GGGGGGGTCG	exonic	OVOL2	nonsynonymous SNV	OVOL2:ENST00000278780.6:exon2:c.G133A:p.E45K,	20p11.23	.
20	20493466	C	T	AACATCATCC,CCTCCTCAAC	exonic	RALGAPA2	nonsynonymous SNV	RALGAPA2:ENST00000202677.7:exon32:c.G4547A:p.G1516E,	20p11.23	.
20	23548954	C	A	AGGGAGGTAA,GAGCCATGAC	exonic	CST9L	nonsynonymous SNV	CST9L:ENST00000376979.3:exon1:c.G134T:p.R45L,	20p11.21	.
20	30606805	G	A	CGCAGGACGC,ACCCCGGCCC	exonic	CCM2L	nonsynonymous SNV	CCM2L:ENST00000262659.8:exon5:c.G526A:p.D176N,CCM2L:ENST00000300415.8:exon5:c.G526A:p.D176N,	20q11.21	.
20	31025100	G	A	TGACTGTATT,GACCCTCAAA	exonic	ASXL1	nonsynonymous SNV	ASXL1:ENST00000306058.5:exon12:c.G4570A:p.G1524R,ASXL1:ENST00000375687.4:exon13:c.G4585A:p.G1529R,	20q11.21	.
20	31659930	G	A	TAGAAGAATG,CTCAGCCATG	exonic	BPIFB3	stopgain	BPIFB3:ENST00000375494.3:exon13:c.G1281A:p.W427X,	20q11.21	.
20	31671578	G	A	GGCCTGCTCG,CGGAGGTGGT	exonic	BPIFB4	nonsynonymous SNV	BPIFB4:ENST00000375483.3:exon3:c.G575A:p.G192D,	20q11.21	.
20	40040896	G	A	TGGTTTGTCT,AAAAATTCTA	exonic	CHD6	nonsynonymous SNV	CHD6:ENST00000373233.3:exon36:c.C7139T:p.S2380L,	20q12	.
20	43251498	C	T	CTGCCGCAGC,TGTTATAAAG	exonic	ADA	nonsynonymous SNV	ADA:ENST00000536532.1:exon8:c.G752A:p.R251K,ADA:ENST00000372874.4:exon8:c.G752A:p.R251K,ADA:ENST00000537820.1:exon7:c.G680A:p.R227K,	20q13.12	.
20	43567778	C	T	AAAGGAAATC,TCGCTTCCAT	exonic	PABPC1L	nonsynonymous SNV	PABPC1L:ENST00000372822.2:exon5:c.C356T:p.P119L,	20q13.12	.
20	43959113	G	A	GATTCTCTTG,GGATAACCTC	exonic	SDC4	nonsynonymous SNV	SDC4:ENST00000372733.3:exon4:c.C338T:p.P113L,SDC4:ENST00000537976.1:exon3:c.C122T:p.P41L,	20q13.12	.
20	44035230	C	T	GCCTCTTCCT,GTTCCCGGCT	exonic	DBNDD2	nonsynonymous SNV	DBNDD2:ENST00000372720.3:exon1:c.C139T:p.R47C,	20q13.12	.
20	44038736	G	A	GGATGAAGAG,AGGAAAGGGG	exonic	DBNDD2	nonsynonymous SNV	DBNDD2:ENST00000372710.3:exon3:c.G748A:p.E250K,DBNDD2:ENST00000357275.2:exon4:c.G442A:p.E148K,DBNDD2:ENST00000372712.2:exon4:c.G442A:p.E148K,DBNDD2:ENST00000372720.3:exon4:c.G736A:p.E246K,DBNDD2:ENST00000360981.4:exon4:c.G442A:p.E148K,DBNDD2:ENST00000372723.3	20q13.12	.
20	44845489	C	T	GGCGGGTTGT,ATTGACGTCG	exonic	CDH22	nonsynonymous SNV	CDH22:ENST00000372262.3:exon4:c.G814A:p.D272N,CDH22:ENST00000537909.1:exon5:c.G814A:p.D272N,	20q13.12	.

22	21340161	G	A	CCTGCGGTTC,ATGTGAAAGA	exonic	LZTR1	nonsynonymous SNV	LZTR1:ENST00000215739.8:exon3:c.G295A:p.D99N,LZTR1:ENST00000414985.1:exon3:c.G295A:p.D99N,	22q11.21	LZTR1;basechange=c.295G>A;AAchange=p.D99N;tissue=endometrium
22	22843189	C	G	CTGTTTACTT,GAAAGTGGAA	exonic	ZNF280B	nonsynonymous SNV	ZNF280B:ENST00000360412.2:exon4:c.G535C:p.E179Q,ZNF280B:ENST00000406426.1:exon4:c.G535C:p.E179Q,	22q11.22	.
22	22890665	G	A	AGGGTACCAT,GATGTCCTCA	exonic	PRAME	nonsynonymous SNV	PRAME:ENST00000398741.1:exon6:c.C1354T:p.H452Y,PRAME:ENST00000539862.1:exon4:c.C1306T:p.H436Y,PRAME:ENST00000543184.1:exon5:c.C1354T:p.H452Y,PRAME:ENST00000402697.1:exon6:c.C1354T:p.H452Y,PRAME:ENST00000398743.2:exon6:c.C1354T:p.H452Y,PRAME:ENST00000405655.3:e	22q11.22	.
22	23047198	G	A	ATCCCTGAAC,ATTCTCTGGG	exonic	IGLV3-22	nonsynonymous SNV	IGLV3-22:ENST00000390307.2:exon2:c.G236A:p.R79Q,	22q11.22	.
22	23247094	C	T	CCAGCTTCCT,CTTCACAGCT	exonic	IGLJ3	nonsynonymous SNV	IGLJ3:ENST00000390324.2:exon1:c.C40T:p.P14S,	22q11.22	.
22	24724831	C	T	TAAAAAACTC,AGGACCAAAA	exonic	SPECC1L,SP	stopgain	SPECC1L:ENST00000541492.1:exon6:c.C2164T:p.Q722X,SPECC1L:ENST00000421374.1:exon6:c.C2164T:p.Q722X,SPECC1L-ADORA2A:ENST00000358654.2:exon7:c.C2164T:p.Q722X,SPECC1L:ENST00000437398.1:exon6:c.C2164T:p.Q722X,SPECC1L:ENST00000314328.9:exon7:c.C2164T:p.Q722X,	22q11.23	.
22	26688621	C	T	GCCTTGCCCC,CAAGAAGAAA	exonic	SEZ6L	nonsynonymous SNV	SEZ6L:ENST00000248933.6:exon2:c.C344T:p.P115L,SEZ6L:ENST00000343706.4:exon2:c.C344T:p.P115L,SEZ6L:ENST00000404234.3:exon2:c.C344T:p.P115L,SEZ6L:ENST00000529632.2:exon2:c.C344T:p.P115L,SEZ6L:ENST00000360929.3:exon2:c.C344T:p.P115L,	22q12.1	.
22	26895474	C	T	GCCTTGGCCT,TTTGCCAGAC	exonic	TFIP11	nonsynonymous SNV	TFIP11:ENST00000407431.1:exon8:c.G925A:p.E309K,TFIP11:ENST00000407690.1:exon9:c.G925A:p.E309K,TFIP11:ENST00000407148.1:exon8:c.G925A:p.E309K,TFIP11:ENST00000405938.1:exon10:c.G925A:p.E309K,	22q12.1	.
22	29446847	C	T	CCTGGCTGCC,TCCGGAGGAG	exonic	ZNRF3	nonsynonymous SNV	ZNRF3:ENST00000332811.4:exon8:c.C2378T:p.P793L,ZNRF3:ENST00000402174.1:exon8:c.C2378T:p.P793L,ZNRF3:ENST00000544604.2:exon8:c.C2678T:p.P893L,ZNRF3:ENST00000406323.3:exon7:c.C2378T:p.P793L,	22q12.1	.
22	30780429	C	T	AACTTCCACT,GATATTGGCA	exonic	RNF215	nonsynonymous SNV	RNF215:ENST00000382363.3:exon5:c.G643A:p.E215K,	22q12.2	.
22	30921653	G	A	GAGGAGCCGC,GCCCACGGAC	exonic	SEC14L6	nonsynonymous SNV	SEC14L6:ENST00000402034.2:exon10:c.C856T:p.R286C,	22q12.2	.
22	32630901	C	T	ATGGGCATTC,AAATATAATT	exonic	SLC5A4	nonsynonymous SNV	SLC5A4:ENST00000266086.4:exon8:c.G844A:p.G282R,	22q12.3	.
22	32651264	G	A	GTCAGACAAT,GAGGTGGCTC	exonic	SLC5A4	nonsynonymous SNV	SLC5A4:ENST00000266086.4:exon1:c.C53T:p.P18L,	22q12.3	.
22	32831816	G	A	AGCACAAAAG,AACTGGTGAG	exonic	BPIFC	nonsynonymous SNV	BPIFC:ENST00000300399.3:exon8:c.C799T:p.P267S,BPIFC:ENST00000432451.2:exon9:c.C241T:p.P81S,BPIFC:ENST00000397452.1:exon9:c.C799T:p.P267S,	22q12.3	.
22	36142011	G	A	GCAAGGGCAT,GTAGGGGTCG	exonic	RBFOX2	nonsynonymous SNV	RBFOX2:ENST00000449924.2:exon12:c.C1057T:p.H353Y,RBFOX2:ENST00000416721.2:exon12:c.C1045T:p.H349Y,RBFOX2:ENST00000262829.7:exon11:c.C991T:p.H331Y,RBFOX2:ENST00000397303.2:exon11:c.C988T:p.H330Y,RBFOX2:ENST00000438146.2:exon13:c.C1270T:p.H424Y,	22q12.3	.
22	37407121	G	A	GGGGCCCGGC,AAACCACTCG	exonic	TST	nonsynonymous SNV	TST:ENST00000249042.3:exon3:c.C841T:p.R281C,TST:ENST00000403892.3:exon2:c.C841T:p.R281C,	22q12.3	.
22	37407346	G	A	ACGGCACCAC,GATATGGCCC	exonic	TST	nonsynonymous SNV	TST:ENST00000249042.3:exon3:c.C616T:p.R206C,TST:ENST00000403892.3:exon2:c.C616T:p.R206C,	22q12.3	.
22	38026006	C	A	TCCCCACAGT,GTCGGATGCC	exonic	GGA1	stopgain	GGA1:ENST00000406772.1:exon14:c.C941A:p.S314X,GGA1:ENST00000381756.5:exon13:c.C1211A:p.S404X,GGA1:ENST00000343632.4:exon13:c.C1160A:p.S387X,GGA1:ENST00000325180.8:exon11:c.C899A:p.S300X,GGA1:ENST00000337437.4:exon12:c.C1061A:p.S354X,	22q13.1	.
22	38121210	C	T	ACCATCATCT,CCCACCGCTC	exonic	TRIOBP	nonsynonymous SNV	TRIOBP:ENST00000406386.3:exon7:c.C2647T:p.P883S,	22q13.1	.
22	38129408	C	T	CAGCCCTGCC,CCAGCAGGCA	exonic	TRIOBP	nonsynonymous SNV	TRIOBP:ENST00000406386.3:exon8:c.C4051T:p.P1351S,	22q13.1	.
22	38208926	C	T	CAAAAATAGC,CGCTTCCACC	exonic	GCAT	nonsynonymous SNV	GCAT:ENST00000426858.1:exon2:c.C229T:p.P77S,	22q13.1	.
22	38208927	C	T	AAAAATAGCC,GCTTCCACCA	exonic	GCAT	nonsynonymous SNV	GCAT:ENST00000445195.1:exon3:c.C439T:p.R147C,GCAT:ENST00000248924.6:exon3:c.C361T:p.R121C,GCAT:ENST00000323205.6:exon3:c.C439T:p.R147C,GCAT:ENST00000426858.1:exon2:c.C230T:p.P77L,	22q13.1	.
22	39122114	G	A	CAGGGAGGAG,AGCCTGCTGA	exonic	GTPBP1	nonsynonymous SNV	GTPBP1:ENST00000216044.5:exon7:c.G1177A:p.E393K,	22q13.1	.
22	39388197	C	T	TCGGCCTCCC,CTCCTCCCCG	exonic	APOBEC3B	nonsynonymous SNV	APOBEC3B:ENST00000402182.3:exon7:c.C1177T:p.P393S,	22q13.1	.
22	40801227	G	A	CGCCTGCGCA,GGTGCTCCGG	exonic	SGSM3	nonsynonymous SNV	SGSM3:ENST00000248929.9:exon7:c.G557A:p.R186K,SGSM3:ENST00000457767.1:exon6:c.G356A:p.R119K,SGSM3:ENST00000454798.2:exon6:c.G356A:p.R119K,	22q13.1	.
22	41525914	C	T	TGCATCTTCT,GACAAATCAT	exonic	EP300	stopgain	EP300:ENST00000263253.7:exon5:c.C1189T:p.R397X,	22q13.2	.
22	41551083	G	A	CTTCCCTTTC,TCAACCTGTG	exonic	EP300	nonsynonymous SNV	EP300:ENST00000263253.7:exon17:c.G3227A:p.R1076H,	22q13.2	.
22	41566486	C	A	TCCTCCTGAC,AGAAGATACC	exonic	EP300	nonsynonymous SNV	EP300:ENST00000263253.7:exon27:c.C4363A:p.Q1455K,	22q13.2	.
22	45785750	G	A	TTATGAATAG,ATGACACAGG	exonic	SMC1B	nonsynonymous SNV	SMC1B:ENST00000404354.3:exon10:c.C1573T:p.P525S,SMC1B:ENST00000357450.4:exon10:c.C1573T:p.P525S,	22q13.31	.
22	45802545	C	T	CTACAGTTCC,TTTTAAAAAC	splicing	SMC1B(ENST	.	.	22q13.31	.
22	50678757	G	A	GACGCTGACT,GAATCCTTCG	exonic	TUBGCP6	stopgain	TUBGCP6:ENST00000439308.2:exon2:c.C781T:p.Q261X,TUBGCP6:ENST00000248846.5:exon2:c.C781T:p.Q261X,	22q13.33	.
22	50694115	G	A	TCCTTCAGCT,GTCCAGGTCT	exonic	MAPK12	stopgain	MAPK12:ENST00000215659.8:exon9:c.C700T:p.Q234X,MAPK12:ENST00000395780.1:exon7:c.C430T:p.Q144X,	22q13.33	.
X	2408233	C	A	CGGCCCCGTA,TTCTCAGGGA	exonic	ZBED1	nonsynonymous SNV	ZBED1:ENST00000461691.1:exon2:c.G528T:p.K176N,ZBED1:ENST00000381218.3:exon2:c.G528T:p.K176N,ZBED1:ENST00000381223.4:exon2:c.G528T:p.K176N,ZBED1:ENST00000381222.2:exon2:c.G528T:p.K176N,	Xp22.33	.
X	2924714	G	A	TGGAGTGGGG,ATTTGTGCTG	exonic	ARSH	nonsynonymous SNV	ARSH:ENST00000381130.2:exon1:c.G61A:p.D21N,	Xp22.33	.
X	5821743	G	A	TGCTGGATGA,CTCCTTGTAG	exonic	NLGN4X	nonsynonymous SNV	NLGN4X:ENST00000381095.3:exon5:c.C976T:p.L326F,NLGN4X:ENST00000538097.1:exon5:c.C976T:p.L326F,NLGN4X:ENST00000381092.1:exon6:c.C976T:p.L326F,NLGN4X:ENST00000275857.6:exon5:c.C976T:p.L326F,NLGN4X:ENST00000381093.2:exon6:c.C1036T:p.L346F,	Xp22.32	.
X	26157440	C	T	AAAGTAGTGT,GCTGGTGCAT	exonic	MAGEB18	nonsynonymous SNV	MAGEB18:ENST00000325250.1:exon2:c.C338T:p.S113L,	Xp21.3	.
X	27839451	C	T	GAGTAAACTC,GTGCCAGGGA	exonic	MAGEB10	nonsynonymous SNV	MAGEB10:ENST00000356790.2:exon3:c.C28T:p.R10C,	Xp21.3	.
X	29935700	G	A	TCGAGTTTGG,AAAGTGACAT	exonic	IL1RAPL1	nonsynonymous SNV	IL1RAPL1:ENST00000302196.4:exon6:c.G898A:p.E300K,IL1RAPL1:ENST00000378993.1:exon7:c.G898A:p.E300K,	Xp21.2	.
X	37969637	C	T	GCACTATGAT,GATTTGGACG	exonic	SYTL5	stopgain	SYTL5:ENST00000357972.5:exon13:c.C1498T:p.R500X,SYTL5:ENST00000297875.2:exon13:c.C1498T:p.R500X,SYTL5:ENST00000456733.2:exon13:c.C1564T:p.R522X,	Xp11.4	.
X	41332893	G	A	GCTCCCGTGC,AGGCGGTCTC	exonic	NYX	nonsynonymous SNV	NYX:ENST00000378220.1:exon3:c.G187A:p.E63K,NYX:ENST00000342595.2:exon2:c.G187A:p.E63K,	Xp11.4	.
X	50377325	C	T	CTCACTCTTC,GCCGGTTTTG	exonic	SHROOM4	nonsynonymous SNV	SHROOM4:ENST00000460112.3:exon3:c.G1400A:p.R467Q,SHROOM4:ENST00000376020.2:exon4:c.G1748A:p.R583Q,SHROOM4:ENST00000289292.7:exon4:c.G1748A:p.R583Q,	Xp11.22	.
X	51487478	G	T	CCTGGTATTT,TCCTGGGCCT	exonic	GSPT2	nonsynonymous SNV	GSPT2:ENST00000340438.4:exon1:c.G756T:p.L252F,	Xp11.22	.
X	51488077	C	T	AAGCTGGAAT,CGGGTCCATT	exonic	GSPT2	nonsynonymous SNV	GSPT2:ENST00000340438.4:exon1:c.C1355T:p.S452F,	Xp11.22	.
X	53284082	G	A	GCTCAGGAAG,AGCCCCCATA	exonic	IQSEC2	nonsynonymous SNV	IQSEC2:ENST00000396435.3:exon4:c.C1031T:p.S344F,IQSEC2:ENST00000375368.5:exon3:c.C1001T:p.S334F,IQSEC2:ENST00000375365.2:exon4:c.C416T:p.S139F,	Xp11.22	.
X	54177781	G	A	GTCACAGGGA,GAAGAACCAG	exonic	FAM120C	nonsynonymous SNV	FAM120C:ENST00000375180.2:exon4:c.C1055T:p.P352L,FAM120C:ENST00000328235.4:exon4:c.C1055T:p.P352L,	Xp11.22	.
X	54177782	G	A	TCACAGGGAG,AAGAACCAGC	exonic	FAM120C	nonsynonymous SNV	FAM120C:ENST00000375180.2:exon4:c.C1054T:p.P352S,FAM120C:ENST00000328235.4:exon4:c.C1054T:p.P352S,	Xp11.22	.
X	55650271	G	A	CTCTTTAGCT,AGCAAATCCT	exonic	FOXR2	nonsynonymous SNV	FOXR2:ENST00000339140.3:exon1:c.G127A:p.E43K,	Xp11.21	.
X	63444876	G	A	CTTGGGACAC,AGCCAATAGG	exonic	ASB12,MTMF	nonsynonymous SNV	MTMR8:ENST00000453546.1:exon10:c.C1780T:p.R594C,ASB12:ENST00000396130.2:exon1:c.C628T:p.R210C,ASB12:ENST00000362002.2:exon2:c.C655T:p.R219C,	Xq11.2	ASB12;basechange=c.628C>T;AAchange=p.R210C;tissue=endometrium&MTMR8_ENST00000453546;basechange=c.1780C>T;AAchange=p.R594C;tissue=endometrium&ASB12_ENST00000362002;basechange=c.655C>T;AAchange=p.R219C;tissue=endometrium
X	86919862	T	C	CTCTACGTGG,TGGAGGATAT	exonic	KLHL4	nonsynonymous SNV	KLHL4:ENST00000373114.4:exon10:c.T2024C:p.V675A,KLHL4:ENST00000373119.4:exon10:c.T2024C:p.V675A,	Xq21.31	.
X	103294713	G	A	GACAGCTTCG,GGACAGCTTC	exonic	H2BFM	nonsynonymous SNV	H2BFM:ENST00000355016.3:exon1:c.G170A:p.G57E,H2BFM:ENST00000243297.5:exon3:c.G479A:p.G160E,	Xq22.2	.
X	104984558	G	A	AGAGCATCTT,GAGAAAAAGA	exonic	IL1RAPL2	nonsynonymous SNV	IL1RAPL2:ENST00000372582.1:exon8:c.G922A:p.G308R,IL1RAPL2:ENST00000344799.4:exon7:c.G922A:p.G308R,	Xq22.3	.
X	105868477	C	T	CAGCCTGTCC,CGTGGATCCA	exonic	CXorf57	nonsynonymous SNV	CXorf57:ENST00000421550.1:exon3:c.C368T:p.P123L,CXorf57:ENST00000372544.2:exon3:c.C944T:p.P315L,CXorf57:ENST00000372548.4:exon3:c.C944T:p.P315L,CXorf57:ENST00000461251.1:exon3:c.C944T:p.P315L,	Xq22.3	.
X	107418431	G	A	ACTGGCCCCG,ATTGCCTCTG	exonic	COL4A6	nonsynonymous SNV	COL4A6:ENST00000334504.7:exon30:c.C2845T:p.P949S,COL4A6:ENST00000394872.2:exon30:c.C2848T:p.P950S,COL4A6:ENST00000545689.1:exon30:c.C2845T:p.P949S,COL4A6:ENST00000372216.4:exon30:c.C2848T:p.P950S,COL4A6:ENST00000538570.1:exon30:c.C2845T:p.P949S,	Xq22.3	.
X	109931860	C	T	GCAGCATTTT,CGTCTATTTT	exonic	CHRDL1	nonsynonymous SNV	CHRDL1:ENST00000218054.4:exon9:c.G947A:p.G316E,CHRDL1:ENST00000434224.1:exon7:c.G710A:p.G237E,CHRDL1:ENST00000372042.1:exon9:c.G950A:p.G317E,CHRDL1:ENST00000394797.4:exon9:c.G947A:p.G316E,CHRDL1:ENST00000372045.1:exon9:c.G929A:p.G310E,CHRDL1:ENST00000482160.1:	Xq23	.
X	114425877	G	A	CCACCGCTAC,GAGGAGGAGG	exonic	RBMXL3	nonsynonymous SNV	RBMXL3:ENST00000424776.3:exon1:c.G1873A:p.G625R,	Xq23	RBMXL3;basechange=c.1873G>A;AAchange=p.G625R;tissue=skin
X	117744305	C	T	GCAATAATTC,CCATGTGACT	exonic	DOCK11	nonsynonymous SNV	DOCK11:ENST00000276204.6:exon28:c.C3020T:p.P1007L,DOCK11:ENST00000276202.7:exon28:c.C3020T:p.P1007L,	Xq24	.
X	131762679	C	T	ACACTAGAGG,CCTAGTGGTA	exonic	HS6ST2	nonsynonymous SNV	HS6ST2:ENST00000406696.3:exon2:c.G568A:p.A190T,HS6ST2:ENST00000521489.1:exon6:c.G1510A:p.A504T,HS6ST2:ENST00000370833.2:exon4:c.G1072A:p.A358T,HS6ST2:ENST00000370836.2:exon4:c.G1390A:p.A464T,	Xq26.2	.
X	136112323	G	A	AAAAGTAGCA,AATCGTAGGA	exonic	GPR101	nonsynonymous SNV	GPR101:ENST00000298110.1:exon1:c.C1511T:p.S504F,	Xq26.3	.
X	149681260	G	A	GCTGATCGAC,ACATTTTGGA	exonic	MAMLD1	nonsynonymous SNV	MAMLD1:ENST00000432680.2:exon5:c.G2914A:p.D972N,	Xq28	.
X	150912754	G	A	CAACCAGCAT,GAGGTCGACG	exonic	CNGA2	nonsynonymous SNV	CNGA2:ENST00000329903.4:exon6:c.G1779A:p.M593I,	Xq28	.
X	153694734	C	G	AACGTTTGAC,AAGTGAGTCC	exonic	PLXNA3	nonsynonymous SNV	PLXNA3:ENST00000369682.3:exon16:c.C2815G:p.Q939E,	Xq28	.
X	153927718	G	A	ACCAGCCTGG,GGCTCATGGG	exonic	GAB3	nonsynonymous SNV	GAB3:ENST00000369568.4:exon6:c.C1196T:p.P399L,GAB3:ENST00000424127.2:exon6:c.C1196T:p.P399L,GAB3:ENST00000369575.3:exon6:c.C1193T:p.P398L,	Xq28	.

C000-S5ZHWR

Chromosom	Position (hg'	Reference al	Mutant allele	Sequence context	ANNOVAR fu	Gene	Mutation type	ANNOVAR output (alternative transcripts)
1	1355494	C	T	TCCCGGGCGT,CACCCGCGCC	exonic	ANKRD65	nonsynonymous SNV	ANKRD65:ENST00000537107.1:exon3:c.G688A:p.D230N,
1	16263924	C	G	CGACTTCCTT,CCCTCCCCTG	exonic	SPEN	nonsynonymous SNV	SPEN:ENST00000375759.3:exon12:c.C10293G:p.F3431L,
1	19524507	G	A	GCTGAGGCTT,ATCTGTAGAT	exonic	UBR4	stopgain	UBR4:ENST00000375254.3:exon6:c.C685T:p.Q229X,UBR4:ENST00000375217.2:exon6:c.C685T:p.Q229X,UBR4:ENST00000375267.2:exon6:c.C685T:p.Q229X,UBR4:ENST00000375226.2:exon6:c.C685T:p.Q229X,
1	55567259	C	G	GAAATCACCT,AGGGAGCCCA	exonic	USP24	nonsynonymous SNV	USP24:ENST00000294383.6:exon43:c.G5143C:p.E1715Q,USP24:ENST00000407756.1:exon40:c.G4663C:p.E1555Q,
1	162722929	G	C	CCAGATTCCA,ATGAGGACAT	exonic	DDR2	nonsynonymous SNV	DDR2:ENST00000446985.1:exon4:c.G127C:p.D43H,DDR2:ENST00000415555.1:exon4:c.G127C:p.D43H,DDR2:ENST00000367922.3:exon5:c.G127C:p.D43H,DDR2:ENST00000367921.3:exon4:c.G127C:p.D43H,
1	168034972	C	T	GCAGCCACAT,CGTTTGACCC	exonic	DCAF6	nonsynonymous SNV	DCAF6:ENST00000312263.6:exon16:c.C2311T:p.P771S,DCAF6:ENST00000432587.2:exon18:c.C2491T:p.P831S,DCAF6:ENST00000367843.3:exon17:c.C2371T:p.P791S,DCAF6:ENST00000367840.3:exon19:c.C2584T:p.P862S,
1	242271125	A	C	TTTGCATCCA,GTCTGGCCAG	exonic	PLD5	nonsynonymous SNV	PLD5:ENST00000536534.2:exon8:c.T1087G:p.L363V,PLD5:ENST00000427495.1:exon8:c.T901G:p.L301V,PLD5:ENST00000442594.2:exon9:c.T811G:p.L271V,
1	242287957	A	T	GATGACACCG,GTTCTTTCAT	exonic	PLD5	nonsynonymous SNV	PLD5:ENST00000536534.2:exon6:c.T746A:p.L249H,PLD5:ENST00000427495.1:exon6:c.T560A:p.L187H,PLD5:ENST00000442594.2:exon7:c.T470A:p.L157H,
2	25358336	G	A	TCCCTTGCAG,TATCCACAGG	exonic	EFR3B	nonsynonymous SNV	EFR3B:ENST00000401432.3:exon13:c.G1312A:p.V438I,EFR3B:ENST00000264719.5:exon9:c.G949A:p.V317I,EFR3B:ENST00000405108.1:exon10:c.G868A:p.V290I,EFR3B:ENST00000402191.1:exon13:c.G1207A:p.V403I,EFR3B:ENST00000403714.3:exon13:c.G1312A:p.V4
2	54120833	G	A	AAAACGTCGT,GATTAAACTT	exonic	PSME4	nonsynonymous SNV	PSME4:ENST00000421748.2:exon15:c.C1448T:p.P483L,PSME4:ENST00000404125.1:exon35:c.C4016T:p.P1339L,
2	54120834	G	A	AAACGTCGTG,ATTAAACTTA	exonic	PSME4	nonsynonymous SNV	PSME4:ENST00000421748.2:exon15:c.C1447T:p.P483S,PSME4:ENST00000404125.1:exon35:c.C4015T:p.P1339S,
2	97790279	C	T	TGTGCTTTCT,GAGATGCGTT	exonic	ANKRD36	stopgain	ANKRD36:ENST00000461153.2:exon5:c.C676T:p.R226X,ANKRD36:ENST00000420699.2:exon5:c.C676T:p.R226X,
2	98887171	A	G	CCAATACAGT,CTTGGAAAAC	exonic	VWA3B	nonsynonymous SNV	VWA3B:ENST00000477737.1:exon22:c.A2870G:p.Y957C,
2	116598332	T	G	GCAGCAAAAG,TCATTTCCAA	exonic	DPP10	nonsynonymous SNV	DPP10:ENST00000409163.1:exon26:c.T2039G:p.V680G,DPP10:ENST00000410059.1:exon25:c.T2189G:p.V730G,DPP10:ENST00000310323.8:exon25:c.T2168G:p.V723G,DPP10:ENST00000393147.2:exon25:c.T2201G:p.V734G,
2	133489388	G	C	GATGTGGAAT,AACAATGGCG	exonic	NCKAP5	nonsynonymous SNV	NCKAP5:ENST00000405974.3:exon13:c.C1408G:p.H470D,NCKAP5:ENST00000409213.1:exon15:c.C1408G:p.H470D,NCKAP5:ENST00000317721.6:exon15:c.C5365G:p.H1789D,NCKAP5:ENST00000409261.1:exon17:c.C5365G:p.H1789D,
2	160006959	T	G	CGATAGCCCC,GCTCAACCTT	exonic	TANC1	nonsynonymous SNV	TANC1:ENST00000263635.6:exon7:c.T574G:p.C192G,
2	166788363	A	T	AGCTTGGTGG,AGCCTAAAAC	exonic	TTC21B	nonsynonymous SNV	TTC21B:ENST00000243344.7:exon8:c.T799A:p.S267T,
2	167133576	T	G	TCCCACATGG,CTCTATCCAC	exonic	SCN9A	nonsynonymous SNV	SCN9A:ENST00000375387.4:exon16:c.A2794C:p.T932P,SCN9A:ENST00000409435.1:exon15:c.A2791C:p.T931P,SCN9A:ENST00000303354.6:exon16:c.A2794C:p.T932P,SCN9A:ENST00000409672.1:exon16:c.A2758C:p.T920P,
2	171243692	C	T	TCATGCACTG,CATCAATGAC	exonic	MYO3B	nonsynonymous SNV	MYO3B:ENST00000409044.3:exon14:c.C1451T:p.A484V,MYO3B:ENST00000408978.4:exon14:c.C1451T:p.A484V,MYO3B:ENST00000317935.6:exon14:c.C1451T:p.A484V,MYO3B:ENST00000334231.6:exon14:c.C1478T:p.A493V,MYO3B:ENST00000484338.2:exon14:c.C14
2	178364352	G	A	GTAGTTTAAA,GATTTGACCC	exonic	AGPS	nonsynonymous SNV	AGPS:ENST00000264167.4:exon14:c.G1369A:p.G457R,
2	178364353	G	A	TAGTTTAAAG,ATTTGACCCA	exonic	AGPS	nonsynonymous SNV	AGPS:ENST00000264167.4:exon14:c.G1370A:p.G457E,
2	208633332	G	C	GGTTGTAGCC,ATGCCGCGGC	exonic	FZD5	nonsynonymous SNV	FZD5:ENST00000295417.3:exon2:c.C132G:p.I44M,
2	210559038	G	A	ATAGCCCTTG,ATTTAACTTT	exonic	MAP2	nonsynonymous SNV	MAP2:ENST00000360351.4:exon7:c.G2144A:p.G715E,MAP2:ENST00000447185.1:exon4:c.G2132A:p.G711E,
2	210561016	T	A	ATTACAAAGA,GAGACCACCA	exonic	MAP2	nonsynonymous SNV	MAP2:ENST00000360351.4:exon7:c.T4122A:p.D1374E,MAP2:ENST00000447185.1:exon4:c.T4110A:p.D1370E,
2	216977781	A	T	TACCATGAGT,ACTCCATTCC	exonic	XRCC5	nonsynonymous SNV	XRCC5:ENST00000417391.1:exon3:c.A25T:p.N9Y,XRCC5:ENST00000392133.3:exon4:c.A64T:p.N22Y,XRCC5:ENST00000392132.2:exon2:c.A64T:p.N22Y,
2	227875166	T	G	GCCACCGAGG,ATCCAGGGCC	exonic	COL4A4	nonsynonymous SNV	COL4A4:ENST00000329662.7:exon45:c.A4376C:p.Y1459S,COL4A4:ENST00000396625.3:exon46:c.A4385C:p.Y1462S,
3	42680981	T	C	CCACAAGGCT,GAGAATAGAA	exonic	NKTR	nonsynonymous SNV	NKTR:ENST00000232978.8:exon13:c.T3785C:p.L1262S,
3	53853648	C	A	TGGGGGACCC,AGGCTGGCTG	exonic	CHDH	nonsynonymous SNV	CHDH:ENST00000315251.6:exon7:c.G1174T:p.G392W,
3	69169145	C	T	TTGAGCTTTT,TTTTAAATAC	exonic	LMOD3	nonsynonymous SNV	LMOD3:ENST00000420581.2:exon2:c.G361A:p.E121K,LMOD3:ENST00000475434.1:exon3:c.G361A:p.E121K,LMOD3:ENST00000489031.1:exon3:c.G361A:p.E121K,
3	81695619	G	A	GCCATCAACT,AATGCAGTTG	exonic	GBE1	stopgain	GBE1:ENST00000489715.1:exon6:c.C583T:p.Q195X,GBE1:ENST00000429644.2:exon6:c.C706T:p.Q236X,
3	97983369	A	G	ATTCCTTGGG,GTTTAGCCTT	exonic	OR5H6	nonsynonymous SNV	OR5H6:ENST00000383696.2:exon1:c.A241G:p.S81G,
3	112358356	G	A	GACCCCGAAG,GAAACGCAAC	exonic	CCDC80	nonsynonymous SNV	CCDC80:ENST00000206423.3:exon2:c.C397T:p.P133S,CCDC80:ENST00000439685.2:exon2:c.C397T:p.P133S,
3	121190917	G	A	CCGCTGAAGG,GAAAGACCAC	exonic	POLQ	nonsynonymous SNV	POLQ:ENST00000264233.5:exon22:c.C6638T:p.P2213L,
3	121341852	C	T	AAACCATTTC,GTCCCCCAGG	exonic	FBXO40	nonsynonymous SNV	FBXO40:ENST00000338040.4:exon3:c.C1576T:p.R526C,
3	121412864	A	T	GTTCAAAGTA,CCTGAATCTC	exonic	GOLGB1	nonsynonymous SNV	GOLGB1:ENST00000340645.5:exon13:c.T6491A:p.V2164D,GOLGB1:ENST00000393667.3:exon13:c.T6506A:p.V2169D,
3	122422726	G	A	CTGTCAGCAT,GGCACAGTGC	exonic	PARP14	nonsynonymous SNV	PARP14:ENST00000460683.1:exon4:c.G2742A:p.M914I,PARP14:ENST00000474629.2:exon7:c.G3219A:p.M1073I,
3	122842927	G	C	TGGCCGGGAT,AATGTCTACT	exonic	PDIA5	nonsynonymous SNV	PDIA5:ENST00000489923.1:exon9:c.G624C:p.M208I,PDIA5:ENST00000316218.7:exon9:c.G624C:p.M208I,
3	137843566	C	T	ACTAGAGTAC,GAGAAGCCTG	exonic	A4GNT	nonsynonymous SNV	A4GNT:ENST00000236709.3:exon3:c.G563A:p.R188Q,
3	154836563	C	T	GGCCTCCCTT,TAGAGATTAC	exonic	MME	nonsynonymous SNV	MME:ENST00000360490.2:exon8:c.C683T:p.S228F,MME:ENST00000492661.1:exon8:c.C683T:p.S228F,MME:ENST00000460393.1:exon8:c.C683T:p.S228F,MME:ENST00000462745.1:exon8:c.C683T:p.S228F,MME:ENST00000493237.1:exon8:c.C683T:p.S228F,MME:ENST
3	155311896	C	T	CAGCTGGGGT,GAAGTTCCCC	exonic	PLCH1	nonsynonymous SNV	PLCH1:ENST00000460012.1:exon4:c.G214A:p.D72N,PLCH1:ENST00000494598.1:exon3:c.G268A:p.D90N,PLCH1:ENST00000447496.2:exon3:c.G268A:p.D90N,PLCH1:ENST00000340059.7:exon3:c.G268A:p.D90N,PLCH1:ENST00000414191.1:exon3:c.G214A:p.D72N,PLCH
3	158415744	T	C	AAATCAATTG,ATCATCATTA	exonic	RARRES1	nonsynonymous SNV	RARRES1:ENST00000237696.5:exon5:c.A688G:p.T230A,
3	173998866	C	T	GTCCATTCAT,CACATGAGGT	exonic	NLGN1	nonsynonymous SNV	NLGN1:ENST00000361589.4:exon6:c.C2245T:p.P749S,NLGN1:ENST00000401917.3:exon7:c.C2365T:p.P789S,NLGN1:ENST00000545397.1:exon5:c.C2245T:p.P749S,NLGN1:ENST00000457714.1:exon7:c.C2245T:p.P749S,
3	183524803	G	A	GAAGGAGCAG,AAGAGAAACA	exonic	YEATS2	nonsynonymous SNV	YEATS2:ENST00000305135.5:exon28:c.G3934A:p.E1312K,
3	185183581	G	A	GCTTGAGCGG,CGAATAATTT	exonic	MAP3K13	nonsynonymous SNV	MAP3K13:ENST00000265026.3:exon9:c.G1435A:p.A479T,MAP3K13:ENST00000424227.1:exon10:c.G1435A:p.A479T,MAP3K13:ENST00000443863.1:exon9:c.G1003A:p.A335T,MAP3K13:ENST00000446828.1:exon8:c.G814A:p.A272T,MAP3K13:ENST00000535426.1:exon8:
3	196888552	C	A	TCAGCAGGGA,TGGCAGGACA	exonic	DLG1	nonsynonymous SNV	DLG1:ENST00000448528.2:exon6:c.G541T:p.V181F,DLG1:ENST00000346964.2:exon6:c.G541T:p.V181F,DLG1:ENST00000419354.1:exon6:c.G541T:p.V181F,DLG1:ENST00000392380.2:exon6:c.G541T:p.V181F,DLG1:ENST00000392381.3:exon6:c.G541T:p.V181F,
4	57877037	C	T	AGAAATTTCT,CTGCAGCTAT	exonic	POLR2B	nonsynonymous SNV	POLR2B:ENST00000314595.5:exon12:c.C1672T:p.P558S,POLR2B:ENST00000381227.1:exon13:c.C1672T:p.P558S,POLR2B:ENST00000431623.2:exon11:c.C1447T:p.P483S,POLR2B:ENST00000441246.2:exon13:c.C1651T:p.P551S,
4	156283217	C	T	AGATGCGTTT,CCTCAGATCC	exonic	MAP9	nonsynonymous SNV	MAP9:ENST00000311277.4:exon6:c.G785A:p.G262E,MAP9:ENST00000433024.1:exon6:c.G782A:p.G261E,MAP9:ENST00000515654.1:exon6:c.G785A:p.G262E,MAP9:ENST00000424373.1:exon6:c.G785A:p.G262E,
4	170674842	G	A	GGCTTTTCAG,ATCAAGTTCT	exonic	C4orf27	nonsynonymous SNV	C4orf27:ENST00000393381.2:exon2:c.C193T:p.P65S,
5	240473	G	T	TTTGTTTTAG,AGATAAAGTC	exonic	SDHA	nonsynonymous SNV	SDHA:ENST00000264932.6:exon11:c.G1433T:p.G478V,SDHA:ENST00000510361.1:exon10:c.G1289T:p.G430V,SDHA:ENST00000504309.1:exon11:c.G1433T:p.G478V,
5	7804732	A	G	GGAGTTGAAA,GATTAAGACC	exonic	ADCY2	nonsynonymous SNV	ADCY2:ENST00000537121.1:exon19:c.A2270G:p.K757R,ADCY2:ENST00000338316.4:exon22:c.A2810G:p.K937R,
5	13769649	T	G	GCTCCTTTTC,TTCGCTTCCA	exonic	DNAH5	nonsynonymous SNV	DNAH5:ENST00000265104.4:exon57:c.A9681C:p.K3227N,
5	34823290	C	G	TTAGAGAGCT,TGAAGCAGAG	exonic	RAI14	nonsynonymous SNV	RAI14:ENST00000397449.1:exon14:c.C1322G:p.S441C,RAI14:ENST00000512629.1:exon14:c.C1256G:p.S419C,RAI14:ENST00000428746.2:exon15:c.C1343G:p.S448C,RAI14:ENST00000503673.1:exon16:c.C1343G:p.S448C,RAI14:ENST00000265109.3:exon15:c.C1343G:p
5	36958268	C	T	TTGTTGCAGG,CGTCCTGGCA	exonic	NIPBL	nonsynonymous SNV	NIPBL:ENST00000282516.8:exon4:c.C293T:p.A98V,NIPBL:ENST00000448238.2:exon4:c.C293T:p.A98V,
5	36976244	G	A	GATATAAACC,CCCACTAAAT	exonic	NIPBL	nonsynonymous SNV	NIPBL:ENST00000282516.8:exon9:c.G1235A:p.R412H,NIPBL:ENST00000448238.2:exon9:c.G1235A:p.R412H,
5	37020666	C	T	AGGAACACAT,ATGCAAAGGA	exonic	NIPBL	nonsynonymous SNV	NIPBL:ENST00000282516.8:exon26:c.C5116T:p.H1706Y,NIPBL:ENST00000448238.2:exon26:c.C5116T:p.H1706Y,
5	37157793	G	A	AAATTATGTG,GGGAACAGCA	exonic	C5orf42	nonsynonymous SNV	C5orf42:ENST00000514429.1:exon25:c.C5134T:p.P1712S,C5orf42:ENST00000274258.7:exon40:c.C4630T:p.P1544S,C5orf42:ENST00000509849.1:exon25:c.C5002T:p.P1668S,C5orf42:ENST00000508244.1:exon39:c.C7936T:p.P2646S,C5orf42:ENST00000425232.2:exon40:
5	41007535	T	A	CTTGCAAACA,TTTAAAGTAG	exonic	MROH2B	nonsynonymous SNV	MROH2B:ENST00000506092.2:exon24:c.A2295T:p.K765N,MROH2B:ENST00000399564.4:exon34:c.A3630T:p.K1210N,
5	41047851	G	A	CCATAGAACG,TACTGATGTT	exonic	MROH2B	nonsynonymous SNV	MROH2B:ENST00000506092.2:exon7:c.C365T:p.T122I,MROH2B:ENST00000399564.4:exon17:c.C1700T:p.T567I,
5	74016969	A	T	CAACCTCTTT,TGCTGGATAT	exonic	HEXB	nonsynonymous SNV	HEXB:ENST00000509579.1:exon3:c.A77T:p.Y26F,HEXB:ENST00000511181.1:exon14:c.A965T:p.Y322F,HEXB:ENST00000261416.7:exon14:c.A1640T:p.Y547F,
5	108521924	G	A	AGAGAGTGAC,TGTGGAGCTT	exonic	FER	nonsynonymous SNV	FER:ENST00000281092.4:exon19:c.G2227A:p.V743M,FER:ENST00000438717.2:exon15:c.G1702A:p.V568M,
5	134785515	C	T	TGCCCCCGTC,GAGAAGCAGA	exonic	TIFAB	nonsynonymous SNV	TIFAB:ENST00000537858.1:exon2:c.G115A:p.G39R,
5	137223057	G	A	TGGACTCTAT,AAAGTGAAGA	exonic	MYOT	nonsynonymous SNV	MYOT:ENST00000421631.2:exon10:c.G928A:p.E310K,MYOT:ENST00000515645.1:exon11:c.G1135A:p.E379K,MYOT:ENST00000239926.4:exon10:c.G1480A:p.E494K,
5	150664228	C	G	GCAAGAAGGT,TTCCAGTTTG	exonic	SLC36A3	nonsynonymous SNV	SLC36A3:ENST00000335230.3:exon7:c.G753C:p.K251N,SLC36A3:ENST00000377713.3:exon8:c.G876C:p.K292N,
5	167303041	C	G	ATCTGCTCAG,TGCCTAGCTC	exonic	TENM2	nonsynonymous SNV	TENM2:ENST00000519204.1:exon2:c.C190G:p.L64V,TENM2:ENST00000403607.2:exon1:c.C52G:p.L18V,TENM2:ENST00000518659.1:exon3:c.C553G:p.L185V,TENM2:ENST00000545108.1:exon3:c.C553G:p.L185V,
5	174936087	T	C	GAGAGCAAAG,ACATCTATGA	exonic	SFXN1	nonsynonymous SNV	SFXN1:ENST00000506963.1:exon4:c.T217C:p.Y73H,SFXN1:ENST00000502393.1:exon3:c.T217C:p.Y73H,SFXN1:ENST00000507017.1:exon3:c.T217C:p.Y73H,SFXN1:ENST00000321442.5:exon3:c.T217C:p.Y73H,
6	29342286	C	T	TGAAGCAGGA,GAATATATGT	exonic	OR12D3	nonsynonymous SNV	OR12D3:ENST00000396806.3:exon1:c.G779A:p.R260H,
6	31239621	T	G	CACGGCGGTG,CGAAATACCT	exonic	HLA-C	nonsynonymous SNV	HLA-C:ENST00000376237.4:exon2:c.A98C:p.D33A,HLA-C:ENST00000383329.3:exon2:c.A98C:p.D33A,HLA-C:ENST00000376228.5:exon2:c.A98C:p.D33A,
6	32489852	A	G	GTCGAAGCGC,AGTCCTCCTC	exonic	HLA-DRB5	nonsynonymous SNV	HLA-DRB5:ENST00000374975.3:exon2:c.T200C:p.L67S,
6	43528738	C	T	ACATCAGAAT,TGCACCCTGT	exonic	XPO5	nonsynonymous SNV	XPO5:ENST00000496341.2:exon2:c.G94A:p.D32N,XPO5:ENST00000265351.7:exon10:c.G1018A:p.D340N,
6	51907686	A	G	AGGCTCCACC,TATCCAGTCT	exonic	PKHD1	nonsynonymous SNV	PKHD1:ENST00000340994.4:exon27:c.T3068C:p.M1023T,PKHD1:ENST00000371117.3:exon27:c.T3068C:p.M1023T,
6	56468081	C	G	AGGCGGTCTT,AAACTCTGGT	exonic	DST	nonsynonymous SNV	DST:ENST00000361203.3:exon38:c.G10446C:p.L3482F,DST:ENST00000446842.2:exon28:c.G9468C:p.L3156F,DST:ENST00000312431.6:exon38:c.G10446C:p.L3482F,DST:ENST00000370769.4:exon38:c.G10446C:p.L3482F,DST:ENST00000370754.5:exon41:c.G10980C:p
6	70976427	C	T	TACCTTGTGT,CTTGTCTTCC	exonic	COL9A1	nonsynonymous SNV	COL9A1:ENST00000370499.4:exon12:c.G605A:p.G202E,COL9A1:ENST00000320755.7:exon12:c.G605A:p.G202E,COL9A1:ENST00000357250.6:exon18:c.G1334A:p.G445E,
6	74229740	C	T	TGAGTCTTTT,CTTTCCCATT	exonic	EEF1A1	nonsynonymous SNV	EEF1A1:ENST00000455918.1:exon2:c.G10A:p.E4K,EEF1A1:ENST00000331523.2:exon2:c.G10A:p.E4K,EEF1A1:ENST00000356303.2:exon2:c.G10A:p.E4K,EEF1A1:ENST00000316292.9:exon1:c.G10A:p.E4K,EEF1A1:ENST00000309268.6:exon2:c.G10A:p.E4K,
6	109714130	C	T	TCTGTTCCTT,AATCAGTTGC	exonic	PPIL6	nonsynonymous SNV	PPIL6:ENST00000440797.2:exon9:c.G913A:p.E305K,PPIL6:ENST00000424445.2:exon7:c.G739A:p.E247K,PPIL6:ENST00000521072.2:exon8:c.G835A:p.E279K,
6	133072613	C	T	GTCTTCATGT,ATAATGATAC	exonic	VNN2	nonsynonymous SNV	VNN2:ENST00000392389.2:exon5:c.G867A:p.M289I,VNN2:ENST00000525270.1:exon6:c.G712A:p.D238N,VNN2:ENST00000326499.6:exon5:c.G871A:p.D291N,VNN2:ENST00000525674.1:exon4:c.G582A:p.M194I,
6	138583997	G	T	TGCGCCTTGA,GAGCTGAAGG	exonic	KIAA1244	nonsynonymous SNV	KIAA1244:ENST00000251691.4:exon12:c.G1377T:p.E459D,
7	12664680	G	A	GGTGGCAGAA,AAAACCCCTT	exonic	SCIN	nonsynonymous SNV	SCIN:ENST00000341757.5:exon6:c.G805A:p.E269K,SCIN:ENST00000297029.5:exon6:c.G805A:p.E269K,SCIN:ENST00000519209.1:exon4:c.G64A:p.E22K,SCIN:ENST00000445618.2:exon3:c.G64A:p.E22K,
7	20685666	T	G	TGCTGTGTAC,TCTTTATGAA	exonic	ABCB5	nonsynonymous SNV	ABCB5:ENST00000404938.2:exon9:c.T886G:p.F296V,
7	22233138	C	G	CTGCTCCATT,ATCGTCCTCA	exonic	RAPGEF5	nonsynonymous SNV	RAPGEF5:ENST00000401957.2:exon1:c.G142C:p.E48Q,RAPGEF5:ENST00000451559.1:exon1:c.G142C:p.E48Q,
7	44180104	C	T	TCCCTCCATC,TGGCCACAGG	exonic	MYL7	nonsynonymous SNV	MYL7:ENST00000447951.1:exon3:c.G203A:p.R68K,
7	77033897	A	G	TGGTTTGACA,TCATATAATC	exonic	GSAP	nonsynonymous SNV	GSAP:ENST00000430584.1:exon3:c.T74C:p.I25T,
7	87280137	G	A	TTTTTTCATA,GTTTTCTGTG	splicing	RUNDC3B(E!	.	.
7	89854482	G	A	AAAGATGCAA,GAAGGTCACT	exonic	STEAP2	nonsynonymous SNV	STEAP2:ENST00000402625.2:exon2:c.G86A:p.R29K,STEAP2:ENST00000394632.1:exon3:c.G86A:p.R29K,STEAP2:ENST00000394629.2:exon3:c.G86A:p.R29K,STEAP2:ENST00000394621.2:exon3:c.G86A:p.R29K,STEAP2:ENST00000287908.3:exon2:c.G86A:p.R29K,STE/
7	95216347	C	T	AGCATCTGTT,CATATCCTGA	exonic	PDK4	nonsynonymous SNV	PDK4:ENST00000005178.5:exon10:c.G1070A:p.G357E,
7	99436744	G	A	TTCTGCCAGG,TCTTTGGAAT	exonic	CYP3A43	nonsynonymous SNV	CYP3A43:ENST00000434806.1:exon3:c.G167A:p.G56D,CYP3A43:ENST00000312017.5:exon3:c.G167A:p.G56D,CYP3A43:ENST00000436834.1:exon2:c.G73A:p.V25I,CYP3A43:ENST00000417625.1:exon4:c.G289A:p.V97I,CYP3A43:ENST00000222382.5:exon3:c.G167A:p.G
7	103126863	C	T	GCAGTCATCA,CTAGAGGACA	exonic	RELN	nonsynonymous SNV	RELN:ENST00000343529.5:exon61:c.G9764A:p.G3255D,RELN:ENST00000428762.1:exon61:c.G9764A:p.G3255D,RELN:ENST00000424685.2:exon61:c.G9764A:p.G3255D,
7	146741006	C	T	CAGGCATTTC,CGGAAACATT	exonic	CNTNAP2	nonsynonymous SNV	CNTNAP2:ENST00000361727.3:exon4:c.C410T:p.P137L,
8	2808705	A	C	GTTGCATTGA,CCAGTCAACA	exonic	CSMD1	nonsynonymous SNV	CSMD1:ENST00000520002.1:exon67:c.T10135G:p.F3379V,CSMD1:ENST00000542608.1:exon63:c.T9601G:p.F3201V,CSMD1:ENST00000602557.1:exon67:c.T10135G:p.F3379V,CSMD1:ENST00000602723.1:exon64:c.T9604G:p.F3202V,CSMD1:ENST00000400186.3:exon6
8	2886864	C	T	CCTCTCATCT,CTATGTTCCA	exonic	CSMD1	nonsynonymous SNV	CSMD1:ENST00000520002.1:exon52:c.G7835A:p.G2612E,CSMD1:ENST00000542608.1:exon51:c.G7832A:p.G2611E,CSMD1:ENST00000602557.1:exon52:c.G7835A:p.G2612E,CSMD1:ENST00000602723.1:exon52:c.G7835A:p.G2612E,CSMD1:ENST00000400186.3:exon5
8	3046416	C	T	CACCTGAATT,CCGAGCCCTC	exonic	CSMD1	nonsynonymous SNV	CSMD1:ENST00000539096.1:exon35:c.G5516A:p.G1839E,CSMD1:ENST00000520002.1:exon36:c.G5519A:p.G1840E,CSMD1:ENST00000542608.1:exon35:c.G5516A:p.G1839E,CSMD1:ENST00000602557.1:exon36:c.G5519A:p.G1840E,CSMD1:ENST00000602723.1:exon3
8	25708118	C	T	ACCTCTGAAT,CATTTCCATT	exonic	EBF2	nonsynonymous SNV	EBF2:ENST00000408929.3:exon14:c.G1244A:p.G415E,EBF2:ENST00000520164.1:exon15:c.G1688A:p.G563E,
8	35624420	G	A	TCCCCTGCAG,AAGTCCCGTT	exonic	UNC5D	nonsynonymous SNV	UNC5D:ENST00000404895.2:exon15:c.G2314A:p.E772K,UNC5D:ENST00000453357.2:exon15:c.G2299A:p.E767K,UNC5D:ENST00000449677.1:exon7:c.G1042A:p.E348K,UNC5D:ENST00000420357.1:exon13:c.G2113A:p.E705K,UNC5D:ENST00000416672.1:exon16:c.G23
8	41791558	C	T	TCGTGGTCGT,CTCAGACCCA	exonic	KAT6A	nonsynonymous SNV	KAT6A:ENST00000265713.2:exon17:c.G4180A:p.D1394N,KAT6A:ENST00000396930.3:exon18:c.G4180A:p.D1394N,KAT6A:ENST00000406337.1:exon18:c.G4180A:p.D1394N,
8	54155443	G	A	TTCATCACAT,GTTTTGTCGT	exonic	OPRK1	nonsynonymous SNV	OPRK1:ENST00000522508.1:exon3:c.C287T:p.P96L,
8	73944295	C	G	AGAATCTTTT,TTATCTAAGT	exonic	TERF1	nonsynonymous SNV	TERF1:ENST00000276602.6:exon7:c.C906G:p.F302L,TERF1:ENST00000276603.5:exon8:c.C966G:p.F322L,
8	77764346	T	C	GAAGCACTGC,GCAGTTTCAG	exonic	ZFHX4	nonsynonymous SNV	ZFHX4:ENST00000455469.2:exon10:c.T5054C:p.L1685P,ZFHX4:ENST00000518282.1:exon9:c.T5111C:p.L1704P,ZFHX4:ENST00000050961.6:exon10:c.T5054C:p.L1685P,ZFHX4:ENST00000521891.2:exon10:c.T5189C:p.L1730P,
8	87235242	G	A	AGTCAGATAG,AAATGTTAAC	exonic	SLC7A13	nonsynonymous SNV	SLC7A13:ENST00000419776.2:exon2:c.C749T:p.S250F,SLC7A13:ENST00000297524.3:exon2:c.C776T:p.S259F,
8	87645093	G	A	ATTAAAGTTC,AACTGCCCAA	exonic	CNGB3	stopgain	CNGB3:ENST00000320005.5:exon11:c.C1207T:p.R403X,
8	101153889	G	A	ATTTGCCCTG,AAAAACCTGA	exonic	FBXO43	nonsynonymous SNV	FBXO43:ENST00000428847.2:exon2:c.C593T:p.S198F,
8	120255661	T	A	TCTTAGATTT,TGCCTTTATG	exonic	MAL2	nonsynonymous SNV	MAL2:ENST00000276681.6:exon5:c.T464A:p.F155Y,
8	144789321	G	C	GGTGGGCTGG,AGAAGGCCGG	exonic	CCDC166	nonsynonymous SNV	CCDC166:ENST00000542437.1:exon2:c.C863G:p.S288C,
9	14619665	C	G	GACATCTTTT,AATAGATGTT	exonic	ZDHHC21	nonsynonymous SNV	ZDHHC21:ENST00000380916.4:exon9:c.G637C:p.E213Q,
9	26997891	G	C	AGGCTTCTTA,AGGCACAAAT	exonic	LRRC19	nonsynonymous SNV	LRRC19:ENST00000380055.5:exon3:c.C430G:p.L144V,
9	32634889	G	A	CCCAGCCAAT,GAAGGGTCAG	exonic	TAF1L	nonsynonymous SNV	TAF1L:ENST00000242310.4:exon1:c.C689T:p.P230L,
9	33529047	G	A	CTGAAACGTG,CGCCAATCCA	exonic	ANKRD18B	nonsynonymous SNV	ANKRD18B:ENST00000290943.6:exon3:c.G371A:p.G124D,
9	37441894	G	A	CCATTCTCCG,TGTAACATCT	exonic	ZBTB5	nonsynonymous SNV	ZBTB5:ENST00000307750.4:exon2:c.C655T:p.P219S,
9	37974631	G	A	GCCAGGGCTG,GATGGTGACC	exonic	SHB	nonsynonymous SNV	SHB:ENST00000377707.3:exon3:c.C1042T:p.P348S,
9	71503969	A	G	TTTTGTGACC,GTGATGATGA	exonic	PIP5K1B	nonsynonymous SNV	PIP5K1B:ENST00000440050.1:exon7:c.A391G:p.S131G,PIP5K1B:ENST00000478500.1:exon8:c.A511G:p.S171G,PIP5K1B:ENST00000265382.3:exon7:c.A391G:p.S131G,PIP5K1B:ENST00000437200.1:exon7:c.A391G:p.S131G,PIP5K1B:ENST00000541509.1:exon6:c.A391G
9	72347117	T	C	GCATTATAGT,GCTTGGGTAT	exonic	PTAR1	nonsynonymous SNV	PTAR1:ENST00000340434.4:exon5:c.A580G:p.N194D,PTAR1:ENST00000377200.5:exon3:c.A343G:p.N115D,
9	87342592	G	A	CACATTTCTC,AATCTCCAAC	exonic	NTRK2	nonsynonymous SNV	NTRK2:ENST00000376213.1:exon11:c.G877A:p.E293K,NTRK2:ENST00000323115.4:exon8:c.G877A:p.E293K,NTRK2:ENST00000376214.1:exon11:c.G877A:p.E293K,NTRK2:ENST00000304053.6:exon9:c.G877A:p.E293K,NTRK2:ENST00000376208.1:exon9:c.G877A:p.E29
9	96019341	C	T	TCCAGCCTCC,AGGTGGGGGC	exonic	WNK2	stopgain	WNK2:ENST00000427277.2:exon10:c.C1138T:p.Q380X,WNK2:ENST00000448039.1:exon9:c.C2146T:p.Q716X,WNK2:ENST00000395477.2:exon10:c.C2302T:p.Q768X,WNK2:ENST00000349097.3:exon10:c.C1138T:p.Q380X,WNK2:ENST00000297954.4:exon10:c.C2302T:p.
9	101235582	G	A	GTACCAGCCC,GAATGATCCA	exonic	GABBR2	nonsynonymous SNV	GABBR2:ENST00000259455.2:exon6:c.C845T:p.P282L,
9	104238230	G	A	CACGAGGTTC,GCTCCACTAC	exonic	TMEM246	nonsynonymous SNV	TMEM246:ENST00000374847.1:exon3:c.C1145T:p.P382L,TMEM246:ENST00000374848.3:exon2:c.C1145T:p.P382L,TMEM246:ENST00000374851.1:exon4:c.C1145T:p.P382L,
9	104238231	G	A	ACGAGGTTCG,CTCCACTACA	exonic	TMEM246	nonsynonymous SNV	TMEM246:ENST00000374847.1:exon3:c.C1144T:p.P382S,TMEM246:ENST00000374848.3:exon2:c.C1144T:p.P382S,TMEM246:ENST00000374851.1:exon4:c.C1144T:p.P382S,
9	113096533	C	T	GGGACCACAC,GTTTCGAAGA	exonic	TXNDC8	nonsynonymous SNV	TXNDC8:ENST00000374507.4:exon2:c.G92A:p.R31Q,TXNDC8:ENST00000423740.2:exon2:c.G92A:p.R31Q,TXNDC8:ENST00000374511.3:exon2:c.G92A:p.R31Q,TXNDC8:ENST00000374510.4:exon2:c.G92A:p.R31Q,
9	123182071	G	A	CTTACCTTGA,AAAAACTGTT	exonic	CDK5RAP2	nonsynonymous SNV	CDK5RAP2:ENST00000360822.3:exon26:c.C4076T:p.S1359F,CDK5RAP2:ENST00000359309.3:exon26:c.C4049T:p.S1350F,CDK5RAP2:ENST00000416449.1:exon12:c.C2354T:p.S785F,CDK5RAP2:ENST00000349780.4:exon27:c.C4172T:p.S1391F,CDK5RAP2:ENST00000
9	125613450	A	G	TTCCACTGCC,TTCCATTTAG	exonic	RC3H2	nonsynonymous SNV	RC3H2:ENST00000357244.2:exon20:c.T3290C:p.M1097T,RC3H2:ENST00000373670.1:exon19:c.T3290C:p.M1097T,
9	131013090	C	T	GCTCCTGGGC,TCCGCCTGCT	exonic	DNM1	nonsynonymous SNV	DNM1:ENST00000475805.1:exon21:c.C2405T:p.P802L,DNM1:ENST00000372923.3:exon21:c.C2405T:p.P802L,DNM1:ENST00000341179.7:exon21:c.C2405T:p.P802L,DNM1:ENST00000393594.3:exon21:c.C2405T:p.P802L,DNM1:ENST00000486160.1:exon21:c.C2405T:p.P
9	131885403	T	C	GACCTTCGAG,ACAGAGTCTC	exonic	PPP2R4	nonsynonymous SNV	PPP2R4:ENST00000348141.5:exon3:c.T220C:p.Y74H,PPP2R4:ENST00000445241.1:exon3:c.T202C:p.Y68H,PPP2R4:ENST00000358994.4:exon4:c.T202C:p.Y68H,PPP2R4:ENST00000417728.1:exon3:c.T97C:p.Y33H,PPP2R4:ENST00000440346.1:exon3:c.T97C:p.Y33H,PF
9	134098287	G	A	GGCATCCCAA,GCTTTGGGTT	exonic	NUP214	nonsynonymous SNV	NUP214:ENST00000411637.2:exon32:c.G5842A:p.G1948S,NUP214:ENST00000483497.2:exon7:c.G2350A:p.G784S,NUP214:ENST00000359428.5:exon32:c.G5872A:p.G1958S,NUP214:ENST00000451030.1:exon32:c.G5875A:p.G1959S,
9	134098288	G	A	GCATCCCAAG,CTTTGGGTTT	exonic	NUP214	nonsynonymous SNV	NUP214:ENST00000411637.2:exon32:c.G5843A:p.G1948D,NUP214:ENST00000483497.2:exon7:c.G2351A:p.G784D,NUP214:ENST00000359428.5:exon32:c.G5873A:p.G1958D,NUP214:ENST00000451030.1:exon32:c.G5876A:p.G1959D,
9	137591911	G	A	GACCACACGG,GAAGCCTGGC	exonic	COL5A1	nonsynonymous SNV	COL5A1:ENST00000371817.3:exon3:c.G434A:p.G145E,
10	298328	G	A	CACTGCTGCT,GAACACATCC	exonic	ZMYND11	stopgain	ZMYND11:ENST00000381604.4:exon15:c.G1607A:p.W536X,ZMYND11:ENST00000381591.1:exon15:c.G1727A:p.W576X,ZMYND11:ENST00000309776.4:exon15:c.G1607A:p.W536X,ZMYND11:ENST00000397959.3:exon13:c.G1472A:p.W491X,ZMYND11:ENST0000038158
10	11985143	C	T	TCTTCTCCCT,ATCATCATCA	exonic	UPF2	nonsynonymous SNV	UPF2:ENST00000397053.2:exon17:c.G3199A:p.E1067K,UPF2:ENST00000357604.5:exon17:c.G3199A:p.E1067K,UPF2:ENST00000356352.2:exon16:c.G3199A:p.E1067K,
10	15561384	G	A	GAAACATTCG,AGTTGCCCAA	exonic	ITGA8	nonsynonymous SNV	ITGA8:ENST00000378076.3:exon29:c.C3010T:p.P1004S,
10	32197249	G	C	CCGGGAAAAT,ACCAAATGAG	exonic	ARHGAP12	nonsynonymous SNV	ARHGAP12:ENST00000396144.4:exon3:c.C535G:p.H179D,ARHGAP12:ENST00000344936.2:exon3:c.C535G:p.H179D,ARHGAP12:ENST00000375245.4:exon3:c.C535G:p.H179D,ARHGAP12:ENST00000311380.4:exon1:c.C535G:p.H179D,ARHGAP12:ENST00000375250.5:
10	47681709	C	T	TGTCAAGGAG,CACCACCTGT	exonic	ANTXRL	nonsynonymous SNV	ANTXRL:ENST00000537271.1:exon14:c.C1147T:p.P383S,ANTXRL:ENST00000447511.2:exon14:c.C1147T:p.P383S,
10	70719727	C	G	GGAGCCATCT,AAAATGACAT	exonic	DDX21	nonsynonymous SNV	DDX21:ENST00000354185.4:exon2:c.C253G:p.Q85E,
10	72513705	G	C	CCTGAGGCCC,ACGGCCCTGT	exonic	ADAMTS14	nonsynonymous SNV	ADAMTS14:ENST00000373207.1:exon19:c.G2879C:p.R960P,ADAMTS14:ENST00000373208.1:exon19:c.G2888C:p.R963P,
10	98133446	C	T	ACCGTGGGGT,TGGTTTCTTG	exonic	TLL2	nonsynonymous SNV	TLL2:ENST00000357947.3:exon19:c.G2569A:p.D857N,
10	101646361	G	A	ATTTAAGGGG,AGATGACAAG	exonic	DNMBP	nonsynonymous SNV	DNMBP:ENST00000342239.3:exon13:c.C3386T:p.S1129F,DNMBP:ENST00000324109.4:exon13:c.C3314T:p.S1105F,DNMBP:ENST00000543621.1:exon10:c.C1052T:p.S351F,DNMBP:ENST00000540316.1:exon5:c.C122T:p.S41F,
11	605709	C	T	AGGCCCGTCT,CGTGGGGCTT	exonic	PHRF1	nonsynonymous SNV	PHRF1:ENST00000534320.1:exon12:c.C1439T:p.S480F,PHRF1:ENST00000413872.2:exon12:c.C1433T:p.S478F,PHRF1:ENST00000416188.2:exon12:c.C1436T:p.S479F,PHRF1:ENST00000264555.5:exon12:c.C1439T:p.S480F,PHRF1:ENST00000533464.1:exon12:c.C1427
11	618683	T	C	CCGGGTGTGG,TGGTTGGTGG	exonic	CDHR5	nonsynonymous SNV	CDHR5:ENST00000397542.2:exon13:c.A1876G:p.T626A,CDHR5:ENST00000358353.3:exon14:c.A1876G:p.T626A,
11	1298462	G	A	CACCATGCCG,GGCTACAGAC	exonic	TOLLIP	nonsynonymous SNV	TOLLIP:ENST00000527886.1:exon6:c.C425T:p.P142L,TOLLIP:ENST00000263646.7:exon6:c.C548T:p.P183L,TOLLIP:ENST00000530541.1:exon5:c.C482T:p.P161L,TOLLIP:ENST00000542915.1:exon5:c.C482T:p.P161L,TOLLIP:ENST00000525159.1:exon5:c.C449T:p.P150L,
11	1605926	C	T	ACCGCAGCCC,CCTTGCAGCC	exonic	KRTAP5-1	nonsynonymous SNV	KRTAP5-1:ENST00000382171.2:exon1:c.G554A:p.G185E,
11	5011056	C	T	ACCTCCATCT,GCCAGGAAGA	exonic	MMP26	nonsynonymous SNV	MMP26:ENST00000380390.1:exon3:c.C278T:p.S93L,MMP26:ENST00000300762.1:exon2:c.C278T:p.S93L,
11	5255396	C	T	GGACAGATCC,CAAAGGACTC	exonic	HBD	nonsynonymous SNV	HBD:ENST00000429817.1:exon3:c.G140A:p.G47E,HBD:ENST00000380299.3:exon2:c.G140A:p.G47E,HBD:ENST00000292901.3:exon2:c.G140A:p.G47E,
11	5841693	G	A	GCTGTCGTGG,GAACTGTGGG	exonic	OR52N2	nonsynonymous SNV	OR52N2:ENST00000317037.2:exon1:c.G128A:p.G43E,
11	6898767	G	A	AAGGAATAAA,AAGTGAAGGC	exonic	OR10A4	nonsynonymous SNV	OR10A4:ENST00000379829.2:exon1:c.G889A:p.E297K,
11	11941727	C	T	TGCATTTATT,AGCCAGAGAT	exonic	USP47	stopgain	USP47:ENST00000339865.5:exon8:c.C790T:p.Q264X,USP47:ENST00000399455.2:exon10:c.C1054T:p.Q352X,USP47:ENST00000527733.1:exon9:c.C994T:p.Q332X,
11	17191236	G	A	TGTTGGTTCC,GATGTGAAGA	exonic	PIK3C2A	nonsynonymous SNV	PIK3C2A:ENST00000265970.7:exon1:c.C53T:p.P18L,PIK3C2A:ENST00000532035.1:exon3:c.C53T:p.P18L,PIK3C2A:ENST00000533645.1:exon2:c.C53T:p.P18L,
11	18313393	G	C	TCCCTTTTTT,ATTCATTAAC	exonic	HPS5	stopgain	HPS5:ENST00000438420.2:exon15:c.C1694G:p.S565X,HPS5:ENST00000349215.3:exon16:c.C2036G:p.S679X,HPS5:ENST00000396253.3:exon15:c.C1694G:p.S565X,
11	18424397	G	C	TGGATATCTT,ACCTACGTGG	exonic	LDHA	stoploss	LDHA:ENST00000536528.1:exon3:c.G137C:p.X46S,
11	18591778	G	A	CAGTTTTGGA,ATAGGGCAAA	exonic	UEVLD	nonsynonymous SNV	UEVLD:ENST00000379387.4:exon3:c.G274T:p.L92F,UEVLD:ENST00000543987.1:exon4:c.C340T:p.L114F,UEVLD:ENST00000535484.1:exon4:c.C226T:p.L76F,UEVLD:ENST00000300038.7:exon4:c.C340T:p.L114F,UEVLD:ENST00000320750.6:exon3:c.C274T:p.L92F,UEVL
11	20409300	C	T	GCCATGTGCT,GTTAGCGTCA	exonic	PRMT3	nonsynonymous SNV	PRMT3:ENST00000331079.6:exon1:c.C8T:p.S3L,PRMT3:ENST00000437750.2:exon1:c.C8T:p.S3L,PRMT3:ENST00000330796.5:exon1:c.C8T:p.S3L,PRMT3:ENST00000525188.1:exon1:c.C8T:p.S3L,PRMT3:ENST00000526583.1:exon1:c.C8T:p.S3L,
11	22301180	A	C	AGAAAAGTTA,TGACTATCAA	exonic	ANO5	nonsynonymous SNV	ANO5:ENST00000324559.8:exon22:c.A2611C:p.M871L,
11	30352661	A	T	GAACAATACA,TTTGCTTTCA	exonic	ARL14EP	nonsynonymous SNV	ARL14EP:ENST00000530909.1:exon1:c.A166T:p.I56F,ARL14EP:ENST00000282032.3:exon2:c.A166T:p.I56F,
11	33620431	G	A	TCAGGGAGAC,CTCACCCCAG	exonic	KIAA1549L	nonsynonymous SNV	KIAA1549L:ENST00000321505.4:exon12:c.G3926A:p.R1309H,KIAA1549L:ENST00000389726.3:exon12:c.G3944A:p.R1315H,
11	46393300	G	A	TCCTTCAAGA,GAAGTCCAGC	exonic	DGKZ	nonsynonymous SNV	DGKZ:ENST00000395574.3:exon9:c.G704A:p.R235K,DGKZ:ENST00000318201.8:exon8:c.G737A:p.R246K,DGKZ:ENST00000343674.6:exon9:c.G854A:p.R285K,DGKZ:ENST00000456247.2:exon9:c.G803A:p.R268K,DGKZ:ENST00000454345.1:exon10:c.G1370A:p.R457K,D
11	51412038	G	A	GCCACATAGC,ATCACAGGCC	exonic	OR4A5	nonsynonymous SNV	OR4A5:ENST00000319760.6:exon1:c.C358T:p.R120C,
11	55321970	A	C	TTTGTAACTG,GTTTGTCCTC	exonic	OR4C15	nonsynonymous SNV	OR4C15:ENST00000314644.2:exon1:c.A188C:p.E63A,
11	57886477	C	T	GACATAGGCT,CTACCACCAG	exonic	OR9I1	nonsynonymous SNV	OR9I1:ENST00000302610.1:exon1:c.G440A:p.G147E,
11	62435095	T	G	GGTTGGTGGC,TCCAGACATG	exonic	C11orf48	nonsynonymous SNV	C11orf48:ENST00000532208.1:exon4:c.A438C:p.E146D,C11orf48:ENST00000354588.3:exon4:c.A438C:p.E146D,C11orf48:ENST00000528115.1:exon2:c.A47C:p.K16T,C11orf48:ENST00000415855.2:exon4:c.A47C:p.K16T,C11orf48:ENST00000431002.2:exon2:c.A516C:p.E1
11	63988591	G	A	CCCGACTTCG,CATCTCCTAT	exonic	FERMT3	nonsynonymous SNV	FERMT3:ENST00000345728.5:exon13:c.G1649A:p.G550D,FERMT3:ENST00000279227.5:exon13:c.G1661A:p.G554D,
11	65373447	G	A	AGGCTTGGGG,AACTGGGACC	exonic	MAP3K11	nonsynonymous SNV	MAP3K11:ENST00000309100.3:exon7:c.C1709T:p.S570F,MAP3K11:ENST00000530153.1:exon7:c.C938T:p.S313F,
11	71169488	G	A	TACGGATGTT,GGATCATTAT	exonic	NADSYN1	stopgain	NADSYN1:ENST00000528509.1:exon3:c.G161A:p.W54X,NADSYN1:ENST00000529120.1:exon3:c.G161A:p.W54X,NADSYN1:ENST00000319023.2:exon3:c.G161A:p.W54X,
11	74875135	C	T	CTGCTGGCCT,CTTCAACGAG	exonic	SLCO2B1	nonsynonymous SNV	SLCO2B1:ENST00000531713.1:exon3:c.C209T:p.S70F,SLCO2B1:ENST00000289575.5:exon3:c.C275T:p.S92F,SLCO2B1:ENST00000428359.2:exon3:c.C209T:p.S70F,SLCO2B1:ENST00000527180.1:exon3:c.C209T:p.S70F,SLCO2B1:ENST00000534186.1:exon3:c.C209T:p.
11	82879773	G	A	GGACAAATGG,GGGAGGAGGC	exonic	PCF11	nonsynonymous SNV	PCF11:ENST00000298281.4:exon8:c.G2396A:p.G799E,PCF11:ENST00000530660.1:exon8:c.G2789A:p.G930E,
11	100998573	T	C	GGCTGCGGGG,TGGCACCGGC	exonic	PGR	nonsynonymous SNV	PGR:ENST00000263463.5:exon1:c.A1229G:p.N410S,PGR:ENST00000325455.5:exon1:c.A1229G:p.N410S,PGR:ENST00000528960.1:exon1:c.A1229G:p.N410S,PGR:ENST00000526300.1:exon1:c.A1229G:p.N410S,PGR:ENST00000534780.1:exon1:c.A1229G:p.N410S,
11	107729481	A	T	CCCGCTGGCG,GTCTGCCTCC	exonic	SLC35F2	nonsynonymous SNV	SLC35F2:ENST00000429869.1:exon4:c.T13A:p.S5T,SLC35F2:ENST00000524991.1:exon1:c.T13A:p.S5T,SLC35F2:ENST00000532513.2:exon1:c.T13A:p.S5T,SLC35F2:ENST00000525071.1:exon4:c.T13A:p.S5T,SLC35F2:ENST00000525815.1:exon1:c.T13A:p.S5T,SLC35F2:
11	108214022	T	G	GAATTTCTTG,TAACAATGAA	exonic	ATM	nonsynonymous SNV	ATM:ENST00000452508.2:exon58:c.T8342G:p.V2781G,ATM:ENST00000278616.4:exon57:c.T8342G:p.V2781G,
11	108594143	G	A	AAGGTGAAGC,GCATAATGTG	exonic	DDX10	nonsynonymous SNV	DDX10:ENST00000322536.3:exon13:c.G1919A:p.R640Q,DDX10:ENST00000526794.1:exon13:c.G1919A:p.R640Q,
11	118772986	G	A	GGGGTGCCCA,GCACTTCATG	exonic	BCL9L	nonsynonymous SNV	BCL9L:ENST00000334801.3:exon6:c.C1466T:p.P489L,
11	120322404	C	T	GCCTCTTTTT,CCAGGAAGGA	exonic	ARHGEF12	nonsynonymous SNV	ARHGEF12:ENST00000532993.1:exon22:c.C1718T:p.S573F,ARHGEF12:ENST00000356641.3:exon21:c.C1970T:p.S657F,ARHGEF12:ENST00000397843.2:exon22:c.C2027T:p.S676F,
11	123848091	C	T	GATCACCTTC,CATCCAGAGT	exonic	OR10S1	nonsynonymous SNV	OR10S1:ENST00000531945.1:exon1:c.G308A:p.G103E,
11	123894648	G	A	CATTCTCAGG,AGAATAAATA	exonic	OR10G9	nonsynonymous SNV	OR10G9:ENST00000375024.1:exon1:c.G929A:p.G310E,
11	124134942	G	A	TGTAGTCACA,TGCTGGGGAA	exonic	OR8G5	nonsynonymous SNV	OR8G5:ENST00000524943.2:exon1:c.G220A:p.V74M,
11	124749208	C	T	AGCCCCAGTC,TGCCCCTAGC	exonic	ROBO3	nonsynonymous SNV	ROBO3:ENST00000538940.1:exon23:c.C3590T:p.P1197L,ROBO3:ENST00000397801.1:exon24:c.C3656T:p.P1219L,
11	128839572	G	A	TCTGCCTCGG,ATGCCTCCTA	exonic	ARHGAP32	nonsynonymous SNV	ARHGAP32:ENST00000392657.3:exon13:c.C4447T:p.P1483S,ARHGAP32:ENST00000310343.9:exon22:c.C5494T:p.P1832S,ARHGAP32:ENST00000527272.1:exon12:c.C4447T:p.P1483S,
12	5916528	T	C	GATCAGAGAG,TAATACCTAC	exonic	ANO2	nonsynonymous SNV	ANO2:ENST00000546188.1:exon8:c.A890G:p.N297S,ANO2:ENST00000327087.8:exon8:c.A887G:p.N296S,ANO2:ENST00000356134.5:exon9:c.A890G:p.N297S,
12	40645297	C	T	AAATAATCTC,TTATGTACCA	exonic	LRRK2	nonsynonymous SNV	LRRK2:ENST00000343742.2:exon10:c.C1132T:p.L378F,LRRK2:ENST00000298910.7:exon10:c.C1132T:p.L378F,
12	55945219	C	T	TTAGAAATTT,CTTCACATCC	exonic	OR6C4	nonsynonymous SNV	OR6C4:ENST00000394256.2:exon1:c.C209T:p.S70F,
12	112229119	C	T	GGCTGGCTTT,CCCCTGGTGT	exonic	ALDH2,RP11	nonsynonymous SNV	ALDH2:ENST00000261733.2:exon7:c.C691T:p.P231S,ALDH2:ENST00000416293.3:exon6:c.C550T:p.P184S,
12	126138278	A	C	CAGAGGGTGA,GGACAAGGGC	exonic	TMEM132B	nonsynonymous SNV	TMEM132B:ENST00000299308.3:exon9:c.A2259C:p.E753D,TMEM132B:ENST00000535886.1:exon5:c.A795C:p.E265D,
13	28622497	T	C	GGGTAGGCTT,AAACCTGACA	exonic	FLT3	nonsynonymous SNV	FLT3:ENST00000380982.4:exon9:c.A1120G:p.K374E,FLT3:ENST00000380987.2:exon9:c.A1120G:p.K374E,FLT3:ENST00000241453.7:exon9:c.A1120G:p.K374E,FLT3:ENST00000537084.1:exon9:c.A1120G:p.K374E,
13	52701527	A	G	CTCTTTTATG,CACAGTGCTT	exonic	NEK5	nonsynonymous SNV	NEK5:ENST00000355568.4:exon3:c.T92C:p.V31A,NEK5:ENST00000465811.3:exon1:c.T92C:p.V31A,
13	103388489	C	T	TCTGCTTTTT,CTGCACCTGA	exonic	CCDC168	nonsynonymous SNV	CCDC168:ENST00000322527.2:exon1:c.G671A:p.G224E,
14	38218158	G	A	GCATCTCATG,AATACTTCCG	exonic	TTC6	nonsynonymous SNV	TTC6:ENST00000553443.1:exon11:c.G2378A:p.G793E,
14	39517932	C	T	AAATTTCTGA,ACTAAATAAA	exonic	SEC23A	nonsynonymous SNV	SEC23A:ENST00000536508.1:exon14:c.G1283A:p.C428Y,SEC23A:ENST00000545328.2:exon14:c.G1574A:p.C525Y,SEC23A:ENST00000307712.6:exon15:c.G1661A:p.C554Y,SEC23A:ENST00000537403.1:exon11:c.G1055A:p.C352Y,
14	62542100	G	A	CCTCCATGTG,AGTCCAGAGG	exonic	SYT16	stopgain	SYT16:ENST00000430451.2:exon3:c.G984A:p.W328X,SYT16:ENST00000555409.1:exon3:c.G984A:p.W328X,SYT16:ENST00000446982.2:exon4:c.G984A:p.W328X,
14	74764760	C	G	GTGAACTGAT,AAAGCTCTTC	exonic	ABCD4	stoploss	ABCD4:ENST00000553486.1:exon3:c.G170C:p.X57S,ABCD4:ENST00000460308.2:exon3:c.G170C:p.X57S,ABCD4:ENST00000470637.1:exon3:c.G170C:p.X57S,ABCD4:ENST00000481935.1:exon3:c.G170C:p.X57S,
14	79175765	G	A	TTTGACTTCC,CACCACAGAG	exonic	NRXN3	nonsynonymous SNV	NRXN3:ENST00000553631.1:exon3:c.G140A:p.R47H,NRXN3:ENST00000557081.1:exon3:c.G140A:p.R47H,NRXN3:ENST00000335750.5:exon4:c.G308A:p.R103H,NRXN3:ENST00000554738.1:exon6:c.G1421A:p.R474H,NRXN3:ENST00000554719.1:exon4:c.G308A:p.R103H,
14	90433745	A	T	TGGCTCGTAA,ACAGTATCCA	exonic	TDP1	nonsynonymous SNV	TDP1:ENST00000553617.1:exon4:c.A341T:p.K114I,TDP1:ENST00000393452.3:exon5:c.A638T:p.K213I,TDP1:ENST00000393454.2:exon4:c.A638T:p.K213I,TDP1:ENST00000335725.4:exon5:c.A638T:p.K213I,TDP1:ENST00000555178.1:exon4:c.A638T:p.K213I,TDP1:ENST0
14	95583961	C	T	TAAGTTACCT,TTCCTGTTTT	exonic	DICER1	nonsynonymous SNV	DICER1:ENST00000343455.3:exon9:c.G1507A:p.E503K,DICER1:ENST00000541352.1:exon8:c.G1507A:p.E503K,DICER1:ENST00000527414.1:exon9:c.G1507A:p.E503K,DICER1:ENST00000526495.1:exon11:c.G1507A:p.E503K,DICER1:ENST00000393063.1:exon10:c.G15
14	106691865	C	T	GGACCCAGTT,ATGCTATAGC	exonic	IGHV3-21	nonsynonymous SNV	IGHV3-21:ENST00000390607.2:exon2:c.G159A:p.M53I,
15	34049784	G	A	TAAGGAGAAA,AAATGGTGGC	exonic	RYR3	nonsynonymous SNV	RYR3:ENST00000389232.4:exon60:c.G8692A:p.E2898K,RYR3:ENST00000415757.3:exon60:c.G8692A:p.E2898K,
15	35045173	C	T	CTGGTGTTCT,TGTGTTCTGC	exonic	GJD2	nonsynonymous SNV	GJD2:ENST00000290374.4:exon2:c.G472A:p.E158K,
15	43773177	C	G	TCTCCCTTCT,TTCCTCCACA	exonic	TP53BP1	nonsynonymous SNV	TP53BP1:ENST00000382044.4:exon5:c.G415C:p.E139Q,TP53BP1:ENST00000382039.3:exon5:c.G415C:p.E139Q,TP53BP1:ENST00000413546.1:exon5:c.G415C:p.E139Q,TP53BP1:ENST00000450115.2:exon5:c.G415C:p.E139Q,TP53BP1:ENST00000263801.3:exon5:c.G4
15	50741678	T	A	AAGCCTTAAA,TAAGGTACAG	exonic	USP8	nonsynonymous SNV	USP8:ENST00000396444.3:exon4:c.T331A:p.L111I,USP8:ENST00000558091.1:exon4:c.T331A:p.L111I,USP8:ENST00000433963.1:exon5:c.T331A:p.L111I,USP8:ENST00000559242.1:exon2:c.T186A:p.N62K,USP8:ENST00000307179.4:exon4:c.T331A:p.L111I,USP8:ENST0
15	63009877	C	T	GCAGCTGGTC,AGAGTTGCAA	exonic	TLN2	stopgain	TLN2:ENST00000561311.1:exon23:c.C2866T:p.Q956X,TLN2:ENST00000306829.6:exon21:c.C2866T:p.Q956X,
15	63970044	C	T	CAGATCATAC,TGATAGTAAT	exonic	HERC1	nonsynonymous SNV	HERC1:ENST00000443617.2:exon37:c.G7070A:p.S2357N,
15	86261282	G	T	GGGAGACTTT,AGATTGAGTC	exonic	AKAP13	stopgain	AKAP13:ENST00000394518.2:exon22:c.G5893T:p.E1965X,AKAP13:ENST00000361243.2:exon22:c.G5905T:p.E1969X,AKAP13:ENST00000394510.2:exon9:c.G628T:p.E210X,
16	2374439	G	A	GTGGTTGAAG,GGTGCTCGAA	exonic	ABCA3	nonsynonymous SNV	ABCA3:ENST00000301732.5:exon6:c.C413T:p.P138L,ABCA3:ENST00000382381.3:exon6:c.C413T:p.P138L,ABCA3:ENST00000567910.1:exon5:c.C413T:p.P138L,
16	3339818	G	A	CAAGTGCCTT,AATGTGGGAA	exonic	ZNF263	nonsynonymous SNV	ZNF263:ENST00000538765.1:exon2:c.G256A:p.E86K,ZNF263:ENST00000219069.5:exon6:c.G1312A:p.E438K,
16	21073963	G	A	CTTTGTCTCG,ACAAGATTTC	exonic	DNAH3	nonsynonymous SNV	DNAH3:ENST00000261383.3:exon25:c.C3560T:p.S1187F,DNAH3:ENST00000415178.1:exon25:c.C3560T:p.S1187F,
16	51172894	G	A	AGATGACAAC,AGTTGGCGGG	exonic	SALL1	nonsynonymous SNV	SALL1:ENST00000440970.1:exon2:c.C2948T:p.S983L,SALL1:ENST00000251020.4:exon2:c.C3239T:p.S1080L,SALL1:ENST00000570206.1:exon2:c.C2948T:p.S983L,
16	69177222	C	T	TTCTGGGACT,AGCCACTGGG	exonic	CIRH1A	nonsynonymous SNV	CIRH1A:ENST00000567460.1:exon5:c.C419T:p.S140L,CIRH1A:ENST00000352319.4:exon6:c.C668T:p.S223L,CIRH1A:ENST00000562237.1:exon6:c.C710T:p.S237L,CIRH1A:ENST00000563094.1:exon6:c.C668T:p.S223L,CIRH1A:ENST00000566227.1:exon6:c.C524T:p.S175
16	70697723	C	T	GTGGGGGTGG,CGACAGAGCA	exonic	MTSS1L	nonsynonymous SNV	MTSS1L:ENST00000338779.6:exon15:c.G2101A:p.A701T,
16	76482703	T	G	CTGGTCAATC,CACCCTGGGC	exonic	CNTNAP4	nonsynonymous SNV	CNTNAP4:ENST00000377504.4:exon7:c.T779G:p.L260R,CNTNAP4:ENST00000307431.8:exon7:c.T779G:p.L260R,CNTNAP4:ENST00000476707.1:exon5:c.T791G:p.L264R,CNTNAP4:ENST00000478060.1:exon6:c.T707G:p.L236R,
16	81097379	A	T	TCCAATAATA,ACTCTTTGGC	exonic	C16orf46	nonsynonymous SNV	C16orf46:ENST00000378611.4:exon2:c.T182A:p.F61Y,C16orf46:ENST00000565253.1:exon3:c.T182A:p.F61Y,C16orf46:ENST00000299578.5:exon3:c.T182A:p.F61Y,

16	88932249	G	A	CCCGGCCGCG,TGCCCTCCTC	exonic	PABPN1L	nonsynonymous SNV	PABPN1L:ENST00000378358.4:exon2:c.C356T:p.T119I,PABPN1L:ENST00000419291.2:exon2:c.C356T:p.T119I,PABPN1L:ENST00000547152.1:exon2:c.C356T:p.T119I,PABPN1L:ENST00000411789.2:exon2:c.C356T:p.T119I,PABPN1L:ENST00000427766.1:exon2:c.C356T:
17	29548909	G	A	TTGATTTGTG,AATCCTGATG	exonic	NF1	stopgain	NF1:ENST00000358273.4:exon15:c.G1683A:p.W561X,NF1:ENST00000456735.2:exon7:c.G681A:p.W227X,NF1:ENST00000356175.3:exon15:c.G1683A:p.W561X,NF1:ENST00000431387.4:exon15:c.G1683A:p.W561X,NF1:ENST00000579081.1:exon15:c.G1785A:p.W595X,
17	30376355	C	G	CAACAAAAAA,AAATTATATT	exonic	LRRC37B	nonsynonymous SNV	LRRC37B:ENST00000543378.2:exon13:c.C2372G:p.T791R,LRRC37B:ENST00000394713.3:exon8:c.C2465G:p.T822R,LRRC37B:ENST00000327564.7:exon10:c.C2699G:p.T900R,LRRC37B:ENST00000584368.1:exon8:c.C2501G:p.T834R,LRRC37B:ENST00000341671.7:exo
17	34893339	C	T	CCAGCCATCT,CTGTTTCCGT	exonic	PIGW	nonsynonymous SNV	PIGW:ENST00000328396.2:exon2:c.C389T:p.S130F,PIGW:ENST00000592983.1:exon2:c.C389T:p.S130F,PIGW:ENST00000429467.2:exon2:c.C389T:p.S130F,
17	39846152	G	C	AGGGATCGCT,ATGATTACGA	exonic	EIF1	nonsynonymous SNV	EIF1:ENST00000469257.1:exon2:c.G154C:p.D52H,EIF1:ENST00000591776.1:exon2:c.G154C:p.D52H,
17	45452118	G	C	CATATTCAAA,AATGGCATAA	exonic	EFCAB13	nonsynonymous SNV	EFCAB13:ENST00000517484.1:exon10:c.G870C:p.K290N,EFCAB13:ENST00000331493.2:exon12:c.G1158C:p.K386N,
17	61958098	C	T	GCCAGTGGGG,CCCAGGATTG	exonic	GH2	nonsynonymous SNV	GH2:ENST00000332800.7:exon4:c.G490A:p.A164T,
18	21487603	C	T	CACAACTCTC,GAGATGGTCT	exonic	LAMA3	stopgain	LAMA3:ENST00000269217.6:exon16:c.C1981T:p.R661X,LAMA3:ENST00000399516.3:exon52:c.C6640T:p.R2214X,LAMA3:ENST00000313654.9:exon53:c.C6808T:p.R2270X,LAMA3:ENST00000587184.1:exon15:c.C1813T:p.R605X,
18	30257282	G	A	ATTACATCAA,GTGGGAGAAA	exonic	KLHL14	nonsynonymous SNV	KLHL14:ENST00000359358.4:exon8:c.C1600T:p.L534F,
18	53018104	C	T	GAATACCTTA,CCATGGCACT	splicing	TCF4(ENST0	.	.
18	53018105	C	T	AATACCTTAC,CATGGCACTA	exonic	TCF4	nonsynonymous SNV	TCF4:ENST00000566286.1:exon6:c.G493A:p.E165K,TCF4:ENST00000568147.1:exon6:c.G427A:p.E143K,TCF4:ENST00000564999.1:exon7:c.G499A:p.E167K,TCF4:ENST00000566777.1:exon3:c.G109A:p.E37K,TCF4:ENST00000568186.1:exon2:c.G109A:p.E37K,TCF4:ENS
18	64211364	G	A	GAAAGTGGTG,AAGCCTCAGT	exonic	CDH19	nonsynonymous SNV	CDH19:ENST00000262150.2:exon7:c.C1058T:p.S353F,CDH19:ENST00000540086.1:exon7:c.C1058T:p.S353F,CDH19:ENST00000579658.1:exon7:c.C1058T:p.S353F,CDH19:ENST00000454642.3:exon6:c.C1025T:p.S342F,
18	66354975	C	T	CCTGTTGATC,ATGATGACAG	exonic	TMX3	stopgain	TMX3:ENST00000299608.2:exon10:c.G665A:p.W222X,
18	71825644	A	T	TTTTCGATAT,TATTTGTAGA	exonic	TIMM21	nonsynonymous SNV	TIMM21:ENST00000169551.6:exon6:c.A676T:p.I226L,
19	2877734	G	C	GAGACCGTAT,AGTGCAAGCA	exonic	ZNF556	nonsynonymous SNV	ZNF556:ENST00000307635.2:exon4:c.G778C:p.E260Q,ZNF556:ENST00000586426.1:exon4:c.G775C:p.E259Q,
19	3740687	G	A	ACCACTCAGC,GAGCCGTGAG	exonic	TJP3	nonsynonymous SNV	TJP3:ENST00000587686.1:exon13:c.G1826A:p.R609Q,TJP3:ENST00000541714.2:exon14:c.G1769A:p.R590Q,TJP3:ENST00000589378.1:exon14:c.G1796A:p.R599Q,TJP3:ENST00000382008.3:exon14:c.G1811A:p.R604Q,TJP3:ENST00000539908.2:exon13:c.G1661A:p.R5
19	5260814	A	C	GGAGCCTCTT,CCTCGAATCG	splicing	PTPRS(ENST	.	.
19	9067062	G	A	TTTCTCTATA,AAGGAGGGAT	exonic	MUC16	nonsynonymous SNV	MUC16:ENST00000397910.4:exon3:c.C20384T:p.S6795F,
19	10108108	C	T	GCCAACCACC,CCTGTTGGGG	exonic	COL5A3	nonsynonymous SNV	COL5A3:ENST00000264828.3:exon11:c.G1202A:p.G401E,
19	10935865	C	T	CGACCTCATG,CAAAGACCAT	exonic	DNM2	nonsynonymous SNV	DNM2:ENST00000408974.4:exon17:c.C2014T:p.P672S,DNM2:ENST00000355667.6:exon18:c.C2026T:p.P676S,DNM2:ENST00000585892.1:exon18:c.C2026T:p.P676S,DNM2:ENST00000359692.6:exon17:c.C2014T:p.P672S,DNM2:ENST00000389253.4:exon18:c.C2026T:p.P
19	10935866	C	T	GACCTCATGC,AAAGACCATC	exonic	DNM2	nonsynonymous SNV	DNM2:ENST00000408974.4:exon17:c.C2015T:p.P672L,DNM2:ENST00000355667.6:exon18:c.C2027T:p.P676L,DNM2:ENST00000585892.1:exon18:c.C2027T:p.P676L,DNM2:ENST00000359692.6:exon17:c.C2015T:p.P672L,DNM2:ENST00000389253.4:exon18:c.C2027T:p.P
19	11303996	C	T	TCTGGGGGAT,GGGGAGGTCC	exonic	KANK2	nonsynonymous SNV	KANK2:ENST00000586659.1:exon4:c.G760A:p.D254N,KANK2:ENST00000589359.1:exon4:c.G760A:p.D254N,KANK2:ENST00000589894.1:exon2:c.G760A:p.D254N,KANK2:ENST00000432929.2:exon4:c.G760A:p.D254N,KANK2:ENST00000355150.5:exon2:c.G760A:p.D254N,
19	13220202	G	A	TACCGCAACT,CAGGAGGCTT	exonic	TRMT1	stopgain	TRMT1:ENST00000221504.8:exon10:c.C1303T:p.Q435X,TRMT1:ENST00000592062.1:exon13:c.C1390T:p.Q464X,TRMT1:ENST00000437766.1:exon11:c.C1390T:p.Q464X,TRMT1:ENST00000357720.4:exon12:c.C1390T:p.Q464X,
19	14938630	C	T	ACCAGCAGTC,ACAGAGGTGA	exonic	OR7A5	nonsynonymous SNV	OR7A5:ENST00000594432.1:exon1:c.G424A:p.G142R,OR7A5:ENST00000322301.3:exon2:c.G424A:p.G142R,
19	17169406	G	A	TGAGAGAGGA,AAGCCTGCGC	exonic	HAUS8	nonsynonymous SNV	HAUS8:ENST00000448593.2:exon8:c.C595T:p.L199F,HAUS8:ENST00000593360.1:exon7:c.C415T:p.L139F,HAUS8:ENST00000253669.5:exon8:c.C598T:p.L200F,
19	17322805	G	A	CTTCGTAACG,TCAGAGTGAA	exonic	MYO9B	nonsynonymous SNV	MYO9B:ENST00000595641.1:exon38:c.G6112A:p.V2038I,MYO9B:ENST00000594824.1:exon40:c.G6160A:p.V2054I,
19	18547642	G	C	CAGCACCTTC,CGCGCCGCAT	exonic	ISYNA1	nonsynonymous SNV	ISYNA1:ENST00000457269.4:exon4:c.C299G:p.A100G,ISYNA1:ENST00000545187.1:exon2:c.C11G:p.A4G,ISYNA1:ENST00000578963.1:exon2:c.C77G:p.A26G,ISYNA1:ENST00000338128.8:exon5:c.C461G:p.A154G,
19	21477362	G	A	ATTTTGCTCT,GGTAGTTGTC	exonic	ZNF708	stopgain	ZNF708:ENST00000601295.1:exon3:c.C214T:p.Q72X,ZNF708:ENST00000356929.3:exon4:c.C406T:p.Q136X,ZNF708:ENST00000602023.1:exon4:c.C178T:p.Q60X,
19	21910476	G	A	CATGCAAAAT,ATTTTTCACA	exonic	ZNF100	nonsynonymous SNV	ZNF100:ENST00000305570.6:exon4:c.C446T:p.S149L,ZNF100:ENST00000358296.6:exon5:c.C638T:p.S213L,
19	37038346	C	A	AAAGCCTTCC,ACATTCCTTA	exonic	ZNF529	nonsynonymous SNV	ZNF529:ENST00000334116.7:exon6:c.G799T:p.G267W,ZNF529:ENST00000590656.1:exon1:c.G85T:p.G29W,ZNF529:ENST00000591340.1:exon5:c.G1114T:p.G372W,
19	42927583	C	G	TTGGCCTTGT,TTTTCGCCAC	exonic	LIPE	nonsynonymous SNV	LIPE:ENST00000599783.1:exon1:c.G106C:p.D36H,
19	44111815	C	T	GTGCAGCTCC,CCAGGTTGTC	exonic	ZNF428	nonsynonymous SNV	ZNF428:ENST00000300811.3:exon3:c.G521A:p.G174E,ZNF428:ENST00000598676.1:exon4:c.G620A:p.G207E,
19	45649265	C	T	TGAGCCGCCC,CGGGGCCTGA	exonic	PPP1R37	nonsynonymous SNV	PPP1R37:ENST00000221462.4:exon11:c.C1951T:p.P651S,PPP1R37:ENST00000421905.1:exon11:c.C1939T:p.P647S,
19	47258913	C	T	CTGGTAGACT,CTTCCTGCAG	exonic	FKRP	nonsynonymous SNV	FKRP:ENST00000602181.1:exon4:c.C206T:p.S69F,FKRP:ENST00000600629.1:exon5:c.C206T:p.S69F,FKRP:ENST00000595868.1:exon5:c.C206T:p.S69F,FKRP:ENST00000593875.1:exon4:c.C206T:p.S69F,FKRP:ENST00000593800.1:exon4:c.C206T:p.S69F,FKRP:ENST0
19	48048405	G	A	GGGCCGGGCG,GGACTCCTCC	exonic	ZNF541	nonsynonymous SNV	ZNF541:ENST00000448976.1:exon3:c.C1381T:p.P461S,ZNF541:ENST00000314121.4:exon3:c.C1381T:p.P461S,ZNF541:ENST00000391901.3:exon3:c.C1381T:p.P461S,
19	48517451	C	T	ATGTGTCTTT,CTTTCACCTA	exonic	ELSPBP1	nonsynonymous SNV	ELSPBP1:ENST00000339841.2:exon3:c.C94T:p.P32S,
19	49442746	G	A	GAGGCTCTGA,GTCTGTTTTG	exonic	DHDH	nonsynonymous SNV	DHDH:ENST00000221403.2:exon4:c.G407A:p.R136K,DHDH:ENST00000522614.1:exon4:c.G407A:p.R136K,
19	49442747	G	A	AGGCTCTGAG,TCTGTTTTGG	exonic	DHDH	nonsynonymous SNV	DHDH:ENST00000520557.1:exon3:c.G208A:p.V70I,
19	49965292	C	T	GACCGCGGCC,GGTGAGACCC	exonic	ALDH16A1	nonsynonymous SNV	ALDH16A1:ENST00000433981.2:exon5:c.C416T:p.P139L,ALDH16A1:ENST00000293350.4:exon7:c.C911T:p.P304L,ALDH16A1:ENST00000540132.1:exon4:c.C422T:p.P141L,
19	54804217	C	G	GATGAGCCCT,GGTGCTGGCA	exonic	LILRA3	nonsynonymous SNV	LILRA3:ENST00000391745.1:exon5:c.G8C:p.R3P,
19	55449523	G	C	GTTGCTGTTT,AGCTGAAGAG	exonic	NLRP7	stopgain	NLRP7:ENST00000592784.1:exon5:c.C2018G:p.S673X,NLRP7:ENST00000586379.1:exon5:c.C2018G:p.S673X,NLRP7:ENST00000340844.2:exon5:c.C2018G:p.S673X,NLRP7:ENST00000448121.2:exon5:c.C1934G:p.S645X,NLRP7:ENST00000590030.1:exon4:c.C2018G:p.S
19	55870791	G	A	CGCAGCTGAG,CCTTCCGGGA	exonic	CTD-2105E1:	nonsynonymous SNV	FAM71E2:ENST00000424985.3:exon9:c.C1445T:p.A482V,
19	56953498	C	T	CTTTTTGAAG,CTCTCCCACA	exonic	ZNF667	nonsynonymous SNV	ZNF667:ENST00000504904.3:exon7:c.G866A:p.G289D,ZNF667:ENST00000342634.3:exon7:c.G1250A:p.G417D,ZNF667:ENST00000292069.6:exon5:c.G866A:p.G289D,
19	56953688	C	T	TCAAGAATTT,CTTTCCATCA	exonic	ZNF667	nonsynonymous SNV	ZNF667:ENST00000504904.3:exon7:c.G676A:p.E226K,ZNF667:ENST00000342634.3:exon7:c.G1060A:p.E354K,ZNF667:ENST00000292069.6:exon5:c.G676A:p.E226K,
19	58301684	G	A	CTTTTCCCTG,AGGAATGGAG	exonic	ZNF586	nonsynonymous SNV	ZNF586:ENST00000598183.1:exon2:c.G79A:p.E27K,ZNF586:ENST00000598885.1:exon2:c.G79A:p.E27K,
19	58928201	G	A	AGTGGAGGAT,AGAGAGCCCA	exonic	ZNF584	nonsynonymous SNV	ZNF584:ENST00000593920.1:exon4:c.G181A:p.E61K,ZNF584:ENST00000306910.4:exon4:c.G316A:p.E106K,ZNF584:ENST00000322834.7:exon5:c.G414A:p.M138I,
20	4162877	C	T	GGAGGGCATC,CTGCCCACGT	exonic	SMOX	nonsynonymous SNV	SMOX:ENST00000305958.4:exon5:c.C751T:p.P251S,SMOX:ENST00000379460.2:exon5:c.C751T:p.P251S,SMOX:ENST00000278795.3:exon5:c.C751T:p.P251S,SMOX:ENST00000457205.1:exon3:c.C322T:p.P108S,SMOX:ENST00000339123.6:exon5:c.C751T:p.P251S,
20	30898646	C	T	TGCCCTCCTT,GAGAATTCCA	exonic	KIF3B	stopgain	KIF3B:ENST00000418717.2:exon1:c.C130T:p.R44X,KIF3B:ENST00000375712.3:exon2:c.C1066T:p.R356X,
20	33173287	G	A	ACGTTGATCT,AAACACACAT	exonic	PIGU	stopgain	PIGU:ENST00000452740.2:exon9:c.C880T:p.Q294X,PIGU:ENST00000217446.3:exon9:c.C880T:p.Q294X,PIGU:ENST00000374820.2:exon8:c.C820T:p.Q274X,
20	45131155	G	A	GTCTTCACAC,AAAAGTTTTC	exonic	ZNF334	nonsynonymous SNV	ZNF334:ENST00000593880.1:exon4:c.C892T:p.R298C,ZNF334:ENST00000457685.2:exon6:c.C709T:p.R237C,ZNF334:ENST00000347606.4:exon5:c.C823T:p.R275C,
20	50049254	G	A	GATCAGAGTG,GGTCATATTC	exonic	NFATC2	nonsynonymous SNV	NFATC2:ENST00000396009.3:exon9:c.C2072T:p.P691L,NFATC2:ENST00000610033.1:exon9:c.C1415T:p.P472L,NFATC2:ENST00000609943.1:exon9:c.C2012T:p.P671L,NFATC2:ENST00000371564.3:exon9:c.C2072T:p.P691L,NFATC2:ENST00000414705.1:exon9:c.C2012
20	50049255	G	A	ATCAGAGTGG,GTCATATTCA	exonic	NFATC2	nonsynonymous SNV	NFATC2:ENST00000396009.3:exon9:c.C2071T:p.P691S,NFATC2:ENST00000610033.1:exon9:c.C1414T:p.P472S,NFATC2:ENST00000609943.1:exon9:c.C2011T:p.P671S,NFATC2:ENST00000371564.3:exon9:c.C2071T:p.P691S,NFATC2:ENST00000414705.1:exon9:c.C201
20	56728670	C	T	TTTAACAACC,CTTTTGAGGA	exonic	C20orf85	nonsynonymous SNV	C20orf85:ENST00000371168.3:exon2:c.C139T:p.P47S,
20	56728671	C	T	TTAACAACCC,TTTTGAGGAG	exonic	C20orf85	nonsynonymous SNV	C20orf85:ENST00000371168.3:exon2:c.C140T:p.P47L,
20	57485133	G	A	TACTCCTGAG,ATGGTGTGTA	exonic	GNAS	nonsynonymous SNV	GNAS:ENST00000265620.7:exon10:c.G922A:p.D308N,GNAS:ENST00000306090.10:exon11:c.G925A:p.D309N,GNAS:ENST00000371095.3:exon10:c.G925A:p.D309N,GNAS:ENST00000371100.4:exon11:c.G2896A:p.D966N,GNAS:ENST00000371085.3:exon11:c.G967A:p.D
20	61938848	C	T	CCAGCCGGCC,CCAGTTCCGC	exonic	COL20A1	nonsynonymous SNV	COL20A1:ENST00000422202.1:exon5:c.C524T:p.P175L,COL20A1:ENST00000358894.6:exon6:c.C503T:p.P168L,COL20A1:ENST00000435874.1:exon6:c.C524T:p.P175L,COL20A1:ENST00000326996.6:exon6:c.C503T:p.P168L,
21	17242448	A	T	ATTGGGCTAG,AAATTTTCAA	exonic	USP25	nonsynonymous SNV	USP25:ENST00000400183.2:exon23:c.A2867T:p.E956V,USP25:ENST00000285679.6:exon21:c.A2657T:p.E886V,USP25:ENST00000285681.2:exon22:c.A2753T:p.E918V,USP25:ENST00000351097.5:exon8:c.A842T:p.E281V,
21	41457622	G	A	TTCACCGTGC,AATAATGAAG	exonic	DSCAM	nonsynonymous SNV	DSCAM:ENST00000400454.1:exon23:c.C4039T:p.R1347C,DSCAM:ENST00000404019.2:exon19:c.C3295T:p.R1099C,
21	45390675	C	T	AGTCAAGTGC,TCCGGGGGAC	exonic	AGPAT3	nonsynonymous SNV	AGPAT3:ENST00000398058.1:exon7:c.C652T:p.L218F,AGPAT3:ENST00000291572.8:exon6:c.C652T:p.L218F,AGPAT3:ENST00000457068.1:exon5:c.C652T:p.L218F,AGPAT3:ENST00000546158.1:exon5:c.C652T:p.L218F,AGPAT3:ENST00000398063.2:exon5:c.C652T:p.L2
21	48078833	G	A	GCGCTCTGAA,TAAGTGTCCA	splicing	PRMT2(ENST	.	.
22	19709850	G	A	TTATCAGGAT,AAGGATGAGG	exonic	SEPT5	nonsynonymous SNV	SEPT5:ENST00000455784.2:exon11:c.G1041A:p.M347I,SEPT5:ENST00000412544.1:exon10:c.G900A:p.M300I,SEPT5:ENST00000383045.3:exon10:c.G1068A:p.M356I,SEPT5:ENST00000431044.1:exon10:c.G900A:p.M300I,SEPT5:ENST00000455843.1:exon10:c.G1068A:p.
22	25243708	C	T	GAACTTCCCG,CGGCTGAGGA	exonic	SGSM1	nonsynonymous SNV	SGSM1:ENST00000400358.4:exon4:c.C247T:p.P83S,SGSM1:ENST00000400359.4:exon4:c.C247T:p.P83S,
X	3248115	G	A	GCAGGTCCAC,GATTTCCCTG	exonic	MXRA5	nonsynonymous SNV	MXRA5:ENST00000217939.6:exon4:c.C653T:p.P218L,
X	3248116	G	A	CAGGTCCACG,ATTTCCCTGC	exonic	MXRA5	nonsynonymous SNV	MXRA5:ENST00000217939.6:exon4:c.C652T:p.P218S,
X	10535521	G	C	GCGCAGGGCA,TAGAAGAGGG	exonic	MID1	nonsynonymous SNV	MID1:ENST00000380779.1:exon2:c.C67G:p.L23V,MID1:ENST00000413894.1:exon2:c.C67G:p.L23V,MID1:ENST00000380787.1:exon2:c.C67G:p.L23V,MID1:ENST00000453318.2:exon2:c.C67G:p.L23V,MID1:ENST00000380785.1:exon3:c.C67G:p.L23V,MID1:ENST0000380
X	11783864	G	A	TTCATTCTCG,GTGCCCCAGG	exonic	MSL3	nonsynonymous SNV	MSL3:ENST00000337339.2:exon9:c.G1187A:p.G396E,
X	15841198	G	A	AAGAAATAGG,ACCGCAGCAG	exonic	ZRSR2	nonsynonymous SNV	ZRSR2:ENST00000307771.7:exon11:c.G1282A:p.D428N,
X	19533352	C	T	AGGGCCCCGG,CGGAGCATTC	exonic	MAP3K15	nonsynonymous SNV	MAP3K15:ENST00000338883.4:exon1:c.G28A:p.A10T,
X	29973554	G	A	CATTACACAT,AGCAGGCTTT	exonic	IL1RAPL1	nonsynonymous SNV	IL1RAPL1:ENST00000378993.1:exon11:c.G1708A:p.E570K,IL1RAPL1:ENST00000302196.4:exon10:c.G1708A:p.E570K,
X	32429969	G	A	TAAGTGTAAG,ATTTTTCAGT	exonic	DMD	nonsynonymous SNV	DMD:ENST00000357033.4:exon30:c.C4133T:p.S1378F,DMD:ENST00000378677.2:exon30:c.C4121T:p.S1374F,
X	34148225	G	A	AGGTTCATCA,GCTTTTTAAG	exonic	FAM47A	nonsynonymous SNV	FAM47A:ENST00000346193.3:exon1:c.C2171T:p.P724L,
X	34148226	G	A	GGTTCATCAG,CTTTTTAAGT	exonic	FAM47A	nonsynonymous SNV	FAM47A:ENST00000346193.3:exon1:c.C2170T:p.P724S,
X	40572175	C	T	CCTCCTGTTT,CTTATCCTCA	exonic	MED14	nonsynonymous SNV	MED14:ENST00000324817.1:exon6:c.G772A:p.E258K,
X	41084016	C	T	CTTCCCAATC,TTTTGGTGAT	exonic	USP9X	nonsynonymous SNV	USP9X:ENST00000378308.2:exon40:c.C6773T:p.P2258L,USP9X:ENST00000324545.8:exon40:c.C6773T:p.P2258L,
X	46433934	G	A	TCTTACCACG,CCGCCCTCTT	exonic	CHST7	nonsynonymous SNV	CHST7:ENST00000276055.3:exon1:c.G568A:p.A190T,
X	52826333	G	A	TTTGCTGTTG,AGTCACAGGG	exonic	SPANXN5	nonsynonymous SNV	SPANXN5:ENST00000375511.3:exon1:c.C56T:p.S19F,
X	54955915	G	A	TATCTGCTTC,GTGGCTCTCC	exonic	TRO	nonsynonymous SNV	TRO:ENST00000375041.2:exon11:c.G1567A:p.G523S,TRO:ENST00000173898.7:exon12:c.G2758A:p.G920S,TRO:ENST00000420798.2:exon10:c.G1351A:p.G451S,
X	55247874	C	T	CAAGAGTCTT,CCAGCCAGTT	exonic	PAGE5	nonsynonymous SNV	PAGE5:ENST00000374955.3:exon2:c.C59T:p.S20F,PAGE5:ENST00000289619.5:exon2:c.C119T:p.S40F,PAGE5:ENST00000374952.1:exon2:c.C59T:p.S20F,
X	65819631	C	T	GCACTGGTCT,CTTGCTGGGT	exonic	EDA2R	nonsynonymous SNV	EDA2R:ENST00000396050.1:exon5:c.G589A:p.E197K,EDA2R:ENST00000451436.2:exon4:c.G217A:p.E73K,EDA2R:ENST00000456230.2:exon6:c.G589A:p.E197K,EDA2R:ENST00000253392.5:exon6:c.G652A:p.E218K,EDA2R:ENST00000374719.3:exon6:c.G589A:p.E197K,
X	70514087	C	T	GAAACCCGAA,CCTAGCGGAG	exonic	NONO	nonsynonymous SNV	NONO:ENST00000276079.8:exon5:c.C359T:p.T120I,NONO:ENST00000413858.1:exon5:c.C359T:p.T120I,NONO:ENST00000373856.3:exon6:c.C359T:p.T120I,NONO:ENST00000420903.1:exon6:c.C308T:p.T103I,NONO:ENST00000535149.1:exon3:c.C92T:p.T31I,NONO:ENS
X	99917295	C	T	CCGGGGCTTT,GATTGATTGG	exonic	SRPX2	stopgain	SRPX2:ENST00000373004.3:exon4:c.C286T:p.R96X,
X	102318190	C	T	GCTCGTTTCT,TTTGGACTCC	exonic	BEX1	nonsynonymous SNV	BEX1:ENST00000372728.3:exon3:c.G13A:p.E5K,
X	105153494	C	T	AGGATCACCT,AGGCACAGGC	exonic	NRK	stopgain	NRK:ENST00000243300.9:exon13:c.C1861T:p.Q621X,NRK:ENST00000428173.2:exon13:c.C1864T:p.Q622X,
X	114426823	G	A	CATGGCCGGA,CCACCGCTAC	exonic	RBMXL3	nonsynonymous SNV	RBMXL3:ENST00000424776.3:exon1:c.G2819A:p.S940N,
X	117695382	C	T	GACCCAACTT,CTGACGGTTC	exonic	DOCK11	nonsynonymous SNV	DOCK11:ENST00000276204.6:exon7:c.C595T:p.P199S,DOCK11:ENST00000276202.7:exon7:c.C595T:p.P199S,
X	118603697	C	T	GTCCGTATTC,CAAGGAGCAG	exonic	SLC25A5	nonsynonymous SNV	SLC25A5:ENST00000317881.8:exon2:c.C185T:p.P62L,
X	119512579	C	T	TACCCAGAGT,GGCTTCTTTT	exonic	ATP1B4	nonsynonymous SNV	ATP1B4:ENST00000539306.1:exon6:c.C734T:p.S245L,ATP1B4:ENST00000361319.3:exon7:c.C851T:p.S284L,ATP1B4:ENST00000218008.3:exon7:c.C863T:p.S288L,
X	123785886	A	C	AGTTAAGATC,GGTTCCGAGG	exonic	TENM1	nonsynonymous SNV	TENM1:ENST00000422452.2:exon8:c.T1457G:p.L486R,TENM1:ENST00000371130.3:exon8:c.T1457G:p.L486R,
X	129148169	C	T	GGGGTTTCCT,CACTCTTACG	exonic	BCORL1	nonsynonymous SNV	BCORL1:ENST00000303743.5:exon4:c.C1421T:p.S474F,BCORL1:ENST00000540052.1:exon3:c.C1421T:p.S474F,BCORL1:ENST00000218147.7:exon4:c.C1421T:p.S474F,BCORL1:ENST00000359304.2:exon4:c.C1421T:p.S474F,BCORL1:ENST00000456822.1:exon1:c.C22
X	134715478	T	G	CCTAATTCAG,ACCTTGAGAA	exonic	DDX26B	nonsynonymous SNV	DDX26B:ENST00000370752.4:exon17:c.T2503G:p.Y835D,
X	135428387	G	A	ACTGTGACAA,AAAAGAAGCA	exonic	GPR112	nonsynonymous SNV	GPR112:ENST00000394143.1:exon6:c.G2522A:p.R841K,GPR112:ENST00000370652.1:exon4:c.G2522A:p.R841K,GPR112:ENST00000287534.4:exon4:c.G2333A:p.R778K,GPR112:ENST00000394141.1:exon3:c.G1907A:p.R636K,GPR112:ENST00000412101.1:exon3:c.G19
X	149639476	C	T	AACACCAAGC,CTTGTCCCAT	exonic	MAMLD1	nonsynonymous SNV	MAMLD1:ENST00000455522.2:exon1:c.C74T:p.P25L,MAMLD1:ENST00000262858.5:exon3:c.C1631T:p.P544L,MAMLD1:ENST00000426613.2:exon2:c.C1556T:p.P519L,MAMLD1:ENST00000370401.2:exon4:c.C1631T:p.P544L,MAMLD1:ENST00000432680.2:exon3:c.C1556
X	153642468	G	A	GAGAATGAGG,GAAAGGTGTT	exonic	TAZ	nonsynonymous SNV	TAZ:ENST00000426834.1:exon5:c.G455A:p.G152E,TAZ:ENST00000351413.4:exon5:c.G401A:p.G134E,TAZ:ENST00000475699.1:exon5:c.G401A:p.G134E,TAZ:ENST00000299328.5:exon5:c.G401A:p.G134E,

estin

testin

96V;tissue=oesophagu

61568E;tissue=large_intestine&CSMD1_ENST00000537824;basechange=c.5516G>A;AAchange=p.G1839E;tissue=large_intest

SG00000156509;basechange=c.491C>T;AAchange=p.S164F;tissue=sk

stin

ur

gu

r

riur

673*;tissue=endometrium&NLRP7;basechange=c.1934C>G;AAchange=p.S645*;tissue=endometri

intestin

.E570*;tissue=lung&IL1RAPL1;basechange=c.1708G>T;AAchange=p.E570*;tissue=lu

BEX1_ENST00000372728;basechange=c.13G>T;AAchange=p.E5*;tissue=large_intest

C000-W91YB6

Chromosom	Position (hg	Reference al	Mutant allele	Sequence context	ANNOVAR fu	Gene	Mutation type	ANNOVAR output (alternative transcripts)
1	152882979	C	T	GCTGGAGCTC,CACAGCAGCA	exonic	IVL	nonsynonymous SNV	IVL:ENST00000368764.3:exon2:c.C706T:p.P236S,IVL:ENST00000392667.2:exon3:c.C268T:p.P90S,
1	168510311	G	A	TCTCACCCAC,TGGCTTGTGG	exonic	XCL2	nonsynonymous SNV	XCL2:ENST00000367819.2:exon3:c.C224T:p.T75M,
2	167141144	C	T	GTTACTGCTG,GTCGCTCCTG	exonic	SCN9A	nonsynonymous SNV	SCN9A:ENST00000409672.1:exon12:c.G1793A:p.R598H,SCN9A:ENST00000454569.1:exon9:c.G1388A:p.R463H,SCN9A:ENST00000409435.1:exon11:c.G1793A:p.R598H,SCN9A:ENST00000375387.4:exon12:c.G1796A:p.R599H,SCN9A:ENST000004
3	119198789	G	C	ATATATCTTT,ACAATGTGAC	exonic	POGLUT1	stoploss	POGLUT1:ENST00000486607.1:exon5:c.G491C:p.X164S,
3	169684623	A	G	GGCGGAACGC,GGAGACACAA	exonic	SEC62	nonsynonymous SNV	SEC62:ENST00000337002.4:exon1:c.A13G:p.R5G,SEC62:ENST00000469515.2:exon1:c.A13G:p.R5G,SEC62:ENST00000480708.1:exon1:c.A13G:p.R5G,SEC62:ENST00000487736.1:exon1:c.A13G:p.R5G,SEC62:ENST00000460513.1:exon1:c.A13G:p.
6	87726137	G	A	ATTAGATGCC,AGAGCATACT	exonic	HTR1E	nonsynonymous SNV	HTR1E:ENST00000305344.5:exon2:c.G1085A:p.R362Q,
7	122221277	C	T	CCAGTGCTTT,TGTGAAGAGT	exonic	CADPS2	nonsynonymous SNV	CADPS2:ENST00000412584.2:exon7:c.G1291A:p.E431K,CADPS2:ENST00000334010.7:exon7:c.G1291A:p.E431K,CADPS2:ENST00000313070.7:exon7:c.G1291A:p.E431K,CADPS2:ENST00000449022.2:exon7:c.G1291A:p.E431K,
7	137266651	G	C	GGCTGAAGTA,TTATTGAAAA	exonic	DGKI	nonsynonymous SNV	DGKI:ENST00000453654.2:exon15:c.C687G:p.N229K,DGKI:ENST00000446122.1:exon15:c.C1587G:p.N529K,DGKI:ENST00000424189.2:exon15:c.C1587G:p.N529K,DGKI:ENST00000288490.5:exon15:c.C1587G:p.N529K,
7	150217958	C	T	AAGTTTTATT,TTCCTAATTT	exonic	GIMAP7	nonsynonymous SNV	GIMAP7:ENST00000313543.4:exon2:c.C896T:p.S299F,
7	150217961	C	T	TTTTATTCTT,CTAATTTACT	exonic	GIMAP7	nonsynonymous SNV	GIMAP7:ENST00000313543.4:exon2:c.C899T:p.S300F,
8	110527432	C	G	TTCCACACTT,AACGTTTGGA	exonic	PKHD1L1	nonsynonymous SNV	PKHD1L1:ENST00000378402.5:exon72:c.C11587G:p.Q3863E,PKHD1L1:ENST00000526472.1:exon18:c.C2371G:p.Q791E,
11	64757197	G	C	TGCACGTGCA,GGTCCGGCTC	exonic	BATF2	nonsynonymous SNV	BATF2:ENST00000534177.1:exon3:c.C226G:p.L76V,BATF2:ENST00000527716.1:exon2:c.C157G:p.L53V,BATF2:ENST00000301887.4:exon3:c.C229G:p.L77V,
12	11138642	G	A	TGAGTCGAAT,CAAGATATAT	exonic	TAS2R50	nonsynonymous SNV	TAS2R50:ENST00000506868.1:exon1:c.C818T:p.A273V,
12	50045835	C	T	CATGCCCTCA,TCAGCTCTGC	exonic	FMNL3	nonsynonymous SNV	FMNL3:ENST00000352151.5:exon13:c.G1331A:p.S444N,FMNL3:ENST00000335154.5:exon14:c.G1484A:p.S495N,FMNL3:ENST00000550488.1:exon14:c.G1484A:p.S495N,FMNL3:ENST00000293590.5:exon14:c.G1484A:p.S495N,
12	85441073	G	A	GAAAAATGTA,ACAGTCTTTT	exonic	LRRIQ1	nonsynonymous SNV	LRRIQ1:ENST00000393217.2:exon6:c.G503A:p.R168K,
14	101347482	C	T	GCGGTTCTGA,GCAGGGCAGG	exonic	RTL1	nonsynonymous SNV	RTL1:ENST00000534062.1:exon1:c.G3644A:p.R1215H,
15	40253993	G	A	AGGCGAGAAC,TCAGTATTCT	exonic	EIF2AK4	nonsynonymous SNV	EIF2AK4:ENST00000382727.2:exon7:c.G752A:p.R251H,EIF2AK4:ENST00000263791.5:exon7:c.G752A:p.R251H,EIF2AK4:ENST00000559624.1:exon7:c.G752A:p.R251H,
16	4812577	T	C	TACTCACCTC,CTCTGGCCAC	exonic	ZNF500	nonsynonymous SNV	ZNF500:ENST00000545009.1:exon3:c.A595G:p.R199G,ZNF500:ENST00000219478.6:exon3:c.A595G:p.R199G,
17	39254126	C	T	CAGCTGGACA,ACAGCAGCTG	exonic	KRTAP4-8	nonsynonymous SNV	KRTAP4-8:ENST00000333822.4:exon1:c.G211A:p.V71M,
20	18440840	G	C	CCATCAGGCA,AGTAATAGGT	exonic	DZANK1	nonsynonymous SNV	DZANK1:ENST00000608192.1:exon3:c.C220G:p.L74V,DZANK1:ENST00000262547.5:exon3:c.C220G:p.L74V,DZANK1:ENST00000329494.5:exon3:c.C220G:p.L74V,DZANK1:ENST00000609267.1:exon3:c.C220G:p.L74V,DZANK1:ENST00000377630.5

e,large_intestir

29N;tissue=endometrium&DGKI;basechange=c.1587C>T;AAchange=p.N529N;tissue=endometr

triur

C000-X88E7Z

Chromosom	Position (hg1	Reference all	Mutant allele	Sequence context	ANNOVAR function	Gene	Mutation type	ANNOVAR output (alternative transcripts)
1	883537	C	G	AGCACCAGCT,CGGGAAGCCG	exonic	NOC2L	nonsynonymous SNV	NOC2L:ENST00000327044.6:exon14:c.G1633C:p.E545Q,
1	1152969	C	A	TTGAGCACCT,CTCGGGCTCC	exonic	SDF4	stopgain	SDF4:ENST00000360001.6:exon7:c.G1012T:p.E338X,
1	1853779	C	A	GTCTCCTTCA,ATCCCCCCTT	exonic	C1orf222	nonsynonymous SNV	C1orf222:ENST00000493964.1:exon21:c.G2551T:p.V851L,
1	2450628	G	A	CATGGTCACG,GGTGGCCCCG	exonic	PANK4	nonsynonymous SNV	PANK4:ENST00000435556.3:exon7:c.C872T:p.P291L,[illegible]
1	3313158	G	A	GGCCTGGACG,TAAGACCCCT	splicing	PRDM16(ENST00000511072.1:exon5:c.679+1G>A		.
1	6206393	C	T	TTGCCGTCTT,ATCCCCAGAG	exonic	CHD5	nonsynonymous SNV	CHD5:ENST00000262450.3:exon11:c.G1681A:p.E561K,[illegible]
1	6273236	G	A	GGAGCGGCTG,AGCCCAGGTG	exonic	RNF207	nonsynonymous SNV	RNF207:ENST00000377939.4:exon16:c.G1645A:p.E549K,
1	6534177	C	T	GACTTGGAGT,CTTCATGCCC	exonic	PLEKHG5	nonsynonymous SNV	PLEKHG5:ENST00000377725.1:exon7:c.G487A:p.D163N,[illegible]
1	10436620	G	A	CTTTCCCGCA,ATGCCCGAGC	exonic	KIF1B	nonsynonymous SNV	KIF1B:ENST00000263934.6:exon47:c.G5288A:p.R1763K,[illegible]
1	10477082	G	C	ATCTCTGAAG,ATGAGAGAAT	exonic	PGD	nonsynonymous SNV	PGD:ENST00000270776.8:exon9:c.G883C:p.D295H,PGD:ENST00000483936.1:exon5:c.G448C:p.D150H,[illegible]
1	10689968	G	A	CGCCGGGGCG,GGATGGGCAG	exonic	PEX14	nonsynonymous SNV	PEX14:ENST00000356607.4:exon9:c.G1058A:p.G353E,PEX14:ENST00000538836.1:exon8:c.G866A:p.G289E,
1	11106667	C	T	TTGGAGTAGT,GGAGCGGAAG	exonic	MASP2	nonsynonymous SNV	MASP2:ENST00000400898.3:exon3:c.G358A:p.D120N,MASP2:ENST00000400897.3:exon3:c.G358A:p.D120N,
1	11562778	T	A	TCTACTGGTA,GTGGATGAGG	exonic	PTCHD2	stopgain	PTCHD2:ENST00000294484.6:exon3:c.T1140A:p.Y380X,PTCHD2:ENST00000389575.3:exon3:c.T1140A:p.Y380X,
1	12907323	C	T	GCCTCTTTTT,ATCATCCTTG	exonic	HNRNPCL1	nonsynonymous SNV	HNRNPCL1:ENST00000317869.6:exon2:c.G820A:p.E274K,
1	16344424	T	C	GAAACTTCTC,CCGCTCGGAA	exonic	HSPB7	nonsynonymous SNV	HSPB7:ENST00000311890.9:exon1:c.A35G:p.E12G,HSPB7:ENST00000406363.2:exon1:c.A35G:p.E12G,[illegible]
1	17264899	C	T	CTCCAGGAAT,CCTGCGGCTA	exonic	CROCC	nonsynonymous SNV	CROCC:ENST00000375541.5:exon11:c.C1295T:p.S432F,
1	18152362	C	G	GTGGTTGATT,TGGCTATGGC	exonic	ACTL8	nonsynonymous SNV	ACTL8:ENST00000375406.1:exon3:c.C449G:p.S150C,
1	18691831	G	A	TCTGCACCGT,ACCTGGATGA	exonic	IGSF21	nonsynonymous SNV	IGSF21:ENST00000251296.1:exon6:c.G655A:p.D219N,
1	21546614	G	A	TGTGCGGACG,AGCACCAGAC	exonic	ECE1	nonsynonymous SNV	ECE1:ENST00000357071.4:exon17:c.C2111T:p.S704F,ECE1:ENST00000264205.6:exon18:c.C2138T:p.S713F,[illegible]
1	21938533	G	A	CCAAGCTTCT,ATAAATGACG	exonic	RAP1GAP	stopgain	RAP1GAP:ENST00000359708.4:exon11:c.C790T:p.Q264X,RAP1GAP:ENST00000542643.2:exon12:c.C598T:p.Q200X,[illegible]
1	23720439	G	A	CCGCAGGCCG,GGTTCCTGGG	exonic	TCEA3	nonsynonymous SNV	TCEA3:ENST00000450454.2:exon8:c.C752T:p.P251L,
1	26873710	G	C	CACCCGGCCT,ACAGTGGGCA	exonic	RPS6KA1	nonsynonymous SNV	RPS6KA1:ENST00000374168.2:exon4:c.G256C:p.D86H,RPS6KA1:ENST00000374163.1:exon3:c.G283C:p.D95H,[illegible]
1	27190168	G	A	AGGAGGCCAT,GACATCAGCA	exonic	SFN	nonsynonymous SNV	SFN:ENST00000339276.4:exon1:c.G465A:p.M155I,
1	27736327	G	A	GGGGGCCCCG,AGGAGGAGGA	exonic	WASF2	nonsynonymous SNV	WASF2:ENST00000430629.2:exon8:c.C1198T:p.P400S,
1	27741385	C	G	TTCATTTTCT,CCACTCTTCC	exonic	WASF2	nonsynonymous SNV	WASF2:ENST00000536657.1:exon6:c.G610C:p.E204Q,WASF2:ENST00000430629.2:exon6:c.G610C:p.E204Q,
1	27877635	G	A	GAGTGCCTGG,GGTCAAGCAG	exonic	AHDC1	nonsynonymous SNV	AHDC1:ENST00000374011.2:exon6:c.C992T:p.P331L,AHDC1:ENST00000247087.5:exon5:c.C992T:p.P331L,
1	31381352	G	A	AGCAGCCCGC,GGCCCCGGGC	exonic	SDC3	nonsynonymous SNV	SDC3:ENST00000339394.6:exon1:c.C82T:p.R28C,
1	34102008	C	T	CCATCAGGCC,CAGGATGCAG	exonic	CSMD2	nonsynonymous SNV	CSMD2:ENST00000373380.1:exon9:c.G1540A:p.G514R,CSMD2:ENST00000241312.4:exon30:c.G4801A:p.G1601R,[illegible]
1	35260447	C	G	TCTTCATCAT,TTCATGTTGG	exonic	GJA4	nonsynonymous SNV	GJA4:ENST00000450137.1:exon2:c.C633G:p.I211M,GJA4:ENST00000342280.4:exon2:c.C633G:p.I211M,
1	36605377	G	A	ACATTTGACC,AGCCAAGAAA	exonic	TRAPPC3	nonsynonymous SNV	TRAPPC3:ENST00000373162.1:exon3:c.C46T:p.R16W,TRAPPC3:ENST00000373166.3:exon3:c.C184T:p.R62W,[illegible]
1	36932092	G	A	GGGCTGGCCT,GAACCAGAGG	exonic	CSF3R	stopgain	CSF3R:ENST00000418048.2:exon15:c.C2377T:p.Q793X,CSF3R:ENST00000373103.1:exon17:c.C2458T:p.Q820X,[illegible]
1	40887650	G	A	CCTTTCAGAT,ACCCAGCAGA	exonic	SMAP2	nonsynonymous SNV	SMAP2:ENST00000372718.3:exon10:c.G1167A:p.M389I,SMAP2:ENST00000539317.1:exon10:c.G927A:p.M309I,[illegible]
1	43162468	G	C	GTGGGGAAAA,AACGAGGGAT	exonic	YBX1	nonsynonymous SNV	YBX1:ENST00000332220.6:exon4:c.G420C:p.K140N,YBX1:ENST00000321358.7:exon5:c.G510C:p.K170N,
1	43851807	C	G	GCCTGAAGGT,TCCAATTAGA	exonic	MED8	nonsynonymous SNV	MED8:ENST00000372455.4:exon6:c.G317C:p.R106T,MED8:ENST00000372457.4:exon6:c.G584C:p.R195T,[illegible]
1	43900930	G	C	TGGTGGCCGA,AGTGAAGAAG	exonic	SZT2	nonsynonymous SNV	SZT2:ENST00000562955.1:exon41:c.G5793C:p.E1931D,SZT2:ENST00000372442.1:exon27:c.G3267C:p.E1089D,
1	44085088	G	C	ACATGGTGCA,ACGGAGGACC	exonic	PTPRF	nonsynonymous SNV	PTPRF:ENST00000372407.4:exon16:c.G1992C:p.Q664H,PTPRF:ENST00000422171.2:exon19:c.G2853C:p.Q951H,[illegible]
1	44433353	G	C	GCTGACTACT,AGGGGTGCCC	exonic	IPO13	stoploss	IPO13:ENST00000372343.3:exon20:c.G2891C:p.X964S,IPO13:ENST00000372339.3:exon5:c.G545C:p.X182S,
1	44687268	G	C	ATGAAGCCTC,ATAGGCGAGT	exonic	ERI3	nonsynonymous SNV	ERI3:ENST00000537474.1:exon7:c.C445G:p.R149G,ERI3:ENST00000372257.2:exon9:c.C976G:p.R326G,[illegible]
1	47581230	C	G	TATTTGGGCT,TTCACCACAA	exonic	CYP4Z1	nonsynonymous SNV	CYP4Z1:ENST00000334194.3:exon10:c.C1231G:p.L411V,
1	52499019	C	T	CCGTCCTCCT,AGCGCGTGGC	exonic	KTI12	nonsynonymous SNV	KTI12:ENST00000371614.1:exon1:c.G415A:p.E139K,
1	54371951	G	A	ATCCAGGAGG,CAGGATCCTC	exonic	DIO1	nonsynonymous SNV	DIO1:ENST00000388876.3:exon2:c.G521A:p.G174D,DIO1:ENST00000524406.1:exon4:c.G278A:p.G93D,
1	55145649	C	T	CTGCCCGTCT,CCTCCTGGCT	exonic	MROH7,MROH7-TTC4	nonsynonymous SNV	MROH7:ENST00000421030.2:exon13:c.C2312T:p.S771F,MROH7:ENST00000339553.5:exon13:c.C2312T:p.S771F,[illegible]
1	57476883	G	A	TCTGTGGTAG,ATCACTGGCA	exonic	DAB1	nonsynonymous SNV	DAB1:ENST00000414851.2:exon11:c.C1453T:p.P485S,DAB1:ENST00000439789.2:exon10:c.C1249T:p.P417S,[illegible]
1	60506665	C	G	ATACACACCT,AGGTTGGTCA	exonic	C1orf87	nonsynonymous SNV	C1orf87:ENST00000371201.3:exon4:c.G481C:p.E161Q,
1	62586864	G	C	GTAGTCAGCA,AGTGCACACA	exonic	INADL	nonsynonymous SNV	INADL:ENST00000484937.1:exon26:c.G3274C:p.E1092Q,INADL:ENST00000371158.2:exon38:c.G4902C:p.Q1634H,
1	64606094	G	C	TCCCATGGCA,ATCCTATAAA	exonic	ROR1	nonsynonymous SNV	ROR1:ENST00000371079.1:exon6:c.G913C:p.D305H,ROR1:ENST00000371080.1:exon6:c.G913C:p.D305H,
1	67359063	G	A	TAAAGAGGTC,GGGAGTAACA	exonic	WDR78	stopgain	WDR78:ENST00000371022.3:exon3:c.C379T:p.R127X,WDR78:ENST00000371023.3:exon3:c.C379T:p.R127X,[illegible]
1	75037922	C	T	GGCGTTGCTT,AAATATGGGA	exonic	C1orf173	nonsynonymous SNV	C1orf173:ENST00000326665.5:exon14:c.G3472A:p.E1158K,
1	75608862	G	A	GACTGGGTCC,GAGAGCCAAG	exonic	LHX8	nonsynonymous SNV	LHX8:ENST00000294638.5:exon6:c.G449A:p.R150Q,LHX8:ENST00000356261.3:exon5:c.G419A:p.R140Q,
1	86890039	G	A	ACAGCTTCAA,ACAATGGGTA	exonic	CLCA2	nonsynonymous SNV	CLCA2:ENST00000370565.4:exon1:c.G109A:p.D37N,
1	89847315	G	A	GCCTTGTCTG,AGAATGCAGT	exonic	GBP6	nonsynonymous SNV	GBP6:ENST00000370456.4:exon7:c.G934A:p.E312K,GBP6:ENST00000535065.1:exon5:c.G544A:p.E182K,
1	91841190	C	T	GCAGGGAAAT,CAAGGACCAC	exonic	HFM1	nonsynonymous SNV	HFM1:ENST00000370424.3:exon8:c.G527A:p.G176E,HFM1:ENST00000370425.3:exon12:c.G1490A:p.G497E,
1	100346653	C	G	GTATGATGCT,TTCCAAGTAC	exonic	AGL	nonsynonymous SNV	AGL:ENST00000361915.3:exon15:c.C1921G:p.L641V,AGL:ENST00000370161.2:exon14:c.C1873G:p.L625V,[illegible]
1	100983813	C	T	ACAGTCCCTT,AGTCTGAATA	exonic	CDC14A	stopgain	CDC14A:ENST00000336454.3:exon16:c.C1762T:p.Q588X,
1	103356041	C	T	ACCTTTGAGA,CAGGTAAGCC	exonic	COL11A1	nonsynonymous SNV	COL11A1:ENST00000370096.3:exon58:c.G4322A:p.G1441D,COL11A1:ENST00000512756.1:exon56:c.G3974A:p.G1325D,[illegible]
1	103356042	C	T	CCTTTGAGAC,AGGTAAGCCA	exonic	COL11A1	nonsynonymous SNV	COL11A1:ENST00000370096.3:exon58:c.G4321A:p.G1441S,COL11A1:ENST00000512756.1:exon56:c.G3973A:p.G1325S,[illegible]
1	109466721	G	A	GCTAGTTTCA,TAATTTGCCA	exonic	GPSM2	nonsynonymous SNV	GPSM2:ENST00000406462.2:exon15:c.G1700A:p.S567N,GPSM2:ENST00000264126.3:exon14:c.G1700A:p.S567N,
1	109825144	C	G	TGGTACCTGT,AGATGGTGAC	exonic	PSRC1	nonsynonymous SNV	PSRC1:ENST00000369907.3:exon3:c.G73C:p.D25H,PSRC1:ENST00000369904.3:exon3:c.G73C:p.D25H,[illegible]
1	113162492	C	T	GATCGTGTGT,GGATGAGGAG	exonic	CAPZA1	nonsynonymous SNV	CAPZA1:ENST00000263168.3:exon1:c.C26T:p.S9L,
1	116226683	G	C	GGCGAGTAAA,AAGCGGAAAG	exonic	VANGL1	nonsynonymous SNV	VANGL1:ENST00000310260.3:exon6:c.G1065C:p.K355N,VANGL1:ENST00000355485.2:exon6:c.G1065C:p.K355N,[illegible]
1	116227966	G	A	TCTCCAGGCT,AGGAGCAGCA	exonic	VANGL1	nonsynonymous SNV	VANGL1:ENST00000310260.3:exon7:c.G1132A:p.E378K,VANGL1:ENST00000355485.2:exon7:c.G1132A:p.E378K,[illegible]
1	117663380	C	G	CTTTGCAGGT,TGACACCTCT	exonic	TRIM45	nonsynonymous SNV	TRIM45:ENST00000256649.4:exon1:c.G444C:p.Q148H,TRIM45:ENST00000369464.3:exon1:c.G444C:p.Q148H,[illegible]
1	147380890	G	A	TCAGCTCCTA,AAGAAGAGAA	exonic	GJA8	nonsynonymous SNV	GJA8:ENST00000240986.4:exon2:c.G808A:p.E270K,GJA8:ENST00000369235.1:exon1:c.G808A:p.E270K,
1	151205108	G	C	CAGCGACGAT,AGTTCATTAT	exonic	PIP5K1A	nonsynonymous SNV	PIP5K1A:ENST00000349792.5:exon6:c.G529C:p.E177Q,PIP5K1A:ENST00000409426.1:exon6:c.G532C:p.E178Q,[illegible]
1	151259260	C	T	CCTGTTTGCT,ATTTTGGCCC	exonic	ZNF687	nonsynonymous SNV	ZNF687:ENST00000449313.1:exon2:c.C493T:p.H165Y,ZNF687:ENST00000324048.5:exon3:c.C493T:p.H165Y,[illegible]
1	151787725	G	A	GAGCCCAGGG,CAGCTGCCCG	exonic	RORC	nonsynonymous SNV	RORC:ENST00000356728.6:exon4:c.C412T:p.P138S,RORC:ENST00000318247.6:exon5:c.C475T:p.P159S,[illegible]
1	152080826	C	T	TGTTCGTCTT,GCGGAATTTT	exonic	TCHH	nonsynonymous SNV	TCHH:ENST00000368804.1:exon2:c.G4867A:p.E1623K,
1	152081876	C	G	TCTTGCTGTT,ACCCAGCAGG	exonic	TCHH	nonsynonymous SNV	TCHH:ENST00000368804.1:exon2:c.G3817C:p.E1273Q,
1	152082044	C	T	CGCTGATCCT,ATCCCGGTAT	exonic	TCHH	nonsynonymous SNV	TCHH:ENST00000368804.1:exon2:c.G3649A:p.E1217K,
1	152082282	C	G	GATATTGCCT,TCCAGCTCCT	exonic	TCHH	nonsynonymous SNV	TCHH:ENST00000368804.1:exon2:c.G3411C:p.E1137D,
1	152082488	C	G	CTTCTCGTCT,CCGTTCCTCT	exonic	TCHH	nonsynonymous SNV	TCHH:ENST00000368804.1:exon2:c.G3205C:p.E1069Q,
1	152082533	C	G	TGCAGCTCCT,TTCCTCCCGA	exonic	TCHH	nonsynonymous SNV	TCHH:ENST00000368804.1:exon2:c.G3160C:p.E1054Q,
1	152082713	C	G	TGCAACTCCT,TTCCTCGCGG	exonic	TCHH	nonsynonymous SNV	TCHH:ENST00000368804.1:exon2:c.G2980C:p.E994Q,
1	152082920	C	G	CTCTCCTGTT,TTGGCGCCTT	exonic	TCHH	nonsynonymous SNV	TCHH:ENST00000368804.1:exon2:c.G2773C:p.E925Q,
1	152324884	G	A	AGTGGTCCTG,ACCCTGTCTG	exonic	FLG2	nonsynonymous SNV	FLG2:ENST00000388718.5:exon3:c.C5378T:p.S1793F,
1	154909149	C	T	GGGGCGCCTC,CAGCGGGGCC	exonic	PMVK	nonsynonymous SNV	PMVK:ENST00000368467.3:exon1:c.G13A:p.G5R,
1	155261553	C	T	TCACCACTTT,AATGCCAAAT	exonic	PKLR	nonsynonymous SNV	PKLR:ENST00000342741.4:exon10:c.G1612A:p.E538K,PKLR:ENST00000392414.3:exon10:c.G1519A:p.E507K,
1	155701777	C	G	GACTGGGTCT,TCTTTAAGCC	exonic	DAP3	nonsynonymous SNV	DAP3:ENST00000535183.1:exon10:c.C823G:p.L275V,DAP3:ENST00000475056.1:exon3:c.C262G:p.L88V,[illegible]
1	155911976	G	A	TTCTGGGCCC,AATAGCCACC	exonic	RXFP4	nonsynonymous SNV	RXFP4:ENST00000368318.3:exon1:c.G476A:p.R159Q,
1	156444993	G	C	GTGAGCGAAT,AGTAGACTGG	exonic	MEF2D	nonsynonymous SNV	MEF2D:ENST00000464356.2:exon7:c.C889G:p.H297D,MEF2D:ENST00000348159.4:exon9:c.C913G:p.H305D,[illegible]
1	156510601	C	T	CTAACTACCT,TTCCTGGAGC	exonic	IQGAP3	nonsynonymous SNV	IQGAP3:ENST00000361170.2:exon23:c.G2638A:p.E880K,IQGAP3:ENST00000491900.1:exon22:c.G2509A:p.E837K,
1	158299249	G	T	CACATCCAGG,TTGCTCGGAG	exonic	CD1B	nonsynonymous SNV	CD1B:ENST00000368168.3:exon4:c.C797A:p.T266N,
1	158746740	T	A	TGATGCTGTT,TTATCTTCAG	exonic	OR6N2	nonsynonymous SNV	OR6N2:ENST00000339258.1:exon1:c.A686T:p.K229I,
1	159504996	C	T	AGGTCTTTTT,TATTGAACTT	exonic	OR10J5	nonsynonymous SNV	OR10J5:ENST00000334857.2:exon1:c.G802A:p.E268K,
1	162124202	G	C	CAGTACGTAG,AAGCCTGGAC	exonic	NOS1AP	nonsynonymous SNV	NOS1AP:ENST00000361897.5:exon2:c.G113C:p.G38A,NOS1AP:ENST00000530878.1:exon2:c.G113C:p.G38A,[illegible]
1	166039968	C	T	TTTTACTCTC,CTTCCCTCAA	exonic	FAM78B	nonsynonymous SNV	FAM78B:ENST00000354422.3:exon2:c.G296A:p.G99E,FAM78B:ENST00000456900.1:exon2:c.G296A:p.G99E,[illegible]
1	169588426	C	T	TCTCTGGAAT,TCTGGTACAA	exonic	SELP	nonsynonymous SNV	SELP:ENST00000367791.2:exon2:c.G35A:p.R12K,SELP:ENST00000458599.2:exon2:c.G35A:p.R12K,[illegible]
1	169761771	C	G	AATCCTTCAT,AATTATTTTG	exonic	METTL18	nonsynonymous SNV	METTL18:ENST00000310392.4:exon2:c.G1066C:p.D356H,METTL18:ENST00000303469.2:exon2:c.G1066C:p.D356H,
1	171501600	C	T	TCAGAAATTT,TGCAGCAGTA	exonic	PRRC2C	nonsynonymous SNV	PRRC2C:ENST00000426496.2:exon12:c.C1367T:p.S456F,PRRC2C:ENST00000338920.4:exon12:c.C1367T:p.S456F,[illegible]
1	171510486	G	C	AGAGAGGAAC,GCCTGAAAAC	exonic	PRRC2C	nonsynonymous SNV	PRRC2C:ENST00000426496.2:exon16:c.G3875C:p.R1292P,PRRC2C:ENST00000338920.4:exon16:c.G3875C:p.R1292P,[illegible]
1	173569296	G	C	CACGAATCCT,ATAGAGAAAG	exonic	SLC9C2	stopgain	SLC9C2:ENST00000367714.3:exon3:c.C188G:p.S63X,
1	178820358	C	T	GTGCTTGCTT,TGTAATGGCC	exonic	ANGPTL1	nonsynonymous SNV	ANGPTL1:ENST00000234816.2:exon6:c.G1382A:p.R461K,ANGPTL1:ENST00000367629.1:exon5:c.G1382A:p.R461K,
1	179623477	G	A	AGAACCAAAC,ATCTGAAGGA	exonic	TDRD5	nonsynonymous SNV	TDRD5:ENST00000444136.1:exon14:c.G2302A:p.D768N,TDRD5:ENST00000417329.1:exon5:c.G670A:p.D224N,
1	181019301	C	G	CTCACACCAT,AAGCAGGCAT	exonic	MR1	nonsynonymous SNV	MR1:ENST00000367580.5:exon3:c.C483G:p.I161M,MR1:ENST00000282990.6:exon3:c.C483G:p.I161M,[illegible]
1	182554720	G	C	CTGCTTTGCA,ACAAGAGACT	exonic	RNASEL	nonsynonymous SNV	RNASEL:ENST00000444138.1:exon1:c.C1222G:p.L408V,RNASEL:ENST00000539397.1:exon2:c.C1222G:p.L408V,[illegible]
1	184680942	G	A	GGGTCATTAG,AAAGAGGATC	exonic	EDEM3	nonsynonymous SNV	EDEM3:ENST00000318130.8:exon15:c.C1606T:p.P536S,EDEM3:ENST00000367512.3:exon15:c.C1477T:p.P493S,
1	196295932	C	T	TTATATAATC,ATTTCCAGCT	exonic	KCNT2	nonsynonymous SNV	KCNT2:ENST00000367433.5:exon19:c.G2191A:p.G731R,KCNT2:ENST00000294725.9:exon19:c.G2191A:p.G731R,[illegible]
1	196392191	T	A	GATGATGTCC,ATCCACTTCA	exonic	KCNT2	nonsynonymous SNV	KCNT2:ENST00000367433.5:exon12:c.A1174T:p.R392W,KCNT2:ENST00000294725.9:exon12:c.A1174T:p.R392W,[illegible]
1	196695612	C	T	CAAGTACAAT,ATGTGGTCCA	exonic	CFH	nonsynonymous SNV	CFH:ENST00000367429.4:exon13:c.C1886T:p.S629L,
1	197113131	G	A	AATATAGCTT,GTGTTTCAGA	exonic	ASPM	stopgain	ASPM:ENST00000294732.7:exon2:c.C397T:p.Q133X,ASPM:ENST00000367409.4:exon2:c.C397T:p.Q133X,
1	198711037	C	T	GACTCACATT,AGTTCACCAG	exonic	PTPRC	stopgain	PTPRC:ENST00000348564.6:exon21:c.C1960T:p.Q654X,PTPRC:ENST00000352140.3:exon23:c.C2293T:p.Q765X,[illegible]
1	200376355	G	C	TTCTCAATCT,ATACGTTGTT	exonic	ZNF281	nonsynonymous SNV	ZNF281:ENST00000367352.3:exon3:c.C2371G:p.Q791E,ZNF281:ENST00000294740.3:exon2:c.C2479G:p.Q827E,[illegible]
1	200376478	G	C	GTCAATTTCT,AGATGAGGAC	exonic	ZNF281	nonsynonymous SNV	ZNF281:ENST00000367352.3:exon3:c.C2248G:p.Q750E,ZNF281:ENST00000294740.3:exon2:c.C2356G:p.Q786E,[illegible]
1	200377473	C	T	TTCATCTATT,CAGTGCCAAT	exonic	ZNF281	nonsynonymous SNV	ZNF281:ENST00000367352.3:exon3:c.G1253A:p.G418E,ZNF281:ENST00000294740.3:exon2:c.G1361A:p.G454E,[illegible]
1	200842212	G	A	TCAGCGCCCT,GGACTACTCG	exonic	GPR25	stopgain	GPR25:ENST00000304244.2:exon1:c.G47A:p.W16X,
1	201169392	G	A	AGAAAAAGAT,GACCTTGAGC	exonic	IGFN1	nonsynonymous SNV	IGFN1:ENST00000451870.2:exon8:c.G486A:p.M162I,IGFN1:ENST00000335211.4:exon8:c.G486A:p.M162I,[illegible]
1	202464743	C	T	AAGAGGCTGT,CATCCGAGAG	exonic	PPP1R12B	nonsynonymous SNV	PPP1R12B:ENST00000367270.4:exon5:c.C59T:p.S20F,PPP1R12B:ENST00000336894.4:exon17:c.C2381T:p.S794F,[illegible]
1	203154477	C	T	GTACTGGGAC,AGCTGGTGTA	exonic	CHI3L1	stopgain	CHI3L1:ENST00000255409.3:exon3:c.G92A:p.W31X,
1	204438702	C	T	AGCTTGAGGT,GGTCCGCCTT	exonic	PIK3C2B	nonsynonymous SNV	PIK3C2B:ENST00000367187.3:exon3:c.G229A:p.D77N,PIK3C2B:ENST00000424712.2:exon3:c.G229A:p.D77N,[illegible]
1	207087280	T	C	CTTGATGAAG,TGGTGGTGGA	exonic	FAIM3	nonsynonymous SNV	FAIM3:ENST00000529560.1:exon3:c.A197G:p.N66S,FAIM3:ENST00000463473.2:exon2:c.A197G:p.N66S,[illegible]
1	207110499	G	A	CTGACCATCC,AATGGGCTCC	exonic	PIGR	nonsynonymous SNV	PIGR:ENST00000356495.4:exon4:c.C986T:p.S329L,
1	207644199	G	A	GTGCTGGTGG,AGAAGAATCC	exonic	CR2	nonsynonymous SNV	CR2:ENST00000367059.3:exon7:c.G1340A:p.G447E,CR2:ENST00000367058.3:exon7:c.G1340A:p.G447E,[illegible]
1	210577997	C	G	TGGGCCCATC,TCAGCTTCTC	exonic	HHAT	nonsynonymous SNV	HHAT:ENST00000426968.1:exon3:c.C274G:p.L92V,HHAT:ENST00000261458.3:exon6:c.C658G:p.L220V,[illegible]
1	212860321	C	T	CTCTCATATT,CTGGGGGAGA	exonic	BATF3	nonsynonymous SNV	BATF3:ENST00000243440.1:exon3:c.G196A:p.E66K,
1	216246451	G	A	GTAAAAGGCT,GAGACCACCC	exonic	USH2A	stopgain	USH2A:ENST00000307340.3:exon28:c.C5764T:p.Q1922X,USH2A:ENST00000366943.2:exon28:c.C5764T:p.Q1922X,
1	223717539	G	A	GCTGTTGAGG,TGAAACCTGA	exonic	CAPN8	nonsynonymous SNV	CAPN8:ENST00000430824.2:exon5:c.C278T:p.T93I,CAPN8:ENST00000366872.5:exon18:c.C1853T:p.T618I,
1	231076271	G	C	GTCATTCACT,CTTCATTAAT	exonic	TTC13	nonsynonymous SNV	TTC13:ENST00000366661.4:exon7:c.C704G:p.A235G,TTC13:ENST00000414259.1:exon5:c.C545G:p.A182G,[illegible]
1	231114438	C	A	GAGTAGTGCT,GGTGGCCAGG	exonic	TTC13	stopgain	TTC13:ENST00000366661.4:exon1:c.G139T:p.E47X,TTC13:ENST00000414259.1:exon1:c.G139T:p.E47X,[illegible]
1	233314921	C	T	CTCCACGGTT,CTAAAGAAAA	exonic	PCNXL2	nonsynonymous SNV	PCNXL2:ENST00000258229.9:exon16:c.G3067A:p.E1023K,PCNXL2:ENST00000488780.2:exon6:c.G466A:p.E156K,
1	235840790	C	G	CTGTTTAAAT,CCATATGATG	exonic	LYST	nonsynonymous SNV	LYST:ENST00000389793.2:exon49:c.G10930C:p.D3644H,LYST:ENST00000389794.3:exon49:c.G10930C:p.D3644H,
1	236413206	C	T	GTTAATTGCT,CCAGTTTATT	exonic	ERO1LB	nonsynonymous SNV	ERO1LB:ENST00000354619.5:exon5:c.G410A:p.G137E,ERO1LB:ENST00000327333.8:exon5:c.G410A:p.G137E,[illegible]
1	240370738	G	A	CCTGGGCATG,TGCCTCCCCC	exonic	FMN2	nonsynonymous SNV	FMN2:ENST00000319653.9:exon5:c.G2626A:p.V876M,
1	241901757	G	A	TTTACTCTAT,ATATATGATG	exonic	WDR64	nonsynonymous SNV	WDR64:ENST00000437684.2:exon10:c.G1257A:p.M419I,WDR64:ENST00000468967.2:exon10:c.G1257A:p.M419I,[illegible]
1	244755018	C	T	CATGATTTTT,ATACGTGATT	exonic	C1orf101	nonsynonymous SNV	C1orf101:ENST00000366533.4:exon15:c.C2174T:p.S725L,C1orf101:ENST00000366531.3:exon12:c.C1721T:p.S574L,[illegible]
1	247607988	G	A	CACTGCTGCT,GGATCTTTCC	exonic	NLRP3	stopgain	NLRP3:ENST00000391828.3:exon10:c.G2876A:p.W959X,NLRP3:ENST00000366496.2:exon7:c.G2705A:p.W902X,[illegible]
1	248059061	C	T	CTCCACACCC,CATGTACTTC	exonic	OR2W3	nonsynonymous SNV	OR2W3:ENST00000360358.3:exon1:c.C173T:p.P58L,OR2W3:ENST00000537741.1:exon3:c.C173T:p.P58L,
1	248436923	G	A	GTCCATGAGG,AAAGTTGGCT	exonic	OR2T33	nonsynonymous SNV	OR2T33:ENST00000318021.2:exon1:c.C194T:p.S65F,
2	1457561	C	G	GGCTGGAACC,CGGCTTCTTG	exonic	TPO	nonsynonymous SNV	TPO:ENST00000382198.1:exon5:c.C578G:p.P193R,TPO:ENST00000337415.3:exon6:c.C578G:p.P193R,[illegible]
2	10729394	C	T	ACTTGGTTTT,CTTCCGGCTC	exonic	NOL10	nonsynonymous SNV	NOL10:ENST00000538384.1:exon18:c.G1541A:p.G514E,NOL10:ENST00000381685.5:exon19:c.G1619A:p.G540E,[illegible]
2	10917778	C	T	AAGGTAACCC,GCTAGGTAAC	exonic	ATP6V1C2	nonsynonymous SNV	ATP6V1C2:ENST00000272238.4:exon11:c.C893T:p.P298L,
2	24092590	G	A	TTCCTCATCA,AAGAAGTTGT	exonic	ATAD2B	nonsynonymous SNV	ATAD2B:ENST00000439915.1:exon10:c.C1061T:p.S354F,ATAD2B:ENST00000238789.5:exon9:c.C1019T:p.S340F,
2	27361179	C	T	AGCACCTGGT,CTTGTTTTGA	exonic	C2orf53	nonsynonymous SNV	C2orf53:ENST00000432962.1:exon3:c.G19A:p.D7N,C2orf53:ENST00000335524.3:exon3:c.G19A:p.D7N,
2	32667191	C	T	TGTTAATACT,TTTGCAGAAA	exonic	BIRC6	nonsynonymous SNV	BIRC6:ENST00000421745.2:exon18:c.C4003T:p.L1335F,
2	37483825	C	T	AATTTCTCTC,ATGGATTTGG	exonic	PRKD3	stopgain	PRKD3:ENST00000379066.1:exon17:c.G2393A:p.W798X,PRKD3:ENST00000234179.2:exon16:c.G2393A:p.W798X,
2	39262607	C	T	CAAAATATCT,GAGCATACGA	exonic	SOS1	nonsynonymous SNV	SOS1:ENST00000402219.2:exon7:c.G899A:p.R300Q,SOS1:ENST00000395038.2:exon7:c.G899A:p.R300Q,[illegible]
2	39405889	C	G	GAAAGGCAGA,TTAGGTGCCT	exonic	CDKL4	nonsynonymous SNV	CDKL4:ENST00000395035.3:exon9:c.G939C:p.K313N,
2	44934537	G	T	TTTTTTTTCA,GTTGCTATTT	splicing	CAMKMT(ENST00000378494.3:exon6:c.493-1G>T		.
2	47797409	G	A	GCAGCAGCAG,AGGGGCGCGG	exonic	KCNK12	nonsynonymous SNV	KCNK12:ENST00000327876.4:exon1:c.C62T:p.S21F,
2	56098157	G	A	AGAATGTAGG,ATCTTGACAA	exonic	EFEMP1	nonsynonymous SNV	EFEMP1:ENST00000394555.2:exon9:c.C1102T:p.P368S,EFEMP1:ENST00000355426.3:exon10:c.C1102T:p.P368S,[illegible]
2	61190012	C	T	AACTGCCATT,TAGTGAATAC	exonic	PUS10	nonsynonymous SNV	PUS10:ENST00000316752.6:exon9:c.G737A:p.R246K,PUS10:ENST00000407787.1:exon9:c.G737A:p.R246K,
2	65296637	C	T	AAGGCCCAGT,CTATGGGAGA	exonic	CEP68	nonsynonymous SNV	CEP68:ENST00000546106.1:exon2:c.C59T:p.S20F,CEP68:ENST00000377990.2:exon2:c.C59T:p.S20F,[illegible]
2	69040510	T	C	GTACGAAGGG,TCCTGCTCTG	exonic	ARHGAP25	nonsynonymous SNV	ARHGAP25:ENST00000409030.3:exon5:c.T742C:p.F248L,ARHGAP25:ENST00000463483.1:exon7:c.T688C:p.F230L,[illegible]
2	70387817	C	T	TCTATACGAC,GTAGGTTTCT	exonic	C2orf42	nonsynonymous SNV	C2orf42:ENST00000420306.1:exon7:c.G1456A:p.G486S,C2orf42:ENST00000264434.2:exon9:c.G1456A:p.G486S,
2	71709015	G	A	CCAGGGATTT,AATGGGACCT	exonic	DYSF	nonsynonymous SNV	DYSF:ENST00000394120.2:exon3:c.G154A:p.E52K,DYSF:ENST00000410020.3:exon3:c.G154A:p.E52K,[illegible]
2	72725668	G	A	TGATTTACAG,GAAACCATAC	exonic	EXOC6B	nonsynonymous SNV	EXOC6B:ENST00000272427.6:exon13:c.C1252T:p.P418S,EXOC6B:ENST00000410104.1:exon13:c.C1252T:p.P418S,
2	85014290	G	C	CATTTTAGGA,AGAAAGAAGT	exonic	DNAH6	nonsynonymous SNV	DNAH6:ENST00000237449.6:exon67:c.G11103C:p.E3701D,DNAH6:ENST00000389394.3:exon68:c.G11103C:p.E3701D,
2	89417262	C	T	ACCTGGGAAC,AGAGCAGCAG	exonic	IGKV1-17	stopgain	IGKV1-17:ENST00000490686.1:exon1:c.G47A:p.W16X,
2	95537511	G	A	CGACCAGTCG,AGCGGCAGCG	exonic	TEKT4	nonsynonymous SNV	TEKT4:ENST00000295201.4:exon1:c.G187A:p.E63K,TEKT4:ENST00000427593.2:exon1:c.G187A:p.E63K,
2	100623152	G	A	GGCAGGCTCC,GCTTGCTGGC	exonic	AFF3	nonsynonymous SNV	AFF3:ENST00000409236.2:exon5:c.C815T:p.P272L,AFF3:ENST00000409579.1:exon7:c.C890T:p.P297L,[illegible]
2	100623153	G	A	GCAGGCTCCG,CTTGCTGGCA	exonic	AFF3	nonsynonymous SNV	AFF3:ENST00000409236.2:exon5:c.C814T:p.P272S,AFF3:ENST00000409579.1:exon7:c.C889T:p.P297S,[illegible]
2	101652543	G	A	ACGAGCTTCC,AATCTTCTCT	exonic	TBC1D8	nonsynonymous SNV	TBC1D8:ENST00000376840.4:exon9:c.C1495T:p.R499W,TBC1D8:ENST00000409318.1:exon9:c.C1540T:p.R514W,
2	102955317	G	A	ATGGGGCCTG,AAAATGAGGC	exonic	IL1RL1	nonsynonymous SNV	IL1RL1:ENST00000427077.1:exon3:c.G82A:p.E28K,IL1RL1:ENST00000409584.1:exon3:c.G82A:p.E28K,[illegible]
2	111721246	G	T	GTGTGACACA,ATGTGTTTGC	exonic	ACOXL	nonsynonymous SNV	ACOXL:ENST00000443586.1:exon1:c.G46T:p.D16Y,ACOXL:ENST00000389811.4:exon13:c.G1105T:p.D369Y,
2	113067739	G	T	GTTCAGCAAG,TAAGTTTGAA	splicing	ZC3H6(ENST00000409871.1:exon4:c.613+1G>T,E		.
2	118577261	C	T	AAAGGGAAAT,TGAAGAAGAA	exonic	DDX18	nonsynonymous SNV	DDX18:ENST00000263239.2:exon3:c.C407T:p.S136F,
2	128046960	C	T	TCTTCTTCTT,ATCCTTGTCC	exonic	ERCC3	nonsynonymous SNV	ERCC3:ENST00000493187.2:exon6:c.G583A:p.E195K,ERCC3:ENST00000285398.2:exon6:c.G775A:p.E259K,
2	131521788	G	A	CAGGGGCCCT,ACATGCTGGA	exonic	AMER3	nonsynonymous SNV	AMER3:ENST00000321420.4:exon2:c.G2143A:p.D715N,AMER3:ENST00000423981.1:exon2:c.G2143A:p.D715N,
2	133540387	C	T	GGGAGACTCT,CAACATGGGA	exonic	NCKAP5	nonsynonymous SNV	NCKAP5:ENST00000317721.6:exon12:c.G3997A:p.E1333K,NCKAP5:ENST00000409261.1:exon14:c.G3997A:p.E1333K,
2	135925276	C	T	ACTGAATCTT,TGATGAGGTA	exonic	RAB3GAP1	nonsynonymous SNV	RAB3GAP1:ENST00000442034.1:exon24:c.C2723T:p.S908F,
2	136566094	C	T	AGCCCCACTC,GTTTTCGGTG	exonic	LCT	nonsynonymous SNV	LCT:ENST00000452974.1:exon2:c.G2119A:p.G707R,LCT:ENST00000264162.2:exon8:c.G3823A:p.G1275R,
2	141680541	C	T	TACCTGTACT,CAACAGGAAA	exonic	LRP1B	stopgain	LRP1B:ENST00000434794.1:exon6:c.G747A:p.W249X,LRP1B:ENST00000389484.3:exon21:c.G3312A:p.W1104X,
2	141751699	G	A	GCTTCTCCAG,ATTAACTAGA	exonic	LRP1B	nonsynonymous SNV	LRP1B:ENST00000389484.3:exon16:c.C2509T:p.P837S,
2	145154147	C	G	TGTGCTCCAT,AAGCAATTCT	exonic	ZEB2	nonsynonymous SNV	ZEB2:ENST00000539609.3:exon8:c.G2827C:p.D943H,ZEB2:ENST00000303660.4:exon9:c.G2899C:p.D967H,[illegible]
2	160206242	G	A	AAAGCAAATG,ATTTAAGCCA	exonic	BAZ2B	nonsynonymous SNV	BAZ2B:ENST00000343439.5:exon27:c.C4540T:p.P1514S,BAZ2B:ENST00000392783.2:exon28:c.C4840T:p.P1614S,[illegible]
2	162760564	G	A	CCATACTTCT,GAGTGACTTC	exonic	SLC4A10	stopgain	SLC4A10:ENST00000272716.5:exon12:c.G1403A:p.W468X,SLC4A10:ENST00000421911.1:exon13:c.G1493A:p.W498X,[illegible]
2	165584560	G	C	TCCTGCGATT,ATAATCTTTC	exonic	COBLL1	nonsynonymous SNV	COBLL1:ENST00000409184.3:exon5:c.C694G:p.Q232E,COBLL1:ENST00000456693.1:exon4:c.C505G:p.Q169E,[illegible]
2	167328878	G	A	ACCGAGGAAG,AAAATGATCC	exonic	SCN7A	nonsynonymous SNV	SCN7A:ENST00000441411.1:exon5:c.C521T:p.S174F,SCN7A:ENST00000409855.1:exon5:c.C521T:p.S174F,[illegible]
2	170063446	G	A	CCATTGATAC,AATCCTTGCA	exonic	LRP2	nonsynonymous SNV	LRP2:ENST00000263816.3:exon39:c.C6784T:p.R2262C,
2	173916485	G	C	TTAGAGCCTC,TCGACCATAG	exonic	RAPGEF4	nonsynonymous SNV	RAPGEF4:ENST00000540783.1:exon28:c.G2567C:p.R856P,RAPGEF4:ENST00000264111.6:exon31:c.G3023C:p.R1008P,[illegible]
2	178576600	C	T	TCTTGAAACC,AGCAGTCTGG	exonic	PDE11A	nonsynonymous SNV	PDE11A:ENST00000286063.6:exon13:c.G2050A:p.G684R,PDE11A:ENST00000389683.3:exon10:c.G718A:p.G240R,[illegible]
2	179499912	C	T	CTTAGAAGTT,TCCTTTCAGT	exonic	TTN	nonsynonymous SNV	TTN:ENST00000591111.1:exon178:c.G37081A:p.E12361K,TTN:ENST00000342992.6:exon177:c.G34300A:p.E11434K,[illegible]
2	179543528	C	G	ACTTTCTCCT,TGGCACAGGT	exonic	TTN	nonsynonymous SNV	TTN:ENST00000591111.1:exon141:c.G32821C:p.E10941Q,TTN:ENST00000342992.6:exon140:c.G30040C:p.E10014Q,[illegible]
2	179569689	A	G	TATTTCCTCA,TTTCCTGAGA	exonic	TTN	nonsynonymous SNV	TTN:ENST00000591111.1:exon102:c.T28658C:p.I9553T,TTN:ENST00000342992.6:exon101:c.T25877C:p.I8626T,[illegible]
2	179596608	G	A	GATCTCAAAG,GAGGAGTGCC	exonic	TTN	nonsynonymous SNV	TTN:ENST00000591111.1:exon56:c.C16043T:p.P5348L,TTN:ENST00000342992.6:exon55:c.C13262T:p.P4421L,[illegible]
2	179596609	G	A	ATCTCAAAGG,AGGAGTGCCT	exonic	TTN	nonsynonymous SNV	TTN:ENST00000591111.1:exon56:c.C16042T:p.P5348S,TTN:ENST00000342992.6:exon55:c.C13261T:p.P4421S,[illegible]
2	179610805	C	T	ACAGACATTT,GAATTTTAAG	exonic	TTN	nonsynonymous SNV	TTN:ENST00000360870.5:exon46:c.G16322A:p.R5441Q,
2	179611564	G	A	TTTGGTCAGA,GTTCAATAAA	exonic	TTN	nonsynonymous SNV	TTN:ENST00000360870.5:exon46:c.C15563T:p.P5188L,
2	179612276	A	T	TGGTTGTAGT,TTCATACACA	exonic	TTN	nonsynonymous SNV	TTN:ENST00000360870.5:exon46:c.T14851A:p.Y4951N,
2	179659878	G	A	AGGCACTTCA,GACCTGTGGC	exonic	TTN	nonsynonymous SNV	TTN:ENST00000591111.1:exon7:c.C1016T:p.P339L,TTN:ENST00000360870.5:exon7:c.C1016T:p.P339L,[illegible]
2	186654199	C	T	GAGATGTTTT,ACAAGATTTG	exonic	FSIP2	nonsynonymous SNV	FSIP2:ENST00000424728.1:exon16:c.C2336T:p.S779L,FSIP2:ENST00000343098.5:exon16:c.C2603T:p.S868L,
2	196759755	C	T	ACTAGTTTTT,CTCCAAATGG	exonic	DNAH7	nonsynonymous SNV	DNAH7:ENST00000312428.6:exon30:c.G4841A:p.G1614E,
2	196836977	C	T	TTCAGACCTT,TTGATATTGT	exonic	DNAH7	nonsynonymous SNV	DNAH7:ENST00000312428.6:exon16:c.G2047A:p.E683K,
2	198949883	C	T	GCCTTCTGAT,CAGATGTGTT	exonic	PLCL1	nonsynonymous SNV	PLCL1:ENST00000487695.2:exon2:c.C1420T:p.P474S,PLCL1:ENST00000428675.1:exon2:c.C1642T:p.P548S,[illegible]
2	198949884	C	T	CCTTCTGATC,AGATGTGTTA	exonic	PLCL1	nonsynonymous SNV	PLCL1:ENST00000487695.2:exon2:c.C1421T:p.P474L,PLCL1:ENST00000428675.1:exon2:c.C1643T:p.P548L,[illegible]
2	202744806	C	T	AAAAGGCTTT,CCAGAGACCG	exonic	CDK15	nonsynonymous SNV	CDK15:ENST00000410091.3:exon13:c.C961T:p.P321S,CDK15:ENST00000374598.4:exon12:c.C1114T:p.P372S,[illegible]
2	203806596	G	C	TTAAATAATA,AAGAAAATGG	splicing	CARF	.	.
2	205829953	C	T	TCGGCAGAGC,CAGATGCTTT	exonic	PARD3B	nonsynonymous SNV	PARD3B:ENST00000349953.3:exon3:c.C301T:p.P101S,PARD3B:ENST00000406610.2:exon3:c.C301T:p.P101S,[illegible]
2	205829954	C	T	CGGCAGAGCC,AGATGCTTTT	exonic	PARD3B	nonsynonymous SNV	PARD3B:ENST00000349953.3:exon3:c.C302T:p.P101L,PARD3B:ENST00000406610.2:exon3:c.C302T:p.P101L,[illegible]
2	209358053	C	T	AAAAGGACAC,GCCATGTGGC	exonic	PTH2R	nonsynonymous SNV	PTH2R:ENST00000272847.2:exon13:c.C1322T:p.P441L,
2	217315769	G	A	CCAAGGAAAT,ACCACCAAGG	exonic	SMARCAL1	nonsynonymous SNV	SMARCAL1:ENST00000392128.2:exon9:c.G1578A:p.M526I,SMARCAL1:ENST00000357276.4:exon12:c.G2052A:p.M684I,[illegible]
2	217525435	C	T	TGAGCAGCAC,GGCAGATGGG	exonic	IGFBP2	nonsynonymous SNV	IGFBP2:ENST00000434997.1:exon2:c.C100T:p.R34W,IGFBP2:ENST00000233809.4:exon2:c.C598T:p.R200W,[illegible]
2	218683369	G	A	CTGGGCAGAC,GGCTGGGCTG	exonic	TNS1	nonsynonymous SNV	TNS1:ENST00000419504.1:exon22:c.C3335T:p.P1112L,TNS1:ENST00000430930.1:exon21:c.C3311T:p.P1104L,[illegible]
2	219747011	T	A	CACCCTGATG,GGCTGCCTCA	exonic	WNT10A	nonsynonymous SNV	WNT10A:ENST00000258411.3:exon2:c.T242A:p.V81E,
2	220172252	C	T	CTGCCCCACT,CCACCTGGCA	exonic	PTPRN	nonsynonymous SNV	PTPRN:ENST00000440552.1:exon3:c.G95A:p.G32E,PTPRN:ENST00000409251.3:exon3:c.G194A:p.G65E,[illegible]
2	227662397	C	G	GGCGTGGGTT,TGTTGGTGCT	exonic	IRS1	nonsynonymous SNV	IRS1:ENST00000305123.5:exon1:c.G1058C:p.R353T,
2	228881906	C	A	GACAGCAGGC,GGCTGTCCAA	exonic	SPHKAP	nonsynonymous SNV	SPHKAP:ENST00000344657.5:exon7:c.G3664T:p.G1222C,SPHKAP:ENST00000392056.3:exon7:c.G3664T:p.G1222C,
2	228996733	C	T	CGGGCCGCTT,CTGAGCTGCC	exonic	SPHKAP	nonsynonymous SNV	SPHKAP:ENST00000344657.5:exon2:c.G101A:p.G34E,SPHKAP:ENST00000392056.3:exon2:c.G101A:p.G34E,
2	231134267	C	T	ACTAGAAAAT,ACCCAATGAA	exonic	SP140	nonsynonymous SNV	SP140:ENST00000486687.2:exon11:c.C1033T:p.H345Y,SP140:ENST00000420434.3:exon12:c.C1180T:p.H394Y,[illegible]
2	233127938	C	T	ATGGTTTGGC,GGACCATCAT	exonic	DIS3L2	nonsynonymous SNV	DIS3L2:ENST00000325385.7:exon13:c.C1447T:p.R483W,DIS3L2:ENST00000390005.5:exon13:c.C1447T:p.R483W,[illegible]
3	1134577	G	A	GCTCCGACAG,AATGCAGCAG	exonic	CNTN6	nonsynonymous SNV	CNTN6:ENST00000413210.1:exon1:c.G158A:p.G53E,CNTN6:ENST00000397479.2:exon1:c.G158A:p.G53E,
3	4738930	G	C	GAAATTGTTT,AGAATTTCCT	exonic	ITPR1	nonsynonymous SNV	ITPR1:ENST00000456211.2:exon33:c.G4309C:p.E1437Q,ITPR1:ENST00000423119.2:exon34:c.G4354C:p.E1452Q,[illegible]
3	4856114	C	T	CAGAGCTGGT,CCTGCAGAAG	exonic	AC018816.3,ITPR1	nonsynonymous SNV	AC018816.3:ENST00000449914.1:exon4:c.G235A:p.D79N,AC018816.3:ENST00000441894.1:exon4:c.G118A:p.D40N,
3	4856115	C	T	AGAGCTGGTC,CTGCAGAAGA	exonic	AC018816.3,ITPR1	nonsynonymous SNV	ITPR1:ENST00000456211.2:exon54:c.C7381T:p.P2461S,ITPR1:ENST00000423119.2:exon55:c.C7426T:p.P2476S,[illegible]
3	10142946	G	C	ACTCTGATTA,ACCCCAGATA	exonic	FANCD2	stoploss	FANCD2:ENST00000383807.1:exon44:c.G4356C:p.X1452Y,FANCD2:ENST00000419585.1:exon44:c.G4356C:p.X1452Y,
3	10953809	C	T	ATCTTTTTCT,CTATGCCATT	exonic	SLC6A11	nonsynonymous SNV	SLC6A11:ENST00000254488.2:exon7:c.C926T:p.S309F,
3	12560690	G	A	GTACGGGACA,ATTTACGTAA	exonic	TSEN2	nonsynonymous SNV	TSEN2:ENST00000446004.1:exon8:c.G1093A:p.D365N,TSEN2:ENST00000402228.3:exon8:c.G1093A:p.D365N,[illegible]
3	19925900	G	A	CATACAAGTA,AAGGAATGGC	exonic	EFHB	nonsynonymous SNV	EFHB:ENST00000295824.9:exon11:c.C2141T:p.S714F,EFHB:ENST00000344838.4:exon13:c.C1751T:p.S584F,
3	27343220	C	T	TCTTGTATTT,CCTAGGGCTC	exonic	NEK10	nonsynonymous SNV	NEK10:ENST00000341435.5:exon14:c.G1135A:p.E379K,NEK10:ENST00000429845.2:exon14:c.G1135A:p.E379K,
3	30891555	T	A	ATCAGGACAA,ATTTGTATCT	exonic	GADL1	nonsynonymous SNV	GADL1:ENST00000282538.5:exon6:c.A584T:p.Y195F,GADL1:ENST00000454381.3:exon6:c.A584T:p.Y195F,
3	33481230	C	T	CCCAGTACCT,ATGCTGTAAG	exonic	UBP1	nonsynonymous SNV	UBP1:ENST00000447368.2:exon1:c.G111A:p.M37I,UBP1:ENST00000456378.1:exon2:c.G111A:p.M37I,[illegible]
3	33725860	G	A	CCTAGCAGGG,GAATTCCTCT	exonic	CLASP2	nonsynonymous SNV	CLASP2:ENST00000359576.5:exon6:c.C635T:p.P212L,CLASP2:ENST00000468888.2:exon6:c.C635T:p.P212L,[illegible]
3	38748786	G	A	CCGTGGGATG,GCTTCTGGGG	exonic	SCN10A	nonsynonymous SNV	SCN10A:ENST00000449082.2:exon25:c.C4370T:p.P1457L,
3	38793886	G	A	TGGTCTCCAG,AAAGACTCCA	exonic	SCN10A	nonsynonymous SNV	SCN10A:ENST00000449082.2:exon11:c.C1579T:p.P527S,
3	44442708	C	T	CAGATATGCT,CAAGCTTGCA	exonic	TCAIM	nonsynonymous SNV	TCAIM:ENST00000342649.4:exon10:c.C1132T:p.P378S,TCAIM:ENST00000417237.1:exon10:c.C1132T:p.P378S,
3	48465824	G	A	GGACACTGTG,CCTCCAGCTG	exonic	PLXNB1	nonsynonymous SNV	PLXNB1:ENST00000456774.1:exon1:c.C197T:p.A66V,PLXNB1:ENST00000296440.6:exon3:c.C197T:p.A66V,[illegible]
3	48683054	G	A	GGTGAACACA,CCAGCAGCTC	exonic	CELSR3	nonsynonymous SNV	CELSR3:ENST00000544264.1:exon25:c.C7634T:p.A2545V,CELSR3:ENST00000164024.4:exon24:c.C7619T:p.A2540V,
3	52263169	C	T	GGGTGCACCT,GTCGTAGAGG	exonic	TWF2	nonsynonymous SNV	TWF2:ENST00000305533.5:exon9:c.G931A:p.E311K,
3	52941090	G	A	GGTACCTTTG,TGAAGGAGGT	exonic	SFMBT1	nonsynonymous SNV	SFMBT1:ENST00000394752.3:exon19:c.C2326T:p.P776S,SFMBT1:ENST00000296295.6:exon19:c.C2326T:p.P776S,[illegible]
3	73453461	A	T	TCTTCTCAAC,CCTGCACCAC	exonic	PDZRN3	nonsynonymous SNV	PDZRN3:ENST00000535920.1:exon2:c.T170A:p.V57E,PDZRN3:ENST00000263666.4:exon4:c.T1004A:p.V335E,[illegible]
3	97806407	C	T	CATATGTAAT,CCTTGCTTTA	exonic	OR5AC2	nonsynonymous SNV	OR5AC2:ENST00000358642.2:exon1:c.C391T:p.P131S,
3	97868379	G	A	CTGTCATCTG,AAAGACCCTC	exonic	OR5H14	stopgain	OR5H14:ENST00000437310.1:exon1:c.G150A:p.W50X,
3	100594363	C	T	TAGTATCACT,CTCCCAGTGG	exonic	ABI3BP	nonsynonymous SNV	ABI3BP:ENST00000284322.5:exon8:c.G827A:p.G276E,ABI3BP:ENST00000495063.1:exon8:c.G827A:p.G276E,[illegible]
3	105422972	G	A	TGGGCAAGGG,AGAGGATCCT	exonic	CBLB	nonsynonymous SNV	CBLB:ENST00000394027.3:exon11:c.C1519T:p.P507S,CBLB:ENST00000405772.1:exon11:c.C1453T:p.P485S,[illegible]
3	108074124	C	G	GATTCTTTTT,TATTAACAGC	exonic	HHLA2	nonsynonymous SNV	HHLA2:ENST00000357759.5:exon5:c.C581G:p.S194C,HHLA2:ENST00000491820.1:exon6:c.C581G:p.S194C,[illegible]
3	108335421	G	A	GAATGGTGAG,AAATTGTTCC	exonic	DZIP3	nonsynonymous SNV	DZIP3:ENST00000479138.1:exon5:c.G292A:p.E98K,DZIP3:ENST00000495008.1:exon5:c.G292A:p.E98K,[illegible]
3	111780672	G	A	ACAATTTTTC,AGTGCCCAGC	exonic	TMPRSS7	nonsynonymous SNV	TMPRSS7:ENST00000452346.2:exon11:c.G1349A:p.R450Q,TMPRSS7:ENST00000419127.1:exon9:c.G971A:p.R324Q,
3	113166977	C	G	CAATTGTCCT,TTTGCTCCTT	exonic	SPICE1	nonsynonymous SNV	SPICE1:ENST00000295872.4:exon16:c.G2334C:p.K778N,
3	119134981	G	A	ACACCAGAAG,GGTTACACTT	exonic	ARHGAP31	nonsynonymous SNV	ARHGAP31:ENST00000264245.4:exon12:c.G4205A:p.G1402E,
3	119255995	T	C	TGTATTCCAG,TGAAGGTCTG	exonic	CD80	nonsynonymous SNV	CD80:ENST00000383669.3:exon3:c.A689G:p.N230S,CD80:ENST00000478182.1:exon4:c.A689G:p.N230S,[illegible]
3	119263493	G	A	TCGTCAGATG,GCGCAGAGCC	exonic	CD80	nonsynonymous SNV	CD80:ENST00000383669.3:exon2:c.C322T:p.P108S,CD80:ENST00000383668.3:exon2:c.C322T:p.P108S,[illegible]
3	119306589	G	A	GCTGGCTGCT,GTGGGGAGTT	exonic	ADPRH	stopgain	ADPRH:ENST00000478927.1:exon4:c.G938A:p.W313X,ADPRH:ENST00000478399.1:exon4:c.G938A:p.W313X,[illegible]
3	120924778	C	G	AGGAAAAAGT,AAAGAGAAGG	exonic	STXBP5L	nonsynonymous SNV	STXBP5L:ENST00000471262.1:exon9:c.C886G:p.Q296E,STXBP5L:ENST00000492541.1:exon10:c.C886G:p.Q296E,[illegible]
3	121647280	G	A	AATGGCCCCA,CCCAGCCAGG	exonic	SLC15A2	nonsynonymous SNV	SLC15A2:ENST00000295605.2:exon14:c.G1126A:p.A376T,SLC15A2:ENST00000489711.1:exon15:c.G1219A:p.A407T,
3	122331927	G	T	GACAACTGTA,AAGTGCTATC	exonic	PARP15	stopgain	PARP15:ENST00000464300.2:exon4:c.G583T:p.E195X,
3	122578959	C	T	GAAACTAATT,TTCTCCTCCT	exonic	DIRC2	nonsynonymous SNV	DIRC2:ENST00000261038.5:exon7:c.C1048T:p.L350F,
3	122578972	T	G	CTCCTCCTGT,TTCGGGAGCT	exonic	DIRC2	nonsynonymous SNV	DIRC2:ENST00000261038.5:exon7:c.T1061G:p.F354C,
3	122641210	C	G	TGCACGGCCT,GCTCATCAGG	exonic	SEMA5B	nonsynonymous SNV	SEMA5B:ENST00000393583.2:exon11:c.G1357C:p.E453Q,SEMA5B:ENST00000357599.3:exon11:c.G1357C:p.E453Q,[illegible]
3	125694443	G	A	GACGCCTCCG,TGAGAGAGCG	exonic	ROPN1B	nonsynonymous SNV	ROPN1B:ENST00000513830.1:exon5:c.G154A:p.V52M,ROPN1B:ENST00000251776.4:exon3:c.G154A:p.V52M,[illegible]
3	128631370	G	A	AAACCTAGAT,AGCAGATTAA	exonic	ACAD9	nonsynonymous SNV	ACAD9:ENST00000508971.1:exon12:c.G1075A:p.E359K,ACAD9:ENST00000308982.7:exon18:c.G1786A:p.E596K,
3	130159460	G	A	AATTATGAGA,AAAAGAATTT	exonic	COL6A5	nonsynonymous SNV	COL6A5:ENST00000373157.4:exon1:c.G107A:p.R36K,COL6A5:ENST00000265379.6:exon35:c.G6278A:p.R2093K,[illegible]
3	130289748	G	A	TATCATGAAG,ATTTTATGAT	exonic	COL6A6	nonsynonymous SNV	COL6A6:ENST00000453409.2:exon6:c.G2488A:p.D830N,COL6A6:ENST00000358511.6:exon6:c.G2488A:p.D830N,
3	133473498	G	T	CCTGAGCCAC,TAAACCTCTT	exonic	TF	nonsynonymous SNV	TF:ENST00000402696.3:exon4:c.G485T:p.R162L,TF:ENST00000264998.3:exon3:c.G104T:p.R35L,TF:ENST00000482271.1:exon3:c.G104T:p.R35L,
3	133478148	C	T	GCAGAGACCA,CGAAGACTGC	exonic	TF	nonsynonymous SNV	TF:ENST00000402696.3:exon9:c.C1178T:p.T393I,TF:ENST00000264998.3:exon8:c.C797T:p.T266I,
3	134090329	C	T	CACCTGGCCC,AGAGCACCTG	exonic	AMOTL2	nonsynonymous SNV	AMOTL2:ENST00000514516.1:exon2:c.G121A:p.G41R,
3	134256013	C	T	CGTCAGAAAT,GCTGGACTGG	exonic	CEP63	nonsynonymous SNV	CEP63:ENST00000332047.5:exon6:c.C458T:p.S153L,CEP63:ENST00000606977.1:exon6:c.C458T:p.S153L,[illegible]
3	138724809	G	A	GAGGACCTCT,GGATCAGGAT	exonic	PRR23A	nonsynonymous SNV	PRR23A:ENST00000383163.2:exon1:c.C302T:p.P101L,
3	[illegible]	C	T	[illegible]	exonic	CLSTN2	nonsynonymous SNV	CLSTN2:ENST00000458420.3:exon12:c.C1907T:p.T636I [illegible]

3	153840472	C	T	CCTCGAGAAT,CTTCCGTGGT	exonic	ARHGEF26	nonsynonymous SNV	ARHGEF26:ENST00000356448.4:exon2:c.C691T:p.P231S,ARHGEF26:ENST00000465817.1:exon2:c.C691T:p.P231S,ARHGEF26:ENST00000465093.1:exon2:c.C691T:p.P231S,ARHGEF26:ENST00000496710.1:exon2:c.C691T:p.P231S,
3	156413772	G	A	ATAAAAAGGA,ACCCCTCTTC	exonic	TIPARP	nonsynonymous SNV	TIPARP:ENST00000461166.1:exon4:c.G1205A:p.R402K,TIPARP:ENST00000486483.1:exon4:c.G1205A:p.R402K,TIPARP:ENST00000473702.1:exon4:c.G1205A:p.R402K,TIPARP:ENST00000481853.1:exon4:c.G1205A:p.R402K,TIPARP:ENST00000542783.1:exon4:c.G1205A:p.R402K
3	157160160	G	A	TCTAGGTTGT,AAACAGCTAT	exonic	PTX3	nonsynonymous SNV	PTX3:ENST00000295927.3:exon3:c.G538A:p.E180K,
3	159713209	G	T	TTTCAACAGT,AGACTGTGCC	exonic	IL12A	stopgain	IL12A:ENST00000480787.1:exon6:c.G511T:p.E171X,IL12A:ENST00000466512.1:exon6:c.G583T:p.E195X,IL12A:ENST00000305579.2:exon7:c.G625T:p.E209X,
3	160786724	G	C	CCTGACCTTT,ACCTGGACAA	exonic	PPM1L	nonsynonymous SNV	PPM1L:ENST00000464260.1:exon5:c.G325C:p.D109H,PPM1L:ENST00000498165.1:exon4:c.G862C:p.D288H,PPM1L:ENST00000295839.9:exon4:c.G481C:p.D161H,
3	169099234	G	A	ATTGAGGGAG,GAGTGCTGGC	exonic	MECOM	nonsynonymous SNV	MECOM:ENST00000486748.1:exon3:c.C188T:p.P63L,MECOM:ENST00000494292.1:exon2:c.C116T:p.P39L,
3	169550939	G	A	ATACCTCAAG,AAAACAGAAA	exonic	LRRIQ4	nonsynonymous SNV	LRRIQ4:ENST00000340806.6:exon4:c.G1498A:p.E500K,
3	169706055	T	C	GCCACCCTCT,CCCCCTTTGG	exonic	SEC62	nonsynonymous SNV	SEC62:ENST00000337002.4:exon7:c.T638C:p.F213S,SEC62:ENST00000480708.1:exon7:c.T638C:p.F213S,
3	169866990	G	A	GCCTGGGAAC,GCTTATTAAC	exonic	PHC3	nonsynonymous SNV	PHC3:ENST00000479467.1:exon5:c.C440T:p.P147L,PHC3:ENST00000474275.1:exon5:c.C409T:p.R137C,PHC3:ENST00000475729.1:exon5:c.C457T:p.R153C,PHC3:ENST00000494943.1:exon5:c.C421T:p.R141C,PHC3:ENST00000495893.2:exon5:c.C457T:p.R153C,
3	169866991	G	A	CCTGGGAACG,CTTATTAACT	exonic	PHC3	nonsynonymous SNV	PHC3:ENST00000479467.1:exon5:c.C439T:p.P147S,
3	169940537	G	C	CACCAGGTCC,GGTGAAAGCC	exonic	PRKCI	nonsynonymous SNV	PRKCI:ENST00000295797.4:exon1:c.G80C:p.R27P,
3	170198660	C	T	GAAAACTCAT,CCCCTCACTC	exonic	SLC7A14	nonsynonymous SNV	SLC7A14:ENST00000231706.5:exon7:c.G1411A:p.D471N,
3	171431832	C	T	TCACTATTAA,CATCTGTAAG	exonic	PLD1	stopgain	PLD1:ENST00000340989.4:exon9:c.G762A:p.W254X,PLD1:ENST00000351298.4:exon9:c.G762A:p.W254X,PLD1:ENST00000356327.5:exon9:c.G762A:p.W254X,PLD1:ENST00000342215.6:exon9:c.G762A:p.W254X,
3	172634255	G	A	CACACCTGAG,ATGCAGGAAA	exonic	SPATA16	nonsynonymous SNV	SPATA16:ENST00000351008.3:exon9:c.C1355T:p.S452F,
3	172694796	C	T	ATGCTCTCTT,CCTTCCTCCA	exonic	SPATA16	nonsynonymous SNV	SPATA16:ENST00000351008.3:exon5:c.G895A:p.E299K,
3	173998951	C	A	CCCTTAATGA,ACCCAACACC	exonic	NLGN1	nonsynonymous SNV	NLGN1:ENST00000545397.1:exon5:c.C2330A:p.T777K,NLGN1:ENST00000457714.1:exon7:c.C2330A:p.T777K,NLGN1:ENST00000401917.3:exon7:c.C2450A:p.T817K,NLGN1:ENST00000361589.4:exon6:c.C2330A:p.T777K,
3	176750919	G	A	AGAATCAAAG,ATGCACTGAA	exonic	TBL1XR1	nonsynonymous SNV	TBL1XR1:ENST00000430069.1:exon14:c.C1256T:p.S419F,TBL1XR1:ENST00000457928.2:exon14:c.C1256T:p.S419F,
3	182577091	G	A	GTATCATGAA,AATCAGATCA	exonic	ATP11B	nonsynonymous SNV	ATP11B:ENST00000323116.5:exon12:c.G1144A:p.E382K,
3	183888355	C	T	GAGCTACGGT,CTCCCGGAGT	exonic	DVL3	nonsynonymous SNV	DVL3:ENST00000431765.1:exon15:c.C1912T:p.P638S,DVL3:ENST00000313143.3:exon15:c.C1963T:p.P655S,
3	183888356	C	T	AGCTACGGTC,TCCCGGAGTG	exonic	DVL3	nonsynonymous SNV	DVL3:ENST00000431765.1:exon15:c.C1913T:p.P638L,DVL3:ENST00000313143.3:exon15:c.C1964T:p.P655L,
3	185979494	C	T	GTACCTTTCT,CTTGTCTCCC	exonic	DGKG	nonsynonymous SNV	DGKG:ENST00000344484.4:exon15:c.G1343A:p.G448E,DGKG:ENST00000382164.4:exon14:c.G1226A:p.G409E,DGKG:ENST00000544847.1:exon13:c.G1166A:p.G389E,DGKG:ENST00000265022.3:exon15:c.G1343A:p.G448E,
3	185979495	C	T	TACCTTTCTC,TTGTCTCCCT	exonic	DGKG	nonsynonymous SNV	DGKG:ENST00000344484.4:exon15:c.G1342A:p.G448R,DGKG:ENST00000382164.4:exon14:c.G1225A:p.G409R,DGKG:ENST00000544847.1:exon13:c.G1165A:p.G389R,DGKG:ENST00000265022.3:exon15:c.G1342A:p.G448R,
3	186769125	G	A	TTCGCCTGAT,AACTCTCAGG	exonic	ST6GAL1	nonsynonymous SNV	ST6GAL1:ENST00000442023.1:exon2:c.G3A:p.M1I,ST6GAL1:ENST00000427315.1:exon2:c.G3A:p.M1I,ST6GAL1:ENST00000455441.1:exon3:c.G3A:p.M1I,ST6GAL1:ENST00000169298.3:exon5:c.G696A:p.M232I,ST6GAL1:ENST00000457772.2:exon4:c.G3A:p.M1I,ST6GAL1:ENST000
3	193040299	G	A	TTTTGGTCCT,GTTTTATGAT	exonic	ATP13A5	nonsynonymous SNV	ATP13A5:ENST00000342358.4:exon15:c.C1739T:p.P580L,
3	194991385	C	T	TTAAGCACCT,GTGCGCCTCG	exonic	XXYLT1	nonsynonymous SNV	XXYLT1:ENST00000310380.6:exon1:c.G403A:p.E135K,
3	195507590	C	T	GAGGAAGTGT,CGTGACAGGA	exonic	MUC4	nonsynonymous SNV	MUC4:ENST00000480843.1:exon2:c.G10861A:p.D3621N,MUC4:ENST00000478156.1:exon2:c.G10861A:p.D3621N,MUC4:ENST00000466475.1:exon2:c.G10861A:p.D3621N,MUC4:ENST00000477086.1:exon2:c.G10861A:p.D3621N,MUC4:ENST00000479406.1:exon2:c.G10861A:p.D36
3	195512786	G	C	GTGACAGGAA,AGGGGTGGTG	exonic	MUC4	nonsynonymous SNV	MUC4:ENST00000480843.1:exon2:c.C5665G:p.L1889V,MUC4:ENST00000478156.1:exon2:c.C5665G:p.L1889V,MUC4:ENST00000466475.1:exon2:c.C5665G:p.L1889V,MUC4:ENST00000477086.1:exon2:c.C5665G:p.L1889V,MUC4:ENST00000479406.1:exon2:c.C5665G:p.L1889V,MU
3	195513377	C	T	GTGGTGTCAT,TGTGGTAGCT	exonic	MUC4	nonsynonymous SNV	MUC4:ENST00000480843.1:exon2:c.G5074A:p.D1692N,MUC4:ENST00000478156.1:exon2:c.G5074A:p.D1692N,MUC4:ENST00000466475.1:exon2:c.G5074A:p.D1692N,MUC4:ENST00000477086.1:exon2:c.G5074A:p.D1692N,MUC4:ENST00000479406.1:exon2:c.G5074A:p.D1692N,M
3	195515737	G	A	GGCCATCCGG,AAATGGCGGC	exonic	MUC4	nonsynonymous SNV	MUC4:ENST00000480843.1:exon2:c.C2714T:p.S905F,MUC4:ENST00000478156.1:exon2:c.C2714T:p.S905F,MUC4:ENST00000466475.1:exon2:c.C2714T:p.S905F,MUC4:ENST00000477086.1:exon2:c.C2714T:p.S905F,MUC4:ENST00000479406.1:exon2:c.C2714T:p.S905F,MUC4:EN
4	435768	T	A	CCAGTATGAA,TCTCCTATGT	exonic	ZNF721	nonsynonymous SNV	ZNF721:ENST00000338977.5:exon2:c.A2452T:p.I818F,ZNF721:ENST00000511833.2:exon3:c.A2488T:p.I830F,
4	2065670	C	T	CACAACTACT,CGCGGTGGTG	exonic	NAT8L	nonsynonymous SNV	NAT8L:ENST00000331662.3:exon3:c.C221T:p.S74F,NAT8L:ENST00000423729.2:exon3:c.C725T:p.S242F,
4	3156048	C	G	ACAAACCCCC,TTCTCTAAGT	exonic	HTT	nonsynonymous SNV	HTT:ENST00000355072.5:exon27:c.C3527G:p.P1176R,
4	16504480	C	T	AGTTGGCTCT,CTACCACCAT	exonic	LDB2	nonsynonymous SNV	LDB2:ENST00000515064.1:exon8:c.G902A:p.G301E,LDB2:ENST00000304523.5:exon8:c.G908A:p.G303E,
4	20715097	C	G	ATTGAATGCT,TAGTTCAGCT	exonic	PACRGL	nonsynonymous SNV	PACRGL:ENST00000511089.1:exon6:c.C385G:p.L129V,PACRGL:ENST00000444671.2:exon4:c.C250G:p.L84V,PACRGL:ENST00000506702.1:exon7:c.C544G:p.L182V,PACRGL:ENST00000502374.1:exon5:c.C385G:p.L129V,PACRGL:ENST00000471979.2:exon7:c.C544G:p.L182V,PA
4	22436941	G	C	TACCTCTTTT,ATTTTTCCTC	exonic	GPR125	stopgain	GPR125:ENST00000502482.1:exon10:c.C1436G:p.S479X,GPR125:ENST00000508133.1:exon5:c.C758G:p.S253X,GPR125:ENST00000334304.5:exon10:c.C1436G:p.S479X,
4	39408886	G	A	GATGGAAAAG,ACCTTCTATA	exonic	KLB	nonsynonymous SNV	KLB:ENST00000257408.4:exon1:c.G317A:p.G106E,
4	52777317	C	T	GCCCCTTTTT,CAGTTTTTCA	exonic	DCUN1D4	nonsynonymous SNV	DCUN1D4:ENST00000451288.2:exon9:c.C829T:p.P277S,DCUN1D4:ENST00000381437.4:exon10:c.C517T:p.P173S,DCUN1D4:ENST00000334635.5:exon9:c.C697T:p.P233S,DCUN1D4:ENST00000510808.1:exon2:c.C127T:p.P43S,
4	55139748	C	G	AACAATGTCT,AAACATCATC	exonic	PDGFRA	stopgain	PDGFRA:ENST00000509490.1:exon10:c.C1409G:p.S470X,PDGFRA:ENST00000257290.5:exon10:c.C1409G:p.S470X,
4	56875950	C	T	AAACAGCCTC,GGCGCCAGCT	exonic	CEP135	nonsynonymous SNV	CEP135:ENST00000257287.4:exon19:c.C2386T:p.R796W,
4	57325688	G	C	AAGAAAATCA,AGAATGTAAT	exonic	PAICS	nonsynonymous SNV	PAICS:ENST00000512576.1:exon9:c.G1262C:p.R421T,PAICS:ENST00000514888.1:exon10:c.G986C:p.R329T,PAICS:ENST00000264221.2:exon10:c.G1262C:p.R421T,PAICS:ENST00000399688.3:exon10:c.G1283C:p.R428T,
4	57797117	T	C	CAGACGGAGG,TGCCCAAATG	exonic	REST	nonsynonymous SNV	REST:ENST00000309042.7:exon4:c.T2093C:p.V698A,
4	66356139	C	T	CTGCCGGGCT,CTGGGCTCAA	exonic	EPHA5	nonsynonymous SNV	EPHA5:ENST00000511294.1:exon5:c.G1358A:p.G453E,EPHA5:ENST00000354839.4:exon5:c.G1358A:p.G453E,EPHA5:ENST00000273854.3:exon5:c.G1358A:p.G453E,
4	68527912	C	G	GTCACCTTCT,TTCCAAGGTT	exonic	UBA6	nonsynonymous SNV	UBA6:ENST00000322244.5:exon13:c.G1099C:p.E367Q,UBA6:ENST00000420827.2:exon13:c.G1099C:p.E367Q,
4	71068046	G	A	TTGCTGTGAT,GTAAGATTCC	exonic	ODAM	nonsynonymous SNV	ODAM:ENST00000514097.1:exon4:c.G459A:p.M153I,ODAM:ENST00000396094.2:exon8:c.G648A:p.M216I,ODAM:ENST00000510709.2:exon7:c.G606A:p.M202I,
4	71508794	G	A	ATACCCTGAG,AAATCCCTTC	exonic	ENAM	nonsynonymous SNV	ENAM:ENST00000396073.3:exon9:c.G1651A:p.E551K,
4	71509696	C	A	TCCCTAACTT,ATCCCACCAA	exonic	ENAM	nonsynonymous SNV	ENAM:ENST00000396073.3:exon9:c.C2553A:p.F851L,
4	71659596	G	A	TCTGCAGCTG,AAGTCGAGGA	exonic	RUFY3	nonsynonymous SNV	RUFY3:ENST00000381006.3:exon13:c.G1432A:p.E478K,RUFY3:ENST00000502653.1:exon14:c.G1273A:p.E425K,
4	72897621	G	A	AGCGGGATAT,AATAGCTTCT	exonic	NPFFR2	nonsynonymous SNV	NPFFR2:ENST00000344413.5:exon1:c.G3A:p.M1I,NPFFR2:ENST00000308744.6:exon1:c.G3A:p.M1I,
4	73280645	G	A	TCTTTCCAAG,GTTCAATGAA	exonic	ADAMTS3	nonsynonymous SNV	ADAMTS3:ENST00000286657.4:exon4:c.C548T:p.P183L,
4	73956389	G	A	CTCACCTGAG,GACTTGGAGC	exonic	ANKRD17	nonsynonymous SNV	ANKRD17:ENST00000330838.6:exon28:c.C6203T:p.P2068L,ANKRD17:ENST00000509867.2:exon29:c.C6617T:p.P2206L,ANKRD17:ENST00000358602.4:exon29:c.C6956T:p.P2319L,
4	77676093	C	T	GGGAGGATCT,CCTCCGAATA	exonic	SHROOM3	nonsynonymous SNV	SHROOM3:ENST00000296043.6:exon7:c.C4457T:p.S1486F,
4	77817637	G	A	CTGAGACCCT,AGGGCTCCGG	exonic	SOWAHB	stopgain	SOWAHB:ENST00000334306.2:exon1:c.C1366T:p.Q456X,
4	85656233	C	T	TACTAAGTCA,GAACAACAGC	exonic	WDFY3	nonsynonymous SNV	WDFY3:ENST00000322366.6:exon43:c.G6956A:p.R2319H,WDFY3:ENST00000295888.4:exon43:c.G6956A:p.R2319H,
4	87023060	C	T	GTGAATAATT,CAGCAGAATG	exonic	MAPK10	nonsynonymous SNV	MAPK10:ENST00000361569.2:exon7:c.G551A:p.G184E,MAPK10:ENST00000449047.2:exon6:c.G116A:p.G39E,MAPK10:ENST00000395166.1:exon7:c.G437A:p.G146E,MAPK10:ENST00000359221.3:exon7:c.G551A:p.G184E,MAPK10:ENST00000395161.2:exon7:c.G551A:p.G184E,M/
4	87729048	C	A	TGAAGATATT,AGGTAAGTGA	exonic	PTPN13	nonsynonymous SNV	PTPN13:ENST00000411767.2:exon45:c.C7081A:p.Q2361K,PTPN13:ENST00000316707.6:exon42:c.C6508A:p.Q2170K,PTPN13:ENST00000511467.1:exon44:c.C7096A:p.Q2366K,PTPN13:ENST00000436978.1:exon45:c.C7096A:p.Q2366K,PTPN13:ENST00000427191.2:exon44:c.C70:
4	88411533	C	G	GTCTGATTGT,AGTGCCACAA	exonic	SPARCL1	nonsynonymous SNV	SPARCL1:ENST00000418378.1:exon8:c.G1423C:p.D475H,SPARCL1:ENST00000282470.6:exon7:c.G1423C:p.D475H,SPARCL1:ENST00000503414.1:exon8:c.G1048C:p.D350H,
4	90756800	C	T	TTTGAAAGTC,TTTCATGAAT	exonic	SNCA	nonsynonymous SNV	SNCA:ENST00000506691.1:exon3:c.G19A:p.G7R,SNCA:ENST00000502987.1:exon1:c.G19A:p.G7R,SNCA:ENST00000336904.3:exon2:c.G19A:p.G7R,SNCA:ENST00000394986.1:exon2:c.G19A:p.G7R,SNCA:ENST00000506244.1:exon2:c.G19A:p.G7R,SNCA:ENST00000345009.4:exo
4	100503179	C	T	TACCGCATTT,CTCCAACGTG	exonic	MTTP	nonsynonymous SNV	MTTP:ENST00000511045.1:exon2:c.C260T:p.S87F,MTTP:ENST00000506883.1:exon2:c.C209T:p.S70F,MTTP:ENST00000265517.5:exon2:c.C179T:p.S60F,MTTP:ENST00000422897.2:exon2:c.C179T:p.S60F,MTTP:ENST00000457717.1:exon3:c.C179T:p.S60F,
4	100574389	G	A	CTGTCAGAGG,GCCACTGCCC	exonic	RP11-766F14.2	nonsynonymous SNV	RP11-766F14.2:ENST00000511828.1:exon1:c.C1417T:p.P473S,
4	106155502	G	T	AAATCCAGGT,AAAGCAGTCA	exonic	TET2	stopgain	TET2:ENST00000305737.2:exon3:c.G403T:p.E135X,TET2:ENST00000265149.5:exon3:c.G403T:p.E135X,TET2:ENST00000540549.1:exon3:c.G403T:p.E135X,TET2:ENST00000380013.4:exon3:c.G403T:p.E135X,TET2:ENST00000545826.1:exon3:c.G403T:p.E135X,TET2:ENST000005
4	114203844	C	T	GGCTATACTC,GTTACATATT	exonic	ANK2	nonsynonymous SNV	ANK2:ENST00000357077.4:exon18:c.C1895T:p.P632L,ANK2:ENST00000504454.1:exon19:c.C1940T:p.P647L,ANK2:ENST00000264366.6:exon18:c.C1895T:p.P632L,ANK2:ENST00000503271.1:exon19:c.C1832T:p.P611L,ANK2:ENST00000394537.3:exon18:c.C1895T:p.P632L,ANK2:E
4	123302238	G	C	AATTTATAAT,AAATACAAAC	exonic	ADAD1	nonsynonymous SNV	ADAD1:ENST00000388725.2:exon3:c.G210C:p.M70I,ADAD1:ENST00000446706.1:exon3:c.G264C:p.M88I,ADAD1:ENST00000296513.2:exon4:c.G264C:p.M88I,ADAD1:ENST00000439307.1:exon3:c.G264C:p.M88I,ADAD1:ENST00000388724.2:exon3:c.G264C:p.M88I,
4	124323317	G	C	CAAGTGTGGA,AATGCACTGC	exonic	SPRY1	nonsynonymous SNV	SPRY1:ENST00000394339.2:exon2:c.G571C:p.E191Q,SPRY1:ENST00000339241.1:exon2:c.G571C:p.E191Q,SPRY1:ENST00000507703.1:exon2:c.G571C:p.E191Q,
4	126337631	C	G	GATCGAGGAT,TAACAGCAAA	exonic	FAT4	nonsynonymous SNV	FAT4:ENST00000394329.3:exon6:c.C6872G:p.S2291C,FAT4:ENST00000335110.5:exon6:c.C1766G:p.S589C,
4	162307150	C	T	AAGAGCACAT,AGTTTGTGTG	exonic	FSTL5	nonsynonymous SNV	FSTL5:ENST00000427802.2:exon15:c.G2263A:p.D755N,FSTL5:ENST00000536695.1:exon15:c.G2290A:p.D764N,FSTL5:ENST00000306100.5:exon16:c.G2293A:p.D765N,FSTL5:ENST00000379164.4:exon16:c.G2290A:p.D764N,
4	166300554	G	A	CGAGCTGCGC,AGGCGCTCGT	exonic	CPE	nonsynonymous SNV	CPE:ENST00000402744.4:exon1:c.G181A:p.E61K,
4	175598335	G	A	GATCCAGAAT,AAACCCAGGA	exonic	GLRA3	nonsynonymous SNV	GLRA3:ENST00000274093.3:exon7:c.C821T:p.S274L,GLRA3:ENST00000340217.5:exon7:c.C821T:p.S274L,
5	1038063	G	C	GCCAGCCTCG,AGCCTGCTGC	exonic	NKD2	nonsynonymous SNV	NKD2:ENST00000296849.5:exon10:c.G931C:p.E311Q,
5	5146258	C	T	GACCTGGTCT,TGCCTACGAG	exonic	ADAMTS16	nonsynonymous SNV	ADAMTS16:ENST00000511368.1:exon3:c.C191T:p.S64F,ADAMTS16:ENST00000274181.7:exon3:c.C191T:p.S64F,
5	14369618	G	A	GGAGCAGAAG,AGGCATTCCT	exonic	TRIO	nonsynonymous SNV	TRIO:ENST00000344204.4:exon18:c.G3202A:p.E1068K,TRIO:ENST00000513206.1:exon14:c.G2401A:p.E801K,TRIO:ENST00000509967.2:exon18:c.G3055A:p.E1019K,TRIO:ENST00000537187.1:exon18:c.G3202A:p.E1068K,TRIO:ENST00000512070.2:exon18:c.G3025A:p.E1009K,
5	15936637	C	T	AAACAGATTT,CATCCGCTAC	exonic	FBXL7	nonsynonymous SNV	FBXL7:ENST00000329673.7:exon3:c.C782T:p.S261F,FBXL7:ENST00000510662.1:exon4:c.C677T:p.S226F,FBXL7:ENST00000504595.1:exon4:c.C818T:p.S273F,
5	19483542	G	A	CTGATGGTGA,GGTGCTGCTG	exonic	CDH18	nonsynonymous SNV	CDH18:ENST00000507958.1:exon14:c.C1750T:p.L584F,CDH18:ENST00000382275.1:exon12:c.C1750T:p.L584F,CDH18:ENST00000274170.4:exon12:c.C1750T:p.L584F,
5	26903744	A	G	GTGAAAAGAA,CCTGTTTGAC	splicing	CDH9(ENST00000231021.4:exon7:c.999+2T>C)	.	.
5	33937773	G	A	CGCGGTAGCC,GAGGACGCCC	exonic	RXFP3	nonsynonymous SNV	RXFP3:ENST00000330120.3:exon1:c.G928A:p.G310R,
5	33937774	G	A	GCGGTAGCCG,AGGACGCCCG	exonic	RXFP3	nonsynonymous SNV	RXFP3:ENST00000330120.3:exon1:c.G929A:p.G310E,
5	34008105	G	C	CTCCACGACC,AGATGCCCTG	exonic	AMACR	nonsynonymous SNV	AMACR:ENST00000502637.1:exon1:c.C20G:p.S7W,AMACR:ENST00000382085.3:exon1:c.C20G:p.S7W,AMACR:ENST00000506639.1:exon1:c.C20G:p.S7W,AMACR:ENST00000382068.3:exon1:c.C20G:p.S7W,AMACR:ENST00000426255.2:exon1:c.C20G:p.S7W,AMACR:ENST00000
5	35910521	C	T	CCATCTTTAT,AAACCTCCGG	exonic	CAPSL	nonsynonymous SNV	CAPSL:ENST00000513623.1:exon3:c.G262A:p.D88N,CAPSL:ENST00000514524.1:exon3:c.G262A:p.D88N,CAPSL:ENST00000397367.2:exon3:c.G262A:p.D88N,CAPSL:ENST00000397366.1:exon3:c.G262A:p.D88N,
5	39134329	C	G	TCATCCTGCT,TGCAACATCA	exonic	FYB	nonsynonymous SNV	FYB:ENST00000351578.6:exon9:c.G1798C:p.E600Q,FYB:ENST00000505428.1:exon9:c.G1798C:p.E600Q,FYB:ENST00000540520.1:exon9:c.G1828C:p.E610Q,FYB:ENST00000512982.1:exon9:c.G1798C:p.E600Q,FYB:ENST00000515010.1:exon8:c.G1798C:p.E600Q,
5	43299001	C	G	AAATAGATCT,AAGGGCAACA	exonic	HMGCS1	nonsynonymous SNV	HMGCS1:ENST00000511774.1:exon2:c.G67C:p.E23Q,HMGCS1:ENST00000325110.6:exon3:c.G67C:p.E23Q,HMGCS1:ENST00000433297.2:exon2:c.G67C:p.E23Q,
5	43529903	G	A	ACCAGCCCCG,ATAAATCTGT	exonic	PAIP1	nonsynonymous SNV	PAIP1:ENST00000338972.4:exon10:c.C995T:p.S332F,PAIP1:ENST00000436644.2:exon10:c.C1094T:p.S365F,PAIP1:ENST00000514816.1:exon2:c.C134T:p.S45F,PAIP1:ENST00000306846.3:exon10:c.C1331T:p.S444F,
5	54635941	C	G	TGAAGAATTT,AAGATGTTGG	exonic	SKIV2L2	nonsynonymous SNV	SKIV2L2:ENST00000545714.1:exon4:c.C316G:p.Q106E,SKIV2L2:ENST00000230640.5:exon6:c.C619G:p.Q207E,
5	54987018	G	A	CATATTTACC,GATTCTCCAT	exonic	SLC38A9	nonsynonymous SNV	SLC38A9:ENST00000512595.1:exon2:c.C31T:p.R11W,
5	55164672	T	A	TAGGAAAAAT,TAACCTGCAC	exonic	IL31RA	nonsynonymous SNV	IL31RA:ENST00000359040.5:exon3:c.T208A:p.L70I,IL31RA:ENST00000447346.2:exon3:c.T208A:p.L70I,IL31RA:ENST00000396836.2:exon3:c.T208A:p.L70I,IL31RA:ENST00000396834.1:exon5:c.T151A:p.L51I,IL31RA:ENST00000354961.4:exon3:c.T151A:p.L51I,
5	55439666	C	T	AAGTCTTTAT,CACAAGGGTG	exonic	ANKRD55	nonsynonymous SNV	ANKRD55:ENST00000341048.4:exon7:c.G574A:p.D192N,ANKRD55:ENST00000513241.2:exon6:c.G487A:p.D163N,
5	65458366	C	T	GACCAAAATT,TGTACCAAGG	exonic	SREK1	nonsynonymous SNV	SREK1:ENST00000334121.6:exon5:c.C701T:p.S234F,SREK1:ENST00000380918.3:exon6:c.C353T:p.S118F,
5	68805317	C	T	CTATACAGAC,CAAGAGCAGC	exonic	OCLN	nonsynonymous SNV	OCLN:ENST00000380766.2:exon3:c.C400T:p.P134S,OCLN:ENST00000396442.2:exon3:c.C400T:p.P134S,OCLN:ENST00000355237.2:exon3:c.C400T:p.P134S,
5	76633109	C	A	TCAAATAGAA,ATGGGGAAGT	exonic	PDE8B	nonsynonymous SNV	PDE8B:ENST00000342343.4:exon5:c.C706A:p.H236N,PDE8B:ENST00000503963.1:exon4:c.C52A:p.H18N,PDE8B:ENST00000346042.3:exon6:c.C766A:p.H256N,PDE8B:ENST00000264917.5:exon6:c.C766A:p.H256N,PDE8B:ENST00000333194.4:exon6:c.C766A:p.H256N,PDE8B:EN!
5	77714722	G	T	TACTACTTGT,GATGTGTGAG	exonic	SCAMP1	nonsynonymous SNV	SCAMP1:ENST00000538629.1:exon5:c.G467T:p.W156L,
5	79732617	C	T	GCATATGATT,TAACCACTGC	exonic	ZFYVE16	nonsynonymous SNV	ZFYVE16:ENST00000510158.1:exon4:c.C113T:p.S38F,ZFYVE16:ENST00000338008.5:exon3:c.C113T:p.S38F,ZFYVE16:ENST00000505560.1:exon4:c.C113T:p.S38F,
5	82834607	C	T	CACAGGTTTT,CTTTGGAGGA	exonic	VCAN	nonsynonymous SNV	VCAN:ENST00000343200.5:exon7:c.C2824T:p.P942S,VCAN:ENST00000513960.1:exon7:c.C2824T:p.P942S,VCAN:ENST00000265077.3:exon8:c.C5785T:p.P1929S,
5	89910681	C	G	ATTAGTAAAT,TTCTTTCAGC	exonic	GPR98	nonsynonymous SNV	GPR98:ENST00000405460.2:exon2:c.C52G:p.L18V,GPR98:ENST00000508842.1:exon2:c.C64G:p.L22V,
5	92924066	C	T	CCGCATCTTC,AGGAGCAGGT	exonic	NR2F1	stopgain	NR2F1:ENST00000327111.3:exon2:c.C907T:p.Q303X,
5	107197425	G	A	GGAGCGCTAG,AGGAGGCGGC	exonic	FBXL17	nonsynonymous SNV	FBXL17:ENST00000359660.5:exon9:c.C908T:p.S303F,FBXL17:ENST00000542267.1:exon9:c.C2102T:p.S701F,
5	107717164	C	T	GGCTCCTCCT,TGGGCCGGCG	exonic	FBXL17	nonsynonymous SNV	FBXL17:ENST00000542267.1:exon1:c.G229A:p.E77K,
5	110074831	G	A	ATGCATCCGC,GCGCCCGGAC	exonic	SLC25A46	nonsynonymous SNV	SLC25A46:ENST00000355943.3:exon1:c.G11A:p.R4Q,SLC25A46:ENST00000447245.2:exon1:c.G11A:p.R4Q,
5	112179285	C	T	GAGGACTGTC,CATTAACAAT	exonic	APC	nonsynonymous SNV	APC:ENST00000257430.4:exon16:c.C7994T:p.P2665L,APC:ENST00000457016.1:exon16:c.C7994T:p.P2665L,APC:ENST00000508376.2:exon17:c.C7994T:p.P2665L,
5	113808751	G	A	TTGTTTTCAG,GTGCTGGTTG	exonic	KCNN2	nonsynonymous SNV	KCNN2:ENST00000512097.3:exon6:c.G1144A:p.G382S,KCNN2:ENST00000503706.1:exon3:c.G100A:p.G34S,KCNN2:ENST00000505491.1:exon3:c.G100A:p.G34S,KCNN2:ENST00000264773.3:exon5:c.G1144A:p.G382S,
5	113808752	G	A	TGTTTTCAGG,TGCTGGTTGC	exonic	KCNN2	nonsynonymous SNV	KCNN2:ENST00000512097.3:exon6:c.G1145A:p.G382D,KCNN2:ENST00000503706.1:exon3:c.G101A:p.G34D,KCNN2:ENST00000505491.1:exon3:c.G101A:p.G34D,KCNN2:ENST00000264773.3:exon5:c.G1145A:p.G382D,
5	115146989	G	A	AAAGCAGTGG,AGTTGGTATG	exonic	CDO1	nonsynonymous SNV	CDO1:ENST00000250535.4:exon3:c.C272T:p.S91F,
5	115783236	G	C	GTGGCGTGAG,ATGGCCTCCG	exonic	SEMA6A	nonsynonymous SNV	SEMA6A:ENST00000513137.1:exon2:c.C447G:p.I149M,SEMA6A:ENST00000510263.1:exon19:c.C2166G:p.I722M,SEMA6A:ENST00000257414.8:exon20:c.C2217G:p.I739M,SEMA6A:ENST00000282394.6:exon6:c.C597G:p.I199M,SEMA6A:ENST00000503865.1:exon2:c.C303G:p.I101I
5	115803294	G	A	TTGTCTTGGT,ATTATGGAAA	exonic	SEMA6A	nonsynonymous SNV	SEMA6A:ENST00000513137.1:exon1:c.C160T:p.H54Y,SEMA6A:ENST00000510263.1:exon18:c.C1879T:p.H627Y,SEMA6A:ENST00000257414.8:exon19:c.C1930T:p.H644Y,SEMA6A:ENST00000343348.6:exon18:c.C1879T:p.H627Y,
5	126394626	C	G	AGAGCAATTT,TCAAGAAGAG	exonic	C5orf63	nonsynonymous SNV	C5orf63:ENST00000508527.1:exon3:c.G62C:p.R21T,C5orf63:ENST00000296662.5:exon3:c.G62C:p.R21T,C5orf63:ENST00000509733.3:exon3:c.G62C:p.R21T,C5orf63:ENST00000606937.1:exon2:c.G62C:p.R21T,C5orf63:ENST00000535381.1:exon3:c.G62C:p.R21T,C5orf63:ENST000
5	126784851	G	T	GAGAGGCCCT,TGGGGGACTG	exonic	MEGF10	nonsynonymous SNV	MEGF10:ENST00000503335.2:exon22:c.G2917T:p.V973L,MEGF10:ENST00000274473.6:exon23:c.G2917T:p.V973L,
5	131325798	G	A	CTCACCTCCT,GTAGGACAGC	exonic	ACSL6	stopgain	ACSL6:ENST00000379272.2:exon4:c.C370T:p.Q124X,ACSL6:ENST00000416557.1:exon4:c.C370T:p.Q124X,ACSL6:ENST00000543479.1:exon4:c.C370T:p.Q124X,ACSL6:ENST00000544770.1:exon4:c.C97T:p.Q33X,ACSL6:ENST00000296869.4:exon4:c.C445T:p.Q149X,ACSL6:ENST
5	132334408	G	A	GGCTGAGGAT,AGGAACTAGA	exonic	ZCCHC10	nonsynonymous SNV	ZCCHC10:ENST00000509437.1:exon5:c.C446T:p.S149L,ZCCHC10:ENST00000355372.2:exon5:c.C428T:p.S143L,ZCCHC10:ENST00000324170.3:exon4:c.C380T:p.S127L,ZCCHC10:ENST00000513848.1:exon3:c.C338T:p.S113L,
5	140208187	A	G	CTTAACCTAT,AACTCAGTTC	exonic	PCDHA6	nonsynonymous SNV	PCDHA6:ENST00000529310.1:exon1:c.A511G:p.K171E,PCDHA6:ENST00000527624.1:exon1:c.A511G:p.K171E,
5	140229968	G	A	GTACACGGGC,AGATCAGCAC	exonic	PCDHA9	nonsynonymous SNV	PCDHA9:ENST00000532602.1:exon1:c.G1888A:p.E630K,PCDHA9:ENST00000378122.3:exon1:c.G1888A:p.E630K,
5	140250453	G	A	TGCGGGCCAC,TGGTGGCGAA	exonic	PCDHA11	nonsynonymous SNV	PCDHA11:ENST00000398640.2:exon1:c.G1765A:p.V589M,
5	140502738	T	A	CTATTCCAGA,AATCTACCGT	exonic	PCDHB4	nonsynonymous SNV	PCDHB4:ENST00000194152.1:exon1:c.T1158A:p.D386E,
5	140552586	G	A	TTAGGGGTAG,GGAACTGAGA	exonic	PCDHB7	nonsynonymous SNV	PCDHB7:ENST00000231137.3:exon1:c.G170A:p.G57E,
5	140712520	G	A	TTCCCACGAG,TCTCCCTCAC	exonic	PCDHGA1	nonsynonymous SNV	PCDHGA1:ENST00000517417.1:exon1:c.G2269A:p.V757I,PCDHGA1:ENST00000378105.3:exon1:c.G2269A:p.V757I,
5	140755068	C	T	TCCATCTTCT,TGTGAATGCA	exonic	PCDHGA6	nonsynonymous SNV	PCDHGA6:ENST00000517434.1:exon1:c.C1418T:p.S473F,
5	140769228	G	C	AGTGGCGGTG,ACGCAGACTC	exonic	PCDHGB4	nonsynonymous SNV	PCDHGB4:ENST00000519479.1:exon1:c.G1777C:p.D593H,
5	140769325	G	A	GGCGAAGTGC,CACAGCGCGT	exonic	PCDHGB4	nonsynonymous SNV	PCDHGB4:ENST00000519479.1:exon1:c.G1874A:p.R625H,
5	141024696	G	C	CTCAAAGTCA,AAAGCTCAGC	exonic	FCHSD1	nonsynonymous SNV	FCHSD1:ENST00000522126.1:exon13:c.C1103G:p.S368C,FCHSD1:ENST00000435817.2:exon14:c.C1331G:p.S444C,FCHSD1:ENST00000518499.1:exon3:c.C380G:p.S127C,FCHSD1:ENST00000522783.1:exon13:c.C1109G:p.S370C,
5	141041798	C	T	GCCCCCTTTC,GGTATACACC	exonic	ARAP3	nonsynonymous SNV	ARAP3:ENST00000513878.1:exon14:c.G1811A:p.R604Q,ARAP3:ENST00000239440.4:exon20:c.G2825A:p.R942Q,
5	145834800	C	T	GCCAATGCCT,CTCCAGGAGG	exonic	TCERG1	nonsynonymous SNV	TCERG1:ENST00000296702.5:exon2:c.C241T:p.P81S,TCERG1:ENST00000549332.1:exon2:c.C241T:p.P81S,TCERG1:ENST00000394421.2:exon2:c.C241T:p.P81S,
5	150496711	C	T	CGTGCCTGGT,CAGGTTTTCT	exonic	ANXA6	nonsynonymous SNV	ANXA6:ENST00000521512.1:exon14:c.G928A:p.D310N,ANXA6:ENST00000523714.1:exon19:c.G1453A:p.D485N,ANXA6:ENST00000377751.5:exon8:c.G520A:p.D174N,ANXA6:ENST00000356496.5:exon20:c.G1549A:p.D517N,ANXA6:ENST00000354546.5:exon20:c.G1549A:p.D517N,
5	159437830	G	A	ATACCTAATA,AACTGGAAAA	exonic	TTC1	nonsynonymous SNV	TTC1:ENST00000231238.5:exon2:c.G295A:p.E99K,TTC1:ENST00000522793.1:exon2:c.G295A:p.E99K,
5	167525117	G	A	ATTTCTAGGA,CAGACTGTGC	exonic	TENM2	nonsynonymous SNV	TENM2:ENST00000519204.1:exon8:c.G1435A:p.A479T,TENM2:ENST00000518659.1:exon9:c.G1798A:p.A600T,TENM2:ENST00000403607.2:exon7:c.G1297A:p.A433T,TENM2:ENST00000545108.1:exon9:c.G1798A:p.A600T,TENM2:ENST00000520394.1:exon6:c.G1102A:p.A368T,
5	169680139	G	A	CTCCGCGGGG,ATGGGGGCCG	exonic	LCP2	nonsynonymous SNV	LCP2:ENST00000521416.1:exon10:c.C614T:p.S205F,LCP2:ENST00000046794.5:exon18:c.C1229T:p.S410F,
5	172585825	G	T	AACTTCTTCA,GGAGGAGATC	exonic	BNIP1	nonsynonymous SNV	BNIP1:ENST00000231668.9:exon5:c.G477T:p.Q159H,BNIP1:ENST00000351486.5:exon4:c.G348T:p.Q116H,
5	172585826	G	A	ACTTCTTCAG,GAGGAGATCT	exonic	BNIP1	nonsynonymous SNV	BNIP1:ENST00000231668.9:exon5:c.G478A:p.G160R,BNIP1:ENST00000351486.5:exon4:c.G349A:p.G117R,
5	172744894	C	T	TCTTCCCACT,GCTGCTTCCG	exonic	STC2	nonsynonymous SNV	STC2:ENST00000265087.4:exon4:c.G865A:p.E289K,
5	175995997	G	A	CTCCGTGAGC,ACCCCTACAT	exonic	CDHR2	nonsynonymous SNV	CDHR2:ENST00000510636.1:exon5:c.G301A:p.D101N,CDHR2:ENST00000261944.5:exon5:c.G301A:p.D101N,CDHR2:ENST00000506348.1:exon4:c.G301A:p.D101N,
5	179263492	G	A	GGGCTTCTCT,ATGAAGGCGG	exonic	SQSTM1	nonsynonymous SNV	SQSTM1:ENST00000402874.3:exon8:c.G970A:p.D324N,SQSTM1:ENST00000376929.3:exon9:c.G970A:p.D324N,SQSTM1:ENST00000360718.5:exon7:c.G970A:p.D324N,SQSTM1:ENST00000389805.4:exon8:c.G1222A:p.D408N,
5	180057060	C	A	CCCCGCCGGT,ATCCCACACC	exonic	FLT4	nonsynonymous SNV	FLT4:ENST00000261937.6:exon5:c.G559T:p.D187Y,FLT4:ENST00000502649.1:exon5:c.G559T:p.D187Y,FLT4:ENST00000393347.3:exon5:c.G559T:p.D187Y,
5	180276661	G	C	TTCTCCCCAA,ATGAACTTTT	exonic	ZFP62	nonsynonymous SNV	ZFP62:ENST00000359141.6:exon3:c.C1654G:p.L552V,ZFP62:ENST00000502412.1:exon2:c.C1834G:p.L612V,ZFP62:ENST00000512132.1:exon3:c.C1735G:p.L579V,
6	1313064	C	G	TCCCTGGGCT,AGATGGGGAC	exonic	FOXQ1	stopgain	FOXQ1:ENST00000296839.2:exon1:c.C125G:p.S42X,
6	1611669	C	T	GAGCTCAGCT,CGGCCTTCTG	exonic	FOXC1	nonsynonymous SNV	FOXC1:ENST00000380874.2:exon1:c.C989T:p.S330F,
6	3850877	A	C	GCGCCTGCTC,GCGACGCCAC	exonic	FAM50B	nonsynonymous SNV	FAM50B:ENST00000380274.1:exon1:c.A832C:p.S278R,FAM50B:ENST00000380272.3:exon2:c.A832C:p.S278R,
6	7393354	G	C	CTTGAAGACA,TCAAAGAGAA	exonic	RIOK1	nonsynonymous SNV	RIOK1:ENST00000379834.2:exon2:c.G94C:p.V32L,RIOK1:ENST00000475351.1:exon2:c.G94C:p.V32L,
6	21065326	G	C	GGTTTTCCTG,AGAAACAGAT	exonic	CDKAL1	nonsynonymous SNV	CDKAL1:ENST00000378610.1:exon10:c.G1103C:p.G368A,CDKAL1:ENST00000274695.4:exon12:c.G1103C:p.G368A,CDKAL1:ENST00000378624.4:exon11:c.G893C:p.G298A,
6	26465517	C	T	CAACAAACTC,AAAAGGAAAA	exonic	BTN2A1	stopgain	BTN2A1:ENST00000541522.1:exon4:c.C634T:p.Q212X,BTN2A1:ENST00000429381.1:exon5:c.C817T:p.Q273X,BTN2A1:ENST00000312541.5:exon5:c.C817T:p.Q273X,BTN2A1:ENST00000469185.1:exon5:c.C817T:p.Q273X,
6	30458264	G	C	AATACCTGGA,AAGGGGAAGG	exonic	HLA-E	nonsynonymous SNV	HLA-E:ENST00000376630.4:exon3:c.G582C:p.E194D,
6	30917650	A	G	ACAGCCAACG,GAAGACCACA	exonic	DPCR1	nonsynonymous SNV	DPCR1:ENST00000462446.1:exon2:c.A1409G:p.E470G,
6	30997314	C	G	ACCACCGCCT,TACTGAAGGC	exonic	MUC22	nonsynonymous SNV	MUC22:ENST00000561890.1:exon2:c.C4106G:p.S1369C,
6	31079598	G	T	GAATTAGAAC,GGGCAGTCGT	exonic	C6orf15	nonsynonymous SNV	C6orf15:ENST00000259870.3:exon2:c.C538A:p.R180S,
6	31623816	G	A	CCAGTGCCCT,AGCACAGTCA	exonic	APOM	nonsynonymous SNV	APOM:ENST00000375916.3:exon1:c.G73A:p.E25K,
6	31696755	C	G	GGTGGCAGTT,TAGCAGCTGT	exonic	DDAH2	nonsynonymous SNV	DDAH2:ENST00000436437.1:exon2:c.G184C:p.E62Q,DDAH2:ENST00000375792.3:exon2:c.G184C:p.E62Q,DDAH2:ENST00000375789.2:exon1:c.G184C:p.E62Q,DDAH2:ENST00000416410.1:exon2:c.G184C:p.E62Q,DDAH2:ENST00000375787.2:exon2:c.G184C:p.E62Q,
6	31696878	C	G	GCCAGGCTCT,TGGGACTCCC	exonic	DDAH2	nonsynonymous SNV	DDAH2:ENST00000436437.1:exon2:c.G61C:p.E21Q,DDAH2:ENST00000375792.3:exon2:c.G61C:p.E21Q,DDAH2:ENST00000375789.2:exon1:c.G61C:p.E21Q,DDAH2:ENST00000416410.1:exon2:c.G61C:p.E21Q,DDAH2:ENST00000375787.2:exon2:c.G61C:p.E21Q,
6	31733760	G	A	CATCACCACG,AATCCGGGGC	exonic	VWA7	nonsynonymous SNV	VWA7:ENST00000375688.4:exon16:c.C2399T:p.S800F,VWA7:ENST00000375686.3:exon16:c.C2399T:p.S800F,
6	31922117	G	A	GTTTCCAAAA,GAGAGAAGGC	exonic	NELFE	nonsynonymous SNV	NELFE:ENST00000454913.1:exon7:c.C845T:p.P282L,NELFE:ENST00000444811.2:exon8:c.C755T:p.P252L,NELFE:ENST00000375425.5:exon8:c.C866T:p.P289L,NELFE:ENST00000375429.3:exon8:c.C845T:p.P282L,NELFE:ENST00000441998.1:exon8:c.C830T:p.P277L,
6	31922118	G	A	TTTCCAAAAG,AGAGAAGGCC	exonic	NELFE	nonsynonymous SNV	NELFE:ENST00000454913.1:exon7:c.C844T:p.P282S,NELFE:ENST00000444811.2:exon8:c.C754T:p.P252S,NELFE:ENST00000375425.5:exon8:c.C865T:p.P289S,NELFE:ENST00000375429.3:exon8:c.C844T:p.P282S,NELFE:ENST00000441998.1:exon8:c.C829T:p.P277S,
6	32609207	C	T	AAGGAGACTG,CTGGCGGTGG	exonic	HLA-DQA1	nonsynonymous SNV	HLA-DQA1:ENST00000395363.1:exon2:c.C203T:p.A68V,HLA-DQA1:ENST00000343139.5:exon2:c.C203T:p.A68V,HLA-DQA1:ENST00000496318.1:exon2:c.C203T:p.A68V,HLA-DQA1:ENST00000374949.2:exon2:c.C203T:p.A68V,
6	32609297	T	G	AACTTGAACA,CATGATTAAA	exonic	HLA-DQA1	nonsynonymous SNV	HLA-DQA1:ENST00000395363.1:exon2:c.T293G:p.I98S,HLA-DQA1:ENST00000343139.5:exon2:c.T293G:p.I98S,HLA-DQA1:ENST00000496318.1:exon2:c.T293G:p.I98S,HLA-DQA1:ENST00000374949.2:exon2:c.T293G:p.I98S,
6	33036827	C	T	GGTCCAAGCC,CAGTGCTCCA	exonic	HLA-DPA1	stopgain	HLA-DPA1:ENST00000419277.1:exon4:c.G597A:p.W199X,HLA-DPA1:ENST00000428995.1:exon3:c.G597A:p.W199X,HLA-DPA1:ENST00000453337.1:exon4:c.G597A:p.W199X,
6	33036828	C	T	GTCCAAGCCC,AGTGCTCCAC	exonic	HLA-DPA1	stopgain	HLA-DPA1:ENST00000419277.1:exon4:c.G596A:p.W199X,HLA-DPA1:ENST00000428995.1:exon3:c.G596A:p.W199X,HLA-DPA1:ENST00000453337.1:exon4:c.G596A:p.W199X,
6	34957089	C	T	TTGACAGCTT,TGAGGTAGAG	exonic	ANKS1A	nonsynonymous SNV	ANKS1A:ENST00000360359.3:exon9:c.C1298T:p.S433F,
6	35586951	C	G	CCTCCATCTT,AATAAATCC	exonic	FKBP5	nonsynonymous SNV	FKBP5:ENST00000539068.1:exon5:c.G430C:p.E144Q,FKBP5:ENST00000542713.1:exon5:c.G430C:p.E144Q,FKBP5:ENST00000357266.4:exon5:c.G430C:p.E144Q,FKBP5:ENST00000536438.1:exon6:c.G430C:p.E144Q,
6	38738239	G	T	ATAAAACAAG,AAACTGATTT	exonic	DNAH8	nonsynonymous SNV	DNAH8:ENST00000449981.2:exon12:c.G1668T:p.R556S,DNAH8:ENST00000359357.3:exon10:c.G1017T:p.R339S,DNAH8:ENST00000441566.1:exon8:c.G1017T:p.R339S,DNAH8:ENST00000327475.6:exon11:c.G1632T:p.R544S,
6	42712806	G	A	TCTTCAGGAA,AATACTCCAG	exonic	TBCC	nonsynonymous SNV	TBCC:ENST00000244625.2:exon2:c.C1006T:p.L336F,TBCC:ENST00000372876.1:exon1:c.C1006T:p.L336F,
6	43018971	G	C	GGACCGTGCT,AGCTCCTTGG	exonic	CUL7	stopgain	CUL7:ENST00000535468.1:exon4:c.C1220G:p.S407X,CUL7:ENST00000265348.3:exon4:c.C968G:p.S323X,
6	70506751	G	A	CAGCTCCGCC,AGGCCGCGCC	exonic	LMBRD1	nonsynonymous SNV	LMBRD1:ENST00000370577.3:exon1:c.C23T:p.S8L,LMBRD1:ENST00000472827.1:exon1:c.C23T:p.S8L,
6	72960130	C	T	CCTCGAAATC,CTATGTAAAA	exonic	RIMS1	nonsynonymous SNV	RIMS1:ENST00000491071.2:exon13:c.C2339T:p.P780L,RIMS1:ENST00000401910.3:exon8:c.C761T:p.P254L,RIMS1:ENST00000523963.1:exon8:c.C761T:p.P254L,RIMS1:ENST00000521978.1:exon13:c.C2339T:p.P780L,RIMS1:ENST00000518273.1:exon13:c.C2339T:p.P780L,RIMS1:
6	75977465	C	T	TATAATCAAT,TGCAAGAGAA	splicing	TMEM30A(ENST00000230461.6:exon3:c.238-1G>	.	.
6	100838568	C	T	ACTACTTATC,GAGATAGTGC	exonic	SIM1	nonsynonymous SNV	SIM1:ENST00000369208.3:exon12:c.G1970A:p.R657Q,SIM1:ENST00000262901.4:exon11:c.G1970A:p.R657Q,
6	101215161	C	G	TAGGCAGTCT,AAACACTATT	exonic	ASCC3	nonsynonymous SNV	ASCC3:ENST00000522650.1:exon9:c.G1456C:p.E486Q,ASCC3:ENST00000369162.2:exon9:c.G1456C:p.E486Q,
6	118791747	C	T	TCTTTCTTTT,CAGCTCTTCC	exonic	CEP85L	nonsynonymous SNV	CEP85L:ENST00000368491.3:exon11:c.G1975A:p.E659K,CEP85L:ENST00000368488.5:exon12:c.G1984A:p.E662K,
6	137814748	C	T	TGAGAGCGCG,CGCCCAAGAT	exonic	OLIG3	nonsynonymous SNV	OLIG3:ENST00000367734.2:exon1:c.G560A:p.G187D,
6	146350873	T	C	CAGGGAGCAG,ATGGCATCCA	exonic	GRM1	nonsynonymous SNV	GRM1:ENST00000355289.4:exon1:c.T220C:p.Y74H,GRM1:ENST00000492807.2:exon2:c.T220C:p.Y74H,GRM1:ENST00000361719.2:exon2:c.T220C:p.Y74H,GRM1:ENST00000282753.1:exon1:c.T220C:p.Y74H,GRM1:ENST00000392299.2:exon2:c.T220C:p.Y74H,GRM1:ENST000005(
6	150267533	G	A	AGGCAAGGAT,TCTTGTGAGC	exonic	ULBP2	nonsynonymous SNV	ULBP2:ENST00000367351.3:exon3:c.G375A:p.M125I,
6	159029421	G	A	GATGCTGGAT,ACCTCTTTCA	exonic	TMEM181	nonsynonymous SNV	TMEM181:ENST00000367090.3:exon9:c.G1141A:p.D381N,
6	159084346	G	C	GTTAGAACGC,AGGCCATTCT	exonic	SYTL3	nonsynonymous SNV	SYTL3:ENST00000297239.9:exon3:c.G46C:p.E16Q,SYTL3:ENST00000360448.3:exon5:c.G46C:p.E16Q,
6	161143583	G	A	GAAGACCCCA,AAAACTACCC	exonic	PLG	nonsynonymous SNV	PLG:ENST00000308192.9:exon10:c.G1240A:p.E414K,
6	170117937	C	T	CACTGAGTTT,AGTAATGACA	exonic	PHF10	nonsynonymous SNV	PHF10:ENST00000339209.4:exon4:c.G391A:p.E131K,PHF10:ENST00000366780.4:exon4:c.G385A:p.E129K,
7	5540758	G	A	GTTGCAGCAG,ACGCCACCAG	exonic	FBXL18	nonsynonymous SNV	FBXL18:ENST00000453700.3:exon3:c.C1142T:p.S381F,FBXL18:ENST00000415009.1:exon3:c.C1142T:p.S381F,FBXL18:ENST00000382368.3:exon3:c.C1142T:p.S381F,
7	10973266	G	T	ATTTCATTTA,AAATCTGGAC	exonic	NDUFA4	nonsynonymous SNV	NDUFA4:ENST00000339600.5:exon4:c.C243A:p.F81L,
7	18067374	G	A	GTCCTGGTGG,AGCTGCCGCT	exonic	PRPS1L1	nonsynonymous SNV	PRPS1L1:ENST00000506618.2:exon1:c.C32T:p.S11F,
7	30951862	C	G	GCCATCCTCT,AGGCATCACC	exonic	AQP1	stopgain	AQP1:ENST00000434909.2:exon7:c.C518G:p.S173X,AQP1:ENST00000311813.4:exon1:c.C338G:p.S113X,AQP1:ENST00000509504.1:exon7:c.C869G:p.S290X,
7	31614329	A	T	AAAGCAGCGA,TGGACATTGA	exonic	CCDC129	nonsynonymous SNV	CCDC129:ENST00000407970.3:exon7:c.A571T:p.M191L,CCDC129:ENST00000319386.3:exon7:c.A571T:p.M191L,CCDC129:ENST00000451887.2:exon7:c.A649T:p.M217L,CCDC129:ENST00000409210.1:exon5:c.A295T:p.M99L,
7	32909018	G	C	CCCATCCGTT,AAAAAAGATA	exonic	KBTBD2	stopgain	KBTBD2:ENST00000304056.4:exon4:c.C1811G:p.S604X,
7	33057212	C	T	TCATATCCTT,TCTGTAAGAT	exonic	NT5C3A	nonsynonymous SNV	NT5C3A:ENST00000405342.1:exon7:c.G430A:p.E144K,NT5C3A:ENST00000610140.1:exon7:c.G532A:p.E178K,NT5C3A:ENST00000396152.2:exon8:c.G430A:p.E144K,NT5C3A:ENST00000409787.1:exon7:c.G430A:p.E144K,NT5C3A:ENST00000242210.7:exon7:c.G547A:p.E183K,NT5
7	63726355	A	T	CCAAGAAGAT,TGGAAAAAGT	exonic	ZNF679	nonsynonymous SNV	ZNF679:ENST00000255746.4:exon4:c.A344T:p.Y115F,ZNF679:ENST00000421025.1:exon5:c.A344T:p.Y115F,
7	77807390	G	A	ATCCTCCGAA,ATGAACATCC	exonic	MAGI2	nonsynonymous SNV	MAGI2:ENST00000419488.1:exon13:c.C2299T:p.L767F,MAGI2:ENST00000522391.1:exon14:c.C2341T:p.L781F,MAGI2:ENST00000354212.4:exon14:c.C2341T:p.L781F,
7	81765956	G	A	CTCACATCGA,ATCATCCTTT	exonic	CACNA2D1	nonsynonymous SNV	CACNA2D1:ENST00000356253.5:exon5:c.C391T:p.L131F,CACNA2D1:ENST00000423588.1:exon5:c.C391T:p.L131F,CACNA2D1:ENST00000356860.3:exon5:c.C391T:p.L131F,
7	82584580	C	T	GATTGCTCAT,TGTTGGTGAG	exonic	PCLO	nonsynonymous SNV	PCLO:ENST00000423517.2:exon5:c.G5689A:p.D1897N,PCLO:ENST00000333891.9:exon5:c.G5689A:p.D1897N,
7	82586058	G	A	TGAGGGGCTG,AAGGGCTGCT	exonic	PCLO	nonsynonymous SNV	PCLO:ENST00000423517.2:exon5:c.C4211T:p.S1404F,PCLO:ENST00000333891.9:exon5:c.C4211T:p.S1404F,
7	88964442	C	T	GGACACATCC,CTAGCAGCAT	exonic	ZNF804B	nonsynonymous SNV	ZNF804B:ENST00000333190.4:exon4:c.C2146T:p.P716S,
7	88964489	C	G	GTCATAGATT,AATGGTAATA	exonic	ZNF804B	nonsynonymous SNV	ZNF804B:ENST00000333190.4:exon4:c.C2193G:p.F731L,
7	91708789	G	C	AAGCATACCA,AGAATAGTGT	exonic	AKAP9	nonsynonymous SNV	AKAP9:ENST00000359028.2:exon32:c.G7378C:p.E2460Q,AKAP9:ENST00000358100.2:exon32:c.G7378C:p.E2460Q,AKAP9:ENST00000356239.3:exon31:c.G7342C:p.E2448Q,AKAP9:ENST00000394534.2:exon5:c.G880C:p.E294Q,
7	92887559	C	G	GATTGGACTT,TGAAATCTCT	exonic	CCDC132	nonsynonymous SNV	CCDC132:ENST00000458530.1:exon7:c.C508G:p.L170V,CCDC132:ENST00000251739.5:exon7:c.C511G:p.L171V,CCDC132:ENST00000544910.1:exon8:c.C421G:p.L141V,CCDC132:ENST00000305866.5:exon7:c.C511G:p.L171V,
7	99690754	G	A	TGGTCTCTGA,TCCTGAAGGA	exonic	MCM7	nonsynonymous SNV	MCM7:ENST00000303887.5:exon15:c.C1961T:p.T654I,MCM7:ENST00000354230.3:exon14:c.C1433T:p.T478I,
7	99818176	C	G	CCAGGGCTCT,TGCCCCGCCG	exonic	PVRIG	nonsynonymous SNV	PVRIG:ENST00000317271.2:exon4:c.C476G:p.S159C,
7	99999580	G	A	AGGAGCTGTG,ATTTCCTGCC	exonic	ZCWPW1	nonsynonymous SNV	ZCWPW1:ENST00000398027.2:exon17:c.C1556T:p.S519F,
7	100253431	G	A	CTCACCTTGG,ACAGTCCTCC	exonic	ACTL6B	nonsynonymous SNV	ACTL6B:ENST00000160382.5:exon2:c.C97T:p.P33S,
7	100279376	G	C	CTCACCAGAA,ATCCGTGCAG	exonic	GIGYF1	nonsynonymous SNV	GIGYF1:ENST00000275732.5:exon24:c.C3074G:p.S1025C,
7	100663433	C	T	AGGCCAGGGA,CATGGCGCTG	exonic	MUC17	nonsynonymous SNV	MUC17:ENST00000306151.4:exon1:c.C17T:p.T6I,MUC17:ENST00000379439.3:exon1:c.C17T:p.T6I,
7	100883023	G	A	GGGGAGAACA,GGAAAGGACA	exonic	FIS1	nonsynonymous SNV	FIS1:ENST00000435848.1:exon4:c.C386T:p.P129L,
7	105108839	C	G	CTTCTATCCT,TTGCTTACCA	exonic	PUS7	nonsynonymous SNV	PUS7:ENST00000356362.2:exon12:c.G1470C:p.K490N,PUS7:ENST00000469408.1:exon12:c.G1470C:p.K490N,
7	106786811	G	C	TGGTAACTAT,ATAATCGTGG	exonic	PRKAR2B	nonsynonymous SNV	PRKAR2B:ENST00000265717.4:exon6:c.G646C:p.D216H,
7	107255739	G	C	AAAGGAAGTA,AAAGCTTTTA	exonic	BCAP29	nonsynonymous SNV	BCAP29:ENST00000379121.2:exon5:c.G457C:p.E153Q,
7	111368493	G	A	TAGCGGGACG,GGCGCCGCAG	exonic	DOCK4	nonsynonymous SNV	DOCK4:ENST00000494651.2:exon22:c.C2387T:p.P796L,DOCK4:ENST00000428084.1:exon53:c.C5765T:p.P1922L,DOCK4:ENST00000437633.1:exon52:c.C5738T:p.P1913L,
7	121651366	G	A	ATACAATGGT,AGACACCTCT	exonic	PTPRZ1	nonsynonymous SNV	PTPRZ1:ENST00000393386.2:exon12:c.G2266A:p.E756K,
7	123101473	G	A	TAGAGCTGTT,AAGTAAACAT	exonic	IQUB	stopgain	IQUB:ENST00000324698.6:exon11:c.C1945T:p.Q649X,IQUB:ENST00000466202.1:exon11:c.C1945T:p.Q649X,
7	123302805	C	A	TAGCTCTTCA,CTTATGTATC	exonic	LMOD2	nonsynonymous SNV	LMOD2:ENST00000458573.2:exon2:c.C1165A:p.P389T,
7	128487879	G	A	CAGTCAAGCT,GCCGGGCGCC	exonic	FLNC	nonsynonymous SNV	FLNC:ENST00000325888.8:exon25:c.G4417A:p.G1473S,FLNC:ENST00000346177.6:exon25:c.G4417A:p.G1473S,
7	128487880	G	A	AGTCAAGCTG,CCGGGCGCCC	exonic	FLNC	nonsynonymous SNV	FLNC:ENST00000325888.8:exon25:c.G4418A:p.G1473D,FLNC:ENST00000346177.6:exon25:c.G4418A:p.G1473D,
7	131195039	G	A	CAGATCACCC,AGGTCAGGAG	exonic	PODXL	nonsynonymous SNV	PODXL:ENST00000541194.1:exon3:c.C764T:p.S255L,PODXL:ENST00000378555.3:exon3:c.C758T:p.S253L,
7	133002139	C	G	GCTGGAAGCT,AAGTGGTAAG	exonic	EXOC4	stopgain	EXOC4:ENST00000539845.1:exon5:c.C455G:p.S152X,EXOC4:ENST00000393161.2:exon5:c.C758G:p.S253X,EXOC4:ENST00000253861.4:exon5:c.C758G:p.S253X,
7	137076063	C	T	CTGCTGTGCT,TTTCTTGAGG	exonic	DGKI	nonsynonymous SNV	DGKI:ENST00000446122.1:exon33:c.G3047A:p.R1016K,DGKI:ENST00000288490.5:exon34:c.G3101A:p.R1034K,DGKI:ENST00000424189.2:exon34:c.G3140A:p.R1047K,DGKI:ENST00000453654.2:exon33:c.G2108A:p.R703K,
7	141727435	C	T	GCCCTTCCCT,CTACTGGGCG	exonic	MGAM	nonsynonymous SNV	MGAM:ENST00000549489.2:exon10:c.C1121T:p.S374F,MGAM:ENST00000475668.2:exon10:c.C1121T:p.S374F,
7	142750060	C	T	GTCCTCAGCT,CTTCCTGGTG	exonic	OR6V1	nonsynonymous SNV	OR6V1:ENST00000418316.1:exon1:c.C623T:p.S208F,
7	148802433	G	A	GCGCCCTGGG,TCTCCCGAGG	exonic	ZNF425	nonsynonymous SNV	ZNF425:ENST00000378061.2:exon4:c.C530T:p.T177I,
7	148978841	C	T	GCGGGCATTC,CCTGCCCCGA	exonic	ZNF783	nonsynonymous SNV	ZNF783:ENST00000434415.1:exon6:c.C1048T:p.P350S,
7	150555934	C	T	CGTGGTCCAG,CAACTCTGGA	exonic	AOC1	nonsynonymous SNV	AOC1:ENST00000493429.1:exon5:c.C1654T:p.P552S,AOC1:ENST00000416793.2:exon3:c.C1654T:p.P552S,AOC1:ENST00000360937.4:exon3:c.C1654T:p.P552S,AOC1:ENST00000467291.1:exon5:c.C1654T:p.P552S,
7	150652584	G	A	CCCGGCTGGG,CCGCCATGGA	exonic	KCNH2	nonsynonymous SNV	KCNH2:ENST00000330883.4:exon1:c.C8T:p.A3V,
7	150761783	G	T	AGATGAGGAT,AGGCCAGCGA	exonic	SLC4A2	stopgain	SLC4A2:ENST00000483786.1:exon4:c.G388T:p.E130X,SLC4A2:ENST00000310317.5:exon4:c.G142T:p.E48X,SLC4A2:ENST00000392826.2:exon3:c.G361T:p.E121X,SLC4A2:ENST00000490898.1:exon4:c.G388T:p.E130X,SLC4A2:ENST00000463414.1:exon4:c.G388T:p.E130X,SLC4A
7	151078424	G	A	TCAGGGATGG,GAGGGAGGAC	exonic	WDR86	nonsynonymous SNV	WDR86:ENST00000469830.2:exon6:c.C1439T:p.P480L,
7	151097234	G	A	ACACTGCCCG,TCAGCACGTC	exonic	WDR86	nonsynonymous SNV	WDR86:ENST00000334493.6:exon2:c.C257T:p.T86I,WDR86:ENST00000469830.2:exon2:c.C257T:p.T86I,
7	151859595	G	C	TTGCTTGTGA,AAATCACTAT	exonic	KMT2C	nonsynonymous SNV	KMT2C:ENST00000262189.6:exon43:c.C11067G:p.F3689L,KMT2C:ENST00000355193.2:exon43:c.C11067G:p.F3689L,KMT2C:ENST00000424877.1:exon1:c.C825G:p.F275L,
7	154862818	C	A	CGTGTACGCA,CTTCCACCGC	exonic	HTR5A,HTR5A-AS1	nonsynonymous SNV	HTR5A-AS1:ENST00000395731.2:exon1:c.G196T:p.V66L,HTR5A:ENST00000287907.2:exon1:c.C209A:p.T70N,HTR5A-AS1:ENST00000543018.1:exon1:c.G196T:p.V66L,
7	156754903	C	T	CGCAAAATTC,AGGCTATGAC	exonic	NOM1	nonsynonymous SNV	NOM1:ENST00000275820.3:exon5:c.C1691T:p.P564L,
8	2857542	C	T	CACGGAAGTT,CCACAAGCCG	exonic	CSMD1	nonsynonymous SNV	CSMD1:ENST00000520002.1:exon54:c.G8144A:p.G2715E,CSMD1:ENST00000602723.1:exon53:c.G7970A:p.G2657E,CSMD1:ENST00000537824.1:exon53:c.G8141A:p.G2714E,CSMD1:ENST00000542608.1:exon52:c.G7967A:p.G2656E,CSMD1:ENST00000602557.1:exon54:c.G8144
8	3432529	G	A	TACTGAACCG,ATAATTAGGG	exonic	CSMD1	nonsynonymous SNV	CSMD1:ENST00000539096.1:exon10:c.C1282T:p.P428S,CSMD1:ENST00000520002.1:exon11:c.C1285T:p.P429S,CSMD1:ENST00000602723.1:exon11:c.C1285T:p.P429S,CSMD1:ENST00000537824.1:exon10:c.C1282T:p.P428S,CSMD1:ENST00000542608.1:exon10:c.C1282T:p.P42
8	27922053	C	T	TTCCACATCT,GTCCCTCTTG	exonic	NUGGC	nonsynonymous SNV	NUGGC:ENST00000413272.2:exon7:c.G907A:p.E303K,NUGGC:ENST00000341513.6:exon7:c.G907A:p.E303K,
8	28981619	G	A	CATTTTCTAC,AAGTCTCTCT	exonic	KIF13B	nonsynonymous SNV	KIF13B:ENST00000524189.1:exon27:c.C3274T:p.R1092C,
8	41582063	T	A	TGCAGGGGCG,GAATCCCGTC	exonic	ANK1	nonsynonymous SNV	ANK1:ENST00000396942.1:exon7:c.A622T:p.T208S,ANK1:ENST00000347528.4:exon7:c.A622T:p.T208S,ANK1:ENST00000396945.1:exon7:c.A622T:p.T208S,ANK1:ENST00000289734.7:exon7:c.A622T:p.T208S,ANK1:ENST00000352337.4:exon7:c.A622T:p.T208S,ANK1:ENST000003
8	42868496	C	T	AACAAAGAGT,AGAGAGAGAT	exonic	HOOK3	stopgain	HOOK3:ENST00000533539.1:exon7:c.C403T:p.Q135X,HOOK3:ENST00000307602.4:exon21:c.C1969T:p.Q657X,
8	42873604	C	T	AGCAGAAGAT,ATACCCAGGC	exonic	HOOK3,RP11-598P20.5	nonsynonymous SNV	HOOK3:ENST00000307602.4:exon22:c.C2120T:p.S707L,RP11-598P20.5:ENST00000534420.1:exon1:c.C38T:p.S13L,
8	48715992	G	C	TCTGGTTTTT,ACTCTTTATG	exonic	PRKDC	stopgain	PRKDC:ENST00000314191.2:exon71:c.C9794G:p.S3265X,PRKDC:ENST00000338368.3:exon71:c.C9794G:p.S3265X,
8	52370130	G	A	GCCATGCACT,ATAGACACCT	exonic	PXDNL	stopgain	PXDNL:ENST00000543296.1:exon9:c.C910T:p.Q304X,PXDNL:ENST00000356297.4:exon9:c.C910T:p.Q304X,
8	61773502	G	A	AGAGATATA,AGACCCCACC	exonic	CHD7	nonsynonymous SNV	CHD7:ENST00000423902.2:exon35:c.G7648A:p.E2550K,
8	67578125	G	A	CCTACCAAGG,GATATAATGG	exonic	VCPIP1	nonsynonymous SNV	VCPIP1:ENST00000310421.4:exon1:c.C1069T:p.P357S,
8	67763093	G	C	TCCTTTTTTT,AATCACTCAG	exonic	C8orf44-SGK3,SGK3	nonsynonymous SNV	C8orf44-SGK3:ENST00000519289.1:exon18:c.G1258C:p.E420Q,SGK3:ENST00000345714.4:exon15:c.G1258C:p.E420Q,SGK3:ENST00000396596.1:exon16:c.G1258C:p.E420Q,SGK3:ENST00000522398.1:exon17:c.G1258C:p.E420Q,SGK3:ENST00000521198.2:exon16:c.G1258C:p.E
8	72184033	C	T	ATTATTGTTT,TTCGGCCCCG	exonic	EYA1	nonsynonymous SNV	EYA1:ENST00000388741.2:exon9:c.G824A:p.R275K,EYA1:ENST00000388742.4:exon9:c.G926A:p.R309K,EYA1:ENST00000388743.2:exon9:c.G923A:p.R308K,EYA1:ENST00000303824.7:exon10:c.G908A:p.R303K,EYA1:ENST00000419131.1:exon9:c.G911A:p.R304K,EYA1:ENST000
8	75276358	C	T	AACTTGGAAA,CTATTACGAG	exonic	GDAP1	nonsynonymous SNV	GDAP1:ENST00000434412.2:exon6:c.C629T:p.T210I,GDAP1:ENST00000220822.7:exon6:c.C833T:p.T278I,
8	77620075	G	A	CAGCTACACT,CAAGACTGAT	exonic	ZFHX4	nonsynonymous SNV	ZFHX4:ENST00000455469.2:exon3:c.G2807A:p.C936Y,ZFHX4:ENST00000050961.6:exon3:c.G2807A:p.C936Y,ZFHX4:ENST00000521891.2:exon3:c.G2885A:p.C962Y,ZFHX4:ENST00000518282.1:exon2:c.G2807A:p.C936Y,
8	77766544	C	T	CGGAAGACCT,CCTCGGCCTC	exonic	ZFHX4	nonsynonymous SNV	ZFHX4:ENST00000455469.2:exon10:c.C7252T:p.P2418S,ZFHX4:ENST00000050961.6:exon10:c.C7252T:p.P2418S,ZFHX4:ENST00000521891.2:exon10:c.C7387T:p.P2463S,ZFHX4:ENST00000518282.1:exon9:c.C7309T:p.P2437S,
8	87507171	C	G	CCTCCACACT,TACCCTCAGT	exonic	RMDN1	nonsynonymous SNV	RMDN1:ENST00000522804.1:exon3:c.G272C:p.R91T,RMDN1:ENST00000523370.1:exon3:c.G140C:p.R47T,
8	95844388	G	A	CAAAACCGAA,AAATGCAGTG	exonic	INTS8	nonsynonymous SNV	INTS8:ENST00000343161.4:exon6:c.G739A:p.E247K,INTS8:ENST00000447247.1:exon6:c.G739A:p.E247K,INTS8:ENST00000523731.1:exon6:c.G739A:p.E247K,INTS8:ENST00000524333.1:exon6:c.G739A:p.E247K,INTS8:ENST00000523206.1:exon6:c.G739A:p.E247K,
8	98288979	A	G	TATCTCCACA,TCTTGTCAGA	exonic	TSPYL5	nonsynonymous SNV	TSPYL5:ENST00000322128.3:exon1:c.T1094C:p.I365T,
8	99718799	G	T	CCTATTTCTT,AATCACCTCA	exonic	STK3	nonsynonymous SNV	STK3:ENST00000523601.1:exon8:c.C664A:p.Q222K,STK3:ENST00000419617.2:exon6:c.C580A:p.Q194K,
8	107719207	A	T	ACAAAGGAAA,CAAGGAAAGG	exonic	OXR1	nonsynonymous SNV	OXR1:ENST00000312046.6:exon7:c.A1437T:p.K479N,OXR1:ENST00000442977.2:exon8:c.A1461T:p.K487N,OXR1:ENST00000531443.1:exon8:c.A1458T:p.K486N,OXR1:ENST00000517566.2:exon8:c.A1458T:p.K486N,OXR1:ENST00000445937.1:exon9:c.A1458T:p.K486N,OXR1:ENS
8	110308626	G	T	CCATTTTTCA,AAGCGCTATT	exonic	NUDCD1	nonsynonymous SNV	NUDCD1:ENST00000239690.4:exon3:c.C446A:p.S149Y,NUDCD1:ENST00000427660.2:exon3:c.C359A:p.S120Y,
8	121219260	G	A	ATGGTTAACT,GACTCATGCC	exonic	COL14A1	stopgain	COL14A1:ENST00000498051.2:exon13:c.G1118A:p.W373X,COL14A1:ENST00000309791.4:exon10:c.G1118A:p.W373X,COL14A1:ENST00000537875.1:exon11:c.G1118A:p.W373X,COL14A1:ENST00000247781.3:exon8:c.G833A:p.W278X,COL14A1:ENST00000434620.1:exon5:c.G55
8	124265639	C	T	TCTGTTTCTT,TTCATCTCTCT	exonic	ZHX1	nonsynonymous SNV	ZHX1:ENST00000395571.3:exon3:c.G2548A:p.E850K,ZHX1:ENST00000522655.1:exon3:c.G2548A:p.E850K,ZHX1:ENST00000297857.2:exon3:c.G2548A:p.E850K,
8	125551447	G	T	TTGGCGTCGG,ACCCTACCTG	exonic	NDUFB9	nonsynonymous SNV	NDUFB9:ENST00000518008.1:exon1:c.G20T:p.G7V,NDUFB9:ENST00000517367.1:exon1:c.G20T:p.G7V,NDUFB9:ENST00000276689.3:exon1:c.G20T:p.G7V,NDUFB9:ENST00000522532.1:exon1:c.G20T:p.G7V,
8	133092096	C	G	GGGAGAGGAT,TCAGAGCAGA	exonic	HHLA1	nonsynonymous SNV	HHLA1:ENST00000434736.2:exon12:c.G902C:p.R301T,HHLA1:ENST00000414222.1:exon10:c.G794C:p.R265T,
8	144789524	G	C	TGTCCGCTTT,ATGGCCTGCG	exonic	CCDC166	nonsynonymous SNV	CCDC166:ENST00000542437.1:exon2:c.C660G:p.I220M,
8	144789598	G	A	TGCACGCGCT,ACGCGCCTCG	exonic	CCDC166	stopgain	CCDC166:ENST00000542437.1:exon2:c.C586T:p.Q196X,
8	144789600	C	G	CACGCGCTGA,GCGCCTCGCG	exonic	CCDC166	nonsynonymous SNV	CCDC166:ENST00000542437.1:exon2:c.G584C:p.R195P,
8	144809389	G	C	CCGCCGCCGG,ATCACGGGCT	exonic	FAM83H	nonsynonymous SNV	FAM83H:ENST00000388913.3:exon5:c.C2242G:p.P748A,
8	144996365	C	G	CGTTCCTGCT,CATCTGCTGC	exonic	PLEC	nonsynonymous SNV	PLEC:ENST00000322810.4:exon32:c.G8035C:p.E2679Q,PLEC:ENST00000354589.3:exon32:c.G7624C:p.E2542Q,PLEC:ENST00000357649.2:exon32:c.G7636C:p.E2546Q,PLEC:ENST00000398774.2:exon32:c.G7528C:p.E2510Q,PLEC:ENST00000345136.3:exon32:c.G7624C:p.E254:
8	145004664	G	C	GGCCAGGCCT,AGAGGTGGCC	exonic	PLEC	stopgain	PLEC:ENST00000322810.4:exon20:c.C2756G:p.S919X,PLEC:ENST00000354589.3:exon20:c.C2345G:p.S782X,PLEC:ENST00000357649.2:exon20:c.C2357G:p.S786X,PLEC:ENST00000398774.2:exon20:c.C2249G:p.S750X,PLEC:ENST00000345136.3:exon20:c.C2345G:p.S782X,PLE
8	145694661	G	A	CAGGTGTCCT,GGCCTTGGGG	exonic	KIFC2	stopgain	KIFC2:ENST00000301331.5:exon7:c.G365A:p.W122X,KIFC2:ENST00000301332.2:exon11:c.G1121A:p.W374X,
8	145999601	G	A	TTCCCAGTAG,AATTTTCTGA	exonic	ZNF34	nonsynonymous SNV	ZNF34:ENST00000343459.4:exon6:c.C733T:p.P245S,ZNF34:ENST00000534337.1:exon4:c.C550T:p.P184S,ZNF34:ENST00000429371.2:exon6:c.C670T:p.P224S,
8	146067504	G	A	TGGTTGTCGT,AGTGTGGGAA	exonic	ZNF7	nonsynonymous SNV	ZNF7:ENST00000325241.6:exon5:c.G1012A:p.E338K,ZNF7:ENST00000446747.2:exon5:c.G1045A:p.E349K,ZNF7:ENST00000544249.1:exon4:c.G724A:p.E242K,ZNF7:ENST00000528372.1:exon5:c.G1012A:p.E338K,
9	4841254	C	G	TGTGTCACCT,AGATGGCGAA	exonic	RCL1	nonsynonymous SNV	RCL1:ENST00000448872.2:exon2:c.C49G:p.Q17E,RCL1:ENST00000441844.1:exon2:c.C49G:p.Q17E,RCL1:ENST00000381728.1:exon2:c.C49G:p.Q17E,RCL1:ENST00000381750.4:exon6:c.C607G:p.Q203E,RCL1:ENST00000381730.1:exon2:c.C49G:p.Q17E,RCL1:ENST00000442869
9	14819230	A	[illegible]	AAATGTTCTA,CCTAACTTTT	splicing	FREM1(ENST00000380881.4:exon16:c.2549+2T>[illegible]	[illegible]	[illegible]

9	73442829	A	T	TCTGCTCCAT,TTTTCCAGTG	exonic	TRPM3	nonsynonymous SNV	TRPM3:ENST00000396283.1:exon6:c.T448A:p.Y150N,TRPM3:ENST00000377111.2:exon6:c.T907A:p.Y303N,TRPM3:ENST00000360823.2:exon6:c.T448A:p.Y150N,TRPM3:ENST00000377110.3:exon6:c.T907A:p.Y303N,TRPM3:ENST00000377101.1:exon6:c.T448A:p.Y150N,TRPM3:E
9	74332946	G	C	CTGTCAATTA,AGCAGCAACA	exonic	TMEM2	nonsynonymous SNV	TMEM2:ENST00000377044.4:exon13:c.C2317G:p.L773V,TMEM2:ENST00000377066.5:exon12:c.C2128G:p.L710V,
9	78547391	G	A	CATTTCAATG,AACCAAAGGT	exonic	PCSK5	nonsynonymous SNV	PCSK5:ENST00000376752.4:exon2:c.G289A:p.E97K,PCSK5:ENST00000376767.3:exon2:c.G289A:p.E97K,PCSK5:ENST00000545128.1:exon2:c.G289A:p.E97K,
9	78965768	C	T	AATTGTTTAT,CTGTGTGTGG	exonic	PCSK5	nonsynonymous SNV	PCSK5:ENST00000545128.1:exon35:c.C4010T:p.S1337F,PCSK5:ENST00000424854.2:exon29:c.C4010T:p.S1337F,
9	79936112	G	T	AATGGCCATT,TTGAGTCAGA	exonic	VPS13A	nonsynonymous SNV	VPS13A:ENST00000376634.4:exon43:c.G5443T:p.V1815F,VPS13A:ENST00000360280.3:exon43:c.G5443T:p.V1815F,VPS13A:ENST00000357409.5:exon43:c.G5443T:p.V1815F,VPS13A:ENST00000376636.3:exon42:c.G5326T:p.V1776F,
9	91947824	G	A	GCTTTTTAGG,TGAAATAGTG	exonic	SECISBP2	nonsynonymous SNV	SECISBP2:ENST00000375807.3:exon6:c.G803A:p.G268D,SECISBP2:ENST00000339901.4:exon6:c.G584A:p.G195D,SECISBP2:ENST00000534113.2:exon6:c.G599A:p.G200D,SECISBP2:ENST00000425851.1:exon3:c.G308A:p.G103D,
9	94172347	C	G	CTCTCTAATT,CATCGGCTCT	exonic	NFIL3	nonsynonymous SNV	NFIL3:ENST00000297689.3:exon2:c.G670C:p.E224Q,
9	101536352	C	G	GAGGGGAGCT,CGTTTCGAAG	exonic	ANKS6	nonsynonymous SNV	ANKS6:ENST00000353234.4:exon9:c.G1628C:p.G543A,ANKS6:ENST00000375019.2:exon8:c.G725C:p.G242A,ANKS6:ENST00000540940.1:exon8:c.G1043C:p.G348A,ANKS6:ENST00000375018.1:exon9:c.G1628C:p.G543A,
9	101778331	C	T	GGTGAAGAGT,CGGCAGCCCT	exonic	COL15A1	nonsynonymous SNV	COL15A1:ENST00000375001.3:exon11:c.C1577T:p.S526F,
9	107550726	G	C	TTCTTTCTCT,GGACTCCTCT	exonic	ABCA1	nonsynonymous SNV	ABCA1:ENST00000374736.3:exon45:c.C6050G:p.P2017R,
9	107553269	G	A	CTTGAAAGTT,ATGATTTTCC	exonic	ABCA1	nonsynonymous SNV	ABCA1:ENST00000374736.3:exon44:c.C5861T:p.S1954L,
9	109690200	C	T	CGGAGGCATC,AGAACACTAT	exonic	ZNF462	nonsynonymous SNV	ZNF462:ENST00000441147.2:exon1:c.C542T:p.P181L,ZNF462:ENST00000374686.2:exon1:c.C656T:p.P219L,ZNF462:ENST00000277225.5:exon3:c.C4007T:p.P1336L,ZNF462:ENST00000457913.1:exon3:c.C4007T:p.P1336L,
9	116812241	C	T	CCAGGCTACC,CTGATCTCTT	exonic	ZNF618	nonsynonymous SNV	ZNF618:ENST00000288466.7:exon14:c.C2380T:p.P794S,ZNF618:ENST00000374126.5:exon15:c.C2659T:p.P887S,
9	117815101	C	G	AGCCAGTGTT,TCCACCAAGC	exonic	TNC	nonsynonymous SNV	TNC:ENST00000350763.4:exon1:c.G3681C:p.E1227D,
9	123724985	G	T	AAAGTGGCAG,ACTGAGAAAC	exonic	C5	nonsynonymous SNV	C5:ENST00000223642.1:exon36:c.C4468A:p.P1490T,
9	125827835	G	C	ATTTGTATTT,ATGTTCAAGA	exonic	RABGAP1	stoploss	RABGAP1:ENST00000456584.1:exon15:c.G1799C:p.X600S,
9	129796770	G	A	TAACGTTGTG,CCTTTACCCG	exonic	RALGPS1	nonsynonymous SNV	RALGPS1:ENST00000259351.5:exon6:c.G277A:p.A93T,RALGPS1:ENST00000373434.1:exon4:c.G277A:p.A93T,RALGPS1:ENST00000373436.1:exon5:c.G277A:p.A93T,RALGPS1:ENST00000424082.2:exon5:c.G277A:p.A93T,RALGPS1:ENST00000394022.3:exon5:c.G277A:p.A93T,R
9	129979233	G	C	CCACGAGGTG,ACCACCTGGA	exonic	RALGPS1	nonsynonymous SNV	RALGPS1:ENST00000373434.1:exon17:c.G1543C:p.D515H,RALGPS1:ENST00000424082.2:exon18:c.G1567C:p.D523H,
9	130217331	G	C	TGAGCTCTCA,AGGTAAACTG	exonic	LRSAM1	nonsynonymous SNV	LRSAM1:ENST00000373322.1:exon3:c.G127C:p.E43Q,LRSAM1:ENST00000300417.6:exon4:c.G127C:p.E43Q,LRSAM1:ENST00000373324.4:exon4:c.G127C:p.E43Q,LRSAM1:ENST00000323301.4:exon3:c.G127C:p.E43Q,
9	130710467	G	A	GAGGAATCCT,GCCTGGGATG	exonic	FAM102A	stopgain	FAM102A:ENST00000373084.4:exon3:c.C73T:p.Q25X,FAM102A:ENST00000373095.1:exon6:c.C499T:p.Q167X,
9	130942729	A	C	CGGACGCCTC,CAAGGCTCAG	exonic	CIZ1	nonsynonymous SNV	CIZ1:ENST00000372938.5:exon7:c.T756G:p.C252W,CIZ1:ENST00000277465.4:exon6:c.T756G:p.C252W,CIZ1:ENST00000538431.1:exon7:c.T756G:p.C252W,CIZ1:ENST00000357558.5:exon7:c.T756G:p.C252W,CIZ1:ENST00000372948.3:exon7:c.T756G:p.C252W,CIZ1:ENST00000
9	131584722	G	T	TGGGCAAATT,AGCTCCGCAC	exonic	ENDOG	stopgain	ENDOG:ENST00000372642.4:exon3:c.G727T:p.E243X,
9	131765229	G	A	GACTTCGTGG,TGTCCACCAG	exonic	NUP188	nonsynonymous SNV	NUP188:ENST00000372577.2:exon37:c.G4271A:p.G1424D,
9	133805147	G	T	CTGGTGCTCT,TCAGCGCCTG	exonic	FIBCD1	nonsynonymous SNV	FIBCD1:ENST00000448616.1:exon3:c.C359A:p.T120K,FIBCD1:ENST00000451466.1:exon3:c.C359A:p.T120K,FIBCD1:ENST00000372338.4:exon2:c.C359A:p.T120K,
9	133987017	G	C	CAGTGATGAA,AGAACCTTCC	exonic	AIF1L	nonsynonymous SNV	AIF1L:ENST00000372312.3:exon2:c.G142C:p.E48Q,AIF1L:ENST00000372298.1:exon3:c.G127C:p.E43Q,AIF1L:ENST00000372300.1:exon3:c.G127C:p.E43Q,AIF1L:ENST00000372302.1:exon3:c.G127C:p.E43Q,AIF1L:ENST00000247291.3:exon3:c.G127C:p.E43Q,AIF1L:ENST000003
9	136536765	G	A	GACGCCTTTG,AATGTGCAGC	exonic	SARDH	nonsynonymous SNV	SARDH:ENST00000371868.1:exon6:c.C502T:p.P168S,SARDH:ENST00000371872.4:exon6:c.C2218T:p.P740S,SARDH:ENST00000422262.2:exon17:c.C1714T:p.P572S,SARDH:ENST00000439388.1:exon18:c.C2218T:p.P740S,
9	136555541	G	C	CAGGTCCTCG,AGCTGTCGAT	exonic	SARDH	nonsynonymous SNV	SARDH:ENST00000371868.1:exon4:c.C314G:p.S105C,SARDH:ENST00000371872.4:exon16:c.C2030G:p.S677C,SARDH:ENST00000422262.2:exon15:c.C1526G:p.S509C,SARDH:ENST00000439388.1:exon16:c.C2030G:p.S677C,
9	139618005	G	A	GCAGTGCAAG,GCGACCTCAT	exonic	FAM69B	nonsynonymous SNV	FAM69B:ENST00000371691.1:exon3:c.G814A:p.G272S,FAM69B:ENST00000371692.4:exon5:c.G1075A:p.G359S,
9	139618006	G	A	CAGTGCAAGG,CGACCTCATC	exonic	FAM69B	nonsynonymous SNV	FAM69B:ENST00000371691.1:exon3:c.G815A:p.G272D,FAM69B:ENST00000371692.4:exon5:c.G1076A:p.G359D,
9	139658367	C	T	CTCTGTGGGC,TGATGGCCCT	exonic	LCN15	nonsynonymous SNV	LCN15:ENST00000316144.5:exon2:c.G191A:p.R64K,
10	12077131	G	C	TCTTCCTCTT,ATGTTTCTTT	exonic	UPF2	nonsynonymous SNV	UPF2:ENST00000356352.2:exon1:c.C292G:p.Q98E,UPF2:ENST00000397053.2:exon2:c.C292G:p.Q98E,UPF2:ENST00000357604.5:exon2:c.C292G:p.Q98E,
10	12206295	G	A	TGCGTTTGGC,GTTTGTGCAT	exonic	SEC61A2	nonsynonymous SNV	SEC61A2:ENST00000298428.9:exon12:c.G1273A:p.G425S,SEC61A2:ENST00000379033.3:exon11:c.G1207A:p.G403S,SEC61A2:ENST00000379020.4:exon11:c.G1075A:p.G359S,
10	12206296	G	A	GCGTTTGGCG,TTTGTGCATT	exonic	SEC61A2	nonsynonymous SNV	SEC61A2:ENST00000298428.9:exon12:c.G1274A:p.G425D,SEC61A2:ENST00000379033.3:exon11:c.G1208A:p.G403D,SEC61A2:ENST00000379020.4:exon11:c.G1076A:p.G359D,
10	13698641	G	A	CTCACCTGAC,TGGCCTTGCA	exonic	FRMD4A	nonsynonymous SNV	FRMD4A:ENST00000358621.4:exon20:c.C2903T:p.T968M,FRMD4A:ENST00000357447.2:exon22:c.C2948T:p.T983M,FRMD4A:ENST00000378503.1:exon21:c.C2948T:p.T983M,
10	13698642	T	A	TCACCTGACG,GGCCTTGCAC	exonic	FRMD4A	nonsynonymous SNV	FRMD4A:ENST00000358621.4:exon20:c.A2902T:p.T968S,FRMD4A:ENST00000357447.2:exon22:c.A2947T:p.T983S,FRMD4A:ENST00000378503.1:exon21:c.A2947T:p.T983S,
10	13717030	C	G	TGCGAGCTAT,TGATTCCTGA	exonic	FRMD4A	nonsynonymous SNV	FRMD4A:ENST00000358621.4:exon14:c.G1087C:p.D363H,FRMD4A:ENST00000264546.6:exon15:c.G1231C:p.D411H,FRMD4A:ENST00000357447.2:exon16:c.G1132C:p.D378H,FRMD4A:ENST00000378503.1:exon15:c.G1132C:p.D378H,
10	22002839	C	T	CTCTCTCCCT,AGCTGTGTCA	exonic	MLLT10	nonsynonymous SNV	MLLT10:ENST00000307729.7:exon14:c.C1838T:p.S613L,MLLT10:ENST00000446906.2:exon14:c.C1838T:p.S613L,MLLT10:ENST00000377072.3:exon15:c.C1886T:p.S629L,MLLT10:ENST00000377059.3:exon13:c.C1838T:p.S613L,MLLT10:ENST00000438473.1:exon7:c.C815T:p.S272
10	25288348	C	T	AGAGTTTTTT,CTTTGAATGT	exonic	ENKUR	nonsynonymous SNV	ENKUR:ENST00000331161.4:exon2:c.G205A:p.E69K,ENKUR:ENST00000496261.2:exon2:c.G205A:p.E69K,ENKUR:ENST00000376363.1:exon2:c.G205A:p.E69K,
10	25839938	C	T	AAGCACATTT,GCTGTATTCT	exonic	GPR158	nonsynonymous SNV	GPR158:ENST00000376351.3:exon6:c.C1438T:p.R480C,
10	30316262	C	T	CCGCCACCTT,TTCCACGCGA	exonic	KIAA1462	nonsynonymous SNV	KIAA1462:ENST00000375377.1:exon3:c.G2815A:p.E939K,
10	37488699	A	T	GGTTGAAGAA,ATTCTTGGGA	exonic	ANKRD30A	nonsynonymous SNV	ANKRD30A:ENST00000361713.1:exon30:c.A2593T:p.N865Y,ANKRD30A:ENST00000602533.1:exon30:c.A2593T:p.N865Y,ANKRD30A:ENST00000374660.1:exon36:c.A2950T:p.N984Y,
10	37508022	A	T	ATACCAGGAA,AGGAAAATAA	exonic	ANKRD30A	stopgain	ANKRD30A:ENST00000361713.1:exon34:c.A3214T:p.K1072X,ANKRD30A:ENST00000602533.1:exon34:c.A3214T:p.K1072X,ANKRD30A:ENST00000374660.1:exon40:c.A3571T:p.K1191X,
10	44112365	C	T	TCAGAAAATC,ATACTGGTGA	exonic	ZNF485	nonsynonymous SNV	ZNF485:ENST00000374435.3:exon5:c.C874T:p.H292Y,ZNF485:ENST00000374437.2:exon3:c.C601T:p.H201Y,ZNF485:ENST00000361807.3:exon5:c.C874T:p.H292Y,
10	49400868	C	T	TCTCTGAATC,AAATGAGAGG	exonic	FRMPD2	stopgain	FRMPD2:ENST00000407470.4:exon15:c.G1928A:p.W643X,FRMPD2:ENST00000305531.3:exon14:c.G1949A:p.W650X,FRMPD2:ENST00000374201.3:exon16:c.G2024A:p.W675X,
10	60996243	G	A	TCATTCCTAG,ATGTTCCCAC	exonic	PHYHIPL	nonsynonymous SNV	PHYHIPL:ENST00000373878.3:exon3:c.G226A:p.D76N,PHYHIPL:ENST00000373880.4:exon3:c.G304A:p.D102N,
10	72503874	G	A	GACAAGTGTG,AGTCTGCGGG	exonic	ADAMTS14	nonsynonymous SNV	ADAMTS14:ENST00000373208.1:exon14:c.G2120A:p.G707E,ADAMTS14:ENST00000373207.1:exon14:c.G2111A:p.G704E,
10	72513698	G	A	GGACCGGCCT,AGGCCCGACG	exonic	ADAMTS14	nonsynonymous SNV	ADAMTS14:ENST00000373208.1:exon19:c.G2881A:p.E961K,ADAMTS14:ENST00000373207.1:exon19:c.G2872A:p.E958K,
10	73822556	C	T	TCGCCTGCCT,GCCCTCCTCC	exonic	SPOCK2	nonsynonymous SNV	SPOCK2:ENST00000373109.2:exon11:c.G1237A:p.E413K,SPOCK2:ENST00000317376.4:exon12:c.G1237A:p.E413K,SPOCK2:ENST00000536168.1:exon11:c.G1237A:p.E413K,
10	76789605	A	G	CAGTATCGAG,GCACAACTGA	exonic	KAT6B	nonsynonymous SNV	KAT6B:ENST00000372725.1:exon17:c.A4147G:p.S1383G,KAT6B:ENST00000287239.4:exon18:c.A5302G:p.S1768G,KAT6B:ENST00000372724.1:exon18:c.A5026G:p.S1676G,KAT6B:ENST00000372711.1:exon17:c.A4474G:p.S1492G,KAT6B:ENST00000372714.1:exon17:c.A4147G:p.
10	76789982	G	C	GAATGGGTCA,AGTGATTTTG	exonic	KAT6B	nonsynonymous SNV	KAT6B:ENST00000372725.1:exon17:c.G4524C:p.Q1508H,KAT6B:ENST00000372724.1:exon18:c.G5403C:p.Q1801H,KAT6B:ENST00000287239.4:exon18:c.G5400C:p.Q1800H,KAT6B:ENST00000372711.1:exon17:c.G4851C:p.Q1617H,KAT6B:ENST00000372714.1:exon17:c.G4524C:p.
10	76790470	G	C	ACGATGCAAA,AGGCATGAAC	exonic	KAT6B	nonsynonymous SNV	KAT6B:ENST00000372725.1:exon17:c.G5012C:p.R1671T,KAT6B:ENST00000372724.1:exon18:c.G5891C:p.R1964T,KAT6B:ENST00000287239.4:exon18:c.G5888C:p.R1963T,KAT6B:ENST00000372711.1:exon17:c.G5339C:p.R1780T,KAT6B:ENST00000372714.1:exon17:c.G5012C:p.
10	76790477	G	A	AAAGAGGCAT,AACATGAGTG	exonic	KAT6B	nonsynonymous SNV	KAT6B:ENST00000372725.1:exon17:c.G5019A:p.M1673I,KAT6B:ENST00000372724.1:exon18:c.G5898A:p.M1966I,KAT6B:ENST00000287239.4:exon18:c.G5895A:p.M1965I,KAT6B:ENST00000372711.1:exon17:c.G5346A:p.M1782I,KAT6B:ENST00000372714.1:exon17:c.G5019A:p.M1
10	76790534	G	A	CTGTGAACAT,AACATGAACA	exonic	KAT6B	nonsynonymous SNV	KAT6B:ENST00000372725.1:exon17:c.G5076A:p.M1692I,KAT6B:ENST00000372724.1:exon18:c.G5955A:p.M1985I,KAT6B:ENST00000287239.4:exon18:c.G5952A:p.M1984I,KAT6B:ENST00000372711.1:exon17:c.G5403A:p.M1801I,KAT6B:ENST00000372714.1:exon17:c.G5076A:p.M1
10	78761203	G	A	AACAGAGACA,AAGAAAGTGG	exonic	KCNMA1	nonsynonymous SNV	KCNMA1:ENST00000404771.3:exon19:c.C2228T:p.S743F,KCNMA1:ENST00000354353.5:exon18:c.C2150T:p.S717F,KCNMA1:ENST00000406533.3:exon20:c.C2240T:p.S747F,KCNMA1:ENST00000286628.8:exon19:c.C2228T:p.S743F,KCNMA1:ENST00000372437.1:exon19:c.C2033T
10	78870083	G	A	TTTTCCCATG,GTCCCCTGAA	exonic	KCNMA1	nonsynonymous SNV	KCNMA1:ENST00000404857.1:exon8:c.C979T:p.P327S,KCNMA1:ENST00000372440.1:exon9:c.C979T:p.P327S,KCNMA1:ENST00000286627.5:exon8:c.C979T:p.P327S,KCNMA1:ENST00000354353.5:exon8:c.C979T:p.P327S,KCNMA1:ENST00000404771.3:exon8:c.C979T:p.P327S,K
10	86131740	C	G	GGTGTAACTT,TACTTTAGGT	exonic	CCSER2	nonsynonymous SNV	CCSER2:ENST00000359979.4:exon2:c.C932G:p.S311C,CCSER2:ENST00000372088.2:exon2:c.C932G:p.S311C,CCSER2:ENST00000224756.8:exon2:c.C932G:p.S311C,
10	91162456	G	C	TCCAGAAATA,ACTGTGAGGA	exonic	IFIT1	nonsynonymous SNV	IFIT1:ENST00000371804.3:exon2:c.G424C:p.D142H,IFIT1:ENST00000546318.1:exon2:c.G331C:p.D111H,
10	93011081	G	T	ACCGAAAGTA,ATGAAGAAGG	exonic	PCGF5	nonsynonymous SNV	PCGF5:ENST00000336126.5:exon6:c.G358T:p.D120Y,PCGF5:ENST00000543648.1:exon6:c.G358T:p.D120Y,
10	94274787	G	A	AGTCTCCAGA,TATATTTGTT	exonic	IDE	nonsynonymous SNV	IDE:ENST00000265986.6:exon5:c.C674T:p.T225I,IDE:ENST00000371581.5:exon11:c.C632T:p.T211I,
10	96163219	G	C	GCCGCGGTCA,AGCGCCCGCG	exonic	TBC1D12	nonsynonymous SNV	TBC1D12:ENST00000225235.4:exon1:c.G849C:p.Q283H,
10	96535202	G	A	TCTCCCTCAT,ACGCTGCGGA	exonic	CYP2C19	nonsynonymous SNV	CYP2C19:ENST00000371321.3:exon3:c.G387A:p.M129I,
10	96541593	C	T	CAATAATTTT,CCACTATCAT	exonic	CYP2C19	nonsynonymous SNV	CYP2C19:ENST00000371321.3:exon5:c.C658T:p.P220S,
10	96827307	G	C	GTAATTCTTT,AGATATTGGG	exonic	CYP2C8	nonsynonymous SNV	CYP2C8:ENST00000371270.3:exon2:c.C310G:p.Q104E,CYP2C8:ENST00000527420.1:exon2:c.C310G:p.Q104E,
10	97763986	T	C	TTTGATGAAA,GATGACTGAA	exonic	CC2D2B	nonsynonymous SNV	CC2D2B:ENST00000410012.2:exon3:c.T2C:p.M1T,CC2D2B:ENST00000371198.2:exon11:c.T275C:p.M92T,CC2D2B:ENST00000424464.1:exon3:c.T2C:p.M1T,CC2D2B:ENST00000344386.3:exon2:c.T2C:p.M1T,
10	98079055	C	T	CAACCCAGGC,CCCCGAAGAC	exonic	DNTT	nonsynonymous SNV	DNTT:ENST00000349175.1:exon3:c.C415T:p.P139S,DNTT:ENST00000371174.2:exon3:c.C415T:p.P139S,
10	99221790	C	T	TGGCTGGAAT,TGCTCGGGAA	exonic	MMS19	nonsynonymous SNV	MMS19:ENST00000327238.7:exon19:c.G1805A:p.R602K,MMS19:ENST00000355839.6:exon20:c.G1970A:p.R657K,MMS19:ENST00000370782.2:exon22:c.G2099A:p.R700K,MMS19:ENST00000438925.2:exon21:c.G2099A:p.R700K,MMS19:ENST00000327277.7:exon21:c.G1007A:p.R
10	99236670	C	A	TTTTCCCCAT,CATCACCTGG	exonic	MMS19	nonsynonymous SNV	MMS19:ENST00000327238.7:exon7:c.G544T:p.D182Y,MMS19:ENST00000439048.1:exon7:c.G500T:p.G167V,MMS19:ENST00000437002.1:exon7:c.G544T:p.D182Y,MMS19:ENST00000422685.1:exon8:c.G661T:p.D221Y,MMS19:ENST00000370782.2:exon8:c.G544T:p.D182Y,MMS1
10	102762429	C	T	CCAGCTCCTC,CCGCCACCAC	exonic	LZTS2	nonsynonymous SNV	LZTS2:ENST00000465680.1:exon2:c.C134T:p.P45L,LZTS2:ENST00000370223.3:exon2:c.C134T:p.P45L,LZTS2:ENST00000544422.1:exon2:c.C134T:p.P45L,LZTS2:ENST00000481129.1:exon2:c.C134T:p.P45L,LZTS2:ENST00000429732.1:exon2:c.C134T:p.P45L,LZTS2:ENST00000
10	103826243	G	A	GCTGGACATG,GCAGTGGGCA	exonic	HPS6	nonsynonymous SNV	HPS6:ENST00000299238.5:exon1:c.G1012A:p.G338S,
10	104160997	C	G	CCCCCTACCT,TGATAGCGAC	exonic	NFKB2	nonsynonymous SNV	NFKB2:ENST00000428099.1:exon19:c.C2132G:p.S711C,NFKB2:ENST00000369966.3:exon19:c.C2132G:p.S711C,NFKB2:ENST00000189444.6:exon19:c.C2132G:p.S711C,
10	115534602	C	T	AAAGAGACAT,CCATGAGTCT	exonic	PLEKHS1	nonsynonymous SNV	PLEKHS1:ENST00000369309.3:exon4:c.C239T:p.S80F,PLEKHS1:ENST00000361048.1:exon10:c.C797T:p.S266F,PLEKHS1:ENST00000369310.3:exon9:c.C779T:p.S260F,PLEKHS1:ENST00000369312.4:exon10:c.C533T:p.S178F,PLEKHS1:ENST00000453462.3:exon3:c.C29T:p.S10F,
10	115636441	C	G	CTGGCCAACT,TAGTCATACT	exonic	NHLRC2	nonsynonymous SNV	NHLRC2:ENST00000369301.3:exon3:c.C493G:p.L165V,
10	125434339	G	A	TGCAGTGTGC,GGAACGCTGT	exonic	GPR26	nonsynonymous SNV	GPR26:ENST00000284674.1:exon2:c.G674A:p.R225Q,
10	127418840	G	C	AATAACACTA,AAATGTATAT	splicing	C10orf137(ENST00000481600.1:exon9:c.1017-1G	.	.
10	127542537	C	G	TACCTTTCTT,TTTCCACACC	exonic	DHX32	nonsynonymous SNV	DHX32:ENST00000284690.3:exon4:c.G1085C:p.R362T,
10	129904586	G	A	CATGGTGTCT,GAAAAGCTCT	exonic	MKI67	stopgain	MKI67:ENST00000368653.3:exon12:c.C4438T:p.Q1480X,MKI67:ENST00000368654.3:exon13:c.C5518T:p.Q1840X,
10	129906332	G	A	GTGTCCACTG,GTCTGACTGT	exonic	MKI67	nonsynonymous SNV	MKI67:ENST00000368653.3:exon12:c.C2692T:p.P898S,MKI67:ENST00000368654.3:exon13:c.C3772T:p.P1258S,
10	134011960	G	C	CTGGCCCCGA,GGCCACGTGC	exonic	DPYSL4	nonsynonymous SNV	DPYSL4:ENST00000338492.4:exon7:c.G663C:p.E221D,
10	134896125	T	C	GAGGAGAAGG,ATCTGAGGCC	exonic	GPR123	nonsynonymous SNV	GPR123:ENST00000607359.1:exon6:c.T1211C:p.V404A,
11	380857	G	A	TGGCCTTTTT,GGATCTACAA	exonic	B4GALNT4	nonsynonymous SNV	B4GALNT4:ENST00000329962.6:exon19:c.G2902A:p.G968R,
11	380858	G	A	GGCCTTTTTG,GATCTACAAG	exonic	B4GALNT4	nonsynonymous SNV	B4GALNT4:ENST00000329962.6:exon19:c.G2903A:p.G968E,
11	607644	G	A	CACACGGGCA,AGAGCGAGGC	exonic	PHRF1	nonsynonymous SNV	PHRF1:ENST00000264555.5:exon14:c.G2188A:p.E730K,PHRF1:ENST00000534320.1:exon14:c.G2188A:p.E730K,PHRF1:ENST00000533464.1:exon14:c.G2176A:p.E726K,PHRF1:ENST00000413872.2:exon14:c.G2182A:p.E728K,PHRF1:ENST00000416188.2:exon14:c.G2185A:p.E72
11	607889	G	C	AGAGGAAGGA,AACCCCTCAC	exonic	PHRF1	nonsynonymous SNV	PHRF1:ENST00000264555.5:exon14:c.G2433C:p.E811D,PHRF1:ENST00000534320.1:exon14:c.G2433C:p.E811D,PHRF1:ENST00000533464.1:exon14:c.G2421C:p.E807D,PHRF1:ENST00000413872.2:exon14:c.G2427C:p.E809D,PHRF1:ENST00000416188.2:exon14:c.G2430C:p.E81
11	694942	C	G	TCCTCCGCCT,GCCTCCTGCC	exonic	DEAF1	nonsynonymous SNV	DEAF1:ENST00000382409.3:exon1:c.G106C:p.E36Q,DEAF1:ENST00000338675.6:exon1:c.G106C:p.E36Q,
11	760132	G	C	GCTCTCCTTT,ATAAAGATGC	exonic	TALDO1	stoploss	TALDO1:ENST00000319006.3:exon4:c.G281C:p.X94S,
11	977172	C	T	GATCTTGTCC,CATGGGCGAC	exonic	AP2A2	nonsynonymous SNV	AP2A2:ENST00000525796.1:exon3:c.C71T:p.P24L,AP2A2:ENST00000534485.1:exon5:c.C521T:p.P174L,AP2A2:ENST00000332231.5:exon5:c.C551T:p.P184L,AP2A2:ENST00000534328.1:exon5:c.C551T:p.P184L,AP2A2:ENST00000528815.1:exon5:c.C551T:p.P184L,AP2A2:ENST000
11	1081806	C	G	CCCTGAACAT,GAGAGCGGTG	exonic	MUC2	nonsynonymous SNV	MUC2:ENST00000359061.5:exon13:c.C1734G:p.I578M,MUC2:ENST00000441003.2:exon13:c.C1734G:p.I578M,
11	1157735	G	A	CATCTGTGAG,AGCTCCTGCA	exonic	MUC5AC	nonsynonymous SNV	MUC5AC:ENST00000356191.2:exon13:c.G793A:p.E265K,MUC5AC:ENST00000534821.1:exon8:c.G802A:p.E268K,
11	1651158	G	A	CGGCTGTGGA,GCTGTGGCTC	exonic	KRTAP5-5	nonsynonymous SNV	KRTAP5-5:ENST00000399676.2:exon1:c.G88A:p.G30S,
11	1983485	C	T	CACCCTCGGT,CCTGCCTCTC	exonic	MRPL23	nonsynonymous SNV	MRPL23:ENST00000381514.3:exon5:c.C314T:p.S105F,
11	5153513	C	T	GATCCAGGGC,ATTGCCAGAA	exonic	OR52A5	nonsynonymous SNV	OR52A5:ENST00000307388.1:exon1:c.G360A:p.M120I,
11	6244872	G	A	GCGATGTAGC,GCCCACAGTG	exonic	FAM160A2	nonsynonymous SNV	FAM160A2:ENST00000449352.2:exon3:c.C745T:p.R249C,FAM160A2:ENST00000524416.1:exon3:c.C745T:p.R249C,FAM160A2:ENST00000265978.4:exon3:c.C745T:p.R249C,
11	19970365	G	A	CTCCAAGCAG,AGACGCCCCC	exonic	NAV2	nonsynonymous SNV	NAV2:ENST00000349880.4:exon10:c.G2384A:p.G795E,NAV2:ENST00000360655.4:exon10:c.G2192A:p.G731E,NAV2:ENST00000396087.3:exon11:c.G2453A:p.G818E,NAV2:ENST00000396085.1:exon10:c.G2384A:p.G795E,NAV2:ENST00000527559.2:exon12:c.G2240A:p.G747E,NAV
11	21594765	C	T	CTCACTTGCC,CAACTTGAGC	exonic	NELL1	nonsynonymous SNV	NELL1:ENST00000298925.5:exon20:c.C2276T:p.P759L,NELL1:ENST00000357134.5:exon19:c.C2192T:p.P731L,NELL1:ENST00000325319.5:exon18:c.C2021T:p.P674L,NELL1:ENST00000532434.1:exon18:c.C2051T:p.P684L,
11	30925094	G	A	TTGTTTCTCA,AGTCTCTCTG	exonic	DCDC1	nonsynonymous SNV	DCDC1:ENST00000339794.5:exon13:c.C1682T:p.S561F,DCDC1:ENST00000597505.1:exon30:c.C4445T:p.S1482F,DCDC1:ENST00000406071.2:exon15:c.C1650T:p.S217F,
11	32858302	G	C	CCTAGAAAGA,AGTGCAATGA	exonic	PRRG4	nonsynonymous SNV	PRRG4:ENST00000257836.3:exon2:c.G202C:p.E68Q,
11	33565789	C	T	CTTGGGATAT,ACTCTGCTGC	exonic	KIAA1549L	nonsynonymous SNV	KIAA1549L:ENST00000321505.4:exon11:c.C1789T:p.H597Y,KIAA1549L:ENST00000265654.5:exon11:c.C1789T:p.H597Y,KIAA1549L:ENST00000389726.3:exon11:c.C1789T:p.H597Y,
11	45924510	C	T	GAGCCTGCGG,CGTGCTTCGG	exonic	MAPK8IP1	nonsynonymous SNV	MAPK8IP1:ENST00000395629.2:exon2:c.C1162T:p.R388S,MAPK8IP1:ENST00000241014.2:exon5:c.C1192T:p.R398S,
11	45924511	C	T	AGCCTGCGGC,GTGCTTCGGA	exonic	MAPK8IP1	nonsynonymous SNV	MAPK8IP1:ENST00000395629.2:exon5:c.C1163T:p.S388L,MAPK8IP1:ENST00000241014.2:exon5:c.C1193T:p.S398L,
11	46717606	C	T	TTCAGAGCCT,GCTGGTGTCA	exonic	ARHGAP1	nonsynonymous SNV	ARHGAP1:ENST00000311956.4:exon2:c.G52A:p.E18K,ARHGAP1:ENST00000525488.1:exon2:c.G52A:p.E18K,
11	46797822	G	A	CTCAAGAAAG,ATTTTCCTTT	exonic	CKAP5	nonsynonymous SNV	CKAP5:ENST00000415402.1:exon24:c.C2974T:p.P992S,CKAP5:ENST00000529230.1:exon24:c.C2974T:p.P992S,CKAP5:ENST00000312055.5:exon24:c.C2974T:p.P992S,CKAP5:ENST00000354558.3:exon23:c.C2974T:p.P992S,
11	48510888	T	A	CATGTATCCC,TATTGAAACT	exonic	OR4A47	nonsynonymous SNV	OR4A47:ENST00000446524.1:exon1:c.T544A:p.L182I,
11	55418432	G	A	CTTTCTCAGA,CCCAGAGATT	exonic	OR4S2	nonsynonymous SNV	OR4S2:ENST00000312422.2:exon1:c.G53A:p.S18N,
11	55433415	G	A	TTGTACATGA,GCCTGTGGTC	exonic	OR4C6	nonsynonymous SNV	OR4C6:ENST00000314259.3:exon1:c.G773A:p.R258K,
11	56019823	G	A	CTTCACAGAT,ATTTTGAGCT	exonic	OR5T3	nonsynonymous SNV	OR5T3:ENST00000303039.3:exon1:c.G148A:p.D50N,
11	56510593	G	A	GTTGACATAG,AAATCAGGAT	exonic	OR9G4	nonsynonymous SNV	OR9G4:ENST00000302957.3:exon1:c.C695T:p.S232F,
11	57374016	C	G	ACTTTGAAAG,CAAGGTAAGT	exonic	SERPING1	nonsynonymous SNV	SERPING1:ENST00000378323.4:exon2:c.C869G:p.A290G,SERPING1:ENST00000340687.6:exon6:c.C1025G:p.A342G,SERPING1:ENST00000378324.2:exon6:c.C1040G:p.A347G,SERPING1:ENST00000528996.1:exon1:c.C137G:p.A46G,SERPING1:ENST00000278407.4:exon6:c.C10
11	58980167	C	T	CGTCCCATGT,CACATTCCGC	exonic	MPEG1	nonsynonymous SNV	MPEG1:ENST00000361050.3:exon1:c.G172A:p.D58N,
11	59856269	C	T	GAGAGCAAAT,TTGCTCTCCC	exonic	MS4A2	nonsynonymous SNV	MS4A2:ENST00000524868.1:exon2:c.C31T:p.L11F,MS4A2:ENST00000278888.3:exon1:c.C31T:p.L11F,
11	62284883	G	A	CTCACGGCCA,AGAAGGTAAA	exonic	AHNAK	nonsynonymous SNV	AHNAK:ENST00000378024.4:exon5:c.C17006T:p.S5669F,
11	62284977	G	A	CTCAGCCCAG,AGCCTGGAGT	exonic	AHNAK	nonsynonymous SNV	AHNAK:ENST00000378024.4:exon5:c.C16912T:p.P5638S,
11	62288347	G	C	GTGGGCTTTT,AAATGTACAT	exonic	AHNAK	nonsynonymous SNV	AHNAK:ENST00000378024.4:exon5:c.C13542G:p.F4514L,
11	62370332	C	G	GAGTTGATGT,GGTCCCATCG	exonic	EML3	nonsynonymous SNV	EML3:ENST00000278845.4:exon21:c.G2389C:p.D797H,EML3:ENST00000394773.2:exon21:c.G2386C:p.D796H,EML3:ENST00000531557.1:exon18:c.G1735C:p.D579H,
11	64022488	C	A	AACTTCATGG,CGTGCAGGAT	exonic	PLCB3	nonsynonymous SNV	PLCB3:ENST00000540288.1:exon4:c.C365A:p.A122D,PLCB3:ENST00000325234.5:exon2:c.C164A:p.A55D,PLCB3:ENST00000279230.6:exon4:c.C365A:p.A122D,
11	65346836	C	T	GCCGGGCATC,AGAACCCATA	exonic	EHBP1L1	stopgain	EHBP1L1:ENST00000309295.4:exon3:c.C187T:p.Q63X,EHBP1L1:ENST00000533237.1:exon3:c.C187T:p.Q63X,
11	66358302	G	A	TCCCGCAGCT,AATGGGCTTC	exonic	CCDC87	stopgain	CCDC87:ENST00000333861.3:exon1:c.C2185T:p.Q729X,
11	67925186	C	T	CTGATCTTCT,TTCTCCTTGA	exonic	SUV420H1	nonsynonymous SNV	SUV420H1:ENST00000304363.4:exon11:c.G2627A:p.R876K,
11	74800493	A	G	AAGCAAGAAT,AGGACAGCAT	exonic	OR2AT4	nonsynonymous SNV	OR2AT4:ENST00000305159.3:exon1:c.T266C:p.L89S,
11	75851783	C	T	CAGTGCAATC,CTGTTCCTAA	exonic	UVRAG	nonsynonymous SNV	UVRAG:ENST00000531818.1:exon9:c.C310T:p.P104S,UVRAG:ENST00000356136.3:exon15:c.C1426T:p.P476S,UVRAG:ENST00000538870.1:exon3:c.C97T:p.P32S,UVRAG:ENST00000539288.1:exon9:c.C310T:p.P104S,UVRAG:ENST00000528420.1:exon15:c.C1123T:p.P375S,UVRA
11	92616244	C	T	CAATGGCATC,CGTTCCGGAA	exonic	FAT3	nonsynonymous SNV	FAT3:ENST00000533797.1:exon6:c.C1627T:p.P543S,FAT3:ENST00000298047.6:exon23:c.C12622T:p.P4208S,FAT3:ENST00000409404.2:exon23:c.C12622T:p.P4208S,FAT3:ENST00000525166.1:exon23:c.C12172T:p.P4058S,
11	94320221	C	T	TAAAGATTAT,CCTTTGGCCT	exonic	PIWIL4	nonsynonymous SNV	PIWIL4:ENST00000446230.2:exon7:c.C641T:p.S214F,PIWIL4:ENST00000543336.1:exon7:c.C515T:p.S172F,PIWIL4:ENST00000299001.6:exon7:c.C722T:p.S241F,
11	100847594	C	T	TCTGTAGGTT,CAAGGAGACA	exonic	ARHGAP42	nonsynonymous SNV	ARHGAP42:ENST00000298815.8:exon21:c.C2285T:p.S762F,ARHGAP42:ENST00000524892.2:exon20:c.C2183T:p.S728F,
11	102195958	C	G	AAATCAGCTT,AAGACACTTC	exonic	BIRC3	nonsynonymous SNV	BIRC3:ENST00000532808.1:exon3:c.C718G:p.Q240E,BIRC3:ENST00000527309.1:exon1:c.C7G:p.Q3E,BIRC3:ENST00000263464.3:exon2:c.C718G:p.Q240E,
11	108079299	G	A	TGGGCAACAG,GTGAGACCCT	exonic	NPAT	nonsynonymous SNV	NPAT:ENST00000351384.1:exon2:c.C50T:p.P17L,
11	108079300	G	A	GGGCAACAGG,TGAGACCCTG	exonic	NPAT	nonsynonymous SNV	NPAT:ENST00000351384.1:exon2:c.C49T:p.P17S,
11	114270762	C	T	AGATCGGGTT,AACGGCAGGG	exonic	C11orf71	nonsynonymous SNV	C11orf71:ENST00000325636.4:exon1:c.G292A:p.E98K,
11	119058067	G	A	GCTCAGCTGG,AGAAGATGCC	exonic	PDZD3	nonsynonymous SNV	PDZD3:ENST00000525131.1:exon5:c.G380A:p.G127E,PDZD3:ENST00000355547.5:exon5:c.G419A:p.G140E,PDZD3:ENST00000392817.2:exon2:c.G617A:p.G206E,PDZD3:ENST00000531114.1:exon3:c.G617A:p.G206E,PDZD3:ENST00000322712.4:exon5:c.G419A:p.G140E,
11	121037337	G	A	AGCCCAAATG,AAGTGTCCAT	exonic	TECTA	nonsynonymous SNV	TECTA:ENST00000264037.2:exon17:c.G5434A:p.E1812K,TECTA:ENST00000392793.1:exon18:c.G5434A:p.E1812K,
12	6127612	G	A	GCCTCTCGGG,GAGCGTCTCA	exonic	VWF	nonsynonymous SNV	VWF:ENST00000261405.5:exon28:c.C4972T:p.P1658S,
12	6457151	G	A	GGCTGGAGAG,GAGCAGGGCC	exonic	SCNN1A	nonsynonymous SNV	SCNN1A:ENST00000228916.2:exon13:c.C1898T:p.P633L,SCNN1A:ENST00000358945.3:exon13:c.C1964T:p.P655L,SCNN1A:ENST00000360168.3:exon12:c.C2075T:p.P692L,SCNN1A:ENST00000543768.1:exon13:c.C1967T:p.P656L,SCNN1A:ENST00000540037.1:exon11:c.C998T:p
12	6658937	C	T	ACATCTGGAT,ATGTCCTCGC	exonic	IFFO1	nonsynonymous SNV	IFFO1:ENST00000436152.2:exon5:c.G135A:p.M45I,IFFO1:ENST00000396840.2:exon4:c.G1056A:p.M352I,IFFO1:ENST00000465801.1:exon5:c.G135A:p.M45I,IFFO1:ENST00000336604.4:exon4:c.G1056A:p.M352I,IFFO1:ENST00000356896.4:exon4:c.G1056A:p.M352I,
12	6923370	C	T	AGGAACTTT,CCCTGATCAT	exonic	CD4	nonsynonymous SNV	CD4:ENST00000541982.1:exon3:c.C112T:p.P38S,CD4:ENST00000011653.4:exon4:c.C277T:p.P93S,
12	7459334	G	A	GCTTGCATAC,AACAGGTCAG	exonic	ACSM4	nonsynonymous SNV	ACSM4:ENST00000399422.4:exon2:c.G407A:p.R136Q,
12	7528485	A	C	AATTCTCTGC,CAGCACATTG	exonic	CD163L1	nonsynonymous SNV	CD163L1:ENST00000416109.2:exon10:c.T2527G:p.C843G,CD163L1:ENST00000396630.1:exon10:c.T2497G:p.C833G,CD163L1:ENST00000313599.3:exon10:c.T2497G:p.C833G,
12	7805431	C	T	CGATTCTTCT,CTGAAATACA	exonic	APOBEC1	nonsynonymous SNV	APOBEC1:ENST00000467171.2:exon2:c.G17A:p.G6E,
12	8757459	C	T	TGCAGCCCTT,CCAGGCTTTG	exonic	AICDA	nonsynonymous SNV	AICDA:ENST00000229335.6:exon4:c.G487A:p.E163K,AICDA:ENST00000537228.1:exon4:c.G457A:p.E153K,
12	12022610	C	T	TCAGTGTCTC,CATGGAGAAT	exonic	ETV6	nonsynonymous SNV	ETV6:ENST00000396373.4:exon5:c.C716T:p.P239L,
12	13724763	C	T	AGCAATGCAT,ATCTACACCC	exonic	GRIN2B	nonsynonymous SNV	GRIN2B:ENST00000609686.1:exon10:c.G2146A:p.D716N,
12	20858999	C	T	TGCAATGCCT,AGTTCTTCAT	exonic	SLCO1C1	stopgain	SLCO1C1:ENST00000545102.1:exon2:c.C34T:p.Q12X,SLCO1C1:ENST00000266509.2:exon4:c.C388T:p.Q130X,SLCO1C1:ENST00000540229.1:exon6:c.C388T:p.Q130X,SLCO1C1:ENST00000381552.1:exon4:c.C388T:p.Q130X,SLCO1C1:ENST00000540354.1:exon5:c.C388T:p.Q130
12	21036407	C	G	AGAAATTACT,AGCACACTTG	exonic	LST3,SLCO1B3	stopgain	SLCO1B3:ENST00000261196.2:exon12:c.C1553G:p.S518X,SLCO1B3:ENST00000553473.1:exon11:c.C1553G:p.S518X,SLCO1B3:ENST00000381545.3:exon13:c.C1553G:p.S518X,LST3:ENST00000540229.1:exon11:c.C1553G:p.S518X,LST3:ENST00000544370.1:exon7:c.C1025G:
12	21207494	G	C	GAAATCTTAC,TTTATTTTGT	exonic	LST3,SLCO1B7	nonsynonymous SNV	SLCO1B7:ENST00000421593.2:exon10:c.G1466C:p.V489L,SLCO1B7:ENST00000554957.1:exon11:c.G1606C:p.V536L,LST3:ENST00000381541.3:exon11:c.G1606C:p.V536L,
12	21392010	G	A	TCAAGAGAAA,ATATCAATGC	exonic	SLCO1B1	nonsynonymous SNV	SLCO1B1:ENST00000256958.2:exon15:c.G1963A:p.D655N,
12	25232596	G	A	ACAGATCCTG,ATTAGAAATT	exonic	LRMP	nonsynonymous SNV	LRMP:ENST00000547044.1:exon4:c.G143A:p.G48E,LRMP:ENST00000557489.1:exon9:c.G143A:p.G48E,LRMP:ENST00000548766.1:exon7:c.G143A:p.G48E,LRMP:ENST00000361433.2:exon8:c.G143A:p.G48E,LRMP:ENST00000550945.1:exon8:c.G143A:p.G48E,LRMP:ENST00000
12	25362828	G	C	CTAATGTATA,AAGGCATCAT	exonic	KRAS	nonsynonymous SNV	KRAS:ENST00000557334.1:exon3:c.C170G:p.F43L,KRAS:ENST00000311936.3:exon5:c.C468G:p.F156L,
12	30888083	C	T	TTTTTCTTCT,AGCCTCAAGT	exonic	CAPRIN2	nonsynonymous SNV	CAPRIN2:ENST00000541765.1:exon6:c.G19A:p.E7K,CAPRIN2:ENST00000548676.1:exon3:c.G19A:p.E7K,CAPRIN2:ENST00000417045.1:exon4:c.G628A:p.E210K,CAPRIN2:ENST00000543380.1:exon4:c.G19A:p.E7K,CAPRIN2:ENST00000298892.5:exon4:c.G628A:p.E210K,CAPRI
12	51685938	C	G	ATTTCCTCCT,TTCTAAGAGC	exonic	BIN2	nonsynonymous SNV	BIN2:ENST00000452142.2:exon9:c.G856C:p.E286Q,BIN2:ENST00000544402.1:exon10:c.G874C:p.E292Q,BIN2:ENST00000267012.4:exon10:c.G952C:p.E318Q,BIN2:ENST00000604560.1:exon9:c.G871C:p.E291Q,
12	52822125	C	G	TCCTGGTACT,ACGCAGGAGC	exonic	KRT75	nonsynonymous SNV	KRT75:ENST00000252245.5:exon7:c.G1297C:p.E433Q,
12	52822233	C	G	TGCTCTGCAT,AGCAATGGCC	exonic	KRT75	nonsynonymous SNV	KRT75:ENST00000252245.5:exon7:c.G1189C:p.D397H,
12	54339012	C	T	GTCAGCAAAT,GAAAGCGCCT	exonic	HOXC13	nonsynonymous SNV	HOXC13:ENST00000243056.3:exon2:c.C965T:p.S322L,
12	55420767	C	G	TCAGTCTGTC,TCCTGGAGAA	exonic	NEUROD4	nonsynonymous SNV	NEUROD4:ENST00000242994.3:exon2:c.C544G:p.L182V,
12	55688235	G	C	TTCTTTTGCA,ATGGTTTAAT	exonic	OR6C6	nonsynonymous SNV	OR6C6:ENST00000358433.2:exon1:c.C782G:p.S261C,
12	56115082	A	T	GCTGTGACTC,GGCTTTGGGC	exonic;splicing	RDH5;RDH5	synonymous SNV	RDH5:ENST00000257895.5:exon2:c.A114T:p.S38S,RDH5:ENST00000548082.1:exon2:c.A114T:p.S38S,
12	56524860	C	T	TCACCCAGGT,AGAGAAAAGA	exonic	ESYT1	stopgain	ESYT1:ENST00000541590.1:exon4:c.C604T:p.Q202X,ESYT1:ENST00000551790.1:exon1:c.C241T:p.Q81X,ESYT1:ENST00000267113.4:exon4:c.C604T:p.Q202X,ESYT1:ENST00000394048.5:exon4:c.C604T:p.Q202X,
12	56995848	G	A	CCTCGGGCCC,GGCAGGAGAA	exonic	BAZ2A	nonsynonymous SNV	BAZ2A:ENST00000379441.3:exon21:c.G3469T:p.R1157W,BAZ2A:ENST00000549787.1:exon4:c.C367T:p.R123W,BAZ2A:ENST00000549884.1:exon20:c.C3553T:p.R1185W,BAZ2A:ENST00000551812.1:exon20:c.C3559T:p.R1187W,BAZ2A:ENST00000179765.5:exon21:c.C3463T:p.R1
12	57406518	C	T	TCCTGTCTTT,CCTCTGGTGA	exonic	TAC3	nonsynonymous SNV	TAC3:ENST00000393867.1:exon6:c.G296A:p.G99E,TAC3:ENST00000357616.3:exon6:c.G296A:p.G99E,
12	57432779	G	C	ACATGGCCAG,ATACCTCGCT	exonic	MYO1A	nonsynonymous SNV	MYO1A:ENST00000554234.1:exon12:c.C861G:p.I287M,MYO1A:ENST00000300119.3:exon16:c.C1347G:p.I449M,MYO1A:ENST00000442789.2:exon17:c.C1347G:p.I449M,MYO1A:ENST00000544473.1:exon12:c.C861G:p.I287M,
12	66850114	G	A	TCGCTCCTCT,AATTTTCACT	exonic	GRIP1	stopgain	GRIP1:ENST00000359742.4:exon10:c.C1051T:p.Q351X,GRIP1:ENST00000286445.7:exon10:c.C1051T:p.Q351X,
12	69990984	G	C	GTGAAGCTTG,AAGTTGCAAA	exonic	CCT2	nonsynonymous SNV	CCT2:ENST00000550010.1:exon11:c.G1031C:p.G344A,CCT2:ENST00000543146.2:exon11:c.G890C:p.G297A,CCT2:ENST00000299300.6:exon11:c.G1031C:p.G344A,CCT2:ENST00000544368.2:exon11:c.G1031C:p.G344A,
12	73012712	G	T	GAGATTATCA,ATACCTGTCT	exonic	TRHDE	nonsynonymous SNV	TRHDE:ENST00000261180.4:exon13:c.G2228T:p.R743L,
12	77216267	G	A	GCAAGCAAAA,GATATGACAA	exonic	ZDHHC17	nonsynonymous SNV	ZDHHC17:ENST00000426126.2:exon8:c.G853A:p.G285R,ZDHHC17:ENST00000550876.1:exon5:c.G364A:p.G122R,
12	78225435	G	A	TGTGAATTTG,AGAGAAGAAA	exonic	NAV3	nonsynonymous SNV	NAV3:ENST00000266692.7:exon1:c.G194A:p.G65E,NAV3:ENST00000397909.2:exon1:c.G194A:p.G65E,NAV3:ENST00000228327.6:exon1:c.G194A:p.G65E,NAV3:ENST00000549464.1:exon1:c.G194A:p.G65E,NAV3:ENST00000536525.2:exon1:c.G194A:p.G65E,
12	79747310	C	A	ATCTGCTTCT,CCTTCGCTAC	exonic	SYT1	nonsynonymous SNV	SYT1:ENST00000261205.4:exon9:c.C839A:p.S280Y,SYT1:ENST00000393240.3:exon10:c.C839A:p.S280Y,SYT1:ENST00000552744.1:exon8:c.C839A:p.S280Y,SYT1:ENST00000457153.2:exon8:c.C830A:p.S277Y,
12	81528772	G	A	TAGTCGCATT,ATCATGTAAA	exonic	ACSS3	nonsynonymous SNV	ACSS3:ENST00000548058.1:exon13:c.G631A:p.D211N,ACSS3:ENST00000261206.3:exon13:c.G631A:p.D211N,
12	85695041	C	T	TCACTCGCCT,GGACAGATTC	exonic	ALX1	nonsynonymous SNV	ALX1:ENST00000316824.3:exon4:c.C769T:p.R257W,
12	86374104	C	T	ACTGAAATTT,CTTCAGCTCT	exonic	MGAT4C	nonsynonymous SNV	MGAT4C:ENST00000604798.1:exon6:c.G400A:p.E134K,MGAT4C:ENST00000547225.1:exon6:c.G400A:p.E134K,MGAT4C:ENST00000393205.2:exon4:c.G487A:p.E163K,MGAT4C:ENST00000548651.1:exon4:c.G400A:p.E134K,MGAT4C:ENST00000552808.2:exon6:c.G400A:p.E134K,
12	88508256	G	A	TTAACATCAG,ATCTTTCTGC	exonic	CEP290	nonsynonymous SNV	CEP290:ENST00000309041.7:exon20:c.C1999T:p.P667S,CEP290:ENST00000552810.1:exon20:c.C1999T:p.P667S,CEP290:ENST00000604024.1:exon12:c.C1252T:p.P418S,
12	88582639	G	A	GTGCCCATAT,AATGTAGGAA	exonic	TMTC3	nonsynonymous SNV	TMTC3:ENST00000266712.6:exon14:c.G1452A:p.M484I,
12	100774643	C	T	TTCTGCATTT,CTTTGGGATC	exonic	SLC17A8	nonsynonymous SNV	SLC17A8:ENST00000323346.5:exon2:c.C266T:p.S89F,SLC17A8:ENST00000392989.3:exon2:c.C266T:p.S89F,
12	101520688	C	T	CACCTCATTT,ATATCTGATC	exonic	ANO4	nonsynonymous SNV	ANO4:ENST00000392977.3:exon27:c.C2708T:p.S903L,ANO4:ENST00000299222.9:exon26:c.C1268T:p.S423L,ANO4:ENST00000392979.3:exon26:c.C2603T:p.S868L,ANO4:ENST00000550015.1:exon14:c.C1268T:p.S423L,
12	105514945	C	A	TATTGATAAT,ATATCACACT	exonic	KIAA1033	nonsynonymous SNV	KIAA1033:ENST00000332180.5:exon6:c.C628A:p.H210N,
12	106853091	C	T	GACTCAGATT,CTTTGGAAGG	exonic	POLR3B	nonsynonymous SNV	POLR3B:ENST00000539066.1:exon22:c.C2341T:p.P781S,POLR3B:ENST00000228347.4:exon22:c.C2515T:p.P839S,
12	106853092	C	T	ACTCAGATTC,TTTGGAAGGA	exonic	POLR3B	nonsynonymous SNV	POLR3B:ENST00000539066.1:exon22:c.C2342T:p.P781L,POLR3B:ENST00000228347.4:exon22:c.C2516T:p.P839L,
12	111800607	G	A	GCCCGGCGGC,AGGCGGTGGT	exonic	FAM109A	nonsynonymous SNV	FAM109A:ENST00000392658.5:exon3:c.C625T:p.R209C,FAM109A:ENST00000548163.1:exon2:c.C625T:p.R209C,FAM109A:ENST00000361483.3:exon4:c.C664T:p.R222C,FAM109A:ENST00000547838.2:exon2:c.C625T:p.R209C,
12	112600893	T	A	CGGGGGCACA,GGGCAGTGTC	exonic	HECTD4	nonsynonymous SNV	HECTD4:ENST00000377560.5:exon74:c.A12557T:p.H4186L,HECTD4:ENST00000430131.2:exon74:c.A11807T:p.H3936L,HECTD4:ENST00000550722.1:exon75:c.A12635T:p.H4212L,
12	117977608	G	A	GGAGGGAGGG,GGGTGCTGGC	exonic	KSR2	nonsynonymous SNV	KSR2:ENST00000339824.5:exon10:c.C1603T:p.P535S,KSR2:ENST00000425217.1:exon10:c.C1516T:p.P506S,
12	117977622	G	C	TGCTGGCGAG,AGGGCGTGGA	exonic	KSR2	nonsynonymous SNV	KSR2:ENST00000339824.5:exon10:c.C1589G:p.S530C,KSR2:ENST00000425217.1:exon10:c.C1502G:p.S501C,
12	119583277	G	A	TACGACTCAG,AAATGACACG	exonic	SRRM4	nonsynonymous SNV	SRRM4:ENST00000267260.4:exon4:c.G863A:p.G288E,
12	120499526	G	C	ATCAGAAGTT,AGAGACAACT	exonic	CCDC64	nonsynonymous SNV	CCDC64:ENST00000397558.2:exon3:c.G658C:p.E220Q,
12	122702928	G	A	ACTACTAAGG,AATGAGGCTC	exonic	DIABLO,RP11-512M8.5	nonsynonymous SNV	DIABLO:ENST00000443649.3:exon4:c.C200T:p.S67F,DIABLO:ENST00000464942.2:exon2:c.C41T:p.S14F,DIABLO:ENST00000267169.6:exon3:c.C41T:p.S14F,
12	123481896	G	C	AAAGGCGTCA,AGCAGACGGG	exonic	PITPNM2	nonsynonymous SNV	PITPNM2:ENST00000320201.4:exon10:c.C1448G:p.S483C,PITPNM2:ENST00000392428.2:exon8:c.C611G:p.S204C,PITPNM2:ENST00000542749.1:exon9:c.C1448G:p.S483C,PITPNM2:ENST00000280562.5:exon10:c.C1448G:p.S483C,
12	129181802	G	T	CATCCTGGCA,AGAAGACAAT	exonic	TMEM132C	stopgain	TMEM132C:ENST00000435159.2:exon6:c.G1963T:p.E655X,TMEM132C:ENST00000315208.8:exon6:c.G811T:p.E271X,
12	130898744	C	T	GTGAGCGGGT,GTAGTCAAAG	exonic	RIMBP2	nonsynonymous SNV	RIMBP2:ENST00000261655.4:exon14:c.G2578A:p.D860N,
12	130926629	G	A	GAGCTGGGCG,AGATCTGCGT	exonic	RIMBP2	nonsynonymous SNV	RIMBP2:ENST00000261655.4:exon10:c.C1217T:p.A406V,RIMBP2:ENST00000535703.1:exon10:c.G941T:p.S314I,RIMBP2:ENST00000536002.1:exon2:c.C941T:p.S314I,
13	21381609	C	G	ATCACAGAAT,TGTTCTGTTG	exonic	XPO4	nonsynonymous SNV	XPO4:ENST00000255305.6:exon13:c.G1804C:p.D602H,XPO4:ENST00000406602.2:exon13:c.G1804C:p.D602H,
13	25671168	C	T	GGAACTTAAG,GCACATTTGA	exonic	PABPC3	nonsynonymous SNV	PABPC3:ENST00000281589.3:exon1:c.C832T:p.R278C,
13	33111074	C	A	ACATACGACT,TGTGGTTGAC	exonic	N4BP2L2	stopgain	N4BP2L2:ENST00000446957.2:exon1:c.G91T:p.E31X,N4BP2L2:ENST00000505213.1:exon2:c.G91T:p.E31X,N4BP2L2:ENST00000267068.3:exon2:c.G91T:p.E31X,
13	36397009	C	T	AAAAGGTCTC,CCCCTGAGAA	exonic	DCLK1	nonsynonymous SNV	DCLK1:ENST00000379893.1:exon7:c.G490A:p.G164R,DCLK1:ENST00000255448.4:exon12:c.G1411A:p.G471R,DCLK1:ENST00000360631.3:exon11:c.G1411A:p.G471R,
13	36410201	T	A	ACAGCAAAAT,TCCATCTCCT	exonic	DCLK1	nonsynonymous SNV	DCLK1:ENST00000379893.1:exon4:c.A277T:p.N93Y,DCLK1:ENST00000255448.4:exon8:c.A1198T:p.N400Y,DCLK1:ENST00000360631.3:exon8:c.A1198T:p.N400Y,
13	39264026	G	A	CATCCTGAGT,AGACAGAGTT	exonic	FREM2	nonsynonymous SNV	FREM2:ENST00000280481.7:exon1:c.G2545A:p.E849K,
13	39420790	G	A	GTGGAGAACG,GCACTGACCT	exonic	FREM2	nonsynonymous SNV	FREM2:ENST00000280481.7:exon7:c.G6100A:p.G2034S,
13	49841644	C	T	AACCGAATTT,ATACTGGCCT	exonic	CDADC1	nonsynonymous SNV	CDADC1:ENST00000251108.6:exon5:c.C449T:p.S150L,
13	50062630	G	C	GCATGTCAAA,ACAGCAGGTA	exonic	SETDB2	nonsynonymous SNV	SETDB2:ENST00000354234.4:exon12:c.G1781C:p.R594T,SETDB2:ENST00000317257.8:exon13:c.G1817C:p.R606T,
13	50062647	G	A	GGTATTTTGT,ATGAAGAGTT	exonic	SETDB2	nonsynonymous SNV	SETDB2:ENST00000354234.4:exon12:c.G1798A:p.D600N,SETDB2:ENST00000317257.8:exon13:c.G1834A:p.D612N,
13	67800798	C	T	TGTGATTACC,CCACAGTACT	exonic	PCDH9	nonsynonymous SNV	PCDH9:ENST00000377865.2:exon2:c.G1775A:p.G592E,PCDH9:ENST00000456367.1:exon2:c.G1775A:p.G592E,PCDH9:ENST00000377861.3:exon2:c.G1775A:p.G592E,PCDH9:ENST00000328454.5:exon1:c.G1775A:p.G592E,PCDH9:ENST00000544246.1:exon2:c.G1775A:p.G592E,
13	72147685	C	T	GTTTCCTTAT,CCAGGAACCA	exonic	DACH1	nonsynonymous SNV	DACH1:ENST00000359684.2:exon4:c.G1153A:p.D385N,
13	77655728	G	C	AGGTCGGCTT,AAGTTTTTAT	exonic	MYCBP2	stopgain	MYCBP2:ENST00000407578.2:exon65:c.C11252G:p.S3751X,MYCBP2:ENST00000357337.6:exon66:c.C11138G:p.S3713X,MYCBP2:ENST00000544440.2:exon65:c.C11138G:p.S3713X,
13	108518482	C	A	AGAAAAAGAG,CTTGCCCCGG	exonic	FAM155A	nonsynonymous SNV	FAM155A:ENST00000375915.2:exon1:c.G463T:p.A155S,
13	113832535	G	A	GACAGGGGAG,AAATGGGTTC	exonic	PCID2	nonsynonymous SNV	PCID2:ENST00000375479.2:exon14:c.C1177T:p.P393S,PCID2:ENST00000375457.2:exon14:c.C1171T:p.P391S,PCID2:ENST00000375477.1:exon14:c.C1171T:p.P391S,PCID2:ENST00000375459.1:exon14:c.C1171T:p.P391S,PCID2:ENST00000337344.4:exon14:c.C1177T:p.P393S,P
14	20215969	G	T	GTTGCCATCT,TAACCCTTTG	exonic	OR4Q3	nonsynonymous SNV	OR4Q3:ENST00000331723.1:exon1:c.G383T:p.C128F,
14	22102619	C	A	TGCTAGGTAC,TGTCATAGGC	exonic	OR10G2	nonsynonymous SNV	OR10G2:ENST00000542433.1:exon1:c.G380T:p.R127M,
14	22134093	C	G	ACCAGCTTCT,CATTGACAAG	exonic	OR4E2	nonsynonymous SNV	OR4E2:ENST00000408935.1:exon1:c.C797G:p.S266C,
14	23817394	C	T	TGGGCCTCCT,CCCCAGCATC	exonic	SLC22A17	nonsynonymous SNV	SLC22A17:ENST00000354772.9:exon6:c.G814A:p.E272K,SLC22A17:ENST00000397260.3:exon5:c.G481A:p.E161K,SLC22A17:ENST00000397267.1:exon6:c.G814A:p.E272K,SLC22A17:ENST00000206544.8:exon5:c.G814A:p.E272K,
14	23817731	G	A	AACAGGAAGA,GATGCAGGGA	exonic	SLC22A17	nonsynonymous SNV	SLC22A17:ENST00000354772.9:exon5:c.C676T:p.L226F,SLC22A17:ENST00000397260.3:exon4:c.C343T:p.L115F,SLC22A17:ENST00000397267.1:exon5:c.C676T:p.L226F,SLC22A17:ENST00000206544.8:exon4:c.C676T:p.L226F,
14	23842554	C	T	TCCTGTAGGG,CAGTGAAGAT	exonic	IL25	nonsynonymous SNV	IL25:ENST00000329715.2:exon1:c.C227T:p.A76V,IL25:ENST00000397242.2:exon2:c.C179T:p.A60V,
14	23996086	C	T	GGTGTAGGCT,TGGCCCATGA	exonic	ZFHX2	nonsynonymous SNV	ZFHX2:ENST00000419474.3:exon8:c.G3232A:p.E1078K,
14	31585641	C	T	TCGACTTCTT,CAAGAGCTCT	exonic	HECTD1	nonsynonymous SNV	HECTD1:ENST00000399332.1:exon30:c.G5419A:p.E1807K,HECTD1:ENST00000553700.1:exon30:c.G5419A:p.E1807K,HECTD1:ENST00000399332.1:exon30:c.G5419A:p.E1807K,
14	32623918	C	T	TTTTGAAAAT,GAGAGTTTCT	exonic	ARHGAP5	stopgain	ARHGAP5:ENST00000556611.1:exon2:c.C4270T:p.R1424X,ARHGAP5:ENST00000432921.1:exon2:c.C4270T:p.R1425X,ARHGAP5:ENST00000345122.3:exon2:c.C4273T:p.R1425X,ARHGAP5:ENST00000433497.1:exon6:c.
14	33684756	G	A	TACAAATGTG,AAATCTGAAC	exonic	NPAS3	stopgain	NPAS3:ENST00000357798.5:exon3:c.G309A:p.W103X,
14	35871632	C	T	AACTCTGACT,TGTGTCATAG	exonic	NFKBIA	nonsynonymous SNV	NFKBIA:ENST00000216797.5:exon5:c.G874A:p.E292K,NFKBIA:ENST00000557140.1:exon5:c.G745A:p.E249K,
14	38061073	C	T	AGGGGCGAGT,GGCGCTGGGG	exonic	FOXA1	nonsynonymous SNV	FOXA1:ENST00000250448.2:exon2:c.G817A:p.D273N,FOXA1:ENST00000545425.2:exon2:c.G616A:p.D206N,
14	38265498	G	A	ATTTCTTCCA,AGGACAATAT	splicing	TTC6(ENST00000553443.1:exon19:c.3792-1G>A)	.	.
14	38266034	C	T	CTTATTTATT,ATTTTGTGAA	exonic	TTC6	nonsynonymous SNV	TTC6:ENST00000267368.7:exon3:c.C104T:p.S35L,TTC6:ENST00000553443.1:exon20:c.C3911T:p.S1304L,TTC6:ENST00000382320.3:exon3:c.C53T:p.S18L,TTC6:ENST00000478811.2:exon3:c.C104T:p.S35L,TTC6:ENST00000476979.1:exon4:c.C104T:p.S35L,
14	47120345	A	G	CTGGGAACGT,CTTGACTCCA	exonic	RPL10L	nonsynonymous SNV	RPL10L:ENST00000298283.3:exon1:c.T595C:p.Y199H,
14	50081108	C	T	CATTCTGTTG,CCACACTGTG	exonic	LRR1	nonsynonymous SNV	LRR1:ENST00000298288.6:exon4:c.C1139T:p.A380V,
14	52507522	C	A	CGAACCGTCT,CTCTCCCGGG	exonic	NID2	stopgain	NID2:ENST00000216286.5:exon8:c.G1873T:p.E625X,NID2:ENST00000541773.1:exon9:c.G1714T:p.E572X,
14	52534630	C	T	CTACCTGCTC,CAGGTGGCCA	exonic	NID2	stopgain	NID2:ENST00000216286.5:exon2:c.G480A:p.W160X,NID2:ENST00000541773.1:exon3:c.G321A:p.W107X,
14	53187672	C	T	TGCTTTGCTG,GTCCAGGAAG	exonic	PSMC6	nonsynonymous SNV	PSMC6:ENST00000445930.2:exon11:c.G871T:p.P291S,PSMC6:ENST00000606149.1:exon11:c.C871T:p.P291S,
14	53570499	C	A	AATTAAATTA,TTTCTTCCTC	exonic	DDHD1	nonsynonymous SNV	DDHD1:ENST00000395606.1:exon2:c.G914T:p.S305I,DDHD1:ENST00000556910.1:exon2:c.G56T:p.S19I,DDHD1:ENST00000323669.5:exon2:c.G914T:p.S305I,DDHD1:ENST00000357758.3:exon2:c.G914T:p.S305I,
14	55610175	C	G	GAGTCTCGCT,TGTCGCCAGG	exonic	LGALS3	nonsynonymous SNV	LGALS3:ENST00000554715.1:exon6:c.G610T:p.L203V,
14	60448579	C	T	AAGGCACCCT,TTTATTTTCT	exonic	LRRC9	nonsynonymous SNV	LRRC9:ENST00000446360.1:exon16:c.C1892T:p.S631F,LRRC9:ENST00000254271.4:exon16:c.C1892T:p.S631F,
14	61746818	C	T	AATCACTCCT,GTCGTCGTCG	exonic	TMEM30B	nonsynonymous SNV	TMEM30B:ENST00000555868.1:exon1:c.G1048A:p.E350K,TMEM30B:ENST00000355702.2:exon1:c.G1048A:p.E350K,
14	64457709	C	T	ACTGACGTTT,ACCAGATTTG	exonic	SYNE2	nonsynonymous SNV	SYNE2:ENST00000554584.1:exon20:c.C2522T:p.S841L,SYNE2:ENST00000358025.3:exon21:c.C2522T:p.S841L,SYNE2:ENST00000344113.4:exon21:c.C2522T:p.S841L,
14	64522958	C	G	AGGAAGCTTT,AAAGCACAGG	exonic	SYNE2	nonsynonymous SNV	SYNE2:ENST00000554584.1:exon49:c.C10040G:p.F3380L,SYNE2:ENST00000358025.3:exon49:c.C10041G:p.F3347L,SYNE2:ENST00000344113.4:exon49:c.C10041G:p.F3347L,
14	64988751	G	A	AGAGGCTGAT,AAGAGTCTTT	exonic	ZBTB1	nonsynonymous SNV	ZBTB1:ENST00000394712.2:exon3:c.G529A:p.E177K,ZBTB1:ENST00000554015.1:exon3:c.G529A:p.E177K,ZBTB1:ENST00000358738.3:exon2:c.G529A:p.E177K,
14	67940336	G	A	GTAGTCCCAG,GGCAGGCGTT	exonic	TMEM229B	nonsynonymous SNV	TMEM229B:ENST00000357461.2:exon3:c.C305T:p.P102L,TMEM229B:ENST00000554278.1:exon3:c.C305T:p.P102L,TMEM229B:ENST00000557006.1:exon4:c.C305T:p.P102L,TMEM229B:ENST00000555638.1:exon3:c.C305T:p.P102L,
14	68228256	G	A	TGCGTCCGAA,GGTAGCTTCC	exonic	ZFYVE26	nonsynonymous SNV	ZFYVE26:ENST00000554452.1:exon35:c.C6415T:p.L2139F,ZFYVE26:ENST00000347230.4:exon35:c.C6415T:p.L2139F,
14	70039705	G	A	GGCGGAGGAG,GACTAGGGGA	exonic	CCDC177	nonsynonymous SNV	CCDC177:ENST00000599174.1:exon1:c.C635T:p.P212L,
14	70175609	C	G	GCCTGCTCCT,TGCAGGTGGA	exonic	KIAA0247	nonsynonymous SNV	KIAA0247:ENST00000342745.4:exon6:c.C674G:p.S225C,
14	73989067	C	T	GTCTCTTCTG,CTCCAGCTGT	exonic	HEATR4	nonsynonymous SNV	HEATR4:ENST00000553558.1:exon3:c.G790A:p.A264T,HEATR4:ENST00000560393.1:exon2:c.G649A:p.A217T,
14	74726405	G	C	GCCATGGTGC,GCACTCCATC	exonic	VSX2	nonsynonymous SNV	VSX2:ENST00000261980.2:exon4:c.G680C:p.R227P,
14	75200839	C	T	CCGTAAGATT,CTGGAGTTCC	exonic	FCF1	nonsynonymous SNV	FCF1:ENST00000554938.2:exon7:c.C478T:p.P160S,FCF1:ENST00000553615.1:exon7:c.C469T:p.P157S,FCF1:ENST00000341162.4:exon7:c.C514T:p.P172S,
14	75515107	C	G	TTTACGTTTT,TGCAGTAGTT	exonic	MLH3	nonsynonymous SNV	MLH3:ENST00000556257.1:exon2:c.G1252C:p.E418Q,MLH3:ENST00000556740.1:exon1:c.G1252C:p.E418Q,MLH3:ENST00000355774.2:exon2:c.G1252C:p.E418Q,MLH3:ENST00000238662.7:exon2:c.G1252C:p.E418Q,
14	75515155	C	G	TTAAACATCT,ATAGGAATCT	exonic	MLH3	nonsynonymous SNV	MLH3:ENST00000556257.1:exon2:c.G1204C:p.E402Q,MLH3:ENST00000556740.1:exon1:c.G1204C:p.E402Q,MLH3:ENST00000355774.2:exon2:c.G1204C:p.E402Q,MLH3:ENST00000238662.7:exon2:c.G1204C:p.E402Q,
14	75515407	C	G	ATGCACACAT,ATACTCACAG	exonic	MLH3	nonsynonymous SNV	MLH3:ENST00000556257.1:exon2:c.G952C:p.D318H,MLH3:ENST00000556740.1:exon1:c.G952C:p.D318H,MLH3:ENST00000355774.2:exon2:c.G952C:p.D318H,MLH3:ENST00000238662.7:exon2:c.G952C:p.D318H,
14	76429705	C	T	CTCTGACCCC,CTGGCCCGGG	exonic	TGFB3	nonsynonymous SNV	TGFB3:ENST00000556285.1:exon5:c.G880A:p.G294R,TGFB3:ENST00000238682.3:exon5:c.G880A:p.G294R,
14	77746782	C	T	AACTCATTGT,CTTGGGTTTG	exonic	POMT2	nonsynonymous SNV	POMT2:ENST00000261534.4:exon16:c.G1678A:p.D560N,
14	86089651	C	T	AAGGACAACT,CATCCTGGAG	exonic	FLRT2	nonsynonymous SNV	FLRT2:ENST00000330753.4:exon2:c.C1793T:p.S598F,FLRT2:ENST00000554746.1:exon2:c.C1793T:p.S598F,
14	88938731	C	T	CACTCCCCAT,AACTAGCCGT	exonic	PTPN21	nonsynonymous SNV	PTPN21:ENST00000328736.3:exon14:c.G2728A:p.D910N,PTPN21:ENST00000556564.1:exon15:c.G2728A:p.D910N,
14	90650895	T	A	CCTCTATCGC,TTGCCAACTT	exonic	KCNK13	nonsynonymous SNV	KCNK13:ENST00000282146.4:exon2:c.T775A:p.F259I,
14	94079392	C	G	ATGCGCCTGT,AACTTGCTTT	exonic	UNC79	stopgain	UNC79:ENST00000553484.1:exon28:c.C4070G:p.S1357X,UNC79:ENST00000555664.1:exon27:c.C4004G:p.S1335X,UNC79:ENST00000393151.2:exon27:c.C4004G:p.S1335X,UNC79:ENST00000256339.4:exon29:c.C3473G:p.S1158X,
14	94423164	G	A	ATGACCCCTT,GAACAAGCCC	exonic	ASB2	stopgain	ASB2:ENST00000315988.4:exon1:c.C115T:p.Q39X,ASB2:ENST00000555287.1:exon2:c.C13T:p.Q5X,ASB2:ENST00000555019.1:exon3:c.C259T:p.Q87X,
14	100820867	C	G	AAAATGGCTT,TTATTTTCAT	exonic	WARS	nonsynonymous SNV	WARS:ENST00000556338.1:exon4:c.G336C:p.K112N,WARS:ENST00000344102.5:exon4:c.G336C:p.K112N,WARS:ENST00000556435.1:exon4:c.G336C:p.K112N,WARS:ENST00000553395.1:exon4:c.G336C:p.K112N,WARS:ENST00000557297.1:exon6:c.G336C:p.K112N,WARS:ENST0000
14	102675339	G	A	CAAGTACATC,TGACAGGTGG	exonic	WDR20	nonsynonymous SNV	WDR20:ENST00000335263.5:exon2:c.G649A:p.V217M,WDR20:ENST00000499851.2:exon4:c.G461A:p.V21M,WDR20:ENST00000445663.1:exon3:c.G313A:p.V105M,WDR20:ENST00000424963.2:exon3:c.G460A:p.V154M,WDR20:ENST00000454394.2:exon5:c.G925A:p.V309M,WDR20
15	23684817	G	T	GATCTCCTGT,CAGTGGGGTT	exonic	GOLGA6L2	nonsynonymous SNV	GOLGA6L2:ENST00000567107.1:exon8:c.C1828A:p.A609E,
15	23932234	G	T	GCCTAGAGGA,GGCTCTGCGG	exonic	NDN	nonsynonymous SNV	NDN:ENST00000331837.4:exon1:c.C131A:p.P44H,
15	27182365	G	A	GTTTACGTCT,GACCAACGGC	exonic	GABRA5	stopgain	GABRA5:ENST00000335625.5:exon6:c.G614A:p.W205X,GABRA5:ENST00000400081.3:exon8:c.G614A:p.W205X,GABRA5:ENST00000355395.5:exon7:c.G614A:p.W205X,
15	29398834	G	A	GCACAAGGGC,AGATCCTGGG	exonic	APBA2	nonsynonymous SNV	APBA2:ENST00000558259.1:exon11:c.G1729A:p.E577K,APBA2:ENST00000558330.1:exon12:c.G1693A:p.E565K,APBA2:ENST00000411764.1:exon10:c.G1693A:p.E565K,APBA2:ENST00000561069.1:exon12:c.G1729A:p.E577K,APBA2:ENST00000558402.1:exon13:c.G1729A:p.E577K,
15	31776159	C	T	GAGGCGCGCT,CGGGACCGGC	exonic	OTUD7A	nonsynonymous SNV	OTUD7A:ENST00000382902.1:exon11:c.G2140A:p.E714K,OTUD7A:ENST00000307050.4:exon11:c.G2119A:p.E707K,
15	35830507	C	G	TAGAGATCTT,AACCTCATCA	exonic	DPH6	nonsynonymous SNV	DPH6:ENST00000440392.2:exon3:c.G280C:p.E94Q,DPH6:ENST00000561411.1:exon2:c.G136C:p.E46Q,DPH6:ENST00000256538.4:exon3:c.G280C:p.E94Q,
15	36989592	G	T	TCCTTCCTAG,TAAGTATTAT	splicing	C15orf41(ENST00000566621.1:exon8:c.544+1G>]		

15	45661648	C	G	CAGCCTTTTT,AAATGATCTT	exonic	GATM	nonsynonymous SNV	GATM:ENST00000558336.1:exon3:c.G360C:p.L120F,GATM:ENST00000558163.1:exon
15	51772985	C	G	GCAGATCACT,TCTACTTTGG	exonic	DMXL2	nonsynonymous SNV	DMXL2:ENST00000449909.3:exon22:c.G4410C:p.E1470D,DMXL2:ENST00000251076.
15	56208566	G	C	ATCACTGGTT,AAAAGTTACT	exonic	NEDD4	stopgain	NEDD4:ENST00000506154.1:exon1:c.C464G:p.S155X,NEDD4:ENST00000338963.2:ex
15	57910315	C	T	AGATCAAAAT,AGCAGAAAGA	exonic	GCOM1,MYZAP	stopgain	GCOM1:ENST00000477282.1:exon3:c.C247T:p.Q83X,MYZAP:ENST00000380565.4:ex
15	64017635	C	G	ATTGTTCTTT,TAGATCCACA	exonic	HERC1	nonsynonymous SNV	HERC1:ENST00000443617.2:exon18:c.G3424C:p.E1142Q,
15	66791804	G	C	TTCTTTTCAG,GGCTGGTTTC	exonic	RPL4	nonsynonymous SNV	RPL4:ENST00000568588.1:exon11:c.C943G:p.P315A,RPL4:ENST00000307961.6:exon
15	77759545	G	A	GTTCATGAAT,AGCGTCGAGA	exonic	HMG20A	nonsynonymous SNV	HMG20A:ENST00000381714.3:exon5:c.G346A:p.E116K,HMG20A:ENST00000558176.1
15	79750498	C	T	CACAGTGGCT,CCACGGACCC	exonic	KIAA1024	nonsynonymous SNV	KIAA1024:ENST00000559272.1:exon1:c.C2009T:p.S670F,KIAA1024:ENST0000030542
15	81046596	C	T	GAGCGCTGTC,CCGAGCCGTG	exonic	ABHD17C	nonsynonymous SNV	ABHD17C:ENST00000560609.1:exon3:c.C170T:p.P57L,ABHD17C:ENST00000258884.
15	83438603	G	C	GTTTGTCACT,AGCAATATGT	exonic	FSD2	stopgain	FSD2:ENST00000334574.8:exon8:c.C1301G:p.S434X,
15	83932106	C	T	GTCTCCCTCT,TGAATTGTGT	exonic	BNC1	nonsynonymous SNV	BNC1:ENST00000569704.1:exon4:c.G1876A:p.E626K,BNC1:ENST00000345382.2:exon
15	89395189	G	A	TGCCATCCTA,AGTTCACCAC	exonic	ACAN	nonsynonymous SNV	ACAN:ENST00000561243.1:exon10:c.G2191A:p.E731K,ACAN:ENST00000352105.7:ex
15	90903699	T	G	GCAAGGGGTG,GAGAAGAAAT	exonic	ZNF774	nonsynonymous SNV	ZNF774:ENST00000354377.3:exon4:c.T636G:p.C212W,
15	91347415	G	C	TATGGAAACA,AAAATTCCAG	exonic	BLM	nonsynonymous SNV	BLM:ENST00000355112.3:exon19:c.G3577C:p.E1193Q,
15	94942239	A	G	AAAGATTTAG,ACAAGCTTTT	exonic	MCTP2	nonsynonymous SNV	MCTP2:ENST00000557742.1:exon7:c.A602G:p.E201G,MCTP2:ENST00000451018.3:ex
16	2815702	C	T	CCCAAGGCAC,GGAGAAGTCC	exonic	SRRM2	nonsynonymous SNV	SRRM2:ENST00000301740.8:exon11:c.C5173T:p.R1725W,
16	3100454	G	A	CGGGTTGGGG,GCACCCTAGC	exonic	MMP25	nonsynonymous SNV	MMP25:ENST00000336577.4:exon4:c.G568A:p.G190S,
16	3434728	C	G	CACACGGCCT,TCCTCACTTT	exonic	ZSCAN32	nonsynonymous SNV	ZSCAN32:ENST00000439568.2:exon2:c.G98C:p.R33T,ZSCAN32:ENST00000396846.3
16	3591889	G	C	CCTGGCCCCG,AGTCCCCAAT	exonic	NLRC3	nonsynonymous SNV	NLRC3:ENST00000359128.5:exon20:c.C3047G:p.S1016C,NLRC3:ENST00000301749.
16	3639732	C	T	TGTGCGACTT,GTTCCCTTCC	exonic	SLX4	nonsynonymous SNV	SLX4:ENST00000294008.3:exon12:c.G3907A:p.E1303K,
16	21008782	C	T	ATGTTGAGGT,ATCTGGGGAA	exonic	DNAH3	nonsynonymous SNV	DNAH3:ENST00000261383.3:exon44:c.G6424A:p.D2142N,
16	23646533	T	C	ACTTGCATCC,TATTTTTATT	exonic	PALB2	nonsynonymous SNV	PALB2:ENST00000261584.4:exon4:c.A1334G:p.K445R,
16	24942653	G	A	TGTTCCTGAA,AACTCTGGCC	exonic	ARHGAP17	nonsynonymous SNV	ARHGAP17:ENST00000303665.5:exon18:c.C1733T:p.S578F,ARHGAP17:ENST000002
16	55983760	G	C	GAGGAGGCTG,ATAAAAATAG	exonic	CES5A	nonsynonymous SNV	CES5A:ENST00000521992.1:exon2:c.C97G:p.P33A,
16	66583906	C	T	CCCGCGACTT,CCGGCCCAAA	exonic	TK2	nonsynonymous SNV	TK2:ENST00000545043.2:exon1:c.G59A:p.G20E,TK2:ENST00000451102.2:exon1:c.G5
16	68002421	C	G	CGCCGCGCCT,GGCCCGTCCA	exonic	SLC12A4	nonsynonymous SNV	SLC12A4:ENST00000422611.2:exon1:c.G137C:p.R46P,
16	68056442	C	T	GGTTGGGCCA,AGCCCGCACC	exonic	DDX28	nonsynonymous SNV	DDX28:ENST00000332395.5:exon1:c.G664A:p.V222M,
16	71610150	G	A	ACAATGGCTC,GATGGGGTTG	exonic	TAT	stopgain	TAT:ENST00000355962.4:exon2:c.C169T:p.R57X,
16	88496939	G	A	CCTCCCCGAG,AGACCCGCAG	exonic	ZNF469	nonsynonymous SNV	ZNF469:ENST00000565624.1:exon1:c.G3061A:p.E1021K,ZNF469:ENST00000437464.
16	88503047	G	C	TGAGATGCAA,ACCTGTGCTT	exonic	ZNF469	nonsynonymous SNV	ZNF469:ENST00000565624.1:exon1:c.G9169C:p.D3057H,ZNF469:ENST00000437464.
16	89795707	G	C	CGAGTCCTTT,AGCCTTACCC	exonic	ZNF276	nonsynonymous SNV	ZNF276:ENST00000443381.2:exon6:c.G1150C:p.E384Q,ZNF276:ENST00000289816.5
16	90025620	C	T	TTCCTGCCTC,GCTGGGCTCA	exonic	DEF8	nonsynonymous SNV	DEF8:ENST00000418391.2:exon6:c.C571T:p.R191C,
17	6018	C	T	CACGTTTTCA,GTAGGGGTCC	exonic	DOC2B	nonsynonymous SNV	DOC2B:ENST00000343572.7:exon6:c.G916A:p.V306M,
17	986762	C	T	CGGCCTACCT,TGAGATCTTC	exonic	ABR	nonsynonymous SNV	ABR:ENST00000570441.1:exon4:c.G367A:p.E123K,ABR:ENST00000291107.2:exon4:c.
17	1936905	G	T	ACAACTTTGA,ATCCCCAAGA	exonic	DPH1	nonsynonymous SNV	DPH1:ENST00000263083.6:exon2:c.G183T:p.E61D,
17	3181523	C	T	TGAACGGATT,GTAGAACTGC	exonic	OR3A2	nonsynonymous SNV	OR3A2:ENST00000408891.2:exon1:c.G707A:p.R236Q,
17	3195698	G	A	GTACATGGGG,TGTGGAGTTT	exonic	OR3A1	nonsynonymous SNV	OR3A1:ENST00000323404.1:exon1:c.C179T:p.T60I,
17	3828717	C	G	TCCCAGCGCT,ACTTCTGGCT	exonic	ATP2A3	stoploss	ATP2A3:ENST00000359983.3:exon23:c.G3158C:p.X1053S,ATP2A3:ENST0000039704
17	4496244	G	A	TGGACCCCCT,AGATTGCCCA	exonic	SMTNL2	nonsynonymous SNV	SMTNL2:ENST00000389313.4:exon3:c.G508A:p.E170K,SMTNL2:ENST00000338859.4
17	4883766	G	A	GTGTGCCTTG,GGAGCTCTGG	exonic	CAMTA2	nonsynonymous SNV	CAMTA2:ENST00000361571.5:exon8:c.C848T:p.P283L,CAMTA2:ENST00000572543.1
17	7096870	G	A	GTCCTTGGTG,GCCCAAGGAT	exonic	DLG4	nonsynonymous SNV	DLG4:ENST00000302955.6:exon16:c.C1616T:p.P539L,DLG4:ENST00000399510.2:exo
17	7229588	G	C	AACTCATCCA,ACTGAATTCG	exonic	NEURL4	nonsynonymous SNV	NEURL4:ENST00000573651.1:exon3:c.C316G:p.L106V,NEURL4:ENST00000570460.1
17	7250410	G	A	AATGGCCAGG,GAAGGGAGCC	exonic	ACAP1	nonsynonymous SNV	ACAP1:ENST00000158762.3:exon14:c.G1192A:p.G398R,
17	7534006	C	A	AGAACCTCCT,CTCCTTTGAG	exonic	SHBG	nonsynonymous SNV	SHBG:ENST00000575729.1:exon3:c.C38A:p.S13Y,SHBG:ENST00000416273.3:exon3:c
17	7577610	T	A	AGAGCCAACC,AGGAGATAAC	splicing	TP53(ENST00000413465.2:exon7:c.673-2A>T,EN	.	.
17	7678188	C	T	TACTTCTTGT,CAATGATGAC	exonic	DNAH2	nonsynonymous SNV	DNAH2:ENST00000389173.2:exon28:c.C4613T:p.S1538F,DNAH2:ENST00000572933.
17	8215376	C	T	CCAGTCCCTT,CTGCAGCAAC	exonic	ARHGEF15	nonsynonymous SNV	ARHGEF15:ENST00000361926.3:exon2:c.C19T:p.P7S,ARHGEF15:ENST00000579439
17	8424550	C	T	TATGCGGAGC,AAAAGCTGTC	exonic	MYH10	nonsynonymous SNV	MYH10:ENST00000379980.4:exon17:c.G1966A:p.G656S,MYH10:ENST00000269243.4
17	12639642	G	A	AGGTTCTCTG,GGACAAACCA	exonic	AC005358.1,MYOCD	nonsynonymous SNV	MYOCD:ENST00000343344.4:exon6:c.G580A:p.G194R,MYOCD:ENST00000425538.1
17	12647507	C	T	CAGTCCAAAT,CTTGGGTGAC	exonic	AC005358.1,MYOCD	nonsynonymous SNV	MYOCD:ENST00000343344.4:exon8:c.C725T:p.S242F,MYOCD:ENST00000425538.1:e
17	12656433	G	A	CCAGCCTCTT,GAAATGCTCA	exonic	AC005358.1,MYOCD	nonsynonymous SNV	MYOCD:ENST00000343344.4:exon10:c.G1828A:p.G610R,MYOCD:ENST00000425538
17	15973811	G	A	AGATTGACCT,AGTTGTTGTC	exonic	NCOR1	nonsynonymous SNV	NCOR1:ENST00000395851.1:exon30:c.C4229T:p.S1410L,NCOR1:ENST00000268712.
17	16395180	C	T	GCCCGCGCGC,CCGCCTTGTC	exonic	FAM211A	nonsynonymous SNV	FAM211A:ENST00000470794.1:exon1:c.G125A:p.G42E,FAM211A:ENST00000409083.
17	18682140	G	C	CCAACTTGAA,ATCTCTTTGC	exonic	FBXW10	nonsynonymous SNV	FBXW10:ENST00000395667.1:exon14:c.G2685C:p.K895N,FBXW10:ENST0000030879
17	18881189	G	C	GGAGCTGCCT,AGTGGCTGTC	exonic	FAM83G	stopgain	FAM83G:ENST00000585154.2:exon4:c.C1790G:p.S597X,FAM83G:ENST00000345041.
17	26715283	C	T	AACTCAGGTT,CCAGCTGGCC	exonic	SARM1	nonsynonymous SNV	SARM1:ENST00000457710.3:exon6:c.C1619T:p.S540F,
17	27052565	C	A	ACTAACATGT,AGGAGTCTGC	exonic	TLCD1	nonsynonymous SNV	TLCD1:ENST00000292090.3:exon2:c.G211T:p.D71Y,TLCD1:ENST00000394933.3:exon
17	29183993	A	G	GTGGCATTTG,AACCACCCTC	exonic	ATAD5	nonsynonymous SNV	ATAD5:ENST00000321990.4:exon8:c.A2656G:p.K886E,ATAD5:ENST00000578295.1:ex
17	30274665	C	G	GATATGTTAT,AAAAGTAGAG	exonic	SUZ12	stopgain	SUZ12:ENST00000322652.5:exon4:c.C416G:p.S139X,
17	30621325	C	T	TGCCTTTTTC,TCTACAATGG	exonic	RHBDL3	nonsynonymous SNV	RHBDL3:ENST00000538145.1:exon4:c.C508T:p.L170F,RHBDL3:ENST00000269051.4
17	30694909	C	T	TCTACAGCTT,AACAACTAGT	exonic	ZNF207	nonsynonymous SNV	ZNF207:ENST00000394670.4:exon10:c.C1040T:p.S347L,ZNF207:ENST00000577908.1
17	34964142	G	A	GGCCGGCACG,TGGGCTGCCC	exonic	MRM1	nonsynonymous SNV	MRM1:ENST00000585770.1:exon3:c.G88A:p.V30M,MRM1:ENST00000250156.7:exon3
17	35936511	C	T	GGAAATACTC,CACACCTAGA	exonic	SYNRG	nonsynonymous SNV	SYNRG:ENST00000585472.1:exon7:c.G592A:p.G198R,SYNRG:ENST00000339208.6:e
17	36731149	G	A	GCCCCGGAGG,GGCGGAGTCG	exonic	SRCIN1	nonsynonymous SNV	SRCIN1:ENST00000578925.1:exon3:c.C397T:p.P133S,SRCIN1:ENST00000584266.1:e
17	37439073	G	A	GTCACACCAG,AAATGTTCAA	exonic	FBXL20	nonsynonymous SNV	FBXL20:ENST00000577399.1:exon8:c.C536T:p.S179F,FBXL20:ENST00000394294.3:e
17	37686913	C	T	CCCCGGACCT,CACCGCCGCC	exonic	CDK12	nonsynonymous SNV	CDK12:ENST00000430627.2:exon14:c.C3790T:p.P1264S,CDK12:ENST00000447079.4
17	40447783	C	G	ACTTCATCAT,CAGTACCAGG	exonic	STAT5A	nonsynonymous SNV	STAT5A:ENST00000546010.2:exon4:c.C432G:p.I144M,STAT5A:ENST00000345506.4:e
17	41003806	C	T	CTGCCTCACC,CTCCTACATG	exonic	AOC3	nonsynonymous SNV	AOC3:ENST00000308423.2:exon1:c.C446T:p.P149L,
17	42882087	C	A	GCCTTCTTCT,CCGGGGACCA	exonic	GJC1	stopgain	GJC1:ENST00000592524.1:exon3:c.G1099T:p.E367X,GJC1:ENST00000587113.1:exon
17	44847163	C	T	CGGCCCGCCT,GTTGTTGTGC	exonic	WNT3	nonsynonymous SNV	WNT3:ENST00000225512.5:exon3:c.G574A:p.E192K,
17	46050900	G	A	CTGGTGGACA,AAGGCACTGC	exonic	CDK5RAP3	nonsynonymous SNV	CDK5RAP3:ENST00000338399.4:exon3:c.G68A:p.R23K,CDK5RAP3:ENST0000058499
17	47218648	C	G	TGGTCATAAT,CTGGTACTTG	exonic	B4GALNT2	nonsynonymous SNV	B4GALNT2:ENST00000393354.2:exon2:c.C54G:p.I18M,B4GALNT2:ENST0000030040
17	56285475	C	T	CCAGAAGATT,ATCCTCATGG	exonic	MKS1	nonsynonymous SNV	MKS1:ENST00000393119.2:exon13:c.G1156A:p.E386K,MKS1:ENST00000337050.7:ex
17	58303554	C	G	TTCCTCCATT,AAAACAGATG	exonic	USP32	nonsynonymous SNV	USP32:ENST00000590133.1:exon13:c.G1269C:p.L423F,USP32:ENST00000592339.1:e
17	60088536	G	T	TGTTGTTGCT,ACCTAAAGAT	exonic	MED13	nonsynonymous SNV	MED13:ENST00000397786.2:exon9:c.C1342A:p.Q448K,
17	61903476	G	C	GGAGAGGCTT,ATTGGAACCA	exonic	FTSJ3	nonsynonymous SNV	FTSJ3:ENST00000427159.2:exon5:c.C240G:p.I80M,FTSJ3:ENST00000581209.1:exon5
17	71231635	C	T	AGTGATAATC,ACCCAGAATG	exonic	C17orf80	nonsynonymous SNV	C17orf80:ENST00000582391.1:exon2:c.C14T:p.P5L,C17orf80:ENST00000585109.1:ex
17	73733427	C	T	GTGCGCTGCT,CTTCCGGGAC	exonic	ITGB4	nonsynonymous SNV	ITGB4:ENST00000449880.2:exon16:c.C2015T:p.S672F,ITGB4:ENST00000339591.3:ex
17	74470489	G	A	GATGCATCCG,AGTGGTCATT	exonic	RHBDF2	nonsynonymous SNV	RHBDF2:ENST00000389760.4:exon12:c.C1430T:p.S477F,RHBDF2:ENST00000313080
17	76183457	G	A	ACGCCATGAT,GATGTGTCTG	exonic	AFMID	nonsynonymous SNV	AFMID:ENST00000327898.5:exon1:c.G6A:p.M2I,AFMID:ENST00000588800.1:exon1:c.
17	76183458	G	A	CGCCATGATG,ATGTGTCTGG	exonic	AFMID	nonsynonymous SNV	AFMID:ENST00000327898.5:exon1:c.G7A:p.D3N,AFMID:ENST00000588800.1:exon1:c.
17	76458991	C	T	GCAACAAGTT,CGTTCTCTTC	exonic	DNAH17	nonsynonymous SNV	DNAH17:ENST00000389840.5:exon57:c.G9067A:p.E3023K,DNAH17:ENST0000058532
17	78317667	G	C	GTGCAGACTG,AATTTGGGTG	exonic	RNF213	nonsynonymous SNV	RNF213:ENST00000336301.6:exon3:c.G413C:p.G138A,RNF213:ENST00000582970.1
17	79682611	G	A	GTGTTGCTGG,CTCCGTCAGC	exonic	SLC25A10	nonsynonymous SNV	SLC25A10:ENST00000350690.5:exon3:c.G317A:p.G106D,SLC25A10:ENST000005458
17	79846193	C	A	GCCGGCACCT,TGCCTCTTCC	exonic	ALYREF	nonsynonymous SNV	ALYREF:ENST00000505490.2:exon5:c.G704T:p.R235I,ALYREF:ENST00000331204.4:e
17	79846276	C	T	GGTTTCTAGT,ATGCCACCTC	exonic	ALYREF	nonsynonymous SNV	ALYREF:ENST00000505490.2:exon5:c.G621A:p.M207I,ALYREF:ENST00000331204.4:e
17	79918493	G	C	GCAGGTCACC,AGGTGTTGAG	exonic	NOTUM	nonsynonymous SNV	NOTUM:ENST00000425009.1:exon2:c.C293G:p.S98W,NOTUM:ENST00000409678.3:e
18	8718542	A	T	AAACTGCCGA,TCCTGCAGTA	exonic	SOGA2	nonsynonymous SNV	SOGA2:ENST00000359865.3:exon3:c.A94T:p.I32F,SOGA2:ENST00000523122.1:exon2
18	12802142	C	T	AAAGTTCTTT,CATCGTTTCT	exonic	PTPN2	stopgain	PTPN2:ENST00000353319.4:exon8:c.G867A:p.W289X,PTPN2:ENST00000327283.3:ex
18	12802143	C	T	AAGTTCTTTC,ATCGTTTCTA	exonic	PTPN2	stopgain	PTPN2:ENST00000353319.4:exon8:c.G866A:p.W289X,PTPN2:ENST00000327283.3:ex
18	18533582	G	C	CGGAAAGACT,ATTTGCAGTG	exonic	ROCK1	nonsynonymous SNV	ROCK1:ENST00000399799.2:exon32:c.C4018G:p.Q1340E,ROCK1:ENST00000578051.
18	18533669	G	A	TTTACTAAAT,AGTTACCCAT	exonic	ROCK1	nonsynonymous SNV	ROCK1:ENST00000399799.2:exon32:c.C3931T:p.H1311Y,ROCK1:ENST00000578051.
18	19031107	C	T	AAAACCACAT,ATTAGGTGAG	exonic	GREB1L	nonsynonymous SNV	GREB1L:ENST00000431264.1:exon13:c.C1844T:p.S615L,GREB1L:ENST00000580732
18	19378157	C	A	AAGGTGGCAT,TGCAGGATCA	exonic	MIB1	nonsynonymous SNV	MIB1:ENST00000261537.6:exon8:c.C1205A:p.S402Y,
18	21437860	T	A	TTCCGGGTTT,ACCGCTTTCC	exonic	LAMA3	nonsynonymous SNV	LAMA3:ENST00000313654.9:exon33:c.T4189A:p.Y1397N,LAMA3:ENST00000399516.3
18	30314160	G	A	TCTGAACTCT,GTGAGCTTGC	exonic	KLHL14	nonsynonymous SNV	KLHL14:ENST00000358095.4:exon4:c.C1118T:p.P373L,
18	30314161	G	A	CTGAACTCTG,TGAGCTTGCA	exonic	KLHL14	nonsynonymous SNV	KLHL14:ENST00000358095.4:exon4:c.C1117T:p.P373S,
18	30926349	C	T	GTTTCCATTT,CTGCTTTAAC	exonic	CCDC178	nonsynonymous SNV	CCDC178:ENST00000399177.3:exon8:c.G484A:p.E162K,CCDC178:ENST0000040330
18	31319046	G	A	AGAACATAAG,AGTCAGAAAC	exonic	ASXL3	nonsynonymous SNV	ASXL3:ENST00000269197.5:exon11:c.G1678A:p.E560K,
18	31323516	C	T	AATAAAAATT,TGTCCCTGTA	exonic	ASXL3	nonsynonymous SNV	ASXL3:ENST00000269197.5:exon12:c.C3704T:p.S1235F,
18	34387860	A	T	CATCAGGTAA,AGTTCTTCAC	exonic	TPGS2	nonsynonymous SNV	TPGS2:ENST00000589049.1:exon3:c.T203A:p.F68Y,TPGS2:ENST00000334295.4:exon
18	44771315	C	T	CCAGAAGCAT,AGAGTCTGTG	exonic	SKOR2	nonsynonymous SNV	SKOR2:ENST00000425639.1:exon3:c.G2716A:p.D906N,
18	44775185	C	G	CACAGACGCT,GGCCTCGCGT	exonic	SKOR2	nonsynonymous SNV	SKOR2:ENST00000425639.1:exon1:c.G370C:p.E124Q,SKOR2:ENST00000400404.1:ex
18	57364559	G	A	CCCCGGCTCG,AGGCGGCGGC	exonic	CCBE1	nonsynonymous SNV	CCBE1:ENST00000439986.4:exon1:c.C16T:p.P6S,
18	74625773	G	A	GCACACCGGG,AAAAGCCCTA	exonic	ZNF236	nonsynonymous SNV	ZNF236:ENST00000320610.9:exon18:c.G2980A:p.E994K,ZNF236:ENST00000253159.
18	74625777	A	C	ACCGGGGAAA,GCCCTACAAG	exonic	ZNF236	nonsynonymous SNV	ZNF236:ENST00000320610.9:exon18:c.A2984C:p.K995T,ZNF236:ENST00000253159.
18	77092936	T	A	CTGCCATCCC,GCAGCCTCCA	exonic	ATP9B	nonsynonymous SNV	ATP9B:ENST00000589732.1:exon4:c.T383A:p.L128Q,
18	77170727	C	T	TCCAAACGGT,CCCCTCCACG	exonic	NFATC1	nonsynonymous SNV	NFATC1:ENST00000586434.1:exon2:c.C413T:p.S138F,NFATC1:ENST00000587635.1
19	1220629	C	A	AGCCAGGGCT,CCCGGCTTTC	exonic	STK11	nonsynonymous SNV	STK11:ENST00000326873.7:exon5:c.C647A:p.S216Y,
19	1230933	G	A	GTCCATGCTG,AGAGGTGGCC	exonic	C19orf26	nonsynonymous SNV	C19orf26:ENST00000590083.1:exon9:c.C1321T:p.P441S,C19orf26:ENST00000215376.
19	1234215	G	A	AGAGTCGCTA,ATGCCGAGGG	exonic	C19orf26	nonsynonymous SNV	C19orf26:ENST00000590083.1:exon7:c.C743T:p.S248F,C19orf26:ENST00000215376.6
19	1371384	C	T	TTCCCAGCCT,GGTGGGTCAG	exonic	MUM1	nonsynonymous SNV	MUM1:ENST00000415183.3:exon12:c.C2141T:p.S714L,
19	3734319	C	T	TCCTCTCCCC,TGCAGACATC	exonic	TJP3	nonsynonymous SNV	TJP3:ENST00000262968.9:exon7:c.C971T:p.P324L,TJP3:ENST00000382008.3:exon8:c
19	4174718	G	A	CCGTTGTGCT,GGCGCAGGGC	exonic	SIRT6	stopgain	SIRT6:ENST00000305232.6:exon7:c.C883T:p.Q295X,SIRT6:ENST00000381935.3:exon
19	4501289	G	C	TCGGGACTCG,AGGAGGGGCC	exonic	HDGFRP2	nonsynonymous SNV	HDGFRP2:ENST00000592417.1:exon6:c.G379C:p.E127Q,HDGFRP2:ENST0000058642
19	4511538	C	T	TGGACGGCCC,CTTGGCCACA	exonic	PLIN4	nonsynonymous SNV	PLIN4:ENST00000301286.3:exon3:c.G2392A:p.G798R,
19	4511736	C	T	TGGATGGCCC,TTTGGCCACA	exonic	PLIN4	nonsynonymous SNV	PLIN4:ENST00000301286.3:exon3:c.G2194A:p.G732R,
19	6026207	G	C	TGTGTGATGT,ATTCCTTCGC	exonic	RFX2	nonsynonymous SNV	RFX2:ENST00000586302.1:exon5:c.C474G:p.I158M,RFX2:ENST00000586806.1:exon2
19	7985396	G	A	GGACGCCACC,AGCTGGCCCG	exonic	SNAPC2	nonsynonymous SNV	SNAPC2:ENST00000221573.6:exon1:c.G145A:p.E49K,
19	9066891	G	A	ACTGGTTGTA,GTTCTGGGCT	exonic	MUC16	nonsynonymous SNV	MUC16:ENST00000397910.4:exon3:c.C20555T:p.P6852L,
19	9525228	C	T	CGTTGCTCTC,AGTAGAGGTT	exonic	ZNF266	nonsynonymous SNV	ZNF266:ENST00000592904.1:exon5:c.G373A:p.G125R,ZNF266:ENST00000592292.1:e
19	10334798	G	A	GGGCAGGAGT,GACGGGACAG	exonic	S1PR2	nonsynonymous SNV	S1PR2:ENST00000590320.1:exon2:c.C784T:p.H262Y,
19	11409608	C	G	AGTGGTCCTT,TTTTCTTTCC	exonic	TSPAN16	nonsynonymous SNV	TSPAN16:ENST00000590327.1:exon3:c.C328G:p.L110V,TSPAN16:ENST00000316737
19	11616577	C	T	GCCTCCGCCT,GGGCTCTAGC	exonic	ZNF653	nonsynonymous SNV	ZNF653:ENST00000293771.5:exon1:c.G25A:p.E9K,
19	11651320	G	C	AAGAGGGAGA,AGACATACAG	exonic	CNN1	nonsynonymous SNV	CNN1:ENST00000585869.1:exon1:c.G80C:p.R27T,
19	11941219	C	G	AACCTGACCT,TTTAGGTAAG	exonic	ZNF440	nonsynonymous SNV	ZNF440:ENST00000414255.1:exon1:c.C131G:p.S44C,ZNF440:ENST00000427505.1:ex
19	11943478	C	A	GTAGTTCCTT,AGTAGTTCCA	exonic	ZNF440	stopgain	ZNF440:ENST00000304060.5:exon4:c.C1487A:p.S496X,
19	12460520	G	C	CACATTTATA,AGTATCTCTC	exonic	ZNF442	nonsynonymous SNV	ZNF442:ENST00000438182.1:exon3:c.C1672G:p.L558V,ZNF442:ENST00000242804.4
19	12460637	G	C	TCATGTCCTC,AAGGAAACGA	exonic	ZNF442	nonsynonymous SNV	ZNF442:ENST00000438182.1:exon3:c.C1555G:p.R519G,ZNF442:ENST00000242804.4
19	12989346	C	T	AAGATCCCTT,CAGCTGGTAG	exonic	DNASE2	nonsynonymous SNV	DNASE2:ENST00000222219.3:exon5:c.G559A:p.E187K,DNASE2:ENST00000592506.1
19	13008629	C	A	TGACGAGTAT,ACGTGATCCG	exonic	GCDH	nonsynonymous SNV	GCDH:ENST00000422947.2:exon11:c.C1063A:p.H355N,GCDH:ENST00000591470.1:ex
19	13356080	C	T	GGAAATAATT,TAGAATGGGG	splicing	CACNA1A(ENST00000360228.5:exon32:c.4867-1(	.	.
19	14522369	G	C	TGCTGATCTG,AAGGCCAGCT	exonic	DDX39A	nonsynonymous SNV	DDX39A:ENST00000454233.2:exon3:c.C378G:p.F126L,DDX39A:ENST00000242776.4
19	14674047	C	G	TTGCGGAGAT,AAGAACCTCT	exonic	TECR	nonsynonymous SNV	TECR:ENST00000598333.1:exon3:c.C96G:p.I32M,TECR:ENST00000598918.1:exon3:c
19	14675621	C	T	ACTGGGGCTT,GCCGCGTGGA	exonic	TECR	nonsynonymous SNV	TECR:ENST00000601187.1:exon2:c.C83T:p.S28L,
19	14710483	G	A	TGGATGGGTA,GATGGGTTCT	exonic	CLEC17A	nonsynonymous SNV	CLEC17A:ENST00000551730.1:exon11:c.G664A:p.G222R,
19	15636205	C	T	GACGGCGTTC,GCATATACGC	exonic	CYP4F22	nonsynonymous SNV	CYP4F22:ENST00000269703.3:exon3:c.C58T:p.R20C,CYP4F22:ENST00000601005.2
19	15807773	A	T	CCTGGCGTTG,TGCTGCTGCA	exonic	CYP4F12	nonsynonymous SNV	CYP4F12:ENST00000324632.10:exon12:c.A1453T:p.M485L,CYP4F12:ENST0000055
19	17780454	G	A	GATGACCTGG,AGTCCAGCGT	exonic	UNC13A	nonsynonymous SNV	UNC13A:ENST00000519716.2:exon5:c.C302T:p.S101F,UNC13A:ENST00000428389.2
19	19766340	C	A	CGCTTACCTT,AATCCATACA	exonic	ATP13A1	stopgain	ATP13A1:ENST00000357324.6:exon10:c.G1393T:p.E465X,ATP13A1:ENST00000291
19	20118034	C	G	TGGAATGAAT,TTGCATGCAC	exonic	ZNF682	nonsynonymous SNV	ZNF682:ENST00000601100.1:exon4:c.G181C:p.D61H,ZNF682:ENST00000358523.5:ex
19	20118064	C	G	AAAAGGTCTT,AGTGTAATGA	exonic	ZNF682	stoploss	ZNF682:ENST00000593468.1:exon5:c.G311C:p.X104S,
19	20728403	T	A	ATTCTTCACA,TTGAAGGGTT	exonic	ZNF737	nonsynonymous SNV	ZNF737:ENST00000427401.4:exon4:c.A606T:p.K202N,ZNF737:ENST00000594419.1:e
19	21239491	C	T	ATAAAAGATT,TTTCCAAGAA	exonic	ZNF430	nonsynonymous SNV	ZNF430:ENST00000261560.5:exon5:c.C377T:p.S126F,
19	21991740	C	G	TAGAATTTCT,TGCAGTATGG	exonic	ZNF43	nonsynonymous SNV	ZNF43:ENST00000595461.1:exon7:c.G1081C:p.E361Q,ZNF43:ENST00000354959.4:ex
19	22697266	G	A	GCTCCACATG,AACTTGTAGG	exonic	ZNF98	nonsynonymous SNV	ZNF98:ENST00000593802.1:exon2:c.C215T:p.S72F,
19	23158640	C	T	AGTATGAATT,TCTTATGTTC	exonic	ZNF728	nonsynonymous SNV	ZNF728:ENST00000594710.1:exon4:c.G1499A:p.R500K,
19	23937670	C	G	GTCCAAGGCT,TTTTTCTTGT	exonic	ZNF681	nonsynonymous SNV	ZNF681:ENST00000402377.3:exon3:c.G181C:p.E61Q,
19	34263804	C	T	CCTGACCTTC,CCCGGTTCAA	exonic	CHST8	nonsynonymous SNV	CHST8:ENST00000262622.4:exon4:c.C1111T:p.P371S,CHST8:ENST00000438847.3:ex
19	35422810	C	T	AGCCTTCTCC,AGCAGGAGTG	exonic	ZNF30	stopgain	ZNF30:ENST00000601957.1:exon3:c.C73T:p.Q25X,ZNF30:ENST00000601540.1:exon3
19	35823671	C	T	TGGGAAGGTT,CTTCTGAGCA	exonic	CD22	nonsynonymous SNV	CD22:ENST00000594250.1:exon2:c.C256T:p.P86S,CD22:ENST00000599811.1:exon3:c
19	37406819	C	T	AGCTCCCCAT,GGTCTTCGGT	exonic	ZNF829	nonsynonymous SNV	ZNF829:ENST00000520965.1:exon1:c.G38A:p.R13Q,
19	37903595	T	A	CACCTGTATG,TTTCTCATAT	exonic	ZNF569	nonsynonymous SNV	ZNF569:ENST00000392150.2:exon2:c.A1488T:p.K496N,ZNF569:ENST00000392149.2
19	37903596	T	C	ACCTGTATGT,TTCTCATATG	exonic	ZNF569	nonsynonymous SNV	ZNF569:ENST00000392150.2:exon2:c.A1487G:p.K496R,ZNF569:ENST00000392149.2
19	38934890	G	A	TGTCTCCTCC,AGCGCTACCT	exonic	RYR1	nonsynonymous SNV	RYR1:ENST00000360985.3:exon6:c.G526A:p.E176K,RYR1:ENST00000355481.4:exon6
19	39104902	T	C	TCCCTGTGTA,CTTCTTCTGT	exonic	MAP4K1	nonsynonymous SNV	MAP4K1:ENST00000586296.1:exon6:c.A400G:p.I134V,MAP4K1:ENST00000591517.1
19	39226769	C	T	TGGCGGCGCT,GGAGAAGACA	exonic	CAPN12	nonsynonymous SNV	CAPN12:ENST00000328867.4:exon12:c.G1564A:p.E522K,CAPN12:ENST00000601953
19	39322376	C	T	GTCAGTAGGT,GCGGAGTCTG	exonic	ECH1	nonsynonymous SNV	ECH1:ENST00000221418.4:exon1:c.G37A:p.D13N,ECH1:ENST00000595470.1:exon1:c
19	40030427	G	A	CCTGGCCGCC,CCGCCGCCGG	exonic	EID2	nonsynonymous SNV	EID2:ENST00000390658.2:exon1:c.C293T:p.A98V,
19	40329827	C	G	GCTCGGTACT,AATTTTGTCA	exonic	FBL	nonsynonymous SNV	FBL:ENST00000598417.1:exon6:c.G358C:p.E120Q,FBL:ENST00000599134.1:exon5:c.
19	40876131	G	T	ATGGACTGGC,TTCACTGACC	exonic	PLD3	nonsynonymous SNV	PLD3:ENST00000409281.1:exon8:c.G665T:p.R222L,PLD3:ENST00000356508.5:exon8
19	41040128	G	A	CAGCAAAGCA,ACCAGCTGGT	exonic	SPTBN4	nonsynonymous SNV	SPTBN4:ENST00000392025.1:exon4:c.G466A:p.D156N,SPTBN4:ENST00000595535.1
19	41076417	C	G	GCGGACACCT,GGCCGGACCG	exonic	SPTBN4	nonsynonymous SNV	SPTBN4:ENST00000392025.1:exon17:c.C3331G:p.R1111G,SPTBN4:ENST000005982
19	41114455	G	T	GCCCGGCCCT,AGCTTCCCTT	exonic	LTBP4	stopgain	LTBP4:ENST00000204005.9:exon12:c.G1462T:p.E488X,LTBP4:ENST00000396819.3:e
19	42906932	C	T	AGGCCTCTGT,CATGGGGCTC	exonic	LIPE	nonsynonymous SNV	LIPE:ENST00000244289.4:exon9:c.G2794A:p.D932N,
19	44612340	G	A	GTCCACATGG,AGAGAAAACA	exonic	ZNF224	nonsynonymous SNV	ZNF224:ENST00000336976.6:exon6:c.G2027A:p.G676E,
19	44981416	G	A	TGAGTTCTTT,ATGCACAACA	exonic	ZNF180	stopgain	ZNF180:ENST00000391956.4:exon4:c.C1207T:p.Q403X,ZNF180:ENST00000592529.1
19	45853775	C	G	CCCCTAGGTG,CCCCAACACA	exonic	KLC3	nonsynonymous SNV	KLC3:ENST00000470402.1:exon9:c.C1280G:p.A427G,KLC3:ENST00000585434.1:exon
19	46029409	G	C	CTTCGTCCAG,AGCTGAGGAA	exonic	VASP	nonsynonymous SNV	VASP:ENST00000245932.6:exon13:c.G1117C:p.E373Q,
19	50365638	G	A	CGGATCAAAG,CTGGGAGCTC	exonic	PNKP	nonsynonymous SNV	PNKP:ENST00000322344.3:exon11:c.C1019T:p.A340V,PNKP:ENST00000600910.1:ex
19	50384530	G	C	CCTAGGTGCA,AGCCCAGCTG	exonic	TBC1D17	nonsynonymous SNV	TBC1D17:ENST00000535102.2:exon4:c.G226C:p.E76Q,TBC1D17:ENST00000221543.
19	51361834	G	A	CTGGACAGGG,GCAAAAGCAC	exonic	KLK3	nonsynonymous SNV	KLK3:ENST00000360617.3:exon4:c.G613A:p.G205S,KLK3:ENST00000593997.1:exon4
19	51871157	G	A	GAAGAGGAAG,CGCTCGTGGT	exonic	CLDND2,CTD-2616J11.11	nonsynonymous SNV	CLDND2:ENST00000601435.1:exon3:c.C293T:p.A98V,CTD-2616J11.11:ENST000006
19	53058281	G	A	ACAGTGGAAT,AAACCTTACA	exonic	ZNF808	nonsynonymous SNV	ZNF808:ENST00000487863.1:exon3:c.G1905A:p.M635I,ZNF808:ENST00000359798.4
19	55019399	G	A	GCTGGTGAAA,GTAAGGACGT	exonic	LAIR2	nonsynonymous SNV	LAIR2:ENST00000351841.2:exon3:c.G364A:p.G122R,LAIR2:ENST00000301202.2:exon
19	55421386	G	C	AGGCGACATT,AGAACACCAG	exonic	NCR1	nonsynonymous SNV	NCR1:ENST00000350790.5:exon4:c.G358C:p.E120Q,NCR1:ENST00000594765.1:exon
19	55915682	C	T	ATGCCCAGCT,AGCCGTCCAG	exonic	UBE2S	nonsynonymous SNV	UBE2S:ENST00000587845.1:exon3:c.G316A:p.E106K,UBE2S:ENST00000264552.9:ex
19	57640197	C	T	AAGAATTTTT,AGCTGAGCAA	exonic	USP29	stopgain	USP29:ENST00000598197.1:exon1:c.C154T:p.Q52X,USP29:ENST00000254181.4:exon
20	1600560	G	A	AAAGGACTAG,AAGGTGGGGC	exonic	RP4-576H24.4,SIRPB1	nonsynonymous SNV	RP4-576H24.4:ENST00000564763.1:exon1:c.C31T:p.P11S,SIRPB1:ENST0000038160
20	2736291	C	T	TCTAGCCCCC,GCTGGCGGCT	exonic	EBF4	nonsynonymous SNV	EBF4:ENST00000609451.1:exon15:c.C1559T:p.P520L,EBF4:ENST00000380648.4:exon
20	3674327	C	T	AGCCCCGGGC,ACACCTGCAC	exonic	SIGLEC1	stopgain	SIGLEC1:ENST00000344754.4:exon13:c.G3275A:p.W1092X,SIGLEC1:ENST0000020
20	5903464	C	T	GCCGGGCATT,TCAGGAGAAG	exonic	CHGB	nonsynonymous SNV	CHGB:ENST00000378961.4:exon4:c.C674T:p.S225F,CHGB:ENST00000455042.1:exon
20	5965450	G	A	CTACTATCCA,ATTTGATTTG	exonic	MCM8	nonsynonymous SNV	MCM8:ENST00000378896.3:exon15:c.G1757A:p.R586K,MCM8:ENST00000265187.4:e
20	10601961	G	C	TTTTGGAGTA,ACAGAAAGAG	splicing	SLX4IP(ENST00000334534.5:exon7:c.406-1G>C)	.	.
20	17600312	G	A	CTGGCACTCG,CGCTGGCGGC	exonic	RRBP1	nonsynonymous SNV	RRBP1:ENST00000377807.2:exon20:c.C2342T:p.A781V,RRBP1:ENST00000377813.1
20	19955916	C	G	CCGCCCAGCT,TGAATCACGG	exonic	RIN2	nonsynonymous SNV	RIN2:ENST00000255006.6:exon8:c.C1394G:p.S465C,
20	25457411	C	T	GCCCTCCTGG,CACTTCCTGC	exonic	NINL	nonsynonymous SNV	NINL:ENST00000278886.6:exon17:c.G2516A:p.G839D,
20	32265342	C	T	GTCACGTAGG,CAGGGTTGGC	exonic	E2F1	nonsynonymous SNV	E2F1:ENST00000343380.5:exon5:c.G730A:p.A244T,
20	33609061	C	G	TACATGATCT,ATGCATAATC	exonic	TRPC4AP	nonsynonymous SNV	TRPC4AP:ENST00000432634.2:exon8:c.G1033C:p.E345Q,TRPC4AP:ENST00000451
20	35651155	C	G	ACGTCCATAT,TTCCTTCGTA	exonic	RBL1	nonsynonymous SNV	RBL1:ENST00000344359.3:exon17:c.G2457C:p.K819N,RBL1:ENST00000373664.3:ex
20	36149761	G	C	GGCCTCGGCT,AACTGCTCAT	exonic	NNAT	nonsynonymous SNV	NNAT:ENST00000062104.2:exon1:c.G28C:p.E10Q,NNAT:ENST00000346199.2:exon1:c
20	36845761	G	C	CCGCTGGGTA,AGGCAAAGGC	exonic	KIAA1755	nonsynonymous SNV	KIAA1755:ENST00000279024.4:exon13:c.C2795G:p.S932C,KIAA1755:ENST0000043
20	37356920	G	A	CGAGGTGCTG,AGAAGTCGCT	exonic	SLC32A1	nonsynonymous SNV	SLC32A1:ENST00000217420.1:exon2:c.G1216A:p.E406K,
20	40944562	G	A	CACGGGCACC,AAAAGCACTC	exonic	PTPRT	nonsynonymous SNV	PTPRT:ENST00000373198.4:exon12:c.C1940T:p.S647L,PTPRT:ENST00000373193.3:e
20	42695486	C	T	AGTGTGGCAT,AGCACCTGCA	exonic	TOX2	nonsynonymous SNV	TOX2:ENST00000413823.1:exon2:c.C293T:p.S98L,
20	43058231	G	A	GCTCCTGCCG,GAGCCGTCGC	exonic	HNF4A	nonsynonymous SNV	HNF4A:ENST00000316673.4:exon10:c.G1285A:p.G429R,HNF4A:ENST00000415691.2
20	43385652	C	A	ATGCCATTCT,TAGCAGGTAG	exonic	RIMS4	stopgain	RIMS4:ENST00000541604.2:exon5:c.G481T:p.E161X,RIMS4:ENST00000372851.3:exon
20	48257181	G	A	AGCATGGCTC,ATTAAAGGGT	exonic	B4GALT5	stopgain	B4GALT5:ENST00000371711.4:exon6:c.C628T:p.R210X,
20	61511058	C	T	TGCCTCCCTT,GAAAGTCTGG	exonic	DIDO1	nonsynonymous SNV	DIDO1:ENST00000266070.4:exon16:c.G6250A:p.E2084K,DIDO1:ENST00000395343.1
20	61917010	A	G	GTGCAGGTGA,GGAGGGAAAG	exonic	ARFGAP1	nonsynonymous SNV	ARFGAP1:ENST00000523460.1:exon3:c.A47G:p.K16R,ARFGAP1:ENST00000522403.2
20	61945122	A	G	CCACCCTCCA,CCTGGCCCTG	exonic	COL20A1	nonsynonymous SNV	COL20A1:ENST00000326996.6:exon18:c.A2237G:p.N746S,COL20A1:ENST000004222
20	62338086	A	C	CTGCTCCAGG,AGGTCTGAGG	exonic	ARFRP1	nonsynonymous SNV	ARFRP1:ENST00000610136.1:exon3:c.T98G:p.F33C,ARFRP1:ENST00000440854.1:ex
20	62599294	G	A	ACGCAGAAAG,ATCCGTCATC	exonic	ZNF512B	nonsynonymous SNV	ZNF512B:ENST00000450537.1:exon2:c.C10T:p.P4S,ZNF512B:ENST00000369888.1:ex
20	62839178	G	A	AGCGAGGAGG,TGAAAAGGGC	exonic	MYT1	nonsynonymous SNV	MYT1:ENST00000328439.1:exon7:c.G629A:p.G210D,MYT1:ENST00000536311.1:exon
20	62899301	C	A	CTTGCTGTTT,TTTTGCTCCT	exonic	PCMTD2	nonsynonymous SNV	PCMTD2:ENST00000308824.6:exon5:c.C644A:p.S215Y,PCMTD2:ENST00000299468.7
21	15599369	C	T	TGACCTTTAT,AGATGACAGC	exonic	RBM11	stopgain	RBM11:ENST00000400577.3:exon5:c.C601T:p.Q201X,
21	15954576	G	A	CATGTCCAGA,AGGGTTTTCA	exonic	SAMSN1	nonsynonymous SNV	SAMSN1:ENST00000285670.2:exon2:c.C142T:p.L48F,
21	19635117	C	T	GGTATAATTC,CAATCTAATT	exonic	CHODL	nonsynonymous SNV	CHODL:ENST00000543733.1:exon5:c.C587T:p.P196L,CHODL:ENST00000400128.1:ex
21	30521536	C	T	AGACTCCATT,CTTTGGTCTT	exonic	MAP3K7CL	nonsynonymous SNV	MAP3K7CL:ENST00000339024.4:exon5:c.C97T:p.P33S,MAP3K7CL:ENST0000054593
21	41033393	C	T	AGAGAATTCC,GGGGGGAAGA	exonic	B3GALT5	nonsynonymous SNV	B3GALT5:ENST00000398714.2:exon2:c.C907T:p.R303W,B3GALT5:ENST0000038062
21	42629222	G	T	GAGCCCAGAA,AGGGTGGGCT	exonic	BACE2	nonsynonymous SNV	BACE2:ENST00000347667.5:exon7:c.G1122T:p.K374N,BACE2:ENST00000330333.6:e
21	42773932	G	A	TGAAAAATAT,AAAAGCAGTA	exonic	MX2	nonsynonymous SNV	MX2:ENST00000330714.3:exon11:c.G1450A:p.E484K,
21	43529698	G	A	GGACAGCGCG,AACACGACTG	exonic	UMODL1	nonsynonymous SNV	UMODL1:ENST00000400427.1:exon10:c.G1330A:p.E444K,UMODL1:ENST000004004
21	43854990	G	A	CTGCCAAGAC,GAGTCGTGGG	exonic	UBASH3A	nonsynonymous SNV	UBASH3A:ENST00000319294.6:exon10:c.G1319A:p.R440Q,UBASH3A:ENST0000039
21	44324278	G	C	AGTTCCTGTT,AGAATAACCA	exonic	NDUFV3	nonsynonymous SNV	NDUFV3:ENST00000354250.2:exon3:c.G1156C:p.E386Q,
21	47419121	G	A	CCGGGGTCCC,AGGGCCCCCA	exonic	COL6A1	nonsynonymous SNV	COL6A1:ENST00000361866.3:exon26:c.G1729A:p.E577K,
21	47832780	G	T	TGGTGACGTT,AAGGATGCAC	exonic	PCNT	nonsynonymous SNV	PCNT:ENST00000359568.5:exon29:c.G6024T:p.L2008F,
22	18020316	G	A	GCAGCCCATG,AGAATGGAGG	exonic	CECR2	nonsynonymous SNV	CECR2:ENST00000262608.8:exon13:c.G1648A:p.E550K,CECR2:ENST00000400573.5
22	18370096	G	A	TACCTTGGGA,AACGCTTCCG	exonic	MICAL3	nonsynonymous SNV	MICAL3:ENST00000383094.3:exon13:c.C1997T:p.S666F,MICAL3:ENST00000429452.1
22	21213432	G	C	CAATCCGTTC,ACGACGACGG	exonic	SNAP29	nonsynonymous SNV	SNAP29:ENST00000215730.7:exon1:c.G34C:p.D12H,
22	22707325	C	T	GTTCCTCTCT,ACTGCACAGG	exonic	IGLV5-48	nonsynonymous SNV	IGLV5-48:ENST00000390293.1:exon1:c.C37T:p.H13Y,
22	24037146	G	A	GCAAAAGCAT,AAAGAGCTAA	exonic	RGL4	nonsynonymous SNV	RGL4:ENST00000290691.5:exon6:c.G1026A:p.M342I,RGL4:ENST00000441897.1:exon
22	24487774	G	C	CAACCCACCT,AGCTGGCCAT	exonic	CABIN1	nonsynonymous SNV	CABIN1:ENST00000398319.2:exon24:c.G3763C:p.E1255Q,CABIN1:ENST0000026311
22	24573745	C	T	CTGCTCTCCC,CAAAGGCAGC	exonic	CABIN1	nonsynonymous SNV	CABIN1:ENST00000398319.2:exon36:c.C6479T:p.P2160L,CABIN1:ENST00000263119.
22	26701947	C	G	TGGATCAGCT,CTTGTGGAGG	exonic	SEZ6L	nonsynonymous SNV	SEZ6L:ENST00000529632.2:exon6:c.C1351G:p.P451A,SEZ6L:ENST00000343706.4:ex
22	29684726	C	T	GGGATTTGAT,GTGGAGGCAT	exonic	EWSR1	nonsynonymous SNV	EWSR1:ENST00000406548.1:exon8:c.C925T:p.R309C,EWSR1:ENST00000333395.6:e
22	29885787	G	A	AAAGTCCCCT,AGAAGGCCAA	exonic	NEFH	nonsynonymous SNV	NEFH:ENST00000310624.6:exon4:c.G2158A:p.E720K,
22	31685244	G	C	CACAAAGTTT,ACTTTGTGTA	exonic	PIK3IP1	stopgain	PIK3IP1:ENST00000402249.3:exon5:c.C644G:p.S215X,
22	33712132	C	G	GGCTCATACT,GTAGTGCAGG	exonic	LARGE	nonsynonymous SNV	LARGE:ENST00000402320.1:exon10:c.G1234C:p.E412Q,LARGE:ENST00000608642.1
22	36556730	C	G	GGAAGCTTCT,TTGAAAGAGT	exonic	APOL3	nonsynonymous SNV	APOL3:ENST00000397289.2:exon1:c.G210C:p.K70N,APOL3:ENST00000432700.1:exon
22	37271838	G	A	CTGTGGCCTG,GAGGGAGGGG	exonic	NCF4	stopgain	NCF4:ENST00000397147.4:exon8:c.G771A:p.W257X,NCF4:ENST00000447071.1:exon
22	38028662	A	T	CCAGACCTAC,ACGAGATGGG	exonic	GGA1	nonsynonymous SNV	GGA1:ENST00000325180.8:exon15:c.A1603T:p.N535Y,GGA1:ENST00000343632.4:ex
22	41926767	G	A	GGGCCCTGTG,GGGACGTGTC	exonic	POLR3H	nonsynonymous SNV	POLR3H:ENST00000396504.2:exon6:c.C485T:p.P162L,POLR3H:ENST00000407461.1
22	41926768	G	A	GGCCCTGTGG,GGACGTGTCA	exonic	POLR3H	nonsynonymous SNV	POLR3H:ENST00000396504.2:exon6:c.C484T:p.P162S,POLR3H:ENST00000407461.1
22	43539185	C	T	GCCCGGCATT,GCCGCTCCGT	exonic	MCAT	nonsynonymous SNV	MCAT:ENST00000327555.5:exon1:c.G170A:p.R57Q,MCAT:ENST00000290429.6:exon1
22	43625080	C	T	CTGCAGACCT,CGCAGTGGGT	exonic	SCUBE1	nonsynonymous SNV	SCUBE1:ENST00000360835.4:exon9:c.G1082A:p.G361E,
22	45595772	G	A	TCGTCCTCCC,GAAGAACTCC	exonic	KIAA0930	nonsynonymous SNV	KIAA0930:ENST00000251993.7:exon8:c.C1012T:p.R338W,KIAA0930:ENST00000336
22	45758801	G	C	TACCTTTCTG,ATAGTTTCTT	exonic	SMC1B	nonsynonymous SNV	SMC1B:ENST00000404354.3:exon16:c.C2526G:p.I842M,SMC1B:ENST00000357450.4
X	1506197	C	G	ACTGCATCAT,ATGCGCCGCC	exonic	SLC25A6	nonsynonymous SNV	SLC25A6:ENST00000381401.5:exon3:c.G714C:p.M238I,
X	7894027	C	T	AACCAACGAT,CCGCAGACGC	exonic	PNPLA4	nonsynonymous SNV	PNPLA4:ENST00000381042.4:exon2:c.G134A:p.G45E,PNPLA4:ENST00000444736.1:e
X	9660253	G	T	GGGGGGCCAT,ACGTCCCGAG	exonic	TBL1X	nonsynonymous SNV	TBL1X:ENST00000217964.7:exon9:c.G850T:p.D284Y,TBL1X:ENST00000536365.1:exo
X	12906533	T	C	AATTTTAAGA,AGCATTTTAC	exonic	TLR7	nonsynonymous SNV	TLR7:ENST00000380659.3:exon3:c.T2906C:p.I969T,
X	13061293	C	G	TCCCCATGCT,ATCAGTTACA	exonic	FAM9C	nonsynonymous SNV	FAM9C:ENST00000542843.1:exon3:c.G136C:p.E46Q,FAM9C:ENST00000333995.3:ex
X	17394364	C	T	GCTCACCCAC,GCCGCGTCTG	exonic	NHS	nonsynonymous SNV	NHS:ENST00000380060.3:exon1:c.C484T:p.R162C,
X	19031816	G	T	GTCTGGACAG,AGGTGCGCTG	exonic	GPR64	nonsynonymous SNV	GPR64:ENST00000354791.3:exon15:c.C1039A:p.P347T,GPR64:ENST00000357544.3
X	20190968	G	A	TCAGTAAACT,AATACTGTTC	exonic	RPS6KA3	stopgain	RPS6KA3:ENST00000544447.1:exon16:c.C1165T:p.Q389X,RPS6KA3:ENST00000379
X	21450748	G	A	CTTGGAAACA,AAAATCTAAA	exonic	CNKSR2	nonsynonymous SNV	CNKSR2:ENST00000279451.4:exon3:c.G247A:p.E83K,CNKSR2:ENST00000543067.1
X	23412019	G	A	GAAGTGCATG,GGTAGCTATT	exonic	PTCHD1	nonsynonymous SNV	PTCHD1:ENST00000379361.4:exon3:c.G2384A:p.G795E,
X	30237637	G	A	TTTGAAAGAT,AAGAGAAAGC	exonic	MAGEB2	nonsynonymous SNV	MAGEB2:ENST00000378988.4:exon2:c.G940A:p.E314K,
X	30877656	C	T	GAAACGTTGT,GAAGATCATG	exonic	TAB3	nonsynonymous SNV	TAB3:ENST00000378932.2:exon6:c.G50A:p.R17Q,TAB3:ENST00000467136.1:exon4:c

X	37545217	G	A	GCGCGGAGAT,AAATTCCCGG	exonic	XK	nonsynonymous SNV	XK:ENST00000378616.3:exon1:c.G3A:p.M1I,
X	41555743	G	A	CATGCCTTTC,ATTCATCTAC	exonic	GPR34	nonsynonymous SNV	GPR34:ENST00000378138.5:exon3:c.G857A:p.R286Q,GPR34:ENST00000378142.4:exon3:c.G857A:p.R286Q,
X	47511482	C	T	GCTCTCACCT,TAGCAACATG	exonic	UXT	nonsynonymous SNV	UXT:ENST00000335890.2:exon5:c.G454A:p.E152K,UXT:ENST00000333119.3:exon6:c.G418A:p.E140K,
X	48434979	G	C	ACGTTCCAGA,ACTATAATGG	exonic	RBM3	nonsynonymous SNV	RBM3:ENST00000354480.2:exon2:c.G318C:p.E106D,RBM3:ENST00000376755.1:exon4:c.G400C:p.D134H,RBM3:ENST00000376759.3:exon5:c.G400C:p.D134H,RBM3:ENST00000430348.2:exon4:c.G318C:p.E106D,
X	48543994	C	T	TCCACCCCCA,CCCCTTCTTC	exonic	WAS	nonsynonymous SNV	WAS:ENST00000376701.4:exon3:c.C332T:p.T111I,WAS:ENST00000450772.1:exon5:c.C332T:p.T111I,
X	48565911	C	T	GAAGCGGGTC,GTATTGAATG	exonic	SUV39H1	nonsynonymous SNV	SUV39H1:ENST00000337852.6:exon6:c.C1222T:p.R408C,SUV39H1:ENST00000376687.3:exon6:c.C1189T:p.R397C,
X	48896765	C	T	GGCCTGTTCC,GACGCTCACG	exonic	TFE3	nonsynonymous SNV	TFE3:ENST00000315869.7:exon3:c.G401A:p.R134Q,
X	49066404	C	T	AGCTCACCGT,TGGTGGGGGG	exonic	CACNA1F	nonsynonymous SNV	CACNA1F:ENST00000376251.1:exon40:c.G4525A:p.D1509N,CACNA1F:ENST00000376265.2:exon40:c.G4720A:p.D1574N,CACNA1F:ENST00000323022.5:exon40:c.G4687A:p.D1563N,
X	53283848	C	T	CCGGTGGCTC,GGGCACCAGC	exonic	IQSEC2	nonsynonymous SNV	IQSEC2:ENST00000375365.2:exon4:c.G650A:p.R217Q,IQSEC2:ENST00000396435.3:exon4:c.G1265A:p.R422Q,IQSEC2:ENST00000375368.5:exon3:c.G1235A:p.R412Q,
X	62855285	G	A	TGAGGAAAAG,GAGCTCAGGG	exonic	ARHGEF9	nonsynonymous SNV	ARHGEF9:ENST00000374878.1:exon10:c.C1351T:p.P451S,
X	67940227	C	G	GAGCATTCAG,AAGCCATGCG	exonic	STARD8	nonsynonymous SNV	STARD8:ENST00000374599.3:exon8:c.C2011G:p.Q671E,STARD8:ENST00000252336.6:exon7:c.C1771G:p.Q591E,STARD8:ENST00000374597.3:exon7:c.C1771G:p.Q591E,
X	67941564	C	T	CTCAATGTCT,TAAGAAGGAT	exonic	STARD8	nonsynonymous SNV	STARD8:ENST00000374599.3:exon10:c.C2435T:p.S812F,STARD8:ENST00000252336.6:exon9:c.C2195T:p.S732F,STARD8:ENST00000374597.3:exon9:c.C2195T:p.S732F,
X	69353912	C	T	CCGGATAGTC,AGGAGAAGGT	exonic	IGBP1	stopgain	IGBP1:ENST00000356413.4:exon2:c.C115T:p.Q39X,IGBP1:ENST00000342206.6:exon1:c.C115T:p.Q39X,
X	69421915	G	A	TGCAAACAGG,TGGGTTCCAA	splicing	DGAT2L6(ENST00000333026.3:exon5:c.647+1G>	.	.
X	70339188	G	A	CTTCCCCTAA,GAAAAAACAA	splicing	MED12(ENST00000429213.1:exon2:c.20-1G>A)	.	.
X	73811873	G	C	AGTAGGCTCT,AGTCGCTATC	exonic	RLIM	stopgain	RLIM:ENST00000332687.6:exon4:c.C1277G:p.S426X,RLIM:ENST00000349225.2:exon5:c.C1277G:p.S426X,
X	76937199	C	G	TTCTCTTTTT,TCCTTGACAA	exonic	ATRX	nonsynonymous SNV	ATRX:ENST00000373344.5:exon9:c.G3549C:p.E1183D,ATRX:ENST00000395603.3:exon8:c.G3435C:p.E1145D,
X	83129181	G	A	AATGGAATCT,ATTTGGAGTT	exonic	CYLC1	nonsynonymous SNV	CYLC1:ENST00000329312.4:exon4:c.G1465A:p.D489N,
X	84526170	G	A	TGTATTTTCA,GTGTGCAGAT	exonic	ZNF711	nonsynonymous SNV	ZNF711:ENST00000360700.4:exon10:c.G1760A:p.R587K,ZNF711:ENST00000542798.1:exon7:c.G1148A:p.R383K,ZNF711:ENST00000276123.3:exon10:c.G1622A:p.R541K,ZNF711:ENST00000395402.1:exon9:c.G1646A:p.R549K,ZNF711:ENST00000373165.3:exon9:c.G1622A:p.R54
X	86890582	C	T	TATTGGTGGA,GTGATGGAAG	exonic	KLHL4	nonsynonymous SNV	KLHL4:ENST00000373119.4:exon9:c.C1732T:p.R578C,KLHL4:ENST00000373114.4:exon9:c.C1732T:p.R578C,
X	102471224	G	A	AAGCCTGGAG,AAATATCAGG	exonic	BEX4	nonsynonymous SNV	BEX4:ENST00000372691.3:exon2:c.G143A:p.G48E,BEX4:ENST00000372695.5:exon3:c.G143A:p.G48E,
X	104463988	G	A	ATGAGGCAGG,AGCTGGACAG	exonic	TEX13A	nonsynonymous SNV	TEX13A:ENST00000372575.1:exon3:c.C890T:p.S297F,TEX13A:ENST00000372578.3:exon3:c.C890T:p.S297F,
X	106396510	A	C	CTGCTGTGAC,GGATAAAATC	exonic	NUP62CL	nonsynonymous SNV	NUP62CL:ENST00000372466.4:exon7:c.T422G:p.L141R,
X	108691314	G	A	GCCAAAGTGG,GATTGATAAA	exonic	GUCY2F	nonsynonymous SNV	GUCY2F:ENST00000218006.2:exon6:c.C1553T:p.P518L,
X	112022236	G	A	TGTTTTGTCT,GATTGCAGGT	exonic	AMOT	nonsynonymous SNV	AMOT:ENST00000371962.1:exon10:c.C2450T:p.P817L,AMOT:ENST00000524145.1:exon11:c.C3146T:p.P1049L,AMOT:ENST00000304758.1:exon11:c.C1919T:p.P640L,AMOT:ENST00000371959.3:exon10:c.C3146T:p.P1049L,
X	114426634	C	T	AGGGGCCGAT,GCCCGATGCC	exonic	RBMXL3	nonsynonymous SNV	RBMXL3:ENST00000424776.3:exon1:c.C2630T:p.S877L,
X	117033075	T	A	CCACATAGAT,TTATTTTCGA	exonic	KLHL13	nonsynonymous SNV	KLHL13:ENST00000371882.1:exon7:c.A1611T:p.K537N,KLHL13:ENST00000371878.1:exon7:c.A1611T:p.K537N,KLHL13:ENST00000262820.3:exon7:c.A1764T:p.K588N,KLHL13:ENST00000541812.1:exon8:c.A1716T:p.K572N,KLHL13:ENST00000371876.1:exon6:c.A1611T:p.K537N,K
X	125685733	G	A	TTCCACAGGT,GAAGTAGCCG	exonic	DCAF12L1	nonsynonymous SNV	DCAF12L1:ENST00000371126.1:exon1:c.C859T:p.H287Y,
X	129168538	G	A	CAGCTCAGAG,AGGCCTCAGA	exonic	BCORL1	nonsynonymous SNV	BCORL1:ENST00000456822.1:exon6:c.G3214A:p.E1072K,BCORL1:ENST00000303743.5:exon9:c.G4414A:p.E1472K,
X	129203540	C	G	GCTGTGGCTT,GCTGCTCTCA	exonic	ELF4	nonsynonymous SNV	ELF4:ENST00000335997.7:exon8:c.G922C:p.E308Q,ELF4:ENST00000308167.5:exon8:c.G922C:p.E308Q,
X	130220344	G	A	AGCGCGTGTG,AAGTCCAGCC	exonic	ARHGAP36	stopgain	ARHGAP36:ENST00000412432.2:exon10:c.G1230A:p.W410X,ARHGAP36:ENST00000370922.1:exon10:c.G1287A:p.W429X,ARHGAP36:ENST00000370921.1:exon7:c.G915A:p.W305X,ARHGAP36:ENST00000276211.5:exon10:c.G1323A:p.W441X,
X	130409948	C	T	TCACCCAGAT,ATAAGGGGCA	exonic	IGSF1	nonsynonymous SNV	IGSF1:ENST00000370903.3:exon15:c.G2898A:p.M966I,IGSF1:ENST00000370904.1:exon21:c.G2856A:p.M952I,IGSF1:ENST00000370910.1:exon14:c.G2856A:p.M952I,IGSF1:ENST00000361420.3:exon15:c.G2883A:p.M961I,
X	132161857	G	A	TTTGTGGAAT,AAGTTTTGTT	exonic	USP26	nonsynonymous SNV	USP26:ENST00000406273.1:exon2:c.C392T:p.S131L,USP26:ENST00000511190.1:exon6:c.C392T:p.S131L,USP26:ENST00000370832.1:exon1:c.C392T:p.S131L,
X	133378945	G	A	AACGACTGCA,ATAGCAGCAA	exonic	CCDC160	nonsynonymous SNV	CCDC160:ENST00000370809.4:exon2:c.G115A:p.D39N,CCDC160:ENST00000517294.1:exon3:c.G115A:p.D39N,
X	135574131	G	A	ATTGAATCCC,AAAGAGAATT	exonic	BRS3	nonsynonymous SNV	BRS3:ENST00000370648.3:exon3:c.G797A:p.R266Q,
X	138856874	G	A	GTACTTTTAG,AAACTCATGC	exonic	ATP11C	nonsynonymous SNV	ATP11C:ENST00000361648.2:exon19:c.C2200T:p.P734S,ATP11C:ENST00000327569.3:exon19:c.C2200T:p.P734S,ATP11C:ENST00000359686.2:exon19:c.C2200T:p.P734S,ATP11C:ENST00000370557.1:exon19:c.C2191T:p.P731S,ATP11C:ENST00000370543.1:exon19:c.C2200T:p.
X	139038351	C	T	TGACAAGTTT,AGCTAATTCA	exonic	CXorf66	nonsynonymous SNV	CXorf66:ENST00000370540.1:exon3:c.G790A:p.E264K,
X	140995322	C	T	CTCCATTTTC,TCAGAGTCCT	exonic	MAGEC1	nonsynonymous SNV	MAGEC1:ENST00000285879.4:exon4:c.C2132T:p.P711L,
X	147743661	C	T	CCAATGCCTC,ACCTTCTGTT	exonic	AFF2	nonsynonymous SNV	AFF2:ENST00000370458.1:exon3:c.C401T:p.P134L,AFF2:ENST00000370457.5:exon3:c.C401T:p.P134L,AFF2:ENST00000370460.2:exon3:c.C413T:p.P138L,AFF2:ENST00000342251.3:exon3:c.C401T:p.P134L,
X	148035252	C	T	TGAGGCACCT,GTGTGGCAAC	exonic	AFF2	nonsynonymous SNV	AFF2:ENST00000286437.5:exon7:c.C463T:p.R155C,AFF2:ENST00000370457.5:exon9:c.C1441T:p.R481C,AFF2:ENST00000370460.2:exon10:c.C1540T:p.R514C,AFF2:ENST00000342251.3:exon9:c.C1441T:p.R481C,
X	149638792	G	A	AGCAAGCAGG,GTCTGCTACA	exonic	MAMLD1	nonsynonymous SNV	MAMLD1:ENST00000432680.2:exon3:c.G872A:p.G291E,MAMLD1:ENST00000370401.2:exon4:c.G947A:p.G316E,MAMLD1:ENST00000262858.5:exon3:c.G947A:p.G316E,MAMLD1:ENST00000426613.2:exon2:c.G872A:p.G291E,
X	149924320	C	T	ACGATGGAAT,CACGGATGAG	exonic	MTMR1	nonsynonymous SNV	MTMR1:ENST00000445323.2:exon15:c.C1840T:p.P614S,MTMR1:ENST00000541925.1:exon15:c.C1534T:p.P512S,MTMR1:ENST00000370390.3:exon14:c.C1816T:p.P606S,MTMR1:ENST00000544228.1:exon14:c.C1816T:p.P606S,
X	151900576	G	C	GAGTATAGTT,ATGGTAGTGG	exonic	MAGEA12	nonsynonymous SNV	MAGEA12:ENST00000357916.4:exon2:c.C225G:p.I75M,MAGEA12:ENST00000393900.3:exon3:c.C225G:p.I75M,MAGEA12:ENST00000393869.3:exon3:c.C225G:p.I75M,
X	153129458	G	A	CCAGCAGGAG,GAGCCTCACG	exonic	L1CAM	nonsynonymous SNV	L1CAM:ENST00000538883.1:exon25:c.C3343T:p.P1115S,L1CAM:ENST00000543994.1:exon25:c.C3343T:p.P1115S,L1CAM:ENST00000370055.1:exon25:c.C3322T:p.P1108S,L1CAM:ENST00000361981.3:exon24:c.C3322T:p.P1108S,L1CAM:ENST00000370057.3:exon25:c.C3337T:p.
X	155004137	G	C	CTCCACTGAT,ATGAAGACAA	exonic	SPRY3	nonsynonymous SNV	SPRY3:ENST00000302805.2:exon2:c.G604C:p.D202H,

=large_intestine

c.13262C>A;AAchange=p.P4421H;tissue=lung

135G>A;AAchange=p.E379K;tissue=large_intestine&NEK10_ENST00000429845;basechange=c.1135G>A;AAchange=p.E379K;tissue=large_intestine

ge=c.5074G>A;AAchange=p.D1692N;tissue=lung

>T;AAchange=p.V757F;tissue=kidney

ge=c.1282C>A;AAchange=p.P428T;tissue=lung

change=p.V1815F;tissue=lung

asechange=c.1838C>T;AAchange=p.S613L;tissue=urinary_tract

=urinary_tract

>C;AAchange=p.N400H;tissue=large_intestine&DCLK1_ENST00000379893;basechange=c.277A>C;AAchange=p.N93H;tissue=large_intestine

nge=c.3704C>T;AAchange=p.S1235F;tissue=urinary_tract,large_intestine

>T;AAchange=p.R309C;tissue=large_intestine

C000-ZZP8UU

Chromosome	Position (hg	Reference al	Mutant allele	Sequence context	ANNOVAR fu	Gene	Mutation type	ANNOVAR output (alternative transcripts)	Cytoband	COSMIC
1	1163943	G	T	CCTCCTGGTG,AAGCCGCGAT	exonic	SDF4	nonsynonymous SNV	SDF4:ENST00000263741.7:exon2:c.C231A:p.F77L,SDF4:ENST00000360001.6:exon2:c.C231A:p.F77L,SDF4:ENST00000545427.1:exon2:c.C231A:p.F77L,SDF4:ENST00000465727.1:exon2:c.C231A:p.F77L,	1p36.33	.
1	1168401	G	T	CTGGGCGCCT,GCTGGCGCCG	exonic	B3GALT6	nonsynonymous SNV	B3GALT6:ENST00000379198.2:exon1:c.G743T:p.W248L,	1p36.33	.
1	12338017	G	A	CCCACCTTCC,GGTCTGGCAG	exonic	VPS13D	nonsynonymous SNV	VPS13D:ENST00000358136.3:exon19:c.G4372A:p.G1458R,VPS13D:ENST00000356315.4:exon19:c.G4372A:p.G1458R,	1p36.22	.
1	14107360	T	A	TCCAATTCTG,CCCCAACAGT	exonic	PRDM2	nonsynonymous SNV	PRDM2:ENST00000311066.5:exon8:c.T3070A:p.S1024T,PRDM2:ENST00000343137.4:exon4:c.T2467A:p.S823T,PRDM2:ENST00000413440.1:exon3:c.T2467A:p.S823T,PRDM2:ENST00000235372.7:exon8:c.T3070A:p.S1024T,	1p36.21	.
1	16255038	C	T	AGCTGTAGCT,ACTCTCCCCT	exonic	SPEN	nonsynonymous SNV	SPEN:ENST00000375759.3:exon11:c.C2303T:p.S768L,	1p36.13	.
1	16256619	T	G	GAAAAAGTCC,CCCCTATTCT	exonic	SPEN	nonsynonymous SNV	SPEN:ENST00000375759.3:exon11:c.T3884G:p.L1295R,	1p36.13	.
1	16261382	C	T	CGTGGCCACC,ATTCCACGTT	exonic	SPEN	nonsynonymous SNV	SPEN:ENST00000375759.3:exon11:c.C8647T:p.H2883Y,	1p36.13	.
1	19447860	G	T	CAGGAGAGCA,TTGCAGCAGC	exonic	UBR4	nonsynonymous SNV	UBR4:ENST00000375267.2:exon68:c.C9964A:p.L3322M,UBR4:ENST00000425413.1:exon21:c.C2788A:p.L930M,UBR4:ENST00000417040.1:exon41:c.C6022A:p.L2008M,UBR4:ENST00000375254.3:exon68:c.C9964A:p.L3322M,UBR4:ENST00000375217.2:exon68:c.C9943A:p.L3315M,UBR4:ENST000003	1p36.13	.
1	23285296	G	C	CCAGCGGGTT,TTAATGGTGA	exonic	LACTBL1	nonsynonymous SNV	LACTBL1:ENST00000426928.2:exon4:c.C435G:p.N145K,	1p36.12	.
1	23810597	G	T	TGACGGCCAG,AACTCGGCGA	exonic	ASAP3	nonsynonymous SNV	ASAP3:ENST00000437606.2:exon1:c.C30A:p.F10L,ASAP3:ENST00000608765.1:exon2:c.C30A:p.F10L,ASAP3:ENST00000336689.3:exon1:c.C30A:p.F10L,ASAP3:ENST00000492982.2:exon1:c.C30A:p.F10L,	1p36.12	.
1	24194727	A	G	GAGCAGCAGC,GCAACAGCGC	exonic	FUCA1	nonsynonymous SNV	FUCA1:ENST00000374479.3:exon1:c.T50C:p.L17P,	1p36.11	.
1	25883705	C	A	CGCCCAGAGC,AGCACAACCA	exonic	LDLRAP1	nonsynonymous SNV	LDLRAP1:ENST00000374338.4:exon4:c.C406A:p.Q136K,	1p36.11	.
1	28268785	G	A	TCTGTCTGCA,AGGAAGCGCA	splicing	SMPDL3B(EN	.	.	1p35.3	.
1	29475560	C	T	GGTTTCCCGA,ATTGTCATTG	exonic	SRSF4	nonsynonymous SNV	SRSF4:ENST00000373795.4:exon6:c.G847A:p.V283I,	1p35.3	.
1	33236755	G	A	TGACAGGTTT,TCATACCTCC	exonic	KIAA1522	nonsynonymous SNV	KIAA1522:ENST00000401073.2:exon6:c.G1975A:p.V659I,KIAA1522:ENST00000373480.1:exon6:c.G1798A:p.V600I,KIAA1522:ENST00000373481.3:exon6:c.G1831A:p.V611I,	1p35.1	.
1	36563703	G	A	GGGGGTCCCG,GGGCCCGGGA	exonic	COL8A2	nonsynonymous SNV	COL8A2:ENST00000397799.1:exon4:c.C1579T:p.P527S,COL8A2:ENST00000481785.1:exon2:c.C1384T:p.P462S,COL8A2:ENST00000303143.4:exon2:c.C1579T:p.P527S,	1p34.3	.
1	43296195	C	T	GCTGTCTGTG,ATGTGTCAGG	exonic	ERMAP	nonsynonymous SNV	ERMAP:ENST00000372514.3:exon2:c.C76T:p.H26Y,ERMAP:ENST00000372517.2:exon3:c.C76T:p.H26Y,	1p34.2	.
1	43651024	C	G	GCAGAGAAAT,AGGGTAAGGC	exonic	WDR65	nonsynonymous SNV	WDR65:ENST00000528956.1:exon5:c.C966G:p.I322M,WDR65:ENST00000372492.4:exon5:c.C966G:p.I322M,	1p34.2	.
1	43831270	A	C	CAAGTTCACA,CAGCCTCCAT	exonic	ELOVL1	nonsynonymous SNV	ELOVL1:ENST00000372458.3:exon2:c.T11G:p.V4G,ELOVL1:ENST00000413844.2:exon2:c.T11G:p.V4G,	1p34.2	.
1	43908593	G	T	GGGTCCTCTC,TGGTGGGGGT	exonic	SZT2	nonsynonymous SNV	SZT2:ENST00000372442.1:exon44:c.G5558T:p.R1853L,SZT2:ENST00000562955.1:exon58:c.G8084T:p.R2695L,	1p34.2	.
1	44069659	G	A	GGAGAGGAAC,GGCGCATCAT	exonic	PTPRF	nonsynonymous SNV	PTPRF:ENST00000372413.3:exon13:c.G2809A:p.G937R,PTPRF:ENST00000438120.1:exon15:c.G2809A:p.G937R,PTPRF:ENST00000422171.2:exon6:c.G880A:p.G294R,PTPRF:ENST00000372414.3:exon14:c.G2836A:p.G946R,PTPRF:ENST00000359947.4:exon16:c.G2836A:p.G946R,	1p34.2	.
1	52863470	C	A	CGTTTACAGG,AGGGACTTCA	exonic	ORC1	nonsynonymous SNV	ORC1:ENST00000371568.3:exon4:c.G289T:p.A97S,ORC1:ENST00000371566.1:exon4:c.G289T:p.A97S,	1p32.3	.
1	53236803	G	T	AAAGCTTTCT,CCACCACAAG	exonic	ZYG11B	nonsynonymous SNV	ZYG11B:ENST00000545132.1:exon3:c.G308T:p.C103F,ZYG11B:ENST00000443756.2:exon3:c.G308T:p.C103F,ZYG11B:ENST00000294353.6:exon3:c.G308T:p.C103F,	1p32.3	.
1	54427793	A	T	GACAGGGCTC,AGGTAAGACT	exonic	LRRC42	stopgain	LRRC42:ENST00000371370.3:exon6:c.A811T:p.K271X,LRRC42:ENST00000319223.4:exon5:c.A811T:p.K271X,	1p32.3	.
1	60499266	A	C	AATCTCCAAC,GACTCCTGTT	exonic	C1orf87	nonsynonymous SNV	C1orf87:ENST00000371201.3:exon7:c.T911G:p.L304R,	1p32.1	.
1	75102007	G	A	TGAGGTTTTT,ATCTGGACCT	exonic	C1orf173	nonsynonymous SNV	C1orf173:ENST00000326665.5:exon6:c.C560T:p.S187L,	1p31.1	.
1	93645791	G	T	GCCAGATCTT,TCGCCCATCC	exonic	TMED5	nonsynonymous SNV	TMED5:ENST00000370282.3:exon1:c.C9A:p.D3E,TMED5:ENST00000370280.1:exon1:c.C9A:p.D3E,TMED5:ENST00000479918.1:exon1:c.C9A:p.D3E,TMED5:ENST00000370290.3:exon1:c.C9A:p.D3E,	1p22.1	.
1	110086987	G	A	GCCTCACCCC,GCTGGAGTCA	exonic	GPR61	nonsynonymous SNV	GPR61:ENST00000527748.1:exon2:c.G1343A:p.R448Q,GPR61:ENST00000469383.2:exon2:c.G1343A:p.R448Q,GPR61:ENST00000404129.2:exon2:c.G1343A:p.R448Q,	1p13.3	.
1	120301829	A	T	AAAGCTTCCC,TCCACTATTG	exonic	HMGCS2	nonsynonymous SNV	HMGCS2:ENST00000544913.2:exon3:c.T636A:p.D212E,HMGCS2:ENST00000369406.3:exon4:c.T762A:p.D254E,	1p12	.
1	154303982	G	C	TCAATGGAAT,TGAGTGCCTG	splicing	ATP8B2(ENS	.	.	1q21.3	.
1	155263350	G	T	TGCCCTCGTT,GCCGGGGCTT	exonic	PKLR	nonsynonymous SNV	PKLR:ENST00000342741.4:exon8:c.C1148A:p.P383Q,PKLR:ENST00000392414.3:exon8:c.C1055A:p.P352Q,	1q22	.
1	156641701	T	G	TTGAGTCTCT,TTTCAAGAGT	exonic	NES	nonsynonymous SNV	NES:ENST00000368223.3:exon4:c.A2279C:p.K760T,	1q23.1	.
1	156641711	T	G	TTTTCAAGAG,TCTCAATGTC	exonic	NES	nonsynonymous SNV	NES:ENST00000368223.3:exon4:c.A2269C:p.T757P,	1q23.1	.
1	156838389	G	T	GCGGGGCCTG,AGCAGGCCGG	exonic	NTRK1	stopgain	NTRK1:ENST00000524377.1:exon6:c.G667T:p.E223X,NTRK1:ENST00000358660.3:exon6:c.G667T:p.E223X,NTRK1:ENST00000368196.3:exon6:c.G667T:p.E223X,NTRK1:ENST00000392302.2:exon7:c.G577T:p.E193X,NTRK1:ENST00000497019.2:exon7:c.G577T:p.E193X,	1q23.1	.
1	156907135	C	T	GCGGCTGGGG,GTCTTGGATC	exonic	ARHGEF11	nonsynonymous SNV	ARHGEF11:ENST00000361409.2:exon38:c.G4226A:p.R1409H,ARHGEF11:ENST00000368194.3:exon39:c.G4346A:p.R1449H,ARHGEF11:ENST00000315174.8:exon18:c.G2474A:p.R825H,	1q23.1	ARHGEF11;basechange=c.4346G>T;AAchange=p.R1449L;tissue=lung&ARHGEF11;basechange=c.4346G>A;AAchange=p.R1449H;tissue=lung
1	157738374	G	C	TCTGTACCAG,AGAATGTGAC	exonic	FCRL2	nonsynonymous SNV	FCRL2:ENST00000392274.3:exon5:c.C713G:p.S238C,FCRL2:ENST00000361516.3:exon5:c.C713G:p.S238C,	1q23.1	.
1	161771988	A	T	CCCTTCAGCG,ATAGCCCAGT	exonic	ATF6	nonsynonymous SNV	ATF6:ENST00000367942.3:exon7:c.A835T:p.N279Y,	1q23.3	.
1	161987249	G	C	AGCTTCAGGA,ATCCAGGCCA	exonic	OLFML2B	nonsynonymous SNV	OLFML2B:ENST00000294794.3:exon3:c.C487G:p.L163V,OLFML2B:ENST00000367940.2:exon3:c.C487G:p.L163V,	1q23.3	OLFML2B;basechange=c.487C>T;AAchange=p.L163F;tissue=skin
1	162825082	C	A	TTGAGGCCAG,ATCATGTGAA	exonic	C1orf110	nonsynonymous SNV	C1orf110:ENST00000367911.2:exon4:c.G367T:p.A123S,C1orf110:ENST00000367910.1:exon4:c.G382T:p.A128S,C1orf110:ENST00000367912.2:exon4:c.G379T:p.A127S,	1q23.3	.
1	169510872	C	G	TTTCACTGAG,TCTGGAGAAG	exonic	F5	nonsynonymous SNV	F5:ENST00000367796.3:exon13:c.G3471C:p.E1157D,F5:ENST00000367797.3:exon13:c.G3456C:p.E1152D,	1q24.2	.
1	173516871	G	C	CTCCATTAAA,CTTCATCTGT	exonic	SLC9C2	nonsynonymous SNV	SLC9C2:ENST00000536496.1:exon12:c.C1208G:p.A403G,SLC9C2:ENST00000367714.3:exon13:c.C1514G:p.A505G,	1q25.1	.
1	176809388	C	A	TGCTGCTCTT,CACACTCTCC	exonic	PAPPA2	nonsynonymous SNV	PAPPA2:ENST00000367662.3:exon22:c.C5282A:p.S1761Y,	1q25.2	.
1	177902740	G	C	GGAACTCTCA,AAATACTTCG	exonic	SEC16B	nonsynonymous SNV	SEC16B:ENST00000308284.6:exon21:c.C2603G:p.S868C,	1q25.2	.
1	180461436	C	T	AGTTTTTTAA,TACTGCGATA	exonic	ACBD6	nonsynonymous SNV	ACBD6:ENST00000367595.3:exon3:c.G352A:p.V118I,	1q25.3	.
1	186276796	G	T	TGAGGAGCTC,CACCCACCAC	exonic	PRG4	nonsynonymous SNV	PRG4:ENST00000367483.4:exon6:c.G1822T:p.A608S,PRG4:ENST00000367485.4:exon5:c.G1666T:p.A556S,PRG4:ENST00000367482.4:exon4:c.G1543T:p.A515S,PRG4:ENST00000445192.2:exon7:c.G1945T:p.A649S,PRG4:ENST00000367486.3:exon6:c.G1816T:p.A606S,	1q31.1	PRG4;basechange=c.1945G>A;AAchange=p.A649T;tissue=endometrium
1	201043667	T	A	CTCCTCAGCC,TGGCCTTCTG	exonic	CACNA1S	nonsynonymous SNV	CACNA1S:ENST00000362061.3:exon14:c.A2030T:p.K677M,CACNA1S:ENST00000367338.3:exon14:c.A2030T:p.K677M,	1q32.1	.
1	201056996	C	T	GAAGAAGGAT,CCAGCAAAAT	exonic	CACNA1S	nonsynonymous SNV	CACNA1S:ENST00000362061.3:exon7:c.G962A:p.G321E,CACNA1S:ENST00000367338.3:exon7:c.G962A:p.G321E,	1q32.1	.
1	201177538	G	T	ATGCCCTGGT,CTAAGGCCTC	exonic	IGFN1	nonsynonymous SNV	IGFN1:ENST00000335211.4:exon12:c.G3517T:p.A1173S,	1q32.1	.
1	201825040	A	C	ATTATCCTGT,CATGCAAATC	exonic	IPO9	nonsynonymous SNV	IPO9:ENST00000361565.4:exon10:c.A1100C:p.Y367S,	1q32.1	.
1	202719815	T	C	GTTAACAGCC,CAGCAAAATT	exonic	KDM5B	nonsynonymous SNV	KDM5B:ENST00000235790.4:exon13:c.A1319G:p.E440G,KDM5B:ENST00000367265.3:exon13:c.A1793G:p.E598G,KDM5B:ENST00000367264.2:exon14:c.A1901G:p.E634G,	1q32.1	.
1	204948522	C	A	AGACCAGTTC,AACCTGGGGT	exonic	NFASC	nonsynonymous SNV	NFASC:ENST00000367171.4:exon18:c.C1966A:p.Q656K,NFASC:ENST00000367172.4:exon19:c.C2011A:p.Q671K,NFASC:ENST00000367170.4:exon19:c.C2011A:p.Q671K,NFASC:ENST00000513543.1:exon16:c.C1999A:p.Q667K,NFASC:ENST00000430393.1:exon17:c.C1972A:p.Q658K,NFASC:ENST00	1q32.1	.
1	205129317	G	A	GAGGGCACAA,GGAAGTGTCC	exonic	DSTYK	nonsynonymous SNV	DSTYK:ENST00000367162.3:exon8:c.C2030T:p.P677L,DSTYK:ENST00000367161.3:exon8:c.C2030T:p.P677L,	1q32.1	.
1	208390424	G	A	TTGCAGAGCC,CACGATGCGT	exonic	PLXNA2	nonsynonymous SNV	PLXNA2:ENST00000367033.3:exon2:c.C844T:p.R282W,	1q32.2	.
1	209878364	A	C	GCAAACGAGG,ATTCAGACCA	exonic	HSD11B1	nonsynonymous SNV	HSD11B1:ENST00000367028.2:exon2:c.A77C:p.E26A,HSD11B1:ENST00000367027.3:exon1:c.A77C:p.E26A,HSD11B1:ENST00000261465.1:exon2:c.A77C:p.E26A,	1q32.2	.
1	222826581	C	G	CTTTGACTAA,TGCATTACAC	exonic	MIA3	nonsynonymous SNV	MIA3:ENST00000344441.6:exon15:c.C4221G:p.N1407K,MIA3:ENST00000340535.7:exon10:c.C855G:p.N285K,MIA3:ENST00000344922.5:exon15:c.C4221G:p.N1407K,	1q41	.
1	228547947	C	T	CCTTGGGGAT,GTGACCCCAA	exonic	OBSCN	nonsynonymous SNV	OBSCN:ENST00000366709.4:exon81:c.C10711T:p.R3571C,OBSCN:ENST00000284548.11:exon81:c.C19354T:p.R6452C,	1q42.13	.
1	230371818	C	G	GATCACGTTT,ACAATGAAGC	exonic	GALNT2	nonsynonymous SNV	GALNT2:ENST00000543760.1:exon4:c.C319G:p.H107D,GALNT2:ENST00000366672.4:exon4:c.C433G:p.H145D,GALNT2:ENST00000541865.1:exon3:c.C163G:p.H55D,	1q42.13	.
1	232597940	G	A	AGCACTCACC,AGCATAGTTT	exonic	SIPA1L2	nonsynonymous SNV	SIPA1L2:ENST00000308942.4:exon1:c.C41T:p.S14L,	1q42.2	.
1	235324383	C	G	AGGATTCTCT,TAGAAATGGA	splicing	ARID4B(ENS	.	.	1q42.3	.
1	240255695	G	C	CTCCCGCGAA,ATGTACTGGA	exonic	FMN2	nonsynonymous SNV	FMN2:ENST00000319653.9:exon1:c.G286C:p.D96H,	1q43	.
1	247588405	C	T	TTTGAAGCTT,CCAGCCGAGA	exonic	NLRP3	nonsynonymous SNV	NLRP3:ENST00000366496.2:exon3:c.C1660T:p.P554S,NLRP3:ENST00000348069.2:exon3:c.C1660T:p.P554S,NLRP3:ENST00000366497.2:exon4:c.C1660T:p.P554S,NLRP3:ENST00000391827.2:exon3:c.C1660T:p.P554S,NLRP3:ENST00000391828.3:exon5:c.C1660T:p.P554S,NLRP3:ENST0000033611	1q44	.
2	1271345	T	G	AGAACCGGGG,AAGTGAACAA	splicing	SNTG2(ENS	.	.	2p25.3	.
2	1926506	G	T	TCTCCTGCGG,TTGGTCTCAC	exonic	MYT1L	nonsynonymous SNV	MYT1L:ENST00000428368.2:exon10:c.C1035A:p.N345K,MYT1L:ENST00000399161.2:exon10:c.C1035A:p.N345K,	2p25.3	.
2	25141322	A	G	GTGATGAAGA,GGAGAAGACA	exonic	ADCY3	nonsynonymous SNV	ADCY3:ENST00000435135.1:exon2:c.T535C:p.F179L,ADCY3:ENST00000260600.5:exon1:c.T535C:p.F179L,	2p23.3	.
2	27324222	G	C	GTTTCCCCTG,AAGTTCCTTG	exonic	CGREF1	nonsynonymous SNV	CGREF1:ENST00000405600.1:exon6:c.C877G:p.P293A,CGREF1:ENST00000404694.3:exon5:c.C1243G:p.P415A,CGREF1:ENST00000312734.4:exon7:c.C877G:p.P293A,CGREF1:ENST00000402394.1:exon6:c.C877G:p.P293A,CGREF1:ENST00000260595.5:exon7:c.C826G:p.P276A,	2p23.3	.
2	27724008	T	G	TCAGGCTCCC,TTGTGGCAGG	exonic	GCKR	nonsynonymous SNV	GCKR:ENST00000453813.1:exon7:c.T472G:p.F158V,GCKR:ENST00000264717.2:exon8:c.T556G:p.F186V,	2p23.3	.
2	37193546	C	A	AGCCCCTTGG,ACCGCCGGCG	exonic	STRN	nonsynonymous SNV	STRN:ENST00000379213.2:exon1:c.G61T:p.A21S,STRN:ENST00000263918.4:exon1:c.G61T:p.A21S,	2p22.2	.
2	44428913	T	C	ATACAACGTG,TAATGGTTCA	exonic	PPM1B	nonsynonymous SNV	PPM1B:ENST00000409432.3:exon2:c.T575C:p.V192A,PPM1B:ENST00000419807.1:exon3:c.T575C:p.V192A,PPM1B:ENST00000409473.2:exon1:c.T575C:p.V192A,PPM1B:ENST00000282412.4:exon2:c.T575C:p.V192A,PPM1B:ENST00000378551.2:exon2:c.T575C:p.V192A,PPM1B:ENST00000409895.4:e	2p21	.
2	45640374	C	A	GTGACCTCAA,GTCTGCTGAA	exonic	SRBD1	nonsynonymous SNV	SRBD1:ENST00000263736.4:exon19:c.G2392T:p.V798F,SRBD1:ENST00000535761.1:exon11:c.G949T:p.V317F,	2p21	.
2	55177799	G	T	GCTGGCTCTA,ACCACGTGTT	exonic	EML6	nonsynonymous SNV	EML6:ENST00000356458.6:exon29:c.G4096T:p.D1366Y,	2p16.1	.
2	70188676	T	C	CGAAGCAGAG,GGGGATGACT	exonic	ASPRV1	nonsynonymous SNV	ASPRV1:ENST00000320256.4:exon1:c.A145G:p.T49A,	2p13.3	.
2	113832312	T	C	AAGATCTGCA,ACTTCCTAAC	exonic	IL1F10	nonsynonymous SNV	IL1F10:ENST00000337569.3:exon3:c.T131C:p.I44T,IL1F10:ENST00000341010.2:exon4:c.T131C:p.I44T,IL1F10:ENST00000393197.2:exon3:c.T131C:p.I44T,	2q13	.
2	114691908	C	T	GGAGAACGGA,GTTGACCGGT	exonic	ACTR3	nonsynonymous SNV	ACTR3:ENST00000263238.2:exon6:c.C485T:p.T162M,ACTR3:ENST00000536059.1:exon5:c.C299T:p.T100M,ACTR3:ENST00000535589.2:exon6:c.C332T:p.T111M,	2q14.1	.
2	124979318	C	T	CTGCTCTCTC,AATGGCTTTT	exonic	CNTNAP5	nonsynonymous SNV	CNTNAP5:ENST00000431078.1:exon2:c.C119T:p.P40L,	2q14.3	.
2	135694412	T	A	TTTCTGCAGA,AATATCGTCT	exonic	CCNT2	nonsynonymous SNV	CCNT2:ENST00000295238.6:exon3:c.T242A:p.I81K,CCNT2:ENST00000452839.1:exon3:c.T242A:p.I81K,CCNT2:ENST00000419781.1:exon3:c.T242A:p.I81K,CCNT2:ENST00000264157.5:exon3:c.T242A:p.I81K,CCNT2:ENST00000417175.1:exon3:c.T242A:p.I81K,	2q21.3	.
2	149542363	G	A	TTGTGCACAA,CAGTCCTCAG	exonic	EPC2	nonsynonymous SNV	EPC2:ENST00000258484.6:exon13:c.G2144A:p.S715N,	2q23.1	.
2	165561008	C	T	TCTGGAGGAA,TCCATCTACT	exonic	COBLL1	nonsynonymous SNV	COBLL1:ENST00000392717.2:exon9:c.G1285A:p.V429I,COBLL1:ENST00000342193.4:exon8:c.G1171A:p.V391I,COBLL1:ENST00000194871.6:exon11:c.G1369A:p.V457I,COBLL1:ENST00000375458.2:exon8:c.G1171A:p.V391I,COBLL1:ENST00000409184.3:exon9:c.G1285A:p.V429I,COBLL1:ENST00000	2q24.3	.
2	165561011	C	T	GGAGGAACTC,ATCTACTGGC	exonic	COBLL1	nonsynonymous SNV	COBLL1:ENST00000392717.2:exon9:c.G1282A:p.G428R,COBLL1:ENST00000342193.4:exon8:c.G1168A:p.G390R,COBLL1:ENST00000194871.6:exon11:c.G1366A:p.G456R,COBLL1:ENST00000375458.2:exon8:c.G1168A:p.G390R,COBLL1:ENST00000409184.3:exon9:c.G1282A:p.G428R,COBLL1:ENST	2q24.3	.
2	175201168	G	T	CGCGGCGGCA,CGGCCGGGGT	exonic	AC018470.1,	nonsynonymous SNV	SP9:ENST00000394967.2:exon2:c.G355T:p.A119S,	2q31.1	.
2	180810294	C	A	GTTTTTCTGA,CTTCTTTCTC	exonic	CWC22	nonsynonymous SNV	CWC22:ENST00000295749.6:exon20:c.G2289T:p.R763S,CWC22:ENST00000410053.3:exon20:c.G2289T:p.R763S,	2q31.3	.
2	191379259	C	G	CATAGGCAAA,TGAGGCACGG	exonic	TMEM194B	nonsynonymous SNV	TMEM194B:ENST00000409150.3:exon7:c.G873C:p.Q291H,	2q32.2	.
2	203052947	G	A	ACCCAAAAGC,AGAGAGAAGG	exonic	AC079354.1	nonsynonymous SNV	AC079354.1:ENST00000498697.1:exon11:c.G1198A:p.E400K,	2q33.1	.
2	211159081	G	T	GGTCCTTGTT,TTGGAAATGG	exonic	MYL1	nonsynonymous SNV	MYL1:ENST00000341685.4:exon4:c.C234A:p.N78K,MYL1:ENST00000352451.3:exon4:c.C366A:p.N122K,	2q34	MYL1;basechange=c.366C>A;AAchange=p.N122K;tissue=lung
2	219130327	C	A	AAGGACTCCA,CGAGTTGGAC	exonic	AAMP	nonsynonymous SNV	AAMP:ENST00000420660.1:exon7:c.G901T:p.V301L,AAMP:ENST00000248450.4:exon8:c.G958T:p.V320L,AAMP:ENST00000444053.1:exon8:c.G961T:p.V321L,	2q35	.
2	219494283	G	A	GTGGATGTAT,GGATGGACCT	exonic	PLCD4	stopgain	PLCD4:ENST00000450993.2:exon8:c.G1016A:p.W339X,PLCD4:ENST00000432688.1:exon8:c.G1016A:p.W339X,PLCD4:ENST00000417849.1:exon8:c.G1016A:p.W339X,	2q35	.
2	234669464	C	A	CACTGCCATG,AGCCTGGAAT	exonic	UGT1A1,UGT	stopgain	UGT1A8:ENST00000305208.5:exon1:c.C531A:p.C177X,UGT1A1:ENST00000608383.1:exon1:c.C531A:p.C177X,UGT1A1:ENST00000360418.3:exon1:c.C531A:p.C177X,	2q37.1	.
2	242573211	T	C	GCTCCTCTGC,GGTGGCGGCA	exonic	THAP4	nonsynonymous SNV	THAP4:ENST00000407315.1:exon2:c.A361G:p.S121G,	2q37.3	.
3	9517758	C	A	AGTCAAGACT,AGACGGGACT	exonic	SETD5	nonsynonymous SNV	SETD5:ENST00000402466.1:exon24:c.C4018A:p.Q1340K,SETD5:ENST00000302463.6:exon22:c.C4018A:p.Q1340K,SETD5:ENST00000402198.1:exon23:c.C4312A:p.Q1438K,SETD5:ENST00000407969.1:exon21:c.C4369A:p.Q1457K,SETD5:ENST00000406341.1:exon22:c.C4312A:p.Q1438K,	3p25.3	.
3	12963695	G	A	TTTCCTGAGG,CCTCATCCAG	exonic	IQSEC1	nonsynonymous SNV	IQSEC1:ENST00000273221.4:exon5:c.C1820T:p.A607V,IQSEC1:ENST00000429247.1:exon5:c.C1778T:p.A593V,	3p25.2	.
3	14725884	G	A	GGACAGTTGT,GAAAGGTGGG	exonic	C3orf20	stopgain	C3orf20:ENST00000435614.1:exon4:c.G254A:p.W85X,C3orf20:ENST00000253697.3:exon4:c.G620A:p.W207X,C3orf20:ENST00000412910.1:exon4:c.G254A:p.W85X,	3p25.1	.
3	37053568	A	G	TATTCGCTCC,TCTTTGGAAA	exonic	MLH1	nonsynonymous SNV	MLH1:ENST00000435176.1:exon8:c.A361G:p.I121V,MLH1:ENST00000231790.2:exon8:c.A655G:p.I219V,	3p22.2	MLH1;basechange=c.655A>G;AAchange=p.I219V;tissue=prostate
3	38739046	G	A	TCATCTGGGA,TGATGCAGCC	exonic	SCN10A	nonsynonymous SNV	SCN10A:ENST00000449082.2:exon27:c.C5665T:p.L1889F,	3p22.2	.
3	42787480	G	C	GATAGGAGCT,CTGAACGTTG	exonic	CCDC13	nonsynonymous SNV	CCDC13:ENST00000310232.6:exon7:c.C760G:p.Q254E,	3p22.1	.
3	44284944	A	G	TCAACATGAA,AAAATAAATA	exonic	TOPAZ1	nonsynonymous SNV	TOPAZ1:ENST00000309765.4:exon2:c.A946G:p.K316E,	3p21.31	.
3	45565528	G	T	CCCCGAGTTT,AGGATGCTTT	exonic	LARS2	stopgain	LARS2:ENST00000415258.1:exon19:c.G2332T:p.E778X,LARS2:ENST00000414984.1:exon18:c.G2203T:p.E735X,LARS2:ENST00000265537.3:exon20:c.G2332T:p.E778X,	3p21.31	.
3	45565529	A	T	CCCGAGTTTG,GGATGCTTTG	exonic	LARS2	nonsynonymous SNV	LARS2:ENST00000415258.1:exon19:c.A2333T:p.E778V,LARS2:ENST00000414984.1:exon18:c.A2204T:p.E735V,LARS2:ENST00000265537.3:exon20:c.A2333T:p.E778V,	3p21.31	.
3	47446551	C	G	TACGAGCAGG,CTGTATTCTC	exonic	PTPN23	nonsynonymous SNV	PTPN23:ENST00000265562.4:exon4:c.C344G:p.A115G,PTPN23:ENST00000456221.1:exon3:c.C269G:p.A90G,	3p21.31	.
3	48423247	G	A	ATCTGGATGG,AGCCAGTGAT	exonic	FBXW12	nonsynonymous SNV	FBXW12:ENST00000436231.1:exon6:c.G572A:p.G191E,FBXW12:ENST00000415155.1:exon8:c.G833A:p.G278E,FBXW12:ENST00000445170.1:exon8:c.G986A:p.G329E,FBXW12:ENST00000296438.5:exon9:c.G1043A:p.G348E,	3p21.31	.
3	48716317	G	T	CACCCCCTCT,TTCAGGAGCA	exonic	NCKIPSD	nonsynonymous SNV	NCKIPSD:ENST00000341520.4:exon11:c.C1785A:p.N595K,NCKIPSD:ENST00000416649.2:exon11:c.C1764A:p.N588K,NCKIPSD:ENST00000294129.2:exon11:c.C1785A:p.N595K,NCKIPSD:ENST00000413374.1:exon2:c.C153A:p.N51K,	3p21.31	.
3	49053257	T	C	TGTACCCGGT,GTAGTAGGAG	exonic	DALRD3	nonsynonymous SNV	DALRD3:ENST00000440857.1:exon11:c.A1091G:p.Q364R,DALRD3:ENST00000395462.4:exon12:c.A991G:p.N331D,DALRD3:ENST00000313778.5:exon11:c.A991G:p.N331D,DALRD3:ENST00000341949.4:exon11:c.A1492G:p.N498D,DALRD3:ENST00000441576.2:exon11:c.A1466G:p.Q489R,	3p21.31	.
3	49699480	G	C	GTCTCTCGGG,CAGGAAGTTC	exonic	BSN	nonsynonymous SNV	BSN:ENST00000296452.4:exon6:c.G10202C:p.G3401A,	3p21.31	.
3	49755494	G	A	ACTGCCAGCT,CACGCGGCCA	exonic	AMIGO3	stopgain	AMIGO3:ENST00000535833.1:exon10:c.C1405T:p.Q469X,AMIGO3:ENST00000320431.7:exon1:c.C1405T:p.Q469X,	3p21.31	.
3	49832788	G	A	CTCTAGGGGC,TGGTGGTCCG	exonic	CDHR4	nonsynonymous SNV	CDHR4:ENST00000412678.2:exon8:c.C872T:p.T291M,	3p21.31	.
3	51694099	C	A	ACTGGCAAAC,AAAGGTTCCT	exonic	RAD54L2	nonsynonymous SNV	RAD54L2:ENST00000409535.2:exon21:c.C3374A:p.P1125Q,RAD54L2:ENST00000296477.3:exon17:c.C2456A:p.P819Q,	3p21.2	.
3	53842745	C	A	GTCCCGTCGT,TCCCATCTTC	exonic	CACNA1D	nonsynonymous SNV	CACNA1D:ENST00000288139.4:exon47:c.C5879A:p.S1960Y,CACNA1D:ENST00000350061.5:exon46:c.C5819A:p.S1940Y,CACNA1D:ENST00000422281.2:exon44:c.C5747A:p.S1916Y,CACNA1D:ENST00000544977.1:exon7:c.C956A:p.S319Y,CACNA1D:ENST00000481478.1:exon41:c.C4898A:p.S1633Y,	3p21.1	.
3	57274571	G	C	TAAAGAAAGA,ATCTGAAAGG	exonic	APPL1	nonsynonymous SNV	APPL1:ENST00000495803.1:exon4:c.G292C:p.D98H,APPL1:ENST00000288266.3:exon5:c.G364C:p.D122H,APPL1:ENST00000444459.1:exon6:c.G313C:p.D105H,	3p14.3	.
3	89448644	C	G	TGTATTATTT,AATGCAGTCT	exonic	EPHA3	nonsynonymous SNV	EPHA3:ENST00000452448.2:exon7:c.C1608G:p.F536L,	3p11.1	.
3	112724619	A	G	GTACAGTGTG,GAGTCTCCAC	exonic	C3orf17	nonsynonymous SNV	C3orf17:ENST00000393857.2:exon8:c.T1060C:p.S354P,C3orf17:ENST00000383675.2:exon7:c.T1258C:p.S420P,C3orf17:ENST00000314400.5:exon9:c.T1468C:p.S490P,	3q13.2	C3orf17;basechange=c.1468T>C;AAchange=p.S490P;tissue=haematopoietic_and_lymphoid_tissue
3	119301081	G	A	TACTACAATG,GAAGTGGGAG	exonic	ADPRH	nonsynonymous SNV	ADPRH:ENST00000478927.1:exon2:c.G65A:p.G22E,ADPRH:ENST00000481816.1:exon3:c.G65A:p.G22E,ADPRH:ENST00000478399.1:exon2:c.G65A:p.G22E,ADPRH:ENST00000465513.1:exon1:c.G65A:p.G22E,ADPRH:ENST00000357003.3:exon3:c.G65A:p.G22E,	3q13.33	.
3	119528930	C	T	ACGCAACGCC,GGCTGAGGTG	exonic	NR1I2	nonsynonymous SNV	NR1I2:ENST00000466380.1:exon3:c.C220T:p.R74W,NR1I2:ENST00000393716.2:exon3:c.C220T:p.R74W,NR1I2:ENST00000337940.4:exon3:c.C337T:p.R113W,	3q13.33	.
3	121418718	G	T	AGAGTAATCT,AGAAGACAGT	exonic	GOLGB1	nonsynonymous SNV	GOLGB1:ENST00000393667.3:exon12:c.C1534A:p.Q512K,GOLGB1:ENST00000340645.5:exon12:c.C1519A:p.Q507K,GOLGB1:ENST00000494517.1:exon11:c.C1411A:p.Q471K,	3q13.33	.
3	135741921	A	T	GATGATGATC,AGGAAACATC	exonic	PPP2R3A	stopgain	PPP2R3A:ENST00000334546.2:exon1:c.A10T:p.K4X,	3q22.3	.
3	140397213	C	T	CAAAGATGAG,GGAACTGCCA	exonic	TRIM42	nonsynonymous SNV	TRIM42:ENST00000286349.3:exon1:c.C142T:p.R48W,	3q23	TRIM42;basechange=c.142C>A;AAchange=p.R48R;tissue=lung&TRIM42;basechange=c.142C>T;AAchange=p.R48W;tissue=large_intestine
3	141163572	C	G	AAGAAAACTA,ATCACATACC	exonic	ZBTB38	nonsynonymous SNV	ZBTB38:ENST00000441582.2:exon2:c.C2342G:p.T781R,ZBTB38:ENST00000514251.1:exon4:c.C2342G:p.T781R,ZBTB38:ENST00000321464.5:exon2:c.C2345G:p.T782R,	3q23	.
3	141163584	G	A	TCACATACCA,GGGAGAAATA	exonic	ZBTB38	nonsynonymous SNV	ZBTB38:ENST00000441582.2:exon2:c.G2354A:p.R785K,ZBTB38:ENST00000514251.1:exon4:c.G2354A:p.R785K,ZBTB38:ENST00000321464.5:exon2:c.G2357A:p.R786K,	3q23	.
3	142753779	C	T	TGCCACCTAT,GTACAATTCA	exonic	U2SURP	nonsynonymous SNV	U2SURP:ENST00000473835.2:exon19:c.C1903T:p.R635C,U2SURP:ENST00000480029.1:exon6:c.C604T:p.R202C,U2SURP:ENST00000493598.2:exon19:c.C1900T:p.R634C,U2SURP:ENST00000397933.2:exon18:c.C676T:p.R226C,	3q23	U2SURP;basechange=c.1903C>T;AAchange=p.R635C;tissue=ovary,large_intestine
3	146307541	C	A	ACATCATTTA,AAATCCTGAC	exonic	PLSCR5	nonsynonymous SNV	PLSCR5:ENST00000492200.1:exon6:c.G676T:p.V226L,PLSCR5:ENST00000482567.1:exon6:c.G640T:p.V214L,PLSCR5:ENST00000443512.1:exon6:c.G676T:p.V226L,	3q24	.
3	151056192	T	A	AATGCCCAGA,GACAACAGAG	exonic	P2RY12	nonsynonymous SNV	P2RY12:ENST00000302632.3:exon3:c.A442T:p.I148F,	3q25.1	.
3	151056254	G	T	AAATGGCCTG,TGGTCTTCTG	exonic	P2RY12	nonsynonymous SNV	P2RY12:ENST00000302632.3:exon3:c.C380A:p.T127N,	3q25.1	.
3	155628609	G	A	AACCTTCACC,TGCAGAACAG	exonic	GMPS	nonsynonymous SNV	GMPS:ENST00000496455.2:exon6:c.G655A:p.V219M,GMPS:ENST00000295920.7:exon4:c.G358A:p.V120M,	3q25.31	.
3	158539876	C	T	AGCAGCCACT,TTGTGTCCCA	exonic	MFSD1	nonsynonymous SNV	MFSD1:ENST00000392813.4:exon10:c.C1054T:p.L352F,MFSD1:ENST00000264266.8:exon11:c.C1024T:p.L342F,MFSD1:ENST00000477743.1:exon5:c.C469T:p.L157F,MFSD1:ENST00000415822.2:exon11:c.C1171T:p.L391F,	3q25.32	.
3	164700108	T	A	TTGACAGGAG,AGTTCCTTTC	exonic	SI	nonsynonymous SNV	SI:ENST00000264382.3:exon47:c.A5338T:p.T1780S,	3q26.1	.
3	164906088	C	T	TGAAACCCCA,CAACTGCTGG	exonic	SLITRK3	nonsynonymous SNV	SLITRK3:ENST00000475390.1:exon2:c.G2531A:p.G844D,SLITRK3:ENST00000241274.3:exon2:c.G2531A:p.G844D,	3q26.1	.
3	172835442	G	T	TTTCTTGCTT,TGTTTATCTT	exonic	SPATA16	nonsynonymous SNV	SPATA16:ENST00000351008.3:exon2:c.C80A:p.T27K,	3q26.31	.
3	173322479	C	T	CCCATTGACT,TCTGTATGTT	exonic	NLGN1	nonsynonymous SNV	NLGN1:ENST00000361589.4:exon2:c.C91T:p.L31F,NLGN1:ENST00000415045.1:exon2:c.C91T:p.L31F,NLGN1:ENST00000401917.3:exon1:c.C91T:p.L31F,NLGN1:ENST00000457714.1:exon3:c.C91T:p.L31F,NLGN1:ENST00000545397.1:exon1:c.C91T:p.L31F,	3q26.31	.
3	185643324	A	T	GATAATCTCG,CTGTAAGACC	exonic	TRA2B	nonsynonymous SNV	TRA2B:ENST00000453386.2:exon3:c.T261A:p.S87R,	3q27.2	.
3	194168588	A	C	TACCTCATTT,AAATGCTATT	exonic	ATP13A3	stopgain	ATP13A3:ENST00000256031.4:exon12:c.T1301G:p.L434X,ATP13A3:ENST00000439040.1:exon13:c.T1301G:p.L434X,	3q29	.
4	2260762	C	T	CCAGCCAGGG,TCCCGGGCCT	exonic	MXD4	nonsynonymous SNV	MXD4:ENST00000510822.1:exon3:c.G187A:p.A63T,	4p16.3	.
4	3039150	T	C	CAGTTCTCGG,GGTGAAAGGG	exonic	GRK4	nonsynonymous SNV	GRK4:ENST00000504933.1:exon14:c.T1457C:p.V486A,GRK4:ENST00000345167.6:exon13:c.T1361C:p.V454A,GRK4:ENST00000398052.4:exon14:c.T1457C:p.V486A,GRK4:ENST00000398051.4:exon13:c.T1361C:p.V454A,	4p16.3	.
4	5570268	G	A	GCCAGCAGCT,AGGCTGTGAG	exonic	EVC2	stopgain	EVC2:ENST00000310917.2:exon20:c.C3220T:p.Q1074X,EVC2:ENST00000475313.1:exon20:c.C3220T:p.Q1074X,EVC2:ENST00000344938.1:exon20:c.C3460T:p.Q1154X,EVC2:ENST00000344408.5:exon20:c.C3460T:p.Q1154X,	4p16.2	.
4	5838524	C	T	GTCATCCGCT,CTCTATCCCG	exonic	CRMP1	nonsynonymous SNV	CRMP1:ENST00000512574.1:exon10:c.G1072A:p.E358K,CRMP1:ENST00000397890.2:exon10:c.G1078A:p.E360K,CRMP1:ENST00000324989.7:exon10:c.G1420A:p.E474K,	4p16.2	.
4	6302519	G	A	CTTCTTCATC,TCAGCAACCT	exonic	WFS1	nonsynonymous SNV	WFS1:ENST00000503569.1:exon8:c.G997A:p.V333I,WFS1:ENST00000226760.1:exon8:c.G997A:p.V333I,	4p16.1	.
4	7725583	G	A	AGGCCACGAT,AGGCGGTGCT	exonic	SORCS2	nonsynonymous SNV	SORCS2:ENST00000329016.9:exon19:c.G2068A:p.E690K,SORCS2:ENST00000507866.2:exon19:c.G2584A:p.E862K,	4p16.1	.
4	13601337	G	A	CACGGGACCC,GTTCTTTGCC	exonic	BOD1L1	nonsynonymous SNV	BOD1L1:ENST00000040738.5:exon10:c.C7187T:p.P2396L,	4p15.33	BOD1L;basechange=c.7187C>T;AAchange=p.P2396L;tissue=stomach
4	37848730	G	A	TTTTCATTTT,AGGTAGGGTG	exonic	PGM2	nonsynonymous SNV	PGM2:ENST00000381967.4:exon9:c.G1186A:p.E396K,PGM2:ENST00000544359.1:exon10:c.G769A:p.E257K,PGM2:ENST00000537241.1:exon7:c.G706A:p.E236K,PGM2:ENST00000510084.2:exon1:c.G70A:p.E24K,	4p14	.
4	39448586	C	T	GCGGACTGGG,GGAACCCGCC	exonic	KLB	nonsynonymous SNV	KLB:ENST00000257408.4:exon4:c.C2240T:p.A747V,	4p14	.
4	41622535	G	C	GAAGCCTTCC,ATGGTGGGTG	exonic	LIMCH1	nonsynonymous SNV	LIMCH1:ENST00000503057.1:exon7:c.G553C:p.D185H,	4p13	.
4	42487657	C	T	AAGGGTAGTA,AGTGACGACT	exonic	ATP8A1	nonsynonymous SNV	ATP8A1:ENST00000381668.5:exon25:c.G2180A:p.C727Y,ATP8A1:ENST00000264449.10:exon24:c.G2135A:p.C712Y,	4p13	.
4	42571221	G	C	TCAGGGACAT,GCTTCAGAAA	exonic	ATP8A1	nonsynonymous SNV	ATP8A1:ENST00000381668.5:exon15:c.C1297G:p.H433D,	4p13	.
4	47887546	T	A	TGCTTGATCA,GACACGGAGC	exonic	NFXL1	nonsynonymous SNV	NFXL1:ENST00000329043.3:exon14:c.A1793T:p.H598L,NFXL1:ENST00000507489.1:exon14:c.A1793T:p.H598L,NFXL1:ENST00000381538.3:exon14:c.A1793T:p.H598L,NFXL1:ENST00000464756.2:exon14:c.A1793T:p.H598L,	4p12	.
4	47887553	G	A	TCATGACACG,AGCAGGACAC	exonic	NFXL1	nonsynonymous SNV	NFXL1:ENST00000329043.3:exon14:c.C1786T:p.P596S,NFXL1:ENST00000507489.1:exon14:c.C1786T:p.P596S,NFXL1:ENST00000381538.3:exon14:c.C1786T:p.P596S,NFXL1:ENST00000464756.2:exon14:c.C1786T:p.P596S,	4p12	.
4	71495242	G	A	GTGCAGGCTT,GAACCTCTTT	exonic	ENAM	nonsynonymous SNV	ENAM:ENST00000396073.3:exon2:c.G25A:p.G9R,	4q13.3	.
4	85639682	A	T	CCTCACTGGT,TCTAGGCCCT	exonic	WDFY3	nonsynonymous SNV	WDFY3:ENST00000322366.6:exon47:c.T7596A:p.D2532E,WDFY3:ENST00000295888.4:exon48:c.T7647A:p.D2549E,WDFY3:ENST00000514711.1:exon4:c.T456A:p.D152E,	4q21.23	.
4	96104115	C	G	ACAGAGTTTG,AAACCAGCTC	exonic	UNC5C	nonsynonymous SNV	UNC5C:ENST00000453304.1:exon14:c.G2384C:p.C795S,	4q22.3	.
4	96762047	G	T	TTTATCCCTG,GCTAAAGGTC	exonic	PDHA2	nonsynonymous SNV	PDHA2:ENST00000295266.4:exon1:c.G746T:p.G249V,	4q22.3	.
4	100570948	G	C	AGATAGTACT,GCCTGTTGTC	exonic	RP11-766F14	nonsynonymous SNV	RP11-766F14.2:ENST00000511828.1:exon1:c.C4858G:p.Q1620E,	4q23	.
4	100571919	C	A	CCTCTCCTCA,TGGCAATGAG	exonic	RP11-766F14	nonsynonymous SNV	RP11-766F14.2:ENST00000511828.1:exon1:c.G3887T:p.S1296I,	4q23	.
4	110890258	C	G	GCCGTAGATT,TATTGGACAG	exonic	EGF	nonsynonymous SNV	EGF:ENST00000509793.1:exon10:c.C1581G:p.F527L,EGF:ENST00000265171.5:exon11:c.C1707G:p.F569L,EGF:ENST00000503392.1:exon11:c.C1707G:p.F569L,	4q25	.
4	114274850	A	T	GTACACAGAA,CAGCACAAAC	exonic	ANK2	nonsynonymous SNV	ANK2:ENST00000503423.1:exon37:c.A4815T:p.K1605N,ANK2:ENST00000264366.6:exon37:c.A4977T:p.K1659N,ANK2:ENST00000504454.1:exon39:c.A5121T:p.K1707N,ANK2:ENST00000357077.4:exon38:c.A5076T:p.K1692N,	4q26	.
4	114275605	G	A	CCCTCCGGAA,AACGGACAAG	exonic	ANK2	nonsynonymous SNV	ANK2:ENST00000264366.6:exon37:c.G5732A:p.R1911K,ANK2:ENST00000357077.4:exon38:c.G5831A:p.R1944K,	4q26	.
4	114276880	T	C	GATAGTGAGG,TCAAGAATCC	exonic	ANK2	nonsynonymous SNV	ANK2:ENST00000264366.6:exon37:c.T7007C:p.V2336A,ANK2:ENST00000357077.4:exon38:c.T7106C:p.V2369A,	4q26	.
4	154557483	C	T	AAGCTTGTCT,CAATGTCTGG	exonic	KIAA0922	nonsynonymous SNV	KIAA0922:ENST00000409663.3:exon35:c.C4585T:p.P1529S,KIAA0922:ENST00000240487.5:exon29:c.C3919T:p.P1307S,KIAA0922:ENST00000409959.3:exon35:c.C4588T:p.P1530S,KIAA0922:ENST00000440693.1:exon33:c.C4336T:p.P1446S,	4q31.3	.
4	169145429	C	A	AAATCATCAT,AAAGTTCCCA	exonic	DDX60	nonsynonymous SNV	DDX60:ENST00000393743.3:exon35:c.G4789T:p.D1597Y,	4q32.3	.
4	169842823	G	A	ACGTGATGCC,GCATCTACAC	exonic	PALLD	nonsynonymous SNV	PALLD:ENST00000512127.1:exon16:c.G1792A:p.G598S,PALLD:ENST00000505667.1:exon18:c.G2989A:p.G997S,PALLD:ENST00000507735.1:exon9:c.G1477A:p.G493S,PALLD:ENST00000335742.7:exon18:c.G2464A:p.G822S,PALLD:ENST00000261509.6:exon17:c.G2938A:p.G980S,	4q32.3	.
5	3600288	C	G	CTTCCTGCAC,TCCACCACCG	exonic	IRX1	nonsynonymous SNV	IRX1:ENST00000302006.3:exon2:c.C1226G:p.P409R,	5p15.33	.
5	6742712	T	A	TACATGAAGG,ACTGTGCTTG	splicing	PAPD7(ENST	.	.	5p15.31	.
5	33576644	T	C	GGCTGTTCTC,TTCTTCCCCT	exonic	ADAMTS12	nonsynonymous SNV	ADAMTS12:ENST00000352040.3:exon17:c.A3232G:p.R1078G,ADAMTS12:ENST00000504830.1:exon19:c.A3487G:p.R1163G,	5p13.3	.
5	35641591	G	A	TAACCTTCTG,GTGTGCAGTT	exonic	SPEF2	nonsynonymous SNV	SPEF2:ENST00000356031.3:exon3:c.G220A:p.G74S,SPEF2:ENST00000505847.1:exon3:c.G220A:p.G74S,SPEF2:ENST00000440995.2:exon3:c.G220A:p.G74S,SPEF2:ENST00000509059.1:exon3:c.G220A:p.G74S,SPEF2:ENST00000282469.6:exon3:c.G220A:p.G74S,SPEF2:ENST00000510777.1:exon3:c	5p13.2	.
5	79366511	A	G	AGATGCAGAC,GAGATGGCAT	exonic	THBS4	nonsynonymous SNV	THBS4:ENST00000511733.1:exon12:c.A1225G:p.R409G,THBS4:ENST00000350881.2:exon12:c.A1498G:p.R500G,	5q14.1	.
5	89821054	C	T	TACTTGACCA,TTGACTGAAC	exonic	LYSMD3	nonsynonymous SNV	LYSMD3:ENST00000509384.1:exon2:c.G53A:p.S18N,LYSMD3:ENST00000315948.6:exon2:c.G53A:p.S18N,LYSMD3:ENST00000453259.2:exon2:c.G53A:p.S18N,LYSMD3:ENST00000500869.2:exon3:c.G53A:p.S18N,	5q14.3	.
5	96129535	T	C	GCCACAGTCA,TGTGATGCCA	exonic	ERAP1	nonsynonymous SNV	ERAP1:ENST00000296754.3:exon6:c.A1045G:p.M349V,ERAP1:ENST00000443439.2:exon6:c.A1045G:p.M349V,	5q15	.
5	98128903	G	A	TGGGGACAGC,ATGCCAAGAG	exonic	RGMB	nonsynonymous SNV	RGMB:ENST00000308234.7:exon5:c.G883A:p.D295N,RGMB:ENST00000513185.1:exon3:c.G760A:p.D254N,	5q15	.
5	112090699	G	T	TACAAAACTG,AAACTGAGGC	exonic	APC	stopgain	APC:ENST00000457016.1:exon2:c.G112T:p.E38X,APC:ENST00000257430.4:exon2:c.G112T:p.E38X,APC:ENST00000512211.2:exon2:c.G112T:p.E38X,APC:ENST00000508624.1:exon2:c.G112T:p.E38X,APC:ENST00000509732.1:exon2:c.G112T:p.E38X,APC:ENST00000508376.2:exon3:c.G112T:p.E38X,	5q22.2	.
5	112920015	C	A	GATGAAATCT,CATCTCCAGC	exonic	YTHDC2	nonsynonymous SNV	YTHDC2:ENST00000161863.4:exon26:c.C3664A:p.P1222T,	5q22.2	.
5	112920090	C	T	TACTGAGGAC,GATCAGATCA	exonic	YTHDC2	stopgain	YTHDC2:ENST00000161863.4:exon26:c.C3739T:p.R1247X,	5q22.2	.
5	128956429	G	A	ATGTAGCAAG,AAGATTTGGA	exonic	ADAMTS19	nonsynonymous SNV	ADAMTS19:ENST00000274487.4:exon9:c.G1579A:p.E527K,	5q23.3	.
5	129520429	A	G	AGTTAACCCC,TGCACGGGGT	exonic	CHSY3	nonsynonymous SNV	CHSY3:ENST00000305031.4:exon3:c.A1594G:p.M532V,	5q23.3	.
5	131927038	G	T	ACCAGGAGAT,GAGCAGTTAA	exonic	RAD50	nonsynonymous SNV	RAD50:ENST00000265335.6:exon10:c.G1575T:p.M525I,RAD50:ENST00000378823.3:exon10:c.G1158T:p.M386I,RAD50:ENST00000423956.1:exon10:c.G1575T:p.M525I,	5q31.1	.
5	132096884	G	C	ACAAACATCT,CCTCATCTCT	exonic	SEPT8	nonsynonymous SNV	SEPT8:ENST00000378706.1:exon8:c.C1036G:p.Q346E,SEPT8:ENST00000448933.1:exon8:c.C856G:p.Q286E,SEPT8:ENST00000378699.2:exon9:c.C856G:p.Q286E,SEPT8:ENST00000296873.7:exon8:c.C1036G:p.Q346E,SEPT8:ENST00000378719.2:exon8:c.C1036G:p.Q346E,SEPT8:ENST0000045848	5q31.1	.
5	132197649	C	T	GAAGCTGGGC,CAAGGGCTTC	exonic	GDF9	nonsynonymous SNV	GDF9:ENST00000296875.2:exon2:c.G997A:p.G333S,GDF9:ENST00000378673.2:exon3:c.G997A:p.G333S,	5q31.1	.
5	139947635	C	T	GCCGTGCTCT,AGCAGTCCTG	exonic	SLC35A4	nonsynonymous SNV	SLC35A4:ENST00000323146.3:exon3:c.C881T:p.S294L,SLC35A4:ENST00000514199.1:exon2:c.C881T:p.S294L,	5q31.3	SLC35A4;basechange=c.881C>T;AAchange=p.S294L;tissue=urinary_tract
5	140573239	G	T	TAAGATTAAT,ACAGAGACTC	exonic	PCDHB10	nonsynonymous SNV	PCDHB10:ENST00000239446.4:exon1:c.G1114T:p.D372Y,	5q31.3	.
5	140811559	T	A	TAGTCTTGGA,AGGGAACAGG	exonic	PCDHGA12	nonsynonymous SNV	PCDHGA12:ENST00000252085.3:exon1:c.T1233A:p.D411E,	5q31.3	.
5	140869291	G	C	GGATCCGGAT,TGGGCACCAA	exonic	PCDHGC5	nonsynonymous SNV	PCDHGC5:ENST00000252087.1:exon1:c.G484C:p.V162L,	5q31.3	.

5	150908813	G	A	AATTGGGGCC,GTGTTCATTG	exonic	FAT2	nonsynonymous SNV	FAT2:ENST00000261800.5:exon14:c.C9952T:p.R3318W,	5q33.1	.
5	154275786	G	A	TCCACGAAAG,CCCTTCGGTG	exonic	GEMIN5	nonsynonymous SNV	GEMIN5:ENST00000285873.7:exon24:c.C3463T:p.P1155S,	5q33.2	.
5	154346296	G	A	CCGCTACCAT,GCAGAGGTCG	exonic	MRPL22	nonsynonymous SNV	MRPL22:ENST00000523037.1:exon7:c.G460A:p.G154S,MRPL22:ENST00000439747.3:exon6:c.G538A:p.G180S,MRPL22:ENST00000265229.8:exon6:c.G220A:p.G74S,MRPL22:ENST00000522038.1:exon7:c.G478A:p.G160S,	5q33.2	.
5	160757976	A	C	CCCCTCCCAA,GAAGATGTAG	exonic	GABRB2	nonsynonymous SNV	GABRB2:ENST00000274547.2:exon9:c.T991G:p.F331V,GABRB2:ENST00000353437.6:exon9:c.T991G:p.F331V,GABRB2:ENST00000393959.1:exon8:c.T991G:p.F331V,GABRB2:ENST00000517901.1:exon8:c.T802G:p.F268V,GABRB2:ENST00000517547.1:exon6:c.T511G:p.F171V,GABRB2:ENST000005	5q34	.
5	167857146	G	A	TTTCAGGTAT,ATGAGAAGAA	exonic	WWC1	nonsynonymous SNV	WWC1:ENST00000521089.1:exon14:c.G2005A:p.D669N,WWC1:ENST00000265293.4:exon14:c.G2005A:p.D669N,	5q34	.
5	176637627	C	T	TCACAAACCC,AGTCAGATTT	exonic	NSD1	stopgain	NSD1:ENST00000439151.2:exon5:c.C2227T:p.Q743X,NSD1:ENST00000361032.4:exon2:c.C1918T:p.Q640X,NSD1:ENST00000354179.4:exon6:c.C1420T:p.Q474X,NSD1:ENST00000347982.4:exon6:c.C1420T:p.Q474X,	5q35.3	.
5	176638426	G	A	CCTGCTTCTG,TAAAAGTCGT	exonic	NSD1	nonsynonymous SNV	NSD1:ENST00000439151.2:exon5:c.G3026A:p.G1009D,NSD1:ENST00000361032.4:exon2:c.G2717A:p.G906D,NSD1:ENST00000354179.4:exon6:c.G2219A:p.G740D,NSD1:ENST00000347982.4:exon6:c.G2219A:p.G740D,	5q35.3	.
5	179041942	T	A	GTAGCTGCTG,TCACTGCTCC	exonic	HNRNPH1	nonsynonymous SNV	HNRNPH1:ENST00000329433.6:exon12:c.A1319T:p.N440I,	5q35.3	.
6	7387309	G	A	CATGGTATCC,ATTCTGACAT	exonic	CAGE1	nonsynonymous SNV	CAGE1:ENST00000379918.4:exon2:c.C98T:p.S33L,CAGE1:ENST00000512691.1:exon2:c.C134T:p.S45L,CAGE1:ENST00000512086.1:exon2:c.C98T:p.S33L,CAGE1:ENST00000442019.2:exon2:c.C98T:p.S33L,CAGE1:ENST00000338150.4:exon2:c.C98T:p.S33L,CAGE1:ENST00000458291.2:exon2:c.C98	6p24.3	.
6	7559511	C	T	CATCAGTGTC,CTCGAGTCCG	exonic	DSP	nonsynonymous SNV	DSP:ENST00000379802.3:exon4:c.C475T:p.P159S,DSP:ENST00000418664.2:exon4:c.C475T:p.P159S,	6p24.3	.
6	7584981	C	A	CTTCAAAGAA,TGTGTGAGCA	exonic	DSP	nonsynonymous SNV	DSP:ENST00000379802.3:exon24:c.C7486A:p.L2496M,DSP:ENST00000418664.2:exon24:c.C5689A:p.L1897M,	6p24.3	.
6	27279852	T	C	GTGAAGGGGC,GAGGTGGCCG	exonic	POM121L2	nonsynonymous SNV	POM121L2:ENST00000377451.2:exon1:c.A98G:p.Q33R,POM121L2:ENST00000444565.1:exon1:c.A98G:p.Q33R,	6p22.1	.
6	30553071	G	A	GCTGATGCTG,ATGAGCCCAC	exonic	ABCF1	nonsynonymous SNV	ABCF1:ENST00000376545.3:exon14:c.G1312A:p.D438N,ABCF1:ENST00000326195.8:exon15:c.G1426A:p.D476N,	6p21.33	.
6	30918734	T	G	GGACAGCCAA,GAGAAGACCA	exonic	DPCR1	nonsynonymous SNV	DPCR1:ENST00000462446.1:exon2:c.T2493G:p.N831K,	6p21.33	.
6	31681637	G	A	TGCACCCCGA,ATCCCGGGAT	exonic	LY6G6E	nonsynonymous SNV	LY6G6E:ENST00000409239.1:exon1:c.C28T:p.L10F,	6p21.33	.
6	31911254	C	T	CTGACAGCAG,TCATTGCTTC	exonic	C2,CFB	nonsynonymous SNV	C2:ENST00000497706.1:exon9:c.C878T:p.A293V,CFB:ENST00000556679.1:exon9:c.C1058T:p.A353V,C2:ENST00000442278.2:exon10:c.C1121T:p.A374V,CFB:ENST00000456570.1:exon9:c.C1058T:p.A353V,C2:ENST00000299367.5:exon12:c.C1517T:p.A506V,C2:ENST00000469372.1:exon8:c.C779T:p.A	6p21.33	.
6	31911260	G	T	GCAGCTCATT,CTTCCGCGAT	exonic	C2,CFB	nonsynonymous SNV	C2:ENST00000497706.1:exon9:c.G884T:p.C295F,CFB:ENST00000556679.1:exon9:c.G1064T:p.C355F,C2:ENST00000442278.2:exon10:c.G1127T:p.C376F,CFB:ENST00000456570.1:exon9:c.G1064T:p.C355F,C2:ENST00000299367.5:exon12:c.G1523T:p.C508F,C2:ENST00000469372.1:exon8:c.G785T:p.	6p21.33	.
6	33137249	C	A	CCTTTCTCTC,ACGTTCCCCG	exonic	COL11A2	stopgain	COL11A2:ENST00000374712.1:exon49:c.G3466T:p.G1156X,COL11A2:ENST00000357486.1:exon50:c.G3646T:p.G1216X,COL11A2:ENST00000361917.1:exon48:c.G3388T:p.G1130X,COL11A2:ENST00000374708.4:exon49:c.G3451T:p.G1151X,COL11A2:ENST00000395197.1:exon50:c.G3529T:p.G1177X	6p21.32	.
6	35209630	G	C	CAGCCCCAGG,CTGCTCCAGC	exonic	SCUBE3	nonsynonymous SNV	SCUBE3:ENST00000274938.7:exon12:c.G1360C:p.A454P,SCUBE3:ENST00000394681.1:exon13:c.G1408C:p.A470P,	6p21.31	.
6	36293083	G	A	TCTGTCCAGC,GAAGCTCCTC	exonic	C6orf222	nonsynonymous SNV	C6orf222:ENST00000437635.2:exon6:c.C1145T:p.P382L,	6p21.31	.
6	36449527	T	A	GGGGGAGGAA,ATTCCCAAAG	exonic	KCTD20	nonsynonymous SNV	KCTD20:ENST00000373731.2:exon6:c.T847A:p.Y283N,KCTD20:ENST00000449081.2:exon3:c.T349A:p.Y117N,KCTD20:ENST00000536244.1:exon5:c.T412A:p.Y138N,KCTD20:ENST00000544295.1:exon4:c.T109A:p.Y37N,	6p21.31	.
6	36931332	C	G	CCCAATACCT,TGCCACCGCT	exonic	PI16	nonsynonymous SNV	PI16:ENST00000373674.3:exon5:c.C1214G:p.S405C,	6p21.2	.
6	37443124	A	C	GATCAGTGCC,TCCACATCCT	exonic	CMTR1	nonsynonymous SNV	CMTR1:ENST00000373451.4:exon19:c.A1972C:p.I658L,	6p21.2	.
6	41029342	T	C	GACCGCATTA,CAAAACCCTT	exonic	APOBEC2	nonsynonymous SNV	APOBEC2:ENST00000244669.2:exon2:c.T407C:p.I136T,	6p21.1	.
6	41895168	G	C	GCCCACCCTG,AGAAGGCTGC	exonic	BYSL	nonsynonymous SNV	BYSL:ENST00000489290.1:exon2:c.G325C:p.E109Q,BYSL:ENST00000230340.4:exon2:c.G325C:p.E109Q,	6p21.1	.
6	43322794	G	C	ATGTGAAACT,AGATAAAGCA	exonic	ZNF318	nonsynonymous SNV	ZNF318:ENST00000605935.1:exon4:c.C2278G:p.Q760E,ZNF318:ENST00000318149.3:exon4:c.C2278G:p.Q760E,ZNF318:ENST00000361428.2:exon4:c.C2278G:p.Q760E,	6p21.1	.
6	43516188	C	A	TAAAGCATGT,AAAATTGGGC	exonic	XPO5	nonsynonymous SNV	XPO5:ENST00000265351.7:exon18:c.G1870T:p.D624Y,	6p21.1	.
6	46424652	T	G	TCCTCCATCT,CAGGGACGTG	exonic	RCAN2	nonsynonymous SNV	RCAN2:ENST00000405162.1:exon1:c.A62C:p.E21A,RCAN2:ENST00000306764.7:exon2:c.A62C:p.E21A,RCAN2:ENST00000371374.1:exon2:c.A62C:p.E21A,	6p12.3	.
6	49986739	C	T	GTAAGCAATC,TAATTTCATT	exonic	DEFB110	nonsynonymous SNV	DEFB110:ENST00000371148.2:exon2:c.G155A:p.R52K,	6p12.3	.
6	52938358	C	T	GAAAATCGAC,TTGCAGAGCA	exonic	FBXO9	nonsynonymous SNV	FBXO9:ENST00000370939.3:exon3:c.C68T:p.P23L,FBXO9:ENST00000498744.1:exon4:c.C68T:p.P23L,FBXO9:ENST00000473337.2:exon3:c.C68T:p.P23L,FBXO9:ENST00000459845.2:exon5:c.C68T:p.P23L,FBXO9:ENST00000323557.7:exon3:c.C170T:p.P57L,FBXO9:ENST00000244426.6:exon2:c.C2001	6p12.1	.
6	84885026	C	A	CTGGTTTATC,AAAGCTGCAG	exonic	KIAA1009	nonsynonymous SNV	KIAA1009:ENST00000403245.3:exon14:c.G1704T:p.L568F,KIAA1009:ENST00000257766.4:exon14:c.G1476T:p.L492F,	6q14.2	.
6	87970483	C	G	TCTGAGTGTA,AAGCAGATTT	exonic	ZNF292	nonsynonymous SNV	ZNF292:ENST00000339907.4:exon8:c.C7121G:p.T2374R,ZNF292:ENST00000369577.3:exon8:c.C7136G:p.T2379R,	6q14.3	.
6	99374640	C	G	CCAAATTCCA,ATAGTATAGG	exonic	FBXL4	nonsynonymous SNV	FBXL4:ENST00000229971.1:exon3:c.G225C:p.M75I,FBXL4:ENST00000369244.2:exon4:c.G225C:p.M75I,	6q16.1	.
6	106552831	C	G	AGGAAAGGAC,TCTACCGTTC	exonic	PRDM1	nonsynonymous SNV	PRDM1:ENST00000369091.2:exon5:c.C688G:p.L230V,PRDM1:ENST00000450060.1:exon3:c.C433G:p.L145V,PRDM1:ENST00000369089.3:exon3:c.C394G:p.L132V,PRDM1:ENST00000369096.4:exon5:c.C796G:p.L266V,	6q21	.
6	110763875	A	G	CAGGGTTCCA,CTGCAAAAAA	exonic	SLC22A16	nonsynonymous SNV	SLC22A16:ENST00000451557.1:exon3:c.T506C:p.V169A,SLC22A16:ENST00000434949.1:exon4:c.T245C:p.V82A,SLC22A16:ENST00000330550.4:exon5:c.T653C:p.V218A,SLC22A16:ENST00000437378.1:exon4:c.T626C:p.V209A,SLC22A16:ENST00000368919.3:exon4:c.T755C:p.V252A,SLC22A16:ENS	6q21	.
6	112390742	G	T	TTAAATGGAA,ATGCTGTGGA	exonic	WISP3	nonsynonymous SNV	WISP3:ENST00000368663.3:exon5:c.G915T:p.K305N,WISP3:ENST00000361714.1:exon5:c.G1038T:p.K346N,WISP3:ENST00000230529.5:exon6:c.G984T:p.K328N,WISP3:ENST00000604763.1:exon6:c.G984T:p.K328N,WISP3:ENST00000368666.2:exon5:c.G984T:p.K328N,WISP3:ENST00000409166.1:ex	6q21	.
6	119345816	T	G	AAAACTTGAA,CTTTCTTCTA	exonic	FAM184A	nonsynonymous SNV	FAM184A:ENST00000521531.1:exon2:c.A322C:p.I108L,FAM184A:ENST00000338891.7:exon2:c.A322C:p.I108L,	6q22.31	.
6	122779786	C	T	AGGACAGCAT,GGCATAGCAA	exonic	SERINC1	nonsynonymous SNV	SERINC1:ENST00000339697.4:exon2:c.G80A:p.R27Q,	6q22.31	.
6	127768665	C	T	GGAGGAGGAA,TGGTGGAGTT	exonic	KIAA0408	nonsynonymous SNV	KIAA0408:ENST00000483725.3:exon5:c.G799A:p.V267I,	6q22.33	.
6	138199835	T	G	CAAAACAAAC,CCCAAAGCTG	exonic	TNFAIP3	nonsynonymous SNV	TNFAIP3:ENST00000237289.4:exon7:c.T1253G:p.L418R,	6q23.3	.
6	142691549	A	C	TTCTGATTCA,AATGTTTGTT	exonic	GPR126	nonsynonymous SNV	GPR126:ENST00000367609.3:exon4:c.A688C:p.K230Q,GPR126:ENST00000541199.1:exon4:c.A685C:p.K229Q,GPR126:ENST00000230173.6:exon4:c.A688C:p.K230Q,GPR126:ENST00000296932.8:exon4:c.A688C:p.K230Q,GPR126:ENST00000367608.2:exon4:c.A688C:p.K230Q,	6q24.1	.
6	148664232	G	A	GCTGGCCCGG,GCCGAGGCCT	exonic	SASH1	nonsynonymous SNV	SASH1:ENST00000367467.3:exon1:c.G29A:p.G10E,	6q24.3	.
6	152646425	C	T	GAGTTAACCA,TGACACTAAA	exonic	SYNE1	nonsynonymous SNV	SYNE1:ENST00000367255.5:exon81:c.G15451A:p.V5151M,SYNE1:ENST00000265368.4:exon81:c.G15451A:p.V5151M,SYNE1:ENST00000341594.5:exon83:c.G14530A:p.V4844M,SYNE1:ENST00000448038.1:exon81:c.G15238A:p.V5080M,SYNE1:ENST00000423061.1:exon80:c.G15238A:p.V5080M,	6q25.2	.
6	167797869	C	G	ATCACCTCAC,TGGTCCCCGC	exonic	TCP10	nonsynonymous SNV	TCP10:ENST00000460930.2:exon1:c.G52C:p.G18R,	6q27	.
7	5364815	G	A	GGCCTCAGAG,GCAAGGCAGG	exonic	TNRC18	nonsynonymous SNV	TNRC18:ENST00000399537.4:exon20:c.C6212T:p.P2071L,TNRC18:ENST00000430969.1:exon20:c.C6212T:p.P2071L,	7p22.1	.
7	22767143	G	A	ACCCCCAGGA,AAGATTCCAA	exonic	IL6	nonsynonymous SNV	IL6:ENST00000426291.1:exon2:c.G100A:p.E34K,IL6:ENST00000258743.5:exon2:c.G100A:p.E34K,IL6:ENST00000420258.2:exon1:c.G262A:p.E88K,IL6:ENST00000406575.1:exon2:c.G100A:p.E34K,IL6:ENST00000401630.3:exon2:c.G31A:p.E11K,IL6:ENST00000404625.1:exon3:c.G100A:p.E34K,	7p15.3	IL6;basechange=c.100G>T;AAchange=p.E34*;tissue=endometrium
7	29544429	T	C	AAAATGGCAT,TGACAGAGGT	exonic	CHN2	nonsynonymous SNV	CHN2:ENST00000539389.1:exon7:c.T551C:p.F184S,CHN2:ENST00000446446.1:exon4:c.T458C:p.F153S,CHN2:ENST00000439711.2:exon4:c.T575C:p.F192S,CHN2:ENST00000495789.2:exon11:c.T1022C:p.F341S,CHN2:ENST00000409041.4:exon4:c.T575C:p.F192S,CHN2:ENST00000546235.1:exon10:	7p14.3	.
7	42949908	G	T	TGATCTAACA,AGCGTTGACT	exonic	C7orf25	nonsynonymous SNV	C7orf25:ENST00000447342.1:exon2:c.C592A:p.L198M,C7orf25:ENST00000438029.1:exon2:c.C592A:p.L198M,C7orf25:ENST00000431882.2:exon2:c.C766A:p.L256M,C7orf25:ENST00000350427.4:exon2:c.C592A:p.L198M,	7p14.1	.
7	47944077	A	G	CCAGCACTCA,AGGCCACACT	exonic	PKD1L1	nonsynonymous SNV	PKD1L1:ENST00000289672.2:exon12:c.T1829C:p.F610S,	7p12.3	.
7	48390407	T	A	GCAGAACAGC,TCTTGGCCAG	exonic	ABCA13	nonsynonymous SNV	ABCA13:ENST00000435803.1:exon30:c.T10372A:p.S3458I,	7p12.3	.
7	72754830	G	A	TTCAGGTGTG,ACAGGTGAGT	exonic	FKBP6	nonsynonymous SNV	FKBP6:ENST00000413573.2:exon5:c.G689A:p.G230E,FKBP6:ENST00000252037.4:exon6:c.G779A:p.G260E,FKBP6:ENST00000431982.2:exon6:c.G764A:p.G255E,FKBP6:ENST00000442793.1:exon5:c.G644A:p.G215E,	7q11.23	.
7	73011985	A	G	TTCAGGAAGG,GGAAATCAGA	exonic	MLXIPL	nonsynonymous SNV	MLXIPL:ENST00000354613.1:exon9:c.T1130C:p.L377P,MLXIPL:ENST00000395189.1:exon8:c.T851C:p.L284P,MLXIPL:ENST00000429400.2:exon9:c.T1130C:p.L377P,MLXIPL:ENST00000313375.3:exon9:c.T1130C:p.L377P,MLXIPL:ENST00000453275.1:exon4:c.T629C:p.L210P,MLXIPL:ENST0000043432	7q11.23	.
7	86416186	C	T	GCAAAAGTTT,AGTGCAGCCT	exonic	GRM3	stopgain	GRM3:ENST00000439827.1:exon3:c.C1078T:p.Q360X,GRM3:ENST00000394720.2:exon3:c.C1072T:p.Q358X,GRM3:ENST00000361669.2:exon3:c.C1078T:p.Q360X,GRM3:ENST00000536043.1:exon2:c.C694T:p.Q232X,	7q21.12	GRM3;basechange=c.1078C>A;AAchange=p.Q360K;tissue=large_intestine
7	91630628	G	A	ATGAAAACAC,GCATAAGGGA	exonic	AKAP9	nonsynonymous SNV	AKAP9:ENST00000356239.3:exon8:c.G1397A:p.R466Q,AKAP9:ENST00000359028.2:exon9:c.G1433A:p.R478Q,AKAP9:ENST00000358100.2:exon9:c.G1433A:p.R478Q,	7q21.2	.
7	91699452	G	A	TGAAGAAATA,AGAAACTGGA	exonic	AKAP9	nonsynonymous SNV	AKAP9:ENST00000356239.3:exon27:c.G6439A:p.E2147K,AKAP9:ENST00000359028.2:exon28:c.G6475A:p.E2159K,AKAP9:ENST00000394534.2:exon1:c.G1A:p.E1K,AKAP9:ENST00000358100.2:exon28:c.G6475A:p.E2159K,	7q21.2	.
7	94058557	A	T	ACTTGCCTTC,TGCGCCTGCT	exonic	COL1A2	nonsynonymous SNV	COL1A2:ENST00000297268.6:exon51:c.A3769T:p.M1257L,	7q21.3	.
7	96339050	T	A	CAGACCTAAG,CTACCGGCTG	exonic	SHFM1	nonsynonymous SNV	SHFM1:ENST00000449279.1:exon1:c.A26T:p.D9V,SHFM1:ENST00000444799.1:exon1:c.A26T:p.D9V,SHFM1:ENST00000248566.2:exon1:c.A26T:p.D9V,SHFM1:ENST00000413065.1:exon1:c.A26T:p.D9V,SHFM1:ENST00000417009.1:exon1:c.A26T:p.D9V,	7q21.3	.
7	99662237	G	A	GAGAAGCACC,TAACTTTCAA	exonic	ZSCAN21	nonsynonymous SNV	ZSCAN21:ENST00000456748.2:exon5:c.G1316A:p.R439H,ZSCAN21:ENST00000543588.1:exon6:c.G1316A:p.R439H,	7q22.1	.
7	100391591	A	G	TGGCACCCAG,GCCTCCCCTG	exonic	ZAN	nonsynonymous SNV	ZAN:ENST00000542585.1:exon43:c.A7999G:p.S2667G,	7q22.1	.
7	100686605	G	A	AACTTCCACT,AACTAAACAC	exonic	MUC17	nonsynonymous SNV	MUC17:ENST00000306151.4:exon3:c.G11908A:p.E3970K,MUC17:ENST00000379439.3:exon3:c.G11908A:p.E3970K,	7q22.1	.
7	102982303	C	T	TCTTTCTTAT,CTCCAGTTCC	exonic	DNAJC2	nonsynonymous SNV	DNAJC2:ENST00000249270.7:exon2:c.G163A:p.D55N,DNAJC2:ENST00000412522.1:exon2:c.G163A:p.D55N,DNAJC2:ENST00000379263.3:exon2:c.G163A:p.D55N,DNAJC2:ENST00000379257.3:exon2:c.G163A:p.D55N,	7q22.1	.
7	104767466	G	T	GTCTTCCCCA,AATGTGGTTC	exonic	SRPK2	nonsynonymous SNV	SRPK2:ENST00000465072.1:exon2:c.C47A:p.S16Y,SRPK2:ENST00000393651.3:exon14:c.C1796A:p.S599Y,SRPK2:ENST00000357311.3:exon13:c.C1763A:p.S588Y,SRPK2:ENST00000489828.1:exon15:c.C1763A:p.S588Y,	7q22.3	.
7	107866678	T	G	AGAAATAGGT,GCTTCTGCTG	exonic	NRCAM	nonsynonymous SNV	NRCAM:ENST00000425651.2:exon6:c.A695C:p.Q232P,NRCAM:ENST00000379022.4:exon10:c.A695C:p.Q232P,NRCAM:ENST00000379028.3:exon9:c.A695C:p.Q232P,NRCAM:ENST00000379024.4:exon9:c.A695C:p.Q232P,NRCAM:ENST00000413765.2:exon10:c.A695C:p.Q232P,NRCAM:ENST0000041	7q31.1	.
7	113518020	C	T	GATTCCTTTT,ACCTGAAGTG	exonic	PPP1R3A	nonsynonymous SNV	PPP1R3A:ENST00000284601.3:exon4:c.G3127A:p.E1043K,	7q31.1	.
7	113519772	C	G	ATTAGTTGAT,TGAAGAGGGG	exonic	PPP1R3A	nonsynonymous SNV	PPP1R3A:ENST00000449795.1:exon4:c.G412C:p.D138H,PPP1R3A:ENST00000284601.3:exon4:c.G1375C:p.D459H,	7q31.1	.
7	117232027	G	C	CTAGGATTTT,GTCACTTCTA	exonic	CFTR	nonsynonymous SNV	CFTR:ENST00000003084.6:exon14:c.G1806C:p.L602F,CFTR:ENST00000426809.1:exon13:c.G1716C:p.L572F,CFTR:ENST00000454343.1:exon13:c.G1623C:p.L541F,	7q31.2	.
7	119915782	G	A	GTATACCATC,TCACCATGAC	exonic	KCND2	nonsynonymous SNV	KCND2:ENST00000331113.4:exon1:c.G1096A:p.V366I,	7q31.31	KCND2;basechange=c.1096G>A;AAchange=p.V366I;tissue=breast
7	128415741	T	A	TGCCTGGAGG,AGAAGGCCCA	exonic	OPN1SW	nonsynonymous SNV	OPN1SW:ENST00000249389.2:exon1:c.A104T:p.Y35F,	7q32.1	.
7	128415787	G	A	CCATCCCACG,CCCCACTGAA	exonic	OPN1SW	nonsynonymous SNV	OPN1SW:ENST00000249389.2:exon1:c.C58T:p.P20S,	7q32.1	.
7	128493011	G	A	AGCAAGGTGC,GGTCTGGGGC	exonic	FLNC	nonsynonymous SNV	FLNC:ENST00000346177.6:exon36:c.G6035A:p.R2012Q,FLNC:ENST00000325888.8:exon37:c.G6134A:p.R2045Q,	7q32.1	.
7	135387612	G	A	CATCAGAGTG,TGAGGTCTGC	exonic	SLC13A4	nonsynonymous SNV	SLC13A4:ENST00000354042.4:exon6:c.C611T:p.T204I,	7q33	.
7	139285231	G	T	GCCGCATCAC,TGGGCCACAC	exonic	HIPK2	nonsynonymous SNV	HIPK2:ENST00000428878.2:exon11:c.C2286A:p.H762Q,HIPK2:ENST00000406875.3:exon11:c.C2367A:p.H789Q,HIPK2:ENST00000342645.6:exon11:c.C2367A:p.H789Q,	7q34	.
7	142919946	A	T	CTACATTTTC,ATGCAATTGC	exonic	TAS2R40	nonsynonymous SNV	TAS2R40:ENST00000408947.3:exon1:c.A775T:p.N259Y,	7q34	.
7	148768830	C	T	CTGGGGCAGG,GCGAGCCTGG	exonic	ZNF786	nonsynonymous SNV	ZNF786:ENST00000316286.9:exon3:c.G776A:p.R259H,ZNF786:ENST00000491431.1:exon4:c.G1034A:p.R345H,ZNF786:ENST00000451334.3:exon5:c.G923A:p.R308H,	7q36.1	.
7	149558728	G	A	CCGGGCCAAA,GGATGCTGAA	exonic	ZNF862	nonsynonymous SNV	ZNF862:ENST00000223210.4:exon7:c.G2479A:p.G827R,	7q36.1	.
7	151292537	A	T	TGAGTCTTCT,CTGCTAAAAG	exonic	PRKAG2	stoploss	PRKAG2:ENST00000488258.1:exon5:c.T688A:p.X230K,	7q36.1	.
7	158443549	A	T	ACTTATCTCA,CCACAGGCCC	exonic	NCAPG2	nonsynonymous SNV	NCAPG2:ENST00000356309.3:exon24:c.T3050A:p.V1017D,NCAPG2:ENST00000409339.3:exon24:c.T3050A:p.V1017D,NCAPG2:ENST00000449727.2:exon25:c.T3050A:p.V1017D,NCAPG2:ENST00000409423.1:exon25:c.T3050A:p.V1017D,	7q36.3	.
8	3351167	C	T	GATCTGGTGT,TCCCACCTTC	exonic	CSMD1	nonsynonymous SNV	CSMD1:ENST00000542608.1:exon11:c.G1426A:p.D476N,CSMD1:ENST00000602557.1:exon12:c.G1429A:p.D477N,CSMD1:ENST00000537824.1:exon11:c.G1426A:p.D476N,CSMD1:ENST00000539096.1:exon11:c.G1426A:p.D476N,CSMD1:ENST00000400186.3:exon12:c.G1429A:p.D477N,CSMD1:ENST	8p23.2	.
8	20022619	C	G	CCTTGGCACT,TGAGAACATG	splicing	SLC18A1(EN	.	.	8p21.3	.
8	22106718	G	T	GCAGCTGCCA,ACACCCTCCC	exonic	POLR3D	nonsynonymous SNV	POLR3D:ENST00000397802.4:exon6:c.G817T:p.D273Y,POLR3D:ENST00000306433.4:exon7:c.G817T:p.D273Y,	8p21.3	.
8	22273443	G	T	TGGACGAGAA,GTCATTGTGG	exonic	SLC39A14	nonsynonymous SNV	SLC39A14:ENST00000359741.5:exon6:c.G912T:p.K304N,SLC39A14:ENST00000240095.6:exon6:c.G912T:p.K304N,SLC39A14:ENST00000381237.1:exon6:c.G912T:p.K304N,SLC39A14:ENST00000517370.1:exon3:c.G381T:p.K127N,SLC39A14:ENST00000289952.5:exon6:c.G912T:p.K304N,	8p21.3	.
8	28321324	C	A	GAGAGTCTGT,CATTTGTCAA	exonic	FBXO16	nonsynonymous SNV	FBXO16:ENST00000517673.1:exon4:c.G147T:p.W49C,FBXO16:ENST00000346498.2:exon3:c.G111T:p.W37C,FBXO16:ENST00000380254.2:exon4:c.G147T:p.W49C,FBXO16:ENST00000521548.1:exon4:c.G147T:p.W49C,FBXO16:ENST00000518734.1:exon3:c.G111T:p.W37C,	8p21.1	.
8	39840234	A	G	GCTCCCTCCT,TCCTGGTCCA	exonic	IDO2	nonsynonymous SNV	IDO2:ENST00000502986.2:exon5:c.A418G:p.I140V,IDO2:ENST00000389060.4:exon4:c.A379G:p.I127V,	8p11.21	.
8	41530344	T	A	GCCACCTCAT,CCAGTACTGG	exonic	ANK1	nonsynonymous SNV	ANK1:ENST00000379758.2:exon38:c.A4624T:p.N1542Y,ANK1:ENST00000289734.7:exon38:c.A4624T:p.N1542Y,ANK1:ENST00000396945.1:exon38:c.A4624T:p.N1542Y,ANK1:ENST00000265709.8:exon39:c.A4747T:p.N1583Y,ANK1:ENST00000347528.4:exon38:c.A4624T:p.N1542Y,ANK1:ENST0000039	8p11.21	.
8	55533561	T	C	TTTTCCATCA,TCATCCTACG	exonic	RP1	nonsynonymous SNV	RP1:ENST00000220676.1:exon2:c.T35C:p.I12T,	8q12.1	.
8	72964914	G	T	CCTGCTGCTT,TTCAGGACTA	exonic	TRPA1	nonsynonymous SNV	TRPA1:ENST00000523582.1:exon11:c.C1287A:p.N429K,TRPA1:ENST00000262209.4:exon14:c.C1731A:p.N577K,	8q13.3	.
8	75227133	C	A	CTGGCCATGG,AGCTGCCCTT	exonic	JPH1	nonsynonymous SNV	JPH1:ENST00000342232.4:exon2:c.G1102T:p.A368S,	8q21.11	.
8	77616405	G	A	GACACAACTT,ATAATGAGGT	exonic	ZFHX4	nonsynonymous SNV	ZFHX4:ENST00000521891.2:exon2:c.G82A:p.D28N,ZFHX4:ENST00000050961.6:exon2:c.G82A:p.D28N,ZFHX4:ENST00000523885.1:exon2:c.G82A:p.D28N,ZFHX4:ENST00000520307.1:exon2:c.G82A:p.D28N,ZFHX4:ENST00000518282.1:exon1:c.G82A:p.D28N,ZFHX4:ENST00000517585.1:exon2:c.G82A	8q21.11	.
8	88298844	A	T	GGCCTACTTT,TCCATATATG	exonic	CNBD1	nonsynonymous SNV	CNBD1:ENST00000518476.1:exon8:c.A987T:p.L329F,	8q21.3	.
8	95680203	C	T	AGCCCAGTTT,TCTCCAAGGA	exonic	ESRP1	nonsynonymous SNV	ESRP1:ENST00000517610.1:exon7:c.C535T:p.L179F,ESRP1:ENST00000454170.2:exon10:c.C958T:p.L320F,ESRP1:ENST00000358397.5:exon10:c.C958T:p.L320F,ESRP1:ENST00000433389.2:exon10:c.C958T:p.L320F,ESRP1:ENST00000423620.2:exon10:c.C958T:p.L320F,	8q22.1	.
8	100865934	C	G	AGTCCAATTT,CACTTTGCTG	exonic	VPS13B	nonsynonymous SNV	VPS13B:ENST00000358544.2:exon56:c.C10392G:p.F3464L,VPS13B:ENST00000357162.2:exon56:c.C10317G:p.F3439L,	8q22.2	.
8	105257242	G	C	GGCAAGCCGA,AGTCTACAGA	exonic	RIMS2	nonsynonymous SNV	RIMS2:ENST00000406091.3:exon20:c.G3433C:p.E1145Q,RIMS2:ENST00000436393.2:exon24:c.G3487C:p.E1163Q,RIMS2:ENST00000339750.2:exon2:c.G241C:p.E81Q,RIMS2:ENST00000523362.1:exon2:c.G241C:p.E81Q,RIMS2:ENST00000262231.10:exon17:c.G2950C:p.E984Q,RIMS2:ENST00000507	8q22.3	.
8	110657533	C	T	CCCAGCCAAG,CAGTGCGTCA	exonic	RP11-422N1(	nonsynonymous SNV	RP11-422N16.3:ENST00000499579.1:exon2:c.C284T:p.A95V,	8q23.2	.
8	114186077	C	T	TCCCCGACGT,GAATCTTGTG	exonic	CSMD3	nonsynonymous SNV	CSMD3:ENST00000455883.2:exon4:c.G583A:p.D195N,CSMD3:ENST00000297405.5:exon4:c.G583A:p.D195N,CSMD3:ENST00000352409.3:exon4:c.G583A:p.D195N,CSMD3:ENST00000343508.3:exon5:c.G463A:p.D155N,	8q23.3	.
8	131124559	T	C	TCTTTCTTGA,AGGGCTTGGC	exonic	ASAP1	nonsynonymous SNV	ASAP1:ENST00000357668.1:exon23:c.A2182G:p.I728V,ASAP1:ENST00000518721.1:exon24:c.A2182G:p.I728V,	8q24.21	ASAP1;basechange=c.2182A>G;AAchange=p.I728V;tissue=stomach
8	144790152	T	C	GTCCAGCTGC,CCGAGAGCAG	exonic	CCDC166	nonsynonymous SNV	CCDC166:ENST00000542437.1:exon1:c.A128G:p.E43G,	8q24.3	.
8	145057848	C	T	ACAGGCTCCT,CTCCTCATGC	exonic	PARP10	nonsynonymous SNV	PARP10:ENST00000524918.1:exon8:c.G1882A:p.E628K,PARP10:ENST00000313028.7:exon8:c.G1909A:p.E637K,PARP10:ENST00000525773.1:exon7:c.G1945A:p.E649K,PARP10:ENST00000527262.1:exon8:c.G1909A:p.E637K,	8q24.3	.
9	2083361	G	A	CTATCTAACT,GACATATGAA	exonic	SMARCA2	stopgain	SMARCA2:ENST00000382203.1:exon16:c.G2363A:p.W788X,SMARCA2:ENST00000382194.1:exon15:c.G2363A:p.W788X,SMARCA2:ENST00000357248.2:exon16:c.G2363A:p.W788X,SMARCA2:ENST00000349721.2:exon16:c.G2363A:p.W788X,	9p24.3	.
9	21333649	C	G	CGCCCACCAA,TGCATACAAA	exonic	KLHL9	nonsynonymous SNV	KLHL9:ENST00000359039.4:exon1:c.G1210C:p.V404L,KLHL9:ENST00000537938.1:exon2:c.G1006C:p.V336L,	9p21.3	.
9	33221004	C	T	GAGCTCACCT,ACCTTCTCTG	exonic	SPINK4	nonsynonymous SNV	SPINK4:ENST00000379725.1:exon2:c.C28T:p.H10Y,SPINK4:ENST00000379723.1:exon1:c.C28T:p.H10Y,	9p13.3	.
9	34397596	G	C	TGTAGGTACT,ATAGGGGTCC	exonic	C9orf24	nonsynonymous SNV	C9orf24:ENST00000297623.2:exon1:c.C36G:p.I12M,	9p13.3	.
9	35378404	G	C	TTGGAAACTT,AATCCTGTTT	exonic	UNC13B	nonsynonymous SNV	UNC13B:ENST00000378495.3:exon16:c.G1929C:p.L643F,UNC13B:ENST00000396787.1:exon17:c.G1965C:p.L655F,UNC13B:ENST00000378496.4:exon16:c.G1929C:p.L643F,	9p13.3	.
9	35560794	G	A	GGCATCAAGT,GGGACACCTC	exonic	RUSC2	stopgain	RUSC2:ENST00000361226.3:exon10:c.G4157A:p.W1386X,RUSC2:ENST00000455600.1:exon10:c.G4157A:p.W1386X,	9p13.3	.
9	36665487	T	G	AAGAGTACTT,ATGTTTCCTG	exonic	MELK	nonsynonymous SNV	MELK:ENST00000536860.1:exon13:c.T1173G:p.F391L,MELK:ENST00000536987.1:exon11:c.T924G:p.F308L,MELK:ENST00000538311.1:exon14:c.T735G:p.F245L,MELK:ENST00000545008.1:exon12:c.T1104G:p.F368L,MELK:ENST00000543751.1:exon13:c.T1221G:p.F407L,MELK:ENST00000298048.2:e	9p13.2	.
9	93375803	G	T	TCGCAGATTT,TTCGTAGATG	exonic	DIRAS2	nonsynonymous SNV	DIRAS2:ENST00000375765.3:exon2:c.C307A:p.Q103K,	9q22.2	.
9	97062765	A	C	CACTAATCAC,AGAGGATTCA	exonic	ZNF169	nonsynonymous SNV	ZNF169:ENST00000395395.2:exon5:c.A925C:p.K309Q,	9q22.32	.
9	115336778	G	A	AGAATATGAA,ATGAGAACAC	exonic	KIAA1958	nonsynonymous SNV	KIAA1958:ENST00000337530.6:exon2:c.G418A:p.D140N,KIAA1958:ENST00000536272.1:exon2:c.G418A:p.D140N,KIAA1958:ENST00000374244.3:exon2:c.G418A:p.D140N,	9q32	.
9	117853056	G	A	GCTGGGCTCT,AAGGCGGTGC	exonic	TNC	nonsynonymous SNV	TNC:ENST00000345230.3:exon2:c.C242T:p.S81L,TNC:ENST00000537320.1:exon1:c.C242T:p.S81L,TNC:ENST00000341037.4:exon1:c.C242T:p.S81L,TNC:ENST00000346706.3:exon2:c.C242T:p.S81L,TNC:ENST00000534839.1:exon3:c.C242T:p.S81L,TNC:ENST00000542877.1:exon1:c.C242T:p.S81L,TN	9q33.1	.
9	125863897	G	A	AAAGAAGGGC,TGTAAAAGGC	exonic	RABGAP1	nonsynonymous SNV	RABGAP1:ENST00000373643.5:exon13:c.G959A:p.R320H,RABGAP1:ENST00000373647.4:exon25:c.G2942A:p.R981H,	9q33.3	.
9	126132483	G	T	ATCTGCAGGT,CCCAGAGACC	exonic	CRB2	nonsynonymous SNV	CRB2:ENST00000359999.3:exon7:c.G1151T:p.C384F,CRB2:ENST00000373629.2:exon2:c.G155T:p.C52F,CRB2:ENST00000460253.1:exon2:c.G155T:p.C52F,CRB2:ENST00000373631.3:exon7:c.G1151T:p.C384F,	9q33.3	.
9	129595735	A	T	GAGCAGGTGG,TTTCTATGGC	exonic	ZBTB43	nonsynonymous SNV	ZBTB43:ENST00000373464.4:exon3:c.A947T:p.D316V,ZBTB43:ENST00000373457.1:exon1:c.A947T:p.D316V,ZBTB43:ENST00000449886.1:exon2:c.A947T:p.D316V,	9q33.3	.
9	135158682	C	T	GAAGGGGACA,CCCCTTGCCC	exonic	SETX	nonsynonymous SNV	SETX:ENST00000372169.2:exon19:c.G6515A:p.G2172D,SETX:ENST00000224140.5:exon19:c.G6515A:p.G2172D,SETX:ENST00000436441.1:exon9:c.G1241A:p.G414D,SETX:ENST00000393220.1:exon19:c.G6515A:p.G2172D,	9q34.13	.
9	135602889	A	G	CCGCTCCATG,TGGAATCAAA	exonic	AK8	nonsynonymous SNV	AK8:ENST00000298545.3:exon12:c.T1154C:p.I385T,	9q34.13	.
9	135772638	C	T	CCATCTTTCT,CAACCTGCCA	exonic	TSC1	nonsynonymous SNV	TSC1:ENST00000545250.1:exon19:c.G2755A:p.E919K,TSC1:ENST00000440111.2:exon20:c.G2908A:p.E970K,TSC1:ENST00000298552.3:exon22:c.G2908A:p.E970K,	9q34.13	.
9	139649962	C	T	GTGGATCTCT,TGTGGCCTTC	exonic	LCN8	nonsynonymous SNV	LCN8:ENST00000371688.3:exon4:c.G233A:p.R78K,	9q34.3	.
10	1149606	C	G	GGGGATGTGT,CAGCGACTGC	exonic	WDR37	nonsynonymous SNV	WDR37:ENST00000263150.4:exon10:c.C791G:p.S264C,WDR37:ENST00000358220.1:exon10:c.C791G:p.S264C,	10p15.3	.
10	7239583	T	A	TTTGTTCAGG,AAGGGCCTGA	exonic	SFMBT2	nonsynonymous SNV	SFMBT2:ENST00000397167.1:exon15:c.A1625T:p.Y542F,SFMBT2:ENST00000361972.4:exon15:c.A1625T:p.Y542F,	10p14	.
10	8006962	G	C	TTCTCCGTCA,TGTCTCCTCC	exonic	TAF3	nonsynonymous SNV	TAF3:ENST00000344293.5:exon3:c.G1489C:p.V497L,	10p14	.
10	19676553	A	G	GATAATACTG,TGAAAATGAG	exonic	C10orf112	nonsynonymous SNV	C10orf112:ENST00000454679.2:exon12:c.A2426G:p.D809G,C10orf112:ENST00000377266.3:exon13:c.A2465G:p.D822G,C10orf112:ENST00000441070.1:exon2:c.A227G:p.D76G,	10p12.31	.
10	19676561	G	A	TGATGAAAAT,AGTGTGGTAG	exonic	C10orf112	nonsynonymous SNV	C10orf112:ENST00000454679.2:exon12:c.G2434A:p.E812K,C10orf112:ENST00000377266.3:exon13:c.G2473A:p.E825K,C10orf112:ENST00000441070.1:exon2:c.G235A:p.E79K,	10p12.31	.
10	22028961	A	C	TGCCTAAGGA,CAACATCAAG	exonic	MLLT10	nonsynonymous SNV	MLLT10:ENST00000307729.7:exon22:c.A2958C:p.E986D,	10p12.31	.
10	26856274	C	A	CGCCAGGCCG,CCCCCGCGGT	exonic	APBB1IP	nonsynonymous SNV	APBB1IP:ENST00000376236.4:exon15:c.C1858A:p.P620T,	10p12.1	.
10	28228864	C	T	TGCAGCTGCT,ATTCTCACTA	exonic	ARMC4	nonsynonymous SNV	ARMC4:ENST00000305242.5:exon14:c.G2059A:p.E687K,ARMC4:ENST00000537576.1:exon9:c.G1135A:p.E379K,ARMC4:ENST00000545014.1:exon7:c.G634A:p.E212K,	10p12.1	.
10	30316307	G	T	GCACGTGGGT,GCCAGGCTGC	exonic	KIAA1462	nonsynonymous SNV	KIAA1462:ENST00000375377.1:exon3:c.C2770A:p.H924N,	10p11.23	.
10	61829104	T	A	CTCCAAGAAC,TTCTGCTTAT	exonic	ANK3	nonsynonymous SNV	ANK3:ENST00000280772.2:exon37:c.A11535T:p.K3845N,	10q21.2	.
10	75673435	A	C	TACAGGAGGC,CCGGGGGGGC	exonic	PLAU	nonsynonymous SNV	PLAU:ENST00000372764.3:exon7:c.A599C:p.H200P,PLAU:ENST00000446342.1:exon6:c.A548C:p.H183P,PLAU:ENST00000372762.4:exon6:c.A491C:p.H164P,	10q22.2	.
10	81049646	G	C	CCCAGAGCCA,GTAAGAGCCT	exonic	ZMIZ1	nonsynonymous SNV	ZMIZ1:ENST00000334512.5:exon9:c.G540C:p.Q180H,	10q22.3	.
10	94239158	G	A	ACAGTGCAAG,GGTCCACATA	exonic	IDE	nonsynonymous SNV	IDE:ENST00000265986.6:exon15:c.C1760T:p.P587L,IDE:ENST00000371581.5:exon5:c.C95T:p.P32L,	10q23.33	.
10	94399615	A	T	AAGTGTGAAA,TATACAGAAA	exonic	KIF11	nonsynonymous SNV	KIF11:ENST00000260731.3:exon17:c.A2225T:p.N742I,	10q23.33	.
10	104129653	T	A	GTGAGCTGGC,AACACTGAGT	exonic	GBF1	nonsynonymous SNV	GBF1:ENST00000369983.3:exon26:c.T3248A:p.L1083Q,	10q24.32	.
10	123843484	C	A	GGAGAGGTTC,TGCCCCATCA	exonic	TACC2	nonsynonymous SNV	TACC2:ENST00000515603.1:exon4:c.C1469A:p.P490H,TACC2:ENST00000369005.1:exon4:c.C1469A:p.P490H,TACC2:ENST00000515273.1:exon4:c.C1469A:p.P490H,TACC2:ENST00000334433.3:exon3:c.C1469A:p.P490H,TACC2:ENST00000453444.2:exon3:c.C1469A:p.P490H,	10q26.13	.
10	123843492	T	C	TCCTGCCCCA,CACCCCAGGA	exonic	TACC2	nonsynonymous SNV	TACC2:ENST00000515603.1:exon4:c.T1477C:p.S493P,TACC2:ENST00000369005.1:exon4:c.T1477C:p.S493P,TACC2:ENST00000515273.1:exon4:c.T1477C:p.S493P,TACC2:ENST00000334433.3:exon3:c.T1477C:p.S493P,TACC2:ENST00000453444.2:exon3:c.T1477C:p.S493P,	10q26.13	.
10	124608764	C	A	TTTCCTAACT,AGAGGCCCTC	exonic	FAM24B	stoploss	FAM24B:ENST00000368898.3:exon4:c.G284T:p.X95L,FAM24B:ENST00000368896.1:exon4:c.G284T:p.X95L,	10q26.13	.
10	135236804	C	A	CGCGTGGGTC,AGCGCCCGAA	exonic	SPRN	stopgain	SPRN:ENST00000414069.2:exon2:c.G382T:p.G128X,SPRN:ENST00000541506.1:exon2:c.G382T:p.G128X,	10q26.3	.
11	284304	C	T	CCCGCACTGA,GGAGCTGGGC	exonic	NLRP6	nonsynonymous SNV	NLRP6:ENST00000534750.1:exon6:c.C2273T:p.T758M,NLRP6:ENST00000312165.5:exon6:c.C2276T:p.T759M,	11p15.5	.
11	294736	C	G	ACTGTGGACC,TGCCTCTGAA	exonic	ATHL1	nonsynonymous SNV	ATHL1:ENST00000409548.2:exon14:c.C2201G:p.P734R,ATHL1:ENST00000409655.1:exon13:c.C1457G:p.P486R,ATHL1:ENST00000409479.1:exon13:c.C2282G:p.P761R,	11p15.5	.
11	3249525	G	A	TGGCGGCTGG,CTCCCCGAAG	exonic	MRGPRE	nonsynonymous SNV	MRGPRE:ENST00000436689.2:exon2:c.C502T:p.P168S,MRGPRE:ENST00000389832.5:exon2:c.C505T:p.P169S,	11p15.4	.
11	4133158	G	A	ATATGTTGAT,AGAGTATCTG	exonic	RRM1	nonsynonymous SNV	RRM1:ENST00000300738.5:exon7:c.G516A:p.M172I,RRM1:ENST00000423050.2:exon6:c.G225A:p.M75I,	11p15.4	.
11	4944870	G	A	AGTCGCTCCT,GTGGGAGGCA	exonic	OR51G1	stopgain	OR51G1:ENST00000321961.2:exon1:c.C700T:p.Q234X,	11p15.4	.
11	5068339	G	A	GGAAACATTC,TATCAATGGT	exonic	OR52J3	nonsynonymous SNV	OR52J3:ENST00000380370.1:exon1:c.G584A:p.R195H,	11p15.4	.
11	5529920	G	C	AGGTTGGCTG,TGGTGGTGGT	exonic	UBQLN3	nonsynonymous SNV	UBQLN3:ENST00000311659.4:exon2:c.C869G:p.T290S,	11p15.4	.
11	17035718	A	G	CGGTGCGCGG,TGCAGCCAGG	exonic	PLEKHA7	nonsynonymous SNV	PLEKHA7:ENST00000532079.1:exon2:c.T53C:p.I18T,	11p15.1	.
11	44069611	C	T	AGACACCCTT,CTGTGCCCTC	exonic	ACCSL	nonsynonymous SNV	ACCSL:ENST00000378832.1:exon1:c.C25T:p.P9S,ACCSL:ENST00000527145.1:exon1:c.C25T:p.P9S,	11p11.2	.
11	46786724	G	T	TTTTCCATTT,GAACAACAAT	exonic	CKAP5	nonsynonymous SNV	CKAP5:ENST00000415402.1:exon28:c.C3494A:p.P1165Q,CKAP5:ENST00000312055.5:exon28:c.C3494A:p.P1165Q,CKAP5:ENST00000354558.3:exon27:c.C3494A:p.P1165Q,CKAP5:ENST00000529230.1:exon28:c.C3494A:p.P1165Q,	11p11.2	.
11	56128237	A	G	TGCTCTTCTA,TATAATCAAT	exonic	OR8J1	nonsynonymous SNV	OR8J1:ENST00000303039.3:exon1:c.A515G:p.N172S,	11q12.1	.
11	56310306	A	T	AAATGTGGCC,AGCAGATGCA	exonic	OR5M11	stopgain	OR5M11:ENST00000528616.2:exon1:c.T428A:p.L143X,	11q12.1	.
11	57095250	C	T	ATCCGTGATG,TGATGCCAGG	exonic	SSRP1	nonsynonymous SNV	SSRP1:ENST00000278412.2:exon14:c.G1718A:p.S573N,	11q12.1	.
11	57367416	A	T	AGTTTGCAAG,CAGAGGCGAA	exonic	SERPING1	nonsynonymous SNV	SERPING1:ENST00000457869.1:exon3:c.A218T:p.D73V,SERPING1:ENST00000340687.6:exon3:c.A116T:p.D39V,SERPING1:ENST00000403558.1:exon2:c.A218T:p.D73V,SERPING1:ENST00000278407.4:exon3:c.A116T:p.D39V,SERPING1:ENST00000405496.1:exon3:c.A116T:p.D39V,SERPING1:ENST(	11q12.1	.
11	57428280	T	C	AGGTGTGAGG,GAACCGAAGG	exonic	CLP1	nonsynonymous SNV	CLP1:ENST00000302731.4:exon3:c.T458C:p.V153A,CLP1:ENST00000529430.1:exon3:c.T683C:p.V228A,CLP1:ENST00000533682.1:exon3:c.T650C:p.V217A,CLP1:ENST00000525602.1:exon3:c.T650C:p.V217A,	11q12.1	.
11	60637307	G	C	ACCTACCCTC,CCACCCTGGC	exonic	ZP1	nonsynonymous SNV	ZP1:ENST00000278853.5:exon3:c.G616C:p.A206P,	11q12.2	.
11	61539408	G	C	CCTGTACGAT,CTAACTACAA	exonic	MYRF	nonsynonymous SNV	MYRF:ENST00000278836.5:exon7:c.G1099C:p.A367P,MYRF:ENST00000265460.5:exon7:c.G1072C:p.A358P,	11q12.2	.
11	62342976	C	T	CCCTTGGGCC,CCGCTCTGCC	exonic	TUT1	nonsynonymous SNV	TUT1:ENST00000308436.7:exon9:c.G2329A:p.G777R,TUT1:ENST00000476907.1:exon9:c.G2215A:p.G739R,	11q12.3	.
11	62545805	C	T	CCTCAAGGAT,GGGTAAGGGG	exonic	TAF6L	nonsynonymous SNV	TAF6L:ENST00000294168.3:exon5:c.C434T:p.S145L,TAF6L:ENST00000529509.1:exon5:c.C434T:p.S145L,	11q12.3	.
11	62554674	C	T	AGCCCGGCCT,GCGCTACGTG	exonic	TAF6L	nonsynonymous SNV	TAF6L:ENST00000294168.3:exon11:c.C1775T:p.S592L,	11q12.3	.
11	63414003	A	T	GGAAAATTTC,TCCATTGCCA	exonic	ATL3	nonsynonymous SNV	ATL3:ENST00000538786.1:exon6:c.T540A:p.D180E,ATL3:ENST00000398868.3:exon6:c.T594A:p.D198E,ATL3:ENST00000332645.4:exon8:c.T675A:p.D225E,	11q13.1	.
11	63681629	G	C	TTCAGGGGCC,AGAGGGTAGA	exonic	RCOR2	nonsynonymous SNV	RCOR2:ENST00000301459.4:exon8:c.C688G:p.R230G,	11q13.1	RCOR2;basechange=c.688C>T;AAchange=p.R230W;tissue=large_intestine
11	64119828	T	A	ATGCGGGCAC,GGAGCTGGCC	exonic	CCDC88B	nonsynonymous SNV	CCDC88B:ENST00000356786.5:exon19:c.T3326A:p.L1109Q,CCDC88B:ENST00000359902.2:exon6:c.T782A:p.L261Q,	11q13.1	.
11	64564643	G	T	GTTGGGGCCT,AAGGAGGTGG	exonic	MAP4K2	stopgain	MAP4K2:ENST00000294066.2:exon19:c.C1298A:p.S433X,	11q13.1	.
11	65561590	G	C	CTTTGAACAT,AGCCTTCGAG	exonic	OVOL1	nonsynonymous SNV	OVOL1:ENST00000532448.1:exon1:c.G3C:p.M1I,OVOL1:ENST00000335987.3:exon2:c.G189C:p.M63I,	11q13.1	.
11	66617882	C	T	GCGTACAGTC,CCGAGCCCCC	exonic	PC	nonsynonymous SNV	PC:ENST00000393960.1:exon19:c.G2527A:p.G843R,PC:ENST00000393955.2:exon17:c.G2527A:p.G843R,PC:ENST00000393958.2:exon18:c.G2527A:p.G843R,	11q13.2	.
11	66617914	A	T	ACTGTAGTCA,ACACGCGCTC	exonic	PC	nonsynonymous SNV	PC:ENST00000393960.1:exon19:c.T2495A:p.F832Y,PC:ENST00000393955.2:exon17:c.T2495A:p.F832Y,PC:ENST00000393958.2:exon18:c.T2495A:p.F832Y,	11q13.2	.
11	69063510	G	A	ATGTGGGCGC,AATGGATGTG	exonic	MYEOV	nonsynonymous SNV	MYEOV:ENST00000535407.1:exon2:c.G419A:p.R140Q,MYEOV:ENST00000308946.3:exon3:c.G593A:p.R198Q,MYEOV:ENST00000441339.2:exon3:c.G593A:p.R198Q,	11q13.3	MYEOV;basechange=c.593G>A;AAchange=p.R198Q;tissue=large_intestine
11	78565314	C	G	CTCCGCGCCT,CTGGGTTAGG	exonic	TENM4	nonsynonymous SNV	TENM4:ENST00000278550.7:exon12:c.G1516C:p.E506Q,	11q14.1	.
11	85435448	A	C	AACCACAAAA,TCTACCAATA	exonic	SYTL2	nonsynonymous SNV	SYTL2:ENST00000354566.3:exon1:c.T2052G:p.D684E,SYTL2:ENST00000359152.5:exon1:c.T3624G:p.D1208E,SYTL2:ENST00000389959.4:exon1:c.T2052G:p.D684E,SYTL2:ENST00000525423.1:exon1:c.T2052G:p.D684E,SYTL2:ENST00000530351.1:exon1:c.T309G:p.D103E,	11q14.1	.
11	85436047	C	A	GACATTTGGG,AGCTAAATAA	exonic	SYTL2	nonsynonymous SNV	SYTL2:ENST00000354566.3:exon1:c.G1453T:p.A485S,SYTL2:ENST00000359152.5:exon1:c.G3025T:p.A1009S,SYTL2:ENST00000389959.4:exon1:c.G1453T:p.A485S,SYTL2:ENST00000525423.1:exon1:c.G1453T:p.A485S,	11q14.1	.
11	113560976	C	T	GCCCTTCCGT,CAGGTAGCCA	exonic	TMPRSS5	nonsynonymous SNV	TMPRSS5:ENST00000544634.1:exon9:c.G976A:p.D326N,TMPRSS5:ENST00000545579.1:exon11:c.G1156A:p.D386N,TMPRSS5:ENST00000540540.1:exon9:c.G406A:p.D136N,TMPRSS5:ENST00000299882.5:exon11:c.G1183A:p.D395N,TMPRSS5:ENST00000538955.1:exon10:c.G1051A:p.D351N,TMPR	11q23.2	.
11	113561002	G	T	AAGCATGCGG,GGGTGAGGGC	exonic	TMPRSS5	nonsynonymous SNV	TMPRSS5:ENST00000544634.1:exon9:c.C950A:p.P317H,TMPRSS5:ENST00000545579.1:exon11:c.C1130A:p.P377H,TMPRSS5:ENST00000540540.1:exon9:c.C380A:p.P127H,TMPRSS5:ENST00000299882.5:exon11:c.C1157A:p.P386H,TMPRSS5:ENST00000538955.1:exon10:c.C1025A:p.P342H,TMPRS	11q23.2	.
11	116691675	T	C	AGGGAGAGAG,CTTGTCCTGG	exonic	APOA4	nonsynonymous SNV	APOA4:ENST00000357780.3:exon3:c.A1099G:p.T367A,	11q23.3	.
11	119003668	C	T	CTTCAGTGCC,GATCCCTCTG	exonic	HINFP	stopgain	HINFP:ENST00000350777.2:exon8:c.C970T:p.R324X,HINFP:ENST00000527410.1:exon8:c.C970T:p.R324X,	11q23.3	.
11	119003693	A	C	ATCAAGTCCC,TTACCGCAAA	exonic	HINFP	nonsynonymous SNV	HINFP:ENST00000350777.2:exon8:c.A995C:p.H332P,HINFP:ENST00000527410.1:exon8:c.A995C:p.H332P,	11q23.3	.
11	119043656	C	T	CGCTCCCCCA,CCGGGAGGCA	exonic	NLRX1	nonsynonymous SNV	NLRX1:ENST00000292199.2:exon4:c.C187T:p.P63S,NLRX1:ENST00000422249.1:exon4:c.C187T:p.P63S,NLRX1:ENST00000409265.4:exon4:c.C187T:p.P63S,NLRX1:ENST00000525863.1:exon3:c.C187T:p.P63S,NLRX1:ENST00000454811.1:exon4:c.C187T:p.P63S,NLRX1:ENST00000409991.1:exon4:c.(	11q23.3	.
12	6174365	G	A	AGCAGGTACT,GCAGATCCCA	exonic	VWF	stopgain	VWF:ENST00000261405.5:exon11:c.C1231T:p.Q411X,	12p13.31	.
12	21918716	T	A	TTTGGTACCA,GAGGGAAGAA	exonic	KCNJ8	nonsynonymous SNV	KCNJ8:ENST00000240662.2:exon3:c.A1216T:p.M406L,	12p12.1	.
12	21958975	A	T	TGTCATTACT,CTTTTTGCAA	exonic	ABCC9	nonsynonymous SNV	ABCC9:ENST00000345162.2:exon36:c.T4361A:p.V1454E,ABCC9:ENST00000261201.4:exon37:c.T4469A:p.V1490E,ABCC9:ENST00000544039.1:exon30:c.T3350A:p.V1117E,ABCC9:ENST00000261200.4:exon37:c.T4469A:p.V1490E,	12p12.1	.
12	22778176	G	T	TTCAGCTGCT,TCCAGACCCG	exonic	ETNK1	nonsynonymous SNV	ETNK1:ENST00000335148.3:exon1:c.G79T:p.V27F,ETNK1:ENST00000266517.4:exon1:c.G79T:p.V27F,	12p12.1	.
12	31135579	T	C	ACAGTGGTGG,GCGCTGCGGC	exonic	TSPAN11	nonsynonymous SNV	TSPAN11:ENST00000261177.9:exon6:c.T569C:p.V190A,TSPAN11:ENST00000544427.1:exon5:c.T539C:p.V180A,TSPAN11:ENST00000546076.1:exon6:c.T569C:p.V190A,TSPAN11:ENST00000535215.1:exon5:c.T356C:p.V119A,	12p11.21	TSPAN11;basechange=c.569T>C;AAchange=p.V190A;tissue=breast
12	31815016	G	T	GAAGCTTTTT,GACCCTGAGA	exonic	METTL20	nonsynonymous SNV	METTL20:ENST00000539633.1:exon2:c.G129T:p.L43F,METTL20:ENST00000412352.2:exon2:c.G129T:p.L43F,METTL20:ENST00000395763.3:exon2:c.G129T:p.L43F,METTL20:ENST00000357721.3:exon2:c.G129T:p.L43F,METTL20:ENST00000538391.1:exon1:c.G129T:p.L43F,METTL20:ENST0000053841	12p11.21	.
12	32908675	C	T	GAACAGACCT,GAGCCTTCTG	exonic	YARS2	nonsynonymous SNV	YARS2:ENST00000548490.1:exon1:c.G56A:p.R19Q,YARS2:ENST00000324868.8:exon1:c.G134A:p.R45Q,	12p11.21	.

12	40872922	T	G	CAGGGAATTA,GGACAATCAT	exonic	MUC19	stopgain	MUC19:ENST00000454784.4:exon49:c.T4467G:p.Y1489X,	12q12	.
12	49089809	C	T	GTCACAGTGG,GTCAACATAC	exonic	CCNT1	nonsynonymous SNV	CCNT1:ENST00000261900.3:exon7:c.G679A:p.A227T,	12q13.11	.
12	49221588	A	G	AACGGGCATG,CCCCCAAGAC	exonic	CACNB3	nonsynonymous SNV	CACNB3:ENST00000547230.1:exon12:c.A1238G:p.D413G,CACNB3:ENST00000301050.2:exon13:c.A1361G:p.D454G, [illegible]	12q13.12	.
12	49437175	C	A	CTCGAGGCCA,GGGATTCTTC	exonic	KMT2D	nonsynonymous SNV	KMT2D:ENST00000301067.7:exon24:c.G5504T:p.R1835L,	12q13.12	.
12	50748022	T	G	AGCCATGCCT,TTTTTCTTCC	exonic	FAM186A	nonsynonymous SNV	FAM186A:ENST00000543111.1:exon4:c.A2593C:p.K865Q,FAM186A:ENST00000327337.5:exon4:c.A2593C:p.K865Q,	12q13.12	.
12	52913665	C	T	CTCTTGGATA,CTCCAGGAGG	exonic	KRT5	nonsynonymous SNV	KRT5:ENST00000551275.1:exon2:c.G311A:p.G104D,KRT5:ENST00000549420.1:exon2:c.G86A:p.G29D,KRT5:ENST0000025224 [illegible]	12q13.13	.
12	52913701	A	G	AGGGCCACCG,AGCCACCTCC	exonic	KRT5	nonsynonymous SNV	KRT5:ENST00000546577.1:exon2:c.T380C:p.F127S,KRT5:ENST00000551275.1:exon2:c.T275C:p.F92S,KRT5:ENST0000054942 [illegible]	12q13.13	.
12	52913702	A	C	GGGCCACCGA,GCCACCTCCA	exonic	KRT5	nonsynonymous SNV	KRT5:ENST00000546577.1:exon2:c.T379G:p.F127V,KRT5:ENST00000551275.1:exon2:c.T274G:p.F92V,KRT5:ENST0000054942 [illegible]	12q13.13	.
12	53497992	T	G	CACATGGAGG,GAGGGATGTC	splicing	SOAT2(ENST	.	.	12q13.13	.
12	53552421	C	T	GGTAGCCAAT,ATCATGGAGC	exonic	CSAD	nonsynonymous SNV	CSAD:ENST00000267085.4:exon17:c.G1437A:p.M479I,CSAD:ENST00000379843.3:exon13:c.G915A:p.M305I,CSAD:ENST00000453446.2:exon16:c.G1356A:p.M452I,CSAD:ENST00000444623.1:exon17: [illegible]	12q13.13	.
12	54379596	G	T	CCCGCGCGCC,AACATCTGGA	exonic	HOXC10	stopgain	HOXC10:ENST00000303460.4:exon1:c.G553T:p.E185X,HOXC10:ENST00000515593.1:exon2:c.G217T:p.E73X,	12q13.13	.
12	56093699	G	A	CCTGGGAGCC,GTCAGCAGGG	exonic	ITGA7	nonsynonymous SNV	ITGA7:ENST00000257880.7:exon6:c.C877T:p.R293W,ITGA7:ENST00000257879.6:exon5:c.C745T:p.R249W,ITGA7:ENST00000557257.1:exon3:c.C271T:p.R91W,ITGA7:ENST00000347027.6:exon5:c.C7 [illegible]	12q13.2	.
12	56748382	G	A	CAGTGCTTTG,AGGCATCCAG	exonic	STAT2	nonsynonymous SNV	STAT2:ENST00000418572.2:exon8:c.C638T:p.S213F,STAT2:ENST00000314128.4:exon8:c.C650T:p.S217F,STAT2:ENST00000557235.1:exon8:c.C638T:p.S213F,	12q13.3	.
12	56755412	T	A	GCCCAGGTTG,AGAAGGTTCC	exonic	APOF	nonsynonymous SNV	APOF:ENST00000541105.1:exon2:c.A524T:p.Y175F,APOF:ENST00000398189.3:exon2:c.A578T:p.Y193F,	12q13.3	.
12	57493869	A	C	GCCCAGCAGG,TCTCCTAGGG	exonic	STAT6	nonsynonymous SNV	STAT6:ENST00000555375.1:exon3:c.T215G:p.I72S,STAT6:ENST00000537215.2:exon13:c.T1187G:p.I396S,STAT6:ENST00000543873.2:exon14:c.T1517G:p.I506S,STAT6:ENST00000454075.3:exon14:c [illegible]	12q13.3	.
12	57929586	G	A	GCAGCATTAG,ATTGACAATG	exonic	DCTN2	nonsynonymous SNV	DCTN2:ENST00000548249.1:exon3:c.C148T:p.P50S,DCTN2:ENST00000537439.1:exon3:c.C79T:p.P27S,DCTN2:ENST00000550201.1:exon3:c.C148T:p.P50S,DCTN2:ENST00000550954.1:exon4:c.C190 [illegible]	12q13.3	.
12	68642639	C	G	TTGCTTTGAT,TCTCCACTCT	exonic	IL22	nonsynonymous SNV	IL22:ENST00000328087.4:exon5:c.G480C:p.E160D,IL22:ENST00000538666.1:exon6:c.G480C:p.E160D,	12q15	IL22;basechange=c.480G>C;AAchange=p.E160D;tissue=breast
12	76425392	C	T	CGGGCCCCCT,GCGGCGCTTT	exonic	PHLDA1	nonsynonymous SNV	PHLDA1:ENST00000266671.5:exon1:c.G130A:p.E44K,	12q21.2	.
12	76425404	G	T	CGGCGCTTTT,AATGGGCCAT	exonic	PHLDA1	nonsynonymous SNV	PHLDA1:ENST00000266671.5:exon1:c.C118A:p.Q40K,	12q21.2	.
12	94658978	C	G	GCTGTTGTGG,AGGTTCCGGA	exonic	PLXNC1	nonsynonymous SNV	PLXNC1:ENST00000258526.4:exon21:c.C3574G:p.Q1192E,PLXNC1:ENST00000549217.1:exon7:c.C697G:p.Q233E,PLXNC1:ENST00000547057.1:exon6:c.C715G:p.Q239E,	12q22	.
12	109927736	G	A	GAAAGTCTTC,ACCAGCGATG	exonic	UBE3B	nonsynonymous SNV	UBE3B:ENST00000340074.5:exon8:c.G557A:p.R186Q,UBE3B:ENST00000539599.1:exon7:c.G557A:p.R186Q,UBE3B:ENST00000280774.5:exon8:c.G557A:p.R186Q,UBE3B:ENST00000342494.3:exon8: [illegible]	12q24.11	.
12	110376977	C	A	TACCCCATGC,ATCTGGCTCC	exonic	GIT2	nonsynonymous SNV	GIT2:ENST00000355312.3:exon17:c.G1807T:p.G603C,GIT2:ENST00000338373.5:exon16:c.G1513T:p.G505C,GIT2:ENST00000343646.5:exon15:c.G1477T:p.G493C,GIT2:ENST00000457474.2:exon16:c. [illegible]	12q24.11	.
12	110461909	G	A	TAATGGTTAC,AAGCAAAGGT	exonic	ANKRD13A	nonsynonymous SNV	ANKRD13A:ENST00000261739.4:exon7:c.G793A:p.E265K,	12q24.11	.
12	110968352	C	G	TTCTTTGGAG,AGTTTCTTCT	exonic	RAD9B	nonsynonymous SNV	RAD9B:ENST00000409300.1:exon11:c.C1148G:p.A383G,RAD9B:ENST00000392672.4:exon11:c.C1148G:p.A383G,RAD9B:ENST00000409246.1:exon11:c.C932G:p.A311G,RAD9B:ENST00000409425.1: [illegible]	12q24.11	.
12	111158905	C	T	GCTTTGTGAT,ATACCCCTTG	exonic	PPP1CC	nonsynonymous SNV	PPP1CC:ENST00000335007.5:exon7:c.G948A:p.M316I,PPP1CC:ENST00000551582.1:exon3:c.G193A:p.D65N,PPP1CC:ENST00000546933.1:exon6:c.G975A:p.M325I,	12q24.11	.
12	112924322	G	T	TTTCGGACCT,GCCGGACCAC	exonic	PTPN11	nonsynonymous SNV	PTPN11:ENST00000392597.1:exon11:c.G1268T:p.W423L,PTPN11:ENST00000351677.2:exon11:c.G1268T:p.W423L,	12q24.13	.
12	113425039	A	C	AAGTCCCTTG,TGGGTTCACC	exonic	OAS2	nonsynonymous SNV	OAS2:ENST00000342315.4:exon2:c.A374C:p.D125A,OAS2:ENST00000392583.2:exon2:c.A374C:p.D125A,OAS2:ENST00000449768.2:exon2:c.A374C:p.D125A,	12q24.13	.
12	113727991	G	A	CAACAGGACC,TGGTGGAGGA	exonic	TPCN1	nonsynonymous SNV	TPCN1:ENST00000392569.4:exon21:c.G1651A:p.V551M,TPCN1:ENST00000541517.1:exon22:c.G2071A:p.V691M,TPCN1:ENST00000335509.6:exon22:c.G1855A:p.V619M,TPCN1:ENST00000550785.1: [illegible]	12q24.13	.
12	122017948	C	T	GAGTCTTTCT,TGCCTCCATT	exonic	KDM2B	nonsynonymous SNV	KDM2B:ENST00000377069.4:exon1:c.G10A:p.E4K,	12q24.31	.
12	133381494	C	T	GTGTCCACCT,CGAGCTCAGC	exonic	GOLGA3	nonsynonymous SNV	GOLGA3:ENST00000450791.2:exon6:c.G1405A:p.E469K,GOLGA3:ENST00000204726.3:exon7:c.G1405A:p.E469K,GOLGA3:ENST00000456883.2:exon7:c.G1405A:p.E469K,GOLGA3:ENST0000053745 [illegible]	12q24.33	.
13	20608496	C	T	GAAGACTCTT,ATGAAACAGT	exonic	ZMYM2	nonsynonymous SNV	ZMYM2:ENST00000382883.3:exon6:c.C517T:p.H173Y,ZMYM2:ENST00000382871.2:exon12:c.C2071T:p.H691Y,ZMYM2:ENST00000382869.3:exon11:c.C2071T:p.H691Y,ZMYM2:ENST00000382874.2:e [illegible]	13q12.11	.
13	23915711	A	T	TTCTGTTTTC,TCAAATGGAT	exonic	SACS	nonsynonymous SNV	SACS:ENST00000382298.3:exon10:c.T2304A:p.D768E,SACS:ENST00000382292.3:exon9:c.T2304A:p.D768E,SACS:ENST00000402364.1:exon8:c.T54A:p.D18E,	13q12.12	.
13	44455459	C	G	CACAGGAAGA,ATTAATGAAA	exonic	LACC1	nonsynonymous SNV	LACC1:ENST00000441843.1:exon2:c.C338G:p.T113R,LACC1:ENST00000425906.1:exon2:c.C338G:p.T113R,LACC1:ENST00000325686.6:exon2:c.C338G:p.T113R,	13q14.11	.
13	45008909	A	C	TCTTCAGCAG,TTGTTCTCCT	exonic	TSC22D1	nonsynonymous SNV	TSC22D1:ENST00000458659.2:exon3:c.T3075G:p.N1025K,TSC22D1:ENST00000261489.2:exon3:c.T288G:p.N96K,	13q14.11	.
13	49705420	C	T	GCCTCCACCA,GTCATATGTA	exonic	FNDC3A	nonsynonymous SNV	FNDC3A:ENST00000484074.1:exon5:c.C400T:p.R134C,FNDC3A:ENST00000398316.3:exon3:c.C232T:p.R78C,FNDC3A:ENST00000492622.2:exon5:c.C400T:p.R134C,FNDC3A:ENST00000541916.1:ex [illegible]	13q14.2	.
13	52992189	C	A	CTGGTGAGAG,AACTAATAGG	exonic	VPS36	nonsynonymous SNV	VPS36:ENST00000378060.4:exon11:c.G843T:p.L281F,	13q14.3	.
13	60616936	G	A	GAGATCTGTC,GCTTCTCTTA	exonic	DIAPH3	stopgain	DIAPH3:ENST00000400330.1:exon5:c.C514T:p.R172X,DIAPH3:ENST00000400320.1:exon3:c.C376T:p.R126X,DIAPH3:ENST00000377908.2:exon4:c.C481T:p.R161X,DIAPH3:ENST00000400319.1:exon3 [illegible]	13q21.2	DIAPH3;basechange=c.514C>T;AAchange=p.R172*;tissue=skin
13	79929476	G	T	TTCGTATGTT,CAAATTGGAT	exonic	RBM26	nonsynonymous SNV	RBM26:ENST00000267229.7:exon12:c.C1733A:p.A578E,RBM26:ENST00000438724.1:exon12:c.C1733A:p.A578E,RBM26:ENST00000438737.2:exon12:c.C1733A:p.A578E,	13q31.1	.
13	101258641	G	T	TGCAGCATTT,GATTTGCTTT	exonic	TMTC4	nonsynonymous SNV	TMTC4:ENST00000376234.3:exon17:c.C2048A:p.P683Q,TMTC4:ENST00000342624.5:exon18:c.C2105A:p.P702Q,TMTC4:ENST00000328767.5:exon15:c.C1715A:p.P572Q,	13q32.3	.
13	101707699	C	T	AGTCAGCTGC,GGGTCCACCA	exonic	NALCN	nonsynonymous SNV	NALCN:ENST00000251127.6:exon44:c.G5165A:p.R1722Q,	13q33.1	.
14	21819371	C	T	GATTTGTTTT,ATGAAGGAAC	exonic	RPGRIP1	nonsynonymous SNV	RPGRIP1:ENST00000307974.4:exon12:c.C1934T:p.S645L,RPGRIP1:ENST00000557771.1:exon24:c.C3743T:p.S1248L,RPGRIP1:ENST00000400017.2:exon24:c.C3857T:p.S1286L,RPGRIP1:ENST00000 [illegible]	14q11.2	.
14	21992295	C	T	TTTTCATCAG,TTTATTCTTG	exonic	SALL2	nonsynonymous SNV	SALL2:ENST00000327430.3:exon2:c.G1567A:p.A523T,SALL2:ENST00000450879.2:exon3:c.G1156A:p.A386T,	14q11.2	.
14	24534228	C	A	GCCTCCACTC,AGAAGAAGAG	exonic	LRRC16B	nonsynonymous SNV	LRRC16B:ENST00000342740.5:exon33:c.C3142A:p.Q1048K,LRRC16B:ENST00000334420.7:exon33:c.C430A:p.Q144K,	14q11.2	LRRC16B;basechange=c.3142C>T;AAchange=p.Q1048*;tissue=skin
14	31077196	G	T	TTGTTTAACT,CCTTGTTTAT	exonic	G2E3	nonsynonymous SNV	G2E3:ENST00000438909.2:exon11:c.G1283T:p.C428F,G2E3:ENST00000553504.1:exon12:c.G1511T:p.C504F,G2E3:ENST00000206595.6:exon12:c.G1421T:p.C474F,	14q12	.
14	31588942	G	T	TAGATCAGTT,TCTGCTGGAC	exonic	HECTD1	nonsynonymous SNV	HECTD1:ENST00000553700.1:exon29:c.C5369A:p.T1790K,HECTD1:ENST00000399332.1:exon29:c.C5369A:p.T1790K,HECTD1:ENST00000553957.1:exon20:c.C3650A:p.T1217K,	14q12	.
14	35480809	C	G	TACAAGTGGC,GCCACAAACA	exonic	SRP54	nonsynonymous SNV	SRP54:ENST00000556994.1:exon9:c.C580G:p.R194G,SRP54:ENST00000546080.1:exon7:c.C433G:p.R145G,SRP54:ENST00000555557.1:exon7:c.C388G:p.R130G,SRP54:ENST00000216774.6:exon8:c [illegible]	14q13.2	.
14	38724374	G	A	GGTCACACAA,AGCGGCCGTC	exonic	CLEC14A	nonsynonymous SNV	CLEC14A:ENST00000342213.2:exon1:c.C854T:p.S285F,	14q21.1	.
14	45564687	A	C	CCAGAATATG,AAAATTTTGG	exonic	PRPF39	nonsynonymous SNV	PRPF39:ENST00000554439.1:exon1:c.A245C:p.E82A,PRPF39:ENST00000355765.6:exon2:c.A245C:p.E82A,PRPF39:ENST00000553605.1:exon2:c.A245C:p.E82A,PRPF39:ENST00000554429.1:exon1:c [illegible]	14q21.2	.
14	45716251	A	T	AGCCTCTGTT,GCATAGTTGA	exonic	MIS18BP1	nonsynonymous SNV	MIS18BP1:ENST00000451174.1:exon3:c.T239A:p.L80Q,MIS18BP1:ENST00000454990.1:exon2:c.T239A:p.L80Q,MIS18BP1:ENST00000310806.4:exon2:c.T239A:p.L80Q,	14q21.2	.
14	55467285	G	T	ATTTCCTTCC,CATCAGTATA	exonic	WDHD1	nonsynonymous SNV	WDHD1:ENST00000421192.1:exon10:c.C509A:p.A170E,WDHD1:ENST00000360586.3:exon10:c.C878A:p.A293E,WDHD1:ENST00000420358.2:exon9:c.C509A:p.A170E,	14q22.2	.
14	55510773	G	C	TTGAACAGTG,AATCACAACT	exonic	SOCS4	nonsynonymous SNV	SOCS4:ENST00000339298.2:exon2:c.G1014C:p.W338C,SOCS4:ENST00000395472.2:exon2:c.G1014C:p.W338C,SOCS4:ENST00000555846.1:exon3:c.G1014C:p.W338C,	14q22.3	.
14	56585410	C	A	GAACACTGCG,CCCCAATAAG	exonic	PELI2	nonsynonymous SNV	PELI2:ENST00000267460.4:exon1:c.C32A:p.A11D,	14q22.3	.
14	61113269	G	A	CTTGTTGGAG,AGGAGTTATT	exonic	SIX1	nonsynonymous SNV	SIX1:ENST00000247182.6:exon2:c.C587T:p.S196F,SIX1:ENST00000554986.1:exon2:c.C68T:p.S23F,	14q23.1	.
14	74876275	C	G	CTGCAGGGAC,CTGGGTCCAG	exonic	SYNDIG1L	nonsynonymous SNV	SYNDIG1L:ENST00000331628.3:exon2:c.G173C:p.G58A,SYNDIG1L:ENST00000554953.1:exon2:c.G173C:p.G58A,SYNDIG1L:ENST00000554823.1:exon1:c.G173C:p.G58A,	14q24.3	.
14	75022345	C	A	CAGTGCGGGA,AACCCCACGT	exonic	LTBP2	nonsynonymous SNV	LTBP2:ENST00000261978.4:exon4:c.G882T:p.L294F,LTBP2:ENST00000553939.1:exon4:c.G882T:p.L294F,LTBP2:ENST00000556690.1:exon4:c.G882T:p.L294F,	14q24.3	.
14	75230733	C	G	GACCTCATCT,AGCCCTACCT	exonic	YLPM1	nonsynonymous SNV	YLPM1:ENST00000325680.7:exon1:c.C541G:p.Q181E,YLPM1:ENST00000552421.1:exon1:c.C541G:p.Q181E,YLPM1:ENST00000238571.3:exon1:c.C541G:p.Q181E,	14q24.3	.
14	75329568	G	A	TCCTGACAGG,TTTGGGGGTC	exonic	PROX2	nonsynonymous SNV	PROX2:ENST00000556489.2:exon1:c.C970T:p.P324S,PROX2:ENST00000445876.1:exon1:c.C970T:p.P324S,	14q24.3	.
14	75747739	C	T	CCCAAGCCCT,AGTGGAACCT	exonic	FOS	nonsynonymous SNV	FOS:ENST00000555686.1:exon3:c.C413T:p.S138L,FOS:ENST00000555672.1:exon2:c.C305T:p.S102L,FOS:ENST00000555347.1:exon2:c.C311T:p.S104L,FOS:ENST00000303562.4:exon4:c.C755T:p.S2 [illegible]	14q24.3	.
14	94044269	C	T	GAGTCCGTTT,GGAGTCCTTT	exonic	UNC79	nonsynonymous SNV	UNC79:ENST00000555664.1:exon18:c.C2293T:p.R765W,UNC79:ENST00000393151.2:exon18:c.C2293T:p.R765W,UNC79:ENST00000256339.4:exon18:c.C1762T:p.R588W,UNC79:ENST00000553484.1 [illegible]	14q32.12	KIAA1409;basechange=c.1762C>T;AAchange=p.R588W;tissue=endometrium&UNC79;basechange=c.2293C>T;AAchange=p.R765W;tissue=endometrium
14	96848724	C	T	GTTCTCTTTG,TGTTAACAAC	exonic	GSKIP	nonsynonymous SNV	GSKIP:ENST00000556095.1:exon3:c.C140T:p.A47V,GSKIP:ENST00000555757.1:exon2:c.C140T:p.A47V,GSKIP:ENST00000438650.1:exon2:c.C140T:p.A47V,GSKIP:ENST00000554182.1:exon3:c.C140T [illegible]	14q32.2	.
14	104618742	C	G	CCAGCAGTGC,TGGAGGGCAT	exonic	KIF26A	nonsynonymous SNV	KIF26A:ENST00000423312.2:exon3:c.C679G:p.L227V,KIF26A:ENST00000315264.7:exon2:c.C262G:p.L88V,	14q32.33	.
14	105612163	C	G	CTCGGTGGCT,TTCTGCGCCG	exonic	JAG2	nonsynonymous SNV	JAG2:ENST00000331782.3:exon23:c.G2857C:p.E953Q,JAG2:ENST00000347004.2:exon22:c.G2743C:p.E915Q,	14q32.33	.
14	105639489	C	G	TGCACCACCT,AATGAGCTCA	exonic	NUDT14	nonsynonymous SNV	NUDT14:ENST00000392568.2:exon5:c.G538C:p.E180Q,	14q32.33	.
15	22969164	A	C	GTGCCACAGG,GCTGGATGGC	exonic	CYFIP1	nonsynonymous SNV	CYFIP1:ENST00000560848.1:exon23:c.A2390C:p.E797A,CYFIP1:ENST00000313077.7:exon22:c.A2390C:p.E797A,CYFIP1:ENST00000435939.2:exon7:c.A1097C:p.E366A,	15q11.2	.
15	35083320	T	C	TTTACCTTAA,CTTCATGGTG	exonic	ACTC1	nonsynonymous SNV	ACTC1:ENST00000290378.4:exon6:c.A985G:p.I329V,	15q14	.
15	40913973	C	A	CAAGATCAAT,AAATGTGCAA	exonic	CASC5	stopgain	CASC5:ENST00000399668.2:exon10:c.C1511A:p.S504X,CASC5:ENST00000346991.5:exon11:c.C1589A:p.S530X,	15q15.1	.
15	42028393	T	A	GAGCTGGAAG,CTTCCTGCAA	exonic	MGA	nonsynonymous SNV	MGA:ENST00000545763.1:exon13:c.T3931A:p.S1311T,MGA:ENST00000389936.4:exon13:c.T3931A:p.S1311T,MGA:ENST00000566586.1:exon12:c.T3931A:p.S1311T,MGA:ENST00000570161.1:exon12 [illegible]	15q15.1	.
15	42693997	G	T	CATTGGCTTC,CCATCTACGA	exonic	CAPN3	nonsynonymous SNV	CAPN3:ENST00000357568.3:exon11:c.G1513T:p.A505S,CAPN3:ENST00000356316.3:exon15:c.G1252T:p.A418S,CAPN3:ENST00000397163.3:exon11:c.G1513T:p.A505S,CAPN3:ENST00000318023.7:e [illegible]	15q15.1	.
15	44216507	T	A	TTGGCATCTC,CTGCAAAGAA	exonic	FRMD5	stopgain	FRMD5:ENST00000421674.1:exon2:c.A103T:p.R35X,FRMD5:ENST00000449926.1:exon1:c.A1T:p.R1X,FRMD5:ENST00000417257.1:exon2:c.A103T:p.R35X,FRMD5:ENST00000451277.1:exon2:c.A103T [illegible]	15q15.3	.
15	44671972	C	A	CCTCTCTCCC,AAATATGCCT	exonic	CASC4	nonsynonymous SNV	CASC4:ENST00000345795.2:exon7:c.C887A:p.P296Q,CASC4:ENST00000299957.6:exon7:c.C887A:p.P296Q,	15q15.3	.
15	44967839	C	G	AACTGGCAGG,AGACACCAGC	exonic	PATL2	nonsynonymous SNV	PATL2:ENST00000560775.1:exon2:c.G67C:p.A23P,PATL2:ENST00000434130.1:exon3:c.G67C:p.A23P,	15q21.1	.
15	59359062	G	A	CCATGTGGAG,GTCGTCACAG	exonic	RNF111	nonsynonymous SNV	RNF111:ENST00000559209.1:exon6:c.G1466A:p.G489E,RNF111:ENST00000557998.1:exon6:c.G1466A:p.G489E,RNF111:ENST00000434298.1:exon6:c.G1466A:p.G489E,RNF111:ENST00000561186.1: [illegible]	15q22.2	.
15	59981525	G	T	ACAGTGAGAA,CCCCGGCGGA	exonic	BNIP2	nonsynonymous SNV	BNIP2:ENST00000267859.3:exon1:c.C115A:p.L39I,	15q22.2	.
15	64966446	A	C	GCGTGTCCGT,CTAATTCCAT	exonic	ZNF609	nonsynonymous SNV	ZNF609:ENST00000326648.3:exon4:c.A1393C:p.T465P,	15q22.31	.
15	86124084	G	A	ACAAGATAAG,ATAAAGCGGT	exonic	AKAP13	nonsynonymous SNV	AKAP13:ENST00000559362.1:exon7:c.G2785A:p.D929N,AKAP13:ENST00000361243.2:exon7:c.G2785A:p.D929N,AKAP13:ENST00000394518.2:exon7:c.G2785A:p.D929N,	15q25.3	.
15	90348585	C	T	GCTGTCAGGT,CTTGGGGTGG	exonic	ANPEP	nonsynonymous SNV	ANPEP:ENST00000300060.6:exon3:c.G724A:p.D242N,ANPEP:ENST00000560028.1:exon1:c.G19A:p.D7N,	15q26.1	.
15	100269737	G	A	GCCGGCCTGG,CACCGGTGCC	exonic	LYSMD4	nonsynonymous SNV	LYSMD4:ENST00000332728.4:exon4:c.C482T:p.A161V,LYSMD4:ENST00000545021.1:exon4:c.C104T:p.A35V,LYSMD4:ENST00000344791.2:exon6:c.C485T:p.A162V,LYSMD4:ENST00000409796.1:exo [illegible]	15q26.3	.
15	100269749	G	A	ACCGGTGCCC,CGCCTGCTCT	exonic	LYSMD4	nonsynonymous SNV	LYSMD4:ENST00000332728.4:exon4:c.C470T:p.A157V,LYSMD4:ENST00000545021.1:exon4:c.C92T:p.A31V,LYSMD4:ENST00000344791.2:exon6:c.C473T:p.A158V,LYSMD4:ENST00000409796.1:exon3 [illegible]	15q26.3	LYSMD4;basechange=c.473C>T;AAchange=p.A158V;tissue=central_nervous_system
16	863812	C	G	CCGCTGGGGT,TCCCGCCGTC	exonic	PRR25	nonsynonymous SNV	PRR25:ENST00000301698.1:exon3:c.C1160G:p.S387C,	16p13.3	.
16	1412694	A	G	ACCCAGCTGG,GGGAGGTCCT	exonic	GNPTG	nonsynonymous SNV	GNPTG:ENST00000204679.4:exon9:c.A692G:p.E231G,	16p13.3	.
16	1505137	C	G	CAGGTTACCT,CGAGTCAAAC	exonic	CLCN7	nonsynonymous SNV	CLCN7:ENST00000262318.8:exon11:c.G1024C:p.E342Q,CLCN7:ENST00000382745.4:exon12:c.G1096C:p.E366Q,CLCN7:ENST00000448525.1:exon11:c.G1024C:p.E342Q,	16p13.3	.
16	1537455	T	C	GGGGGGCCCC,GTGCTCAGAG	exonic	PTX4	nonsynonymous SNV	PTX4:ENST00000293922.1:exon2:c.A643G:p.R215G,PTX4:ENST00000447419.2:exon2:c.A658G:p.R220G,	16p13.3	.
16	2285562	G	A	GGTGCAGACG,TCATCGTCTA	exonic	E4F1	nonsynonymous SNV	E4F1:ENST00000565090.1:exon12:c.G1813A:p.V605I,E4F1:ENST00000301727.4:exon14:c.G2344A:p.V782I,	16p13.3	.
16	2569391	C	G	ATGACGACAT,AGCCTCTACA	exonic	ATP6V0C	nonsynonymous SNV	ATP6V0C:ENST00000564973.1:exon1:c.C123G:p.I41M,ATP6V0C:ENST00000330398.4:exon2:c.C252G:p.I84M,ATP6V0C:ENST00000565223.1:exon2:c.C123G:p.I41M,	16p13.3	.
16	2816500	C	T	TCCGGTCACC,GAAGGAGATC	exonic	SRRM2	stopgain	SRRM2:ENST00000301740.8:exon11:c.C5971T:p.R1991X,	16p13.3	SRRM2;basechange=c.5971C>A;AAchange=p.R1991R;tissue=endometrium
16	2817508	T	C	TCCCTCCAGC,CCAGAACACC	exonic	SRRM2	nonsynonymous SNV	SRRM2:ENST00000301740.8:exon11:c.T6979C:p.S2327P,	16p13.3	.
16	3293922	A	T	AGGGAAAAAA,TCCTGAGCAT	exonic	MEFV	nonsynonymous SNV	MEFV:ENST00000541159.1:exon8:c.T1272A:p.D424E,	16p13.3	.
16	4925055	G	T	GTCCTCTTCT,CCCTGAGCCA	exonic	UBN1	nonsynonymous SNV	UBN1:ENST00000590769.1:exon15:c.G2644T:p.A882S,UBN1:ENST00000396658.4:exon14:c.G2644T:p.A882S,UBN1:ENST00000545171.1:exon15:c.G2644T:p.A882S,UBN1:ENST00000262376.6:exon1 [illegible]	16p13.3	.
16	11000885	C	A	GCTTCCTGCA,AGCACGTGCG	exonic	CIITA	nonsynonymous SNV	CIITA:ENST00000324288.8:exon11:c.C1536A:p.H512Q,	16p13.13	.
16	11038614	G	T	CGGGGGCCAT,GCAAGACTTC	exonic	CLEC16A	nonsynonymous SNV	CLEC16A:ENST00000409552.3:exon1:c.G40T:p.G14C,CLEC16A:ENST00000409790.1:exon1:c.G40T:p.G14C,	16p13.13	.
16	24043493	T	A	GATCCACACG,ACTCCAGCCC	exonic	PRKCB	nonsynonymous SNV	PRKCB:ENST00000321728.7:exon4:c.T325A:p.Y109N,PRKCB:ENST00000303531.7:exon4:c.T325A:p.Y109N,	16p12.2	PRKCB_ENST00000303531;basechange=c.325T>C;AAchange=p.Y109H;tissue=upper_aerodigestive_tract
16	27514259	C	T	CGGTAAGTTT,GTGTGGTTTG	exonic	GTF3C1	nonsynonymous SNV	GTF3C1:ENST00000561623.1:exon11:c.G1828A:p.E610K,GTF3C1:ENST00000356183.4:exon11:c.G1828A:p.E610K,	16p12.1	.
16	29908148	C	A	CTCACCTGGG,TGGTCACCGT	exonic	SEZ6L2	nonsynonymous SNV	SEZ6L2:ENST00000308713.5:exon3:c.G506T:p.S169I,SEZ6L2:ENST00000346932.5:exon3:c.G506T:p.S169I,SEZ6L2:ENST00000350527.3:exon3:c.G296T:p.S99I,SEZ6L2:ENST00000537485.1:exon2:c.G [illegible]	16p11.2	.
16	30544276	C	G	AGCCGTGACG,TGGTCAGCTC	exonic	ZNF747	nonsynonymous SNV	ZNF747:ENST00000568028.1:exon3:c.G537C:p.Q179H,ZNF747:ENST00000535210.1:exon3:c.G537C:p.Q179H,ZNF747:ENST00000569360.1:exon3:c.G537C:p.Q179H,	16p11.2	.
16	30734031	C	T	ACCACCCCTG,CCCTACTGGC	exonic	SRCAP	nonsynonymous SNV	SRCAP:ENST00000262518.4:exon23:c.C3854T:p.A1285V,	16p11.2	.
16	31382674	G	T	TGTGCTCTGG,TGGGGGTGAG	exonic	ITGAX	nonsynonymous SNV	ITGAX:ENST00000562522.1:exon16:c.G1861T:p.V621L,ITGAX:ENST00000268296.4:exon16:c.G1861T:p.V621L,	16p11.2	.
16	31471028	C	G	TCGAGCGCTT,CGGGCACGCG	exonic	ARMC5	nonsynonymous SNV	ARMC5:ENST00000268314.4:exon1:c.C183G:p.F61L,ARMC5:ENST00000457010.2:exon1:c.C183G:p.F61L,ARMC5:ENST00000408912.3:exon3:c.C468G:p.F156L,ARMC5:ENST00000563544.1:exon2:c.C [illegible]	16p11.2	.
16	67709582	G	A	CGGATGCCAC,GATGGCAGCG	exonic	GFOD2	nonsynonymous SNV	GFOD2:ENST00000268797.7:exon3:c.C634T:p.R212C,	16q22.1	.
16	68289836	G	A	CCGGGCCTTC,TGTCACTGGG	exonic	PLA2G15	nonsynonymous SNV	PLA2G15:ENST00000564827.2:exon6:c.G641A:p.R214H,PLA2G15:ENST00000568082.1:exon5:c.G730A:p.V244M,PLA2G15:ENST00000413021.2:exon4:c.G388A:p.V130M,PLA2G15:ENST00000566188. [illegible]	16q22.1	.
16	75269722	C	A	TACACGTCCT,GGCCGGCGGG	exonic	BCAR1	stopgain	BCAR1:ENST00000162330.5:exon5:c.G1075T:p.E359X,BCAR1:ENST00000393422.2:exon5:c.G1129T:p.E377X,BCAR1:ENST00000538440.2:exon5:c.G1075T:p.E359X,BCAR1:ENST00000546196.1:exon [illegible]	16q23.1	.
16	84132766	G	A	TCTTTTATCT,AATCACCTCA	exonic	MBTPS1	stopgain	MBTPS1:ENST00000343411.3:exon3:c.C313T:p.Q105X,	16q23.3	.
16	87723386	G	A	GACCCCCGAC,ACAGCCCCCT	exonic	JPH3	nonsynonymous SNV	JPH3:ENST00000284262.2:exon4:c.G1420A:p.D474N,	16q24.2	.
16	88052214	C	T	TCCAACCTCT,GGGGCAGGGC	exonic	BANP	nonsynonymous SNV	BANP:ENST00000393207.1:exon7:c.C812T:p.S271L,BANP:ENST00000355022.4:exon6:c.C719T:p.S240L,BANP:ENST00000479780.2:exon6:c.C719T:p.S240L,BANP:ENST00000393208.2:exon6:c.C719T [illegible]	16q24.2	.
16	89614500	G	A	CTTCGAGTAC,CCGTGGAGCG	exonic	SPG7	nonsynonymous SNV	SPG7:ENST00000561911.1:exon2:c.G187A:p.A63T,SPG7:ENST00000268704.2:exon12:c.G1642A:p.A548T,	16q24.3	.
16	89805060	T	G	CAGCCTGCTG,CTGCTCTGGA	exonic	FANCA	nonsynonymous SNV	FANCA:ENST00000561722.1:exon5:c.A537C:p.R179S,FANCA:ENST00000389301.3:exon43:c.A4317C:p.R1439S,	16q24.3	.
16	89805092	C	A	ACCTCTGGGT,GCAGTCCCCA	exonic	FANCA	nonsynonymous SNV	FANCA:ENST00000561722.1:exon5:c.G505T:p.D169Y,FANCA:ENST00000389301.3:exon43:c.G4285T:p.D1429Y,	16q24.3	.
17	1628488	G	T	CTGTCCCCGC,CAGAGGGCCT	exonic	WDR81	nonsynonymous SNV	WDR81:ENST00000409644.1:exon1:c.G235T:p.A79S,	17p13.3	.
17	2218939	C	A	CACACCAGTG,TAACAAGCTC	exonic	SRR	nonsynonymous SNV	SRR:ENST00000570662.2:exon2:c.C85A:p.L29I,SRR:ENST00000575840.1:exon3:c.C85A:p.L29I,SRR:ENST00000574229.1:exon1:c.C85A:p.L29I,SRR:ENST00000572709.1:exon2:c.C85A:p.L29I,SRR:EN [illegible]	17p13.3	.
17	7154582	T	C	GCCACGAACG,GCGCAGCCCC	exonic	CTDNEP1	nonsynonymous SNV	CTDNEP1:ENST00000574322.1:exon1:c.A34G:p.T12A,CTDNEP1:ENST00000571409.1:exon2:c.A34G:p.T12A,CTDNEP1:ENST00000575783.1:exon2:c.A34G:p.T12A,CTDNEP1:ENST00000318988.6:exo [illegible]	17p13.1	CTDNEP1;basechange=c.34A>G;AAchange=p.T12A;tissue=breast
17	7578263	G	A	CCTTCCACTC,GATAAGATGC	exonic	TP53	stopgain	TP53:ENST00000413465.2:exon5:c.C586T:p.R196X,TP53:ENST00000359597.4:exon5:c.C586T:p.R196X,TP53:ENST00000514944.1:exon5:c.C307T:p.R103X,TP53:ENST00000445888.2:exon6:c.C586T:p [illegible]	17p13.1	TP53;basechange=c.586C>A;AAchange=p.R196R;tissue=oesophagus,central_nervous_system&TP53_ENST00000545858;basechange=c.307C>T;AAchange=p.R103*;tissue=breas [illegible]
17	8141520	A	G	ACGGGGGAAC,GAAAAAGATG	exonic	CTC1	nonsynonymous SNV	CTC1:ENST00000315684.8:exon4:c.T476C:p.L159P,CTC1:ENST00000449476.2:exon4:c.T476C:p.L159P,	17p13.1	.
17	8243661	G	A	ACTATTGGTC,TGGCCTTCTC	exonic	ODF4	nonsynonymous SNV	ODF4:ENST00000328248.2:exon1:c.G292A:p.V98M,	17p13.1	ODF4;basechange=c.292G>A;AAchange=p.V98M;tissue=stomach
17	16526537	G	A	CATTTATAAG,TTTCTCTCCT	exonic	ZNF624	nonsynonymous SNV	ZNF624:ENST00000311331.7:exon6:c.C1663T:p.P555S,	17p11.2	.
17	17124829	G	A	CCAAGGCCCC,GCAACAGCAC	exonic	FLCN	nonsynonymous SNV	FLCN:ENST00000389169.5:exon8:c.C893T:p.P298L,	17p11.2	.
17	17723836	C	G	TGAAGCATGT,TGTGAAAAGG	splicing	SREBF1(ENS	.	.	17p11.2	.
17	18022943	C	T	CTACGGCGAC,ACTACTACGG	exonic	MYO15A	nonsynonymous SNV	MYO15A:ENST00000205890.5:exon2:c.C829T:p.H277Y,	17p11.2	.
17	19290121	G	A	GGCGTGGAGA,AAGCAGCAGC	exonic	MFAP4	nonsynonymous SNV	MFAP4:ENST00000497081.2:exon1:c.C112T:p.L38F,MFAP4:ENST00000395592.2:exon2:c.C109T:p.L37F,MFAP4:ENST00000299610.4:exon2:c.C37T:p.L13F,	17p11.2	.
17	26955373	G	C	CCCCGCTTTG,CTTTTTGGCT	exonic	KIAA0100	nonsynonymous SNV	KIAA0100:ENST00000544884.1:exon24:c.C4075G:p.P1359A,KIAA0100:ENST00000389003.3:exon24:c.C4075G:p.P1359A,KIAA0100:ENST00000528896.2:exon24:c.C4504G:p.P1502A,	17q11.2	.
17	36622669	C	A	GGGGATGAGC,AGCCCCGCCC	exonic	ARHGAP23	nonsynonymous SNV	ARHGAP23:ENST00000431231.2:exon7:c.C745A:p.Q249K,ARHGAP23:ENST00000437668.3:exon7:c.C745A:p.Q249K,ARHGAP23:ENST00000443378.1:exon7:c.C463A:p.Q155K,	17q12	.
17	37815015	G	T	GTGGACCCCT,CTGTTCTCCG	exonic	STARD3	nonsynonymous SNV	STARD3:ENST00000578577.1:exon6:c.G521T:p.C174F,	17q12	.
17	38990945	G	A	TAAAGCCATG,ATTTTTGTGG	exonic	TMEM99	stopgain	TMEM99:ENST00000436612.1:exon3:c.G177A:p.W59X,TMEM99:ENST00000301665.3:exon3:c.G177A:p.W59X,	17q21.2	.
17	39992207	C	A	TCAGGGTGCT,ACCTGTCCCG	exonic	NT5C3B	stopgain	NT5C3B:ENST00000445655.1:exon2:c.G118T:p.E40X,	17q21.2	.
17	54933967	C	G	TCTCTGTTTT,TAGCAGAATT	exonic	DGKE	nonsynonymous SNV	DGKE:ENST00000284061.3:exon8:c.C1193G:p.S398C,	17q22	DGKE;basechange=c.1193C>A;AAchange=p.S398Y;tissue=large_intestine
17	55184399	C	T	GAGACCAGCT,GAGCCCCAGG	exonic	AKAP1	nonsynonymous SNV	AKAP1:ENST00000571629.1:exon3:c.C1574T:p.S525L,AKAP1:ENST00000337714.3:exon2:c.C1574T:p.S525L,AKAP1:ENST00000572557.1:exon2:c.C1574T:p.S525L,AKAP1:ENST00000573326.1:exon1: [illegible]	17q22	.
17	61888459	C	T	TGCAACTTTT,GGAAGAAGAT	exonic	DDX42	nonsynonymous SNV	DDX42:ENST00000359353.5:exon12:c.C967T:p.R323W,DDX42:ENST00000457800.2:exon12:c.C1324T:p.R442W,DDX42:ENST00000389924.2:exon13:c.C1324T:p.R442W,DDX42:ENST00000583590.1:e [illegible]	17q23.3	.
17	65936711	G	A	CAACCCCCTC,CCCCCAACAA	exonic	BPTF	nonsynonymous SNV	BPTF:ENST00000321892.4:exon21:c.G6794A:p.R2265H,BPTF:ENST00000424123.3:exon21:c.G6377A:p.R2126H,BPTF:ENST00000306378.6:exon19:c.G6416A:p.R2139H,BPTF:ENST00000335221.5:ex [illegible]	17q24.2	.
17	73824949	C	G	CCGGGCACCT,ACGCAGCGGC	exonic	UNC13D	nonsynonymous SNV	UNC13D:ENST00000412096.2:exon31:c.G3070C:p.E1024Q,UNC13D:ENST00000207549.4:exon31:c.G3070C:p.E1024Q,	17q25.1	.
17	74287204	C	G	CTACTGAGCT,TCCCATCAGA	exonic	QRICH2	nonsynonymous SNV	QRICH2:ENST00000262765.5:exon4:c.G3106C:p.E1036Q,QRICH2:ENST00000447564.2:exon1:c.G130C:p.E44Q,	17q25.1	.
17	76495164	G	T	GCTCCCGTCT,GGCCAGGCCC	exonic	DNAH17	nonsynonymous SNV	DNAH17:ENST00000585328.1:exon37:c.C5656A:p.Q1886K,DNAH17:ENST00000389840.5:exon37:c.C5629A:p.Q1877K,	17q25.3	.
17	78059929	C	A	GTCAAGGTGA,ACAGGAGCAG	exonic	CCDC40	nonsynonymous SNV	CCDC40:ENST00000374877.3:exon14:c.C2363A:p.T788K,CCDC40:ENST00000397545.4:exon14:c.C2363A:p.T788K,	17q25.3	.
18	346328	C	T	ATGAGCTGAC,ATGCTTGGAG	exonic	COLEC12	nonsynonymous SNV	COLEC12:ENST00000400256.3:exon5:c.G1294A:p.G432S,	18p11.32	.
18	5891204	G	A	GCCGCGGGGT,AGGAGGCCAC	exonic	TMEM200C	nonsynonymous SNV	TMEM200C:ENST00000383490.2:exon1:c.C859T:p.H287Y,TMEM200C:ENST00000581347.2:exon3:c.C859T:p.H287Y,	18p11.31	.
18	12337375	C	T	GTGAGAAGAG,TACTGTTCTT	exonic	AFG3L2	nonsynonymous SNV	AFG3L2:ENST00000269143.3:exon16:c.G2140A:p.A714T,	18p11.21	AFG3L2;basechange=c.2140G>A;AAchange=p.A714T;tissue=endometrium
18	21355845	G	A	AGACAACTGT,AGAAGTGTGC	exonic	LAMA3	nonsynonymous SNV	LAMA3:ENST00000313654.9:exon10:c.G1363A:p.E455K,LAMA3:ENST00000399516.3:exon10:c.G1363A:p.E455K,LAMA3:ENST00000585600.1:exon10:c.G1363A:p.E455K,	18q11.2	.
18	29867662	G	T	ACAGTCAAGT,CAAAGGAAAG	exonic	GAREM	nonsynonymous SNV	GAREM:ENST00000399218.4:exon4:c.C898A:p.H300N,GAREM:ENST00000269209.6:exon4:c.C898A:p.H300N,	18q12.1	.
18	33767568	C	A	GGGACCCGAG,GCACCGCGGC	exonic	MOCOS	nonsynonymous SNV	MOCOS:ENST00000261326.5:exon1:c.C66A:p.S22R,	18q12.2	.
18	33831181	G	A	TTTTTTGGCC,TCCTTGTCAT	exonic	MOCOS	nonsynonymous SNV	MOCOS:ENST00000261326.5:exon11:c.G2099A:p.R700H,	18q12.2	.
18	52265308	A	C	TGAATCTACA,CTTCAACAGC	exonic	DYNAP	nonsynonymous SNV	DYNAP:ENST00000321600.1:exon3:c.A565C:p.T189P,DYNAP:ENST00000585973.1:exon3:c.A409C:p.T137P,	18q21.2	.
18	72343535	A	C	ACAGTTCTCA,ATGCAGCATC	exonic	ZNF407	nonsynonymous SNV	ZNF407:ENST00000577538.1:exon1:c.A560C:p.K187T,ZNF407:ENST00000309902.6:exon1:c.A560C:p.K187T,ZNF407:ENST00000582337.1:exon2:c.A560C:p.K187T,ZNF407:ENST00000299687.5:exon1 [illegible]	18q22.3	.
18	72344384	C	A	CAGATGAAAA,ACACGATGCA	exonic	ZNF407	nonsynonymous SNV	ZNF407:ENST00000577538.1:exon1:c.C1409A:p.T470K,ZNF407:ENST00000309902.6:exon1:c.C1409A:p.T470K,ZNF407:ENST00000582337.1:exon2:c.C1409A:p.T470K,ZNF407:ENST00000299687.5:ex [illegible]	18q22.3	.
19	603765	A	T	ATCAAGAAGA,GTATCTGCGC	exonic	HCN2	nonsynonymous SNV	HCN2:ENST00000251287.2:exon2:c.A854T:p.K285M,	19p13.3	.
19	4329958	G	A	GGACACTCAC,AGGGTGTCTC	exonic	STAP2	nonsynonymous SNV	STAP2:ENST00000600324.1:exon5:c.C455T:p.S152L,STAP2:ENST00000594605.1:exon5:c.C455T:p.S152L,STAP2:ENST00000601482.1:exon5:c.C455T:p.S152L,	19p13.3	.
19	5785647	C	A	GAGCAGAAAG,GCCGGGTCTT	exonic	DUS3L	nonsynonymous SNV	DUS3L:ENST00000309061.7:exon11:c.G1718T:p.R573L,DUS3L:ENST00000320699.8:exon10:c.G992T:p.R331L,	19p13.3	.
19	6678278	C	T	CGCTGCTGTC,AACCTGCACC	exonic	C3	nonsynonymous SNV	C3:ENST00000245907.6:exon40:c.G4735A:p.G1579R,	19p13.3	.
19	9049955	G	T	ATTGAAAACA,ATGGGGTTGT	exonic	MUC16	nonsynonymous SNV	MUC16:ENST00000397910.4:exon5:c.C31676A:p.S10559Y,	19p13.2	.
19	9066247	G	T	TCTGAGTGTG,AAATCTCTGA	exonic	MUC16	nonsynonymous SNV	MUC16:ENST00000397910.4:exon3:c.C21199A:p.P7067T,	19p13.2	.
19	9083770	G	A	GGTAGTCTCT,GGACTGTGCC	exonic	MUC16	nonsynonymous SNV	MUC16:ENST00000397910.4:exon1:c.C8045T:p.P2682L,	19p13.2	.
19	9089062	C	G	TCTGAGCTCA,TCACTAGGTG	exonic	MUC16	nonsynonymous SNV	MUC16:ENST00000397910.4:exon1:c.G2753C:p.S918T,	19p13.2	.
19	10079093	C	T	GGTCCCTGCA,GCCTGGCAAC	exonic	COL5A3	nonsynonymous SNV	COL5A3:ENST00000264828.3:exon59:c.G4282A:p.V1428M,	19p13.2	.
19	10401846	C	T	CAACTGCCCT,GGCCGGAGCG	exonic	ICAM5	nonsynonymous SNV	ICAM5:ENST00000221980.4:exon2:c.C181T:p.R61W,	19p13.2	.
19	10676445	C	G	GCTGCTGTCT,GGTCCCCGTA	exonic	KRI1	nonsynonymous SNV	KRI1:ENST00000544397.1:exon2:c.G125C:p.R42P,KRI1:ENST00000361821.5:exon1:c.G122C:p.R41P,KRI1:ENST00000312962.6:exon2:c.G134C:p.R45P,KRI1:ENST00000539027.1:exon2:c.G107C:p.R3 [illegible]	19p13.2	.
19	12383675	A	T	TTGTTTCATG,CTTTGAAAGG	exonic	ZNF44	nonsynonymous SNV	ZNF44:ENST00000355684.5:exon4:c.T1395A:p.S465R,ZNF44:ENST00000356109.5:exon5:c.T1539A:p.S513R,ZNF44:ENST00000397742.3:exon4:c.T1395A:p.S465R,ZNF44:ENST00000393337.3:exon5: [illegible]	19p13.2	.
19	14044770	T	C	GTCTGGCGGC,CAGGGCTCCT	exonic	PODNL1	nonsynonymous SNV	PODNL1:ENST00000591353.1:exon2:c.A157G:p.S53G,PODNL1:ENST00000538517.2:exon5:c.A436G:p.S146G,PODNL1:ENST00000339560.5:exon7:c.A709G:p.S237G,PODNL1:ENST00000586075.1:ex [illegible]	19p13.12	.
19	14211681	C	T	ATCTCCCCGC,GGGCACGTAC	exonic	PRKACA	nonsynonymous SNV	PRKACA:ENST00000593092.1:exon4:c.G325A:p.G109S,PRKACA:ENST00000589994.1:exon5:c.G352A:p.G118S,PRKACA:ENST00000308677.4:exon5:c.G376A:p.G126S,	19p13.12	.
19	14262026	C	G	GTGCAGCTCT,CGACTCGTCC	exonic	LPHN1	nonsynonymous SNV	LPHN1:ENST00000340736.6:exon24:c.G4084C:p.E1362Q,LPHN1:ENST00000361434.3:exon23:c.G4069C:p.E1357Q,	19p13.12	.
19	15067348	G	C	GAAGGGGAAG,GGTTCCGGTG	exonic	SLC1A6	nonsynonymous SNV	SLC1A6:ENST00000430939.2:exon6:c.C917G:p.P306R,SLC1A6:ENST00000221742.3:exon6:c.C1109G:p.P370R,	19p13.12	.
19	17448753	C	T	ATCGAGCCCT,GGTCTACTTG	exonic	GTPBP3	nonsynonymous SNV	GTPBP3:ENST00000600610.1:exon2:c.C124T:p.R42W,	19p13.11	.
19	17627025	T	G	AGCCAGGTGA,CACCATTAAC	exonic	PGLS	nonsynonymous SNV	PGLS:ENST00000595782.1:exon2:c.T140G:p.I47S,PGLS:ENST00000600923.1:exon3:c.T356G:p.I119S,PGLS:ENST00000252603.2:exon2:c.T332G:p.I111S,PGLS:ENST00000594761.1:exon2:c.T332G:p. [illegible]	19p13.11	.
19	18971654	G	C	TGCGAACGTG,AGAAGATCAC	exonic	UPF1	nonsynonymous SNV	UPF1:ENST00000599848.1:exon17:c.G2353C:p.E785Q,UPF1:ENST00000262803.5:exon17:c.G2320C:p.E774Q,	19p13.11	.
19	21239785	A	G	ACTCAACATA,AAGAATTCAT	exonic	ZNF430	nonsynonymous SNV	ZNF430:ENST00000261560.5:exon5:c.A671G:p.K224R,	19p12	.
19	30314670	C	T	GAGCAGCGGG,CGGAAATGGC	exonic	CCNE1	nonsynonymous SNV	CCNE1:ENST00000444983.2:exon10:c.C1174T:p.P392S,CCNE1:ENST00000576532.1:exon7:c.C838T:p.P280S,CCNE1:ENST00000357943.5:exon11:c.C1090T:p.P364S,CCNE1:ENST00000262643.3:ex [illegible]	19q12	.
19	35524836	G	A	TCCAGCCCAC,GAGAGGTCAA	exonic	SCN1B	nonsynonymous SNV	SCN1B:ENST00000415950.3:exon3:c.G641A:p.R214Q,	19q13.12	.
19	36017854	C	T	CCAGGTTGGA,ACCATGAACT	exonic	SBSN	nonsynonymous SNV	SBSN:ENST00000452271.2:exon1:c.G1330A:p.V444I,	19q13.12	.
19	36293115	A	C	AGACGTGGTC,CACATGCCCA	exonic	PRODH2	nonsynonymous SNV	PRODH2:ENST00000589835.1:exon3:c.T192G:p.C64W,PRODH2:ENST00000301175.3:exon10:c.T1404G:p.C468W,	19q13.12	.
19	36883974	T	A	TTCCTTACAG,CATAGGGCTT	exonic	ZFP82	nonsynonymous SNV	ZFP82:ENST00000392161.3:exon5:c.A1268T:p.D423V,ZFP82:ENST00000392171.1:exon4:c.A1268T:p.D423V,	19q13.12	.
19	37853491	A	G	AGCCTCCAGA,GACACAAACT	exonic	HKR1	nonsynonymous SNV	HKR1:ENST00000589392.1:exon5:c.A740G:p.K247R,HKR1:ENST00000324411.4:exon6:c.A794G:p.K265R,HKR1:ENST00000392153.3:exon5:c.A737G:p.K246R,HKR1:ENST00000541583.2:exon4:c.A611 [illegible]	19q13.12	.
19	38103688	A	G	ATGTGGGAAG,CCTTTAGATT	exonic	ZNF540	nonsynonymous SNV	ZNF540:ENST00000316433.4:exon5:c.A1507G:p.T503A,ZNF540:ENST00000586134.1:exon6:c.A1507G:p.T503A,ZNF540:ENST00000343599.5:exon5:c.A1507G:p.T503A,ZNF540:ENST00000589117.1:ex [illegible]	19q13.12	.
19	38379258	G	T	TCCCCTCTTT,GGCCAGTGTT	exonic	WDR87	nonsynonymous SNV	WDR87:ENST00000303868.5:exon6:c.C4936A:p.Q1646K,WDR87:ENST00000447313.2:exon6:c.C5053A:p.Q1685K,	19q13.13	.
19	38379830	G	A	CTGCCAAGAT,GTGTCCTCTC	exonic	WDR87	nonsynonymous SNV	WDR87:ENST00000303868.5:exon6:c.C4364T:p.P1455L,WDR87:ENST00000447313.2:exon6:c.C4481T:p.P1494L,	19q13.13	.
19	39871418	G	C	GGAGGCCAGG,CCGACAGGTA	exonic	SAMD4B	nonsynonymous SNV	SAMD4B:ENST00000314471.6:exon13:c.G1841C:p.G614A,SAMD4B:ENST00000598913.1:exon10:c.G1841C:p.G614A,	19q13.2	.
19	41858757	G	T	ACCTCCCCCT,GCTCGGGGGG	exonic	TGFB1	nonsynonymous SNV	TGFB1:ENST00000221930.5:exon1:c.C193A:p.Q65K,	19q13.2	.
19	42083797	A	G	AATATCACCC,GTGGAGATCT	exonic	CEACAM21	nonsynonymous SNV	CEACAM21:ENST00000187608.9:exon2:c.A310G:p.S104G,CEACAM21:ENST00000457737.1:exon2:c.A310G:p.S104G,CEACAM21:ENST00000401445.2:exon2:c.A310G:p.S104G,	19q13.2	.
19	42801343	G	T	CGGCTCAGAT,CAGGTAATCA	exonic	PAFAH1B3	nonsynonymous SNV	PAFAH1B3:ENST00000262890.3:exon5:c.C583A:p.H195N,PAFAH1B3:ENST00000538771.1:exon6:c.C583A:p.H195N,	19q13.2	.
19	44278690	C	G	CCACACACCA,CAGCTCCAGC	exonic	KCNN4	nonsynonymous SNV	KCNN4:ENST00000262888.3:exon3:c.G337C:p.V113L,	19q13.31	.
19	44570745	T	C	AAATTACACA,GGGAGAGAAA	exonic	ZNF223	nonsynonymous SNV	ZNF223:ENST00000591850.1:exon3:c.T656C:p.M219T,ZNF223:ENST00000591793.1:exon7:c.T1094C:p.M365T,ZNF223:ENST00000434772.3:exon5:c.T764C:p.M255T,	19q13.31	.
19	44570746	G	T	AATTACACAT,GGAGAGAAAC	exonic	ZNF223	nonsynonymous SNV	ZNF223:ENST00000591850.1:exon3:c.G657T:p.M219I,ZNF223:ENST00000591793.1:exon7:c.G1095T:p.M365I,ZNF223:ENST00000434772.3:exon5:c.G765T:p.M255I,	19q13.31	.
19	44571072	G	A	ATCCATCAGC,AGTCCACACT	exonic	ZNF223	nonsynonymous SNV	ZNF223:ENST00000591793.1:exon7:c.G1421A:p.R474Q,ZNF223:ENST00000434772.3:exon5:c.G1091A:p.R364Q,	19q13.31	ZNF223;basechange=c.1091G>C;AAchange=p.R364P;tissue=upper_aerodigestive_tract
19	44660921	G	C	ACTGTACATT,CAAATTACAC	exonic	ZNF234	nonsynonymous SNV	ZNF234:ENST00000592437.1:exon6:c.G752C:p.C251S,ZNF234:ENST00000426739.2:exon6:c.G752C:p.C251S,	19q13.31	.

19	45821197	A	C	AGGACTCCTC,TCACCAGCCA	exonic	CKM	nonsynonymous SNV	CKM:ENST00000221476.3:exon3:c.T234G:p.D78E,	19q13.32	.
19	47983614	G	C	GCCCAGAGCT,AGAGGCGCCG	exonic	KPTN	stopgain	KPTN:ENST00000338134.3:exon7:c.C650G:p.S217X,KPTN:ENST00000595554.1:exon5:c.C482G:p.S161X,	19q13.32	.
19	48377955	C	T	ATAATCAACA,TCAGGAGGGA	exonic	SULT2A1	nonsynonymous SNV	SULT2A1:ENST00000222002.3:exon5:c.G704A:p.S235N,	19q13.33	.
19	48800751	C	A	TGGCGCTGCG,CTCCGCCTGC	exonic	CCDC114	nonsynonymous SNV	CCDC114:ENST00000315396.7:exon14:c.G1495T:p.A499S,	19q13.33	.
19	49404211	C	T	GCCCTGCTAT,CAGCCAGGTG	exonic	NUCB1	nonsynonymous SNV	NUCB1:ENST00000443560.1:exon2:c.C158T:p.S53F,	19q13.33	.
19	49878196	G	A	GGACGTCCTA,AAGAGGGGAC	exonic	DKKL1	nonsynonymous SNV	DKKL1:ENST00000597546.1:exon4:c.G547A:p.E183K,DKKL1:ENST00000594268.1:exon2:c.G214A:p.E72K,DKKL1:ENST00000221498.2:exon5:c.G640A:p.E214K,DKKL1:ENST00000597873.1:exon5:c.G415A:p.E139K,	19q13.33	.
19	50365514	G	A	GGGAGGCAGA,AGGCCCTGAG	exonic	PNKP	nonsynonymous SNV	PNKP:ENST00000596014.1:exon11:c.C1054T:p.L352F,PNKP:ENST00000600573.1:exon11:c.C961T:p.L321F,PNKP:ENST00000600910.1:exon12:c.C1054T:p.L352F,PNKP:ENST00000322344.3:exon12:c.C1054T:p.L352F,	19q13.33	.
19	50413010	C	A	TTTGCAGTGC,AAACGTGAAC	exonic	NUP62	nonsynonymous SNV	NUP62:ENST00000600645.1:exon2:c.G55T:p.G19C,NUP62:ENST00000352066.3:exon3:c.G55T:p.G19C,NUP62:ENST00000599788.1:exon3:c.G55T:p.G19C,NUP62:ENST00000597723.1:exon3:c.G55T:p.G19C,NUP62:ENST00000422090.2:exon2:c.G55T:p.G19C,NUP62:ENST00000594673.1:exon4:c.G55[illegible]	19q13.33	.
19	51844823	T	G	GAATTTTGAA,CTGGGGCCTC	exonic	VSIG10L	nonsynonymous SNV	VSIG10L:ENST00000335624.4:exon2:c.A479C:p.D160A,	19q13.41	.
19	53384564	G	T	ACATTTGTAA,GTTTCTCTCC	exonic	ZNF320	nonsynonymous SNV	ZNF320:ENST00000595635.1:exon8:c.C815A:p.P272H,ZNF320:ENST00000391781.2:exon3:c.C815A:p.P272H,	19q13.41	.
19	54692322	A	G	GGGGATGGAG,TAAGAAGAAC	exonic	MBOAT7	nonsynonymous SNV	MBOAT7:ENST00000453320.1:exon2:c.T41C:p.I14T,MBOAT7:ENST00000391754.1:exon2:c.T41C:p.I14T,MBOAT7:ENST00000245615.1:exon2:c.T41C:p.I14T,MBOAT7:ENST00000414665.1:exon2:c.T41C:p.I14T,MBOAT7:ENST00000437868.1:exon2:c.T41C:p.I14T,	19q13.42	.
19	55527071	T	C	GTCTGACTCC,TTGGACTGGC	exonic	GP6	nonsynonymous SNV	GP6:ENST00000417454.1:exon7:c.A755G:p.K252R,GP6:ENST00000310373.3:exon7:c.A755G:p.K252R,GP6:ENST00000333884.2:exon6:c.A701G:p.K234R,	19q13.42	.
19	55751381	C	T	CAGCAGCTGC,GCAGCTGCTC	exonic	PPP6R1	nonsynonymous SNV	PPP6R1:ENST00000412770.2:exon12:c.G1463A:p.R488Q,PPP6R1:ENST00000587283.1:exon11:c.G1463A:p.R488Q,	19q13.42	.
19	56459614	G	C	AATGTGTGTT,TAGTCCGCAG	exonic	NLRP8	nonsynonymous SNV	NLRP8:ENST00000291971.3:exon1:c.G346C:p.V116L,NLRP8:ENST00000590542.1:exon1:c.G346C:p.V116L,	19q13.43	.
19	56572875	G	A	GAAGATGACC,GTACTGGTGG	exonic	NLRP5	nonsynonymous SNV	NLRP5:ENST00000390649.3:exon15:c.G3584A:p.R1195Q,	19q13.43	.
19	57932488	G	C	AATCATATTA,ACATCGGAGA	exonic	ZNF17	nonsynonymous SNV	ZNF17:ENST00000601808.1:exon3:c.G1628C:p.R543T,ZNF17:ENST00000307658.7:exon4:c.G1634C:p.R545T,	19q13.43	.
19	58511985	G	T	TTCCCCAGCT,TGGCAGAGCA	exonic	ZNF606	nonsynonymous SNV	ZNF606:ENST00000546715.1:exon3:c.C154A:p.Q52K,ZNF606:ENST00000552184.1:exon3:c.C154A:p.Q52K,	19q13.43	.
19	58868695	C	T	CAGCCCGGCT,CTCTGGGAGA	exonic	ZNF497	nonsynonymous SNV	ZNF497:ENST00000425453.3:exon2:c.G307A:p.E103K,ZNF497:ENST00000311044.3:exon3:c.G307A:p.E103K,	19q13.43	.
19	58928847	C	T	AGAGTTCACA,TGGAGAAAGG	exonic	ZNF584	nonsynonymous SNV	ZNF584:ENST00000306910.4:exon4:c.C962T:p.T321I,ZNF584:ENST00000593920.1:exon4:c.C827T:p.T276I,	19q13.43	.
19	59029010	G	T	TTCTGCAGGT,TATGTGATGC	exonic	ZBTB45	nonsynonymous SNV	ZBTB45:ENST00000600990.1:exon2:c.C31A:p.H11N,ZBTB45:ENST00000600130.1:exon2:c.C31A:p.H11N,ZBTB45:ENST00000596739.1:exon2:c.C31A:p.H11N,ZBTB45:ENST00000354590.3:exon2:c.C31A:p.H11N,ZBTB45:ENST00000594051.1:exon2:c.C31A:p.H11N,	19q13.43	.
20	2969076	C	T	GGAAATTAAC,GGAGAATGGC	exonic	PTPRA	nonsynonymous SNV	PTPRA:ENST00000358719.4:exon9:c.C262T:p.R88W,PTPRA:ENST00000356147.3:exon8:c.C667T:p.R223W,PTPRA:ENST00000216877.6:exon8:c.C667T:p.R223W,PTPRA:ENST00000455631.1:exon6:c.C667T:p.R223W,PTPRA:ENST00000425918.2:exon8:c.C727T:p.R243W,PTPRA:ENST00000318266.5[illegible]	20p13	.
20	3103022	G	T	TCTGCTAGAT,AGGCAGATGT	exonic	UBOX5	stopgain	UBOX5:ENST00000449731.1:exon4:c.C263A:p.S88X,UBOX5:ENST00000348031.2:exon3:c.C263A:p.S88X,UBOX5:ENST00000217173.2:exon3:c.C263A:p.S88X,	20p13	.
20	3928917	A	T	TGTGTGCTTG,TGTCTTAGCC	exonic	RNF24	nonsynonymous SNV	RNF24:ENST00000545616.2:exon4:c.T216A:p.H72Q,RNF24:ENST00000432261.2:exon3:c.T216A:p.H72Q,RNF24:ENST00000358395.6:exon3:c.T153A:p.H51Q,RNF24:ENST00000336095.6:exon3:c.T153A:p.H51Q,	20p13	.
20	7963242	G	A	TCAGCTCTAT,AGCCTCCTCT	exonic	TMX4	nonsynonymous SNV	TMX4:ENST00000246024.2:exon8:c.C706T:p.H236Y,	20p12.3	.
20	7963244	G	A	AGCTCTATGA,CCTCCTCTGA	exonic	TMX4	nonsynonymous SNV	TMX4:ENST00000246024.2:exon8:c.C704T:p.A235V,	20p12.3	.
20	35491161	G	C	GAAGGCAAGC,AGGCGCTGAG	exonic	SOGA1	nonsynonymous SNV	SOGA1:ENST00000237536.4:exon1:c.C587G:p.S196W,	20q11.23	.
20	44483822	G	C	TAGCAGTGCA,GGAGTGCACG	exonic	ACOT8	nonsynonymous SNV	ACOT8:ENST00000217455.4:exon2:c.C238G:p.L80V,ACOT8:ENST00000481938.1:exon2:c.C238G:p.L80V,ACOT8:ENST00000461272.1:exon2:c.C238G:p.L80V,ACOT8:ENST00000486165.1:exon2:c.C238G:p.L80V,ACOT8:ENST00000426915.1:exon2:c.C232G:p.L78V,	20q13.12	.
20	44505821	T	G	TCCAGAGCAG,AAGATGACCG	exonic	ZSWIM3	nonsynonymous SNV	ZSWIM3:ENST00000454862.2:exon3:c.T606G:p.S202R,ZSWIM3:ENST00000255152.2:exon2:c.T624G:p.S208R,	20q13.12	.
20	47864967	T	G	GGGGGTCGGT,GCAGGGCTCA	exonic	ZNFX1	nonsynonymous SNV	ZNFX1:ENST00000396105.1:exon14:c.A4594C:p.N1532H,ZNFX1:ENST00000371752.1:exon14:c.A4594C:p.N1532H,	20q13.13	.
20	47888204	G	A	CTTCCAGGGT,GCTGGCTCCT	exonic	ZNFX1	nonsynonymous SNV	ZNFX1:ENST00000371744.1:exon3:c.C145T:p.H49Y,ZNFX1:ENST00000396105.1:exon3:c.C145T:p.H49Y,ZNFX1:ENST00000371752.1:exon3:c.C145T:p.H49Y,ZNFX1:ENST00000371754.4:exon3:c.C145T:p.H49Y,	20q13.13	.
20	50241823	C	G	AGTTCCATCT,CATCTCCAGG	exonic	ATP9A	nonsynonymous SNV	ATP9A:ENST00000311637.5:exon13:c.G1516C:p.E506Q,ATP9A:ENST00000402822.1:exon13:c.G1561C:p.E521Q,ATP9A:ENST00000338821.5:exon18:c.G1924C:p.E642Q,	20q13.2	.
20	51871004	C	G	CCCGATTCAA,CACAGGATCT	exonic	TSHZ2	nonsynonymous SNV	TSHZ2:ENST00000603338.2:exon2:c.C998G:p.T333S,TSHZ2:ENST00000329613.6:exon2:c.C998G:p.T333S,TSHZ2:ENST00000371497.5:exon2:c.C1007G:p.T336S,	20q13.2	.
20	52645128	C	T	CCCCCTGTCT,AGGTGGGAGA	exonic	BCAS1	nonsynonymous SNV	BCAS1:ENST00000411563.1:exon2:c.G235A:p.E79K,BCAS1:ENST00000448484.1:exon1:c.G112A:p.E38K,BCAS1:ENST00000371440.3:exon4:c.G526A:p.E176K,BCAS1:ENST00000371435.2:exon4:c.G526A:p.E176K,BCAS1:ENST00000395961.3:exon4:c.G526A:p.E176K,	20q13.2	.
20	54824710	A	T	CTGCCCCACC,ACCCCTACTG	exonic	MC3R	nonsynonymous SNV	MC3R:ENST00000243911.2:exon1:c.A811T:p.N271Y,	20q13.2	.
20	54972322	C	T	TACTGTTGCC,CTGGCACAGG	exonic	CSTF1	nonsynonymous SNV	CSTF1:ENST00000428552.1:exon3:c.C229T:p.P77S,CSTF1:ENST00000452950.1:exon3:c.C229T:p.P77S,CSTF1:ENST00000415828.1:exon3:c.C229T:p.P77S,CSTF1:ENST00000217109.4:exon3:c.C229T:p.P77S,	20q13.2	.
20	62153051	G	T	GGTCATTGGT,GGCCAGCTTC	exonic	PPDPF	nonsynonymous SNV	PPDPF:ENST00000370179.3:exon4:c.G164T:p.W55L,PPDPF:ENST00000370177.1:exon3:c.G242T:p.W81L,	20q13.33	.
21	34635504	G	A	TCCACGGAGG,GTCTGGGGGC	exonic	IFNAR2	nonsynonymous SNV	IFNAR2:ENST00000382241.3:exon9:c.G1247A:p.G416E,IFNAR2:ENST00000342136.4:exon9:c.G1247A:p.G416E,	21q22.11	.
21	34922647	G	T	CCACAGCGTT,GAGCTGCAGG	exonic	SON	nonsynonymous SNV	SON:ENST00000300278.4:exon3:c.G1110T:p.L370F,SON:ENST00000356577.4:exon3:c.G1110T:p.L370F,SON:ENST00000381679.4:exon3:c.G1110T:p.L370F,SON:ENST00000455528.1:exon3:c.G1110T:p.L370F,SON:ENST00000290239.6:exon3:c.G1110T:p.L370F,	21q22.11	.
21	39672015	G	T	GAAGGAGTTT,AGCTTGTGGT	exonic	KCNJ15	stopgain	KCNJ15:ENST00000398932.1:exon4:c.G832T:p.E278X,KCNJ15:ENST00000398930.1:exon4:c.G832T:p.E278X,KCNJ15:ENST00000398934.1:exon3:c.G832T:p.E278X,KCNJ15:ENST00000398938.2:exon3:c.G832T:p.E278X,KCNJ15:ENST00000328656.4:exon4:c.G832T:p.E278X,	21q22.13	.
21	42752020	C	A	GGATCAGCTA,CGGAACACCG	exonic	MX2	stopgain	MX2:ENST00000330714.3:exon4:c.C519A:p.Y173X,	21q22.3	.
21	42824675	G	C	CAGAAGGTCA,AGAGAAGGAG	exonic	MX1	nonsynonymous SNV	MX1:ENST00000398598.3:exon16:c.G1637C:p.R546T,MX1:ENST00000398600.2:exon18:c.G1637C:p.R546T,MX1:ENST00000455164.2:exon14:c.G1637C:p.R546T,MX1:ENST00000288383.6:exon12:c.G1568C:p.R523T,	21q22.3	.
21	44838409	G	T	GGTGGAGGGG,AGACGACTAT	exonic	SIK1	nonsynonymous SNV	SIK1:ENST00000270162.6:exon12:c.C1475A:p.S492Y,	21q22.3	.
21	45649543	G	C	ACCCGCCCTG,ACACCCCACG	exonic	ICOSLG	nonsynonymous SNV	ICOSLG:ENST00000400379.3:exon6:c.C1292G:p.S431C,	21q22.3	.
21	46086635	G	C	CAGCTCACGG,CAGGCACACG	exonic	KRTAP12-2	nonsynonymous SNV	KRTAP12-2:ENST00000360770.3:exon1:c.C169G:p.P57A,	21q22.3	.
22	19178926	A	T	AGGGCTCTGT,ACAGAGCTCG	exonic	CLTCL1	nonsynonymous SNV	CLTCL1:ENST00000427926.1:exon28:c.T4213A:p.Y1405N,CLTCL1:ENST00000263200.10:exon27:c.T4213A:p.Y1405N,CLTCL1:ENST00000353891.5:exon27:c.T4213A:p.Y1405N,	22q11.21	.
22	19492953	A	C	CGGAACGAGG,TGAGGAGAAC	exonic	CDC45	nonsynonymous SNV	CDC45:ENST00000263201.1:exon10:c.A773C:p.D258A,CDC45:ENST00000407835.1:exon11:c.A773C:p.D258A,CDC45:ENST00000437685.2:exon11:c.A869C:p.D290A,CDC45:ENST00000404724.3:exon9:c.A635C:p.D212A,	22q11.21	.
22	19808769	C	T	GAGGAGCGGG,GCCCCTGAGC	exonic	GNB1L	nonsynonymous SNV	GNB1L:ENST00000453108.1:exon2:c.G110A:p.R37H,GNB1L:ENST00000329517.6:exon3:c.G110A:p.R37H,GNB1L:ENST00000405009.1:exon3:c.G110A:p.R37H,GNB1L:ENST00000403325.1:exon2:c.G110A:p.R37H,	22q11.21	.
22	29440838	T	G	GTCTGCCTCA,CCTCCTTGTC	exonic	ZNRF3	nonsynonymous SNV	ZNRF3:ENST00000402174.1:exon5:c.T404G:p.I135S,ZNRF3:ENST00000406323.3:exon4:c.T404G:p.I135S,ZNRF3:ENST00000544604.2:exon5:c.T704G:p.I235S,ZNRF3:ENST00000332811.4:exon5:c.T404G:p.I135S,	22q12.1	.
22	31011399	T	A	GAGGAGATCT,GAAGGCCCAG	exonic	TCN2	stopgain	TCN2:ENST00000407817.3:exon5:c.T611A:p.L204X,TCN2:ENST00000215838.3:exon5:c.T692A:p.L231X,TCN2:ENST00000405742.3:exon5:c.T680A:p.L227X,	22q12.2	.
22	31740976	T	A	GGCTGCATGC,GCCGGCGATG	exonic	PATZ1	nonsynonymous SNV	PATZ1:ENST00000215919.3:exon1:c.A613T:p.S205C,PATZ1:ENST00000266269.5:exon1:c.A613T:p.S205C,PATZ1:ENST00000405309.3:exon1:c.A613T:p.S205C,PATZ1:ENST00000351933.4:exon1:c.A613T:p.S205C,	22q12.2	.
22	32795682	C	T	TCCTTGTCTT,AGCCCAGGCA	exonic	RTCB	nonsynonymous SNV	RTCB:ENST00000216038.5:exon6:c.G562A:p.E188K,	22q12.3	.
22	33264993	G	A	GGAGTAGAGA,AGTTGACAGC	exonic	SYN3	nonsynonymous SNV	SYN3:ENST00000358763.2:exon5:c.C581T:p.S194F,SYN3:ENST00000332840.5:exon4:c.C581T:p.S194F,	22q12.3	.
22	38155464	C	A	CAGACAAGGC,ACTGAGCTCT	exonic	TRIOBP	nonsynonymous SNV	TRIOBP:ENST00000407319.2:exon8:c.C1226A:p.P409Q,	22q13.1	.
22	38864270	G	C	GGCGACCTGA,CCACCTCCTG	exonic	KDELR3	nonsynonymous SNV	KDELR3:ENST00000216014.4:exon1:c.G32C:p.S11T,KDELR3:ENST00000409006.3:exon1:c.G32C:p.S11T,	22q13.1	.
22	41257383	C	T	TGATCACTTT,AATAATTTTC	exonic	DNAJB7	nonsynonymous SNV	DNAJB7:ENST00000307221.4:exon1:c.G616A:p.E206K,	22q13.2	DNAJB7;basechange=c.616G>A;AAchange=p.E206K;tissue=breast
22	41904000	G	C	CATCCACTGT,ACCATCTGAT	exonic	ACO2	nonsynonymous SNV	ACO2:ENST00000216254.4:exon3:c.G379C:p.D127H,ACO2:ENST00000396512.3:exon3:c.G379C:p.D127H,	22q13.2	.
22	45757732	G	T	TCAACAGCCA,AAACTTCTTC	exonic	SMC1B	nonsynonymous SNV	SMC1B:ENST00000357450.4:exon17:c.C2695A:p.L899M,SMC1B:ENST00000404354.3:exon17:c.C2695A:p.L899M,	22q13.31	.
22	45944619	G	T	GGCCGCAACT,CCAAGGTGAG	exonic	FBLN1	nonsynonymous SNV	FBLN1:ENST00000262722.7:exon13:c.G1568T:p.C523F,FBLN1:ENST00000327858.6:exon13:c.G1568T:p.C523F,FBLN1:ENST00000442170.2:exon13:c.G1568T:p.C523F,FBLN1:ENST00000340923.5:exon13:c.G1568T:p.C523F,FBLN1:ENST00000402984.3:exon14:c.G1682T:p.C561F,FBLN1:ENST000003[illegible]	22q13.31	.
22	50616546	A	T	GAAGACGGCC,CGGACACGCT	exonic	PANX2	nonsynonymous SNV	PANX2:ENST00000159647.5:exon2:c.A1405T:p.T469S,PANX2:ENST00000395842.2:exon2:c.A1405T:p.T469S,	22q13.33	.
X	12905616	T	A	ATTCCCTAAG,TTCTTGCCTT	exonic	TLR7	nonsynonymous SNV	TLR7:ENST00000380659.3:exon3:c.T1989A:p.S663R,	Xp22.2	.
X	15588452	C	T	TAGGCTTATC,TCACTTTGAT	exonic	ACE2	nonsynonymous SNV	ACE2:ENST00000427411.1:exon15:c.G1862A:p.R621K,ACE2:ENST00000252519.3:exon14:c.G1862A:p.R621K,	Xp22.2	.
X	19017149	A	C	CAGATACATG,AGGTCACGTT	exonic	GPR64	nonsynonymous SNV	GPR64:ENST00000379869.3:exon26:c.T2579G:p.F860C,GPR64:ENST00000379878.3:exon25:c.T2531G:p.F844C,GPR64:ENST00000379873.2:exon26:c.T2579G:p.F860C,GPR64:ENST00000356606.4:exon25:c.T2537G:p.F846C,GPR64:ENST00000340581.3:exon21:c.T2222G:p.F741C,GPR64:ENST0000[illegible]	Xp22.13	.
X	20044018	G	A	GGAGACCCTG,GTAGGGAGGT	exonic	MAP7D2	nonsynonymous SNV	MAP7D2:ENST00000379643.5:exon9:c.C1060T:p.P354S,MAP7D2:ENST00000443379.3:exon7:c.C802T:p.P268S,MAP7D2:ENST00000379651.3:exon8:c.C937T:p.P313S,MAP7D2:ENST00000543767.1:exon6:c.C592T:p.P198S,MAP7D2:ENST00000452324.3:exon8:c.C781T:p.P261S,	Xp22.12	.
X	22292216	G	T	TCAGTTCACC,AAAATCAAGA	exonic	ZNF645	stopgain	ZNF645:ENST00000323684.1:exon1:c.G1108T:p.E370X,	Xp22.11	.
X	26157157	C	T	CCACCAGGCT,GTTGTGAGAA	exonic	MAGEB18	nonsynonymous SNV	MAGEB18:ENST00000325250.1:exon2:c.C55T:p.R19C,	Xp21.3	.
X	26157705	G	A	GCCTCCTGAT,ATTGCACTGG	exonic	MAGEB18	nonsynonymous SNV	MAGEB18:ENST00000325250.1:exon2:c.G603A:p.M201I,	Xp21.3	.
X	37027444	G	C	TCTCCGCCCA,AGCCTTCTGA	exonic	FAM47C	nonsynonymous SNV	FAM47C:ENST00000358047.3:exon1:c.G961C:p.E321Q,	Xp21.1	.
X	41010197	A	T	AAAAGATCCA,TGGATAGATC	exonic	USP9X	nonsynonymous SNV	USP9X:ENST00000324545.8:exon13:c.A1650T:p.Q550H,USP9X:ENST00000378308.2:exon13:c.A1650T:p.Q550H,	Xp11.4	.
X	44919984	G	A	CCTGCCAAGA,GAAAAGAACA	exonic	KDM6A	nonsynonymous SNV	KDM6A:ENST00000382899.4:exon13:c.G1325A:p.R442K,	Xp11.3	.
X	45013157	T	G	CTGAGTACCT,CCTGCTTGTC	exonic	CXorf36	nonsynonymous SNV	CXorf36:ENST00000398000.2:exon4:c.A959C:p.E320A,	Xp11.3	.
X	48970829	C	T	ATCACACGGT,ACCCTCTGCC	exonic	GPKOW	nonsynonymous SNV	GPKOW:ENST00000156109.5:exon10:c.G1258A:p.D420N,	Xp11.23	.
X	48970831	C	T	CACACGGTCA,CCTCTGCCTT	exonic	GPKOW	nonsynonymous SNV	GPKOW:ENST00000156109.5:exon10:c.G1256A:p.G419D,	Xp11.23	.
X	54470948	G	A	GGATATAGTG,AAGATGGCTG	exonic	TSR2	nonsynonymous SNV	TSR2:ENST00000375151.4:exon5:c.G541A:p.E181K,	Xp11.22	.
X	65428077	C	T	TGGCCACTGG,TGCTGAGCCT	exonic	HEPH	nonsynonymous SNV	HEPH:ENST00000419594.1:exon12:c.C1985T:p.A662V,HEPH:ENST00000343002.2:exon14:c.C2552T:p.A851V,HEPH:ENST00000441993.2:exon15:c.C2561T:p.A854V,HEPH:ENST00000336279.5:exon14:c.C1751T:p.A584V,HEPH:ENST00000519389.1:exon15:c.C2714T:p.A905V,HEPH:ENST0000037472[illegible]	Xq12	.
X	70525021	G	T	AGGAGGAAGA,GAGGAAGCAA	exonic	ITGB1BP2	nonsynonymous SNV	ITGB1BP2:ENST00000373829.3:exon11:c.G1023T:p.E341D,ITGB1BP2:ENST00000538820.1:exon10:c.G969T:p.E323D,	Xq13.1	.
X	70756210	T	C	GCTGGACAGG,AGGAGATGTT	splicing	OGT(ENST0[illegible]	.	.	Xq13.1	.
X	75003656	T	G	ATCAGGAAGA,CAAACCTAGG	exonic	MAGEE2	nonsynonymous SNV	MAGEE2:ENST00000373359.2:exon1:c.A1231C:p.I411L,	Xq13.3	.
X	75397477	G	A	CTTTGCCATT,AAATTGCTCG	exonic	PBDC1	nonsynonymous SNV	PBDC1:ENST00000373358.3:exon6:c.G436A:p.E146K,	Xq13.3	.
X	92964870	C	G	GAATCAGAAT,AGAGAGCAAG	exonic	FAM133A	stopgain	FAM133A:ENST00000355813.5:exon4:c.C452G:p.S151X,FAM133A:ENST00000538690.1:exon5:c.C452G:p.S151X,FAM133A:ENST00000322139.4:exon3:c.C452G:p.S151X,FAM133A:ENST00000332647.4:exon4:c.C452G:p.S151X,	Xq21.32	.
X	99661971	G	A	GCTTTGCAGT,AGGGAAGGCC	exonic	PCDH19	nonsynonymous SNV	PCDH19:ENST00000420881.2:exon1:c.C1625T:p.S542L,PCDH19:ENST00000373034.4:exon1:c.C1625T:p.S542L,PCDH19:ENST00000255531.7:exon1:c.C1625T:p.S542L,	Xq22.1	.
X	99887545	C	T	CTATAATGGT,ATCACCTTTA	exonic	TSPAN6	nonsynonymous SNV	TSPAN6:ENST00000373020.4:exon6:c.G606A:p.M202I,	Xq22.1	.
X	109697147	A	C	AAAGCCACAG,CTCTGGAGAG	exonic	RGAG1	nonsynonymous SNV	RGAG1:ENST00000540313.1:exon1:c.A3302C:p.D1101A,RGAG1:ENST00000465301.2:exon3:c.A3302C:p.D1101A,	Xq23	.
X	110497499	C	A	AGGGATAATA,CTTTGTCCAA	splicing	CAPN6(ENS[illegible]	.	.	Xq23	.
X	118974647	C	T	AACCTGTGTA,CTGAAGACAG	exonic	UPF3B	nonsynonymous SNV	UPF3B:ENST00000276201.2:exon8:c.G808A:p.V270I,	Xq24	.
X	129155048	G	A	AATGGAGTCA,GGGAAAGCAC	exonic	BCORL1	nonsynonymous SNV	BCORL1:ENST00000540052.1:exon4:c.G3530A:p.R1177K,BCORL1:ENST00000303743.5:exon5:c.G3530A:p.R1177K,BCORL1:ENST00000218147.7:exon5:c.G3530A:p.R1177K,BCORL1:ENST00000456822.1:exon2:c.G2330A:p.R777K,BCORL1:ENST00000359304.2:exon5:c.G3530A:p.R1177K,	Xq26.1	.
X	135126834	C	G	ATGGATGATT,TGAACCCCCG	exonic	SLC9A6	nonsynonymous SNV	SLC9A6:ENST00000370701.1:exon17:c.C1901G:p.S634C,SLC9A6:ENST00000370698.3:exon16:c.C1961G:p.S654C,SLC9A6:ENST00000370695.4:exon16:c.C2057G:p.S686C,	Xq26.3	.
X	135429795	C	A	TTTCCAGTCA,CAAACACATT	exonic	GPR112	nonsynonymous SNV	GPR112:ENST00000287534.4:exon4:c.C3741A:p.H1247Q,GPR112:ENST00000394141.1:exon3:c.C3315A:p.H1105Q,GPR112:ENST00000412101.1:exon3:c.C3315A:p.H1105Q,GPR112:ENST00000394143.1:exon6:c.C3930A:p.H1310Q,GPR112:ENST00000370652.1:exon4:c.C3930A:p.H1310Q,	Xq26.3	.
X	135572387	T	C	GTGTCTATGA,ATTTGCTCTA	exonic	BRS3	nonsynonymous SNV	BRS3:ENST00000370648.3:exon2:c.T530C:p.I177T,	Xq26.3	.
X	148685720	G	A	AATGTTGACA,AACACAGGAT	exonic	TMEM185A	nonsynonymous SNV	TMEM185A:ENST00000536359.1:exon3:c.C263T:p.S88F,TMEM185A:ENST00000507237.1:exon4:c.C440T:p.S147F,TMEM185A:ENST00000316916.8:exon4:c.C440T:p.S147F,	Xq28	.
X	153707187	C	A	GCCCCCGCGG,AGCTGTGGCC	exonic	LAGE3	nonsynonymous SNV	LAGE3:ENST00000407062.1:exon1:c.G68T:p.C23F,LAGE3:ENST00000357360.4:exon1:c.G68T:p.C23F,	Xq28	.
X	154157860	G	A	AGATCTATTT,CTTGAGGGAT	exonic	F8	nonsynonymous SNV	F8:ENST00000360256.4:exon14:c.C4205T:p.A1402V,	Xq28	.

196*;tissue=ovary,breast,stomach,haematopoietic_and_lymphoid_tissue,urinary_tract,kidney,pancreas,liver,oesophagus,skin,lung,upper_aerodigestive_tract,large_intestine,central_nervous_system,biliary_tract&TP53_ENST00000269305;basechange=c.586C>T;AAchange=p.R196*;tissue=breast,stomach,haematopoietic_and_lymphoid_tissue,oesophagus,lung,upper_aerodigestive_tract,large_intestine,central_nervous_system,biliary_tract&TP53_ENST00000455263;basechange=c.586C>T;AAchange=p.R196*;tissue=stomach,lung,large_intestine&TP53_ENST00000413465;basechange=c.586C>T;AAchange=p.R196*;tissue=breast,haematopoietic_and_lymphoid_tissue,stomach,oesophagus,lung,upper_aerodigestive_tract,large_intestine

Supplemental Table 3: indel_C000-2SXE39_somatic_funct

Patient ID	Chromosome	Position (hg19)	Reference allele	Mutant allele	ANNOVAR function	Gene	Mutation type	ANNOVAR output (alternative transcripts)	Cytoband	COSMIC
C000-2SXE39	1	45813636	CAG	C	exonic	TESK2	frameshift deletion	TESK2:ENST00000538496.1:exon5:c.326_327del:p.S109fs,TESK2:ENST00000341771.6:exon6:c.575_576del:p.S192fs,TESK2:ENST00000372084.1:exon5:c.575_576del:p.S192fs,TESK2:ENST00000372086.3:exon6:c.575_576del:p.S192fs,	1p34.1	.
C000-2SXE39	2	80773113	TG	T	exonic	CTNNA2	frameshift deletion	CTNNA2:ENST00000343114.3:exon5:c.503delG:p.W168fs,CTNNA2:ENST00000540488.1:exon10:c.1466delG:p.W489fs,CTNNA2:ENST00000402739.4:exon10:c.1466delG:p.W489fs,CTNNA2:ENST00000496558.1:exon11:c.1466delG:p.W489fs,CTNNA2:ENST00000541047.1:exon10:c.1466delG:p.W489fs,CTNNA2:ENST00000466387.1:exon15:c.1466delG:p.W489fs,CTNNA	2p12	.
C000-2SXE39	2	152534076	CA	C	splicing	NEB(ENST00000409198.1:exon34:c.3774+2T>-,ENS	.	.	2q23.3	.
C000-2SXE39	2	157184438	C	CG	exonic	NR4A2	frameshift insertion	NR4A2:ENST00000426264.1:exon5:c.892dupC:p.P298fs,NR4A2:ENST00000409108.2:exon4:c.1081dupC:p.P361fs,NR4A2:ENST00000409572.1:exon5:c.1081dupC:p.P361fs,NR4A2:ENST00000417972.1:exon5:c.892dupC:p.P298fs,NR4A2:ENST00000417764.1:exon4:c.892dupC:p.P298fs,NR4A2:ENST00000539077.1:exon4:c.1114dupC:p.P372fs,NR4A2:ENST0000042937	2q24.1	.
C000-2SXE39	3	123699222	C	CA	exonic	ROPN1	frameshift insertion	ROPN1:ENST00000405845.3:exon2:c.106dupT:p.W36fs,ROPN1:ENST00000460743.1:exon4:c.106dupT:p.W36fs,ROPN1:ENST00000484329.1:exon3:c.106dupT:p.W36fs,ROPN1:ENST00000496145.1:exon3:c.106dupT:p.W36fs,ROPN1:ENST00000459660.1:exon3:c.106dupT:p.W36fs,ROPN1:ENST00000467907.1:exon3:c.106dupT:p.W36fs,ROPN1:ENST00000479867.1:exo	3q21.1	.
C000-2SXE39	3	169684617	G	GA	exonic	SEC62	frameshift insertion	SEC62:ENST00000460513.1:exon1:c.8dupA:p.E3fs,SEC62:ENST00000337002.4:exon1:c.8dupA:p.E3fs,SEC62:ENST00000469515.2:exon1:c.8dupA:p.E3fs,SEC62:ENST00000480708.1:exon1:c.8dupA:p.E3fs,SEC62:ENST00000487736.1:exon1:c.8dupA:p.E3fs,	3q26.2	SEC62;basechange=c.7G>A;AAchange=p.E3K;tissues=breast
C000-2SXE39	4	144618280	AG	A	exonic	FREM3	frameshift deletion	FREM3:ENST00000329798.5:exon1:c.3548delC:p.P1183fs,	4q31.21	.
C000-2SXE39	4	156632028	TC	T	exonic	GUCY1A3	frameshift deletion	GUCY1A3:ENST00000455639.2:exon6:c.712delC:p.P238fs,GUCY1A3:ENST00000511108.1:exon7:c.712delC:p.P238fs,GUCY1A3:ENST00000513574.1:exon5:c.712delC:p.P238fs,GUCY1A3:ENST00000511507.1:exon6:c.712delC:p.P238fs,GUCY1A3:ENST00000506455.1:exon6:c.712delC:p.P238fs,GUCY1A3:ENST00000296518.7:exon6:c.712delC:p.P238fs,	4q32.1	.
C000-2SXE39	5	61673530	G	GTT	exonic	KIF2A	frameshift insertion	KIF2A:ENST00000512006.2:exon4:c.365_366insTT:p.V122fs,KIF2A:ENST00000381103.2:exon19:c.1781_1782insTT:p.V594fs,KIF2A:ENST00000401507.3:exon18:c.1841_1842insTT:p.V614fs,KIF2A:ENST00000407818.3:exon19:c.1955_1956insTT:p.V652fs,KIF2A:ENST00000506857.1:exon18:c.1703_1704insTT:p.V568fs,	5q12.1	.
C000-2SXE39	5	102469213	CA	C	exonic	PPIP5K2	frameshift deletion	PPIP5K2:ENST00000358359.3:exon3:c.172delA:p.K58fs,PPIP5K2:ENST00000321521.9:exon3:c.172delA:p.K58fs,PPIP5K2:ENST00000414217.1:exon2:c.172delA:p.K58fs,PPIP5K2:ENST00000515845.1:exon3:c.172delA:p.K58fs,	5q21.1	.
C000-2SXE39	6	108042020	GC	G	exonic	SCML4	frameshift deletion	SCML4:ENST00000369021.3:exon5:c.772delG:p.A258fs,SCML4:ENST00000369022.2:exon5:c.685delG:p.A229fs,SCML4:ENST00000369020.3:exon6:c.859delG:p.A287fs,SCML4:ENST00000369025.2:exon2:c.133delG:p.A45fs,	6q21	.
C000-2SXE39	8	125499109	AGTTAT	A	exonic	RNF139	frameshift deletion	RNF139:ENST00000303545.3:exon2:c.1220_1224del:p.S407fs,	8q24.13	.
C000-2SXE39	10	108431121	TC	T	exonic	SORCS1	frameshift deletion	SORCS1:ENST00000369698.1:exon8:c.667delG:p.E223fs,SORCS1:ENST00000263054.6:exon16:c.2062delG:p.E688fs,SORCS1:ENST00000344440.6:exon16:c.2062delG:p.E688fs,	10q25.1	.
C000-2SXE39	11	5510674	C	CA	exonic	OR52D1	frameshift insertion	OR52D1:ENST00000322641.5:exon1:c.740dupA:p.H247fs,	11p15.4	OR52D1;basechange=c.738C>A;AAchange=p.S246S;tissues=soft_tissue,lung
C000-2SXE39	12	15262411	TC	T	exonic	RERG	frameshift deletion	RERG:ENST00000256953.2:exon5:c.232delG:p.E78fs,RERG:ENST00000546331.1:exon4:c.175delG:p.E59fs,RERG:ENST00000545567.1:exon4:c.271delG:p.E91fs,RERG:ENST00000538313.1:exon4:c.232delG:p.E78fs,RERG:ENST00000536465.1:exon5:c.232delG:p.E78fs,	12p12.3	RERG;basechange=c.233A>G;AAchange=p.E78G;tissues=large_intestine&RERG;basechange=c.232G>A;AAchange=p.E78K;tissues=skin,upper_aerodigestive_tract
C000-2SXE39	12	52200888	TC	T	exonic	SCN8A	frameshift deletion	SCN8A:ENST00000545061.1:exon25:c.5496delC:p.F1832fs,SCN8A:ENST00000354534.6:exon27:c.5619delC:p.F1873fs,	12q13.13	SCN8A_ENST00000354534;basechange=c.5619C>T;AAchange=p.F1873F;tissues=skin,endometrium&SCN8A;basechange=c.5619C>T;AAchange=p.F1873F;tissues=skin,endometrium
C000-2SXE39	13	101760069	CT	C	exonic	NALCN	frameshift deletion	NALCN:ENST00000251127.6:exon21:c.2435delA:p.K812fs,	13q33.1	.
C000-2SXE39	15	87531228	TC	T	exonic	AGBL1	frameshift deletion	AGBL1:ENST00000441037.2:exon23:c.3095delC:p.S1032fs,AGBL1:ENST00000421325.2:exon22:c.3095delC:p.S1032fs,AGBL1:ENST00000389298.3:exon16:c.2288delC:p.S763fs,	15q25.3	.
C000-2SXE39	16	31090553	C	CT	exonic	ZNF646	frameshift insertion	ZNF646:ENST00000300850.5:exon2:c.2909dupT:p.L970fs,ZNF646:ENST00000394979.2:exon1:c.2909dupT:p.L970fs,	16p11.2	.
C000-2SXE39	16	53279692	TAA	T	exonic	CHD9	frameshift deletion	CHD9:ENST00000564845.1:exon15:c.3385_3386del:p.N1129fs,CHD9:ENST00000565803.1:exon14:c.1963_1964del:p.N655fs,CHD9:ENST00000447540.1:exon15:c.3385_3386del:p.N1129fs,CHD9:ENST00000566029.1:exon15:c.3385_3386del:p.N1129fs,CHD9:ENST00000398510.3:exon14:c.3385_3386del:p.N1129fs,	16q12.2	.
C000-2SXE39	16	88798759	TAGA	T	exonic	PIEZO1	nonframeshift deletion	PIEZO1:ENST00000301015.9:exon21:c.2972_2974del:p.991_992del,	16q24.3	.
C000-2SXE39	17	76421613	TG	T	exonic	DNAH17	frameshift deletion	DNAH17:ENST00000585328.1:exon80:c.12939delC:p.T4313fs,DNAH17:ENST00000389840.5:exon80:c.13023delC:p.T4341fs,	17q25.3	.
C000-2SXE39	X	51486946	GC	G	exonic	GSPT2	frameshift deletion	GSPT2:ENST00000340438.4:exon1:c.225delC:p.G75fs,	Xp11.22	.
C000-2SXE39	X	72433663	GTCC	G	exonic	NAP1L2	nonframeshift deletion	NAP1L2:ENST00000536638.1:exon2:c.237_239del:p.79_80del,NAP1L2:ENST00000373517.3:exon1:c.663_665del:p.221_222del,	Xq13.2	.
C000-6KNGDL	6	31600299	TC	T	exonic	PRRC2A	frameshift deletion	PRRC2A:ENST00000376007.4:exon16:c.3850delC:p.P1284fs,PRRC2A:ENST00000376033.2:exon16:c.3850delC:p.P1284fs,	6p21.33	.
C000-6KNGDL	8	101724623	C	CA	exonic	PABPC1	frameshift insertion	PABPC1:ENST00000318607.5:exon7:c.937_938insT:p.E313fs,PABPC1:ENST00000522387.1:exon8:c.841_842insT:p.E281fs,PABPC1:ENST00000519004.1:exon8:c.802_803insT:p.E268fs,	8q22.3	.
C000-6KNGDL	8	101724625	CTT	C	exonic	PABPC1	frameshift deletion	PABPC1:ENST00000318607.5:exon7:c.935_936del:p.K312fs,PABPC1:ENST00000522387.1:exon8:c.839_840del:p.K280fs,PABPC1:ENST00000519004.1:exon8:c.800_801del:p.K267fs,	8q22.3	.
C000-6KNGDL	11	2979735	GA	G	exonic	NAP1L4	frameshift deletion	NAP1L4:ENST00000526115.1:exon11:c.785delT:p.V262fs,NAP1L4:ENST00000492685.1:exon4:c.353delT:p.V118fs,NAP1L4:ENST00000532325.2:exon8:c.440delT:p.V147fs,NAP1L4:ENST00000448187.1:exon10:c.821delT:p.V274fs,NAP1L4:ENST00000380542.4:exon10:c.785delT:p.V262fs,	11p15.4	.
C000-EXKBCS	1	57192256	TCTGAG	T	splicing	C1orf168	.	.	1p32.2	.
C000-HZGGV2	1	114515717	GTTT	G	exonic	HIPK1	nonframeshift deletion	HIPK1:ENST00000369555.2:exon15:c.3082_3084del:p.1028_1028del,HIPK1:ENST00000369553.1:exon14:c.2035_2037del:p.679_679del,HIPK1:ENST00000369554.2:exon15:c.3082_3084del:p.1028_1028del,HIPK1:ENST00000369561.4:exon16:c.3115_3117del:p.1039_1039del,HIPK1:ENST00000340480.4:exon15:c.2095_2097del:p.699_699del,HIPK1:ENST00000426820.2:e	1p13.2	HIPK1_ENST00000406344;basechange=c.2034G>A;AAchange=p.A678A;tissues=breast&HIPK1_ENST00000369558;basechange=c.3216G>A;AAchange=p.A1072A;tissues=breast,thyroid
C000-HZGGV2	2	73678878	CCT	C	exonic	ALMS1	frameshift deletion	ALMS1:ENST00000423048.1:exon1:c.56_57del:p.P19fs,ALMS1:ENST00000264448.6:exon8:c.5222_5223del:p.P1741fs,ALMS1:ENST00000377715.1:exon8:c.5222_5223del:p.P1741fs,ALMS1:ENST00000409009.1:exon7:c.5096_5097del:p.P1699fs,	2p13.1	.
C000-HZGGV2	6	117243195	TCCTGGGTAG	T	splicing	RFX6	.	.	6q22.1	.
C000-HZGGV2	9	112172556	TC	T	exonic	PTPN3	frameshift deletion	PTPN3:ENST00000262539.3:exon13:c.990delG:p.V330fs,PTPN3:ENST00000412145.1:exon10:c.1059delG:p.V353fs,PTPN3:ENST00000446349.1:exon9:c.924delG:p.V308fs,PTPN3:ENST00000374541.2:exon15:c.1452delG:p.V484fs,	9q31.3	.
C000-HZGGV2	11	65620371	TCTC	T	exonic	SNX32	nonframeshift deletion	SNX32:ENST00000308342.6:exon12:c.1101_1103del:p.367_368del,	11q13.1	.
C000-HZGGV2	12	72026281	AT	A	exonic	ZFC3H1	stopgain	ZFC3H1:ENST00000378743.3:exon15:c.2830delA:p.M944X,ZFC3H1:ENST00000552994.1:exon15:c.2830delA:p.M944X,	12q21.1	.
C000-HZGGV2	17	7318427	C	CCCTCCCGGG	exonic	NLGN2	nonframeshift insertion	NLGN2:ENST00000302926.2:exon5:c.998_999insCCTCCCGGG:p.P333delinsPLPG,NLGN2:ENST00000575301.1:exon6:c.998_999insCCTCCCGGG:p.P333delinsPLPG,	17p13.1	.
C000-HZGGV2	17	7577063	TTCTTGCGGAGATTCTCTTCC	T	exonic	TP53	frameshift deletion	TP53:ENST00000269305.4:exon8:c.855_874del:p.E285fs,TP53:ENST00000445888.2:exon8:c.855_874del:p.E285fs,TP53:ENST00000420246.2:exon8:c.855_874del:p.E285fs,TP53:ENST00000359597.4:exon7:c.855_874del:p.E285fs,TP53:ENST00000509690.1:exon5:c.459_478del:p.E153fs,TP53:ENST00000455263.2:exon8:c.855_874del:p.E285fs,	17p13.1	TP53;basechange=c.875A>G;AAchange=p.K292R;tissues=stomach,oesophagus,thyroid,cervix,large_intestine&TP53;basechange=c.875A>C;AAchange=p.K292T;tissues=upper_aerodigestive_tract,large_intestine&TP53_ENST00000269305;basechange=c.874A>T;AAchange=p.K292*;tissues=oesophagus&TP53;basechange=c.874A>T;AAchange=p.K292*;tissues=urinary_tract,oesophagus,upper_aerodigestive_tract&TP53;basechange=c.874A>G;AAchange=p.K292E;tissues=urinary_tract&TP53_ENST00000455263;basechange=c.873G>A;AAchange=p.K291K;tissues=cervix&TP53_ENST00000420246;basechange=c.873G>A;AAchange=p.K291K;tissues=cervix&TP53;basechange=c.873G>A;AAchange=p.K291K;tissues=urinary_tract,cervix&TP53;basechange=c.873G>C;AAchange=p.K291N;tissues=breast,urinary_tract,pancreas&TP53_ENST00000269305;basechange=c.873G>A;AAchange=p.K291K;tissues=cervix&TP53;basechange=c.872A>C;AAchange=p.K291T;tissues=urinary_tract,prostate,lung&TP53;basechange=c.872A>G;AAchange=p.K291R;tissues=lung,upper_aerodigestive_tract,central_nervous_system&TP53;basechange=c.872A>T;AAchange=p.K291M;tissues=ovary&TP53_ENST00000269305;basechange=c.871A>T;AAchange=p.K291*;tissues=ovary&TP53;basechange=c.871A>T;AAchange=p.K291*;tissues=ovary,urinary_tract,liver,upper_aerodigestive_tract,large_intestine&TP53;basechange=c.871A>G;AAchange=p.K291E;tissues=lung,biliary_tract&TP53;basechange=c.871A>C;AAchange=p.K291Q;tissues=soft_tissue&TP53_ENST00000420246;basechange=c.871A>T;AAchange=p.K291*;tissues=ovary&TP53_ENST00000455263;basechange=c.871A>T;AAchange=p.K291*;tissues=ovary&TP53;basechange=c.870C>A;AAchange=p.R290R;tissues=oesophagus&TP53;basechange=c.870C>G;AAchange=p.R290R;tissues=skin&TP53_ENST00000455263;basechange=c.869G>A;AAchange=p.R290H;tissues=haematopoietic_and_lymphoid_tissue&TP53_ENST00000269305;basechange=c.869G>A;AAchange=p.R290H;tissues=haematopoietic_and_lymphoid_tissue&TP53;basechange=c.869G>T;AAchange=p.R290L;tissues=ovary,vulva,upper_aerodigestive_tract&TP53_ENST00000420246;basechange=c.869G>A;AAchange=p.R290H;tissues=haematopoietic_and_lymphoid_tissue&TP53;basechange=c.869G>A;AAchange=p.R290H;tissues=breast,stomach,haematopoietic_and_lymphoid_tissue,urinary_tract,skin,upper_aerodigestive_tract,large_intestine,central_nervous_system&TP53_ENST00000420246;basechange=c.868C>T;AAchange=p.R290C;tissues=skin,upper_aerodigestive_tract&TP53;basechange=c.868C>T;AAchange=p.R290C;tissues=haematopoietic_and_lymphoid_tissue,skin,thyroid,upper_aerodigestive_tract&TP53_ENST00000455263;basechange=c.868C>T;AAchange=p.R290C;tissues=skin,upper_aerodigestive_tract&TP53;basechange=c.868C>A;AAchange=p.R290S;tissues=cervix&TP53_ENST00000269305;basechange=c.868C>T;AAchange=p.R290C;tissues=skin,upper_aerodigestive_tract&TP53_ENST00000455263;basechange=c.867C>T;AAchange=p.L289L;tissues=skin&TP53_ENST00000269305;basechange=c.867C>T;AAchange=p.L289L;tissues=skin&TP53;basechange=c.867C>T;AAchange=p.L289L;tissues=soft_tissue,skin,upper_aerodigestive_tract&TP53_ENST00000420246;basechange=c.867C>T;AAchange=p.L289L;tissues=skin&TP53;basechange=c.866T>G;AAchange=p.L289R;tissues=central_nervous_system&TP53;basechange=c.866T>C;AAchange=p.L289P;tissues=oesophagus,lung&TP53_ENST00000455263;basechange=c.865C>T;AAchange=p.L289F;tissues=breast&TP53_ENST00000269305;basechange=c.865C>T;AAchange=p.L289F;tissues=breast&TP53;basechange=c.865C>T;AAchange=p.L289F;tissues=breast,haematopoietic_and_lymphoid_tissue,large_intestine&TP53_ENST00000420246;basechange=c.865C>T;AAchange=p.L289F;tissues=breast&TP53;basechange=c.865C>G;AAchange=p.L289V;tissues=lung&TP53;basechange=c.864T>A;AAchange=p.N288K;tissues=stomach&TP53;basechange=c.863A>G;AAchange=p.N288S;tissues=urinary_tract,prostate,lung,upper_aerodigestive_tract,biliary_tract&TP53;basechange=c.862A>G;AAchange=p.N288D;tissues=urinary_tract&TP53;basechange=c.862A>T;AAchange=p.N288Y;tissues=stomach,soft_tissue,liver&TP53_ENST00000455263;basechange=c.861G>A;AAchange=p.E287E;tissues=urinary_tract,cervix&TP53_ENST00000420246;basechange=c.861G>A;AAchange=p.E287E;tissues=urinary_tract,cervix&TP53;basechange=c.861G>A;AAchange=p.E287E;tissues=stomach,urinary_tract,cervix&TP53_ENST00000269305;basechange=c.861G>C;AAchange=p.E287D;tissues=cervix&TP53_ENST00000420246;basechange=c.861G>T;AAchange=p.E287D;tissues=breast&TP53_ENST00000269305;basechange=c.861G>T;AAchange=p.E287D;tissues=breast&TP53_ENST00000455263;basechange=c.861G>C;AAchange=p.E287D;tissues=cervix&TP53_ENST00000455263;basechange=c.861G>T;AAchange=p.E287D;tissues=breast&TP53;basechange=c.861G>C;AAchange=p.E287D;tissues=vulva,lung,cervix&TP53_ENST00000269305;basechange=c.861G>A;AAchange=p.E287E;tissues=urinary_tract,cervix&TP53;basechange=c.861G>T;AAchange=p.E287D;tissues=breast,haematopoietic_and_lymphoid_tissue,liver&TP53_ENST00000420246;basechange=c.861G>C;AAchange=p.E287D;tissues=cervix&TP53;basechange=c.860A>G;AAchange=p.E287G;tissues=breast&TP53;basechange=c.860A>C;AAchange=p.E287A;tissues=upper_aerodigestive_tract&TP53;basechange=c.860A>T;AAchange=p.E287V;tissues=bone&TP53_ENST00000269305;basechange=c.859G>T;AAchange=p.E287*;tissues=breast,liver,oesophagus,lung,large_intestine,biliary_tract&TP53_ENST00000455263;basechange=c.859G>T;AAchange=p.E287*;tissues=liver,oesophagus,lung,large_intestine,biliary_tract&TP53_ENST00000420246;basechange=c.859G>T;AAchange=p.E287*;tissues=liver,oesophagus,lung,large_intestine,biliary_tract&TP53;basechange=c.859G>A;AAchange=p.E287K;tissues=breast,stomach,urinary_tract,liver,skin&TP53;basechange=c.859G>T;AAchange=p.E287*;tissues=breast,urinary_tract,pancreas,liver,oesophagus,lung,large_intestine,biliary_tract&TP53;basechange=c.858A>C;AAchange=p.E286D;tissues=liver&TP53;basechange=c.858A>T;AAchange=p.E286D;tissues=urinary_tract&TP53_ENST00000455263;basechange=c.858A>C;AAchange=p.E286D;tissues=liver&TP53;basechange=c.858A>G;AAchange=p.E286E;tissues=pancreas,liver&TP53_ENST00000420246;basechange=c.858A>C;AAchange=p.E286D;tissues=liver&TP53_ENST00000269305;basechange=c.858A>C;AAchange=p.E286D;tissues=liver&TP53;basechange=c.857A>C;AAchange=p.E286A;tissues=breast,kidney,large_intestine&TP53_ENST00000269305;basechange=c.857A>T;AAchange=p.E286V;tissues=liver,oesophagus&TP53_ENST00000420246;basechange=c.857A>C;AAchange=p.E286A;tissues=breast,kidney&TP53_ENST00000269305;basechange=c.857A>C;AAchange=p.E286A;tissues=breast,kidney&TP53_ENST00000420246;basechange=c.857A>T;AAchange=p.E286V;tissues=oesophagus&TP53_ENST00000420246;basechange=c.857A>G;AAchange=p.E286G;tissues=lung,large_intestine,central_nervous_system&TP53;basechange=c.857A>G;AAchange=p.E286G;tissu
C000-HZGGV2	19	59063199	G	GA	exonic	CHMP2A	unknow n	UNKNOWN	19q13.43	.
C000-HZGGV2	20	51870204	TCC	T	exonic	TSHZ2	frameshift deletion	TSHZ2:ENST00000371497.5:exon2:c.208_209del:p.P70fs,TSHZ2:ENST00000329613.6:exon2:c.199_200del:p.P67fs,TSHZ2:ENST00000603338.2:exon2:c.199_200del:p.P67fs,	20q13.2	.
C000-JMUDMJ	21	31691917	TG	T	exonic	KRTAP26-1	frameshift deletion	KRTAP26-1:ENST00000360542.3:exon1:c.436delC:p.Q146fs,	21q22.11	KRTAP26-1;basechange=c.436C>A;AAchange=p.Q146K;tissues=liver
C000-RCWM1J	1	43308820	GC	G	exonic	ERMAP	frameshift deletion	ERMAP:ENST00000372514.3:exon11:c.1346delC:p.A449fs,ERMAP:ENST00000328249.3:exon9:c.1076delC:p.A359fs,ERMAP:ENST00000372517.2:exon12:c.1346delC:p.A449fs,	1p34.2	.
C000-RCWM1J	11	33566538	TCCATACAGCCTTC	T	exonic	KIAA1549L	frameshift deletion	KIAA1549L:ENST00000265654.5:exon2:c.2127_2139del:p.V709fs,KIAA1549L:ENST00000321505.4:exon2:c.2109_2121del:p.V703fs,KIAA1549L:ENST00000389726.3:exon2:c.2127_2139del:p.V709fs,	11p13	C11orf41;basechange=c.2132C>A;AAchange=p.T711K;tissues=lung&C11orf41_ENST00000321505;basechange=c.2114C>A;AAchange=p.T705K;tissues=lung
C000-RCWM1J	16	19566929	GGCCGCTGCGGGGCGGCCAGCT	G	exonic	C16orf62	nonframeshift deletion	C16orf62:ENST00000542263.1:exon1:c.146_166del:p.49_56del,C16orf62:ENST00000538853.1:exon1:c.146_166del:p.49_56del,C16orf62:ENST00000438132.3:exon1:c.146_166del:p.49_56del,C16orf62:ENST00000539322.1:exon1:c.146_166del:p.49_56del,	16p12.3	C16orf62_ENST00000538853;basechange=c.166T>A;AAchange=p.C56S;tissues=prostate&C16orf62_ENST00000438132;basechange=c.166T>A;AAchange=p.C56S;tissues=prostate
C000-RCWM1J	19	3611932	AG	A	exonic	CACTIN	frameshift deletion	CACTIN:ENST00000585942.1:exon10:c.2265delC:p.R755fs,CACTIN:ENST00000221899.3:exon10:c.2061delC:p.R687fs,CACTIN:ENST00000248420.5:exon10:c.2265delC:p.R755fs,CACTIN:ENST00000591726.1:exon1:c.342delC:p.R114fs,CACTIN:ENST00000429344.2:exon10:c.2265delC:p.R755fs,	19p13.3	.
C000-S5ZHWR	3	167035375	C	CT	exonic	ZBBX	frameshift insertion	ZBBX:ENST00000307529.5:exon10:c.992dupA:p.Q331fs,ZBBX:ENST00000392766.2:exon13:c.992dupA:p.Q331fs,ZBBX:ENST00000392767.2:exon10:c.992dupA:p.Q331fs,ZBBX:ENST00000455345.2:exon12:c.992dupA:p.Q331fs,ZBBX:ENST00000392764.1:exon13:c.905dupA:p.Q302fs,	3q26.1	.
C000-S5ZHWR	12	109834281	CAG	C	exonic	MYO1H	frameshift deletion	MYO1H:ENST00000310903.5:exon4:c.336_337del:p.T112fs,MYO1H:ENST00000431443.2:exon3:c.336_337del:p.T112fs,	12q24.11	.
C000-X88E7Z	4	2702234	TAAG	T	exonic	FAM193A	nonframeshift deletion	FAM193A:ENST00000502458.1:exon18:c.3529_3531del:p.1177_1177del,FAM193A:ENST00000505311.1:exon17:c.3463_3465del:p.1155_1155del,FAM193A:ENST00000382839.3:exon17:c.3463_3465del:p.1155_1155del,FAM193A:ENST00000324666.5:exon17:c.3463_3465del:p.1155_1155del,FAM193A:ENST00000545951.1:exon17:c.3463_3465del:p.1155_1155del,	4p16.3	.
C000-X88E7Z	8	113349950	C	CA	exonic	CSMD3	frameshift insertion	CSMD3:ENST00000455883.2:exon42:c.6349dupT:p.L2117fs,CSMD3:ENST00000297405.5:exon43:c.6661dupT:p.L2221fs,CSMD3:ENST00000339701.3:exon28:c.4471dupT:p.L1491fs,CSMD3:ENST00000343508.3:exon44:c.6541dupT:p.L2181fs,CSMD3:ENST00000352409.3:exon41:c.6451dupT:p.L2151fs,	8q23.3	.
C000-X88E7Z	16	3434576	G	GT	exonic	ZSCAN32	frameshift insertion	ZSCAN32:ENST00000573327.1:exon6:c.1115dupA:p.E372fs,ZSCAN32:ENST00000439568.2:exon2:c.248dupA:p.E83fs,ZSCAN32:ENST00000573830.1:exon2:c.248dupA:p.E83fs,ZSCAN32:ENST00000304926.3:exon5:c.479dupA:p.E160fs,ZSCAN32:ENST00000422427.2:exon4:c.479dupA:p.E160fs,ZSCAN32:ENST00000396852.4:exon6:c.1115dupA:p.E372fs,ZSCAN32	16p13.3	.
C000-X88E7Z	17	80789858	CG	C	exonic	ZNF750	frameshift deletion	ZNF750:ENST00000269394.3:exon2:c.472delC:p.R158fs,	17q25.3	.
C000-X88E7Z	20	36845781	TG	T	exonic	KIAA1755	frameshift deletion	KIAA1755:ENST00000435901.1:exon6:c.671delC:p.P224fs,KIAA1755:ENST00000279024.4:exon13:c.2774delC:p.P925fs,	20q11.23	.

Supplemental Table 4

cytoband	1q21.3	8q24.3	7p22.1	21q22.3
q value	0,0006579	0,0006579	0,091895	0,091895
residual q value	0,0006579	0,0006579	0,091895	0,091895
wide peak boundaries	chr1:152265562-152329851	chr8:144980719-145001933	chr7:6744791-6864178	chr21:45953569-46082924
genes in wide peak	FLG	PLEC	ZNF12	TSPEAR
	FLG2		CCZ1B	KRTAP10-10
			RSPH10B	KRTAP10-4
			PMS2CL	KRTAP10-6
			RSPH10B2	KRTAP10-7
				KRTAP10-9
				KRTAP10-1
				KRTAP10-11
				KRTAP10-2
				KRTAP10-5
				KRTAP10-8
				KRTAP10-3
				KRTAP12-3
				KRTAP12-4

cytoband	5q22.2	2q32.2	2q36.3	9q22.33	15q21.1	1p21.1	6q13	10q23.31	12q24.33	3p24.1	4q13.3	13q13.1	22q11.21	10p12.33	1p22.3
q value	1,6726E-08	1,6726E-08	0,000072259	0,00081909	0,0021785	0,0044551	0,0062561	0,0062561	0,013069	0,013306	0,029455	0,041181	0,060988	0,085556	0,099783
residual q value	1,6726E-08	1,7698E-08	0,00042243	0,00081909	0,0021785	0,0044551	0,0062561	0,0062561	0,013069	0,013306	0,029354	0,041181	0,060988	0,085556	0,10347
wide peak boundaries	chr5:111747667-112200958	chr2:189876882-190014430	chr2:227853164-228192407	chr9:101829555-101983418	chr15:48630195-49034995	chr1:102454027-104074629	chr6:70502782-71133026	chr10:89611276-90034788	chr12:133457637-133851895	chr3:30043344-31205400	chr4:70620736-71204641	chr13:32875788-32993270	chr22:21380710-21922059	chr10:17754611-18292099	chr1:86166705-86820520
genes in wide peak	APC<br>FLJ11235	hsa-mir-3129<br>MIR3129	COL4A3<br>COL4A4	TGFBR1	FBN1	COL11A1	COL9A1<br>COL19A1	PTEN<br>KLLN	ZNF10<br>ZNF26<br>ZNF84<br>ZNF140<br>ZNF268<br>LOC647589<br>ZNF605	hsa-mir-466<br>TGFBR2<br>GADL1	CSN1S1<br>CSN2<br>CSN3<br>HTN1<br>HTN3<br>STATH<br>SULT1E1<br>ODAM<br>CABS1<br>FDCSP<br>CSN1S2AP<br>C4orf40<br>CSN1S2BP	BRCA2<br>ZAR1L	hsa-mir-649<br>SLC7A4<br>HIC2<br>POM121L8P<br>RIMBP3C<br>PI4KAP2<br>LOC400891<br>BCRP2<br>P2RX6P<br>RIMBP3B<br>TMEM191C	hsa-mir-511-2<br>hsa-mir-511-1<br>MRC1<br>MIR511-1<br>MIR511-2<br>TMEM236	COL24A1

Supplemental Table 5

	AC1	AC2	AC3	AC4	AC6	AC7	AC8	AC11	AC12	AC13	AC14	AC17	AC19	AC21	AC23	AC25	AC27	AC29	AC30
**C000-6KNGDL**						60		25					15						
**C000-X88E7Z**		17				39		17		26									
**C000-RCWM1J**				7		52		41											
**C000-EXKBCS**				15		49		29							7				
**C000-HZGGV2**		5		5		49		32		7		2							
**C000-ESMKCH**				15		41		44											
**C000-S5ZHWR**		7				40		31		12		10							
**C000-2SXE39**			30	30						4			11	9		12	3		
**C000-ZZP8UU**	5	15	66										11				4		
**C000-CFLP5N**	4	11	44			6		16					8						10
**C000-JMUDMJ**		16				28		22		20				13					
**C000-648QQH**	4	11	18		6	9	26		27										
**C000-W91YB6**	43	10						16		31									
**C000-AX1WLG**						61				9	8							22	

Relative signature contributions (% of SNVs attributed to the signature)
